# Aberrant APOBEC3C expression induces characteristic genomic instability in pancreatic ductal adenocarcinoma

**DOI:** 10.1038/s41389-022-00411-9

**Published:** 2022-06-24

**Authors:** Yunzhen Qian, Yitao Gong, Xuan Zou, Yu Liu, Yusheng Chen, Ruijie Wang, Zhengjie Dai, Yesiboli Tasiheng, Xuan Lin, Xu Wang, Guopei Luo, Xianjun Yu, He Cheng, Chen Liu

**Affiliations:** 1 Department of Pancreatic Surgery, Fudan University Shanghai Cancer Center, 200032 Shanghai, China; 2 Department of Oncology, Shanghai Medical College, Fudan University, 200032 Shanghai, China; 3 Shanghai Pancreatic Cancer Institute, 200032 Shanghai, China; 4 Pancreatic Cancer Institute, Fudan University, 200032 Shanghai, China

**Keywords:** Pancreatic cancer, Tumour heterogeneity, Immunotherapy, Genomic instability, DNA mismatch repair

## Abstract

Pancreatic ductal adenocarcinoma (PDAC) is a well-known lethal and heterogeneous disease. Apolipoprotein B mRNA-editing enzyme catalytic polypeptide-like (APOBEC) is an important mutagenic driver that has seldom been investigated in PDAC. Therefore, this study investigated the significance of APOBEC3C in PDAC. First, cytosine deamination-associated mutation signatures were identified in the PDAC cohorts from TCGA and Fudan University Shanghai Cancer Center (FUSCC) datasets, and C > X-enriched kataegis regions were identified in the FUSCC cohort (12 to 27 counts per sample). Patients were stratified according to APOBEC3C expression, and high APOBEC3C expression was found to correlate with a higher motif enrichment score of 5’-CC-3’ and an elevated kataegis count within *PCSK5* and *NES* genes. Second, we compared APOBEC expression in PDAC and normal pancreatic tissues and found that APOBEC3C was substantially upregulated in PDAC. APOBEC3C-overexpressing cell lines were generated to substantiate the effects of APOBEC3C on PDAC genome, including alterations in single-nucleotide variant (SNV) classes (higher proportion of C > T conversions) and the formation of kataegis regions (newly occurring kataegis regions detected in *ACHE and MUC6* genes). Three different PDAC cohorts (FUSCC, TCGA, and QCMG) were analysed to evaluate the prognostic value of APOBEC3C, and APOBEC3C overexpression predicted shorter survival. Finally, the APOBEC3C overexpression correalted with the PDAC tumour microenvironment (TME) remodelling, APOBEC3C expression was associated with the invasion of CD4 + T lymphocytes and CD8 + T lymphocytes (cytotoxic T lymphocytes, CTLs), indicating enhanced immune activity and validating the practicality of APOBEC3C for guiding immunotherapy.

## Introduction

Pancreatic ductal adenocarcinoma (PDAC) is a notorious malignancy with a poor prognosis and increasing incidence. PDAC has the lowest 5-year survival rate among all types of cancers [1] and is estimated to become the second leading cause of cancer-related death by 2030. The short survival of patients with PDAC might be largely attributed to systemic therapy resistance [2] and a high recurrence rate; accordingly, studies aiming to determine the mechanisms through which new phenotypes of PDAC develop are urgently needed.

Notably, genomic instability is one of the hallmarks of PDAC [3, 4]. PDAC is characterised by high intratumour heterogeneity and is composed of various subpopulations with diverse somatic mutations [5]. Genomic mutations (such as *KRAS* and *TP53*, Fig. S1A) drive the process of tumorigenesis [6] and enhance cancer cell plasticity in response to evolutionary pressures such as treatment [7]. Therefore, elucidating the biological processes that generate mutations has great importance. The underlying mechanisms for generating mutations [8] include exogenous mutagens, DNA replicative infidelity, DNA repair deficiency and enzyme-induced DNA alterations. With the advancement of next-generation sequencing, the genomic landscape of PDAC and the characteristic mutational signatures created by particular mutagenic components [9, 10] have been unveiled. The predominant mutational signatures present in PDAC are associated with age, *BRCA* mutations, DNA mismatch repair deficiency (dMMR) and apolipoprotein B mRNA-editing enzyme catalytic polypeptide-like (APOBEC) proteins [11].

The APOBEC family comprises activation-induced cytidine deaminase (AICDA), APOBEC1, APOBEC2, APOBEC4 and seven APOBEC3 subfamily homologues [12]. APOBEC deaminates cytosine in single-stranded DNA (ssDNA) or mRNA, resulting in cytosine (C) to uracil (U) conversions [13], and U·G mispairs are generally transformed to C > T single point mutations at replication forks. Moreover, along with the participation of uracyl deglycosylases in base excision repair and mismatch repair (MMR), high levels of C > X mutations and strand breaks lead to chromosome translocation. Thus, APOBEC is capable of causing genomic instability. Physically, APOBEC is involved in biological processes of somatic hypermutation and class switch recombination for B-cell maturation [14], innate immunity to remove foreign retroviruses [15], and epigenetic reprogramming of DNA demethylation [16, 17].

Accumulating evidences suggested APOBEC also promotes tumorigenesis in diverse organs [18–20]. APOBEC is implicated not only in haematological malignancies [21–23] but also in various solid tumours [24–27]. APOBEC induces a high tumour mutational burden (TMB), nourishing neoantigens that supplement tumour evolution and adaptation to therapy [28–30]. However, research on the role of APOBEC in PDAC is scarce. Here, we performed deep whole-exon sequencing (WES) in 124 PDAC tissues to identify mutational signatures and characteristic kataegis regions and substantiated the effect of APOBEC3C on the PDAC genome by generating APOBEC3C-overexpressing cell lines. Publicly available datasets were used to confirm our conclusions. In addition, the overall survival (OS) of patients with PDAC differed between the subgroups stratified by APOBEC3C expression. Finally, we showed that APOBEC3C expression correlated with PDAC tumour microenvironment (TME) remodelling. This is the first investigation reporting prognostic and therapeutic values of APOBEC3C in PDAC, and our study also contributes to deciphering PDAC genomic features [31].

## Methods

### Online databases

Open access data (RNA expression and corresponding patient survival times) were sourced from The Cancer Genome Atlas (TCGA) and Queensland Centre for Medical Genomics (QCMG) [32] using Xena browser (https://xenabrowser.net) and cBioPortal (http://www.cbioportal.org/). Comparison of APOBEC expression in TCGA and Genotype-Tissue Expression project (GTEx) databases was performed using GEPIA2 (http://gepia2.cancer-pku.cn) [33], which normalised data by the maximum median expression value across all blocks and then made comparison between PDAC and normal pancreatic tissues. The maf file bcgsc.ca_PAAD.IlluminaHiSeq_DNASeq.1.somatic.maf (*n* = 147) was downloaded from GDC (https://portal.gdc.cancer.gov).

### Cell culture

The human pancreatic cancer cell lines BxPC-3, CFPAC-1, MIA PaCa-2 and SW 1990 were obtained from the National Collection of Authenticated Cell Cultures, Capan-1, Capan-2, and SU.86.86 were obtained from the American Type Culture Collection. The human pancreatic ductal epithelial cell line HPDE6c7 (H6c7) was obtained from Kyushu University. Cell lines were authenticated by STR and tested for mycoplasma contamination.

BxPC-3 and SU.86.86 were cultured in RPMI 1640, Capan-1 and CFPAC-1 were cultured in IMDM, Capan-2 was cultured in McCoy’s 5A modified medium, H6c7 and MIA PaCa-2 were cultured in Dulbecco’s modified Eagle medium (DMEM), and SW1990 was cultured in L15 medium. All cell culture media were supplemented with 10% heat-inactivated foetal bovine serum and 100 U/mL penicillin–streptomycin, and DMEM used for culturing MIA PaCa-2 was additionally supplemented with 2.5% horse serum. All cells were cultured at 37°C with 5% CO_2_.

### Overexpression vector construction and transfection

Wild-type APOBEC3C consensus coding sequence (CCDS) (APOBEC3C-pENTER vector, WZ Biosciences) was amplified by PCR (forward primer: 5’-CCGTCAGATCCGCTAGTAATAC-3’, reverse primer: 5’-CCGGAATTCTGTGGTATGGCTGATTATGATC-3’). Then, BamHI and EcoRI were employed to generate sticky ends. The pCDH-CMV-puro plasmid was digested with the same enzymes, and restriction enzyme-digested products were purified by electrophoresis and Universal DNA Purification Kits (Cat#DP214-03, Tiangen, China). The CCDS fragment and pCDH plasmid were ligated using DNA Ligation Kits (Cat# 6021, Takara, Japan) and then transformed into DH5α cells for further selection and amplification.

EndoFree Mini Plasmid Kit II (Cat# DP118-02, Tiangen) was used to extract pCDH-APOBEC3C (pCDH-A3C). Then, 7.5 µg pCDH-A3C or pCDH backbone plus 5.625 µg psPAX2 and 1.875 µg pMD2.G were incubated with 30 µL Hieff Trans Liposomal Transfection Reagent (Cat# 40802ES03, Yeason, China) in 1 mL DMEM. The mixture was added to 293T cells to generate lentiviruses, which were used to transfect pancreatic cell lines. Puromycin was added to culture mediums (4 µg/mL) for selection. Four months after transfection, total DNA was extracted from A3C-overexpressing cell lines and subjected to Sanger sequencing to verify that A3C sequence in the plasmid did not undergo genetic modifications.

### Clonogenic assay

Cells were suspended in PBS, and its density was counted by BECKMAN COULTER (Cat# AW29395, System 7221901). Then, PBS containing 1500 cells was added to 6 mL medium. Cells were resuspended before being transferred into three wells of a 6-well plate and cultured for two weeks. Clones were fixed with 4% paraformaldehyde and stained with crystal violet. Clones were counted, and Welch’s *t*-test was used to compare the differences.

### Patients and specimens

Pancreatic tumour specimens for WES and transcriptomics were obtained from 124 other patients, defined as FUSCC cohort A. Pancreatic tumour specimens for quantitative real-time polymerase chain reaction (qRT–PCR) and survival analyses were obtained from 124 other patients, defined as FUSCC cohort B. Sixty tumour centre tissues and six normal tissues remote from tumour sites for immunohistochemical (IHC) staining were also obtained from FUSCC cohort B, defined as FUSCC cohort B1. All patients were pathologically diagnosed with PDAC after radical surgical resection in the Department of Pancreatic Surgery Shanghai Cancer Center, Fudan University, China.

OS was defined as the length of time from the beginning of diagnosis to death from any cause or the last follow-up. The follow-up period of cohort B ended in November 2019. This study was approved by the Ethics Board of Shanghai Cancer Center, Fudan University, and all patients involved in this study provided written informed consent for use of their specimens and personal data for research purposes.

### Mutational signature and SNV distribution analysis

DeconstructSigs R package (version 1.8.0) was used to analyse mutational signatures [34] and compare the results to COSMIC reference signatures (SBS v3.2, March 2021). Ramtools R package (version 2.4.0) was used to obtain flanking sequences centred on each mutation, and APOBEC motif enrichment scores were calculated using the formulas [35]. MeichunCai/KataegisPortal R package (version 1.0.3) [36] was applied to detect and locate kataegis regions and calculate the proportion of C > X mutations in kataegis. Maftools R package (version 2.4.12) [37] was used to detect the differentially altered genes between non-APOBEC-enriched samples and APOBEC-enriched samples in TCGA PDAC cohort.

### Quantitative real-time polymerase chain reaction

Total RNA was extracted from cell lines and PDAC tissues using EZ-press RNA Purification Kits (Cat# B0004D-100, EZBioscience, USA) and Tissue RNA Purification Kit PLUS (Cat# EZB-RN001-plus, EZBioscience), respectively. RNA was excluded from further experiments if its 260/280 nm optical density ratio was less than 1.85 or greater than 2.15 or its concentration was less than 100 ng/µL. Reverse transcription was conducted using 4× EZscript Reverse Transcription Mix II (Cat#EZB-RT2GQ, EZBioscience).

qRT–PCR was performed using a QuantStudio™ 7 Flex Real-Time PCR system (Cat#4485701, Applied Biosystems) and 2×Colour SYBR Green qPCR Master Mix (Cat#A0012-R2, EZBioscience). ACTB was used as an internal control, and one PDAC sample was used as an interplate control. All reactions were run in triplicate. All primers for qRT–PCR were designed by Primer Premier. Primer specificity was checked using Basic Local Alignment Search Tool (https://blast.ncbi.nlm.nih.gov/Blast.cgi). All primers produced single-peak melting curves. The primer sequences are listed in Table S1.

### Immunohistochemistry

Formalin-fixed, paraffin-embedded (FFPE) pancreatic specimens were sectioned into 4-micron-thick slices, including 60 tumour tissues and 6 normal tissues. After deparaffinization and rehydration, 3% H_2_O_2_ was used to block endogenous peroxidases for 15 min. Then, slices were heated in Tris-EDTA buffer (pH = 9.0) for 10 min to retrieve antigens and blocked with 2.5% goat serum for 1 h. The following primary antibodies were used: polyclonal antibody against APOBEC3C (10591-1-AP, Proteintech, USA), recombinant antibody against CD4 (ab133616, Abcam, England), and polyclonal antibody against CD8 (ab4055, Abcam). The isotype control was ab37415 (polyclonal rabbit IgG, Abcam). Colouration was performed using a GTVision^TM^ III Detection System/Mo&Rb (Cat#GK500710, Gene Tech, China), and haematoxylin was used for counterstaining. Finally, sections were dehydrated and re-embedded for observation. Expression was considered positive only when positive reaction products were localised in the expected cellular compartment. The counts of immune cells were defined as the mean number of immune cells in three representative high-power fields (20×). Immune cells were counted manually.

### Survival analysis

Continuous variables (APOBEC3C mRNA expression level) were dichotomised by optimal cutoff values ascertained by survminer R package (version 0.4.9). Kaplan–Meier method was employed to plot the survival curves. Survival curves of different groups were compared using log-rank (Mantel–Cox) tests, and hazard ratios (HRs) were calculated using Mantel–Haenszel method.

### ESTIMATE and CIBERSORT analyses

ESTIMATE method [38] was applied to infer the proportions of immune and stromal components in the TME of TCGA PDAC samples. Single-sample gene set enrichment analysis (ssGSEA) output the immune score, which represents the level of infiltrating immune cells, and the stromal score, which represents the level of stromal cells in tumour tissues; then, the immune score and the stromal score were integrated into the ESTIMATE score, which represents the purity of tumour samples.

CIBERSORT (https://cibersort.stanford.edu/) was used to estimate the fractions of 22 types of immune cells by deconvoluting bulk tumour gene expression profiles based on the characteristic gene signature file “LM22”. The normalised gene expression matrix of TCGA PDAC dataset was uploaded to generate corresponding leucocyte proportion matrix. Spearman’s rank correlation coefficient was calculated to measure the correlation [39].

## Results

### APOBEC contributes to shaping mutational signatures in PDAC

**Fig. 1:**
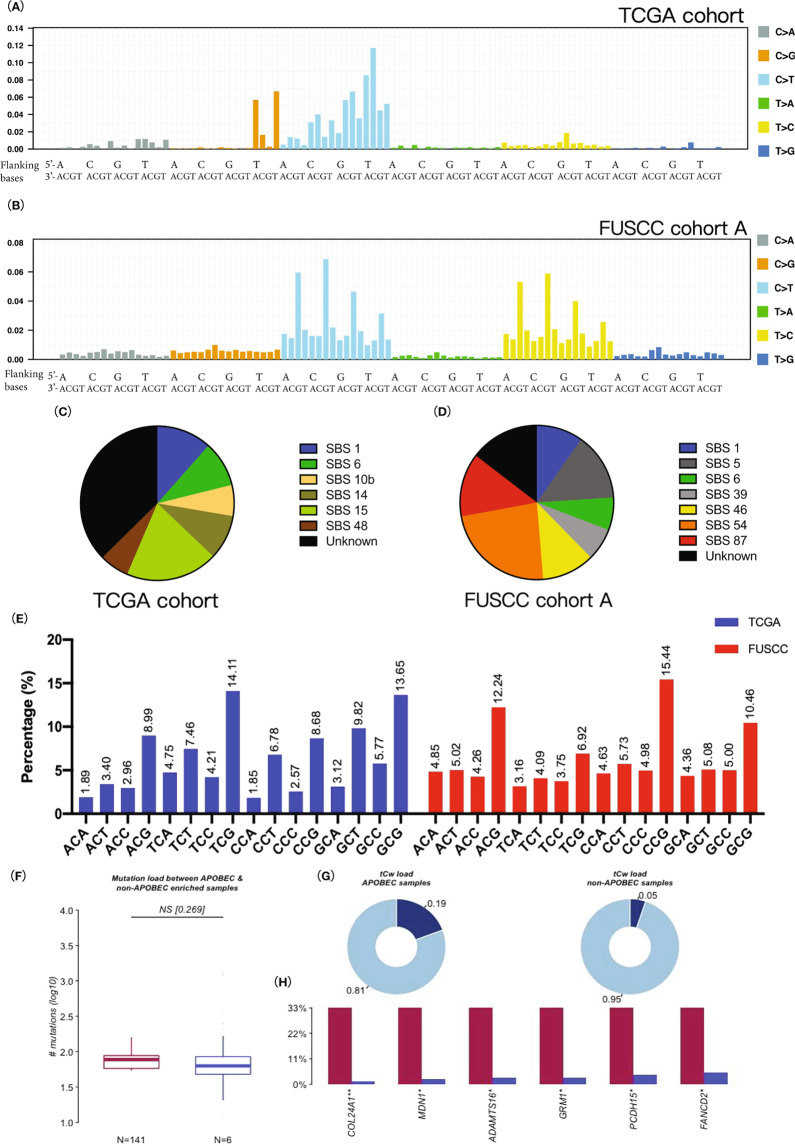
Mutational signatures and SNV distribution in the PDAC genome. **A**, **B** Mutational signatures deconstructed from TCGA PDAC cohort (**A**) and FUSCC PDAC cohort A (**B**). The upper *x*-axis represents the nucleotide in the 5’ end flanking the SNVs, and the lower *x*-axis represents the nucleotide in the 3’ end flanking the SNVs. **C**, **D** Comparison between the deconstructed mutational signatures and validated SBS signatures (v3.2, March 2021). In TCGA cohort (**C**), the SBS1 weight was 0.115, SBS6 and SBS15 weights were 0.0955 and 0.193, respectively, SBS10b weight was 0.0649, and SBS14 weight was 0.0935. SBS48 correlated with potential artefacts and its weight was 0.0618. In FUSCC cohort A (**D**), the SBS1 weight was 0.0975, SBS5 and SBS39 weights were 0.142 and 0.068, respectively, SBS6 weight was 0.0685, and SBS87 weight was 0.133. SBS46 and SBS54 correlated with potential artefacts and their weights were 0.111 and 0.234, respectively. **E** Summary of trinucleotide motifs of all C > X mutations in TCGA cohort and FUSCC cohort A. **F**, **G**, **H** APOBEC-enriched samples referred to those samples with an enrichment score E-TCA > 2. The comparison between non-APOBEC-enriched samples (TCGA PDAC cohort, *n* = 141) and APOBEC-enriched samples (*n* = 6) was performed using the “plotApobecDiff” function of the maftools R package, including comparisons of the mutation burden (**G**), tCw burden (**H**) and differentially altered genes (**I**). Detailed information was shown in Table S2. SNV, single-nucleotide variant; PDAC, pancreatic ductal adenocarcinoma; SBS, single-base substitution; tCw, w = A or T.

Mutational signatures in the tumour genome indicate corresponding aetiology. Therefore, we extracted mutational signatures from TCGA PDAC cohort and FUSCC cohort A by referring to the latest COSMIC single-base-substitution signatures (SBS, v3.2, Fig. 1A–D). The two cohorts concurrently reflected that SNVs in the PDAC genome were primarily composed of C > T conversions, consistent with the biological function of APOBEC.

In TCGA PDAC cohort, SBS1 (associated with spontaneous or enzymatic deamination of 5-methylcytosine) had a weight of 0.115. SBS6 and SBS15 (associated with dMMR) had weights of 0.0955 and 0.193, respectively, SBS10b (associated with polymerase epsilon (POLE) exonuclease domain mutations) had a weight of 0.0649, and SBS14 (associated with concurrent dMMR and POLE mutation) had a weight of 0.0935.

In FUSCC cohort A, SBS1 had a weight of 0.0975, SBS6 had a weight of 0.0685 and SBS87 (associated with thiopurine chemotherapy treatment) had a weight of 0.133. The remaining deconstructed signatures have not yet been correlated with a definite mutagen.

We additionally compared deconstructed mutational signatures of TCGA PDAC genome with classical reference mutational signatures (COSMIC v1.0, August 2013) and matched reference signatures 1, 2, 5, 6, 14 and 18. Note that signature 1 is associated with age, signature 2 is associated with APOBEC and signature 6 is associated with dMMR (Fig. S2E).

Briefly, age, APOBEC, dMMR and POLE mutations established the mutational signature of the PDAC genome.

### Single-nucleotide polymorphisms in PDAC match preferred targets of APOBEC3 subfamily

Members of the APOBEC family have a lax sequence preference; thus, we analysed flanking bases of mutated sites to identify the trinucleotide motif of the C > X mutation. The APOBEC3 subfamily has been reported to prefer the 5’-TC-3’ and 5’-CC-3’ sequences [13]. Coincidently, we found that C > X mutations mostly occurred in 5’-TCG-3’ motif in TCGA PDAC cohort. In FUSCC cohort A, however, C > X mutations mainly occurred in 5’-CCG-3’ motif (Fig. 1E).

We selected 25 samples with the highest APOBEC3C expression and 25 samples with the lowest APOBEC3C expression from FUSCC cohort A, calculated motif enrichment scores and made comparisons. The high APOBEC3C expression subgroup had a higher 5’-CC-3’ enrichment score (Table S3, 1.319 versus 1.310, *p* = 0.0093). However, significant differences in 5’-TC-3’-related motif enrichment scores were not observed.

### APOBEC3C is the most abundant member of the APOBEC family in PDAC

**Fig. 2:**
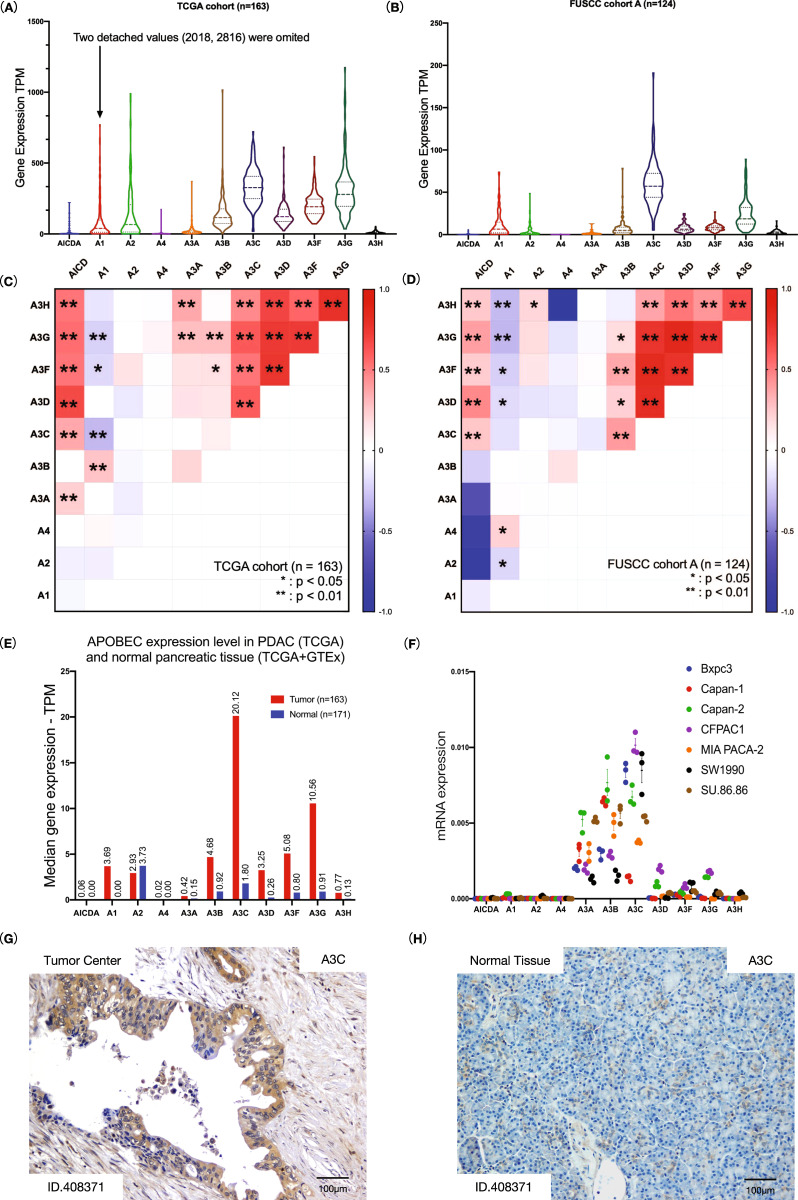
APOBEC3C is expressed at the highest level among APOBECs in PDAC. **A**, **B** The mRNA expression levels of APOBECs in TCGA (*n* = 163) and FUSCC cohort A (*n* = 124). **C**, **D** The Pearson correlation coefficients were calculated to examine the correlations between APOBEC expression in TCGA cohort (**C**) and FUSCC cohort A (**D**). *p < 0.05, **p < 0.01. Detailed information was shown in Table S5. **E** The height of the bar represents the median mRNA expression level of a specific APOBEC. GEPIA2 normalised data by the maximum median expression value across all blocks and then compared data between pancreatic tumour tissues (TCGA database, *n* = 163) and normal tissues (TCGA and GTEx databases, *n* = 171) using one-way ANOVA, *p-value < 0.01. Corresponding box plots are displayed in Fig. S3. **F** Expression of APOBEC enzymes in PDAC cell lines. The mRNA expression level was determined by performing qRT–PCR on validated cDNAs in triplicate. **G**, **H** Representative images (patient ID.408371, FUSCC cohort B1) of IHC staining for APOBEC3 in PDAC (**G**) and normal pancreatic tissues (**H**). Normal pancreatic tissues from six patients with PDAC were stained, and the results are presented in Fig. S4. PDAC, pancreatic ductal adenocarcinoma; TPM: transcripts per million mapped reads.

We compared the mRNA expression levels of the APOBEC family in PDAC tissues to determine the predominant APOBEC in PDAC and found that APOBEC3C had the highest average mRNA expression level (Fig. 2A, B and Fig. S3A). We were concerned that APOBEC isoforms share similarity in their sequences, thus confounding their transcriptomics quantification results (Table S4). Therefore, we further tested the correlation between the transcripts per million mapped reads (TPM) of APOBEC family members. We found that the APOBEC3C TPM was correlated with the TPM values of AICDA, A1 and other APOBEC3 family members (Fig. 2C, D and Table S5). Whereas, multiple linear regression models showed that only APOBEC3D TPM correlated with APOBEC3C TPM (Table S6, *β* > 0.8, *p* < 0.001).

Afterwards, we compared the median TPM of each APOBEC enzyme in PDAC and normal pancreatic tissues from TCGA and GTEx datasets and obtained consistent results (Fig. 2E and Fig. S3B). Compared to normal pancreatic tissue, almost all APOBEC enzymes were upregulated in PDAC, among which APOBEC3C displayed the highest expression level. APOBEC3C expression in PDAC and normal pancreatic tissues was 20.12 and 1.80 TPM, respectively (*p* < 0.05).

We then investigated the mRNA expression levels of the APOBEC family in seven PDAC cell lines. APOBEC3C tended to be the main APOBEC enzyme expressed in CFPAC-1, Bxpc-3, and Capan-2 (Fig. 2F and Fig. S3C). Interestingly, the comparatively low expression of APOBEC3C was accompanied by relatively high expression of APOBEC3B and APOBEC3A in Capan-1.

We performed IHC to corroborate the in silico findings by staining for APOBEC3C in PDAC and normal pancreatic tissues and found apparent upregulated expression of APOBEC3C in PDAC (Fig. 2G, H and Fig. S4A).

### APOBEC3C is associated with the kataegis distribution pattern in the PDAC genome

Remarkably, APOBECs provides impetus to the formation of kataegis events (Greek for thunderstorms) [40]. Kataegis refers to mutation showers with clustered mutations in small localised genomic regions [41]. Putative kataegis is defined as genomic segments containing six or more consecutive mutations with an average intermutational distance of less than or equal to 1000 bp [11]. Considering the biological function of APOBEC, kataegis with enriched C > X substitutions is regarded as a consequence of APOBEC attack on the genome. Kataegis was observed in normal B lymphocytes [42], haematological malignancies such as B-cell lymphomas [42] and multiple myeloma [43], as well as solid tumours such as breast cancer [40] and osteosarcomas [25]. A recent pancancer analysis also reported the presence of kataegis in PDAC [31].

**Fig. 3:**
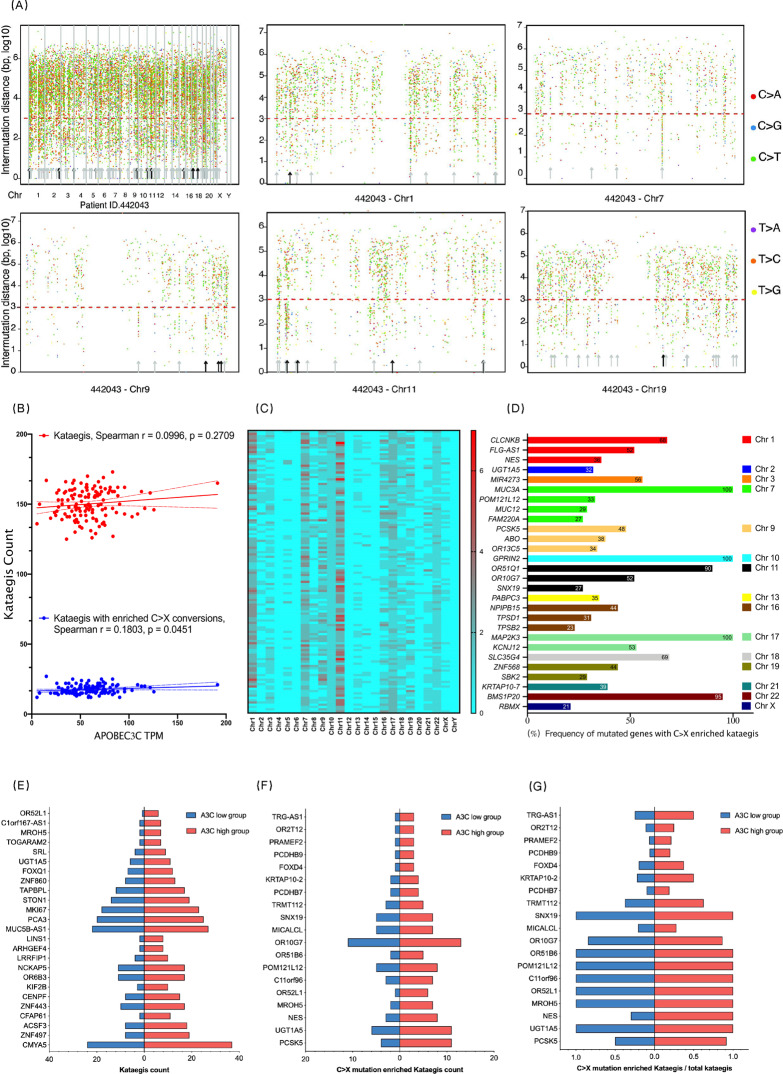
The chromosomal distribution of kataegis regions with enriched C > X substitutions is correlated with APOBEC3C expression. The MeiChuncai/KataegisPortal R package was used to detect kataegis in FUSCC cohort A (*n* = 124) and calculate the C > X mutation proportion in each kataegis region. The Ramtools R package was used to obtain trinucleotide motifs of mutations. Each PDAC sample harboured 125 to 173 kataegis regions. Detailed information about kataegis in FUSCC cohort A is recorded in Tables S7-10. **A** Representative rainfall plot showing a PDAC sample (Patient ID: 442043) harbouring 159 kataegis regions. The *x*-axis represents the SNV position, and the *y*-axis represents the intermutational distance. As the density of the mutation distribution may influence the observation of kataegis, we separately display kataegis regions on chromosomes 1, 7, 9, 11, and 17 to show the kataegis regions with enriched C > X conversions. Kataegis were pointed out by arrows, and kataegis with enriched C > X mutations were labelled by solid arrows. **B** Nonparametric Spearman correlation test to determine whether the count of kataegis regions with enriched C > X substitutions or the count of total kataegis regions correlated with APOBEC3C expression. **C** Heatmap showing the chromosomal distribution of kataegis regions with enriched C > X substitutions (more than 5 C > X substitutions per 1000 bp). The colour ladder was based on the counts of kataegis regions. The *y*-axis represents the hospital ID of each sample in FUSCC. **D** Genes with enriched C > X kataegis regions. Genes with frequencies lower than 20% were omitted from this figure. The complete list is recorded in Table S9. SNV, single-nucleotide variant; PDAC, pancreatic ductal adenocarcinoma; chr, chromosome; r, correlation. **E**, **F**, **G** Comparison of total kataegis count (**E**), C > X mutation-enriched kataegis count (**F**) and proportion of C > X mutation-enriched kataegis in total kataegis (**G**) between APOBEC3C highest expressed samples (*n* = 25) and APOBEC3C lowest expressed samples (*n* = 25) from FUSCC cohort A.

We performed WES (>200×) in FUSCC cohort A to sensitively and accurately detect SNVs and analyse the presence of kataegis. A deep sequencing depth was essential because tumour-derived DNA was mixed with normal DNA from tumour adjacent tissues, and mutations with a low variant allele frequency (VAF) are only detectable using deep sequencing [44, 45]. The total kataegis count was 125 to 173 per FUSCC cohort A sample (Fig. 3A and Table S7) and did not show an association with the APOBEC3C TPM (Fig. 3B, *r* = 0.0996, *p* = 0.2709). We further discerned the kataegis with more than five C > X conversions per 1000 bp to more clearly reflect the possible effect of APOBEC. The count of kataegis with enriched C > X conversions ranged from 12 to 27 per sample and was marginally correlated with the APOBEC3C TPM (*r* = 0.1803, *p* = 0.0451). Kataegis with enriched C > X conversions were primarily located on chromosomes 1, 7, 9, 11, and 17 within *MAP2K3*, *GPRIN2*, *MUC3A*, and *BMS1P20* genes (Fig. 3C, D; Tables S8 and S9). We then used a stricter filtering condition to examine whether TpC to TpX mutations formed kataegis in the PDAC genome. Kataegis completely consisting of TpC to TpX mutations were observed in *MIR4273* gene and etc., accounting for 0 to 2 counts per sample (Fig. S2F and Table S10).

We then compared the kataegis distribution of the 25 samples with the highest APOBEC3C expression and the 25 samples with the lowest APOBEC3C expression from FUSCC cohort A. Higher kataegis counts were observed within *CMYA5*, *ZNF49*, and *ACSF3* genes in the A3C high subgroup. The counts of kataegis with enriched C > X conversions were higher in *PCSK5*, *UGT1A5*, *NES*, *OR52L1*, *MROH5* genes in A3C high subgroup, the proportion of kataegis with enriched C > X conversions among total kataegis increased within *PCSK5*, *NES*, *TRMT112*, *KRTAP10-2*, and *TRG-AS1* genes (Fig. 3E, F, G). Therefore, we speculated that APOBEC3C may influence the formation of kataegis within *PCSK5, NES* genes and etc.

### APOBEC3C overexpression results in newly occruing SNVs and kataegis in pancreatic cell lines

**Fig. 4:**
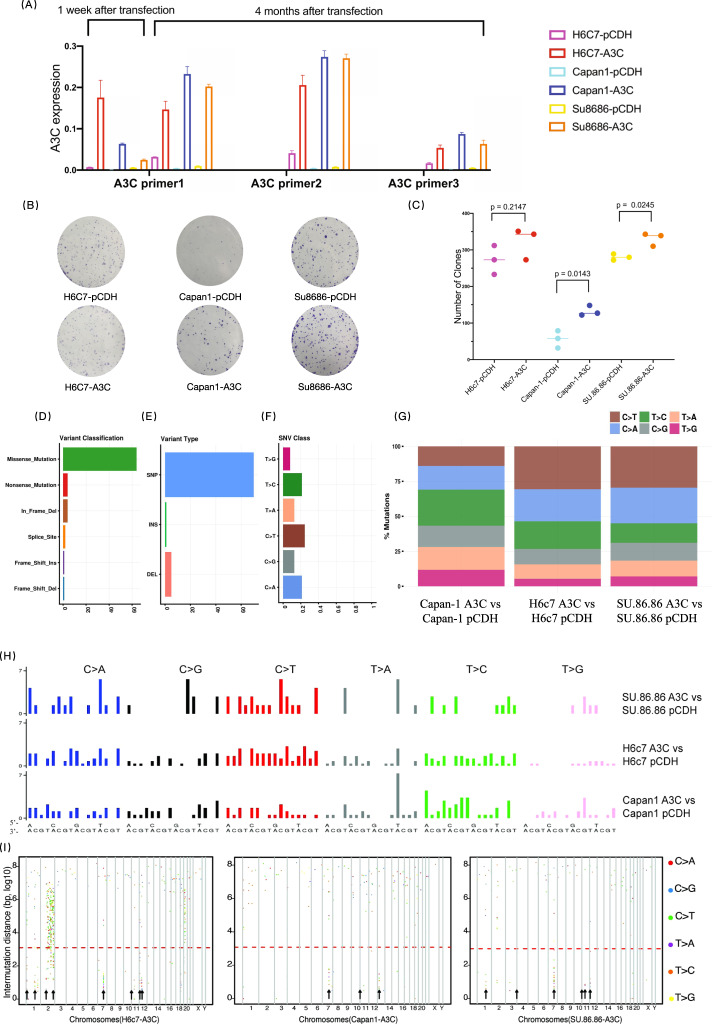
Transfection of pCDH-APOBEC3C into pancreatic cell lines causes characteristic genomic alterations. **A** APOBEC3C-overexpressing cell lines were generated by transfecting a pCDH plasmid loaded with the APOBEC3C CCDS, and the control group was transfected with the pCDH backbone plasmid. Multiple APOBEC3C primer pairs were applied to perform qRT–PCR in triplicate at different time points after transfection to validate that the cell lines transfected with the pCDH-A3C plasmid had a steadily elevated APOBEC3C mRNA expression level compared to the corresponding control cell lines. The corresponding western blotting results are shown in Fig. S4. **B** Representative images of the clonogenic assay. The assay was repeated in triplicate. **C** Summary of clone counts for H6c7-pCDH (mean = 272.7, SD = 39.5), H6c7-A3C (mean = 322.3, SD = 42.91), Capan-1-pCDH (mean = 56.33, SD = 23.54), Capan-1-A3C (mean = 132.3, SD = 13.8), SU.86.86-pCDH (mean = 280.3, SD = 8.505) and SU.86.86-A3C (mean = 280.3, SD = 8.505) cells. Welch’s *t*-test was applied to perform statistical analyses and obtain a two-tailed *p*-value. **D**, **E**, **F** Classification of newly occurring variants in APOBEC3C-overexpressing cell lines. Missense mutation was the major variant type (**D**), SNV was more prevalent than INS and DEL (**E**), and C > T mutation was the primary SNV type (**F**). **G** Mutation spectrum of newly occurring SNVs in APOBEC3C-overexpressing cell lines compared to corresponding control cell lines. **H** Mutational signatures of newly occurring SNVs in H6c7, SU.86.86 and Capan-1 cells. The upper *x*-axis represents the nucleotide at the 5’ end flanking the mutated sites, and the lower *x*-axis represents the nucleotide at the 3’ end flanking the mutated sites. **I** Rainfall plots showing that some of the newly occurring SNVs in APOBEC3C-overexpressing cell lines had short intermutational distances and formed characteristic kataegis regions on chromosomes 1, 2, 3, 7, 11, 12, and 13. The *x*-axis represents the SNV position, and the *y*-axis represents the intermutational distance. The complete list of newly formed kataegis regions is recorded in Table S12. CCDS, consensus coding sequence; SNV/SNP, single-nucleotide variant/polymorphism; INS, insertion; DEL, deletion.

A normal human pancreatic ductal epithelial cell line (H6c7) and two PDAC cell lines (SU.86.86 and Capan-1) with relatively low expression of APOBEC3C were used to construct APOBEC3C-overexpressing cell lines and simulate the effect of APOBEC3C on the genome. We excluded MIA PaCa-2 because they have a hypotriploid karyotype with a few absent normal chromosomes and thus lack representativeness. Ectopic APOBEC3C overexpression was validated by qRT–PCR and western blotting (Fig. 4A and Fig. S4B). Cell lines with APOBEC3C overexpression tended to form more and larger clones in the colony formation assay (Fig. 4B, C, increases in Capan-1 A3C and SU.86.86-A3C, and a tendency to increase in H6c7 A3C).

Cell lines were cultured for another four months to accumulate mutations before WES. We extracted newly occurring SNVs in APOBEC3C-overexpressing cell lines compared to the corresponding control cell line (Table S11). In diploid cell lines (H6c7 and SU.86.86), the newly occurring SNVs were primarily composed of C > T conversions, while in Capan-1, an even distribution of the mutational spectrum of newly occurring SNVs was observed (Fig. 4D–H). In addition, the mutational signature reflected that most newly occurring C > X substitutions tended to be located in TpCpX trinucleotides in APOBEC3C-overexpressing cell lines, consistent with the preferred APOBEC3C target sequence. All three APOBEC3C-overexpressing cell lines had newly occurring kataegis regions in *ACHE* and *MUC6* genes (Fig. 4I and Table S12). Additionally, APOBEC3C-overexpressing cell lines had newly occurring kataegis regions in *HRNR* (H6c7 and SU.86.86), *PABPC3* (Capan-1) and *TNS1* (H6c7) genes. Kataegis oriented within *HRNR*, *PABPC3*, and *TNS1* genes were also observed in FUSCC cohort A. Therefore, we speculated that APOBEC3C overexpression was capable of inducing C > X substitution in pancreatic cell lines and generating characteristic kataegis.

### A high level of APOBEC3C expression predicts shorter overall survival for patients with PDAC

**Fig. 5:**
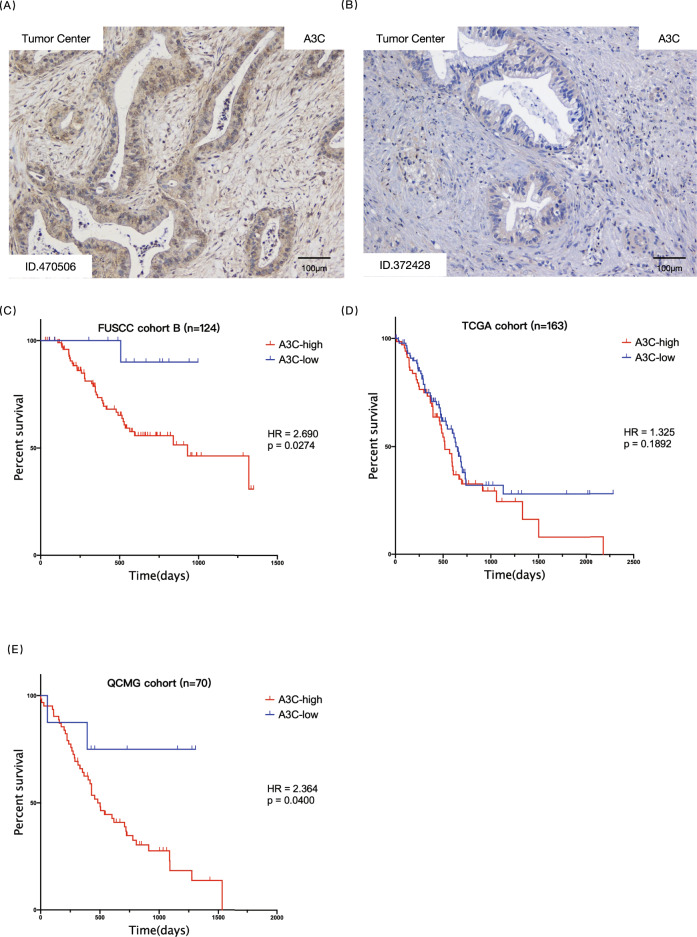
Universal high APOBEC3C expression in PDAC denotes shorter OS. The Mantel–Cox test was used to compare Kaplan–Meier survival curves. **A**, **B** Representative IHC images showing relatively high expression (**A** patient ID: 470506) and relatively low expression (**B** patient ID: 372428) of APOBEC3C in PDAC tissues. Sixty PDAC specimens (FUSCC cohort B1) underwent IHC staining with an APOBEC3C antibody to corroborate the qRT–PCR results. Positive APOBEC3C staining was located in the nucleus and cytoplasm of pancreatic tumour cells. **C** A cutoff value of 0.011802837 (qRT–PCR result) stratified the patients in FUSCC cohort B into the high APOBEC3C expression subgroup (*n* = 109) and low APOBEC3C expression subgroup (*n* = 15) with a difference in OS. The mRNA expression level was determined by performing qRT–PCR with cDNAs in triplicate, and the normalised mean value was used for analysis. **D** A cutoff value of 353.34 TPM stratified TCGA cohort into the high APOBEC3C expression subgroup (*n* = 69) and the low APOBEC3C expression subgroup (*n* = 94) with a trend towards a difference in OS. **E** A cutoff value of 372.77 TPM stratified the QCMG cohort into the high APOBEC3C expression subgroup (*n* = 62) and the low APOBEC3C expression subgroup (*n* = 8) with a difference in OS. PDAC, pancreatic ductal adenocarcinoma; A3C, APOBEC3C; HR, hazard ratio.

Given the importance of A3C in causing PDAC genomic instability, we further investigated the correlation between APOBEC3C expression and the survival of patients with PDAC. We quantified the APOBEC3C mRNA expression level in FUSCC cohort B by performing qRT–PCR with APOBEC3C primer 1 (targeting A3C specific sequence to avoid other APOBECs’ confounding) and obtained its correlation with patient prognosis. The APOBEC3C mRNA expression level measured by qRT–PCR was cross-validated at protein level by performing IHC in 60 FFPE tumour tissues (FUSCC cohort B1, Fig. 5A, B).

We calculated the optimal cutoff values and then stratified the three cohorts accordingly. FUSCC cohort B was divided into high and low APOBEC3C expression groups (*n* = 109 and *n* = 15, respectively), and patients without ectopic APOBEC3C expression experienced a longer OS (Fig. 5C, mean days of survival = 947.1 days versus 856.7, HR = 2.690, *p* = 0.0274). TCGA PDAC cohort was also stratified into high and low APOBEC3C expression subgroups (*n* = 69 and *n* = 94, respectively), and patients with comparatively low APOBEC3C expression tended to experience longer OS (Fig. 5D, mean days of survival = 634.0 days versus 518.0 days, HR = 1.325, *p* = 0.1892). QCMG PDAC dataset includes 70 patients with PDAC who have complete survival data and APOBEC3C expression data, and they were stratified into high and low APOBEC3C expression groups (*n* = 62 and *n* = 8, respectively). The latter group had a longer OS (Fig. 5E, mean days of survival were 1190.8 days versus 645.5 days, HR = 2.982, *p* = 0.0022). Stratification of FUSCC and QCMG datasets were in accordance with the result that most PDAC samples overexpressed APOBEC3C and few PDAC samples did not (Fig. 2A, B, E and Fig. S3A, B).

As APOBEC3D expression correlated with APOBEC3C expression in transcriptomics (Tables S5 and 6), we further performed survival analysis based on APOBEC3D expression to assess possible confounding effects. Patients with comparatively low APOBEC3D expression in TCGA cohort and QCMG cohort experienced longer survival (Fig. S5A, TCGA cohort, mean days of survival = 614.0 days versus 517.0 days, HR = 1.408, *p* = 0.1776; B, QCMG cohort, mean days of survival = 912.0 days versus 429.0 days, HR = 2.106, *p* = 0.0148). However, multivariable Cox analysis showed that neither APOBEC3C nor APOBEC3D expression correlated with survival when analysing transcriptimics data (Table S13).

### APOBEC3C remodels the PDAC immune microenvironment

**Fig. 6:**
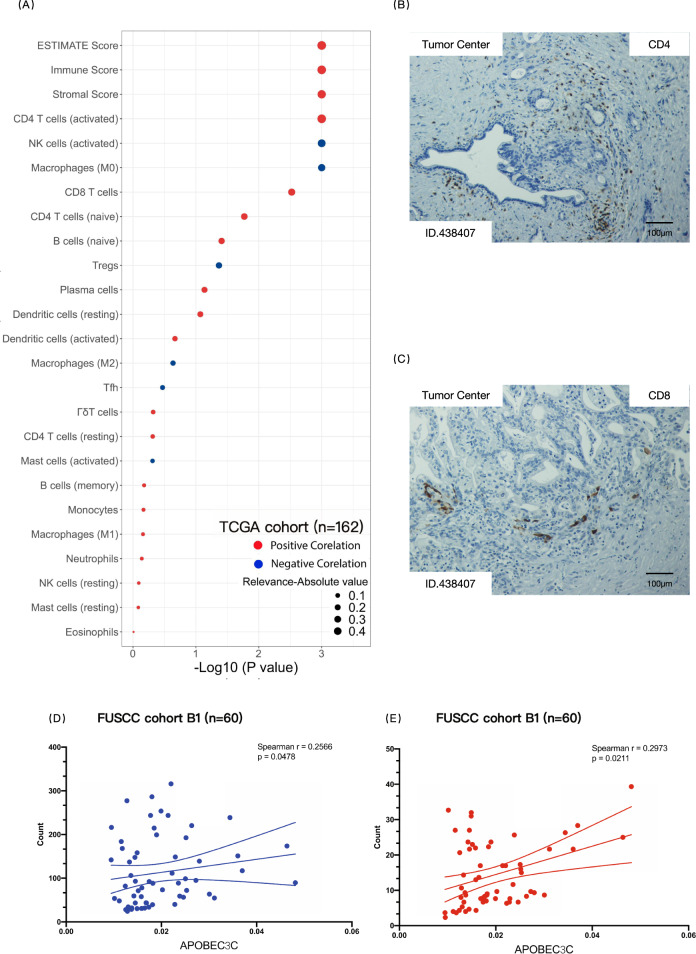
APOBEC3C expression in PDAC correlates with TME remodelling. **A** ssGSEA was used to analyse the stroma of TCGA PDAC samples (*n* = 162) and output the immune score and the stromal score. The immune score and the stromal score were integrated into the ESTIMATE score. The CIBERSORT analytical tool was used to estimate the fractions of 22 types of immune cells in the matrix of TCGA PDAC dataset (based on the characteristic gene signature file “LM22”). Spearman’s rank correlation coefficient was calculated to measure the correlation between APOBEC3C expression and leucocyte fractions in the PDAC TME. **B**, **C** Representative images showing CD4 IHC staining (**B**) and CD8 IHC staining (**C**) in FUSCC cohort B1 (*n* = 60). For each sample, immune cells were manually counted in three representative high-power fields. **D**, **E** Nonparametric Spearman’s correlation test to determine whether the count of CD4 T cells (**D**) or the count of CD8 T cells (**E**) correlated with APOBEC3C expression in FUSCC cohort B1 (*n* = 60). The APOBEC3C expression level was quantified using qRT–PCR. The immune cells were counted manually, and taken the mean values of three high-power fields (20×). PDAC, pancreatic ductal adenocarcinoma; ssGSEA, single-sample gene set enrichment analysis; NK cells, natural killer cells; Tfh, follicular helper T cells; Tregs, regulatory T cells; *r*, correlation.

ESTIMATE and CIBERSORT analyses revealed that APOBEC3C was correlated with PDAC stromal modification and immune cell distribution in TCGA dataset (Fig. 6A, *r* = 0.364 and 0.404, respectively, ESTIMATE score *r* = 0.415, *p* < 0.001). Activated CD4 + T cells and CTLs were marginally positively correlated with APOBEC3C expression (relevance = 0.397 and 0.230, respectively, *p* < 0.05), while natural killer cells and M0 macrophages were marginally negatively correlated with APOBEC3C expression (relevance = –0.309 and –0.251, respectively, *p* < 0.05).

We then counted the numbers of infiltrated CD4 + T cells and CD8 + T cells in PDAC stroma and correlated them with APOBEC3C expression in FUSCC cohort B1 (*n* = 60, Fig. 6B–E). APOBEC3C expression marginally correlated with CD4 + T cell counts (*r* = 0.2566, *p* = 0.0478) and CD8 + T cell counts (*r* = 0.2973, *p* = 0.0211).

## Discussion

The dismal prognosis of PDAC has not improved for decades, which might largely be attributed to therapeutic resistance and ubiquitous recurrence [46]. Notably, PDAC features high intratumor heterogeneity [5, 47], which accounts for its phenotypes development and adaptation to evolutionary pressure [48]. Patients have an increased mutational burden after first-line treatment [49], and the heterogeneous polyclonality of PDAC contributes to metastatic progression [50]. Accordingly, investigations of the factors resulting in PDAC genomic instability and promoting PDAC heterogeneity are urgently needed.

Remarkably, APOBEC3, along with MMR, promotes focal hypermutation in the tumour genome [51]. We analysed TCGA and FUSCC cohort A, and mutational signatures of the two cohorts coincidently showed a predominance of C > T mutations. Moreover, C > T mutations more frequently occurred in APOBEC3 preferred motifs, indicating that APOBEC3 participates in modelling mutational features in the PDAC genome. We located characteristic kataegis regions and correlated the count of C > X mutation-enriched kataegis regions to APOBEC3C mRNA expression level. We compared the high APOBEC3C expression subgroup and the low APOBEC3C expression subgroup and speculated that kataegis located within *PCSK5* and *NES* genes were fuelled by APOBEC3C. Vector transfected APOBEC3C overexpression in cell lines also resulted in altered SNV distribution and new kataegis occurrence. The alteration of SNV distributions in diploid cell lines met our expectation, whereas it did not in the hypotriploid cell line (Capan-1). The seemingly paradoxical results may be attributed to the comprehensive effects of multiple mutagens, such as ADAR, POLE and POLH (Fig. S5C–H).

Notably, ssDNA is more vulnerable to DNA-modifying enzymes that cause clustered DNA damage, thus forming kataegis regions [52]; hence, the kataegis location reflects the ssDNA region, which is more likely to be translated or modified. Kataegis is not only observable evidence of hypermutagenic factors but is also associated with structural or functional alterations in the genome. Previous studies have reported that kataegis stabilises the expression of neighbouring genes [41] and colocalizes with genomic rearrangements [20] or is accompanied by chromothripsis [53].

Furthermore, this research revealed that APOBEC3C is the predominant APOBEC enzyme expressed in PDAC and that universal ectopic expression of APOBEC3C occurs in PDAC. We established survival curves showing that universally elevated APOBEC3C expression was related to shorter OS of PDAC patients. High APOBEC3C expression contributes to tumour plasticity; thus, tumours are able to adapt more easily to evolutionary pressure, such as chemotherapy [54], and are more prone to developing new phenotypes for recurrence or metastasis, which account for the worse prognosis.

Notably, factors involved in inducing genomic instability are also promising actionable targets for precise therapy in PDAC, such as *BRCA* mutations [55, 56] and dMMR [57, 58]. A real-world study revealed that patients with PDAC receiving matched precise therapy (including PARP inhibitors and PD-1 inhibitors) experienced prolonged survival [59], which reinforces the importance of identifying precise therapeutic targets to overcome the low survival rate of patients with PDAC. Regarding APOBEC, its increased TMB also suggests the potential for immune therapy [60–62]. APOBEC-induced genomic instability nourishes neoantigens, which are promising targets for immune cells to recognise and attack tumour cells. Our study revealed a positive correlation between APOBEC3C expression and the invasion of activated CD4 + T cells and CTLs, which are major effector lymphocytes involved in antitumour immune processes. Therefore, APOBEC3C expression denotes enhanced immune activity in the PDAC TME and predicts immunotherapy responses of PDAC. A previous pancancer analysis also revealed that APOBEC and kataegis are associated with programmed death-ligand 1/2 [63], and the potential of APOBEC for guiding immunotherapy has been affirmed in other tumours [64, 65]. In addition, ongoing clinical trials are investigating the practicality of APOBEC in guiding precise tumour therapy [66], such as NCT02576444 (olaparib, phase II) and NCT03989089 (pembrolizumab, phase II). Further studies on the application of APOBEC in PDAC immunotherapy are anticipated.

Given the importance of APOBEC for guiding immunotherapy, the potential to inhibit APOBEC family members for the purpose of limiting tumour adaptation also exists [19]. However, APOBEC has numerous isoforms, and each single isoform should be evaluated thoroughly to ensure the effect of inhibitors. The APOBEC expression burden may also be applied as an indicator for the risk of developing cancerous disease in the future. Moreover, APOBECs are promising base editing tools and probably have therapeutic implications in gene therapy [67].

In conclusion, APOBEC3C, a mutagenic driver, plays a dual role in PDAC treatment. On the one hand, its induction of genomic instability supplements tumour heterogeneity and evolution; on the other hand, it is a promising target for precise therapy.

## Supplementary information

Supplementary information

Reference Mutational Signature

Reference Mutational Signature

Reference Mutational Signature

Reference Mutational Signature

Reference Mutational Signature

Reference Mutational Signature

Reference Mutational Signature

Reference Mutational Signature

Reference Mutational Signature

Reference Mutational Signature

FUSCC cohort patient ID list

FUSCC cohort A transcriptomics data

Survival data of FUSCC cohort A

FUSCC kataegis stratified by A3C expression

FUSCC cohort A SNP

FUSCC cohort A SNP summary

FUSCC cohort A trinucleotide motif information

FUSCC cohort B1 immune cell counts

TCGA PAAD maf file

TCGA-SNP

TCGA PDAC APOBEC and OS

GEPIA sample list

CCLE APOBEC expression

QCMG PDAC APOBEC and OS

Cibersort and ESTIMATE output

Motif enrichment analysis

Sanger sequencing results.

Capan-1 A3C newly occured SNVs

H6c7 A3C newly occured SNVs

SU.86.86-A3C newly occured SNVs

## Data Availability

The datasets used and analysed during this study available from the corresponding author on reasonable request.
