# Supplementary material for: Aberrant APOBEC3C expression induces characteristic genomic instability in pancreatic ductal adenocarcinoma
Source: Oncogenesis. 2022 Jun 24;11(1):35. doi: 10.1038/s41389-022-00411-9 (PMC9232547; doi:10.1038/s41389-022-00411-9)
Supplement: Supplementary file 1 — Supplementary information [file 41389_2022_411_MOESM1_ESM.pdf]

Supplementary materials and methods

Next-generation sequencing

WES and transcriptome analyses were performed using the DNBSEQ platform (BGI Tech company, China), including 3 A3C-overexpressing pancreatic cell lines and matched controls, 124 specimens from patients with PDAC and matched blood samples. Reference genomes were chosen according to the instructions provided by BGI Tech Company (GRCh37/hg19 for cell lines and GRCh38/hg38 for tissue samples). Burrows–Wheeler Aligner (v 0.7.15) was used to align clean reads to the reference genome; the average mapping ratio with the reference genome was 88.89%, and the mean sequencing depth was 222×. Bam-matcher[1] was applied to ascertain that the matched sequencing samples had the same source, GATK(v 3.7) HaplotypeCaller was used to detect mutations, SnpEff was used to annotate variants, and 92.80% of identified variants were reported in dbSNP v141. The RSEM method was applied for isoform quantification. Sequence data are shown in the supplementary information, and were also uploaded to BGI company's database.

Protein extraction and western blot analysis

Cells were washed with ice-cold PBS and lysed in RIPA buffer for 10 minutes. Cell debris were removed by centrifugation at 12,000 rpm for 20 minutes at 4°C. A BCA Protein Quantification Kit (Cat# 20201ES76, Yeason) was used to quantify the protein concentrations, and different volumes of RIPA buffer were added to each sample to obtain equal final protein concentrations. Then, 10 μL of each protein lysate sample were denatured and electrophoretically separated on a 10% SDS polyacrylamide gel. Separated proteins were transferred to a membrane and subsequently blotted with the APOBEC3C antibody (10591-1-AP). The efficiency of 10591-1-AP for staining APOBEC3C was validated by rabbit anti-HA antibody (Cat#C29F4, Cell signaling)[2]. An antibody against ACTB was used for blotting as

as the internal control (HRP-conjugated monoclonal antibody against ACTB, Cat# HRP-60008, Proteintech).

Reference

1. Wang PPS, Parker WT, Branford S, Schreiber AW. BAM-matcher: A tool for rapid NGS sample matching. *Bioinformatics*. 2016;32(17):2699-2701.

2. Anderson BD, Ikeda T, Moghadasi SA, Martin AS, Brown WL, Harris RS. Natural APOBEC3C variants can elicit differential HIV-1 restriction activity. *Retrovirology*. 2018;15(1):78.

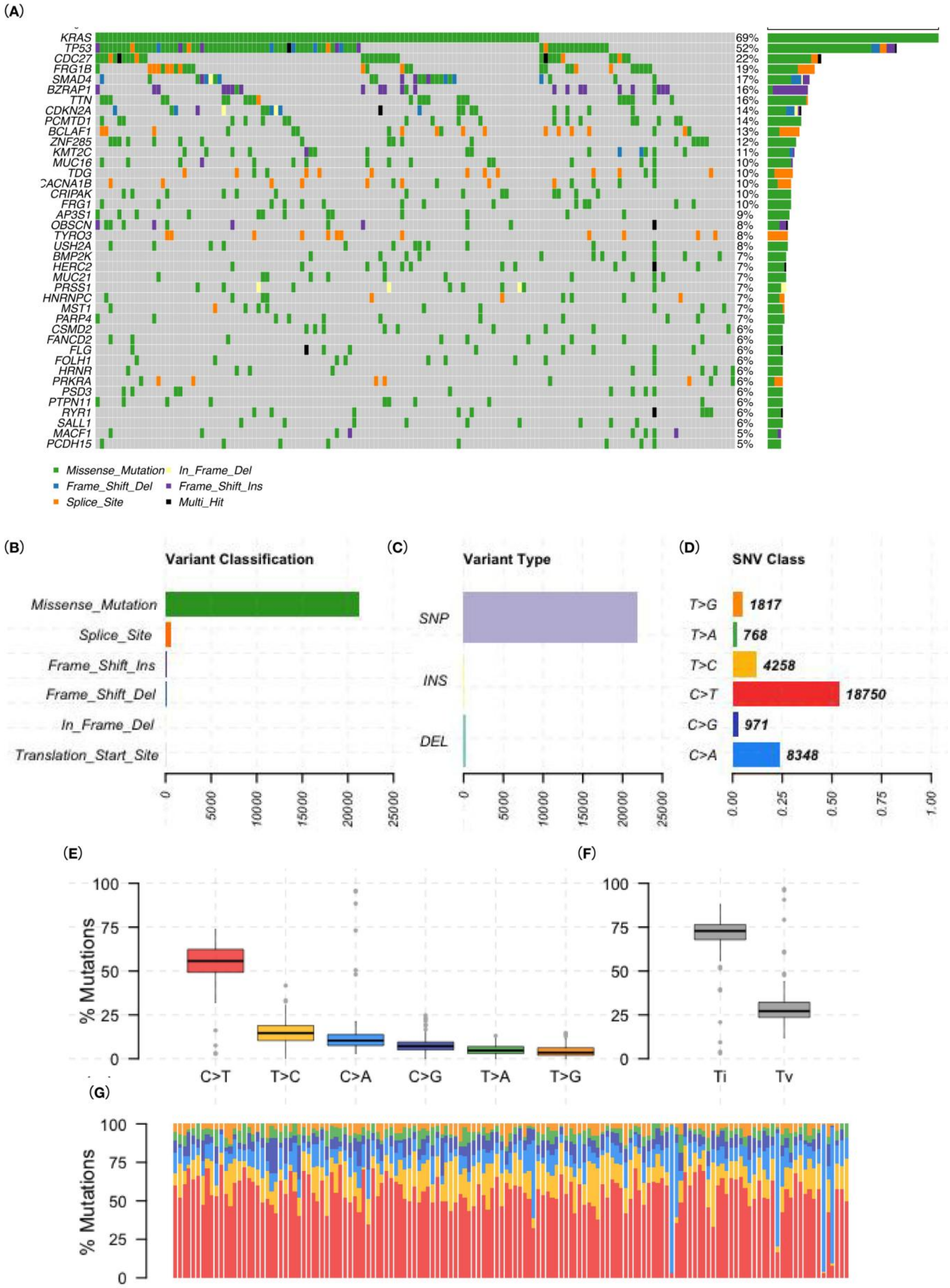

Supplementary Figure S1. Mutational features of TCGA PDAC cohort.

Maftools was used to analyse bcgsc.ca_PAAD.IlluminaHiSeq_DNASeq.1.somatic.maf (n =

147) and outputted the results listed below.

A: Waterfall plot showing the top 40 most frequently mutated genes in TCGA cohort. *KRAS* and *TP53* alterations were the most common genomic mutational events in PDAC, and more than 80% of patients with PDAC harboured *KRAS* or *TP53* mutations. The most common SNV of *KRAS* was reported to be rs121913529 (allele: C>A, G, T), and the most common SNV of *TP53* was rs28934578 (allele: C>A, T).

B, C, D: Classification of mutations. Missense mutation was the major variant type (B), SNV was more common than INS and DEL (C), and C>T mutation was the primary SNV type (D).

E, F: Overall distribution of six types of SNVs. Transition (Ti, C>T, T>C, A>G, or G>A) was more prevalent than transversion (Tv, the other types of base conversion).

G: Stacked bar plot showing the fraction of conversions in each sample.

Abbreviations: SNV, single nucleotide variant; INS, insertion; DEL, deletion.

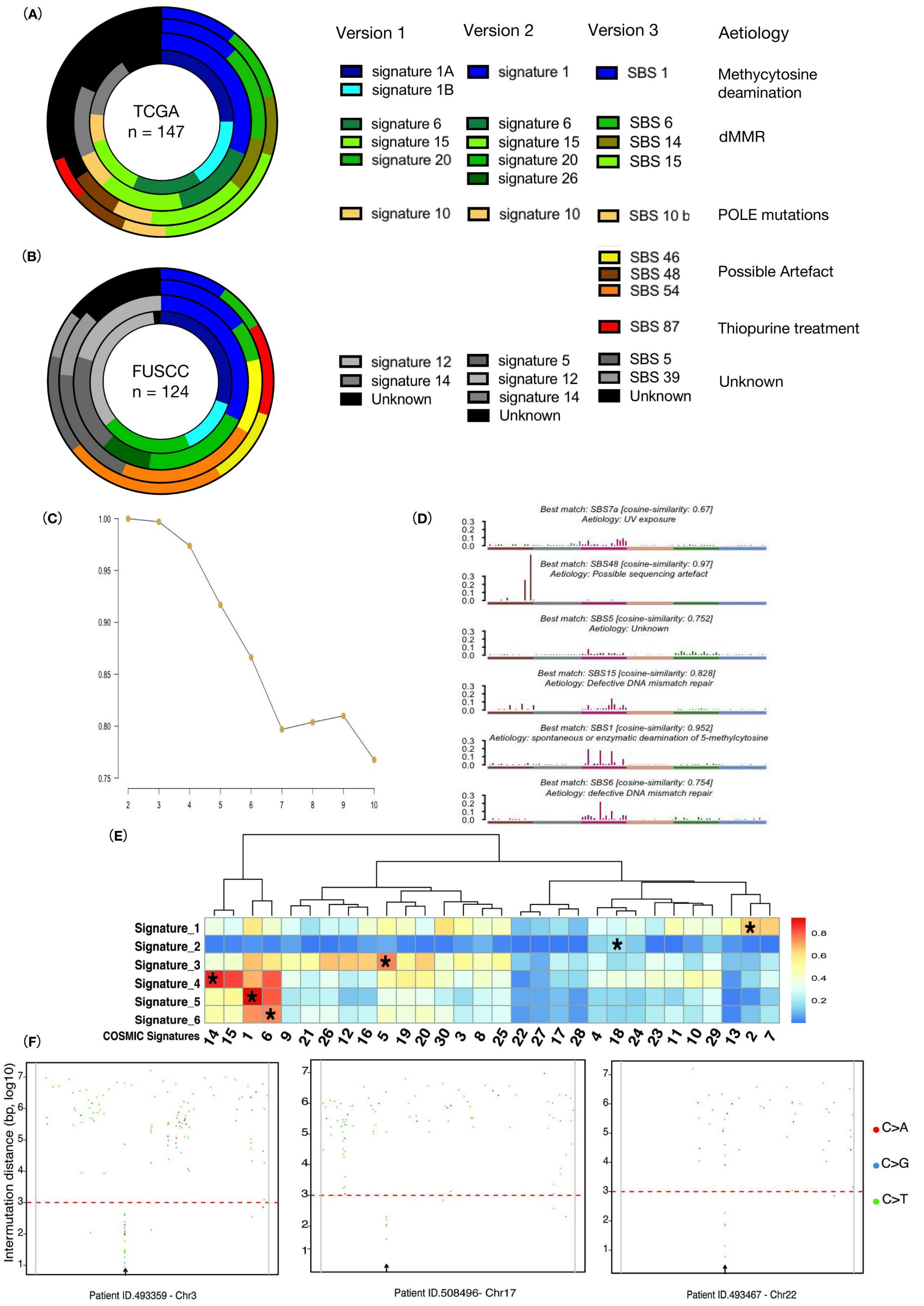

Supplementary Figure S2. Mutational signatures compared to the classical reference

and characterized kataegis.

A, B: DeconstrutSigs compared earlier COSMIC reference mutational signatures and extracted mutational signatures from TCGA PDAC cohort (A, n= 147) and FUSCC cohort A (B, n = 124). Rings from the inner to the outer represent COSMIC reference mutational signature version 1, version 2, version 3.0 and version 3.1, respectively.

C: The optimal counts of mutational signatures comprising TCGA PDAC genomic alteration characteristics were measured by calculating the cophenetic correlation coefficient. The most faithful signature count was the value at which cophenetic correlation decreased significantly (n = 6).

D: Maftools compared deconstructed mutational signatures (NMF method) from TCGA PDAC genome and reference mutational signatures (COSMIC version 3.0, May 2019). SBS1 is associated with spontaneous or enzymatic deamination of 5-methylcytosine (age- or APOBEC-induced), SBS6 and SBS15 are associated with DNA MMR deficiency, and SBS7a is associated with UV exposure.

E: Maftools compared deconstructed mutational signatures (NMF method) from TCGA PDAC genome and reference mutational signatures (COSMIC version 1, August 2013). The cosine similarity between signature 1 and COSMIC signature 2 was 0.665, that between signature 2 and COSMIC signature 18 was 0.176, that between signature 3 and COSMIC signature 5 was 0.761, that between signature 4 and COSMIC signature 14 was 0.885, that between signature 5 and COSMIC signature 1 was 0.941, and that between signature 6 and COSMIC signature 6 was 0.749.

F: Representative rainfall plots showing that kataegis completely consisted of TpC to TpX mutations within the *MIR4273* (patient ID: 493359, chromosome 3), *GGTLC2* (patient ID: 493467, chromosome 22) and *PCDH11X* (patient ID: 624549, chromosome X) genes in FUSCC cohort A.

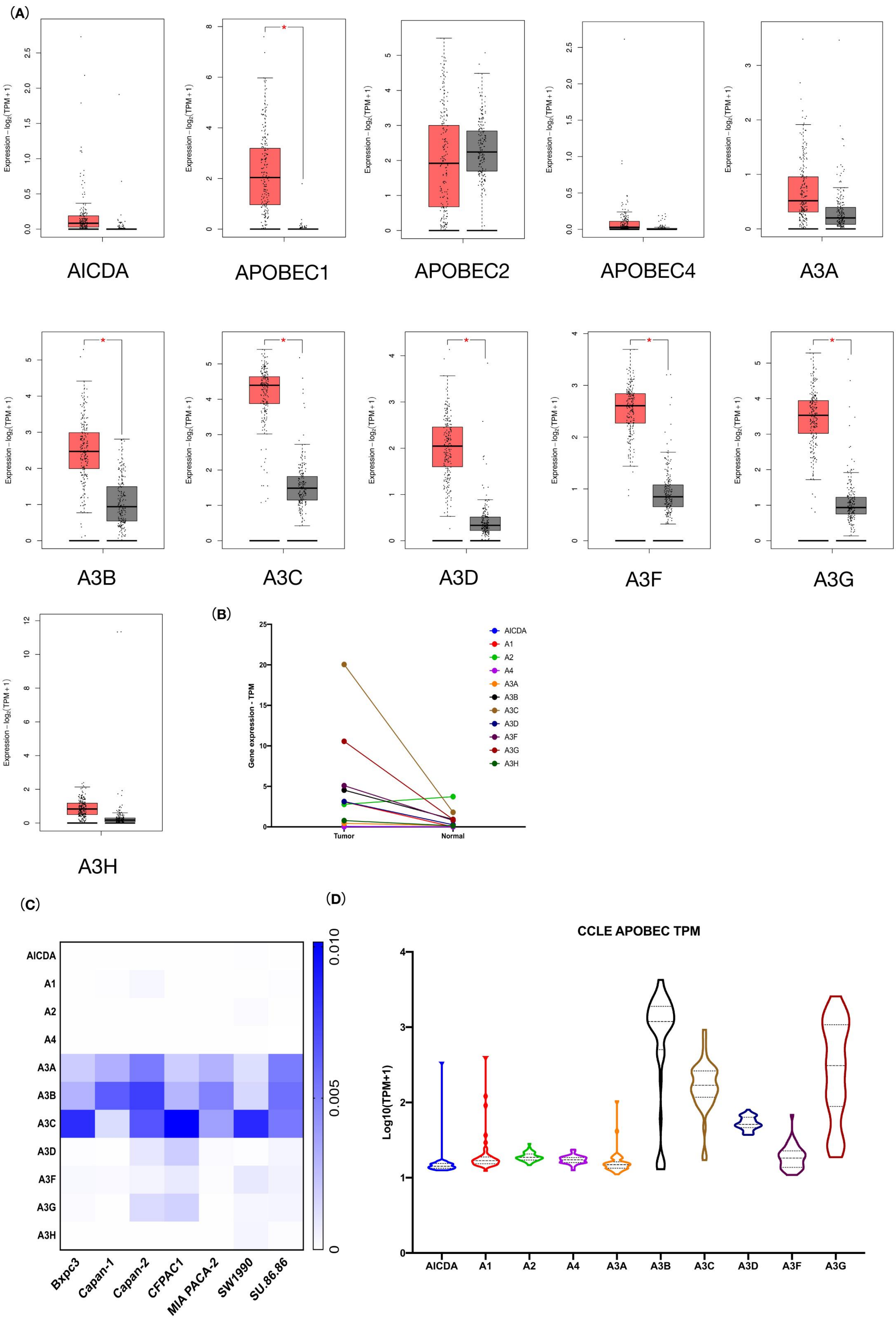

Supplementary Figure S3. APOBEC expression in pancreatic tissue and cell lines.

A: Boxplots comparing APOBEC mRNA expression between PDAC and normal pancreatic tissues were generated using the GEPIA2 website. One-way ANOVA was used to test the statistical significance. The red box represents the mRNA expression level in PDAC (TCGA database, n = 163), and the grey box represents the mRNA expression level in normal pancreatic tissue (TCGA and GTEx databases, n = 171). *: p value < 0.01.

B: Summary of the median mRNA expression levels of APOBECs in PDAC and normal pancreatic tissues. The original data were the same as those shown in Figure 2 E and Figure S3 A.

C: Heatmap showing the expression of APOBECs in FUSCC PDAC cell lines. The original data were the same as those shown in Figure 2 F.

D: Summary of APOBEC expression in pancreatic cancer cell lines. The expression data were sourced from the Cancer Cell Line Encyclopaedia database (CCLE).

Abbreviations: PDAC, pancreatic ductal adenocarcinoma.

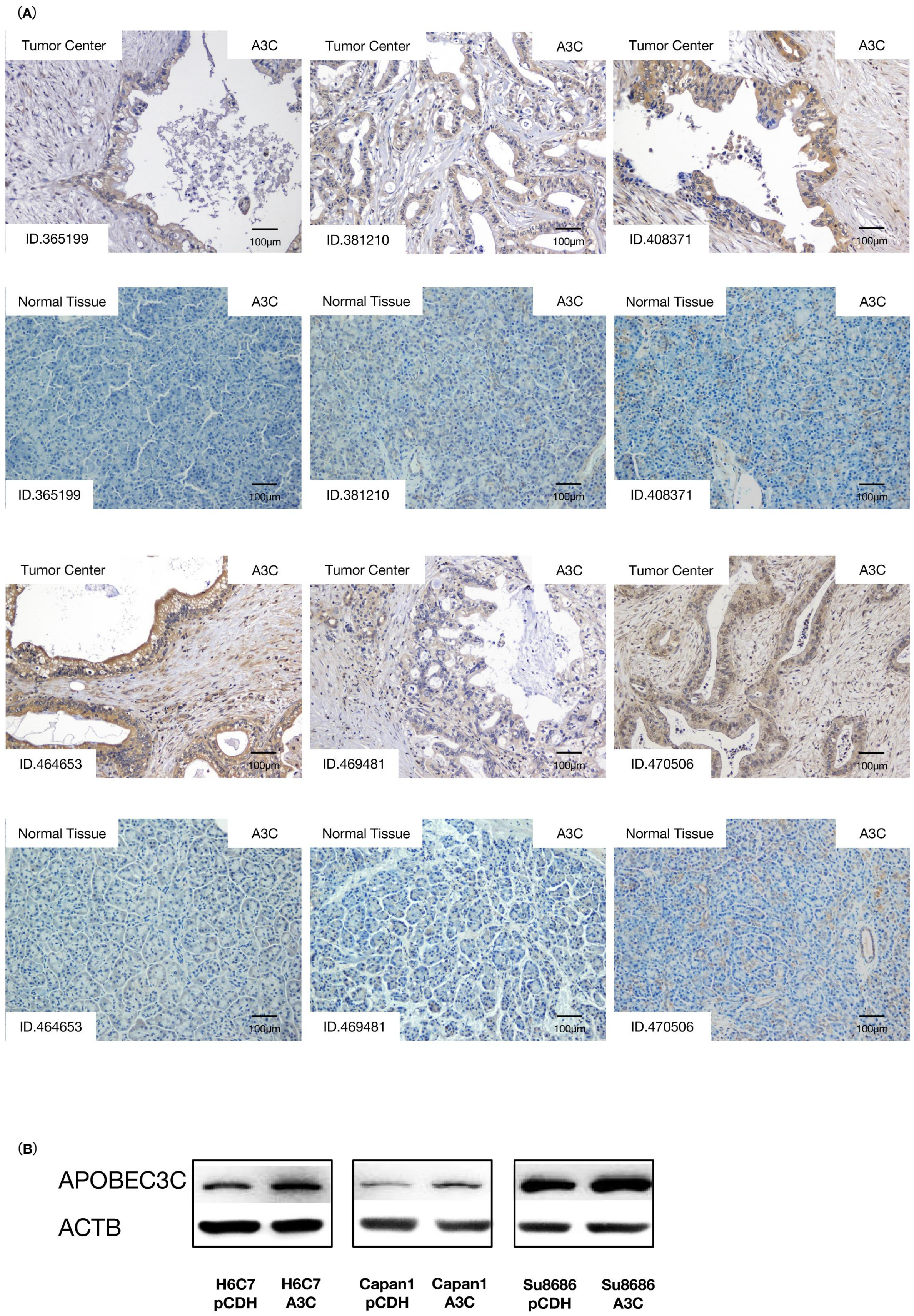

Supplementary Figure S4. Comparison of APOBEC3C expression at the protein level.

A: IHC staining showing that APOBEC3C was much more abundant in PDAC than in

normal pancreatic tissues (FUSCC cohort B1).

B: Western blot showing that APOBEC3C levels were increased in H6c7 A3C, Capan-1 A3C, and SU.86.86 A3C cells compared to the corresponding control cell lines.

Abbreviations: PDAC, pancreatic ductal adenocarcinoma; ACTB, β-actin; A3C, APOBEC3C.

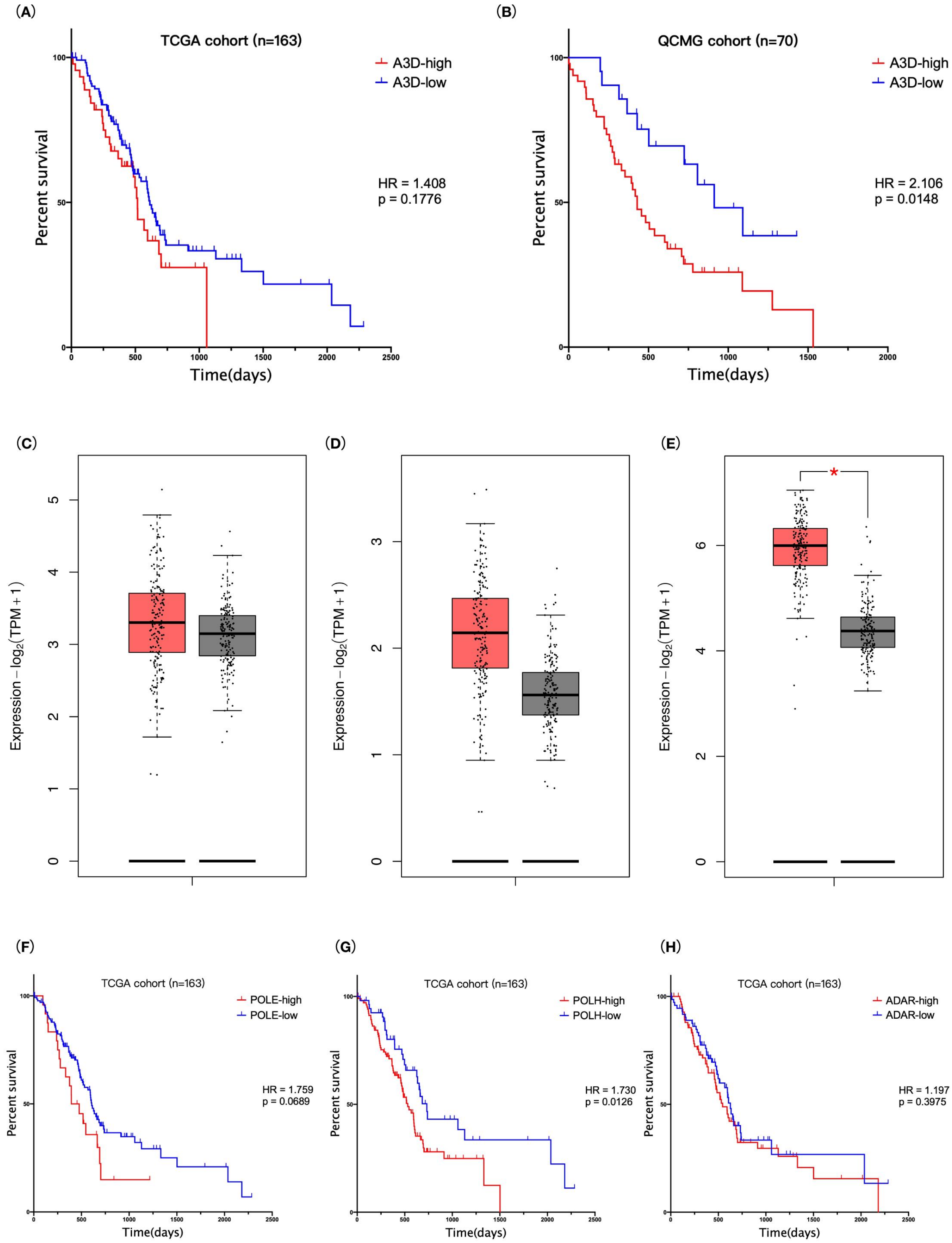

Supplementary Figure S5. Additional analysis of other mutagenic drivers in PDAC.

Mantel–Cox test was used to compare Kaplan–Meier survival curves.

A: A cut-off value of 230.68 TPM stratified TCGA PDAC cohort into the high APOBEC3D expression subgroup (n = 46) and the low APOBEC3D expression subgroup (n = 117), with a trend towards a difference in OS.

B: A cut-off value of 69.31 TPM stratified the QCMG PDAC cohort into the high APOBEC3D expression subgroup (n = 49) and the low APOBEC3D expression subgroup (n = 21) with a difference in OS.

C, D, E: Boxplots comparing the expression of *POLE* (C), and *POLH* (D) and *ADAR* (E) between PDAC and normal pancreatic tissues. One-way ANOVA was used to test the statistical significance. The red box represents the mRNA expression level in PDAC (TCGA database, n = 163), and the grey box represents the mRNA expression level in normal pancreatic tissues (TCGA and GTEx databases, n = 171). *: p value < 0.01.

F: TCGA PDAC cohort were stratified into POLE high expression subgroup (n = 24) and POLE low expression subgroup (n = 139) with an overall survival difference tendency.

G: TCGA PDAC cohort were stratified into POLH high expression subgroup (n = 107) and POLH low expression subgroup (n = 56) with an overall survival difference.

H: TCGA PDAC cohort were stratified into ADAR high expression subgroup (n = 86) and ADAR low expression subgroup (n = 77) with an overall survival difference tendency.

Abbreviations: PDAC, pancreatic ductal adenocarcinoma; A3D, APOBEC3D; HR, hazard ratio; ADAR, adenosine deaminase acting on RNA; POLE, DNA polymerase epsilon; POLH, DNA polymerase eta.

Table S1. Primers used for qRT-PCR in this study.
F: forward, R: reverse

Gene name	Serial number	Primer sequence
ACTB	1.F	5-CGTGCGTGACATTAAGGAAGAGT-3
	1.R	5-GGAAGGAAGGCTGGAAGAGT-3
AICDA	1.F	5-CGTAGTGAAGAGGCGTGACA-3
	1.R	5-ATGTAGCGGAGGAAGAGCAAT-3
APOBEC1	1.F	5-CCTGGTTCTTGTCCTGGAGT-3
	1.R	5-CCCTGAGACCTTGCCGATT-3
APOBEC2	1.F	5-AACCTGCGTCTGCTCATTCT-3
	1.R	5-GTCTGCCAACTTCTCCTCGTA-3
APOBEC3A	1.F	5-ATAAGACCTACCTGTGCTACGA-3
	1.R	5-TCCAGGAGATGAACCAAGTGA-3
APOBEC3B	1.F	5-CCAGACCTACTTGTGCTATGAG-3
	1.R	5-GGCTCCAGGAGATGAACCA-3
APOBEC3C	1.F	5-TGGTTCTGCGACGACATACT-3
	1.R	5-GGCGGTGAAGATGGTGAGA-3
	2.F	5-TCCAGGCACATTCTACTTCCA-3
	2.R	5-TGTCGTCGCAGAACCAAGA-3
	3.F	5-TCACCTGGTACACATCTTGGA-3
	3.R	5-AGGCTTGAATGGCTCATTATCA-3
APOBEC3D/3E	1.F	5-ACCGACTGCCTGCTAACA-3
	1.R	5-CCACCGCCAATCTCTATCC-3
APOBEC3F	1.F	5-AAGCACCACTCACCTGTCTC-3
	1.R	5-TGTCGTCACAGAACCAAGAGA-3
APOBEC3G	1.F	5-GCATCGTGACCAGGAGTATGA-3
	1.R	5-GTAGTAGAGGCGGGCAACAA-3
APOBEC3H	1.F	5-GTTTGCTGACTGCTGGGAAA-3
	1.R	5-CGCTTTATGGCTCGACTGTT-3
APOBEC4	1.F	5-GAACAGGTGAAGAAGCAAGAGT-3
	1.R	5-ACCAGGCTACCAGAAGAAGTT-3

Table S2. Differentially altered genes in relation to APOBEC enrichment score in TCGA PDAC cohort. Samples with motif enrichment score of 5'-TCA-3' > 2 were considered as APOBEC enriched.
CI: confidence interval of odds ratio.

Gene	Mutated samples		p value	Odds ratio	95%CI.LOW	95%CI.UP
	APOBEC enriched group	non-APOBEC enriched group				
COL24A1	2	2	0.008	31.530	1.870	542.830
MDN1	2	3	0.013	21.367	1.427	252.877
ADAMTS16	2	4	0.019	16.147	1.151	163.940
GRM1	2	4	0.019	16.147	1.151	163.940
PCDH15	2	6	0.035	10.795	0.826	95.646
FANCD2	2	7	0.044	9.236	0.721	78.931
HNRNPC	2	8	0.054	8.057	0.640	66.992
PRSS1	2	9	0.065	7.133	0.573	58.275
MUC16	2	13	0.115	4.833	0.401	37.675
ACVR2A	1	2	0.118	13.154	0.197	294.972
ADAMTS3	1	2	0.118	13.154	0.197	294.972
ADAMTS7	1	2	0.118	13.154	0.197	294.972
ADAMTSL3	1	2	0.118	13.154	0.197	294.972
AGBL2	1	2	0.118	13.154	0.197	294.972
ARMC12	1	2	0.118	13.154	0.197	294.972
ATP2B2	1	2	0.118	13.154	0.197	294.972
ATXN2L	1	2	0.118	13.154	0.197	294.972
BAI2	1	2	0.118	13.154	0.197	294.972
C10orf71	1	2	0.118	13.154	0.197	294.972
CACNA1I	1	2	0.118	13.154	0.197	294.972
CHL1	1	2	0.118	13.154	0.197	294.972
CIC	1	2	0.118	13.154	0.197	294.972
CLK2	1	2	0.118	13.154	0.197	294.972
CNTNAP4	1	2	0.118	13.154	0.197	294.972
COG3	1	2	0.118	13.154	0.197	294.972
COL3A1	1	2	0.118	13.154	0.197	294.972
COL4A5	1	2	0.118	13.154	0.197	294.972
CTNNB1	1	2	0.118	13.154	0.197	294.972
DCHS1	1	2	0.118	13.154	0.197	294.972
DDX31	1	2	0.118	13.154	0.197	294.972
DLEC1	1	2	0.118	13.154	0.197	294.972
DUOXA2	1	2	0.118	13.154	0.197	294.972
EIF2AK2	1	2	0.118	13.154	0.197	294.972
FAM135B	1	2	0.118	13.154	0.197	294.972
FAM183B	1	2	0.118	13.154	0.197	294.972
FBXO40	1	2	0.118	13.154	0.197	294.972
GABRB1	1	2	0.118	13.154	0.197	294.972
GAK	1	2	0.118	13.154	0.197	294.972
GIF	1	2	0.118	13.154	0.197	294.972
IFT122	1	2	0.118	13.154	0.197	294.972
INPP5F	1	2	0.118	13.154	0.197	294.972
KCNA5	1	2	0.118	13.154	0.197	294.972
KCNB1	1	2	0.118	13.154	0.197	294.972
KDM1A	1	2	0.118	13.154	0.197	294.972
KIF1B	1	2	0.118	13.154	0.197	294.972
KLHL38	1	2	0.118	13.154	0.197	294.972
KMT2E	1	2	0.118	13.154	0.197	294.972
LILRB1	1	2	0.118	13.154	0.197	294.972
LRCH1	1	2	0.118	13.154	0.197	294.972
MAP7D3	1	2	0.118	13.154	0.197	294.972
MST1R	1	2	0.118	13.154	0.197	294.972
NLGN3	1	2	0.118	13.154	0.197	294.972
NT5C3B	1	2	0.118	13.154	0.197	294.972

OR8B12	1	2	0.118	13.154	0.197	294.972
PARD3B	1	2	0.118	13.154	0.197	294.972
PHKB	1	2	0.118	13.154	0.197	294.972
PKD1L1	1	2	0.118	13.154	0.197	294.972
PRKDC	1	2	0.118	13.154	0.197	294.972
PRL	1	2	0.118	13.154	0.197	294.972
PTH2	1	2	0.118	13.154	0.197	294.972
PTPRD	1	2	0.118	13.154	0.197	294.972
SECISBP2L	1	2	0.118	13.154	0.197	294.972
SEZ6L	1	2	0.118	13.154	0.197	294.972
SLC2A9	1	2	0.118	13.154	0.197	294.972
SRGAP1	1	2	0.118	13.154	0.197	294.972
STK10	1	2	0.118	13.154	0.197	294.972
TBC1D32	1	2	0.118	13.154	0.197	294.972
TRIOBP	1	2	0.118	13.154	0.197	294.972
UNC50	1	2	0.118	13.154	0.197	294.972
XRN1	1	2	0.118	13.154	0.197	294.972
ZNF521	1	2	0.118	13.154	0.197	294.972
ZNF645	1	2	0.118	13.154	0.197	294.972
KMT2C	2	14	0.129	4.460	0.372	34.495
ABLIM1	1	3	0.155	8.849	0.146	136.689
ADNP	1	3	0.155	8.849	0.146	136.689
AQP7	1	3	0.155	8.849	0.146	136.689
ARID1A	1	3	0.155	8.849	0.146	136.689
BTN3A1	1	3	0.155	8.849	0.146	136.689
C1S	1	3	0.155	8.849	0.146	136.689
C7orf10	1	3	0.155	8.849	0.146	136.689
CDH11	1	3	0.155	8.849	0.146	136.689
CEP164	1	3	0.155	8.849	0.146	136.689
DOCK7	1	3	0.155	8.849	0.146	136.689
ECT2	1	3	0.155	8.849	0.146	136.689
ELMO1	1	3	0.155	8.849	0.146	136.689
HDAC9	1	3	0.155	8.849	0.146	136.689
HIVEP1	1	3	0.155	8.849	0.146	136.689
IGSF22	1	3	0.155	8.849	0.146	136.689
KCNQ3	1	3	0.155	8.849	0.146	136.689
KIF26B	1	3	0.155	8.849	0.146	136.689
KRTAP10-2	1	3	0.155	8.849	0.146	136.689
OTOP1	1	3	0.155	8.849	0.146	136.689
PIGQ	1	3	0.155	8.849	0.146	136.689
RIMS2	1	3	0.155	8.849	0.146	136.689
SCN1A	1	3	0.155	8.849	0.146	136.689
SVIL	1	3	0.155	8.849	0.146	136.689
THOC5	1	3	0.155	8.849	0.146	136.689
TMEM198	1	3	0.155	8.849	0.146	136.689
TRIM42	1	3	0.155	8.849	0.146	136.689
TULP4	1	3	0.155	8.849	0.146	136.689
UBR2	1	3	0.155	8.849	0.146	136.689
XYLT1	1	3	0.155	8.849	0.146	136.689
ZFYVE26	1	3	0.155	8.849	0.146	136.689
AFF3	1	4	0.190	6.651	0.116	87.695
AHNAK	1	4	0.190	6.651	0.116	87.695
ATP12A	1	4	0.190	6.651	0.116	87.695
CNTNAP5	1	4	0.190	6.651	0.116	87.695
COL6A2	1	4	0.190	6.651	0.116	87.695
DEK	1	4	0.190	6.651	0.116	87.695
DMBT1	1	4	0.190	6.651	0.116	87.695
HYDIN	1	4	0.190	6.651	0.116	87.695
KCNA6	1	4	0.190	6.651	0.116	87.695
MSLNL	1	4	0.190	6.651	0.116	87.695
NCKAP5	1	4	0.190	6.651	0.116	87.695
PCDH9	1	4	0.190	6.651	0.116	87.695
PLEKHH2	1	4	0.190	6.651	0.116	87.695
PREX1	1	4	0.190	6.651	0.116	87.695
STK31	1	4	0.190	6.651	0.116	87.695
THSD7B	1	4	0.190	6.651	0.116	87.695

TOX2	1	4	0.190	6.651	0.116	87.695
ZNF606	1	4	0.190	6.651	0.116	87.695
APOB	1	5	0.225	5.315	0.096	64.197
BRWD3	1	5	0.225	5.315	0.096	64.197
CSMD3	1	5	0.225	5.315	0.096	64.197
FLNA	1	5	0.225	5.315	0.096	64.197
FRG2	1	5	0.225	5.315	0.096	64.197
GREB1	1	5	0.225	5.315	0.096	64.197
MUC5B	1	5	0.225	5.315	0.096	64.197
TMEM132D	1	5	0.225	5.315	0.096	64.197
TTN	2	22	0.253	2.680	0.229	20.049
SPTA1	1	6	0.258	4.415	0.082	50.445
SYNE1	1	6	0.258	4.415	0.082	50.445
SYNE2	1	6	0.258	4.415	0.082	50.445
HECW2	1	7	0.289	3.769	0.071	41.489
PCLO	1	7	0.289	3.769	0.071	41.489
ZNF626	1	7	0.289	3.769	0.071	41.489
CSMD2	1	8	0.320	3.281	0.063	35.150
PSD3	1	8	0.320	3.281	0.063	35.150
FRG1B	2	26	0.321	2.197	0.189	16.288
KRAS	3	99	0.371	0.427	0.055	3.319
BMP2K	1	10	0.378	2.595	0.050	26.808
MUC21	1	10	0.378	2.595	0.050	26.808
TYRO3	1	11	0.406	2.345	0.046	23.902
USH2A	1	11	0.406	2.345	0.046	23.902
AP3S1	1	12	0.432	2.135	0.042	21.536
CACNA1B	1	13	0.457	1.958	0.039	19.570
CRIPAK	1	13	0.457	1.958	0.039	19.570
BCLAF1	1	18	0.571	1.363	0.027	13.242
CDKN2A	1	19	0.591	1.282	0.026	12.400
AC011841.1	0	6	1.000	0.000	0.000	24.041
ANKRD30A	0	6	1.000	0.000	0.000	24.041
ATP10A	0	6	1.000	0.000	0.000	24.041
BTBD11	0	6	1.000	0.000	0.000	24.041
CHEK2	0	6	1.000	0.000	0.000	24.041
CPAMD8	0	6	1.000	0.000	0.000	24.041
DNAJC13	0	6	1.000	0.000	0.000	24.041
FAT2	0	6	1.000	0.000	0.000	24.041
FN1	0	6	1.000	0.000	0.000	24.041
FRY	0	6	1.000	0.000	0.000	24.041
HLA-DQB2	0	6	1.000	0.000	0.000	24.041
HMCN1	0	6	1.000	0.000	0.000	24.041
KCNB2	0	6	1.000	0.000	0.000	24.041
KIAA1109	0	6	1.000	0.000	0.000	24.041
KIR2DL3	0	6	1.000	0.000	0.000	24.041
KRTAP4-9	0	6	1.000	0.000	0.000	24.041
LRRK1	0	6	1.000	0.000	0.000	24.041
MYO3A	0	6	1.000	0.000	0.000	24.041
MYO5B	0	6	1.000	0.000	0.000	24.041
PAPPA2	0	6	1.000	0.000	0.000	24.041
PCDHB7	0	6	1.000	0.000	0.000	24.041
PHIP	0	6	1.000	0.000	0.000	24.041
PRRC2A	0	6	1.000	0.000	0.000	24.041
PSG1	0	6	1.000	0.000	0.000	24.041
PTCHD2	0	6	1.000	0.000	0.000	24.041
RP1L1	0	6	1.000	0.000	0.000	24.041
RYR3	0	6	1.000	0.000	0.000	24.041
SCN9A	0	6	1.000	0.000	0.000	24.041
TBC1D29	0	6	1.000	0.000	0.000	24.041
TNXB	0	6	1.000	0.000	0.000	24.041
AADACL3	0	2	1.000	0.000	0.000	134.508
AADACL4	0	3	1.000	0.000	0.000	64.152
AAGAB	0	2	1.000	0.000	0.000	134.508
AASDH	0	3	1.000	0.000	0.000	64.152
AASS	0	3	1.000	0.000	0.000	64.152
ABCA1	0	3	1.000	0.000	0.000	64.152

ABCA12	0	4	1.000	0.000	0.000	41.489
ABCA13	0	5	1.000	0.000	0.000	30.501
ABCA4	0	2	1.000	0.000	0.000	134.508
ABCA5	0	2	1.000	0.000	0.000	134.508
ABCA6	0	2	1.000	0.000	0.000	134.508
ABCA9	0	2	1.000	0.000	0.000	134.508
ABCB1	0	4	1.000	0.000	0.000	41.489
ABCB11	0	3	1.000	0.000	0.000	64.152
ABCB4	0	2	1.000	0.000	0.000	134.508
ABCB5	0	5	1.000	0.000	0.000	30.501
ABCB6	0	3	1.000	0.000	0.000	64.152
ABCC1	0	2	1.000	0.000	0.000	134.508
ABCC10	0	2	1.000	0.000	0.000	134.508
ABCC11	0	2	1.000	0.000	0.000	134.508
ABCC4	0	2	1.000	0.000	0.000	134.508
ABCC6	0	2	1.000	0.000	0.000	134.508
ABCC9	0	3	1.000	0.000	0.000	64.152
ABCD3	0	2	1.000	0.000	0.000	134.508
ABCG1	0	2	1.000	0.000	0.000	134.508
ABCG4	0	2	1.000	0.000	0.000	134.508
ABCG8	0	3	1.000	0.000	0.000	64.152
ABR	0	2	1.000	0.000	0.000	134.508
ABRA	0	2	1.000	0.000	0.000	134.508
AC003682.1	0	2	1.000	0.000	0.000	134.508
AC069154.2	0	3	1.000	0.000	0.000	64.152
AC131180.1	0	3	1.000	0.000	0.000	64.152
ACACA	0	2	1.000	0.000	0.000	134.508
ACACB	0	2	1.000	0.000	0.000	134.508
ACAD10	0	2	1.000	0.000	0.000	134.508
ACAN	0	3	1.000	0.000	0.000	64.152
ACAP1	0	3	1.000	0.000	0.000	64.152
ACBD3	0	2	1.000	0.000	0.000	134.508
ACCS	0	2	1.000	0.000	0.000	134.508
ACE	0	2	1.000	0.000	0.000	134.508
ACHE	0	2	1.000	0.000	0.000	134.508
ACIN1	0	2	1.000	0.000	0.000	134.508
ACLY	0	3	1.000	0.000	0.000	64.152
ACOT12	0	2	1.000	0.000	0.000	134.508
ACSF3	0	2	1.000	0.000	0.000	134.508
ACSL3	0	2	1.000	0.000	0.000	134.508
ACSL4	0	2	1.000	0.000	0.000	134.508
ACSL5	0	2	1.000	0.000	0.000	134.508
ACSM1	0	2	1.000	0.000	0.000	134.508
ACSM3	0	2	1.000	0.000	0.000	134.508
ACSS3	0	3	1.000	0.000	0.000	64.152
ACTA1	0	2	1.000	0.000	0.000	134.508
ACTC1	0	2	1.000	0.000	0.000	134.508
ACTG2	0	2	1.000	0.000	0.000	134.508
ACTL9	0	4	1.000	0.000	0.000	41.489
ADAD1	0	2	1.000	0.000	0.000	134.508
ADAM15	0	2	1.000	0.000	0.000	134.508
ADAM17	0	3	1.000	0.000	0.000	64.152
ADAM18	0	2	1.000	0.000	0.000	134.508
ADAM2	0	4	1.000	0.000	0.000	41.489
ADAM21	0	2	1.000	0.000	0.000	134.508
ADAM28	0	2	1.000	0.000	0.000	134.508
ADAM7	0	2	1.000	0.000	0.000	134.508
ADAMTS1	0	2	1.000	0.000	0.000	134.508
ADAMTS12	0	7	1.000	0.000	0.000	19.777
ADAMTS14	0	4	1.000	0.000	0.000	41.489
ADAMTS15	0	3	1.000	0.000	0.000	64.152
ADAMTS17	0	3	1.000	0.000	0.000	64.152
ADAMTS18	0	4	1.000	0.000	0.000	41.489
ADAMTS19	0	3	1.000	0.000	0.000	64.152
ADAMTS2	0	2	1.000	0.000	0.000	134.508
ADAMTS20	0	2	1.000	0.000	0.000	134.508

ADAMTS4	0	4	1.000	0.000	0.000	41.489
ADAMTS5	0	2	1.000	0.000	0.000	134.508
ADAMTS8	0	2	1.000	0.000	0.000	134.508
ADAMTS9	0	2	1.000	0.000	0.000	134.508
ADAMTSL1	0	2	1.000	0.000	0.000	134.508
ADAMTSL4	0	4	1.000	0.000	0.000	41.489
ADARB1	0	2	1.000	0.000	0.000	134.508
ADARB2	0	2	1.000	0.000	0.000	134.508
ADCY1	0	3	1.000	0.000	0.000	64.152
ADCY10	0	2	1.000	0.000	0.000	134.508
ADCY2	0	2	1.000	0.000	0.000	134.508
ADCY3	0	2	1.000	0.000	0.000	134.508
ADCY5	0	2	1.000	0.000	0.000	134.508
ADCY8	0	3	1.000	0.000	0.000	64.152
ADCY9	0	2	1.000	0.000	0.000	134.508
ADD3	0	2	1.000	0.000	0.000	134.508
ADORA2A	0	2	1.000	0.000	0.000	134.508
ADORA3	0	2	1.000	0.000	0.000	134.508
ADRA1A	0	2	1.000	0.000	0.000	134.508
ADRA2B	0	2	1.000	0.000	0.000	134.508
ADRBK2	0	2	1.000	0.000	0.000	134.508
AEBP1	0	2	1.000	0.000	0.000	134.508
AFF2	0	4	1.000	0.000	0.000	41.489
AGL	0	3	1.000	0.000	0.000	64.152
AGMO	0	2	1.000	0.000	0.000	134.508
AGR2	0	2	1.000	0.000	0.000	134.508
AGT	0	2	1.000	0.000	0.000	134.508
AGTPBP1	0	2	1.000	0.000	0.000	134.508
AGXT	0	2	1.000	0.000	0.000	134.508
AHCTF1	0	2	1.000	0.000	0.000	134.508
AHI1	0	2	1.000	0.000	0.000	134.508
AHNAK2	0	7	1.000	0.000	0.000	19.777
AHRR	0	4	1.000	0.000	0.000	41.489
AIM1	0	3	1.000	0.000	0.000	64.152
AJAP1	0	3	1.000	0.000	0.000	64.152
AK7	0	2	1.000	0.000	0.000	134.508
AKAP10	0	2	1.000	0.000	0.000	134.508
AKAP11	0	2	1.000	0.000	0.000	134.508
AKAP12	0	3	1.000	0.000	0.000	64.152
AKAP13	0	2	1.000	0.000	0.000	134.508
AKAP6	0	4	1.000	0.000	0.000	41.489
AKAP9	0	2	1.000	0.000	0.000	134.508
AKNAD1	0	3	1.000	0.000	0.000	64.152
AKR1E2	0	2	1.000	0.000	0.000	134.508
AKR7A3	0	3	1.000	0.000	0.000	64.152
AKT3	0	3	1.000	0.000	0.000	64.152
AL354822.1	0	2	1.000	0.000	0.000	134.508
AL360154.1	0	2	1.000	0.000	0.000	134.508
AL592183.1	0	5	1.000	0.000	0.000	30.501
ALAS2	0	3	1.000	0.000	0.000	64.152
ALCAM	0	2	1.000	0.000	0.000	134.508
ALDH16A1	0	3	1.000	0.000	0.000	64.152
ALDH18A1	0	2	1.000	0.000	0.000	134.508
ALDH1A3	0	2	1.000	0.000	0.000	134.508
ALDH1L1	0	2	1.000	0.000	0.000	134.508
ALDH1L2	0	2	1.000	0.000	0.000	134.508
ALDH3A1	0	2	1.000	0.000	0.000	134.508
ALDH4A1	0	3	1.000	0.000	0.000	64.152
ALDOB	0	2	1.000	0.000	0.000	134.508
ALG12	0	2	1.000	0.000	0.000	134.508
ALK	0	2	1.000	0.000	0.000	134.508
ALMS1	0	3	1.000	0.000	0.000	64.152
ALOX15	0	2	1.000	0.000	0.000	134.508
ALPK1	0	2	1.000	0.000	0.000	134.508
ALPK2	0	3	1.000	0.000	0.000	64.152
ALS2	0	3	1.000	0.000	0.000	64.152

ALS2CL	0	3	1.000	0.000	0.000	64.152
ALX4	0	2	1.000	0.000	0.000	134.508
AMBP	0	2	1.000	0.000	0.000	134.508
AMD1	0	2	1.000	0.000	0.000	134.508
AMER1	0	3	1.000	0.000	0.000	64.152
AMFR	0	4	1.000	0.000	0.000	41.489
AMPD1	0	2	1.000	0.000	0.000	134.508
AMPD3	0	2	1.000	0.000	0.000	134.508
AMZ2	0	2	1.000	0.000	0.000	134.508
ANAPC1	0	2	1.000	0.000	0.000	134.508
ANAPC2	0	2	1.000	0.000	0.000	134.508
ANK1	0	3	1.000	0.000	0.000	64.152
ANK2	0	4	1.000	0.000	0.000	41.489
ANK3	0	5	1.000	0.000	0.000	30.501
ANKAR	0	3	1.000	0.000	0.000	64.152
ANKDD1A	0	2	1.000	0.000	0.000	134.508
ANKEF1	0	2	1.000	0.000	0.000	134.508
ANKHD1	0	2	1.000	0.000	0.000	134.508
ANKRD12	0	3	1.000	0.000	0.000	64.152
ANKRD13C	0	2	1.000	0.000	0.000	134.508
ANKRD24	0	3	1.000	0.000	0.000	64.152
ANKRD27	0	2	1.000	0.000	0.000	134.508
ANKRD30B	0	2	1.000	0.000	0.000	134.508
ANKRD35	0	3	1.000	0.000	0.000	64.152
ANKRD50	0	2	1.000	0.000	0.000	134.508
ANKS1B	0	4	1.000	0.000	0.000	41.489
ANO3	0	3	1.000	0.000	0.000	64.152
ANO5	0	2	1.000	0.000	0.000	134.508
ANPEP	0	2	1.000	0.000	0.000	134.508
ANXA1	0	3	1.000	0.000	0.000	64.152
ANXA11	0	2	1.000	0.000	0.000	134.508
ANXA6	0	2	1.000	0.000	0.000	134.508
AOC3	0	8	1.000	0.000	0.000	16.757
AP3B1	0	3	1.000	0.000	0.000	64.152
AP4B1	0	2	1.000	0.000	0.000	134.508
APBA2	0	4	1.000	0.000	0.000	41.489
APC	0	3	1.000	0.000	0.000	64.152
APCDD1	0	2	1.000	0.000	0.000	134.508
APLF	0	2	1.000	0.000	0.000	134.508
APLP2	0	3	1.000	0.000	0.000	64.152
APOA4	0	2	1.000	0.000	0.000	134.508
APOBEC3B	0	2	1.000	0.000	0.000	134.508
APOH	0	2	1.000	0.000	0.000	134.508
APPBP2	0	2	1.000	0.000	0.000	134.508
AQR	0	2	1.000	0.000	0.000	134.508
AR	0	2	1.000	0.000	0.000	134.508
ARAF	0	2	1.000	0.000	0.000	134.508
ARAP1	0	2	1.000	0.000	0.000	134.508
ARCN1	0	3	1.000	0.000	0.000	64.152
AREL1	0	2	1.000	0.000	0.000	134.508
ARFIP2	0	2	1.000	0.000	0.000	134.508
ARG2	0	2	1.000	0.000	0.000	134.508
ARHGAP12	0	3	1.000	0.000	0.000	64.152
ARHGAP15	0	2	1.000	0.000	0.000	134.508
ARHGAP17	0	2	1.000	0.000	0.000	134.508
ARHGAP18	0	3	1.000	0.000	0.000	64.152
ARHGAP21	0	5	1.000	0.000	0.000	30.501
ARHGAP24	0	2	1.000	0.000	0.000	134.508
ARHGAP25	0	2	1.000	0.000	0.000	134.508
ARHGAP31	0	4	1.000	0.000	0.000	41.489
ARHGAP36	0	3	1.000	0.000	0.000	64.152
ARHGAP5	0	4	1.000	0.000	0.000	41.489
ARHGAP6	0	2	1.000	0.000	0.000	134.508
ARHGAP9	0	2	1.000	0.000	0.000	134.508
ARHGEF10	0	3	1.000	0.000	0.000	64.152
ARHGEF10L	0	2	1.000	0.000	0.000	134.508

ARHGEF11	0	2	1.000	0.000	0.000	134.508
ARHGEF12	0	2	1.000	0.000	0.000	134.508
ARHGEF17	0	3	1.000	0.000	0.000	64.152
ARHGEF37	0	2	1.000	0.000	0.000	134.508
ARID2	0	3	1.000	0.000	0.000	64.152
ARID3A	0	2	1.000	0.000	0.000	134.508
ARID4A	0	2	1.000	0.000	0.000	134.508
ARID4B	0	2	1.000	0.000	0.000	134.508
ARMC3	0	2	1.000	0.000	0.000	134.508
ARMC7	0	2	1.000	0.000	0.000	134.508
ARMCX1	0	2	1.000	0.000	0.000	134.508
ARPC5	0	2	1.000	0.000	0.000	134.508
ARRDC1	0	2	1.000	0.000	0.000	134.508
ARSI	0	2	1.000	0.000	0.000	134.508
ARSK	0	2	1.000	0.000	0.000	134.508
ASB15	0	4	1.000	0.000	0.000	41.489
ASB8	0	2	1.000	0.000	0.000	134.508
ASB9	0	2	1.000	0.000	0.000	134.508
ASCC3	0	2	1.000	0.000	0.000	134.508
ASGR2	0	2	1.000	0.000	0.000	134.508
ASH1L	0	3	1.000	0.000	0.000	64.152
ASH2L	0	2	1.000	0.000	0.000	134.508
ASIC1	0	2	1.000	0.000	0.000	134.508
ASIC3	0	2	1.000	0.000	0.000	134.508
ASMTL	0	2	1.000	0.000	0.000	134.508
ASPH	0	2	1.000	0.000	0.000	134.508
ASPM	0	5	1.000	0.000	0.000	30.501
ASPRV1	0	2	1.000	0.000	0.000	134.508
ASPSCR1	0	2	1.000	0.000	0.000	134.508
ASTN1	0	4	1.000	0.000	0.000	41.489
ASTN2	0	4	1.000	0.000	0.000	41.489
ASUN	0	2	1.000	0.000	0.000	134.508
ASXL3	0	2	1.000	0.000	0.000	134.508
ATAD2B	0	2	1.000	0.000	0.000	134.508
ATAD5	0	3	1.000	0.000	0.000	64.152
ATF1	0	2	1.000	0.000	0.000	134.508
ATF6B	0	2	1.000	0.000	0.000	134.508
ATF7IP2	0	2	1.000	0.000	0.000	134.508
ATG16L2	0	2	1.000	0.000	0.000	134.508
ATG2A	0	2	1.000	0.000	0.000	134.508
ATG2B	0	3	1.000	0.000	0.000	64.152
ATM	0	4	1.000	0.000	0.000	41.489
ATN1	0	2	1.000	0.000	0.000	134.508
ATP10B	0	3	1.000	0.000	0.000	64.152
ATP11A	0	2	1.000	0.000	0.000	134.508
ATP11B	0	4	1.000	0.000	0.000	41.489
ATP13A3	0	2	1.000	0.000	0.000	134.508
ATP13A5	0	2	1.000	0.000	0.000	134.508
ATP2A1	0	3	1.000	0.000	0.000	64.152
ATP2B1	0	2	1.000	0.000	0.000	134.508
ATP2C1	0	2	1.000	0.000	0.000	134.508
ATP2C2	0	2	1.000	0.000	0.000	134.508
ATP4A	0	2	1.000	0.000	0.000	134.508
ATP5G3	0	2	1.000	0.000	0.000	134.508
ATP6V0A4	0	2	1.000	0.000	0.000	134.508
ATP7A	0	2	1.000	0.000	0.000	134.508
ATP8A1	0	4	1.000	0.000	0.000	41.489
ATP8B2	0	2	1.000	0.000	0.000	134.508
ATP8B3	0	2	1.000	0.000	0.000	134.508
ATP8B4	0	3	1.000	0.000	0.000	64.152
ATP9A	0	2	1.000	0.000	0.000	134.508
ATR	0	3	1.000	0.000	0.000	64.152
ATRIP	0	2	1.000	0.000	0.000	134.508
ATRNL1	0	4	1.000	0.000	0.000	41.489
ATXN1	0	2	1.000	0.000	0.000	134.508
AUNIP	0	2	1.000	0.000	0.000	134.508

AURKA	0	2	1.000	0.000	0.000	134.508
AVL9	0	2	1.000	0.000	0.000	134.508
AVPR2	0	3	1.000	0.000	0.000	64.152
AXDND1	0	4	1.000	0.000	0.000	41.489
AXIN1	0	4	1.000	0.000	0.000	41.489
AXL	0	3	1.000	0.000	0.000	64.152
AZU1	0	2	1.000	0.000	0.000	134.508
B4GALNT4	0	2	1.000	0.000	0.000	134.508
BAALC	0	2	1.000	0.000	0.000	134.508
BACH1	0	3	1.000	0.000	0.000	64.152
BAHD1	0	2	1.000	0.000	0.000	134.508
BAI1	0	2	1.000	0.000	0.000	134.508
BANK1	0	2	1.000	0.000	0.000	134.508
BAZ1A	0	2	1.000	0.000	0.000	134.508
BAZ2B	0	2	1.000	0.000	0.000	134.508
BBS9	0	2	1.000	0.000	0.000	134.508
BCAM	0	2	1.000	0.000	0.000	134.508
BCAN	0	2	1.000	0.000	0.000	134.508
BCAT2	0	2	1.000	0.000	0.000	134.508
BCHE	0	3	1.000	0.000	0.000	64.152
BCL2L13	0	3	1.000	0.000	0.000	64.152
BCL6	0	3	1.000	0.000	0.000	64.152
BCO2	0	2	1.000	0.000	0.000	134.508
BCORL1	0	2	1.000	0.000	0.000	134.508
BDP1	0	2	1.000	0.000	0.000	134.508
BEND2	0	3	1.000	0.000	0.000	64.152
BEND3	0	2	1.000	0.000	0.000	134.508
BEND7	0	3	1.000	0.000	0.000	64.152
BEST1	0	3	1.000	0.000	0.000	64.152
BEST3	0	2	1.000	0.000	0.000	134.508
BFAR	0	3	1.000	0.000	0.000	64.152
BFSP2	0	3	1.000	0.000	0.000	64.152
BIRC6	0	3	1.000	0.000	0.000	64.152
BLNK	0	2	1.000	0.000	0.000	134.508
BMP1	0	3	1.000	0.000	0.000	64.152
BMP2	0	2	1.000	0.000	0.000	134.508
BMP5	0	2	1.000	0.000	0.000	134.508
BMPR1A	0	2	1.000	0.000	0.000	134.508
BMS1	0	5	1.000	0.000	0.000	30.501
BMX	0	2	1.000	0.000	0.000	134.508
BNC2	0	2	1.000	0.000	0.000	134.508
BOD1L1	0	3	1.000	0.000	0.000	64.152
BPIFB2	0	2	1.000	0.000	0.000	134.508
BPIFB6	0	2	1.000	0.000	0.000	134.508
BPNT1	0	2	1.000	0.000	0.000	134.508
BPTF	0	2	1.000	0.000	0.000	134.508
BRCA1	0	3	1.000	0.000	0.000	64.152
BRCA2	0	2	1.000	0.000	0.000	134.508
BRD4	0	2	1.000	0.000	0.000	134.508
BRF1	0	2	1.000	0.000	0.000	134.508
BRINP2	0	4	1.000	0.000	0.000	41.489
BRINP3	0	3	1.000	0.000	0.000	64.152
BRPF3	0	2	1.000	0.000	0.000	134.508
BRSK1	0	2	1.000	0.000	0.000	134.508
BRWD1	0	3	1.000	0.000	0.000	64.152
BSG	0	2	1.000	0.000	0.000	134.508
BTAF1	0	3	1.000	0.000	0.000	64.152
BTBD16	0	2	1.000	0.000	0.000	134.508
BTN1A1	0	2	1.000	0.000	0.000	134.508
BTNL2	0	2	1.000	0.000	0.000	134.508
BTNL8	0	2	1.000	0.000	0.000	134.508
BTRC	0	2	1.000	0.000	0.000	134.508
BUB1	0	2	1.000	0.000	0.000	134.508
BVES	0	2	1.000	0.000	0.000	134.508
BZRAP1	1	23	1.000	1.026	0.021	9.807
C10orf137	0	2	1.000	0.000	0.000	134.508

C10orf76	0	2	1.000	0.000	0.000	134.508
C10orf90	0	2	1.000	0.000	0.000	134.508
C11orf30	0	2	1.000	0.000	0.000	134.508
C11orf70	0	2	1.000	0.000	0.000	134.508
C14orf39	0	4	1.000	0.000	0.000	41.489
C16orf70	0	2	1.000	0.000	0.000	134.508
C16orf72	0	2	1.000	0.000	0.000	134.508
C17orf49	0	2	1.000	0.000	0.000	134.508
C17orf53	0	2	1.000	0.000	0.000	134.508
C17orf74	0	2	1.000	0.000	0.000	134.508
C17orf97	0	5	1.000	0.000	0.000	30.501
C18orf8	0	2	1.000	0.000	0.000	134.508
C19orf57	0	2	1.000	0.000	0.000	134.508
C1QTNF7	0	2	1.000	0.000	0.000	134.508
C1orf173	0	3	1.000	0.000	0.000	64.152
C1orf43	0	2	1.000	0.000	0.000	134.508
C1orf94	0	2	1.000	0.000	0.000	134.508
C20orf195	0	2	1.000	0.000	0.000	134.508
C2CD5	0	2	1.000	0.000	0.000	134.508
C2orf16	0	5	1.000	0.000	0.000	30.501
C2orf71	0	2	1.000	0.000	0.000	134.508
C3	0	4	1.000	0.000	0.000	41.489
C3orf58	0	2	1.000	0.000	0.000	134.508
C4A	0	2	1.000	0.000	0.000	134.508
C4orf21	0	2	1.000	0.000	0.000	134.508
C4orf22	0	2	1.000	0.000	0.000	134.508
C4orf40	0	2	1.000	0.000	0.000	134.508
C4orf50	0	2	1.000	0.000	0.000	134.508
C5orf42	0	2	1.000	0.000	0.000	134.508
C5orf51	0	2	1.000	0.000	0.000	134.508
C5orf54	0	2	1.000	0.000	0.000	134.508
C6	0	2	1.000	0.000	0.000	134.508
C6orf118	0	2	1.000	0.000	0.000	134.508
C7	0	2	1.000	0.000	0.000	134.508
C7orf31	0	3	1.000	0.000	0.000	64.152
C7orf69	0	2	1.000	0.000	0.000	134.508
C8orf34	0	2	1.000	0.000	0.000	134.508
C9	0	2	1.000	0.000	0.000	134.508
C9orf139	0	2	1.000	0.000	0.000	134.508
C9orf3	0	2	1.000	0.000	0.000	134.508
C9orf43	0	2	1.000	0.000	0.000	134.508
C9orf50	0	3	1.000	0.000	0.000	64.152
C9orf84	0	2	1.000	0.000	0.000	134.508
C9orf96	0	2	1.000	0.000	0.000	134.508
CA6	0	2	1.000	0.000	0.000	134.508
CA9	0	2	1.000	0.000	0.000	134.508
CABIN1	0	2	1.000	0.000	0.000	134.508
CACNA1A	0	2	1.000	0.000	0.000	134.508
CACNA1C	0	3	1.000	0.000	0.000	64.152
CACNA1D	0	2	1.000	0.000	0.000	134.508
CACNA1E	0	2	1.000	0.000	0.000	134.508
CACNA1G	0	3	1.000	0.000	0.000	64.152
CACNA1H	0	3	1.000	0.000	0.000	64.152
CACNA1S	0	2	1.000	0.000	0.000	134.508
CACNA2D3	0	2	1.000	0.000	0.000	134.508
CACNB2	0	2	1.000	0.000	0.000	134.508
CACNG2	0	2	1.000	0.000	0.000	134.508
CACNG3	0	2	1.000	0.000	0.000	134.508
CAD	0	2	1.000	0.000	0.000	134.508
CADM2	0	3	1.000	0.000	0.000	64.152
CADM3	0	3	1.000	0.000	0.000	64.152
CADPS	0	2	1.000	0.000	0.000	134.508
CADPS2	0	2	1.000	0.000	0.000	134.508
CALML5	0	3	1.000	0.000	0.000	64.152
CAMK1D	0	2	1.000	0.000	0.000	134.508
CAMK1G	0	2	1.000	0.000	0.000	134.508

CAMK4	0	2	1.000	0.000	0.000	134.508
CAMSAP3	0	3	1.000	0.000	0.000	64.152
CAND1	0	2	1.000	0.000	0.000	134.508
CAND2	0	3	1.000	0.000	0.000	64.152
CAPN3	0	2	1.000	0.000	0.000	134.508
CAPN5	0	2	1.000	0.000	0.000	134.508
CAPNS1	0	2	1.000	0.000	0.000	134.508
CAPZA3	0	4	1.000	0.000	0.000	41.489
CARD11	0	2	1.000	0.000	0.000	134.508
CARD9	0	4	1.000	0.000	0.000	41.489
CARM1	0	2	1.000	0.000	0.000	134.508
CASD1	0	2	1.000	0.000	0.000	134.508
CATSPER1	0	2	1.000	0.000	0.000	134.508
CATSPERD	0	2	1.000	0.000	0.000	134.508
CBFA2T2	0	2	1.000	0.000	0.000	134.508
CBLL1	0	2	1.000	0.000	0.000	134.508
CBX1	0	2	1.000	0.000	0.000	134.508
CCAR1	0	2	1.000	0.000	0.000	134.508
CCDC108	0	3	1.000	0.000	0.000	64.152
CCDC11	0	2	1.000	0.000	0.000	134.508
CCDC116	0	2	1.000	0.000	0.000	134.508
CCDC132	0	3	1.000	0.000	0.000	64.152
CCDC135	0	3	1.000	0.000	0.000	64.152
CCDC138	0	3	1.000	0.000	0.000	64.152
CCDC144NL	0	4	1.000	0.000	0.000	41.489
CCDC150	0	2	1.000	0.000	0.000	134.508
CCDC171	0	2	1.000	0.000	0.000	134.508
CCDC18	0	2	1.000	0.000	0.000	134.508
CCDC180	0	2	1.000	0.000	0.000	134.508
CCDC40	0	2	1.000	0.000	0.000	134.508
CCDC60	0	3	1.000	0.000	0.000	64.152
CCDC73	0	2	1.000	0.000	0.000	134.508
CCDC8	0	4	1.000	0.000	0.000	41.489
CCDC85A	0	3	1.000	0.000	0.000	64.152
CCDC87	0	2	1.000	0.000	0.000	134.508
CCDC88B	0	2	1.000	0.000	0.000	134.508
CCDC90B	0	2	1.000	0.000	0.000	134.508
CCDC93	0	3	1.000	0.000	0.000	64.152
CCHCR1	0	2	1.000	0.000	0.000	134.508
CCKAR	0	2	1.000	0.000	0.000	134.508
CCL1	0	2	1.000	0.000	0.000	134.508
CCNA1	0	2	1.000	0.000	0.000	134.508
CCNF	0	2	1.000	0.000	0.000	134.508
CCNL1	0	2	1.000	0.000	0.000	134.508
CCNO	0	2	1.000	0.000	0.000	134.508
CCNYL1	0	3	1.000	0.000	0.000	64.152
CCSER1	0	2	1.000	0.000	0.000	134.508
CCT3	0	3	1.000	0.000	0.000	64.152
CD163L1	0	3	1.000	0.000	0.000	64.152
CD19	0	2	1.000	0.000	0.000	134.508
CD1D	0	3	1.000	0.000	0.000	64.152
CD207	0	2	1.000	0.000	0.000	134.508
CD248	0	2	1.000	0.000	0.000	134.508
CD274	0	2	1.000	0.000	0.000	134.508
CD33	0	3	1.000	0.000	0.000	64.152
CD46	0	2	1.000	0.000	0.000	134.508
CD81	0	2	1.000	0.000	0.000	134.508
CD8B	0	2	1.000	0.000	0.000	134.508
CD99	0	3	1.000	0.000	0.000	64.152
CDC14A	0	2	1.000	0.000	0.000	134.508
CDC20B	0	2	1.000	0.000	0.000	134.508
CDC27	1	31	1.000	0.711	0.015	6.701
CDC42BPA	0	2	1.000	0.000	0.000	134.508
CDC6	0	2	1.000	0.000	0.000	134.508
CDC73	0	3	1.000	0.000	0.000	64.152
CDCA2	0	2	1.000	0.000	0.000	134.508

CDH1	0	2	1.000	0.000	0.000	134.508
CDH10	0	4	1.000	0.000	0.000	41.489
CDH15	0	2	1.000	0.000	0.000	134.508
CDH17	0	2	1.000	0.000	0.000	134.508
CDH18	0	3	1.000	0.000	0.000	64.152
CDH2	0	4	1.000	0.000	0.000	41.489
CDH20	0	2	1.000	0.000	0.000	134.508
CDH22	0	2	1.000	0.000	0.000	134.508
CDH23	0	5	1.000	0.000	0.000	30.501
CDH26	0	2	1.000	0.000	0.000	134.508
CDH4	0	3	1.000	0.000	0.000	64.152
CDH8	0	4	1.000	0.000	0.000	41.489
CDHR1	0	2	1.000	0.000	0.000	134.508
CDHR2	0	2	1.000	0.000	0.000	134.508
CDIPT	0	2	1.000	0.000	0.000	134.508
CDK14	0	2	1.000	0.000	0.000	134.508
CDK5RAP1	0	2	1.000	0.000	0.000	134.508
CDK5RAP2	0	2	1.000	0.000	0.000	134.508
CDKN2AIP	0	2	1.000	0.000	0.000	134.508
CDYL2	0	2	1.000	0.000	0.000	134.508
CEACAM3	0	2	1.000	0.000	0.000	134.508
CEACAM6	0	3	1.000	0.000	0.000	64.152
CEBPZ	0	2	1.000	0.000	0.000	134.508
CECR2	0	2	1.000	0.000	0.000	134.508
CELF2	0	2	1.000	0.000	0.000	134.508
CELF3	0	2	1.000	0.000	0.000	134.508
CELF4	0	2	1.000	0.000	0.000	134.508
CELF6	0	2	1.000	0.000	0.000	134.508
CELSR1	0	3	1.000	0.000	0.000	64.152
CELSR2	0	2	1.000	0.000	0.000	134.508
CELSR3	0	5	1.000	0.000	0.000	30.501
CENPC	0	2	1.000	0.000	0.000	134.508
CENPF	0	2	1.000	0.000	0.000	134.508
CENPJ	0	4	1.000	0.000	0.000	41.489
CEP104	0	2	1.000	0.000	0.000	134.508
CEP112	0	2	1.000	0.000	0.000	134.508
CEP250	0	2	1.000	0.000	0.000	134.508
CEP350	0	4	1.000	0.000	0.000	41.489
CEP44	0	2	1.000	0.000	0.000	134.508
CEP63	0	2	1.000	0.000	0.000	134.508
CEP70	0	3	1.000	0.000	0.000	64.152
CEP76	0	2	1.000	0.000	0.000	134.508
CEP85	0	2	1.000	0.000	0.000	134.508
CEP89	0	2	1.000	0.000	0.000	134.508
CEP97	0	2	1.000	0.000	0.000	134.508
CERCAM	0	2	1.000	0.000	0.000	134.508
CFB	0	2	1.000	0.000	0.000	134.508
CFH	0	2	1.000	0.000	0.000	134.508
CFTR	0	2	1.000	0.000	0.000	134.508
CGNL1	0	2	1.000	0.000	0.000	134.508
CGRRF1	0	2	1.000	0.000	0.000	134.508
CHAT	0	2	1.000	0.000	0.000	134.508
CHD3	0	2	1.000	0.000	0.000	134.508
CHD4	0	2	1.000	0.000	0.000	134.508
CHD5	0	2	1.000	0.000	0.000	134.508
CHD6	0	2	1.000	0.000	0.000	134.508
CHD8	0	3	1.000	0.000	0.000	64.152
CHFR	0	2	1.000	0.000	0.000	134.508
CHGA	0	3	1.000	0.000	0.000	64.152
CHIA	0	2	1.000	0.000	0.000	134.508
CHML	0	3	1.000	0.000	0.000	64.152
CHPF2	0	2	1.000	0.000	0.000	134.508
CHRFAM7A	0	2	1.000	0.000	0.000	134.508
CHRM3	0	2	1.000	0.000	0.000	134.508
CHRNA2	0	2	1.000	0.000	0.000	134.508
CHST11	0	3	1.000	0.000	0.000	64.152

CHST5	0	2	1.000	0.000	0.000	134.508
CHSY3	0	2	1.000	0.000	0.000	134.508
CIRH1A	0	2	1.000	0.000	0.000	134.508
CIT	0	2	1.000	0.000	0.000	134.508
CKAP2L	0	4	1.000	0.000	0.000	41.489
CKAP5	0	3	1.000	0.000	0.000	64.152
CKMT2	0	3	1.000	0.000	0.000	64.152
CLCA1	0	3	1.000	0.000	0.000	64.152
CLCA2	0	2	1.000	0.000	0.000	134.508
CLCN6	0	2	1.000	0.000	0.000	134.508
CLEC1B	0	2	1.000	0.000	0.000	134.508
CLHC1	0	2	1.000	0.000	0.000	134.508
CLIP1	0	2	1.000	0.000	0.000	134.508
CLIP4	0	2	1.000	0.000	0.000	134.508
CLPTM1	0	2	1.000	0.000	0.000	134.508
CLSTN2	0	4	1.000	0.000	0.000	41.489
CLSTN3	0	2	1.000	0.000	0.000	134.508
CLTC	0	2	1.000	0.000	0.000	134.508
CLTCL1	0	2	1.000	0.000	0.000	134.508
CLVS1	0	2	1.000	0.000	0.000	134.508
CMTR1	0	2	1.000	0.000	0.000	134.508
CMYA5	0	5	1.000	0.000	0.000	30.501
CNBD1	0	2	1.000	0.000	0.000	134.508
CNBD2	0	2	1.000	0.000	0.000	134.508
CNGA3	0	3	1.000	0.000	0.000	64.152
CNGA4	0	2	1.000	0.000	0.000	134.508
CNIH3	0	3	1.000	0.000	0.000	64.152
CNKSR2	0	2	1.000	0.000	0.000	134.508
CNN2	0	2	1.000	0.000	0.000	134.508
CNNM4	0	2	1.000	0.000	0.000	134.508
CNOT1	0	3	1.000	0.000	0.000	64.152
CNOT10	0	2	1.000	0.000	0.000	134.508
CNOT3	0	2	1.000	0.000	0.000	134.508
CNOT4	0	3	1.000	0.000	0.000	64.152
CNOT6L	0	3	1.000	0.000	0.000	64.152
CNTLN	0	2	1.000	0.000	0.000	134.508
CNTN1	0	4	1.000	0.000	0.000	41.489
CNTN3	0	3	1.000	0.000	0.000	64.152
CNTN4	0	4	1.000	0.000	0.000	41.489
CNTN5	0	2	1.000	0.000	0.000	134.508
CNTN6	0	2	1.000	0.000	0.000	134.508
CNTNAP2	0	2	1.000	0.000	0.000	134.508
CNTRL	0	3	1.000	0.000	0.000	64.152
COCH	0	2	1.000	0.000	0.000	134.508
COG1	0	2	1.000	0.000	0.000	134.508
COL11A1	0	7	1.000	0.000	0.000	19.777
COL11A2	0	2	1.000	0.000	0.000	134.508
COL12A1	0	2	1.000	0.000	0.000	134.508
COL14A1	0	3	1.000	0.000	0.000	64.152
COL18A1	0	3	1.000	0.000	0.000	64.152
COL1A1	0	3	1.000	0.000	0.000	64.152
COL1A2	0	3	1.000	0.000	0.000	64.152
COL22A1	0	4	1.000	0.000	0.000	41.489
COL25A1	0	2	1.000	0.000	0.000	134.508
COL27A1	0	3	1.000	0.000	0.000	64.152
COL28A1	0	2	1.000	0.000	0.000	134.508
COL2A1	0	2	1.000	0.000	0.000	134.508
COL4A2	0	3	1.000	0.000	0.000	64.152
COL4A4	0	3	1.000	0.000	0.000	64.152
COL5A1	0	4	1.000	0.000	0.000	41.489
COL5A2	0	4	1.000	0.000	0.000	41.489
COL5A3	0	2	1.000	0.000	0.000	134.508
COL6A3	0	4	1.000	0.000	0.000	41.489
COL6A6	0	3	1.000	0.000	0.000	64.152
COL9A1	0	2	1.000	0.000	0.000	134.508
COLEC11	0	2	1.000	0.000	0.000	134.508

CORIN	0	2	1.000	0.000	0.000	134.508
CORO1B	0	2	1.000	0.000	0.000	134.508
CORO2B	0	2	1.000	0.000	0.000	134.508
COX4I2	0	2	1.000	0.000	0.000	134.508
CPE	0	2	1.000	0.000	0.000	134.508
CPEB4	0	2	1.000	0.000	0.000	134.508
CPN2	0	2	1.000	0.000	0.000	134.508
CPNE2	0	2	1.000	0.000	0.000	134.508
CPNE4	0	2	1.000	0.000	0.000	134.508
CPNE5	0	2	1.000	0.000	0.000	134.508
CPO	0	3	1.000	0.000	0.000	64.152
CPT1B	0	3	1.000	0.000	0.000	64.152
CPXM2	0	2	1.000	0.000	0.000	134.508
CR1	0	3	1.000	0.000	0.000	64.152
CR1L	0	2	1.000	0.000	0.000	134.508
CR2	0	5	1.000	0.000	0.000	30.501
CRB2	0	2	1.000	0.000	0.000	134.508
CREBBP	0	2	1.000	0.000	0.000	134.508
CREBZF	0	2	1.000	0.000	0.000	134.508
CRMP1	0	2	1.000	0.000	0.000	134.508
CRNN	0	3	1.000	0.000	0.000	64.152
CROCC	0	3	1.000	0.000	0.000	64.152
CROT	0	3	1.000	0.000	0.000	64.152
CRYBB1	0	2	1.000	0.000	0.000	134.508
CSE1L	0	2	1.000	0.000	0.000	134.508
CSF1	0	2	1.000	0.000	0.000	134.508
CSF3R	0	2	1.000	0.000	0.000	134.508
CSGALNACT2	0	3	1.000	0.000	0.000	64.152
CSMD1	0	3	1.000	0.000	0.000	64.152
CSPG4	0	2	1.000	0.000	0.000	134.508
CSPP1	0	3	1.000	0.000	0.000	64.152
CSRNP1	0	2	1.000	0.000	0.000	134.508
CSRNP3	0	2	1.000	0.000	0.000	134.508
CST9L	0	2	1.000	0.000	0.000	134.508
CTAGE5	0	4	1.000	0.000	0.000	41.489
CTBP2	0	4	1.000	0.000	0.000	41.489
CTCF	0	2	1.000	0.000	0.000	134.508
CTNNA2	0	3	1.000	0.000	0.000	64.152
CTNNA3	0	3	1.000	0.000	0.000	64.152
CTNND2	0	3	1.000	0.000	0.000	64.152
CTR9	0	3	1.000	0.000	0.000	64.152
CTSB	0	2	1.000	0.000	0.000	134.508
CTSE	0	2	1.000	0.000	0.000	134.508
CTSG	0	2	1.000	0.000	0.000	134.508
CTSS	0	2	1.000	0.000	0.000	134.508
CUBN	0	5	1.000	0.000	0.000	30.501
CUL1	0	2	1.000	0.000	0.000	134.508
CUL2	0	2	1.000	0.000	0.000	134.508
CUL3	0	2	1.000	0.000	0.000	134.508
CUL4B	0	2	1.000	0.000	0.000	134.508
CUX1	0	2	1.000	0.000	0.000	134.508
CX3CL1	0	2	1.000	0.000	0.000	134.508
CXCR1	0	3	1.000	0.000	0.000	64.152
CXCR4	0	2	1.000	0.000	0.000	134.508
CXorf21	0	2	1.000	0.000	0.000	134.508
CXorf22	0	3	1.000	0.000	0.000	64.152
CYB5R1	0	2	1.000	0.000	0.000	134.508
CYB5R3	0	3	1.000	0.000	0.000	64.152
CYFIP1	0	2	1.000	0.000	0.000	134.508
CYLC1	0	2	1.000	0.000	0.000	134.508
CYLD	0	2	1.000	0.000	0.000	134.508
CYP11B1	0	3	1.000	0.000	0.000	64.152
CYP19A1	0	2	1.000	0.000	0.000	134.508
CYP1A1	0	2	1.000	0.000	0.000	134.508
CYP20A1	0	2	1.000	0.000	0.000	134.508
CYP2A13	0	3	1.000	0.000	0.000	64.152

CYP2C18	0	2	1.000	0.000	0.000	134.508
CYP4B1	0	2	1.000	0.000	0.000	134.508
CYP4F11	0	2	1.000	0.000	0.000	134.508
CYP4F22	0	2	1.000	0.000	0.000	134.508
CYP7A1	0	2	1.000	0.000	0.000	134.508
CYP8B1	0	2	1.000	0.000	0.000	134.508
D2HGDH	0	3	1.000	0.000	0.000	64.152
DAAM1	0	3	1.000	0.000	0.000	64.152
DACH2	0	2	1.000	0.000	0.000	134.508
DACT1	0	2	1.000	0.000	0.000	134.508
DAGLA	0	3	1.000	0.000	0.000	64.152
DAPK1	0	2	1.000	0.000	0.000	134.508
DAPP1	0	2	1.000	0.000	0.000	134.508
DAW1	0	2	1.000	0.000	0.000	134.508
DBNL	0	2	1.000	0.000	0.000	134.508
DCAF12L2	0	3	1.000	0.000	0.000	64.152
DCAF4	0	2	1.000	0.000	0.000	134.508
DCAF8	0	2	1.000	0.000	0.000	134.508
DCHS2	0	3	1.000	0.000	0.000	64.152
DCLK3	0	2	1.000	0.000	0.000	134.508
DCLRE1B	0	2	1.000	0.000	0.000	134.508
DCP1B	0	4	1.000	0.000	0.000	41.489
DCPS	0	2	1.000	0.000	0.000	134.508
DCST1	0	2	1.000	0.000	0.000	134.508
DCST2	0	2	1.000	0.000	0.000	134.508
DCSTAMP	0	3	1.000	0.000	0.000	64.152
DCTD	0	2	1.000	0.000	0.000	134.508
DCTN1	0	2	1.000	0.000	0.000	134.508
DDB1	0	2	1.000	0.000	0.000	134.508
DDHD1	0	3	1.000	0.000	0.000	64.152
DDHD2	0	2	1.000	0.000	0.000	134.508
DDI1	0	2	1.000	0.000	0.000	134.508
DDO	0	2	1.000	0.000	0.000	134.508
DDX11	0	2	1.000	0.000	0.000	134.508
DDX18	0	2	1.000	0.000	0.000	134.508
DDX4	0	2	1.000	0.000	0.000	134.508
DDX46	0	2	1.000	0.000	0.000	134.508
DDX50	0	2	1.000	0.000	0.000	134.508
DDX59	0	2	1.000	0.000	0.000	134.508
DDX60	0	3	1.000	0.000	0.000	64.152
DDX60L	0	2	1.000	0.000	0.000	134.508
DEAF1	0	3	1.000	0.000	0.000	64.152
DEF8	0	2	1.000	0.000	0.000	134.508
DEFB119	0	4	1.000	0.000	0.000	41.489
DENND2A	0	2	1.000	0.000	0.000	134.508
DENND2D	0	2	1.000	0.000	0.000	134.508
DENND4C	0	3	1.000	0.000	0.000	64.152
DENND5A	0	3	1.000	0.000	0.000	64.152
DENND5B	0	2	1.000	0.000	0.000	134.508
DEPDC1	0	3	1.000	0.000	0.000	64.152
DEPDC4	0	2	1.000	0.000	0.000	134.508
DET1	0	2	1.000	0.000	0.000	134.508
DFNB31	0	2	1.000	0.000	0.000	134.508
DGCR2	0	3	1.000	0.000	0.000	64.152
DGKB	0	3	1.000	0.000	0.000	64.152
DGKH	0	2	1.000	0.000	0.000	134.508
DGKI	0	4	1.000	0.000	0.000	41.489
DGKQ	0	2	1.000	0.000	0.000	134.508
DHRS1	0	3	1.000	0.000	0.000	64.152
DHRS2	0	2	1.000	0.000	0.000	134.508
DHRS4	0	3	1.000	0.000	0.000	64.152
DHRS4L2	0	2	1.000	0.000	0.000	134.508
DHRS9	0	3	1.000	0.000	0.000	64.152
DHX15	0	2	1.000	0.000	0.000	134.508
DHX32	0	2	1.000	0.000	0.000	134.508
DHX35	0	2	1.000	0.000	0.000	134.508

DHX36	0	4	1.000	0.000	0.000	41.489
DHX40	0	2	1.000	0.000	0.000	134.508
DHX8	0	3	1.000	0.000	0.000	64.152
DIAPH1	0	2	1.000	0.000	0.000	134.508
DIAPH2	0	3	1.000	0.000	0.000	64.152
DIAPH3	0	2	1.000	0.000	0.000	134.508
DICER1	0	3	1.000	0.000	0.000	64.152
DIDO1	0	3	1.000	0.000	0.000	64.152
DIO3	0	4	1.000	0.000	0.000	41.489
DIP2A	0	2	1.000	0.000	0.000	134.508
DISC1	0	3	1.000	0.000	0.000	64.152
DISP1	0	4	1.000	0.000	0.000	41.489
DKKL1	0	3	1.000	0.000	0.000	64.152
DLC1	0	2	1.000	0.000	0.000	134.508
DLG2	0	2	1.000	0.000	0.000	134.508
DLG3	0	2	1.000	0.000	0.000	134.508
DLG4	0	2	1.000	0.000	0.000	134.508
DLGAP1	0	2	1.000	0.000	0.000	134.508
DLGAP2	0	5	1.000	0.000	0.000	30.501
DLGAP4	0	2	1.000	0.000	0.000	134.508
DLK2	0	2	1.000	0.000	0.000	134.508
DLL4	0	2	1.000	0.000	0.000	134.508
DMD	0	7	1.000	0.000	0.000	19.777
DMGDH	0	2	1.000	0.000	0.000	134.508
DMXL1	0	3	1.000	0.000	0.000	64.152
DMXL2	0	2	1.000	0.000	0.000	134.508
DNA2	0	3	1.000	0.000	0.000	64.152
DNAH1	0	2	1.000	0.000	0.000	134.508
DNAH10	0	7	1.000	0.000	0.000	19.777
DNAH11	0	7	1.000	0.000	0.000	19.777
DNAH17	0	4	1.000	0.000	0.000	41.489
DNAH2	0	3	1.000	0.000	0.000	64.152
DNAH3	0	7	1.000	0.000	0.000	19.777
DNAH5	0	5	1.000	0.000	0.000	30.501
DNAH6	0	2	1.000	0.000	0.000	134.508
DNAH7	0	3	1.000	0.000	0.000	64.152
DNAH8	0	5	1.000	0.000	0.000	30.501
DNAH9	0	4	1.000	0.000	0.000	41.489
DNAJC6	0	2	1.000	0.000	0.000	134.508
DND1	0	3	1.000	0.000	0.000	64.152
DNER	0	4	1.000	0.000	0.000	41.489
DNHD1	0	2	1.000	0.000	0.000	134.508
DNM1L	0	2	1.000	0.000	0.000	134.508
DNM2	0	2	1.000	0.000	0.000	134.508
DNMBP	0	2	1.000	0.000	0.000	134.508
DNMT3A	0	3	1.000	0.000	0.000	64.152
DNMT3B	0	3	1.000	0.000	0.000	64.152
DNTT	0	2	1.000	0.000	0.000	134.508
DOCK1	0	3	1.000	0.000	0.000	64.152
DOCK10	0	4	1.000	0.000	0.000	41.489
DOCK11	0	2	1.000	0.000	0.000	134.508
DOCK2	0	3	1.000	0.000	0.000	64.152
DOCK3	0	2	1.000	0.000	0.000	134.508
DOCK4	0	3	1.000	0.000	0.000	64.152
DOCK8	0	3	1.000	0.000	0.000	64.152
DOCK9	0	3	1.000	0.000	0.000	64.152
DOK5	0	2	1.000	0.000	0.000	134.508
DOPEY1	0	2	1.000	0.000	0.000	134.508
DOPEY2	0	3	1.000	0.000	0.000	64.152
DOT1L	0	3	1.000	0.000	0.000	64.152
DPF2	0	2	1.000	0.000	0.000	134.508
DPP10	0	3	1.000	0.000	0.000	64.152
DPP6	0	3	1.000	0.000	0.000	64.152
DPY19L2	0	2	1.000	0.000	0.000	134.508
DPY19L3	0	2	1.000	0.000	0.000	134.508
DPYD	0	4	1.000	0.000	0.000	41.489

DPYS	0	2	1.000	0.000	0.000	134.508
DPYSL5	0	2	1.000	0.000	0.000	134.508
DRD1	0	2	1.000	0.000	0.000	134.508
DROSHA	0	2	1.000	0.000	0.000	134.508
DRP2	0	4	1.000	0.000	0.000	41.489
DSC1	0	4	1.000	0.000	0.000	41.489
DSC3	0	2	1.000	0.000	0.000	134.508
DSCAM	0	3	1.000	0.000	0.000	64.152
DSCAML1	0	4	1.000	0.000	0.000	41.489
DSCC1	0	2	1.000	0.000	0.000	134.508
DSCR4	0	2	1.000	0.000	0.000	134.508
DSEL	0	2	1.000	0.000	0.000	134.508
DSG2	0	4	1.000	0.000	0.000	41.489
DSG4	0	2	1.000	0.000	0.000	134.508
DSP	0	3	1.000	0.000	0.000	64.152
DST	0	3	1.000	0.000	0.000	64.152
DTX2	0	2	1.000	0.000	0.000	134.508
DUOX1	0	3	1.000	0.000	0.000	64.152
DUSP13	0	2	1.000	0.000	0.000	134.508
DYM	0	2	1.000	0.000	0.000	134.508
DYNC1H1	0	3	1.000	0.000	0.000	64.152
DYNC2H1	0	5	1.000	0.000	0.000	30.501
DYRK1A	0	3	1.000	0.000	0.000	64.152
DYSF	0	5	1.000	0.000	0.000	30.501
DZIP3	0	2	1.000	0.000	0.000	134.508
E2F4	0	2	1.000	0.000	0.000	134.508
E4F1	0	2	1.000	0.000	0.000	134.508
EBAG9	0	2	1.000	0.000	0.000	134.508
EED	0	2	1.000	0.000	0.000	134.508
EEPD1	0	2	1.000	0.000	0.000	134.508
EFCAB6	0	2	1.000	0.000	0.000	134.508
EFEMP2	0	2	1.000	0.000	0.000	134.508
EFHC1	0	3	1.000	0.000	0.000	64.152
EFS	0	3	1.000	0.000	0.000	64.152
EFTUD2	0	4	1.000	0.000	0.000	41.489
EGF	0	4	1.000	0.000	0.000	41.489
EGFLAM	0	2	1.000	0.000	0.000	134.508
EGR1	0	2	1.000	0.000	0.000	134.508
EHMT2	0	2	1.000	0.000	0.000	134.508
EIF3A	0	2	1.000	0.000	0.000	134.508
EIF4ENIF1	0	2	1.000	0.000	0.000	134.508
ELAVL2	0	2	1.000	0.000	0.000	134.508
ELAVL4	0	3	1.000	0.000	0.000	64.152
ELL	0	2	1.000	0.000	0.000	134.508
ELMOD2	0	2	1.000	0.000	0.000	134.508
ELOVL6	0	2	1.000	0.000	0.000	134.508
ELP4	0	2	1.000	0.000	0.000	134.508
ELTD1	0	4	1.000	0.000	0.000	41.489
EML1	0	4	1.000	0.000	0.000	41.489
EML5	0	2	1.000	0.000	0.000	134.508
ENAM	0	2	1.000	0.000	0.000	134.508
ENC1	0	3	1.000	0.000	0.000	64.152
ENGASE	0	3	1.000	0.000	0.000	64.152
ENOX1	0	3	1.000	0.000	0.000	64.152
ENPP4	0	2	1.000	0.000	0.000	134.508
ENPP7	0	2	1.000	0.000	0.000	134.508
ENTPD2	0	2	1.000	0.000	0.000	134.508
ENTPD4	0	4	1.000	0.000	0.000	41.489
ENTPD8	0	2	1.000	0.000	0.000	134.508
EP300	0	3	1.000	0.000	0.000	64.152
EP400	0	3	1.000	0.000	0.000	64.152
EPB41L1	0	2	1.000	0.000	0.000	134.508
EPB41L3	0	2	1.000	0.000	0.000	134.508
EPB42	0	2	1.000	0.000	0.000	134.508
EPC1	0	2	1.000	0.000	0.000	134.508
EPC2	0	2	1.000	0.000	0.000	134.508

EPHA1	0	2	1.000	0.000	0.000	134.508
EPHA4	0	2	1.000	0.000	0.000	134.508
EPHA5	0	2	1.000	0.000	0.000	134.508
EPHA6	0	3	1.000	0.000	0.000	64.152
EPHA7	0	3	1.000	0.000	0.000	64.152
EPHA8	0	4	1.000	0.000	0.000	41.489
EPHB2	0	3	1.000	0.000	0.000	64.152
EPHB4	0	2	1.000	0.000	0.000	134.508
EPHX1	0	2	1.000	0.000	0.000	134.508
EPO	0	2	1.000	0.000	0.000	134.508
EPPK1	0	2	1.000	0.000	0.000	134.508
EPRS	0	2	1.000	0.000	0.000	134.508
EPS8L3	0	2	1.000	0.000	0.000	134.508
ERAP2	0	3	1.000	0.000	0.000	64.152
ERBB2	0	2	1.000	0.000	0.000	134.508
ERBB2IP	0	2	1.000	0.000	0.000	134.508
ERBB4	0	2	1.000	0.000	0.000	134.508
ERC1	0	2	1.000	0.000	0.000	134.508
ERCC2	0	2	1.000	0.000	0.000	134.508
ERCC4	0	2	1.000	0.000	0.000	134.508
ERCC5	0	2	1.000	0.000	0.000	134.508
ERCC6L2	0	2	1.000	0.000	0.000	134.508
ERN2	0	2	1.000	0.000	0.000	134.508
ESCO1	0	2	1.000	0.000	0.000	134.508
ESPL1	0	2	1.000	0.000	0.000	134.508
ESR2	0	2	1.000	0.000	0.000	134.508
ESRRA	0	2	1.000	0.000	0.000	134.508
ETAA1	0	2	1.000	0.000	0.000	134.508
ETFDH	0	2	1.000	0.000	0.000	134.508
ETNPPL	0	3	1.000	0.000	0.000	64.152
ETV5	0	2	1.000	0.000	0.000	134.508
EXO1	0	2	1.000	0.000	0.000	134.508
EXOC1	0	2	1.000	0.000	0.000	134.508
EXOC5	0	2	1.000	0.000	0.000	134.508
EXOSC8	0	2	1.000	0.000	0.000	134.508
EXPH5	0	3	1.000	0.000	0.000	64.152
EXTL2	0	2	1.000	0.000	0.000	134.508
EXTL3	0	2	1.000	0.000	0.000	134.508
EYS	0	3	1.000	0.000	0.000	64.152
F13A1	0	3	1.000	0.000	0.000	64.152
F5	0	2	1.000	0.000	0.000	134.508
F8	0	2	1.000	0.000	0.000	134.508
FADS3	0	2	1.000	0.000	0.000	134.508
FAM107A	0	2	1.000	0.000	0.000	134.508
FAM114A1	0	2	1.000	0.000	0.000	134.508
FAM120C	0	2	1.000	0.000	0.000	134.508
FAM129B	0	2	1.000	0.000	0.000	134.508
FAM129C	0	4	1.000	0.000	0.000	41.489
FAM13B	0	2	1.000	0.000	0.000	134.508
FAM155A	0	2	1.000	0.000	0.000	134.508
FAM160A2	0	2	1.000	0.000	0.000	134.508
FAM166A	0	2	1.000	0.000	0.000	134.508
FAM167A	0	2	1.000	0.000	0.000	134.508
FAM169B	0	2	1.000	0.000	0.000	134.508
FAM178A	0	2	1.000	0.000	0.000	134.508
FAM179B	0	3	1.000	0.000	0.000	64.152
FAM208A	0	2	1.000	0.000	0.000	134.508
FAM208B	0	4	1.000	0.000	0.000	41.489
FAM35A	0	2	1.000	0.000	0.000	134.508
FAM47A	0	8	1.000	0.000	0.000	16.757
FAM47C	0	2	1.000	0.000	0.000	134.508
FAM49A	0	2	1.000	0.000	0.000	134.508
FAM53A	0	2	1.000	0.000	0.000	134.508
FAM65A	0	3	1.000	0.000	0.000	64.152
FAM71A	0	3	1.000	0.000	0.000	64.152
FAM71B	0	5	1.000	0.000	0.000	30.501

FAM83B	0	2	1.000	0.000	0.000	134.508
FAM8A1	0	2	1.000	0.000	0.000	134.508
FAN1	0	2	1.000	0.000	0.000	134.508
FANCA	0	2	1.000	0.000	0.000	134.508
FANCM	0	2	1.000	0.000	0.000	134.508
FAR1	0	2	1.000	0.000	0.000	134.508
FARP2	0	3	1.000	0.000	0.000	64.152
FASTKD1	0	3	1.000	0.000	0.000	64.152
FASTKD3	0	2	1.000	0.000	0.000	134.508
FAT1	0	7	1.000	0.000	0.000	19.777
FAT3	0	8	1.000	0.000	0.000	16.757
FAT4	0	5	1.000	0.000	0.000	30.501
FBN1	0	4	1.000	0.000	0.000	41.489
FBN2	0	4	1.000	0.000	0.000	41.489
FBN3	0	4	1.000	0.000	0.000	41.489
FBXL17	0	2	1.000	0.000	0.000	134.508
FBXL4	0	2	1.000	0.000	0.000	134.508
FBXO11	0	2	1.000	0.000	0.000	134.508
FBXO15	0	3	1.000	0.000	0.000	64.152
FBXO24	0	2	1.000	0.000	0.000	134.508
FBXO38	0	2	1.000	0.000	0.000	134.508
FBXO47	0	2	1.000	0.000	0.000	134.508
FBXO5	0	2	1.000	0.000	0.000	134.508
FBXW10	0	2	1.000	0.000	0.000	134.508
FBXW11	0	2	1.000	0.000	0.000	134.508
FBXW5	0	2	1.000	0.000	0.000	134.508
FCGBP	0	3	1.000	0.000	0.000	64.152
FERMT1	0	3	1.000	0.000	0.000	64.152
FGD4	0	2	1.000	0.000	0.000	134.508
FGD6	0	2	1.000	0.000	0.000	134.508
FGF13	0	2	1.000	0.000	0.000	134.508
FGF6	0	2	1.000	0.000	0.000	134.508
FGFR4	0	2	1.000	0.000	0.000	134.508
FHOD3	0	4	1.000	0.000	0.000	41.489
FIGN	0	3	1.000	0.000	0.000	64.152
FIP1L1	0	2	1.000	0.000	0.000	134.508
FKBP6	0	2	1.000	0.000	0.000	134.508
FKTN	0	2	1.000	0.000	0.000	134.508
FLG	0	9	1.000	0.000	0.000	14.507
FLG2	0	3	1.000	0.000	0.000	64.152
FLII	0	2	1.000	0.000	0.000	134.508
FLNB	0	2	1.000	0.000	0.000	134.508
FLNC	0	5	1.000	0.000	0.000	30.501
FLOT1	0	4	1.000	0.000	0.000	41.489
FLRT2	0	4	1.000	0.000	0.000	41.489
FLT1	0	3	1.000	0.000	0.000	64.152
FLT3	0	3	1.000	0.000	0.000	64.152
FLT4	0	5	1.000	0.000	0.000	30.501
FLVCR1	0	2	1.000	0.000	0.000	134.508
FMN2	0	5	1.000	0.000	0.000	30.501
FMNL3	0	4	1.000	0.000	0.000	41.489
FMR1NB	0	2	1.000	0.000	0.000	134.508
FNDC1	0	3	1.000	0.000	0.000	64.152
FOLH1	0	9	1.000	0.000	0.000	14.507
FOSL2	0	2	1.000	0.000	0.000	134.508
FOXG1	0	2	1.000	0.000	0.000	134.508
FOXI1	0	2	1.000	0.000	0.000	134.508
FOXJ3	0	2	1.000	0.000	0.000	134.508
FOXN4	0	2	1.000	0.000	0.000	134.508
FPGS	0	2	1.000	0.000	0.000	134.508
FPGT-TNNI3K	0	3	1.000	0.000	0.000	64.152
FPR1	0	4	1.000	0.000	0.000	41.489
FRAS1	0	2	1.000	0.000	0.000	134.508
FREM1	0	2	1.000	0.000	0.000	134.508
FREM2	0	4	1.000	0.000	0.000	41.489
FRG1	0	14	1.000	0.000	0.000	8.533

FRG2B	0	3	1.000	0.000	0.000	64.152
FRMD1	0	2	1.000	0.000	0.000	134.508
FRMD7	0	2	1.000	0.000	0.000	134.508
FRMPD1	0	4	1.000	0.000	0.000	41.489
FRMPD2	0	2	1.000	0.000	0.000	134.508
FRMPD3	0	2	1.000	0.000	0.000	134.508
FRMPD4	0	3	1.000	0.000	0.000	64.152
FRYL	0	3	1.000	0.000	0.000	64.152
FSCB	0	2	1.000	0.000	0.000	134.508
FSD2	0	2	1.000	0.000	0.000	134.508
FSHR	0	2	1.000	0.000	0.000	134.508
FSTL5	0	3	1.000	0.000	0.000	64.152
FTCD	0	2	1.000	0.000	0.000	134.508
FTHL17	0	2	1.000	0.000	0.000	134.508
FTSJ3	0	3	1.000	0.000	0.000	64.152
FURIN	0	2	1.000	0.000	0.000	134.508
FUT2	0	2	1.000	0.000	0.000	134.508
FXR1	0	2	1.000	0.000	0.000	134.508
FYCO1	0	3	1.000	0.000	0.000	64.152
FZD10	0	4	1.000	0.000	0.000	41.489
FZD3	0	2	1.000	0.000	0.000	134.508
FZD6	0	4	1.000	0.000	0.000	41.489
G3BP2	0	2	1.000	0.000	0.000	134.508
G6PC2	0	3	1.000	0.000	0.000	64.152
GAB3	0	2	1.000	0.000	0.000	134.508
GABBR1	0	2	1.000	0.000	0.000	134.508
GABBR2	0	2	1.000	0.000	0.000	134.508
GABPA	0	2	1.000	0.000	0.000	134.508
GABRA4	0	2	1.000	0.000	0.000	134.508
GABRB3	0	2	1.000	0.000	0.000	134.508
GABRD	0	2	1.000	0.000	0.000	134.508
GABRE	0	2	1.000	0.000	0.000	134.508
GABRG3	0	2	1.000	0.000	0.000	134.508
GABRQ	0	2	1.000	0.000	0.000	134.508
GAD1	0	2	1.000	0.000	0.000	134.508
GAD2	0	3	1.000	0.000	0.000	64.152
GAL3ST3	0	2	1.000	0.000	0.000	134.508
GALC	0	2	1.000	0.000	0.000	134.508
GALNT10	0	3	1.000	0.000	0.000	64.152
GALNT18	0	2	1.000	0.000	0.000	134.508
GALNT2	0	2	1.000	0.000	0.000	134.508
GALNT5	0	2	1.000	0.000	0.000	134.508
GALNT7	0	2	1.000	0.000	0.000	134.508
GALNTL6	0	2	1.000	0.000	0.000	134.508
GALR1	0	2	1.000	0.000	0.000	134.508
GAN	0	2	1.000	0.000	0.000	134.508
GANAB	0	2	1.000	0.000	0.000	134.508
GAPVD1	0	2	1.000	0.000	0.000	134.508
GARS	0	2	1.000	0.000	0.000	134.508
GAS2L2	0	4	1.000	0.000	0.000	41.489
GAS6	0	2	1.000	0.000	0.000	134.508
GAS7	0	3	1.000	0.000	0.000	64.152
GATA3	0	2	1.000	0.000	0.000	134.508
GATAD2B	0	2	1.000	0.000	0.000	134.508
GBA2	0	2	1.000	0.000	0.000	134.508
GBF1	0	2	1.000	0.000	0.000	134.508
GBP7	0	2	1.000	0.000	0.000	134.508
GCC2	0	2	1.000	0.000	0.000	134.508
GCDH	0	2	1.000	0.000	0.000	134.508
GCN1L1	0	2	1.000	0.000	0.000	134.508
GDPD4	0	2	1.000	0.000	0.000	134.508
GDPD5	0	2	1.000	0.000	0.000	134.508
GDPGP1	0	2	1.000	0.000	0.000	134.508
GFAP	0	2	1.000	0.000	0.000	134.508
GFER	0	3	1.000	0.000	0.000	64.152
GFM2	0	2	1.000	0.000	0.000	134.508

GFPT1	0	2	1.000	0.000	0.000	134.508
GFPT2	0	2	1.000	0.000	0.000	134.508
GFRAL	0	2	1.000	0.000	0.000	134.508
GGCX	0	2	1.000	0.000	0.000	134.508
GGNBP2	0	2	1.000	0.000	0.000	134.508
GGT5	0	2	1.000	0.000	0.000	134.508
GJC1	0	2	1.000	0.000	0.000	134.508
GK2	0	3	1.000	0.000	0.000	64.152
GLB1	0	2	1.000	0.000	0.000	134.508
GLB1L	0	3	1.000	0.000	0.000	64.152
GLB1L2	0	2	1.000	0.000	0.000	134.508
GLB1L3	0	2	1.000	0.000	0.000	134.508
GLCCI1	0	2	1.000	0.000	0.000	134.508
GLI1	0	2	1.000	0.000	0.000	134.508
GLI2	0	2	1.000	0.000	0.000	134.508
GLI3	0	5	1.000	0.000	0.000	30.501
GLIS3	0	2	1.000	0.000	0.000	134.508
GLMN	0	3	1.000	0.000	0.000	64.152
GLRA1	0	2	1.000	0.000	0.000	134.508
GLRA2	0	2	1.000	0.000	0.000	134.508
GLTSCR1L	0	2	1.000	0.000	0.000	134.508
GLTSCR2	0	3	1.000	0.000	0.000	64.152
GLYATL1	0	2	1.000	0.000	0.000	134.508
GNAO1	0	2	1.000	0.000	0.000	134.508
GNAQ	0	4	1.000	0.000	0.000	41.489
GNAS	0	7	1.000	0.000	0.000	19.777
GNB3	0	2	1.000	0.000	0.000	134.508
GNB4	0	2	1.000	0.000	0.000	134.508
GNPTAB	0	2	1.000	0.000	0.000	134.508
GOLGA1	0	3	1.000	0.000	0.000	64.152
GOLGA3	0	3	1.000	0.000	0.000	64.152
GOLGB1	0	2	1.000	0.000	0.000	134.508
GOLPH3	0	2	1.000	0.000	0.000	134.508
GOLPH3L	0	2	1.000	0.000	0.000	134.508
GOLT1B	0	2	1.000	0.000	0.000	134.508
GP2	0	2	1.000	0.000	0.000	134.508
GP6	0	2	1.000	0.000	0.000	134.508
GPC4	0	2	1.000	0.000	0.000	134.508
GPC5	0	2	1.000	0.000	0.000	134.508
GPER1	0	2	1.000	0.000	0.000	134.508
GPI	0	3	1.000	0.000	0.000	64.152
GPLD1	0	2	1.000	0.000	0.000	134.508
GPR111	0	2	1.000	0.000	0.000	134.508
GPR112	0	7	1.000	0.000	0.000	19.777
GPR124	0	2	1.000	0.000	0.000	134.508
GPR125	0	3	1.000	0.000	0.000	64.152
GPR133	0	4	1.000	0.000	0.000	41.489
GPR137B	0	2	1.000	0.000	0.000	134.508
GPR151	0	2	1.000	0.000	0.000	134.508
GPR156	0	2	1.000	0.000	0.000	134.508
GPR158	0	3	1.000	0.000	0.000	64.152
GPR171	0	2	1.000	0.000	0.000	134.508
GPR174	0	3	1.000	0.000	0.000	64.152
GPR179	0	2	1.000	0.000	0.000	134.508
GPR26	0	2	1.000	0.000	0.000	134.508
GPR39	0	2	1.000	0.000	0.000	134.508
GPR4	0	2	1.000	0.000	0.000	134.508
GPR64	0	2	1.000	0.000	0.000	134.508
GPR98	0	3	1.000	0.000	0.000	64.152
GPRASP1	0	2	1.000	0.000	0.000	134.508
GPRASP2	0	2	1.000	0.000	0.000	134.508
GPRC5B	0	2	1.000	0.000	0.000	134.508
GPRC6A	0	2	1.000	0.000	0.000	134.508
GPRIN2	0	2	1.000	0.000	0.000	134.508
GPX5	0	3	1.000	0.000	0.000	64.152
GRAMD1A	0	2	1.000	0.000	0.000	134.508

GRAMD1C	0	2	1.000	0.000	0.000	134.508
GRHL1	0	2	1.000	0.000	0.000	134.508
GRHL2	0	2	1.000	0.000	0.000	134.508
GRIA4	0	3	1.000	0.000	0.000	64.152
GRID1	0	3	1.000	0.000	0.000	64.152
GRID2	0	3	1.000	0.000	0.000	64.152
GRIK2	0	5	1.000	0.000	0.000	30.501
GRIK3	0	3	1.000	0.000	0.000	64.152
GRIK5	0	2	1.000	0.000	0.000	134.508
GRIN2A	0	2	1.000	0.000	0.000	134.508
GRIN2B	0	2	1.000	0.000	0.000	134.508
GRIN2D	0	2	1.000	0.000	0.000	134.508
GRIN3B	0	2	1.000	0.000	0.000	134.508
GRIP1	0	3	1.000	0.000	0.000	64.152
GRK6	0	2	1.000	0.000	0.000	134.508
GRK7	0	2	1.000	0.000	0.000	134.508
GRM4	0	2	1.000	0.000	0.000	134.508
GRM8	0	3	1.000	0.000	0.000	64.152
GRPR	0	2	1.000	0.000	0.000	134.508
GRSF1	0	2	1.000	0.000	0.000	134.508
GRXCR1	0	2	1.000	0.000	0.000	134.508
GSDMA	0	2	1.000	0.000	0.000	134.508
GSDMD	0	2	1.000	0.000	0.000	134.508
GSG2	0	2	1.000	0.000	0.000	134.508
GSTA3	0	2	1.000	0.000	0.000	134.508
GTF2F1	0	2	1.000	0.000	0.000	134.508
GTF2IRD1	0	2	1.000	0.000	0.000	134.508
GTF3C1	0	2	1.000	0.000	0.000	134.508
GTF3C2	0	2	1.000	0.000	0.000	134.508
GTPBP10	0	3	1.000	0.000	0.000	64.152
GTSE1	0	2	1.000	0.000	0.000	134.508
GUCY2F	0	2	1.000	0.000	0.000	134.508
GXYLT1	0	5	1.000	0.000	0.000	30.501
H6PD	0	3	1.000	0.000	0.000	64.152
HACE1	0	3	1.000	0.000	0.000	64.152
HADHB	0	2	1.000	0.000	0.000	134.508
HAPLN3	0	2	1.000	0.000	0.000	134.508
HAS2	0	2	1.000	0.000	0.000	134.508
HAUS5	0	3	1.000	0.000	0.000	64.152
HAUS7	0	2	1.000	0.000	0.000	134.508
HAVCR1	0	2	1.000	0.000	0.000	134.508
HBS1L	0	2	1.000	0.000	0.000	134.508
HCN1	0	2	1.000	0.000	0.000	134.508
HCN4	0	3	1.000	0.000	0.000	64.152
HEATR1	0	4	1.000	0.000	0.000	41.489
HEATR2	0	2	1.000	0.000	0.000	134.508
HEATR3	0	2	1.000	0.000	0.000	134.508
HECTD1	0	2	1.000	0.000	0.000	134.508
HECTD2	0	2	1.000	0.000	0.000	134.508
HECTD4	0	2	1.000	0.000	0.000	134.508
HECW1	0	4	1.000	0.000	0.000	41.489
HELB	0	2	1.000	0.000	0.000	134.508
HELZ	0	2	1.000	0.000	0.000	134.508
HEPHL1	0	2	1.000	0.000	0.000	134.508
HERC1	0	5	1.000	0.000	0.000	30.501
HERC2	0	11	1.000	0.000	0.000	11.391
HERC3	0	2	1.000	0.000	0.000	134.508
HERC5	0	2	1.000	0.000	0.000	134.508
HEXIM1	0	2	1.000	0.000	0.000	134.508
HIF3A	0	2	1.000	0.000	0.000	134.508
HINFP	0	2	1.000	0.000	0.000	134.508
HIPK3	0	3	1.000	0.000	0.000	64.152
HIST1H1B	0	3	1.000	0.000	0.000	64.152
HIST1H2AC	0	2	1.000	0.000	0.000	134.508
HIST1H2BJ	0	2	1.000	0.000	0.000	134.508
HIST1H3I	0	2	1.000	0.000	0.000	134.508

HIVEP3	0	3	1.000	0.000	0.000	64.152
HK3	0	3	1.000	0.000	0.000	64.152
HLA-A	0	5	1.000	0.000	0.000	30.501
HLA-DQA2	0	5	1.000	0.000	0.000	30.501
HLA-DRB1	0	8	1.000	0.000	0.000	16.757
HLA-DRB5	0	2	1.000	0.000	0.000	134.508
HLA-G	0	2	1.000	0.000	0.000	134.508
HLCS	0	2	1.000	0.000	0.000	134.508
HLX	0	2	1.000	0.000	0.000	134.508
HMG20B	0	2	1.000	0.000	0.000	134.508
HNF1A	0	2	1.000	0.000	0.000	134.508
HNRNPCL1	0	2	1.000	0.000	0.000	134.508
HNRNPF	0	2	1.000	0.000	0.000	134.508
HNRNPH3	0	2	1.000	0.000	0.000	134.508
HNRNPR	0	3	1.000	0.000	0.000	64.152
HOXA3	0	3	1.000	0.000	0.000	64.152
HOXA4	0	2	1.000	0.000	0.000	134.508
HOXB3	0	4	1.000	0.000	0.000	41.489
HPR	0	4	1.000	0.000	0.000	41.489
HPS3	0	2	1.000	0.000	0.000	134.508
HPSE2	0	2	1.000	0.000	0.000	134.508
HR	0	2	1.000	0.000	0.000	134.508
HRASLS	0	2	1.000	0.000	0.000	134.508
HRASLS5	0	3	1.000	0.000	0.000	64.152
HRNR	0	9	1.000	0.000	0.000	14.507
HS3ST2	0	2	1.000	0.000	0.000	134.508
HSD17B13	0	2	1.000	0.000	0.000	134.508
HSD17B2	0	2	1.000	0.000	0.000	134.508
HSF2	0	2	1.000	0.000	0.000	134.508
HSF5	0	2	1.000	0.000	0.000	134.508
HSP90AA1	0	2	1.000	0.000	0.000	134.508
HSPA12A	0	2	1.000	0.000	0.000	134.508
HSPA5	0	2	1.000	0.000	0.000	134.508
HSPA9	0	2	1.000	0.000	0.000	134.508
HSPG2	0	2	1.000	0.000	0.000	134.508
HSPH1	0	2	1.000	0.000	0.000	134.508
HTATSF1	0	2	1.000	0.000	0.000	134.508
HTR2A	0	2	1.000	0.000	0.000	134.508
HTR2C	0	2	1.000	0.000	0.000	134.508
HTR3B	0	2	1.000	0.000	0.000	134.508
HTT	0	2	1.000	0.000	0.000	134.508
HYKK	0	2	1.000	0.000	0.000	134.508
IARS	0	2	1.000	0.000	0.000	134.508
IBSP	0	2	1.000	0.000	0.000	134.508
ICK	0	2	1.000	0.000	0.000	134.508
IFI44L	0	2	1.000	0.000	0.000	134.508
IFNAR1	0	2	1.000	0.000	0.000	134.508
IFNGR1	0	3	1.000	0.000	0.000	64.152
IFT140	0	2	1.000	0.000	0.000	134.508
IFT46	0	2	1.000	0.000	0.000	134.508
IFT80	0	3	1.000	0.000	0.000	64.152
IFT88	0	3	1.000	0.000	0.000	64.152
IGBP1	0	3	1.000	0.000	0.000	64.152
IGDCC4	0	2	1.000	0.000	0.000	134.508
IGF1R	0	2	1.000	0.000	0.000	134.508
IGF2BP2	0	2	1.000	0.000	0.000	134.508
IGF2R	0	3	1.000	0.000	0.000	64.152
IGSF1	0	3	1.000	0.000	0.000	64.152
IGSF10	0	5	1.000	0.000	0.000	30.501
IGSF3	0	3	1.000	0.000	0.000	64.152
IGSF9	0	2	1.000	0.000	0.000	134.508
IGSF9B	0	2	1.000	0.000	0.000	134.508
IKBKAP	0	3	1.000	0.000	0.000	64.152
IKBKB	0	2	1.000	0.000	0.000	134.508
IKBKE	0	2	1.000	0.000	0.000	134.508
IKZF2	0	2	1.000	0.000	0.000	134.508

IL1F10	0	2	1.000	0.000	0.000	134.508
IL20RA	0	2	1.000	0.000	0.000	134.508
IL21	0	2	1.000	0.000	0.000	134.508
IL4R	0	2	1.000	0.000	0.000	134.508
IL6ST	0	3	1.000	0.000	0.000	64.152
ILF3	0	2	1.000	0.000	0.000	134.508
IMPDH1	0	2	1.000	0.000	0.000	134.508
IMPDH2	0	2	1.000	0.000	0.000	134.508
IMPG1	0	2	1.000	0.000	0.000	134.508
IMPG2	0	2	1.000	0.000	0.000	134.508
INADL	0	2	1.000	0.000	0.000	134.508
INCENP	0	2	1.000	0.000	0.000	134.508
ING1	0	2	1.000	0.000	0.000	134.508
INPP5A	0	2	1.000	0.000	0.000	134.508
INSR	0	3	1.000	0.000	0.000	64.152
INSRR	0	2	1.000	0.000	0.000	134.508
INTS1	0	3	1.000	0.000	0.000	64.152
INTS10	0	2	1.000	0.000	0.000	134.508
IPO5	0	4	1.000	0.000	0.000	41.489
IQGAP1	0	2	1.000	0.000	0.000	134.508
IQGAP3	0	2	1.000	0.000	0.000	134.508
IRAK3	0	2	1.000	0.000	0.000	134.508
IRS1	0	3	1.000	0.000	0.000	64.152
IRS4	0	2	1.000	0.000	0.000	134.508
IRX2	0	2	1.000	0.000	0.000	134.508
IRX4	0	2	1.000	0.000	0.000	134.508
IRX6	0	2	1.000	0.000	0.000	134.508
ISY1	0	2	1.000	0.000	0.000	134.508
ITGA1	0	2	1.000	0.000	0.000	134.508
ITGA4	0	2	1.000	0.000	0.000	134.508
ITGA6	0	2	1.000	0.000	0.000	134.508
ITGA7	0	3	1.000	0.000	0.000	64.152
ITGAD	0	2	1.000	0.000	0.000	134.508
ITGAE	0	2	1.000	0.000	0.000	134.508
ITGAM	0	2	1.000	0.000	0.000	134.508
ITGAX	0	2	1.000	0.000	0.000	134.508
ITGB2	0	2	1.000	0.000	0.000	134.508
ITGB4	0	3	1.000	0.000	0.000	64.152
ITGB8	0	2	1.000	0.000	0.000	134.508
ITIH5	0	2	1.000	0.000	0.000	134.508
ITK	0	3	1.000	0.000	0.000	64.152
ITLN2	0	2	1.000	0.000	0.000	134.508
ITPKB	0	2	1.000	0.000	0.000	134.508
ITPR1	0	5	1.000	0.000	0.000	30.501
ITPR2	0	4	1.000	0.000	0.000	41.489
ITPRIP	0	2	1.000	0.000	0.000	134.508
ITSN1	0	2	1.000	0.000	0.000	134.508
ITSN2	0	4	1.000	0.000	0.000	41.489
IVL	0	2	1.000	0.000	0.000	134.508
JAGN1	0	2	1.000	0.000	0.000	134.508
JAKMIP1	0	4	1.000	0.000	0.000	41.489
JAKMIP2	0	2	1.000	0.000	0.000	134.508
JAM2	0	2	1.000	0.000	0.000	134.508
JARID2	0	2	1.000	0.000	0.000	134.508
JMJD1C	0	2	1.000	0.000	0.000	134.508
JPH1	0	3	1.000	0.000	0.000	64.152
JUP	0	2	1.000	0.000	0.000	134.508
KALRN	0	3	1.000	0.000	0.000	64.152
KANSL1	0	3	1.000	0.000	0.000	64.152
KAT6A	0	2	1.000	0.000	0.000	134.508
KBTBD2	0	2	1.000	0.000	0.000	134.508
KBTBD6	0	2	1.000	0.000	0.000	134.508
KCNA1	0	4	1.000	0.000	0.000	41.489
KCNA3	0	2	1.000	0.000	0.000	134.508
KCNA4	0	5	1.000	0.000	0.000	30.501
KCNA7	0	3	1.000	0.000	0.000	64.152

KCNC1	0	2	1.000	0.000	0.000	134.508
KCND1	0	2	1.000	0.000	0.000	134.508
KCNE4	0	2	1.000	0.000	0.000	134.508
KCNG3	0	3	1.000	0.000	0.000	64.152
KCNH1	0	3	1.000	0.000	0.000	64.152
KCNH3	0	4	1.000	0.000	0.000	41.489
KCNH4	0	3	1.000	0.000	0.000	64.152
KCNH5	0	3	1.000	0.000	0.000	64.152
KCNH6	0	4	1.000	0.000	0.000	41.489
KCNH7	0	3	1.000	0.000	0.000	64.152
KCNH8	0	2	1.000	0.000	0.000	134.508
KCNJ12	0	5	1.000	0.000	0.000	30.501
KCNJ15	0	2	1.000	0.000	0.000	134.508
KCNJ2	0	2	1.000	0.000	0.000	134.508
KCNK1	0	2	1.000	0.000	0.000	134.508
KCNK3	0	2	1.000	0.000	0.000	134.508
KCNK6	0	2	1.000	0.000	0.000	134.508
KCNMA1	0	3	1.000	0.000	0.000	64.152
KCNN1	0	2	1.000	0.000	0.000	134.508
KCNQ2	0	3	1.000	0.000	0.000	64.152
KCNQ5	0	2	1.000	0.000	0.000	134.508
KCNS3	0	2	1.000	0.000	0.000	134.508
KCNT1	0	2	1.000	0.000	0.000	134.508
KCNT2	0	3	1.000	0.000	0.000	64.152
KCNU1	0	2	1.000	0.000	0.000	134.508
KCTD10	0	2	1.000	0.000	0.000	134.508
KCTD11	0	2	1.000	0.000	0.000	134.508
KDM2B	0	2	1.000	0.000	0.000	134.508
KDM3A	0	2	1.000	0.000	0.000	134.508
KDM4C	0	2	1.000	0.000	0.000	134.508
KDM5A	0	2	1.000	0.000	0.000	134.508
KDM5B	0	2	1.000	0.000	0.000	134.508
KDM6A	0	4	1.000	0.000	0.000	41.489
KDM6B	0	3	1.000	0.000	0.000	64.152
KEAP1	0	2	1.000	0.000	0.000	134.508
KHDRBS2	0	2	1.000	0.000	0.000	134.508
KHNYN	0	2	1.000	0.000	0.000	134.508
KIAA0100	0	3	1.000	0.000	0.000	64.152
KIAA0226	0	2	1.000	0.000	0.000	134.508
KIAA0226L	0	3	1.000	0.000	0.000	64.152
KIAA0355	0	2	1.000	0.000	0.000	134.508
KIAA0408	0	2	1.000	0.000	0.000	134.508
KIAA0556	0	3	1.000	0.000	0.000	64.152
KIAA0586	0	4	1.000	0.000	0.000	41.489
KIAA0895	0	2	1.000	0.000	0.000	134.508
KIAA0947	0	3	1.000	0.000	0.000	64.152
KIAA1045	0	3	1.000	0.000	0.000	64.152
KIAA1210	0	3	1.000	0.000	0.000	64.152
KIAA1211	0	3	1.000	0.000	0.000	64.152
KIAA1324	0	2	1.000	0.000	0.000	134.508
KIAA1377	0	2	1.000	0.000	0.000	134.508
KIAA1432	0	3	1.000	0.000	0.000	64.152
KIAA1462	0	3	1.000	0.000	0.000	64.152
KIAA1468	0	3	1.000	0.000	0.000	64.152
KIAA1524	0	2	1.000	0.000	0.000	134.508
KIAA1549	0	2	1.000	0.000	0.000	134.508
KIAA1755	0	2	1.000	0.000	0.000	134.508
KIAA1804	0	2	1.000	0.000	0.000	134.508
KIAA2022	0	2	1.000	0.000	0.000	134.508
KIDINS220	0	2	1.000	0.000	0.000	134.508
KIF15	0	2	1.000	0.000	0.000	134.508
KIF16B	0	2	1.000	0.000	0.000	134.508
KIF17	0	3	1.000	0.000	0.000	64.152
KIF1A	0	3	1.000	0.000	0.000	64.152
KIF1C	0	2	1.000	0.000	0.000	134.508
KIF23	0	3	1.000	0.000	0.000	64.152

KIF26A	0	3	1.000	0.000	0.000	64.152
KIF27	0	3	1.000	0.000	0.000	64.152
KIF3C	0	2	1.000	0.000	0.000	134.508
KIF4A	0	3	1.000	0.000	0.000	64.152
KIF4B	0	3	1.000	0.000	0.000	64.152
KIF5A	0	2	1.000	0.000	0.000	134.508
KIF5B	0	3	1.000	0.000	0.000	64.152
KIFAP3	0	3	1.000	0.000	0.000	64.152
KIFC3	0	2	1.000	0.000	0.000	134.508
KIR3DX1	0	2	1.000	0.000	0.000	134.508
KIRREL3	0	2	1.000	0.000	0.000	134.508
KLB	0	2	1.000	0.000	0.000	134.508
KLF4	0	2	1.000	0.000	0.000	134.508
KLF5	0	2	1.000	0.000	0.000	134.508
KLHDC2	0	2	1.000	0.000	0.000	134.508
KLHDC7A	0	2	1.000	0.000	0.000	134.508
KLHDC8A	0	3	1.000	0.000	0.000	64.152
KLHL31	0	2	1.000	0.000	0.000	134.508
KLHL32	0	2	1.000	0.000	0.000	134.508
KLHL36	0	2	1.000	0.000	0.000	134.508
KLK15	0	4	1.000	0.000	0.000	41.489
KLRC3	0	2	1.000	0.000	0.000	134.508
KMT2A	0	4	1.000	0.000	0.000	41.489
KMT2B	0	7	1.000	0.000	0.000	19.777
KNDC1	0	4	1.000	0.000	0.000	41.489
KPRP	0	2	1.000	0.000	0.000	134.508
KRIT1	0	2	1.000	0.000	0.000	134.508
KRT24	0	3	1.000	0.000	0.000	64.152
KRT31	0	2	1.000	0.000	0.000	134.508
KRT32	0	2	1.000	0.000	0.000	134.508
KRT34	0	2	1.000	0.000	0.000	134.508
KRT37	0	2	1.000	0.000	0.000	134.508
KRT38	0	2	1.000	0.000	0.000	134.508
KRT40	0	2	1.000	0.000	0.000	134.508
KRT6A	0	2	1.000	0.000	0.000	134.508
KRT74	0	2	1.000	0.000	0.000	134.508
KRT75	0	2	1.000	0.000	0.000	134.508
KRT77	0	2	1.000	0.000	0.000	134.508
KRT8	0	3	1.000	0.000	0.000	64.152
KRT83	0	3	1.000	0.000	0.000	64.152
KRT86	0	5	1.000	0.000	0.000	30.501
KRTAP12-2	0	3	1.000	0.000	0.000	64.152
KRTAP12-3	0	2	1.000	0.000	0.000	134.508
KRTAP24-1	0	2	1.000	0.000	0.000	134.508
KRTAP4-3	0	2	1.000	0.000	0.000	134.508
KRTAP4-5	0	2	1.000	0.000	0.000	134.508
KRTAP4-7	0	4	1.000	0.000	0.000	41.489
KRTAP4-8	0	2	1.000	0.000	0.000	134.508
KRTDAP	0	2	1.000	0.000	0.000	134.508
KSR2	0	2	1.000	0.000	0.000	134.508
KYNU	0	3	1.000	0.000	0.000	64.152
L1CAM	0	2	1.000	0.000	0.000	134.508
L3HYPDH	0	2	1.000	0.000	0.000	134.508
L3MBTL1	0	2	1.000	0.000	0.000	134.508
L3MBTL2	0	2	1.000	0.000	0.000	134.508
L3MBTL4	0	2	1.000	0.000	0.000	134.508
LAMA1	0	5	1.000	0.000	0.000	30.501
LAMA4	0	3	1.000	0.000	0.000	64.152
LAMB2	0	2	1.000	0.000	0.000	134.508
LAMB3	0	3	1.000	0.000	0.000	64.152
LAMP2	0	2	1.000	0.000	0.000	134.508
LAPTM4B	0	3	1.000	0.000	0.000	64.152
LARP4	0	3	1.000	0.000	0.000	64.152
LARP4B	0	2	1.000	0.000	0.000	134.508
LATS1	0	3	1.000	0.000	0.000	64.152
LCMT1	0	2	1.000	0.000	0.000	134.508

LCN8	0	2	1.000	0.000	0.000	134.508
LCP1	0	3	1.000	0.000	0.000	64.152
LCT	0	3	1.000	0.000	0.000	64.152
LDB1	0	2	1.000	0.000	0.000	134.508
LDLRAD4	0	2	1.000	0.000	0.000	134.508
LEF1	0	2	1.000	0.000	0.000	134.508
LEPR	0	3	1.000	0.000	0.000	64.152
LEPRE1	0	2	1.000	0.000	0.000	134.508
LHCGR	0	2	1.000	0.000	0.000	134.508
LHX3	0	2	1.000	0.000	0.000	134.508
LHX8	0	2	1.000	0.000	0.000	134.508
LIF	0	2	1.000	0.000	0.000	134.508
LILRA1	0	3	1.000	0.000	0.000	64.152
LILRA3	0	3	1.000	0.000	0.000	64.152
LILRA6	0	2	1.000	0.000	0.000	134.508
LILRB3	0	2	1.000	0.000	0.000	134.508
LIMCH1	0	2	1.000	0.000	0.000	134.508
LIN54	0	2	1.000	0.000	0.000	134.508
LIN9	0	3	1.000	0.000	0.000	64.152
LMBR1	0	2	1.000	0.000	0.000	134.508
LMO7	0	3	1.000	0.000	0.000	64.152
LMOD1	0	2	1.000	0.000	0.000	134.508
LMTK2	0	2	1.000	0.000	0.000	134.508
LNPEP	0	2	1.000	0.000	0.000	134.508
LNX2	0	2	1.000	0.000	0.000	134.508
LONP1	0	2	1.000	0.000	0.000	134.508
LPA	0	2	1.000	0.000	0.000	134.508
LPCAT3	0	2	1.000	0.000	0.000	134.508
LPHN1	0	2	1.000	0.000	0.000	134.508
LPHN2	0	3	1.000	0.000	0.000	64.152
LPIN1	0	2	1.000	0.000	0.000	134.508
LPO	0	2	1.000	0.000	0.000	134.508
LPP	0	2	1.000	0.000	0.000	134.508
LPPR4	0	2	1.000	0.000	0.000	134.508
LRBA	0	3	1.000	0.000	0.000	64.152
LRCH3	0	2	1.000	0.000	0.000	134.508
LRFN1	0	2	1.000	0.000	0.000	134.508
LRFN5	0	4	1.000	0.000	0.000	41.489
LRIF1	0	2	1.000	0.000	0.000	134.508
LRIG1	0	2	1.000	0.000	0.000	134.508
LRIG2	0	3	1.000	0.000	0.000	64.152
LRIG3	0	2	1.000	0.000	0.000	134.508
LRIT1	0	2	1.000	0.000	0.000	134.508
LRIT3	0	2	1.000	0.000	0.000	134.508
LRMP	0	2	1.000	0.000	0.000	134.508
LRP1	0	5	1.000	0.000	0.000	30.501
LRP1B	0	5	1.000	0.000	0.000	30.501
LRP2	0	3	1.000	0.000	0.000	64.152
LRP4	0	4	1.000	0.000	0.000	41.489
LRP5	0	3	1.000	0.000	0.000	64.152
LRP6	0	3	1.000	0.000	0.000	64.152
LRRC25	0	2	1.000	0.000	0.000	134.508
LRRC43	0	2	1.000	0.000	0.000	134.508
LRRC55	0	2	1.000	0.000	0.000	134.508
LRRC7	0	4	1.000	0.000	0.000	41.489
LRRC8B	0	2	1.000	0.000	0.000	134.508
LRRC8D	0	2	1.000	0.000	0.000	134.508
LRRCC1	0	3	1.000	0.000	0.000	64.152
LRRIQ1	0	3	1.000	0.000	0.000	64.152
LRRIQ3	0	2	1.000	0.000	0.000	134.508
LRRIQ4	0	2	1.000	0.000	0.000	134.508
LRRK2	0	3	1.000	0.000	0.000	64.152
LRRN2	0	2	1.000	0.000	0.000	134.508
LRRN3	0	2	1.000	0.000	0.000	134.508
LSM11	0	2	1.000	0.000	0.000	134.508
LTB4R	0	2	1.000	0.000	0.000	134.508

LTB4R2	0	2	1.000	0.000	0.000	134.508
LTBP2	0	3	1.000	0.000	0.000	64.152
LTBP4	0	2	1.000	0.000	0.000	134.508
LTN1	0	2	1.000	0.000	0.000	134.508
LUC7L2	0	3	1.000	0.000	0.000	64.152
LUM	0	2	1.000	0.000	0.000	134.508
LUZP1	0	2	1.000	0.000	0.000	134.508
LY75	0	2	1.000	0.000	0.000	134.508
LYPD5	0	2	1.000	0.000	0.000	134.508
LYST	0	2	1.000	0.000	0.000	134.508
LYZL2	0	2	1.000	0.000	0.000	134.508
MACF1	0	8	1.000	0.000	0.000	16.757
MAEA	0	2	1.000	0.000	0.000	134.508
MAGEA8	0	3	1.000	0.000	0.000	64.152
MAGEB6	0	4	1.000	0.000	0.000	41.489
MAGEC1	0	3	1.000	0.000	0.000	64.152
MAGEL2	0	2	1.000	0.000	0.000	134.508
MAGI2	0	3	1.000	0.000	0.000	64.152
MAK	0	2	1.000	0.000	0.000	134.508
MAMDC2	0	2	1.000	0.000	0.000	134.508
MAN2A1	0	2	1.000	0.000	0.000	134.508
MAN2B2	0	3	1.000	0.000	0.000	64.152
MAP10	0	2	1.000	0.000	0.000	134.508
MAP1B	0	2	1.000	0.000	0.000	134.508
MAP1LC3A	0	2	1.000	0.000	0.000	134.508
MAP2	0	3	1.000	0.000	0.000	64.152
MAP2K3	0	2	1.000	0.000	0.000	134.508
MAP2K4	0	4	1.000	0.000	0.000	41.489
MAP2K7	0	2	1.000	0.000	0.000	134.508
MAP3K1	0	2	1.000	0.000	0.000	134.508
MAP3K13	0	2	1.000	0.000	0.000	134.508
MAP3K15	0	2	1.000	0.000	0.000	134.508
MAP3K4	0	2	1.000	0.000	0.000	134.508
MAP3K7CL	0	2	1.000	0.000	0.000	134.508
MAP3K9	0	2	1.000	0.000	0.000	134.508
MAP4	0	2	1.000	0.000	0.000	134.508
MAP4K1	0	2	1.000	0.000	0.000	134.508
MAP4K2	0	2	1.000	0.000	0.000	134.508
MAP4K3	0	2	1.000	0.000	0.000	134.508
MAP6	0	2	1.000	0.000	0.000	134.508
MAPK12	0	2	1.000	0.000	0.000	134.508
MAPK13	0	2	1.000	0.000	0.000	134.508
MAPK6	0	2	1.000	0.000	0.000	134.508
MAPK8IP3	0	2	1.000	0.000	0.000	134.508
MAPT	0	3	1.000	0.000	0.000	64.152
6-Mar	0	3	1.000	0.000	0.000	64.152
MARCO	0	2	1.000	0.000	0.000	134.508
MAST1	0	2	1.000	0.000	0.000	134.508
MAST3	0	2	1.000	0.000	0.000	134.508
MATN4	0	2	1.000	0.000	0.000	134.508
MAZ	0	2	1.000	0.000	0.000	134.508
MBD1	0	2	1.000	0.000	0.000	134.508
MBTD1	0	3	1.000	0.000	0.000	64.152
MC3R	0	2	1.000	0.000	0.000	134.508
MC5R	0	3	1.000	0.000	0.000	64.152
MCCC1	0	2	1.000	0.000	0.000	134.508
MCEE	0	2	1.000	0.000	0.000	134.508
MCF2L	0	3	1.000	0.000	0.000	64.152
MCF2L2	0	3	1.000	0.000	0.000	64.152
MCHR1	0	2	1.000	0.000	0.000	134.508
MCHR2	0	2	1.000	0.000	0.000	134.508
MCM2	0	2	1.000	0.000	0.000	134.508
MCM3AP	0	2	1.000	0.000	0.000	134.508
MCMBP	0	2	1.000	0.000	0.000	134.508
MCOLN1	0	2	1.000	0.000	0.000	134.508
MCPH1	0	2	1.000	0.000	0.000	134.508

MDGA1	0	2	1.000	0.000	0.000	134.508
MDGA2	0	2	1.000	0.000	0.000	134.508
MDH1B	0	2	1.000	0.000	0.000	134.508
MDM1	0	3	1.000	0.000	0.000	64.152
MDM2	0	2	1.000	0.000	0.000	134.508
ME2	0	2	1.000	0.000	0.000	134.508
MECOM	0	2	1.000	0.000	0.000	134.508
MECR	0	2	1.000	0.000	0.000	134.508
MED1	0	2	1.000	0.000	0.000	134.508
MED12	0	2	1.000	0.000	0.000	134.508
MED12L	0	4	1.000	0.000	0.000	41.489
MED13L	0	3	1.000	0.000	0.000	64.152
MED14	0	2	1.000	0.000	0.000	134.508
MED15	0	2	1.000	0.000	0.000	134.508
MED16	0	2	1.000	0.000	0.000	134.508
MEF2A	0	2	1.000	0.000	0.000	134.508
MEF2C	0	2	1.000	0.000	0.000	134.508
MEF2D	0	2	1.000	0.000	0.000	134.508
MEFV	0	3	1.000	0.000	0.000	64.152
MEGF10	0	2	1.000	0.000	0.000	134.508
MEGF8	0	4	1.000	0.000	0.000	41.489
MELK	0	3	1.000	0.000	0.000	64.152
MEOX1	0	2	1.000	0.000	0.000	134.508
MEP1A	0	2	1.000	0.000	0.000	134.508
MERTK	0	2	1.000	0.000	0.000	134.508
MET	0	2	1.000	0.000	0.000	134.508
METAP1	0	2	1.000	0.000	0.000	134.508
METTL13	0	2	1.000	0.000	0.000	134.508
METTL25	0	2	1.000	0.000	0.000	134.508
METTL3	0	2	1.000	0.000	0.000	134.508
MEX3C	0	2	1.000	0.000	0.000	134.508
MFAP3L	0	2	1.000	0.000	0.000	134.508
MFN1	0	2	1.000	0.000	0.000	134.508
MFSD7	0	2	1.000	0.000	0.000	134.508
MGA	0	2	1.000	0.000	0.000	134.508
MGAM	0	4	1.000	0.000	0.000	41.489
MGAT1	0	2	1.000	0.000	0.000	134.508
MGAT5	0	2	1.000	0.000	0.000	134.508
MIB1	0	2	1.000	0.000	0.000	134.508
MICAL2	0	3	1.000	0.000	0.000	64.152
MICALCL	0	3	1.000	0.000	0.000	64.152
MISP	0	2	1.000	0.000	0.000	134.508
MIXL1	0	2	1.000	0.000	0.000	134.508
MKI67	0	4	1.000	0.000	0.000	41.489
MKL2	0	3	1.000	0.000	0.000	64.152
MKLN1	0	2	1.000	0.000	0.000	134.508
MLH1	0	2	1.000	0.000	0.000	134.508
MLH3	0	2	1.000	0.000	0.000	134.508
MLKL	0	3	1.000	0.000	0.000	64.152
MLLT10	0	2	1.000	0.000	0.000	134.508
MLN	0	2	1.000	0.000	0.000	134.508
MLPH	0	2	1.000	0.000	0.000	134.508
MMP14	0	2	1.000	0.000	0.000	134.508
MMP16	0	3	1.000	0.000	0.000	64.152
MMP21	0	2	1.000	0.000	0.000	134.508
MMS19	0	2	1.000	0.000	0.000	134.508
MMS22L	0	2	1.000	0.000	0.000	134.508
MOB3A	0	2	1.000	0.000	0.000	134.508
MOCS1	0	2	1.000	0.000	0.000	134.508
MOGS	0	2	1.000	0.000	0.000	134.508
MOK	0	3	1.000	0.000	0.000	64.152
MON2	0	2	1.000	0.000	0.000	134.508
MOS	0	2	1.000	0.000	0.000	134.508
MOSPD2	0	2	1.000	0.000	0.000	134.508
MOV10L1	0	3	1.000	0.000	0.000	64.152
MPDZ	0	2	1.000	0.000	0.000	134.508

MPHOSPH8	0	3	1.000	0.000	0.000	64.152
MPO	0	2	1.000	0.000	0.000	134.508
MPP2	0	2	1.000	0.000	0.000	134.508
MRGPRX1	0	2	1.000	0.000	0.000	134.508
MROH7	0	4	1.000	0.000	0.000	41.489
MROH8	0	2	1.000	0.000	0.000	134.508
MROH9	0	2	1.000	0.000	0.000	134.508
MRPL15	0	2	1.000	0.000	0.000	134.508
MRPS36	0	2	1.000	0.000	0.000	134.508
MSGN1	0	2	1.000	0.000	0.000	134.508
MSI2	0	2	1.000	0.000	0.000	134.508
MSRB3	0	2	1.000	0.000	0.000	134.508
MST1	0	10	1.000	0.000	0.000	12.777
MST1L	0	5	1.000	0.000	0.000	30.501
MT1M	0	2	1.000	0.000	0.000	134.508
MTFR1	0	2	1.000	0.000	0.000	134.508
MTG1	0	2	1.000	0.000	0.000	134.508
MTG2	0	2	1.000	0.000	0.000	134.508
MTHFD1L	0	3	1.000	0.000	0.000	64.152
MTMR12	0	3	1.000	0.000	0.000	64.152
MTMR4	0	2	1.000	0.000	0.000	134.508
MTMR7	0	2	1.000	0.000	0.000	134.508
MTOR	0	3	1.000	0.000	0.000	64.152
MTPAP	0	2	1.000	0.000	0.000	134.508
MTRR	0	2	1.000	0.000	0.000	134.508
MTUS1	0	2	1.000	0.000	0.000	134.508
MTUS2	0	3	1.000	0.000	0.000	64.152
MUC17	0	7	1.000	0.000	0.000	19.777
MUC2	0	3	1.000	0.000	0.000	64.152
MUC20	0	2	1.000	0.000	0.000	134.508
MUC4	0	2	1.000	0.000	0.000	134.508
MUC5AC	0	2	1.000	0.000	0.000	134.508
MUC6	0	7	1.000	0.000	0.000	19.777
MUS81	0	2	1.000	0.000	0.000	134.508
MVB12B	0	3	1.000	0.000	0.000	64.152
MYADM	0	2	1.000	0.000	0.000	134.508
MYCN	0	2	1.000	0.000	0.000	134.508
MYEF2	0	4	1.000	0.000	0.000	41.489
MYH1	0	3	1.000	0.000	0.000	64.152
MYH10	0	3	1.000	0.000	0.000	64.152
MYH11	0	3	1.000	0.000	0.000	64.152
MYH13	0	3	1.000	0.000	0.000	64.152
MYH14	0	2	1.000	0.000	0.000	134.508
MYH15	0	4	1.000	0.000	0.000	41.489
MYH3	0	2	1.000	0.000	0.000	134.508
MYH4	0	4	1.000	0.000	0.000	41.489
MYH6	0	2	1.000	0.000	0.000	134.508
MYH7	0	4	1.000	0.000	0.000	41.489
MYH7B	0	3	1.000	0.000	0.000	64.152
MYH9	0	2	1.000	0.000	0.000	134.508
MYLK	0	4	1.000	0.000	0.000	41.489
MYO10	0	2	1.000	0.000	0.000	134.508
MYO15A	0	3	1.000	0.000	0.000	64.152
MYO16	0	5	1.000	0.000	0.000	30.501
MYO18B	0	5	1.000	0.000	0.000	30.501
MYO1C	0	3	1.000	0.000	0.000	64.152
MYO1D	0	2	1.000	0.000	0.000	134.508
MYO1E	0	2	1.000	0.000	0.000	134.508
MYO3B	0	3	1.000	0.000	0.000	64.152
MYO5A	0	2	1.000	0.000	0.000	134.508
MYO6	0	2	1.000	0.000	0.000	134.508
MYO7A	0	2	1.000	0.000	0.000	134.508
MYO7B	0	2	1.000	0.000	0.000	134.508
MYO9A	0	4	1.000	0.000	0.000	41.489
MYO9B	0	2	1.000	0.000	0.000	134.508
MYOG	0	2	1.000	0.000	0.000	134.508

MYOM2	0	2	1.000	0.000	0.000	134.508
MYOT	0	3	1.000	0.000	0.000	64.152
MYRF	0	3	1.000	0.000	0.000	64.152
MYRIP	0	2	1.000	0.000	0.000	134.508
MYT1	0	2	1.000	0.000	0.000	134.508
MYT1L	0	2	1.000	0.000	0.000	134.508
N4BP2	0	2	1.000	0.000	0.000	134.508
N4BP2L2	0	2	1.000	0.000	0.000	134.508
NAA16	0	3	1.000	0.000	0.000	64.152
NAA25	0	3	1.000	0.000	0.000	64.152
NAA60	0	2	1.000	0.000	0.000	134.508
NAAA	0	2	1.000	0.000	0.000	134.508
NAALAD2	0	3	1.000	0.000	0.000	64.152
NACA	0	3	1.000	0.000	0.000	64.152
NACC2	0	2	1.000	0.000	0.000	134.508
NADSYN1	0	2	1.000	0.000	0.000	134.508
NAGLU	0	2	1.000	0.000	0.000	134.508
NALCN	0	2	1.000	0.000	0.000	134.508
NAP1L3	0	2	1.000	0.000	0.000	134.508
NARG2	0	2	1.000	0.000	0.000	134.508
NASP	0	7	1.000	0.000	0.000	19.777
NBAS	0	2	1.000	0.000	0.000	134.508
NBEA	0	2	1.000	0.000	0.000	134.508
NBEAL1	0	2	1.000	0.000	0.000	134.508
NBEAL2	0	2	1.000	0.000	0.000	134.508
NCAM1	0	2	1.000	0.000	0.000	134.508
NCAPD2	0	2	1.000	0.000	0.000	134.508
NCAPG	0	2	1.000	0.000	0.000	134.508
NCAPH	0	3	1.000	0.000	0.000	64.152
NCKAP1	0	2	1.000	0.000	0.000	134.508
NCKAP5L	0	2	1.000	0.000	0.000	134.508
NCKIPSD	0	2	1.000	0.000	0.000	134.508
NCOA2	0	2	1.000	0.000	0.000	134.508
NCOA3	0	2	1.000	0.000	0.000	134.508
NCOR1	0	3	1.000	0.000	0.000	64.152
NDST3	0	3	1.000	0.000	0.000	64.152
NDUFS1	0	2	1.000	0.000	0.000	134.508
NEB	0	4	1.000	0.000	0.000	41.489
NEBL	0	2	1.000	0.000	0.000	134.508
NEK10	0	2	1.000	0.000	0.000	134.508
NEK5	0	2	1.000	0.000	0.000	134.508
NELFA	0	2	1.000	0.000	0.000	134.508
NELFB	0	2	1.000	0.000	0.000	134.508
NELL1	0	2	1.000	0.000	0.000	134.508
NELL2	0	3	1.000	0.000	0.000	64.152
NEMF	0	2	1.000	0.000	0.000	134.508
NES	0	2	1.000	0.000	0.000	134.508
NEURL4	0	3	1.000	0.000	0.000	64.152
NEUROD6	0	2	1.000	0.000	0.000	134.508
NEXN	0	2	1.000	0.000	0.000	134.508
NFASC	0	2	1.000	0.000	0.000	134.508
NFAT5	0	2	1.000	0.000	0.000	134.508
NFATC1	0	2	1.000	0.000	0.000	134.508
NFATC2	0	2	1.000	0.000	0.000	134.508
NFE2L1	0	2	1.000	0.000	0.000	134.508
NFKBIZ	0	4	1.000	0.000	0.000	41.489
NGF	0	2	1.000	0.000	0.000	134.508
NGLY1	0	2	1.000	0.000	0.000	134.508
NHLRC3	0	2	1.000	0.000	0.000	134.508
NID1	0	2	1.000	0.000	0.000	134.508
NINL	0	2	1.000	0.000	0.000	134.508
NIPA2	0	2	1.000	0.000	0.000	134.508
NIPBL	0	2	1.000	0.000	0.000	134.508
NKD1	0	2	1.000	0.000	0.000	134.508
NKX2-4	0	3	1.000	0.000	0.000	64.152
NLGN1	0	3	1.000	0.000	0.000	64.152

NLK	0	2	1.000	0.000	0.000	134.508
NLRC4	0	2	1.000	0.000	0.000	134.508
NLRC5	0	4	1.000	0.000	0.000	41.489
NLRP10	0	3	1.000	0.000	0.000	64.152
NLRP12	0	3	1.000	0.000	0.000	64.152
NLRP13	0	2	1.000	0.000	0.000	134.508
NLRP2	0	3	1.000	0.000	0.000	64.152
NLRP3	0	2	1.000	0.000	0.000	134.508
NLRP4	0	3	1.000	0.000	0.000	64.152
NLRP8	0	5	1.000	0.000	0.000	30.501
NMD3	0	2	1.000	0.000	0.000	134.508
NMS	0	2	1.000	0.000	0.000	134.508
NOBOX	0	3	1.000	0.000	0.000	64.152
NOC4L	0	2	1.000	0.000	0.000	134.508
NOL4	0	2	1.000	0.000	0.000	134.508
NOM1	0	2	1.000	0.000	0.000	134.508
NOS1	0	5	1.000	0.000	0.000	30.501
NOS3	0	3	1.000	0.000	0.000	64.152
NOTCH1	0	2	1.000	0.000	0.000	134.508
NOTCH2	0	2	1.000	0.000	0.000	134.508
NOTCH3	0	2	1.000	0.000	0.000	134.508
NOTCH4	0	3	1.000	0.000	0.000	64.152
NOVA1	0	2	1.000	0.000	0.000	134.508
NOX1	0	2	1.000	0.000	0.000	134.508
NPAP1	0	4	1.000	0.000	0.000	41.489
NPAS2	0	2	1.000	0.000	0.000	134.508
NPAS3	0	4	1.000	0.000	0.000	41.489
NPAS4	0	2	1.000	0.000	0.000	134.508
NPAT	0	4	1.000	0.000	0.000	41.489
NPC1	0	4	1.000	0.000	0.000	41.489
NPHP1	0	2	1.000	0.000	0.000	134.508
NPHP3	0	3	1.000	0.000	0.000	64.152
NPHS1	0	2	1.000	0.000	0.000	134.508
NPHS2	0	2	1.000	0.000	0.000	134.508
NPY1R	0	3	1.000	0.000	0.000	64.152
NR0B1	0	2	1.000	0.000	0.000	134.508
NR5A1	0	2	1.000	0.000	0.000	134.508
NRG1	0	3	1.000	0.000	0.000	64.152
NRIP3	0	2	1.000	0.000	0.000	134.508
NRK	0	3	1.000	0.000	0.000	64.152
NRN1	0	2	1.000	0.000	0.000	134.508
NRSN1	0	2	1.000	0.000	0.000	134.508
NRXN1	0	2	1.000	0.000	0.000	134.508
NRXN2	0	2	1.000	0.000	0.000	134.508
NRXN3	0	3	1.000	0.000	0.000	64.152
NSD1	0	2	1.000	0.000	0.000	134.508
NSDHL	0	2	1.000	0.000	0.000	134.508
NSMCE2	0	2	1.000	0.000	0.000	134.508
NSUN4	0	4	1.000	0.000	0.000	41.489
NT5DC3	0	2	1.000	0.000	0.000	134.508
NTF3	0	4	1.000	0.000	0.000	41.489
NTM	0	2	1.000	0.000	0.000	134.508
NTNG1	0	2	1.000	0.000	0.000	134.508
NTRK1	0	2	1.000	0.000	0.000	134.508
NUAK1	0	3	1.000	0.000	0.000	64.152
NUAK2	0	2	1.000	0.000	0.000	134.508
NUDT17	0	2	1.000	0.000	0.000	134.508
NUDT18	0	2	1.000	0.000	0.000	134.508
NUDT6	0	2	1.000	0.000	0.000	134.508
NUP155	0	2	1.000	0.000	0.000	134.508
NUP160	0	2	1.000	0.000	0.000	134.508
NUP205	0	3	1.000	0.000	0.000	64.152
NUP210L	0	3	1.000	0.000	0.000	64.152
NUP214	0	2	1.000	0.000	0.000	134.508
NUP37	0	2	1.000	0.000	0.000	134.508
NUP50	0	2	1.000	0.000	0.000	134.508

NUP98	0	3	1.000	0.000	0.000	64.152
NUPL2	0	2	1.000	0.000	0.000	134.508
NVL	0	2	1.000	0.000	0.000	134.508
NWD1	0	2	1.000	0.000	0.000	134.508
NXPH1	0	2	1.000	0.000	0.000	134.508
NYAP2	0	3	1.000	0.000	0.000	64.152
NYNRIN	0	3	1.000	0.000	0.000	64.152
OARD1	0	2	1.000	0.000	0.000	134.508
OAT	0	2	1.000	0.000	0.000	134.508
OBSCN	0	12	1.000	0.000	0.000	10.263
OBSL1	0	4	1.000	0.000	0.000	41.489
OCA2	0	2	1.000	0.000	0.000	134.508
OCRL	0	2	1.000	0.000	0.000	134.508
ODF1	0	2	1.000	0.000	0.000	134.508
ODF2	0	2	1.000	0.000	0.000	134.508
OGDH	0	2	1.000	0.000	0.000	134.508
OGFR	0	2	1.000	0.000	0.000	134.508
OGT	0	2	1.000	0.000	0.000	134.508
OLFM1	0	2	1.000	0.000	0.000	134.508
OLFML2B	0	3	1.000	0.000	0.000	64.152
OLIG3	0	2	1.000	0.000	0.000	134.508
ONECUT2	0	2	1.000	0.000	0.000	134.508
OOEP	0	2	1.000	0.000	0.000	134.508
OPA1	0	2	1.000	0.000	0.000	134.508
OPCML	0	2	1.000	0.000	0.000	134.508
OPN1LW	0	3	1.000	0.000	0.000	64.152
OR10C1	0	3	1.000	0.000	0.000	64.152
OR10G9	0	4	1.000	0.000	0.000	41.489
OR10H3	0	2	1.000	0.000	0.000	134.508
OR10J1	0	2	1.000	0.000	0.000	134.508
OR10J3	0	2	1.000	0.000	0.000	134.508
OR10Q1	0	2	1.000	0.000	0.000	134.508
OR10S1	0	2	1.000	0.000	0.000	134.508
OR11H12	0	2	1.000	0.000	0.000	134.508
OR11H6	0	2	1.000	0.000	0.000	134.508
OR12D2	0	2	1.000	0.000	0.000	134.508
OR13F1	0	3	1.000	0.000	0.000	64.152
OR13H1	0	2	1.000	0.000	0.000	134.508
OR14K1	0	2	1.000	0.000	0.000	134.508
OR1C1	0	3	1.000	0.000	0.000	64.152
OR2A2	0	2	1.000	0.000	0.000	134.508
OR2AE1	0	2	1.000	0.000	0.000	134.508
OR2F1	0	2	1.000	0.000	0.000	134.508
OR2G2	0	2	1.000	0.000	0.000	134.508
OR2J1	0	2	1.000	0.000	0.000	134.508
OR2J3	0	4	1.000	0.000	0.000	41.489
OR2K2	0	3	1.000	0.000	0.000	64.152
OR2L3	0	3	1.000	0.000	0.000	64.152
OR2L8	0	3	1.000	0.000	0.000	64.152
OR2M4	0	2	1.000	0.000	0.000	134.508
OR2M5	0	3	1.000	0.000	0.000	64.152
OR2S2	0	2	1.000	0.000	0.000	134.508
OR2T2	0	2	1.000	0.000	0.000	134.508
OR2T33	0	2	1.000	0.000	0.000	134.508
OR2T8	0	2	1.000	0.000	0.000	134.508
OR2V1	0	2	1.000	0.000	0.000	134.508
OR2W3	0	2	1.000	0.000	0.000	134.508
OR4A47	0	2	1.000	0.000	0.000	134.508
OR4C11	0	2	1.000	0.000	0.000	134.508
OR4C3	0	3	1.000	0.000	0.000	64.152
OR4D5	0	2	1.000	0.000	0.000	134.508
OR4D9	0	2	1.000	0.000	0.000	134.508
OR4N2	0	2	1.000	0.000	0.000	134.508
OR4N4	0	2	1.000	0.000	0.000	134.508
OR4Q3	0	3	1.000	0.000	0.000	64.152
OR4S2	0	4	1.000	0.000	0.000	41.489

OR51A2	0	2	1.000	0.000	0.000	134.508
OR51A7	0	2	1.000	0.000	0.000	134.508
OR51E1	0	2	1.000	0.000	0.000	134.508
OR51I2	0	2	1.000	0.000	0.000	134.508
OR51M1	0	2	1.000	0.000	0.000	134.508
OR51Q1	0	3	1.000	0.000	0.000	64.152
OR51T1	0	2	1.000	0.000	0.000	134.508
OR52A1	0	2	1.000	0.000	0.000	134.508
OR52E8	0	2	1.000	0.000	0.000	134.508
OR52I2	0	2	1.000	0.000	0.000	134.508
OR5A1	0	3	1.000	0.000	0.000	64.152
OR5B17	0	2	1.000	0.000	0.000	134.508
OR5D13	0	3	1.000	0.000	0.000	64.152
OR5F1	0	2	1.000	0.000	0.000	134.508
OR5H1	0	3	1.000	0.000	0.000	64.152
OR5L2	0	2	1.000	0.000	0.000	134.508
OR5M11	0	2	1.000	0.000	0.000	134.508
OR5M3	0	2	1.000	0.000	0.000	134.508
OR5P2	0	2	1.000	0.000	0.000	134.508
OR5P3	0	2	1.000	0.000	0.000	134.508
OR6C6	0	2	1.000	0.000	0.000	134.508
OR6K6	0	2	1.000	0.000	0.000	134.508
OR6S1	0	2	1.000	0.000	0.000	134.508
OR7D4	0	3	1.000	0.000	0.000	64.152
OR8I2	0	2	1.000	0.000	0.000	134.508
OR8J3	0	2	1.000	0.000	0.000	134.508
OR8U1	0	2	1.000	0.000	0.000	134.508
OSBPL11	0	2	1.000	0.000	0.000	134.508
OSBPL1A	0	3	1.000	0.000	0.000	64.152
OSBPL6	0	2	1.000	0.000	0.000	134.508
OSBPL8	0	2	1.000	0.000	0.000	134.508
OSBPL9	0	2	1.000	0.000	0.000	134.508
OSGIN1	0	2	1.000	0.000	0.000	134.508
OSMR	0	3	1.000	0.000	0.000	64.152
OSTF1	0	2	1.000	0.000	0.000	134.508
OTOF	0	5	1.000	0.000	0.000	30.501
OTOGL	0	2	1.000	0.000	0.000	134.508
OTOP3	0	2	1.000	0.000	0.000	134.508
OTUD4	0	2	1.000	0.000	0.000	134.508
OTX2	0	2	1.000	0.000	0.000	134.508
OVCH1	0	2	1.000	0.000	0.000	134.508
OVGP1	0	3	1.000	0.000	0.000	64.152
OXCT1	0	2	1.000	0.000	0.000	134.508
OXTR	0	2	1.000	0.000	0.000	134.508
P2RY13	0	2	1.000	0.000	0.000	134.508
P2RY2	0	2	1.000	0.000	0.000	134.508
P2RY4	0	2	1.000	0.000	0.000	134.508
P2RY8	0	3	1.000	0.000	0.000	64.152
P4HA1	0	4	1.000	0.000	0.000	41.489
PABPC1	0	4	1.000	0.000	0.000	41.489
PABPC3	0	8	1.000	0.000	0.000	16.757
PACSIN1	0	2	1.000	0.000	0.000	134.508
PADI3	0	2	1.000	0.000	0.000	134.508
PAG1	0	2	1.000	0.000	0.000	134.508
PAK2	0	5	1.000	0.000	0.000	30.501
PALD1	0	2	1.000	0.000	0.000	134.508
PALLD	0	5	1.000	0.000	0.000	30.501
PALM2-AKAP2	0	2	1.000	0.000	0.000	134.508
PANK4	0	2	1.000	0.000	0.000	134.508
PANX2	0	2	1.000	0.000	0.000	134.508
PAPLN	0	2	1.000	0.000	0.000	134.508
PAPOLB	0	2	1.000	0.000	0.000	134.508
PAPPA	0	2	1.000	0.000	0.000	134.508
PAQR9	0	2	1.000	0.000	0.000	134.508
PARK2	0	2	1.000	0.000	0.000	134.508
PARP1	0	2	1.000	0.000	0.000	134.508

PARP15	0	2	1.000	0.000	0.000	134.508
PARP4	0	10	1.000	0.000	0.000	12.777
PARP9	0	3	1.000	0.000	0.000	64.152
PARVA	0	2	1.000	0.000	0.000	134.508
PARVB	0	2	1.000	0.000	0.000	134.508
PASD1	0	2	1.000	0.000	0.000	134.508
PASK	0	3	1.000	0.000	0.000	64.152
PAXBP1	0	2	1.000	0.000	0.000	134.508
PBRM1	0	3	1.000	0.000	0.000	64.152
PC	0	2	1.000	0.000	0.000	134.508
PCBP3	0	2	1.000	0.000	0.000	134.508
PCDH10	0	2	1.000	0.000	0.000	134.508
PCDH11X	0	3	1.000	0.000	0.000	64.152
PCDH12	0	4	1.000	0.000	0.000	41.489
PCDH17	0	2	1.000	0.000	0.000	134.508
PCDH18	0	3	1.000	0.000	0.000	64.152
PCDH19	0	3	1.000	0.000	0.000	64.152
PCDHA1	0	2	1.000	0.000	0.000	134.508
PCDHA10	0	2	1.000	0.000	0.000	134.508
PCDHA2	0	5	1.000	0.000	0.000	30.501
PCDHA3	0	3	1.000	0.000	0.000	64.152
PCDHA4	0	3	1.000	0.000	0.000	64.152
PCDHA7	0	3	1.000	0.000	0.000	64.152
PCDHA8	0	2	1.000	0.000	0.000	134.508
PCDHB10	0	3	1.000	0.000	0.000	64.152
PCDHB11	0	2	1.000	0.000	0.000	134.508
PCDHB12	0	4	1.000	0.000	0.000	41.489
PCDHB13	0	3	1.000	0.000	0.000	64.152
PCDHB14	0	2	1.000	0.000	0.000	134.508
PCDHB3	0	4	1.000	0.000	0.000	41.489
PCDHB4	0	2	1.000	0.000	0.000	134.508
PCDHB5	0	2	1.000	0.000	0.000	134.508
PCDHB6	0	2	1.000	0.000	0.000	134.508
PCDHB8	0	3	1.000	0.000	0.000	64.152
PCDHGA10	0	2	1.000	0.000	0.000	134.508
PCDHGA12	0	2	1.000	0.000	0.000	134.508
PCDHGA2	0	4	1.000	0.000	0.000	41.489
PCDHGA5	0	2	1.000	0.000	0.000	134.508
PCDHGA6	0	3	1.000	0.000	0.000	64.152
PCDHGA7	0	2	1.000	0.000	0.000	134.508
PCDHGA8	0	2	1.000	0.000	0.000	134.508
PCDHGB4	0	2	1.000	0.000	0.000	134.508
PCDHGB6	0	3	1.000	0.000	0.000	64.152
PCDHGB7	0	3	1.000	0.000	0.000	64.152
PCDHGC5	0	2	1.000	0.000	0.000	134.508
PCM1	0	3	1.000	0.000	0.000	64.152
PCMTD1	0	20	1.000	0.000	0.000	5.547
PCNX	0	3	1.000	0.000	0.000	64.152
PCNXL2	0	3	1.000	0.000	0.000	64.152
PCOLCE	0	2	1.000	0.000	0.000	134.508
PCYT1B	0	3	1.000	0.000	0.000	64.152
PCYT2	0	2	1.000	0.000	0.000	134.508
PDCD11	0	4	1.000	0.000	0.000	41.489
PDCD6IP	0	3	1.000	0.000	0.000	64.152
PDE10A	0	3	1.000	0.000	0.000	64.152
PDE3A	0	5	1.000	0.000	0.000	30.501
PDE3B	0	2	1.000	0.000	0.000	134.508
PDE4C	0	2	1.000	0.000	0.000	134.508
PDE4DIP	0	5	1.000	0.000	0.000	30.501
PDE5A	0	2	1.000	0.000	0.000	134.508
PDE6C	0	3	1.000	0.000	0.000	64.152
PDE8B	0	2	1.000	0.000	0.000	134.508
PDGFC	0	2	1.000	0.000	0.000	134.508
PDGFRA	0	2	1.000	0.000	0.000	134.508
PDGFRB	0	2	1.000	0.000	0.000	134.508
PDS5B	0	2	1.000	0.000	0.000	134.508

PDYN	0	2	1.000	0.000	0.000	134.508
PDZD2	0	2	1.000	0.000	0.000	134.508
PDZRN3	0	5	1.000	0.000	0.000	30.501
PDZRN4	0	3	1.000	0.000	0.000	64.152
PEAK1	0	2	1.000	0.000	0.000	134.508
PEG10	0	2	1.000	0.000	0.000	134.508
PEG3	0	5	1.000	0.000	0.000	30.501
PER1	0	2	1.000	0.000	0.000	134.508
PERP	0	2	1.000	0.000	0.000	134.508
PES1	0	3	1.000	0.000	0.000	64.152
PEX12	0	2	1.000	0.000	0.000	134.508
PEX16	0	3	1.000	0.000	0.000	64.152
PEX6	0	2	1.000	0.000	0.000	134.508
PEX7	0	2	1.000	0.000	0.000	134.508
PFAS	0	2	1.000	0.000	0.000	134.508
PFKFB4	0	3	1.000	0.000	0.000	64.152
PFKL	0	2	1.000	0.000	0.000	134.508
PGBD1	0	2	1.000	0.000	0.000	134.508
PGBD5	0	2	1.000	0.000	0.000	134.508
PGC	0	2	1.000	0.000	0.000	134.508
PHACTR1	0	4	1.000	0.000	0.000	41.489
PHACTR3	0	5	1.000	0.000	0.000	30.501
PHACTR4	0	3	1.000	0.000	0.000	64.152
PHB	0	2	1.000	0.000	0.000	134.508
PHC2	0	2	1.000	0.000	0.000	134.508
PHEX	0	2	1.000	0.000	0.000	134.508
PHF10	0	2	1.000	0.000	0.000	134.508
PHF11	0	2	1.000	0.000	0.000	134.508
PHF12	0	2	1.000	0.000	0.000	134.508
PHF15	0	2	1.000	0.000	0.000	134.508
PHF20L1	0	3	1.000	0.000	0.000	64.152
PHF3	0	2	1.000	0.000	0.000	134.508
PHGDH	0	2	1.000	0.000	0.000	134.508
PHKA2	0	3	1.000	0.000	0.000	64.152
PHLDB2	0	2	1.000	0.000	0.000	134.508
PHLPP1	0	2	1.000	0.000	0.000	134.508
PHOSPHO1	0	2	1.000	0.000	0.000	134.508
PHTF1	0	2	1.000	0.000	0.000	134.508
PIBF1	0	2	1.000	0.000	0.000	134.508
PIGO	0	2	1.000	0.000	0.000	134.508
PIK3C2A	0	2	1.000	0.000	0.000	134.508
PIK3C3	0	2	1.000	0.000	0.000	134.508
PIK3CA	0	5	1.000	0.000	0.000	30.501
PIK3CB	0	4	1.000	0.000	0.000	41.489
PIK3CG	0	2	1.000	0.000	0.000	134.508
PIK3R1	0	2	1.000	0.000	0.000	134.508
PIK3R2	0	2	1.000	0.000	0.000	134.508
PIK3R3	0	2	1.000	0.000	0.000	134.508
PIK3R4	0	4	1.000	0.000	0.000	41.489
PIK3R5	0	2	1.000	0.000	0.000	134.508
PIKFYVE	0	3	1.000	0.000	0.000	64.152
PITPNC1	0	3	1.000	0.000	0.000	64.152
PITPNM2	0	2	1.000	0.000	0.000	134.508
PIWIL1	0	2	1.000	0.000	0.000	134.508
PIWIL3	0	3	1.000	0.000	0.000	64.152
PIWIL4	0	2	1.000	0.000	0.000	134.508
PKD1L2	0	3	1.000	0.000	0.000	64.152
PKD2	0	2	1.000	0.000	0.000	134.508
PKDREJ	0	2	1.000	0.000	0.000	134.508
PKHD1	0	3	1.000	0.000	0.000	64.152
PKHD1L1	0	4	1.000	0.000	0.000	41.489
PKP1	0	2	1.000	0.000	0.000	134.508
PKP2	0	3	1.000	0.000	0.000	64.152
PKP4	0	2	1.000	0.000	0.000	134.508
PLA2G4D	0	2	1.000	0.000	0.000	134.508
PLA2G7	0	2	1.000	0.000	0.000	134.508

PLCB2	0	2	1.000	0.000	0.000	134.508
PLCB3	0	2	1.000	0.000	0.000	134.508
PLCB4	0	3	1.000	0.000	0.000	64.152
PLCD4	0	2	1.000	0.000	0.000	134.508
PLCE1	0	4	1.000	0.000	0.000	41.489
PLCG1	0	3	1.000	0.000	0.000	64.152
PLD5	0	4	1.000	0.000	0.000	41.489
PLEC	0	5	1.000	0.000	0.000	30.501
PLEKHA4	0	2	1.000	0.000	0.000	134.508
PLEKHA5	0	3	1.000	0.000	0.000	64.152
PLEKHA8	0	2	1.000	0.000	0.000	134.508
PLEKHG2	0	2	1.000	0.000	0.000	134.508
PLEKHG3	0	2	1.000	0.000	0.000	134.508
PLEKHH1	0	2	1.000	0.000	0.000	134.508
PLIN4	0	3	1.000	0.000	0.000	64.152
PLK4	0	3	1.000	0.000	0.000	64.152
PLOD2	0	2	1.000	0.000	0.000	134.508
PLS1	0	2	1.000	0.000	0.000	134.508
PLSCR2	0	2	1.000	0.000	0.000	134.508
PLXDC1	0	2	1.000	0.000	0.000	134.508
PLXDC2	0	2	1.000	0.000	0.000	134.508
PLXNA1	0	2	1.000	0.000	0.000	134.508
PLXNA2	0	5	1.000	0.000	0.000	30.501
PLXNA3	0	2	1.000	0.000	0.000	134.508
PLXNB1	0	2	1.000	0.000	0.000	134.508
PLXNB2	0	3	1.000	0.000	0.000	64.152
PLXNB3	0	3	1.000	0.000	0.000	64.152
PLXNC1	0	2	1.000	0.000	0.000	134.508
PLXND1	0	2	1.000	0.000	0.000	134.508
PM20D1	0	2	1.000	0.000	0.000	134.508
PMEPA1	0	4	1.000	0.000	0.000	41.489
PMFBP1	0	2	1.000	0.000	0.000	134.508
PMPCB	0	3	1.000	0.000	0.000	64.152
PMS1	0	2	1.000	0.000	0.000	134.508
PNLDC1	0	2	1.000	0.000	0.000	134.508
PNLIP	0	2	1.000	0.000	0.000	134.508
PNMA5	0	2	1.000	0.000	0.000	134.508
POLDIP3	0	2	1.000	0.000	0.000	134.508
POLE	0	2	1.000	0.000	0.000	134.508
POLE2	0	2	1.000	0.000	0.000	134.508
POLQ	0	2	1.000	0.000	0.000	134.508
POLR1B	0	3	1.000	0.000	0.000	64.152
POLR2B	0	3	1.000	0.000	0.000	64.152
POM121L12	0	5	1.000	0.000	0.000	30.501
POMGNT1	0	2	1.000	0.000	0.000	134.508
PON1	0	2	1.000	0.000	0.000	134.508
POSTN	0	2	1.000	0.000	0.000	134.508
POTEG	0	3	1.000	0.000	0.000	64.152
POTEM	0	2	1.000	0.000	0.000	134.508
PPARGC1A	0	2	1.000	0.000	0.000	134.508
PPARGC1B	0	2	1.000	0.000	0.000	134.508
PPFIA1	0	2	1.000	0.000	0.000	134.508
PPFIA2	0	3	1.000	0.000	0.000	64.152
PPIC	0	2	1.000	0.000	0.000	134.508
PPIG	0	2	1.000	0.000	0.000	134.508
PPIP5K1	0	2	1.000	0.000	0.000	134.508
PPL	0	2	1.000	0.000	0.000	134.508
PPM1G	0	2	1.000	0.000	0.000	134.508
PPP1R12C	0	2	1.000	0.000	0.000	134.508
PPP1R16B	0	3	1.000	0.000	0.000	64.152
PPP1R26	0	3	1.000	0.000	0.000	64.152
PPP1R3A	0	2	1.000	0.000	0.000	134.508
PPP1R9A	0	3	1.000	0.000	0.000	64.152
PPP2R1A	0	2	1.000	0.000	0.000	134.508
PPP2R1B	0	3	1.000	0.000	0.000	64.152
PPP2R5C	0	2	1.000	0.000	0.000	134.508

PPP6R3	0	2	1.000	0.000	0.000	134.508
PPWD1	0	2	1.000	0.000	0.000	134.508
PRAMEF1	0	2	1.000	0.000	0.000	134.508
PRAMEF11	0	2	1.000	0.000	0.000	134.508
PRAMEF4	0	2	1.000	0.000	0.000	134.508
PRC1	0	2	1.000	0.000	0.000	134.508
PRCC	0	2	1.000	0.000	0.000	134.508
PRDM10	0	2	1.000	0.000	0.000	134.508
PRDM15	0	2	1.000	0.000	0.000	134.508
PRDM2	0	2	1.000	0.000	0.000	134.508
PRDM5	0	2	1.000	0.000	0.000	134.508
PREX2	0	3	1.000	0.000	0.000	64.152
PRG4	0	4	1.000	0.000	0.000	41.489
PRICKLE1	0	2	1.000	0.000	0.000	134.508
PRKAG3	0	2	1.000	0.000	0.000	134.508
PRKCD	0	3	1.000	0.000	0.000	64.152
PRKCG	0	2	1.000	0.000	0.000	134.508
PRKCH	0	2	1.000	0.000	0.000	134.508
PRKCQ	0	2	1.000	0.000	0.000	134.508
PRKCZ	0	2	1.000	0.000	0.000	134.508
PRKD2	0	3	1.000	0.000	0.000	64.152
PRKRA	0	9	1.000	0.000	0.000	14.507
PRKRIR	0	2	1.000	0.000	0.000	134.508
PRLR	0	2	1.000	0.000	0.000	134.508
PRMT10	0	2	1.000	0.000	0.000	134.508
PRND	0	2	1.000	0.000	0.000	134.508
PRODH2	0	2	1.000	0.000	0.000	134.508
PRPF3	0	2	1.000	0.000	0.000	134.508
PRPF39	0	2	1.000	0.000	0.000	134.508
PRPF8	0	2	1.000	0.000	0.000	134.508
PRPS1L1	0	2	1.000	0.000	0.000	134.508
PRR14	0	2	1.000	0.000	0.000	134.508
PRR16	0	2	1.000	0.000	0.000	134.508
PRR21	0	4	1.000	0.000	0.000	41.489
PRR5L	0	3	1.000	0.000	0.000	64.152
PRRT2	0	2	1.000	0.000	0.000	134.508
PRSS21	0	2	1.000	0.000	0.000	134.508
PRSS23	0	2	1.000	0.000	0.000	134.508
PRSS35	0	3	1.000	0.000	0.000	64.152
PRSS45	0	2	1.000	0.000	0.000	134.508
PRTFDC1	0	2	1.000	0.000	0.000	134.508
PSD	0	2	1.000	0.000	0.000	134.508
PSG3	0	5	1.000	0.000	0.000	30.501
PSG4	0	3	1.000	0.000	0.000	64.152
PSG6	0	3	1.000	0.000	0.000	64.152
PSG8	0	2	1.000	0.000	0.000	134.508
PSMD1	0	2	1.000	0.000	0.000	134.508
PSMD11	0	2	1.000	0.000	0.000	134.508
PSME4	0	4	1.000	0.000	0.000	41.489
PTBP3	0	2	1.000	0.000	0.000	134.508
PTCD3	0	2	1.000	0.000	0.000	134.508
PTCH1	0	3	1.000	0.000	0.000	64.152
PTCH2	0	3	1.000	0.000	0.000	64.152
PTCHD3	0	2	1.000	0.000	0.000	134.508
PTCHD4	0	2	1.000	0.000	0.000	134.508
PTDSS1	0	2	1.000	0.000	0.000	134.508
PTF1A	0	2	1.000	0.000	0.000	134.508
PTGIS	0	2	1.000	0.000	0.000	134.508
PTPDC1	0	2	1.000	0.000	0.000	134.508
PTPN11	0	9	1.000	0.000	0.000	14.507
PTPN12	0	2	1.000	0.000	0.000	134.508
PTPN13	0	3	1.000	0.000	0.000	64.152
PTPN14	0	2	1.000	0.000	0.000	134.508
PTPN21	0	3	1.000	0.000	0.000	64.152
PTPN23	0	2	1.000	0.000	0.000	134.508
PTPN4	0	2	1.000	0.000	0.000	134.508

PTPN7	0	2	1.000	0.000	0.000	134.508
PTPRB	0	2	1.000	0.000	0.000	134.508
PTPRE	0	2	1.000	0.000	0.000	134.508
PTPRF	0	2	1.000	0.000	0.000	134.508
PTPRG	0	3	1.000	0.000	0.000	64.152
PTPRM	0	2	1.000	0.000	0.000	134.508
PTPRN2	0	3	1.000	0.000	0.000	64.152
PTPRS	0	3	1.000	0.000	0.000	64.152
PTPRT	0	4	1.000	0.000	0.000	41.489
PTPRU	0	2	1.000	0.000	0.000	134.508
PTPRZ1	0	3	1.000	0.000	0.000	64.152
PTX4	0	3	1.000	0.000	0.000	64.152
PUM2	0	2	1.000	0.000	0.000	134.508
PUS3	0	3	1.000	0.000	0.000	64.152
PUS7L	0	3	1.000	0.000	0.000	64.152
PWP1	0	2	1.000	0.000	0.000	134.508
PXDN	0	3	1.000	0.000	0.000	64.152
PXDNL	0	4	1.000	0.000	0.000	41.489
PYGL	0	4	1.000	0.000	0.000	41.489
QDPR	0	2	1.000	0.000	0.000	134.508
QRICH2	0	4	1.000	0.000	0.000	41.489
QSER1	0	2	1.000	0.000	0.000	134.508
RAB19	0	2	1.000	0.000	0.000	134.508
RAB28	0	2	1.000	0.000	0.000	134.508
RAB3GAP1	0	2	1.000	0.000	0.000	134.508
RAB3IP	0	2	1.000	0.000	0.000	134.508
RAB4A	0	2	1.000	0.000	0.000	134.508
RAD21	0	2	1.000	0.000	0.000	134.508
RADIL	0	2	1.000	0.000	0.000	134.508
RAG1	0	2	1.000	0.000	0.000	134.508
RAI1	0	3	1.000	0.000	0.000	64.152
RALGAPA1	0	3	1.000	0.000	0.000	64.152
RALGAPA2	0	3	1.000	0.000	0.000	64.152
RALGAPB	0	2	1.000	0.000	0.000	134.508
RALGPS1	0	2	1.000	0.000	0.000	134.508
RANBP2	0	4	1.000	0.000	0.000	41.489
RANBP3	0	2	1.000	0.000	0.000	134.508
RANGAP1	0	2	1.000	0.000	0.000	134.508
RAPGEF2	0	4	1.000	0.000	0.000	41.489
RARRES3	0	2	1.000	0.000	0.000	134.508
RARS	0	2	1.000	0.000	0.000	134.508
RARS2	0	2	1.000	0.000	0.000	134.508
RASA1	0	3	1.000	0.000	0.000	64.152
RASA2	0	3	1.000	0.000	0.000	64.152
RASGEF1C	0	2	1.000	0.000	0.000	134.508
RASGRF2	0	2	1.000	0.000	0.000	134.508
RASSF2	0	2	1.000	0.000	0.000	134.508
RAVER2	0	2	1.000	0.000	0.000	134.508
RB1CC1	0	3	1.000	0.000	0.000	64.152
RBBP6	0	2	1.000	0.000	0.000	134.508
RBFA	0	2	1.000	0.000	0.000	134.508
RBFOX1	0	2	1.000	0.000	0.000	134.508
RBKS	0	2	1.000	0.000	0.000	134.508
RBM10	0	3	1.000	0.000	0.000	64.152
RBM12B	0	2	1.000	0.000	0.000	134.508
RBM15	0	2	1.000	0.000	0.000	134.508
RBM17	0	2	1.000	0.000	0.000	134.508
RBM23	0	2	1.000	0.000	0.000	134.508
RBM25	0	2	1.000	0.000	0.000	134.508
RBM26	0	2	1.000	0.000	0.000	134.508
RBM27	0	3	1.000	0.000	0.000	64.152
RBM28	0	2	1.000	0.000	0.000	134.508
RBM45	0	2	1.000	0.000	0.000	134.508
RBM6	0	2	1.000	0.000	0.000	134.508
RBMS1	0	2	1.000	0.000	0.000	134.508
RBP3	0	2	1.000	0.000	0.000	134.508

RC3H2	0	2	1.000	0.000	0.000	134.508
RECK	0	2	1.000	0.000	0.000	134.508
RECQL	0	2	1.000	0.000	0.000	134.508
RELN	0	7	1.000	0.000	0.000	19.777
REPS1	0	2	1.000	0.000	0.000	134.508
RET	0	2	1.000	0.000	0.000	134.508
RETSAT	0	4	1.000	0.000	0.000	41.489
REV1	0	2	1.000	0.000	0.000	134.508
REV3L	0	4	1.000	0.000	0.000	41.489
RFPL1	0	2	1.000	0.000	0.000	134.508
RFTN2	0	2	1.000	0.000	0.000	134.508
RGS12	0	3	1.000	0.000	0.000	64.152
RGS22	0	3	1.000	0.000	0.000	64.152
RHBDF1	0	2	1.000	0.000	0.000	134.508
RHBG	0	2	1.000	0.000	0.000	134.508
RHOBTB1	0	2	1.000	0.000	0.000	134.508
RICTOR	0	3	1.000	0.000	0.000	64.152
RIF1	0	2	1.000	0.000	0.000	134.508
RIMBP2	0	2	1.000	0.000	0.000	134.508
RIMS4	0	4	1.000	0.000	0.000	41.489
RIPPLY2	0	2	1.000	0.000	0.000	134.508
RLF	0	2	1.000	0.000	0.000	134.508
RLN1	0	2	1.000	0.000	0.000	134.508
RLTPR	0	2	1.000	0.000	0.000	134.508
RND1	0	2	1.000	0.000	0.000	134.508
RND3	0	2	1.000	0.000	0.000	134.508
RNF10	0	2	1.000	0.000	0.000	134.508
RNF111	0	3	1.000	0.000	0.000	64.152
RNF13	0	2	1.000	0.000	0.000	134.508
RNF145	0	7	1.000	0.000	0.000	19.777
RNF152	0	2	1.000	0.000	0.000	134.508
RNF213	0	5	1.000	0.000	0.000	30.501
RNF31	0	2	1.000	0.000	0.000	134.508
RNF38	0	2	1.000	0.000	0.000	134.508
RNF40	0	2	1.000	0.000	0.000	134.508
RNF43	0	8	1.000	0.000	0.000	16.757
RNF6	0	2	1.000	0.000	0.000	134.508
ROBO1	0	3	1.000	0.000	0.000	64.152
ROBO2	0	3	1.000	0.000	0.000	64.152
ROBO4	0	2	1.000	0.000	0.000	134.508
ROCK1	0	4	1.000	0.000	0.000	41.489
ROR2	0	2	1.000	0.000	0.000	134.508
ROS1	0	2	1.000	0.000	0.000	134.508
RP1	0	4	1.000	0.000	0.000	41.489
RP11-368J21.2	0	2	1.000	0.000	0.000	134.508
RPL10L	0	2	1.000	0.000	0.000	134.508
RPL14	0	2	1.000	0.000	0.000	134.508
RPL3L	0	2	1.000	0.000	0.000	134.508
RPL4	0	3	1.000	0.000	0.000	64.152
RPRD1B	0	2	1.000	0.000	0.000	134.508
RPRD2	0	2	1.000	0.000	0.000	134.508
RPS11	0	2	1.000	0.000	0.000	134.508
RPS18	0	2	1.000	0.000	0.000	134.508
RPS6KA2	0	2	1.000	0.000	0.000	134.508
RPS6KC1	0	2	1.000	0.000	0.000	134.508
RPTOR	0	2	1.000	0.000	0.000	134.508
RREB1	0	5	1.000	0.000	0.000	30.501
RSC1A1	0	3	1.000	0.000	0.000	64.152
RSPRY1	0	2	1.000	0.000	0.000	134.508
RSRC2	0	2	1.000	0.000	0.000	134.508
RTN2	0	2	1.000	0.000	0.000	134.508
RTN3	0	2	1.000	0.000	0.000	134.508
RTN4	0	2	1.000	0.000	0.000	134.508
RTTN	0	2	1.000	0.000	0.000	134.508
RUFY1	0	3	1.000	0.000	0.000	64.152
RUSC2	0	2	1.000	0.000	0.000	134.508

RYR1	0	9	1.000	0.000	0.000	14.507
RYR2	0	5	1.000	0.000	0.000	30.501
S100A10	0	3	1.000	0.000	0.000	64.152
SACS	0	3	1.000	0.000	0.000	64.152
SAFB	0	2	1.000	0.000	0.000	134.508
SALL1	0	9	1.000	0.000	0.000	14.507
SAMD15	0	3	1.000	0.000	0.000	64.152
SAMD9	0	2	1.000	0.000	0.000	134.508
SAMHD1	0	2	1.000	0.000	0.000	134.508
SAMM50	0	3	1.000	0.000	0.000	64.152
SASH1	0	2	1.000	0.000	0.000	134.508
SBF2	0	2	1.000	0.000	0.000	134.508
SBSPON	0	2	1.000	0.000	0.000	134.508
SCAF11	0	2	1.000	0.000	0.000	134.508
SCAMP3	0	2	1.000	0.000	0.000	134.508
SCARB2	0	3	1.000	0.000	0.000	64.152
SCARF1	0	2	1.000	0.000	0.000	134.508
SCD5	0	2	1.000	0.000	0.000	134.508
SCFD1	0	2	1.000	0.000	0.000	134.508
SCML1	0	2	1.000	0.000	0.000	134.508
SCN11A	0	2	1.000	0.000	0.000	134.508
SCN4A	0	4	1.000	0.000	0.000	41.489
SCN5A	0	5	1.000	0.000	0.000	30.501
SCN8A	0	3	1.000	0.000	0.000	64.152
SCNN1B	0	2	1.000	0.000	0.000	134.508
SCOC	0	2	1.000	0.000	0.000	134.508
SCRIB	0	3	1.000	0.000	0.000	64.152
SCUBE1	0	3	1.000	0.000	0.000	64.152
SDCBP	0	2	1.000	0.000	0.000	134.508
SDCCAG8	0	2	1.000	0.000	0.000	134.508
SDHA	0	4	1.000	0.000	0.000	41.489
SDK1	0	4	1.000	0.000	0.000	41.489
SDR39U1	0	3	1.000	0.000	0.000	64.152
SEC14L3	0	2	1.000	0.000	0.000	134.508
SEC14L4	0	2	1.000	0.000	0.000	134.508
SEC16B	0	3	1.000	0.000	0.000	64.152
SEC24A	0	2	1.000	0.000	0.000	134.508
SEC24B	0	2	1.000	0.000	0.000	134.508
SEC24C	0	2	1.000	0.000	0.000	134.508
SEC31B	0	3	1.000	0.000	0.000	64.152
SEL1L2	0	2	1.000	0.000	0.000	134.508
SEL1L3	0	2	1.000	0.000	0.000	134.508
SEMA3A	0	3	1.000	0.000	0.000	64.152
SEMA4C	0	2	1.000	0.000	0.000	134.508
SEMA4G	0	2	1.000	0.000	0.000	134.508
SEMA5A	0	2	1.000	0.000	0.000	134.508
SEMA5B	0	3	1.000	0.000	0.000	64.152
SEPP1	0	2	1.000	0.000	0.000	134.508
SERPINA10	0	2	1.000	0.000	0.000	134.508
SERPINA7	0	3	1.000	0.000	0.000	64.152
SERPINA9	0	2	1.000	0.000	0.000	134.508
SERPINB10	0	3	1.000	0.000	0.000	64.152
SERPINB2	0	3	1.000	0.000	0.000	64.152
SERPINB5	0	2	1.000	0.000	0.000	134.508
SESN2	0	2	1.000	0.000	0.000	134.508
SETBP1	0	2	1.000	0.000	0.000	134.508
SETD1A	0	2	1.000	0.000	0.000	134.508
SETD2	0	2	1.000	0.000	0.000	134.508
SETDB1	0	2	1.000	0.000	0.000	134.508
SETX	0	3	1.000	0.000	0.000	64.152
SF1	0	2	1.000	0.000	0.000	134.508
SF3B1	0	4	1.000	0.000	0.000	41.489
SF3B2	0	2	1.000	0.000	0.000	134.508
SF3B3	0	2	1.000	0.000	0.000	134.508
SFI1	0	2	1.000	0.000	0.000	134.508
SFRP4	0	2	1.000	0.000	0.000	134.508

SFTPC	0	2	1.000	0.000	0.000	134.508
SGCD	0	2	1.000	0.000	0.000	134.508
SGIP1	0	4	1.000	0.000	0.000	41.489
SGK1	0	3	1.000	0.000	0.000	64.152
SGK223	0	3	1.000	0.000	0.000	64.152
SGSM1	0	3	1.000	0.000	0.000	64.152
SGSM3	0	2	1.000	0.000	0.000	134.508
SH2D3A	0	2	1.000	0.000	0.000	134.508
SH2D3C	0	3	1.000	0.000	0.000	64.152
SH2D4A	0	2	1.000	0.000	0.000	134.508
SH3TC1	0	3	1.000	0.000	0.000	64.152
SH3TC2	0	2	1.000	0.000	0.000	134.508
SHANK1	0	2	1.000	0.000	0.000	134.508
SHD	0	2	1.000	0.000	0.000	134.508
SHMT2	0	2	1.000	0.000	0.000	134.508
SHOX2	0	2	1.000	0.000	0.000	134.508
SHROOM3	0	5	1.000	0.000	0.000	30.501
SHROOM4	0	3	1.000	0.000	0.000	64.152
SI	0	2	1.000	0.000	0.000	134.508
SIAH2	0	2	1.000	0.000	0.000	134.508
SIGIRR	0	2	1.000	0.000	0.000	134.508
SIGLEC1	0	3	1.000	0.000	0.000	64.152
SIGLEC5	0	2	1.000	0.000	0.000	134.508
SIGLEC6	0	3	1.000	0.000	0.000	64.152
SIGLEC8	0	2	1.000	0.000	0.000	134.508
SIM2	0	2	1.000	0.000	0.000	134.508
SIN3A	0	2	1.000	0.000	0.000	134.508
SIPA1L2	0	2	1.000	0.000	0.000	134.508
SIPA1L3	0	2	1.000	0.000	0.000	134.508
SIRPA	0	2	1.000	0.000	0.000	134.508
SIRPB1	0	2	1.000	0.000	0.000	134.508
SIRPG	0	2	1.000	0.000	0.000	134.508
SKIV2L2	0	3	1.000	0.000	0.000	64.152
SLAIN1	0	2	1.000	0.000	0.000	134.508
SLC10A6	0	2	1.000	0.000	0.000	134.508
SLC12A2	0	5	1.000	0.000	0.000	30.501
SLC12A3	0	3	1.000	0.000	0.000	64.152
SLC12A5	0	2	1.000	0.000	0.000	134.508
SLC12A6	0	3	1.000	0.000	0.000	64.152
SLC12A7	0	4	1.000	0.000	0.000	41.489
SLC13A4	0	2	1.000	0.000	0.000	134.508
SLC18A2	0	2	1.000	0.000	0.000	134.508
SLC19A1	0	2	1.000	0.000	0.000	134.508
SLC1A3	0	2	1.000	0.000	0.000	134.508
SLC22A12	0	2	1.000	0.000	0.000	134.508
SLC22A2	0	2	1.000	0.000	0.000	134.508
SLC22A23	0	2	1.000	0.000	0.000	134.508
SLC25A12	0	2	1.000	0.000	0.000	134.508
SLC25A13	0	2	1.000	0.000	0.000	134.508
SLC25A36	0	2	1.000	0.000	0.000	134.508
SLC25A48	0	2	1.000	0.000	0.000	134.508
SLC26A6	0	2	1.000	0.000	0.000	134.508
SLC26A7	0	2	1.000	0.000	0.000	134.508
SLC28A1	0	2	1.000	0.000	0.000	134.508
SLC2A10	0	2	1.000	0.000	0.000	134.508
SLC30A8	0	3	1.000	0.000	0.000	64.152
SLC35B3	0	2	1.000	0.000	0.000	134.508
SLC35G5	0	2	1.000	0.000	0.000	134.508
SLC35G6	0	3	1.000	0.000	0.000	64.152
SLC36A3	0	2	1.000	0.000	0.000	134.508
SLC38A10	0	4	1.000	0.000	0.000	41.489
SLC38A8	0	3	1.000	0.000	0.000	64.152
SLC39A12	0	2	1.000	0.000	0.000	134.508
SLC41A3	0	2	1.000	0.000	0.000	134.508
SLC44A5	0	2	1.000	0.000	0.000	134.508
SLC45A4	0	2	1.000	0.000	0.000	134.508

SLC4A10	0	2	1.000	0.000	0.000	134.508
SLC52A1	0	2	1.000	0.000	0.000	134.508
SLC5A2	0	2	1.000	0.000	0.000	134.508
SLC5A3	0	2	1.000	0.000	0.000	134.508
SLC5A8	0	2	1.000	0.000	0.000	134.508
SLC5A9	0	2	1.000	0.000	0.000	134.508
SLC6A18	0	2	1.000	0.000	0.000	134.508
SLC6A2	0	3	1.000	0.000	0.000	64.152
SLC6A3	0	2	1.000	0.000	0.000	134.508
SLC6A4	0	2	1.000	0.000	0.000	134.508
SLC6A5	0	2	1.000	0.000	0.000	134.508
SLC7A1	0	2	1.000	0.000	0.000	134.508
SLC7A2	0	3	1.000	0.000	0.000	64.152
SLC7A3	0	2	1.000	0.000	0.000	134.508
SLC7A7	0	2	1.000	0.000	0.000	134.508
SLC8A2	0	2	1.000	0.000	0.000	134.508
SLC8A3	0	2	1.000	0.000	0.000	134.508
SLC9A3	0	2	1.000	0.000	0.000	134.508
SLC9A6	0	3	1.000	0.000	0.000	64.152
SLC9A9	0	2	1.000	0.000	0.000	134.508
SLC9B2	0	2	1.000	0.000	0.000	134.508
SLC9C2	0	2	1.000	0.000	0.000	134.508
SLCO1B3	0	4	1.000	0.000	0.000	41.489
SLCO1C1	0	2	1.000	0.000	0.000	134.508
SLCO5A1	0	2	1.000	0.000	0.000	134.508
SLCO6A1	0	2	1.000	0.000	0.000	134.508
SLIT1	0	4	1.000	0.000	0.000	41.489
SLIT2	0	3	1.000	0.000	0.000	64.152
SLIT3	0	2	1.000	0.000	0.000	134.508
SLK	0	2	1.000	0.000	0.000	134.508
SLTM	0	2	1.000	0.000	0.000	134.508
SLU7	0	2	1.000	0.000	0.000	134.508
SMAD4	1	24	1.000	0.975	0.020	9.301
SMARCA2	0	4	1.000	0.000	0.000	41.489
SMARCA4	0	2	1.000	0.000	0.000	134.508
SMARCC2	0	2	1.000	0.000	0.000	134.508
SMARCE1	0	3	1.000	0.000	0.000	64.152
SMC2	0	3	1.000	0.000	0.000	64.152
SMC3	0	2	1.000	0.000	0.000	134.508
SMC4	0	2	1.000	0.000	0.000	134.508
SMC5	0	3	1.000	0.000	0.000	64.152
SMCHD1	0	2	1.000	0.000	0.000	134.508
SMEK1	0	2	1.000	0.000	0.000	134.508
SMEK2	0	2	1.000	0.000	0.000	134.508
SMG1	0	2	1.000	0.000	0.000	134.508
SMPD4	0	2	1.000	0.000	0.000	134.508
SMPDL3A	0	2	1.000	0.000	0.000	134.508
SMTN	0	2	1.000	0.000	0.000	134.508
SMTNL2	0	2	1.000	0.000	0.000	134.508
SNAPC3	0	2	1.000	0.000	0.000	134.508
SNAPC4	0	2	1.000	0.000	0.000	134.508
SNCAIP	0	2	1.000	0.000	0.000	134.508
SNRPE	0	2	1.000	0.000	0.000	134.508
SNX2	0	2	1.000	0.000	0.000	134.508
SNX29	0	2	1.000	0.000	0.000	134.508
SNX6	0	3	1.000	0.000	0.000	64.152
SOCS5	0	4	1.000	0.000	0.000	41.489
SOGA3	0	2	1.000	0.000	0.000	134.508
SON	0	3	1.000	0.000	0.000	64.152
SORCS1	0	2	1.000	0.000	0.000	134.508
SORCS2	0	2	1.000	0.000	0.000	134.508
SORCS3	0	2	1.000	0.000	0.000	134.508
SORL1	0	3	1.000	0.000	0.000	64.152
SORT1	0	2	1.000	0.000	0.000	134.508
SOS1	0	2	1.000	0.000	0.000	134.508
SP140	0	3	1.000	0.000	0.000	64.152

SP4	0	2	1.000	0.000	0.000	134.508
SPACA3	0	2	1.000	0.000	0.000	134.508
SPAG1	0	2	1.000	0.000	0.000	134.508
SPAG16	0	2	1.000	0.000	0.000	134.508
SPAG17	0	4	1.000	0.000	0.000	41.489
SPAG5	0	2	1.000	0.000	0.000	134.508
SPATA18	0	2	1.000	0.000	0.000	134.508
SPATA31D1	0	3	1.000	0.000	0.000	64.152
SPATA31E1	0	2	1.000	0.000	0.000	134.508
SPATA9	0	2	1.000	0.000	0.000	134.508
SPECC1L	0	2	1.000	0.000	0.000	134.508
SPEF2	0	3	1.000	0.000	0.000	64.152
SPEN	0	2	1.000	0.000	0.000	134.508
SPESP1	0	2	1.000	0.000	0.000	134.508
SPG11	0	2	1.000	0.000	0.000	134.508
SPG20	0	2	1.000	0.000	0.000	134.508
SPHKAP	0	3	1.000	0.000	0.000	64.152
SPINK5	0	3	1.000	0.000	0.000	64.152
SPOCD1	0	2	1.000	0.000	0.000	134.508
SPPL3	0	2	1.000	0.000	0.000	134.508
SPTB	0	2	1.000	0.000	0.000	134.508
SPTBN1	0	3	1.000	0.000	0.000	64.152
SPTBN2	0	3	1.000	0.000	0.000	64.152
SPTBN5	0	2	1.000	0.000	0.000	134.508
SQSTM1	0	3	1.000	0.000	0.000	64.152
SRC	0	2	1.000	0.000	0.000	134.508
SRCAP	0	3	1.000	0.000	0.000	64.152
SRGAP3	0	3	1.000	0.000	0.000	64.152
SRL	0	2	1.000	0.000	0.000	134.508
SRP72	0	2	1.000	0.000	0.000	134.508
SRPX2	0	2	1.000	0.000	0.000	134.508
SRR	0	2	1.000	0.000	0.000	134.508
SRRM1	0	2	1.000	0.000	0.000	134.508
SRRM2	0	2	1.000	0.000	0.000	134.508
SRRM4	0	2	1.000	0.000	0.000	134.508
SRSF6	0	2	1.000	0.000	0.000	134.508
SSBP4	0	2	1.000	0.000	0.000	134.508
SSFA2	0	2	1.000	0.000	0.000	134.508
SSMEM1	0	2	1.000	0.000	0.000	134.508
SSPO	0	2	1.000	0.000	0.000	134.508
SST	0	2	1.000	0.000	0.000	134.508
SSTR4	0	2	1.000	0.000	0.000	134.508
SSTR5	0	2	1.000	0.000	0.000	134.508
SSX2IP	0	2	1.000	0.000	0.000	134.508
SSX7	0	2	1.000	0.000	0.000	134.508
SSX9	0	3	1.000	0.000	0.000	64.152
ST18	0	4	1.000	0.000	0.000	41.489
ST6GAL2	0	2	1.000	0.000	0.000	134.508
ST7L	0	2	1.000	0.000	0.000	134.508
ST8SIA4	0	2	1.000	0.000	0.000	134.508
ST8SIA5	0	2	1.000	0.000	0.000	134.508
ST8SIA6	0	2	1.000	0.000	0.000	134.508
STAB1	0	4	1.000	0.000	0.000	41.489
STAG2	0	3	1.000	0.000	0.000	64.152
STAT1	0	2	1.000	0.000	0.000	134.508
STIL	0	3	1.000	0.000	0.000	64.152
STK11	0	2	1.000	0.000	0.000	134.508
STK38L	0	4	1.000	0.000	0.000	41.489
STOX2	0	3	1.000	0.000	0.000	64.152
STRIP2	0	2	1.000	0.000	0.000	134.508
STS	0	3	1.000	0.000	0.000	64.152
STX4	0	2	1.000	0.000	0.000	134.508
STXBP1	0	2	1.000	0.000	0.000	134.508
STXBP4	0	3	1.000	0.000	0.000	64.152
SUCO	0	2	1.000	0.000	0.000	134.508
SUGP2	0	2	1.000	0.000	0.000	134.508

SULF1	0	3	1.000	0.000	0.000	64.152
SULT1C4	0	2	1.000	0.000	0.000	134.508
SULT6B1	0	2	1.000	0.000	0.000	134.508
SUPT5H	0	2	1.000	0.000	0.000	134.508
SUSD2	0	2	1.000	0.000	0.000	134.508
SUSD4	0	2	1.000	0.000	0.000	134.508
SV2C	0	2	1.000	0.000	0.000	134.508
SVEP1	0	4	1.000	0.000	0.000	41.489
SWAP70	0	2	1.000	0.000	0.000	134.508
SYNJ2	0	2	1.000	0.000	0.000	134.508
SYNPO2	0	3	1.000	0.000	0.000	64.152
SYNPO2L	0	2	1.000	0.000	0.000	134.508
SYNRG	0	2	1.000	0.000	0.000	134.508
SYT14	0	2	1.000	0.000	0.000	134.508
SYT15	0	2	1.000	0.000	0.000	134.508
SYT3	0	3	1.000	0.000	0.000	64.152
SYT4	0	2	1.000	0.000	0.000	134.508
SYTL2	0	2	1.000	0.000	0.000	134.508
TAB2	0	2	1.000	0.000	0.000	134.508
TACC2	0	4	1.000	0.000	0.000	41.489
TAF1	0	3	1.000	0.000	0.000	64.152
TAF15	0	2	1.000	0.000	0.000	134.508
TAF1C	0	2	1.000	0.000	0.000	134.508
TAF1L	0	2	1.000	0.000	0.000	134.508
TAF4	0	3	1.000	0.000	0.000	64.152
TAF5L	0	2	1.000	0.000	0.000	134.508
TAF6	0	2	1.000	0.000	0.000	134.508
TANC1	0	3	1.000	0.000	0.000	64.152
TAOK1	0	2	1.000	0.000	0.000	134.508
TAOK2	0	2	1.000	0.000	0.000	134.508
TARDBP	0	2	1.000	0.000	0.000	134.508
TARS	0	2	1.000	0.000	0.000	134.508
TARSL2	0	2	1.000	0.000	0.000	134.508
TAS1R1	0	2	1.000	0.000	0.000	134.508
TAS2R19	0	5	1.000	0.000	0.000	30.501
TAS2R50	0	2	1.000	0.000	0.000	134.508
TASP1	0	2	1.000	0.000	0.000	134.508
TAX1BP1	0	2	1.000	0.000	0.000	134.508
TBC1D10A	0	2	1.000	0.000	0.000	134.508
TBC1D24	0	2	1.000	0.000	0.000	134.508
TBC1D25	0	2	1.000	0.000	0.000	134.508
TBC1D2B	0	2	1.000	0.000	0.000	134.508
TBC1D31	0	2	1.000	0.000	0.000	134.508
TBC1D5	0	2	1.000	0.000	0.000	134.508
TBC1D8	0	2	1.000	0.000	0.000	134.508
TBC1D9	0	2	1.000	0.000	0.000	134.508
TBCK	0	2	1.000	0.000	0.000	134.508
TBL3	0	2	1.000	0.000	0.000	134.508
TBX15	0	2	1.000	0.000	0.000	134.508
TBX22	0	2	1.000	0.000	0.000	134.508
TBX5	0	2	1.000	0.000	0.000	134.508
TBXAS1	0	2	1.000	0.000	0.000	134.508
TCEB3B	0	2	1.000	0.000	0.000	134.508
TCF4	0	5	1.000	0.000	0.000	30.501
TCF7L1	0	2	1.000	0.000	0.000	134.508
TCHH	0	3	1.000	0.000	0.000	64.152
TCN1	0	2	1.000	0.000	0.000	134.508
TCN2	0	2	1.000	0.000	0.000	134.508
TCTE1	0	2	1.000	0.000	0.000	134.508
TDG	0	15	1.000	0.000	0.000	7.856
TDP2	0	2	1.000	0.000	0.000	134.508
TDRD10	0	2	1.000	0.000	0.000	134.508
TDRD5	0	3	1.000	0.000	0.000	64.152
TDRD6	0	2	1.000	0.000	0.000	134.508
TEAD2	0	2	1.000	0.000	0.000	134.508
TECTA	0	3	1.000	0.000	0.000	64.152

TEKT3	0	2	1.000	0.000	0.000	134.508
TEKT4	0	3	1.000	0.000	0.000	64.152
TENM1	0	3	1.000	0.000	0.000	64.152
TENM2	0	3	1.000	0.000	0.000	64.152
TENM3	0	5	1.000	0.000	0.000	30.501
TENM4	0	4	1.000	0.000	0.000	41.489
TEP1	0	2	1.000	0.000	0.000	134.508
TERT	0	2	1.000	0.000	0.000	134.508
TESC	0	2	1.000	0.000	0.000	134.508
TET2	0	2	1.000	0.000	0.000	134.508
TET3	0	2	1.000	0.000	0.000	134.508
TEX11	0	2	1.000	0.000	0.000	134.508
TEX13A	0	2	1.000	0.000	0.000	134.508
TEX14	0	2	1.000	0.000	0.000	134.508
TEX15	0	4	1.000	0.000	0.000	41.489
TEX19	0	2	1.000	0.000	0.000	134.508
TEX2	0	2	1.000	0.000	0.000	134.508
TFAP2C	0	2	1.000	0.000	0.000	134.508
TFB2M	0	2	1.000	0.000	0.000	134.508
TFPI	0	2	1.000	0.000	0.000	134.508
TG	0	2	1.000	0.000	0.000	134.508
TGFBR1	0	2	1.000	0.000	0.000	134.508
TGFBR2	0	5	1.000	0.000	0.000	30.501
TGFBRAP1	0	2	1.000	0.000	0.000	134.508
TGIF2LX	0	2	1.000	0.000	0.000	134.508
TGM4	0	2	1.000	0.000	0.000	134.508
TGM5	0	2	1.000	0.000	0.000	134.508
TGM7	0	2	1.000	0.000	0.000	134.508
THADA	0	2	1.000	0.000	0.000	134.508
THAP4	0	2	1.000	0.000	0.000	134.508
THAP9	0	2	1.000	0.000	0.000	134.508
THBS1	0	2	1.000	0.000	0.000	134.508
THBS4	0	3	1.000	0.000	0.000	64.152
THOC2	0	2	1.000	0.000	0.000	134.508
THOP1	0	2	1.000	0.000	0.000	134.508
THRAP3	0	2	1.000	0.000	0.000	134.508
TIAM1	0	5	1.000	0.000	0.000	30.501
TIAM2	0	3	1.000	0.000	0.000	64.152
TICRR	0	2	1.000	0.000	0.000	134.508
TIE1	0	2	1.000	0.000	0.000	134.508
TIGD2	0	2	1.000	0.000	0.000	134.508
TIGD7	0	2	1.000	0.000	0.000	134.508
TIMELESS	0	3	1.000	0.000	0.000	64.152
TIMM44	0	2	1.000	0.000	0.000	134.508
TINF2	0	2	1.000	0.000	0.000	134.508
TLE3	0	2	1.000	0.000	0.000	134.508
TLE4	0	2	1.000	0.000	0.000	134.508
TLK2	0	3	1.000	0.000	0.000	64.152
TLL2	0	3	1.000	0.000	0.000	64.152
TLN1	0	2	1.000	0.000	0.000	134.508
TLN2	0	2	1.000	0.000	0.000	134.508
TLR6	0	3	1.000	0.000	0.000	64.152
TLR8	0	2	1.000	0.000	0.000	134.508
TM9SF1	0	2	1.000	0.000	0.000	134.508
TM9SF2	0	2	1.000	0.000	0.000	134.508
TMBIM4	0	2	1.000	0.000	0.000	134.508
TMC4	0	2	1.000	0.000	0.000	134.508
TMC5	0	3	1.000	0.000	0.000	64.152
TMEM104	0	2	1.000	0.000	0.000	134.508
TMEM106C	0	2	1.000	0.000	0.000	134.508
TMEM120B	0	2	1.000	0.000	0.000	134.508
TMEM131	0	2	1.000	0.000	0.000	134.508
TMEM132B	0	3	1.000	0.000	0.000	64.152
TMEM14B	0	4	1.000	0.000	0.000	41.489
TMEM175	0	3	1.000	0.000	0.000	64.152
TMEM177	0	2	1.000	0.000	0.000	134.508

TMEM2	0	2	1.000	0.000	0.000	134.508
TMEM200A	0	2	1.000	0.000	0.000	134.508
TMEM45B	0	2	1.000	0.000	0.000	134.508
TMEM52	0	2	1.000	0.000	0.000	134.508
TMEM57	0	2	1.000	0.000	0.000	134.508
TMEM87A	0	2	1.000	0.000	0.000	134.508
TMF1	0	4	1.000	0.000	0.000	41.489
TMIGD2	0	2	1.000	0.000	0.000	134.508
TMPRSS11D	0	2	1.000	0.000	0.000	134.508
TMPRSS11E	0	2	1.000	0.000	0.000	134.508
TMPRSS3	0	2	1.000	0.000	0.000	134.508
TMPRSS6	0	2	1.000	0.000	0.000	134.508
TMX4	0	2	1.000	0.000	0.000	134.508
TNC	0	4	1.000	0.000	0.000	41.489
TNFRSF11A	0	2	1.000	0.000	0.000	134.508
TNFRSF11B	0	2	1.000	0.000	0.000	134.508
TNFRSF1A	0	2	1.000	0.000	0.000	134.508
TNFSF14	0	2	1.000	0.000	0.000	134.508
TNIK	0	2	1.000	0.000	0.000	134.508
TNIP3	0	2	1.000	0.000	0.000	134.508
TNKS	0	4	1.000	0.000	0.000	41.489
TNKS1BP1	0	2	1.000	0.000	0.000	134.508
TNN	0	2	1.000	0.000	0.000	134.508
TNNI3	0	2	1.000	0.000	0.000	134.508
TNNT3	0	2	1.000	0.000	0.000	134.508
TNPO2	0	3	1.000	0.000	0.000	64.152
TNR	0	4	1.000	0.000	0.000	41.489
TNRC6B	0	2	1.000	0.000	0.000	134.508
TNS1	0	2	1.000	0.000	0.000	134.508
TONSL	0	2	1.000	0.000	0.000	134.508
TOP2B	0	2	1.000	0.000	0.000	134.508
TOPBP1	0	2	1.000	0.000	0.000	134.508
TOR1AIP1	0	2	1.000	0.000	0.000	134.508
TOX4	0	2	1.000	0.000	0.000	134.508
TP53	3	74	1.000	0.906	0.117	6.999
TP53BP2	0	2	1.000	0.000	0.000	134.508
TP53I13	0	2	1.000	0.000	0.000	134.508
TP63	0	3	1.000	0.000	0.000	64.152
TPCN1	0	2	1.000	0.000	0.000	134.508
TPH1	0	2	1.000	0.000	0.000	134.508
TPM1	0	2	1.000	0.000	0.000	134.508
TPO	0	5	1.000	0.000	0.000	30.501
TPR	0	3	1.000	0.000	0.000	64.152
TPSAB1	0	2	1.000	0.000	0.000	134.508
TPSD1	0	3	1.000	0.000	0.000	64.152
TPTE	0	5	1.000	0.000	0.000	30.501
TPTE2	0	3	1.000	0.000	0.000	64.152
TRAK1	0	2	1.000	0.000	0.000	134.508
TRAM1L1	0	2	1.000	0.000	0.000	134.508
TRANK1	0	2	1.000	0.000	0.000	134.508
TRAPPC10	0	2	1.000	0.000	0.000	134.508
TRAPPC8	0	4	1.000	0.000	0.000	41.489
TRAPPC9	0	2	1.000	0.000	0.000	134.508
TRH	0	2	1.000	0.000	0.000	134.508
TRHDE	0	2	1.000	0.000	0.000	134.508
TRIM16	0	2	1.000	0.000	0.000	134.508
TRIM16L	0	2	1.000	0.000	0.000	134.508
TRIM2	0	2	1.000	0.000	0.000	134.508
TRIM26	0	2	1.000	0.000	0.000	134.508
TRIM33	0	2	1.000	0.000	0.000	134.508
TRIM36	0	2	1.000	0.000	0.000	134.508
TRIM37	0	3	1.000	0.000	0.000	64.152
TRIM38	0	2	1.000	0.000	0.000	134.508
TRIM49	0	3	1.000	0.000	0.000	64.152
TRIM5	0	2	1.000	0.000	0.000	134.508
TRIM51	0	3	1.000	0.000	0.000	64.152

TRIM58	0	2	1.000	0.000	0.000	134.508
TRIO	0	3	1.000	0.000	0.000	64.152
TRIP12	0	3	1.000	0.000	0.000	64.152
TRNAU1AP	0	2	1.000	0.000	0.000	134.508
TRO	0	2	1.000	0.000	0.000	134.508
TROAP	0	2	1.000	0.000	0.000	134.508
TRPA1	0	2	1.000	0.000	0.000	134.508
TRPC3	0	3	1.000	0.000	0.000	64.152
TRPC4	0	2	1.000	0.000	0.000	134.508
TRPC6	0	3	1.000	0.000	0.000	64.152
TRPC7	0	4	1.000	0.000	0.000	41.489
TRPM1	0	2	1.000	0.000	0.000	134.508
TRPM3	0	3	1.000	0.000	0.000	64.152
TRPM4	0	3	1.000	0.000	0.000	64.152
TRPM6	0	4	1.000	0.000	0.000	41.489
TRPS1	0	3	1.000	0.000	0.000	64.152
TRPV3	0	2	1.000	0.000	0.000	134.508
TRPV4	0	2	1.000	0.000	0.000	134.508
TRPV6	0	3	1.000	0.000	0.000	64.152
TRRAP	0	2	1.000	0.000	0.000	134.508
TSC1	0	2	1.000	0.000	0.000	134.508
TSC2	0	2	1.000	0.000	0.000	134.508
TSHZ2	0	4	1.000	0.000	0.000	41.489
TSHZ3	0	3	1.000	0.000	0.000	64.152
TSPAN11	0	2	1.000	0.000	0.000	134.508
TSPAN17	0	2	1.000	0.000	0.000	134.508
TTC18	0	3	1.000	0.000	0.000	64.152
TTC21B	0	2	1.000	0.000	0.000	134.508
TTC3	0	2	1.000	0.000	0.000	134.508
TTC37	0	2	1.000	0.000	0.000	134.508
TTC39C	0	2	1.000	0.000	0.000	134.508
TTC8	0	2	1.000	0.000	0.000	134.508
TTC9	0	2	1.000	0.000	0.000	134.508
TTI1	0	2	1.000	0.000	0.000	134.508
TTK	0	2	1.000	0.000	0.000	134.508
TTLL4	0	2	1.000	0.000	0.000	134.508
TTLL5	0	3	1.000	0.000	0.000	64.152
TTLL7	0	3	1.000	0.000	0.000	64.152
TTLL8	0	2	1.000	0.000	0.000	134.508
TTYH2	0	2	1.000	0.000	0.000	134.508
TUBA3C	0	4	1.000	0.000	0.000	41.489
TUBA8	0	2	1.000	0.000	0.000	134.508
TUBB1	0	2	1.000	0.000	0.000	134.508
TUBGCP3	0	2	1.000	0.000	0.000	134.508
TVP23C	0	2	1.000	0.000	0.000	134.508
TXK	0	3	1.000	0.000	0.000	64.152
TXLNB	0	2	1.000	0.000	0.000	134.508
TYR	0	2	1.000	0.000	0.000	134.508
U2AF1	0	3	1.000	0.000	0.000	64.152
U2SURP	0	3	1.000	0.000	0.000	64.152
UBA2	0	3	1.000	0.000	0.000	64.152
UBA5	0	2	1.000	0.000	0.000	134.508
UBA7	0	3	1.000	0.000	0.000	64.152
UBAP2	0	2	1.000	0.000	0.000	134.508
UBASH3B	0	4	1.000	0.000	0.000	41.489
UBE2D4	0	4	1.000	0.000	0.000	41.489
UBE2J1	0	2	1.000	0.000	0.000	134.508
UBE3A	0	2	1.000	0.000	0.000	134.508
UBE3C	0	2	1.000	0.000	0.000	134.508
UBN2	0	2	1.000	0.000	0.000	134.508
UBQLN3	0	2	1.000	0.000	0.000	134.508
UBR4	0	2	1.000	0.000	0.000	134.508
UBTF	0	2	1.000	0.000	0.000	134.508
UBXN11	0	3	1.000	0.000	0.000	64.152
UGCG	0	2	1.000	0.000	0.000	134.508
UGDH	0	2	1.000	0.000	0.000	134.508

UGGT2	0	2	1.000	0.000	0.000	134.508
UGP2	0	3	1.000	0.000	0.000	64.152
UGT1A4	0	2	1.000	0.000	0.000	134.508
UGT3A2	0	2	1.000	0.000	0.000	134.508
UHRF1BP1L	0	3	1.000	0.000	0.000	64.152
ULK1	0	2	1.000	0.000	0.000	134.508
ULK4	0	2	1.000	0.000	0.000	134.508
UMOD	0	3	1.000	0.000	0.000	64.152
UMODL1	0	2	1.000	0.000	0.000	134.508
UNC13A	0	2	1.000	0.000	0.000	134.508
UNC13C	0	3	1.000	0.000	0.000	64.152
UNC5B	0	2	1.000	0.000	0.000	134.508
UNC5D	0	2	1.000	0.000	0.000	134.508
UNC79	0	4	1.000	0.000	0.000	41.489
UPF3A	0	2	1.000	0.000	0.000	134.508
UROC1	0	2	1.000	0.000	0.000	134.508
USHBP1	0	2	1.000	0.000	0.000	134.508
USP10	0	2	1.000	0.000	0.000	134.508
USP14	0	2	1.000	0.000	0.000	134.508
USP16	0	2	1.000	0.000	0.000	134.508
USP2	0	2	1.000	0.000	0.000	134.508
USP24	0	2	1.000	0.000	0.000	134.508
USP25	0	3	1.000	0.000	0.000	64.152
USP26	0	3	1.000	0.000	0.000	64.152
USP28	0	2	1.000	0.000	0.000	134.508
USP31	0	2	1.000	0.000	0.000	134.508
USP33	0	2	1.000	0.000	0.000	134.508
USP34	0	2	1.000	0.000	0.000	134.508
USP35	0	2	1.000	0.000	0.000	134.508
USP37	0	2	1.000	0.000	0.000	134.508
USP40	0	2	1.000	0.000	0.000	134.508
USP48	0	2	1.000	0.000	0.000	134.508
USP6	0	3	1.000	0.000	0.000	64.152
USP8	0	3	1.000	0.000	0.000	64.152
USP9X	0	2	1.000	0.000	0.000	134.508
UTP11L	0	2	1.000	0.000	0.000	134.508
UTP15	0	2	1.000	0.000	0.000	134.508
UTRN	0	3	1.000	0.000	0.000	64.152
UVRAG	0	5	1.000	0.000	0.000	30.501
UXS1	0	2	1.000	0.000	0.000	134.508
VAC14	0	2	1.000	0.000	0.000	134.508
VANGL1	0	2	1.000	0.000	0.000	134.508
VAV2	0	2	1.000	0.000	0.000	134.508
VCAN	0	4	1.000	0.000	0.000	41.489
VCL	0	3	1.000	0.000	0.000	64.152
VENTX	0	2	1.000	0.000	0.000	134.508
VIPR1	0	2	1.000	0.000	0.000	134.508
VKORC1	0	2	1.000	0.000	0.000	134.508
VPS13A	0	2	1.000	0.000	0.000	134.508
VPS13B	0	4	1.000	0.000	0.000	41.489
VPS13C	0	2	1.000	0.000	0.000	134.508
VPS13D	0	8	1.000	0.000	0.000	16.757
VPS25	0	2	1.000	0.000	0.000	134.508
VPS33B	0	2	1.000	0.000	0.000	134.508
VPS37B	0	2	1.000	0.000	0.000	134.508
VSX2	0	2	1.000	0.000	0.000	134.508
VWA3B	0	3	1.000	0.000	0.000	64.152
VWA5A	0	2	1.000	0.000	0.000	134.508
VWA7	0	2	1.000	0.000	0.000	134.508
VWA8	0	5	1.000	0.000	0.000	30.501
VWA9	0	3	1.000	0.000	0.000	64.152
VWF	0	4	1.000	0.000	0.000	41.489
WAC	0	2	1.000	0.000	0.000	134.508
WASF3	0	2	1.000	0.000	0.000	134.508
WASL	0	2	1.000	0.000	0.000	134.508
WBP11	0	2	1.000	0.000	0.000	134.508

WBSCR17	0	3	1.000	0.000	0.000	64.152
WDHD1	0	2	1.000	0.000	0.000	134.508
WDR17	0	4	1.000	0.000	0.000	41.489
WDR3	0	2	1.000	0.000	0.000	134.508
WDR31	0	2	1.000	0.000	0.000	134.508
WDR33	0	2	1.000	0.000	0.000	134.508
WDR35	0	3	1.000	0.000	0.000	64.152
WDR36	0	2	1.000	0.000	0.000	134.508
WDR37	0	2	1.000	0.000	0.000	134.508
WDR48	0	3	1.000	0.000	0.000	64.152
WDR52	0	2	1.000	0.000	0.000	134.508
WDR6	0	2	1.000	0.000	0.000	134.508
WDR62	0	3	1.000	0.000	0.000	64.152
WDR63	0	2	1.000	0.000	0.000	134.508
WDR78	0	5	1.000	0.000	0.000	30.501
WDR89	0	2	1.000	0.000	0.000	134.508
WDR90	0	2	1.000	0.000	0.000	134.508
WEE2	0	2	1.000	0.000	0.000	134.508
WFIKKN2	0	2	1.000	0.000	0.000	134.508
WIPI1	0	2	1.000	0.000	0.000	134.508
WIPI2	0	2	1.000	0.000	0.000	134.508
WNK4	0	2	1.000	0.000	0.000	134.508
WNT2	0	2	1.000	0.000	0.000	134.508
WNT2B	0	2	1.000	0.000	0.000	134.508
WNT3A	0	4	1.000	0.000	0.000	41.489
WNT5A	0	2	1.000	0.000	0.000	134.508
WNT7B	0	2	1.000	0.000	0.000	134.508
WRN	0	2	1.000	0.000	0.000	134.508
WSB2	0	2	1.000	0.000	0.000	134.508
WTAP	0	3	1.000	0.000	0.000	64.152
WWC3	0	2	1.000	0.000	0.000	134.508
XCR1	0	2	1.000	0.000	0.000	134.508
XDH	0	2	1.000	0.000	0.000	134.508
XIRP1	0	4	1.000	0.000	0.000	41.489
XIRP2	0	3	1.000	0.000	0.000	64.152
XPC	0	2	1.000	0.000	0.000	134.508
XPO7	0	3	1.000	0.000	0.000	64.152
XRRA1	0	2	1.000	0.000	0.000	134.508
YLPM1	0	2	1.000	0.000	0.000	134.508
YTHDC1	0	3	1.000	0.000	0.000	64.152
YTHDF1	0	3	1.000	0.000	0.000	64.152
ZAN	0	4	1.000	0.000	0.000	41.489
ZBBX	0	2	1.000	0.000	0.000	134.508
ZBED4	0	2	1.000	0.000	0.000	134.508
ZBTB11	0	3	1.000	0.000	0.000	64.152
ZBTB22	0	2	1.000	0.000	0.000	134.508
ZBTB41	0	2	1.000	0.000	0.000	134.508
ZBTB45	0	2	1.000	0.000	0.000	134.508
ZC2HC1A	0	3	1.000	0.000	0.000	64.152
ZC3H12A	0	3	1.000	0.000	0.000	64.152
ZC3H12B	0	2	1.000	0.000	0.000	134.508
ZC3H12C	0	2	1.000	0.000	0.000	134.508
ZC3H15	0	3	1.000	0.000	0.000	64.152
ZC3H7A	0	2	1.000	0.000	0.000	134.508
ZC3HC1	0	3	1.000	0.000	0.000	64.152
ZCCHC11	0	2	1.000	0.000	0.000	134.508
ZCCHC14	0	2	1.000	0.000	0.000	134.508
ZCCHC4	0	2	1.000	0.000	0.000	134.508
ZCCHC6	0	2	1.000	0.000	0.000	134.508
ZDBF2	0	3	1.000	0.000	0.000	64.152
ZDHHC5	0	2	1.000	0.000	0.000	134.508
ZEB1	0	3	1.000	0.000	0.000	64.152
ZEB2	0	2	1.000	0.000	0.000	134.508
ZFAT	0	2	1.000	0.000	0.000	134.508
ZFC3H1	0	2	1.000	0.000	0.000	134.508
ZFHX3	0	3	1.000	0.000	0.000	64.152

ZFHX4	0	4	1.000	0.000	0.000	41.489
ZFP42	0	2	1.000	0.000	0.000	134.508
ZFP57	0	2	1.000	0.000	0.000	134.508
ZFP69	0	2	1.000	0.000	0.000	134.508
ZFP91	0	3	1.000	0.000	0.000	64.152
ZFPM2	0	3	1.000	0.000	0.000	64.152
ZFYVE16	0	2	1.000	0.000	0.000	134.508
ZFYVE20	0	2	1.000	0.000	0.000	134.508
ZFYVE9	0	3	1.000	0.000	0.000	64.152
ZHX3	0	2	1.000	0.000	0.000	134.508
ZIK1	0	2	1.000	0.000	0.000	134.508
ZIM3	0	2	1.000	0.000	0.000	134.508
ZKSCAN2	0	2	1.000	0.000	0.000	134.508
ZKSCAN7	0	3	1.000	0.000	0.000	64.152
ZMAT1	0	2	1.000	0.000	0.000	134.508
ZMIZ2	0	3	1.000	0.000	0.000	64.152
ZMYM3	0	2	1.000	0.000	0.000	134.508
ZMYND11	0	2	1.000	0.000	0.000	134.508
ZMYND12	0	2	1.000	0.000	0.000	134.508
ZMYND8	0	3	1.000	0.000	0.000	64.152
ZNF106	0	2	1.000	0.000	0.000	134.508
ZNF114	0	3	1.000	0.000	0.000	64.152
ZNF136	0	3	1.000	0.000	0.000	64.152
ZNF141	0	4	1.000	0.000	0.000	41.489
ZNF142	0	4	1.000	0.000	0.000	41.489
ZNF148	0	2	1.000	0.000	0.000	134.508
ZNF155	0	2	1.000	0.000	0.000	134.508
ZNF175	0	2	1.000	0.000	0.000	134.508
ZNF180	0	2	1.000	0.000	0.000	134.508
ZNF184	0	3	1.000	0.000	0.000	64.152
ZNF208	0	3	1.000	0.000	0.000	64.152
ZNF215	0	2	1.000	0.000	0.000	134.508
ZNF217	0	2	1.000	0.000	0.000	134.508
ZNF224	0	2	1.000	0.000	0.000	134.508
ZNF229	0	2	1.000	0.000	0.000	134.508
ZNF239	0	2	1.000	0.000	0.000	134.508
ZNF257	0	2	1.000	0.000	0.000	134.508
ZNF264	0	2	1.000	0.000	0.000	134.508
ZNF274	0	2	1.000	0.000	0.000	134.508
ZNF28	0	3	1.000	0.000	0.000	64.152
ZNF280D	0	4	1.000	0.000	0.000	41.489
ZNF285	0	17	1.000	0.000	0.000	6.757
ZNF292	0	3	1.000	0.000	0.000	64.152
ZNF3	0	3	1.000	0.000	0.000	64.152
ZNF311	0	2	1.000	0.000	0.000	134.508
ZNF318	0	3	1.000	0.000	0.000	64.152
ZNF326	0	2	1.000	0.000	0.000	134.508
ZNF335	0	2	1.000	0.000	0.000	134.508
ZNF345	0	2	1.000	0.000	0.000	134.508
ZNF365	0	2	1.000	0.000	0.000	134.508
ZNF366	0	2	1.000	0.000	0.000	134.508
ZNF382	0	2	1.000	0.000	0.000	134.508
ZNF385B	0	2	1.000	0.000	0.000	134.508
ZNF385D	0	3	1.000	0.000	0.000	64.152
ZNF420	0	2	1.000	0.000	0.000	134.508
ZNF423	0	2	1.000	0.000	0.000	134.508
ZNF430	0	2	1.000	0.000	0.000	134.508
ZNF431	0	2	1.000	0.000	0.000	134.508
ZNF441	0	2	1.000	0.000	0.000	134.508
ZNF443	0	2	1.000	0.000	0.000	134.508
ZNF45	0	2	1.000	0.000	0.000	134.508
ZNF470	0	2	1.000	0.000	0.000	134.508
ZNF471	0	4	1.000	0.000	0.000	41.489
ZNF473	0	2	1.000	0.000	0.000	134.508
ZNF480	0	3	1.000	0.000	0.000	64.152
ZNF485	0	2	1.000	0.000	0.000	134.508

ZNF491	0	2	1.000	0.000	0.000	134.508
ZNF492	0	2	1.000	0.000	0.000	134.508
ZNF493	0	3	1.000	0.000	0.000	64.152
ZNF496	0	3	1.000	0.000	0.000	64.152
ZNF497	0	2	1.000	0.000	0.000	134.508
ZNF500	0	3	1.000	0.000	0.000	64.152
ZNF502	0	2	1.000	0.000	0.000	134.508
ZNF512B	0	2	1.000	0.000	0.000	134.508
ZNF516	0	3	1.000	0.000	0.000	64.152
ZNF527	0	2	1.000	0.000	0.000	134.508
ZNF528	0	2	1.000	0.000	0.000	134.508
ZNF532	0	2	1.000	0.000	0.000	134.508
ZNF536	0	2	1.000	0.000	0.000	134.508
ZNF544	0	2	1.000	0.000	0.000	134.508
ZNF565	0	2	1.000	0.000	0.000	134.508
ZNF568	0	2	1.000	0.000	0.000	134.508
ZNF569	0	2	1.000	0.000	0.000	134.508
ZNF585A	0	4	1.000	0.000	0.000	41.489
ZNF585B	0	3	1.000	0.000	0.000	64.152
ZNF586	0	2	1.000	0.000	0.000	134.508
ZNF600	0	2	1.000	0.000	0.000	134.508
ZNF607	0	2	1.000	0.000	0.000	134.508
ZNF611	0	2	1.000	0.000	0.000	134.508
ZNF613	0	2	1.000	0.000	0.000	134.508
ZNF616	0	2	1.000	0.000	0.000	134.508
ZNF619	0	2	1.000	0.000	0.000	134.508
ZNF624	0	2	1.000	0.000	0.000	134.508
ZNF625	0	2	1.000	0.000	0.000	134.508
ZNF628	0	2	1.000	0.000	0.000	134.508
ZNF638	0	2	1.000	0.000	0.000	134.508
ZNF644	0	2	1.000	0.000	0.000	134.508
ZNF648	0	2	1.000	0.000	0.000	134.508
ZNF662	0	3	1.000	0.000	0.000	64.152
ZNF667	0	2	1.000	0.000	0.000	134.508
ZNF671	0	2	1.000	0.000	0.000	134.508
ZNF675	0	2	1.000	0.000	0.000	134.508
ZNF676	0	3	1.000	0.000	0.000	64.152
ZNF681	0	3	1.000	0.000	0.000	64.152
ZNF695	0	2	1.000	0.000	0.000	134.508
ZNF70	0	3	1.000	0.000	0.000	64.152
ZNF701	0	2	1.000	0.000	0.000	134.508
ZNF708	0	2	1.000	0.000	0.000	134.508
ZNF709	0	2	1.000	0.000	0.000	134.508
ZNF721	0	2	1.000	0.000	0.000	134.508
ZNF729	0	2	1.000	0.000	0.000	134.508
ZNF749	0	2	1.000	0.000	0.000	134.508
ZNF750	0	2	1.000	0.000	0.000	134.508
ZNF75A	0	2	1.000	0.000	0.000	134.508
ZNF772	0	2	1.000	0.000	0.000	134.508
ZNF773	0	2	1.000	0.000	0.000	134.508
ZNF777	0	2	1.000	0.000	0.000	134.508
ZNF780B	0	2	1.000	0.000	0.000	134.508
ZNF783	0	2	1.000	0.000	0.000	134.508
ZNF787	0	2	1.000	0.000	0.000	134.508
ZNF790	0	3	1.000	0.000	0.000	64.152
ZNF804A	0	3	1.000	0.000	0.000	64.152
ZNF804B	0	2	1.000	0.000	0.000	134.508
ZNF813	0	2	1.000	0.000	0.000	134.508
ZNF814	0	2	1.000	0.000	0.000	134.508
ZNF83	0	3	1.000	0.000	0.000	64.152
ZNF831	0	2	1.000	0.000	0.000	134.508
ZNF835	0	3	1.000	0.000	0.000	64.152
ZNF845	0	2	1.000	0.000	0.000	134.508
ZNF860	0	2	1.000	0.000	0.000	134.508
ZNF880	0	2	1.000	0.000	0.000	134.508
ZNF99	0	3	1.000	0.000	0.000	64.152

ZNFX1	0	2	1.000	0.000	0.000	134.508
ZNRF4	0	4	1.000	0.000	0.000	41.489
ZSCAN22	0	2	1.000	0.000	0.000	134.508
ZSWIM2	0	2	1.000	0.000	0.000	134.508
ZW10	0	3	1.000	0.000	0.000	64.152
ZWINT	0	2	1.000	0.000	0.000	134.508
ZXDC	0	2	1.000	0.000	0.000	134.508
ZYG11B	0	2	1.000	0.000	0.000	134.508
ZZZ3	0	5	1.000	0.000	0.000	30.501

Table S3. Motif enrichment scores of FUSCC cohort A stratified by A3C expression.
Motif enrichment scores were calculated according to Dmitry's formulas.

Mutations at C:G bp were treated as C>X mutations, and C>A mutations were excluded. Let MutC = number of C>X mutations, MutTCA = number of TCA>TXA mutations, ConC = number of C or G within the 41-mers centred on each mutation, and ConTCA = number of TCA or TGA with in the 41-mers centred on each mutation. E-TCA = (MutTCA × ConC)/(MutC × ConTCA). E-TC and E-TCW were calculated analogously.

A3C high group consisted of 25 samples with highest A3C expression in FUSCC cohort A.
A3C low group consisted of 25 samples with lowest A3C expression in FUSCC cohort A.
Enrichment score ≤1 denoted no enrichment.
p values were calculated by Welch's t test.

	A3C high group		A3C low group		
Motif	Enrichment score	SD	Enrichment score	SD	p value
CG	3.417	0.02438	3.424	0.02635	0.2968
AC	1.470	0.01359	1.480	0.01455	0.0193
CC	1.319	0.01278	1.310	0.01141	0.0093
TC	0.789	0.00926	0.791	0.00926	0.5151
TCW	0.587	0.01623	0.587	0.00938	0.9196
TCA	0.508	0.02064	0.506	0.01606	0.6058
YTCA	0.521	0.02522	0.522	0.01916	0.8853
RTCA	0.534	0.02669	0.526	0.02603	0.3082

Table S4. CCDS of APOBECs and their identities to APOBEC3C.
CCDS: consensus coding sequence; MW: molecular weight.

Gene	Location	Gene region (FASTA)	CCDS ID	MW (kd)	Identity to A3C
AICDA	12p13.31	8602170 to 8612970	41747.1	23.96	74%
			81662.1	22.62	74%
APOBEC1	12p13.31	7649400 to 7670599	8579.1	28.2	<60%
APOBEC2	6p21.1	41053202 to 41064891	4848.1	25.71	<60%
APOBEC3A	22q13.1	38957609 to 38963184	13981.1	23.02	<60%
APOBEC3B	22q13.1	38982347 to 38992779	13982.1	45.93	72%
			58807.1	43.09	72%
APOBEC3C	22q13.1	39014257 to 39020352	13983.1	22.83	100%
APOBEC3D	22q13.1	39021127 to 39033277	46709.1	46.6	91%
	22q13.1		87027.1	24.15	93%
APOBEC3F	22q13.1	39019081 to 39055972	33648.1	45.02	90%
	22q13.1		33649.1	11.83	90%
APOBEC3G	22q13.1	39077005 to 39087743	13984.1	46.41	71%
APOBEC3H	22q13.2	39097203 to 39104067	13985.1	21.66	64%
	22q13.1		54530.1	23.54	64%
	22q13.1		54531.1	21.53	64%
	22q13.1		54532.1	17.79	64%
APOBEC4	1q25.3	183646275 to 183653316	1358.1	41.59	<60%

Table S5. Correlation of APOBEC isoforms expression in transcriptomics.

*: Correlation is significant with p value < 0.05 (2-tailed),

**: Correlation is significant with p value < 0.01 level (2-tailed),

r: pearson correlation test.

The AICDA, A3A, A3H, A4 expression data of QCMG PDAC cohort was missing (downloaded from cBioPortal).

			AICDA	A1	A2	A4	A3A	A3B	A3C	A3D	A3F	A3G
TCGA cohort (n = 163)	A3H	r	0.519**	-0.148	0.010	-0.044	0.357**	0.045	0.455**	0.679**	0.567**	0.784**
		p	<0.001	0.06	0.898	0.577	<0.001	0.572	<0.001	<0.001	<0.001	<0.001
	A3G	r	0.539**	-0.219**	0.008	0.077	0.286**	0.286**	0.606**	0.736**	0.725**	
		p	<0.001	0.005	0.921	0.328	<0.001	<0.001	<0.001	<0.001	<0.001	
	A3F	r	0.520**	-0.195*	0.152	-0.042	0.151	0.157*	0.516**	0.755**		
		p	<0.001	0.013	0.052	0.591	0.055	0.045	<0.001	<0.001		
	A3D	r	0.664**	-0.025	-0.134	-0.041	0.147	0.136	0.594**			
		p	<0.001	0.752	0.088	0.602	0.061	0.084	<0.001			
	A3C	r	0.357**	-0.309**	-0.042	0.022	0.004	0.094				
		p	<0.001	<0.001	0.593	0.785	0.964	0.231				
	A3B	r	-0.006	0.265**	-0.068	0.008	0.198*					
		p	0.994	0.001	0.392	0.923	0.011					
	A3A	r	0.223**	-0.020	-0.110	0.002						
		p	0.004	0.800	0.162	0.976						
	A4	r	-0.027	0.059	-0.060							
		p	0.736	0.454	0.445							
	A2	r	-0.104	-0.108								
		p	0.187	0.171								
	A1	r	-0.077									
		p	0.326									

			AICDA	A1	A2	A4	A3A	A3B	A3C	A3D	A3F	A3G
FUSCC cohort A (n = 124)	A3H	r	0.245**	-0.303**	0.193*	-0.098	0.017	-0.103	0.365**	0.512**	0.426**	0.640**
		p	0.006	0.001	0.032	0.278	0.852	0.254	<0.001	<0.001	<0.001	<0.001
	A3G	r	0.382**	-0.312**	0.175	-0.154	-0.060	0.178*	0.764**	0.841**	0.749**	
		p	<0.001	<0.001	0.051	0.088	0.506	0.048	<0.001	<0.001	<0.001	
	A3F	r	0.236**	-0.230*	0.142	-0.101	0.046	0.360**	0.827**	0.778**		
		p	0.008	0.010	0.116	0.262	0.615	<0.001	<0.001	<0.001		
	A3D	r	0.465**	-0.185*	-0.015	-0.140	-0.042	0.216*	0.806**			
		p	<0.001	0.040	0.868	0.121	0.642	0.016	<0.001			
	A3C	r	0.260**	-0.165	-0.006	-0.073	-0.126	0.400**				
		p	0.004	0.068	0.949	0.423	0.163	<0.001				
	A3B	r	-0.023	-0.023	-0.037	0.154	-0.012					
		p	0.804	0.798	0.685	0.088	0.897					
	A3A	r	-0.067	0.029	-0.036	-0.040						
		p	0.459	0.751	0.695	0.659						
	A4	r	-0.087	0.213*	-0.040							
		p	0.335	0.017	0.656							
	A2	r	-0.093	-0.203*								
		p	0.307	0.023								
	A1	r	-0.119									
		p	0.188									

			A1	A2	A3B	A3C	A3D	A3F
QCMG cohort (n = 70)	A3G	r	-0.083	-0.077	-0.020	0.002	0.070	0.151
		p	0.494	0.528	0.868	0.990	0.563	0,211
	A3F	r	-0.051	-0.053	0.772**	0.796**	0.805**	
		p	0.676	0.660	<0.001	<0.001	<0.001	
	A3D	r	0.024	-0.071	0.782**	0.717**		
		p	0.841	0.559	<0.001	<0.001		
	A3C	r	-0.029	0.009	0.966**			
		p	0.813	0.938	<0.001			
	A3B	r	-0.055	-0.048				
		p	0.654	0.692				
	A2	r	-0.055					
		p	0.652					

Table S6. Multiple linear regression of APOBECs expression correlating to A3C expression. APOBECs whose expression correlated with A3C expression (Table S5, p<0.05) were included in multiple linear regression

CI: confidence interval.

QCMG PDAC dataset was excluded from multiple linear regression because of data missing.

	R square = 0.485, F = 24.466, p < 0.001			
	Multiple center uploaded data with unknown methods for isoforms quantification			
	Gene	B	95% CI	P value
TCGA cohort (n = 163)	AICDA	-0.536	(-1.291, 0.219)	0.163
	A1	-0.104	(-0.154, -0.055)	<0.001
	A3D	**0.809**	(0.469, 1.149)	**<0.001**
	A3F	-0.114	(-0.399, 0.172)	0.433
	A3G	0.262	(0.111, 0.414)	0.001
	A3H	-1.821	(-4.151, 0.509)	0.125

	R square = 0.788, F = 72.545, p < 0.001			
	RSEM method for isoforms quantification			
	Gene	B	95% CI	P value
FUSCC cohort A (n = 124)	AICDA	-2.275	(-5.813, 1.262)	0.205
	A3B	0.361	(0.098, 0.623)	0.008
	A3D	**1.905**	(0.950, 2.860)	**<0.001**
	A3F	**2.589**	(1.536, 3.643)	**<0.001**
	A3G	0.381	(0.106, 0.656)	0.007
	A3H	-1.268	(-2.567, 0.031)	0.056

Table S7. Location of all kataegis detected in FUSCC cohort A.
SNVs: single nucleotide variants; TSS: Translation Start Site.

Patient ID	Chrom arm	Start	End	Length	SNVs	weight.C>X	Annotation	Distance To TSS	Gene	Gene ID
354287	1p	978953	979847	895	6	0.5	Promoter(1-2kb)	1182	PERM1	84808
354287	1p	12893187	12893472	286	6	0.5	Exon(exon4of4)	4798	PRAMEF10	343071
354287	1p	16058491	16060000	1510	11	0.909	Exon(exon5of7)	6168	CLCNKB	1188
354287	1p	18481403	18482217	815	6	0.667	Promoter(<=1kb)	421	KLHDC7A	127707
354287	1p	23874604	23875430	827	9	0.556	Exon(exon2of2)	-6310	FUCA1	2517
354287	1p	40067594	40067675	82	6	0	Promoter(<=1kb)	324	CAP1	10487
354287	1q	145872200	145874909	2710	6	0.667	Exon(exon6of12)	10957	ANKRD35	148741
354287	1q	152213286	152213347	62	8	0.5	Exon(exon3of3)	10846	HRNR	388697
354287	1q	152219233	152221375	2143	16	0.75	Promoter(2-3kb)	2818	HRNR	388697
354287	1q	158765805	158766655	851	6	0.5	Promoter(<=1kb)	47	OR6N1	128372
354287	1q	169540901	169542882	1982	10	0.3	Exon(exon13of25)	-25156	F5	2153
354287	1q	201206099	201209342	3244	9	0.667	Promoter(1-2kb)	1017	IGFN1	91156
354287	1q	201210956	201212792	1837	11	0.455	Promoter(<=1kb)	0	IGFN1	91156
354287	1q	228315976	228318038	2063	8	0.625	Exon(exon50of81)	6492	OBSCN	84033
354287	1q	233379090	233379667	578	6	0.833	Exon(exon9of10)	3307	MAP3K21	84451
354287	1q	247841312	247841582	271	6	0.833	Promoter(<=1kb)	314	OR11L1	391189
354287	1q	247921132	247921847	716	6	0.833	Promoter(<=1kb)	114	OR2T8	343172
354287	1q	247949325	247949738	414	10	0.3	Promoter(<=1kb)	467	OR2L8	391190
354287	1q	248273309	248273670	362	6	0.5	Promoter(<=1kb)	166	OR2T33	391195
354287	1q	248294677	248295458	782	7	0.571	Promoter(<=1kb)	142	OR2T12	127064
354287	1q	248559358	248559487	130	8	0.375	Promoter(<=1kb)	9	OR2T5	401993
354287	2q	102351547	102351902	356	7	0.429	Exon(exon11of11)	-4027	IL18R1	8809
354287	2q	132783032	132785125	2094	8	0.625	Promoter(1-2kb)	-1009	NCKAP5	344148
354287	2q	177617090	177618246	1157	6	0.5	Promoter(<=1kb)	496	TTC30A	92104
354287	2q	178739433	178741811	2379	6	0.5	Exon(exon45of191)	26014	TTN	7273
354287	2q	184936178	184937636	1459	6	0.333	Exon(exon4of4)	69813	ZNF804A	91752
354287	2q	185789865	185794632	4768	10	0.8	Promoter(<=1kb)	0	FSIP2	401024
354287	2q	185805377	185808170	2794	6	0.333	Promoter(<=1kb)	0	FSIP2	401024
354287	2q	217847583	217848746	1164	7	0.857	Exon(exon19of33)	-5423	TNS1	7145
354287	2q	219539659	219540599	941	6	0.667	Promoter(2-3kb)	2498	CHPF	79586
354287	2q	238130271	238131546	1276	7	0.286	Promoter(1-2kb)	1323	ESPNL	339768
354287	3p	31989532	31990905	1374	7	0.286	Exon(exon2of2)	7761	ZNF860	344787
354287	3p	75737070	75739243	2174	54	0.63	Promoter(<=1kb)	0	MIR4273	100422955
354287	3q	194341097	194342571	1475	7	0.714	Exon(exon2of2)	8747	CPN2	1370
354287	4p	5988383	5989749	1367	7	0.571	Promoter(<=1kb)	0	C4orf50	389197
354287	4p	6300792	6302360	1569	7	0.857	Exon(exon8of8)	6021	WFS1	7466
354287	4p	8227004	8228508	1505	8	0.125	Promoter(<=1kb)	-24	SH3TC1	54436
354287	4q	121036404	121037542	1139	6	0.333	Promoter(1-2kb)	1442	NDNF	79625
354287	4q	185458217	185460011	1795	8	0.625	Promoter(<=1kb)	0	CCDC110	256309
354287	5q	79728956	79730716	1761	7	0.286	Exon(exon2of13)	-7426	CMYA5	202333
354287	5q	79731782	79734523	2742	13	0.308	Exon(exon2of13)	-3619	CMYA5	202333
354287	5q	83537326	83539905	2580	6	0.333	Promoter(1-2kb)	1712	VCAN	1462
354287	5q	112891825	112893068	1244	6	0.5	3'UTR	27216	SRP19	6728
354287	5q	140848579	140850786	2208	8	0.5	Promoter(<=1kb)	807	PCDHA9	9752
354287	5q	141122595	141123655	1061	6	0.833	Promoter(<=1kb)	777	PCDHB4	56131
354287	5q	141150948	141152584	1637	6	0.833	Promoter(<=1kb)	846	PCDHB6	56130
354287	5q	141172977	141175025	2049	11	0.818	Promoter(<=1kb)	333	PCDHB7	56129
354287	5q	141178745	141180333	1589	9	0.778	Promoter(<=1kb)	955	PCDHB8	56128
354287	5q	141183598	141184688	1091	10	0.9	Promoter(2-3kb)	2199	PCDHB16	57717
354287	5q	141187690	141189425	1736	8	0.25	Promoter(<=1kb)	529	PCDHB9	56127
354287	5q	141246961	141247287	327	6	0.667	Promoter(1-2kb)	1566	PCDHB15	56121
354287	5q	148826877	148828070	1194	6	1	Promoter(1-2kb)	1632	ADRB2	154
354287	5q	151521550	151522069	520	6	0.667	Promoter(<=1kb)	79	MIR6499	102465246
354287	5q	151565922	151568158	2237	9	0.778	Promoter(<=1kb)	786	FAT2	2196
354287	6p	16327099	16327837	739	6	0.333	Exon(exon8of9)	37154	GMPR	2766
354287	6p	26370344	26370520	177	7	0.571	Promoter(<=1kb)	0	BTN3A2	11118
354287	6p	46858771	46859389	619	6	0.5	Exon(exon17of21)	3915	ADGRF5	221395
354287	6q	159233455	159234370	916	10	0.5	Exon(exon11of23)	15158	FNDC1	84624
354287	7p	38353718	38353991	274	8	0.625	Exon(exon2of2)	3699	TRG-AS1	100506776
354287	7p	45082865	45084866	2002	7	0.429	Promoter(1-2kb)	-1465	NACAD	23148
354287	7p	56021087	56021209	123	6	0.5	Exon(exon2of7)	12947	PSPH	5723
354287	7q	64991278	64992758	1481	7	0.571	Promoter(<=1kb)	-242	ZNF117	51351
354287	7q	100958977	100960873	1897	56	0.446	Promoter(1-2kb)	1012	MUC3A	4584
354287	7q	100991195	100993127	1933	8	0.625	Exon(exon5of15)	-19927	MUC12	10071
354287	7q	100995575	100995785	211	6	0.833	Exon(exon5of15)	-17269	MUC12	10071
354287	8p	10607375	10608261	887	6	0.5	Exon(exon4of4)	46882	RP1L1	94137
354287	8p	11331194	11332082	889	9	0.556	Promoter(<=1kb)	306	SLC35G5	83650
354287	8p	12137448	12138641	1194	6	0.833	Promoter(<=1kb)	436	USP17L2	377630

354287	8p	13021128	13022030	903	8	0.25	Exon(exon5of5)	9115	TRMT9B	57604
354287	8q	143869993	143872650	2658	8	0.625	Exon(exon2of2)	5814	EPPK1	83481
354287	8q	143916360	143919209	2850	7	0.143	Exon(exon32of32)	20381	PLEC	5339
354287	9p	21206764	21207074	311	6	0.5	Promoter(<=1kb)	69	IFNA10	3446
354287	9q	76175237	76175296	60	10	0.8	Exon(exon14of14)	-13343	PCSK5	5125
354287	9q	76705179	76707804	2626	8	0.5	Promoter(<=1kb)	121	PCA3	50652
354287	9q	76709263	76710846	1584	9	0.556	Promoter(<=1kb)	0	PRUNE2	158471
354287	9q	87887543	87888819	1277	6	0.5	Exon(exon4of4)	4666	SPATA31E1	286234
354287	9q	104598545	104599361	817	12	0.583	Promoter(<=1kb)	52	OR13C5	138799
354287	9q	122553263	122554071	809	8	0.5	Promoter(<=1kb)	93	OR1N2	138882
354287	9q	122749914	122750547	634	6	0.833	Promoter(<=1kb)	174	OR1L6	392390
354287	9q	124855684	124856809	1126	7	0.571	Promoter(2-3kb)	2208	WDR38	401551
354287	9q	133255626	133256205	580	8	0.875	3'UTR	19009	ABO	28
354287	9q	135484803	135487213	2411	9	0.333	Promoter(1-2kb)	1440	PPP1R26	9858
354287	10p	47663	48605	943	7	0.857	Promoter(<=1kb)	664	TUBB8	347688
354287	10q	46549378	46550723	1346	26	0.654	Exon(exon3of3)	4807	GPRIN2	9721
354287	10q	122084988	122087840	2853	8	0.75	Exon(exon4of23)	-25190	TACC2	10579
354287	11p	244106	244197	92	8	0.5	Promoter(<=1kb)	-232	PSMD13	5719
354287	11p	1246332	1247378	1047	6	0.5	Promoter(2-3kb)	2298	MUC5B-AS1	112577518
354287	11p	5046754	5047432	679	7	0.571	Promoter(<=1kb)	228	OR52J3	119679
354287	11p	5177978	5178478	501	6	0.167	Promoter(<=1kb)	186	OR52Z1	283110
354287	11p	5323362	5324256	895	7	0.429	Promoter(<=1kb)	41	OR51B2	79345
354287	11p	5389704	5390350	647	7	0.429	Promoter(<=1kb)	327	OR51M1	390059
354287	11p	5422212	5423123	912	11	0.636	Promoter(<=1kb)	101	OR51Q1	390061
354287	11p	5581045	5581738	694	8	0.375	Promoter(<=1kb)	168	OR52B6	340980
354287	11p	5841302	5841883	582	9	0.333	Promoter(<=1kb)	14	OR52E6	390078
354287	11p	5884818	5885061	244	7	0.429	Promoter(<=1kb)	547	OR52E4	390081
354287	11p	11351961	11352736	776	8	0.25	Promoter(<=1kb)	514	CSNK2A3	283106
354287	11p	12293639	12294368	730	6	0.833	Exon(exon29of35)	6739	MICALCL	84953
354287	11q	54603136	54603820	685	6	0.667	Promoter(<=1kb)	178	OR4C46	119749
354287	11q	58214757	58215722	966	8	0.25	Promoter(<=1kb)	12	OR1S1	219959
354287	11q	85724687	85725825	1139	6	0.5	Promoter(<=1kb)	0	SYTL2	54843
354287	11q	124015601	124016477	877	9	0.222	Promoter(<=1kb)	26	OR10G4	390264
354287	11q	124023038	124023849	812	9	0.444	Promoter(<=1kb)	25	OR10G9	219870
354287	11q	124038392	124038988	597	6	0.833	Promoter(<=1kb)	13	OR10G7	390265
354287	12p	4626571	4628549	1979	8	0.5	Exon(exon5of6)	14054	DYRK4	8798
354287	12p	6453119	6453670	552	6	0.667	Promoter(<=1kb)	633	TAPBPL	55080
354287	12p	8222174	8223514	1341	6	0.667	Exon(exon5of6)	4073	FAM90A1	55138
354287	12q	52571389	52573652	2264	7	0.571	Promoter(<=1kb)	164	KRT74	121391
354287	13q	25096659	25097182	524	12	0.417	Promoter(<=1kb)	791	PABPC3	5042
354287	13q	102732474	102733933	1460	6	0.333	Exon(exon4of4)	25139	CCDC168	643677
354287	14q	20060048	20060884	837	8	0.625	Promoter(<=1kb)	3	OR4L1	122742
354287	14q	20143999	20144604	606	8	0.5	Promoter(<=1kb)	263	OR4N5	390437
354287	14q	20640982	20641567	586	6	0.5	Promoter(<=1kb)	124	OR6S1	341799
354287	14q	63599655	63599684	30	6	0.667	Exon(exon2of2)	41723	WDR89	112840
354287	14q	70457520	70458540	1021	12	0.333	Exon(exon2of2)	5346	ADAM21	8747
354287	14q	104175275	104177810	2536	10	0.3	Exon(exon12of15)	36235	KIF26A	26153
354287	14q	104939262	104942618	3357	10	0.2	5'UTR	7102	PLD4	122618
354287	14q	104943622	104945444	1823	7	0.571	Exon(exon6of6)	9958	AHNAK2	113146
354287	14q	104947901	104953878	5978	32	0.5	Promoter(1-2kb)	1524	AHNAK2	113146
354287	15q	23439979	23442067	2089	9	0.667	5'UTR	5167	GOLGA6L2	283685
354287	15q	78765997	78766626	630	7	0.429	Promoter(<=1kb)	-884	ADAMTS7	11173
354287	15q	85579423	85582073	2651	15	0.6	Promoter(<=1kb)	-837	AKAP13	11214
354287	15q	88857108	88859365	2258	6	0	Exon(exon12of18)	9865	ACAN	176
354287	15q	99129423	99132517	3095	7	0.429	Exon(exon4of5)	7225	TTC23	64927
354287	16p	1486371	1488463	2093	8	0.75	Promoter(<=1kb)	4	PTX4	390667
354287	16q	74391416	74391928	513	8	0.75	Exon(exon7of7)	13538	NPIPB15	440348
354287	16q	89100686	89101050	365	8	0.625	Promoter(<=1kb)	24	ACSF3	197322
354287	17p	744946	746966	2021	6	1	3'UTR	5072	GEMIN4	50628
354287	17p	10638198	10641099	2902	7	0.286	Exon(exon19of41)	-8169	MYH3	4621
354287	17p	21300581	21300978	398	12	0.75	3'UTR	9112	MAP2K3	5606
354287	17p	21415470	21416370	901	9	0.778	Exon(exon3of3)	10334	KCNJ12	3768
354287	17q	76293419	76294016	598	6	0.5	Promoter(2-3kb)	-2167	QRICH2	84074
354287	17q	81645135	81645417	283	6	0.333	Promoter(2-3kb)	2722	TSPAN10	83882
354287	18p	11609904	11610469	566	6	0.667	Promoter(<=1kb)	308	SLC35G4	646000
354287	18q	58535186	58537515	2330	10	0.4	Promoter(<=1kb)	0	ALPK2	115701
354287	19p	8948231	8952171	3941	11	0.455	Exon(exon3of84)	29171	MUC16	94025
354287	19p	8959403	8961248	1846	6	0.667	Exon(exon3of84)	20094	MUC16	94025
354287	19p	12075333	12077046	1714	6	0.167	Promoter(<=1kb)	6	ZNF788P	388507
354287	19p	15087213	15088040	828	11	0.273	Promoter(<=1kb)	233	OR1I1	126370
354287	19p	18264798	18267409	2612	12	0.583	5'UTR	7002	IQCN	80726
354287	19p	21971930	21974500	2571	10	0.7	Exon(exon4of4)	14408	ZNF208	7757
354287	19p	22756294	22759533	3240	9	0.667	3'UTR	10449	ZNF99	7652
354287	19q	36996730	36997597	868	10	0.6	Exon(exon10of10)	5677	ZNF568	374900
354287	19q	39877222	39877880	659	6	0.5	Exon(exon20of28)	9412	FCGBP	8857
354287	19q	39886240	39886422	183	6	0.667	Promoter(<=1kb)	870	FCGBP	8857
354287	19q	43913423	43914878	1456	9	0.556	Exon(exon10of10)	4861	ZNF45	7596

354287	19q	43966037	43967171	1135	6	0.167	Promoter(<=1kb)	-691	ZNF155	7711
354287	19q	43996326	43997366	1041	6	0.5	Exon(exon5of5)	5419	LOC10192806:	101928063
354287	19q	44106512	44108078	1567	8	0.125	Exon(exon6of6)	-4103	ZNF225	7768
354287	19q	52437918	52439242	1325	7	0.429	Exon(exon4of4)	6504	ZNF534	147658
354287	19q	55517821	55518189	369	9	1	Exon(exon14of14)	18114	SBK2	646643
354287	19q	55911888	55913077	1190	6	0.667	Exon(exon5of12)	19234	NLRP13	126204
354287	19q	57639918	57641641	1724	7	0.429	3'UTR	6693	ZNF211	10520
354287	19q	58368293	58368875	583	7	0.429	Exon(exon3of3)	-5445	ZNF497	162968
354287	20q	63559968	63565531	5564	15	0.667	Promoter(<=1kb)	0	HELZ2	85441
354287	21q	44550835	44551416	582	7	0.857	Promoter(<=1kb)	89	KRTAP10-2	386679
354287	22q	22352950	22353365	416	15	0.533	Exon(exon1of2)	30478	BMS1P20	96610
354287	22q	36191154	36191906	753	6	0.667	3'UTR	9971	APOL4	80832
354287	22q	36265284	36265796	513	6	1	Exon(exon6of6)	12120	APOL1	8542
354287	23p	35802148	35803010	863	7	0.571	5'UTR	3357	MAGEB16	139604
354287	23q	141906066	141906494	429	8	0.5	Promoter(1-2kb)	1264	MAGEC1	9947
355614	1p	12893204	12893425	222	6	0.167	Exon(exon4of4)	4845	PRAMEF10	343071
355614	1p	16048038	16049824	1787	6	0.5	Promoter(<=1kb)	0	CLCNKB	1188
355614	1p	16058491	16060000	1510	10	0.9	Exon(exon5of7)	6168	CLCNKB	1188
355614	1p	18481042	18482217	1176	12	0.75	Promoter(<=1kb)	60	KLHDC7A	127707
355614	1p	23874604	23875430	827	8	0.5	Exon(exon2of2)	-6310	FUCA1	2517
355614	1p	40067594	40067675	82	6	0	Promoter(<=1kb)	324	CAP1	10487
355614	1q	152213274	152213320	47	8	0.5	Exon(exon3of3)	10873	HRNR	388697
355614	1q	152218469	152221375	2907	14	0.643	Promoter(2-3kb)	2818	HRNR	388697
355614	1q	152303673	152313891	10219	37	0.595	Promoter(<=1kb)	0	FLG-AS1	339400
355614	1q	156669844	156670886	1043	6	1	Exon(exon4of4)	6521	NES	10763
355614	1q	158398525	158399274	750	6	0.333	Promoter(<=1kb)	192	OR10T2	128360
355614	1q	158765805	158766655	851	6	0.5	Promoter(<=1kb)	47	OR6N1	128372
355614	1q	228315976	228318026	2051	6	0.5	Exon(exon50of81)	6492	OBSCN	84033
355614	1q	247841312	247841582	271	6	0.833	Promoter(<=1kb)	314	OR11L1	391189
355614	1q	247895796	247896502	707	7	0.571	Promoter(<=1kb)	209	OR2W3	343171
355614	1q	247949443	247949738	296	10	0.3	Promoter(<=1kb)	585	OR2L8	391190
355614	1q	248573992	248574210	219	6	0.833	Promoter(<=1kb)	547	OR2T34	127068
355614	1q	248681658	248682198	541	7	0.571	Promoter(<=1kb)	130	OR14I1	401994
355614	2p	48580657	48582454	1798	7	0.571	Promoter(<=1kb)	0	STON1	11037
355614	2q	132783534	132785001	1468	7	0.429	Promoter(1-2kb)	-1511	NCKAP5	344148
355614	2q	167246794	167248478	1685	7	0.714	Promoter(<=1kb)	-204	XIRP2	129446
355614	2q	178739433	178741811	2379	6	0.5	Exon(exon45of191)	26014	TTN	7273
355614	2q	217847583	217848559	977	6	0.833	Exon(exon19of33)	-5423	TNS1	7145
355614	2q	238130271	238131546	1276	7	0.286	Promoter(1-2kb)	1323	ESPNL	339768
355614	3p	75737230	75739007	1778	9	0.556	Promoter(<=1kb)	0	MIR4273	100422955
355614	3q	98264413	98265098	686	7	0.571	Promoter(<=1kb)	128	OR5H6	79295
355614	3q	194359607	194360906	1300	9	0.889	Exon(exon2of2)	-8279	CPN2	1370
355614	4p	5988383	5989749	1367	7	0.571	Promoter(<=1kb)	0	C4orf50	389197
355614	4p	6300792	6302360	1569	8	0.875	Exon(exon8of8)	6021	WFS1	7466
355614	4p	8227004	8228508	1505	8	0.125	Promoter(<=1kb)	-24	SH3TC1	54436
355614	4p	10443803	10446224	2422	7	0.429	Exon(exon3of3)	10952	ZNF518B	85460
355614	4q	154489498	154491312	1815	9	0.556	Promoter(<=1kb)	22	DCHS2	54798
355614	4q	185458217	185460011	1795	8	0.625	Promoter(<=1kb)	0	CCDC110	256309
355614	4q	186617176	186621582	4407	11	0.455	Exon(exon10of27)	-7106	FAT1	2195
355614	4q	186706638	186709436	2799	7	0.714	Exon(exon2of27)	14397	FAT1	2195
355614	5p	795818	796237	420	6	0.667	3'UTR	4908	ZDHHC11	79844
355614	5q	79728956	79734523	5568	21	0.333	Exon(exon2of13)	-3619	CMYA5	202333
355614	5q	83537326	83539905	2580	6	0.333	Promoter(1-2kb)	1712	VCAN	1462
355614	5q	140848579	140850786	2208	6	0.5	Promoter(<=1kb)	807	PCDHA9	9752
355614	5q	141174000	141175025	1026	6	0.833	Promoter(1-2kb)	1356	PCDHB7	56129
355614	6p	16327099	16327837	739	6	0.333	Exon(exon8of9)	37154	GMPR	2766
355614	6p	46858771	46859502	732	7	0.571	Exon(exon17of21)	3802	ADGRF5	221395
355614	6q	159231899	159234370	2472	12	0.583	Exon(exon11of23)	13602	FNDC1	84624
355614	7p	6330446	6330944	499	6	1	Exon(exon2of2)	7749	FAM220A	84792
355614	7p	12369637	12370736	1100	6	0.667	3'UTR	-13307	VWDE	221806
355614	7p	28956184	28957525	1342	6	0.833	Promoter(<=1kb)	805	TRIL	9865
355614	7p	56021087	56021209	123	6	0.5	Exon(exon2of7)	12947	PSPH	5723
355614	7q	64991321	64992758	1438	7	0.714	Promoter(<=1kb)	-285	ZNF117	51351
355614	7q	100958721	100960873	2153	58	0.431	Promoter(<=1kb)	756	MUC3A	4584
355614	7q	100991195	100993102	1908	8	0.625	Exon(exon5of15)	-19952	MUC12	10071
355614	7q	100995575	100995785	211	7	0.714	Exon(exon5of15)	-17269	MUC12	10071
355614	7q	101004258	101004836	579	7	0.857	Exon(exon5of15)	-8218	MUC12	10071
355614	7q	101034361	101040583	6223	38	0.5	Exon(exon3of12)	-3128	MUC17	140453
355614	8p	8376561	8377994	1434	6	1	Exon(exon2of5)	3753	PRAG1	157285
355614	8p	10607375	10608261	887	6	0.5	Exon(exon4of4)	46882	RP1L1	94137
355614	8p	13021128	13022030	903	8	0.25	Exon(exon5of5)	9115	TRMT9B	57604
355614	8q	123651655	123652634	980	7	0.571	Promoter(<=1kb)	316	KLHL38	340359
355614	8q	138151505	138153046	1542	6	0.667	Promoter(<=1kb)	0	FAM135B	51059
355614	8q	143916360	143919209	2850	7	0.143	Exon(exon32of32)	20381	PLEC	5339
355614	8q	143923488	143925516	2029	8	0.625	Exon(exon31of32)	14074	PLEC	5339
355614	8q	144415811	144416618	808	6	0.667	Promoter(<=1kb)	0	SLC39A4	55630
355614	9p	116832	118032	1201	6	0.667	Promoter(<=1kb)	172	FOXD4	2298

355614	9p	39078723	39078846	124	6	0.667	Exon(exon22of24)	7302	CNTNAP3	79937
355614	9q	87886533	87888398	1866	6	0.667	Exon(exon4of4)	3656	SPATA31E1	286234
355614	9q	104598545	104599361	817	12	0.583	Promoter(<=1kb)	52	OR13C5	138799
355614	9q	104694482	104695444	963	6	0.5	Promoter(<=1kb)	0	OR13D1	286365
355614	9q	122553278	122554071	794	6	0.333	Promoter(<=1kb)	108	OR1N2	138882
355614	9q	122628595	122629398	804	7	0.286	Promoter(<=1kb)	175	OR1B1	347169
355614	9q	122749914	122750547	634	6	0.833	Promoter(<=1kb)	174	OR1L6	392390
355614	9q	133255635	133256205	571	7	1	3'UTR	19009	ABO	28
355614	9q	135484803	135487213	2411	8	0.25	Promoter(1-2kb)	1440	PPP1R26	9858
355614	10q	46549378	46550723	1346	25	0.64	Exon(exon3of3)	4807	GPRIN2	9721
355614	10q	89737450	89738561	1112	6	0	Exon(exon20of33)	13874	KIF20B	9585
355614	10q	122084988	122087840	2853	8	0.75	Exon(exon4of23)	-25190	TACC2	10579
355614	11p	244106	244197	92	8	0.5	Promoter(<=1kb)	-232	PSMD13	5719
355614	11p	1246095	1247378	1284	8	0.375	Promoter(2-3kb)	2298	MUC5B-AS1	112577518
355614	11p	1248397	1251628	3232	9	0.778	Promoter(<=1kb)	0	MUC5B-AS1	112577518
355614	11p	5177978	5178478	501	6	0.167	Promoter(<=1kb)	186	OR52Z1	283110
355614	11p	5323362	5324256	895	7	0.429	Promoter(<=1kb)	41	OR51B2	79345
355614	11p	5389704	5390350	647	7	0.429	Promoter(<=1kb)	327	OR51M1	390059
355614	11p	5402638	5403471	834	12	0.417	Promoter(<=1kb)	41	OR51J1	79470
355614	11p	5422212	5423123	912	10	0.7	Promoter(<=1kb)	101	OR51Q1	390061
355614	11p	5515185	5515931	747	6	0.333	Promoter(<=1kb)	768	UBQLNL	143630
355614	11p	5581045	5581738	694	8	0.375	Promoter(<=1kb)	168	OR52B6	340980
355614	11p	5841302	5841883	582	9	0.333	Promoter(<=1kb)	14	OR52E6	390078
355614	11p	5884818	5885061	244	7	0.429	Promoter(<=1kb)	547	OR52E4	390081
355614	11p	11351961	11352736	776	7	0.143	Promoter(<=1kb)	514	CSNK2A3	283106
355614	11p	12293639	12294538	900	8	0.625	Exon(exon29of35)	6739	MICALCL	84953
355614	11p	34916266	34916763	498	6	0.667	Promoter(<=1kb)	0	APIP	51074
355614	11q	55827536	55827640	105	6	0.667	Promoter(<=1kb)	317	OR5L2	26338
355614	11q	58214757	58215722	966	8	0.25	Promoter(<=1kb)	12	OR1S1	219959
355614	11q	85724687	85725825	1139	6	0.5	Promoter(<=1kb)	0	SYTL2	54843
355614	11q	123906790	123907324	535	6	0.667	Promoter(<=1kb)	644	OR8D4	338662
355614	11q	124038366	124038988	623	7	1	Promoter(<=1kb)	13	OR10G7	390265
355614	11q	124382526	124383285	760	8	0.625	Promoter(<=1kb)	58	OR8B2	26595
355614	12p	4626568	4628549	1982	11	0.455	Exon(exon5of6)	14051	DYRK4	8798
355614	12p	6453119	6453670	552	6	0.667	Promoter(<=1kb)	633	TAPBPL	55080
355614	12p	31981130	31983004	1875	8	0.25	Exon(exon4of6)	-4162	RESF1	55196
355614	12q	52316096	52317765	1670	6	0.667	Exon(exon4of9)	3633	KRT83	3889
355614	13q	24434450	24435347	898	7	0.571	Exon(exon31of34)	19787	PARP4	143
355614	13q	25096730	25097003	274	9	0.444	Promoter(<=1kb)	862	PABPC3	5042
355614	13q	102732474	102733933	1460	6	0.333	Exon(exon4of4)	25139	CCDC168	643677
355614	14q	20060048	20060884	837	8	0.625	Promoter(<=1kb)	3	OR4L1	122742
355614	14q	20640750	20641620	871	8	0.625	Promoter(<=1kb)	71	OR6S1	341799
355614	14q	21634137	21634589	453	9	0.556	Promoter(<=1kb)	351	OR10G2	26534
355614	14q	44505064	44506403	1340	6	0.667	Promoter(<=1kb)	880	FSCB	84075
355614	14q	70457520	70458540	1021	7	0.429	Exon(exon2of2)	5346	ADAM21	8747
355614	14q	104175275	104177810	2536	10	0.3	Exon(exon12of15)	36235	KIF26A	26153
355614	14q	104939262	104945444	6183	20	0.4	5'UTR	7102	PLD4	122618
355614	14q	104946867	104951938	5072	24	0.583	Exon(exon6of6)	3464	AHNAK2	113146
355614	15q	20534501	20535056	556	8	0.5	Exon(exon8of9)	6744	GOLGA6L6	727832
355614	15q	23439979	23442067	2089	12	0.5	5'UTR	5167	GOLGA6L2	283685
355614	15q	40621642	40623696	2055	7	0.429	Promoter(<=1kb)	0	KNL1	57082
355614	15q	73702465	73703760	1296	6	0.833	Promoter(<=1kb)	-149	CD276	80381
355614	15q	85579423	85582073	2651	16	0.625	Promoter(<=1kb)	-837	AKAP13	11214
355614	15q	99129423	99132517	3095	6	0.333	Exon(exon4of5)	7225	TTC23	64927
355614	16p	1256345	1256980	636	6	0.833	Promoter(<=1kb)	276	TPSD1	23430
355614	16q	74391650	74391928	279	6	0.667	Exon(exon7of7)	13772	NPIPB15	440348
355614	16q	88428539	88431889	3351	12	0.417	Exon(exon3of3)	-21391	ZFPM1	161882
355614	16q	88434626	88436097	1472	6	0.5	Exon(exon3of3)	-17183	ZFPM1	161882
355614	16q	88714632	88717113	2482	7	0.429	Promoter(<=1kb)	0	MIR4722	100616167
355614	16q	89100686	89101050	365	7	0.571	Promoter(<=1kb)	24	ACSF3	197322
355614	16q	89226863	89228419	1557	9	0.556	Promoter(2-3kb)	2229	ZNF778	197320
355614	17p	21300581	21300978	398	12	0.75	3'UTR	9112	MAP2K3	5606
355614	17p	21415470	21416499	1030	10	0.8	Exon(exon3of3)	10334	KCNJ12	3768
355614	17q	41586466	41586829	364	6	0.833	Promoter(<=1kb)	66	KRT14	3861
355614	17q	53823368	53824891	1524	6	0.833	Promoter(<=1kb)	441	KIF2B	84643
355614	17q	81645135	81645417	283	6	0.333	Promoter(2-3kb)	2722	TSPAN10	83882
355614	18p	11609646	11610444	799	13	0.769	Promoter(<=1kb)	50	SLC35G4	646000
355614	18p	14542649	14543140	492	6	0.5	Promoter(<=1kb)	6	POTEC	388468
355614	18q	47033473	47035259	1787	8	0.875	Promoter(<=1kb)	362	ELOA2	51224
355614	18q	58535186	58537515	2330	10	0.4	Promoter(<=1kb)	0	ALPK2	115701
355614	19p	4511338	4513547	2210	8	0.25	Exon(exon3of6)	4157	PLIN4	729359
355614	19p	5455600	5456439	840	7	0.571	Promoter(<=1kb)	183	ZNRF4	148066
355614	19p	8946313	8951868	5556	15	0.6	Exon(exon3of84)	29474	MUC16	94025
355614	19p	8959116	8962299	3184	10	0.7	Exon(exon3of84)	19043	MUC16	94025
355614	19p	8972751	8978096	5346	15	0.467	Exon(exon1of84)	3246	MUC16	94025
355614	19p	12430157	12432437	2281	9	0.333	3'UTR	8584	ZNF443	10224
355614	19p	21971930	21974500	2571	10	0.7	Exon(exon4of4)	14408	ZNF208	7757

355614	19p	22756294	22759533	3240	11	0.545	3'UTR	10449	ZNF99	7652
355614	19p	23743906	23745300	1395	6	0.333	Exon(exon4of4)	13537	ZNF681	148213
355614	19q	39886240	39886422	183	6	0.667	Promoter(<=1kb)	870	FCGBP	8857
355614	19q	43913423	43914878	1456	8	0.5	Exon(exon10of10)	4861	ZNF45	7596
355614	19q	43966037	43967171	1135	6	0.167	Promoter(<=1kb)	-691	ZNF155	7711
355614	19q	44106512	44108078	1567	7	0	Exon(exon6of6)	-4103	ZNF225	7768
355614	19q	51745958	51746963	1006	6	0.333	Exon(exon3of3)	3848	FPR1	2357
355614	19q	52437918	52439242	1325	7	0.429	Exon(exon4of4)	6504	ZNF534	147658
355614	19q	52840798	52842068	1271	8	0.375	3'UTR	7457	ZNF468	90333
355614	19q	55911888	55913077	1190	6	0.667	Exon(exon5of12)	19234	NLRP13	126204
355614	20p	5922421	5923394	974	7	0.571	Exon(exon4of5)	6923	CHGB	1114
355614	20q	63349752	63350772	1021	6	0.5	3'UTR	3794	CHRNA4	1137
355614	20q	63561666	63565531	3866	12	0.667	Promoter(<=1kb)	-61	HELZ2	85441
355614	21q	44637474	44638041	568	9	0.333	Promoter(<=1kb)	118	KRTAP10-10	353333
355614	22q	22352950	22353380	431	16	0.5	Exon(exon1of2)	30478	BMS1P20	96610
355614	22q	36191154	36191906	753	6	0.667	3'UTR	9971	APOL4	80832
355614	23p	8170039	8170141	103	6	0.5	Promoter(1-2kb)	1126	VCX2	51480
355614	23p	35802148	35803010	863	7	0.571	5'UTR	3357	MAGEB16	139604
355614	23q	102937373	102937765	393	7	0.571	Promoter(<=1kb)	101	RAB40AL	282808
355614	23q	141905856	141907529	1674	13	0.615	Promoter(1-2kb)	1054	MAGEC1	9947
355903	1p	16058491	16060000	1510	10	0.9	Exon(exon5of7)	6168	CLCNKB	1188
355903	1p	40067594	40067675	82	6	0	Promoter(<=1kb)	324	CAP1	10487
355903	1q	152218469	152221375	2907	14	0.643	Promoter(2-3kb)	2818	HRNR	388697
355903	1q	152302822	152313891	11070	37	0.568	Promoter(<=1kb)	0	FLG-AS1	339400
355903	1q	156669844	156670886	1043	6	1	Exon(exon4of4)	6521	NES	10763
355903	1q	158765805	158766655	851	6	0.5	Promoter(<=1kb)	47	OR6N1	128372
355903	1q	169540901	169542882	1982	10	0.3	Exon(exon13of25)	-25156	F5	2153
355903	1q	201206099	201209342	3244	10	0.6	Promoter(1-2kb)	1017	IGFN1	91156
355903	1q	202318028	202319165	1138	6	0.667	3'UTR	22771	UBE2T	29089
355903	1q	214640144	214642954	2811	12	0.5	Exon(exon12of20)	-5013	CENPF	1063
355903	1q	214644872	214647181	2310	10	0.4	Promoter(<=1kb)	-786	CENPF	1063
355903	1q	226735683	226737239	1557	7	0.857	Promoter(<=1kb)	219	ITPKB	3707
355903	1q	228315976	228318049	2074	8	0.625	Exon(exon50of81)	6492	OBSCN	84033
355903	1q	232805117	232806800	1684	7	0.429	Promoter(<=1kb)	225	MAP10	54627
355903	1q	247841312	247841582	271	6	0.833	Promoter(<=1kb)	314	OR11L1	391189
355903	1q	247949325	247949738	414	11	0.273	Promoter(<=1kb)	467	OR2L8	391190
355903	2p	48580657	48582454	1798	7	0.571	Promoter(<=1kb)	0	STON1	11037
355903	2q	132781078	132784972	3895	10	0.5	Promoter(<=1kb)	0	NCKAP5	344148
355903	2q	185789865	185794632	4768	10	0.8	Promoter(<=1kb)	0	FSIP2	401024
355903	2q	217847567	217848746	1180	7	0.857	Exon(exon19of33)	-5407	TNS1	7145
355903	2q	233713134	233713783	650	13	0.692	Promoter(<=1kb)	142	UGT1A5	54579
355903	2q	233840612	233842185	1574	6	0.667	Promoter(<=1kb)	0	HJURP	55355
355903	2q	237762685	237764060	1376	10	0.5	Exon(exon8of8)	-4137	LRRFIP1	9208
355903	2q	240041845	240042811	967	8	0.375	Downstream(2-3kb)	3261	OR6B3	150681
355903	3p	75737051	75739243	2193	60	0.633	Promoter(<=1kb)	0	MIR4273	100422955
355903	3q	98264413	98265098	686	7	0.571	Promoter(<=1kb)	128	OR5H6	79295
355903	3q	194341177	194342571	1395	6	0.667	Exon(exon2of2)	8747	CPN2	1370
355903	3q	194359607	194361038	1432	11	0.818	Exon(exon2of2)	-8279	CPN2	1370
355903	4p	1394625	1395373	749	8	0.25	Exon(exon1of1)	9813	UVSSA	57654
355903	4p	5988383	5989749	1367	8	0.625	Promoter(<=1kb)	0	C4orf50	389197
355903	4p	6300792	6302360	1569	6	0.833	Exon(exon8of8)	6021	WFS1	7466
355903	4q	154489498	154491312	1815	9	0.556	Promoter(<=1kb)	22	DCHS2	54798
355903	4q	186706638	186708616	1979	6	0.667	Exon(exon2of27)	15217	FAT1	2195
355903	5q	79728956	79730716	1761	7	0.286	Exon(exon2of13)	-7426	CMYA5	202333
355903	5q	79731782	79734523	2742	13	0.308	Exon(exon2of13)	-3619	CMYA5	202333
355903	5q	83537326	83539905	2580	6	0.333	Promoter(1-2kb)	1712	VCAN	1462
355903	5q	140807352	140807737	386	6	0.833	Promoter(<=1kb)	271	PCDHA4	56144
355903	5q	141174000	141175025	1026	6	0.833	Promoter(1-2kb)	1356	PCDHB7	56129
355903	5q	151521550	151522069	520	6	0.667	Promoter(<=1kb)	79	MIR6499	102465246
355903	5q	151565922	151568158	2237	9	0.778	Promoter(<=1kb)	786	FAT2	2196
355903	6p	26370344	26370479	136	6	0.5	Promoter(<=1kb)	0	BTN3A2	11118
355903	6p	46858771	46859502	732	8	0.5	Exon(exon17of21)	3802	ADGRF5	221395
355903	6q	64591274	64591961	688	10	0.5	Exon(exon26of43)	121374	EYS	346007
355903	6q	149888581	149889987	1407	8	0.75	Promoter(<=1kb)	-116	RAET1E-AS1	100652739
355903	6q	159231899	159234370	2472	13	0.615	Exon(exon11of23)	13602	FNDC1	84624
355903	7p	38353718	38353991	274	8	0.625	Exon(exon2of2)	3699	TRG-AS1	100506776
355903	7p	45082865	45084866	2002	6	0.333	Promoter(1-2kb)	-1465	NACAD	23148
355903	7q	64991278	64992758	1481	6	0.667	Promoter(<=1kb)	-242	ZNF117	51351
355903	7q	100958977	100960873	1897	50	0.46	Promoter(1-2kb)	1012	MUC3A	4584
355903	7q	100991195	100992398	1204	7	0.571	Exon(exon5of15)	-20656	MUC12	10071
355903	7q	100995575	100996039	465	8	0.75	Exon(exon5of15)	-17015	MUC12	10071
355903	7q	101004538	101004836	299	6	0.833	Exon(exon5of15)	-8218	MUC12	10071
355903	7q	101034361	101040583	6223	32	0.469	Exon(exon3of12)	-3128	MUC17	140453
355903	8p	10609650	10612307	2658	8	0.875	Exon(exon4of4)	42836	RP1L1	94137
355903	8p	11331234	11332082	849	6	0.667	Promoter(<=1kb)	346	SLC35G5	83650
355903	8p	13021128	13022030	903	8	0.125	Exon(exon5of5)	9115	TRMT9B	57604
355903	8q	123651655	123652634	980	6	0.5	Promoter(<=1kb)	316	KLHL38	340359

355903	9p	39078723	39078846	124	6	0.667	Exon(exon22of24)	7302	CNTNAP3	79937
355903	9q	76175237	76175296	60	9	0.778	Exon(exon14of14)	-13343	PCSK5	5125
355903	9q	76703555	76707804	4250	13	0.538	Promoter(<=1kb)	0	PCA3	50652
355903	9q	76709830	76710843	1014	6	0.667	Promoter(<=1kb)	0	PRUNE2	158471
355903	9q	104598641	104599361	721	8	0.625	Promoter(<=1kb)	52	OR13C5	138799
355903	9q	122553263	122554071	809	8	0.5	Promoter(<=1kb)	93	OR1N2	138882
355903	9q	122628595	122629398	804	7	0.286	Promoter(<=1kb)	175	OR1B1	347169
355903	9q	122749914	122750547	634	6	0.833	Promoter(<=1kb)	174	OR1L6	392390
355903	10p	47663	48605	943	7	0.857	Promoter(<=1kb)	664	TUBB8	347688
355903	10q	46549378	46550723	1346	26	0.654	Exon(exon3of3)	4807	GPRIN2	9721
355903	10q	89737450	89738561	1112	6	0	Exon(exon20of33)	13874	KIF20B	9585
355903	10q	128103129	128104830	1702	10	0.6	Promoter(<=1kb)	-1	MKI67	4288
355903	11p	244106	244197	92	8	0.5	Promoter(<=1kb)	-232	PSMD13	5719
355903	11p	1194354	1196902	2549	7	0.571	Exon(exon34of49)	-26164	MUC5B	727897
355903	11p	1241677	1243593	1917	6	0.333	Exon(exon31of49)	6083	MUC5B-AS1	112577518
355903	11p	1244757	1248605	3849	17	0.706	Promoter(1-2kb)	1071	MUC5B-AS1	112577518
355903	11p	1250091	1251628	1538	9	0.778	Promoter(<=1kb)	-415	MUC5B-AS1	112577518
355903	11p	5046754	5047432	679	7	0.571	Promoter(<=1kb)	228	OR52J3	119679
355903	11p	5177978	5178478	501	6	0.167	Promoter(<=1kb)	186	OR52Z1	283110
355903	11p	5389704	5390350	647	7	0.429	Promoter(<=1kb)	327	OR51M1	390059
355903	11p	5402638	5403322	685	10	0.5	Promoter(<=1kb)	41	OR51J1	79470
355903	11p	5422212	5423123	912	10	0.7	Promoter(<=1kb)	101	OR51Q1	390061
355903	11p	5581045	5581738	694	8	0.375	Promoter(<=1kb)	168	OR52B6	340980
355903	11p	5841302	5841883	582	9	0.333	Promoter(<=1kb)	14	OR52E6	390078
355903	11p	11351961	11352736	776	9	0.222	Promoter(<=1kb)	514	CSNK2A3	283106
355903	11p	12293639	12294842	1204	7	0.714	Exon(exon29of35)	6739	MICALCL	84953
355903	11p	43942293	43943348	1056	9	0.778	Promoter(<=1kb)	0	C11orf96	387763
355903	11q	55572176	55572903	728	6	0.667	Promoter(<=1kb)	48	OR4C16	219428
355903	11q	58402523	58403265	743	8	0.5	Promoter(<=1kb)	144	OR5B3	441608
355903	11q	64116513	64118232	1720	7	0.714	Exon(exon2of2)	8702	MACROD1	28992
355903	11q	85724687	85725825	1139	6	0.5	Promoter(<=1kb)	0	SYTL2	54843
355903	11q	124015600	124016477	878	9	0.333	Promoter(<=1kb)	25	OR10G4	390264
355903	11q	124023038	124023849	812	10	0.4	Promoter(<=1kb)	25	OR10G9	219870
355903	12p	4626568	4628549	1982	11	0.455	Exon(exon5of6)	14051	DYRK4	8798
355903	12p	6453119	6453670	552	6	0.667	Promoter(<=1kb)	633	TAPBPL	55080
355903	13q	25096639	25097182	544	23	0.522	Promoter(<=1kb)	771	PABPC3	5042
355903	13q	102732474	102733933	1460	6	0.333	Exon(exon4of4)	25139	CCDC168	643677
355903	14q	20060048	20060884	837	8	0.625	Promoter(<=1kb)	3	OR4L1	122742
355903	14q	21634137	21634589	453	9	0.556	Promoter(<=1kb)	351	OR10G2	26534
355903	14q	70457520	70458238	719	11	0.364	Exon(exon2of2)	5346	ADAM21	8747
355903	14q	94587512	94587839	328	6	0.5	Exon(exon2of2)	-4219	SERPINA3	12
355903	14q	104175275	104177810	2536	6	0.333	Exon(exon12of15)	36235	KIF26A	26153
355903	15q	20534480	20535014	535	7	0.429	Exon(exon8of9)	6786	GOLGA6L6	727832
355903	15q	23439979	23442067	2089	10	0.6	5'UTR	5167	GOLGA6L2	283685
355903	15q	85579423	85582073	2651	15	0.6	Promoter(<=1kb)	-837	AKAP13	11214
355903	15q	99129423	99132517	3095	6	0.333	Exon(exon4of5)	7225	TTC23	64927
355903	16p	1486371	1488463	2093	9	0.778	Promoter(<=1kb)	4	PTX4	390667
355903	16q	74391549	74392004	456	9	0.667	Exon(exon7of7)	13671	NPIPB15	440348
355903	16q	88428539	88429600	1062	7	0.429	Exon(exon3of3)	-23680	ZFPM1	161882
355903	16q	88430623	88436097	5475	13	0.615	Exon(exon3of3)	-17183	ZFPM1	161882
355903	16q	88714632	88717113	2482	7	0.429	Promoter(<=1kb)	0	MIR4722	100616167
355903	16q	89100686	89101050	365	8	0.625	Promoter(<=1kb)	24	ACSF3	197322
355903	16q	89226863	89228419	1557	10	0.6	Promoter(2-3kb)	2229	ZNF778	197320
355903	17p	744946	746966	2021	6	1	3'UTR	5072	GEMIN4	50628
355903	17p	10638198	10641099	2902	7	0.286	Exon(exon19of41)	-8169	MYH3	4621
355903	17p	21300581	21300954	374	8	0.75	3'UTR	9112	MAP2K3	5606
355903	17p	21415470	21416370	901	12	0.75	Exon(exon3of3)	10334	KCNJ12	3768
355903	17q	53823368	53824891	1524	6	0.833	Promoter(<=1kb)	441	KIF2B	84643
355903	17q	76293419	76294016	598	6	0.5	Promoter(2-3kb)	-2167	QRICH2	84074
355903	17q	81645135	81645417	283	6	0.333	Promoter(2-3kb)	2722	TSPAN10	83882
355903	18p	11609646	11610350	705	10	0.8	Promoter(<=1kb)	50	SLC35G4	646000
355903	18q	58535186	58537515	2330	9	0.333	Promoter(<=1kb)	0	ALPK2	115701
355903	19p	1008684	1009586	903	6	0.667	Promoter(2-3kb)	2406	TMEM259	91304
355903	19p	4510548	4513547	3000	17	0.471	Exon(exon3of6)	4157	PLIN4	729359
355903	19p	5455600	5456439	840	7	0.571	Promoter(<=1kb)	183	ZNRF4	148066
355903	19p	8937644	8939234	1591	6	0.667	Exon(exon5of84)	-41554	MUC16	94025
355903	19p	8946313	8952171	5859	20	0.6	Exon(exon3of84)	29171	MUC16	94025
355903	19p	8959116	8962299	3184	12	0.75	Exon(exon3of84)	19043	MUC16	94025
355903	19p	8971838	8978096	6259	15	0.533	Exon(exon1of84)	3246	MUC16	94025
355903	19p	15087213	15088040	828	11	0.273	Promoter(<=1kb)	233	OR1I1	126370
355903	19p	18264753	18267409	2657	8	0.5	5'UTR	7002	IQCN	80726
355903	19p	21971930	21974500	2571	10	0.7	Exon(exon4of4)	14408	ZNF208	7757
355903	19p	23743906	23745300	1395	6	0.333	Exon(exon4of4)	13537	ZNF681	148213
355903	19q	36996730	36997597	868	10	0.6	Exon(exon10of10)	5677	ZNF568	374900
355903	19q	40875742	40878770	3029	7	0.286	Promoter(<=1kb)	0	CYP2A7	1549
355903	19q	43913423	43914878	1456	8	0.5	Exon(exon10of10)	4861	ZNF45	7596
355903	19q	44106512	44108078	1567	7	0	Exon(exon6of6)	-4103	ZNF225	7768

355903	19q	52437918	52439242	1325	7	0.429	Exon(exon4of4)	6504	ZNF534	147658
355903	19q	55912302	55913166	865	6	0.667	Exon(exon5of12)	19145	NLRP13	126204
355903	20p	20052354	20052736	383	6	0	Promoter(<=1kb)	0	CFAP61	26074
355903	20q	63561666	63565531	3866	11	0.636	Promoter(<=1kb)	-61	HELZ2	85441
355903	21q	44539312	44540035	724	6	0.833	Promoter(<=1kb)	160	KRTAP10-1	386677
355903	22q	22352950	22353298	349	13	0.538	Exon(exon1of2)	30478	BMS1P20	96610
355903	22q	36191154	36191906	753	6	0.667	3'UTR	9971	APOL4	80832
355903	23p	8170039	8170141	103	6	0.5	Promoter(1-2kb)	1126	VCX2	51480
355903	23p	35802148	35803010	863	7	0.571	5'UTR	3357	MAGEB16	139604
355903	23p	38285603	38287917	2315	6	1	3'UTR	-11661	RPGR	6103
355903	1p	11766028	11768307	2280	6	0.833	Promoter(<=1kb)	0	C1orf167	284498
357201	1p	16058491	16060000	1510	10	0.9	Exon(exon5of7)	6168	CLCNKB	1188
357201	1p	18481403	18482217	815	6	0.667	Promoter(<=1kb)	421	KLHDC7A	127707
357201	1p	23874604	23875430	827	8	0.5	Exon(exon2of2)	-6310	FUCA1	2517
357201	1p	40067594	40067675	82	6	0	Promoter(<=1kb)	324	CAP1	10487
357201	1p	89186388	89186419	32	9	0.556	Promoter(<=1kb)	107	GBP4	115361
357201	1q	152218469	152221375	2907	17	0.706	Promoter(2-3kb)	2818	HRNR	388697
357201	1q	152303673	152313891	10219	37	0.595	Promoter(<=1kb)	0	FLG-AS1	339400
357201	1q	156669844	156670886	1043	6	1	Exon(exon4of4)	6521	NES	10763
357201	1q	158765805	158766655	851	6	0.5	Promoter(<=1kb)	47	OR6N1	128372
357201	1q	183647749	183648558	810	7	0.429	Exon(exon2of2)	4758	APOBEC4	403314
357201	1q	197101312	197101771	460	6	0.5	Exon(exon18of28)	33373	ASPM	259266
357201	1q	201206099	201209342	3244	9	0.667	Promoter(1-2kb)	1017	IGFN1	91156
357201	1q	201210956	201215147	4192	9	0.556	Promoter(<=1kb)	0	IGFN1	91156
357201	1q	228315976	228318026	2051	7	0.571	Exon(exon50of81)	6492	OBSCN	84033
357201	1q	247841312	247841582	271	6	0.833	Promoter(<=1kb)	314	OR11L1	391189
357201	1q	247949325	247949738	414	10	0.3	Promoter(<=1kb)	467	OR2L8	391190
357201	2p	27578536	27580412	1877	6	0.667	Promoter(2-3kb)	2014	C2orf16	84226
357201	2q	130914528	130917154	2627	7	0.429	Promoter(<=1kb)	0	ARHGEF4	50649
357201	2q	132783032	132785012	1981	10	0.6	Promoter(1-2kb)	-1009	NCKAP5	344148
357201	2q	217847583	217848559	977	6	0.833	Exon(exon19of33)	-5423	TNS1	7145
357201	2q	233713134	233713664	531	8	0.75	Promoter(<=1kb)	142	UGT1A5	54579
357201	2q	233840612	233842185	1574	6	0.667	Promoter(<=1kb)	0	HJURP	55355
357201	2q	238130416	238131546	1131	7	0.429	Promoter(1-2kb)	1468	ESPNL	339768
357201	3p	75736880	75739243	2364	47	0.617	Promoter(<=1kb)	0	MIR4273	100422955
357201	3q	98264413	98265098	686	7	0.571	Promoter(<=1kb)	128	OR5H6	79295
357201	4p	5988383	5989749	1367	7	0.571	Promoter(<=1kb)	0	C4orf50	389197
357201	4p	6300780	6302360	1581	7	0.714	Exon(exon8of8)	6009	WFS1	7466
357201	4q	154489498	154491312	1815	9	0.556	Promoter(<=1kb)	22	DCHS2	54798
357201	4q	185458217	185460011	1795	8	0.625	Promoter(<=1kb)	0	CCDC110	256309
357201	4q	186706638	186708616	1979	7	0.571	Exon(exon2of27)	15217	FAT1	2195
357201	5q	140802063	140803473	1411	6	0.5	Promoter(1-2kb)	1006	PCDHA3	56145
357201	5q	140848579	140850786	2208	7	0.571	Promoter(<=1kb)	807	PCDHA9	9752
357201	5q	141173841	141175025	1185	7	0.857	Promoter(1-2kb)	1197	PCDHB7	56129
357201	5q	141187690	141189425	1736	6	0.5	Promoter(<=1kb)	529	PCDHB9	56127
357201	5q	141955356	141957660	2305	7	0.714	Promoter(<=1kb)	-668	RNF14	9604
357201	5q	151565922	151568158	2237	9	0.778	Promoter(<=1kb)	786	FAT2	2196
357201	6p	46858771	46859389	619	6	0.5	Exon(exon17of21)	3915	ADGRF5	221395
357201	6q	149888581	149890867	2287	7	0.714	Promoter(<=1kb)	0	RAET1E-AS1	100652739
357201	6q	159233455	159234370	916	10	0.5	Exon(exon11of23)	15158	FNDC1	84624
357201	7p	53035678	53036385	708	7	1	Promoter(<=1kb)	45	POM121L12	285877
357201	7q	64991278	64992758	1481	6	0.667	Promoter(<=1kb)	-242	ZNF117	51351
357201	7q	89334686	89336565	1880	7	0.571	Exon(exon4of4)	116532	ZNF804B	219578
357201	7q	100958977	100960873	1897	56	0.446	Promoter(1-2kb)	1012	MUC3A	4584
357201	7q	100991195	100992398	1204	7	0.571	Exon(exon5of15)	-20656	MUC12	10071
357201	7q	100995575	100995785	211	7	0.714	Exon(exon5of15)	-17269	MUC12	10071
357201	7q	101034221	101041312	7092	44	0.523	Promoter(2-3kb)	-2399	MUC17	140453
357201	8p	8376561	8377198	638	6	1	Exon(exon2of5)	4549	PRAG1	157285
357201	8p	10607245	10608432	1188	8	0.5	Exon(exon4of4)	46711	RP1L1	94137
357201	8p	10609614	10610662	1049	8	0.5	Exon(exon4of4)	44481	RP1L1	94137
357201	8p	11331194	11332082	889	7	0.714	Promoter(<=1kb)	306	SLC35G5	83650
357201	8p	13021128	13022030	903	7	0.143	Exon(exon5of5)	9115	TRMT9B	57604
357201	8q	123651873	123652634	762	6	0.333	Promoter(<=1kb)	316	KLHL38	340359
357201	8q	138151949	138153046	1098	6	0.667	Promoter(<=1kb)	0	FAM135B	51059
357201	9q	76175237	76175296	60	10	0.8	Exon(exon14of14)	-13343	PCSK5	5125
357201	9q	76705724	76707804	2081	6	0.5	Promoter(<=1kb)	666	PCA3	50652
357201	9q	87885490	87888819	3330	8	0.625	Promoter(2-3kb)	2613	SPATA31E1	286234
357201	9q	104598545	104599361	817	6	0.5	Promoter(<=1kb)	52	OR13C5	138799
357201	9q	122553263	122554071	809	7	0.429	Promoter(<=1kb)	93	OR1N2	138882
357201	9q	122749914	122750547	634	6	0.833	Promoter(<=1kb)	174	OR1L6	392390
357201	9q	133255635	133256205	571	8	0.875	3'UTR	19009	ABO	28
357201	9q	135484803	135487375	2573	10	0.4	Promoter(1-2kb)	1440	PPP1R26	9858
357201	10p	30027143	30029020	1878	6	0.333	Exon(exon3of4)	30566	JCAD	57608
357201	10q	46549378	46550723	1346	25	0.64	Exon(exon3of3)	4807	GPRIN2	9721
357201	10q	49323169	49326817	3649	17	0.588	Exon(exon3of3)	23895	C10orf71	118461
357201	10q	128102594	128105201	2608	6	0.667	Promoter(<=1kb)	0	MKI67	4288
357201	11p	244106	244197	92	8	0.5	Promoter(<=1kb)	-232	PSMD13	5719

357201	11p	1241677	1243593	1917	7	0.286	Exon(exon31of49)	6083	MUC5B-AS1	112577518
357201	11p	1244757	1248605	3849	14	0.643	Promoter(1-2kb)	1071	MUC5B-AS1	112577518
357201	11p	1250091	1251628	1538	9	0.778	Promoter(<=1kb)	-415	MUC5B-AS1	112577518
357201	11p	5177978	5178478	501	6	0.167	Promoter(<=1kb)	186	OR52Z1	283110
357201	11p	5323362	5324256	895	7	0.429	Promoter(<=1kb)	41	OR51B2	79345
357201	11p	5422212	5423123	912	11	0.636	Promoter(<=1kb)	101	OR51Q1	390061
357201	11p	5515185	5516362	1178	7	0.429	Promoter(<=1kb)	337	UBQLNL	143630
357201	11p	5581045	5581738	694	8	0.375	Promoter(<=1kb)	168	OR52B6	340980
357201	11p	5841302	5841883	582	9	0.333	Promoter(<=1kb)	14	OR52E6	390078
357201	11p	5884818	5885061	244	7	0.429	Promoter(<=1kb)	547	OR52E4	390081
357201	11p	11351961	11352736	776	9	0.222	Promoter(<=1kb)	514	CSNK2A3	283106
357201	11p	12293639	12294368	730	6	0.833	Exon(exon29of35)	6739	MICALCL	84953
357201	11p	43942293	43943348	1056	9	0.778	Promoter(<=1kb)	0	C11orf96	387763
357201	11q	58214757	58215722	966	7	0.286	Promoter(<=1kb)	12	OR1S1	219959
357201	11q	58402523	58403265	743	8	0.5	Promoter(<=1kb)	144	OR5B3	441608
357201	11q	64116513	64118232	1720	7	0.714	Exon(exon2of2)	8702	MACROD1	28992
357201	11q	82732630	82733184	555	6	0.833	Promoter(<=1kb)	680	FAM181B	220382
357201	11q	85724687	85725825	1139	6	0.5	Promoter(<=1kb)	0	SYTL2	54843
357201	11q	124015601	124016477	877	9	0.333	Promoter(<=1kb)	26	OR10G4	390264
357201	11q	124023038	124023849	812	9	0.444	Promoter(<=1kb)	25	OR10G9	219870
357201	11q	124382526	124383285	760	8	0.625	Promoter(<=1kb)	58	OR8B2	26595
357201	12p	4626571	4628549	1979	9	0.556	Exon(exon5of6)	14054	DYRK4	8798
357201	13q	102732474	102733933	1460	6	0.333	Exon(exon4of4)	25139	CCDC168	643677
357201	14q	20060048	20060884	837	8	0.625	Promoter(<=1kb)	3	OR4L1	122742
357201	14q	20224029	20224484	456	6	0.5	Promoter(<=1kb)	319	OR11H6	122748
357201	14q	22633879	22634450	572	9	0.333	Exon(exon2of2)	32212	ABHD4	63874
357201	14q	70457532	70458540	1009	11	0.273	Exon(exon2of2)	5358	ADAM21	8747
357201	14q	94587512	94587839	328	6	0.5	Exon(exon2of2)	-4219	SERPINA3	12
357201	14q	104939262	104942618	3357	10	0.2	5'UTR	7102	PLD4	122618
357201	14q	104943622	104945444	1823	7	0.571	Exon(exon6of6)	9958	AHNAK2	113146
357201	14q	104947901	104950428	2528	16	0.625	Exon(exon6of6)	4974	AHNAK2	113146
357201	14q	104951557	104953878	2322	9	0.333	Promoter(1-2kb)	1524	AHNAK2	113146
357201	15q	23439979	23442067	2089	15	0.467	5'UTR	5167	GOLGA6L2	283685
357201	15q	40621642	40623696	2055	6	0.333	Promoter(<=1kb)	0	KNL1	57082
357201	15q	73702465	73703760	1296	6	0.833	Promoter(<=1kb)	-149	CD276	80381
357201	15q	85579207	85581800	2594	14	0.643	Promoter(1-2kb)	-1110	AKAP13	11214
357201	15q	88857633	88859365	1733	6	0.167	Exon(exon12of18)	10390	ACAN	176
357201	15q	99129423	99132517	3095	6	0.333	Exon(exon4of5)	7225	TTC23	64927
357201	15q	100569472	100570060	589	6	0.667	Promoter(<=1kb)	571	LINS1	55180
357201	16p	789084	790597	1514	6	0.167	Promoter(<=1kb)	0	CHTF18	63922
357201	16p	1228744	1229622	879	6	0.833	Promoter(<=1kb)	540	TPSB2	64499
357201	16p	1256345	1256896	552	6	0.667	Promoter(<=1kb)	276	TPSD1	23430
357201	16p	1486322	1488463	2142	13	0.692	Promoter(<=1kb)	4	PTX4	390667
357201	16p	4207130	4208004	875	6	0.5	Exon(exon2of7)	31739	SRL	6345
357201	16p	28495551	28497395	1845	6	0.333	Promoter(<=1kb)	0	CLN3	1201
357201	16q	84175984	84176258	275	6	0.5	Promoter(<=1kb)	0	DNAAF1	123872
357201	16q	88433131	88436097	2967	6	0.5	Exon(exon3of3)	-17183	ZFPM1	161882
357201	16q	88714717	88717113	2397	9	0.556	Promoter(<=1kb)	0	MIR4722	100616167
357201	16q	89226863	89228289	1427	8	0.625	Promoter(2-3kb)	2229	ZNF778	197320
357201	17p	413503	413831	329	6	0.667	Promoter(<=1kb)	-120	LOC10537143(	105371430
357201	17p	10638198	10641099	2902	7	0.429	Exon(exon19of41)	-8169	MYH3	4621
357201	17p	21300581	21300978	398	12	0.75	3'UTR	9112	MAP2K3	5606
357201	17p	21415470	21416370	901	7	0.857	Exon(exon3of3)	10334	KCNJ12	3768
357201	17q	81645135	81645417	283	6	0.333	Promoter(2-3kb)	2722	TSPAN10	83882
357201	18p	11609728	11610164	437	14	0.643	Promoter(<=1kb)	132	SLC35G4	646000
357201	18p	11644365	11644565	201	7	0.857	Exon(exon1of1)	10376	MIR7153	102465690
357201	18q	58535186	58538030	2845	18	0.556	Promoter(<=1kb)	0	ALPK2	115701
357201	19p	4510548	4513547	3000	22	0.5	Exon(exon3of6)	4157	PLIN4	729359
357201	19p	5455600	5456439	840	7	0.571	Promoter(<=1kb)	183	ZNRF4	148066
357201	19p	8937644	8939234	1591	6	0.667	Exon(exon5of84)	-41554	MUC16	94025
357201	19p	8946313	8952171	5859	20	0.6	Exon(exon3of84)	29171	MUC16	94025
357201	19p	8959116	8962299	3184	12	0.75	Exon(exon3of84)	19043	MUC16	94025
357201	19p	8971838	8978096	6259	16	0.562	Exon(exon1of84)	3246	MUC16	94025
357201	19p	12430157	12432437	2281	12	0.333	3'UTR	8584	ZNF443	10224
357201	19p	15087213	15088040	828	9	0.333	Promoter(<=1kb)	233	OR1I1	126370
357201	19p	21971930	21974500	2571	7	0.714	Exon(exon4of4)	14408	ZNF208	7757
357201	19p	23743906	23745300	1395	6	0.333	Exon(exon4of4)	13537	ZNF681	148213
357201	19q	37886924	37889059	2136	6	0.833	Exon(exon6of6)	17535	WDR87	83889
357201	19q	38504270	38506350	2081	6	0.5	Exon(exon50of105)	-5221	RYR1	6261
357201	19q	39877222	39877880	659	6	0.5	Exon(exon20of28)	9412	FCGBP	8857
357201	19q	43913423	43914878	1456	8	0.5	Exon(exon10of10)	4861	ZNF45	7596
357201	19q	43966037	43967171	1135	6	0.167	Promoter(<=1kb)	-691	ZNF155	7711
357201	19q	44106512	44108078	1567	8	0.125	Exon(exon6of6)	-4103	ZNF225	7768
357201	19q	52365769	52366744	976	6	1	Exon(exon6of6)	-3173	ZNF880	400713
357201	19q	52437918	52439242	1325	7	0.429	Exon(exon4of4)	6504	ZNF534	147658
357201	19q	55358736	55359651	916	6	0.667	Promoter(<=1kb)	0	FAM71E2	284418
357201	19q	55517821	55518642	822	6	1	Exon(exon14of14)	17661	SBK2	646643

357201	19q	55912302	55913166	865	6	0.667	Exon(exon5of12)	19145	NLRP13	126204
357201	19q	58416932	58417769	838	6	0.5	3'UTR	8155	ZNF584	201514
357201	20p	5922421	5923394	974	9	0.556	Exon(exon4of5)	6923	CHGB	1114
357201	20q	63349752	63350772	1021	6	0.5	3'UTR	3794	CHRNA4	1137
357201	21q	44591587	44592359	773	6	0.333	Promoter(<=1kb)	146	KRTAP10-6	386674
357201	21q	44600627	44601692	1066	10	0.8	Promoter(<=1kb)	30	KRTAP10-7	386675
357201	21q	44637474	44638143	670	6	0.5	Promoter(<=1kb)	118	KRTAP10-10	353333
357201	22q	22352950	22353298	349	13	0.538	Exon(exon1of2)	30478	BMS1P20	96610
357201	22q	36191154	36191906	753	6	0.667	3'UTR	9971	APOL4	80832
357201	23p	3320126	3323750	3625	9	0.556	Exon(exon5of7)	22902	MXRA5	25878
357201	23p	8170039	8170141	103	6	0.5	Promoter(1-2kb)	1126	VCX2	51480
357201	23p	35802148	35803010	863	7	0.571	5'UTR	3357	MAGEB16	139604
357201	23q	136874183	136874416	234	8	0.75	Promoter(<=1kb)	-201	RBMX	27316
357725	1p	12847526	12847995	470	11	0.455	Promoter(<=1kb)	730	HNRNPCL1	343069
357725	1p	12859036	12860079	1044	6	0.333	Promoter(1-2kb)	1950	PRAMEF2	65122
357725	1p	13369166	13369564	399	10	0.8	Promoter(2-3kb)	2336	PRAMEF19	645414
357725	1p	13370686	13371119	434	9	0.444	Promoter(<=1kb)	781	PRAMEF19	645414
357725	1p	16058491	16060000	1510	13	0.846	Exon(exon5of7)	6168	CLCNKB	1188
357725	1p	18481403	18482308	906	9	0.667	Promoter(<=1kb)	421	KLHDC7A	127707
357725	1p	23874604	23875430	827	9	0.556	Exon(exon2of2)	-6310	FUCA1	2517
357725	1p	40067594	40067675	82	6	0	Promoter(<=1kb)	324	CAP1	10487
357725	1p	89186388	89186419	32	9	0.556	Promoter(<=1kb)	107	GBP4	115361
357725	1q	152213274	152213320	47	8	0.5	Exon(exon3of3)	10873	HRNR	388697
357725	1q	152218469	152221375	2907	16	0.688	Promoter(2-3kb)	2818	HRNR	388697
357725	1q	152303673	152313891	10219	38	0.605	Promoter(<=1kb)	0	FLG-AS1	339400
357725	1q	156669844	156671817	1974	8	1	Exon(exon4of4)	5590	NES	10763
357725	1q	158765805	158766655	851	6	0.5	Promoter(<=1kb)	47	OR6N1	128372
357725	1q	169542317	169542882	566	6	0.167	Exon(exon13of25)	-26572	F5	2153
357725	1q	201206099	201209342	3244	12	0.667	Promoter(1-2kb)	1017	IGFN1	91156
357725	1q	240207634	240207680	47	7	0.571	Exon(exon5of18)	-28091	FMN2	56776
357725	1q	247841312	247841582	271	6	0.833	Promoter(<=1kb)	314	OR11L1	391189
357725	1q	247948924	247949759	836	9	0.444	Promoter(<=1kb)	66	OR2L8	391190
357725	1q	248294677	248295458	782	7	0.571	Promoter(<=1kb)	142	OR2T12	127064
357725	1q	248573992	248574210	219	6	0.833	Promoter(<=1kb)	547	OR2T34	127068
357725	1q	248681658	248682198	541	7	0.571	Promoter(<=1kb)	130	OR14I1	401994
357725	2p	48580657	48582454	1798	7	0.571	Promoter(<=1kb)	0	STON1	11037
357725	2q	95944651	95945173	523	6	0.5	Promoter(<=1kb)	0	ANKRD36C	400986
357725	2q	130193878	130194376	499	6	0.833	Exon(exon4of5)	4063	TUBA3E	112714
357725	2q	132783534	132785001	1468	7	0.429	Promoter(1-2kb)	-1511	NCKAP5	344148
357725	2q	178739433	178741811	2379	6	0.5	Exon(exon45of191)	26014	TTN	7273
357725	2q	185789865	185794632	4768	12	0.75	Promoter(<=1kb)	0	FSIP2	401024
357725	2q	185805377	185808170	2794	6	0.333	Promoter(<=1kb)	0	FSIP2	401024
357725	2q	233713134	233713783	650	11	0.636	Promoter(<=1kb)	142	UGT1A5	54579
357725	2q	240041845	240042154	310	6	0.167	Downstream(2-3kb)	3918	OR6B3	150681
357725	3p	75736929	75739007	2079	14	0.643	Promoter(<=1kb)	0	MIR4273	100422955
357725	3q	98264413	98265098	686	7	0.571	Promoter(<=1kb)	128	OR5H6	79295
357725	3q	194341097	194342571	1475	7	0.714	Exon(exon2of2)	8747	CPN2	1370
357725	4p	5988383	5989749	1367	7	0.571	Promoter(<=1kb)	0	C4orf50	389197
357725	4p	6300792	6302360	1569	7	0.714	Exon(exon8of8)	6021	WFS1	7466
357725	4p	8227004	8228508	1505	8	0.25	Promoter(<=1kb)	-24	SH3TC1	54436
357725	4p	10443803	10446224	2422	6	0.333	Exon(exon3of3)	10952	ZNF518B	85460
357725	4q	154489498	154491312	1815	9	0.556	Promoter(<=1kb)	22	DCHS2	54798
357725	4q	186619481	186621601	2121	7	0.286	Exon(exon10of27)	-9411	FAT1	2195
357725	5q	140802149	140803473	1325	6	0.667	Promoter(1-2kb)	1092	PCDHA3	56145
357725	5q	140807352	140807737	386	6	0.833	Promoter(<=1kb)	271	PCDHA4	56144
357725	5q	141174000	141175025	1026	6	0.833	Promoter(1-2kb)	1356	PCDHB7	56129
357725	5q	141955356	141957660	2305	6	0.667	Promoter(<=1kb)	-668	RNF14	9604
357725	5q	151565922	151568158	2237	9	0.778	Promoter(<=1kb)	786	FAT2	2196
357725	6p	1312843	1313745	903	6	0.5	Promoter(<=1kb)	745	FOXQ1	94234
357725	6p	16326440	16327684	1245	7	0.429	Exon(exon8of9)	36495	GMPR	2766
357725	6p	46858240	46859389	1150	7	0.429	Exon(exon17of21)	3915	ADGRF5	221395
357725	6q	64591274	64591961	688	10	0.5	Exon(exon26of43)	121374	EYS	346007
357725	6q	149888581	149890867	2287	7	0.714	Promoter(<=1kb)	0	RAET1E-AS1	100652739
357725	6q	159233455	159234370	916	10	0.5	Exon(exon11of23)	15158	FNDC1	84624
357725	7p	6330446	6330944	499	6	1	Exon(exon2of2)	7749	FAM220A	84792
357725	7p	12369637	12370736	1100	6	0.667	3'UTR	-13307	VWDE	221806
357725	7p	45082865	45084866	2002	7	0.429	Promoter(1-2kb)	-1465	NACAD	23148
357725	7p	53035678	53036385	708	7	1	Promoter(<=1kb)	45	POM121L12	285877
357725	7q	100958977	100960873	1897	56	0.446	Promoter(1-2kb)	1012	MUC3A	4584
357725	7q	100991195	100992398	1204	7	0.571	Exon(exon5of15)	-20656	MUC12	10071
357725	7q	100995575	100996058	484	9	0.778	Exon(exon5of15)	-16996	MUC12	10071
357725	7q	101004421	101004836	416	6	0.667	Exon(exon5of15)	-8218	MUC12	10071
357725	7q	101034361	101040583	6223	33	0.485	Exon(exon3of12)	-3128	MUC17	140453
357725	8p	10607245	10608432	1188	8	0.5	Exon(exon4of4)	46711	RP1L1	94137
357725	8p	10609614	10611045	1432	9	0.556	Exon(exon4of4)	44098	RP1L1	94137
357725	8q	123651655	123652634	980	7	0.571	Promoter(<=1kb)	316	KLHL38	340359
357725	8q	142664564	142665970	1407	9	0.889	Exon(exon2of2)	4000	JRK	8629

357725	9q	76175237	76175296	60	10	0.8	Exon(exon14of14)	-13343	PCSK5	5125
357725	9q	76703555	76707804	4250	13	0.462	Promoter(<=1kb)	0	PCA3	50652
357725	9q	76709263	76710843	1581	8	0.5	Promoter(<=1kb)	0	PRUNE2	158471
357725	9q	104598545	104599361	817	12	0.583	Promoter(<=1kb)	52	OR13C5	138799
357725	9q	104694482	104695444	963	6	0.667	Promoter(<=1kb)	0	OR13D1	286365
357725	9q	122553263	122554071	809	8	0.5	Promoter(<=1kb)	93	OR1N2	138882
357725	9q	122628595	122629398	804	7	0.286	Promoter(<=1kb)	175	OR1B1	347169
357725	9q	122749914	122750547	634	6	0.833	Promoter(<=1kb)	174	OR1L6	392390
357725	10q	46549378	46550723	1346	25	0.64	Exon(exon3of3)	4807	GPRIN2	9721
357725	10q	89737450	89738561	1112	6	0	Exon(exon20of33)	13874	KIF20B	9585
357725	10q	128102594	128105201	2608	12	0.583	Promoter(<=1kb)	0	MKI67	4288
357725	10q	128106210	128109280	3071	8	0.875	Exon(exon12of14)	-3082	MKI67	4288
357725	11p	244106	244197	92	8	0.5	Promoter(<=1kb)	-232	PSMD13	5719
357725	11p	1194354	1196902	2549	7	0.571	Exon(exon34of49)	-26164	MUC5B	727897
357725	11p	1246687	1249735	3049	10	0.5	Promoter(<=1kb)	0	MUC5B-AS1	112577518
357725	11p	5177978	5178478	501	6	0.167	Promoter(<=1kb)	186	OR52Z1	283110
357725	11p	5389704	5390350	647	6	0.5	Promoter(<=1kb)	327	OR51M1	390059
357725	11p	5402638	5403322	685	9	0.444	Promoter(<=1kb)	41	OR51J1	79470
357725	11p	5422212	5423123	912	11	0.636	Promoter(<=1kb)	101	OR51Q1	390061
357725	11p	5440604	5441472	869	6	0.833	Promoter(<=1kb)	42	OR51I1	390063
357725	11p	5515185	5515931	747	6	0.333	Promoter(<=1kb)	768	UBQLNL	143630
357725	11p	5581045	5581738	694	8	0.375	Promoter(<=1kb)	168	OR52B6	340980
357725	11p	5841302	5841883	582	9	0.333	Promoter(<=1kb)	14	OR52E6	390078
357725	11p	11351961	11352736	776	9	0.222	Promoter(<=1kb)	514	CSNK2A3	283106
357725	11p	12293639	12294842	1204	8	0.75	Exon(exon29of35)	6739	MICALCL	84953
357725	11p	18173280	18173901	622	6	0.333	Promoter(<=1kb)	443	MRGPRX4	117196
357725	11q	58214757	58215722	966	8	0.25	Promoter(<=1kb)	12	OR1S1	219959
357725	11q	64116513	64118232	1720	6	0.667	Exon(exon2of2)	8702	MACROD1	28992
357725	11q	64315804	64315856	53	7	0.714	Promoter(1-2kb)	1485	TRMT112	51504
357725	11q	82732630	82733184	555	6	0.833	Promoter(<=1kb)	680	FAM181B	220382
357725	11q	85724687	85725825	1139	6	0.5	Promoter(<=1kb)	0	SYTL2	54843
357725	11q	123906790	123907324	535	6	0.667	Promoter(<=1kb)	644	OR8D4	338662
357725	11q	124038366	124038988	623	8	0.875	Promoter(<=1kb)	13	OR10G7	390265
357725	11q	124382526	124383285	760	8	0.625	Promoter(<=1kb)	58	OR8B2	26595
357725	12p	4626568	4628549	1982	11	0.455	Exon(exon5of6)	14051	DYRK4	8798
357725	12p	6453119	6453670	552	7	0.714	Promoter(<=1kb)	633	TAPBPL	55080
357725	12q	48525773	48526013	241	6	0.333	Promoter(<=1kb)	141	OR8S1	341568
357725	12q	52316096	52317765	1670	6	0.667	Exon(exon4of9)	3633	KRT83	3889
357725	13q	24434450	24435347	898	7	0.571	Exon(exon31of34)	19787	PARP4	143
357725	13q	25096713	25097136	424	22	0.545	Promoter(<=1kb)	845	PABPC3	5042
357725	13q	102731670	102733933	2264	7	0.286	Exon(exon4of4)	25139	CCDC168	643677
357725	14q	20060048	20060523	476	6	0.5	Promoter(<=1kb)	3	OR4L1	122742
357725	14q	20640982	20641567	586	6	0.5	Promoter(<=1kb)	124	OR6S1	341799
357725	14q	44504986	44506403	1418	8	0.75	Promoter(<=1kb)	880	FSCB	84075
357725	14q	70457520	70458948	1429	13	0.385	Exon(exon2of2)	5346	ADAM21	8747
357725	14q	77026548	77027532	985	6	0.5	Promoter(1-2kb)	1176	IRF2BPL	64207
357725	14q	104175275	104178588	3314	10	0.3	Exon(exon12of15)	36235	KIF26A	26153
357725	14q	104939262	104942618	3357	11	0.364	5'UTR	7102	PLD4	122618
357725	14q	104943622	104953878	10257	47	0.596	Promoter(1-2kb)	1524	AHNAK2	113146
357725	15q	23439979	23442067	2089	11	0.545	5'UTR	5167	GOLGA6L2	283685
357725	15q	40621642	40623696	2055	6	0.333	Promoter(<=1kb)	0	KNL1	57082
357725	15q	42690621	42692660	2040	6	0.5	Promoter(1-2kb)	1026	STARD9	57519
357725	15q	59206980	59208056	1077	7	0.714	Promoter(<=1kb)	137	LDHAL6B	92483
357725	15q	85579083	85582073	2991	16	0.562	Promoter(<=1kb)	-837	AKAP13	11214
357725	16p	376432	377820	1389	6	0.5	Promoter(<=1kb)	0	PGAP6	58986
357725	16p	1228744	1229716	973	9	0.667	Promoter(<=1kb)	446	TPSB2	64499
357725	16p	1256345	1256980	636	7	0.857	Promoter(<=1kb)	276	TPSD1	23430
357725	16p	1486371	1488463	2093	8	0.75	Promoter(<=1kb)	4	PTX4	390667
357725	16p	4207130	4208004	875	6	0.5	Exon(exon2of7)	31739	SRL	6345
357725	16q	74391416	74391928	513	8	0.75	Exon(exon7of7)	13538	NPIPB15	440348
357725	16q	88428539	88431889	3351	11	0.364	Exon(exon3of3)	-21391	ZFPM1	161882
357725	16q	88433131	88436097	2967	6	0.667	Exon(exon3of3)	-17183	ZFPM1	161882
357725	16q	89226863	89228289	1427	8	0.625	Promoter(2-3kb)	2229	ZNF778	197320
357725	17p	2299649	2300159	511	6	0.167	Exon(exon2of19)	-3224	SRR	63826
357725	17p	21300581	21300978	398	12	0.75	3'UTR	9112	MAP2K3	5606
357725	17q	76293419	76294016	598	6	0.5	Promoter(2-3kb)	-2167	QRICH2	84074
357725	17q	81645135	81645417	283	6	0.333	Promoter(2-3kb)	2722	TSPAN10	83882
357725	18p	11609904	11610469	566	9	0.667	Promoter(<=1kb)	308	SLC35G4	646000
357725	18p	11644365	11644684	320	7	0.857	Exon(exon1of1)	10257	MIR7153	102465690
357725	18q	58535186	58538030	2845	18	0.556	Promoter(<=1kb)	0	ALPK2	115701
357725	19p	4510548	4513547	3000	19	0.526	Exon(exon3of6)	4157	PLIN4	729359
357725	19p	5455600	5456439	840	7	0.571	Promoter(<=1kb)	183	ZNRF4	148066
357725	19p	8948231	8950136	1906	6	0.667	Exon(exon3of84)	31206	MUC16	94025
357725	19p	8959518	8962066	2549	6	0.333	Exon(exon3of84)	19276	MUC16	94025
357725	19p	8964274	8967127	2854	19	0.632	Exon(exon3of84)	14215	MUC16	94025
357725	19p	14841061	14841331	271	6	0.5	Promoter(<=1kb)	546	OR7A10	390892
357725	19p	18264753	18267409	2657	8	0.5	5'UTR	7002	IQCN	80726

357725	19p	22314389	22314412	24	6	0	Exon(exon4of4)	27948	ZNF729	100287226
357725	19p	23743906	23745300	1395	6	0.333	Exon(exon4of4)	13537	ZNF681	148213
357725	19q	36996730	36997597	868	10	0.6	Exon(exon10of10)	5677	ZNF568	374900
357725	19q	38504270	38506350	2081	6	0.5	Exon(exon50of105)	-5221	RYR1	6261
357725	19q	39877222	39877880	659	7	0.429	Exon(exon20of28)	9412	FCGBP	8857
357725	19q	40880174	40880622	449	7	0.429	Promoter(<=1kb)	-84	CYP2A7	1549
357725	19q	43913423	43914878	1456	9	0.556	Exon(exon10of10)	4861	ZNF45	7596
357725	19q	43996326	43997366	1041	6	0.5	Exon(exon5of5)	5419	LOC10192806:	101928063
357725	19q	44106512	44108078	1567	8	0.125	Exon(exon6of6)	-4103	ZNF225	7768
357725	19q	52365769	52366744	976	6	1	Exon(exon6of6)	-3173	ZNF880	400713
357725	19q	52437918	52439242	1325	7	0.429	Exon(exon4of4)	6504	ZNF534	147658
357725	19q	55912302	55913166	865	6	0.667	Exon(exon5of12)	19145	NLRP13	126204
357725	19q	57639918	57641641	1724	7	0.429	3'UTR	6693	ZNF211	10520
357725	20p	5922421	5923394	974	6	0.5	Exon(exon4of5)	6923	CHGB	1114
357725	20q	63561666	63565531	3866	12	0.667	Promoter(<=1kb)	-61	HELZ2	85441
357725	21q	41740862	41741990	1129	6	0.833	Exon(exon2of2)	4851	MIR6814	102465488
357725	21q	44550835	44551416	582	6	0.833	Promoter(<=1kb)	89	KRTAP10-2	386679
357725	21q	44600627	44601692	1066	10	0.7	Promoter(<=1kb)	30	KRTAP10-7	386675
357725	21q	44637474	44638041	568	9	0.333	Promoter(<=1kb)	118	KRTAP10-10	353333
357725	22q	22352950	22353380	431	16	0.5	Exon(exon1of2)	30478	BMS1P20	96610
357725	22q	36191154	36191906	753	6	0.667	3'UTR	9971	APOL4	80832
357725	22q	39100331	39102033	1703	6	0.833	Promoter(<=1kb)	52	APOBEC3H	164668
357725	23p	8170039	8170141	103	6	0.5	Promoter(1-2kb)	1126	VCX2	51480
357725	23p	35802148	35803010	863	7	0.571	5'UTR	3357	MAGEB16	139604
357725	23q	101494051	101494598	548	6	0.5	Exon(exon2of2)	6007	ARMCX4	100131755
357725	23q	136874183	136874416	234	8	0.75	Promoter(<=1kb)	-201	RBMX	27316
379890	1p	11765960	11768307	2348	7	0.857	Promoter(<=1kb)	0	C1orf167	284498
379890	1p	11778784	11779941	1158	7	0.714	Promoter(<=1kb)	0	C1orf167-AS1	102724659
379890	1p	12029085	12030097	1013	6	0.833	Promoter(<=1kb)	0	MIR6729	102466982
379890	1p	12795695	12795957	263	6	0.5	Promoter(1-2kb)	1004	PRAMEF1	65121
379890	1p	12847526	12847995	470	10	0.4	Promoter(<=1kb)	730	HNRNPCL1	343069
379890	1p	12859036	12860079	1044	6	0.333	Promoter(1-2kb)	1950	PRAMEF2	65122
379890	1p	13369166	13369564	399	7	0.714	Promoter(2-3kb)	2336	PRAMEF19	645414
379890	1p	13370686	13371119	434	8	0.375	Promoter(<=1kb)	781	PRAMEF19	645414
379890	1p	13391965	13392142	178	6	0.333	Promoter(2-3kb)	2333	PRAMEF17	391004
379890	1p	16058491	16060000	1510	13	0.846	Exon(exon5of7)	6168	CLCNKB	1188
379890	1p	18481403	18482217	815	6	0.667	Promoter(<=1kb)	421	KLHDC7A	127707
379890	1p	23874604	23875430	827	8	0.5	Exon(exon2of2)	-6310	FUCA1	2517
379890	1p	40067594	40067675	82	6	0	Promoter(<=1kb)	324	CAP1	10487
379890	1p	89186388	89186419	32	9	0.556	Promoter(<=1kb)	107	GBP4	115361
379890	1q	152219233	152221375	2143	17	0.765	Promoter(2-3kb)	2818	HRNR	388697
379890	1q	158765805	158766655	851	6	0.5	Promoter(<=1kb)	47	OR6N1	128372
379890	1q	228315976	228318038	2063	9	0.667	Exon(exon50of81)	6492	OBSCN	84033
379890	1q	247841312	247841582	271	6	0.833	Promoter(<=1kb)	314	OR11L1	391189
379890	1q	247949325	247949738	414	9	0.333	Promoter(<=1kb)	467	OR2L8	391190
379890	2p	27578536	27579938	1403	6	0.333	Promoter(2-3kb)	2014	C2orf16	84226
379890	2q	97547464	97547725	262	6	0.5	3'UTR	-35855	ANKRD36B	57730
379890	2q	130914528	130916712	2185	6	0.333	Promoter(<=1kb)	0	ARHGEF4	50649
379890	2q	132783534	132785001	1468	7	0.429	Promoter(1-2kb)	-1511	NCKAP5	344148
379890	2q	167246794	167248478	1685	7	0.714	Promoter(<=1kb)	-204	XIRP2	129446
379890	2q	184936178	184937636	1459	8	0.375	Exon(exon4of4)	69813	ZNF804A	91752
379890	2q	233681970	233682638	669	6	0.5	Promoter(<=1kb)	0	UGT1A7	54577
379890	2q	233840612	233842185	1574	6	0.5	Promoter(<=1kb)	0	HJURP	55355
379890	2q	237762685	237764060	1376	10	0.5	Exon(exon8of8)	-4137	LRRFIP1	9208
379890	2q	238130271	238131546	1276	7	0.286	Promoter(1-2kb)	1323	ESPNL	339768
379890	2q	240041845	240042131	287	6	0.167	Downstream(2-3kb)	3941	OR6B3	150681
379890	3p	31989532	31990905	1374	6	0.333	Exon(exon2of2)	7761	ZNF860	344787
379890	3p	75736880	75739243	2364	68	0.632	Promoter(<=1kb)	0	MIR4273	100422955
379890	3q	98264413	98265098	686	7	0.571	Promoter(<=1kb)	128	OR5H6	79295
379890	4p	1394625	1395373	749	9	0.333	Exon(exon1of1)	9813	UVSSA	57654
379890	4p	5988383	5989749	1367	7	0.571	Promoter(<=1kb)	0	C4orf50	389197
379890	4p	6300792	6302360	1569	6	0.833	Exon(exon8of8)	6021	WFS1	7466
379890	4p	10443803	10446224	2422	6	0.333	Exon(exon3of3)	10952	ZNF518B	85460
379890	4p	38774449	38775552	1104	9	0.556	Promoter(<=1kb)	644	TLR10	81793
379890	4q	121036404	121037542	1139	6	0.333	Promoter(1-2kb)	1442	NDNF	79625
379890	4q	186706638	186708616	1979	6	0.667	Exon(exon2of27)	15217	FAT1	2195
379890	5q	79731782	79735549	3768	15	0.333	Promoter(2-3kb)	-2593	CMYA5	202333
379890	5q	83537326	83539905	2580	6	0.333	Promoter(1-2kb)	1712	VCAN	1462
379890	5q	140807352	140807737	386	6	0.833	Promoter(<=1kb)	271	PCDHA4	56144
379890	5q	140848579	140850786	2208	6	0.5	Promoter(<=1kb)	807	PCDHA9	9752
379890	5q	151521550	151522069	520	6	0.667	Promoter(<=1kb)	79	MIR6499	102465246
379890	5q	155015598	155017546	1949	6	0.833	Promoter(1-2kb)	1843	KIF4B	285643
379890	6p	26370344	26370479	136	6	0.5	Promoter(<=1kb)	0	BTN3A2	11118
379890	6p	46858771	46859502	732	8	0.5	Exon(exon17of21)	3802	ADGRF5	221395
379890	6q	64591274	64591961	688	10	0.5	Exon(exon26of43)	121374	EYS	346007
379890	6q	149888581	149890867	2287	7	0.714	Promoter(<=1kb)	0	RAET1E-AS1	100652739
379890	6q	159231899	159234370	2472	12	0.583	Exon(exon11of23)	13602	FNDC1	84624

379890	7p	38353718	38353991	274	9	0.667	Exon(exon2of2)	3699	TRG-AS1	100506776
379890	7p	53035678	53036385	708	7	1	Promoter(<=1kb)	45	POM121L12	285877
379890	7q	100958977	100960873	1897	56	0.446	Promoter(1-2kb)	1012	MUC3A	4584
379890	7q	100991195	100992398	1204	8	0.625	Exon(exon5of15)	-20656	MUC12	10071
379890	7q	100995547	100996058	512	9	0.889	Exon(exon5of15)	-16996	MUC12	10071
379890	7q	101003685	101004836	1152	7	0.714	Exon(exon5of15)	-8218	MUC12	10071
379890	7q	149818015	149819792	1778	6	0.667	Promoter(2-3kb)	-2352	SSPO	23145
379890	8p	8376561	8377994	1434	6	1	Exon(exon2of5)	3753	PRAG1	157285
379890	8p	10607245	10608432	1188	8	0.5	Exon(exon4of4)	46711	RP1L1	94137
379890	8p	10609614	10610662	1049	8	0.5	Exon(exon4of4)	44481	RP1L1	94137
379890	8p	11331194	11332026	833	8	0.625	Promoter(<=1kb)	306	SLC35G5	83650
379890	8p	12132686	12133940	1255	6	0.667	Promoter(<=1kb)	498	USP17L7	392197
379890	8p	13021128	13022030	903	7	0.143	Exon(exon5of5)	9115	TRMT9B	57604
379890	9p	116832	117720	889	9	0.667	Promoter(<=1kb)	484	FOXD4	2298
379890	9p	39078723	39078846	124	6	0.667	Exon(exon22of24)	7302	CNTNAP3	79937
379890	9q	76175237	76175300	64	6	0.5	Exon(exon14of14)	-13339	PCSK5	5125
379890	9q	76705724	76707804	2081	6	0.5	Promoter(<=1kb)	666	PCA3	50652
379890	9q	104504315	104505071	757	6	0.5	Promoter(<=1kb)	52	OR13F1	138805
379890	9q	104598545	104599361	817	12	0.583	Promoter(<=1kb)	52	OR13C5	138799
379890	9q	122553263	122554071	809	8	0.5	Promoter(<=1kb)	93	OR1N2	138882
379890	9q	122628595	122629398	804	7	0.286	Promoter(<=1kb)	175	OR1B1	347169
379890	9q	122749914	122750547	634	6	0.833	Promoter(<=1kb)	174	OR1L6	392390
379890	9q	135484803	135487213	2411	8	0.25	Promoter(1-2kb)	1440	PPP1R26	9858
379890	10p	47663	48753	1091	6	0.833	Promoter(<=1kb)	516	TUBB8	347688
379890	10p	30027143	30029814	2672	7	0.429	Exon(exon3of4)	29772	JCAD	57608
379890	10q	46549378	46550723	1346	26	0.654	Exon(exon3of3)	4807	GPRIN2	9721
379890	10q	49323169	49326817	3649	18	0.611	Exon(exon3of3)	23895	C10orf71	118461
379890	10q	89737450	89738561	1112	6	0	Exon(exon20of33)	13874	KIF20B	9585
379890	11p	244106	244197	92	8	0.5	Promoter(<=1kb)	-232	PSMD13	5719
379890	11p	1246095	1247378	1284	9	0.556	Promoter(2-3kb)	2298	MUC5B-AS1	112577518
379890	11p	4954856	4955581	726	6	0.833	Promoter(<=1kb)	132	OR51A2	401667
379890	11p	5177978	5178478	501	6	0.167	Promoter(<=1kb)	186	OR52Z1	283110
379890	11p	5323451	5324256	806	6	0.5	Promoter(<=1kb)	41	OR51B2	79345
379890	11p	5351521	5352416	896	17	0.529	Promoter(<=1kb)	13	OR51B6	390058
379890	11p	5389419	5390350	932	8	0.5	Promoter(<=1kb)	42	OR51M1	390059
379890	11p	5422212	5423123	912	11	0.727	Promoter(<=1kb)	101	OR51Q1	390061
379890	11p	5581045	5581738	694	8	0.375	Promoter(<=1kb)	168	OR52B6	340980
379890	11p	5884818	5885061	244	6	0.333	Promoter(<=1kb)	547	OR52E4	390081
379890	11p	5986042	5986542	501	6	1	Promoter(<=1kb)	443	OR52L1	338751
379890	11p	11351961	11352736	776	8	0.125	Promoter(<=1kb)	514	CSNK2A3	283106
379890	11p	12293639	12294368	730	7	0.857	Exon(exon29of35)	6739	MICALCL	84953
379890	11p	18173280	18173901	622	6	0.333	Promoter(<=1kb)	443	MRGPRX4	117196
379890	11p	34916266	34916763	498	6	0.667	Promoter(<=1kb)	0	APIP	51074
379890	11p	43942293	43943348	1056	9	0.778	Promoter(<=1kb)	0	C11orf96	387763
379890	11q	54603136	54603820	685	6	0.667	Promoter(<=1kb)	178	OR4C46	119749
379890	11q	58214757	58215722	966	6	0.333	Promoter(<=1kb)	12	OR1S1	219959
379890	11q	58402523	58403265	743	8	0.5	Promoter(<=1kb)	144	OR5B3	441608
379890	11q	64116513	64118232	1720	7	0.714	Exon(exon2of2)	8702	MACROD1	28992
379890	11q	64315797	64315856	60	8	0.75	Promoter(1-2kb)	1485	TRMT112	51504
379890	11q	71796106	71796215	110	7	0.429	3'UTR	8425	ALG1L9P	285407
379890	11q	85724687	85725825	1139	6	0.5	Promoter(<=1kb)	0	SYTL2	54843
379890	11q	124015600	124016477	878	10	0.3	Promoter(<=1kb)	25	OR10G4	390264
379890	11q	124023038	124023849	812	10	0.4	Promoter(<=1kb)	25	OR10G9	219870
379890	11q	124185670	124186080	411	6	0.333	Promoter(<=1kb)	454	OR10D3	26497
379890	11q	130914501	130915409	909	10	0.7	Promoter(1-2kb)	1035	SNX19	399979
379890	12p	4626571	4628549	1979	8	0.5	Exon(exon5of6)	14054	DYRK4	8798
379890	12p	6018369	6019277	909	6	0.333	Promoter(2-3kb)	2649	VWF	7450
379890	12p	6453119	6453670	552	6	0.667	Promoter(<=1kb)	633	TAPBPL	55080
379890	12p	8222174	8224062	1889	7	0.714	Exon(exon4of6)	3525	FAM90A1	55138
379890	12q	49795037	49796884	1848	6	0.5	Promoter(<=1kb)	118	NCKAP5L	57701
379890	13q	25096659	25097182	524	24	0.583	Promoter(<=1kb)	791	PABPC3	5042
379890	13q	102732474	102733933	1460	6	0.333	Exon(exon4of4)	25139	CCDC168	643677
379890	14q	20060048	20060884	837	8	0.625	Promoter(<=1kb)	3	OR4L1	122742
379890	14q	20640982	20641567	586	6	0.5	Promoter(<=1kb)	124	OR6S1	341799
379890	14q	21634137	21634589	453	9	0.556	Promoter(<=1kb)	351	OR10G2	26534
379890	14q	44504986	44506403	1418	6	0.667	Promoter(<=1kb)	880	FSCB	84075
379890	14q	70457532	70458540	1009	9	0.333	Exon(exon2of2)	5358	ADAM21	8747
379890	14q	104175195	104177810	2616	7	0.286	Exon(exon12of15)	36155	KIF26A	26153
379890	14q	104939262	104942618	3357	11	0.182	5'UTR	7102	PLD4	122618
379890	14q	104943622	104945444	1823	7	0.571	Exon(exon6of6)	9958	AHNAK2	113146
379890	14q	104947901	104949883	1983	17	0.647	Exon(exon6of6)	5519	AHNAK2	113146
379890	15q	20534501	20535014	514	6	0.5	Exon(exon8of9)	6786	GOLGA6L6	727832
379890	15q	23363730	23365045	1316	7	0.571	Exon(exon14of18)	8884	GOLGA8S	653061
379890	15q	23439979	23442067	2089	14	0.5	5'UTR	5167	GOLGA6L2	283685
379890	15q	85579423	85581800	2378	14	0.571	Promoter(1-2kb)	-1110	AKAP13	11214
379890	15q	88854874	88855594	721	6	0.833	Exon(exon12of18)	7631	ACAN	176
379890	15q	88857108	88859365	2258	6	0	Exon(exon12of18)	9865	ACAN	176

379890	15q	100569472	100570097	626	6	0.833	Promoter(<=1kb)	534	LINS1	55180
379890	16p	1228744	1229731	988	10	0.7	Promoter(<=1kb)	431	TPSB2	64499
379890	16p	1256345	1256985	641	14	0.786	Promoter(<=1kb)	276	TPSD1	23430
379890	16p	1486190	1488463	2274	9	0.778	Promoter(<=1kb)	4	PTX4	390667
379890	16p	4883938	4885635	1698	6	1	Exon(exon22of22)	4701	PPL	5493
379890	16q	88714717	88717113	2397	6	0.5	Promoter(<=1kb)	0	MIR4722	100616167
379890	16q	89226863	89228289	1427	7	0.571	Promoter(2-3kb)	2229	ZNF778	197320
379890	17p	21300581	21300978	398	12	0.75	3'UTR	9112	MAP2K3	5606
379890	17q	81645135	81645595	461	7	0.429	Promoter(2-3kb)	2722	TSPAN10	83882
379890	18p	11609728	11610383	656	7	1	Promoter(<=1kb)	132	SLC35G4	646000
379890	18p	11644365	11644734	370	9	0.778	Exon(exon1of1)	10207	MIR7153	102465690
379890	18q	58535186	58537515	2330	10	0.4	Promoter(<=1kb)	0	ALPK2	115701
379890	19p	1004711	1005532	822	7	0.571	Exon(exon3of9)	4292	GRIN3B	116444
379890	19p	1036457	1036914	458	8	0.625	Exon(exon6of7)	-3187	ABCA7	10347
379890	19p	4510548	4513547	3000	18	0.5	Exon(exon3of6)	4157	PLIN4	729359
379890	19p	5455600	5456439	840	6	0.5	Promoter(<=1kb)	183	ZNRF4	148066
379890	19p	8937644	8939234	1591	6	0.667	Exon(exon5of84)	-41554	MUC16	94025
379890	19p	8946313	8951868	5556	19	0.632	Exon(exon3of84)	29474	MUC16	94025
379890	19p	8959116	8962299	3184	11	0.727	Exon(exon3of84)	19043	MUC16	94025
379890	19p	8971838	8978096	6259	15	0.533	Exon(exon1of84)	3246	MUC16	94025
379890	19p	12430157	12432437	2281	11	0.364	3'UTR	8584	ZNF443	10224
379890	19p	15087213	15088040	828	10	0.3	Promoter(<=1kb)	233	OR1I1	126370
379890	19p	18264798	18267409	2612	12	0.583	5'UTR	7002	IQCN	80726
379890	19p	21971930	21974500	2571	7	0.714	Exon(exon4of4)	14408	ZNF208	7757
379890	19q	39877222	39877880	659	6	0.5	Exon(exon20of28)	9412	FCGBP	8857
379890	19q	40880231	40880622	392	6	0.333	Promoter(<=1kb)	-141	CYP2A7	1549
379890	19q	43203948	43205504	1557	6	0.667	Promoter(<=1kb)	0	PSG4	5672
379890	19q	43913423	43914878	1456	8	0.5	Exon(exon10of10)	4861	ZNF45	7596
379890	19q	44106512	44108078	1567	7	0	Exon(exon6of6)	-4103	ZNF225	7768
379890	19q	51745958	51746963	1006	6	0.5	Exon(exon3of3)	3848	FPR1	2357
379890	19q	52437918	52439242	1325	7	0.429	Exon(exon4of4)	6504	ZNF534	147658
379890	19q	53108623	53109736	1114	6	0.167	5'UTR	-5187	ZNF160	90338
379890	19q	55911888	55913166	1279	7	0.714	Exon(exon5of12)	19145	NLRP13	126204
379890	19q	56190954	56193030	2077	6	1	Promoter(<=1kb)	22	ZSCAN5B	342933
379890	19q	58368293	58368875	583	7	0.429	Exon(exon3of3)	-5445	ZNF497	162968
379890	20p	1635288	1636423	1136	6	0.333	Promoter(1-2kb)	1662	SIRPG-AS1	101929010
379890	20p	5922677	5923382	706	7	0.571	Exon(exon4of5)	7179	CHGB	1114
379890	20p	20052354	20052736	383	6	0.167	Promoter(<=1kb)	0	CFAP61	26074
379890	20q	59191988	59194085	2098	6	0.667	Promoter(<=1kb)	968	ZNF831	128611
379890	20q	63561666	63565531	3866	14	0.643	Promoter(<=1kb)	-61	HELZ2	85441
379890	21q	26843740	26844859	1120	6	0.667	Promoter(<=1kb)	0	ADAMTS1	9510
379890	21q	41740862	41741990	1129	6	0.833	Exon(exon2of2)	4851	MIR6814	102465488
379890	22q	22352950	22353380	431	16	0.5	Exon(exon1of2)	30478	BMS1P20	96610
379890	22q	22646443	22647152	710	7	0.286	Promoter(<=1kb)	0	GGTLC2	91227
379890	22q	49884187	49884994	808	6	0.333	Exon(exon2of2)	22924	ALG12	79087
379890	23p	8170039	8170141	103	6	0.5	Promoter(1-2kb)	1126	VCX2	51480
381208	1p	978953	980295	1343	6	0.5	Promoter(<=1kb)	734	PERM1	84808
381208	1p	16058491	16060000	1510	11	0.818	Exon(5of7)	6168	CLCNKB	1188
381208	1p	18481403	18483159	1757	7	0.714	Promoter(<=1kb)	421	KLHDC7A	127707
381208	1p	23874604	23875430	827	8	0.5	Exon(2of2)	-6310	FUCA1	2517
381208	1p	40067594	40067675	82	6	0	Promoter(<=1kb)	324	CAP1	10487
381208	1p	89186388	89186419	32	9	0.556	Promoter(<=1kb)	107	GBP4	115361
381208	1q	152213286	152213347	62	8	0.5	Exon(3of3)	10846	HRNR	388697
381208	1q	152219233	152221375	2143	17	0.706	Promoter(2-3kb)	2818	HRNR	388697
381208	1q	152303673	152313891	10219	33	0.606	Promoter(<=1kb)	0	FLG-AS1	339400
381208	1q	158765805	158766655	851	6	0.5	Promoter(<=1kb)	47	OR6N1	128372
381208	1q	201206099	201209342	3244	9	0.667	Promoter(1-2kb)	1017	IGFN1	91156
381208	1q	214640144	214642881	2738	9	0.444	Exon(12of20)	-5086	CENPF	1063
381208	1q	214644880	214647181	2302	7	0.286	Promoter(<=1kb)	-786	CENPF	1063
381208	1q	222628664	222629929	1266	6	0.333	Promoter(<=1kb)	190	MIA3	375056
381208	1q	247841312	247841582	271	6	0.833	Promoter(<=1kb)	314	OR11L1	391189
381208	1q	247949443	247949738	296	9	0.333	Promoter(<=1kb)	585	OR2L8	391190
381208	2p	29002636	29003646	1011	6	0.333	Exon(5of20)	-10821	TOGARAM2	165186
381208	2p	29071763	29073000	1238	6	0.667	Promoter(1-2kb)	1523	PCARE	388939
381208	2p	48580657	48582454	1798	7	0.571	Promoter(<=1kb)	0	STON1	11037
381208	2q	130914528	130917154	2627	8	0.5	Promoter(<=1kb)	0	ARHGEF4	50649
381208	2q	132783032	132785012	1981	7	0.571	Promoter(1-2kb)	-1009	NCKAP5	344148
381208	2q	185789865	185794632	4768	10	0.8	Promoter(<=1kb)	0	FSIP2	401024
381208	2q	185805377	185808170	2794	6	0.333	Promoter(<=1kb)	0	FSIP2	401024
381208	2q	233713134	233713664	531	8	0.75	Promoter(<=1kb)	142	UGT1A5	54579
381208	2q	238130271	238131546	1276	7	0.286	Promoter(1-2kb)	1323	ESPNL	339768
381208	2q	240041845	240042180	336	7	0.286	Downstream(2-3kb)	3892	OR6B3	150681
381208	3p	52520288	52524117	3830	7	0.429	Promoter(<=1kb)	0	STAB1	23166
381208	3p	75736880	75739243	2364	66	0.606	Promoter(<=1kb)	0	MIR4273	100422955
381208	3q	98264413	98265137	725	8	0.625	Promoter(<=1kb)	128	OR5H6	79295
381208	4p	5988383	5989749	1367	7	0.571	Promoter(<=1kb)	0	C4orf50	389197
381208	4p	6300792	6302360	1569	6	0.833	Exon(8of8)	6021	WFS1	7466

381208	4q	87811379	87811766	388	6	0.333	Exon(7of7)	-9645	MEPE	56955
381208	4q	121036404	121037542	1139	6	0.333	Promoter(1-2kb)	1442	NDNF	79625
381208	5q	79728956	79730716	1761	7	0.286	Exon(2of13)	-7426	CMYA5	202333
381208	5q	79731782	79734523	2742	13	0.308	Exon(2of13)	-3619	CMYA5	202333
381208	5q	140807352	140807737	386	6	0.833	Promoter(<=1kb)	271	PCDHA4	56144
381208	5q	140848579	140850786	2208	7	0.571	Promoter(<=1kb)	807	PCDHA9	9752
381208	5q	141174000	141175025	1026	6	0.833	Promoter(1-2kb)	1356	PCDHB7	56129
381208	5q	141183999	141184688	690	6	0.833	Promoter(2-3kb)	-2473	PCDHB9	56127
381208	5q	141187690	141189425	1736	6	0.5	Promoter(<=1kb)	529	PCDHB9	56127
381208	5q	141955356	141957660	2305	6	0.667	Promoter(<=1kb)	-668	RNF14	9604
381208	5q	151521550	151522069	520	6	0.667	Promoter(<=1kb)	79	MIR6499	102465246
381208	5q	151565922	151568158	2237	9	0.778	Promoter(<=1kb)	786	FAT2	2196
381208	5q	177209881	177210867	987	6	0.833	Promoter(1-2kb)	-1244	NSD1	64324
381208	6p	1312843	1313745	903	6	0.5	Promoter(<=1kb)	745	FOXQ1	94234
381208	6p	16327099	16327684	586	6	0.333	Exon(8of9)	37154	GMPR	2766
381208	6p	42745312	42746041	730	6	0.667	Promoter(<=1kb)	62	TBCC	6903
381208	6p	46858771	46859502	732	8	0.5	Exon(17of21)	3802	ADGRF5	221395
381208	6q	159231899	159234370	2472	12	0.583	Exon(11of23)	13602	FNDC1	84624
381208	7p	12369637	12370736	1100	6	0.667	3'UTR	-13307	VWDE	221806
381208	7p	45082865	45084866	2002	7	0.429	Promoter(1-2kb)	-1465	NACAD	23148
381208	7q	100958977	100960873	1897	52	0.442	Promoter(1-2kb)	1012	MUC3A	4584
381208	7q	100993912	100995922	2011	13	0.692	Exon(5of15)	-17132	MUC12	10071
381208	7q	101034361	101040583	6223	37	0.514	Exon(3of12)	-3128	MUC17	140453
381208	7q	149818015	149819792	1778	7	0.714	Promoter(2-3kb)	-2352	SSPO	23145
381208	8p	8376561	8377220	660	6	1	Exon(2of5)	4527	PRAG1	157285
381208	8p	11331194	11332026	833	8	0.625	Promoter(<=1kb)	306	SLC35G5	83650
381208	8p	12132477	12133940	1464	10	0.7	Promoter(<=1kb)	498	USP17L7	392197
381208	8p	13021128	13022030	903	8	0.125	Exon(5of5)	9115	TRMT9B	57604
381208	8q	123651655	123652634	980	7	0.571	Promoter(<=1kb)	316	KLHL38	340359
381208	8q	141466455	141467514	1060	8	0.75	3'UTR	29245	MROH5	389690
381208	9p	39078723	39078846	124	6	0.667	Exon(22of24)	7302	CNTNAP3	79937
381208	9q	76705724	76707804	2081	6	0.5	Promoter(<=1kb)	666	PCA3	50652
381208	9q	104598545	104599361	817	12	0.583	Promoter(<=1kb)	52	OR13C5	138799
381208	9q	122553278	122554071	794	7	0.429	Promoter(<=1kb)	108	OR1N2	138882
381208	9q	122628595	122629398	804	6	0.333	Promoter(<=1kb)	175	OR1B1	347169
381208	9q	122749914	122750547	634	6	0.833	Promoter(<=1kb)	174	OR1L6	392390
381208	10q	46549378	46550723	1346	26	0.654	Exon(3of3)	4807	GPRIN2	9721
381208	10q	49322575	49325572	2998	8	0.5	Exon(3of3)	23301	C10orf71	118461
381208	11p	244106	244197	92	8	0.5	Promoter(<=1kb)	-232	PSMD13	5719
381208	11p	1194354	1196902	2549	7	0.571	Exon(34of49)	-26164	MUC5B	727897
381208	11p	1246332	1247378	1047	6	0.5	Promoter(2-3kb)	2298	MUC5B-AS1	112577518
381208	11p	5177811	5178478	668	7	0.143	Promoter(<=1kb)	186	OR52Z1	283110
381208	11p	5323451	5324256	806	6	0.5	Promoter(<=1kb)	41	OR51B2	79345
381208	11p	5351521	5352416	896	17	0.529	Promoter(<=1kb)	13	OR51B6	390058
381208	11p	5389704	5390350	647	7	0.429	Promoter(<=1kb)	327	OR51M1	390059
381208	11p	5422212	5423123	912	11	0.727	Promoter(<=1kb)	101	OR51Q1	390061
381208	11p	5515185	5515931	747	6	0.333	Promoter(<=1kb)	768	UBQLNL	143630
381208	11p	5581045	5581738	694	8	0.375	Promoter(<=1kb)	168	OR52B6	340980
381208	11p	5841302	5841883	582	9	0.333	Promoter(<=1kb)	14	OR52E6	390078
381208	11p	5884818	5885061	244	7	0.429	Promoter(<=1kb)	547	OR52E4	390081
381208	11p	11351961	11352736	776	7	0.143	Promoter(<=1kb)	514	CSNK2A3	283106
381208	11p	12293639	12294842	1204	7	0.714	Exon(29of35)	6739	MICALCL	84953
381208	11p	43942293	43943348	1056	9	0.778	Promoter(<=1kb)	0	C11orf96	387763
381208	11q	58214757	58215722	966	8	0.25	Promoter(<=1kb)	12	OR1S1	219959
381208	11q	58402523	58403265	743	8	0.5	Promoter(<=1kb)	144	OR5B3	441608
381208	11q	64116513	64118232	1720	6	0.667	Exon(2of2)	8702	MACROD1	28992
381208	11q	82732630	82733184	555	6	0.833	Promoter(<=1kb)	680	FAM181B	220382
381208	11q	85724687	85725825	1139	6	0.5	Promoter(<=1kb)	0	SYTL2	54843
381208	11q	101961859	101963037	1179	6	0.667	3'UTR	-15365	CEP126	57562
381208	11q	123906790	123907324	535	6	0.667	Promoter(<=1kb)	644	OR8D4	338662
381208	11q	124015600	124016477	878	7	0.143	Promoter(<=1kb)	25	OR10G4	390264
381208	11q	124038366	124038988	623	6	0.833	Promoter(<=1kb)	13	OR10G7	390265
381208	11q	124382526	124383285	760	8	0.625	Promoter(<=1kb)	58	OR8B2	26595
381208	11q	130914501	130915409	909	10	0.7	Promoter(1-2kb)	1035	SNX19	399979
381208	12p	4626568	4627524	957	7	0.429	Exon(5of6)	14051	DYRK4	8798
381208	12p	6453119	6453670	552	6	0.667	Promoter(<=1kb)	633	TAPBPL	55080
381208	12q	49795819	49797223	1405	6	0.5	Promoter(<=1kb)	0	NCKAP5L	57701
381208	13q	25096730	25097072	343	13	0.615	Promoter(<=1kb)	862	PABPC3	5042
381208	13q	102732474	102733933	1460	6	0.333	Exon(4of4)	25139	CCDC168	643677
381208	14q	19935750	19936577	828	6	0.833	Promoter(<=1kb)	142	OR4K1	79544
381208	14q	20060048	20060884	837	8	0.625	Promoter(<=1kb)	3	OR4L1	122742
381208	14q	20640750	20641567	818	6	0.5	Promoter(<=1kb)	124	OR6S1	341799
381208	14q	21634137	21634589	453	9	0.556	Promoter(<=1kb)	351	OR10G2	26534
381208	14q	70457520	70458948	1429	13	0.385	Exon(2of2)	5346	ADAM21	8747
381208	15q	20534480	20535014	535	7	0.429	Exon(8of9)	6786	GOLGA6L6	727832
381208	15q	23439979	23442067	2089	10	0.6	5'UTR	5167	GOLGA6L2	283685
381208	15q	40621642	40624264	2623	7	0.429	Promoter(<=1kb)	0	KNL1	57082

381208	15q	85579423	85582073	2651	15	0.6	Promoter(<=1kb)	-837	AKAP13	11214
381208	15q	88854874	88855594	721	6	0.833	Exon(12of18)	7631	ACAN	176
381208	15q	88857108	88859365	2258	6	0	Exon(12of18)	9865	ACAN	176
381208	15q	99129423	99132517	3095	6	0.333	Exon(4of5)	7225	TTC23	64927
381208	16p	1486322	1488463	2142	11	0.636	Promoter(<=1kb)	4	PTX4	390667
381208	16p	28495551	28497395	1845	6	0.333	Promoter(<=1kb)	0	CLN3	1201
381208	16q	88428539	88429600	1062	7	0.429	Exon(3of3)	-23680	ZFPM1	161882
381208	16q	88430623	88433932	3310	9	0.667	Exon(3of3)	-19348	ZFPM1	161882
381208	16q	89100686	89101050	365	7	0.571	Promoter(<=1kb)	24	ACSF3	197322
381208	16q	89226863	89228289	1427	8	0.625	Promoter(2-3kb)	2229	ZNF778	197320
381208	17p	10638198	10641099	2902	7	0.286	Exon(19of41)	-8169	MYH3	4621
381208	17p	21300581	21300954	374	8	0.75	3'UTR	9112	MAP2K3	5606
381208	17p	21415470	21416431	962	10	0.7	Exon(3of3)	10334	KCNJ12	3768
381208	17q	81645135	81645417	283	6	0.333	Promoter(2-3kb)	2722	TSPAN10	83882
381208	18p	11609646	11610509	864	16	0.688	Promoter(<=1kb)	50	SLC35G4	646000
381208	18q	58535186	58537257	2072	8	0.375	Promoter(<=1kb)	0	ALPK2	115701
381208	19p	1004711	1005532	822	7	0.571	Exon(3of9)	4292	GRIN3B	116444
381208	19p	4510548	4513547	3000	13	0.385	Exon(3of6)	4157	PLIN4	729359
381208	19p	5455600	5456439	840	8	0.625	Promoter(<=1kb)	183	ZNRF4	148066
381208	19p	12075333	12077046	1714	7	0.143	Promoter(<=1kb)	6	ZNF788P	388507
381208	19p	12430157	12431840	1684	9	0.444	3'UTR	9181	ZNF443	10224
381208	19p	14841061	14841748	688	9	0.444	Promoter(<=1kb)	129	OR7A10	390892
381208	19p	18264798	18267409	2612	12	0.583	5'UTR	7002	IQCN	80726
381208	19p	21971930	21974500	2571	10	0.7	Exon(4of4)	14408	ZNF208	7757
381208	19p	22663799	22665021	1223	6	0.667	Exon(4of4)	29475	ZNF492	57615
381208	19p	22756205	22759523	3319	13	0.615	3'UTR	10459	ZNF99	7652
381208	19q	36996730	36997597	868	10	0.7	Exon(10of10)	5677	ZNF568	374900
381208	19q	39886005	39886439	435	6	0.333	Promoter(<=1kb)	853	FCGBP	8857
381208	19q	43913423	43914878	1456	8	0.5	Exon(10of10)	4861	ZNF45	7596
381208	19q	44106512	44108078	1567	7	0	Exon(6of6)	-4103	ZNF225	7768
381208	19q	44327836	44329698	1863	6	0.5	Exon(4of4)	-22790	ZNF235	9310
381208	19q	48873325	48875925	2601	11	0.545	Promoter(<=1kb)	904	PPP1R15A	23645
381208	19q	52437918	52439242	1325	7	0.429	Exon(4of4)	6504	ZNF534	147658
381208	19q	55481625	55483456	1832	7	0.429	Promoter(1-2kb)	-1732	NAT14	57106
381208	19q	55517821	55518642	822	6	1	Exon(14of14)	17661	SBK2	646643
381208	19q	55911888	55913166	1279	7	0.714	Exon(5of12)	19145	NLRP13	126204
381208	19q	58368293	58368875	583	7	0.429	Exon(3of3)	-5445	ZNF497	162968
381208	20p	20052354	20052736	383	6	0	Promoter(<=1kb)	0	CFAP61	26074
381208	20q	63349752	63350772	1021	6	0.5	3'UTR	3794	CHRNA4	1137
381208	20q	63561666	63565531	3866	11	0.636	Promoter(<=1kb)	-61	HELZ2	85441
381208	21q	44550835	44551416	582	6	0.833	Promoter(<=1kb)	89	KRTAP10-2	386679
381208	22q	22352950	22353348	399	14	0.571	Exon(1of2)	30478	BMS1P20	96610
381208	22q	22758758	22759209	452	6	0.833	Exon(1of2)	-63567	MIR650	723778
381208	22q	36191154	36191906	753	7	0.714	3'UTR	9971	APOL4	80832
381208	22q	39100331	39102033	1703	6	0.833	Promoter(<=1kb)	52	APOBEC3H	164668
381208	23p	8170039	8170141	103	6	0.5	Promoter(1-2kb)	1126	VCX2	51480
381208	23p	35802148	35803010	863	7	0.571	5'UTR	3357	MAGEB16	139604
381208	23q	101494051	101494598	548	8	0.625	Exon(2of2)	6007	ARMCX4	100131755
381888	1p	12847526	12847995	470	10	0.4	Promoter(<=1kb)	730	HNRNPCL1	343069
381888	1p	12859036	12860212	1177	14	0.5	Promoter(1-2kb)	1950	PRAMEF2	65122
381888	1p	12861255	12861478	224	6	0.333	Exon(exon4of4)	4169	PRAMEF2	65122
381888	1p	12893249	12893472	224	6	0.667	Exon(exon4of4)	4798	PRAMEF10	343071
381888	1p	13369166	13369564	399	10	0.8	Promoter(2-3kb)	2336	PRAMEF19	645414
381888	1p	13370686	13371119	434	9	0.444	Promoter(<=1kb)	781	PRAMEF19	645414
381888	1p	16058491	16060000	1510	10	0.9	Exon(exon5of7)	6168	CLCNKB	1188
381888	1p	18481403	18482217	815	7	0.714	Promoter(<=1kb)	421	KLHDC7A	127707
381888	1p	23874604	23875430	827	8	0.5	Exon(exon2of2)	-6310	FUCA1	2517
381888	1p	26343953	26345408	1456	6	0.833	Exon(exon4of22)	-4607	CRYBG2	55057
381888	1p	40067594	40067675	82	6	0	Promoter(<=1kb)	324	CAP1	10487
381888	1p	62273232	62275080	1849	7	0.429	Promoter(<=1kb)	-441	KANK4	163782
381888	1q	152213286	152213347	62	8	0.5	Exon(exon3of3)	10846	HRNR	388697
381888	1q	152219233	152221375	2143	16	0.75	Promoter(2-3kb)	2818	HRNR	388697
381888	1q	183647749	183648558	810	7	0.429	Exon(exon2of2)	4758	APOBEC4	403314
381888	1q	197101312	197101771	460	6	0.5	Exon(exon18of28)	33373	ASPM	259266
381888	1q	201206099	201209849	3751	12	0.583	Promoter(1-2kb)	1017	IGFN1	91156
381888	1q	222628664	222629985	1322	6	0.167	Promoter(<=1kb)	190	MIA3	375056
381888	1q	228315976	228318049	2074	7	0.571	Exon(exon50of81)	6492	OBSCN	84033
381888	1q	247841312	247841582	271	6	0.833	Promoter(<=1kb)	314	OR11L1	391189
381888	1q	247949443	247949738	296	10	0.3	Promoter(<=1kb)	585	OR2L8	391190
381888	1q	248294677	248295415	739	6	0.667	Promoter(<=1kb)	185	OR2T12	127064
381888	2p	48580657	48582454	1798	7	0.571	Promoter(<=1kb)	0	STON1	11037
381888	2q	130193975	130194376	402	7	0.571	Exon(exon4of5)	4063	TUBA3E	112714
381888	2q	132783534	132785001	1468	6	0.333	Promoter(1-2kb)	-1511	NCKAP5	344148
381888	2q	167246794	167248478	1685	7	0.714	Promoter(<=1kb)	-204	XIRP2	129446
381888	2q	184936178	184937636	1459	6	0.333	Exon(exon4of4)	69813	ZNF804A	91752
381888	2q	217847583	217848559	977	6	0.833	Exon(exon19of33)	-5423	TNS1	7145
381888	2q	233713134	233713783	650	13	0.692	Promoter(<=1kb)	142	UGT1A5	54579

381888	2q	240041845	240042811	967	8	0.25	Downstream(2-3kb)	3261	OR6B3	150681
381888	3p	75736880	75739243	2364	70	0.643	Promoter(<=1kb)	0	MIR4273	100422955
381888	3q	98264413	98265098	686	7	0.571	Promoter(<=1kb)	128	OR5H6	79295
381888	3q	194341097	194342571	1475	7	0.714	Exon(exon2of2)	8747	CPN2	1370
381888	4p	5988383	5989749	1367	7	0.571	Promoter(<=1kb)	0	C4orf50	389197
381888	4p	6300792	6302360	1569	7	0.857	Exon(exon8of8)	6021	WFS1	7466
381888	4p	10443803	10446224	2422	7	0.429	Exon(exon3of3)	10952	ZNF518B	85460
381888	4q	112431241	112432293	1053	7	0.429	3'UTR	4735	ALPK1	80216
381888	4q	185458217	185460011	1795	8	0.625	Promoter(<=1kb)	0	CCDC110	256309
381888	4q	186617176	186621582	4407	11	0.364	Exon(exon10of27)	-7106	FAT1	2195
381888	5q	140807352	140807737	386	6	0.833	Promoter(<=1kb)	271	PCDHA4	56144
381888	5q	140848579	140850786	2208	7	0.571	Promoter(<=1kb)	807	PCDHA9	9752
381888	5q	141174000	141175025	1026	6	0.833	Promoter(1-2kb)	1356	PCDHB7	56129
381888	5q	141187690	141189425	1736	7	0.571	Promoter(<=1kb)	529	PCDHB9	56127
381888	5q	151521550	151522069	520	6	0.667	Promoter(<=1kb)	79	MIR6499	102465246
381888	5q	151565922	151568158	2237	9	0.778	Promoter(<=1kb)	786	FAT2	2196
381888	6p	1312843	1313745	903	6	0.5	Promoter(<=1kb)	745	FOXQ1	94234
381888	6p	46858771	46859389	619	6	0.5	Exon(exon17of21)	3915	ADGRF5	221395
381888	6q	64591274	64591961	688	10	0.5	Exon(exon26of43)	121374	EYS	346007
381888	6q	149888581	149889987	1407	6	0.667	Promoter(<=1kb)	-116	RAET1E-AS1	100652739
381888	6q	159231899	159234370	2472	12	0.583	Exon(exon11of23)	13602	FNDC1	84624
381888	7p	6330446	6330944	499	6	1	Exon(exon2of2)	7749	FAM220A	84792
381888	7p	12369637	12370736	1100	6	0.667	3'UTR	-13307	VWDE	221806
381888	7p	38353718	38353991	274	8	0.625	Exon(exon2of2)	3699	TRG-AS1	100506776
381888	7q	100958721	100960873	2153	57	0.456	Promoter(<=1kb)	756	MUC3A	4584
381888	7q	100991195	100994184	2990	12	0.75	Exon(exon5of15)	-18870	MUC12	10071
381888	7q	100995547	100995785	239	7	0.857	Exon(exon5of15)	-17269	MUC12	10071
381888	7q	101003685	101004836	1152	6	0.667	Exon(exon5of15)	-8218	MUC12	10071
381888	7q	149818015	149819792	1778	6	0.667	Promoter(2-3kb)	-2352	SSPO	23145
381888	8p	8376561	8377198	638	6	1	Exon(exon2of5)	4549	PRAG1	157285
381888	8p	10607245	10608261	1017	8	0.625	Exon(exon4of4)	46882	RP1L1	94137
381888	8p	10609650	10612307	2658	8	0.875	Exon(exon4of4)	42836	RP1L1	94137
381888	8p	11331234	11332082	849	6	0.667	Promoter(<=1kb)	346	SLC35G5	83650
381888	8p	12132686	12133940	1255	6	0.667	Promoter(<=1kb)	498	USP17L7	392197
381888	8p	13021128	13022030	903	8	0.25	Exon(exon5of5)	9115	TRMT9B	57604
381888	8q	105801290	105803289	2000	6	0.5	Promoter(2-3kb)	-2881	ZFPM2-AS1	102723356
381888	8q	123651655	123652634	980	7	0.571	Promoter(<=1kb)	316	KLHL38	340359
381888	9p	116800	117428	629	6	0.833	Promoter(<=1kb)	776	FOXD4	2298
381888	9q	76175237	76175300	64	14	0.714	Exon(exon14of14)	-13339	PCSK5	5125
381888	9q	76703555	76707804	4250	13	0.462	Promoter(<=1kb)	0	PCA3	50652
381888	9q	76709263	76710843	1581	9	0.556	Promoter(<=1kb)	0	PRUNE2	158471
381888	9q	104504315	104505071	757	6	0.5	Promoter(<=1kb)	52	OR13F1	138805
381888	9q	104598545	104599361	817	12	0.583	Promoter(<=1kb)	52	OR13C5	138799
381888	9q	122553263	122554071	809	7	0.429	Promoter(<=1kb)	93	OR1N2	138882
381888	9q	122628595	122629130	536	6	0.333	Promoter(<=1kb)	443	OR1B1	347169
381888	9q	122749914	122750547	634	6	0.833	Promoter(<=1kb)	174	OR1L6	392390
381888	9q	135484803	135487213	2411	9	0.333	Promoter(1-2kb)	1440	PPP1R26	9858
381888	10q	46549378	46550723	1346	26	0.654	Exon(exon3of3)	4807	GPRIN2	9721
381888	10q	49323169	49326817	3649	11	0.545	Exon(exon3of3)	23895	C10orf71	118461
381888	10q	128102594	128105328	2735	7	0.714	Promoter(<=1kb)	0	MKI67	4288
381888	11p	244106	244197	92	8	0.5	Promoter(<=1kb)	-232	PSMD13	5719
381888	11p	5177978	5178478	501	6	0.167	Promoter(<=1kb)	186	OR52Z1	283110
381888	11p	5323362	5324256	895	7	0.429	Promoter(<=1kb)	41	OR51B2	79345
381888	11p	5422212	5423123	912	11	0.636	Promoter(<=1kb)	101	OR51Q1	390061
381888	11p	5581045	5581738	694	8	0.375	Promoter(<=1kb)	168	OR52B6	340980
381888	11p	5841302	5841883	582	9	0.333	Promoter(<=1kb)	14	OR52E6	390078
381888	11p	11351961	11352736	776	9	0.222	Promoter(<=1kb)	514	CSNK2A3	283106
381888	11p	12293639	12294368	730	6	0.833	Exon(exon29of35)	6739	MICALCL	84953
381888	11p	18173280	18173901	622	6	0.333	Promoter(<=1kb)	443	MRGPRX4	117196
381888	11q	58214757	58215722	966	8	0.25	Promoter(<=1kb)	12	OR1S1	219959
381888	11q	58402523	58403265	743	8	0.5	Promoter(<=1kb)	144	OR5B3	441608
381888	11q	64315797	64315856	60	8	0.75	Promoter(1-2kb)	1485	TRMT112	51504
381888	11q	85724687	85725825	1139	6	0.5	Promoter(<=1kb)	0	SYTL2	54843
381888	11q	124015600	124016477	878	10	0.3	Promoter(<=1kb)	25	OR10G4	390264
381888	11q	124023038	124023849	812	10	0.4	Promoter(<=1kb)	25	OR10G9	219870
381888	12p	4626568	4627524	957	7	0.429	Exon(exon5of6)	14051	DYRK4	8798
381888	12p	6453119	6453670	552	6	0.667	Promoter(<=1kb)	633	TAPBPL	55080
381888	12p	8222174	8223514	1341	7	0.714	Exon(exon5of6)	4073	FAM90A1	55138
381888	12p	31981306	31982507	1202	6	0.5	Exon(exon4of6)	-4659	RESF1	55196
381888	12q	48525773	48526223	451	6	0.333	Promoter(<=1kb)	141	OR8S1	341568
381888	13q	24434450	24435347	898	8	0.625	Exon(exon31of34)	19787	PARP4	143
381888	13q	25096713	25097182	470	31	0.548	Promoter(<=1kb)	845	PABPC3	5042
381888	13q	102732474	102733933	1460	6	0.333	Exon(exon4of4)	25139	CCDC168	643677
381888	14q	19975713	19976448	736	9	0.444	Promoter(<=1kb)	269	OR4K15	81127
381888	14q	20060048	20060884	837	10	0.6	Promoter(<=1kb)	3	OR4L1	122742
381888	14q	20640982	20641567	586	6	0.5	Promoter(<=1kb)	124	OR6S1	341799
381888	14q	21634137	21634589	453	9	0.556	Promoter(<=1kb)	351	OR10G2	26534

381888	14q	70457520	70458540	1021	13	0.385	Exon(exon2of2)	5346	ADAM21	8747
381888	14q	104939262	104942618	3357	11	0.182	5'UTR	7102	PLD4	122618
381888	14q	104943622	104946886	3265	15	0.6	Exon(exon6of6)	8516	AHNAK2	113146
381888	14q	104947901	104953878	5978	32	0.531	Promoter(1-2kb)	1524	AHNAK2	113146
381888	15q	23439979	23442067	2089	13	0.462	5'UTR	5167	GOLGA6L2	283685
381888	15q	85579083	85582073	2991	17	0.588	Promoter(<=1kb)	-837	AKAP13	11214
381888	15q	99129423	99132517	3095	6	0.333	Exon(exon4of5)	7225	TTC23	64927
381888	15q	100569472	100570097	626	6	0.833	Promoter(<=1kb)	534	LINS1	55180
381888	16p	669592	672548	2957	6	0.5	Promoter(<=1kb)	0	RHOT2	89941
381888	16p	788550	790597	2048	7	0.286	Promoter(<=1kb)	0	CHTF18	63922
381888	16p	1228744	1229622	879	9	0.778	Promoter(<=1kb)	540	TPSB2	64499
381888	16p	1256345	1256896	552	7	0.714	Promoter(<=1kb)	276	TPSD1	23430
381888	16p	1486371	1488463	2093	8	0.75	Promoter(<=1kb)	4	PTX4	390667
381888	16p	4207130	4208004	875	6	0.5	Exon(exon2of7)	31739	SRL	6345
381888	16q	74391650	74392004	355	8	0.625	Exon(exon7of7)	13772	NPIPB15	440348
381888	16q	88428539	88429246	708	6	0.5	Exon(exon3of3)	-24034	ZFPM1	161882
381888	16q	88430623	88433932	3310	9	0.667	Exon(exon3of3)	-19348	ZFPM1	161882
381888	16q	89100686	89101050	365	7	0.571	Promoter(<=1kb)	24	ACSF3	197322
381888	16q	89226863	89228289	1427	7	0.571	Promoter(2-3kb)	2229	ZNF778	197320
381888	17p	744946	746966	2021	6	1	3'UTR	5072	GEMIN4	50628
381888	17p	21300581	21300954	374	8	0.75	3'UTR	9112	MAP2K3	5606
381888	17p	21415470	21416431	962	15	0.733	Exon(exon3of3)	10334	KCNJ12	3768
381888	17q	41727098	41728331	1234	6	0.833	Promoter(1-2kb)	-1201	HAP1	9001
381888	17q	76293419	76294016	598	6	0.5	Promoter(2-3kb)	-2167	QRICH2	84074
381888	17q	81645135	81645417	283	6	0.333	Promoter(2-3kb)	2722	TSPAN10	83882
381888	18p	11609646	11610491	846	14	0.714	Promoter(<=1kb)	50	SLC35G4	646000
381888	18q	58535186	58537515	2330	9	0.333	Promoter(<=1kb)	0	ALPK2	115701
381888	19p	1004688	1005532	845	6	0.5	Exon(exon3of9)	4269	GRIN3B	116444
381888	19p	4510548	4513547	3000	17	0.471	Exon(exon3of6)	4157	PLIN4	729359
381888	19p	5455600	5456439	840	7	0.571	Promoter(<=1kb)	183	ZNRF4	148066
381888	19p	8946313	8951868	5556	15	0.6	Exon(exon3of84)	29474	MUC16	94025
381888	19p	8959116	8962299	3184	10	0.7	Exon(exon3of84)	19043	MUC16	94025
381888	19p	12075333	12077046	1714	7	0.286	Promoter(<=1kb)	6	ZNF788P	388507
381888	19p	12430400	12432437	2038	9	0.444	3'UTR	8584	ZNF443	10224
381888	19p	18264798	18267409	2612	12	0.583	5'UTR	7002	IQCN	80726
381888	19p	23743906	23745300	1395	6	0.333	Exon(exon4of4)	13537	ZNF681	148213
381888	19q	36996730	36997597	868	11	0.636	Exon(exon10of10)	5677	ZNF568	374900
381888	19q	37151928	37153149	1222	6	0.833	Exon(exon5of5)	19287	ZNF585A	199704
381888	19q	37885190	37888806	3617	8	0.625	Exon(exon6of6)	17788	WDR87	83889
381888	19q	39877222	39877880	659	6	0.5	Exon(exon20of28)	9412	FCGBP	8857
381888	19q	39886022	39886439	418	6	0.5	Promoter(<=1kb)	853	FCGBP	8857
381888	19q	40880174	40880622	449	7	0.429	Promoter(<=1kb)	-84	CYP2A7	1549
381888	19q	43913423	43914878	1456	8	0.5	Exon(exon10of10)	4861	ZNF45	7596
381888	19q	44106512	44108078	1567	7	0	Exon(exon6of6)	-4103	ZNF225	7768
381888	19q	48873325	48875925	2601	11	0.545	Promoter(<=1kb)	904	PPP1R15A	23645
381888	19q	52437918	52439242	1325	7	0.429	Exon(exon4of4)	6504	ZNF534	147658
381888	19q	55481625	55483628	2004	7	0.571	Promoter(1-2kb)	-1560	NAT14	57106
381888	19q	55517821	55518176	356	7	1	Exon(exon14of14)	18127	SBK2	646643
381888	19q	58368293	58368875	583	7	0.429	Exon(exon3of3)	-5445	ZNF497	162968
381888	20p	5922421	5923394	974	8	0.5	Exon(exon4of5)	6923	CHGB	1114
381888	20q	63349752	63350772	1021	6	0.5	3'UTR	3794	CHRNA4	1137
381888	20q	63561666	63565531	3866	11	0.636	Promoter(<=1kb)	-61	HELZ2	85441
381888	21q	44637476	44638143	668	9	0.444	Promoter(<=1kb)	120	KRTAP10-10	353333
381888	22q	22352950	22353380	431	16	0.5	Exon(exon1of2)	30478	BMS1P20	96610
381888	22q	36191154	36191906	753	6	0.667	3'UTR	9971	APOL4	80832
381888	22q	36265284	36265796	513	6	1	Exon(exon6of6)	12120	APOL1	8542
381888	23p	35802148	35803010	863	7	0.571	5'UTR	3357	MAGEB16	139604
381888	23q	136347344	136349199	1856	6	0.5	Exon(exon6of26)	41323	ADGRG4	139378
381888	23q	136874183	136874347	165	7	0.714	Promoter(<=1kb)	-201	RBMX	27316
383374	1p	12847526	12847995	470	7	0.429	Promoter(<=1kb)	730	HNRNPCL1	343069
383374	1p	12859036	12860212	1177	13	0.538	Promoter(1-2kb)	1950	PRAMEF2	65122
383374	1p	13369166	13369564	399	9	0.778	Promoter(2-3kb)	2336	PRAMEF19	645414
383374	1p	13370686	13371119	434	9	0.444	Promoter(<=1kb)	781	PRAMEF19	645414
383374	1p	16058547	16060000	1454	7	0.714	Exon(exon5of7)	6224	CLCNKB	1188
383374	1p	18481403	18482217	815	6	0.667	Promoter(<=1kb)	421	KLHDC7A	127707
383374	1p	23874604	23875430	827	8	0.5	Exon(exon2of2)	-6310	FUCA1	2517
383374	1p	40067594	40067675	82	6	0	Promoter(<=1kb)	324	CAP1	10487
383374	1q	152303673	152304920	1248	8	0.5	Exon(exon3of3)	-7656	FLG-AS1	339400
383374	1q	152306380	152313891	7512	30	0.567	Promoter(<=1kb)	0	FLG-AS1	339400
383374	1q	156669844	156670886	1043	6	1	Exon(exon4of4)	6521	NES	10763
383374	1q	158765805	158766655	851	6	0.5	Promoter(<=1kb)	47	OR6N1	128372
383374	1q	169542317	169542882	566	6	0.167	Exon(exon13of25)	-26572	F5	2153
383374	1q	201206099	201209738	3640	11	0.545	Promoter(1-2kb)	1017	IGFN1	91156
383374	1q	201212089	201215147	3059	7	0.571	Promoter(<=1kb)	0	IGFN1	91156
383374	1q	226735683	226737239	1557	8	0.875	Promoter(<=1kb)	219	ITPKB	3707
383374	1q	247841312	247841582	271	6	0.833	Promoter(<=1kb)	314	OR11L1	391189
383374	1q	247949325	247949738	414	9	0.333	Promoter(<=1kb)	467	OR2L8	391190

383374	1q	248294677	248295458	782	6	0.667	Promoter(<=1kb)	142	OR2T12	127064
383374	2p	29002636	29003646	1011	6	0.333	Exon(exon5of20)	-10821	TOGARAM2	165186
383374	2q	102351547	102351902	356	7	0.429	Exon(exon11of11)	-4027	IL18R1	8809
383374	2q	184936178	184937636	1459	6	0.333	Exon(exon4of4)	69813	ZNF804A	91752
383374	2q	185790999	185794632	3634	9	0.778	Promoter(<=1kb)	0	FSIP2	401024
383374	2q	185805377	185807800	2424	6	0.333	Promoter(<=1kb)	0	FSIP2	401024
383374	2q	217847567	217848213	647	6	0.833	Exon(exon19of33)	-5407	TNS1	7145
383374	2q	238130271	238131702	1432	11	0.545	Promoter(1-2kb)	1323	ESPNL	339768
383374	2q	240041845	240042811	967	6	0.333	Downstream(2-3kb)	3261	OR6B3	150681
383374	3p	75665726	75665948	223	6	0.5	Promoter(1-2kb)	1396	FRG2C	100288801
383374	3p	75736880	75738954	2075	55	0.564	Promoter(<=1kb)	0	MIR4273	100422955
383374	3q	98264413	98265098	686	6	0.5	Promoter(<=1kb)	128	OR5H6	79295
383374	3q	194359607	194360906	1300	9	0.889	Exon(exon2of2)	-8279	CPN2	1370
383374	4p	5988383	5989749	1367	7	0.571	Promoter(<=1kb)	0	C4orf50	389197
383374	4p	6300792	6302360	1569	8	0.75	Exon(exon8of8)	6021	WFS1	7466
383374	5q	79728956	79730716	1761	7	0.286	Exon(exon2of13)	-7426	CMYA5	202333
383374	5q	79731782	79734523	2742	13	0.308	Exon(exon2of13)	-3619	CMYA5	202333
383374	5q	83537326	83539905	2580	6	0.333	Promoter(1-2kb)	1712	VCAN	1462
383374	5q	141100771	141102728	1958	8	0.375	Promoter(<=1kb)	298	PCDHB3	56132
383374	5q	141122595	141123949	1355	6	0.833	Promoter(<=1kb)	777	PCDHB4	56131
383374	5q	141174000	141175025	1026	7	0.714	Promoter(1-2kb)	1356	PCDHB7	56129
383374	5q	141187109	141189425	2317	6	0.5	Promoter(<=1kb)	0	PCDHB9	56127
383374	5q	141192663	141194472	1810	6	1	Promoter(<=1kb)	310	PCDHB10	56126
383374	5q	141955356	141957660	2305	6	0.667	Promoter(<=1kb)	-668	RNF14	9604
383374	5q	148826877	148828070	1194	6	1	Promoter(1-2kb)	1632	ADRB2	154
383374	5q	151521550	151522069	520	6	0.667	Promoter(<=1kb)	79	MIR6499	102465246
383374	5q	151565922	151568158	2237	9	0.778	Promoter(<=1kb)	786	FAT2	2196
383374	6p	1312843	1313745	903	6	0.5	Promoter(<=1kb)	745	FOXQ1	94234
383374	6p	42745312	42746041	730	6	0.667	Promoter(<=1kb)	62	TBCC	6903
383374	6p	46858771	46859502	732	8	0.5	Exon(exon17of21)	3802	ADGRF5	221395
383374	6p	56605300	56607197	1898	7	0.571	Exon(exon36of98)	-6787	DST	667
383374	6q	149888581	149890867	2287	7	0.714	Promoter(<=1kb)	0	RAET1E-AS1	100652739
383374	6q	159231899	159234370	2472	10	0.5	Exon(exon11of23)	13602	FNDC1	84624
383374	7p	56021087	56021209	123	6	0.5	Exon(exon2of7)	12947	PSPH	5723
383374	7q	64991278	64992758	1481	6	0.667	Promoter(<=1kb)	-242	ZNF117	51351
383374	7q	100958721	100960873	2153	53	0.453	Promoter(<=1kb)	756	MUC3A	4584
383374	7q	100991195	100993102	1908	8	0.625	Exon(exon5of15)	-19952	MUC12	10071
383374	7q	101034305	101038481	4177	26	0.5	Exon(exon3of12)	-5230	MUC17	140453
383374	7q	129126736	129127452	717	6	0.333	Exon(exon1of2)	-17255	TSPAN33	340348
383374	8p	10607375	10608261	887	6	0.5	Exon(exon4of4)	46882	RP1L1	94137
383374	8p	12132686	12133936	1251	9	0.667	Promoter(<=1kb)	502	USP17L7	392197
383374	8p	13021128	13022030	903	8	0.125	Exon(exon5of5)	9115	TRMT9B	57604
383374	8q	141218050	141219792	1743	6	0.667	5'UTR	8778	SLC45A4	57210
383374	8q	143916360	143919209	2850	7	0.143	Exon(exon32of32)	20381	PLEC	5339
383374	9p	34723747	34725745	1999	7	0.571	Exon(exon4of4)	3743	FAM205A	259308
383374	9q	76705724	76707804	2081	6	0.5	Promoter(<=1kb)	666	PCA3	50652
383374	9q	131474844	131476113	1270	6	0.833	Promoter(<=1kb)	0	PRRC2B	84726
383374	10q	46549378	46550723	1346	25	0.64	Exon(exon3of3)	4807	GPRIN2	9721
383374	10q	49323559	49326497	2939	9	0.444	Exon(exon3of3)	24285	C10orf71	118461
383374	10q	128102594	128105328	2735	7	0.714	Promoter(<=1kb)	0	MKI67	4288
383374	11p	244106	244197	92	8	0.5	Promoter(<=1kb)	-232	PSMD13	5719
383374	11p	1194354	1196902	2549	7	0.571	Exon(exon34of49)	-26164	MUC5B	727897
383374	11p	5177978	5178478	501	6	0.167	Promoter(<=1kb)	186	OR52Z1	283110
383374	11p	5323451	5324256	806	6	0.5	Promoter(<=1kb)	41	OR51B2	79345
383374	11p	5389704	5390350	647	7	0.429	Promoter(<=1kb)	327	OR51M1	390059
383374	11p	5422212	5423123	912	11	0.636	Promoter(<=1kb)	101	OR51Q1	390061
383374	11p	5581045	5581738	694	8	0.375	Promoter(<=1kb)	168	OR52B6	340980
383374	11p	5841302	5841883	582	9	0.333	Promoter(<=1kb)	14	OR52E6	390078
383374	11p	5884818	5885061	244	7	0.429	Promoter(<=1kb)	547	OR52E4	390081
383374	11p	11352040	11352736	697	8	0.25	Promoter(<=1kb)	514	CSNK2A3	283106
383374	11p	12293639	12294368	730	6	0.833	Exon(exon29of35)	6739	MICALCL	84953
383374	11q	58214757	58215722	966	8	0.25	Promoter(<=1kb)	12	OR1S1	219959
383374	11q	58402523	58403265	743	8	0.5	Promoter(<=1kb)	144	OR5B3	441608
383374	11q	64116513	64118232	1720	8	0.75	Exon(exon2of2)	8702	MACROD1	28992
383374	11q	85724687	85725825	1139	6	0.5	Promoter(<=1kb)	0	SYTL2	54843
383374	11q	123906790	123907324	535	6	0.667	Promoter(<=1kb)	644	OR8D4	338662
383374	11q	124015600	124016477	878	7	0.143	Promoter(<=1kb)	25	OR10G4	390264
383374	11q	124038366	124038988	623	8	1	Promoter(<=1kb)	13	OR10G7	390265
383374	11q	124382526	124383285	760	7	0.571	Promoter(<=1kb)	58	OR8B2	26595
383374	12p	4626568	4628549	1982	10	0.5	Exon(exon5of6)	14051	DYRK4	8798
383374	12p	6453119	6453670	552	7	0.714	Promoter(<=1kb)	633	TAPBPL	55080
383374	12q	52316096	52317765	1670	7	0.714	Exon(exon4of9)	3633	KRT83	3889
383374	13q	25096659	25097231	573	13	0.538	Promoter(<=1kb)	791	PABPC3	5042
383374	13q	102732474	102733933	1460	6	0.333	Exon(exon4of4)	25139	CCDC168	643677
383374	14q	20060048	20060884	837	8	0.625	Promoter(<=1kb)	3	OR4L1	122742
383374	14q	21634177	21634589	413	6	0.833	Promoter(<=1kb)	351	OR10G2	26534
383374	14q	22633879	22634450	572	9	0.333	Exon(exon2of2)	32212	ABHD4	63874

383374	14q	70457745	70458238	494	9	0.333	Exon(exon2of2)	5571	ADAM21	8747
383374	15q	23440196	23442067	1872	11	0.636	5'UTR	5167	GOLGA6L2	283685
383374	15q	40621642	40623696	2055	6	0.333	Promoter(<=1kb)	0	KNL1	57082
383374	15q	59206980	59207987	1008	8	0.625	Promoter(<=1kb)	137	LDHAL6B	92483
383374	15q	73702465	73703760	1296	6	0.833	Promoter(<=1kb)	-149	CD276	80381
383374	15q	78766033	78766626	594	6	0.333	Promoter(<=1kb)	-920	ADAMTS7	11173
383374	15q	85579423	85581800	2378	15	0.533	Promoter(1-2kb)	-1110	AKAP13	11214
383374	16p	1256354	1256985	632	11	0.636	Promoter(<=1kb)	285	TPSD1	23430
383374	16p	1998795	2000191	1397	6	0.667	Promoter(<=1kb)	0	ZNF598	90850
383374	16p	4207130	4208004	875	6	0.5	Exon(exon2of7)	31739	SRL	6345
383374	16p	28495551	28497395	1845	6	0.333	Promoter(<=1kb)	0	CLN3	1201
383374	16q	74391650	74391897	248	9	0.778	Exon(exon7of7)	13772	NPIPB15	440348
383374	16q	88428539	88431889	3351	12	0.417	Exon(exon3of3)	-21391	ZFPM1	161882
383374	16q	88712207	88714717	2511	7	0.571	Promoter(<=1kb)	0	CTU2	348180
383374	16q	89226863	89228419	1557	9	0.556	Promoter(2-3kb)	2229	ZNF778	197320
383374	17p	744946	746966	2021	6	1	3'UTR	5072	GEMIN4	50628
383374	17p	7482736	7482961	226	6	0.667	Promoter(1-2kb)	1302	ZBTB4	57659
383374	17p	10638198	10641099	2902	7	0.286	Exon(exon19of41)	-8169	MYH3	4621
383374	17p	21300581	21300954	374	8	0.75	3'UTR	9112	MAP2K3	5606
383374	17p	21415461	21416431	971	10	0.8	Exon(exon3of3)	10325	KCNJ12	3768
383374	17q	76293419	76294016	598	6	0.5	Promoter(2-3kb)	-2167	QRICH2	84074
383374	17q	81645135	81645607	473	7	0.286	Promoter(2-3kb)	2722	TSPAN10	83882
383374	18p	11609646	11610333	688	10	0.8	Promoter(<=1kb)	50	SLC35G4	646000
383374	18q	58535186	58537515	2330	9	0.333	Promoter(<=1kb)	0	ALPK2	115701
383374	19p	1004711	1005532	822	7	0.571	Exon(exon3of9)	4292	GRIN3B	116444
383374	19p	4510548	4513547	3000	14	0.429	Exon(exon3of6)	4157	PLIN4	729359
383374	19p	5455600	5456439	840	7	0.571	Promoter(<=1kb)	183	ZNRF4	148066
383374	19p	8946313	8951868	5556	16	0.625	Exon(exon3of84)	29474	MUC16	94025
383374	19p	8959116	8962299	3184	10	0.7	Exon(exon3of84)	19043	MUC16	94025
383374	19p	8972751	8978096	5346	13	0.462	Exon(exon1of84)	3246	MUC16	94025
383374	19p	12430157	12431840	1684	9	0.444	3'UTR	9181	ZNF443	10224
383374	19p	15087389	15087953	565	6	0.333	Promoter(<=1kb)	409	OR1I1	126370
383374	19p	17281820	17284246	2427	9	0.556	Promoter(<=1kb)	0	ANKLE1	126549
383374	19p	21971930	21974500	2571	10	0.7	Exon(exon4of4)	14408	ZNF208	7757
383374	19q	34943334	34944685	1352	6	0.333	3'UTR	9723	ZNF30	90075
383374	19q	37886924	37888806	1883	6	0.833	Exon(exon6of6)	17788	WDR87	83889
383374	19q	43913423	43914878	1456	9	0.556	Exon(exon10of10)	4861	ZNF45	7596
383374	19q	43996326	43997366	1041	6	0.5	Exon(exon5of5)	5419	LOC10192806:	101928063
383374	19q	52437918	52439242	1325	7	0.429	Exon(exon4of4)	6504	ZNF534	147658
383374	19q	55911888	55913166	1279	7	0.714	Exon(exon5of12)	19145	NLRP13	126204
383374	20p	5922421	5923394	974	7	0.571	Exon(exon4of5)	6923	CHGB	1114
383374	20q	63349752	63350841	1090	6	0.5	3'UTR	3725	CHRNA4	1137
383374	21q	44558207	44558709	503	6	0.5	Promoter(<=1kb)	86	KRTAP10-3	386682
383374	22q	22352950	22353348	399	14	0.571	Exon(exon1of2)	30478	BMS1P20	96610
383374	22q	36191154	36191906	753	6	0.667	3'UTR	9971	APOL4	80832
383374	22q	49883704	49884994	1291	6	0.167	Exon(exon2of2)	22924	ALG12	79087
383374	23p	35802148	35803010	863	7	0.571	5'UTR	3357	MAGEB16	139604
386166	1p	16058491	16060000	1510	10	0.9	Exon(exon5of7)	6168	CLCNKB	1188
386166	1p	18481042	18482217	1176	12	0.75	Promoter(<=1kb)	60	KLHDC7A	127707
386166	1p	23874604	23875430	827	8	0.5	Exon(exon2of2)	-6310	FUCA1	2517
386166	1p	40067594	40067675	82	6	0	Promoter(<=1kb)	324	CAP1	10487
386166	1p	62273232	62274777	1546	7	0.429	Promoter(<=1kb)	-441	KANK4	163782
386166	1q	145872200	145873487	1288	6	0.667	Exon(exon8of12)	-12364	PIAS3	10401
386166	1q	152303673	152313891	10219	38	0.579	Promoter(<=1kb)	0	FLG-AS1	339400
386166	1q	158765805	158766655	851	6	0.5	Promoter(<=1kb)	47	OR6N1	128372
386166	1q	169542317	169542882	566	6	0.167	Exon(exon13of25)	-26572	F5	2153
386166	1q	223393517	223394466	950	6	0.667	Promoter(<=1kb)	102	CCDC185	164127
386166	1q	247840950	247841582	633	7	0.857	Promoter(<=1kb)	314	OR11L1	391189
386166	1q	247949443	247949738	296	10	0.3	Promoter(<=1kb)	585	OR2L8	391190
386166	1q	248273309	248273700	392	6	0.333	Promoter(<=1kb)	136	OR2T33	391195
386166	1q	248294677	248295415	739	6	0.667	Promoter(<=1kb)	185	OR2T12	127064
386166	1q	248573992	248574210	219	6	0.833	Promoter(<=1kb)	547	OR2T34	127068
386166	1q	248681658	248682198	541	7	0.571	Promoter(<=1kb)	130	OR14I1	401994
386166	2p	48580657	48582454	1798	7	0.571	Promoter(<=1kb)	0	STON1	11037
386166	2q	130193876	130194275	400	6	0.667	Exon(exon4of5)	4164	TUBA3E	112714
386166	2q	178739433	178741811	2379	6	0.5	Exon(exon45of191)	26014	TTN	7273
386166	2q	185789865	185794632	4768	10	0.8	Promoter(<=1kb)	0	FSIP2	401024
386166	2q	217847567	217848559	993	7	0.857	Exon(exon19of33)	-5407	TNS1	7145
386166	2q	232378876	232380431	1556	6	0.667	Promoter(<=1kb)	125	ALPP	250
386166	2q	238130271	238131546	1276	8	0.375	Promoter(1-2kb)	1323	ESPNL	339768
386166	3p	31989532	31990905	1374	6	0.333	Exon(exon2of2)	7761	ZNF860	344787
386166	3p	75736880	75739243	2364	48	0.625	Promoter(<=1kb)	0	MIR4273	100422955
386166	3q	194359607	194360906	1300	9	0.889	Exon(exon2of2)	-8279	CPN2	1370
386166	4p	1394625	1395373	749	6	0.167	Exon(exon1of1)	9813	UVSSA	57654
386166	4p	5988383	5989749	1367	8	0.625	Promoter(<=1kb)	0	C4orf50	389197
386166	4p	6300792	6302391	1600	7	0.857	Exon(exon8of8)	6021	WFS1	7466
386166	4p	8227004	8228508	1505	8	0.125	Promoter(<=1kb)	-24	SH3TC1	54436

386166	4q	154489498	154491312	1815	9	0.556	Promoter(<=1kb)	22	DCHS2	54798
386166	5q	79728956	79730716	1761	7	0.571	Exon(exon2of13)	-7426	CMYA5	202333
386166	5q	79731782	79734523	2742	13	0.308	Exon(exon2of13)	-3619	CMYA5	202333
386166	5q	140807352	140807737	386	6	0.833	Promoter(<=1kb)	271	PCDHA4	56144
386166	5q	140848579	140850786	2208	6	0.5	Promoter(<=1kb)	807	PCDHA9	9752
386166	5q	141174000	141175025	1026	6	0.833	Promoter(1-2kb)	1356	PCDHB7	56129
386166	5q	141183999	141184688	690	6	0.833	Promoter(2-3kb)	-2473	PCDHB9	56127
386166	5q	141187690	141189425	1736	6	0.5	Promoter(<=1kb)	529	PCDHB9	56127
386166	6p	1312843	1313745	903	6	0.5	Promoter(<=1kb)	745	FOXQ1	94234
386166	6p	46858771	46859389	619	6	0.5	Exon(exon17of21)	3915	ADGRF5	221395
386166	6q	149888581	149890867	2287	7	0.714	Promoter(<=1kb)	0	RAET1E-AS1	100652739
386166	6q	159233455	159234370	916	10	0.5	Exon(exon11of23)	15158	FNDC1	84624
386166	7p	12369637	12370736	1100	6	0.667	3'UTR	-13307	VWDE	221806
386166	7p	38353718	38353991	274	8	0.625	Exon(exon2of2)	3699	TRG-AS1	100506776
386166	7q	64991278	64992758	1481	6	0.667	Promoter(<=1kb)	-242	ZNF117	51351
386166	7q	100958977	100960873	1897	56	0.429	Promoter(1-2kb)	1012	MUC3A	4584
386166	7q	100991195	100992398	1204	7	0.571	Exon(exon5of15)	-20656	MUC12	10071
386166	7q	100995575	100995785	211	7	0.714	Exon(exon5of15)	-17269	MUC12	10071
386166	7q	101034361	101040583	6223	39	0.513	Exon(exon3of12)	-3128	MUC17	140453
386166	8p	8376561	8377994	1434	6	1	Exon(exon2of5)	3753	PRAG1	157285
386166	8p	11331194	11332082	889	6	0.667	Promoter(<=1kb)	306	SLC35G5	83650
386166	8p	12132686	12133940	1255	6	0.667	Promoter(<=1kb)	498	USP17L7	392197
386166	8p	13021128	13022030	903	9	0.222	Exon(exon5of5)	9115	TRMT9B	57604
386166	8q	138151634	138153046	1413	7	0.714	Promoter(<=1kb)	0	FAM135B	51059
386166	8q	141218050	141219792	1743	6	0.667	5'UTR	8778	SLC45A4	57210
386166	9p	21206764	21207074	311	6	0.5	Promoter(<=1kb)	69	IFNA10	3446
386166	9q	76705179	76707804	2626	8	0.5	Promoter(<=1kb)	121	PCA3	50652
386166	9q	76709263	76710843	1581	8	0.5	Promoter(<=1kb)	0	PRUNE2	158471
386166	9q	104598641	104599361	721	8	0.625	Promoter(<=1kb)	52	OR13C5	138799
386166	9q	122553263	122554071	809	7	0.429	Promoter(<=1kb)	93	OR1N2	138882
386166	9q	122749914	122750547	634	6	0.833	Promoter(<=1kb)	174	OR1L6	392390
386166	9q	133255635	133256205	571	9	0.889	3'UTR	19009	ABO	28
386166	9q	135484803	135487213	2411	8	0.25	Promoter(1-2kb)	1440	PPP1R26	9858
386166	9q	135547960	135548795	836	8	0.625	Promoter(1-2kb)	1805	OBP2A	29991
386166	10p	47663	48605	943	7	0.857	Promoter(<=1kb)	664	TUBB8	347688
386166	10q	46549378	46550723	1346	25	0.64	Exon(exon3of3)	4807	GPRIN2	9721
386166	10q	49323169	49325192	2024	7	0.571	Exon(exon3of3)	23895	C10orf71	118461
386166	10q	128103129	128108204	5076	15	0.667	Promoter(<=1kb)	-1	MKI67	4288
386166	11p	244106	244197	92	8	0.5	Promoter(<=1kb)	-232	PSMD13	5719
386166	11p	1191524	1191729	206	6	0.667	Exon(exon31of49)	-31337	MUC5B	727897
386166	11p	1194354	1197925	3572	9	0.667	Exon(exon34of49)	-25141	MUC5B	727897
386166	11p	1243401	1247378	3978	13	0.308	Promoter(2-3kb)	2298	MUC5B-AS1	112577518
386166	11p	5046754	5047432	679	7	0.571	Promoter(<=1kb)	228	OR52J3	119679
386166	11p	5177978	5178478	501	6	0.167	Promoter(<=1kb)	186	OR52Z1	283110
386166	11p	5422212	5423123	912	11	0.636	Promoter(<=1kb)	101	OR51Q1	390061
386166	11p	5515079	5515931	853	7	0.429	Promoter(<=1kb)	768	UBQLNL	143630
386166	11p	5544676	5545259	584	6	0.5	Promoter(<=1kb)	290	OR52H1	390067
386166	11p	5581045	5581738	694	9	0.444	Promoter(<=1kb)	168	OR52B6	340980
386166	11p	5841302	5841883	582	9	0.333	Promoter(<=1kb)	14	OR52E6	390078
386166	11p	11351961	11352736	776	9	0.222	Promoter(<=1kb)	514	CSNK2A3	283106
386166	11p	18173280	18173901	622	6	0.333	Promoter(<=1kb)	443	MRGPRX4	117196
386166	11q	58214757	58215722	966	8	0.25	Promoter(<=1kb)	12	OR1S1	219959
386166	11q	64116513	64118232	1720	8	0.75	Exon(exon2of2)	8702	MACROD1	28992
386166	11q	64315804	64315856	53	7	0.714	Promoter(1-2kb)	1485	TRMT112	51504
386166	11q	66560202	66562261	2060	6	0.333	Exon(exon14of21)	3873	CTSF	8722
386166	11q	69295669	69296300	632	6	0.667	Promoter(1-2kb)	1495	MYEOV	26579
386166	11q	82732630	82733184	555	6	0.833	Promoter(<=1kb)	680	FAM181B	220382
386166	11q	85724687	85725825	1139	6	0.5	Promoter(<=1kb)	0	SYTL2	54843
386166	11q	124015601	124016477	877	8	0.25	Promoter(<=1kb)	26	OR10G4	390264
386166	11q	124023038	124023849	812	9	0.444	Promoter(<=1kb)	25	OR10G9	219870
386166	11q	124038392	124038988	597	6	0.833	Promoter(<=1kb)	13	OR10G7	390265
386166	11q	124382526	124383285	760	8	0.625	Promoter(<=1kb)	58	OR8B2	26595
386166	11q	130914501	130915409	909	10	0.7	Promoter(1-2kb)	1035	SNX19	399979
386166	12p	4626571	4628549	1979	10	0.5	Exon(exon5of6)	14054	DYRK4	8798
386166	13q	25096889	25097154	266	15	0.467	Promoter(1-2kb)	1021	PABPC3	5042
386166	13q	102732474	102733933	1460	6	0.333	Exon(exon4of4)	25139	CCDC168	643677
386166	14q	20640750	20641567	818	8	0.5	Promoter(<=1kb)	124	OR6S1	341799
386166	14q	22633879	22634450	572	9	0.333	Exon(exon2of2)	32212	ABHD4	63874
386166	14q	70457520	70458540	1021	9	0.333	Exon(exon2of2)	5346	ADAM21	8747
386166	14q	94587512	94587839	328	6	0.5	Exon(exon2of2)	-4219	SERPINA3	12
386166	14q	104175275	104177810	2536	7	0.286	Exon(exon12of15)	36235	KIF26A	26153
386166	14q	104939262	104942618	3357	13	0.231	5'UTR	7102	PLD4	122618
386166	14q	104943622	104946886	3265	16	0.625	Exon(exon6of6)	8516	AHNAK2	113146
386166	14q	104947901	104953878	5978	39	0.487	Promoter(1-2kb)	1524	AHNAK2	113146
386166	15q	23439979	23442067	2089	11	0.545	5'UTR	5167	GOLGA6L2	283685
386166	15q	52609086	52609780	695	6	0.333	Promoter(<=1kb)	-490	FAM214A	56204
386166	15q	85579423	85581800	2378	14	0.571	Promoter(1-2kb)	-1110	AKAP13	11214

386166	16p	669592	672548	2957	7	0.571	Promoter(<=1kb)	0	RHOT2	89941
386166	16p	789084	790597	1514	6	0.167	Promoter(<=1kb)	0	CHTF18	63922
386166	16p	1486371	1488463	2093	8	0.75	Promoter(<=1kb)	4	PTX4	390667
386166	16q	74391650	74392004	355	8	0.625	Exon(exon7of7)	13772	NPIPB15	440348
386166	16q	88428539	88429600	1062	6	0.333	Exon(exon3of3)	-23680	ZFPM1	161882
386166	16q	88433131	88436097	2967	6	0.667	Exon(exon3of3)	-17183	ZFPM1	161882
386166	16q	88712207	88714717	2511	7	0.571	Promoter(<=1kb)	0	CTU2	348180
386166	17p	10638198	10641099	2902	6	0.333	Exon(exon19of41)	-8169	MYH3	4621
386166	17p	21300581	21300954	374	8	0.75	3'UTR	9112	MAP2K3	5606
386166	17p	21415470	21416370	901	10	0.7	Exon(exon3of3)	10334	KCNJ12	3768
386166	17q	73236548	73236991	444	6	0.5	Promoter(<=1kb)	0	FAM104A	84923
386166	17q	81645135	81645417	283	6	0.333	Promoter(2-3kb)	2722	TSPAN10	83882
386166	18p	11609904	11610469	566	9	0.667	Promoter(<=1kb)	308	SLC35G4	646000
386166	19p	4511338	4513547	2210	10	0.4	Exon(exon3of6)	4157	PLIN4	729359
386166	19p	5455600	5456439	840	7	0.571	Promoter(<=1kb)	183	ZNRF4	148066
386166	19p	8937644	8939234	1591	6	0.667	Exon(exon5of84)	-41554	MUC16	94025
386166	19p	8946313	8952882	6570	20	0.6	Exon(exon3of84)	28460	MUC16	94025
386166	19p	8959116	8962299	3184	12	0.75	Exon(exon3of84)	19043	MUC16	94025
386166	19p	8971838	8978096	6259	16	0.5	Exon(exon1of84)	3246	MUC16	94025
386166	19p	12075333	12077046	1714	6	0.167	Promoter(<=1kb)	6	ZNF788P	388507
386166	19p	14766987	14767045	59	7	0.857	Exon(exon6of21)	5329	ADGRE2	30817
386166	19p	15087213	15088040	828	9	0.333	Promoter(<=1kb)	233	OR1I1	126370
386166	19p	17281820	17284246	2427	9	0.556	Promoter(<=1kb)	0	ANKLE1	126549
386166	19p	18264753	18267409	2657	8	0.5	5'UTR	7002	IQCN	80726
386166	19p	21971930	21974500	2571	7	0.714	Exon(exon4of4)	14408	ZNF208	7757
386166	19p	22756294	22759533	3240	11	0.545	3'UTR	10449	ZNF99	7652
386166	19p	23743906	23745300	1395	6	0.333	Exon(exon4of4)	13537	ZNF681	148213
386166	19q	37886924	37889059	2136	6	0.833	Exon(exon6of6)	17535	WDR87	83889
386166	19q	40880231	40880622	392	6	0.333	Promoter(<=1kb)	-141	CYP2A7	1549
386166	19q	43846955	43848536	1582	6	0.833	3'UTR	13450	ZNF283	284349
386166	19q	43913423	43914878	1456	7	0.429	Exon(exon10of10)	4861	ZNF45	7596
386166	19q	43966037	43967171	1135	6	0.167	Promoter(<=1kb)	-691	ZNF155	7711
386166	19q	44106512	44108078	1567	7	0	Exon(exon6of6)	-4103	ZNF225	7768
386166	19q	44327836	44329698	1863	6	0.5	Exon(exon4of4)	-22790	ZNF235	9310
386166	19q	51745958	51746963	1006	6	0.5	Exon(exon3of3)	3848	FPR1	2357
386166	19q	52437918	52439242	1325	7	0.429	Exon(exon4of4)	6504	ZNF534	147658
386166	19q	55358736	55359651	916	6	0.667	Promoter(<=1kb)	0	FAM71E2	284418
386166	19q	55911888	55913077	1190	6	0.667	Exon(exon5of12)	19234	NLRP13	126204
386166	19q	58368293	58368875	583	7	0.429	Exon(exon3of3)	-5445	ZNF497	162968
386166	20p	5922421	5923394	974	6	0.5	Exon(exon4of5)	6923	CHGB	1114
386166	20q	63561666	63567309	5644	14	0.643	Promoter(<=1kb)	-61	HELZ2	85441
386166	21q	44550835	44551416	582	6	0.833	Promoter(<=1kb)	89	KRTAP10-2	386679
386166	22q	22352950	22353298	349	13	0.538	Exon(exon1of2)	30478	BMS1P20	96610
386166	22q	22758758	22759209	452	6	0.833	Exon(exon1of2)	-63567	MIR650	723778
386166	22q	36191154	36191906	753	7	0.714	3'UTR	9971	APOL4	80832
386166	22q	36265284	36265945	662	7	0.857	Exon(exon6of6)	12120	APOL1	8542
386166	22q	49884187	49885214	1028	6	0.333	Exon(exon2of2)	22704	ALG12	79087
386166	23p	8170039	8170141	103	6	0.5	Promoter(1-2kb)	1126	VCX2	51480
386166	23p	35802148	35803010	863	7	0.571	5'UTR	3357	MAGEB16	139604
392211	1p	11778784	11779941	1158	7	0.714	Promoter(<=1kb)	0	C1orf167-AS1	102724659
392211	1p	12847526	12847995	470	7	0.429	Promoter(<=1kb)	730	HNRNPCL1	343069
392211	1p	12859036	12860079	1044	6	0.333	Promoter(1-2kb)	1950	PRAMEF2	65122
392211	1p	12893249	12893472	224	6	0.5	Exon(exon4of4)	4798	PRAMEF10	343071
392211	1p	13369166	13369564	399	7	0.714	Promoter(2-3kb)	2336	PRAMEF19	645414
392211	1p	13370686	13370989	304	9	0.556	Promoter(<=1kb)	911	PRAMEF19	645414
392211	1p	16058491	16059952	1462	7	0.857	Exon(exon5of7)	6168	CLCNKB	1188
392211	1p	18481403	18482217	815	7	0.714	Promoter(<=1kb)	421	KLHDC7A	127707
392211	1p	23874604	23875430	827	8	0.5	Exon(exon2of2)	-6310	FUCA1	2517
392211	1p	40067594	40067675	82	6	0	Promoter(<=1kb)	324	CAP1	10487
392211	1q	152213286	152213316	31	6	0.5	Exon(exon3of3)	10877	HRNR	388697
392211	1q	152219233	152221375	2143	15	0.667	Promoter(2-3kb)	2818	HRNR	388697
392211	1q	156669844	156670886	1043	6	1	Exon(exon4of4)	6521	NES	10763
392211	1q	158765805	158766655	851	6	0.5	Promoter(<=1kb)	47	OR6N1	128372
392211	1q	169542317	169542882	566	6	0.167	Exon(exon13of25)	-26572	F5	2153
392211	1q	197101312	197101771	460	6	0.5	Exon(exon18of28)	33373	ASPM	259266
392211	1q	214640144	214642881	2738	12	0.583	Exon(exon12of20)	-5086	CENPF	1063
392211	1q	214644872	214647181	2310	12	0.5	Promoter(<=1kb)	-786	CENPF	1063
392211	1q	226735563	226737239	1677	8	0.875	Promoter(<=1kb)	219	ITPKB	3707
392211	1q	228315976	228318038	2063	8	0.625	Exon(exon50of81)	6492	OBSCN	84033
392211	1q	247841312	247841582	271	6	0.833	Promoter(<=1kb)	314	OR11L1	391189
392211	1q	247949436	247949759	324	11	0.455	Promoter(<=1kb)	578	OR2L8	391190
392211	1q	248294677	248295458	782	7	0.857	Promoter(<=1kb)	142	OR2T12	127064
392211	1q	248573992	248574210	219	6	0.833	Promoter(<=1kb)	547	OR2T34	127068
392211	2p	48580657	48582454	1798	7	0.571	Promoter(<=1kb)	0	STON1	11037
392211	2q	95944651	95945173	523	6	0.5	Promoter(<=1kb)	0	ANKRD36C	400986
392211	2q	130193975	130194376	402	6	0.667	Exon(exon4of5)	4063	TUBA3E	112714
392211	2q	132783032	132785012	1981	7	0.571	Promoter(1-2kb)	-1009	NCKAP5	344148

392211	2q	178739433	178741811	2379	6	0.5	Exon(exon45of191)	26014	TTN	7273
392211	2q	186828181	186828428	248	6	0.333	Exon(exon9of9)	20742	ZSWIM2	151112
392211	2q	208325289	208326375	1087	6	0.5	3'UTR	-7997	PIKFYVE	200576
392211	2q	217847583	217848559	977	6	0.833	Exon(exon19of33)	-5423	TNS1	7145
392211	2q	233713134	233713783	650	13	0.692	Promoter(<=1kb)	142	UGT1A5	54579
392211	2q	237762685	237764060	1376	10	0.5	Exon(exon8of8)	-4137	LRRFIP1	9208
392211	2q	238130271	238131546	1276	7	0.286	Promoter(1-2kb)	1323	ESPNL	339768
392211	3p	31989532	31990905	1374	6	0.333	Exon(exon2of2)	7761	ZNF860	344787
392211	3p	75736929	75738859	1931	10	0.6	Promoter(<=1kb)	0	MIR4273	100422955
392211	3q	98264413	98265098	686	7	0.571	Promoter(<=1kb)	128	OR5H6	79295
392211	4p	5988383	5989749	1367	7	0.571	Promoter(<=1kb)	0	C4orf50	389197
392211	4p	6300792	6302360	1569	7	0.857	Exon(exon8of8)	6021	WFS1	7466
392211	4p	8227004	8228508	1505	8	0.125	Promoter(<=1kb)	-24	SH3TC1	54436
392211	4q	185458217	185460011	1795	9	0.556	Promoter(<=1kb)	0	CCDC110	256309
392211	4q	186619481	186621582	2102	6	0.333	Exon(exon10of27)	-9411	FAT1	2195
392211	4q	186706638	186708616	1979	6	0.667	Exon(exon2of27)	15217	FAT1	2195
392211	5p	795818	796237	420	7	0.571	3'UTR	4908	ZDHHC11	79844
392211	5q	79728956	79730716	1761	7	0.286	Exon(exon2of13)	-7426	CMYA5	202333
392211	5q	79731782	79734523	2742	13	0.308	Exon(exon2of13)	-3619	CMYA5	202333
392211	5q	83537326	83539905	2580	6	0.333	Promoter(1-2kb)	1712	VCAN	1462
392211	5q	141174000	141175025	1026	6	0.833	Promoter(1-2kb)	1356	PCDHB7	56129
392211	5q	141187690	141189425	1736	6	0.5	Promoter(<=1kb)	529	PCDHB9	56127
392211	5q	141955676	141957660	1985	6	0.5	Promoter(<=1kb)	-668	RNF14	9604
392211	5q	151565922	151568158	2237	9	0.778	Promoter(<=1kb)	786	FAT2	2196
392211	5q	177209881	177211855	1975	7	0.714	Promoter(<=1kb)	-256	NSD1	64324
392211	6p	46858771	46859502	732	8	0.5	Exon(exon17of21)	3802	ADGRF5	221395
392211	6q	159231899	159234370	2472	12	0.583	Exon(exon11of23)	13602	FNDC1	84624
392211	7p	12369637	12370736	1100	6	0.667	3'UTR	-13307	VWDE	221806
392211	7p	53035678	53036385	708	7	1	Promoter(<=1kb)	45	POM121L12	285877
392211	7p	56021087	56021209	123	6	0.5	Exon(exon2of7)	12947	PSPH	5723
392211	7q	100958721	100960873	2153	57	0.439	Promoter(<=1kb)	756	MUC3A	4584
392211	7q	100991195	100992398	1204	6	0.5	Exon(exon5of15)	-20656	MUC12	10071
392211	7q	100995575	100995872	298	6	0.5	Exon(exon5of15)	-17182	MUC12	10071
392211	7q	101034305	101038481	4177	25	0.48	Exon(exon3of12)	-5230	MUC17	140453
392211	7q	156949807	156950570	764	6	0.667	Promoter(<=1kb)	95	NOM1	64434
392211	8p	10609650	10612307	2658	8	0.875	Exon(exon4of4)	42836	RP1L1	94137
392211	8p	11331194	11332082	889	8	0.625	Promoter(<=1kb)	306	SLC35G5	83650
392211	8p	12137207	12138641	1435	8	0.875	Promoter(<=1kb)	436	USP17L2	377630
392211	8p	13021128	13022030	903	7	0.143	Exon(exon5of5)	9115	TRMT9B	57604
392211	8q	123651655	123652634	980	7	0.571	Promoter(<=1kb)	316	KLHL38	340359
392211	9q	76703555	76707804	4250	13	0.462	Promoter(<=1kb)	0	PCA3	50652
392211	9q	76709263	76710843	1581	9	0.556	Promoter(<=1kb)	0	PRUNE2	158471
392211	9q	104598545	104599361	817	6	0.333	Promoter(<=1kb)	52	OR13C5	138799
392211	9q	104604727	104605560	834	6	0.5	Promoter(<=1kb)	67	OR13C2	392376
392211	9q	122553263	122554071	809	8	0.5	Promoter(<=1kb)	93	OR1N2	138882
392211	9q	122628595	122629398	804	7	0.286	Promoter(<=1kb)	175	OR1B1	347169
392211	9q	135484803	135487573	2771	9	0.333	Promoter(1-2kb)	1440	PPP1R26	9858
392211	10q	46549378	46550744	1367	26	0.615	5'UTR	4786	GPRIN2	9721
392211	10q	122084988	122087840	2853	8	0.75	Exon(exon4of23)	-25190	TACC2	10579
392211	10q	128103129	128106296	3168	13	0.615	Promoter(<=1kb)	-1	MKI67	4288
392211	11p	244106	244197	92	8	0.5	Promoter(<=1kb)	-232	PSMD13	5719
392211	11p	1194329	1196902	2574	8	0.625	Exon(exon34of49)	-26164	MUC5B	727897
392211	11p	5046754	5047432	679	7	0.571	Promoter(<=1kb)	228	OR52J3	119679
392211	11p	5177978	5178478	501	6	0.167	Promoter(<=1kb)	186	OR52Z1	283110
392211	11p	5389704	5390350	647	6	0.5	Promoter(<=1kb)	327	OR51M1	390059
392211	11p	5422212	5423123	912	12	0.667	Promoter(<=1kb)	101	OR51Q1	390061
392211	11p	5515185	5515931	747	6	0.333	Promoter(<=1kb)	768	UBQLNL	143630
392211	11p	5581045	5581738	694	8	0.375	Promoter(<=1kb)	168	OR52B6	340980
392211	11p	5788000	5788760	761	8	0.75	Promoter(<=1kb)	56	OR52N1	79473
392211	11p	5841302	5841883	582	9	0.333	Promoter(<=1kb)	14	OR52E6	390078
392211	11p	5986042	5986669	628	7	1	Promoter(<=1kb)	316	OR52L1	338751
392211	11p	11352040	11352736	697	8	0.25	Promoter(<=1kb)	514	CSNK2A3	283106
392211	11p	12293639	12294842	1204	7	0.714	Exon(exon29of35)	6739	MICALCL	84953
392211	11q	58214757	58215722	966	7	0.286	Promoter(<=1kb)	12	OR1S1	219959
392211	11q	58402523	58403265	743	8	0.5	Promoter(<=1kb)	144	OR5B3	441608
392211	11q	85724687	85725825	1139	6	0.5	Promoter(<=1kb)	0	SYTL2	54843
392211	11q	124382526	124383285	760	6	0.5	Promoter(<=1kb)	58	OR8B2	26595
392211	11q	130914501	130915580	1080	12	0.75	Promoter(<=1kb)	864	SNX19	399979
392211	12p	4626568	4628549	1982	11	0.455	Exon(exon5of6)	14051	DYRK4	8798
392211	12p	6453119	6453670	552	6	0.667	Promoter(<=1kb)	633	TAPBPL	55080
392211	13q	24434450	24435347	898	7	0.571	Exon(exon31of34)	19787	PARP4	143
392211	13q	102732474	102733933	1460	6	0.333	Exon(exon4of4)	25139	CCDC168	643677
392211	14q	20060048	20060884	837	8	0.625	Promoter(<=1kb)	3	OR4L1	122742
392211	14q	20640982	20641567	586	6	0.5	Promoter(<=1kb)	124	OR6S1	341799
392211	14q	44504986	44506403	1418	6	0.667	Promoter(<=1kb)	880	FSCB	84075
392211	14q	70457532	70457980	449	7	0.429	Exon(exon2of2)	5358	ADAM21	8747
392211	14q	104175275	104177810	2536	10	0.3	Exon(exon12of15)	36235	KIF26A	26153

392211	14q	104939262	104942618	3357	10	0.2	5'UTR	7102	PLD4	122618
392211	14q	104943622	104945444	1823	7	0.714	Exon(exon6of6)	9958	AHNAK2	113146
392211	15q	23440160	23442067	1908	11	0.636	5'UTR	5167	GOLGA6L2	283685
392211	15q	85579207	85582073	2867	15	0.667	Promoter(<=1kb)	-837	AKAP13	11214
392211	15q	99129423	99132517	3095	6	0.333	Exon(exon4of5)	7225	TTC23	64927
392211	15q	100569472	100570097	626	7	0.714	Promoter(<=1kb)	534	LINS1	55180
392211	16p	789084	790597	1514	7	0.286	Promoter(<=1kb)	0	CHTF18	63922
392211	16p	1256345	1256985	641	14	0.714	Promoter(<=1kb)	276	TPSD1	23430
392211	16p	1486371	1488463	2093	8	0.75	Promoter(<=1kb)	4	PTX4	390667
392211	16q	74391650	74391897	248	9	0.778	Exon(exon7of7)	13772	NPIPB15	440348
392211	16q	89100686	89101050	365	7	0.571	Promoter(<=1kb)	24	ACSF3	197322
392211	16q	89226863	89228419	1557	10	0.6	Promoter(2-3kb)	2229	ZNF778	197320
392211	17p	744917	746966	2050	8	0.875	3'UTR	5072	GEMIN4	50628
392211	17p	10638198	10641099	2902	8	0.375	Exon(exon19of41)	-8169	MYH3	4621
392211	17p	21300581	21300954	374	8	0.75	3'UTR	9112	MAP2K3	5606
392211	17p	21415458	21416416	959	9	0.889	Exon(exon3of3)	10322	KCNJ12	3768
392211	17q	73236508	73236991	484	6	0.333	Promoter(<=1kb)	0	FAM104A	84923
392211	17q	81510690	81511591	902	6	0.667	Promoter(<=1kb)	257	ACTG1	71
392211	17q	81645135	81645417	283	6	0.333	Promoter(2-3kb)	2722	TSPAN10	83882
392211	18p	11609646	11610164	519	13	0.846	Promoter(<=1kb)	50	SLC35G4	646000
392211	18q	58535186	58538030	2845	19	0.579	Promoter(<=1kb)	0	ALPK2	115701
392211	19p	4510548	4513547	3000	17	0.471	Exon(exon3of6)	4157	PLIN4	729359
392211	19p	5455600	5456439	840	6	0.5	Promoter(<=1kb)	183	ZNRF4	148066
392211	19p	8333830	8334965	1136	6	0.667	Promoter(<=1kb)	0	KANK3	256949
392211	19p	8937644	8939234	1591	6	0.833	Exon(exon5of84)	-41554	MUC16	94025
392211	19p	8946306	8951868	5563	18	0.667	Exon(exon3of84)	29474	MUC16	94025
392211	19p	8959116	8962299	3184	12	0.583	Exon(exon3of84)	19043	MUC16	94025
392211	19p	8963397	8967127	3731	21	0.667	Exon(exon3of84)	14215	MUC16	94025
392211	19p	8972751	8978096	5346	13	0.462	Exon(exon1of84)	3246	MUC16	94025
392211	19p	12430400	12432437	2038	8	0.5	3'UTR	8584	ZNF443	10224
392211	19p	14841061	14841331	271	6	0.5	Promoter(<=1kb)	546	OR7A10	390892
392211	19p	14880627	14881233	607	6	0.5	Promoter(<=1kb)	219	OR7A17	26333
392211	19p	17281820	17284246	2427	9	0.556	Promoter(<=1kb)	0	ANKLE1	126549
392211	19p	18264753	18267409	2657	8	0.5	5'UTR	7002	IQCN	80726
392211	19p	21971930	21974500	2571	8	0.625	Exon(exon4of4)	14408	ZNF208	7757
392211	19q	33205409	33207385	1977	6	0.667	Promoter(1-2kb)	1539	LRP3	4037
392211	19q	36996730	36997597	868	10	0.6	Exon(exon10of10)	5677	ZNF568	374900
392211	19q	39877222	39877880	659	6	0.5	Exon(exon20of28)	9412	FCGBP	8857
392211	19q	43204093	43205504	1412	6	0.833	Promoter(<=1kb)	0	PSG4	5672
392211	19q	43913423	43914878	1456	8	0.5	Exon(exon10of10)	4861	ZNF45	7596
392211	19q	43966037	43967171	1135	6	0.167	Promoter(<=1kb)	-691	ZNF155	7711
392211	19q	44106512	44108078	1567	8	0.125	Exon(exon6of6)	-4103	ZNF225	7768
392211	19q	52437918	52439242	1325	7	0.429	Exon(exon4of4)	6504	ZNF534	147658
392211	19q	55911888	55913166	1279	7	0.714	Exon(exon5of12)	19145	NLRP13	126204
392211	19q	58368293	58368875	583	7	0.429	Exon(exon3of3)	-5445	ZNF497	162968
392211	20p	20052354	20052736	383	6	0.167	Promoter(<=1kb)	0	CFAP61	26074
392211	20q	63562750	63565531	2782	8	0.375	Promoter(1-2kb)	-1145	HELZ2	85441
392211	21q	26843740	26844859	1120	6	0.667	Promoter(<=1kb)	0	ADAMTS1	9510
392211	21q	44637474	44638143	670	11	0.455	Promoter(<=1kb)	118	KRTAP10-10	353333
392211	21q	44666460	44666841	382	7	0.714	Promoter(<=1kb)	86	KRTAP12-2	353323
392211	22q	22352950	22353348	399	14	0.571	Exon(exon1of2)	30478	BMS1P20	96610
392211	22q	36191154	36191906	753	6	0.667	3'UTR	9971	APOL4	80832
392211	23p	3320126	3323750	3625	10	0.6	Exon(exon5of7)	22902	MXRA5	25878
392211	23p	8170039	8170141	103	6	0.5	Promoter(1-2kb)	1126	VCX2	51480
392211	23p	35802148	35803010	863	7	0.571	5'UTR	3357	MAGEB16	139604
392211	23q	136874183	136874347	165	7	0.714	Promoter(<=1kb)	-201	RBMX	27316
393057	1p	12029085	12030097	1013	6	0.833	Promoter(<=1kb)	0	MIR6729	102466982
393057	1p	16058680	16060000	1321	8	0.75	Exon(exon5of7)	6357	CLCNKB	1188
393057	1p	23874604	23875430	827	8	0.5	Exon(exon2of2)	-6310	FUCA1	2517
393057	1p	26343953	26345408	1456	6	0.833	Exon(exon4of22)	-4607	CRYBG2	55057
393057	1p	40067594	40067675	82	6	0	Promoter(<=1kb)	324	CAP1	10487
393057	1p	89186388	89186419	32	9	0.556	Promoter(<=1kb)	107	GBP4	115361
393057	1q	152218469	152221375	2907	16	0.688	Promoter(2-3kb)	2818	HRNR	388697
393057	1q	152303673	152304920	1248	8	0.5	Exon(exon3of3)	-7656	FLG-AS1	339400
393057	1q	152306380	152313891	7512	26	0.615	Promoter(<=1kb)	0	FLG-AS1	339400
393057	1q	169542317	169542882	566	6	0.167	Exon(exon13of25)	-26572	F5	2153
393057	1q	201206099	201209738	3640	10	0.6	Promoter(1-2kb)	1017	IGFN1	91156
393057	1q	223393517	223394466	950	6	0.667	Promoter(<=1kb)	102	CCDC185	164127
393057	1q	226735683	226737239	1557	7	0.857	Promoter(<=1kb)	219	ITPKB	3707
393057	1q	228315976	228318049	2074	8	0.625	Exon(exon50of81)	6492	OBSCN	84033
393057	1q	240207775	240208254	480	7	0.714	Exon(exon5of18)	-27517	FMN2	56776
393057	1q	247841312	247841582	271	6	0.833	Promoter(<=1kb)	314	OR11L1	391189
393057	1q	247896121	247896502	382	6	0.5	Promoter(<=1kb)	534	OR2W3	343171
393057	1q	247949325	247949738	414	9	0.333	Promoter(<=1kb)	467	OR2L8	391190
393057	2p	48580657	48582454	1798	7	0.571	Promoter(<=1kb)	0	STON1	11037
393057	2q	130193975	130194465	491	6	0.667	Exon(exon4of5)	3974	TUBA3E	112714
393057	2q	132783534	132785001	1468	7	0.429	Promoter(1-2kb)	-1511	NCKAP5	344148

393057	2q	184936178	184937636	1459	6	0.333	Exon(exon4of4)	69813	ZNF804A	91752
393057	2q	185789865	185794632	4768	11	0.818	Promoter(<=1kb)	0	FSIP2	401024
393057	2q	185805377	185808891	3515	7	0.429	Promoter(<=1kb)	0	FSIP2	401024
393057	2q	233840612	233842185	1574	6	0.667	Promoter(<=1kb)	0	HJURP	55355
393057	2q	240041845	240042811	967	6	0.333	Downstream(2-3kb)	3261	OR6B3	150681
393057	3p	13637703	13638208	506	6	0.833	Promoter(1-2kb)	1258	FBLN2	2199
393057	3p	75736929	75739007	2079	14	0.643	Promoter(<=1kb)	0	MIR4273	100422955
393057	3q	98169021	98169594	574	6	0.667	Exon(exon2of2)	19695	OR5H14	403273
393057	3q	196947388	196948102	715	6	0.5	3'UTR	3828	PIGZ	80235
393057	4p	5988383	5989749	1367	7	0.571	Promoter(<=1kb)	0	C4orf50	389197
393057	4p	6300792	6302360	1569	6	0.833	Exon(exon8of8)	6021	WFS1	7466
393057	4p	8227004	8228508	1505	8	0.125	Promoter(<=1kb)	-24	SH3TC1	54436
393057	4p	10443803	10446224	2422	6	0.333	Exon(exon3of3)	10952	ZNF518B	85460
393057	4q	185458217	185460011	1795	8	0.625	Promoter(<=1kb)	0	CCDC110	256309
393057	5p	795818	796237	420	6	0.667	3'UTR	4908	ZDHHC11	79844
393057	5q	79728956	79730716	1761	7	0.286	Exon(exon2of13)	-7426	CMYA5	202333
393057	5q	79731782	79734523	2742	13	0.308	Exon(exon2of13)	-3619	CMYA5	202333
393057	5q	140848579	140850786	2208	6	0.5	Promoter(<=1kb)	807	PCDHA9	9752
393057	5q	141174000	141175025	1026	6	0.833	Promoter(1-2kb)	1356	PCDHB7	56129
393057	5q	151521550	151522069	520	6	0.667	Promoter(<=1kb)	79	MIR6499	102465246
393057	6p	1312843	1313745	903	6	0.5	Promoter(<=1kb)	745	FOXQ1	94234
393057	6p	46858240	46859502	1263	9	0.444	Exon(exon17of21)	3802	ADGRF5	221395
393057	6q	64591274	64591961	688	10	0.5	Exon(exon26of43)	121374	EYS	346007
393057	6q	149888581	149890867	2287	7	0.714	Promoter(<=1kb)	0	RAET1E-AS1	100652739
393057	6q	159233455	159234370	916	10	0.5	Exon(exon11of23)	15158	FNDC1	84624
393057	6q	168307636	168309048	1413	8	0.5	5'UTR	10662	DACT2	168002
393057	7p	38353718	38353991	274	8	0.625	Exon(exon2of2)	3699	TRG-AS1	100506776
393057	7p	53035678	53036385	708	7	1	Promoter(<=1kb)	45	POM121L12	285877
393057	7p	56021087	56021209	123	6	0.5	Exon(exon2of7)	12947	PSPH	5723
393057	7q	100958721	100960873	2153	57	0.439	Promoter(<=1kb)	756	MUC3A	4584
393057	7q	100991195	100994358	3164	10	0.7	Exon(exon5of15)	-18696	MUC12	10071
393057	7q	149818015	149819792	1778	6	0.667	Promoter(2-3kb)	-2352	SSPO	23145
393057	7q	149824094	149826495	2402	6	0.667	Promoter(<=1kb)	0	SSPO	23145
393057	8p	10607245	10608432	1188	8	0.5	Exon(exon4of4)	46711	RP1L1	94137
393057	8p	10609614	10610662	1049	7	0.429	Exon(exon4of4)	44481	RP1L1	94137
393057	8p	13021128	13022030	903	9	0.111	Exon(exon5of5)	9115	TRMT9B	57604
393057	8q	138151949	138153046	1098	6	0.667	Promoter(<=1kb)	0	FAM135B	51059
393057	8q	141466429	141467514	1086	9	0.778	3'UTR	29245	MROH5	389690
393057	8q	142664552	142665852	1301	6	0.833	Exon(exon2of2)	4118	JRK	8629
393057	8q	144516318	144517737	1420	6	0.667	Promoter(<=1kb)	0	RECQL4	9401
393057	9p	712060	713307	1248	7	0.714	Exon(exon7of16)	5171	KANK1	23189
393057	9q	76175241	76175296	56	8	0.875	Exon(exon14of14)	-13343	PCSK5	5125
393057	9q	76705724	76707804	2081	6	0.5	Promoter(<=1kb)	666	PCA3	50652
393057	9q	122553263	122554071	809	7	0.429	Promoter(<=1kb)	93	OR1N2	138882
393057	9q	135484803	135487573	2771	10	0.4	Promoter(1-2kb)	1440	PPP1R26	9858
393057	10q	46549378	46550723	1346	25	0.64	Exon(exon3of3)	4807	GPRIN2	9721
393057	10q	49323169	49325192	2024	7	0.571	Exon(exon3of3)	23895	C10orf71	118461
393057	10q	125026145	125027175	1031	6	0.5	Promoter(<=1kb)	853	CTBP2	1488
393057	10q	128102802	128104752	1951	10	0.5	Promoter(<=1kb)	0	MKI67	4288
393057	11p	244106	244197	92	8	0.5	Promoter(<=1kb)	-232	PSMD13	5719
393057	11p	5177978	5178478	501	6	0.167	Promoter(<=1kb)	186	OR52Z1	283110
393057	11p	5323451	5324256	806	6	0.5	Promoter(<=1kb)	41	OR51B2	79345
393057	11p	5389704	5390350	647	7	0.429	Promoter(<=1kb)	327	OR51M1	390059
393057	11p	5402638	5403471	834	11	0.455	Promoter(<=1kb)	41	OR51J1	79470
393057	11p	5422212	5423123	912	10	0.7	Promoter(<=1kb)	101	OR51Q1	390061
393057	11p	5581045	5581738	694	8	0.375	Promoter(<=1kb)	168	OR52B6	340980
393057	11p	5788000	5788760	761	8	0.75	Promoter(<=1kb)	56	OR52N1	79473
393057	11p	5841302	5841883	582	9	0.333	Promoter(<=1kb)	14	OR52E6	390078
393057	11p	11352040	11352736	697	7	0.143	Promoter(<=1kb)	514	CSNK2A3	283106
393057	11p	12293639	12294368	730	6	0.833	Exon(exon29of35)	6739	MICALCL	84953
393057	11p	43942293	43943348	1056	9	0.778	Promoter(<=1kb)	0	C11orf96	387763
393057	11q	55827536	55827640	105	6	0.667	Promoter(<=1kb)	317	OR5L2	26338
393057	11q	58214757	58215722	966	8	0.25	Promoter(<=1kb)	12	OR1S1	219959
393057	11q	58402523	58403265	743	8	0.5	Promoter(<=1kb)	144	OR5B3	441608
393057	11q	85724687	85725825	1139	6	0.5	Promoter(<=1kb)	0	SYTL2	54843
393057	11q	124015600	124016477	878	7	0.143	Promoter(<=1kb)	25	OR10G4	390264
393057	12p	4626568	4628549	1982	10	0.5	Exon(exon5of6)	14051	DYRK4	8798
393057	12q	52316096	52317765	1670	6	0.667	Exon(exon4of9)	3633	KRT83	3889
393057	12q	55247291	55248136	846	6	0.667	Promoter(<=1kb)	93	OR6C74	254783
393057	13q	25096713	25097182	470	10	0.4	Promoter(<=1kb)	845	PABPC3	5042
393057	13q	102732474	102733933	1460	6	0.333	Exon(exon4of4)	25139	CCDC168	643677
393057	14q	20060048	20060884	837	9	0.556	Promoter(<=1kb)	3	OR4L1	122742
393057	14q	20640750	20641567	818	7	0.571	Promoter(<=1kb)	124	OR6S1	341799
393057	14q	70457532	70457980	449	8	0.375	Exon(exon2of2)	5358	ADAM21	8747
393057	14q	104947943	104950338	2396	8	0.5	Exon(exon6of6)	5064	AHNAK2	113146
393057	15q	23440370	23442067	1698	9	0.667	5'UTR	5167	GOLGA6L2	283685
393057	15q	59206980	59207987	1008	7	0.571	Promoter(<=1kb)	137	LDHAL6B	92483

393057	15q	73702465	73703760	1296	6	0.833	Promoter(<=1kb)	-149	CD276	80381
393057	15q	85579423	85582073	2651	15	0.667	Promoter(<=1kb)	-837	AKAP13	11214
393057	15q	88857108	88859365	2258	7	0	Exon(exon12of18)	9865	ACAN	176
393057	15q	99129423	99132517	3095	6	0.333	Exon(exon4of5)	7225	TTC23	64927
393057	16p	789027	790599	1573	7	0.429	Promoter(<=1kb)	0	CHTF18	63922
393057	16p	1228806	1229716	911	8	0.625	Promoter(<=1kb)	446	TPSB2	64499
393057	16p	1256345	1256985	641	14	0.714	Promoter(<=1kb)	276	TPSD1	23430
393057	16p	1486371	1488463	2093	8	0.75	Promoter(<=1kb)	4	PTX4	390667
393057	16p	28495551	28497395	1845	6	0.333	Promoter(<=1kb)	0	CLN3	1201
393057	16q	72957492	72958851	1360	6	0.5	Exon(exon2of10)	-67658	ZFHX3	463
393057	16q	88428041	88431977	3937	13	0.385	Exon(exon3of3)	-21303	ZFPM1	161882
393057	16q	88433131	88436097	2967	7	0.714	Exon(exon3of3)	-17183	ZFPM1	161882
393057	16q	89226863	89228289	1427	8	0.625	Promoter(2-3kb)	2229	ZNF778	197320
393057	17p	2299649	2300159	511	6	0.167	Exon(exon2of19)	-3224	SRR	63826
393057	17p	10638198	10641099	2902	7	0.286	Exon(exon19of41)	-8169	MYH3	4621
393057	17p	21300581	21300954	374	8	0.75	3'UTR	9112	MAP2K3	5606
393057	17p	21415458	21416416	959	10	0.9	Exon(exon3of3)	10322	KCNJ12	3768
393057	17q	41586466	41586829	364	6	0.833	Promoter(<=1kb)	66	KRT14	3861
393057	17q	41727098	41728331	1234	6	0.833	Promoter(1-2kb)	-1201	HAP1	9001
393057	17q	53823344	53824891	1548	7	0.857	Promoter(<=1kb)	417	KIF2B	84643
393057	17q	76293419	76294016	598	6	0.5	Promoter(2-3kb)	-2167	QRICH2	84074
393057	17q	81645135	81645417	283	6	0.333	Promoter(2-3kb)	2722	TSPAN10	83882
393057	18q	58535186	58538030	2845	19	0.526	Promoter(<=1kb)	0	ALPK2	115701
393057	19p	4510548	4513547	3000	22	0.409	Exon(exon3of6)	4157	PLIN4	729359
393057	19p	5455600	5456439	840	6	0.5	Promoter(<=1kb)	183	ZNRF4	148066
393057	19p	8333830	8335281	1452	7	0.571	Promoter(<=1kb)	0	KANK3	256949
393057	19p	8946313	8951868	5556	15	0.6	Exon(exon3of84)	29474	MUC16	94025
393057	19p	8959116	8962299	3184	11	0.727	Exon(exon3of84)	19043	MUC16	94025
393057	19p	8972751	8978096	5346	16	0.5	Exon(exon1of84)	3246	MUC16	94025
393057	19p	15087213	15088040	828	10	0.3	Promoter(<=1kb)	233	OR1I1	126370
393057	19p	18264798	18267409	2612	12	0.583	5'UTR	7002	IQCN	80726
393057	19p	23743906	23745300	1395	6	0.333	Exon(exon4of4)	13537	ZNF681	148213
393057	19q	39877222	39877880	659	6	0.5	Exon(exon20of28)	9412	FCGBP	8857
393057	19q	39886005	39886439	435	6	0.5	Promoter(<=1kb)	853	FCGBP	8857
393057	19q	43846955	43848536	1582	6	0.833	3'UTR	13450	ZNF283	284349
393057	19q	44106512	44108078	1567	8	0.125	Exon(exon6of6)	-4103	ZNF225	7768
393057	19q	44327836	44329698	1863	6	0.5	Exon(exon4of4)	-22790	ZNF235	9310
393057	19q	51745958	51746963	1006	6	0.333	Exon(exon3of3)	3848	FPR1	2357
393057	19q	52437918	52439242	1325	7	0.429	Exon(exon4of4)	6504	ZNF534	147658
393057	19q	52840798	52841923	1126	9	0.444	3'UTR	7602	ZNF468	90333
393057	19q	55160006	55162286	2281	6	0.333	Promoter(<=1kb)	0	DNAAF3	352909
393057	19q	55912302	55913166	865	6	0.667	Exon(exon5of12)	19145	NLRP13	126204
393057	19q	58368293	58368994	702	8	0.5	Exon(exon3of3)	-5445	ZNF497	162968
393057	20p	5922421	5923394	974	7	0.571	Exon(exon4of5)	6923	CHGB	1114
393057	20q	63562677	63565531	2855	10	0.6	Promoter(1-2kb)	-1072	HELZ2	85441
393057	21q	26843740	26844859	1120	6	0.667	Promoter(<=1kb)	0	ADAMTS1	9510
393057	21q	44627265	44628008	744	6	0.333	Promoter(<=1kb)	172	KRTAP10-9	386676
393057	21q	45455650	45456803	1154	6	0.667	Promoter(<=1kb)	119	COL18A1	80781
393057	22q	22352950	22353348	399	14	0.571	Exon(exon1of2)	30478	BMS1P20	96610
393057	22q	36191154	36191906	753	8	0.625	3'UTR	9971	APOL4	80832
393057	23p	8170039	8170141	103	6	0.5	Promoter(1-2kb)	1126	VCX2	51480
393057	23q	136874183	136874347	165	7	0.714	Promoter(<=1kb)	-201	RBMX	27316
394028	1p	12847526	12847995	470	6	0.5	Promoter(<=1kb)	730	HNRNPCL1	343069
394028	1p	12859036	12860079	1044	6	0.333	Promoter(1-2kb)	1950	PRAMEF2	65122
394028	1p	13369166	13369564	399	7	0.714	Promoter(2-3kb)	2336	PRAMEF19	645414
394028	1p	13370686	13371119	434	9	0.444	Promoter(<=1kb)	781	PRAMEF19	645414
394028	1p	18481403	18483159	1757	8	0.75	Promoter(<=1kb)	421	KLHDC7A	127707
394028	1p	23874604	23875430	827	8	0.5	Exon(exon2of2)	-6310	FUCA1	2517
394028	1p	40067594	40067675	82	6	0	Promoter(<=1kb)	324	CAP1	10487
394028	1p	89186388	89186419	32	9	0.556	Promoter(<=1kb)	107	GBP4	115361
394028	1q	152218928	152221375	2448	19	0.737	Promoter(2-3kb)	2818	HRNR	388697
394028	1q	152303798	152304692	895	7	0.286	Exon(exon3of3)	-7884	FLG-AS1	339400
394028	1q	152307365	152313650	6286	25	0.6	Promoter(<=1kb)	0	FLG-AS1	339400
394028	1q	152910134	152911235	1102	6	0.667	Promoter(1-2kb)	1588	IVL	3713
394028	1q	214640144	214642954	2811	12	0.5	Exon(exon12of20)	-5013	CENPF	1063
394028	1q	214644872	214647181	2310	10	0.4	Promoter(<=1kb)	-786	CENPF	1063
394028	1q	225345982	225346646	665	6	0.167	Exon(exon48of61)	-43094	DNAH14	127602
394028	1q	240207775	240208254	480	8	0.75	Exon(exon5of18)	-27517	FMN2	56776
394028	1q	247841312	247841582	271	6	0.833	Promoter(<=1kb)	314	OR11L1	391189
394028	1q	247949325	247949738	414	10	0.3	Promoter(<=1kb)	467	OR2L8	391190
394028	1q	248294677	248295458	782	6	0.667	Promoter(<=1kb)	142	OR2T12	127064
394028	2p	48580657	48582454	1798	7	0.571	Promoter(<=1kb)	0	STON1	11037
394028	2q	95944651	95945175	525	7	0.571	Promoter(<=1kb)	0	ANKRD36C	400986
394028	2q	130193975	130194465	491	6	0.667	Exon(exon4of5)	3974	TUBA3E	112714
394028	2q	132783032	132785012	1981	7	0.571	Promoter(1-2kb)	-1009	NCKAP5	344148
394028	2q	184936178	184937636	1459	6	0.333	Exon(exon4of4)	69813	ZNF804A	91752
394028	2q	217847567	217848559	993	7	0.857	Exon(exon19of33)	-5407	TNS1	7145

394028	2q	233713134	233713783	650	14	0.714	Promoter(<=1kb)	142	UGT1A5	54579
394028	2q	238130271	238131546	1276	7	0.286	Promoter(1-2kb)	1323	ESPNL	339768
394028	3p	31989532	31990905	1374	6	0.333	Exon(exon2of2)	7761	ZNF860	344787
394028	3q	98169021	98169594	574	6	0.667	Exon(exon2of2)	19695	OR5H14	403273
394028	3q	98264413	98265137	725	6	0.667	Promoter(<=1kb)	128	OR5H6	79295
394028	3q	196947878	196948448	571	6	0.5	3'UTR	3482	PIGZ	80235
394028	4p	5988383	5989749	1367	7	0.571	Promoter(<=1kb)	0	C4orf50	389197
394028	4p	6300792	6302360	1569	6	0.833	Exon(exon8of8)	6021	WFS1	7466
394028	4p	8227004	8228508	1505	8	0.125	Promoter(<=1kb)	-24	SH3TC1	54436
394028	4p	10443803	10446224	2422	6	0.333	Exon(exon3of3)	10952	ZNF518B	85460
394028	4p	38774018	38775552	1535	6	0.333	Promoter(<=1kb)	644	TLR10	81793
394028	4q	185458217	185460011	1795	8	0.625	Promoter(<=1kb)	0	CCDC110	256309
394028	5p	795818	796237	420	6	0.5	3'UTR	4908	ZDHHC11	79844
394028	5q	79731782	79734523	2742	13	0.308	Exon(exon2of13)	-3619	CMYA5	202333
394028	5q	141174000	141175025	1026	6	0.833	Promoter(1-2kb)	1356	PCDHB7	56129
394028	5q	151565922	151568158	2237	10	0.8	Promoter(<=1kb)	786	FAT2	2196
394028	6p	46858771	46859389	619	6	0.5	Exon(exon17of21)	3915	ADGRF5	221395
394028	6q	149888581	149890867	2287	8	0.625	Promoter(<=1kb)	0	RAET1E-AS1	100652739
394028	6q	159231899	159234370	2472	12	0.583	Exon(exon11of23)	13602	FNDC1	84624
394028	7p	53035678	53036385	708	6	1	Promoter(<=1kb)	45	POM121L12	285877
394028	7p	56021087	56021209	123	6	0.5	Exon(exon2of7)	12947	PSPH	5723
394028	7q	100958977	100960873	1897	50	0.46	Promoter(1-2kb)	1012	MUC3A	4584
394028	7q	101034305	101040583	6279	32	0.562	Exon(exon3of12)	-3128	MUC17	140453
394028	8p	10607245	10608261	1017	6	0.667	Exon(exon4of4)	46882	RP1L1	94137
394028	8p	10609650	10612903	3254	11	0.727	Exon(exon4of4)	42240	RP1L1	94137
394028	8p	12137448	12138641	1194	6	0.833	Promoter(<=1kb)	436	USP17L2	377630
394028	8p	13021128	13022030	903	7	0.143	Exon(exon5of5)	9115	TRMT9B	57604
394028	8q	141466429	141467514	1086	9	0.778	3'UTR	29245	MROH5	389690
394028	8q	143916360	143919209	2850	7	0.143	Exon(exon32of32)	20381	PLEC	5339
394028	9q	76703451	76707804	4354	14	0.429	Promoter(<=1kb)	0	PCA3	50652
394028	9q	76709263	76710843	1581	8	0.5	Promoter(<=1kb)	0	PRUNE2	158471
394028	9q	87884955	87887543	2589	6	0.667	Promoter(2-3kb)	2078	SPATA31E1	286234
394028	9q	122553263	122554071	809	8	0.5	Promoter(<=1kb)	93	OR1N2	138882
394028	9q	122628595	122629130	536	6	0.333	Promoter(<=1kb)	443	OR1B1	347169
394028	9q	133255635	133256205	571	7	1	3'UTR	19009	ABO	28
394028	9q	135484803	135487213	2411	9	0.333	Promoter(1-2kb)	1440	PPP1R26	9858
394028	10q	46549378	46550723	1346	26	0.654	Exon(exon3of3)	4807	GPRIN2	9721
394028	10q	49323559	49326554	2996	6	0.5	Exon(exon3of3)	24285	C10orf71	118461
394028	10q	128103129	128106254	3126	13	0.692	Promoter(<=1kb)	-1	MKI67	4288
394028	11p	244106	244197	92	8	0.5	Promoter(<=1kb)	-232	PSMD13	5719
394028	11p	5177978	5178478	501	6	0.167	Promoter(<=1kb)	186	OR52Z1	283110
394028	11p	5323451	5324256	806	6	0.5	Promoter(<=1kb)	41	OR51B2	79345
394028	11p	5389704	5390350	647	7	0.429	Promoter(<=1kb)	327	OR51M1	390059
394028	11p	5422212	5423123	912	10	0.7	Promoter(<=1kb)	101	OR51Q1	390061
394028	11p	5581045	5581738	694	8	0.375	Promoter(<=1kb)	168	OR52B6	340980
394028	11p	5841302	5841883	582	9	0.333	Promoter(<=1kb)	14	OR52E6	390078
394028	11p	11352040	11352736	697	8	0.25	Promoter(<=1kb)	514	CSNK2A3	283106
394028	11p	12293639	12294368	730	6	0.833	Exon(exon29of35)	6739	MICALCL	84953
394028	11q	55572176	55572903	728	6	0.667	Promoter(<=1kb)	48	OR4C16	219428
394028	11q	58214757	58215722	966	9	0.222	Promoter(<=1kb)	12	OR1S1	219959
394028	11q	58402523	58403265	743	8	0.5	Promoter(<=1kb)	144	OR5B3	441608
394028	11q	64315818	64315856	39	6	0.833	Promoter(1-2kb)	1485	TRMT112	51504
394028	11q	82732630	82733184	555	6	0.833	Promoter(<=1kb)	680	FAM181B	220382
394028	11q	85724687	85725825	1139	6	0.5	Promoter(<=1kb)	0	SYTL2	54843
394028	11q	124015601	124016477	877	7	0.143	Promoter(<=1kb)	26	OR10G4	390264
394028	11q	124023038	124023849	812	7	0.429	Promoter(<=1kb)	25	OR10G9	219870
394028	11q	124038366	124038988	623	8	1	Promoter(<=1kb)	13	OR10G7	390265
394028	11q	124264847	124265676	830	6	0.833	Promoter(<=1kb)	20	OR8G5	219865
394028	11q	124382526	124383285	760	7	0.571	Promoter(<=1kb)	58	OR8B2	26595
394028	12p	4626568	4628549	1982	11	0.545	Exon(exon5of6)	14051	DYRK4	8798
394028	13q	24434450	24435347	898	7	0.571	Exon(exon31of34)	19787	PARP4	143
394028	13q	25096713	25097182	470	13	0.462	Promoter(<=1kb)	845	PABPC3	5042
394028	13q	102732474	102733933	1460	6	0.333	Exon(exon4of4)	25139	CCDC168	643677
394028	14q	19975713	19976448	736	8	0.375	Promoter(<=1kb)	269	OR4K15	81127
394028	14q	20060048	20060884	837	9	0.667	Promoter(<=1kb)	3	OR4L1	122742
394028	14q	70457532	70458238	707	8	0.375	Exon(exon2of2)	5358	ADAM21	8747
394028	14q	94587512	94587839	328	6	0.5	Exon(exon2of2)	-4219	SERPINA3	12
394028	14q	104175275	104177810	2536	8	0.25	Exon(exon12of15)	36235	KIF26A	26153
394028	14q	104939262	104942618	3357	11	0.364	5'UTR	7102	PLD4	122618
394028	14q	104943622	104945444	1823	7	0.714	Exon(exon6of6)	9958	AHNAK2	113146
394028	14q	104946805	104953878	7074	38	0.474	Promoter(1-2kb)	1524	AHNAK2	113146
394028	15q	23440081	23442067	1987	11	0.545	5'UTR	5167	GOLGA6L2	283685
394028	15q	40621642	40623696	2055	6	0.333	Promoter(<=1kb)	0	KNL1	57082
394028	15q	78766033	78766626	594	8	0.5	Promoter(<=1kb)	-920	ADAMTS7	11173
394028	15q	85579423	85581800	2378	15	0.6	Promoter(1-2kb)	-1110	AKAP13	11214
394028	15q	100569472	100570097	626	6	0.833	Promoter(<=1kb)	534	LINS1	55180
394028	16p	768559	770215	1657	8	0.5	Promoter(<=1kb)	0	MIR662	724032

394028	16p	1256354	1256985	632	13	0.615	Promoter(<=1kb)	285	TPSD1	23430
394028	16p	1486371	1488463	2093	8	0.75	Promoter(<=1kb)	4	PTX4	390667
394028	16p	4207130	4208004	875	6	0.5	Exon(exon2of7)	31739	SRL	6345
394028	16q	74391650	74391897	248	9	0.778	Exon(exon7of7)	13772	NPIPB15	440348
394028	16q	88428539	88429600	1062	6	0.333	Exon(exon3of3)	-23680	ZFPM1	161882
394028	16q	89226863	89228289	1427	7	0.571	Promoter(2-3kb)	2229	ZNF778	197320
394028	17p	10638198	10641099	2902	7	0.286	Exon(exon19of41)	-8169	MYH3	4621
394028	17p	21300581	21300954	374	11	0.727	3'UTR	9112	MAP2K3	5606
394028	17p	21415458	21416431	974	11	0.818	Exon(exon3of3)	10322	KCNJ12	3768
394028	17q	76293419	76294016	598	6	0.5	Promoter(2-3kb)	-2167	QRICH2	84074
394028	17q	81645135	81645607	473	7	0.286	Promoter(2-3kb)	2722	TSPAN10	83882
394028	18q	58535186	58537515	2330	9	0.333	Promoter(<=1kb)	0	ALPK2	115701
394028	19p	756985	758497	1513	7	0.429	Promoter(<=1kb)	0	MISP	126353
394028	19p	4510548	4513547	3000	19	0.421	Exon(exon3of6)	4157	PLIN4	729359
394028	19p	5455600	5456439	840	7	0.571	Promoter(<=1kb)	183	ZNRF4	148066
394028	19p	8886745	8886806	62	7	0.857	Exon(exon59of84)	5191	MUC16	94025
394028	19p	8937644	8939234	1591	6	0.833	Exon(exon5of84)	-41554	MUC16	94025
394028	19p	8946306	8951868	5563	18	0.667	Exon(exon3of84)	29474	MUC16	94025
394028	19p	8959116	8962299	3184	12	0.583	Exon(exon3of84)	19043	MUC16	94025
394028	19p	8963397	8967127	3731	22	0.636	Exon(exon3of84)	14215	MUC16	94025
394028	19p	8972751	8978096	5346	13	0.462	Exon(exon1of84)	3246	MUC16	94025
394028	19p	12430400	12432437	2038	8	0.5	3'UTR	8584	ZNF443	10224
394028	19p	18264798	18267409	2612	12	0.583	5'UTR	7002	IQCN	80726
394028	19p	21971930	21974500	2571	8	0.75	Exon(exon4of4)	14408	ZNF208	7757
394028	19p	22757840	22759533	1694	11	0.545	Exon(exon4of4)	10449	ZNF99	7652
394028	19q	39886005	39886439	435	7	0.571	Promoter(<=1kb)	853	FCGBP	8857
394028	19q	43846955	43848536	1582	6	0.833	3'UTR	13450	ZNF283	284349
394028	19q	43966037	43967171	1135	6	0.167	Promoter(<=1kb)	-691	ZNF155	7711
394028	19q	44106645	44108078	1434	6	0	Exon(exon6of6)	-4103	ZNF225	7768
394028	19q	49908289	49909393	1105	6	0.833	Exon(exon2of2)	3697	NUP62	23636
394028	19q	51414858	51417359	2502	7	0.571	Promoter(<=1kb)	0	LOC10012908:	100129083
394028	19q	52437918	52439242	1325	7	0.429	Exon(exon4of4)	6504	ZNF534	147658
394028	19q	52840798	52841923	1126	7	0.429	3'UTR	7602	ZNF468	90333
394028	19q	55358524	55359651	1128	7	0.571	Promoter(<=1kb)	0	FAM71E2	284418
394028	19q	55481625	55483456	1832	6	0.5	Promoter(1-2kb)	-1732	NAT14	57106
394028	19q	55517821	55518176	356	7	1	Exon(exon14of14)	18127	SBK2	646643
394028	19q	55911888	55913077	1190	6	0.667	Exon(exon5of12)	19234	NLRP13	126204
394028	20p	5922421	5923643	1223	9	0.667	Exon(exon4of5)	6923	CHGB	1114
394028	20q	63561666	63565531	3866	11	0.636	Promoter(<=1kb)	-61	HELZ2	85441
394028	21q	26843740	26844859	1120	6	0.667	Promoter(<=1kb)	0	ADAMTS1	9510
394028	21q	44600627	44601692	1066	8	0.875	Promoter(<=1kb)	30	KRTAP10-7	386675
394028	22q	22352950	22353348	399	14	0.571	Exon(exon1of2)	30478	BMS1P20	96610
394028	22q	36191154	36191906	753	7	0.714	3'UTR	9971	APOL4	80832
394028	23p	3320126	3323750	3625	9	0.556	Exon(exon5of7)	22902	MXRA5	25878
394028	23p	8170039	8170141	103	6	0.5	Promoter(1-2kb)	1126	VCX2	51480
394028	23q	141906066	141907529	1464	12	0.75	Promoter(1-2kb)	1264	MAGEC1	9947
398253	1p	16058491	16060000	1510	10	0.9	Exon(exon5of7)	6168	CLCNKB	1188
398253	1p	18481403	18482217	815	6	0.667	Promoter(<=1kb)	421	KLHDC7A	127707
398253	1p	40067594	40067675	82	6	0	Promoter(<=1kb)	324	CAP1	10487
398253	1p	89186388	89186419	32	9	0.556	Promoter(<=1kb)	107	GBP4	115361
398253	1q	145872200	145873487	1288	6	0.667	Exon(exon8of12)	-12364	PIAS3	10401
398253	1q	152213274	152213320	47	8	0.5	Exon(exon3of3)	10873	HRNR	388697
398253	1q	152218469	152221375	2907	17	0.706	Promoter(2-3kb)	2818	HRNR	388697
398253	1q	152302977	152313891	10915	38	0.579	Promoter(<=1kb)	0	FLG-AS1	339400
398253	1q	156669844	156670886	1043	6	1	Exon(exon4of4)	6521	NES	10763
398253	1q	169542317	169542882	566	6	0.167	Exon(exon13of25)	-26572	F5	2153
398253	1q	201206099	201209849	3751	12	0.583	Promoter(1-2kb)	1017	IGFN1	91156
398253	1q	223393517	223394466	950	6	0.667	Promoter(<=1kb)	102	CCDC185	164127
398253	1q	228315976	228317998	2023	6	0.5	Exon(exon50of81)	6492	OBSCN	84033
398253	1q	247841312	247841582	271	6	0.833	Promoter(<=1kb)	314	OR11L1	391189
398253	1q	247896121	247896502	382	6	0.5	Promoter(<=1kb)	534	OR2W3	343171
398253	1q	247949325	247949738	414	10	0.3	Promoter(<=1kb)	467	OR2L8	391190
398253	1q	248294677	248295458	782	7	0.571	Promoter(<=1kb)	142	OR2T12	127064
398253	2p	48580657	48582454	1798	7	0.571	Promoter(<=1kb)	0	STON1	11037
398253	2q	217847583	217848746	1164	7	0.857	Exon(exon19of33)	-5423	TNS1	7145
398253	2q	219271337	219271649	313	6	0.667	Exon(exon4of4)	6227	TUBA4A	7277
398253	2q	233840612	233842185	1574	6	0.667	Promoter(<=1kb)	0	HJURP	55355
398253	2q	238130271	238131546	1276	7	0.286	Promoter(1-2kb)	1323	ESPNL	339768
398253	2q	240041845	240042811	967	8	0.25	Downstream(2-3kb)	3261	OR6B3	150681
398253	3p	31989532	31990905	1374	8	0.375	Exon(exon2of2)	7761	ZNF860	344787
398253	3p	75736880	75739007	2128	65	0.631	Promoter(<=1kb)	0	MIR4273	100422955
398253	3q	98264413	98265098	686	7	0.571	Promoter(<=1kb)	128	OR5H6	79295
398253	3q	194341097	194342571	1475	7	0.714	Exon(exon2of2)	8747	CPN2	1370
398253	3q	196947878	196948448	571	7	0.571	3'UTR	3482	PIGZ	80235
398253	4p	5988383	5989749	1367	7	0.571	Promoter(<=1kb)	0	C4orf50	389197
398253	4p	6300792	6302360	1569	6	0.833	Exon(exon8of8)	6021	WFS1	7466
398253	4p	8227004	8228508	1505	7	0.143	Promoter(<=1kb)	-24	SH3TC1	54436

398253	4q	186617176	186621627	4452	12	0.417	Exon(exon10of27)	-7106	FAT1	2195
398253	5q	83537326	83539905	2580	6	0.333	Promoter(1-2kb)	1712	VCAN	1462
398253	5q	140807352	140808990	1639	10	0.7	Promoter(<=1kb)	271	PCDHA4	56144
398253	5q	140848579	140850786	2208	7	0.571	Promoter(<=1kb)	807	PCDHA9	9752
398253	5q	141174000	141175025	1026	7	0.857	Promoter(1-2kb)	1356	PCDHB7	56129
398253	5q	141183628	141184688	1061	7	0.857	Promoter(2-3kb)	2229	PCDHB16	57717
398253	5q	141187690	141189425	1736	6	0.5	Promoter(<=1kb)	529	PCDHB9	56127
398253	5q	151521550	151522069	520	6	0.667	Promoter(<=1kb)	79	MIR6499	102465246
398253	5q	151565922	151568455	2534	10	0.8	Promoter(<=1kb)	489	FAT2	2196
398253	6p	46858771	46859389	619	6	0.5	Exon(exon17of21)	3915	ADGRF5	221395
398253	6q	159233455	159234370	916	10	0.5	Exon(exon11of23)	15158	FNDC1	84624
398253	7p	6330446	6330944	499	6	1	Exon(exon2of2)	7749	FAM220A	84792
398253	7p	12369637	12370736	1100	6	0.667	3'UTR	-13307	VWDE	221806
398253	7q	100958721	100960873	2153	55	0.473	Promoter(<=1kb)	756	MUC3A	4584
398253	7q	100991195	100993127	1933	8	0.625	Exon(exon5of15)	-19927	MUC12	10071
398253	7q	100995575	100995785	211	6	0.833	Exon(exon5of15)	-17269	MUC12	10071
398253	7q	149818015	149819792	1778	7	0.714	Promoter(2-3kb)	-2352	SSPO	23145
398253	8p	8376561	8377198	638	6	1	Exon(exon2of5)	4549	PRAG1	157285
398253	8p	10609614	10612307	2694	11	0.909	Exon(exon4of4)	42836	RP1L1	94137
398253	8p	12132686	12133940	1255	6	0.833	Promoter(<=1kb)	498	USP17L7	392197
398253	8p	12137448	12138641	1194	6	0.833	Promoter(<=1kb)	436	USP17L2	377630
398253	8p	13021128	13022030	903	8	0.25	Exon(exon5of5)	9115	TRMT9B	57604
398253	8q	123651873	123652634	762	6	0.333	Promoter(<=1kb)	316	KLHL38	340359
398253	8q	138151949	138153046	1098	6	0.667	Promoter(<=1kb)	0	FAM135B	51059
398253	8q	141218050	141219792	1743	7	0.714	5'UTR	8778	SLC45A4	57210
398253	9p	116800	118032	1233	7	0.714	Promoter(<=1kb)	172	FOXD4	2298
398253	9p	712060	713307	1248	7	0.714	Exon(exon7of16)	5171	KANK1	23189
398253	9p	21206764	21207038	275	6	0.667	Promoter(<=1kb)	105	IFNA10	3446
398253	9q	76705724	76710699	4976	13	0.615	Promoter(<=1kb)	0	PRUNE2	158471
398253	9q	104598545	104599396	852	17	0.588	Promoter(<=1kb)	17	OR13C5	138799
398253	9q	122553263	122554071	809	8	0.5	Promoter(<=1kb)	93	OR1N2	138882
398253	9q	122628595	122629130	536	6	0.333	Promoter(<=1kb)	443	OR1B1	347169
398253	9q	122749914	122750547	634	6	0.833	Promoter(<=1kb)	174	OR1L6	392390
398253	10p	47454	48605	1152	8	0.625	Promoter(<=1kb)	664	TUBB8	347688
398253	10q	46549378	46550723	1346	26	0.654	Exon(exon3of3)	4807	GPRIN2	9721
398253	10q	49323169	49325572	2404	8	0.5	Exon(exon3of3)	23895	C10orf71	118461
398253	10q	89737450	89738561	1112	6	0	Exon(exon20of33)	13874	KIF20B	9585
398253	11p	244106	244197	92	8	0.5	Promoter(<=1kb)	-232	PSMD13	5719
398253	11p	1194354	1196902	2549	7	0.571	Exon(exon34of49)	-26164	MUC5B	727897
398253	11p	5177978	5178478	501	6	0.167	Promoter(<=1kb)	186	OR52Z1	283110
398253	11p	5323362	5324256	895	8	0.5	Promoter(<=1kb)	41	OR51B2	79345
398253	11p	5389704	5390350	647	6	0.5	Promoter(<=1kb)	327	OR51M1	390059
398253	11p	5402638	5403322	685	9	0.444	Promoter(<=1kb)	41	OR51J1	79470
398253	11p	5440761	5441472	712	6	0.833	Promoter(<=1kb)	42	OR51I1	390063
398253	11p	5581045	5581738	694	8	0.375	Promoter(<=1kb)	168	OR52B6	340980
398253	11p	5755057	5755393	337	6	0.167	Promoter(<=1kb)	364	OR52N4	390072
398253	11p	5788000	5788581	582	7	0.714	Promoter(<=1kb)	235	OR52N1	79473
398253	11p	11351961	11352736	776	9	0.222	Promoter(<=1kb)	514	CSNK2A3	283106
398253	11p	12293639	12294368	730	6	0.833	Exon(exon29of35)	6739	MICALCL	84953
398253	11q	58214757	58215722	966	7	0.286	Promoter(<=1kb)	12	OR1S1	219959
398253	11q	58402523	58403265	743	8	0.5	Promoter(<=1kb)	144	OR5B3	441608
398253	11q	64315818	64315856	39	6	0.833	Promoter(1-2kb)	1485	TRMT112	51504
398253	11q	66560202	66562261	2060	6	0.333	Exon(exon14of21)	3873	CTSF	8722
398253	11q	85724687	85725825	1139	6	0.5	Promoter(<=1kb)	0	SYTL2	54843
398253	11q	123906595	123907324	730	9	0.444	Promoter(<=1kb)	449	OR8D4	338662
398253	11q	124015600	124016477	878	7	0.143	Promoter(<=1kb)	25	OR10G4	390264
398253	11q	124038366	124038988	623	9	0.889	Promoter(<=1kb)	13	OR10G7	390265
398253	12p	4626568	4628549	1982	9	0.556	Exon(exon5of6)	14051	DYRK4	8798
398253	12q	52316096	52317765	1670	6	0.667	Exon(exon4of9)	3633	KRT83	3889
398253	13q	102732474	102733933	1460	6	0.333	Exon(exon4of4)	25139	CCDC168	643677
398253	14q	20060048	20060884	837	8	0.625	Promoter(<=1kb)	3	OR4L1	122742
398253	14q	21634137	21634589	453	9	0.556	Promoter(<=1kb)	351	OR10G2	26534
398253	14q	44504986	44506403	1418	7	0.714	Promoter(<=1kb)	880	FSCB	84075
398253	14q	70457520	70458540	1021	12	0.333	Exon(exon2of2)	5346	ADAM21	8747
398253	14q	94587512	94587839	328	6	0.5	Exon(exon2of2)	-4219	SERPINA3	12
398253	14q	104175455	104177762	2308	8	0.375	Exon(exon12of15)	36415	KIF26A	26153
398253	14q	104939262	104942618	3357	11	0.273	5'UTR	7102	PLD4	122618
398253	14q	104943622	104945444	1823	7	0.571	Exon(exon6of6)	9958	AHNAK2	113146
398253	14q	104947943	104950520	2578	11	0.455	Exon(exon6of6)	4882	AHNAK2	113146
398253	15q	20534501	20535129	629	6	0.5	Exon(exon8of9)	6671	GOLGA6L6	727832
398253	15q	23439979	23442067	2089	12	0.583	5'UTR	5167	GOLGA6L2	283685
398253	15q	40621642	40623696	2055	6	0.333	Promoter(<=1kb)	0	KNL1	57082
398253	15q	59206980	59208056	1077	7	0.714	Promoter(<=1kb)	137	LDHAL6B	92483
398253	15q	75689209	75690531	1323	6	0.667	Exon(exon3of10)	22317	CSPG4	1464
398253	15q	85579423	85582073	2651	15	0.6	Promoter(<=1kb)	-837	AKAP13	11214
398253	15q	99129423	99132517	3095	6	0.333	Exon(exon4of5)	7225	TTC23	64927
398253	16p	1256345	1256980	636	6	0.833	Promoter(<=1kb)	276	TPSD1	23430

398253	16p	4207130	4208004	875	6	0.5	Exon(exon2of7)	31739	SRL	6345
398253	16q	74391650	74392004	355	8	0.625	Exon(exon7of7)	13772	NPIPB15	440348
398253	16q	88714717	88717113	2397	8	0.5	Promoter(<=1kb)	0	MIR4722	100616167
398253	16q	89226863	89228390	1528	10	0.6	Promoter(2-3kb)	2229	ZNF778	197320
398253	17p	10638198	10641099	2902	7	0.286	Exon(exon19of41)	-8169	MYH3	4621
398253	17p	21300581	21300978	398	12	0.75	3'UTR	9112	MAP2K3	5606
398253	17q	41727098	41728331	1234	6	0.833	Promoter(1-2kb)	-1201	HAP1	9001
398253	17q	43091983	43093449	1467	6	0.333	Promoter(<=1kb)	-132	BRCA1	672
398253	17q	76293419	76294016	598	6	0.5	Promoter(2-3kb)	-2167	QRICH2	84074
398253	17q	81510690	81511591	902	6	0.667	Promoter(<=1kb)	257	ACTG1	71
398253	17q	81645135	81645417	283	6	0.333	Promoter(2-3kb)	2722	TSPAN10	83882
398253	18p	11609646	11610350	705	11	0.818	Promoter(<=1kb)	50	SLC35G4	646000
398253	18p	14542649	14543140	492	6	0.5	Promoter(<=1kb)	6	POTEC	388468
398253	18q	58535186	58538030	2845	18	0.556	Promoter(<=1kb)	0	ALPK2	115701
398253	19p	4511338	4513547	2210	8	0.25	Exon(exon3of6)	4157	PLIN4	729359
398253	19p	5455600	5456439	840	7	0.571	Promoter(<=1kb)	183	ZNRF4	148066
398253	19p	8948231	8951868	3638	12	0.583	Exon(exon3of84)	29474	MUC16	94025
398253	19p	15087452	15087953	502	7	0.143	Promoter(<=1kb)	472	OR1I1	126370
398253	19p	15794192	15794719	528	6	0.333	Promoter(<=1kb)	241	OR10H5	284433
398253	19p	17281820	17284246	2427	9	0.556	Promoter(<=1kb)	0	ANKLE1	126549
398253	19p	18264753	18267409	2657	14	0.643	5'UTR	7002	IQCN	80726
398253	19p	21971930	21974500	2571	7	0.714	Exon(exon4of4)	14408	ZNF208	7757
398253	19p	22756205	22759523	3319	13	0.615	3'UTR	10459	ZNF99	7652
398253	19q	36996730	36997597	868	10	0.6	Exon(exon10of10)	5677	ZNF568	374900
398253	19q	37885190	37888806	3617	8	0.625	Exon(exon6of6)	17788	WDR87	83889
398253	19q	39877222	39877880	659	6	0.5	Exon(exon20of28)	9412	FCGBP	8857
398253	19q	39892073	39894014	1942	7	0.714	Exon(exon12of28)	-4781	FCGBP	8857
398253	19q	43913423	43914878	1456	7	0.429	Exon(exon10of10)	4861	ZNF45	7596
398253	19q	43996326	43997366	1041	6	0.5	Exon(exon5of5)	5419	LOC10192806[illegible]	101928063
398253	19q	44106512	44108078	1567	7	0	Exon(exon6of6)	-4103	ZNF225	7768
398253	19q	52437918	52439242	1325	7	0.429	Exon(exon4of4)	6504	ZNF534	147658
398253	19q	53490416	53491750	1335	6	0.5	Exon(exon4of4)	22673	ZNF813	126017
398253	19q	55911888	55913077	1190	6	0.667	Exon(exon5of12)	19234	NLRP13	126204
398253	20q	62332390	62334004	1615	6	0.333	Promoter(<=1kb)	0	MIR4758	100616340
398253	20q	63349752	63350772	1021	6	0.5	3'UTR	3794	CHRNA4	1137
398253	20q	63561666	63564319	2654	8	0.75	Promoter(<=1kb)	-61	HELZ2	85441
398253	21q	44637474	44638143	670	11	0.455	Promoter(<=1kb)	118	KRTAP10-10	353333
398253	21q	44666460	44666841	382	7	0.714	Promoter(<=1kb)	86	KRTAP12-2	353323
398253	22q	22352950	22353380	431	16	0.5	Exon(exon1of2)	30478	BMS1P20	96610
398253	22q	36191154	36191906	753	6	0.667	3'UTR	9971	APOL4	80832
398253	22q	49884187	49884994	808	6	0.333	Exon(exon2of2)	22924	ALG12	79087
398253	23p	3320126	3323750	3625	9	0.556	Exon(exon5of7)	22902	MXRA5	25878
398253	23p	8170039	8170141	103	6	0.5	Promoter(1-2kb)	1126	VCX2	51480
398253	23p	35802148	35803010	863	7	0.571	5'UTR	3357	MAGEB16	139604
398253	23q	136347344	136349199	1856	6	0.5	Exon(exon6of26)	41323	ADGRG4	139378
402252	1p	16058491	16060000	1510	13	0.846	Exon(exon5of7)	6168	CLCNKB	1188
402252	1p	23874604	23875430	827	8	0.5	Exon(exon2of2)	-6310	FUCA1	2517
402252	1p	40067594	40067675	82	6	0	Promoter(<=1kb)	324	CAP1	10487
402252	1q	152303673	152313891	10219	38	0.579	Promoter(<=1kb)	0	FLG-AS1	339400
402252	1q	158765805	158766655	851	6	0.5	Promoter(<=1kb)	47	OR6N1	128372
402252	1q	169541142	169542882	1741	10	0.3	Exon(exon13of25)	-25397	F5	2153
402252	1q	201206099	201209342	3244	9	0.667	Promoter(1-2kb)	1017	IGFN1	91156
402252	1q	214640144	214642954	2811	12	0.5	Exon(exon12of20)	-5013	CENPF	1063
402252	1q	214644872	214647181	2310	10	0.4	Promoter(<=1kb)	-786	CENPF	1063
402252	1q	228315976	228318049	2074	7	0.571	Exon(exon50of81)	6492	OBSCN	84033
402252	1q	236553647	236555893	2247	6	0.333	Exon(exon39of45)	10351	LGALS8	3964
402252	1q	247841312	247841582	271	6	0.833	Promoter(<=1kb)	314	OR11L1	391189
402252	1q	247949443	247949738	296	9	0.333	Promoter(<=1kb)	585	OR2L8	391190
402252	2q	102351547	102351902	356	7	0.429	Exon(exon11of11)	-4027	IL18R1	8809
402252	2q	184936178	184938637	2460	8	0.375	Exon(exon4of4)	69813	ZNF804A	91752
402252	2q	185789865	185794632	4768	11	0.818	Promoter(<=1kb)	0	FSIP2	401024
402252	2q	217847583	217848559	977	6	0.833	Exon(exon19of33)	-5423	TNS1	7145
402252	2q	233840612	233842185	1574	6	0.667	Promoter(<=1kb)	0	HJURP	55355
402252	2q	238130416	238131546	1131	6	0.333	Promoter(1-2kb)	1468	ESPNL	339768
402252	2q	240041845	240042154	310	6	0.167	Downstream(2-3kb)	3918	OR6B3	150681
402252	3p	31989532	31990905	1374	7	0.286	Exon(exon2of2)	7761	ZNF860	344787
402252	3p	75737230	75739007	1778	9	0.556	Promoter(<=1kb)	0	MIR4273	100422955
402252	3q	98168777	98169561	785	7	0.571	Exon(exon2of2)	19451	OR5H14	403273
402252	3q	98264413	98265098	686	7	0.571	Promoter(<=1kb)	128	OR5H6	79295
402252	4p	5988383	5989749	1367	7	0.571	Promoter(<=1kb)	0	C4orf50	389197
402252	4p	6300792	6302360	1569	6	0.833	Exon(exon8of8)	6021	WFS1	7466
402252	4q	121036404	121037542	1139	6	0.333	Promoter(1-2kb)	1442	NDNF	79625
402252	4q	186619481	186621582	2102	6	0.333	Exon(exon10of27)	-9411	FAT1	2195
402252	5p	795818	796261	444	8	0.75	3'UTR	4884	ZDHHC11	79844
402252	5q	79728956	79730716	1761	6	0.5	Exon(exon2of13)	-7426	CMYA5	202333
402252	5q	79731782	79734523	2742	13	0.308	Exon(exon2of13)	-3619	CMYA5	202333
402252	5q	140807352	140807737	386	6	0.833	Promoter(<=1kb)	271	PCDHA4	56144

402252	5q	141174000	141175025	1026	6	0.833	Promoter(1-2kb)	1356	PCDHB7	56129
402252	5q	141183999	141184688	690	8	1	Promoter(2-3kb)	-2473	PCDHB9	56127
402252	5q	141955356	141957660	2305	6	0.667	Promoter(<=1kb)	-668	RNF14	9604
402252	5q	148826877	148828070	1194	7	0.857	Promoter(1-2kb)	1632	ADRB2	154
402252	6p	1312843	1313745	903	6	0.5	Promoter(<=1kb)	745	FOXQ1	94234
402252	6p	46858771	46859502	732	8	0.5	Exon(exon17of21)	3802	ADGRF5	221395
402252	7p	6330446	6330944	499	6	1	Exon(exon2of2)	7749	FAM220A	84792
402252	7q	100958977	100960873	1897	52	0.462	Promoter(1-2kb)	1012	MUC3A	4584
402252	7q	100991195	100992398	1204	7	0.571	Exon(exon5of15)	-20656	MUC12	10071
402252	7q	100995575	100995785	211	7	0.714	Exon(exon5of15)	-17269	MUC12	10071
402252	7q	101004421	101004836	416	6	0.667	Exon(exon5of15)	-8218	MUC12	10071
402252	7q	101034361	101040583	6223	34	0.5	Exon(exon3of12)	-3128	MUC17	140453
402252	8p	10609614	10612307	2694	10	0.9	Exon(exon4of4)	42836	RP1L1	94137
402252	8p	12132477	12133940	1464	10	0.7	Promoter(<=1kb)	498	USP17L7	392197
402252	8p	13021128	13022030	903	7	0.143	Exon(exon5of5)	9115	TRMT9B	57604
402252	8q	123651655	123652634	980	7	0.571	Promoter(<=1kb)	316	KLHL38	340359
402252	9p	39078723	39078846	124	7	0.714	Exon(exon22of24)	7302	CNTNAP3	79937
402252	9q	76175237	76175296	60	9	0.778	Exon(exon14of14)	-13343	PCSK5	5125
402252	9q	76705724	76707804	2081	6	0.5	Promoter(<=1kb)	666	PCA3	50652
402252	9q	87886533	87888536	2004	7	0.571	Exon(exon4of4)	3656	SPATA31E1	286234
402252	9q	104504315	104505071	757	6	0.333	Promoter(<=1kb)	52	OR13F1	138805
402252	9q	104598545	104599361	817	12	0.583	Promoter(<=1kb)	52	OR13C5	138799
402252	9q	122553263	122554071	809	7	0.429	Promoter(<=1kb)	93	OR1N2	138882
402252	9q	122628595	122629130	536	6	0.333	Promoter(<=1kb)	443	OR1B1	347169
402252	9q	122749914	122750547	634	6	0.833	Promoter(<=1kb)	174	OR1L6	392390
402252	9q	135484803	135487573	2771	9	0.333	Promoter(1-2kb)	1440	PPP1R26	9858
402252	10q	46549378	46550723	1346	26	0.654	Exon(exon3of3)	4807	GPRIN2	9721
402252	10q	49323504	49325572	2069	7	0.429	Exon(exon3of3)	24230	C10orf71	118461
402252	11p	244106	244197	92	8	0.5	Promoter(<=1kb)	-232	PSMD13	5719
402252	11p	1241677	1248605	6929	23	0.565	Promoter(1-2kb)	1071	MUC5B-AS1	112577518
402252	11p	1250091	1251628	1538	9	0.778	Promoter(<=1kb)	-415	MUC5B-AS1	112577518
402252	11p	5177978	5178478	501	6	0.167	Promoter(<=1kb)	186	OR52Z1	283110
402252	11p	5323542	5324256	715	6	0.5	Promoter(<=1kb)	41	OR51B2	79345
402252	11p	5351521	5352416	896	15	0.467	Promoter(<=1kb)	13	OR51B6	390058
402252	11p	5389704	5390350	647	7	0.429	Promoter(<=1kb)	327	OR51M1	390059
402252	11p	5402638	5403322	685	10	0.5	Promoter(<=1kb)	41	OR51J1	79470
402252	11p	5422212	5423123	912	10	0.7	Promoter(<=1kb)	101	OR51Q1	390061
402252	11p	5581045	5581738	694	8	0.375	Promoter(<=1kb)	168	OR52B6	340980
402252	11p	5841302	5841883	582	9	0.333	Promoter(<=1kb)	14	OR52E6	390078
402252	11p	5884818	5885061	244	7	0.429	Promoter(<=1kb)	547	OR52E4	390081
402252	11p	5986042	5986669	628	7	1	Promoter(<=1kb)	316	OR52L1	338751
402252	11p	11351961	11352736	776	9	0.222	Promoter(<=1kb)	514	CSNK2A3	283106
402252	11p	12293639	12294842	1204	7	0.571	Exon(exon29of35)	6739	MICALCL	84953
402252	11p	43942293	43943348	1056	9	0.778	Promoter(<=1kb)	0	C11orf96	387763
402252	11q	55827536	55827640	105	6	0.667	Promoter(<=1kb)	317	OR5L2	26338
402252	11q	58214757	58215722	966	8	0.25	Promoter(<=1kb)	12	OR1S1	219959
402252	11q	64315797	64315856	60	8	0.75	Promoter(1-2kb)	1485	TRMT112	51504
402252	11q	85724687	85725825	1139	6	0.5	Promoter(<=1kb)	0	SYTL2	54843
402252	11q	123906790	123907324	535	6	0.667	Promoter(<=1kb)	644	OR8D4	338662
402252	11q	124038595	124038988	394	6	0.833	Promoter(<=1kb)	13	OR10G7	390265
402252	11q	124382526	124383285	760	7	0.714	Promoter(<=1kb)	58	OR8B2	26595
402252	12p	4626568	4628549	1982	11	0.455	Exon(exon5of6)	14051	DYRK4	8798
402252	13q	102732474	102733933	1460	6	0.333	Exon(exon4of4)	25139	CCDC168	643677
402252	14q	19975713	19976448	736	9	0.444	Promoter(<=1kb)	269	OR4K15	81127
402252	14q	20060048	20060884	837	8	0.625	Promoter(<=1kb)	3	OR4L1	122742
402252	14q	21634137	21634589	453	9	0.556	Promoter(<=1kb)	351	OR10G2	26534
402252	14q	70457532	70458540	1009	10	0.4	Exon(exon2of2)	5358	ADAM21	8747
402252	14q	104175455	104177762	2308	8	0.25	Exon(exon12of15)	36415	KIF26A	26153
402252	14q	104939262	104942618	3357	10	0.2	5'UTR	7102	PLD4	122618
402252	14q	104943622	104945444	1823	7	0.571	Exon(exon6of6)	9958	AHNAK2	113146
402252	14q	104947901	104953878	5978	43	0.512	Promoter(1-2kb)	1524	AHNAK2	113146
402252	15q	20534480	20535129	650	8	0.375	Exon(exon8of9)	6671	GOLGA6L6	727832
402252	15q	23439979	23442067	2089	13	0.538	5'UTR	5167	GOLGA6L2	283685
402252	15q	78766033	78766671	639	9	0.778	Promoter(<=1kb)	-920	ADAMTS7	11173
402252	15q	85579423	85581800	2378	14	0.571	Promoter(1-2kb)	-1110	AKAP13	11214
402252	15q	88857108	88859008	1901	6	0.333	Exon(exon12of18)	9865	ACAN	176
402252	15q	99129423	99132517	3095	6	0.333	Exon(exon4of5)	7225	TTC23	64927
402252	16p	1228744	1229731	988	10	0.7	Promoter(<=1kb)	431	TPSB2	64499
402252	16p	1486371	1488463	2093	8	0.75	Promoter(<=1kb)	4	PTX4	390667
402252	16p	4207130	4208004	875	7	0.571	Exon(exon2of7)	31739	SRL	6345
402252	16q	88428539	88429600	1062	7	0.429	Exon(exon3of3)	-23680	ZFPM1	161882
402252	16q	88430623	88436097	5475	13	0.615	Exon(exon3of3)	-17183	ZFPM1	161882
402252	16q	89100686	89101050	365	8	0.625	Promoter(<=1kb)	24	ACSF3	197322
402252	16q	89226863	89228419	1557	9	0.556	Promoter(2-3kb)	2229	ZNF778	197320
402252	17p	10638198	10641099	2902	7	0.286	Exon(exon19of41)	-8169	MYH3	4621
402252	17p	21300581	21300978	398	11	0.727	3'UTR	9112	MAP2K3	5606
402252	17q	53823368	53824891	1524	6	0.833	Promoter(<=1kb)	441	KIF2B	84643

402252	17q	76293419	76294016	598	6	0.5	Promoter(2-3kb)	-2167	QRICH2	84074
402252	17q	81645135	81645607	473	7	0.286	Promoter(2-3kb)	2722	TSPAN10	83882
402252	18p	11609646	11610350	705	14	0.857	Promoter(<=1kb)	50	SLC35G4	646000
402252	18q	58535186	58538030	2845	19	0.579	Promoter(<=1kb)	0	ALPK2	115701
402252	19p	4510548	4513547	3000	22	0.455	Exon(exon3of6)	4157	PLIN4	729359
402252	19p	5455600	5456439	840	8	0.625	Promoter(<=1kb)	183	ZNRF4	148066
402252	19p	8948231	8952171	3941	13	0.538	Exon(exon3of84)	29171	MUC16	94025
402252	19p	8959403	8961248	1846	6	0.667	Exon(exon3of84)	20094	MUC16	94025
402252	19p	12430718	12432437	1720	8	0.375	3'UTR	8584	ZNF443	10224
402252	19p	17281820	17284246	2427	9	0.556	Promoter(<=1kb)	0	ANKLE1	126549
402252	19p	18264753	18267409	2657	8	0.5	5'UTR	7002	IQCN	80726
402252	19p	21971930	21974500	2571	8	0.625	Exon(exon4of4)	14408	ZNF208	7757
402252	19p	23743906	23745300	1395	6	0.333	Exon(exon4of4)	13537	ZNF681	148213
402252	19q	39877222	39877880	659	6	0.333	Exon(exon20of28)	9412	FCGBP	8857
402252	19q	43913423	43914878	1456	8	0.5	Exon(exon10of10)	4861	ZNF45	7596
402252	19q	44106512	44108078	1567	7	0	Exon(exon6of6)	-4103	ZNF225	7768
402252	19q	51745958	51746963	1006	6	0.333	Exon(exon3of3)	3848	FPR1	2357
402252	19q	52384029	52384992	964	6	0	Exon(exon4of4)	12677	ZNF528-AS1	102724105
402252	19q	52437918	52439242	1325	8	0.5	Exon(exon4of4)	6504	ZNF534	147658
402252	19q	53164551	53166239	1689	6	0.167	Exon(exon4of4)	-5476	ZNF347	84671
402252	19q	55481625	55483456	1832	7	0.429	Promoter(1-2kb)	-1732	NAT14	57106
402252	19q	55911888	55913077	1190	6	0.667	Exon(exon5of12)	19234	NLRP13	126204
402252	19q	58367975	58368875	901	8	0.5	Exon(exon3of3)	-5127	ZNF497	162968
402252	20p	1635288	1636423	1136	6	0.333	Promoter(1-2kb)	1662	SIRPG-AS1	101929010
402252	20p	5922421	5923394	974	6	0.5	Exon(exon4of5)	6923	CHGB	1114
402252	20q	63349752	63350772	1021	6	0.5	3'UTR	3794	CHRNA4	1137
402252	20q	63540866	63543452	2587	10	0.8	Exon(exon1of1)	-3490	PTK6	5753
402252	21q	44637476	44638143	668	6	0.667	Promoter(<=1kb)	120	KRTAP10-10	353333
402252	22q	22352950	22353380	431	16	0.5	Exon(exon1of2)	30478	BMS1P20	96610
402252	22q	36191154	36191906	753	7	0.571	3'UTR	9971	APOL4	80832
402252	23p	8170039	8170141	103	6	0.5	Promoter(1-2kb)	1126	VCX2	51480
402252	23p	35802148	35803010	863	7	0.571	5'UTR	3357	MAGEB16	139604
402252	23q	136874183	136874416	234	8	0.75	Promoter(<=1kb)	-201	RBMX	27316
402252	23q	141906066	141906644	579	14	0.429	Promoter(1-2kb)	1264	MAGEC1	9947
403953	1p	11778784	11779941	1158	7	0.714	Promoter(<=1kb)	0	C1orf167-AS1	102724659
403953	1p	12847526	12847995	470	7	0.429	Promoter(<=1kb)	730	HNRNPCL1	343069
403953	1p	12859036	12860212	1177	14	0.5	Promoter(1-2kb)	1950	PRAMEF2	65122
403953	1p	13370686	13370989	304	8	0.5	Promoter(<=1kb)	911	PRAMEF19	645414
403953	1p	16058491	16059952	1462	7	0.857	Exon(exon5of7)	6168	CLCNKB	1188
403953	1p	18481403	18482217	815	7	0.714	Promoter(<=1kb)	421	KLHDC7A	127707
403953	1p	23874604	23875430	827	8	0.5	Exon(exon2of2)	-6310	FUCA1	2517
403953	1p	40067594	40067675	82	6	0	Promoter(<=1kb)	324	CAP1	10487
403953	1p	89186388	89186419	32	9	0.556	Promoter(<=1kb)	107	GBP4	115361
403953	1q	152303673	152304920	1248	7	0.429	Exon(exon3of3)	-7656	FLG-AS1	339400
403953	1q	152306380	152313891	7512	30	0.567	Promoter(<=1kb)	0	FLG-AS1	339400
403953	1q	158765805	158766655	851	6	0.5	Promoter(<=1kb)	47	OR6N1	128372
403953	1q	223393517	223394466	950	6	0.667	Promoter(<=1kb)	102	CCDC185	164127
403953	1q	228315976	228318026	2051	7	0.571	Exon(exon50of81)	6492	OBSCN	84033
403953	1q	240207775	240208254	480	7	0.714	Exon(exon5of18)	-27517	FMN2	56776
403953	1q	247841312	247841582	271	6	0.833	Promoter(<=1kb)	314	OR11L1	391189
403953	1q	247949325	247949738	414	9	0.333	Promoter(<=1kb)	467	OR2L8	391190
403953	2p	48580657	48582454	1798	7	0.571	Promoter(<=1kb)	0	STON1	11037
403953	2q	184936178	184937636	1459	7	0.429	Exon(exon4of4)	69813	ZNF804A	91752
403953	2q	185789865	185794632	4768	11	0.727	Promoter(<=1kb)	0	FSIP2	401024
403953	2q	217847583	217848559	977	6	0.833	Exon(exon19of33)	-5423	TNS1	7145
403953	2q	233713134	233713733	600	9	0.778	Promoter(<=1kb)	142	UGT1A5	54579
403953	3p	31989532	31990905	1374	6	0.333	Exon(exon2of2)	7761	ZNF860	344787
403953	3p	75737230	75739007	1778	9	0.556	Promoter(<=1kb)	0	MIR4273	100422955
403953	3q	194341097	194342571	1475	7	0.714	Exon(exon2of2)	8747	CPN2	1370
403953	3q	196947388	196948102	715	6	0.5	3'UTR	3828	PIGZ	80235
403953	4p	5988383	5989749	1367	8	0.5	Promoter(<=1kb)	0	C4orf50	389197
403953	4p	6300792	6302360	1569	6	0.833	Exon(exon8of8)	6021	WFS1	7466
403953	4p	8227004	8228508	1505	8	0.125	Promoter(<=1kb)	-24	SH3TC1	54436
403953	4q	112431241	112432293	1053	7	0.429	3'UTR	4735	ALPK1	80216
403953	4q	154489498	154491312	1815	9	0.556	Promoter(<=1kb)	22	DCHS2	54798
403953	4q	186706638	186709751	3114	7	0.714	Exon(exon2of27)	14082	FAT1	2195
403953	5q	79728956	79730716	1761	7	0.286	Exon(exon2of13)	-7426	CMYA5	202333
403953	5q	79731782	79734523	2742	13	0.308	Exon(exon2of13)	-3619	CMYA5	202333
403953	5q	83537326	83539905	2580	6	0.333	Promoter(1-2kb)	1712	VCAN	1462
403953	5q	141174000	141175025	1026	6	0.833	Promoter(1-2kb)	1356	PCDHB7	56129
403953	5q	141955356	141957660	2305	6	0.667	Promoter(<=1kb)	-668	RNF14	9604
403953	5q	151565922	151568158	2237	9	0.778	Promoter(<=1kb)	786	FAT2	2196
403953	6p	1312843	1313745	903	6	0.5	Promoter(<=1kb)	745	FOXQ1	94234
403953	6p	26370344	26370479	136	6	0.5	Promoter(<=1kb)	0	BTN3A2	11118
403953	6p	46858771	46859502	732	8	0.5	Exon(exon17of21)	3802	ADGRF5	221395
403953	6q	64591274	64591961	688	10	0.5	Exon(exon26of43)	121374	EYS	346007
403953	6q	106511549	106512572	1024	6	0.667	Promoter(<=1kb)	0	CRYBG1	202

403953	6q	149888581	149890867	2287	7	0.714	Promoter(<=1kb)	0	RAET1E-AS1	100652739
403953	6q	159231899	159234370	2472	12	0.583	Exon(exon11of23)	13602	FNDC1	84624
403953	7p	6330446	6330944	499	6	1	Exon(exon2of2)	7749	FAM220A	84792
403953	7p	53035678	53036385	708	7	1	Promoter(<=1kb)	45	POM121L12	285877
403953	7q	64991278	64992758	1481	6	0.667	Promoter(<=1kb)	-242	ZNF117	51351
403953	7q	100958721	100960873	2153	55	0.455	Promoter(<=1kb)	756	MUC3A	4584
403953	7q	100991195	100992398	1204	8	0.625	Exon(exon5of15)	-20656	MUC12	10071
403953	7q	100995547	100995896	350	8	0.75	Exon(exon5of15)	-17158	MUC12	10071
403953	7q	149818015	149819792	1778	6	0.667	Promoter(2-3kb)	-2352	SSPO	23145
403953	7q	149824094	149826695	2602	6	0.667	Promoter(<=1kb)	0	SSPO	23145
403953	8p	10607375	10608261	887	6	0.5	Exon(exon4of4)	46882	RP1L1	94137
403953	8p	12132686	12133940	1255	6	0.833	Promoter(<=1kb)	498	USP17L7	392197
403953	8p	12137448	12138641	1194	6	0.833	Promoter(<=1kb)	436	USP17L2	377630
403953	8p	13021128	13022030	903	8	0.125	Exon(exon5of5)	9115	TRMT9B	57604
403953	8q	141218050	141219792	1743	6	0.667	5'UTR	8778	SLC45A4	57210
403953	8q	141466455	141467514	1060	8	0.75	3'UTR	29245	MROH5	389690
403953	9p	34723747	34725745	1999	7	0.571	Exon(exon4of4)	3743	FAM205A	259308
403953	9q	76703555	76706360	2806	8	0.5	Promoter(<=1kb)	0	PCA3	50652
403953	9q	87886533	87888536	2004	7	0.571	Exon(exon4of4)	3656	SPATA31E1	286234
403953	9q	122628595	122629398	804	7	0.286	Promoter(<=1kb)	175	OR1B1	347169
403953	9q	135484695	135487213	2519	9	0.333	Promoter(1-2kb)	1332	PPP1R26	9858
403953	10q	46549378	46550723	1346	26	0.654	Exon(exon3of3)	4807	GPRIN2	9721
403953	10q	125026145	125027175	1031	7	0.571	Promoter(<=1kb)	853	CTBP2	1488
403953	10q	128103129	128104752	1624	8	0.5	Promoter(<=1kb)	-1	MKI67	4288
403953	10q	128106210	128108204	1995	6	1	Exon(exon12of14)	-3082	MKI67	4288
403953	11p	244106	244197	92	8	0.5	Promoter(<=1kb)	-232	PSMD13	5719
403953	11p	5046754	5047432	679	7	0.571	Promoter(<=1kb)	228	OR52J3	119679
403953	11p	5177978	5178478	501	6	0.167	Promoter(<=1kb)	186	OR52Z1	283110
403953	11p	5323362	5324256	895	7	0.429	Promoter(<=1kb)	41	OR51B2	79345
403953	11p	5422212	5423123	912	11	0.636	Promoter(<=1kb)	101	OR51Q1	390061
403953	11p	5581045	5581738	694	8	0.375	Promoter(<=1kb)	168	OR52B6	340980
403953	11p	5841302	5841883	582	9	0.333	Promoter(<=1kb)	14	OR52E6	390078
403953	11p	5884818	5885061	244	7	0.429	Promoter(<=1kb)	547	OR52E4	390081
403953	11p	11352040	11352736	697	8	0.25	Promoter(<=1kb)	514	CSNK2A3	283106
403953	11p	12293628	12294368	741	7	0.857	Exon(exon29of35)	6728	MICALCL	84953
403953	11q	58214757	58215722	966	7	0.286	Promoter(<=1kb)	12	OR1S1	219959
403953	11q	58402523	58403265	743	8	0.5	Promoter(<=1kb)	144	OR5B3	441608
403953	11q	64116513	64118232	1720	7	0.714	Exon(exon2of2)	8702	MACROD1	28992
403953	11q	85724687	85725825	1139	6	0.5	Promoter(<=1kb)	0	SYTL2	54843
403953	11q	123906790	123907324	535	6	0.667	Promoter(<=1kb)	644	OR8D4	338662
403953	11q	123943088	123943782	695	7	0.571	Promoter(<=1kb)	91	OR6T1	219874
403953	11q	124015601	124016477	877	7	0.143	Promoter(<=1kb)	26	OR10G4	390264
403953	11q	124023038	124023849	812	7	0.429	Promoter(<=1kb)	25	OR10G9	219870
403953	11q	124038366	124038988	623	8	1	Promoter(<=1kb)	13	OR10G7	390265
403953	11q	124264847	124265676	830	6	0.833	Promoter(<=1kb)	20	OR8G5	219865
403953	12p	4626568	4628549	1982	11	0.455	Exon(exon5of6)	14051	DYRK4	8798
403953	13q	24434450	24435347	898	7	0.571	Exon(exon31of34)	19787	PARP4	143
403953	13q	25096713	25097231	519	11	0.364	Promoter(<=1kb)	845	PABPC3	5042
403953	13q	102732474	102733933	1460	6	0.333	Exon(exon4of4)	25139	CCDC168	643677
403953	14q	19975713	19976448	736	9	0.444	Promoter(<=1kb)	269	OR4K15	81127
403953	14q	20060048	20060884	837	8	0.625	Promoter(<=1kb)	3	OR4L1	122742
403953	14q	20640982	20641567	586	6	0.5	Promoter(<=1kb)	124	OR6S1	341799
403953	14q	70457745	70458238	494	9	0.333	Exon(exon2of2)	5571	ADAM21	8747
403953	14q	104175275	104177810	2536	9	0.333	Exon(exon12of15)	36235	KIF26A	26153
403953	14q	104939262	104945444	6183	17	0.412	5'UTR	7102	PLD4	122618
403953	14q	104947943	104953878	5936	31	0.452	Promoter(1-2kb)	1524	AHNAK2	113146
403953	15q	23440370	23442067	1698	10	0.7	5'UTR	5167	GOLGA6L2	283685
403953	15q	42690621	42692660	2040	6	0.5	Promoter(1-2kb)	1026	STARD9	57519
403953	15q	59206980	59208023	1044	9	0.556	Promoter(<=1kb)	137	LDHAL6B	92483
403953	15q	78766033	78766638	606	8	0.25	Promoter(<=1kb)	-920	ADAMTS7	11173
403953	15q	85579423	85581800	2378	14	0.571	Promoter(1-2kb)	-1110	AKAP13	11214
403953	15q	88857108	88859365	2258	6	0	Exon(exon12of18)	9865	ACAN	176
403953	15q	99129423	99132517	3095	7	0.429	Exon(exon4of5)	7225	TTC23	64927
403953	15q	100569472	100570097	626	7	0.714	Promoter(<=1kb)	534	LINS1	55180
403953	16p	1229573	1229716	144	7	0.571	Promoter(<=1kb)	446	TPSB2	64499
403953	16p	1256345	1256985	641	14	0.714	Promoter(<=1kb)	276	TPSD1	23430
403953	16p	1486371	1488463	2093	8	0.75	Promoter(<=1kb)	4	PTX4	390667
403953	16p	4207059	4208004	946	7	0.571	Exon(exon2of7)	31739	SRL	6345
403953	16q	74391650	74391897	248	8	0.75	Exon(exon7of7)	13772	NPIPB15	440348
403953	16q	88428539	88429600	1062	6	0.333	Exon(exon3of3)	-23680	ZFPM1	161882
403953	16q	89100686	89101050	365	8	0.625	Promoter(<=1kb)	24	ACSF3	197322
403953	17p	744946	747270	2325	8	0.875	3'UTR	4768	GEMIN4	50628
403953	17p	10638198	10641099	2902	6	0.333	Exon(exon19of41)	-8169	MYH3	4621
403953	17p	21300581	21300978	398	12	0.75	3'UTR	9112	MAP2K3	5606
403953	17p	21415470	21416431	962	9	0.778	Exon(exon3of3)	10334	KCNJ12	3768
403953	17q	53823368	53824891	1524	6	0.833	Promoter(<=1kb)	441	KIF2B	84643
403953	17q	76293419	76294016	598	6	0.5	Promoter(2-3kb)	-2167	QRICH2	84074

403953	17q	78803535	78803816	282	6	0.667	Exon(exon16of21)	-3813	USP36	57602
403953	17q	81645135	81645417	283	6	0.333	Promoter(2-3kb)	2722	TSPAN10	83882
403953	18p	11609728	11610350	623	9	0.778	Promoter(<=1kb)	132	SLC35G4	646000
403953	18p	14542649	14543140	492	6	0.667	Promoter(<=1kb)	6	POTEC	388468
403953	18q	47033929	47035145	1217	6	0.833	Promoter(<=1kb)	476	ELOA2	51224
403953	18q	58535186	58537515	2330	9	0.333	Promoter(<=1kb)	0	ALPK2	115701
403953	19p	4510548	4511943	1396	14	0.429	Exon(exon3of6)	5761	PLIN4	729359
403953	19p	5455600	5456439	840	7	0.571	Promoter(<=1kb)	183	ZNRF4	148066
403953	19p	8333830	8335276	1447	7	0.571	Promoter(<=1kb)	0	KANK3	256949
403953	19p	8946313	8951868	5556	15	0.6	Exon(exon3of84)	29474	MUC16	94025
403953	19p	8959116	8962299	3184	10	0.7	Exon(exon3of84)	19043	MUC16	94025
403953	19p	8972751	8978096	5346	13	0.462	Exon(exon1of84)	3246	MUC16	94025
403953	19p	12430400	12432437	2038	8	0.5	3'UTR	8584	ZNF443	10224
403953	19p	21971930	21974500	2571	7	0.714	Exon(exon4of4)	14408	ZNF208	7757
403953	19q	37886924	37889059	2136	6	0.833	Exon(exon6of6)	17535	WDR87	83889
403953	19q	39877222	39877880	659	6	0.5	Exon(exon20of28)	9412	FCGBP	8857
403953	19q	43913423	43914878	1456	9	0.556	Exon(exon10of10)	4861	ZNF45	7596
403953	19q	43996326	43997366	1041	6	0.5	Exon(exon5of5)	5419	LOC10192806:	101928063
403953	19q	44106512	44108078	1567	7	0	Exon(exon6of6)	-4103	ZNF225	7768
403953	19q	48873325	48875925	2601	10	0.5	Promoter(<=1kb)	904	PPP1R15A	23645
403953	19q	52365769	52366744	976	6	0.833	Exon(exon6of6)	-3173	ZNF880	400713
403953	19q	52384029	52384992	964	7	0.143	Exon(exon4of4)	12677	ZNF528-AS1	102724105
403953	19q	52437918	52439242	1325	7	0.429	Exon(exon4of4)	6504	ZNF534	147658
403953	19q	55481625	55483456	1832	6	0.5	Promoter(1-2kb)	-1732	NAT14	57106
403953	19q	55517821	55518176	356	7	1	Exon(exon14of14)	18127	SBK2	646643
403953	19q	55911888	55913077	1190	6	0.667	Exon(exon5of12)	19234	NLRP13	126204
403953	19q	56190954	56193038	2085	6	1	Promoter(<=1kb)	14	ZSCAN5B	342933
403953	19q	56663753	56665114	1362	6	0.5	Exon(exon2of2)	6643	ZNF835	90485
403953	20p	20052354	20052736	383	6	0	Promoter(<=1kb)	0	CFAP61	26074
403953	20q	63349752	63350772	1021	7	0.429	3'UTR	3794	CHRNA4	1137
403953	20q	63561666	63565531	3866	14	0.714	Promoter(<=1kb)	-61	HELZ2	85441
403953	21q	44600627	44601692	1066	8	0.875	Promoter(<=1kb)	30	KRTAP10-7	386675
403953	21q	44637474	44638041	568	9	0.333	Promoter(<=1kb)	118	KRTAP10-10	353333
403953	22q	22352950	22353348	399	14	0.571	Exon(exon1of2)	30478	BMS1P20	96610
403953	22q	36191154	36191906	753	6	0.667	3'UTR	9971	APOL4	80832
403953	22q	49884187	49884994	808	6	0.333	Exon(exon2of2)	22924	ALG12	79087
403953	23p	3320126	3323750	3625	9	0.556	Exon(exon5of7)	22902	MXRA5	25878
403953	23p	35802148	35803010	863	7	0.571	5'UTR	3357	MAGEB16	139604
404064	1p	978953	979884	932	6	0.5	Promoter(1-2kb)	1145	PERM1	84808
404064	1p	12847526	12847995	470	7	0.571	Promoter(<=1kb)	730	HNRNPCL1	343069
404064	1p	12859036	12860079	1044	6	0.333	Promoter(1-2kb)	1950	PRAMEF2	65122
404064	1p	13370686	13370918	233	6	0.5	Promoter(<=1kb)	982	PRAMEF19	645414
404064	1p	16029143	16030652	1510	7	0.571	Promoter(2-3kb)	2304	CLCNKA	1187
404064	1p	16058491	16059952	1462	8	0.875	Exon(exon5of7)	6168	CLCNKB	1188
404064	1p	18481403	18482217	815	6	0.667	Promoter(<=1kb)	421	KLHDC7A	127707
404064	1p	23874604	23875430	827	8	0.5	Exon(exon2of2)	-6310	FUCA1	2517
404064	1p	40067594	40067675	82	6	0	Promoter(<=1kb)	324	CAP1	10487
404064	1p	89186388	89186419	32	9	0.556	Promoter(<=1kb)	107	GBP4	115361
404064	1q	152303673	152304920	1248	8	0.5	Exon(exon3of3)	-7656	FLG-AS1	339400
404064	1q	152306380	152313891	7512	31	0.581	Promoter(<=1kb)	0	FLG-AS1	339400
404064	1q	156669844	156670886	1043	6	1	Exon(exon4of4)	6521	NES	10763
404064	1q	158765805	158766655	851	6	0.5	Promoter(<=1kb)	47	OR6N1	128372
404064	1q	201206099	201209738	3640	12	0.583	Promoter(1-2kb)	1017	IGFN1	91156
404064	1q	201210956	201212792	1837	6	0.5	Promoter(<=1kb)	0	IGFN1	91156
404064	1q	222628526	222629862	1337	6	0.333	Promoter(<=1kb)	52	MIA3	375056
404064	1q	228315976	228318038	2063	8	0.625	Exon(exon50of81)	6492	OBSCN	84033
404064	1q	247841312	247841582	271	6	0.833	Promoter(<=1kb)	314	OR11L1	391189
404064	1q	247949325	247949738	414	9	0.333	Promoter(<=1kb)	467	OR2L8	391190
404064	2q	95944651	95945173	523	6	0.5	Promoter(<=1kb)	0	ANKRD36C	400986
404064	2q	132783534	132785001	1468	7	0.429	Promoter(1-2kb)	-1511	NCKAP5	344148
404064	2q	184936178	184937636	1459	7	0.429	Exon(exon4of4)	69813	ZNF804A	91752
404064	2q	217847583	217848559	977	6	0.833	Exon(exon19of33)	-5423	TNS1	7145
404064	2q	233840612	233842185	1574	7	0.714	Promoter(<=1kb)	0	HJURP	55355
404064	2q	238130416	238131546	1131	6	0.333	Promoter(1-2kb)	1468	ESPNL	339768
404064	3p	52521941	52524117	2177	6	0.333	Promoter(<=1kb)	0	STAB1	23166
404064	3p	75736880	75739243	2364	54	0.574	Promoter(<=1kb)	0	MIR4273	100422955
404064	4p	5988383	5989749	1367	9	0.667	Promoter(<=1kb)	0	C4orf50	389197
404064	4p	6300792	6302360	1569	6	0.833	Exon(exon8of8)	6021	WFS1	7466
404064	4p	8227004	8228508	1505	8	0.125	Promoter(<=1kb)	-24	SH3TC1	54436
404064	4p	38773164	38774870	1707	7	0.429	Promoter(1-2kb)	1326	TLR10	81793
404064	4q	121036404	121037542	1139	6	0.333	Promoter(1-2kb)	1442	NDNF	79625
404064	4q	154489498	154491312	1815	10	0.6	Promoter(<=1kb)	22	DCHS2	54798
404064	4q	185458217	185460011	1795	9	0.556	Promoter(<=1kb)	0	CCDC110	256309
404064	5p	795818	796237	420	6	0.667	3'UTR	4908	ZDHHC11	79844
404064	5q	79728956	79730716	1761	8	0.25	Exon(exon2of13)	-7426	CMYA5	202333
404064	5q	79731782	79734523	2742	13	0.308	Exon(exon2of13)	-3619	CMYA5	202333
404064	5q	83537326	83539905	2580	6	0.333	Promoter(1-2kb)	1712	VCAN	1462

404064	5q	140848579	140850786	2208	7	0.571	Promoter(<=1kb)	807	PCDHA9	9752
404064	5q	141178745	141180333	1589	7	0.714	Promoter(<=1kb)	955	PCDHB8	56128
404064	5q	141955356	141957660	2305	6	0.667	Promoter(<=1kb)	-668	RNF14	9604
404064	6p	46858771	46859502	732	8	0.5	Exon(exon17of21)	3802	ADGRF5	221395
404064	6q	159233455	159234370	916	10	0.5	Exon(exon11of23)	15158	FNDC1	84624
404064	7p	53035678	53036385	708	7	1	Promoter(<=1kb)	45	POM121L12	285877
404064	7q	64991278	64992758	1481	6	0.667	Promoter(<=1kb)	-242	ZNF117	51351
404064	7q	100958721	100960873	2153	51	0.471	Promoter(<=1kb)	756	MUC3A	4584
404064	7q	100991195	100992398	1204	6	0.5	Exon(exon5of15)	-20656	MUC12	10071
404064	7q	101034305	101038481	4177	25	0.48	Exon(exon3of12)	-5230	MUC17	140453
404064	8p	10607245	10608432	1188	8	0.5	Exon(exon4of4)	46711	RP1L1	94137
404064	8p	10609614	10610662	1049	7	0.429	Exon(exon4of4)	44481	RP1L1	94137
404064	8p	13021128	13022030	903	7	0.143	Exon(exon5of5)	9115	TRMT9B	57604
404064	8q	138151949	138153046	1098	6	0.667	Promoter(<=1kb)	0	FAM135B	51059
404064	8q	144108511	144111231	2721	6	0.667	Promoter(<=1kb)	0	WDR97	340390
404064	9q	76175241	76175296	56	8	0.875	Exon(exon14of14)	-13343	PCSK5	5125
404064	9q	76703451	76707804	4354	14	0.429	Promoter(<=1kb)	0	PCA3	50652
404064	9q	76709263	76710843	1581	8	0.5	Promoter(<=1kb)	0	PRUNE2	158471
404064	9q	122553263	122554071	809	7	0.429	Promoter(<=1kb)	93	OR1N2	138882
404064	9q	122628595	122629398	804	7	0.429	Promoter(<=1kb)	175	OR1B1	347169
404064	9q	135484803	135487573	2771	9	0.333	Promoter(1-2kb)	1440	PPP1R26	9858
404064	9q	135547960	135548795	836	6	0.667	Promoter(1-2kb)	1805	OBP2A	29991
404064	10q	46549378	46550723	1346	26	0.654	Exon(exon3of3)	4807	GPRIN2	9721
404064	10q	49322575	49325572	2998	9	0.556	Exon(exon3of3)	23301	C10orf71	118461
404064	10q	128102594	128105328	2735	7	0.714	Promoter(<=1kb)	0	MKI67	4288
404064	11p	244106	244197	92	8	0.5	Promoter(<=1kb)	-232	PSMD13	5719
404064	11p	1194354	1196902	2549	7	0.571	Exon(exon34of49)	-26164	MUC5B	727897
404064	11p	5177978	5178478	501	6	0.167	Promoter(<=1kb)	186	OR52Z1	283110
404064	11p	5323451	5324256	806	6	0.5	Promoter(<=1kb)	41	OR51B2	79345
404064	11p	5389704	5390350	647	7	0.429	Promoter(<=1kb)	327	OR51M1	390059
404064	11p	5422212	5423123	912	10	0.7	Promoter(<=1kb)	101	OR51Q1	390061
404064	11p	5581045	5581738	694	8	0.375	Promoter(<=1kb)	168	OR52B6	340980
404064	11p	5884818	5885061	244	6	0.333	Promoter(<=1kb)	547	OR52E4	390081
404064	11p	11352040	11352736	697	8	0.25	Promoter(<=1kb)	514	CSNK2A3	283106
404064	11p	12293639	12294368	730	7	0.857	Exon(exon29of35)	6739	MICALCL	84953
404064	11q	58214757	58215722	966	7	0.286	Promoter(<=1kb)	12	OR1S1	219959
404064	11q	64116513	64118232	1720	7	0.714	Exon(exon2of2)	8702	MACROD1	28992
404064	11q	66560202	66562261	2060	6	0.333	Exon(exon14of21)	3873	CTSF	8722
404064	11q	85724687	85725825	1139	6	0.5	Promoter(<=1kb)	0	SYTL2	54843
404064	11q	124015601	124016477	877	7	0.143	Promoter(<=1kb)	26	OR10G4	390264
404064	11q	124264847	124265676	830	6	0.833	Promoter(<=1kb)	20	OR8G5	219865
404064	11q	130914501	130915409	909	10	0.7	Promoter(1-2kb)	1035	SNX19	399979
404064	12p	4626568	4628549	1982	11	0.455	Exon(exon5of6)	14051	DYRK4	8798
404064	12p	8222174	8223514	1341	6	0.667	Exon(exon5of6)	4073	FAM90A1	55138
404064	12q	52571389	52573652	2264	7	0.571	Promoter(<=1kb)	164	KRT74	121391
404064	13q	24434450	24435347	898	7	0.571	Exon(exon31of34)	19787	PARP4	143
404064	13q	25096713	25097231	519	11	0.455	Promoter(<=1kb)	845	PABPC3	5042
404064	13q	102732474	102733933	1460	6	0.333	Exon(exon4of4)	25139	CCDC168	643677
404064	14q	20060048	20060884	837	8	0.625	Promoter(<=1kb)	3	OR4L1	122742
404064	14q	44504986	44506403	1418	6	0.667	Promoter(<=1kb)	880	FSCB	84075
404064	14q	70457532	70458238	707	10	0.3	Exon(exon2of2)	5358	ADAM21	8747
404064	14q	94587512	94587839	328	6	0.5	Exon(exon2of2)	-4219	SERPINA3	12
404064	14q	104947943	104950338	2396	9	0.444	Exon(exon6of6)	5064	AHNAK2	113146
404064	15q	23440370	23442067	1698	9	0.667	5'UTR	5167	GOLGA6L2	283685
404064	15q	42690621	42692660	2040	6	0.5	Promoter(1-2kb)	1026	STARD9	57519
404064	15q	78766033	78766632	600	8	0.25	Promoter(<=1kb)	-920	ADAMTS7	11173
404064	16p	1256354	1256985	632	11	0.636	Promoter(<=1kb)	285	TPSD1	23430
404064	16p	1486371	1488463	2093	9	0.778	Promoter(<=1kb)	4	PTX4	390667
404064	16q	74391650	74391897	248	8	0.75	Exon(exon7of7)	13772	NPIPB15	440348
404064	16q	89100686	89101050	365	8	0.625	Promoter(<=1kb)	24	ACSF3	197322
404064	16q	89226863	89228419	1557	10	0.6	Promoter(2-3kb)	2229	ZNF778	197320
404064	17p	744946	747270	2325	8	0.875	3'UTR	4768	GEMIN4	50628
404064	17p	21300581	21300954	374	11	0.727	3'UTR	9112	MAP2K3	5606
404064	17p	21415458	21416431	974	10	0.8	Exon(exon3of3)	10322	KCNJ12	3768
404064	17q	41586466	41586829	364	6	0.833	Promoter(<=1kb)	66	KRT14	3861
404064	17q	41727098	41728331	1234	6	0.833	Promoter(1-2kb)	-1201	HAP1	9001
404064	17q	76291123	76294016	2894	11	0.455	Promoter(<=1kb)	0	QRICH2	84074
404064	17q	81645135	81645417	283	6	0.333	Promoter(2-3kb)	2722	TSPAN10	83882
404064	18p	9886991	9888072	1082	7	0.857	Promoter(<=1kb)	977	TXNDC2	84203
404064	18p	11609728	11610333	606	7	0.857	Promoter(<=1kb)	132	SLC35G4	646000
404064	18q	58535186	58538030	2845	19	0.526	Promoter(<=1kb)	0	ALPK2	115701
404064	19p	1036457	1036914	458	6	0.5	Exon(exon6of7)	-3187	ABCA7	10347
404064	19p	4510548	4513547	3000	19	0.421	Exon(exon3of6)	4157	PLIN4	729359
404064	19p	5455600	5456439	840	7	0.571	Promoter(<=1kb)	183	ZNRF4	148066
404064	19p	8946092	8951868	5777	16	0.625	Exon(exon3of84)	29474	MUC16	94025
404064	19p	8959116	8962299	3184	10	0.7	Exon(exon3of84)	19043	MUC16	94025
404064	19p	8972751	8978096	5346	14	0.429	Exon(exon1of84)	3246	MUC16	94025

404064	19p	12075333	12077046	1714	6	0.167	Promoter(<=1kb)	6	ZNF788P	388507
404064	19p	15087213	15088040	828	10	0.3	Promoter(<=1kb)	233	OR1I1	126370
404064	19p	18264753	18267409	2657	8	0.5	5'UTR	7002	IQCN	80726
404064	19p	23743906	23745300	1395	6	0.333	Exon(exon4of4)	13537	ZNF681	148213
404064	19q	36996730	36997597	868	10	0.7	Exon(exon10of10)	5677	ZNF568	374900
404064	19q	37151928	37153149	1222	6	0.833	Exon(exon5of5)	19287	ZNF585A	199704
404064	19q	37885190	37888806	3617	8	0.625	Exon(exon6of6)	17788	WDR87	83889
404064	19q	39877222	39877880	659	6	0.5	Exon(exon20of28)	9412	FCGBP	8857
404064	19q	39886005	39886439	435	6	0.333	Promoter(<=1kb)	853	FCGBP	8857
404064	19q	43846955	43848536	1582	6	0.833	3'UTR	13450	ZNF283	284349
404064	19q	43913423	43914878	1456	8	0.5	Exon(exon10of10)	4861	ZNF45	7596
404064	19q	43966037	43967171	1135	6	0.167	Promoter(<=1kb)	-691	ZNF155	7711
404064	19q	43996326	43997366	1041	6	0.5	Exon(exon5of5)	5419	LOC10192806	101928063
404064	19q	44106645	44108078	1434	6	0	Exon(exon6of6)	-4103	ZNF225	7768
404064	19q	52437918	52439242	1325	7	0.429	Exon(exon4of4)	6504	ZNF534	147658
404064	19q	53164551	53166239	1689	6	0.167	Exon(exon4of4)	-5476	ZNF347	84671
404064	19q	55517821	55518176	356	7	1	Exon(exon14of14)	18127	SBK2	646643
404064	19q	55911888	55913077	1190	6	0.667	Exon(exon5of12)	19234	NLRP13	126204
404064	20p	20052354	20052736	383	6	0.167	Promoter(<=1kb)	0	CFAP61	26074
404064	20q	63561666	63564319	2654	8	0.75	Promoter(<=1kb)	-61	HELZ2	85441
404064	21q	44539312	44540035	724	7	0.714	Promoter(<=1kb)	160	KRTAP10-1	386677
404064	21q	44550929	44551452	524	7	0.857	Promoter(<=1kb)	53	KRTAP10-2	386679
404064	21q	44600627	44601692	1066	9	0.778	Promoter(<=1kb)	30	KRTAP10-7	386675
404064	21q	44637474	44638041	568	9	0.333	Promoter(<=1kb)	118	KRTAP10-10	353333
404064	22q	22352950	22353365	416	13	0.462	Exon(exon1of2)	30478	BMS1P20	96610
404064	22q	36191154	36191906	753	6	0.667	3'UTR	9971	APOL4	80832
404064	22q	49884187	49884994	808	6	0.333	Exon(exon2of2)	22924	ALG12	79087
404064	23p	8170039	8170141	103	6	0.5	Promoter(1-2kb)	1126	VCX2	51480
404064	23p	35802148	35803010	863	7	0.571	5'UTR	3357	MAGEB16	139604
406293	1p	11766028	11768307	2280	6	0.833	Promoter(<=1kb)	0	C1orf167	284498
406293	1p	11778784	11779941	1158	7	0.714	Promoter(<=1kb)	0	C1orf167-AS1	102724659
406293	1p	12847526	12847780	255	7	0.429	Promoter(<=1kb)	945	HNRNPCL1	343069
406293	1p	12859036	12860079	1044	6	0.333	Promoter(1-2kb)	1950	PRAMEF2	65122
406293	1p	12893249	12893472	224	7	0.571	Exon(exon4of4)	4798	PRAMEF10	343071
406293	1p	13370686	13371119	434	7	0.429	Promoter(<=1kb)	781	PRAMEF19	645414
406293	1p	16058491	16060000	1510	10	0.9	Exon(exon5of7)	6168	CLCNKB	1188
406293	1p	18481403	18482217	815	7	0.714	Promoter(<=1kb)	421	KLHDC7A	127707
406293	1p	23874604	23875430	827	8	0.5	Exon(exon2of2)	-6310	FUCA1	2517
406293	1p	40067594	40067675	82	6	0	Promoter(<=1kb)	324	CAP1	10487
406293	1p	89186388	89186419	32	9	0.556	Promoter(<=1kb)	107	GBP4	115361
406293	1q	152303673	152313891	10219	36	0.583	Promoter(<=1kb)	0	FLG-AS1	339400
406293	1q	156669844	156670886	1043	6	1	Exon(exon4of4)	6521	NES	10763
406293	1q	169540901	169542882	1982	9	0.222	Exon(exon13of25)	-25156	F5	2153
406293	1q	202318028	202319165	1138	6	0.667	3'UTR	22771	UBE2T	29089
406293	1q	228315976	228317998	2023	6	0.333	Exon(exon50of81)	6492	OBSCN	84033
406293	1q	228371172	228372999	1828	8	0.375	Promoter(<=1kb)	0	OBSCN	84033
406293	1q	247841312	247841582	271	6	0.833	Promoter(<=1kb)	314	OR11L1	391189
406293	1q	247949325	247949738	414	7	0.143	Promoter(<=1kb)	467	OR2L8	391190
406293	1q	248294677	248295458	782	9	0.667	Promoter(<=1kb)	142	OR2T12	127064
406293	1q	248681658	248682198	541	7	0.571	Promoter(<=1kb)	130	OR14I1	401994
406293	2p	48580657	48582454	1798	7	0.571	Promoter(<=1kb)	0	STON1	11037
406293	2q	130193975	130194376	402	6	0.833	Exon(exon4of5)	4063	TUBA3E	112714
406293	2q	184936178	184937636	1459	6	0.333	Exon(exon4of4)	69813	ZNF804A	91752
406293	2q	217847583	217848559	977	6	0.833	Exon(exon19of33)	-5423	TNS1	7145
406293	2q	232379271	232380431	1161	6	0.333	Promoter(<=1kb)	520	ALPP	250
406293	2q	233713134	233713664	531	9	0.778	Promoter(<=1kb)	142	UGT1A5	54579
406293	2q	238130416	238131546	1131	6	0.333	Promoter(1-2kb)	1468	ESPNL	339768
406293	2q	240041845	240042811	967	7	0.286	Downstream(2-3kb)	3261	OR6B3	150681
406293	3p	31989532	31990905	1374	8	0.375	Exon(exon2of2)	7761	ZNF860	344787
406293	3p	75737230	75739007	1778	9	0.556	Promoter(<=1kb)	0	MIR4273	100422955
406293	3q	98264413	98265098	686	7	0.571	Promoter(<=1kb)	128	OR5H6	79295
406293	3q	194341097	194342571	1475	6	0.667	Exon(exon2of2)	8747	CPN2	1370
406293	4p	5988383	5989749	1367	8	0.5	Promoter(<=1kb)	0	C4orf50	389197
406293	4p	6300792	6302360	1569	6	0.833	Exon(exon8of8)	6021	WFS1	7466
406293	4p	8227004	8228508	1505	8	0.125	Promoter(<=1kb)	-24	SH3TC1	54436
406293	4q	112431241	112432293	1053	7	0.429	3'UTR	4735	ALPK1	80216
406293	4q	121036404	121037542	1139	6	0.333	Promoter(1-2kb)	1442	NDNF	79625
406293	4q	154489498	154491312	1815	9	0.556	Promoter(<=1kb)	22	DCHS2	54798
406293	4q	186619481	186621582	2102	6	0.333	Exon(exon10of27)	-9411	FAT1	2195
406293	4q	186706638	186708616	1979	6	0.667	Exon(exon2of27)	15217	FAT1	2195
406293	5q	79728956	79734523	5568	19	0.368	Exon(exon2of13)	-3619	CMYA5	202333
406293	5q	140807352	140807737	386	6	0.833	Promoter(<=1kb)	271	PCDHA4	56144
406293	5q	140848579	140850786	2208	6	0.5	Promoter(<=1kb)	807	PCDHA9	9752
406293	5q	141955356	141957660	2305	6	0.667	Promoter(<=1kb)	-668	RNF14	9604
406293	5q	148826877	148828070	1194	6	1	Promoter(1-2kb)	1632	ADRB2	154
406293	5q	151521550	151522069	520	6	0.667	Promoter(<=1kb)	79	MIR6499	102465246
406293	5q	151565922	151568158	2237	9	0.778	Promoter(<=1kb)	786	FAT2	2196

406293	6p	46858771	46859389	619	6	0.5	Exon(exon17of21)	3915	ADGRF5	221395
406293	6q	159231899	159234370	2472	12	0.583	Exon(exon11of23)	13602	FNDC1	84624
406293	7q	64991278	64992758	1481	6	0.667	Promoter(<=1kb)	-242	ZNF117	51351
406293	7q	100958977	100960873	1897	56	0.446	Promoter(1-2kb)	1012	MUC3A	4584
406293	7q	100991195	100993127	1933	8	0.625	Exon(exon5of15)	-19927	MUC12	10071
406293	7q	100995575	100995785	211	6	0.833	Exon(exon5of15)	-17269	MUC12	10071
406293	7q	101032095	101038994	6900	36	0.444	Exon(exon3of12)	-4717	MUC17	140453
406293	8p	8376561	8377198	638	6	1	Exon(exon2of5)	4549	PRAG1	157285
406293	8p	10607375	10612307	4933	15	0.667	Exon(exon4of4)	42836	RP1L1	94137
406293	8p	13021128	13022030	903	8	0.25	Exon(exon5of5)	9115	TRMT9B	57604
406293	8q	142664552	142665852	1301	6	0.833	Exon(exon2of2)	4118	JRK	8629
406293	9p	116800	117713	914	7	0.714	Promoter(<=1kb)	491	FOXD4	2298
406293	9q	76175237	76175296	60	10	0.8	Exon(exon14of14)	-13343	PCSK5	5125
406293	9q	76705179	76707804	2626	8	0.5	Promoter(<=1kb)	121	PCA3	50652
406293	9q	76709263	76710843	1581	8	0.5	Promoter(<=1kb)	0	PRUNE2	158471
406293	9q	104504315	104505071	757	6	0.5	Promoter(<=1kb)	52	OR13F1	138805
406293	9q	122553278	122554071	794	7	0.429	Promoter(<=1kb)	108	OR1N2	138882
406293	9q	122628595	122629398	804	7	0.286	Promoter(<=1kb)	175	OR1B1	347169
406293	9q	122749914	122750547	634	6	0.833	Promoter(<=1kb)	174	OR1L6	392390
406293	9q	124855684	124856809	1126	7	0.571	Promoter(2-3kb)	2208	WDR38	401551
406293	9q	133255635	133256264	630	8	1	3'UTR	18950	ABO	28
406293	9q	135484803	135487213	2411	12	0.5	Promoter(1-2kb)	1440	PPP1R26	9858
406293	9q	135547960	135548795	836	8	0.625	Promoter(1-2kb)	1805	OBP2A	29991
406293	10q	46549378	46550723	1346	25	0.64	Exon(exon3of3)	4807	GPRIN2	9721
406293	10q	128103129	128104830	1702	10	0.6	Promoter(<=1kb)	-1	MKI67	4288
406293	11p	244106	244197	92	8	0.5	Promoter(<=1kb)	-232	PSMD13	5719
406293	11p	1194354	1196902	2549	7	0.571	Exon(exon34of49)	-26164	MUC5B	727897
406293	11p	1246095	1247378	1284	9	0.444	Promoter(2-3kb)	2298	MUC5B-AS1	112577518
406293	11p	5177978	5178478	501	6	0.167	Promoter(<=1kb)	186	OR52Z1	283110
406293	11p	5323362	5324256	895	7	0.429	Promoter(<=1kb)	41	OR51B2	79345
406293	11p	5389704	5390350	647	7	0.429	Promoter(<=1kb)	327	OR51M1	390059
406293	11p	5422212	5423123	912	11	0.636	Promoter(<=1kb)	101	OR51Q1	390061
406293	11p	5515185	5516015	831	6	0.333	Promoter(<=1kb)	684	UBQLNL	143630
406293	11p	5581045	5581738	694	8	0.375	Promoter(<=1kb)	168	OR52B6	340980
406293	11p	5841302	5841883	582	9	0.333	Promoter(<=1kb)	14	OR52E6	390078
406293	11p	11351961	11352736	776	9	0.222	Promoter(<=1kb)	514	CSNK2A3	283106
406293	11p	12293639	12294538	900	8	0.625	Exon(exon29of35)	6739	MICALCL	84953
406293	11q	58214757	58215722	966	7	0.286	Promoter(<=1kb)	12	OR1S1	219959
406293	11q	66560202	66562261	2060	6	0.333	Exon(exon14of21)	3873	CTSF	8722
406293	11q	82732630	82733184	555	6	0.833	Promoter(<=1kb)	680	FAM181B	220382
406293	11q	85724687	85725825	1139	6	0.5	Promoter(<=1kb)	0	SYTL2	54843
406293	11q	123906790	123907324	535	6	0.667	Promoter(<=1kb)	644	OR8D4	338662
406293	11q	124015600	124016477	878	7	0.143	Promoter(<=1kb)	25	OR10G4	390264
406293	11q	124038366	124038988	623	9	0.889	Promoter(<=1kb)	13	OR10G7	390265
406293	11q	124185670	124186080	411	6	0.333	Promoter(<=1kb)	454	OR10D3	26497
406293	12p	4627329	4628549	1221	8	0.625	Exon(exon5of6)	14812	DYRK4	8798
406293	12p	6453119	6453670	552	6	0.667	Promoter(<=1kb)	633	TAPBPL	55080
406293	13q	25096713	25097182	470	17	0.471	Promoter(<=1kb)	845	PABPC3	5042
406293	13q	102732474	102733933	1460	6	0.333	Exon(exon4of4)	25139	CCDC168	643677
406293	14q	20060048	20060884	837	8	0.625	Promoter(<=1kb)	3	OR4L1	122742
406293	14q	20640982	20641567	586	6	0.5	Promoter(<=1kb)	124	OR6S1	341799
406293	14q	21634137	21634589	453	9	0.556	Promoter(<=1kb)	351	OR10G2	26534
406293	14q	22633879	22634450	572	9	0.333	Exon(exon2of2)	32212	ABHD4	63874
406293	14q	70457532	70459101	1570	12	0.25	Exon(exon2of2)	5358	ADAM21	8747
406293	14q	104175275	104177810	2536	10	0.3	Exon(exon12of15)	36235	KIF26A	26153
406293	15q	20534501	20535129	629	6	0.5	Exon(exon8of9)	6671	GOLGA6L6	727832
406293	15q	23439979	23442067	2089	12	0.5	5'UTR	5167	GOLGA6L2	283685
406293	15q	40621642	40623696	2055	6	0.333	Promoter(<=1kb)	0	KNL1	57082
406293	15q	42690621	42692660	2040	6	0.5	Promoter(1-2kb)	1026	STARD9	57519
406293	15q	85579423	85581800	2378	14	0.571	Promoter(1-2kb)	-1110	AKAP13	11214
406293	15q	88856792	88859365	2574	10	0.3	Exon(exon12of18)	9549	ACAN	176
406293	15q	99129423	99132517	3095	6	0.333	Exon(exon4of5)	7225	TTC23	64927
406293	16p	1228744	1229731	988	10	0.7	Promoter(<=1kb)	431	TPSB2	64499
406293	16p	1256345	1256980	636	7	0.857	Promoter(<=1kb)	276	TPSD1	23430
406293	16p	1486371	1488463	2093	8	0.75	Promoter(<=1kb)	4	PTX4	390667
406293	16p	4207130	4208004	875	6	0.5	Exon(exon2of7)	31739	SRL	6345
406293	16q	74391650	74391928	279	7	0.714	Exon(exon7of7)	13772	NPIPB15	440348
406293	16q	89100686	89101050	365	7	0.571	Promoter(<=1kb)	24	ACSF3	197322
406293	16q	89226863	89228289	1427	8	0.625	Promoter(2-3kb)	2229	ZNF778	197320
406293	17p	5558351	5559959	1609	6	0.833	Exon(exon4of17)	23998	NLRP1	22861
406293	17p	21300581	21300978	398	11	0.727	3'UTR	9112	MAP2K3	5606
406293	17p	21415470	21416404	935	8	0.875	Exon(exon3of3)	10334	KCNJ12	3768
406293	17q	53823368	53824891	1524	6	0.833	Promoter(<=1kb)	441	KIF2B	84643
406293	17q	76291123	76294016	2894	11	0.455	Promoter(<=1kb)	0	QRICH2	84074
406293	17q	81645135	81645595	461	7	0.429	Promoter(2-3kb)	2722	TSPAN10	83882
406293	18p	11609646	11610383	738	13	0.846	Promoter(<=1kb)	50	SLC35G4	646000
406293	18p	14542649	14543140	492	6	0.5	Promoter(<=1kb)	6	POTEC	388468

406293	18q	58535186	58537515	2330	9	0.333	Promoter(<=1kb)	0	ALPK2	115701
406293	19p	4510548	4513547	3000	22	0.455	Exon(exon3of6)	4157	PLIN4	729359
406293	19p	5455724	5456439	716	6	0.5	Promoter(<=1kb)	307	ZNRF4	148066
406293	19p	8946313	8951868	5556	15	0.6	Exon(exon3of84)	29474	MUC16	94025
406293	19p	8959116	8962299	3184	11	0.636	Exon(exon3of84)	19043	MUC16	94025
406293	19p	8972751	8978096	5346	13	0.462	Exon(exon1of84)	3246	MUC16	94025
406293	19p	12075333	12077046	1714	7	0.286	Promoter(<=1kb)	6	ZNF788P	388507
406293	19p	15087213	15088040	828	10	0.3	Promoter(<=1kb)	233	OR1I1	126370
406293	19p	17281820	17284246	2427	9	0.556	Promoter(<=1kb)	0	ANKLE1	126549
406293	19p	18264753	18267409	2657	8	0.5	5'UTR	7002	IQCN	80726
406293	19p	21971930	21974500	2571	8	0.75	Exon(exon4of4)	14408	ZNF208	7757
406293	19p	23743906	23745300	1395	6	0.333	Exon(exon4of4)	13537	ZNF681	148213
406293	19q	37886924	37889059	2136	6	0.833	Exon(exon6of6)	17535	WDR87	83889
406293	19q	39886240	39886422	183	6	0.667	Promoter(<=1kb)	870	FCGBP	8857
406293	19q	43846955	43848536	1582	6	0.833	3'UTR	13450	ZNF283	284349
406293	19q	43913423	43914878	1456	9	0.556	Exon(exon10of10)	4861	ZNF45	7596
406293	19q	43966037	43967171	1135	6	0.167	Promoter(<=1kb)	-691	ZNF155	7711
406293	19q	43996326	43997366	1041	6	0.5	Exon(exon5of5)	5419	LOC10192806[illegible]	101928063
406293	19q	44106512	44108078	1567	7	0	Exon(exon6of6)	-4103	ZNF225	7768
406293	19q	44327836	44329698	1863	6	0.5	Exon(exon4of4)	-22790	ZNF235	9310
406293	19q	51745958	51746963	1006	6	0.333	Exon(exon3of3)	3848	FPR1	2357
406293	19q	52365769	52366744	976	6	0.833	Exon(exon6of6)	-3173	ZNF880	400713
406293	19q	52384029	52384992	964	7	0.143	Exon(exon4of4)	12677	ZNF528-AS1	102724105
406293	19q	52437918	52439242	1325	7	0.429	Exon(exon4of4)	6504	ZNF534	147658
406293	19q	55358736	55359651	916	6	0.667	Promoter(<=1kb)	0	FAM71E2	284418
406293	19q	55771923	55773169	1247	6	0.5	Promoter(2-3kb)	2782	RFPL4AL1	729974
406293	19q	55911888	55913077	1190	6	0.667	Exon(exon5of12)	19234	NLRP13	126204
406293	19q	58367898	58368875	978	8	0.5	Exon(exon3of3)	-5050	ZNF497	162968
406293	20p	5922421	5923394	974	8	0.5	Exon(exon4of5)	6923	CHGB	1114
406293	20q	63561666	63565531	3866	11	0.636	Promoter(<=1kb)	-61	HELZ2	85441
406293	21q	26843740	26844859	1120	6	0.667	Promoter(<=1kb)	0	ADAMTS1	9510
406293	21q	44591429	44592359	931	8	0.375	Promoter(<=1kb)	146	KRTAP10-6	386674
406293	22q	22352950	22353380	431	16	0.5	Exon(exon1of2)	30478	BMS1P20	96610
406293	22q	36191154	36191906	753	8	0.625	3'UTR	9971	APOL4	80832
406293	22q	49883704	49884994	1291	6	0.167	Exon(exon2of2)	22924	ALG12	79087
406293	23p	8170039	8170141	103	6	0.5	Promoter(1-2kb)	1126	VCX2	51480
406293	23p	35802148	35803010	863	7	0.571	5'UTR	3357	MAGEB16	139604
406293	23p	38285569	38287917	2349	7	1	3'UTR	-11627	RPGR	6103
406516	1p	978953	979847	895	6	0.5	Promoter(1-2kb)	1182	PERM1	84808
406516	1p	12847526	12847780	255	7	0.429	Promoter(<=1kb)	945	HNRNPCL1	343069
406516	1p	12859036	12860079	1044	6	0.333	Promoter(1-2kb)	1950	PRAMEF2	65122
406516	1p	12893249	12893472	224	6	0.5	Exon(exon4of4)	4798	PRAMEF10	343071
406516	1p	13369166	13369564	399	7	0.714	Promoter(2-3kb)	2336	PRAMEF19	645414
406516	1p	13370686	13371119	434	7	0.429	Promoter(<=1kb)	781	PRAMEF19	645414
406516	1p	16058491	16060000	1510	13	0.846	Exon(exon5of7)	6168	CLCNKB	1188
406516	1p	18481403	18482217	815	6	0.667	Promoter(<=1kb)	421	KLHDC7A	127707
406516	1p	23874604	23875430	827	8	0.5	Exon(exon2of2)	-6310	FUCA1	2517
406516	1p	40067594	40067675	82	6	0	Promoter(<=1kb)	324	CAP1	10487
406516	1p	74470245	74472144	1900	6	0.333	Exon(exon5of5)	31180	FPGT-TNNI3K	100526835
406516	1p	89186388	89186419	32	9	0.556	Promoter(<=1kb)	107	GBP4	115361
406516	1q	145872200	145873487	1288	6	0.667	Exon(exon8of12)	-12364	PIAS3	10401
406516	1q	152213286	152213347	62	8	0.5	Exon(exon3of3)	10846	HRNR	388697
406516	1q	152218928	152221375	2448	16	0.688	Promoter(2-3kb)	2818	HRNR	388697
406516	1q	152304395	152306226	1832	7	0.429	Exon(exon3of3)	-6350	FLG-AS1	339400
406516	1q	152308456	152309531	1076	8	0.625	Exon(exon3of3)	-3045	FLG-AS1	339400
406516	1q	152310646	152313526	2881	9	0.667	Promoter(<=1kb)	0	FLG-AS1	339400
406516	1q	158765805	158766655	851	6	0.5	Promoter(<=1kb)	47	OR6N1	128372
406516	1q	169542317	169542882	566	6	0.167	Exon(exon13of25)	-26572	F5	2153
406516	1q	197101312	197101771	460	6	0.5	Exon(exon18of28)	33373	ASPM	259266
406516	1q	201206099	201209342	3244	10	0.6	Promoter(1-2kb)	1017	IGFN1	91156
406516	1q	232805207	232806505	1299	6	0.333	Promoter(<=1kb)	315	MAP10	54627
406516	1q	247841312	247841582	271	6	0.833	Promoter(<=1kb)	314	OR11L1	391189
406516	1q	247949443	247949738	296	9	0.333	Promoter(<=1kb)	585	OR2L8	391190
406516	1q	248294677	248295458	782	6	0.667	Promoter(<=1kb)	142	OR2T12	127064
406516	2p	48580657	48582454	1798	7	0.571	Promoter(<=1kb)	0	STON1	11037
406516	2q	130193975	130194376	402	6	0.833	Exon(exon4of5)	4063	TUBA3E	112714
406516	2q	130914528	130916922	2395	6	0.333	Promoter(<=1kb)	0	ARHGEF4	50649
406516	2q	178739433	178741811	2379	6	0.5	Exon(exon45of191)	26014	TTN	7273
406516	2q	184936178	184937815	1638	6	0.333	Exon(exon4of4)	69813	ZNF804A	91752
406516	2q	185790999	185794632	3634	9	0.778	Promoter(<=1kb)	0	FSIP2	401024
406516	2q	185805377	185808170	2794	7	0.286	Promoter(<=1kb)	0	FSIP2	401024
406516	2q	238130271	238131702	1432	12	0.583	Promoter(1-2kb)	1323	ESPNL	339768
406516	2q	240041845	240042131	287	6	0.167	Downstream(2-3kb)	3941	OR6B3	150681
406516	3p	52521941	52524117	2177	6	0.333	Promoter(<=1kb)	0	STAB1	23166
406516	3p	75736929	75739007	2079	14	0.643	Promoter(<=1kb)	0	MIR4273	100422955
406516	4p	5988383	5989749	1367	7	0.571	Promoter(<=1kb)	0	C4orf50	389197
406516	4p	6300792	6302360	1569	6	0.833	Exon(exon8of8)	6021	WFS1	7466

406516	4p	10443803	10446224	2422	6	0.333	Exon(exon3of3)	10952	ZNF518B	85460
406516	4q	121036404	121037542	1139	6	0.333	Promoter(1-2kb)	1442	NDNF	79625
406516	4q	186619481	186621582	2102	6	0.333	Exon(exon10of27)	-9411	FAT1	2195
406516	4q	186706638	186708616	1979	6	0.667	Exon(exon2of27)	15217	FAT1	2195
406516	5q	83537326	83539905	2580	6	0.333	Promoter(1-2kb)	1712	VCAN	1462
406516	5q	140807352	140807737	386	6	0.833	Promoter(<=1kb)	271	PCDHA4	56144
406516	5q	141955676	141957660	1985	6	0.667	Promoter(<=1kb)	-668	RNF14	9604
406516	6p	26370344	26370479	136	6	0.5	Promoter(<=1kb)	0	BTN3A2	11118
406516	6p	46858771	46859502	732	8	0.5	Exon(exon17of21)	3802	ADGRF5	221395
406516	6q	64591274	64591961	688	10	0.5	Exon(exon26of43)	121374	EYS	346007
406516	6q	159231899	159234370	2472	10	0.5	Exon(exon11of23)	13602	FNDC1	84624
406516	7p	6330446	6330944	499	6	1	Exon(exon2of2)	7749	FAM220A	84792
406516	7p	12369637	12370736	1100	6	0.667	3'UTR	-13307	VWDE	221806
406516	7q	100958721	100960873	2153	55	0.455	Promoter(<=1kb)	756	MUC3A	4584
406516	7q	100991195	100993102	1908	8	0.625	Exon(exon5of15)	-19952	MUC12	10071
406516	7q	100995575	100995922	348	9	0.667	Exon(exon5of15)	-17132	MUC12	10071
406516	7q	101003685	101004836	1152	7	0.571	Exon(exon5of15)	-8218	MUC12	10071
406516	7q	101034361	101040583	6223	36	0.5	Exon(exon3of12)	-3128	MUC17	140453
406516	8p	13021128	13022030	903	9	0.111	Exon(exon5of5)	9115	TRMT9B	57604
406516	8p	17754377	17755366	990	6	0.333	Promoter(<=1kb)	0	MTUS1	57509
406516	8q	141466429	141467514	1086	9	0.778	3'UTR	29245	MROH5	389690
406516	9p	34723747	34726473	2727	6	0.833	Exon(exon4of4)	3015	FAM205A	259308
406516	9q	76703555	76706360	2806	8	0.5	Promoter(<=1kb)	0	PCA3	50652
406516	9q	104598545	104599361	817	13	0.538	Promoter(<=1kb)	52	OR13C5	138799
406516	9q	122553263	122554071	809	8	0.5	Promoter(<=1kb)	93	OR1N2	138882
406516	9q	122628595	122629130	536	6	0.333	Promoter(<=1kb)	443	OR1B1	347169
406516	9q	122749914	122750547	634	6	0.833	Promoter(<=1kb)	174	OR1L6	392390
406516	9q	135484803	135487213	2411	8	0.25	Promoter(1-2kb)	1440	PPP1R26	9858
406516	10q	46549378	46550723	1346	25	0.64	Exon(exon3of3)	4807	GPRIN2	9721
406516	10q	49323559	49326554	2996	10	0.5	Exon(exon3of3)	24285	C10orf71	118461
406516	10q	122084988	122087840	2853	8	0.75	Exon(exon4of23)	-25190	TACC2	10579
406516	10q	128102594	128106296	3703	10	0.7	Promoter(<=1kb)	0	MKI67	4288
406516	11p	244106	244197	92	8	0.5	Promoter(<=1kb)	-232	PSMD13	5719
406516	11p	1246332	1248605	2274	10	0.5	Promoter(1-2kb)	1071	MUC5B-AS1	112577518
406516	11p	5177978	5178478	501	6	0.167	Promoter(<=1kb)	186	OR52Z1	283110
406516	11p	5323362	5324256	895	8	0.5	Promoter(<=1kb)	41	OR51B2	79345
406516	11p	5351521	5352416	896	15	0.467	Promoter(<=1kb)	13	OR51B6	390058
406516	11p	5389419	5390350	932	8	0.5	Promoter(<=1kb)	42	OR51M1	390059
406516	11p	5422212	5423123	912	10	0.7	Promoter(<=1kb)	101	OR51Q1	390061
406516	11p	5581045	5581738	694	8	0.375	Promoter(<=1kb)	168	OR52B6	340980
406516	11p	5841302	5841883	582	9	0.333	Promoter(<=1kb)	14	OR52E6	390078
406516	11p	5884818	5885061	244	7	0.429	Promoter(<=1kb)	547	OR52E4	390081
406516	11p	11351961	11352736	776	8	0.25	Promoter(<=1kb)	514	CSNK2A3	283106
406516	11p	12293639	12294538	900	6	0.667	Exon(exon29of35)	6739	MICALCL	84953
406516	11q	58214757	58215722	966	8	0.25	Promoter(<=1kb)	12	OR1S1	219959
406516	11q	58402523	58403265	743	8	0.5	Promoter(<=1kb)	144	OR5B3	441608
406516	11q	64116513	64118232	1720	7	0.714	Exon(exon2of2)	8702	MACROD1	28992
406516	11q	82732630	82733396	767	7	0.857	Promoter(<=1kb)	468	FAM181B	220382
406516	11q	85724687	85725825	1139	6	0.5	Promoter(<=1kb)	0	SYTL2	54843
406516	11q	124382526	124383285	760	8	0.625	Promoter(<=1kb)	58	OR8B2	26595
406516	11q	130914501	130915409	909	10	0.7	Promoter(1-2kb)	1035	SNX19	399979
406516	12p	4626568	4627524	957	7	0.429	Exon(exon5of6)	14051	DYRK4	8798
406516	12p	6453119	6453670	552	7	0.714	Promoter(<=1kb)	633	TAPBPL	55080
406516	12q	52316096	52317765	1670	6	0.667	Exon(exon4of9)	3633	KRT83	3889
406516	13q	25096877	25097260	384	11	0.455	Promoter(1-2kb)	1009	PABPC3	5042
406516	13q	49668389	49668431	43	6	0.167	Exon(exon3of3)	23007	EBPL	84650
406516	13q	102732474	102733933	1460	6	0.333	Exon(exon4of4)	25139	CCDC168	643677
406516	14q	20060048	20060884	837	8	0.625	Promoter(<=1kb)	3	OR4L1	122742
406516	14q	21634137	21634644	508	7	0.429	Promoter(<=1kb)	296	OR10G2	26534
406516	14q	22633879	22634450	572	9	0.333	Exon(exon2of2)	32212	ABHD4	63874
406516	14q	70457532	70458540	1009	9	0.333	Exon(exon2of2)	5358	ADAM21	8747
406516	14q	104939262	104942618	3357	10	0.2	5'UTR	7102	PLD4	122618
406516	14q	104943622	104945444	1823	7	0.571	Exon(exon6of6)	9958	AHNAK2	113146
406516	14q	104948292	104949883	1592	11	0.636	Exon(exon6of6)	5519	AHNAK2	113146
406516	14q	104951428	104953878	2451	14	0.5	Promoter(1-2kb)	1524	AHNAK2	113146
406516	15q	23439979	23442067	2089	12	0.583	5'UTR	5167	GOLGA6L2	283685
406516	15q	40621642	40625415	3774	9	0.333	Promoter(<=1kb)	0	KNL1	57082
406516	15q	88854874	88855594	721	6	0.833	Exon(exon12of18)	7631	ACAN	176
406516	15q	88857108	88859365	2258	6	0	Exon(exon12of18)	9865	ACAN	176
406516	16p	1256345	1256896	552	6	0.667	Promoter(<=1kb)	276	TPSD1	23430
406516	16p	28495551	28497395	1845	6	0.333	Promoter(<=1kb)	0	CLN3	1201
406516	16q	74391416	74392004	589	10	0.6	Exon(exon7of7)	13538	NPIPB15	440348
406516	16q	88428339	88429600	1262	6	0.333	Exon(exon3of3)	-23680	ZFPM1	161882
406516	16q	89226863	89228289	1427	7	0.571	Promoter(2-3kb)	2229	ZNF778	197320
406516	17p	744917	746966	2050	8	0.875	3'UTR	5072	GEMIN4	50628
406516	17p	10638198	10641099	2902	7	0.286	Exon(exon19of41)	-8169	MYH3	4621
406516	17p	21300581	21300954	374	10	0.7	3'UTR	9112	MAP2K3	5606

406516	17p	21415470	21416431	962	15	0.733	Exon(exon3of3)	10334	KCNJ12	3768
406516	17q	76293419	76294016	598	6	0.5	Promoter(2-3kb)	-2167	QRICH2	84074
406516	17q	81645135	81645607	473	7	0.286	Promoter(2-3kb)	2722	TSPAN10	83882
406516	18p	11609637	11610581	945	10	0.8	Promoter(<=1kb)	41	SLC35G4	646000
406516	18q	58535186	58538030	2845	19	0.579	Promoter(<=1kb)	0	ALPK2	115701
406516	18q	75285722	75287404	1683	6	0.333	Exon(exon2of2)	41146	TSHZ1	10194
406516	19p	4510548	4513547	3000	21	0.476	Exon(exon3of6)	4157	PLIN4	729359
406516	19p	5455600	5456439	840	7	0.571	Promoter(<=1kb)	183	ZNRF4	148066
406516	19p	8948231	8951868	3638	10	0.5	Exon(exon3of84)	29474	MUC16	94025
406516	19p	12430157	12432437	2281	7	0.286	3'UTR	8584	ZNF443	10224
406516	19p	18264753	18267409	2657	8	0.5	5'UTR	7002	IQCN	80726
406516	19p	21971930	21974500	2571	7	0.714	Exon(exon4of4)	14408	ZNF208	7757
406516	19q	39877222	39877880	659	6	0.5	Exon(exon20of28)	9412	FCGBP	8857
406516	19q	39886240	39886422	183	6	0.667	Promoter(<=1kb)	870	FCGBP	8857
406516	19q	43913423	43914878	1456	8	0.5	Exon(exon10of10)	4861	ZNF45	7596
406516	19q	44106512	44108078	1567	7	0	Exon(exon6of6)	-4103	ZNF225	7768
406516	19q	44327836	44329698	1863	6	0.5	Exon(exon4of4)	-22790	ZNF235	9310
406516	19q	52437918	52439242	1325	7	0.429	Exon(exon4of4)	6504	ZNF534	147658
406516	19q	55160006	55162286	2281	6	0.333	Promoter(<=1kb)	0	DNAAF3	352909
406516	19q	55481625	55483456	1832	7	0.429	Promoter(1-2kb)	-1732	NAT14	57106
406516	19q	55517821	55518176	356	7	1	Exon(exon14of14)	18127	SBK2	646643
406516	19q	55911888	55913077	1190	6	0.667	Exon(exon5of12)	19234	NLRP13	126204
406516	20p	1635288	1636423	1136	6	0.333	Promoter(1-2kb)	1662	SIRPG-AS1	101929010
406516	20p	5922677	5923382	706	6	0.5	Exon(exon4of5)	7179	CHGB	1114
406516	20q	63561666	63565531	3866	12	0.667	Promoter(<=1kb)	-61	HELZ2	85441
406516	21q	44550835	44551416	582	6	0.833	Promoter(<=1kb)	89	KRTAP10-2	386679
406516	21q	44637474	44638041	568	11	0.364	Promoter(<=1kb)	118	KRTAP10-10	353333
406516	21q	44666460	44666841	382	7	0.714	Promoter(<=1kb)	86	KRTAP12-2	353323
406516	22q	22352950	22353298	349	13	0.538	Exon(exon1of2)	30478	BMS1P20	96610
406516	22q	22758758	22759209	452	6	0.833	Exon(exon1of2)	-63567	MIR650	723778
406516	22q	36191154	36191906	753	6	0.667	3'UTR	9971	APOL4	80832
406516	22q	39100331	39102033	1703	6	0.833	Promoter(<=1kb)	52	APOBEC3H	164668
406516	23p	8170039	8170141	103	6	0.5	Promoter(1-2kb)	1126	VCX2	51480
406516	23q	136874183	136874416	234	6	1	Promoter(<=1kb)	-201	RBMX	27316
407306	1p	978953	979884	932	6	0.5	Promoter(1-2kb)	1145	PERM1	84808
407306	1p	11766028	11768307	2280	6	0.833	Promoter(<=1kb)	0	C1orf167	284498
407306	1p	12795695	12795957	263	6	0.5	Promoter(1-2kb)	1004	PRAMEF1	65121
407306	1p	12847526	12848054	529	11	0.364	Promoter(<=1kb)	671	HNRNPCL1	343069
407306	1p	12859036	12860079	1044	6	0.333	Promoter(1-2kb)	1950	PRAMEF2	65122
407306	1p	13370686	13371119	434	9	0.444	Promoter(<=1kb)	781	PRAMEF19	645414
407306	1p	16058491	16060000	1510	13	0.846	Exon(exon5of7)	6168	CLCNKB	1188
407306	1p	18481403	18482217	815	6	0.667	Promoter(<=1kb)	421	KLHDC7A	127707
407306	1p	23874604	23875430	827	9	0.556	Exon(exon2of2)	-6310	FUCA1	2517
407306	1p	40067594	40067675	82	6	0	Promoter(<=1kb)	324	CAP1	10487
407306	1p	89186388	89186419	32	9	0.556	Promoter(<=1kb)	107	GBP4	115361
407306	1q	152219233	152221375	2143	16	0.75	Promoter(2-3kb)	2818	HRNR	388697
407306	1q	156669844	156671745	1902	7	1	Exon(exon4of4)	5662	NES	10763
407306	1q	169542317	169542882	566	6	0.167	Exon(exon13of25)	-26572	F5	2153
407306	1q	223393517	223394466	950	6	0.667	Promoter(<=1kb)	102	CCDC185	164127
407306	1q	228315976	228318026	2051	8	0.625	Exon(exon50of81)	6492	OBSCN	84033
407306	1q	247841312	247841582	271	6	0.833	Promoter(<=1kb)	314	OR11L1	391189
407306	1q	247949325	247949738	414	10	0.3	Promoter(<=1kb)	467	OR2L8	391190
407306	2p	48580657	48582454	1798	7	0.571	Promoter(<=1kb)	0	STON1	11037
407306	2q	132783032	132785012	1981	7	0.571	Promoter(1-2kb)	-1009	NCKAP5	344148
407306	2q	184936178	184937636	1459	6	0.333	Exon(exon4of4)	69813	ZNF804A	91752
407306	2q	185789865	185794632	4768	10	0.8	Promoter(<=1kb)	0	FSIP2	401024
407306	2q	185805377	185808170	2794	6	0.333	Promoter(<=1kb)	0	FSIP2	401024
407306	2q	217847583	217848642	1060	7	0.857	Exon(exon19of33)	-5423	TNS1	7145
407306	2q	233840612	233842185	1574	6	0.667	Promoter(<=1kb)	0	HJURP	55355
407306	2q	238130392	238131546	1155	6	0.667	Promoter(1-2kb)	1444	ESPNL	339768
407306	3p	75736880	75739243	2364	69	0.667	Promoter(<=1kb)	0	MIR4273	100422955
407306	3q	98264413	98265098	686	7	0.571	Promoter(<=1kb)	128	OR5H6	79295
407306	4p	5988383	5989749	1367	7	0.571	Promoter(<=1kb)	0	C4orf50	389197
407306	4p	6300792	6302360	1569	6	0.833	Exon(exon8of8)	6021	WFS1	7466
407306	4p	8227004	8228508	1505	8	0.125	Promoter(<=1kb)	-24	SH3TC1	54436
407306	4q	154489498	154491312	1815	9	0.556	Promoter(<=1kb)	22	DCHS2	54798
407306	4q	186619481	186621582	2102	6	0.333	Exon(exon10of27)	-9411	FAT1	2195
407306	5p	796157	796237	81	6	0.333	3'UTR	4908	ZDHHC11	79844
407306	5q	79728956	79730716	1761	6	0.333	Exon(exon2of13)	-7426	CMYA5	202333
407306	5q	79731782	79734523	2742	14	0.357	Exon(exon2of13)	-3619	CMYA5	202333
407306	5q	79736843	79738839	1997	6	0.833	Promoter(<=1kb)	0	CMYA5	202333
407306	5q	83537326	83539905	2580	6	0.333	Promoter(1-2kb)	1712	VCAN	1462
407306	5q	140807352	140807737	386	6	0.833	Promoter(<=1kb)	271	PCDHA4	56144
407306	5q	140848579	140850786	2208	6	0.5	Promoter(<=1kb)	807	PCDHA9	9752
407306	5q	141174000	141175025	1026	6	0.833	Promoter(1-2kb)	1356	PCDHB7	56129
407306	5q	141183999	141184688	690	6	0.833	Promoter(2-3kb)	-2473	PCDHB9	56127
407306	5q	141187690	141189425	1736	6	0.5	Promoter(<=1kb)	529	PCDHB9	56127

407306	5q	148826877	148828070	1194	6	1	Promoter(1-2kb)	1632	ADRB2	154
407306	5q	151521550	151522069	520	6	0.667	Promoter(<=1kb)	79	MIR6499	102465246
407306	5q	151565922	151568158	2237	9	0.778	Promoter(<=1kb)	786	FAT2	2196
407306	6p	1312843	1313745	903	6	0.5	Promoter(<=1kb)	745	FOXQ1	94234
407306	6p	16327099	16327837	739	7	0.286	Exon(exon8of9)	37154	GMPR	2766
407306	6p	46858771	46859502	732	7	0.571	Exon(exon17of21)	3802	ADGRF5	221395
407306	6q	149888581	149889987	1407	6	0.667	Promoter(<=1kb)	-116	RAET1E-AS1	100652739
407306	6q	159231899	159234370	2472	12	0.583	Exon(exon11of23)	13602	FNDC1	84624
407306	7p	6330446	6330944	499	6	1	Exon(exon2of2)	7749	FAM220A	84792
407306	7p	38353718	38353991	274	8	0.625	Exon(exon2of2)	3699	TRG-AS1	100506776
407306	7p	45082725	45084866	2142	8	0.5	Promoter(1-2kb)	-1325	NACAD	23148
407306	7p	53035678	53036385	708	7	1	Promoter(<=1kb)	45	POM121L12	285877
407306	7p	56021087	56021209	123	6	0.5	Exon(exon2of7)	12947	PSPH	5723
407306	7q	100958721	100960873	2153	55	0.473	Promoter(<=1kb)	756	MUC3A	4584
407306	7q	100991195	100992398	1204	8	0.625	Exon(exon5of15)	-20656	MUC12	10071
407306	7q	100995547	100996058	512	10	0.9	Exon(exon5of15)	-16996	MUC12	10071
407306	7q	101003685	101004836	1152	6	0.667	Exon(exon5of15)	-8218	MUC12	10071
407306	7q	149818015	149819792	1778	6	0.667	Promoter(2-3kb)	-2352	SSPO	23145
407306	7q	149824094	149826495	2402	6	0.667	Promoter(<=1kb)	0	SSPO	23145
407306	8p	10609614	10612307	2694	10	0.9	Exon(exon4of4)	42836	RP1L1	94137
407306	8p	13021128	13022030	903	7	0.143	Exon(exon5of5)	9115	TRMT9B	57604
407306	9p	712060	713307	1248	7	0.714	Exon(exon7of16)	5171	KANK1	23189
407306	9q	76703451	76707804	4354	14	0.429	Promoter(<=1kb)	0	PCA3	50652
407306	9q	76709263	76710843	1581	8	0.5	Promoter(<=1kb)	0	PRUNE2	158471
407306	9q	87886533	87888819	2287	6	0.667	Exon(exon4of4)	3656	SPATA31E1	286234
407306	9q	104598641	104599361	721	8	0.625	Promoter(<=1kb)	52	OR13C5	138799
407306	9q	122553263	122554071	809	7	0.429	Promoter(<=1kb)	93	OR1N2	138882
407306	9q	122628595	122629130	536	6	0.333	Promoter(<=1kb)	443	OR1B1	347169
407306	9q	122749914	122750547	634	6	0.833	Promoter(<=1kb)	174	OR1L6	392390
407306	9q	133255635	133256205	571	11	0.909	3'UTR	19009	ABO	28
407306	9q	135484803	135487213	2411	8	0.25	Promoter(1-2kb)	1440	PPP1R26	9858
407306	10q	46549378	46550723	1346	26	0.654	Exon(exon3of3)	4807	GPRIN2	9721
407306	10q	49323169	49325572	2404	8	0.5	Exon(exon3of3)	23895	C10orf71	118461
407306	10q	128102594	128106296	3703	8	0.625	Promoter(<=1kb)	0	MKI67	4288
407306	11p	244106	244197	92	8	0.5	Promoter(<=1kb)	-232	PSMD13	5719
407306	11p	1246332	1247378	1047	7	0.571	Promoter(2-3kb)	2298	MUC5B-AS1	112577518
407306	11p	4954783	4955581	799	6	0.667	Promoter(<=1kb)	132	OR51A2	401667
407306	11p	5177978	5178478	501	6	0.167	Promoter(<=1kb)	186	OR52Z1	283110
407306	11p	5323451	5324256	806	6	0.5	Promoter(<=1kb)	41	OR51B2	79345
407306	11p	5389704	5390350	647	6	0.5	Promoter(<=1kb)	327	OR51M1	390059
407306	11p	5422212	5423123	912	11	0.636	Promoter(<=1kb)	101	OR51Q1	390061
407306	11p	5440761	5441472	712	6	0.833	Promoter(<=1kb)	42	OR51I1	390063
407306	11p	5544676	5545259	584	6	0.5	Promoter(<=1kb)	290	OR52H1	390067
407306	11p	5581045	5581738	694	8	0.375	Promoter(<=1kb)	168	OR52B6	340980
407306	11p	5884818	5885061	244	6	0.333	Promoter(<=1kb)	547	OR52E4	390081
407306	11p	11351961	11352736	776	9	0.222	Promoter(<=1kb)	514	CSNK2A3	283106
407306	11p	12293639	12294368	730	6	0.833	Exon(exon29of35)	6739	MICALCL	84953
407306	11p	18173280	18173901	622	6	0.333	Promoter(<=1kb)	443	MRGPRX4	117196
407306	11q	55827393	55827640	248	7	0.714	Promoter(<=1kb)	174	OR5L2	26338
407306	11q	58214757	58215722	966	8	0.25	Promoter(<=1kb)	12	OR1S1	219959
407306	11q	85724687	85725825	1139	6	0.5	Promoter(<=1kb)	0	SYTL2	54843
407306	11q	124015600	124016477	878	7	0.143	Promoter(<=1kb)	25	OR10G4	390264
407306	11q	124382526	124383285	760	8	0.625	Promoter(<=1kb)	58	OR8B2	26595
407306	12p	4626571	4628549	1979	10	0.5	Exon(exon5of6)	14054	DYRK4	8798
407306	12p	8222174	8223514	1341	6	0.667	Exon(exon5of6)	4073	FAM90A1	55138
407306	12q	49795819	49797137	1319	6	0.5	Promoter(<=1kb)	0	NCKAP5L	57701
407306	13q	24434450	24435347	898	7	0.571	Exon(exon31of34)	19787	PARP4	143
407306	13q	25096713	25097182	470	30	0.567	Promoter(<=1kb)	845	PABPC3	5042
407306	13q	102732474	102733933	1460	6	0.333	Exon(exon4of4)	25139	CCDC168	643677
407306	14q	19975713	19976448	736	9	0.444	Promoter(<=1kb)	269	OR4K15	81127
407306	14q	20060048	20060884	837	9	0.667	Promoter(<=1kb)	3	OR4L1	122742
407306	14q	20640750	20641567	818	7	0.571	Promoter(<=1kb)	124	OR6S1	341799
407306	14q	21634137	21634589	453	9	0.556	Promoter(<=1kb)	351	OR10G2	26534
407306	14q	22633879	22634450	572	8	0.375	Exon(exon2of2)	32212	ABHD4	63874
407306	14q	44504986	44506403	1418	6	0.667	Promoter(<=1kb)	880	FSCB	84075
407306	14q	70457520	70458540	1021	13	0.385	Exon(exon2of2)	5346	ADAM21	8747
407306	14q	94587512	94587839	328	6	0.5	Exon(exon2of2)	-4219	SERPINA3	12
407306	14q	104175275	104177810	2536	11	0.364	Exon(exon12of15)	36235	KIF26A	26153
407306	15q	23440160	23442067	1908	13	0.615	5'UTR	5167	GOLGA6L2	283685
407306	15q	40621642	40623696	2055	6	0.333	Promoter(<=1kb)	0	KNL1	57082
407306	15q	85579423	85582073	2651	16	0.625	Promoter(<=1kb)	-837	AKAP13	11214
407306	16p	1228744	1229731	988	10	0.7	Promoter(<=1kb)	431	TPSB2	64499
407306	16p	1256345	1256980	636	7	0.857	Promoter(<=1kb)	276	TPSD1	23430
407306	16q	74391416	74391897	482	6	0.667	Exon(exon7of7)	13538	NPIPB15	440348
407306	16q	88428255	88429600	1346	6	0.333	Exon(exon3of3)	-23680	ZFPM1	161882
407306	16q	88714717	88717113	2397	8	0.5	Promoter(<=1kb)	0	MIR4722	100616167
407306	16q	89226863	89228419	1557	10	0.6	Promoter(2-3kb)	2229	ZNF778	197320

407306	17p	744917	746966	2050	8	0.875	3'UTR	5072	GEMIN4	50628
407306	17p	21300581	21300978	398	11	0.727	3'UTR	9112	MAP2K3	5606
407306	17p	21415470	21416404	935	8	0.875	Exon(exon3of3)	10334	KCNJ12	3768
407306	17q	81645135	81645607	473	7	0.286	Promoter(2-3kb)	2722	TSPAN10	83882
407306	18p	11609646	11610509	864	17	0.647	Promoter(<=1kb)	50	SLC35G4	646000
407306	18q	58535186	58537515	2330	10	0.4	Promoter(<=1kb)	0	ALPK2	115701
407306	19p	4510548	4513547	3000	17	0.471	Exon(exon3of6)	4157	PLIN4	729359
407306	19p	5455600	5456631	1032	9	0.667	Promoter(<=1kb)	183	ZNRF4	148066
407306	19p	8333830	8335281	1452	8	0.625	Promoter(<=1kb)	0	KANK3	256949
407306	19p	8936591	8939050	2460	9	1	Exon(exon5of84)	-40501	MUC16	94025
407306	19p	8946254	8947220	967	9	0.444	Exon(exon3of84)	34122	MUC16	94025
407306	19p	8948231	8950136	1906	6	0.667	Exon(exon3of84)	31206	MUC16	94025
407306	19p	8959518	8962066	2549	6	0.333	Exon(exon3of84)	19276	MUC16	94025
407306	19p	8964274	8967127	2854	19	0.632	Exon(exon3of84)	14215	MUC16	94025
407306	19p	14766987	14767045	59	7	0.857	Exon(exon6of21)	5329	ADGRE2	30817
407306	19p	18264753	18267409	2657	8	0.5	5'UTR	7002	IQCN	80726
407306	19p	21971930	21974500	2571	7	0.714	Exon(exon4of4)	14408	ZNF208	7757
407306	19p	23743906	23745300	1395	6	0.333	Exon(exon4of4)	13537	ZNF681	148213
407306	19q	39877222	39877880	659	6	0.5	Exon(exon20of28)	9412	FCGBP	8857
407306	19q	39886240	39886422	183	7	0.714	Promoter(<=1kb)	870	FCGBP	8857
407306	19q	40880231	40880622	392	6	0.333	Promoter(<=1kb)	-141	CYP2A7	1549
407306	19q	43846955	43848536	1582	6	0.833	3'UTR	13450	ZNF283	284349
407306	19q	43913423	43914878	1456	8	0.5	Exon(exon10of10)	4861	ZNF45	7596
407306	19q	43996326	43997366	1041	6	0.5	Exon(exon5of5)	5419	LOC10192806?	101928063
407306	19q	44106512	44108078	1567	8	0.125	Exon(exon6of6)	-4103	ZNF225	7768
407306	19q	44327836	44329698	1863	6	0.5	Exon(exon4of4)	-22790	ZNF235	9310
407306	19q	47679530	47681217	1688	6	0.667	Promoter(<=1kb)	568	BICRA	29998
407306	19q	52384029	52384992	964	7	0.143	Exon(exon4of4)	12677	ZNF528-AS1	102724105
407306	19q	52437918	52439242	1325	7	0.429	Exon(exon4of4)	6504	ZNF534	147658
407306	19q	55517821	55518642	822	9	1	Exon(exon14of14)	17661	SBK2	646643
407306	19q	55911888	55913077	1190	6	0.667	Exon(exon5of12)	19234	NLRP13	126204
407306	19q	58416932	58417769	838	6	0.5	3'UTR	8155	ZNF584	201514
407306	20p	5922421	5923394	974	9	0.556	Exon(exon4of5)	6923	CHGB	1114
407306	20p	18315086	18316549	1464	6	0.667	Promoter(2-3kb)	2450	ZNF133	7692
407306	21q	26843740	26844859	1120	6	0.667	Promoter(<=1kb)	0	ADAMTS1	9510
407306	21q	44558207	44558709	503	6	0.5	Promoter(<=1kb)	86	KRTAP10-3	386682
407306	21q	44573798	44574611	814	6	0.667	Promoter(<=1kb)	0	TSPEAR	54084
407306	21q	44637474	44638143	670	10	0.4	Promoter(<=1kb)	118	KRTAP10-10	353333
407306	21q	44666460	44666841	382	7	0.714	Promoter(<=1kb)	86	KRTAP12-2	353323
407306	22q	22352950	22353365	416	15	0.533	Exon(exon1of2)	30478	BMS1P20	96610
407306	22q	22758758	22759209	452	6	0.833	Exon(exon1of2)	-63567	MIR650	723778
407306	22q	36191154	36191906	753	6	0.667	3'UTR	9971	APOL4	80832
407306	22q	49884187	49884994	808	6	0.333	Exon(exon2of2)	22924	ALG12	79087
407306	23p	3320126	3323750	3625	10	0.6	Exon(exon5of7)	22902	MXRA5	25878
407306	23p	8170039	8170141	103	6	0.5	Promoter(1-2kb)	1126	VCX2	51480
407306	23p	35802148	35803010	863	8	0.625	5'UTR	3357	MAGEB16	139604
410837	1p	11766028	11768307	2280	6	0.833	Promoter(<=1kb)	0	C1orf167	284498
410837	1p	11778784	11779941	1158	7	0.714	Promoter(<=1kb)	0	C1orf167-AS1	102724659
410837	1p	12893180	12893472	293	8	0.25	Exon(exon4of4)	4798	PRAMEF10	343071
410837	1p	16058491	16060000	1510	12	0.833	Exon(exon5of7)	6168	CLCNKB	1188
410837	1p	18481403	18482217	815	7	0.714	Promoter(<=1kb)	421	KLHDC7A	127707
410837	1p	23874604	23875430	827	8	0.5	Exon(exon2of2)	-6310	FUCA1	2517
410837	1p	40067594	40067675	82	6	0	Promoter(<=1kb)	324	CAP1	10487
410837	1p	89186388	89186419	32	9	0.556	Promoter(<=1kb)	107	GBP4	115361
410837	1q	152213274	152213320	47	8	0.5	Exon(exon3of3)	10873	HRNR	388697
410837	1q	152218469	152221375	2907	16	0.688	Promoter(2-3kb)	2818	HRNR	388697
410837	1q	152303673	152313891	10219	38	0.605	Promoter(<=1kb)	0	FLG-AS1	339400
410837	1q	158765805	158766655	851	6	0.5	Promoter(<=1kb)	47	OR6N1	128372
410837	1q	169542317	169542882	566	6	0.167	Exon(exon13of25)	-26572	F5	2153
410837	1q	197101312	197101771	460	6	0.5	Exon(exon18of28)	33373	ASPM	259266
410837	1q	223393517	223394466	950	6	0.667	Promoter(<=1kb)	102	CCDC185	164127
410837	1q	228315976	228318026	2051	6	0.5	Exon(exon50of81)	6492	OBSCN	84033
410837	1q	232805207	232806505	1299	7	0.429	Promoter(<=1kb)	315	MAP10	54627
410837	1q	247841312	247841582	271	6	0.833	Promoter(<=1kb)	314	OR11L1	391189
410837	1q	247949325	247949738	414	9	0.333	Promoter(<=1kb)	467	OR2L8	391190
410837	1q	248273309	248273670	362	7	0.571	Promoter(<=1kb)	166	OR2T33	391195
410837	1q	248294677	248295458	782	7	0.571	Promoter(<=1kb)	142	OR2T12	127064
410837	2p	48580657	48582454	1798	7	0.571	Promoter(<=1kb)	0	STON1	11037
410837	2q	102351547	102351902	356	7	0.429	Exon(exon11of11)	-4027	IL18R1	8809
410837	2q	130193975	130194376	402	7	0.571	Exon(exon4of5)	4063	TUBA3E	112714
410837	2q	132781993	132784972	2980	8	0.5	Promoter(<=1kb)	0	NCKAP5	344148
410837	2q	167246794	167248478	1685	7	0.714	Promoter(<=1kb)	-204	XIRP2	129446
410837	2q	178739433	178741811	2379	6	0.5	Exon(exon45of191)	26014	TTN	7273
410837	2q	184936178	184937636	1459	6	0.333	Exon(exon4of4)	69813	ZNF804A	91752
410837	2q	185789865	185794632	4768	10	0.8	Promoter(<=1kb)	0	FSIP2	401024
410837	2q	185805377	185808170	2794	7	0.429	Promoter(<=1kb)	0	FSIP2	401024
410837	2q	233713134	233713783	650	13	0.692	Promoter(<=1kb)	142	UGT1A5	54579

410837	2q	233840612	233842185	1574	6	0.667	Promoter(<=1kb)	0	HJURP	55355
410837	2q	238130271	238131546	1276	7	0.286	Promoter(1-2kb)	1323	ESPNL	339768
410837	3p	31989532	31990905	1374	7	0.286	Exon(exon2of2)	7761	ZNF860	344787
410837	3p	75736929	75739007	2079	15	0.6	Promoter(<=1kb)	0	MIR4273	100422955
410837	3q	98264413	98265098	686	7	0.571	Promoter(<=1kb)	128	OR5H6	79295
410837	3q	196947878	196948468	591	6	0.5	3'UTR	3462	PIGZ	80235
410837	4p	5988383	5989749	1367	8	0.625	Promoter(<=1kb)	0	C4orf50	389197
410837	4p	6300792	6302360	1569	6	0.833	Exon(exon8of8)	6021	WFS1	7466
410837	4p	8227004	8228508	1505	8	0.125	Promoter(<=1kb)	-24	SH3TC1	54436
410837	4q	186617176	186621582	4407	12	0.417	Exon(exon10of27)	-7106	FAT1	2195
410837	5q	79731782	79734523	2742	13	0.308	Exon(exon2of13)	-3619	CMYA5	202333
410837	5q	140802063	140803473	1411	6	0.5	Promoter(1-2kb)	1006	PCDHA3	56145
410837	5q	140807352	140807737	386	6	0.833	Promoter(<=1kb)	271	PCDHA4	56144
410837	5q	140848579	140850786	2208	7	0.571	Promoter(<=1kb)	807	PCDHA9	9752
410837	5q	141174000	141175025	1026	6	0.833	Promoter(1-2kb)	1356	PCDHB7	56129
410837	5q	141183999	141184688	690	6	0.833	Promoter(2-3kb)	-2473	PCDHB9	56127
410837	5q	141187690	141189425	1736	7	0.571	Promoter(<=1kb)	529	PCDHB9	56127
410837	5q	141955356	141957660	2305	7	0.714	Promoter(<=1kb)	-668	RNF14	9604
410837	6p	16327099	16327837	739	6	0.333	Exon(exon8of9)	37154	GMPR	2766
410837	6p	46858771	46859502	732	7	0.571	Exon(exon17of21)	3802	ADGRF5	221395
410837	6q	64591274	64591961	688	10	0.5	Exon(exon26of43)	121374	EYS	346007
410837	6q	149888581	149890867	2287	7	0.714	Promoter(<=1kb)	0	RAET1E-AS1	100652739
410837	6q	159233455	159234370	916	10	0.5	Exon(exon11of23)	15158	FNDC1	84624
410837	7p	53035678	53036385	708	8	1	Promoter(<=1kb)	45	POM121L12	285877
410837	7q	100958977	100960873	1897	55	0.473	Promoter(1-2kb)	1012	MUC3A	4584
410837	7q	100991195	100993127	1933	9	0.667	Exon(exon5of15)	-19927	MUC12	10071
410837	7q	100995575	100995785	211	6	0.833	Exon(exon5of15)	-17269	MUC12	10071
410837	7q	149818015	149819792	1778	6	0.667	Promoter(2-3kb)	-2352	SSPO	23145
410837	8p	8376561	8377198	638	6	1	Exon(exon2of5)	4549	PRAG1	157285
410837	8p	10607375	10612307	4933	15	0.6	Exon(exon4of4)	42836	RP1L1	94137
410837	8p	11331234	11332082	849	6	0.667	Promoter(<=1kb)	346	SLC35G5	83650
410837	8p	12132686	12133940	1255	6	0.667	Promoter(<=1kb)	498	USP17L7	392197
410837	8q	141466429	141467514	1086	9	0.778	3'UTR	29245	MROH5	389690
410837	9p	116800	117934	1135	7	0.857	Promoter(<=1kb)	270	FOXD4	2298
410837	9p	39078723	39078846	124	7	0.714	Exon(exon22of24)	7302	CNTNAP3	79937
410837	9q	76703555	76706360	2806	8	0.5	Promoter(<=1kb)	0	PCA3	50652
410837	9q	87887543	87888819	1277	6	0.5	Exon(exon4of4)	4666	SPATA31E1	286234
410837	9q	104598545	104599361	817	12	0.583	Promoter(<=1kb)	52	OR13C5	138799
410837	9q	122749914	122750547	634	6	0.833	Promoter(<=1kb)	174	OR1L6	392390
410837	9q	128907497	128909159	1663	7	0.429	Exon(exon3of4)	-3585	LRRC8A	56262
410837	9q	135484803	135487213	2411	9	0.333	Promoter(1-2kb)	1440	PPP1R26	9858
410837	9q	135547960	135548795	836	8	0.625	Promoter(1-2kb)	1805	OBP2A	29991
410837	10q	46549378	46550723	1346	25	0.64	Exon(exon3of3)	4807	GPRIN2	9721
410837	10q	49323169	49326817	3649	11	0.545	Exon(exon3of3)	23895	C10orf71	118461
410837	10q	89737450	89738561	1112	6	0	Exon(exon20of33)	13874	KIF20B	9585
410837	10q	103602059	103602530	472	6	0.667	Exon(exon15of15)	30382	NEURL1	9148
410837	10q	125026145	125027175	1031	6	0.5	Promoter(<=1kb)	853	CTBP2	1488
410837	11p	244106	244197	92	8	0.5	Promoter(<=1kb)	-232	PSMD13	5719
410837	11p	1246332	1247378	1047	6	0.5	Promoter(2-3kb)	2298	MUC5B-AS1	112577518
410837	11p	5177978	5178478	501	6	0.167	Promoter(<=1kb)	186	OR52Z1	283110
410837	11p	5323542	5324256	715	6	0.5	Promoter(<=1kb)	41	OR51B2	79345
410837	11p	5389704	5390350	647	6	0.5	Promoter(<=1kb)	327	OR51M1	390059
410837	11p	5402638	5403322	685	9	0.444	Promoter(<=1kb)	41	OR51J1	79470
410837	11p	5515185	5516015	831	6	0.333	Promoter(<=1kb)	684	UBQLNL	143630
410837	11p	5581045	5581738	694	8	0.375	Promoter(<=1kb)	168	OR52B6	340980
410837	11p	5884818	5885061	244	6	0.333	Promoter(<=1kb)	547	OR52E4	390081
410837	11p	11351961	11352736	776	9	0.222	Promoter(<=1kb)	514	CSNK2A3	283106
410837	11p	12293639	12294842	1204	7	0.714	Exon(exon29of35)	6739	MICALCL	84953
410837	11p	34916266	34916763	498	6	0.667	Promoter(<=1kb)	0	APIP	51074
410837	11q	54603136	54603820	685	6	0.667	Promoter(<=1kb)	178	OR4C46	119749
410837	11q	58214757	58215722	966	8	0.25	Promoter(<=1kb)	12	OR1S1	219959
410837	11q	64315797	64315856	60	8	0.75	Promoter(1-2kb)	1485	TRMT112	51504
410837	11q	85724479	85725825	1347	7	0.571	Promoter(<=1kb)	0	SYTL2	54843
410837	11q	124382526	124383285	760	8	0.625	Promoter(<=1kb)	58	OR8B2	26595
410837	11q	130914501	130915432	932	11	0.636	Promoter(1-2kb)	1012	SNX19	399979
410837	12p	4626571	4628549	1979	9	0.444	Exon(exon5of6)	14054	DYRK4	8798
410837	12p	6453119	6453670	552	6	0.667	Promoter(<=1kb)	633	TAPBPL	55080
410837	13q	24434450	24435347	898	7	0.571	Exon(exon31of34)	19787	PARP4	143
410837	13q	25096850	25097182	333	11	0.455	Promoter(<=1kb)	982	PABPC3	5042
410837	13q	102732474	102733933	1460	6	0.333	Exon(exon4of4)	25139	CCDC168	643677
410837	14q	20060048	20060884	837	8	0.625	Promoter(<=1kb)	3	OR4L1	122742
410837	14q	21634137	21634589	453	9	0.556	Promoter(<=1kb)	351	OR10G2	26534
410837	14q	44504986	44506403	1418	6	0.667	Promoter(<=1kb)	880	FSCB	84075
410837	14q	70457520	70458540	1021	12	0.333	Exon(exon2of2)	5346	ADAM21	8747
410837	14q	103110058	103110438	381	6	0.667	Promoter(2-3kb)	2436	EXOC3L4	91828
410837	14q	104939262	104945444	6183	23	0.391	5'UTR	7102	PLD4	122618
410837	14q	104946867	104950810	3944	20	0.55	Exon(exon6of6)	4592	AHNAK2	113146

410837	15q	23440160	23442067	1908	12	0.667	5'UTR	5167	GOLGA6L2	283685
410837	15q	42690621	42692660	2040	6	0.5	Promoter(1-2kb)	1026	STARD9	57519
410837	15q	85579423	85582073	2651	15	0.6	Promoter(<=1kb)	-837	AKAP13	11214
410837	15q	88854874	88855594	721	6	0.833	Exon(exon12of18)	7631	ACAN	176
410837	15q	88857108	88859365	2258	6	0	Exon(exon12of18)	9865	ACAN	176
410837	16p	669592	672548	2957	7	0.429	Promoter(<=1kb)	0	RHOT2	89941
410837	16p	768555	770938	2384	10	0.5	Promoter(<=1kb)	0	MIR662	724032
410837	16p	1228744	1229622	879	6	0.667	Promoter(<=1kb)	540	TPSB2	64499
410837	16p	4207130	4208004	875	6	0.5	Exon(exon2of7)	31739	SRL	6345
410837	16p	28495551	28497395	1845	6	0.333	Promoter(<=1kb)	0	CLN3	1201
410837	16q	74391401	74391897	497	9	0.778	Exon(exon7of7)	13523	NPIPB15	440348
410837	16q	89100686	89101050	365	7	0.571	Promoter(<=1kb)	24	ACSF3	197322
410837	16q	89226863	89228289	1427	8	0.625	Promoter(2-3kb)	2229	ZNF778	197320
410837	17p	744917	746966	2050	8	0.875	3'UTR	5072	GEMIN4	50628
410837	17p	10638198	10641099	2902	7	0.286	Exon(exon19of41)	-8169	MYH3	4621
410837	17p	21300581	21300954	374	8	0.75	3'UTR	9112	MAP2K3	5606
410837	17p	21415470	21416550	1081	7	1	Exon(exon3of3)	10334	KCNJ12	3768
410837	17q	53823368	53824891	1524	6	0.833	Promoter(<=1kb)	441	KIF2B	84643
410837	17q	73236548	73236991	444	6	0.5	Promoter(<=1kb)	0	FAM104A	84923
410837	17q	81645135	81645417	283	6	0.333	Promoter(2-3kb)	2722	TSPAN10	83882
410837	18p	11609646	11610509	864	15	0.667	Promoter(<=1kb)	50	SLC35G4	646000
410837	18q	47033473	47035259	1787	10	1	Promoter(<=1kb)	362	ELOA2	51224
410837	18q	58535186	58538030	2845	19	0.579	Promoter(<=1kb)	0	ALPK2	115701
410837	19p	4510548	4513547	3000	21	0.429	Exon(exon3of6)	4157	PLIN4	729359
410837	19p	5455600	5456439	840	6	0.5	Promoter(<=1kb)	183	ZNRF4	148066
410837	19p	8948231	8952171	3941	12	0.5	Exon(exon3of84)	29171	MUC16	94025
410837	19p	8959403	8961248	1846	6	0.667	Exon(exon3of84)	20094	MUC16	94025
410837	19p	15087452	15087953	502	7	0.143	Promoter(<=1kb)	472	OR1I1	126370
410837	19p	15794192	15794719	528	6	0.333	Promoter(<=1kb)	241	OR10H5	284433
410837	19p	17281820	17284246	2427	9	0.556	Promoter(<=1kb)	0	ANKLE1	126549
410837	19p	18264753	18267409	2657	8	0.5	5'UTR	7002	IQCN	80726
410837	19p	21971930	21974500	2571	7	0.714	Exon(exon4of4)	14408	ZNF208	7757
410837	19p	23743906	23745300	1395	6	0.333	Exon(exon4of4)	13537	ZNF681	148213
410837	19q	34943334	34944685	1352	6	0.333	3'UTR	9723	ZNF30	90075
410837	19q	36996730	36997597	868	10	0.6	Exon(exon10of10)	5677	ZNF568	374900
410837	19q	37886924	37888806	1883	6	0.667	Exon(exon6of6)	17788	WDR87	83889
410837	19q	39877222	39877880	659	6	0.5	Exon(exon20of28)	9412	FCGBP	8857
410837	19q	43846955	43848536	1582	6	0.833	3'UTR	13450	ZNF283	284349
410837	19q	43913423	43914878	1456	8	0.5	Exon(exon10of10)	4861	ZNF45	7596
410837	19q	52437918	52439242	1325	7	0.429	Exon(exon4of4)	6504	ZNF534	147658
410837	19q	55481625	55483456	1832	7	0.429	Promoter(1-2kb)	-1732	NAT14	57106
410837	19q	55912302	55913166	865	6	0.667	Exon(exon5of12)	19145	NLRP13	126204
410837	19q	58368293	58368875	583	7	0.429	Exon(exon3of3)	-5445	ZNF497	162968
410837	20q	63561666	63565531	3866	14	0.643	Promoter(<=1kb)	-61	HELZ2	85441
410837	21q	26843740	26844859	1120	6	0.667	Promoter(<=1kb)	0	ADAMTS1	9510
410837	21q	44550835	44551416	582	6	0.833	Promoter(<=1kb)	89	KRTAP10-2	386679
410837	21q	44637474	44638143	670	8	0.5	Promoter(<=1kb)	118	KRTAP10-10	353333
410837	22q	22352950	22353348	399	14	0.571	Exon(exon1of2)	30478	BMS1P20	96610
410837	22q	36191154	36191906	753	7	0.714	3'UTR	9971	APOL4	80832
410837	23p	3320126	3323750	3625	10	0.6	Exon(exon5of7)	22902	MXRA5	25878
410837	23p	8170039	8170141	103	6	0.5	Promoter(1-2kb)	1126	VCX2	51480
410837	23p	35802148	35803010	863	7	0.571	5'UTR	3357	MAGEB16	139604
410837	23q	102937373	102937765	393	7	0.571	Promoter(<=1kb)	101	RAB40AL	282808
413017	1p	11778784	11779941	1158	7	0.714	Promoter(<=1kb)	0	C1orf167-AS1	102724659
413017	1p	12847526	12847995	470	9	0.444	Promoter(<=1kb)	730	HNRNPCL1	343069
413017	1p	12859036	12860079	1044	6	0.333	Promoter(1-2kb)	1950	PRAMEF2	65122
413017	1p	13370686	13370957	272	7	0.429	Promoter(<=1kb)	943	PRAMEF19	645414
413017	1p	16058491	16060000	1510	10	0.9	Exon(exon5of7)	6168	CLCNKB	1188
413017	1p	18481403	18482217	815	6	0.667	Promoter(<=1kb)	421	KLHDC7A	127707
413017	1p	23874604	23875430	827	9	0.556	Exon(exon2of2)	-6310	FUCA1	2517
413017	1p	40067594	40067675	82	6	0	Promoter(<=1kb)	324	CAP1	10487
413017	1q	152302977	152313891	10915	40	0.575	Promoter(<=1kb)	0	FLG-AS1	339400
413017	1q	158765805	158766655	851	6	0.5	Promoter(<=1kb)	47	OR6N1	128372
413017	1q	228315976	228318049	2074	10	0.7	Exon(exon50of81)	6492	OBSCN	84033
413017	1q	247841312	247841582	271	6	0.833	Promoter(<=1kb)	314	OR11L1	391189
413017	1q	247949325	247949738	414	10	0.3	Promoter(<=1kb)	467	OR2L8	391190
413017	1q	248294677	248295458	782	7	0.571	Promoter(<=1kb)	142	OR2T12	127064
413017	2p	29002636	29003646	1011	6	0.333	Exon(exon5of20)	-10821	TOGARAM2	165186
413017	2q	130193975	130194275	301	6	0.5	Exon(exon4of5)	4164	TUBA3E	112714
413017	2q	185789865	185794632	4768	11	0.727	Promoter(<=1kb)	0	FSIP2	401024
413017	2q	185805377	185807800	2424	6	0.333	Promoter(<=1kb)	0	FSIP2	401024
413017	2q	238130416	238131546	1131	6	0.333	Promoter(1-2kb)	1468	ESPNL	339768
413017	2q	240041845	240042811	967	7	0.286	Downstream(2-3kb)	3261	OR6B3	150681
413017	3p	75736880	75738859	1980	55	0.582	Promoter(<=1kb)	0	MIR4273	100422955
413017	3q	98264413	98265098	686	7	0.571	Promoter(<=1kb)	128	OR5H6	79295
413017	4p	5988383	5989749	1367	7	0.571	Promoter(<=1kb)	0	C4orf50	389197
413017	4p	6300792	6302360	1569	7	0.857	Exon(exon8of8)	6021	WFS1	7466

413017	4p	7433331	7434512	1182	7	0.857	Promoter(<=1kb)	418	PSAPL1	768239
413017	4p	8227004	8228508	1505	8	0.125	Promoter(<=1kb)	-24	SH3TC1	54436
413017	4p	10443803	10446224	2422	6	0.333	Exon(exon3of3)	10952	ZNF518B	85460
413017	4p	38774449	38775552	1104	9	0.556	Promoter(<=1kb)	644	TLR10	81793
413017	4p	38827699	38829115	1417	6	0.5	Exon(exon2of2)	-22121	TLR1	7096
413017	4q	185458217	185460011	1795	8	0.625	Promoter(<=1kb)	0	CCDC110	256309
413017	5p	795818	796237	420	6	0.667	3'UTR	4908	ZDHHC11	79844
413017	5q	79731782	79734523	2742	13	0.308	Exon(exon2of13)	-3619	CMYA5	202333
413017	5q	83537326	83539905	2580	6	0.333	Promoter(1-2kb)	1712	VCAN	1462
413017	5q	140807352	140807737	386	6	0.833	Promoter(<=1kb)	271	PCDHA4	56144
413017	5q	140848579	140850786	2208	7	0.571	Promoter(<=1kb)	807	PCDHA9	9752
413017	5q	141174000	141174608	609	7	1	Promoter(1-2kb)	1356	PCDHB7	56129
413017	5q	141178745	141180333	1589	8	0.75	Promoter(<=1kb)	955	PCDHB8	56128
413017	5q	151521550	151522069	520	6	0.667	Promoter(<=1kb)	79	MIR6499	102465246
413017	5q	151565922	151568158	2237	9	0.778	Promoter(<=1kb)	786	FAT2	2196
413017	6p	1312843	1313745	903	6	0.5	Promoter(<=1kb)	745	FOXQ1	94234
413017	6p	46858771	46859502	732	8	0.5	Exon(exon17of21)	3802	ADGRF5	221395
413017	6q	149888581	149889987	1407	6	0.667	Promoter(<=1kb)	-116	RAET1E-AS1	100652739
413017	6q	159231899	159234370	2472	12	0.583	Exon(exon11of23)	13602	FNDC1	84624
413017	7p	12369637	12370736	1100	6	0.667	3'UTR	-13307	VWDE	221806
413017	7p	53035678	53036385	708	7	1	Promoter(<=1kb)	45	POM121L12	285877
413017	7q	100958977	100960873	1897	51	0.471	Promoter(1-2kb)	1012	MUC3A	4584
413017	7q	100991195	100993127	1933	8	0.625	Exon(exon5of15)	-19927	MUC12	10071
413017	7q	100995575	100995785	211	7	0.857	Exon(exon5of15)	-17269	MUC12	10071
413017	8p	11331234	11332082	849	6	0.667	Promoter(<=1kb)	346	SLC35G5	83650
413017	8p	13021128	13022030	903	7	0.143	Exon(exon5of5)	9115	TRMT9B	57604
413017	9p	116832	117720	889	6	0.5	Promoter(<=1kb)	484	FOXD4	2298
413017	9p	34723747	34726527	2781	11	0.727	Promoter(2-3kb)	2961	FAM205A	259308
413017	9q	76175237	76175296	60	10	0.8	Exon(exon14of14)	-13343	PCSK5	5125
413017	9q	76703555	76707804	4250	13	0.462	Promoter(<=1kb)	0	PCA3	50652
413017	9q	76709263	76710843	1581	8	0.5	Promoter(<=1kb)	0	PRUNE2	158471
413017	9q	104598641	104599361	721	8	0.625	Promoter(<=1kb)	52	OR13C5	138799
413017	9q	122553263	122554071	809	7	0.429	Promoter(<=1kb)	93	OR1N2	138882
413017	9q	122628595	122629398	804	6	0.333	Promoter(<=1kb)	175	OR1B1	347169
413017	9q	122749914	122750547	634	6	0.833	Promoter(<=1kb)	174	OR1L6	392390
413017	9q	135484803	135487573	2771	9	0.333	Promoter(1-2kb)	1440	PPP1R26	9858
413017	9q	135547960	135548795	836	8	0.625	Promoter(1-2kb)	1805	OBP2A	29991
413017	10p	47663	48605	943	7	0.857	Promoter(<=1kb)	664	TUBB8	347688
413017	10q	46549378	46550723	1346	25	0.64	Exon(exon3of3)	4807	GPRIN2	9721
413017	10q	89737450	89738561	1112	6	0	Exon(exon20of33)	13874	KIF20B	9585
413017	10q	128102595	128104752	2158	9	0.556	Promoter(<=1kb)	0	MKI67	4288
413017	10q	128106210	128108204	1995	6	1	Exon(exon12of14)	-3082	MKI67	4288
413017	11p	244106	244197	92	8	0.5	Promoter(<=1kb)	-232	PSMD13	5719
413017	11p	5177978	5178478	501	6	0.167	Promoter(<=1kb)	186	OR52Z1	283110
413017	11p	5323542	5324256	715	6	0.5	Promoter(<=1kb)	41	OR51B2	79345
413017	11p	5351521	5352416	896	15	0.467	Promoter(<=1kb)	13	OR51B6	390058
413017	11p	5389704	5390350	647	7	0.429	Promoter(<=1kb)	327	OR51M1	390059
413017	11p	5422212	5423123	912	11	0.636	Promoter(<=1kb)	101	OR51Q1	390061
413017	11p	5515185	5515931	747	6	0.333	Promoter(<=1kb)	768	UBQLNL	143630
413017	11p	5581045	5581738	694	8	0.375	Promoter(<=1kb)	168	OR52B6	340980
413017	11p	5788000	5788760	761	8	0.75	Promoter(<=1kb)	56	OR52N1	79473
413017	11p	5841302	5841883	582	9	0.333	Promoter(<=1kb)	14	OR52E6	390078
413017	11p	11351961	11352736	776	9	0.222	Promoter(<=1kb)	514	CSNK2A3	283106
413017	11p	12293639	12294368	730	7	0.857	Exon(exon29of35)	6739	MICALCL	84953
413017	11p	18173280	18173901	622	6	0.333	Promoter(<=1kb)	443	MRGPRX4	117196
413017	11q	58214757	58215722	966	8	0.25	Promoter(<=1kb)	12	OR1S1	219959
413017	11q	64315797	64315856	60	8	0.75	Promoter(1-2kb)	1485	TRMT112	51504
413017	11q	66560202	66562261	2060	6	0.333	Exon(exon14of21)	3873	CTSF	8722
413017	11q	85724687	85725825	1139	6	0.5	Promoter(<=1kb)	0	SYTL2	54843
413017	11q	123906790	123907324	535	6	0.667	Promoter(<=1kb)	644	OR8D4	338662
413017	11q	124015600	124016477	878	7	0.143	Promoter(<=1kb)	25	OR10G4	390264
413017	11q	124038366	124038988	623	7	1	Promoter(<=1kb)	13	OR10G7	390265
413017	11q	124382526	124383285	760	8	0.625	Promoter(<=1kb)	58	OR8B2	26595
413017	12p	4626571	4628549	1979	9	0.556	Exon(exon5of6)	14054	DYRK4	8798
413017	12p	8222174	8223514	1341	6	0.667	Exon(exon5of6)	4073	FAM90A1	55138
413017	13q	24434450	24435347	898	7	0.571	Exon(exon31of34)	19787	PARP4	143
413017	13q	25096665	25097182	518	17	0.471	Promoter(<=1kb)	797	PABPC3	5042
413017	13q	49668389	49668431	43	6	0.167	Exon(exon3of3)	23007	EBPL	84650
413017	13q	102732474	102733933	1460	6	0.333	Exon(exon4of4)	25139	CCDC168	643677
413017	14q	20060048	20060884	837	8	0.625	Promoter(<=1kb)	3	OR4L1	122742
413017	14q	21634137	21634589	453	9	0.556	Promoter(<=1kb)	351	OR10G2	26534
413017	14q	70457532	70458540	1009	9	0.333	Exon(exon2of2)	5358	ADAM21	8747
413017	14q	104939262	104942618	3357	13	0.154	5'UTR	7102	PLD4	122618
413017	14q	104943622	104946886	3265	16	0.625	Exon(exon6of6)	8516	AHNAK2	113146
413017	14q	104947901	104953878	5978	42	0.5	Promoter(1-2kb)	1524	AHNAK2	113146
413017	15q	23439979	23442067	2089	12	0.583	5'UTR	5167	GOLGA6L2	283685
413017	15q	85579423	85581800	2378	15	0.533	Promoter(1-2kb)	-1110	AKAP13	11214

413017	16p	1228744	1229622	879	6	0.833	Promoter(<=1kb)	540	TPSB2	64499
413017	16p	1486371	1488463	2093	8	0.75	Promoter(<=1kb)	4	PTX4	390667
413017	16q	88428539	88429600	1062	7	0.429	Exon(exon3of3)	-23680	ZFPM1	161882
413017	16q	88714717	88717113	2397	6	0.5	Promoter(<=1kb)	0	MIR4722	100616167
413017	16q	89100686	89101050	365	7	0.571	Promoter(<=1kb)	24	ACSF3	197322
413017	17p	10638198	10641099	2902	7	0.286	Exon(exon19of41)	-8169	MYH3	4621
413017	17p	21300581	21300954	374	8	0.75	3'UTR	9112	MAP2K3	5606
413017	17p	21415470	21416404	935	7	1	Exon(exon3of3)	10334	KCNJ12	3768
413017	17q	76293419	76294016	598	6	0.5	Promoter(2-3kb)	-2167	QRICH2	84074
413017	17q	81645135	81645417	283	6	0.333	Promoter(2-3kb)	2722	TSPAN10	83882
413017	18p	11609904	11610509	606	8	0.625	Promoter(<=1kb)	308	SLC35G4	646000
413017	18q	58535186	58537515	2330	9	0.333	Promoter(<=1kb)	0	ALPK2	115701
413017	19p	1004688	1005532	845	6	0.5	Exon(exon3of9)	4269	GRIN3B	116444
413017	19p	1036457	1036914	458	6	0.5	Exon(exon6of7)	-3187	ABCA7	10347
413017	19p	4510548	4513547	3000	20	0.45	Exon(exon3of6)	4157	PLIN4	729359
413017	19p	5455724	5456439	716	6	0.5	Promoter(<=1kb)	307	ZNRF4	148066
413017	19p	5866643	5867737	1095	7	0.571	Promoter(2-3kb)	2803	FUT5	2527
413017	19p	8937644	8939234	1591	6	0.667	Exon(exon5of84)	-41554	MUC16	94025
413017	19p	8946313	8952171	5859	20	0.6	Exon(exon3of84)	29171	MUC16	94025
413017	19p	8959116	8962299	3184	12	0.75	Exon(exon3of84)	19043	MUC16	94025
413017	19p	8971838	8978096	6259	15	0.533	Exon(exon1of84)	3246	MUC16	94025
413017	19p	11978548	11978990	443	6	0.5	Promoter(1-2kb)	1726	ZNF763	284390
413017	19p	12075333	12077046	1714	6	0.167	Promoter(<=1kb)	6	ZNF788P	388507
413017	19p	17282085	17284246	2162	8	0.5	Promoter(<=1kb)	0	ANKLE1	126549
413017	19p	18264798	18267409	2612	12	0.583	5'UTR	7002	IQCN	80726
413017	19p	21971930	21974500	2571	7	0.714	Exon(exon4of4)	14408	ZNF208	7757
413017	19p	22663799	22665021	1223	6	0.667	Exon(exon4of4)	29475	ZNF492	57615
413017	19p	22756205	22759523	3319	13	0.615	3'UTR	10459	ZNF99	7652
413017	19q	43913423	43914878	1456	8	0.5	Exon(exon10of10)	4861	ZNF45	7596
413017	19q	43966037	43967171	1135	6	0.167	Promoter(<=1kb)	-691	ZNF155	7711
413017	19q	44328359	44329698	1340	6	0.667	Exon(exon4of4)	-23313	ZNF235	9310
413017	19q	51745958	51746963	1006	6	0.333	Exon(exon3of3)	3848	FPR1	2357
413017	19q	52437918	52439242	1325	7	0.429	Exon(exon4of4)	6504	ZNF534	147658
413017	19q	55517821	55518176	356	7	1	Exon(exon14of14)	18127	SBK2	646643
413017	19q	55912302	55913166	865	6	0.667	Exon(exon5of12)	19145	NLRP13	126204
413017	19q	58368293	58368875	583	7	0.429	Exon(exon3of3)	-5445	ZNF497	162968
413017	20p	5922577	5923382	806	8	0.625	Exon(exon4of5)	7079	CHGB	1114
413017	20q	63349752	63350772	1021	6	0.5	3'UTR	3794	CHRNA4	1137
413017	20q	63561666	63565531	3866	14	0.643	Promoter(<=1kb)	-61	HELZ2	85441
413017	21q	36070334	36072675	2342	6	0.833	Promoter(<=1kb)	299	CBR1	873
413017	22q	22352950	22353380	431	16	0.5	Exon(exon1of2)	30478	BMS1P20	96610
413017	22q	36191154	36191906	753	6	0.667	3'UTR	9971	APOL4	80832
413017	22q	49883704	49884994	1291	7	0.286	Exon(exon2of2)	22924	ALG12	79087
413017	23p	8170039	8170141	103	6	0.5	Promoter(1-2kb)	1126	VCX2	51480
413017	23p	35802148	35803010	863	7	0.571	5'UTR	3357	MAGEB16	139604
413017	23q	136874183	136874416	234	6	1	Promoter(<=1kb)	-201	RBMX	27316
421092	1p	16058680	16059952	1273	6	0.667	Exon(exon5of7)	6357	CLCNKB	1188
421092	1p	18481403	18483159	1757	7	0.714	Promoter(<=1kb)	421	KLHDC7A	127707
421092	1p	40067594	40067675	82	6	0	Promoter(<=1kb)	324	CAP1	10487
421092	1p	89186388	89186419	32	9	0.556	Promoter(<=1kb)	107	GBP4	115361
421092	1q	152218469	152221235	2767	15	0.667	Promoter(2-3kb)	2958	HRNR	388697
421092	1q	152303673	152305077	1405	10	0.6	Exon(exon3of3)	-7499	FLG-AS1	339400
421092	1q	152306380	152313891	7512	40	0.65	Promoter(<=1kb)	0	FLG-AS1	339400
421092	1q	158398525	158399274	750	6	0.333	Promoter(<=1kb)	192	OR10T2	128360
421092	1q	158765805	158766655	851	6	0.5	Promoter(<=1kb)	47	OR6N1	128372
421092	1q	201206099	201209342	3244	10	0.6	Promoter(1-2kb)	1017	IGFN1	91156
421092	1q	201212089	201215147	3059	8	0.625	Promoter(<=1kb)	0	IGFN1	91156
421092	1q	223393517	223394466	950	6	0.667	Promoter(<=1kb)	102	CCDC185	164127
421092	1q	226735683	226737239	1557	7	0.857	Promoter(<=1kb)	219	ITPKB	3707
421092	1q	247841312	247841582	271	6	0.833	Promoter(<=1kb)	314	OR11L1	391189
421092	1q	247896121	247896502	382	6	0.5	Promoter(<=1kb)	534	OR2W3	343171
421092	1q	247949325	247949738	414	6	0	Promoter(<=1kb)	467	OR2L8	391190
421092	2q	130193975	130194465	491	6	0.667	Exon(exon4of5)	3974	TUBA3E	112714
421092	2q	167246794	167248478	1685	8	0.75	Promoter(<=1kb)	-204	XIRP2	129446
421092	2q	167249575	167251847	2273	8	0.375	Promoter(<=1kb)	893	XIRP2	129446
421092	2q	178739433	178741811	2379	6	0.5	Exon(exon45of191)	26014	TTN	7273
421092	2q	184936178	184937636	1459	6	0.333	Exon(exon4of4)	69813	ZNF804A	91752
421092	2q	185789865	185794632	4768	11	0.818	Promoter(<=1kb)	0	FSIP2	401024
421092	2q	217847583	217848559	977	6	0.833	Exon(exon19of33)	-5423	TNS1	7145
421092	2q	233713134	233713783	650	13	0.692	Promoter(<=1kb)	142	UGT1A5	54579
421092	2q	233840612	233842185	1574	6	0.5	Promoter(<=1kb)	0	HJURP	55355
421092	2q	238130416	238131546	1131	6	0.333	Promoter(1-2kb)	1468	ESPNL	339768
421092	3p	75737230	75739007	1778	9	0.556	Promoter(<=1kb)	0	MIR4273	100422955
421092	3q	98169021	98169594	574	6	0.667	Exon(exon2of2)	19695	OR5H14	403273
421092	3q	98264413	98265137	725	7	0.714	Promoter(<=1kb)	128	OR5H6	79295
421092	3q	194341097	194342571	1475	7	0.714	Exon(exon2of2)	8747	CPN2	1370
421092	3q	194359607	194360906	1300	9	0.889	Exon(exon2of2)	-8279	CPN2	1370

421092	4p	5988383	5989749	1367	7	0.571	Promoter(<=1kb)	0	C4orf50	389197
421092	4p	6300792	6302360	1569	6	0.833	Exon(exon8of8)	6021	WFS1	7466
421092	4p	8227004	8228508	1505	8	0.125	Promoter(<=1kb)	-24	SH3TC1	54436
421092	4q	185458217	185460011	1795	8	0.625	Promoter(<=1kb)	0	CCDC110	256309
421092	5q	79731782	79734523	2742	14	0.357	Exon(exon2of13)	-3619	CMYA5	202333
421092	5q	140848579	140850786	2208	8	0.5	Promoter(<=1kb)	807	PCDHA9	9752
421092	5q	141174000	141175025	1026	6	0.833	Promoter(1-2kb)	1356	PCDHB7	56129
421092	5q	141187690	141189425	1736	6	0.5	Promoter(<=1kb)	529	PCDHB9	56127
421092	5q	141955676	141957660	1985	6	0.667	Promoter(<=1kb)	-668	RNF14	9604
421092	5q	148826877	148828070	1194	6	1	Promoter(1-2kb)	1632	ADRB2	154
421092	5q	151565922	151568158	2237	9	0.778	Promoter(<=1kb)	786	FAT2	2196
421092	6p	16327099	16327837	739	6	0.333	Exon(exon8of9)	37154	GMPR	2766
421092	6p	46858771	46859502	732	7	0.571	Exon(exon17of21)	3802	ADGRF5	221395
421092	6q	111373870	111375649	1780	6	0.833	3'UTR	-6512	REV3L	5980
421092	6q	159231899	159234370	2472	12	0.583	Exon(exon11of23)	13602	FNDC1	84624
421092	7p	2538147	2538737	591	6	0.667	Promoter(2-3kb)	2287	BRAT1	221927
421092	7p	12369637	12370736	1100	6	0.667	3'UTR	-13307	VWDE	221806
421092	7q	100958721	100960873	2153	51	0.471	Promoter(<=1kb)	756	MUC3A	4584
421092	7q	100991195	100993101	1907	7	0.571	Exon(exon5of15)	-19953	MUC12	10071
421092	7q	101034305	101038481	4177	25	0.48	Exon(exon3of12)	-5230	MUC17	140453
421092	8p	10607245	10608432	1188	8	0.5	Exon(exon4of4)	46711	RP1L1	94137
421092	8p	13021128	13022030	903	8	0.125	Exon(exon5of5)	9115	TRMT9B	57604
421092	8p	22163524	22164421	898	6	0.833	Promoter(<=1kb)	444	SFTPC	6440
421092	8q	138151949	138153046	1098	6	0.667	Promoter(<=1kb)	0	FAM135B	51059
421092	8q	143916274	143919209	2936	8	0.25	Exon(exon32of32)	20381	PLEC	5339
421092	8q	143923488	143925516	2029	9	0.667	Exon(exon31of32)	14074	PLEC	5339
421092	9q	76705724	76707804	2081	6	0.5	Promoter(<=1kb)	666	PCA3	50652
421092	9q	87885490	87887543	2054	6	0.667	Promoter(2-3kb)	2613	SPATA31E1	286234
421092	9q	122553263	122554071	809	7	0.429	Promoter(<=1kb)	93	OR1N2	138882
421092	9q	122628595	122629398	804	6	0.333	Promoter(<=1kb)	175	OR1B1	347169
421092	10q	46549378	46550723	1346	26	0.654	Exon(exon3of3)	4807	GPRIN2	9721
421092	10q	103602059	103602530	472	6	0.667	Exon(exon15of15)	30382	NEURL1	9148
421092	10q	122084988	122087840	2853	8	0.75	Exon(exon4of23)	-25190	TACC2	10579
421092	11p	244106	244197	92	8	0.5	Promoter(<=1kb)	-232	PSMD13	5719
421092	11p	1194354	1196902	2549	7	0.571	Exon(exon34of49)	-26164	MUC5B	727897
421092	11p	5177978	5178478	501	6	0.167	Promoter(<=1kb)	186	OR52Z1	283110
421092	11p	5323542	5324256	715	6	0.5	Promoter(<=1kb)	41	OR51B2	79345
421092	11p	5389704	5390350	647	7	0.429	Promoter(<=1kb)	327	OR51M1	390059
421092	11p	5402638	5403322	685	10	0.5	Promoter(<=1kb)	41	OR51J1	79470
421092	11p	5422212	5423123	912	10	0.7	Promoter(<=1kb)	101	OR51Q1	390061
421092	11p	5581045	5581738	694	8	0.375	Promoter(<=1kb)	168	OR52B6	340980
421092	11p	5841302	5841883	582	9	0.333	Promoter(<=1kb)	14	OR52E6	390078
421092	11p	11352040	11352736	697	8	0.25	Promoter(<=1kb)	514	CSNK2A3	283106
421092	11p	12293639	12294368	730	7	0.857	Exon(exon29of35)	6739	MICALCL	84953
421092	11q	58214757	58215722	966	8	0.25	Promoter(<=1kb)	12	OR1S1	219959
421092	11q	82732630	82733184	555	6	0.833	Promoter(<=1kb)	680	FAM181B	220382
421092	11q	85724687	85725825	1139	7	0.571	Promoter(<=1kb)	0	SYTL2	54843
421092	11q	123906790	123907324	535	6	0.667	Promoter(<=1kb)	644	OR8D4	338662
421092	11q	124264847	124265676	830	6	0.833	Promoter(<=1kb)	20	OR8G5	219865
421092	11q	124382526	124383285	760	6	0.5	Promoter(<=1kb)	58	OR8B2	26595
421092	12p	4626571	4628549	1979	9	0.556	Exon(exon5of6)	14054	DYRK4	8798
421092	12q	48525773	48526223	451	6	0.5	Promoter(<=1kb)	141	OR8S1	341568
421092	12q	52571389	52573652	2264	6	0.667	Promoter(<=1kb)	164	KRT74	121391
421092	13q	24434450	24435347	898	7	0.571	Exon(exon31of34)	19787	PARP4	143
421092	13q	24446725	24447185	461	6	0.333	Exon(exon26of34)	7949	PARP4	143
421092	13q	25096713	25097529	817	19	0.474	Promoter(<=1kb)	845	PABPC3	5042
421092	13q	102732474	102733933	1460	6	0.333	Exon(exon4of4)	25139	CCDC168	643677
421092	14q	20060048	20060884	837	8	0.625	Promoter(<=1kb)	3	OR4L1	122742
421092	14q	44504986	44506403	1418	6	0.667	Promoter(<=1kb)	880	FSCB	84075
421092	14q	63599649	63599713	65	9	0.556	Exon(exon2of2)	41694	WDR89	112840
421092	14q	70457532	70458948	1417	12	0.417	Exon(exon2of2)	5358	ADAM21	8747
421092	14q	104177072	104177810	739	6	0.333	Exon(exon12of15)	38032	KIF26A	26153
421092	14q	104939262	104942618	3357	11	0.273	5'UTR	7102	PLD4	122618
421092	14q	104943622	104945729	2108	8	0.75	Exon(exon6of6)	9673	AHNAK2	113146
421092	14q	104947453	104949750	2298	11	0.727	Exon(exon6of6)	5652	AHNAK2	113146
421092	14q	104950765	104953878	3114	16	0.375	Promoter(1-2kb)	1524	AHNAK2	113146
421092	15q	23440160	23442067	1908	10	0.6	5'UTR	5167	GOLGA6L2	283685
421092	15q	78766033	78766609	577	6	0.333	Promoter(<=1kb)	-920	ADAMTS7	11173
421092	15q	99129423	99132517	3095	6	0.333	Exon(exon4of5)	7225	TTC23	64927
421092	16p	789084	790597	1514	6	0.167	Promoter(<=1kb)	0	CHTF18	63922
421092	16p	1256354	1256985	632	11	0.636	Promoter(<=1kb)	285	TPSD1	23430
421092	16p	1486371	1488463	2093	8	0.75	Promoter(<=1kb)	4	PTX4	390667
421092	16p	28495551	28497830	2280	7	0.429	Promoter(<=1kb)	0	CLN3	1201
421092	16q	74391650	74391897	248	8	0.75	Exon(exon7of7)	13772	NPIPB15	440348
421092	16q	89100686	89101050	365	7	0.571	Promoter(<=1kb)	24	ACSF3	197322
421092	16q	89226863	89228289	1427	7	0.571	Promoter(2-3kb)	2229	ZNF778	197320
421092	17p	7041768	7043011	1244	6	0.667	Promoter(<=1kb)	595	SLC16A11	162515

421092	17p	7482736	7482961	226	6	0.833	Promoter(1-2kb)	1302	ZBTB4	57659
421092	17p	10638198	10641099	2902	7	0.286	Exon(exon19of41)	-8169	MYH3	4621
421092	17p	21300581	21300978	398	12	0.75	3'UTR	9112	MAP2K3	5606
421092	17p	21415470	21416431	962	12	0.833	Exon(exon3of3)	10334	KCNJ12	3768
421092	17q	41586434	41586829	396	7	0.857	Promoter(<=1kb)	66	KRT14	3861
421092	17q	76293419	76294016	598	6	0.5	Promoter(2-3kb)	-2167	QRICH2	84074
421092	17q	81645135	81645607	473	7	0.286	Promoter(2-3kb)	2722	TSPAN10	83882
421092	18q	58535186	58537515	2330	10	0.4	Promoter(<=1kb)	0	ALPK2	115701
421092	19p	4510711	4511943	1233	11	0.545	Exon(exon3of6)	5761	PLIN4	729359
421092	19p	5455600	5456439	840	7	0.571	Promoter(<=1kb)	183	ZNRF4	148066
421092	19p	8946313	8951868	5556	15	0.6	Exon(exon3of84)	29474	MUC16	94025
421092	19p	8959116	8962299	3184	10	0.7	Exon(exon3of84)	19043	MUC16	94025
421092	19p	8972467	8978096	5630	17	0.471	Exon(exon1of84)	3246	MUC16	94025
421092	19p	18264798	18267409	2612	12	0.583	5'UTR	7002	IQCN	80726
421092	19p	21971930	21974500	2571	11	0.636	Exon(exon4of4)	14408	ZNF208	7757
421092	19p	23743906	23745300	1395	6	0.333	Exon(exon4of4)	13537	ZNF681	148213
421092	19q	43913423	43914878	1456	9	0.556	Exon(exon10of10)	4861	ZNF45	7596
421092	19q	43996326	43997366	1041	6	0.5	Exon(exon5of5)	5419	LOC10192806[illegible]	101928063
421092	19q	44106512	44108078	1567	7	0	Exon(exon6of6)	-4103	ZNF225	7768
421092	19q	52437918	52439242	1325	7	0.429	Exon(exon4of4)	6504	ZNF534	147658
421092	19q	55517821	55518176	356	7	1	Exon(exon14of14)	18127	SBK2	646643
421092	20p	5922421	5923394	974	6	0.5	Exon(exon4of5)	6923	CHGB	1114
421092	20p	20052354	20052736	383	6	0	Promoter(<=1kb)	0	CFAP61	26074
421092	20q	63561666	63565531	3866	12	0.667	Promoter(<=1kb)	-61	HELZ2	85441
421092	21q	44600627	44601692	1066	7	0.857	Promoter(<=1kb)	30	KRTAP10-7	386675
421092	21q	44637476	44638143	668	9	0.444	Promoter(<=1kb)	120	KRTAP10-10	353333
421092	22q	22352950	22353348	399	14	0.571	Exon(exon1of2)	30478	BMS1P20	96610
421092	22q	36191154	36191906	753	6	0.667	3'UTR	9971	APOL4	80832
421092	23p	3320126	3323750	3625	9	0.556	Exon(exon5of7)	22902	MXRA5	25878
421092	23p	8170039	8170141	103	6	0.5	Promoter(1-2kb)	1126	VCX2	51480
421092	23p	35802148	35803010	863	7	0.571	5'UTR	3357	MAGEB16	139604
422115	1p	13369166	13369564	399	7	0.714	Promoter(2-3kb)	2336	PRAMEF19	645414
422115	1p	13370686	13371119	434	7	0.429	Promoter(<=1kb)	781	PRAMEF19	645414
422115	1p	13391965	13392142	178	6	0.333	Promoter(2-3kb)	2333	PRAMEF17	391004
422115	1p	16058491	16060000	1510	13	0.846	Exon(exon5of7)	6168	CLCNKB	1188
422115	1p	18481403	18482217	815	6	0.667	Promoter(<=1kb)	421	KLHDC7A	127707
422115	1p	23874604	23875430	827	9	0.556	Exon(exon2of2)	-6310	FUCA1	2517
422115	1p	40067594	40067675	82	6	0	Promoter(<=1kb)	324	CAP1	10487
422115	1p	89186388	89186419	32	9	0.556	Promoter(<=1kb)	107	GBP4	115361
422115	1q	152302977	152313891	10915	45	0.578	Promoter(<=1kb)	0	FLG-AS1	339400
422115	1q	169542317	169542882	566	6	0.167	Exon(exon13of25)	-26572	F5	2153
422115	1q	223393517	223394466	950	6	0.667	Promoter(<=1kb)	102	CCDC185	164127
422115	1q	228315976	228318026	2051	7	0.571	Exon(exon50of81)	6492	OBSCN	84033
422115	1q	247841312	247841582	271	6	0.833	Promoter(<=1kb)	314	OR11L1	391189
422115	1q	247895951	247896502	552	7	0.571	Promoter(<=1kb)	364	OR2W3	343171
422115	1q	247949325	247949738	414	10	0.3	Promoter(<=1kb)	467	OR2L8	391190
422115	1q	248273309	248273670	362	7	0.571	Promoter(<=1kb)	166	OR2T33	391195
422115	1q	248294677	248295458	782	7	0.571	Promoter(<=1kb)	142	OR2T12	127064
422115	2p	29002636	29003646	1011	6	0.333	Exon(exon5of20)	-10821	TOGARAM2	165186
422115	2p	48580657	48582454	1798	8	0.5	Promoter(<=1kb)	0	STON1	11037
422115	2q	132783032	132785012	1981	7	0.571	Promoter(1-2kb)	-1009	NCKAP5	344148
422115	2q	185789591	185794632	5042	11	0.727	Promoter(<=1kb)	0	FSIP2	401024
422115	2q	185805377	185807185	1809	6	0.5	Promoter(<=1kb)	0	FSIP2	401024
422115	2q	217847583	217848559	977	6	0.833	Exon(exon19of33)	-5423	TNS1	7145
422115	2q	237762685	237764060	1376	10	0.5	Exon(exon8of8)	-4137	LRRFIP1	9208
422115	2q	238130271	238131546	1276	7	0.286	Promoter(1-2kb)	1323	ESPNL	339768
422115	3p	75736880	75739007	2128	49	0.592	Promoter(<=1kb)	0	MIR4273	100422955
422115	3q	98264413	98265098	686	7	0.571	Promoter(<=1kb)	128	OR5H6	79295
422115	4p	5988383	5989749	1367	8	0.625	Promoter(<=1kb)	0	C4orf50	389197
422115	4p	6300792	6302360	1569	7	0.857	Exon(exon8of8)	6021	WFS1	7466
422115	4p	7433331	7434512	1182	7	0.857	Promoter(<=1kb)	418	PSAPL1	768239
422115	4p	8227004	8228508	1505	8	0.125	Promoter(<=1kb)	-24	SH3TC1	54436
422115	4q	154489498	154491312	1815	9	0.556	Promoter(<=1kb)	22	DCHS2	54798
422115	5q	67163994	67165736	1743	6	0.5	Exon(exon29of29)	20561	CD180	4064
422115	5q	79731782	79734523	2742	13	0.308	Exon(exon2of13)	-3619	CMYA5	202333
422115	5q	140807352	140807737	386	6	0.833	Promoter(<=1kb)	271	PCDHA4	56144
422115	5q	140848579	140850786	2208	6	0.5	Promoter(<=1kb)	807	PCDHA9	9752
422115	6p	46858771	46859502	732	8	0.5	Exon(exon17of21)	3802	ADGRF5	221395
422115	6q	149888581	149890867	2287	7	0.714	Promoter(<=1kb)	0	RAET1E-AS1	100652739
422115	6q	159233455	159234370	916	10	0.5	Exon(exon11of23)	15158	FNDC1	84624
422115	7p	45082865	45084866	2002	6	0.333	Promoter(1-2kb)	-1465	NACAD	23148
422115	7q	64991278	64992758	1481	7	0.571	Promoter(<=1kb)	-242	ZNF117	51351
422115	7q	100958977	100960873	1897	54	0.444	Promoter(1-2kb)	1012	MUC3A	4584
422115	7q	100991195	100992398	1204	8	0.625	Exon(exon5of15)	-20656	MUC12	10071
422115	7q	100995547	100995896	350	9	0.889	Exon(exon5of15)	-17158	MUC12	10071
422115	8p	10607245	10608261	1017	7	0.571	Exon(exon4of4)	46882	RP1L1	94137
422115	8p	10609650	10610662	1013	7	0.571	Exon(exon4of4)	44481	RP1L1	94137

422115	8p	11331194	11332026	833	7	0.571	Promoter(<=1kb)	306	SLC35G5	83650
422115	8p	12132686	12133940	1255	6	0.833	Promoter(<=1kb)	498	USP17L7	392197
422115	8p	12137448	12138752	1305	7	0.857	Promoter(<=1kb)	325	USP17L2	377630
422115	8p	13021128	13022030	903	9	0.222	Exon(exon5of5)	9115	TRMT9B	57604
422115	8q	123651655	123652634	980	7	0.571	Promoter(<=1kb)	316	KLHL38	340359
422115	8q	143916360	143919209	2850	7	0.143	Exon(exon32of32)	20381	PLEC	5339
422115	9p	38395931	38396505	575	6	0.833	Exon(exon2of2)	3219	ALDH1B1	219
422115	9q	76175237	76175296	60	10	0.8	Exon(exon14of14)	-13343	PCSK5	5125
422115	9q	76703555	76706360	2806	8	0.5	Promoter(<=1kb)	0	PCA3	50652
422115	9q	87886533	87888536	2004	7	0.571	Exon(exon4of4)	3656	SPATA31E1	286234
422115	9q	104504315	104505071	757	6	0.5	Promoter(<=1kb)	52	OR13F1	138805
422115	9q	104598545	104599361	817	12	0.583	Promoter(<=1kb)	52	OR13C5	138799
422115	9q	122553263	122554071	809	8	0.5	Promoter(<=1kb)	93	OR1N2	138882
422115	9q	122628595	122629398	804	7	0.286	Promoter(<=1kb)	175	OR1B1	347169
422115	9q	122749914	122750547	634	7	0.857	Promoter(<=1kb)	174	OR1L6	392390
422115	9q	135484803	135487573	2771	10	0.4	Promoter(1-2kb)	1440	PPP1R26	9858
422115	10q	46549378	46550723	1346	27	0.667	Exon(exon3of3)	4807	GPRIN2	9721
422115	10q	49323169	49326817	3649	14	0.643	Exon(exon3of3)	23895	C10orf71	118461
422115	10q	122084988	122087840	2853	8	0.75	Exon(exon4of23)	-25190	TACC2	10579
422115	10q	128103129	128104830	1702	10	0.6	Promoter(<=1kb)	-1	MKI67	4288
422115	11p	308290	309127	838	6	0.333	Promoter(<=1kb)	0	IFITM2	10581
422115	11p	1246095	1248605	2511	13	0.538	Promoter(1-2kb)	1071	MUC5B-AS1	112577518
422115	11p	5177811	5178478	668	7	0.143	Promoter(<=1kb)	186	OR52Z1	283110
422115	11p	5323542	5324256	715	6	0.5	Promoter(<=1kb)	41	OR51B2	79345
422115	11p	5389704	5390350	647	6	0.5	Promoter(<=1kb)	327	OR51M1	390059
422115	11p	5402638	5403322	685	9	0.444	Promoter(<=1kb)	41	OR51J1	79470
422115	11p	5440761	5441472	712	6	0.833	Promoter(<=1kb)	42	OR51I1	390063
422115	11p	5581045	5581738	694	8	0.375	Promoter(<=1kb)	168	OR52B6	340980
422115	11p	5841302	5841883	582	9	0.333	Promoter(<=1kb)	14	OR52E6	390078
422115	11p	11351961	11352736	776	9	0.222	Promoter(<=1kb)	514	CSNK2A3	283106
422115	11p	12293639	12294842	1204	7	0.714	Exon(exon29of35)	6739	MICALCL	84953
422115	11p	18173280	18173901	622	6	0.333	Promoter(<=1kb)	443	MRGPRX4	117196
422115	11q	58214757	58215722	966	8	0.25	Promoter(<=1kb)	12	OR1S1	219959
422115	11q	82732630	82733184	555	6	0.833	Promoter(<=1kb)	680	FAM181B	220382
422115	11q	85724687	85726853	2167	8	0.375	Promoter(<=1kb)	0	SYTL2	54843
422115	11q	123906790	123907324	535	6	0.667	Promoter(<=1kb)	644	OR8D4	338662
422115	11q	124038366	124038988	623	8	0.875	Promoter(<=1kb)	13	OR10G7	390265
422115	12p	4626568	4627524	957	7	0.429	Exon(exon5of6)	14051	DYRK4	8798
422115	12p	6453119	6453670	552	6	0.667	Promoter(<=1kb)	633	TAPBPL	55080
422115	12p	8222174	8223514	1341	6	0.667	Exon(exon5of6)	4073	FAM90A1	55138
422115	13q	25096713	25097021	309	12	0.417	Promoter(<=1kb)	845	PABPC3	5042
422115	13q	102732474	102733933	1460	6	0.333	Exon(exon4of4)	25139	CCDC168	643677
422115	14q	19975713	19976448	736	9	0.444	Promoter(<=1kb)	269	OR4K15	81127
422115	14q	21634137	21634589	453	9	0.556	Promoter(<=1kb)	351	OR10G2	26534
422115	14q	22633879	22634450	572	9	0.333	Exon(exon2of2)	32212	ABHD4	63874
422115	14q	44504986	44506403	1418	6	0.667	Promoter(<=1kb)	880	FSCB	84075
422115	14q	70457532	70458540	1009	9	0.333	Exon(exon2of2)	5358	ADAM21	8747
422115	14q	94587512	94587839	328	6	0.5	Exon(exon2of2)	-4219	SERPINA3	12
422115	14q	104939262	104945444	6183	20	0.4	5'UTR	7102	PLD4	122618
422115	14q	104946867	104953878	7012	36	0.556	Promoter(1-2kb)	1524	AHNAK2	113146
422115	15q	23439979	23442067	2089	13	0.538	5'UTR	5167	GOLGA6L2	283685
422115	15q	85579423	85582073	2651	15	0.6	Promoter(<=1kb)	-837	AKAP13	11214
422115	15q	99129423	99132517	3095	6	0.333	Exon(exon4of5)	7225	TTC23	64927
422115	15q	100569472	100570097	626	6	0.833	Promoter(<=1kb)	534	LINS1	55180
422115	16p	768559	770215	1657	7	0.429	Promoter(<=1kb)	0	MIR662	724032
422115	16p	1486371	1488463	2093	8	0.75	Promoter(<=1kb)	4	PTX4	390667
422115	16p	4883938	4885649	1712	6	0.833	Exon(exon22of22)	4687	PPL	5493
422115	16p	27362551	27363079	529	6	0.167	3'UTR	7203	IL4R	3566
422115	16q	74391401	74392004	604	9	0.667	Exon(exon7of7)	13523	NPIPB15	440348
422115	16q	88714632	88717113	2482	7	0.429	Promoter(<=1kb)	0	MIR4722	100616167
422115	16q	89100686	89101050	365	8	0.625	Promoter(<=1kb)	24	ACSF3	197322
422115	16q	89226863	89228419	1557	10	0.6	Promoter(2-3kb)	2229	ZNF778	197320
422115	17p	10638198	10641099	2902	7	0.286	Exon(exon19of41)	-8169	MYH3	4621
422115	17p	21300581	21300954	374	8	0.75	3'UTR	9112	MAP2K3	5606
422115	17q	76293419	76294016	598	6	0.5	Promoter(2-3kb)	-2167	QRICH2	84074
422115	17q	81645135	81645417	283	6	0.333	Promoter(2-3kb)	2722	TSPAN10	83882
422115	18p	11609904	11610469	566	8	0.625	Promoter(<=1kb)	308	SLC35G4	646000
422115	18q	58535186	58537515	2330	10	0.3	Promoter(<=1kb)	0	ALPK2	115701
422115	19p	4510548	4513547	3000	17	0.471	Exon(exon3of6)	4157	PLIN4	729359
422115	19p	5455600	5456439	840	7	0.571	Promoter(<=1kb)	183	ZNRF4	148066
422115	19p	8111125	8112061	937	6	0.833	Promoter(<=1kb)	0	FBN3	84467
422115	19p	8937644	8939234	1591	6	0.667	Exon(exon5of84)	-41554	MUC16	94025
422115	19p	8946313	8952171	5859	21	0.619	Exon(exon3of84)	29171	MUC16	94025
422115	19p	8959116	8962299	3184	12	0.75	Exon(exon3of84)	19043	MUC16	94025
422115	19p	8963397	8966905	3509	7	0.857	Exon(exon3of84)	14437	MUC16	94025
422115	19p	8971838	8978096	6259	16	0.562	Exon(exon1of84)	3246	MUC16	94025
422115	19p	17282085	17284246	2162	8	0.5	Promoter(<=1kb)	0	ANKLE1	126549

422115	19p	18264798	18267409	2612	12	0.583	5'UTR	7002	IQCN	80726
422115	19p	21971930	21974500	2571	10	0.7	Exon(exon4of4)	14408	ZNF208	7757
422115	19p	22663799	22665034	1236	6	0.833	Exon(exon4of4)	29475	ZNF492	57615
422115	19p	22756205	22759523	3319	12	0.667	3'UTR	10459	ZNF99	7652
422115	19p	23743906	23745300	1395	6	0.333	Exon(exon4of4)	13537	ZNF681	148213
422115	19q	34943334	34944685	1352	6	0.333	3'UTR	9723	ZNF30	90075
422115	19q	36996730	36997597	868	10	0.6	Exon(exon10of10)	5677	ZNF568	374900
422115	19q	39877222	39877880	659	7	0.429	Exon(exon20of28)	9412	FCGBP	8857
422115	19q	39893102	39894316	1215	8	0.625	Exon(exon12of28)	-5810	FCGBP	8857
422115	19q	40880231	40880622	392	6	0.333	Promoter(<=1kb)	-141	CYP2A7	1549
422115	19q	43913423	43914878	1456	8	0.5	Exon(exon10of10)	4861	ZNF45	7596
422115	19q	53164551	53166239	1689	6	0.167	Exon(exon4of4)	-5476	ZNF347	84671
422115	19q	55912302	55913166	865	6	0.667	Exon(exon5of12)	19145	NLRP13	126204
422115	19q	58368293	58368875	583	8	0.375	Exon(exon3of3)	-5445	ZNF497	162968
422115	20p	5922421	5923394	974	8	0.5	Exon(exon4of5)	6923	CHGB	1114
422115	20q	63349752	63350772	1021	6	0.5	3'UTR	3794	CHRNA4	1137
422115	20q	63561666	63565531	3866	12	0.667	Promoter(<=1kb)	-61	HELZ2	85441
422115	21q	44600627	44601692	1066	10	0.7	Promoter(<=1kb)	30	KRTAP10-7	386675
422115	22q	22352950	22353380	431	16	0.5	Exon(exon1of2)	30478	BMS1P20	96610
422115	22q	36191154	36191906	753	7	0.714	3'UTR	9971	APOL4	80832
422115	22q	39100331	39102033	1703	7	0.714	Promoter(<=1kb)	52	APOBEC3H	164668
422115	23p	8170039	8170141	103	6	0.5	Promoter(1-2kb)	1126	VCX2	51480
422115	23p	35802148	35803010	863	7	0.571	5'UTR	3357	MAGEB16	139604
422115	23q	136874183	136874416	234	8	0.75	Promoter(<=1kb)	-201	RBMX	27316
430477	1p	12029085	12030097	1013	6	0.833	Promoter(<=1kb)	0	MIR6729	102466982
430477	1p	18481042	18482032	991	11	0.727	Promoter(<=1kb)	60	KLHDC7A	127707
430477	1p	23874604	23875430	827	8	0.5	Exon(exon2of2)	-6310	FUCA1	2517
430477	1p	40067594	40067675	82	6	0	Promoter(<=1kb)	324	CAP1	10487
430477	1p	89186388	89186419	32	9	0.556	Promoter(<=1kb)	107	GBP4	115361
430477	1q	152302977	152305228	2252	10	0.6	Exon(exon3of3)	-7348	FLG-AS1	339400
430477	1q	152306380	152313891	7512	30	0.567	Promoter(<=1kb)	0	FLG-AS1	339400
430477	1q	156669844	156670886	1043	6	1	Exon(exon4of4)	6521	NES	10763
430477	1q	240207634	240208254	621	13	0.462	Exon(exon5of18)	-27517	FMN2	56776
430477	1q	247841312	247841582	271	6	0.833	Promoter(<=1kb)	314	OR11L1	391189
430477	1q	247896121	247896502	382	6	0.5	Promoter(<=1kb)	534	OR2W3	343171
430477	1q	247949325	247949738	414	9	0.333	Promoter(<=1kb)	467	OR2L8	391190
430477	1q	248573992	248574210	219	6	0.833	Promoter(<=1kb)	547	OR2T34	127068
430477	1q	248681658	248682198	541	7	0.571	Promoter(<=1kb)	130	OR14I1	401994
430477	2p	48580657	48582454	1798	7	0.571	Promoter(<=1kb)	0	STON1	11037
430477	2p	73450706	73453381	2676	7	0.571	Promoter(<=1kb)	0	ALMS1	7840
430477	2q	102351547	102351902	356	7	0.429	Exon(exon11of11)	-4027	IL18R1	8809
430477	2q	132783534	132785001	1468	7	0.429	Promoter(1-2kb)	-1511	NCKAP5	344148
430477	2q	184936178	184937636	1459	6	0.333	Exon(exon4of4)	69813	ZNF804A	91752
430477	2q	185789865	185794632	4768	11	0.727	Promoter(<=1kb)	0	FSIP2	401024
430477	2q	185805377	185808170	2794	6	0.333	Promoter(<=1kb)	0	FSIP2	401024
430477	2q	238130271	238131546	1276	7	0.286	Promoter(1-2kb)	1323	ESPNL	339768
430477	3p	75737230	75739007	1778	9	0.556	Promoter(<=1kb)	0	MIR4273	100422955
430477	3q	98264413	98265137	725	6	0.667	Promoter(<=1kb)	128	OR5H6	79295
430477	3q	194341097	194342571	1475	7	0.714	Exon(exon2of2)	8747	CPN2	1370
430477	4p	5988383	5989749	1367	7	0.571	Promoter(<=1kb)	0	C4orf50	389197
430477	4p	6300792	6302360	1569	7	0.857	Exon(exon8of8)	6021	WFS1	7466
430477	4p	8227004	8228508	1505	7	0.143	Promoter(<=1kb)	-24	SH3TC1	54436
430477	4q	185458217	185460011	1795	9	0.556	Promoter(<=1kb)	0	CCDC110	256309
430477	5p	795818	796237	420	6	0.5	3'UTR	4908	ZDHHC11	79844
430477	5q	67163994	67165736	1743	6	0.5	Exon(exon29of29)	20561	CD180	4064
430477	5q	83537326	83540001	2676	7	0.286	Promoter(1-2kb)	1712	VCAN	1462
430477	5q	140848579	140850786	2208	9	0.556	Promoter(<=1kb)	807	PCDHA9	9752
430477	5q	151565922	151568158	2237	9	0.778	Promoter(<=1kb)	786	FAT2	2196
430477	6p	26370344	26370479	136	6	0.5	Promoter(<=1kb)	0	BTN3A2	11118
430477	6p	46858240	46859389	1150	7	0.429	Exon(exon17of21)	3915	ADGRF5	221395
430477	6q	64591274	64591961	688	10	0.5	Exon(exon26of43)	121374	EYS	346007
430477	6q	149888581	149889987	1407	6	0.667	Promoter(<=1kb)	-116	RAET1E-AS1	100652739
430477	6q	159231899	159234370	2472	12	0.583	Exon(exon11of23)	13602	FNDC1	84624
430477	7p	12369637	12370736	1100	6	0.667	3'UTR	-13307	VWDE	221806
430477	7p	38353718	38353991	274	8	0.625	Exon(exon2of2)	3699	TRG-AS1	100506776
430477	7p	56021087	56021209	123	6	0.5	Exon(exon2of7)	12947	PSPH	5723
430477	7q	100958721	100960873	2153	55	0.455	Promoter(<=1kb)	756	MUC3A	4584
430477	7q	101034305	101040583	6279	31	0.548	Exon(exon3of12)	-3128	MUC17	140453
430477	7q	149818015	149819792	1778	6	0.667	Promoter(2-3kb)	-2352	SSPO	23145
430477	7q	149824094	149826495	2402	6	0.667	Promoter(<=1kb)	0	SSPO	23145
430477	8p	10607245	10608432	1188	8	0.5	Exon(exon4of4)	46711	RP1L1	94137
430477	8p	10609614	10610142	529	7	0.429	Exon(exon4of4)	45001	RP1L1	94137
430477	8p	12132477	12133601	1125	6	0.833	Promoter(<=1kb)	837	USP17L7	392197
430477	8p	13021128	13022030	903	8	0.25	Exon(exon5of5)	9115	TRMT9B	57604
430477	8q	123651655	123652634	980	7	0.571	Promoter(<=1kb)	316	KLHL38	340359
430477	8q	138151949	138153046	1098	6	0.5	Promoter(<=1kb)	0	FAM135B	51059
430477	8q	141218050	141219792	1743	6	0.667	5'UTR	8778	SLC45A4	57210

430477	9q	76175237	76175271	35	8	0.875	Exon(exon14of14)	-13368	PCSK5	5125
430477	9q	76705724	76707804	2081	6	0.5	Promoter(<=1kb)	666	PCA3	50652
430477	9q	104598545	104599361	817	6	0.333	Promoter(<=1kb)	52	OR13C5	138799
430477	9q	135547960	135548795	836	8	0.625	Promoter(1-2kb)	1805	OBP2A	29991
430477	10q	46549378	46550723	1346	25	0.64	Exon(exon3of3)	4807	GPRIN2	9721
430477	10q	89737450	89738561	1112	7	0	Exon(exon20of33)	13874	KIF20B	9585
430477	10q	128102594	128105201	2608	12	0.583	Promoter(<=1kb)	0	MKI67	4288
430477	10q	128106210	128109280	3071	8	0.875	Exon(exon12of14)	-3082	MKI67	4288
430477	11p	244106	244197	92	8	0.5	Promoter(<=1kb)	-232	PSMD13	5719
430477	11p	1194354	1196902	2549	7	0.571	Exon(exon34of49)	-26164	MUC5B	727897
430477	11p	5177978	5178478	501	6	0.167	Promoter(<=1kb)	186	OR52Z1	283110
430477	11p	5422212	5423123	912	11	0.636	Promoter(<=1kb)	101	OR51Q1	390061
430477	11p	5581045	5581738	694	8	0.375	Promoter(<=1kb)	168	OR52B6	340980
430477	11p	5841302	5841883	582	9	0.333	Promoter(<=1kb)	14	OR52E6	390078
430477	11p	11352040	11352736	697	8	0.25	Promoter(<=1kb)	514	CSNK2A3	283106
430477	11p	12293639	12294368	730	6	0.833	Exon(exon29of35)	6739	MICALCL	84953
430477	11p	34916266	34916763	498	6	0.667	Promoter(<=1kb)	0	APIP	51074
430477	11q	58214757	58215722	966	7	0.286	Promoter(<=1kb)	12	OR1S1	219959
430477	11q	82732630	82733184	555	6	0.833	Promoter(<=1kb)	680	FAM181B	220382
430477	11q	85724687	85725825	1139	6	0.5	Promoter(<=1kb)	0	SYTL2	54843
430477	11q	124015601	124016477	877	8	0.25	Promoter(<=1kb)	26	OR10G4	390264
430477	12p	4626571	4628549	1979	9	0.556	Exon(exon5of6)	14054	DYRK4	8798
430477	12p	6453119	6453670	552	6	0.667	Promoter(<=1kb)	633	TAPBPL	55080
430477	12q	52316096	52317765	1670	6	0.667	Exon(exon4of9)	3633	KRT83	3889
430477	13q	24434450	24435347	898	7	0.571	Exon(exon31of34)	19787	PARP4	143
430477	13q	102732474	102733933	1460	6	0.333	Exon(exon4of4)	25139	CCDC168	643677
430477	14q	20060048	20060884	837	8	0.625	Promoter(<=1kb)	3	OR4L1	122742
430477	14q	20640982	20641567	586	6	0.5	Promoter(<=1kb)	124	OR6S1	341799
430477	14q	21634177	21634589	413	6	0.833	Promoter(<=1kb)	351	OR10G2	26534
430477	14q	22633879	22634450	572	9	0.333	Exon(exon2of2)	32212	ABHD4	63874
430477	14q	63599645	63599684	40	8	0.625	Exon(exon2of2)	41723	WDR89	112840
430477	14q	70457532	70458948	1417	11	0.364	Exon(exon2of2)	5358	ADAM21	8747
430477	14q	104939262	104942618	3357	10	0.2	5'UTR	7102	PLD4	122618
430477	14q	104943622	104945444	1823	6	0.667	Exon(exon6of6)	9958	AHNAK2	113146
430477	14q	104948292	104953878	5587	24	0.542	Promoter(1-2kb)	1524	AHNAK2	113146
430477	15q	23440196	23442067	1872	9	0.556	5'UTR	5167	GOLGA6L2	283685
430477	15q	78766033	78766671	639	7	0.429	Promoter(<=1kb)	-920	ADAMTS7	11173
430477	15q	85579423	85582073	2651	16	0.625	Promoter(<=1kb)	-837	AKAP13	11214
430477	16p	1256354	1256985	632	10	0.6	Promoter(<=1kb)	285	TPSD1	23430
430477	16p	27362551	27363079	529	6	0.167	3'UTR	7203	IL4R	3566
430477	16q	74391650	74391897	248	9	0.778	Exon(exon7of7)	13772	NPIPB15	440348
430477	16q	84178965	84180270	1306	6	0.833	Promoter(1-2kb)	1148	TAF1C	9013
430477	16q	88428539	88431889	3351	11	0.364	Exon(exon3of3)	-21391	ZFPM1	161882
430477	16q	88433131	88436097	2967	6	0.667	Exon(exon3of3)	-17183	ZFPM1	161882
430477	16q	89226863	89228289	1427	7	0.571	Promoter(2-3kb)	2229	ZNF778	197320
430477	17p	21300581	21300954	374	8	0.75	3'UTR	9112	MAP2K3	5606
430477	17p	21415458	21416416	959	9	0.889	Exon(exon3of3)	10322	KCNJ12	3768
430477	17q	76293419	76294016	598	6	0.5	Promoter(2-3kb)	-2167	QRICH2	84074
430477	17q	81645135	81645417	283	6	0.333	Promoter(2-3kb)	2722	TSPAN10	83882
430477	18p	11609646	11610350	705	11	0.818	Promoter(<=1kb)	50	SLC35G4	646000
430477	18q	58535186	58537515	2330	9	0.333	Promoter(<=1kb)	0	ALPK2	115701
430477	19p	4510548	4513547	3000	17	0.412	Exon(exon3of6)	4157	PLIN4	729359
430477	19p	5455600	5456439	840	7	0.571	Promoter(<=1kb)	183	ZNRF4	148066
430477	19p	8946313	8951868	5556	15	0.6	Exon(exon3of84)	29474	MUC16	94025
430477	19p	8959116	8962299	3184	10	0.7	Exon(exon3of84)	19043	MUC16	94025
430477	19p	8972751	8978096	5346	17	0.529	Exon(exon1of84)	3246	MUC16	94025
430477	19p	15087213	15088040	828	11	0.273	Promoter(<=1kb)	233	OR1I1	126370
430477	19p	18264753	18267409	2657	8	0.5	5'UTR	7002	IQCN	80726
430477	19p	23743906	23745300	1395	6	0.333	Exon(exon4of4)	13537	ZNF681	148213
430477	19q	36996730	36997597	868	10	0.7	Exon(exon10of10)	5677	ZNF568	374900
430477	19q	39877222	39877880	659	6	0.5	Exon(exon20of28)	9412	FCGBP	8857
430477	19q	39886005	39886439	435	6	0.5	Promoter(<=1kb)	853	FCGBP	8857
430477	19q	40880128	40880622	495	7	0.286	Promoter(<=1kb)	-38	CYP2A7	1549
430477	19q	43203948	43205504	1557	6	0.667	Promoter(<=1kb)	0	PSG4	5672
430477	19q	43913423	43914878	1456	8	0.5	Exon(exon10of10)	4861	ZNF45	7596
430477	19q	43966037	43967171	1135	6	0.167	Promoter(<=1kb)	-691	ZNF155	7711
430477	19q	44106512	44108078	1567	7	0	Exon(exon6of6)	-4103	ZNF225	7768
430477	19q	48873325	48875925	2601	9	0.556	Promoter(<=1kb)	904	PPP1R15A	23645
430477	19q	51745958	51746963	1006	6	0.5	Exon(exon3of3)	3848	FPR1	2357
430477	19q	55358736	55359651	916	6	0.667	Promoter(<=1kb)	0	FAM71E2	284418
430477	19q	55517821	55518176	356	7	1	Exon(exon14of14)	18127	SBK2	646643
430477	19q	55911888	55913077	1190	6	0.667	Exon(exon5of12)	19234	NLRP13	126204
430477	20q	63349752	63350772	1021	6	0.5	3'UTR	3794	CHRNA4	1137
430477	20q	63561666	63565531	3866	13	0.692	Promoter(<=1kb)	-61	HELZ2	85441
430477	21q	26843740	26844859	1120	6	0.667	Promoter(<=1kb)	0	ADAMTS1	9510
430477	21q	44600627	44601692	1066	9	0.778	Promoter(<=1kb)	30	KRTAP10-7	386675
430477	22q	36191154	36191906	753	7	0.714	3'UTR	9971	APOL4	80832

430477	23p	8170039	8170141	103	6	0.5	Promoter(1-2kb)	1126	VCX2	51480
430477	23q	136874183	136874416	234	6	0.833	Promoter(<=1kb)	-201	RBMX	27316
430477	23q	141906066	141906494	429	7	0.571	Promoter(1-2kb)	1264	MAGEC1	9947
432194	1p	978953	979847	895	6	0.5	Promoter(1-2kb)	1182	PERM1	84808
432194	1p	12847526	12847995	470	9	0.444	Promoter(<=1kb)	730	HNRNPCL1	343069
432194	1p	12859036	12860212	1177	14	0.5	Promoter(1-2kb)	1950	PRAMEF2	65122
432194	1p	12861255	12861740	486	9	0.333	Exon(exon4of4)	4169	PRAMEF2	65122
432194	1p	13370686	13370957	272	7	0.429	Promoter(<=1kb)	943	PRAMEF19	645414
432194	1p	16058491	16060000	1510	10	0.9	Exon(exon5of7)	6168	CLCNKB	1188
432194	1p	40067594	40067675	82	6	0	Promoter(<=1kb)	324	CAP1	10487
432194	1p	89186388	89186419	32	9	0.556	Promoter(<=1kb)	107	GBP4	115361
432194	1q	152218469	152221375	2907	17	0.706	Promoter(2-3kb)	2818	HRNR	388697
432194	1q	152302829	152313891	11063	39	0.615	Promoter(<=1kb)	0	FLG-AS1	339400
432194	1q	156669844	156670886	1043	6	1	Exon(exon4of4)	6521	NES	10763
432194	1q	201206099	201209837	3739	11	0.545	Promoter(1-2kb)	1017	IGFN1	91156
432194	1q	228315976	228317998	2023	6	0.5	Exon(exon50of81)	6492	OBSCN	84033
432194	1q	247841312	247841582	271	6	0.833	Promoter(<=1kb)	314	OR11L1	391189
432194	1q	247895950	247896410	461	6	0.5	Promoter(<=1kb)	363	OR2W3	343171
432194	1q	248294677	248295458	782	7	0.571	Promoter(<=1kb)	142	OR2T12	127064
432194	2p	48580657	48582454	1798	7	0.571	Promoter(<=1kb)	0	STON1	11037
432194	2q	102351547	102351902	356	7	0.429	Exon(exon11of11)	-4027	IL18R1	8809
432194	2q	185789865	185794632	4768	10	0.8	Promoter(<=1kb)	0	FSIP2	401024
432194	2q	217847583	217848559	977	6	0.833	Exon(exon19of33)	-5423	TNS1	7145
432194	2q	233713134	233713783	650	12	0.667	Promoter(<=1kb)	142	UGT1A5	54579
432194	2q	238130416	238131546	1131	6	0.333	Promoter(1-2kb)	1468	ESPNL	339768
432194	3p	75736880	75739243	2364	61	0.623	Promoter(<=1kb)	0	MIR4273	100422955
432194	3q	98169021	98169594	574	6	0.667	Exon(exon2of2)	19695	OR5H14	403273
432194	3q	98264413	98265098	686	7	0.571	Promoter(<=1kb)	128	OR5H6	79295
432194	3q	194341097	194342571	1475	7	0.714	Exon(exon2of2)	8747	CPN2	1370
432194	4p	5988383	5989749	1367	7	0.571	Promoter(<=1kb)	0	C4orf50	389197
432194	4p	6300792	6302360	1569	6	0.833	Exon(exon8of8)	6021	WFS1	7466
432194	4p	8227004	8228508	1505	9	0.222	Promoter(<=1kb)	-24	SH3TC1	54436
432194	4p	10443803	10446224	2422	6	0.333	Exon(exon3of3)	10952	ZNF518B	85460
432194	4q	121036404	121037542	1139	6	0.333	Promoter(1-2kb)	1442	NDNF	79625
432194	5q	83537326	83539905	2580	7	0.286	Promoter(1-2kb)	1712	VCAN	1462
432194	5q	140848579	140850786	2208	6	0.5	Promoter(<=1kb)	807	PCDHA9	9752
432194	5q	141100771	141102758	1988	13	0.308	Promoter(<=1kb)	298	PCDHB3	56132
432194	5q	141122761	141123949	1189	6	0.833	Promoter(<=1kb)	943	PCDHB4	56131
432194	5q	151565922	151568158	2237	9	0.778	Promoter(<=1kb)	786	FAT2	2196
432194	6p	42745312	42746041	730	6	0.667	Promoter(<=1kb)	62	TBCC	6903
432194	6p	46858771	46859502	732	7	0.571	Exon(exon17of21)	3802	ADGRF5	221395
432194	6q	159233455	159234370	916	10	0.5	Exon(exon11of23)	15158	FNDC1	84624
432194	6q	168307821	168309048	1228	8	0.5	5'UTR	10662	DACT2	168002
432194	7p	12369637	12370736	1100	6	0.667	3'UTR	-13307	VWDE	221806
432194	7p	38353718	38353991	274	8	0.625	Exon(exon2of2)	3699	TRG-AS1	100506776
432194	7p	45082865	45085548	2684	7	0.429	Promoter(1-2kb)	-1465	NACAD	23148
432194	7p	53035678	53036385	708	7	1	Promoter(<=1kb)	45	POM121L12	285877
432194	7q	64991278	64992758	1481	6	0.667	Promoter(<=1kb)	-242	ZNF117	51351
432194	7q	100958721	100960873	2153	56	0.464	Promoter(<=1kb)	756	MUC3A	4584
432194	7q	100991195	100993127	1933	8	0.625	Exon(exon5of15)	-19927	MUC12	10071
432194	7q	100995575	100995785	211	6	0.833	Exon(exon5of15)	-17269	MUC12	10071
432194	7q	101004258	101004836	579	9	0.889	Exon(exon5of15)	-8218	MUC12	10071
432194	8p	11331194	11332026	833	6	0.667	Promoter(<=1kb)	306	SLC35G5	83650
432194	8p	12132686	12133940	1255	6	0.833	Promoter(<=1kb)	498	USP17L7	392197
432194	8p	12137448	12138752	1305	7	0.857	Promoter(<=1kb)	325	USP17L2	377630
432194	8p	13021128	13022030	903	8	0.25	Exon(exon5of5)	9115	TRMT9B	57604
432194	9p	34723747	34724799	1053	6	0.833	Exon(exon4of4)	4689	FAM205A	259308
432194	9q	76705724	76707804	2081	6	0.5	Promoter(<=1kb)	666	PCA3	50652
432194	9q	104504315	104505023	709	6	0.5	Promoter(<=1kb)	52	OR13F1	138805
432194	9q	104598545	104599361	817	12	0.583	Promoter(<=1kb)	52	OR13C5	138799
432194	9q	122553263	122554071	809	7	0.429	Promoter(<=1kb)	93	OR1N2	138882
432194	9q	122628595	122629398	804	6	0.333	Promoter(<=1kb)	175	OR1B1	347169
432194	9q	122749914	122750547	634	6	0.833	Promoter(<=1kb)	174	OR1L6	392390
432194	9q	135484803	135487213	2411	11	0.455	Promoter(1-2kb)	1440	PPP1R26	9858
432194	9q	135547960	135548795	836	8	0.625	Promoter(1-2kb)	1805	OBP2A	29991
432194	10p	47663	48605	943	6	0.833	Promoter(<=1kb)	664	TUBB8	347688
432194	10q	46549378	46550723	1346	27	0.667	Exon(exon3of3)	4807	GPRIN2	9721
432194	10q	48913676	48914136	461	6	0.833	Promoter(<=1kb)	96	LRRC18	474354
432194	11p	244106	244197	92	8	0.5	Promoter(<=1kb)	-232	PSMD13	5719
432194	11p	1194354	1196902	2549	7	0.571	Exon(exon34of49)	-26164	MUC5B	727897
432194	11p	1245486	1248605	3120	11	0.455	Promoter(1-2kb)	1071	MUC5B-AS1	112577518
432194	11p	5046754	5047432	679	7	0.571	Promoter(<=1kb)	228	OR52J3	119679
432194	11p	5177811	5178478	668	7	0.143	Promoter(<=1kb)	186	OR52Z1	283110
432194	11p	5323451	5324256	806	7	0.571	Promoter(<=1kb)	41	OR51B2	79345
432194	11p	5389704	5390350	647	7	0.429	Promoter(<=1kb)	327	OR51M1	390059
432194	11p	5422212	5423123	912	11	0.636	Promoter(<=1kb)	101	OR51Q1	390061
432194	11p	5581045	5581738	694	8	0.375	Promoter(<=1kb)	168	OR52B6	340980

432194	11p	5841302	5841883	582	9	0.333	Promoter(<=1kb)	14	OR52E6	390078
432194	11p	5884818	5885061	244	7	0.429	Promoter(<=1kb)	547	OR52E4	390081
432194	11p	11351961	11352736	776	9	0.222	Promoter(<=1kb)	514	CSNK2A3	283106
432194	11p	12293639	12294368	730	6	0.833	Exon(exon29of35)	6739	MICALCL	84953
432194	11p	18173280	18173901	622	6	0.333	Promoter(<=1kb)	443	MRGPRX4	117196
432194	11q	55827393	55827640	248	7	0.714	Promoter(<=1kb)	174	OR5L2	26338
432194	11q	58214757	58215722	966	8	0.25	Promoter(<=1kb)	12	OR1S1	219959
432194	11q	82732630	82733184	555	6	0.833	Promoter(<=1kb)	680	FAM181B	220382
432194	11q	85724687	85725825	1139	6	0.5	Promoter(<=1kb)	0	SYTL2	54843
432194	11q	123906790	123907324	535	6	0.667	Promoter(<=1kb)	644	OR8D4	338662
432194	11q	124038366	124038988	623	7	1	Promoter(<=1kb)	13	OR10G7	390265
432194	11q	124382526	124383285	760	8	0.625	Promoter(<=1kb)	58	OR8B2	26595
432194	12p	4626568	4628549	1982	11	0.545	Exon(exon5of6)	14051	DYRK4	8798
432194	12p	8222174	8223514	1341	6	0.667	Exon(exon5of6)	4073	FAM90A1	55138
432194	12q	52316096	52317765	1670	7	0.571	Exon(exon4of9)	3633	KRT83	3889
432194	13q	24434450	24435347	898	7	0.571	Exon(exon31of34)	19787	PARP4	143
432194	13q	102732474	102733933	1460	6	0.333	Exon(exon4of4)	25139	CCDC168	643677
432194	14q	20060048	20060884	837	8	0.625	Promoter(<=1kb)	3	OR4L1	122742
432194	14q	21634137	21634589	453	9	0.556	Promoter(<=1kb)	351	OR10G2	26534
432194	14q	22633879	22634450	572	9	0.333	Exon(exon2of2)	32212	ABHD4	63874
432194	14q	44504986	44506403	1418	6	0.667	Promoter(<=1kb)	880	FSCB	84075
432194	14q	94587512	94587839	328	6	0.5	Exon(exon2of2)	-4219	SERPINA3	12
432194	14q	104947943	104951938	3996	12	0.333	Exon(exon6of6)	3464	AHNAK2	113146
432194	15q	20534480	20535014	535	8	0.5	Exon(exon8of9)	6786	GOLGA6L6	727832
432194	15q	23439979	23442067	2089	14	0.5	5'UTR	5167	GOLGA6L2	283685
432194	15q	40621642	40623696	2055	6	0.333	Promoter(<=1kb)	0	KNL1	57082
432194	15q	99129423	99132517	3095	6	0.333	Exon(exon4of5)	7225	TTC23	64927
432194	16p	1228744	1229731	988	13	0.692	Promoter(<=1kb)	431	TPSB2	64499
432194	16p	1486371	1488463	2093	8	0.75	Promoter(<=1kb)	4	PTX4	390667
432194	16q	74391401	74392004	604	10	0.7	Exon(exon7of7)	13523	NPIPB15	440348
432194	16q	88428539	88429600	1062	6	0.333	Exon(exon3of3)	-23680	ZFPM1	161882
432194	16q	89227206	89228419	1214	8	0.625	Promoter(2-3kb)	2572	ZNF778	197320
432194	17p	744946	746966	2021	7	1	3'UTR	5072	GEMIN4	50628
432194	17p	21300581	21300978	398	12	0.75	3'UTR	9112	MAP2K3	5606
432194	17q	41586466	41586829	364	6	0.833	Promoter(<=1kb)	66	KRT14	3861
432194	17q	53823723	53824891	1169	6	0.667	Promoter(<=1kb)	796	KIF2B	84643
432194	17q	76293419	76294016	598	6	0.5	Promoter(2-3kb)	-2167	QRICH2	84074
432194	17q	81645135	81645607	473	7	0.286	Promoter(2-3kb)	2722	TSPAN10	83882
432194	18p	11609646	11610491	846	13	0.769	Promoter(<=1kb)	50	SLC35G4	646000
432194	18q	58535186	58537515	2330	9	0.333	Promoter(<=1kb)	0	ALPK2	115701
432194	19p	1004688	1005532	845	6	0.5	Exon(exon3of9)	4269	GRIN3B	116444
432194	19p	5455600	5456439	840	6	0.5	Promoter(<=1kb)	183	ZNRF4	148066
432194	19p	8937644	8939234	1591	6	0.667	Exon(exon5of84)	-41554	MUC16	94025
432194	19p	8946313	8952171	5859	19	0.579	Exon(exon3of84)	29171	MUC16	94025
432194	19p	8959116	8962299	3184	12	0.75	Exon(exon3of84)	19043	MUC16	94025
432194	19p	8971838	8978096	6259	15	0.533	Exon(exon1of84)	3246	MUC16	94025
432194	19p	12430718	12432437	1720	8	0.375	3'UTR	8584	ZNF443	10224
432194	19p	18264753	18267409	2657	8	0.5	5'UTR	7002	IQCN	80726
432194	19p	21971930	21974500	2571	7	0.714	Exon(exon4of4)	14408	ZNF208	7757
432194	19p	22756294	22759533	3240	15	0.533	3'UTR	10449	ZNF99	7652
432194	19q	40394650	40396060	1411	7	0.571	3'UTR	-4469	HIPK4	147746
432194	19q	43846955	43848536	1582	6	0.833	3'UTR	13450	ZNF283	284349
432194	19q	43913423	43914878	1456	9	0.556	Exon(exon10of10)	4861	ZNF45	7596
432194	19q	43996326	43997366	1041	6	0.5	Exon(exon5of5)	5419	LOC10192806	101928063
432194	19q	44106512	44108078	1567	7	0	Exon(exon6of6)	-4103	ZNF225	7768
432194	19q	52365769	52366744	976	6	1	Exon(exon6of6)	-3173	ZNF880	400713
432194	19q	52437918	52439242	1325	7	0.429	Exon(exon4of4)	6504	ZNF534	147658
432194	19q	55912302	55913166	865	6	0.667	Exon(exon5of12)	19145	NLRP13	126204
432194	20p	5922577	5923382	806	6	0.5	Exon(exon4of5)	7079	CHGB	1114
432194	20q	63561666	63565531	3866	11	0.636	Promoter(<=1kb)	-61	HELZ2	85441
432194	21q	26843740	26844859	1120	6	0.667	Promoter(<=1kb)	0	ADAMTS1	9510
432194	22q	22352950	22353380	431	16	0.5	Exon(exon1of2)	30478	BMS1P20	96610
432194	22q	22646443	22647152	710	7	0.286	Promoter(<=1kb)	0	GGTLC2	91227
432194	22q	36191154	36191906	753	6	0.667	3'UTR	9971	APOL4	80832
432194	23p	8170039	8170141	103	6	0.5	Promoter(1-2kb)	1126	VCX2	51480
442043	1p	978953	979847	895	6	0.5	Promoter(1-2kb)	1182	PERM1	84808
442043	1p	16058491	16060000	1510	10	0.9	Exon(exon5of7)	6168	CLCNKB	1188
442043	1p	23874604	23875430	827	9	0.556	Exon(exon2of2)	-6310	FUCA1	2517
442043	1p	40067594	40067675	82	6	0	Promoter(<=1kb)	324	CAP1	10487
442043	1q	152213274	152213320	47	8	0.5	Exon(exon3of3)	10873	HRNR	388697
442043	1q	152219211	152221375	2165	18	0.722	Promoter(2-3kb)	2818	HRNR	388697
442043	1q	152304920	152306213	1294	6	0.667	Exon(exon3of3)	-6363	FLG-AS1	339400
442043	1q	152307253	152307694	442	6	0.667	Exon(exon3of3)	-4882	FLG-AS1	339400
442043	1q	152310318	152313891	3574	14	0.571	Promoter(<=1kb)	0	FLG-AS1	339400
442043	1q	169542317	169542882	566	6	0.167	Exon(exon13of25)	-26572	F5	2153
442043	1q	201206099	201209856	3758	13	0.615	Promoter(1-2kb)	1017	IGFN1	91156
442043	1q	228315976	228317998	2023	7	0.571	Exon(exon50of81)	6492	OBSCN	84033

442043	1q	247841312	247841582	271	6	0.833	Promoter(<=1kb)	314	OR11L1	391189
442043	1q	247949325	247949738	414	10	0.3	Promoter(<=1kb)	467	OR2L8	391190
442043	1q	248626203	248626898	696	6	0.333	Promoter(<=1kb)	292	OR2T11	127077
442043	2p	29002636	29003646	1011	6	0.333	Exon(exon5of20)	-10821	TOGARAM2	165186
442043	2p	48580657	48582454	1798	7	0.571	Promoter(<=1kb)	0	STON1	11037
442043	2q	102351547	102351902	356	7	0.429	Exon(exon11of11)	-4027	IL18R1	8809
442043	2q	178739433	178741811	2379	6	0.5	Exon(exon45of191)	26014	TTN	7273
442043	2q	217817751	217818645	895	6	0.333	Promoter(2-3kb)	-2383	TNS1	7145
442043	2q	217847583	217848559	977	6	0.833	Exon(exon19of33)	-5423	TNS1	7145
442043	2q	238130271	238131546	1276	8	0.375	Promoter(1-2kb)	1323	ESPNL	339768
442043	2q	240041845	240042154	310	6	0.167	Downstream(2-3kb)	3918	OR6B3	150681
442043	3p	75736880	75739243	2364	77	0.636	Promoter(<=1kb)	0	MIR4273	100422955
442043	3q	98264413	98265098	686	7	0.571	Promoter(<=1kb)	128	OR5H6	79295
442043	3q	196947388	196948102	715	6	0.5	3'UTR	3828	PIGZ	80235
442043	4p	5988383	5989749	1367	7	0.571	Promoter(<=1kb)	0	C4orf50	389197
442043	4p	6300792	6302360	1569	6	0.833	Exon(exon8of8)	6021	WFS1	7466
442043	4p	10443803	10446224	2422	6	0.333	Exon(exon3of3)	10952	ZNF518B	85460
442043	4q	154489498	154491312	1815	9	0.556	Promoter(<=1kb)	22	DCHS2	54798
442043	4q	185458217	185460011	1795	9	0.556	Promoter(<=1kb)	0	CCDC110	256309
442043	4q	186619481	186621582	2102	7	0.429	Exon(exon10of27)	-9411	FAT1	2195
442043	5q	54518000	54519665	1666	6	0.333	Promoter(<=1kb)	135	SNX18	112574
442043	5q	79728956	79730716	1761	8	0.25	Exon(exon2of13)	-7426	CMYA5	202333
442043	5q	79731782	79734523	2742	13	0.308	Exon(exon2of13)	-3619	CMYA5	202333
442043	5q	83537326	83539905	2580	6	0.333	Promoter(1-2kb)	1712	VCAN	1462
442043	5q	140848579	140850786	2208	6	0.5	Promoter(<=1kb)	807	PCDHA9	9752
442043	5q	141955676	141957689	2014	6	0.667	Promoter(<=1kb)	-639	RNF14	9604
442043	5q	148826877	148828070	1194	6	1	Promoter(1-2kb)	1632	ADRB2	154
442043	5q	151521550	151522069	520	6	0.667	Promoter(<=1kb)	79	MIR6499	102465246
442043	5q	151565922	151568158	2237	9	0.778	Promoter(<=1kb)	786	FAT2	2196
442043	6p	1312843	1313745	903	6	0.5	Promoter(<=1kb)	745	FOXQ1	94234
442043	6p	26370344	26370479	136	6	0.5	Promoter(<=1kb)	0	BTN3A2	11118
442043	6p	46858771	46859389	619	6	0.5	Exon(exon17of21)	3915	ADGRF5	221395
442043	6q	64591274	64591961	688	10	0.5	Exon(exon26of43)	121374	EYS	346007
442043	6q	149888581	149890867	2287	7	0.714	Promoter(<=1kb)	0	RAET1E-AS1	100652739
442043	6q	159231899	159234370	2472	12	0.583	Exon(exon11of23)	13602	FNDC1	84624
442043	7p	12369637	12370736	1100	6	0.667	3'UTR	-13307	VWDE	221806
442043	7p	45082865	45084866	2002	7	0.429	Promoter(1-2kb)	-1465	NACAD	23148
442043	7q	64991278	64992758	1481	6	0.667	Promoter(<=1kb)	-242	ZNF117	51351
442043	7q	100958977	100960873	1897	56	0.446	Promoter(1-2kb)	1012	MUC3A	4584
442043	7q	100991195	100992398	1204	7	0.571	Exon(exon5of15)	-20656	MUC12	10071
442043	7q	100995575	100995785	211	7	0.714	Exon(exon5of15)	-17269	MUC12	10071
442043	7q	101034361	101041369	7009	40	0.55	Promoter(2-3kb)	-2342	MUC17	140453
442043	8p	10609614	10612307	2694	10	0.9	Exon(exon4of4)	42836	RP1L1	94137
442043	8p	12137448	12138641	1194	6	0.833	Promoter(<=1kb)	436	USP17L2	377630
442043	8p	13021128	13022030	903	7	0.143	Exon(exon5of5)	9115	TRMT9B	57604
442043	8q	123651655	123652634	980	6	0.5	Promoter(<=1kb)	316	KLHL38	340359
442043	9q	76705179	76707804	2626	8	0.5	Promoter(<=1kb)	121	PCA3	50652
442043	9q	76709263	76710843	1581	9	0.556	Promoter(<=1kb)	0	PRUNE2	158471
442043	9q	87885490	87888819	3330	9	0.667	Promoter(2-3kb)	2613	SPATA31E1	286234
442043	9q	104598641	104599361	721	8	0.625	Promoter(<=1kb)	52	OR13C5	138799
442043	9q	122553263	122554071	809	7	0.429	Promoter(<=1kb)	93	OR1N2	138882
442043	9q	122628595	122629398	804	6	0.333	Promoter(<=1kb)	175	OR1B1	347169
442043	9q	122749914	122750547	634	6	0.833	Promoter(<=1kb)	174	OR1L6	392390
442043	9q	131474936	131475956	1021	6	0.833	Promoter(<=1kb)	0	PRRC2B	84726
442043	9q	133255635	133256205	571	7	1	3'UTR	19009	ABO	28
442043	9q	135484803	135487573	2771	9	0.333	Promoter(1-2kb)	1440	PPP1R26	9858
442043	10p	30027143	30029020	1878	6	0.333	Exon(exon3of4)	30566	JCAD	57608
442043	10q	46549378	46550723	1346	27	0.667	Exon(exon3of3)	4807	GPRIN2	9721
442043	10q	49323169	49325192	2024	7	0.571	Exon(exon3of3)	23895	C10orf71	118461
442043	11p	244106	244197	92	8	0.5	Promoter(<=1kb)	-232	PSMD13	5719
442043	11p	1241677	1243593	1917	6	0.333	Exon(exon31of49)	6083	MUC5B-AS1	112577518
442043	11p	1244757	1248605	3849	16	0.625	Promoter(1-2kb)	1071	MUC5B-AS1	112577518
442043	11p	1250091	1251628	1538	9	0.778	Promoter(<=1kb)	-415	MUC5B-AS1	112577518
442043	11p	5177978	5178478	501	6	0.167	Promoter(<=1kb)	186	OR52Z1	283110
442043	11p	5323451	5324256	806	6	0.5	Promoter(<=1kb)	41	OR51B2	79345
442043	11p	5422212	5423123	912	12	0.667	Promoter(<=1kb)	101	OR51Q1	390061
442043	11p	5581045	5581738	694	8	0.375	Promoter(<=1kb)	168	OR52B6	340980
442043	11p	5841302	5841883	582	9	0.333	Promoter(<=1kb)	14	OR52E6	390078
442043	11p	5884818	5885061	244	7	0.429	Promoter(<=1kb)	547	OR52E4	390081
442043	11p	5986042	5986669	628	7	1	Promoter(<=1kb)	316	OR52L1	338751
442043	11p	11351961	11352736	776	9	0.222	Promoter(<=1kb)	514	CSNK2A3	283106
442043	11p	12293639	12294368	730	6	0.833	Exon(exon29of35)	6739	MICALCL	84953
442043	11p	18173280	18173901	622	6	0.333	Promoter(<=1kb)	443	MRGPRX4	117196
442043	11p	34916266	34916763	498	6	0.667	Promoter(<=1kb)	0	APIP	51074
442043	11q	58214757	58215722	966	8	0.25	Promoter(<=1kb)	12	OR1S1	219959
442043	11q	69295669	69296261	593	6	0.833	Promoter(1-2kb)	1495	MYEOV	26579
442043	11q	85724687	85725825	1139	6	0.5	Promoter(<=1kb)	0	SYTL2	54843

442043	11q	123906790	123907324	535	6	0.667	Promoter(<=1kb)	644	OR8D4	338662
442043	11q	124038366	124038988	623	7	1	Promoter(<=1kb)	13	OR10G7	390265
442043	11q	124382526	124383285	760	8	0.625	Promoter(<=1kb)	58	OR8B2	26595
442043	12p	4627329	4628549	1221	8	0.625	Exon(exon5of6)	14812	DYRK4	8798
442043	12p	31981306	31982507	1202	6	0.333	Exon(exon4of6)	-4659	RESF1	55196
442043	12q	49795819	49797137	1319	6	0.5	Promoter(<=1kb)	0	NCKAP5L	57701
442043	12q	52571389	52572653	1265	6	0.5	Promoter(1-2kb)	1163	KRT74	121391
442043	13q	24434450	24435347	898	7	0.571	Exon(exon31of34)	19787	PARP4	143
442043	13q	25096850	25096984	135	7	0.429	Promoter(<=1kb)	982	PABPC3	5042
442043	13q	102732474	102733933	1460	6	0.333	Exon(exon4of4)	25139	CCDC168	643677
442043	14q	19975713	19976448	736	9	0.444	Promoter(<=1kb)	269	OR4K15	81127
442043	14q	20060048	20060884	837	8	0.625	Promoter(<=1kb)	3	OR4L1	122742
442043	14q	21634137	21634589	453	9	0.556	Promoter(<=1kb)	351	OR10G2	26534
442043	14q	70457520	70458540	1021	13	0.385	Exon(exon2of2)	5346	ADAM21	8747
442043	14q	77377020	77378778	1759	7	0.286	Promoter(<=1kb)	0	SAMD15	161394
442043	14q	104175275	104177810	2536	10	0.3	Exon(exon12of15)	36235	KIF26A	26153
442043	14q	104950428	104953878	3451	9	0.333	Promoter(1-2kb)	1524	AHNAK2	113146
442043	15q	20534480	20534954	475	6	0.333	Exon(exon8of9)	6846	GOLGA6L6	727832
442043	15q	23439979	23442067	2089	13	0.538	5'UTR	5167	GOLGA6L2	283685
442043	15q	73702465	73703760	1296	6	0.833	Promoter(<=1kb)	-149	CD276	80381
442043	15q	85579423	85582073	2651	16	0.625	Promoter(<=1kb)	-837	AKAP13	11214
442043	15q	99129423	99132517	3095	7	0.286	Exon(exon4of5)	7225	TTC23	64927
442043	16p	1221890	1223490	1601	6	1	Promoter(1-2kb)	1285	TPSG1	25823
442043	16p	1486371	1488463	2093	8	0.75	Promoter(<=1kb)	4	PTX4	390667
442043	16p	20963445	20965038	1594	7	0.429	Exon(exon53of62)	62341	LYRM1	57149
442043	16p	28495551	28497395	1845	6	0.333	Promoter(<=1kb)	0	CLN3	1201
442043	16q	74391460	74392004	545	9	0.667	Exon(exon7of7)	13582	NPIPB15	440348
442043	16q	88428339	88429600	1262	7	0.429	Exon(exon3of3)	-23680	ZFPM1	161882
442043	16q	88430623	88431889	1267	6	0.5	Exon(exon3of3)	-21391	ZFPM1	161882
442043	16q	89227206	89228289	1084	7	0.571	Promoter(2-3kb)	2572	ZNF778	197320
442043	17p	2299649	2300159	511	6	0.167	Exon(exon2of19)	-3224	SRR	63826
442043	17p	21300581	21300978	398	11	0.727	3'UTR	9112	MAP2K3	5606
442043	17p	21415470	21416485	1016	6	0.833	Exon(exon3of3)	10334	KCNJ12	3768
442043	17q	81645135	81645417	283	6	0.333	Promoter(2-3kb)	2722	TSPAN10	83882
442043	18p	11609728	11610350	623	9	0.889	Promoter(<=1kb)	132	SLC35G4	646000
442043	18q	58535186	58537515	2330	10	0.3	Promoter(<=1kb)	0	ALPK2	115701
442043	19p	4510548	4513547	3000	16	0.438	Exon(exon3of6)	4157	PLIN4	729359
442043	19p	5455600	5456439	840	8	0.625	Promoter(<=1kb)	183	ZNRF4	148066
442043	19p	8937644	8939234	1591	6	0.667	Exon(exon5of84)	-41554	MUC16	94025
442043	19p	8946313	8952171	5859	21	0.619	Exon(exon3of84)	29171	MUC16	94025
442043	19p	8959116	8962299	3184	12	0.75	Exon(exon3of84)	19043	MUC16	94025
442043	19p	8972467	8973623	1157	7	0.714	Exon(exon1of84)	7719	MUC16	94025
442043	19p	12430718	12432437	1720	8	0.375	3'UTR	8584	ZNF443	10224
442043	19p	15087213	15088040	828	9	0.333	Promoter(<=1kb)	233	OR1I1	126370
442043	19p	18264798	18267409	2612	12	0.583	5'UTR	7002	IQCN	80726
442043	19p	21971930	21974500	2571	8	0.625	Exon(exon4of4)	14408	ZNF208	7757
442043	19p	23743906	23745300	1395	7	0.429	Exon(exon4of4)	13537	ZNF681	148213
442043	19q	36996730	36997597	868	10	0.7	Exon(exon10of10)	5677	ZNF568	374900
442043	19q	37151928	37153149	1222	6	0.833	Exon(exon5of5)	19287	ZNF585A	199704
442043	19q	37885190	37888806	3617	8	0.625	Exon(exon6of6)	17788	WDR87	83889
442043	19q	43913423	43914878	1456	7	0.429	Exon(exon10of10)	4861	ZNF45	7596
442043	19q	43996326	43997366	1041	6	0.5	Exon(exon5of5)	5419	LOC10192806:	101928063
442043	19q	44106512	44108078	1567	7	0	Exon(exon6of6)	-4103	ZNF225	7768
442043	19q	51745958	51746963	1006	7	0.429	Exon(exon3of3)	3848	FPR1	2357
442043	19q	52437918	52439242	1325	7	0.429	Exon(exon4of4)	6504	ZNF534	147658
442043	19q	53164551	53166239	1689	6	0.167	Exon(exon4of4)	-5476	ZNF347	84671
442043	19q	57444351	57445460	1110	6	0.667	Exon(exon3of3)	9004	ZNF749	388567
442043	19q	58368293	58368875	583	7	0.429	Exon(exon3of3)	-5445	ZNF497	162968
442043	20p	5922421	5923394	974	6	0.5	Exon(exon4of5)	6923	CHGB	1114
442043	20p	18315086	18316549	1464	6	0.667	Promoter(2-3kb)	2450	ZNF133	7692
442043	20q	62812284	62813587	1304	9	1	Promoter(2-3kb)	-2657	COL9A3	1299
442043	20q	63349752	63350772	1021	6	0.5	3'UTR	3794	CHRNA4	1137
442043	20q	63561666	63565531	3866	13	0.615	Promoter(<=1kb)	-61	HELZ2	85441
442043	21q	44637474	44638041	568	10	0.4	Promoter(<=1kb)	118	KRTAP10-10	353333
442043	22q	22352950	22353348	399	14	0.571	Exon(exon1of2)	30478	BMS1P20	96610
442043	22q	36191154	36191906	753	7	0.571	3'UTR	9971	APOL4	80832
442043	23p	3320126	3323750	3625	10	0.6	Exon(exon5of7)	22902	MXRA5	25878
442043	23p	8170039	8170141	103	6	0.5	Promoter(1-2kb)	1126	VCX2	51480
442043	23p	8466313	8466392	80	7	0.429	Promoter(<=1kb)	887	VCX3B	425054
442043	23p	35802148	35803010	863	7	0.571	5'UTR	3357	MAGEB16	139604
446356	1p	11766028	11768307	2280	6	0.833	Promoter(<=1kb)	0	C1orf167	284498
446356	1p	16058491	16060000	1510	10	0.9	Exon(exon5of7)	6168	CLCNKB	1188
446356	1p	18481042	18482217	1176	12	0.75	Promoter(<=1kb)	60	KLHDC7A	127707
446356	1p	23874604	23875430	827	9	0.556	Exon(exon2of2)	-6310	FUCA1	2517
446356	1p	40067594	40067675	82	6	0	Promoter(<=1kb)	324	CAP1	10487
446356	1p	89186388	89186419	32	9	0.556	Promoter(<=1kb)	107	GBP4	115361
446356	1q	145872200	145873487	1288	6	0.667	Exon(exon8of12)	-12364	PIAS3	10401

446356	1q	152213274	152213320	47	8	0.5	Exon(exon3of3)	10873	HRNR	388697
446356	1q	152219233	152221375	2143	16	0.688	Promoter(2-3kb)	2818	HRNR	388697
446356	1q	152303673	152313891	10219	37	0.595	Promoter(<=1kb)	0	FLG-AS1	339400
446356	1q	158765805	158766655	851	6	0.5	Promoter(<=1kb)	47	OR6N1	128372
446356	1q	169542317	169542882	566	8	0.125	Exon(exon13of25)	-26572	F5	2153
446356	1q	197101312	197101771	460	6	0.5	Exon(exon18of28)	33373	ASPM	259266
446356	1q	201206099	201209837	3739	13	0.538	Promoter(1-2kb)	1017	IGFN1	91156
446356	1q	214640144	214642954	2811	12	0.5	Exon(exon12of20)	-5013	CENPF	1063
446356	1q	214644872	214647181	2310	10	0.4	Promoter(<=1kb)	-786	CENPF	1063
446356	1q	228315976	228318026	2051	7	0.571	Exon(exon50of81)	6492	OBSCN	84033
446356	1q	247841312	247841582	271	6	0.833	Promoter(<=1kb)	314	OR11L1	391189
446356	1q	247949325	247949738	414	10	0.3	Promoter(<=1kb)	467	OR2L8	391190
446356	1q	248294677	248295458	782	7	0.571	Promoter(<=1kb)	142	OR2T12	127064
446356	1q	248323714	248324466	753	6	0.667	Promoter(<=1kb)	102	OR2M7	391196
446356	1q	248681658	248682198	541	8	0.625	Promoter(<=1kb)	130	OR14I1	401994
446356	2q	167246794	167248478	1685	7	0.714	Promoter(<=1kb)	-204	XIRP2	129446
446356	2q	184936178	184937636	1459	7	0.429	Exon(exon4of4)	69813	ZNF804A	91752
446356	2q	185789865	185794632	4768	10	0.8	Promoter(<=1kb)	0	FSIP2	401024
446356	2q	217847583	217848746	1164	7	0.857	Exon(exon19of33)	-5423	TNS1	7145
446356	2q	233713134	233713783	650	9	0.667	Promoter(<=1kb)	142	UGT1A5	54579
446356	2q	238130271	238131546	1276	7	0.286	Promoter(1-2kb)	1323	ESPNL	339768
446356	3p	31989532	31990905	1374	6	0.333	Exon(exon2of2)	7761	ZNF860	344787
446356	3p	75736880	75739243	2364	77	0.623	Promoter(<=1kb)	0	MIR4273	100422955
446356	3q	98264413	98265137	725	6	0.667	Promoter(<=1kb)	128	OR5H6	79295
446356	3q	194341097	194342571	1475	7	0.714	Exon(exon2of2)	8747	CPN2	1370
446356	3q	196947388	196948102	715	6	0.5	3'UTR	3828	PIGZ	80235
446356	4p	6300792	6302360	1569	6	0.833	Exon(exon8of8)	6021	WFS1	7466
446356	4p	8227004	8228508	1505	8	0.125	Promoter(<=1kb)	-24	SH3TC1	54436
446356	4q	121036404	121037542	1139	6	0.333	Promoter(1-2kb)	1442	NDNF	79625
446356	4q	154489498	154491312	1815	9	0.556	Promoter(<=1kb)	22	DCHS2	54798
446356	4q	186619481	186621582	2102	6	0.333	Exon(exon10of27)	-9411	FAT1	2195
446356	4q	186706638	186708616	1979	6	0.667	Exon(exon2of27)	15217	FAT1	2195
446356	5q	79731782	79734523	2742	13	0.308	Exon(exon2of13)	-3619	CMYA5	202333
446356	5q	83537326	83539905	2580	6	0.333	Promoter(1-2kb)	1712	VCAN	1462
446356	5q	140848579	140850786	2208	7	0.571	Promoter(<=1kb)	807	PCDHA9	9752
446356	5q	141100771	141102758	1988	11	0.273	Promoter(<=1kb)	298	PCDHB3	56132
446356	5q	141122595	141123949	1355	7	0.714	Promoter(<=1kb)	777	PCDHB4	56131
446356	5q	141174000	141175025	1026	7	0.714	Promoter(1-2kb)	1356	PCDHB7	56129
446356	5q	141178631	141180333	1703	6	0.833	Promoter(<=1kb)	841	PCDHB8	56128
446356	5q	141183999	141184688	690	6	0.833	Promoter(2-3kb)	-2473	PCDHB9	56127
446356	5q	141955356	141957660	2305	6	0.667	Promoter(<=1kb)	-668	RNF14	9604
446356	6p	42745312	42746041	730	6	0.667	Promoter(<=1kb)	62	TBCC	6903
446356	6p	46858771	46859502	732	7	0.571	Exon(exon17of21)	3802	ADGRF5	221395
446356	6q	159233455	159234370	916	10	0.5	Exon(exon11of23)	15158	FNDC1	84624
446356	7p	12369637	12370736	1100	6	0.667	3'UTR	-13307	VWDE	221806
446356	7p	45082725	45084866	2142	7	0.429	Promoter(1-2kb)	-1325	NACAD	23148
446356	7q	100958721	100960873	2153	56	0.464	Promoter(<=1kb)	756	MUC3A	4584
446356	7q	100991195	100994184	2990	11	0.727	Exon(exon5of15)	-18870	MUC12	10071
446356	7q	100995547	100995785	239	7	0.857	Exon(exon5of15)	-17269	MUC12	10071
446356	8p	10607245	10608432	1188	8	0.5	Exon(exon4of4)	46711	RP1L1	94137
446356	8p	10609614	10610662	1049	8	0.5	Exon(exon4of4)	44481	RP1L1	94137
446356	8p	12137448	12138641	1194	7	0.857	Promoter(<=1kb)	436	USP17L2	377630
446356	8p	13021128	13022030	903	8	0.25	Exon(exon5of5)	9115	TRMT9B	57604
446356	8q	143916360	143919209	2850	7	0.143	Exon(exon32of32)	20381	PLEC	5339
446356	8q	143923488	143925516	2029	9	0.667	Exon(exon31of32)	14074	PLEC	5339
446356	9q	76175237	76175301	65	10	0.7	Exon(exon14of14)	-13338	PCSK5	5125
446356	9q	104598641	104599361	721	8	0.625	Promoter(<=1kb)	52	OR13C5	138799
446356	9q	122553263	122554071	809	7	0.429	Promoter(<=1kb)	93	OR1N2	138882
446356	9q	122749914	122750547	634	6	0.833	Promoter(<=1kb)	174	OR1L6	392390
446356	9q	131474844	131476113	1270	6	0.833	Promoter(<=1kb)	0	PRRC2B	84726
446356	9q	135484803	135487573	2771	9	0.333	Promoter(1-2kb)	1440	PPP1R26	9858
446356	9q	137107684	137108975	1292	6	0.5	Promoter(<=1kb)	312	MAN1B1	11253
446356	10q	46549378	46550744	1367	26	0.615	5'UTR	4786	GPRIN2	9721
446356	10q	49323169	49326817	3649	14	0.643	Exon(exon3of3)	23895	C10orf71	118461
446356	10q	122084988	122087840	2853	9	0.778	Exon(exon4of23)	-25190	TACC2	10579
446356	11p	244106	244197	92	8	0.5	Promoter(<=1kb)	-232	PSMD13	5719
446356	11p	1245466	1247378	1913	8	0.375	Promoter(2-3kb)	2298	MUC5B-AS1	112577518
446356	11p	5177811	5178478	668	7	0.143	Promoter(<=1kb)	186	OR52Z1	283110
446356	11p	5323362	5324256	895	7	0.429	Promoter(<=1kb)	41	OR51B2	79345
446356	11p	5389704	5390350	647	6	0.5	Promoter(<=1kb)	327	OR51M1	390059
446356	11p	5422212	5423123	912	11	0.636	Promoter(<=1kb)	101	OR51Q1	390061
446356	11p	5440761	5441472	712	6	0.833	Promoter(<=1kb)	42	OR51I1	390063
446356	11p	5581045	5581738	694	8	0.375	Promoter(<=1kb)	168	OR52B6	340980
446356	11p	5841302	5841883	582	9	0.333	Promoter(<=1kb)	14	OR52E6	390078
446356	11p	5884818	5885061	244	7	0.429	Promoter(<=1kb)	547	OR52E4	390081
446356	11p	11351961	11352736	776	8	0.125	Promoter(<=1kb)	514	CSNK2A3	283106
446356	11p	12293639	12294368	730	7	0.857	Exon(exon29of35)	6739	MICALCL	84953

446356	11p	18173280	18173901	622	6	0.333	Promoter(<=1kb)	443	MRGPRX4	117196
446356	11p	34916266	34916763	498	6	0.667	Promoter(<=1kb)	0	APIP	51074
446356	11q	58214757	58215722	966	8	0.25	Promoter(<=1kb)	12	OR1S1	219959
446356	11q	85724687	85725825	1139	6	0.5	Promoter(<=1kb)	0	SYTL2	54843
446356	11q	123906790	123907324	535	6	0.667	Promoter(<=1kb)	644	OR8D4	338662
446356	11q	124038366	124038988	623	8	0.875	Promoter(<=1kb)	13	OR10G7	390265
446356	11q	130914501	130915409	909	10	0.7	Promoter(1-2kb)	1035	SNX19	399979
446356	12p	4626568	4628549	1982	11	0.455	Exon(exon5of6)	14051	DYRK4	8798
446356	12p	6453119	6453670	552	6	0.667	Promoter(<=1kb)	633	TAPBPL	55080
446356	12q	48525773	48526223	451	6	0.5	Promoter(<=1kb)	141	OR8S1	341568
446356	13q	25096675	25097182	508	17	0.471	Promoter(<=1kb)	807	PABPC3	5042
446356	13q	102732474	102733933	1460	6	0.333	Exon(exon4of4)	25139	CCDC168	643677
446356	14q	19975713	19976448	736	9	0.444	Promoter(<=1kb)	269	OR4K15	81127
446356	14q	20060048	20060884	837	8	0.625	Promoter(<=1kb)	3	OR4L1	122742
446356	14q	20143999	20144604	606	8	0.5	Promoter(<=1kb)	263	OR4N5	390437
446356	14q	20640982	20641567	586	6	0.5	Promoter(<=1kb)	124	OR6S1	341799
446356	14q	70457532	70458540	1009	9	0.333	Exon(exon2of2)	5358	ADAM21	8747
446356	14q	104939262	104942618	3357	10	0.2	5'UTR	7102	PLD4	122618
446356	14q	104943622	104945444	1823	7	0.571	Exon(exon6of6)	9958	AHNAK2	113146
446356	14q	104947943	104953878	5936	35	0.486	Promoter(1-2kb)	1524	AHNAK2	113146
446356	15q	20534480	20535014	535	7	0.429	Exon(exon8of9)	6786	GOLGA6L6	727832
446356	15q	23439979	23442067	2089	12	0.583	5'UTR	5167	GOLGA6L2	283685
446356	15q	85579423	85581800	2378	15	0.533	Promoter(1-2kb)	-1110	AKAP13	11214
446356	15q	99129423	99132517	3095	6	0.333	Exon(exon4of5)	7225	TTC23	64927
446356	16p	1221890	1223490	1601	7	1	Promoter(1-2kb)	1285	TPSG1	25823
446356	16p	1486371	1488463	2093	8	0.75	Promoter(<=1kb)	4	PTX4	390667
446356	16p	4207130	4208004	875	6	0.5	Exon(exon2of7)	31739	SRL	6345
446356	16p	28495551	28497395	1845	6	0.333	Promoter(<=1kb)	0	CLN3	1201
446356	16q	74391401	74392004	604	10	0.7	Exon(exon7of7)	13523	NPIPB15	440348
446356	16q	88428339	88429600	1262	6	0.333	Exon(exon3of3)	-23680	ZFPM1	161882
446356	16q	88433131	88436097	2967	6	0.667	Exon(exon3of3)	-17183	ZFPM1	161882
446356	16q	89100686	89101050	365	8	0.625	Promoter(<=1kb)	24	ACSF3	197322
446356	16q	89226863	89228419	1557	9	0.556	Promoter(2-3kb)	2229	ZNF778	197320
446356	17p	744946	746966	2021	6	1	3'UTR	5072	GEMIN4	50628
446356	17p	10638198	10641099	2902	7	0.286	Exon(exon19of41)	-8169	MYH3	4621
446356	17p	21300581	21300978	398	12	0.75	3'UTR	9112	MAP2K3	5606
446356	17p	21415470	21416416	947	6	0.833	Exon(exon3of3)	10334	KCNJ12	3768
446356	17q	81645135	81645417	283	6	0.333	Promoter(2-3kb)	2722	TSPAN10	83882
446356	18p	11609728	11610383	656	10	0.9	Promoter(<=1kb)	132	SLC35G4	646000
446356	18p	11644378	11644629	252	6	1	Exon(exon1of1)	10312	MIR7153	102465690
446356	18q	58535186	58538030	2845	19	0.579	Promoter(<=1kb)	0	ALPK2	115701
446356	19p	1036457	1036914	458	6	0.5	Exon(exon6of7)	-3187	ABCA7	10347
446356	19p	4511338	4513547	2210	9	0.333	Exon(exon3of6)	4157	PLIN4	729359
446356	19p	5455600	5456439	840	8	0.625	Promoter(<=1kb)	183	ZNRF4	148066
446356	19p	8948231	8951868	3638	13	0.615	Exon(exon3of84)	29474	MUC16	94025
446356	19p	8959403	8962066	2664	7	0.429	Exon(exon3of84)	19276	MUC16	94025
446356	19p	8964274	8967127	2854	21	0.667	Exon(exon3of84)	14215	MUC16	94025
446356	19p	11978548	11978990	443	6	0.5	Promoter(1-2kb)	1726	ZNF763	284390
446356	19p	12075333	12077092	1760	7	0.286	Promoter(<=1kb)	6	ZNF788P	388507
446356	19p	15087213	15088040	828	9	0.333	Promoter(<=1kb)	233	OR1I1	126370
446356	19p	18264798	18267409	2612	12	0.583	5'UTR	7002	IQCN	80726
446356	19p	21971930	21974500	2571	8	0.75	Exon(exon4of4)	14408	ZNF208	7757
446356	19q	36996730	36997597	868	10	0.6	Exon(exon10of10)	5677	ZNF568	374900
446356	19q	43846955	43848536	1582	6	0.833	3'UTR	13450	ZNF283	284349
446356	19q	43913423	43914878	1456	8	0.5	Exon(exon10of10)	4861	ZNF45	7596
446356	19q	43996326	43997366	1041	6	0.5	Exon(exon5of5)	5419	LOC10192806:	101928063
446356	19q	44106512	44108078	1567	8	0.125	Exon(exon6of6)	-4103	ZNF225	7768
446356	19q	44386492	44387622	1131	7	0.714	Exon(exon4of4)	13953	ZNF285	26974
446356	19q	52437918	52439242	1325	7	0.429	Exon(exon4of4)	6504	ZNF534	147658
446356	19q	53164551	53166239	1689	6	0.167	Exon(exon4of4)	-5476	ZNF347	84671
446356	19q	55160006	55162286	2281	6	0.333	Promoter(<=1kb)	0	DNAAF3	352909
446356	19q	55911813	55913077	1265	7	0.714	Exon(exon5of12)	19234	NLRP13	126204
446356	19q	58368293	58368875	583	7	0.429	Exon(exon3of3)	-5445	ZNF497	162968
446356	20p	5922421	5923394	974	6	0.5	Exon(exon4of5)	6923	CHGB	1114
446356	20p	20052354	20052736	383	6	0.167	Promoter(<=1kb)	0	CFAP61	26074
446356	20q	63561666	63565531	3866	13	0.615	Promoter(<=1kb)	-61	HELZ2	85441
446356	22q	22352950	22353298	349	13	0.538	Exon(exon1of2)	30478	BMS1P20	96610
446356	22q	36191154	36191906	753	7	0.571	3'UTR	9971	APOL4	80832
446356	22q	39100331	39102033	1703	6	0.833	Promoter(<=1kb)	52	APOBEC3H	164668
446356	23p	3320126	3323750	3625	9	0.556	Exon(exon5of7)	22902	MXRA5	25878
446356	23p	35802148	35803010	863	7	0.571	5'UTR	3357	MAGEB16	139604
446356	23q	136874183	136874416	234	8	0.75	Promoter(<=1kb)	-201	RBMX	27316
448565	1p	18481403	18482217	815	6	0.667	Promoter(<=1kb)	421	KLHDC7A	127707
448565	1p	23874604	23875430	827	8	0.5	Exon(exon2of2)	-6310	FUCA1	2517
448565	1p	40067594	40067675	82	6	0	Promoter(<=1kb)	324	CAP1	10487
448565	1p	89186388	89186419	32	9	0.556	Promoter(<=1kb)	107	GBP4	115361
448565	1q	145872200	145873487	1288	6	0.667	Exon(exon8of12)	-12364	PIAS3	10401

448565	1q	152303673	152304920	1248	8	0.5	Exon(exon3of3)	-7656	FLG-AS1	339400
448565	1q	152306380	152313891	7512	29	0.552	Promoter(<=1kb)	0	FLG-AS1	339400
448565	1q	156669844	156670886	1043	7	1	Exon(exon4of4)	6521	NES	10763
448565	1q	201206099	201209738	3640	11	0.545	Promoter(1-2kb)	1017	IGFN1	91156
448565	1q	214640144	214643296	3153	13	0.538	Exon(exon12of20)	-4671	CENPF	1063
448565	1q	228315976	228318049	2074	8	0.625	Exon(exon50of81)	6492	OBSCN	84033
448565	1q	232805117	232806906	1790	7	0.429	Promoter(<=1kb)	225	MAP10	54627
448565	1q	247841312	247841582	271	6	0.833	Promoter(<=1kb)	314	OR11L1	391189
448565	1q	247949325	247949738	414	10	0.3	Promoter(<=1kb)	467	OR2L8	391190
448565	1q	248294677	248295458	782	6	0.667	Promoter(<=1kb)	142	OR2T12	127064
448565	2p	29071763	29073000	1238	6	0.667	Promoter(1-2kb)	1523	PCARE	388939
448565	2p	48580657	48582454	1798	7	0.571	Promoter(<=1kb)	0	STON1	11037
448565	2q	95944651	95945175	525	7	0.571	Promoter(<=1kb)	0	ANKRD36C	400986
448565	2q	132783061	132784972	1912	7	0.429	Promoter(1-2kb)	-1038	NCKAP5	344148
448565	2q	233713134	233713783	650	13	0.692	Promoter(<=1kb)	142	UGT1A5	54579
448565	2q	238130542	238131702	1161	7	0.714	Promoter(1-2kb)	1594	ESPNL	339768
448565	3p	31989532	31990905	1374	7	0.429	Exon(exon2of2)	7761	ZNF860	344787
448565	3p	75736880	75739239	2360	51	0.588	Promoter(<=1kb)	0	MIR4273	100422955
448565	4p	5988383	5989749	1367	8	0.5	Promoter(<=1kb)	0	C4orf50	389197
448565	4q	154489498	154491312	1815	8	0.5	Promoter(<=1kb)	22	DCHS2	54798
448565	4q	185458217	185460011	1795	8	0.625	Promoter(<=1kb)	0	CCDC110	256309
448565	4q	186619361	186621601	2241	8	0.375	Exon(exon10of27)	-9291	FAT1	2195
448565	4q	186706638	186709751	3114	7	0.714	Exon(exon2of27)	14082	FAT1	2195
448565	5q	79728956	79730716	1761	7	0.286	Exon(exon2of13)	-7426	CMYA5	202333
448565	5q	79731782	79734523	2742	13	0.308	Exon(exon2of13)	-3619	CMYA5	202333
448565	5q	83537326	83540746	3421	7	0.286	Promoter(1-2kb)	-1202	VCAN	1462
448565	5q	140841437	140843056	1620	6	0.667	Promoter(<=1kb)	250	PCDHA8	56140
448565	5q	141100771	141102728	1958	6	0.167	Promoter(<=1kb)	298	PCDHB3	56132
448565	5q	141174000	141175025	1026	7	0.714	Promoter(1-2kb)	1356	PCDHB7	56129
448565	5q	141187109	141189425	2317	6	0.5	Promoter(<=1kb)	0	PCDHB9	56127
448565	5q	141192663	141194472	1810	6	1	Promoter(<=1kb)	310	PCDHB10	56126
448565	5q	141955676	141957660	1985	6	0.667	Promoter(<=1kb)	-668	RNF14	9604
448565	5q	151565922	151568455	2534	10	0.8	Promoter(<=1kb)	489	FAT2	2196
448565	6p	1312843	1313745	903	6	0.5	Promoter(<=1kb)	745	FOXQ1	94234
448565	6p	46858771	46859502	732	8	0.5	Exon(exon17of21)	3802	ADGRF5	221395
448565	6q	64591274	64591961	688	10	0.5	Exon(exon26of43)	121374	EYS	346007
448565	6q	149888581	149889987	1407	6	0.667	Promoter(<=1kb)	-116	RAET1E-AS1	100652739
448565	6q	159233455	159234370	916	10	0.5	Exon(exon11of23)	15158	FNDC1	84624
448565	7p	6330446	6330944	499	6	1	Exon(exon2of2)	7749	FAM220A	84792
448565	7p	12369637	12370736	1100	6	0.667	3'UTR	-13307	VWDE	221806
448565	7q	100958721	100960873	2153	55	0.455	Promoter(<=1kb)	756	MUC3A	4584
448565	7q	100991195	100992398	1204	7	0.571	Exon(exon5of15)	-20656	MUC12	10071
448565	8p	10607245	10608432	1188	8	0.5	Exon(exon4of4)	46711	RP1L1	94137
448565	8p	10609614	10610662	1049	8	0.5	Exon(exon4of4)	44481	RP1L1	94137
448565	8p	13021128	13022030	903	7	0.143	Exon(exon5of5)	9115	TRMT9B	57604
448565	8q	141466455	141467514	1060	8	0.75	3'UTR	29245	MROH5	389690
448565	9q	76705179	76707804	2626	8	0.5	Promoter(<=1kb)	121	PCA3	50652
448565	9q	76709263	76710843	1581	8	0.5	Promoter(<=1kb)	0	PRUNE2	158471
448565	9q	122553263	122554071	809	7	0.429	Promoter(<=1kb)	93	OR1N2	138882
448565	10q	46549378	46550723	1346	26	0.654	Exon(exon3of3)	4807	GPRIN2	9721
448565	10q	49323169	49326817	3649	14	0.643	Exon(exon3of3)	23895	C10orf71	118461
448565	10q	89737450	89738561	1112	6	0	Exon(exon20of33)	13874	KIF20B	9585
448565	11p	244106	244197	92	8	0.5	Promoter(<=1kb)	-232	PSMD13	5719
448565	11p	5046754	5047432	679	7	0.571	Promoter(<=1kb)	228	OR52J3	119679
448565	11p	5177978	5178478	501	6	0.167	Promoter(<=1kb)	186	OR52Z1	283110
448565	11p	5323542	5324256	715	6	0.5	Promoter(<=1kb)	41	OR51B2	79345
448565	11p	5389704	5390350	647	6	0.5	Promoter(<=1kb)	327	OR51M1	390059
448565	11p	5402638	5403322	685	9	0.444	Promoter(<=1kb)	41	OR51J1	79470
448565	11p	5422212	5423123	912	11	0.636	Promoter(<=1kb)	101	OR51Q1	390061
448565	11p	5581045	5581738	694	8	0.375	Promoter(<=1kb)	168	OR52B6	340980
448565	11p	11352040	11352736	697	8	0.25	Promoter(<=1kb)	514	CSNK2A3	283106
448565	11p	12293639	12294368	730	6	0.833	Exon(exon29of35)	6739	MICALCL	84953
448565	11q	58214757	58215722	966	7	0.286	Promoter(<=1kb)	12	OR1S1	219959
448565	11q	82732630	82733184	555	6	0.833	Promoter(<=1kb)	680	FAM181B	220382
448565	11q	85724687	85725825	1139	6	0.5	Promoter(<=1kb)	0	SYTL2	54843
448565	11q	123906790	123907324	535	6	0.667	Promoter(<=1kb)	644	OR8D4	338662
448565	11q	124038366	124038988	623	8	0.875	Promoter(<=1kb)	13	OR10G7	390265
448565	11q	124264847	124265676	830	6	0.833	Promoter(<=1kb)	20	OR8G5	219865
448565	11q	124382526	124383285	760	6	0.5	Promoter(<=1kb)	58	OR8B2	26595
448565	12p	4626568	4628549	1982	10	0.5	Exon(exon5of6)	14051	DYRK4	8798
448565	12p	6453119	6453670	552	6	0.667	Promoter(<=1kb)	633	TAPBPL	55080
448565	12q	52571389	52572653	1265	6	0.5	Promoter(1-2kb)	1163	KRT74	121391
448565	13q	24434450	24435347	898	7	0.571	Exon(exon31of34)	19787	PARP4	143
448565	13q	24446725	24447185	461	6	0.333	Exon(exon26of34)	7949	PARP4	143
448565	13q	25096713	25098034	1322	18	0.5	Promoter(<=1kb)	845	PABPC3	5042
448565	13q	102732474	102733933	1460	6	0.333	Exon(exon4of4)	25139	CCDC168	643677
448565	14q	19975713	19976448	736	9	0.444	Promoter(<=1kb)	269	OR4K15	81127

448565	14q	20060048	20060884	837	8	0.625	Promoter(<=1kb)	3	OR4L1	122742
448565	14q	20143999	20144604	606	8	0.5	Promoter(<=1kb)	263	OR4N5	390437
448565	14q	20640750	20641567	818	7	0.571	Promoter(<=1kb)	124	OR6S1	341799
448565	14q	21634177	21634589	413	6	0.833	Promoter(<=1kb)	351	OR10G2	26534
448565	14q	70457532	70458238	707	9	0.333	Exon(exon2of2)	5358	ADAM21	8747
448565	14q	94587512	94587839	328	6	0.5	Exon(exon2of2)	-4219	SERPINA3	12
448565	14q	104175275	104177810	2536	7	0.429	Exon(exon12of15)	36235	KIF26A	26153
448565	14q	104947943	104950338	2396	7	0.429	Exon(exon6of6)	5064	AHNAK2	113146
448565	15q	23439979	23442067	2089	12	0.583	5'UTR	5167	GOLGA6L2	283685
448565	15q	85579423	85582016	2594	16	0.562	Promoter(<=1kb)	-894	AKAP13	11214
448565	15q	88857108	88859365	2258	6	0	Exon(exon12of18)	9865	ACAN	176
448565	16p	669592	672548	2957	7	0.571	Promoter(<=1kb)	0	RHOT2	89941
448565	16p	1256354	1256985	632	11	0.636	Promoter(<=1kb)	285	TPSD1	23430
448565	16q	74391650	74391889	240	7	0.857	Exon(exon7of7)	13772	NPIPB15	440348
448565	16q	89226863	89228289	1427	7	0.571	Promoter(2-3kb)	2229	ZNF778	197320
448565	17p	744946	746966	2021	6	1	3'UTR	5072	GEMIN4	50628
448565	17p	2299649	2300159	511	6	0.167	Exon(exon2of19)	-3224	SRR	63826
448565	17p	21300581	21300978	398	11	0.727	3'UTR	9112	MAP2K3	5606
448565	17p	21415470	21416431	962	8	0.75	Exon(exon3of3)	10334	KCNJ12	3768
448565	17q	76291123	76294016	2894	11	0.455	Promoter(<=1kb)	0	QRICH2	84074
448565	17q	81645135	81645417	283	6	0.333	Promoter(2-3kb)	2722	TSPAN10	83882
448565	18q	58535186	58537515	2330	10	0.4	Promoter(<=1kb)	0	ALPK2	115701
448565	19p	4510548	4513547	3000	17	0.471	Exon(exon3of6)	4157	PLIN4	729359
448565	19p	5455600	5456439	840	6	0.5	Promoter(<=1kb)	183	ZNRF4	148066
448565	19p	8937644	8939234	1591	6	0.667	Exon(exon5of84)	-41554	MUC16	94025
448565	19p	8946313	8951868	5556	19	0.632	Exon(exon3of84)	29474	MUC16	94025
448565	19p	8959116	8962299	3184	12	0.667	Exon(exon3of84)	19043	MUC16	94025
448565	19p	12430400	12432437	2038	8	0.5	3'UTR	8584	ZNF443	10224
448565	19p	17281820	17284246	2427	8	0.5	Promoter(<=1kb)	0	ANKLE1	126549
448565	19p	18264753	18267409	2657	8	0.5	5'UTR	7002	IQCN	80726
448565	19p	21971930	21974500	2571	8	0.75	Exon(exon4of4)	14408	ZNF208	7757
448565	19p	22756205	22759523	3319	10	0.7	3'UTR	10459	ZNF99	7652
448565	19p	23743906	23745300	1395	6	0.333	Exon(exon4of4)	13537	ZNF681	148213
448565	19q	39886005	39886439	435	7	0.571	Promoter(<=1kb)	853	FCGBP	8857
448565	19q	43846955	43848536	1582	6	0.833	3'UTR	13450	ZNF283	284349
448565	19q	43913423	43914878	1456	8	0.5	Exon(exon10of10)	4861	ZNF45	7596
448565	19q	44106512	44108078	1567	9	0.222	Exon(exon6of6)	-4103	ZNF225	7768
448565	19q	52437918	52439242	1325	7	0.429	Exon(exon4of4)	6504	ZNF534	147658
448565	19q	52840798	52841923	1126	8	0.375	3'UTR	7602	ZNF468	90333
448565	19q	55481625	55483456	1832	7	0.429	Promoter(1-2kb)	-1732	NAT14	57106
448565	19q	55517821	55518176	356	7	1	Exon(exon14of14)	18127	SBK2	646643
448565	19q	55911888	55913077	1190	6	0.667	Exon(exon5of12)	19234	NLRP13	126204
448565	19q	57444351	57445460	1110	6	0.667	Exon(exon3of3)	9004	ZNF749	388567
448565	19q	58368293	58368875	583	7	0.429	Exon(exon3of3)	-5445	ZNF497	162968
448565	20p	20052354	20052736	383	6	0	Promoter(<=1kb)	0	CFAP61	26074
448565	20q	63349752	63350772	1021	6	0.5	3'UTR	3794	CHRNA4	1137
448565	20q	63561666	63565531	3866	11	0.636	Promoter(<=1kb)	-61	HELZ2	85441
448565	21q	44637474	44638041	568	6	0.333	Promoter(<=1kb)	118	KRTAP10-10	353333
448565	22q	36191154	36191906	753	6	0.667	3'UTR	9971	APOL4	80832
448565	23p	3320126	3323750	3625	9	0.556	Exon(exon5of7)	22902	MXRA5	25878
448565	23p	8170039	8170141	103	6	0.5	Promoter(1-2kb)	1126	VCX2	51480
448565	23p	35802148	35803010	863	7	0.571	5'UTR	3357	MAGEB16	139604
448565	23q	141906066	141906494	429	6	0.5	Promoter(1-2kb)	1264	MAGEC1	9947
449697	1p	12847526	12847780	255	7	0.429	Promoter(<=1kb)	945	HNRNPCL1	343069
449697	1p	12859036	12860079	1044	6	0.333	Promoter(1-2kb)	1950	PRAMEF2	65122
449697	1p	13370686	13370957	272	7	0.429	Promoter(<=1kb)	943	PRAMEF19	645414
449697	1p	16058491	16060000	1510	10	0.9	Exon(exon5of7)	6168	CLCNKB	1188
449697	1p	18481403	18482217	815	7	0.714	Promoter(<=1kb)	421	KLHDC7A	127707
449697	1p	23874604	23875430	827	8	0.5	Exon(exon2of2)	-6310	FUCA1	2517
449697	1p	40067594	40067675	82	6	0	Promoter(<=1kb)	324	CAP1	10487
449697	1q	152108539	152109592	1054	7	0.429	Exon(exon2of2)	4488	TCHH	7062
449697	1q	152154896	152156768	1873	6	0.333	Promoter(2-3kb)	2460	RPTN	126638
449697	1q	152213286	152213566	281	9	0.444	Exon(exon3of3)	10627	HRNR	388697
449697	1q	152219233	152221375	2143	15	0.667	Promoter(2-3kb)	2818	HRNR	388697
449697	1q	152304413	152309942	5530	11	0.455	Promoter(2-3kb)	-2634	FLG-AS1	339400
449697	1q	152311007	152313526	2520	7	0.571	Promoter(<=1kb)	0	FLG-AS1	339400
449697	1q	156669844	156671745	1902	7	1	Exon(exon4of4)	5662	NES	10763
449697	1q	158765805	158766655	851	6	0.5	Promoter(<=1kb)	47	OR6N1	128372
449697	1q	197101312	197101771	460	6	0.5	Exon(exon18of28)	33373	ASPM	259266
449697	1q	201206099	201209342	3244	10	0.6	Promoter(1-2kb)	1017	IGFN1	91156
449697	1q	223393517	223394466	950	7	0.714	Promoter(<=1kb)	102	CCDC185	164127
449697	1q	228315976	228318049	2074	8	0.625	Exon(exon50of81)	6492	OBSCN	84033
449697	1q	232805117	232807473	2357	8	0.375	Promoter(<=1kb)	225	MAP10	54627
449697	1q	236553647	236555893	2247	6	0.333	Exon(exon39of45)	10351	LGALS8	3964
449697	1q	247841312	247841582	271	6	0.833	Promoter(<=1kb)	314	OR11L1	391189
449697	1q	247949325	247949738	414	10	0.3	Promoter(<=1kb)	467	OR2L8	391190
449697	1q	248294677	248295458	782	7	0.571	Promoter(<=1kb)	142	OR2T12	127064

449697	2q	167243228	167244632	1405	6	0.5	Exon(exon9of11)	-4050	XIRP2	129446
449697	2q	167246794	167248478	1685	8	0.625	Promoter(<=1kb)	-204	XIRP2	129446
449697	2q	184936178	184937636	1459	8	0.375	Exon(exon4of4)	69813	ZNF804A	91752
449697	2q	233713134	233713664	531	8	0.75	Promoter(<=1kb)	142	UGT1A5	54579
449697	2q	233840612	233842185	1574	6	0.667	Promoter(<=1kb)	0	HJURP	55355
449697	2q	238130416	238131546	1131	6	0.333	Promoter(1-2kb)	1468	ESPNL	339768
449697	2q	240041845	240042154	310	6	0.167	Downstream(2-3kb)	3918	OR6B3	150681
449697	3p	31989532	31990905	1374	6	0.333	Exon(exon2of2)	7761	ZNF860	344787
449697	3p	75736880	75739243	2364	62	0.613	Promoter(<=1kb)	0	MIR4273	100422955
449697	4p	5988383	5989749	1367	7	0.571	Promoter(<=1kb)	0	C4orf50	389197
449697	4p	6300792	6302360	1569	6	0.833	Exon(exon8of8)	6021	WFS1	7466
449697	4p	8227004	8228508	1505	8	0.125	Promoter(<=1kb)	-24	SH3TC1	54436
449697	4p	9175308	9175954	647	6	0.667	Exon(exon7of7)	4899	FAM90A26	100287045
449697	4q	186706638	186708616	1979	6	0.667	Exon(exon2of27)	15217	FAT1	2195
449697	5q	79728956	79730716	1761	6	0.333	Exon(exon2of13)	-7426	CMYA5	202333
449697	5q	79731782	79734523	2742	13	0.308	Exon(exon2of13)	-3619	CMYA5	202333
449697	5q	140807352	140807737	386	6	0.833	Promoter(<=1kb)	271	PCDHA4	56144
449697	5q	140848579	140850786	2208	6	0.5	Promoter(<=1kb)	807	PCDHA9	9752
449697	5q	141174000	141175025	1026	6	0.833	Promoter(1-2kb)	1356	PCDHB7	56129
449697	5q	141183201	141184688	1488	7	0.857	Promoter(1-2kb)	1802	PCDHB16	57717
449697	5q	141187690	141189425	1736	6	0.5	Promoter(<=1kb)	529	PCDHB9	56127
449697	6p	1312843	1313745	903	6	0.5	Promoter(<=1kb)	745	FOXQ1	94234
449697	6p	46858771	46859502	732	8	0.5	Exon(exon17of21)	3802	ADGRF5	221395
449697	6q	111373870	111375649	1780	6	0.667	3'UTR	-6512	REV3L	5980
449697	6q	149888581	149889987	1407	6	0.667	Promoter(<=1kb)	-116	RAET1E-AS1	100652739
449697	6q	159233455	159234370	916	10	0.5	Exon(exon11of23)	15158	FNDC1	84624
449697	7p	12369637	12370736	1100	6	0.667	3'UTR	-13307	VWDE	221806
449697	7q	100958721	100960873	2153	58	0.466	Promoter(<=1kb)	756	MUC3A	4584
449697	7q	100991195	100992983	1789	9	0.667	Exon(exon5of15)	-20071	MUC12	10071
449697	7q	100995575	100995785	211	7	0.714	Exon(exon5of15)	-17269	MUC12	10071
449697	7q	101034361	101040583	6223	38	0.5	Exon(exon3of12)	-3128	MUC17	140453
449697	7q	149818015	149819792	1778	6	0.667	Promoter(2-3kb)	-2352	SSPO	23145
449697	8p	10607375	10612307	4933	16	0.625	Exon(exon4of4)	42836	RP1L1	94137
449697	8p	12132686	12133940	1255	6	0.833	Promoter(<=1kb)	498	USP17L7	392197
449697	8p	12137448	12138641	1194	6	0.833	Promoter(<=1kb)	436	USP17L2	377630
449697	8p	13021128	13022030	903	7	0.143	Exon(exon5of5)	9115	TRMT9B	57604
449697	8q	123651693	123652634	942	6	0.5	Promoter(<=1kb)	316	KLHL38	340359
449697	8q	143916360	143919209	2850	7	0.143	Exon(exon32of32)	20381	PLEC	5339
449697	9p	39078723	39078846	124	6	0.667	Exon(exon22of24)	7302	CNTNAP3	79937
449697	9q	76175237	76175296	60	10	0.8	Exon(exon14of14)	-13343	PCSK5	5125
449697	9q	76703555	76707804	4250	13	0.385	Promoter(<=1kb)	0	PCA3	50652
449697	9q	76709263	76710843	1581	8	0.5	Promoter(<=1kb)	0	PRUNE2	158471
449697	9q	104504315	104505071	757	6	0.333	Promoter(<=1kb)	52	OR13F1	138805
449697	9q	104598641	104599361	721	8	0.625	Promoter(<=1kb)	52	OR13C5	138799
449697	9q	122553278	122554071	794	7	0.429	Promoter(<=1kb)	108	OR1N2	138882
449697	9q	122749914	122750547	634	6	0.833	Promoter(<=1kb)	174	OR1L6	392390
449697	9q	131474844	131476113	1270	6	0.833	Promoter(<=1kb)	0	PRRC2B	84726
449697	9q	133255635	133256264	630	8	1	3'UTR	18950	ABO	28
449697	9q	135484803	135487213	2411	8	0.25	Promoter(1-2kb)	1440	PPP1R26	9858
449697	10q	46549378	46550723	1346	25	0.64	Exon(exon3of3)	4807	GPRIN2	9721
449697	10q	49322575	49325572	2998	9	0.556	Exon(exon3of3)	23301	C10orf71	118461
449697	11p	244106	244197	92	8	0.5	Promoter(<=1kb)	-232	PSMD13	5719
449697	11p	1245466	1247378	1913	8	0.5	Promoter(2-3kb)	2298	MUC5B-AS1	112577518
449697	11p	1835943	1837348	1406	6	0.667	Promoter(<=1kb)	0	SYT8	90019
449697	11p	5177978	5178478	501	6	0.167	Promoter(<=1kb)	186	OR52Z1	283110
449697	11p	5323451	5324256	806	6	0.5	Promoter(<=1kb)	41	OR51B2	79345
449697	11p	5389704	5390350	647	6	0.5	Promoter(<=1kb)	327	OR51M1	390059
449697	11p	5402638	5403322	685	9	0.444	Promoter(<=1kb)	41	OR51J1	79470
449697	11p	5515079	5515931	853	7	0.429	Promoter(<=1kb)	768	UBQLNL	143630
449697	11p	5581045	5581738	694	8	0.375	Promoter(<=1kb)	168	OR52B6	340980
449697	11p	5841302	5841883	582	9	0.333	Promoter(<=1kb)	14	OR52E6	390078
449697	11p	5884818	5885061	244	7	0.429	Promoter(<=1kb)	547	OR52E4	390081
449697	11p	11351961	11352736	776	9	0.222	Promoter(<=1kb)	514	CSNK2A3	283106
449697	11p	12293788	12294842	1055	6	0.667	Exon(exon29of35)	6888	MICALCL	84953
449697	11p	18173280	18173901	622	6	0.333	Promoter(<=1kb)	443	MRGPRX4	117196
449697	11p	34916266	34916763	498	6	0.667	Promoter(<=1kb)	0	APIP	51074
449697	11q	55572176	55572903	728	6	0.667	Promoter(<=1kb)	48	OR4C16	219428
449697	11q	58214757	58215722	966	9	0.222	Promoter(<=1kb)	12	OR1S1	219959
449697	11q	58402523	58403265	743	8	0.5	Promoter(<=1kb)	144	OR5B3	441608
449697	11q	82732630	82733184	555	6	0.833	Promoter(<=1kb)	680	FAM181B	220382
449697	11q	85724687	85725825	1139	6	0.5	Promoter(<=1kb)	0	SYTL2	54843
449697	11q	124015600	124016477	878	7	0.143	Promoter(<=1kb)	25	OR10G4	390264
449697	11q	124382526	124383285	760	7	0.571	Promoter(<=1kb)	58	OR8B2	26595
449697	12p	4626571	4628549	1979	9	0.444	Exon(exon5of6)	14054	DYRK4	8798
449697	12p	31981130	31985476	4347	11	0.273	Promoter(1-2kb)	-1690	RESF1	55196
449697	12q	52316096	52317765	1670	6	0.667	Exon(exon4of9)	3633	KRT83	3889
449697	13q	25096713	25097154	442	16	0.5	Promoter(<=1kb)	845	PABPC3	5042

449697	13q	102732474	102734620	2147	7	0.286	Exon(exon4of4)	24452	CCDC168	643677
449697	14q	19935750	19936577	828	6	0.833	Promoter(<=1kb)	142	OR4K1	79544
449697	14q	20060048	20060884	837	8	0.625	Promoter(<=1kb)	3	OR4L1	122742
449697	14q	21634137	21634589	453	9	0.556	Promoter(<=1kb)	351	OR10G2	26534
449697	14q	22633879	22634450	572	9	0.333	Exon(exon2of2)	32212	ABHD4	63874
449697	14q	70457520	70458540	1021	12	0.333	Exon(exon2of2)	5346	ADAM21	8747
449697	14q	94587512	94587839	328	6	0.5	Exon(exon2of2)	-4219	SERPINA3	12
449697	14q	104947943	104950338	2396	6	0.333	Exon(exon6of6)	5064	AHNAK2	113146
449697	15q	23439979	23442067	2089	11	0.636	5'UTR	5167	GOLGA6L2	283685
449697	15q	40621642	40623696	2055	6	0.333	Promoter(<=1kb)	0	KNL1	57082
449697	15q	78765975	78766581	607	8	0.625	Promoter(<=1kb)	-862	ADAMTS7	11173
449697	15q	79456261	79457965	1705	6	0.5	Promoter(<=1kb)	165	MINAR1	23251
449697	15q	85579423	85582073	2651	15	0.6	Promoter(<=1kb)	-837	AKAP13	11214
449697	15q	88857108	88859365	2258	6	0	Exon(exon12of18)	9865	ACAN	176
449697	15q	99129423	99132517	3095	7	0.429	Exon(exon4of5)	7225	TTC23	64927
449697	16p	1256345	1256980	636	6	0.833	Promoter(<=1kb)	276	TPSD1	23430
449697	16q	74391549	74392004	456	10	0.7	Exon(exon7of7)	13671	NPIPB15	440348
449697	16q	89226863	89228289	1427	7	0.571	Promoter(2-3kb)	2229	ZNF778	197320
449697	17p	10638198	10641099	2902	7	0.286	Exon(exon19of41)	-8169	MYH3	4621
449697	17p	21300581	21300954	374	8	0.75	3'UTR	9112	MAP2K3	5606
449697	17p	21415470	21416370	901	6	0.833	Exon(exon3of3)	10334	KCNJ12	3768
449697	17q	53823368	53824891	1524	6	0.833	Promoter(<=1kb)	441	KIF2B	84643
449697	17q	76293419	76294016	598	6	0.5	Promoter(2-3kb)	-2167	QRICH2	84074
449697	17q	81645135	81645607	473	7	0.286	Promoter(2-3kb)	2722	TSPAN10	83882
449697	18p	11609646	11610164	519	9	0.778	Promoter(<=1kb)	50	SLC35G4	646000
449697	18q	58535186	58537515	2330	10	0.4	Promoter(<=1kb)	0	ALPK2	115701
449697	19p	4510548	4513547	3000	21	0.524	Exon(exon3of6)	4157	PLIN4	729359
449697	19p	5455600	5456439	840	8	0.625	Promoter(<=1kb)	183	ZNRF4	148066
449697	19p	8333830	8334965	1136	7	0.714	Promoter(<=1kb)	0	KANK3	256949
449697	19p	8937644	8939234	1591	6	0.833	Exon(exon5of84)	-41554	MUC16	94025
449697	19p	8946306	8951868	5563	18	0.667	Exon(exon3of84)	29474	MUC16	94025
449697	19p	8959116	8962299	3184	12	0.583	Exon(exon3of84)	19043	MUC16	94025
449697	19p	8963397	8967127	3731	21	0.667	Exon(exon3of84)	14215	MUC16	94025
449697	19p	8972751	8978096	5346	13	0.462	Exon(exon1of84)	3246	MUC16	94025
449697	19p	18264753	18267409	2657	14	0.643	5'UTR	7002	IQCN	80726
449697	19p	21971930	21974500	2571	10	0.7	Exon(exon4of4)	14408	ZNF208	7757
449697	19p	23743906	23745300	1395	6	0.333	Exon(exon4of4)	13537	ZNF681	148213
449697	19q	36996730	36997597	868	10	0.6	Exon(exon10of10)	5677	ZNF568	374900
449697	19q	39886005	39886439	435	6	0.333	Promoter(<=1kb)	853	FCGBP	8857
449697	19q	40880231	40880622	392	6	0.333	Promoter(<=1kb)	-141	CYP2A7	1549
449697	19q	43913423	43914878	1456	7	0.429	Exon(exon10of10)	4861	ZNF45	7596
449697	19q	43996326	43997366	1041	6	0.5	Exon(exon5of5)	5419	LOC10192806:	101928063
449697	19q	44327836	44329698	1863	6	0.5	Exon(exon4of4)	-22790	ZNF235	9310
449697	19q	48873325	48875925	2601	12	0.583	Promoter(<=1kb)	904	PPP1R15A	23645
449697	19q	51872787	51873699	913	10	0.5	Promoter(<=1kb)	-940	ZNF577	84765
449697	19q	52437918	52439242	1325	7	0.429	Exon(exon4of4)	6504	ZNF534	147658
449697	19q	55911888	55913077	1190	6	0.667	Exon(exon5of12)	19234	NLRP13	126204
449697	19q	58368293	58368875	583	7	0.429	Exon(exon3of3)	-5445	ZNF497	162968
449697	20p	1635288	1636423	1136	6	0.333	Promoter(1-2kb)	1662	SIRPG-AS1	101929010
449697	20p	5922421	5923394	974	6	0.5	Exon(exon4of5)	6923	CHGB	1114
449697	20q	63349752	63350772	1021	6	0.5	3'UTR	3794	CHRNA4	1137
449697	20q	63561666	63565531	3866	11	0.636	Promoter(<=1kb)	-61	HELZ2	85441
449697	21q	26843740	26844859	1120	6	0.667	Promoter(<=1kb)	0	ADAMTS1	9510
449697	21q	44600627	44601692	1066	9	0.778	Promoter(<=1kb)	30	KRTAP10-7	386675
449697	21q	44637474	44638143	670	7	0.429	Promoter(<=1kb)	118	KRTAP10-10	353333
449697	22q	22352950	22353380	431	16	0.5	Exon(exon1of2)	30478	BMS1P20	96610
449697	22q	36191154	36191906	753	6	0.667	3'UTR	9971	APOL4	80832
449697	22q	50503556	50505734	2179	8	0.875	Promoter(<=1kb)	0	LMF2	91289
449697	23p	35802148	35803010	863	7	0.571	5'UTR	3357	MAGEB16	139604
449697	23q	136874183	136874416	234	8	0.75	Promoter(<=1kb)	-201	RBMX	27316
449697	23q	141906066	141906494	429	6	0.5	Promoter(1-2kb)	1264	MAGEC1	9947
450458	1p	11766028	11768307	2280	6	0.833	Promoter(<=1kb)	0	C1orf167	284498
450458	1p	12847526	12847995	470	8	0.5	Promoter(<=1kb)	730	HNRNPCL1	343069
450458	1p	12859036	12860079	1044	6	0.333	Promoter(1-2kb)	1950	PRAMEF2	65122
450458	1p	12893249	12893472	224	6	0.5	Exon(exon4of4)	4798	PRAMEF10	343071
450458	1p	13369166	13369564	399	7	0.714	Promoter(2-3kb)	2336	PRAMEF19	645414
450458	1p	13370686	13371119	434	7	0.429	Promoter(<=1kb)	781	PRAMEF19	645414
450458	1p	16058491	16060000	1510	13	0.846	Exon(exon5of7)	6168	CLCNKB	1188
450458	1p	18481042	18482217	1176	12	0.75	Promoter(<=1kb)	60	KLHDC7A	127707
450458	1p	23874604	23875430	827	8	0.5	Exon(exon2of2)	-6310	FUCA1	2517
450458	1p	40067594	40067675	82	6	0	Promoter(<=1kb)	324	CAP1	10487
450458	1q	152213274	152213316	43	7	0.571	Exon(exon3of3)	10877	HRNR	388697
450458	1q	152218469	152221375	2907	18	0.722	Promoter(2-3kb)	2818	HRNR	388697
450458	1q	152303673	152313891	10219	34	0.618	Promoter(<=1kb)	0	FLG-AS1	339400
450458	1q	158765805	158766655	851	6	0.5	Promoter(<=1kb)	47	OR6N1	128372
450458	1q	214640144	214642954	2811	12	0.5	Exon(exon12of20)	-5013	CENPF	1063
450458	1q	214644872	214647181	2310	10	0.4	Promoter(<=1kb)	-786	CENPF	1063

450458	1q	223393517	223394466	950	6	0.667	Promoter(<=1kb)	102	CCDC185	164127
450458	1q	228315976	228318038	2063	9	0.667	Exon(exon50of81)	6492	OBSCN	84033
450458	1q	247841312	247841582	271	6	0.833	Promoter(<=1kb)	314	OR11L1	391189
450458	2p	48580657	48582454	1798	7	0.571	Promoter(<=1kb)	0	STON1	11037
450458	2q	178739433	178741811	2379	6	0.5	Exon(exon45of191)	26014	TTN	7273
450458	2q	184936178	184938131	1954	6	0.333	Exon(exon4of4)	69813	ZNF804A	91752
450458	2q	185789865	185794632	4768	11	0.727	Promoter(<=1kb)	0	FSIP2	401024
450458	2q	185805377	185808891	3515	7	0.429	Promoter(<=1kb)	0	FSIP2	401024
450458	2q	217847583	217848559	977	6	0.833	Exon(exon19of33)	-5423	TNS1	7145
450458	2q	233681970	233682693	724	6	0.667	Promoter(<=1kb)	0	UGT1A7	54577
450458	2q	233713134	233713664	531	8	0.75	Promoter(<=1kb)	142	UGT1A5	54579
450458	2q	237762685	237764060	1376	10	0.5	Exon(exon8of8)	-4137	LRRFIP1	9208
450458	2q	238130271	238131546	1276	7	0.286	Promoter(1-2kb)	1323	ESPNL	339768
450458	3p	75736880	75739243	2364	65	0.631	Promoter(<=1kb)	0	MIR4273	100422955
450458	3q	98264413	98265098	686	7	0.571	Promoter(<=1kb)	128	OR5H6	79295
450458	4p	5988383	5989749	1367	8	0.625	Promoter(<=1kb)	0	C4orf50	389197
450458	4p	6300792	6302360	1569	6	0.833	Exon(exon8of8)	6021	WFS1	7466
450458	4p	8227004	8228508	1505	8	0.125	Promoter(<=1kb)	-24	SH3TC1	54436
450458	4q	121036404	121037542	1139	6	0.333	Promoter(1-2kb)	1442	NDNF	79625
450458	4q	185458217	185460011	1795	8	0.625	Promoter(<=1kb)	0	CCDC110	256309
450458	4q	186619481	186621601	2121	8	0.375	Exon(exon10of27)	-9411	FAT1	2195
450458	5q	54518000	54519665	1666	6	0.333	Promoter(<=1kb)	135	SNX18	112574
450458	5q	79728956	79730716	1761	7	0.286	Exon(exon2of13)	-7426	CMYA5	202333
450458	5q	79731782	79734523	2742	13	0.308	Exon(exon2of13)	-3619	CMYA5	202333
450458	5q	83537326	83539905	2580	6	0.333	Promoter(1-2kb)	1712	VCAN	1462
450458	5q	140807352	140807737	386	7	0.857	Promoter(<=1kb)	271	PCDHA4	56144
450458	5q	141174000	141175025	1026	6	0.833	Promoter(1-2kb)	1356	PCDHB7	56129
450458	5q	141187690	141189425	1736	6	0.5	Promoter(<=1kb)	529	PCDHB9	56127
450458	5q	141192583	141194472	1890	7	1	Promoter(<=1kb)	230	PCDHB10	56126
450458	5q	141955356	141957660	2305	6	0.667	Promoter(<=1kb)	-668	RNF14	9604
450458	5q	151521550	151522069	520	6	0.667	Promoter(<=1kb)	79	MIR6499	102465246
450458	5q	151565922	151568158	2237	10	0.8	Promoter(<=1kb)	786	FAT2	2196
450458	6p	46858771	46859389	619	6	0.5	Exon(exon17of21)	3915	ADGRF5	221395
450458	6q	64591274	64591961	688	10	0.5	Exon(exon26of43)	121374	EYS	346007
450458	6q	159231899	159234370	2472	10	0.5	Exon(exon11of23)	13602	FNDC1	84624
450458	6q	168307821	168309048	1228	7	0.429	5'UTR	10662	DACT2	168002
450458	7p	12369637	12370736	1100	6	0.667	3'UTR	-13307	VWDE	221806
450458	7p	45082725	45084866	2142	8	0.5	Promoter(1-2kb)	-1325	NACAD	23148
450458	7q	100958721	100960873	2153	56	0.464	Promoter(<=1kb)	756	MUC3A	4584
450458	7q	100993912	100995785	1874	11	0.727	Exon(exon5of15)	-17269	MUC12	10071
450458	7q	101034361	101041369	7009	41	0.537	Promoter(2-3kb)	-2342	MUC17	140453
450458	8p	10607245	10608261	1017	6	0.667	Exon(exon4of4)	46882	RP1L1	94137
450458	8p	10609650	10612307	2658	10	0.7	Exon(exon4of4)	42836	RP1L1	94137
450458	8p	12132686	12133940	1255	6	0.833	Promoter(<=1kb)	498	USP17L7	392197
450458	8p	12137448	12138752	1305	7	0.857	Promoter(<=1kb)	325	USP17L2	377630
450458	8p	13021128	13022030	903	8	0.25	Exon(exon5of5)	9115	TRMT9B	57604
450458	9p	39078723	39078846	124	6	0.667	Exon(exon22of24)	7302	CNTNAP3	79937
450458	9q	76175237	76175296	60	9	0.778	Exon(exon14of14)	-13343	PCSK5	5125
450458	9q	76705179	76707804	2626	8	0.5	Promoter(<=1kb)	121	PCA3	50652
450458	9q	76709263	76710846	1584	9	0.556	Promoter(<=1kb)	0	PRUNE2	158471
450458	9q	87886533	87888536	2004	7	0.571	Exon(exon4of4)	3656	SPATA31E1	286234
450458	9q	122553263	122554071	809	8	0.5	Promoter(<=1kb)	93	OR1N2	138882
450458	9q	122628595	122629130	536	6	0.333	Promoter(<=1kb)	443	OR1B1	347169
450458	9q	122749914	122750547	634	6	0.833	Promoter(<=1kb)	174	OR1L6	392390
450458	9q	133255635	133256208	574	10	0.9	3'UTR	19006	ABO	28
450458	10q	46549378	46550723	1346	25	0.64	Exon(exon3of3)	4807	GPRIN2	9721
450458	10q	49323169	49326817	3649	13	0.615	Exon(exon3of3)	23895	C10orf71	118461
450458	10q	128102802	128104752	1951	10	0.5	Promoter(<=1kb)	0	MKI67	4288
450458	11p	244106	244197	92	8	0.5	Promoter(<=1kb)	-232	PSMD13	5719
450458	11p	1246095	1247378	1284	6	0.333	Promoter(2-3kb)	2298	MUC5B-AS1	112577518
450458	11p	5177978	5178478	501	6	0.167	Promoter(<=1kb)	186	OR52Z1	283110
450458	11p	5323362	5324256	895	7	0.429	Promoter(<=1kb)	41	OR51B2	79345
450458	11p	5389704	5390350	647	7	0.429	Promoter(<=1kb)	327	OR51M1	390059
450458	11p	5422212	5423123	912	10	0.7	Promoter(<=1kb)	101	OR51Q1	390061
450458	11p	5581045	5581738	694	8	0.375	Promoter(<=1kb)	168	OR52B6	340980
450458	11p	5841302	5841883	582	9	0.333	Promoter(<=1kb)	14	OR52E6	390078
450458	11p	5884818	5885061	244	7	0.429	Promoter(<=1kb)	547	OR52E4	390081
450458	11p	11351961	11352736	776	9	0.222	Promoter(<=1kb)	514	CSNK2A3	283106
450458	11p	12293639	12294368	730	6	0.833	Exon(exon29of35)	6739	MICALCL	84953
450458	11q	58402523	58403265	743	8	0.5	Promoter(<=1kb)	144	OR5B3	441608
450458	11q	64116513	64118232	1720	7	0.714	Exon(exon2of2)	8702	MACROD1	28992
450458	11q	85724687	85725825	1139	6	0.5	Promoter(<=1kb)	0	SYTL2	54843
450458	11q	123906790	123907324	535	6	0.667	Promoter(<=1kb)	644	OR8D4	338662
450458	11q	123943088	123943824	737	8	0.625	Promoter(<=1kb)	49	OR6T1	219874
450458	11q	124015601	124016477	877	8	0.25	Promoter(<=1kb)	26	OR10G4	390264
450458	11q	124023038	124023849	812	9	0.444	Promoter(<=1kb)	25	OR10G9	219870
450458	11q	124382526	124383285	760	8	0.625	Promoter(<=1kb)	58	OR8B2	26595

450458	12p	4626571	4628549	1979	9	0.556	Exon(exon5of6)	14054	DYRK4	8798
450458	13q	25096665	25097182	518	19	0.474	Promoter(<=1kb)	797	PABPC3	5042
450458	13q	102732474	102733933	1460	6	0.333	Exon(exon4of4)	25139	CCDC168	643677
450458	13q	102743469	102745587	2119	6	0.5	Exon(exon1of2)	13485	CCDC168	643677
450458	14q	22633879	22634450	572	8	0.375	Exon(exon2of2)	32212	ABHD4	63874
450458	14q	70457520	70458948	1429	13	0.385	Exon(exon2of2)	5346	ADAM21	8747
450458	14q	104939262	104953878	14617	73	0.507	Promoter(1-2kb)	1524	AHNAK2	113146
450458	15q	23439979	23442067	2089	13	0.538	5'UTR	5167	GOLGA6L2	283685
450458	15q	78765997	78766840	844	6	0.333	Promoter(<=1kb)	-884	ADAMTS7	11173
450458	15q	85579207	85581800	2594	15	0.6	Promoter(1-2kb)	-1110	AKAP13	11214
450458	15q	99129423	99132517	3095	6	0.333	Exon(exon4of5)	7225	TTC23	64927
450458	16p	1228744	1229731	988	11	0.727	Promoter(<=1kb)	431	TPSB2	64499
450458	16p	1486371	1488463	2093	8	0.75	Promoter(<=1kb)	4	PTX4	390667
450458	16q	88428539	88429600	1062	6	0.333	Exon(exon3of3)	-23680	ZFPM1	161882
450458	16q	89226863	89228289	1427	7	0.571	Promoter(2-3kb)	2229	ZNF778	197320
450458	17p	10638198	10641099	2902	7	0.286	Exon(exon19of41)	-8169	MYH3	4621
450458	17p	21300581	21300978	398	12	0.75	3'UTR	9112	MAP2K3	5606
450458	17p	21415470	21416370	901	8	0.75	Exon(exon3of3)	10334	KCNJ12	3768
450458	17q	53823368	53824891	1524	6	0.833	Promoter(<=1kb)	441	KIF2B	84643
450458	17q	76293419	76294016	598	6	0.5	Promoter(2-3kb)	-2167	QRICH2	84074
450458	17q	81645135	81645417	283	6	0.333	Promoter(2-3kb)	2722	TSPAN10	83882
450458	18p	11609904	11610469	566	8	0.625	Promoter(<=1kb)	308	SLC35G4	646000
450458	18q	58535186	58537515	2330	9	0.333	Promoter(<=1kb)	0	ALPK2	115701
450458	18q	75285722	75287404	1683	6	0.333	Exon(exon2of2)	41146	TSHZ1	10194
450458	19p	1036457	1036914	458	6	0.5	Exon(exon6of7)	-3187	ABCA7	10347
450458	19p	4510548	4513547	3000	21	0.429	Exon(exon3of6)	4157	PLIN4	729359
450458	19p	5455600	5456439	840	7	0.571	Promoter(<=1kb)	183	ZNRF4	148066
450458	19p	8937644	8939234	1591	6	0.667	Exon(exon5of84)	-41554	MUC16	94025
450458	19p	8946313	8951868	5556	19	0.632	Exon(exon3of84)	29474	MUC16	94025
450458	19p	8959116	8962299	3184	11	0.727	Exon(exon3of84)	19043	MUC16	94025
450458	19p	8971838	8978096	6259	15	0.533	Exon(exon1of84)	3246	MUC16	94025
450458	19p	12430718	12432437	1720	8	0.375	3'UTR	8584	ZNF443	10224
450458	19p	18264798	18267409	2612	12	0.583	5'UTR	7002	IQCN	80726
450458	19p	21971930	21974500	2571	7	0.714	Exon(exon4of4)	14408	ZNF208	7757
450458	19q	36996730	36997597	868	10	0.6	Exon(exon10of10)	5677	ZNF568	374900
450458	19q	37886924	37888806	1883	6	0.667	Exon(exon6of6)	17788	WDR87	83889
450458	19q	39877222	39877880	659	6	0.5	Exon(exon20of28)	9412	FCGBP	8857
450458	19q	39886240	39886422	183	6	0.667	Promoter(<=1kb)	870	FCGBP	8857
450458	19q	40880231	40880622	392	6	0.333	Promoter(<=1kb)	-141	CYP2A7	1549
450458	19q	43846955	43848536	1582	6	0.833	3'UTR	13450	ZNF283	284349
450458	19q	43913423	43914878	1456	8	0.5	Exon(exon10of10)	4861	ZNF45	7596
450458	19q	44106512	44108078	1567	7	0	Exon(exon6of6)	-4103	ZNF225	7768
450458	19q	52437918	52439242	1325	7	0.429	Exon(exon4of4)	6504	ZNF534	147658
450458	19q	55911888	55913077	1190	6	0.667	Exon(exon5of12)	19234	NLRP13	126204
450458	20p	5922577	5923643	1067	9	0.667	Exon(exon4of5)	7079	CHGB	1114
450458	20q	63349752	63350772	1021	6	0.5	3'UTR	3794	CHRNA4	1137
450458	20q	63561666	63565531	3866	13	0.615	Promoter(<=1kb)	-61	HELZ2	85441
450458	21q	44637474	44638041	568	9	0.333	Promoter(<=1kb)	118	KRTAP10-10	353333
450458	21q	44666460	44666841	382	7	0.714	Promoter(<=1kb)	86	KRTAP12-2	353323
450458	22q	22352950	22353298	349	13	0.538	Exon(exon1of2)	30478	BMS1P20	96610
450458	22q	36191154	36191906	753	6	0.667	3'UTR	9971	APOL4	80832
450458	22q	50503556	50505734	2179	6	0.833	Promoter(<=1kb)	0	LMF2	91289
450458	23p	8170039	8170141	103	6	0.5	Promoter(1-2kb)	1126	VCX2	51480
450458	23p	35802148	35803010	863	7	0.571	5'UTR	3357	MAGEB16	139604
450458	23q	141905856	141906494	639	10	0.7	Promoter(1-2kb)	1054	MAGEC1	9947
451811	1p	11766028	11768307	2280	6	0.833	Promoter(<=1kb)	0	C1orf167	284498
451811	1p	11778784	11779941	1158	7	0.714	Promoter(<=1kb)	0	C1orf167-AS1	102724659
451811	1p	12859108	12860212	1105	10	0.5	Promoter(2-3kb)	2022	PRAMEF2	65122
451811	1p	12861255	12861721	467	7	0.286	Exon(exon4of4)	4169	PRAMEF2	65122
451811	1p	16058491	16060000	1510	13	0.846	Exon(exon5of7)	6168	CLCNKB	1188
451811	1p	40067594	40067675	82	6	0	Promoter(<=1kb)	324	CAP1	10487
451811	1p	89186388	89186419	32	9	0.556	Promoter(<=1kb)	107	GBP4	115361
451811	1q	152213274	152213316	43	6	0.667	Exon(exon3of3)	10877	HRNR	388697
451811	1q	152218469	152221375	2907	16	0.688	Promoter(2-3kb)	2818	HRNR	388697
451811	1q	158765805	158766655	851	6	0.5	Promoter(<=1kb)	47	OR6N1	128372
451811	1q	169542317	169542882	566	6	0.167	Exon(exon13of25)	-26572	F5	2153
451811	1q	197100802	197101771	970	6	0.5	Exon(exon18of28)	33373	ASPM	259266
451811	1q	201206099	201209342	3244	11	0.636	Promoter(1-2kb)	1017	IGFN1	91156
451811	1q	201210956	201212792	1837	6	0.333	Promoter(<=1kb)	0	IGFN1	91156
451811	1q	228315976	228318038	2063	10	0.7	Exon(exon50of81)	6492	OBSCN	84033
451811	1q	236553600	236555893	2294	6	0.333	Exon(exon39of45)	10304	LGALS8	3964
451811	1q	247841312	247841582	271	6	0.833	Promoter(<=1kb)	314	OR11L1	391189
451811	1q	247949443	247949738	296	9	0.333	Promoter(<=1kb)	585	OR2L8	391190
451811	2q	97547464	97547725	262	6	0.5	3'UTR	-35855	ANKRD36B	57730
451811	2q	132783061	132784972	1912	7	0.429	Promoter(1-2kb)	-1038	NCKAP5	344148
451811	2q	167246794	167248478	1685	7	0.714	Promoter(<=1kb)	-204	XIRP2	129446
451811	2q	185789865	185794632	4768	10	0.8	Promoter(<=1kb)	0	FSIP2	401024

451811	2q	217847583	217848969	1387	7	0.857	Exon(exon19of33)	-5423	TNS1	7145
451811	2q	233681970	233682693	724	6	0.667	Promoter(<=1kb)	0	UGT1A7	54577
451811	2q	233713134	233713664	531	8	0.75	Promoter(<=1kb)	142	UGT1A5	54579
451811	2q	233840612	233842185	1574	6	0.667	Promoter(<=1kb)	0	HJURP	55355
451811	2q	238130542	238131702	1161	7	0.714	Promoter(1-2kb)	1594	ESPNL	339768
451811	2q	240041845	240042131	287	6	0.167	Downstream(2-3kb)	3941	OR6B3	150681
451811	3p	31989643	31990470	828	6	0.167	Exon(exon2of2)	7872	ZNF860	344787
451811	3p	75737230	75739007	1778	9	0.556	Promoter(<=1kb)	0	MIR4273	100422955
451811	3q	194341097	194342571	1475	6	0.667	Exon(exon2of2)	8747	CPN2	1370
451811	4p	5988383	5989749	1367	7	0.571	Promoter(<=1kb)	0	C4orf50	389197
451811	4p	6300792	6302360	1569	6	0.833	Exon(exon8of8)	6021	WFS1	7466
451811	4p	8227004	8228508	1505	7	0.143	Promoter(<=1kb)	-24	SH3TC1	54436
451811	4p	10443803	10446224	2422	7	0.429	Exon(exon3of3)	10952	ZNF518B	85460
451811	4p	38774486	38775552	1067	8	0.5	Promoter(<=1kb)	644	TLR10	81793
451811	4q	121036404	121037542	1139	7	0.429	Promoter(1-2kb)	1442	NDNF	79625
451811	5q	140848579	140850786	2208	6	0.5	Promoter(<=1kb)	807	PCDHA9	9752
451811	5q	141100771	141102758	1988	11	0.273	Promoter(<=1kb)	298	PCDHB3	56132
451811	5q	141955356	141957660	2305	7	0.714	Promoter(<=1kb)	-668	RNF14	9604
451811	6p	16327099	16327684	586	6	0.333	Exon(exon8of9)	37154	GMPR	2766
451811	6p	46858771	46859502	732	9	0.556	Exon(exon17of21)	3802	ADGRF5	221395
451811	6q	168307821	168309048	1228	7	0.429	5'UTR	10662	DACT2	168002
451811	7q	64991278	64992758	1481	6	0.667	Promoter(<=1kb)	-242	ZNF117	51351
451811	7q	100958977	100960873	1897	50	0.46	Promoter(1-2kb)	1012	MUC3A	4584
451811	7q	100991359	100993912	2554	9	0.556	Exon(exon5of15)	-19142	MUC12	10071
451811	7q	100995424	100995785	362	7	0.714	Exon(exon5of15)	-17269	MUC12	10071
451811	8p	10609650	10612307	2658	9	0.889	Exon(exon4of4)	42836	RP1L1	94137
451811	8p	11331194	11332082	889	9	0.667	Promoter(<=1kb)	306	SLC35G5	83650
451811	8p	13021128	13022030	903	7	0.143	Exon(exon5of5)	9115	TRMT9B	57604
451811	8q	123651655	123652634	980	7	0.571	Promoter(<=1kb)	316	KLHL38	340359
451811	8q	141218050	141219792	1743	6	0.667	5'UTR	8778	SLC45A4	57210
451811	9p	116800	117696	897	6	0.833	Promoter(<=1kb)	508	FOXD4	2298
451811	9p	214679	215269	591	6	0.5	Promoter(<=1kb)	0	DOCK8	81704
451811	9p	21206764	21207074	311	6	0.5	Promoter(<=1kb)	69	IFNA10	3446
451811	9p	39078723	39078846	124	6	0.667	Exon(exon22of24)	7302	CNTNAP3	79937
451811	9q	76175237	76175301	65	11	0.727	Exon(exon14of14)	-13338	PCSK5	5125
451811	9q	76705724	76707804	2081	6	0.5	Promoter(<=1kb)	666	PCA3	50652
451811	9q	104598641	104599361	721	8	0.625	Promoter(<=1kb)	52	OR13C5	138799
451811	9q	122749914	122750547	634	6	0.833	Promoter(<=1kb)	174	OR1L6	392390
451811	10p	47663	48605	943	6	0.833	Promoter(<=1kb)	664	TUBB8	347688
451811	10q	46549378	46550723	1346	25	0.64	Exon(exon3of3)	4807	GPRIN2	9721
451811	10q	49323559	49326331	2773	9	0.444	Exon(exon3of3)	24285	C10orf71	118461
451811	10q	122084988	122087840	2853	9	0.778	Exon(exon4of23)	-25190	TACC2	10579
451811	10q	128102802	128106296	3495	13	0.538	Promoter(<=1kb)	0	MKI67	4288
451811	11p	244106	244197	92	8	0.5	Promoter(<=1kb)	-232	PSMD13	5719
451811	11p	1242953	1244556	1604	7	0.429	Exon(exon31of49)	5120	MUC5B-AS1	112577518
451811	11p	1246332	1248605	2274	8	0.75	Promoter(1-2kb)	1071	MUC5B-AS1	112577518
451811	11p	5177978	5178478	501	6	0.167	Promoter(<=1kb)	186	OR52Z1	283110
451811	11p	5422212	5423123	912	11	0.636	Promoter(<=1kb)	101	OR51Q1	390061
451811	11p	5515185	5515931	747	6	0.333	Promoter(<=1kb)	768	UBQLNL	143630
451811	11p	5581045	5581738	694	8	0.375	Promoter(<=1kb)	168	OR52B6	340980
451811	11p	5788000	5788760	761	8	0.75	Promoter(<=1kb)	56	OR52N1	79473
451811	11p	5841302	5841883	582	9	0.333	Promoter(<=1kb)	14	OR52E6	390078
451811	11p	11351961	11352736	776	9	0.222	Promoter(<=1kb)	514	CSNK2A3	283106
451811	11p	12293639	12294538	900	8	0.625	Exon(exon29of35)	6739	MICALCL	84953
451811	11q	55827536	55827640	105	6	0.667	Promoter(<=1kb)	317	OR5L2	26338
451811	11q	58214757	58215722	966	8	0.25	Promoter(<=1kb)	12	OR1S1	219959
451811	11q	82732630	82733184	555	6	0.833	Promoter(<=1kb)	680	FAM181B	220382
451811	11q	123906790	123907324	535	7	0.714	Promoter(<=1kb)	644	OR8D4	338662
451811	11q	124015600	124016477	878	7	0.143	Promoter(<=1kb)	25	OR10G4	390264
451811	11q	124038366	124038988	623	9	0.889	Promoter(<=1kb)	13	OR10G7	390265
451811	11q	130914501	130915409	909	10	0.7	Promoter(1-2kb)	1035	SNX19	399979
451811	12p	4626568	4628549	1982	11	0.545	Exon(exon5of6)	14051	DYRK4	8798
451811	12p	8222174	8223514	1341	6	0.667	Exon(exon5of6)	4073	FAM90A1	55138
451811	13q	24434450	24435347	898	7	0.571	Exon(exon31of34)	19787	PARP4	143
451811	13q	25096665	25097231	567	11	0.455	Promoter(<=1kb)	797	PABPC3	5042
451811	13q	102732474	102733933	1460	6	0.333	Exon(exon4of4)	25139	CCDC168	643677
451811	14q	19975713	19976448	736	9	0.444	Promoter(<=1kb)	269	OR4K15	81127
451811	14q	20060048	20060884	837	8	0.625	Promoter(<=1kb)	3	OR4L1	122742
451811	14q	70457532	70458540	1009	9	0.333	Exon(exon2of2)	5358	ADAM21	8747
451811	14q	104947943	104953878	5936	29	0.483	Promoter(1-2kb)	1524	AHNAK2	113146
451811	15q	20534480	20534954	475	6	0.333	Exon(exon8of9)	6846	GOLGA6L6	727832
451811	15q	23439979	23442067	2089	13	0.538	5'UTR	5167	GOLGA6L2	283685
451811	15q	78765975	78766638	664	10	0.5	Promoter(<=1kb)	-862	ADAMTS7	11173
451811	15q	99129423	99132394	2972	9	0.556	Exon(exon4of5)	7348	TTC23	64927
451811	16p	669592	672548	2957	7	0.571	Promoter(<=1kb)	0	RHOT2	89941
451811	16p	768559	770215	1657	7	0.429	Promoter(<=1kb)	0	MIR662	724032
451811	16p	1486371	1488463	2093	8	0.75	Promoter(<=1kb)	4	PTX4	390667

451811	16q	89100686	89101050	365	7	0.571	Promoter(<=1kb)	24	ACSF3	197322
451811	16q	89226863	89228289	1427	8	0.625	Promoter(2-3kb)	2229	ZNF778	197320
451811	17p	21300581	21300978	398	12	0.75	3'UTR	9112	MAP2K3	5606
451811	17p	21415470	21416517	1048	9	1	Exon(exon3of3)	10334	KCNJ12	3768
451811	17q	76293419	76294016	598	6	0.5	Promoter(2-3kb)	-2167	QRICH2	84074
451811	17q	81645135	81645607	473	8	0.375	Promoter(2-3kb)	2722	TSPAN10	83882
451811	18p	11609646	11610491	846	13	0.692	Promoter(<=1kb)	50	SLC35G4	646000
451811	18q	58535186	58538030	2845	19	0.526	Promoter(<=1kb)	0	ALPK2	115701
451811	19p	4510548	4513547	3000	23	0.435	Exon(exon3of6)	4157	PLIN4	729359
451811	19p	5455600	5456439	840	7	0.571	Promoter(<=1kb)	183	ZNRF4	148066
451811	19p	8333830	8334965	1136	6	0.667	Promoter(<=1kb)	0	KANK3	256949
451811	19p	8948231	8951868	3638	11	0.545	Exon(exon3of84)	29474	MUC16	94025
451811	19p	12430718	12432437	1720	8	0.375	3'UTR	8584	ZNF443	10224
451811	19p	15087213	15088040	828	10	0.3	Promoter(<=1kb)	233	OR1I1	126370
451811	19p	17281820	17284246	2427	9	0.556	Promoter(<=1kb)	0	ANKLE1	126549
451811	19p	18264798	18267409	2612	12	0.583	5'UTR	7002	IQCN	80726
451811	19p	21971930	21974500	2571	10	0.7	Exon(exon4of4)	14408	ZNF208	7757
451811	19p	22756205	22759523	3319	13	0.615	3'UTR	10459	ZNF99	7652
451811	19q	33205409	33207385	1977	6	0.667	Promoter(1-2kb)	1539	LRP3	4037
451811	19q	37885190	37888806	3617	6	0.5	Exon(exon6of6)	17788	WDR87	83889
451811	19q	39877222	39878209	988	6	0.5	Exon(exon20of28)	9083	FCGBP	8857
451811	19q	39893459	39894014	556	6	0.667	Exon(exon12of28)	-6167	FCGBP	8857
451811	19q	43913423	43914878	1456	8	0.5	Exon(exon10of10)	4861	ZNF45	7596
451811	19q	44106512	44108078	1567	8	0.125	Exon(exon6of6)	-4103	ZNF225	7768
451811	19q	51745958	51746963	1006	6	0.333	Exon(exon3of3)	3848	FPR1	2357
451811	19q	52437918	52439242	1325	7	0.429	Exon(exon4of4)	6504	ZNF534	147658
451811	19q	55911888	55913077	1190	6	0.667	Exon(exon5of12)	19234	NLRP13	126204
451811	20p	20052354	20052736	383	6	0	Promoter(<=1kb)	0	CFAP61	26074
451811	20q	63561666	63565531	3866	12	0.667	Promoter(<=1kb)	-61	HELZ2	85441
451811	21q	26843740	26844859	1120	6	0.667	Promoter(<=1kb)	0	ADAMTS1	9510
451811	21q	44550738	44551416	679	7	0.857	Promoter(<=1kb)	89	KRTAP10-2	386679
451811	21q	44637474	44638143	670	11	0.455	Promoter(<=1kb)	118	KRTAP10-10	353333
451811	21q	44666460	44666841	382	7	0.714	Promoter(<=1kb)	86	KRTAP12-2	353323
451811	22q	36191154	36191906	753	6	0.667	3'UTR	9971	APOL4	80832
451811	22q	39100331	39102033	1703	6	0.833	Promoter(<=1kb)	52	APOBEC3H	164668
451811	23p	3320126	3323750	3625	9	0.556	Exon(exon5of7)	22902	MXRA5	25878
451811	23p	8170039	8170141	103	6	0.5	Promoter(1-2kb)	1126	VCX2	51480
451811	23q	136874183	136874347	165	7	0.714	Promoter(<=1kb)	-201	RBMX	27316
453143	1p	978953	979847	895	6	0.5	Promoter(1-2kb)	1182	PERM1	84808
453143	1p	12847526	12847995	470	8	0.5	Promoter(<=1kb)	730	HNRNPCL1	343069
453143	1p	12859036	12860079	1044	6	0.333	Promoter(1-2kb)	1950	PRAMEF2	65122
453143	1p	13369166	13369564	399	7	0.714	Promoter(2-3kb)	2336	PRAMEF19	645414
453143	1p	13370686	13371119	434	7	0.429	Promoter(<=1kb)	781	PRAMEF19	645414
453143	1p	18481403	18482217	815	7	0.714	Promoter(<=1kb)	421	KLHDC7A	127707
453143	1p	40067594	40067675	82	6	0	Promoter(<=1kb)	324	CAP1	10487
453143	1q	152218469	152221375	2907	15	0.667	Promoter(2-3kb)	2818	HRNR	388697
453143	1q	152303673	152313891	10219	36	0.583	Promoter(<=1kb)	0	FLG-AS1	339400
453143	1q	156669844	156671561	1718	7	0.857	Exon(exon4of4)	5846	NES	10763
453143	1q	169542317	169542882	566	6	0.167	Exon(exon13of25)	-26572	F5	2153
453143	1q	223393517	223394466	950	6	0.667	Promoter(<=1kb)	102	CCDC185	164127
453143	1q	226735563	226737239	1677	8	0.875	Promoter(<=1kb)	219	ITPKB	3707
453143	1q	228315976	228318026	2051	7	0.571	Exon(exon50of81)	6492	OBSCN	84033
453143	1q	247841312	247841582	271	6	0.833	Promoter(<=1kb)	314	OR11L1	391189
453143	1q	247949325	247949738	414	9	0.333	Promoter(<=1kb)	467	OR2L8	391190
453143	2p	29002636	29003646	1011	6	0.333	Exon(exon5of20)	-10821	TOGARAM2	165186
453143	2q	167246794	167248478	1685	7	0.714	Promoter(<=1kb)	-204	XIRP2	129446
453143	2q	185789865	185794632	4768	11	0.818	Promoter(<=1kb)	0	FSIP2	401024
453143	2q	238130416	238131546	1131	6	0.333	Promoter(1-2kb)	1468	ESPNL	339768
453143	3p	31989532	31990905	1374	6	0.333	Exon(exon2of2)	7761	ZNF860	344787
453143	3p	75736880	75739243	2364	41	0.634	Promoter(<=1kb)	0	MIR4273	100422955
453143	3q	98264413	98265098	686	7	0.571	Promoter(<=1kb)	128	OR5H6	79295
453143	3q	194341097	194342571	1475	7	0.714	Exon(exon2of2)	8747	CPN2	1370
453143	4p	5988383	5989749	1367	7	0.571	Promoter(<=1kb)	0	C4orf50	389197
453143	4p	6300792	6302360	1569	6	0.833	Exon(exon8of8)	6021	WFS1	7466
453143	4p	8227004	8228508	1505	7	0.143	Promoter(<=1kb)	-24	SH3TC1	54436
453143	4q	112431241	112432293	1053	7	0.429	3'UTR	4735	ALPK1	80216
453143	4q	185458217	185460011	1795	8	0.625	Promoter(<=1kb)	0	CCDC110	256309
453143	4q	186617176	186619764	2589	7	0.429	Exon(exon10of27)	-7106	FAT1	2195
453143	4q	186706638	186708616	1979	6	0.667	Exon(exon2of27)	15217	FAT1	2195
453143	5p	795818	796218	401	6	0.833	3'UTR	4927	ZDHHC11	79844
453143	5q	140807352	140807737	386	6	0.833	Promoter(<=1kb)	271	PCDHA4	56144
453143	5q	141100771	141102758	1988	11	0.273	Promoter(<=1kb)	298	PCDHB3	56132
453143	6p	46858771	46859389	619	6	0.5	Exon(exon17of21)	3915	ADGRF5	221395
453143	6q	159231899	159234370	2472	10	0.5	Exon(exon11of23)	13602	FNDC1	84624
453143	7p	6330446	6330944	499	6	1	Exon(exon2of2)	7749	FAM220A	84792
453143	7p	53035678	53036385	708	7	1	Promoter(<=1kb)	45	POM121L12	285877
453143	7p	56021087	56021209	123	6	0.5	Exon(exon2of7)	12947	PSPH	5723

453143	7q	100952225	100953156	932	6	0.833	Promoter(2-3kb)	2600	MUC3A	4584
453143	7q	100958977	100960873	1897	52	0.442	Promoter(1-2kb)	1012	MUC3A	4584
453143	7q	100991195	100994057	2863	15	0.667	Exon(exon5of15)	-18997	MUC12	10071
453143	7q	100995424	100995785	362	7	0.857	Exon(exon5of15)	-17269	MUC12	10071
453143	7q	149818015	149819792	1778	6	0.667	Promoter(2-3kb)	-2352	SSPO	23145
453143	8p	10607375	10612558	5184	17	0.706	Exon(exon4of4)	42585	RP1L1	94137
453143	8p	11331194	11332026	833	6	0.667	Promoter(<=1kb)	306	SLC35G5	83650
453143	8p	13021128	13022030	903	7	0.143	Exon(exon5of5)	9115	TRMT9B	57604
453143	8q	138151949	138153046	1098	6	0.667	Promoter(<=1kb)	0	FAM135B	51059
453143	9p	116800	117696	897	7	0.857	Promoter(<=1kb)	508	FOXD4	2298
453143	9p	39078723	39078753	31	6	0.833	Exon(exon22of24)	7395	CNTNAP3	79937
453143	9q	76175237	76175296	60	10	0.8	Exon(exon14of14)	-13343	PCSK5	5125
453143	9q	76703451	76704082	632	6	0.333	Promoter(<=1kb)	-13	PRUNE2	158471
453143	9q	76705724	76707804	2081	6	0.5	Promoter(<=1kb)	666	PCA3	50652
453143	9q	87886533	87888713	2181	9	0.667	Exon(exon4of4)	3656	SPATA31E1	286234
453143	9q	122553263	122554071	809	7	0.429	Promoter(<=1kb)	93	OR1N2	138882
453143	9q	122628595	122629398	804	7	0.286	Promoter(<=1kb)	175	OR1B1	347169
453143	9q	122749914	122750547	634	6	0.833	Promoter(<=1kb)	174	OR1L6	392390
453143	9q	135484803	135487340	2538	10	0.4	Promoter(1-2kb)	1440	PPP1R26	9858
453143	10q	46549378	46550723	1346	25	0.64	Exon(exon3of3)	4807	GPRIN2	9721
453143	10q	49323559	49326554	2996	11	0.545	Exon(exon3of3)	24285	C10orf71	118461
453143	10q	122084988	122087840	2853	9	0.778	Exon(exon4of23)	-25190	TACC2	10579
453143	10q	128102594	128105201	2608	6	0.667	Promoter(<=1kb)	0	MKI67	4288
453143	11p	244106	244197	92	8	0.5	Promoter(<=1kb)	-232	PSMD13	5719
453143	11p	1194354	1196902	2549	7	0.571	Exon(exon34of49)	-26164	MUC5B	727897
453143	11p	1246095	1247378	1284	7	0.429	Promoter(2-3kb)	2298	MUC5B-AS1	112577518
453143	11p	1250091	1251628	1538	6	0.833	Promoter(<=1kb)	-415	MUC5B-AS1	112577518
453143	11p	5177978	5178478	501	6	0.167	Promoter(<=1kb)	186	OR52Z1	283110
453143	11p	5323451	5324256	806	6	0.5	Promoter(<=1kb)	41	OR51B2	79345
453143	11p	5422212	5423123	912	12	0.667	Promoter(<=1kb)	101	OR51Q1	390061
453143	11p	5581045	5581738	694	8	0.375	Promoter(<=1kb)	168	OR52B6	340980
453143	11p	5841302	5841883	582	9	0.333	Promoter(<=1kb)	14	OR52E6	390078
453143	11p	5884818	5885061	244	7	0.429	Promoter(<=1kb)	547	OR52E4	390081
453143	11p	11351961	11352736	776	9	0.222	Promoter(<=1kb)	514	CSNK2A3	283106
453143	11p	12293639	12294842	1204	7	0.714	Exon(exon29of35)	6739	MICALCL	84953
453143	11p	18173280	18173901	622	6	0.333	Promoter(<=1kb)	443	MRGPRX4	117196
453143	11q	58214757	58215722	966	8	0.25	Promoter(<=1kb)	12	OR1S1	219959
453143	11q	64116513	64118232	1720	7	0.714	Exon(exon2of2)	8702	MACROD1	28992
453143	11q	69295389	69296261	873	6	0.667	Promoter(1-2kb)	1215	MYEOV	26579
453143	11q	123906790	123907324	535	6	0.667	Promoter(<=1kb)	644	OR8D4	338662
453143	11q	123943088	123943782	695	7	0.571	Promoter(<=1kb)	91	OR6T1	219874
453143	11q	124015601	124016477	877	8	0.25	Promoter(<=1kb)	26	OR10G4	390264
453143	11q	124023038	124023849	812	9	0.444	Promoter(<=1kb)	25	OR10G9	219870
453143	11q	124038366	124038988	623	9	0.889	Promoter(<=1kb)	13	OR10G7	390265
453143	11q	130914501	130915409	909	10	0.7	Promoter(1-2kb)	1035	SNX19	399979
453143	12p	4626571	4628549	1979	9	0.444	Exon(exon5of6)	14054	DYRK4	8798
453143	12p	8222174	8223514	1341	6	0.667	Exon(exon5of6)	4073	FAM90A1	55138
453143	12q	52316096	52317765	1670	6	0.667	Exon(exon4of9)	3633	KRT83	3889
453143	13q	24434450	24435347	898	7	0.571	Exon(exon31of34)	19787	PARP4	143
453143	13q	49668389	49668431	43	6	0.167	Exon(exon3of3)	23007	EBPL	84650
453143	13q	102732474	102733933	1460	6	0.333	Exon(exon4of4)	25139	CCDC168	643677
453143	14q	19935750	19936577	828	6	0.833	Promoter(<=1kb)	142	OR4K1	79544
453143	14q	20640750	20641567	818	7	0.571	Promoter(<=1kb)	124	OR6S1	341799
453143	14q	21634137	21634589	453	9	0.556	Promoter(<=1kb)	351	OR10G2	26534
453143	14q	70457532	70458540	1009	9	0.333	Exon(exon2of2)	5358	ADAM21	8747
453143	14q	94587512	94587839	328	6	0.5	Exon(exon2of2)	-4219	SERPINA3	12
453143	14q	104939262	104942618	3357	10	0.2	5'UTR	7102	PLD4	122618
453143	14q	104943622	104945444	1823	7	0.571	Exon(exon6of6)	9958	AHNAK2	113146
453143	14q	104947943	104953878	5936	34	0.5	Promoter(1-2kb)	1524	AHNAK2	113146
453143	15q	23440160	23442067	1908	12	0.583	5'UTR	5167	GOLGA6L2	283685
453143	15q	85579423	85581800	2378	14	0.571	Promoter(1-2kb)	-1110	AKAP13	11214
453143	15q	99129423	99132517	3095	6	0.333	Exon(exon4of5)	7225	TTC23	64927
453143	15q	100569472	100570097	626	6	0.833	Promoter(<=1kb)	534	LINS1	55180
453143	16p	1486322	1488362	2041	7	0.714	Promoter(<=1kb)	105	PTX4	390667
453143	16p	1998795	2000191	1397	7	0.714	Promoter(<=1kb)	0	ZNF598	90850
453143	16q	89100686	89101050	365	7	0.571	Promoter(<=1kb)	24	ACSF3	197322
453143	16q	89226863	89228289	1427	7	0.571	Promoter(2-3kb)	2229	ZNF778	197320
453143	17p	7846688	7848460	1773	6	0.667	Promoter(1-2kb)	1773	KDM6B	23135
453143	17p	10638198	10641099	2902	7	0.286	Exon(exon19of41)	-8169	MYH3	4621
453143	17p	21300581	21300978	398	12	0.75	3'UTR	9112	MAP2K3	5606
453143	17q	53823723	53824891	1169	6	0.833	Promoter(<=1kb)	796	KIF2B	84643
453143	17q	81645135	81645417	283	6	0.333	Promoter(2-3kb)	2722	TSPAN10	83882
453143	18p	11609728	11610164	437	14	0.643	Promoter(<=1kb)	132	SLC35G4	646000
453143	18p	11644365	11644598	234	8	0.875	Exon(exon1of1)	10343	MIR7153	102465690
453143	18q	58535186	58537515	2330	10	0.4	Promoter(<=1kb)	0	ALPK2	115701
453143	19p	4510548	4513547	3000	15	0.467	Exon(exon3of6)	4157	PLIN4	729359
453143	19p	5455600	5456439	840	8	0.625	Promoter(<=1kb)	183	ZNRF4	148066

453143	19p	8946313	8951868	5556	15	0.6	Exon(exon3of84)	29474	MUC16	94025
453143	19p	8959116	8962299	3184	10	0.7	Exon(exon3of84)	19043	MUC16	94025
453143	19p	8972751	8978096	5346	13	0.462	Exon(exon1of84)	3246	MUC16	94025
453143	19p	12430718	12432437	1720	8	0.375	3'UTR	8584	ZNF443	10224
453143	19p	15794192	15794719	528	6	0.333	Promoter(<=1kb)	241	OR10H5	284433
453143	19p	17282085	17284246	2162	8	0.5	Promoter(<=1kb)	0	ANKLE1	126549
453143	19p	18264753	18267409	2657	8	0.5	5'UTR	7002	IQCN	80726
453143	19p	23743906	23745300	1395	6	0.333	Exon(exon4of4)	13537	ZNF681	148213
453143	19q	35125411	35126735	1325	6	0.667	Promoter(2-3kb)	2711	MIR6887	102466205
453143	19q	36996730	36997597	868	11	0.636	Exon(exon10of10)	5677	ZNF568	374900
453143	19q	37151928	37153149	1222	6	0.833	Exon(exon5of5)	19287	ZNF585A	199704
453143	19q	37885190	37888806	3617	8	0.625	Exon(exon6of6)	17788	WDR87	83889
453143	19q	43913423	43914878	1456	8	0.5	Exon(exon10of10)	4861	ZNF45	7596
453143	19q	44106512	44108078	1567	7	0	Exon(exon6of6)	-4103	ZNF225	7768
453143	19q	51745958	51746963	1006	7	0.429	Exon(exon3of3)	3848	FPR1	2357
453143	19q	52437918	52439242	1325	7	0.429	Exon(exon4of4)	6504	ZNF534	147658
453143	19q	55517821	55518189	369	8	1	Exon(exon14of14)	18114	SBK2	646643
453143	19q	55911888	55913077	1190	6	0.667	Exon(exon5of12)	19234	NLRP13	126204
453143	19q	58368293	58368875	583	7	0.429	Exon(exon3of3)	-5445	ZNF497	162968
453143	20p	5922421	5923394	974	6	0.5	Exon(exon4of5)	6923	CHGB	1114
453143	20q	62812284	62813587	1304	6	1	Promoter(2-3kb)	-2657	COL9A3	1299
453143	20q	63349752	63350772	1021	6	0.5	3'UTR	3794	CHRNA4	1137
453143	20q	63564900	63567309	2410	7	0.571	Promoter(<=1kb)	765	HELZ2	85441
453143	21q	26843740	26844859	1120	6	0.667	Promoter(<=1kb)	0	ADAMTS1	9510
453143	21q	44550835	44551416	582	6	0.833	Promoter(<=1kb)	89	KRTAP10-2	386679
453143	21q	44637474	44638041	568	6	0.333	Promoter(<=1kb)	118	KRTAP10-10	353333
453143	22q	22352950	22353380	431	16	0.5	Exon(exon1of2)	30478	BMS1P20	96610
453143	22q	36191154	36191906	753	7	0.571	3'UTR	9971	APOL4	80832
453143	23p	3320126	3323750	3625	9	0.556	Exon(exon5of7)	22902	MXRA5	25878
453143	23p	8170039	8170141	103	6	0.5	Promoter(1-2kb)	1126	VCX2	51480
453143	23p	35802148	35803010	863	7	0.571	5'UTR	3357	MAGEB16	139604
454638	1p	16058491	16060000	1510	10	0.9	Exon(exon5of7)	6168	CLCNKB	1188
454638	1p	18481403	18482217	815	6	0.667	Promoter(<=1kb)	421	KLHDC7A	127707
454638	1p	23874604	23875430	827	9	0.556	Exon(exon2of2)	-6310	FUCA1	2517
454638	1p	40067594	40067675	82	6	0	Promoter(<=1kb)	324	CAP1	10487
454638	1p	89186388	89186419	32	9	0.556	Promoter(<=1kb)	107	GBP4	115361
454638	1q	152213274	152213314	41	6	0.5	Exon(exon3of3)	10879	HRNR	388697
454638	1q	152218469	152221375	2907	17	0.647	Promoter(2-3kb)	2818	HRNR	388697
454638	1q	152303673	152313891	10219	37	0.595	Promoter(<=1kb)	0	FLG-AS1	339400
454638	1q	156669844	156670886	1043	6	1	Exon(exon4of4)	6521	NES	10763
454638	1q	167125926	167128502	2577	6	0.833	Exon(exon6of6)	31076	DUSP27	92235
454638	1q	197101312	197101771	460	6	0.5	Exon(exon18of28)	33373	ASPM	259266
454638	1q	228315976	228318038	2063	9	0.667	Exon(exon50of81)	6492	OBSCN	84033
454638	1q	232805117	232806800	1684	6	0.5	Promoter(<=1kb)	225	MAP10	54627
454638	1q	247841312	247841582	271	6	0.833	Promoter(<=1kb)	314	OR11L1	391189
454638	1q	247895950	247896410	461	6	0.5	Promoter(<=1kb)	363	OR2W3	343171
454638	1q	247949325	247949738	414	10	0.3	Promoter(<=1kb)	467	OR2L8	391190
454638	1q	248294677	248295458	782	7	0.571	Promoter(<=1kb)	142	OR2T12	127064
454638	2p	48580657	48582454	1798	7	0.571	Promoter(<=1kb)	0	STON1	11037
454638	2q	132781993	132785012	3020	9	0.556	Promoter(<=1kb)	0	NCKAP5	344148
454638	2q	184936178	184937636	1459	6	0.333	Exon(exon4of4)	69813	ZNF804A	91752
454638	2q	185789865	185794632	4768	13	0.692	Promoter(<=1kb)	0	FSIP2	401024
454638	2q	185805377	185807800	2424	6	0.333	Promoter(<=1kb)	0	FSIP2	401024
454638	2q	217847567	217848213	647	6	0.833	Exon(exon19of33)	-5407	TNS1	7145
454638	2q	238130271	238131546	1276	8	0.375	Promoter(1-2kb)	1323	ESPNL	339768
454638	3p	75736880	75738859	1980	61	0.623	Promoter(<=1kb)	0	MIR4273	100422955
454638	3q	98169021	98169594	574	6	0.667	Exon(exon2of2)	19695	OR5H14	403273
454638	3q	98264413	98265098	686	7	0.571	Promoter(<=1kb)	128	OR5H6	79295
454638	3q	194341097	194342571	1475	7	0.714	Exon(exon2of2)	8747	CPN2	1370
454638	3q	194359607	194360906	1300	9	0.889	Exon(exon2of2)	-8279	CPN2	1370
454638	4p	6300792	6302360	1569	7	0.857	Exon(exon8of8)	6021	WFS1	7466
454638	4p	8227004	8228508	1505	8	0.125	Promoter(<=1kb)	-24	SH3TC1	54436
454638	4q	154489498	154491312	1815	9	0.556	Promoter(<=1kb)	22	DCHS2	54798
454638	5q	79731782	79734523	2742	13	0.308	Exon(exon2of13)	-3619	CMYA5	202333
454638	5q	83537326	83539905	2580	6	0.333	Promoter(1-2kb)	1712	VCAN	1462
454638	5q	140807352	140807737	386	6	0.833	Promoter(<=1kb)	271	PCDHA4	56144
454638	5q	140848579	140850786	2208	7	0.571	Promoter(<=1kb)	807	PCDHA9	9752
454638	5q	141173599	141175025	1427	7	0.857	Promoter(<=1kb)	955	PCDHB7	56129
454638	5q	141187690	141188770	1081	6	0.333	Promoter(<=1kb)	529	PCDHB9	56127
454638	5q	160565578	160565747	170	6	0.5	Exon(exon21of21)	37544	ATP10B	23120
454638	6p	1312843	1313745	903	6	0.5	Promoter(<=1kb)	745	FOXQ1	94234
454638	6p	46858771	46859389	619	6	0.5	Exon(exon17of21)	3915	ADGRF5	221395
454638	6q	64591274	64591961	688	10	0.5	Exon(exon26of43)	121374	EYS	346007
454638	6q	149888581	149890867	2287	7	0.714	Promoter(<=1kb)	0	RAET1E-AS1	100652739
454638	6q	159231899	159234370	2472	12	0.583	Exon(exon11of23)	13602	FNDC1	84624
454638	7p	12369637	12370736	1100	6	0.667	3'UTR	-13307	VWDE	221806
454638	7p	53035678	53036385	708	6	1	Promoter(<=1kb)	45	POM121L12	285877

454638	7q	100958977	100960873	1897	53	0.453	Promoter(1-2kb)	1012	MUC3A	4584
454638	7q	100991195	100993127	1933	9	0.556	Exon(exon5of15)	-19927	MUC12	10071
454638	7q	100995575	100995785	211	6	0.833	Exon(exon5of15)	-17269	MUC12	10071
454638	8p	10607245	10608432	1188	8	0.5	Exon(exon4of4)	46711	RP1L1	94137
454638	8p	10609614	10610662	1049	8	0.5	Exon(exon4of4)	44481	RP1L1	94137
454638	8p	12137448	12138641	1194	6	0.833	Promoter(<=1kb)	436	USP17L2	377630
454638	8p	13021128	13022030	903	9	0.111	Exon(exon5of5)	9115	TRMT9B	57604
454638	8q	123651873	123652634	762	6	0.333	Promoter(<=1kb)	316	KLHL38	340359
454638	8q	138151949	138153046	1098	6	0.667	Promoter(<=1kb)	0	FAM135B	51059
454638	9p	116800	117713	914	7	0.714	Promoter(<=1kb)	491	FOXD4	2298
454638	9p	214679	215269	591	6	0.5	Promoter(<=1kb)	0	DOCK8	81704
454638	9p	21206764	21207074	311	6	0.5	Promoter(<=1kb)	69	IFNA10	3446
454638	9p	39078723	39078846	124	7	0.714	Exon(exon22of24)	7302	CNTNAP3	79937
454638	9q	76175237	76175300	64	14	0.714	Exon(exon14of14)	-13339	PCSK5	5125
454638	9q	76705179	76707804	2626	8	0.5	Promoter(<=1kb)	121	PCA3	50652
454638	9q	76709263	76710843	1581	8	0.5	Promoter(<=1kb)	0	PRUNE2	158471
454638	9q	104598545	104599318	774	10	0.6	Promoter(<=1kb)	95	OR13C5	138799
454638	9q	122553263	122554071	809	8	0.5	Promoter(<=1kb)	93	OR1N2	138882
454638	9q	122628595	122629398	804	6	0.167	Promoter(<=1kb)	175	OR1B1	347169
454638	9q	122749914	122750547	634	6	0.833	Promoter(<=1kb)	174	OR1L6	392390
454638	9q	133255635	133256205	571	6	1	3'UTR	19009	ABO	28
454638	10q	46549378	46550723	1346	27	0.667	Exon(exon3of3)	4807	GPRIN2	9721
454638	10q	49323169	49326817	3649	11	0.545	Exon(exon3of3)	23895	C10orf71	118461
454638	10q	122084988	122087840	2853	8	0.75	Exon(exon4of23)	-25190	TACC2	10579
454638	10q	133625384	133625511	128	7	0.714	Promoter(1-2kb)	1281	FRG2B	441581
454638	11p	244106	244197	92	8	0.5	Promoter(<=1kb)	-232	PSMD13	5719
454638	11p	1246095	1247378	1284	8	0.375	Promoter(2-3kb)	2298	MUC5B-AS1	112577518
454638	11p	1835440	1837342	1903	6	0.667	Promoter(<=1kb)	0	SYT8	90019
454638	11p	5177978	5178478	501	6	0.167	Promoter(<=1kb)	186	OR52Z1	283110
454638	11p	5422212	5423123	912	12	0.667	Promoter(<=1kb)	101	OR51Q1	390061
454638	11p	5440761	5441472	712	6	0.833	Promoter(<=1kb)	42	OR51I1	390063
454638	11p	5515079	5515931	853	7	0.429	Promoter(<=1kb)	768	UBQLNL	143630
454638	11p	5544676	5545259	584	6	0.5	Promoter(<=1kb)	290	OR52H1	390067
454638	11p	5581208	5581738	531	7	0.429	Promoter(<=1kb)	331	OR52B6	340980
454638	11p	5884818	5885061	244	6	0.333	Promoter(<=1kb)	547	OR52E4	390081
454638	11p	11351961	11352736	776	7	0.143	Promoter(<=1kb)	514	CSNK2A3	283106
454638	11p	12293639	12294842	1204	7	0.714	Exon(exon29of35)	6739	MICALCL	84953
454638	11p	18173280	18173901	622	6	0.5	Promoter(<=1kb)	443	MRGPRX4	117196
454638	11q	58214757	58215722	966	8	0.25	Promoter(<=1kb)	12	OR1S1	219959
454638	11q	64315804	64315856	53	7	0.714	Promoter(1-2kb)	1485	TRMT112	51504
454638	11q	66560202	66562261	2060	6	0.333	Exon(exon14of21)	3873	CTSF	8722
454638	11q	85724687	85725825	1139	6	0.5	Promoter(<=1kb)	0	SYTL2	54843
454638	11q	123906510	123907324	815	7	0.714	Promoter(<=1kb)	364	OR8D4	338662
454638	11q	124038366	124038988	623	7	1	Promoter(<=1kb)	13	OR10G7	390265
454638	11q	124382526	124383285	760	8	0.625	Promoter(<=1kb)	58	OR8B2	26595
454638	12p	4626568	4627524	957	7	0.429	Exon(exon5of6)	14051	DYRK4	8798
454638	13q	24434450	24435347	898	7	0.571	Exon(exon31of34)	19787	PARP4	143
454638	13q	25096713	25097154	442	21	0.524	Promoter(<=1kb)	845	PABPC3	5042
454638	13q	102732474	102733933	1460	6	0.333	Exon(exon4of4)	25139	CCDC168	643677
454638	14q	19975713	19976448	736	8	0.375	Promoter(<=1kb)	269	OR4K15	81127
454638	14q	20060048	20060884	837	8	0.625	Promoter(<=1kb)	3	OR4L1	122742
454638	14q	21634137	21634589	453	9	0.556	Promoter(<=1kb)	351	OR10G2	26534
454638	14q	70457520	70458540	1021	14	0.429	Exon(exon2of2)	5346	ADAM21	8747
454638	14q	94587512	94587839	328	6	0.5	Exon(exon2of2)	-4219	SERPINA3	12
454638	14q	104947943	104951938	3996	14	0.5	Exon(exon6of6)	3464	AHNAK2	113146
454638	15q	20534480	20535014	535	6	0.5	Exon(exon8of9)	6786	GOLGA6L6	727832
454638	15q	23439979	23442067	2089	12	0.583	5'UTR	5167	GOLGA6L2	283685
454638	15q	40621642	40623696	2055	6	0.333	Promoter(<=1kb)	0	KNL1	57082
454638	15q	75206207	75207654	1448	6	0.5	Promoter(<=1kb)	158	C15orf39	56905
454638	15q	78766049	78766671	623	7	0.714	Promoter(<=1kb)	-936	ADAMTS7	11173
454638	15q	85579423	85582073	2651	15	0.6	Promoter(<=1kb)	-837	AKAP13	11214
454638	16p	1228744	1229731	988	9	0.667	Promoter(<=1kb)	431	TPSB2	64499
454638	16p	1256345	1256980	636	7	0.857	Promoter(<=1kb)	276	TPSD1	23430
454638	16p	3589138	3590784	1647	6	0.833	Exon(exon12of15)	-11735	NLRC3	197358
454638	16p	4207130	4208004	875	6	0.5	Exon(exon2of7)	31739	SRL	6345
454638	16q	74391460	74392004	545	9	0.667	Exon(exon7of7)	13582	NPIPB15	440348
454638	16q	88428416	88429600	1185	6	0.333	Exon(exon3of3)	-23680	ZFPM1	161882
454638	16q	88713331	88717113	3783	9	0.444	Promoter(<=1kb)	0	MIR4722	100616167
454638	16q	89226863	89228390	1528	9	0.556	Promoter(2-3kb)	2229	ZNF778	197320
454638	17p	744946	746966	2021	6	1	3'UTR	5072	GEMIN4	50628
454638	17p	21300581	21300954	374	8	0.75	3'UTR	9112	MAP2K3	5606
454638	17q	73236508	73236991	484	6	0.333	Promoter(<=1kb)	0	FAM104A	84923
454638	17q	76293419	76294016	598	6	0.5	Promoter(2-3kb)	-2167	QRICH2	84074
454638	17q	81645135	81645607	473	7	0.286	Promoter(2-3kb)	2722	TSPAN10	83882
454638	18p	11609728	11610509	782	10	0.6	Promoter(<=1kb)	132	SLC35G4	646000
454638	18q	58535186	58538030	2845	19	0.579	Promoter(<=1kb)	0	ALPK2	115701
454638	19p	4510548	4513547	3000	18	0.5	Exon(exon3of6)	4157	PLIN4	729359

454638	19p	5455600	5456439	840	8	0.625	Promoter(<=1kb)	183	ZNRF4	148066
454638	19p	8948231	8952171	3941	12	0.5	Exon(exon3of84)	29171	MUC16	94025
454638	19p	8959403	8961248	1846	6	0.667	Exon(exon3of84)	20094	MUC16	94025
454638	19p	12075333	12077046	1714	6	0.167	Promoter(<=1kb)	6	ZNF788P	388507
454638	19p	12430157	12432437	2281	10	0.4	3'UTR	8584	ZNF443	10224
454638	19p	17282085	17284246	2162	8	0.5	Promoter(<=1kb)	0	ANKLE1	126549
454638	19p	18264753	18267409	2657	8	0.5	5'UTR	7002	IQCN	80726
454638	19p	21971930	21974500	2571	7	0.714	Exon(exon4of4)	14408	ZNF208	7757
454638	19p	23743906	23745300	1395	6	0.333	Exon(exon4of4)	13537	ZNF681	148213
454638	19q	39886240	39886422	183	6	0.667	Promoter(<=1kb)	870	FCGBP	8857
454638	19q	43612804	43614201	1398	6	0.5	Promoter(<=1kb)	716	SRRM5	100170229
454638	19q	43913423	43914878	1456	9	0.556	Exon(exon10of10)	4861	ZNF45	7596
454638	19q	44106512	44108078	1567	7	0	Exon(exon6of6)	-4103	ZNF225	7768
454638	19q	52437918	52439242	1325	7	0.429	Exon(exon4of4)	6504	ZNF534	147658
454638	19q	55481625	55483456	1832	6	0.5	Promoter(1-2kb)	-1732	NAT14	57106
454638	19q	55517821	55518189	369	9	1	Exon(exon14of14)	18114	SBK2	646643
454638	19q	55911888	55913077	1190	6	0.667	Exon(exon5of12)	19234	NLRP13	126204
454638	20p	5922421	5923643	1223	9	0.667	Exon(exon4of5)	6923	CHGB	1114
454638	20q	62812284	62813345	1062	7	0.857	Promoter(2-3kb)	-2899	COL9A3	1299
454638	20q	63561666	63564319	2654	9	0.778	Promoter(<=1kb)	-61	HELZ2	85441
454638	21q	26843740	26844859	1120	6	0.667	Promoter(<=1kb)	0	ADAMTS1	9510
454638	21q	44600627	44601692	1066	10	0.7	Promoter(<=1kb)	30	KRTAP10-7	386675
454638	22q	22352950	22353380	431	16	0.5	Exon(exon1of2)	30478	BMS1P20	96610
454638	22q	36191154	36191906	753	6	0.667	3'UTR	9971	APOL4	80832
454638	22q	39100331	39102033	1703	6	0.833	Promoter(<=1kb)	52	APOBEC3H	164668
454638	22q	49884187	49884994	808	6	0.333	Exon(exon2of2)	22924	ALG12	79087
454638	23p	3320126	3323750	3625	9	0.556	Exon(exon5of7)	22902	MXRA5	25878
454638	23p	8170039	8170141	103	6	0.5	Promoter(1-2kb)	1126	VCX2	51480
454638	23p	35802148	35803010	863	7	0.571	5'UTR	3357	MAGEB16	139604
457042	1p	12847526	12847780	255	7	0.429	Promoter(<=1kb)	945	HNRNPCL1	343069
457042	1p	12859036	12860212	1177	14	0.5	Promoter(1-2kb)	1950	PRAMEF2	65122
457042	1p	12861255	12861684	430	6	0.167	Exon(exon4of4)	4169	PRAMEF2	65122
457042	1p	12879619	12879853	235	6	0.667	Exon(exon4of4)	6348	PRAMEF4	400735
457042	1p	13370686	13371119	434	7	0.429	Promoter(<=1kb)	781	PRAMEF19	645414
457042	1p	16058491	16058689	199	6	1	Exon(exon7of7)	6168	CLCNKB	1188
457042	1p	18481042	18482217	1176	12	0.75	Promoter(<=1kb)	60	KLHDC7A	127707
457042	1p	23874604	23875430	827	9	0.556	Exon(exon2of2)	-6310	FUCA1	2517
457042	1p	40067594	40067675	82	6	0	Promoter(<=1kb)	324	CAP1	10487
457042	1p	109251229	109252986	1758	6	0.5	Promoter(1-2kb)	1690	CELSR2	1952
457042	1q	152219233	152221375	2143	16	0.75	Promoter(2-3kb)	2818	HRNR	388697
457042	1q	158765805	158766655	851	6	0.5	Promoter(<=1kb)	47	OR6N1	128372
457042	1q	169541142	169542882	1741	9	0.222	Exon(exon13of25)	-25397	F5	2153
457042	1q	228315976	228319055	3080	8	0.5	Exon(exon50of81)	6492	OBSCN	84033
457042	1q	228371172	228372999	1828	8	0.375	Promoter(<=1kb)	0	OBSCN	84033
457042	1q	247841312	247841582	271	6	0.833	Promoter(<=1kb)	314	OR11L1	391189
457042	1q	247895625	247896410	786	6	0.5	Promoter(<=1kb)	38	OR2W3	343171
457042	1q	247949436	247949759	324	9	0.333	Promoter(<=1kb)	578	OR2L8	391190
457042	2p	48580657	48582454	1798	8	0.625	Promoter(<=1kb)	0	STON1	11037
457042	2q	130914528	130917154	2627	7	0.429	Promoter(<=1kb)	0	ARHGEF4	50649
457042	2q	217847583	217848559	977	6	0.833	Exon(exon19of33)	-5423	TNS1	7145
457042	2q	219271337	219271649	313	6	0.667	Exon(exon4of4)	6227	TUBA4A	7277
457042	2q	237762685	237764060	1376	10	0.5	Exon(exon8of8)	-4137	LRRFIP1	9208
457042	2q	240041845	240042154	310	6	0.167	Downstream(2-3kb)	3918	OR6B3	150681
457042	3p	75736880	75739243	2364	51	0.627	Promoter(<=1kb)	0	MIR4273	100422955
457042	3q	98264413	98265098	686	7	0.571	Promoter(<=1kb)	128	OR5H6	79295
457042	3q	194341097	194342571	1475	7	0.714	Exon(exon2of2)	8747	CPN2	1370
457042	3q	194359607	194360963	1357	10	0.9	Exon(exon2of2)	-8279	CPN2	1370
457042	4p	5988383	5989749	1367	7	0.571	Promoter(<=1kb)	0	C4orf50	389197
457042	4p	6300792	6302360	1569	7	0.857	Exon(exon8of8)	6021	WFS1	7466
457042	4p	8227004	8228508	1505	9	0.222	Promoter(<=1kb)	-24	SH3TC1	54436
457042	4q	154489498	154491312	1815	9	0.556	Promoter(<=1kb)	22	DCHS2	54798
457042	4q	186706638	186709436	2799	7	0.714	Exon(exon2of27)	14397	FAT1	2195
457042	5q	79731782	79734523	2742	13	0.308	Exon(exon2of13)	-3619	CMYA5	202333
457042	5q	140848579	140850786	2208	6	0.5	Promoter(<=1kb)	807	PCDHA9	9752
457042	5q	141174000	141175025	1026	6	0.833	Promoter(1-2kb)	1356	PCDHB7	56129
457042	5q	148826877	148828070	1194	6	1	Promoter(1-2kb)	1632	ADRB2	154
457042	5q	151521550	151522069	520	6	0.667	Promoter(<=1kb)	79	MIR6499	102465246
457042	5q	151565922	151568190	2269	10	0.8	Promoter(<=1kb)	754	FAT2	2196
457042	6p	1312843	1313745	903	6	0.5	Promoter(<=1kb)	745	FOXQ1	94234
457042	6p	46858771	46859389	619	6	0.5	Exon(exon17of21)	3915	ADGRF5	221395
457042	6q	149888581	149889987	1407	6	0.667	Promoter(<=1kb)	-116	RAET1E-AS1	100652739
457042	6q	159231899	159234370	2472	10	0.5	Exon(exon11of23)	13602	FNDC1	84624
457042	7p	12369637	12370736	1100	6	0.667	3'UTR	-13307	VWDE	221806
457042	7q	64991278	64992758	1481	6	0.667	Promoter(<=1kb)	-242	ZNF117	51351
457042	7q	100952225	100953156	932	6	0.833	Promoter(2-3kb)	2600	MUC3A	4584
457042	7q	100958977	100960873	1897	52	0.442	Promoter(1-2kb)	1012	MUC3A	4584
457042	7q	100995547	100996058	512	7	0.857	Exon(exon5of15)	-16996	MUC12	10071

457042	8p	10609614	10612307	2694	10	0.9	Exon(exon4of4)	42836	RP1L1	94137
457042	8p	11331234	11332082	849	6	0.667	Promoter(<=1kb)	346	SLC35G5	83650
457042	8q	141218050	141219792	1743	6	0.667	5'UTR	8778	SLC45A4	57210
457042	9q	76705179	76707804	2626	8	0.5	Promoter(<=1kb)	121	PCA3	50652
457042	9q	76709263	76710843	1581	9	0.556	Promoter(<=1kb)	0	PRUNE2	158471
457042	9q	87885490	87888819	3330	8	0.625	Promoter(2-3kb)	2613	SPATA31E1	286234
457042	9q	104598545	104599361	817	12	0.583	Promoter(<=1kb)	52	OR13C5	138799
457042	9q	122553263	122554071	809	8	0.5	Promoter(<=1kb)	93	OR1N2	138882
457042	9q	122628595	122629398	804	7	0.286	Promoter(<=1kb)	175	OR1B1	347169
457042	9q	122749914	122750547	634	6	0.833	Promoter(<=1kb)	174	OR1L6	392390
457042	9q	135484803	135487213	2411	8	0.25	Promoter(1-2kb)	1440	PPP1R26	9858
457042	10q	46549378	46550723	1346	25	0.64	Exon(exon3of3)	4807	GPRIN2	9721
457042	10q	49323169	49326817	3649	15	0.533	Exon(exon3of3)	23895	C10orf71	118461
457042	10q	128103129	128108204	5076	15	0.667	Promoter(<=1kb)	-1	MKI67	4288
457042	11p	244106	244197	92	8	0.5	Promoter(<=1kb)	-232	PSMD13	5719
457042	11p	5046754	5047432	679	7	0.571	Promoter(<=1kb)	228	OR52J3	119679
457042	11p	5177978	5178478	501	6	0.167	Promoter(<=1kb)	186	OR52Z1	283110
457042	11p	5323451	5324256	806	6	0.5	Promoter(<=1kb)	41	OR51B2	79345
457042	11p	5389704	5390350	647	7	0.429	Promoter(<=1kb)	327	OR51M1	390059
457042	11p	5422212	5423123	912	10	0.7	Promoter(<=1kb)	101	OR51Q1	390061
457042	11p	5515185	5515931	747	6	0.333	Promoter(<=1kb)	768	UBQLNL	143630
457042	11p	5581045	5581738	694	8	0.375	Promoter(<=1kb)	168	OR52B6	340980
457042	11p	5841302	5841883	582	9	0.333	Promoter(<=1kb)	14	OR52E6	390078
457042	11p	5884818	5885061	244	7	0.429	Promoter(<=1kb)	547	OR52E4	390081
457042	11p	11351961	11352736	776	9	0.222	Promoter(<=1kb)	514	CSNK2A3	283106
457042	11p	12293639	12294842	1204	7	0.571	Exon(exon29of35)	6739	MICALCL	84953
457042	11q	58214757	58215722	966	6	0.333	Promoter(<=1kb)	12	OR1S1	219959
457042	11q	82732630	82733184	555	6	0.833	Promoter(<=1kb)	680	FAM181B	220382
457042	11q	85724687	85725825	1139	6	0.5	Promoter(<=1kb)	0	SYTL2	54843
457042	11q	123906510	123907324	815	7	0.714	Promoter(<=1kb)	364	OR8D4	338662
457042	11q	124038366	124038988	623	7	1	Promoter(<=1kb)	13	OR10G7	390265
457042	11q	124382526	124383285	760	8	0.625	Promoter(<=1kb)	58	OR8B2	26595
457042	12p	4626571	4628549	1979	9	0.444	Exon(exon5of6)	14054	DYRK4	8798
457042	12p	6453119	6453670	552	6	0.667	Promoter(<=1kb)	633	TAPBPL	55080
457042	13q	24434450	24435347	898	7	0.571	Exon(exon31of34)	19787	PARP4	143
457042	13q	25096713	25097076	364	9	0.444	Promoter(<=1kb)	845	PABPC3	5042
457042	13q	102732474	102733933	1460	6	0.333	Exon(exon4of4)	25139	CCDC168	643677
457042	14q	20060048	20060884	837	8	0.625	Promoter(<=1kb)	3	OR4L1	122742
457042	14q	23079970	23080576	607	6	0.5	Promoter(<=1kb)	-377	ACIN1	22985
457042	14q	70457532	70458540	1009	10	0.4	Exon(exon2of2)	5358	ADAM21	8747
457042	14q	94587512	94587839	328	6	0.5	Exon(exon2of2)	-4219	SERPINA3	12
457042	14q	104939262	104942618	3357	10	0.2	5'UTR	7102	PLD4	122618
457042	14q	104943622	104945444	1823	7	0.571	Exon(exon6of6)	9958	AHNAK2	113146
457042	14q	104947943	104953878	5936	41	0.512	Promoter(1-2kb)	1524	AHNAK2	113146
457042	15q	23440160	23442067	1908	10	0.6	5'UTR	5167	GOLGA6L2	283685
457042	15q	40621642	40623696	2055	6	0.333	Promoter(<=1kb)	0	KNL1	57082
457042	15q	85579423	85581800	2378	14	0.571	Promoter(1-2kb)	-1110	AKAP13	11214
457042	16p	1228744	1229622	879	7	0.714	Promoter(<=1kb)	540	TPSB2	64499
457042	16p	1486322	1488463	2142	13	0.692	Promoter(<=1kb)	4	PTX4	390667
457042	16p	4207059	4208004	946	7	0.571	Exon(exon2of7)	31739	SRL	6345
457042	16q	88428339	88429600	1262	6	0.333	Exon(exon3of3)	-23680	ZFPM1	161882
457042	16q	88712207	88714717	2511	7	0.571	Promoter(<=1kb)	0	CTU2	348180
457042	16q	89100686	89101050	365	7	0.571	Promoter(<=1kb)	24	ACSF3	197322
457042	16q	89226863	89228390	1528	9	0.556	Promoter(2-3kb)	2229	ZNF778	197320
457042	17p	2299649	2300159	511	6	0.167	Exon(exon2of19)	-3224	SRR	63826
457042	17p	5558351	5559959	1609	6	0.833	Exon(exon4of17)	23998	NLRP1	22861
457042	17p	10638198	10641099	2902	7	0.286	Exon(exon19of41)	-8169	MYH3	4621
457042	17p	21300581	21300978	398	12	0.75	3'UTR	9112	MAP2K3	5606
457042	17q	76291123	76294016	2894	11	0.545	Promoter(<=1kb)	0	QRICH2	84074
457042	17q	81645135	81645417	283	6	0.333	Promoter(2-3kb)	2722	TSPAN10	83882
457042	18p	11609646	11610350	705	11	0.818	Promoter(<=1kb)	50	SLC35G4	646000
457042	18q	47033929	47035145	1217	6	0.833	Promoter(<=1kb)	476	ELOA2	51224
457042	18q	58535186	58537515	2330	9	0.333	Promoter(<=1kb)	0	ALPK2	115701
457042	19p	4510548	4513547	3000	21	0.429	Exon(exon3of6)	4157	PLIN4	729359
457042	19p	5455600	5456439	840	7	0.571	Promoter(<=1kb)	183	ZNRF4	148066
457042	19p	5866643	5867737	1095	6	0.5	Promoter(2-3kb)	2803	FUT5	2527
457042	19p	8937644	8939234	1591	6	0.833	Exon(exon5of84)	-41554	MUC16	94025
457042	19p	8946306	8951868	5563	19	0.684	Exon(exon3of84)	29474	MUC16	94025
457042	19p	8959116	8962299	3184	12	0.583	Exon(exon3of84)	19043	MUC16	94025
457042	19p	8963397	8967127	3731	21	0.667	Exon(exon3of84)	14215	MUC16	94025
457042	19p	8972448	8978096	5649	15	0.533	Exon(exon1of84)	3246	MUC16	94025
457042	19p	12430157	12432437	2281	9	0.333	3'UTR	8584	ZNF443	10224
457042	19p	17281820	17284246	2427	9	0.556	Promoter(<=1kb)	0	ANKLE1	126549
457042	19p	18264798	18267409	2612	12	0.583	5'UTR	7002	IQCN	80726
457042	19p	21971930	21974500	2571	10	0.7	Exon(exon4of4)	14408	ZNF208	7757
457042	19p	23743906	23745300	1395	6	0.333	Exon(exon4of4)	13537	ZNF681	148213
457042	19q	39886240	39886422	183	6	0.667	Promoter(<=1kb)	870	FCGBP	8857

457042	19q	43913423	43914878	1456	9	0.556	Exon(exon10of10)	4861	ZNF45	7596
457042	19q	43996326	43997366	1041	6	0.5	Exon(exon5of5)	5419	LOC10192806:	101928063
457042	19q	44106512	44108078	1567	7	0	Exon(exon6of6)	-4103	ZNF225	7768
457042	19q	52437918	52439242	1325	7	0.429	Exon(exon4of4)	6504	ZNF534	147658
457042	19q	55481625	55483456	1832	7	0.429	Promoter(1-2kb)	-1732	NAT14	57106
457042	19q	55911888	55913077	1190	6	0.667	Exon(exon5of12)	19234	NLRP13	126204
457042	19q	58368293	58368875	583	7	0.429	Exon(exon3of3)	-5445	ZNF497	162968
457042	20p	5922421	5923394	974	9	0.556	Exon(exon4of5)	6923	CHGB	1114
457042	20q	63349752	63350772	1021	6	0.5	3'UTR	3794	CHRNA4	1137
457042	20q	63561666	63565531	3866	13	0.615	Promoter(<=1kb)	-61	HELZ2	85441
457042	21q	26843740	26844859	1120	6	0.667	Promoter(<=1kb)	0	ADAMTS1	9510
457042	21q	44600627	44601692	1066	10	0.7	Promoter(<=1kb)	30	KRTAP10-7	386675
457042	22q	22352950	22353291	342	12	0.5	Exon(exon1of2)	30478	BMS1P20	96610
457042	22q	36265284	36265796	513	6	1	Exon(exon6of6)	12120	APOL1	8542
457042	23p	3320126	3323750	3625	9	0.556	Exon(exon5of7)	22902	MXRA5	25878
457042	23p	8170039	8170141	103	6	0.5	Promoter(1-2kb)	1126	VCX2	51480
463774	1p	12847526	12847995	470	7	0.429	Promoter(<=1kb)	730	HNRNPCL1	343069
463774	1p	12859036	12860212	1177	13	0.538	Promoter(1-2kb)	1950	PRAMEF2	65122
463774	1p	12893249	12893472	224	6	0.5	Exon(exon4of4)	4798	PRAMEF10	343071
463774	1p	13370686	13370989	304	8	0.5	Promoter(<=1kb)	911	PRAMEF19	645414
463774	1p	18481403	18482217	815	7	0.714	Promoter(<=1kb)	421	KLHDC7A	127707
463774	1p	40067594	40067675	82	6	0	Promoter(<=1kb)	324	CAP1	10487
463774	1q	152218469	152221375	2907	14	0.643	Promoter(2-3kb)	2818	HRNR	388697
463774	1q	152302822	152304920	2099	9	0.444	Exon(exon3of3)	-7656	FLG-AS1	339400
463774	1q	152307444	152313891	6448	23	0.609	Promoter(<=1kb)	0	FLG-AS1	339400
463774	1q	169540901	169542882	1982	9	0.333	Exon(exon13of25)	-25156	F5	2153
463774	1q	201206099	201209738	3640	11	0.636	Promoter(1-2kb)	1017	IGFN1	91156
463774	1q	201210956	201212866	1911	6	0.5	Promoter(<=1kb)	0	IGFN1	91156
463774	1q	226735683	226737239	1557	7	0.857	Promoter(<=1kb)	219	ITPKB	3707
463774	1q	228315976	228318038	2063	9	0.667	Exon(exon50of81)	6492	OBSCN	84033
463774	1q	236553587	236555893	2307	6	0.333	Exon(exon39of45)	10291	LGALS8	3964
463774	1q	247841312	247841582	271	6	0.833	Promoter(<=1kb)	314	OR11L1	391189
463774	1q	247949325	247949738	414	10	0.3	Promoter(<=1kb)	467	OR2L8	391190
463774	2p	48580657	48582454	1798	7	0.571	Promoter(<=1kb)	0	STON1	11037
463774	2q	132783032	132785012	1981	7	0.571	Promoter(1-2kb)	-1009	NCKAP5	344148
463774	2q	178739433	178741811	2379	6	0.5	Exon(exon45of191)	26014	TTN	7273
463774	2q	184936178	184937636	1459	6	0.333	Exon(exon4of4)	69813	ZNF804A	91752
463774	2q	185789865	185794632	4768	10	0.8	Promoter(<=1kb)	0	FSIP2	401024
463774	2q	185805377	185808170	2794	6	0.333	Promoter(<=1kb)	0	FSIP2	401024
463774	2q	238130271	238131546	1276	7	0.286	Promoter(1-2kb)	1323	ESPNL	339768
463774	2q	240041845	240042811	967	6	0.333	Downstream(2-3kb)	3261	OR6B3	150681
463774	3p	75736880	75739007	2128	28	0.5	Promoter(<=1kb)	0	MIR4273	100422955
463774	3q	158732176	158732509	334	6	0.5	Promoter(<=1kb)	0	RARRES1	5918
463774	4p	5988383	5989749	1367	7	0.429	Promoter(<=1kb)	0	C4orf50	389197
463774	4p	6300792	6302360	1569	6	0.833	Exon(exon8of8)	6021	WFS1	7466
463774	4p	8227004	8228508	1505	8	0.125	Promoter(<=1kb)	-24	SH3TC1	54436
463774	4q	185458217	185460011	1795	9	0.556	Promoter(<=1kb)	0	CCDC110	256309
463774	4q	186619481	186621601	2121	8	0.25	Exon(exon10of27)	-9411	FAT1	2195
463774	5q	79731782	79734523	2742	14	0.357	Exon(exon2of13)	-3619	CMYA5	202333
463774	5q	83537326	83539905	2580	6	0.333	Promoter(1-2kb)	1712	VCAN	1462
463774	5q	140848579	140850786	2208	8	0.5	Promoter(<=1kb)	807	PCDHA9	9752
463774	5q	141100771	141102728	1958	6	0.167	Promoter(<=1kb)	298	PCDHB3	56132
463774	5q	141955356	141957660	2305	7	0.714	Promoter(<=1kb)	-668	RNF14	9604
463774	5q	151565922	151568158	2237	9	0.778	Promoter(<=1kb)	786	FAT2	2196
463774	6p	1312843	1313745	903	6	0.5	Promoter(<=1kb)	745	FOXQ1	94234
463774	6p	46858771	46859502	732	8	0.5	Exon(exon17of21)	3802	ADGRF5	221395
463774	6q	64591274	64591961	688	10	0.5	Exon(exon26of43)	121374	EYS	346007
463774	6q	149888581	149889987	1407	6	0.667	Promoter(<=1kb)	-116	RAET1E-AS1	100652739
463774	6q	159233455	159234370	916	10	0.5	Exon(exon11of23)	15158	FNDC1	84624
463774	7p	6330446	6330944	499	6	1	Exon(exon2of2)	7749	FAM220A	84792
463774	7p	56021087	56021209	123	6	0.5	Exon(exon2of7)	12947	PSPH	5723
463774	7q	100958721	100960873	2153	58	0.431	Promoter(<=1kb)	756	MUC3A	4584
463774	7q	101034305	101038481	4177	25	0.56	Exon(exon3of12)	-5230	MUC17	140453
463774	7q	129126736	129127452	717	6	0.333	Exon(exon1of2)	-17255	TSPAN33	340348
463774	8p	10609614	10612307	2694	9	0.889	Exon(exon4of4)	42836	RP1L1	94137
463774	8p	11331194	11332082	889	8	0.625	Promoter(<=1kb)	306	SLC35G5	83650
463774	8p	13021128	13022030	903	7	0.143	Exon(exon5of5)	9115	TRMT9B	57604
463774	8q	123651873	123652634	762	6	0.333	Promoter(<=1kb)	316	KLHL38	340359
463774	8q	138151949	138153046	1098	6	0.667	Promoter(<=1kb)	0	FAM135B	51059
463774	8q	141466429	141467514	1086	9	0.778	3'UTR	29245	MROH5	389690
463774	8q	143916360	143919209	2850	7	0.143	Exon(exon32of32)	20381	PLEC	5339
463774	9q	76705724	76707804	2081	6	0.5	Promoter(<=1kb)	666	PCA3	50652
463774	9q	104504315	104505071	757	6	0.5	Promoter(<=1kb)	52	OR13F1	138805
463774	9q	122553263	122554071	809	8	0.5	Promoter(<=1kb)	93	OR1N2	138882
463774	9q	122628595	122629130	536	6	0.333	Promoter(<=1kb)	443	OR1B1	347169
463774	9q	135484803	135487213	2411	8	0.25	Promoter(1-2kb)	1440	PPP1R26	9858
463774	10q	46549378	46550723	1346	26	0.654	Exon(exon3of3)	4807	GPRIN2	9721

463774	10q	128102802	128104752	1951	10	0.5	Promoter(<=1kb)	0	MKI67	4288
463774	11p	244106	244197	92	8	0.5	Promoter(<=1kb)	-232	PSMD13	5719
463774	11p	5177978	5178478	501	6	0.167	Promoter(<=1kb)	186	OR52Z1	283110
463774	11p	5323451	5324256	806	6	0.5	Promoter(<=1kb)	41	OR51B2	79345
463774	11p	5422212	5423123	912	12	0.667	Promoter(<=1kb)	101	OR51Q1	390061
463774	11p	5440761	5441472	712	6	0.833	Promoter(<=1kb)	42	OR51I1	390063
463774	11p	5515185	5515931	747	6	0.333	Promoter(<=1kb)	768	UBQLNL	143630
463774	11p	5581045	5581738	694	8	0.375	Promoter(<=1kb)	168	OR52B6	340980
463774	11p	5884818	5885061	244	6	0.333	Promoter(<=1kb)	547	OR52E4	390081
463774	11p	5986042	5986542	501	6	1	Promoter(<=1kb)	443	OR52L1	338751
463774	11p	11352040	11352736	697	8	0.25	Promoter(<=1kb)	514	CSNK2A3	283106
463774	11p	12293639	12294842	1204	7	0.571	Exon(exon29of35)	6739	MICALCL	84953
463774	11q	55572176	55572903	728	6	0.667	Promoter(<=1kb)	48	OR4C16	219428
463774	11q	58214757	58215722	966	8	0.25	Promoter(<=1kb)	12	OR1S1	219959
463774	11q	123906790	123907324	535	6	0.667	Promoter(<=1kb)	644	OR8D4	338662
463774	11q	124038366	124038988	623	6	1	Promoter(<=1kb)	13	OR10G7	390265
463774	12p	4627329	4628549	1221	8	0.625	Exon(exon5of6)	14812	DYRK4	8798
463774	12p	6453119	6453670	552	6	0.667	Promoter(<=1kb)	633	TAPBPL	55080
463774	13q	24434450	24435347	898	7	0.571	Exon(exon31of34)	19787	PARP4	143
463774	13q	24446725	24447185	461	6	0.333	Exon(exon26of34)	7949	PARP4	143
463774	13q	25096213	25097182	970	9	0.444	Promoter(<=1kb)	345	PABPC3	5042
463774	13q	102732474	102733933	1460	6	0.333	Exon(exon4of4)	25139	CCDC168	643677
463774	14q	19975713	19976448	736	9	0.444	Promoter(<=1kb)	269	OR4K15	81127
463774	14q	20060048	20060884	837	9	0.667	Promoter(<=1kb)	3	OR4L1	122742
463774	14q	21634177	21634589	413	6	0.833	Promoter(<=1kb)	351	OR10G2	26534
463774	14q	70457784	70458444	661	9	0.333	Exon(exon2of2)	5610	ADAM21	8747
463774	15q	23440196	23442067	1872	11	0.545	5'UTR	5167	GOLGA6L2	283685
463774	15q	40621642	40623696	2055	6	0.333	Promoter(<=1kb)	0	KNL1	57082
463774	15q	85579423	85581800	2378	15	0.6	Promoter(1-2kb)	-1110	AKAP13	11214
463774	15q	100569472	100570097	626	7	0.714	Promoter(<=1kb)	534	LINS1	55180
463774	16p	1256354	1256985	632	11	0.636	Promoter(<=1kb)	285	TPSD1	23430
463774	16p	1486371	1488463	2093	8	0.75	Promoter(<=1kb)	4	PTX4	390667
463774	16q	74391416	74391897	482	10	0.8	Exon(exon7of7)	13538	NPIPB15	440348
463774	16q	79211799	79212064	266	6	0.667	3'UTR	15440	WWOX	51741
463774	16q	88428539	88431889	3351	11	0.364	Exon(exon3of3)	-21391	ZFPM1	161882
463774	16q	89100686	89101050	365	8	0.625	Promoter(<=1kb)	24	ACSF3	197322
463774	16q	89226863	89228419	1557	11	0.636	Promoter(2-3kb)	2229	ZNF778	197320
463774	17p	10638198	10641099	2902	6	0.333	Exon(exon19of41)	-8169	MYH3	4621
463774	17p	21300581	21300954	374	8	0.75	3'UTR	9112	MAP2K3	5606
463774	17p	21415458	21416431	974	11	0.818	Exon(exon3of3)	10322	KCNJ12	3768
463774	17q	76293419	76294016	598	6	0.5	Promoter(2-3kb)	-2167	QRICH2	84074
463774	17q	81645135	81645607	473	7	0.286	Promoter(2-3kb)	2722	TSPAN10	83882
463774	18p	11609646	11610350	705	11	0.818	Promoter(<=1kb)	50	SLC35G4	646000
463774	19p	4510548	4513547	3000	20	0.45	Exon(exon3of6)	4157	PLIN4	729359
463774	19p	5455600	5456439	840	6	0.5	Promoter(<=1kb)	183	ZNRF4	148066
463774	19p	8948231	8953259	5029	16	0.562	Exon(exon3of84)	28083	MUC16	94025
463774	19p	8959403	8962066	2664	8	0.5	Exon(exon3of84)	19276	MUC16	94025
463774	19p	8964274	8967127	2854	22	0.682	Exon(exon3of84)	14215	MUC16	94025
463774	19p	15087451	15087953	503	6	0.333	Promoter(<=1kb)	471	OR1I1	126370
463774	19p	18264753	18267409	2657	8	0.5	5'UTR	7002	IQCN	80726
463774	19p	21971930	21974500	2571	8	0.625	Exon(exon4of4)	14408	ZNF208	7757
463774	19p	22757857	22759533	1677	10	0.5	Exon(exon4of4)	10449	ZNF99	7652
463774	19q	43913423	43914878	1456	8	0.5	Exon(exon10of10)	4861	ZNF45	7596
463774	19q	44106512	44108078	1567	7	0	Exon(exon6of6)	-4103	ZNF225	7768
463774	19q	51745958	51746963	1006	6	0.333	Exon(exon3of3)	3848	FPR1	2357
463774	19q	52437918	52439242	1325	7	0.429	Exon(exon4of4)	6504	ZNF534	147658
463774	19q	55911888	55913077	1190	6	0.667	Exon(exon5of12)	19234	NLRP13	126204
463774	20p	5922421	5923394	974	7	0.571	Exon(exon4of5)	6923	CHGB	1114
463774	20q	53575869	53577159	1291	6	0.833	Promoter(<=1kb)	0	ZNF217	7764
463774	20q	63561666	63565531	3866	12	0.667	Promoter(<=1kb)	-61	HELZ2	85441
463774	22q	22352950	22353348	399	14	0.571	Exon(exon1of2)	30478	BMS1P20	96610
463774	22q	36191154	36191906	753	6	0.667	3'UTR	9971	APOL4	80832
463774	23p	8170039	8170243	205	7	0.571	Promoter(1-2kb)	1024	VCX2	51480
463774	23q	136874183	136874416	234	7	0.857	Promoter(<=1kb)	-201	RBMX	27316
463774	23q	141905911	141906494	584	7	0.571	Promoter(1-2kb)	1109	MAGEC1	9947
464094	1p	12859108	12860212	1105	10	0.5	Promoter(2-3kb)	2022	PRAMEF2	65122
464094	1p	16048038	16049824	1787	6	0.5	Promoter(<=1kb)	0	CLCNKB	1188
464094	1p	23874604	23875430	827	9	0.556	Exon(exon2of2)	-6310	FUCA1	2517
464094	1p	40067594	40067675	82	6	0	Promoter(<=1kb)	324	CAP1	10487
464094	1p	89186388	89186411	24	6	0.667	Promoter(<=1kb)	115	GBP4	115361
464094	1q	152218469	152221375	2907	16	0.688	Promoter(2-3kb)	2818	HRNR	388697
464094	1q	152303673	152304920	1248	8	0.5	Exon(exon3of3)	-7656	FLG-AS1	339400
464094	1q	152307444	152313891	6448	23	0.609	Promoter(<=1kb)	0	FLG-AS1	339400
464094	1q	156906733	156909788	3056	6	0.5	Promoter(<=1kb)	-169	PEAR1	375033
464094	1q	158765805	158766655	851	6	0.5	Promoter(<=1kb)	47	OR6N1	128372
464094	1q	169540901	169542882	1982	9	0.333	Exon(exon13of25)	-25156	F5	2153
464094	1q	228315976	228318038	2063	9	0.667	Exon(exon50of81)	6492	OBSCN	84033

464094	1q	247841312	247841582	271	6	0.833	Promoter(<=1kb)	314	OR11L1	391189
464094	1q	247896121	247896502	382	6	0.5	Promoter(<=1kb)	534	OR2W3	343171
464094	1q	247949443	247949738	296	9	0.333	Promoter(<=1kb)	585	OR2L8	391190
464094	1q	248681658	248682198	541	7	0.571	Promoter(<=1kb)	130	OR14I1	401994
464094	2p	48580657	48582454	1798	7	0.571	Promoter(<=1kb)	0	STON1	11037
464094	2q	102351547	102351902	356	7	0.429	Exon(exon11of11)	-4027	IL18R1	8809
464094	2q	132781078	132784972	3895	10	0.6	Promoter(<=1kb)	0	NCKAP5	344148
464094	2q	184936178	184937636	1459	9	0.333	Exon(exon4of4)	69813	ZNF804A	91752
464094	2q	185789577	185794632	5056	11	0.727	Promoter(<=1kb)	0	FSIP2	401024
464094	2q	185805377	185808891	3515	7	0.429	Promoter(<=1kb)	0	FSIP2	401024
464094	2q	217847567	217848559	993	7	0.857	Exon(exon19of33)	-5407	TNS1	7145
464094	2q	233713134	233713783	650	13	0.692	Promoter(<=1kb)	142	UGT1A5	54579
464094	2q	235041233	235041640	408	6	0.333	Exon(exon4of6)	46393	SH3BP4	23677
464094	2q	238130416	238131546	1131	6	0.333	Promoter(1-2kb)	1468	ESPNL	339768
464094	3p	31989532	31990905	1374	6	0.333	Exon(exon2of2)	7761	ZNF860	344787
464094	3p	75736880	75739243	2364	41	0.61	Promoter(<=1kb)	0	MIR4273	100422955
464094	3q	98264413	98265098	686	6	0.5	Promoter(<=1kb)	128	OR5H6	79295
464094	4p	5988383	5989749	1367	7	0.571	Promoter(<=1kb)	0	C4orf50	389197
464094	4p	6300792	6302360	1569	6	0.833	Exon(exon8of8)	6021	WFS1	7466
464094	4p	8227004	8228508	1505	7	0.143	Promoter(<=1kb)	-24	SH3TC1	54436
464094	4p	10443803	10446224	2422	7	0.429	Exon(exon3of3)	10952	ZNF518B	85460
464094	4q	186619481	186621582	2102	6	0.333	Exon(exon10of27)	-9411	FAT1	2195
464094	4q	186706638	186708616	1979	6	0.667	Exon(exon2of27)	15217	FAT1	2195
464094	5q	83537326	83539905	2580	6	0.333	Promoter(1-2kb)	1712	VCAN	1462
464094	5q	140848579	140850786	2208	6	0.5	Promoter(<=1kb)	807	PCDHA9	9752
464094	5q	141955356	141957660	2305	6	0.667	Promoter(<=1kb)	-668	RNF14	9604
464094	5q	148826877	148828070	1194	6	1	Promoter(1-2kb)	1632	ADRB2	154
464094	5q	151565922	151568158	2237	9	0.778	Promoter(<=1kb)	786	FAT2	2196
464094	6p	26370344	26370479	136	6	0.5	Promoter(<=1kb)	0	BTN3A2	11118
464094	6p	46858771	46859502	732	7	0.571	Exon(exon17of21)	3802	ADGRF5	221395
464094	6q	149888581	149890867	2287	7	0.714	Promoter(<=1kb)	0	RAET1E-AS1	100652739
464094	6q	159231899	159234370	2472	10	0.5	Exon(exon11of23)	13602	FNDC1	84624
464094	7p	6330446	6330944	499	6	1	Exon(exon2of2)	7749	FAM220A	84792
464094	7p	38353718	38353991	274	9	0.667	Exon(exon2of2)	3699	TRG-AS1	100506776
464094	7p	53035678	53036385	708	7	1	Promoter(<=1kb)	45	POM121L12	285877
464094	7p	56021087	56021209	123	6	0.5	Exon(exon2of7)	12947	PSPH	5723
464094	7q	100958721	100960873	2153	55	0.455	Promoter(<=1kb)	756	MUC3A	4584
464094	7q	100991195	100992398	1204	7	0.571	Exon(exon5of15)	-20656	MUC12	10071
464094	7q	149818015	149819792	1778	6	0.667	Promoter(2-3kb)	-2352	SSPO	23145
464094	8p	10606895	10608432	1538	9	0.556	Exon(exon4of4)	46711	RP1L1	94137
464094	8p	10609614	10610662	1049	8	0.5	Exon(exon4of4)	44481	RP1L1	94137
464094	8p	13021128	13022030	903	8	0.25	Exon(exon5of5)	9115	TRMT9B	57604
464094	8q	123651873	123652634	762	6	0.333	Promoter(<=1kb)	316	KLHL38	340359
464094	8q	141218050	141219792	1743	6	0.667	5'UTR	8778	SLC45A4	57210
464094	9q	76703555	76707804	4250	13	0.538	Promoter(<=1kb)	0	PCA3	50652
464094	9q	76709523	76710843	1321	6	0.5	Promoter(<=1kb)	0	PRUNE2	158471
464094	9q	122553263	122554071	809	7	0.429	Promoter(<=1kb)	93	OR1N2	138882
464094	9q	122628595	122629398	804	6	0.333	Promoter(<=1kb)	175	OR1B1	347169
464094	9q	131474871	131475956	1086	6	0.833	Promoter(<=1kb)	0	PRRC2B	84726
464094	9q	135484803	135487213	2411	8	0.25	Promoter(1-2kb)	1440	PPP1R26	9858
464094	9q	135547960	135548795	836	6	0.667	Promoter(1-2kb)	1805	OBP2A	29991
464094	10q	46549378	46550744	1367	26	0.615	5'UTR	4786	GPRIN2	9721
464094	10q	49322575	49326331	3757	10	0.5	Exon(exon3of3)	23301	C10orf71	118461
464094	10q	89737450	89738561	1112	6	0	Exon(exon20of33)	13874	KIF20B	9585
464094	10q	125026129	125027175	1047	7	0.571	Promoter(<=1kb)	853	CTBP2	1488
464094	11p	244106	244197	92	8	0.5	Promoter(<=1kb)	-232	PSMD13	5719
464094	11p	5177978	5178478	501	6	0.167	Promoter(<=1kb)	186	OR52Z1	283110
464094	11p	5323542	5324256	715	6	0.5	Promoter(<=1kb)	41	OR51B2	79345
464094	11p	5351521	5352416	896	16	0.5	Promoter(<=1kb)	13	OR51B6	390058
464094	11p	5389704	5390350	647	7	0.429	Promoter(<=1kb)	327	OR51M1	390059
464094	11p	5422212	5423123	912	11	0.727	Promoter(<=1kb)	101	OR51Q1	390061
464094	11p	5581045	5581738	694	8	0.375	Promoter(<=1kb)	168	OR52B6	340980
464094	11p	5841302	5841883	582	9	0.333	Promoter(<=1kb)	14	OR52E6	390078
464094	11p	5884818	5885061	244	7	0.429	Promoter(<=1kb)	547	OR52E4	390081
464094	11p	5986042	5986669	628	7	1	Promoter(<=1kb)	316	OR52L1	338751
464094	11p	11352040	11352736	697	8	0.25	Promoter(<=1kb)	514	CSNK2A3	283106
464094	11q	55572176	55572903	728	6	0.667	Promoter(<=1kb)	48	OR4C16	219428
464094	11q	58214757	58215722	966	7	0.286	Promoter(<=1kb)	12	OR1S1	219959
464094	11q	69295669	69296300	632	6	0.667	Promoter(1-2kb)	1495	MYEOV	26579
464094	11q	82732630	82733184	555	6	0.833	Promoter(<=1kb)	680	FAM181B	220382
464094	11q	85724687	85725825	1139	6	0.5	Promoter(<=1kb)	0	SYTL2	54843
464094	11q	123906790	123907324	535	6	0.667	Promoter(<=1kb)	644	OR8D4	338662
464094	11q	124015600	124016477	878	7	0.143	Promoter(<=1kb)	25	OR10G4	390264
464094	11q	124038366	124038988	623	8	1	Promoter(<=1kb)	13	OR10G7	390265
464094	11q	124264847	124265766	920	7	0.714	Promoter(<=1kb)	20	OR8G5	219865
464094	12p	4626571	4628549	1979	8	0.5	Exon(exon5of6)	14054	DYRK4	8798
464094	12p	6453119	6453670	552	6	0.667	Promoter(<=1kb)	633	TAPBPL	55080

464094	12q	52316096	52317765	1670	6	0.667	Exon(exon4of9)	3633	KRT83	3889
464094	13q	24446725	24447185	461	6	0.333	Exon(exon26of34)	7949	PARP4	143
464094	13q	102732474	102733933	1460	6	0.333	Exon(exon4of4)	25139	CCDC168	643677
464094	14q	20060048	20060884	837	8	0.625	Promoter(<=1kb)	3	OR4L1	122742
464094	14q	70457520	70459101	1582	7	0.429	Exon(exon2of2)	5346	ADAM21	8747
464094	14q	94587512	94587839	328	6	0.5	Exon(exon2of2)	-4219	SERPINA3	12
464094	14q	104175455	104177762	2308	9	0.333	Exon(exon12of15)	36415	KIF26A	26153
464094	14q	104939262	104942618	3357	12	0.167	5'UTR	7102	PLD4	122618
464094	14q	104943622	104946886	3265	17	0.647	Exon(exon6of6)	8516	AHNAK2	113146
464094	14q	104947901	104949883	1983	14	0.643	Exon(exon6of6)	5519	AHNAK2	113146
464094	14q	104951557	104953878	2322	11	0.455	Promoter(1-2kb)	1524	AHNAK2	113146
464094	15q	23440370	23442067	1698	9	0.667	5'UTR	5167	GOLGA6L2	283685
464094	15q	85579423	85582073	2651	16	0.562	Promoter(<=1kb)	-837	AKAP13	11214
464094	15q	88854874	88855400	527	6	0.833	Exon(exon12of18)	7631	ACAN	176
464094	15q	88857108	88859365	2258	6	0	Exon(exon12of18)	9865	ACAN	176
464094	16p	376432	377820	1389	6	0.5	Promoter(<=1kb)	0	PGAP6	58986
464094	16p	1221890	1223490	1601	7	1	Promoter(1-2kb)	1285	TPSG1	25823
464094	16p	1256354	1256985	632	11	0.545	Promoter(<=1kb)	285	TPSD1	23430
464094	16p	1486371	1488463	2093	8	0.75	Promoter(<=1kb)	4	PTX4	390667
464094	16p	4207130	4208004	875	6	0.5	Exon(exon2of7)	31739	SRL	6345
464094	16q	74391650	74391897	248	9	0.778	Exon(exon7of7)	13772	NPIPB15	440348
464094	16q	89100686	89101050	365	9	0.667	Promoter(<=1kb)	24	ACSF3	197322
464094	16q	89227206	89228419	1214	8	0.625	Promoter(2-3kb)	2572	ZNF778	197320
464094	17p	744946	746966	2021	6	1	3'UTR	5072	GEMIN4	50628
464094	17p	7482135	7482961	827	8	0.75	Promoter(<=1kb)	803	SLC35G6	643664
464094	17p	21300581	21300954	374	8	0.75	3'UTR	9112	MAP2K3	5606
464094	17p	21415458	21416431	974	10	0.8	Exon(exon3of3)	10322	KCNJ12	3768
464094	17q	76293419	76294016	598	6	0.5	Promoter(2-3kb)	-2167	QRICH2	84074
464094	17q	81645135	81645607	473	7	0.286	Promoter(2-3kb)	2722	TSPAN10	83882
464094	18p	11609646	11610333	688	11	0.818	Promoter(<=1kb)	50	SLC35G4	646000
464094	18q	47033121	47035145	2025	8	0.75	Promoter(<=1kb)	476	ELOA2	51224
464094	18q	58535186	58538030	2845	19	0.526	Promoter(<=1kb)	0	ALPK2	115701
464094	19p	4510548	4513547	3000	19	0.474	Exon(exon3of6)	4157	PLIN4	729359
464094	19p	5455600	5456439	840	8	0.625	Promoter(<=1kb)	183	ZNRF4	148066
464094	19p	8937644	8939234	1591	6	0.833	Exon(exon5of84)	-41554	MUC16	94025
464094	19p	8946306	8951868	5563	17	0.647	Exon(exon3of84)	29474	MUC16	94025
464094	19p	8959116	8962299	3184	10	0.7	Exon(exon3of84)	19043	MUC16	94025
464094	19p	8972751	8978096	5346	16	0.5	Exon(exon1of84)	3246	MUC16	94025
464094	19p	12430400	12432437	2038	6	0.5	3'UTR	8584	ZNF443	10224
464094	19p	15087213	15088040	828	9	0.333	Promoter(<=1kb)	233	OR1I1	126370
464094	19p	18264798	18267409	2612	12	0.583	5'UTR	7002	IQCN	80726
464094	19p	22757857	22759533	1677	10	0.5	Exon(exon4of4)	10449	ZNF99	7652
464094	19q	37886924	37889059	2136	6	0.833	Exon(exon6of6)	17535	WDR87	83889
464094	19q	39877222	39877880	659	6	0.5	Exon(exon20of28)	9412	FCGBP	8857
464094	19q	39886005	39886439	435	8	0.5	Promoter(<=1kb)	853	FCGBP	8857
464094	19q	43846955	43848536	1582	6	0.833	3'UTR	13450	ZNF283	284349
464094	19q	43913423	43914878	1456	7	0.429	Exon(exon10of10)	4861	ZNF45	7596
464094	19q	44106512	44108078	1567	9	0.111	Exon(exon6of6)	-4103	ZNF225	7768
464094	19q	44327836	44329698	1863	6	0.5	Exon(exon4of4)	-22790	ZNF235	9310
464094	19q	52437918	52439242	1325	7	0.429	Exon(exon4of4)	6504	ZNF534	147658
464094	19q	55481625	55483456	1832	6	0.5	Promoter(1-2kb)	-1732	NAT14	57106
464094	19q	55911888	55913077	1190	6	0.667	Exon(exon5of12)	19234	NLRP13	126204
464094	21q	26843740	26844859	1120	6	0.667	Promoter(<=1kb)	0	ADAMTS1	9510
464094	21q	41740862	41741990	1129	7	0.857	Exon(exon2of2)	4851	MIR6814	102465488
464094	21q	44539312	44540109	798	6	1	Promoter(<=1kb)	86	KRTAP10-1	386677
464094	21q	44600627	44601692	1066	10	0.7	Promoter(<=1kb)	30	KRTAP10-7	386675
464094	21q	44637474	44638143	670	10	0.4	Promoter(<=1kb)	118	KRTAP10-10	353333
464094	21q	46125432	46126068	637	6	1	Exon(exon25of28)	4872	COL6A2	1292
464094	22q	17817474	17818927	1454	6	0.667	Promoter(<=1kb)	0	MICAL3	57553
464094	22q	22352950	22353348	399	14	0.571	Exon(exon1of2)	30478	BMS1P20	96610
464094	22q	35658986	35659117	132	6	0.833	Exon(exon3of3)	10540	APOL6	80830
464094	23p	8170039	8170141	103	6	0.5	Promoter(1-2kb)	1126	VCX2	51480
464094	23p	35802148	35803010	863	7	0.571	5'UTR	3357	MAGEB16	139604
464094	23q	141905856	141906385	530	7	0.714	Promoter(1-2kb)	1054	MAGEC1	9947
464651	1p	12847526	12847995	470	7	0.429	Promoter(<=1kb)	730	HNRNPCL1	343069
464651	1p	12859036	12860079	1044	6	0.333	Promoter(1-2kb)	1950	PRAMEF2	65122
464651	1p	13370686	13370989	304	8	0.5	Promoter(<=1kb)	911	PRAMEF19	645414
464651	1p	16058491	16059952	1462	10	0.8	Exon(exon5of7)	6168	CLCNKB	1188
464651	1p	18481403	18482217	815	6	0.667	Promoter(<=1kb)	421	KLHDC7A	127707
464651	1p	23874604	23875430	827	9	0.556	Exon(exon2of2)	-6310	FUCA1	2517
464651	1p	40067594	40067675	82	6	0	Promoter(<=1kb)	324	CAP1	10487
464651	1q	152218469	152221375	2907	16	0.688	Promoter(2-3kb)	2818	HRNR	388697
464651	1q	152303673	152304920	1248	7	0.429	Exon(exon3of3)	-7656	FLG-AS1	339400
464651	1q	152306380	152313891	7512	31	0.581	Promoter(<=1kb)	0	FLG-AS1	339400
464651	1q	158765805	158766655	851	6	0.5	Promoter(<=1kb)	47	OR6N1	128372
464651	1q	201206099	201209342	3244	10	0.6	Promoter(1-2kb)	1017	IGFN1	91156
464651	1q	201210956	201212792	1837	7	0.429	Promoter(<=1kb)	0	IGFN1	91156

464651	1q	247841312	247841582	271	6	0.833	Promoter(<=1kb)	314	OR11L1	391189
464651	1q	247895950	247896410	461	6	0.5	Promoter(<=1kb)	363	OR2W3	343171
464651	1q	247949325	247949738	414	9	0.222	Promoter(<=1kb)	467	OR2L8	391190
464651	1q	248573992	248574210	219	6	0.833	Promoter(<=1kb)	547	OR2T34	127068
464651	1q	248681658	248682198	541	7	0.571	Promoter(<=1kb)	130	OR14I1	401994
464651	2p	48580657	48582454	1798	7	0.571	Promoter(<=1kb)	0	STON1	11037
464651	2q	167246794	167248478	1685	7	0.714	Promoter(<=1kb)	-204	XIRP2	129446
464651	2q	178739433	178741811	2379	6	0.5	Exon(exon45of191)	26014	TTN	7273
464651	2q	185790999	185794632	3634	8	0.875	Promoter(<=1kb)	0	FSIP2	401024
464651	2q	185805377	185807185	1809	6	0.5	Promoter(<=1kb)	0	FSIP2	401024
464651	2q	238130271	238131546	1276	7	0.286	Promoter(1-2kb)	1323	ESPNL	339768
464651	3p	75736929	75738859	1931	10	0.6	Promoter(<=1kb)	0	MIR4273	100422955
464651	4p	5988383	5989749	1367	8	0.625	Promoter(<=1kb)	0	C4orf50	389197
464651	4p	6300792	6302360	1569	6	0.833	Exon(exon8of8)	6021	WFS1	7466
464651	4p	8227004	8228508	1505	8	0.125	Promoter(<=1kb)	-24	SH3TC1	54436
464651	4q	121036404	121037542	1139	6	0.333	Promoter(1-2kb)	1442	NDNF	79625
464651	4q	154489498	154491312	1815	9	0.556	Promoter(<=1kb)	22	DCHS2	54798
464651	4q	185458217	185460011	1795	8	0.625	Promoter(<=1kb)	0	CCDC110	256309
464651	4q	186619481	186621601	2121	7	0.286	Exon(exon10of27)	-9411	FAT1	2195
464651	5p	795818	796237	420	6	0.5	3'UTR	4908	ZDHHC11	79844
464651	5q	83537326	83539905	2580	6	0.333	Promoter(1-2kb)	1712	VCAN	1462
464651	5q	140848579	140850786	2208	7	0.429	Promoter(<=1kb)	807	PCDHA9	9752
464651	5q	141174000	141175025	1026	6	0.833	Promoter(1-2kb)	1356	PCDHB7	56129
464651	5q	151521550	151522069	520	6	0.667	Promoter(<=1kb)	79	MIR6499	102465246
464651	5q	151565922	151568158	2237	9	0.778	Promoter(<=1kb)	786	FAT2	2196
464651	6p	1312843	1313745	903	6	0.5	Promoter(<=1kb)	745	FOXQ1	94234
464651	6p	46858771	46859502	732	8	0.5	Exon(exon17of21)	3802	ADGRF5	221395
464651	6q	149888581	149890867	2287	7	0.714	Promoter(<=1kb)	0	RAET1E-AS1	100652739
464651	6q	159231899	159234370	2472	10	0.5	Exon(exon11of23)	13602	FNDC1	84624
464651	7p	12369637	12370736	1100	6	0.667	3'UTR	-13307	VWDE	221806
464651	7q	100958721	100960873	2153	58	0.431	Promoter(<=1kb)	756	MUC3A	4584
464651	7q	100991195	100992866	1672	10	0.6	Exon(exon5of15)	-20188	MUC12	10071
464651	8p	10607245	10612307	5063	15	0.667	Exon(exon4of4)	42836	RP1L1	94137
464651	8p	11331194	11332082	889	8	0.625	Promoter(<=1kb)	306	SLC35G5	83650
464651	8p	12132686	12133940	1255	6	0.667	Promoter(<=1kb)	498	USP17L7	392197
464651	8p	13021128	13022030	903	9	0.111	Exon(exon5of5)	9115	TRMT9B	57604
464651	8q	123651655	123652634	980	7	0.571	Promoter(<=1kb)	316	KLHL38	340359
464651	8q	138151949	138153046	1098	6	0.667	Promoter(<=1kb)	0	FAM135B	51059
464651	9q	76703451	76707804	4354	14	0.429	Promoter(<=1kb)	0	PCA3	50652
464651	9q	76709263	76710843	1581	8	0.5	Promoter(<=1kb)	0	PRUNE2	158471
464651	9q	122553278	122554071	794	6	0.333	Promoter(<=1kb)	108	OR1N2	138882
464651	9q	122628595	122629398	804	7	0.286	Promoter(<=1kb)	175	OR1B1	347169
464651	9q	135484803	135487213	2411	8	0.25	Promoter(1-2kb)	1440	PPP1R26	9858
464651	10q	46549378	46550723	1346	25	0.64	Exon(exon3of3)	4807	GPRIN2	9721
464651	10q	122084988	122087840	2853	8	0.75	Exon(exon4of23)	-25190	TACC2	10579
464651	10q	128102802	128104752	1951	10	0.5	Promoter(<=1kb)	0	MKI67	4288
464651	11p	244106	244197	92	8	0.5	Promoter(<=1kb)	-232	PSMD13	5719
464651	11p	5177978	5178478	501	6	0.167	Promoter(<=1kb)	186	OR52Z1	283110
464651	11p	5323542	5324256	715	6	0.5	Promoter(<=1kb)	41	OR51B2	79345
464651	11p	5389704	5390350	647	7	0.429	Promoter(<=1kb)	327	OR51M1	390059
464651	11p	5422212	5423123	912	11	0.636	Promoter(<=1kb)	101	OR51Q1	390061
464651	11p	5581045	5581738	694	8	0.375	Promoter(<=1kb)	168	OR52B6	340980
464651	11p	5841302	5841883	582	9	0.333	Promoter(<=1kb)	14	OR52E6	390078
464651	11p	6319295	6320454	1160	6	0.667	Promoter(<=1kb)	0	CAVIN3	112464
464651	11p	11352040	11352736	697	8	0.25	Promoter(<=1kb)	514	CSNK2A3	283106
464651	11p	12293639	12294368	730	7	0.857	Exon(exon29of35)	6739	MICALCL	84953
464651	11q	58214757	58215722	966	7	0.286	Promoter(<=1kb)	12	OR1S1	219959
464651	11q	64315818	64315856	39	6	0.833	Promoter(1-2kb)	1485	TRMT112	51504
464651	11q	124015601	124016477	877	6	0.167	Promoter(<=1kb)	26	OR10G4	390264
464651	11q	124023038	124023849	812	7	0.429	Promoter(<=1kb)	25	OR10G9	219870
464651	11q	124382526	124383285	760	6	0.5	Promoter(<=1kb)	58	OR8B2	26595
464651	12p	4626571	4628549	1979	9	0.444	Exon(exon5of6)	14054	DYRK4	8798
464651	12p	6453119	6453670	552	6	0.667	Promoter(<=1kb)	633	TAPBPL	55080
464651	12q	48525773	48526223	451	6	0.5	Promoter(<=1kb)	141	OR8S1	341568
464651	12q	52316096	52317765	1670	6	0.667	Exon(exon4of9)	3633	KRT83	3889
464651	13q	25096713	25097231	519	9	0.444	Promoter(<=1kb)	845	PABPC3	5042
464651	13q	102732474	102733933	1460	6	0.333	Exon(exon4of4)	25139	CCDC168	643677
464651	14q	20224029	20224484	456	6	0.5	Promoter(<=1kb)	319	OR11H6	122748
464651	14q	21634177	21634589	413	6	0.833	Promoter(<=1kb)	351	OR10G2	26534
464651	14q	70457532	70458238	707	9	0.444	Exon(exon2of2)	5358	ADAM21	8747
464651	14q	104175275	104177810	2536	7	0.429	Exon(exon12of15)	36235	KIF26A	26153
464651	14q	104939262	104942618	3357	11	0.273	5'UTR	7102	PLD4	122618
464651	14q	104943622	104945444	1823	7	0.714	Exon(exon6of6)	9958	AHNAK2	113146
464651	14q	104946886	104949673	2788	8	0.625	Exon(exon6of6)	5729	AHNAK2	113146
464651	15q	23440160	23442067	1908	10	0.6	5'UTR	5167	GOLGA6L2	283685
464651	15q	78766033	78766626	594	6	0.333	Promoter(<=1kb)	-920	ADAMTS7	11173
464651	15q	88857108	88859365	2258	6	0	Exon(exon12of18)	9865	ACAN	176

464651	15q	101066056	101067308	1253	6	0.667	Promoter(1-2kb)	1640	LRRK1	79705
464651	16p	669592	672548	2957	7	0.571	Promoter(<=1kb)	0	RHOT2	89941
464651	16p	1229573	1229716	144	7	0.571	Promoter(<=1kb)	446	TPSB2	64499
464651	16p	1256345	1256985	641	14	0.714	Promoter(<=1kb)	276	TPSD1	23430
464651	16q	74391549	74391897	349	9	0.778	Exon(exon7of7)	13671	NPIPB15	440348
464651	16q	88428339	88429600	1262	7	0.429	Exon(exon3of3)	-23680	ZFPM1	161882
464651	16q	89226863	89228419	1557	10	0.6	Promoter(2-3kb)	2229	ZNF778	197320
464651	17p	744946	746966	2021	6	1	3'UTR	5072	GEMIN4	50628
464651	17p	10638198	10641099	2902	6	0.333	Exon(exon19of41)	-8169	MYH3	4621
464651	17p	21300581	21300978	398	11	0.727	3'UTR	9112	MAP2K3	5606
464651	17p	21415458	21416431	974	16	0.875	Exon(exon3of3)	10322	KCNJ12	3768
464651	17q	76293419	76294016	598	6	0.5	Promoter(2-3kb)	-2167	QRICH2	84074
464651	17q	81645135	81645417	283	6	0.333	Promoter(2-3kb)	2722	TSPAN10	83882
464651	18p	11609646	11610350	705	7	0.857	Promoter(<=1kb)	50	SLC35G4	646000
464651	18q	58535186	58537515	2330	9	0.333	Promoter(<=1kb)	0	ALPK2	115701
464651	19p	1004711	1005532	822	7	0.571	Exon(exon3of9)	4292	GRIN3B	116444
464651	19p	1036457	1036914	458	6	0.5	Exon(exon6of7)	-3187	ABCA7	10347
464651	19p	4510548	4513547	3000	19	0.526	Exon(exon3of6)	4157	PLIN4	729359
464651	19p	5455600	5456439	840	8	0.625	Promoter(<=1kb)	183	ZNRF4	148066
464651	19p	8937644	8939234	1591	6	0.667	Exon(exon5of84)	-41554	MUC16	94025
464651	19p	8946313	8952171	5859	20	0.6	Exon(exon3of84)	29171	MUC16	94025
464651	19p	8959116	8962299	3184	12	0.75	Exon(exon3of84)	19043	MUC16	94025
464651	19p	8971838	8978096	6259	15	0.533	Exon(exon1of84)	3246	MUC16	94025
464651	19p	18264753	18267409	2657	8	0.5	5'UTR	7002	IQCN	80726
464651	19p	21971930	21974500	2571	7	0.714	Exon(exon4of4)	14408	ZNF208	7757
464651	19p	23743906	23745300	1395	6	0.333	Exon(exon4of4)	13537	ZNF681	148213
464651	19q	37886924	37889059	2136	6	0.833	Exon(exon6of6)	17535	WDR87	83889
464651	19q	39886005	39886260	256	6	0.667	Promoter(1-2kb)	1032	FCGBP	8857
464651	19q	43913423	43914878	1456	8	0.5	Exon(exon10of10)	4861	ZNF45	7596
464651	19q	44106512	44108078	1567	7	0	Exon(exon6of6)	-4103	ZNF225	7768
464651	19q	52437918	52439242	1325	7	0.429	Exon(exon4of4)	6504	ZNF534	147658
464651	19q	52840798	52842068	1271	7	0.286	3'UTR	7457	ZNF468	90333
464651	19q	55481625	55483456	1832	6	0.5	Promoter(1-2kb)	-1732	NAT14	57106
464651	20p	5922421	5923394	974	6	0.5	Exon(exon4of5)	6923	CHGB	1114
464651	20q	63349752	63350772	1021	6	0.5	3'UTR	3794	CHRNA4	1137
464651	20q	63540866	63543452	2587	10	0.8	Exon(exon1of1)	-3490	PTK6	5753
464651	20q	63561666	63565531	3866	13	0.615	Promoter(<=1kb)	-61	HELZ2	85441
464651	21q	26843740	26844859	1120	6	0.667	Promoter(<=1kb)	0	ADAMTS1	9510
464651	21q	44539312	44540035	724	6	0.833	Promoter(<=1kb)	160	KRTAP10-1	386677
464651	21q	44550835	44551416	582	6	0.833	Promoter(<=1kb)	89	KRTAP10-2	386679
464651	21q	44637476	44638143	668	9	0.444	Promoter(<=1kb)	120	KRTAP10-10	353333
464651	22q	22352950	22353298	349	13	0.538	Exon(exon1of2)	30478	BMS1P20	96610
464651	22q	36191154	36191906	753	6	0.667	3'UTR	9971	APOL4	80832
464651	23p	8170039	8170141	103	6	0.5	Promoter(1-2kb)	1126	VCX2	51480
464651	23p	35802148	35803010	863	7	0.571	5'UTR	3357	MAGEB16	139604
464651	23q	136874183	136874416	234	8	0.75	Promoter(<=1kb)	-201	RBMX	27316
465576	1p	978953	979884	932	6	0.5	Promoter(1-2kb)	1145	PERM1	84808
465576	1p	16058491	16059952	1462	7	0.857	Exon(exon5of7)	6168	CLCNKB	1188
465576	1p	18481403	18483159	1757	7	0.714	Promoter(<=1kb)	421	KLHDC7A	127707
465576	1p	40067594	40067675	82	6	0	Promoter(<=1kb)	324	CAP1	10487
465576	1p	88983230	88983751	522	7	0.429	Exon(exon3of3)	4604	KYAT3	56267
465576	1p	89186388	89186419	32	9	0.556	Promoter(<=1kb)	107	GBP4	115361
465576	1q	152303673	152304920	1248	7	0.429	Exon(exon3of3)	-7656	FLG-AS1	339400
465576	1q	152306380	152313891	7512	30	0.567	Promoter(<=1kb)	0	FLG-AS1	339400
465576	1q	158765805	158766655	851	6	0.5	Promoter(<=1kb)	47	OR6N1	128372
465576	1q	201206099	201209738	3640	11	0.545	Promoter(1-2kb)	1017	IGFN1	91156
465576	1q	214640144	214642954	2811	12	0.5	Exon(exon12of20)	-5013	CENPF	1063
465576	1q	247841312	247841582	271	6	0.833	Promoter(<=1kb)	314	OR11L1	391189
465576	1q	247896121	247896502	382	6	0.5	Promoter(<=1kb)	534	OR2W3	343171
465576	1q	247949325	247949738	414	10	0.3	Promoter(<=1kb)	467	OR2L8	391190
465576	2p	48580657	48582454	1798	7	0.571	Promoter(<=1kb)	0	STON1	11037
465576	2q	184936178	184937636	1459	9	0.333	Exon(exon4of4)	69813	ZNF804A	91752
465576	2q	185789865	185794632	4768	10	0.8	Promoter(<=1kb)	0	FSIP2	401024
465576	2q	217847583	217848559	977	6	0.833	Exon(exon19of33)	-5423	TNS1	7145
465576	2q	238130236	238131546	1311	8	0.375	Promoter(1-2kb)	1288	ESPNL	339768
465576	3p	75736929	75738859	1931	10	0.6	Promoter(<=1kb)	0	MIR4273	100422955
465576	3q	98264413	98265098	686	6	0.5	Promoter(<=1kb)	128	OR5H6	79295
465576	4p	5988383	5989749	1367	8	0.5	Promoter(<=1kb)	0	C4orf50	389197
465576	4p	6300792	6302360	1569	6	0.833	Exon(exon8of8)	6021	WFS1	7466
465576	4q	185458217	185460011	1795	8	0.625	Promoter(<=1kb)	0	CCDC110	256309
465576	4q	186619481	186621582	2102	7	0.286	Exon(exon10of27)	-9411	FAT1	2195
465576	5p	795818	796237	420	6	0.5	3'UTR	4908	ZDHHC11	79844
465576	5q	79728956	79730716	1761	7	0.286	Exon(exon2of13)	-7426	CMYA5	202333
465576	5q	79731782	79734523	2742	14	0.357	Exon(exon2of13)	-3619	CMYA5	202333
465576	5q	83537326	83539905	2580	6	0.333	Promoter(1-2kb)	1712	VCAN	1462
465576	5q	140848579	140850786	2208	10	0.6	Promoter(<=1kb)	807	PCDHA9	9752
465576	5q	141955676	141957660	1985	6	0.667	Promoter(<=1kb)	-668	RNF14	9604

465576	5q	148826877	148828070	1194	6	1	Promoter(1-2kb)	1632	ADRB2	154
465576	5q	151565922	151568158	2237	9	0.778	Promoter(<=1kb)	786	FAT2	2196
465576	6p	26370344	26370479	136	6	0.5	Promoter(<=1kb)	0	BTN3A2	11118
465576	6p	46858771	46859502	732	7	0.571	Exon(exon17of21)	3802	ADGRF5	221395
465576	6q	149888581	149890867	2287	7	0.714	Promoter(<=1kb)	0	RAET1E-AS1	100652739
465576	6q	159231899	159234370	2472	10	0.5	Exon(exon11of23)	13602	FNDC1	84624
465576	7p	45082725	45084866	2142	7	0.429	Promoter(1-2kb)	-1325	NACAD	23148
465576	7p	53035678	53036385	708	7	1	Promoter(<=1kb)	45	POM121L12	285877
465576	7q	100958721	100960873	2153	56	0.464	Promoter(<=1kb)	756	MUC3A	4584
465576	7q	101034305	101038481	4177	23	0.522	Exon(exon3of12)	-5230	MUC17	140453
465576	8p	8376561	8377994	1434	6	1	Exon(exon2of5)	3753	PRAG1	157285
465576	8p	10607375	10608261	887	6	0.5	Exon(exon4of4)	46882	RP1L1	94137
465576	8p	11331194	11332082	889	8	0.625	Promoter(<=1kb)	306	SLC35G5	83650
465576	8q	123651655	123652634	980	7	0.571	Promoter(<=1kb)	316	KLHL38	340359
465576	8q	138151949	138153046	1098	6	0.667	Promoter(<=1kb)	0	FAM135B	51059
465576	9q	76703451	76707804	4354	14	0.429	Promoter(<=1kb)	0	PCA3	50652
465576	9q	76709263	76710843	1581	8	0.5	Promoter(<=1kb)	0	PRUNE2	158471
465576	9q	87886533	87888536	2004	7	0.571	Exon(exon4of4)	3656	SPATA31E1	286234
465576	9q	104598545	104599361	817	6	0.333	Promoter(<=1kb)	52	OR13C5	138799
465576	9q	122553263	122554071	809	8	0.5	Promoter(<=1kb)	93	OR1N2	138882
465576	9q	131474936	131475956	1021	6	0.833	Promoter(<=1kb)	0	PRRC2B	84726
465576	9q	133255635	133256205	571	7	1	3'UTR	19009	ABO	28
465576	9q	135484803	135487213	2411	9	0.333	Promoter(1-2kb)	1440	PPP1R26	9858
465576	10q	46549378	46550723	1346	25	0.64	Exon(exon3of3)	4807	GPRIN2	9721
465576	10q	128102594	128106296	3703	10	0.7	Promoter(<=1kb)	0	MKI67	4288
465576	11p	244106	244197	92	8	0.5	Promoter(<=1kb)	-232	PSMD13	5719
465576	11p	1194354	1196902	2549	7	0.571	Exon(exon34of49)	-26164	MUC5B	727897
465576	11p	1241677	1243593	1917	6	0.333	Exon(exon31of49)	6083	MUC5B-AS1	112577518
465576	11p	1250091	1251628	1538	8	0.75	Promoter(<=1kb)	-415	MUC5B-AS1	112577518
465576	11p	5177978	5178528	551	7	0.143	Promoter(<=1kb)	136	OR52Z1	283110
465576	11p	5323362	5324256	895	7	0.429	Promoter(<=1kb)	41	OR51B2	79345
465576	11p	5389704	5390350	647	7	0.429	Promoter(<=1kb)	327	OR51M1	390059
465576	11p	5422212	5423123	912	11	0.636	Promoter(<=1kb)	101	OR51Q1	390061
465576	11p	5515185	5516015	831	6	0.333	Promoter(<=1kb)	684	UBQLNL	143630
465576	11p	5581045	5581738	694	8	0.375	Promoter(<=1kb)	168	OR52B6	340980
465576	11p	5788000	5788760	761	8	0.75	Promoter(<=1kb)	56	OR52N1	79473
465576	11p	5841302	5841883	582	9	0.333	Promoter(<=1kb)	14	OR52E6	390078
465576	11p	11352040	11352736	697	8	0.25	Promoter(<=1kb)	514	CSNK2A3	283106
465576	11p	12293639	12294538	900	6	0.667	Exon(exon29of35)	6739	MICALCL	84953
465576	11q	55827536	55827640	105	6	0.667	Promoter(<=1kb)	317	OR5L2	26338
465576	11q	58214757	58215722	966	7	0.286	Promoter(<=1kb)	12	OR1S1	219959
465576	11q	85724687	85725825	1139	6	0.5	Promoter(<=1kb)	0	SYTL2	54843
465576	11q	123906790	123907324	535	6	0.667	Promoter(<=1kb)	644	OR8D4	338662
465576	11q	124038366	124038988	623	8	1	Promoter(<=1kb)	13	OR10G7	390265
465576	11q	124382526	124383285	760	6	0.5	Promoter(<=1kb)	58	OR8B2	26595
465576	12p	4626568	4628549	1982	11	0.455	Exon(exon5of6)	14051	DYRK4	8798
465576	12p	6453119	6453670	552	6	0.667	Promoter(<=1kb)	633	TAPBPL	55080
465576	12q	52316096	52317765	1670	6	0.667	Exon(exon4of9)	3633	KRT83	3889
465576	13q	24434450	24435347	898	6	0.5	Exon(exon31of34)	19787	PARP4	143
465576	13q	102731670	102733933	2264	7	0.286	Exon(exon4of4)	25139	CCDC168	643677
465576	14q	19975713	19976448	736	9	0.444	Promoter(<=1kb)	269	OR4K15	81127
465576	14q	20060048	20060884	837	8	0.625	Promoter(<=1kb)	3	OR4L1	122742
465576	14q	20640982	20641567	586	6	0.5	Promoter(<=1kb)	124	OR6S1	341799
465576	14q	22633879	22634450	572	8	0.375	Exon(exon2of2)	32212	ABHD4	63874
465576	14q	70457532	70457980	449	7	0.429	Exon(exon2of2)	5358	ADAM21	8747
465576	14q	104175275	104177810	2536	6	0.333	Exon(exon12of15)	36235	KIF26A	26153
465576	14q	104949015	104950976	1962	6	0.5	Exon(exon6of6)	4426	AHNAK2	113146
465576	15q	23440196	23442067	1872	10	0.6	5'UTR	5167	GOLGA6L2	283685
465576	15q	40621642	40624434	2793	8	0.375	Promoter(<=1kb)	0	KNL1	57082
465576	15q	85579423	85581800	2378	14	0.571	Promoter(1-2kb)	-1110	AKAP13	11214
465576	16p	1256354	1256985	632	10	0.6	Promoter(<=1kb)	285	TPSD1	23430
465576	16p	1486371	1488463	2093	8	0.75	Promoter(<=1kb)	4	PTX4	390667
465576	16q	74391650	74391897	248	9	0.778	Exon(exon7of7)	13772	NPIPB15	440348
465576	16q	89226863	89228289	1427	7	0.571	Promoter(2-3kb)	2229	ZNF778	197320
465576	17p	10638198	10641099	2902	7	0.286	Exon(exon19of41)	-8169	MYH3	4621
465576	17p	21300581	21300954	374	8	0.75	3'UTR	9112	MAP2K3	5606
465576	17p	21415458	21416416	959	10	0.9	Exon(exon3of3)	10322	KCNJ12	3768
465576	17q	76293419	76294016	598	6	0.5	Promoter(2-3kb)	-2167	QRICH2	84074
465576	17q	81645135	81645607	473	7	0.286	Promoter(2-3kb)	2722	TSPAN10	83882
465576	18p	11609646	11610164	519	6	0.833	Promoter(<=1kb)	50	SLC35G4	646000
465576	18q	58535186	58537515	2330	9	0.333	Promoter(<=1kb)	0	ALPK2	115701
465576	19p	4510548	4513547	3000	19	0.421	Exon(exon3of6)	4157	PLIN4	729359
465576	19p	5455600	5456439	840	6	0.5	Promoter(<=1kb)	183	ZNRF4	148066
465576	19p	8948231	8953259	5029	16	0.562	Exon(exon3of84)	28083	MUC16	94025
465576	19p	8959403	8962066	2664	8	0.5	Exon(exon3of84)	19276	MUC16	94025
465576	19p	8964274	8967127	2854	22	0.682	Exon(exon3of84)	14215	MUC16	94025
465576	19p	12075333	12077046	1714	6	0.167	Promoter(<=1kb)	6	ZNF788P	388507

465576	19p	12430400	12432437	2038	8	0.5	3'UTR	8584	ZNF443	10224
465576	19p	17281820	17284246	2427	9	0.556	Promoter(<=1kb)	0	ANKLE1	126549
465576	19p	18264798	18267409	2612	12	0.583	5'UTR	7002	IQCN	80726
465576	19p	21971930	21974500	2571	10	0.7	Exon(exon4of4)	14408	ZNF208	7757
465576	19q	43846955	43848536	1582	6	0.833	3'UTR	13450	ZNF283	284349
465576	19q	43913423	43914878	1456	8	0.5	Exon(exon10of10)	4861	ZNF45	7596
465576	19q	44106512	44108078	1567	8	0.125	Exon(exon6of6)	-4103	ZNF225	7768
465576	19q	52437918	52439242	1325	7	0.429	Exon(exon4of4)	6504	ZNF534	147658
465576	19q	53490416	53491750	1335	6	0.5	Exon(exon4of4)	22673	ZNF813	126017
465576	19q	55911888	55913077	1190	6	0.667	Exon(exon5of12)	19234	NLRP13	126204
465576	19q	57639918	57641641	1724	7	0.429	3'UTR	6693	ZNF211	10520
465576	20p	5922421	5923394	974	6	0.5	Exon(exon4of5)	6923	CHGB	1114
465576	20q	62332390	62334004	1615	6	0.5	Promoter(<=1kb)	0	MIR4758	100616340
465576	20q	63561666	63565531	3866	12	0.667	Promoter(<=1kb)	-61	HELZ2	85441
465576	21q	44600627	44601692	1066	9	0.778	Promoter(<=1kb)	30	KRTAP10-7	386675
465576	22q	22352950	22353365	416	15	0.533	Exon(exon1of2)	30478	BMS1P20	96610
465576	22q	36191154	36191906	753	6	0.667	3'UTR	9971	APOL4	80832
465576	22q	49883704	49884994	1291	6	0.167	Exon(exon2of2)	22924	ALG12	79087
465576	22q	50503556	50505734	2179	7	0.857	Promoter(<=1kb)	0	LMF2	91289
465576	23p	6533793	6533828	36	6	1	Promoter(1-2kb)	1290	VCX3A	51481
465576	23p	35802148	35803010	863	7	0.571	5'UTR	3357	MAGEB16	139604
465576	23q	136874183	136874347	165	7	0.714	Promoter(<=1kb)	-201	RBMX	27316
466472	1p	11766028	11768307	2280	6	0.833	Promoter(<=1kb)	0	C1orf167	284498
466472	1p	12893249	12893472	224	6	0.5	Exon(exon4of4)	4798	PRAMEF10	343071
466472	1p	13370686	13371119	434	7	0.429	Promoter(<=1kb)	781	PRAMEF19	645414
466472	1p	16058491	16060000	1510	10	0.9	Exon(exon5of7)	6168	CLCNKB	1188
466472	1p	18481403	18482217	815	6	0.667	Promoter(<=1kb)	421	KLHDC7A	127707
466472	1p	23874604	23875430	827	8	0.5	Exon(exon2of2)	-6310	FUCA1	2517
466472	1p	40067594	40067675	82	6	0	Promoter(<=1kb)	324	CAP1	10487
466472	1p	62273232	62275080	1849	7	0.429	Promoter(<=1kb)	-441	KANK4	163782
466472	1p	89186388	89186419	32	9	0.556	Promoter(<=1kb)	107	GBP4	115361
466472	1q	152218469	152221071	2603	7	0.429	Exon(exon3of3)	3122	HRNR	388697
466472	1q	152303673	152313891	10219	40	0.6	Promoter(<=1kb)	0	FLG-AS1	339400
466472	1q	152910134	152911235	1102	6	0.667	Promoter(1-2kb)	1588	IVL	3713
466472	1q	156669844	156670886	1043	6	1	Exon(exon4of4)	6521	NES	10763
466472	1q	158398525	158399274	750	6	0.333	Promoter(<=1kb)	192	OR10T2	128360
466472	1q	158765805	158766655	851	6	0.5	Promoter(<=1kb)	47	OR6N1	128372
466472	1q	169542317	169542882	566	6	0.167	Exon(exon13of25)	-26572	F5	2153
466472	1q	201206099	201209856	3758	13	0.615	Promoter(1-2kb)	1017	IGFN1	91156
466472	1q	214640144	214642954	2811	12	0.5	Exon(exon12of20)	-5013	CENPF	1063
466472	1q	214644872	214647181	2310	10	0.4	Promoter(<=1kb)	-786	CENPF	1063
466472	1q	228315976	228318049	2074	7	0.571	Exon(exon50of81)	6492	OBSCN	84033
466472	1q	247841312	247841582	271	6	0.833	Promoter(<=1kb)	314	OR11L1	391189
466472	1q	247895681	247896502	822	7	0.429	Promoter(<=1kb)	94	OR2W3	343171
466472	1q	247949325	247949738	414	10	0.3	Promoter(<=1kb)	467	OR2L8	391190
466472	2p	48580657	48582454	1798	7	0.571	Promoter(<=1kb)	0	STON1	11037
466472	2q	184936178	184937636	1459	6	0.333	Exon(exon4of4)	69813	ZNF804A	91752
466472	2q	185789865	185794632	4768	10	0.8	Promoter(<=1kb)	0	FSIP2	401024
466472	2q	217847583	217848559	977	6	0.833	Exon(exon19of33)	-5423	TNS1	7145
466472	2q	219271337	219271649	313	6	0.667	Exon(exon4of4)	6227	TUBA4A	7277
466472	2q	237762685	237764060	1376	10	0.5	Exon(exon8of8)	-4137	LRRFIP1	9208
466472	2q	238130271	238131546	1276	7	0.286	Promoter(1-2kb)	1323	ESPNL	339768
466472	3p	31989532	31990905	1374	7	0.286	Exon(exon2of2)	7761	ZNF860	344787
466472	3p	75737230	75739007	1778	9	0.556	Promoter(<=1kb)	0	MIR4273	100422955
466472	3q	98264413	98265098	686	7	0.571	Promoter(<=1kb)	128	OR5H6	79295
466472	3q	196947388	196948102	715	6	0.5	3'UTR	3828	PIGZ	80235
466472	4p	5988383	5989749	1367	7	0.571	Promoter(<=1kb)	0	C4orf50	389197
466472	4p	6300792	6302360	1569	6	0.833	Exon(exon8of8)	6021	WFS1	7466
466472	4q	185458217	185460011	1795	9	0.667	Promoter(<=1kb)	0	CCDC110	256309
466472	5q	67163994	67165736	1743	6	0.5	Exon(exon29of29)	20561	CD180	4064
466472	5q	79728956	79730716	1761	6	0.333	Exon(exon2of13)	-7426	CMYA5	202333
466472	5q	79731782	79734523	2742	13	0.308	Exon(exon2of13)	-3619	CMYA5	202333
466472	5q	83537326	83539905	2580	6	0.333	Promoter(1-2kb)	1712	VCAN	1462
466472	5q	140807352	140807737	386	6	0.833	Promoter(<=1kb)	271	PCDHA4	56144
466472	5q	141174000	141175025	1026	6	0.833	Promoter(1-2kb)	1356	PCDHB7	56129
466472	5q	141183999	141184688	690	6	1	Promoter(2-3kb)	-2473	PCDHB9	56127
466472	5q	141955676	141957660	1985	6	0.667	Promoter(<=1kb)	-668	RNF14	9604
466472	5q	148826877	148828070	1194	6	1	Promoter(1-2kb)	1632	ADRB2	154
466472	6p	46858771	46859389	619	6	0.5	Exon(exon17of21)	3915	ADGRF5	221395
466472	6q	149888581	149890867	2287	7	0.714	Promoter(<=1kb)	0	RAET1E-AS1	100652739
466472	7p	53035732	53036253	522	6	1	Promoter(<=1kb)	99	POM121L12	285877
466472	7p	56021087	56021209	123	6	0.5	Exon(exon2of7)	12947	PSPH	5723
466472	7q	64991278	64992758	1481	6	0.667	Promoter(<=1kb)	-242	ZNF117	51351
466472	7q	100958977	100960873	1897	50	0.46	Promoter(1-2kb)	1012	MUC3A	4584
466472	7q	100991195	100994184	2990	11	0.727	Exon(exon5of15)	-18870	MUC12	10071
466472	7q	100995547	100995785	239	7	0.857	Exon(exon5of15)	-17269	MUC12	10071
466472	7q	101003685	101004836	1152	6	0.667	Exon(exon5of15)	-8218	MUC12	10071

466472	7q	149818015	149819792	1778	6	0.667	Promoter(2-3kb)	-2352	SSPO	23145
466472	8p	10607245	10608432	1188	8	0.5	Exon(exon4of4)	46711	RP1L1	94137
466472	8p	10609614	10610662	1049	8	0.5	Exon(exon4of4)	44481	RP1L1	94137
466472	8p	11331194	11332082	889	9	0.667	Promoter(<=1kb)	306	SLC35G5	83650
466472	8p	12132686	12133940	1255	6	0.667	Promoter(<=1kb)	498	USP17L7	392197
466472	8p	13021128	13022030	903	8	0.125	Exon(exon5of5)	9115	TRMT9B	57604
466472	8p	17754377	17755366	990	6	0.333	Promoter(<=1kb)	0	MTUS1	57509
466472	8q	141466429	141467514	1086	9	0.778	3'UTR	29245	MROH5	389690
466472	8q	143916360	143919209	2850	7	0.143	Exon(exon32of32)	20381	PLEC	5339
466472	8q	143923488	143925516	2029	9	0.667	Exon(exon31of32)	14074	PLEC	5339
466472	9p	116800	117800	1001	8	0.75	Promoter(<=1kb)	404	FOXD4	2298
466472	9q	76175241	76175296	56	8	0.875	Exon(exon14of14)	-13343	PCSK5	5125
466472	9q	76703555	76706360	2806	8	0.5	Promoter(<=1kb)	0	PCA3	50652
466472	9q	87885490	87888819	3330	9	0.667	Promoter(2-3kb)	2613	SPATA31E1	286234
466472	9q	104504315	104505071	757	6	0.5	Promoter(<=1kb)	52	OR13F1	138805
466472	9q	104598545	104599361	817	12	0.583	Promoter(<=1kb)	52	OR13C5	138799
466472	9q	122553263	122554071	809	8	0.5	Promoter(<=1kb)	93	OR1N2	138882
466472	9q	122628595	122629398	804	6	0.333	Promoter(<=1kb)	175	OR1B1	347169
466472	9q	122749914	122750547	634	6	0.833	Promoter(<=1kb)	174	OR1L6	392390
466472	9q	133255635	133256264	630	8	1	3'UTR	18950	ABO	28
466472	9q	135484803	135487213	2411	8	0.25	Promoter(1-2kb)	1440	PPP1R26	9858
466472	9q	135547960	135548795	836	8	0.625	Promoter(1-2kb)	1805	OBP2A	29991
466472	10q	46549378	46550723	1346	27	0.667	Exon(exon3of3)	4807	GPRIN2	9721
466472	10q	49323169	49326817	3649	11	0.545	Exon(exon3of3)	23895	C10orf71	118461
466472	11p	244106	244197	92	8	0.5	Promoter(<=1kb)	-232	PSMD13	5719
466472	11p	1194354	1196902	2549	7	0.571	Exon(exon34of49)	-26164	MUC5B	727897
466472	11p	1246095	1247378	1284	8	0.5	Promoter(2-3kb)	2298	MUC5B-AS1	112577518
466472	11p	5177978	5178478	501	6	0.167	Promoter(<=1kb)	186	OR52Z1	283110
466472	11p	5323542	5324256	715	6	0.5	Promoter(<=1kb)	41	OR51B2	79345
466472	11p	5389704	5390350	647	6	0.5	Promoter(<=1kb)	327	OR51M1	390059
466472	11p	5402638	5403322	685	9	0.444	Promoter(<=1kb)	41	OR51J1	79470
466472	11p	5422212	5423123	912	11	0.636	Promoter(<=1kb)	101	OR51Q1	390061
466472	11p	5515079	5515931	853	7	0.429	Promoter(<=1kb)	768	UBQLNL	143630
466472	11p	5581045	5581738	694	8	0.375	Promoter(<=1kb)	168	OR52B6	340980
466472	11p	5788078	5788516	439	6	0.667	Promoter(<=1kb)	300	OR52N1	79473
466472	11p	5841302	5841883	582	9	0.333	Promoter(<=1kb)	14	OR52E6	390078
466472	11p	5884818	5885061	244	7	0.429	Promoter(<=1kb)	547	OR52E4	390081
466472	11p	11351961	11352736	776	9	0.222	Promoter(<=1kb)	514	CSNK2A3	283106
466472	11p	12293639	12294368	730	7	0.857	Exon(exon29of35)	6739	MICALCL	84953
466472	11q	55572176	55572903	728	6	0.667	Promoter(<=1kb)	48	OR4C16	219428
466472	11q	58214757	58215722	966	8	0.25	Promoter(<=1kb)	12	OR1S1	219959
466472	11q	64116513	64118232	1720	7	0.714	Exon(exon2of2)	8702	MACROD1	28992
466472	11q	85724687	85725825	1139	6	0.5	Promoter(<=1kb)	0	SYTL2	54843
466472	11q	123906790	123907324	535	6	0.667	Promoter(<=1kb)	644	OR8D4	338662
466472	11q	124015601	124016477	877	8	0.25	Promoter(<=1kb)	26	OR10G4	390264
466472	11q	124023038	124023849	812	9	0.444	Promoter(<=1kb)	25	OR10G9	219870
466472	11q	124038366	124038988	623	9	0.889	Promoter(<=1kb)	13	OR10G7	390265
466472	11q	124382526	124383285	760	10	0.6	Promoter(<=1kb)	58	OR8B2	26595
466472	12p	4626568	4628549	1982	11	0.455	Exon(exon5of6)	14051	DYRK4	8798
466472	12p	6018369	6019277	909	6	0.333	Promoter(2-3kb)	2649	VWF	7450
466472	12p	8222174	8223514	1341	6	0.667	Exon(exon5of6)	4073	FAM90A1	55138
466472	13q	102732474	102733933	1460	6	0.333	Exon(exon4of4)	25139	CCDC168	643677
466472	14q	20060048	20060884	837	8	0.625	Promoter(<=1kb)	3	OR4L1	122742
466472	14q	21634137	21634589	453	9	0.556	Promoter(<=1kb)	351	OR10G2	26534
466472	14q	103110058	103110438	381	6	0.667	Promoter(2-3kb)	2436	EXOC3L4	91828
466472	14q	104175455	104177810	2356	9	0.444	Exon(exon12of15)	36415	KIF26A	26153
466472	14q	104939262	104942618	3357	11	0.364	5'UTR	7102	PLD4	122618
466472	14q	104943622	104945444	1823	8	0.625	Exon(exon6of6)	9958	AHNAK2	113146
466472	14q	104946867	104953878	7012	37	0.568	Promoter(1-2kb)	1524	AHNAK2	113146
466472	15q	23363730	23365045	1316	7	0.571	Exon(exon14of18)	8884	GOLGA8S	653061
466472	15q	23439979	23442067	2089	11	0.636	5'UTR	5167	GOLGA6L2	283685
466472	15q	78766215	78766632	418	6	0.5	Promoter(1-2kb)	-1102	ADAMTS7	11173
466472	15q	85579423	85581800	2378	14	0.571	Promoter(1-2kb)	-1110	AKAP13	11214
466472	15q	99130060	99132394	2335	6	0.5	Exon(exon4of5)	7348	TTC23	64927
466472	16p	1241159	1241317	159	6	0.667	Promoter(<=1kb)	0	TPSAB1	7177
466472	16p	1256345	1256816	472	6	0.833	Promoter(<=1kb)	276	TPSD1	23430
466472	16p	1486371	1488463	2093	8	0.75	Promoter(<=1kb)	4	PTX4	390667
466472	16q	88428303	88429600	1298	7	0.429	Exon(exon3of3)	-23680	ZFPM1	161882
466472	16q	89226863	89228289	1427	7	0.571	Promoter(2-3kb)	2229	ZNF778	197320
466472	17p	413503	413831	329	6	0.667	Promoter(<=1kb)	-120	LOC10537143(	105371430
466472	17p	744946	746966	2021	7	1	3'UTR	5072	GEMIN4	50628
466472	17p	2299649	2300159	511	6	0.167	Exon(exon2of19)	-3224	SRR	63826
466472	17p	21300581	21300954	374	9	0.667	3'UTR	9112	MAP2K3	5606
466472	17q	76293419	76294016	598	6	0.5	Promoter(2-3kb)	-2167	QRICH2	84074
466472	17q	81645135	81645617	483	8	0.375	Promoter(2-3kb)	2722	TSPAN10	83882
466472	18p	11609728	11610383	656	10	0.9	Promoter(<=1kb)	132	SLC35G4	646000
466472	18q	58535186	58538030	2845	19	0.526	Promoter(<=1kb)	0	ALPK2	115701

466472	19p	4510548	4511943	1396	13	0.538	Exon(exon3of6)	5761	PLIN4	729359
466472	19p	5455600	5456439	840	8	0.625	Promoter(<=1kb)	183	ZNRF4	148066
466472	19p	8948231	8950136	1906	6	0.667	Exon(exon3of84)	31206	MUC16	94025
466472	19p	8959518	8962066	2549	6	0.333	Exon(exon3of84)	19276	MUC16	94025
466472	19p	8964274	8967127	2854	20	0.6	Exon(exon3of84)	14215	MUC16	94025
466472	19p	8972751	8978096	5346	13	0.462	Exon(exon1of84)	3246	MUC16	94025
466472	19p	18264753	18267409	2657	14	0.643	5'UTR	7002	IQCN	80726
466472	19p	21971930	21974500	2571	6	0.667	Exon(exon4of4)	14408	ZNF208	7757
466472	19p	22756205	22759523	3319	9	0.667	3'UTR	10459	ZNF99	7652
466472	19q	34943334	34944685	1352	6	0.333	3'UTR	9723	ZNF30	90075
466472	19q	36996730	36997597	868	10	0.6	Exon(exon10of10)	5677	ZNF568	374900
466472	19q	43913423	43914878	1456	8	0.5	Exon(exon10of10)	4861	ZNF45	7596
466472	19q	44106512	44108078	1567	7	0	Exon(exon6of6)	-4103	ZNF225	7768
466472	19q	52437918	52439242	1325	8	0.5	Exon(exon4of4)	6504	ZNF534	147658
466472	19q	55358317	55359651	1335	6	0.5	Promoter(<=1kb)	0	FAM71E2	284418
466472	19q	55911888	55913077	1190	6	0.667	Exon(exon5of12)	19234	NLRP13	126204
466472	20p	5922421	5923394	974	6	0.5	Exon(exon4of5)	6923	CHGB	1114
466472	20q	63561666	63565531	3866	12	0.667	Promoter(<=1kb)	-61	HELZ2	85441
466472	22q	22352950	22353298	349	13	0.538	Exon(exon1of2)	30478	BMS1P20	96610
466472	22q	22758758	22759209	452	6	0.833	Exon(exon1of2)	-63567	MIR650	723778
466472	22q	25028581	25029472	892	6	0.333	Promoter(<=1kb)	607	KIAA1671	85379
466472	22q	36191154	36191906	753	6	0.667	3'UTR	9971	APOL4	80832
466472	22q	49884187	49884994	808	6	0.167	Exon(exon2of2)	22924	ALG12	79087
466472	23p	8170039	8170141	103	6	0.5	Promoter(1-2kb)	1126	VCX2	51480
466472	23p	35802148	35803010	863	7	0.571	5'UTR	3357	MAGEB16	139604
467998	1p	978953	980029	1077	6	0.5	Promoter(<=1kb)	1000	PERM1	84808
467998	1p	11766028	11768307	2280	6	0.833	Promoter(<=1kb)	0	C1orf167	284498
467998	1p	11778784	11779941	1158	7	0.714	Promoter(<=1kb)	0	C1orf167-AS1	102724659
467998	1p	16058491	16060000	1510	10	0.9	Exon(exon5of7)	6168	CLCNKB	1188
467998	1p	40067594	40067675	82	6	0	Promoter(<=1kb)	324	CAP1	10487
467998	1p	89186388	89186419	32	9	0.556	Promoter(<=1kb)	107	GBP4	115361
467998	1q	152218730	152221375	2646	16	0.688	Promoter(2-3kb)	2818	HRNR	388697
467998	1q	156669844	156670886	1043	6	1	Exon(exon4of4)	6521	NES	10763
467998	1q	158765805	158766655	851	6	0.5	Promoter(<=1kb)	47	OR6N1	128372
467998	1q	169542317	169542882	566	6	0.167	Exon(exon13of25)	-26572	F5	2153
467998	1q	201206099	201209342	3244	10	0.6	Promoter(1-2kb)	1017	IGFN1	91156
467998	1q	226735563	226737239	1677	6	0.833	Promoter(<=1kb)	219	ITPKB	3707
467998	1q	247841312	247841582	271	6	0.833	Promoter(<=1kb)	314	OR11L1	391189
467998	1q	247949325	247949738	414	10	0.3	Promoter(<=1kb)	467	OR2L8	391190
467998	1q	248294677	248295458	782	7	0.571	Promoter(<=1kb)	142	OR2T12	127064
467998	2p	48580657	48582454	1798	7	0.571	Promoter(<=1kb)	0	STON1	11037
467998	2q	132781993	132784972	2980	8	0.5	Promoter(<=1kb)	0	NCKAP5	344148
467998	2q	178739433	178741811	2379	6	0.5	Exon(exon45of191)	26014	TTN	7273
467998	2q	185789865	185794632	4768	10	0.8	Promoter(<=1kb)	0	FSIP2	401024
467998	2q	208325606	208326375	770	7	0.286	3'UTR	-7997	PIKFYVE	200576
467998	2q	217847583	217848559	977	6	0.833	Exon(exon19of33)	-5423	TNS1	7145
467998	2q	240041845	240042154	310	6	0.167	Downstream(2-3kb)	3918	OR6B3	150681
467998	3p	31989643	31990470	828	6	0.167	Exon(exon2of2)	7872	ZNF860	344787
467998	3p	75736880	75738859	1980	68	0.632	Promoter(<=1kb)	0	MIR4273	100422955
467998	3q	98169021	98169561	541	6	0.5	Exon(exon2of2)	19695	OR5H14	403273
467998	3q	98264413	98265098	686	7	0.571	Promoter(<=1kb)	128	OR5H6	79295
467998	3q	194341097	194342571	1475	7	0.714	Exon(exon2of2)	8747	CPN2	1370
467998	3q	194359607	194360963	1357	10	0.9	Exon(exon2of2)	-8279	CPN2	1370
467998	4p	5988383	5989749	1367	7	0.571	Promoter(<=1kb)	0	C4orf50	389197
467998	4p	6300792	6302391	1600	7	0.857	Exon(exon8of8)	6021	WFS1	7466
467998	4p	8227004	8228508	1505	8	0.125	Promoter(<=1kb)	-24	SH3TC1	54436
467998	4q	185458217	185460011	1795	8	0.625	Promoter(<=1kb)	0	CCDC110	256309
467998	4q	186619481	186621582	2102	6	0.333	Exon(exon10of27)	-9411	FAT1	2195
467998	4q	186706638	186708616	1979	6	0.667	Exon(exon2of27)	15217	FAT1	2195
467998	5q	140807352	140807737	386	6	0.833	Promoter(<=1kb)	271	PCDHA4	56144
467998	5q	140848579	140850948	2370	7	0.571	Promoter(<=1kb)	807	PCDHA9	9752
467998	5q	141150357	141152584	2228	7	0.857	Promoter(<=1kb)	255	PCDHB6	56130
467998	5q	141172977	141174900	1924	10	0.9	Promoter(<=1kb)	333	PCDHB7	56129
467998	5q	141178745	141180333	1589	10	0.7	Promoter(<=1kb)	955	PCDHB8	56128
467998	5q	141187668	141188806	1139	8	0.25	Promoter(<=1kb)	507	PCDHB9	56127
467998	5q	141192902	141194383	1482	6	0.833	Promoter(<=1kb)	549	PCDHB10	56126
467998	5q	141245861	141247657	1797	11	0.727	Promoter(<=1kb)	466	PCDHB15	56121
467998	5q	141955356	141957660	2305	7	0.714	Promoter(<=1kb)	-668	RNF14	9604
467998	5q	151521550	151522069	520	6	0.667	Promoter(<=1kb)	79	MIR6499	102465246
467998	6p	27309272	27310722	1451	6	0.833	Promoter(1-2kb)	1448	POM121L2	94026
467998	6p	46858771	46859502	732	8	0.5	Exon(exon17of21)	3802	ADGRF5	221395
467998	6q	64590250	64591961	1712	12	0.5	Exon(exon26of43)	121374	EYS	346007
467998	6q	149888581	149890867	2287	7	0.714	Promoter(<=1kb)	0	RAET1E-AS1	100652739
467998	6q	159233455	159234370	916	10	0.5	Exon(exon11of23)	15158	FNDC1	84624
467998	7p	45082865	45084866	2002	6	0.333	Promoter(1-2kb)	-1465	NACAD	23148
467998	7q	64991278	64992758	1481	6	0.667	Promoter(<=1kb)	-242	ZNF117	51351
467998	7q	100958721	100960873	2153	53	0.491	Promoter(<=1kb)	756	MUC3A	4584

467998	7q	100991195	100992398	1204	7	0.571	Exon(exon5of15)	-20656	MUC12	10071
467998	7q	100995575	100995785	211	7	0.714	Exon(exon5of15)	-17269	MUC12	10071
467998	7q	101034361	101040583	6223	40	0.5	Exon(exon3of12)	-3128	MUC17	140453
467998	8p	10607375	10612307	4933	16	0.625	Exon(exon4of4)	42836	RP1L1	94137
467998	8p	12137448	12138512	1065	6	0.833	Promoter(<=1kb)	565	USP17L2	377630
467998	8p	13021128	13022030	903	7	0.143	Exon(exon5of5)	9115	TRMT9B	57604
467998	8q	123651873	123652634	762	6	0.333	Promoter(<=1kb)	316	KLHL38	340359
467998	8q	142664633	142665852	1220	6	0.833	Exon(exon2of2)	4118	JRK	8629
467998	9p	21206764	21207074	311	6	0.5	Promoter(<=1kb)	69	IFNA10	3446
467998	9p	39078723	39078846	124	6	0.667	Exon(exon22of24)	7302	CNTNAP3	79937
467998	9q	76705724	76707804	2081	7	0.571	Promoter(<=1kb)	666	PCA3	50652
467998	9q	87885490	87888819	3330	8	0.625	Promoter(2-3kb)	2613	SPATA31E1	286234
467998	9q	104598641	104599361	721	8	0.625	Promoter(<=1kb)	52	OR13C5	138799
467998	9q	122628595	122629398	804	7	0.286	Promoter(<=1kb)	175	OR1B1	347169
467998	9q	122749914	122750547	634	6	0.833	Promoter(<=1kb)	174	OR1L6	392390
467998	9q	131474844	131476113	1270	6	0.833	Promoter(<=1kb)	0	PRRC2B	84726
467998	9q	135484695	135486007	1313	6	0.333	Promoter(1-2kb)	1332	PPP1R26	9858
467998	10p	47663	48605	943	7	0.571	Promoter(<=1kb)	664	TUBB8	347688
467998	10q	46549378	46550723	1346	25	0.64	Exon(exon3of3)	4807	GPRIN2	9721
467998	10q	49323169	49325192	2024	7	0.571	Exon(exon3of3)	23895	C10orf71	118461
467998	10q	128103129	128104830	1702	10	0.6	Promoter(<=1kb)	-1	MKI67	4288
467998	11p	244106	244197	92	8	0.5	Promoter(<=1kb)	-232	PSMD13	5719
467998	11p	1194354	1196902	2549	7	0.571	Exon(exon34of49)	-26164	MUC5B	727897
467998	11p	1246095	1247378	1284	7	0.286	Promoter(2-3kb)	2298	MUC5B-AS1	112577518
467998	11p	1250091	1251628	1538	6	0.833	Promoter(<=1kb)	-415	MUC5B-AS1	112577518
467998	11p	5177978	5178478	501	6	0.167	Promoter(<=1kb)	186	OR52Z1	283110
467998	11p	5389704	5390350	647	7	0.429	Promoter(<=1kb)	327	OR51M1	390059
467998	11p	5402638	5403322	685	9	0.444	Promoter(<=1kb)	41	OR51J1	79470
467998	11p	5422212	5423123	912	11	0.636	Promoter(<=1kb)	101	OR51Q1	390061
467998	11p	5440604	5441472	869	6	0.833	Promoter(<=1kb)	42	OR51I1	390063
467998	11p	5515185	5516015	831	6	0.333	Promoter(<=1kb)	684	UBQLNL	143630
467998	11p	5581045	5581738	694	8	0.375	Promoter(<=1kb)	168	OR52B6	340980
467998	11p	5884818	5885061	244	6	0.333	Promoter(<=1kb)	547	OR52E4	390081
467998	11p	11351961	11352736	776	9	0.222	Promoter(<=1kb)	514	CSNK2A3	283106
467998	11p	12293639	12294538	900	8	0.625	Exon(exon29of35)	6739	MICALCL	84953
467998	11q	55827536	55827640	105	6	0.667	Promoter(<=1kb)	317	OR5L2	26338
467998	11q	58214757	58215722	966	6	0.333	Promoter(<=1kb)	12	OR1S1	219959
467998	11q	58402523	58403265	743	8	0.5	Promoter(<=1kb)	144	OR5B3	441608
467998	11q	82732630	82733184	555	6	0.833	Promoter(<=1kb)	680	FAM181B	220382
467998	11q	85724687	85725825	1139	6	0.5	Promoter(<=1kb)	0	SYTL2	54843
467998	11q	123906790	123907324	535	6	0.667	Promoter(<=1kb)	644	OR8D4	338662
467998	11q	124038366	124038988	623	7	0.857	Promoter(<=1kb)	13	OR10G7	390265
467998	11q	124382526	124383285	760	8	0.625	Promoter(<=1kb)	58	OR8B2	26595
467998	12p	4626568	4628549	1982	11	0.455	Exon(exon5of6)	14051	DYRK4	8798
467998	12p	6453119	6453670	552	6	0.667	Promoter(<=1kb)	633	TAPBPL	55080
467998	12p	31981130	31983004	1875	6	0.167	Exon(exon4of6)	-4162	RESF1	55196
467998	13q	24434450	24435347	898	7	0.571	Exon(exon31of34)	19787	PARP4	143
467998	13q	25096713	25097182	470	9	0.333	Promoter(<=1kb)	845	PABPC3	5042
467998	13q	102732424	102733933	1510	6	0.333	Exon(exon4of4)	25139	CCDC168	643677
467998	14q	19975713	19976448	736	9	0.444	Promoter(<=1kb)	269	OR4K15	81127
467998	14q	20060048	20060884	837	8	0.625	Promoter(<=1kb)	3	OR4L1	122742
467998	14q	21634137	21634589	453	9	0.556	Promoter(<=1kb)	351	OR10G2	26534
467998	14q	22633879	22634450	572	9	0.333	Exon(exon2of2)	32212	ABHD4	63874
467998	14q	70457532	70458540	1009	9	0.333	Exon(exon2of2)	5358	ADAM21	8747
467998	14q	104175275	104177810	2536	10	0.3	Exon(exon12of15)	36235	KIF26A	26153
467998	14q	104939262	104954713	15452	71	0.493	Promoter(<=1kb)	689	AHNAK2	113146
467998	15q	23439979	23442067	2089	14	0.5	5'UTR	5167	GOLGA6L2	283685
467998	15q	78766049	78766626	578	6	0.667	Promoter(<=1kb)	-936	ADAMTS7	11173
467998	15q	85579423	85581800	2378	14	0.571	Promoter(1-2kb)	-1110	AKAP13	11214
467998	15q	88857108	88859365	2258	6	0	Exon(exon12of18)	9865	ACAN	176
467998	16p	1228744	1229731	988	10	0.7	Promoter(<=1kb)	431	TPSB2	64499
467998	16p	1486371	1488463	2093	8	0.75	Promoter(<=1kb)	4	PTX4	390667
467998	16p	4207059	4208004	946	7	0.571	Exon(exon2of7)	31739	SRL	6345
467998	16q	74391416	74392004	589	9	0.667	Exon(exon7of7)	13538	NPIPB15	440348
467998	16q	89100686	89101050	365	7	0.571	Promoter(<=1kb)	24	ACSF3	197322
467998	16q	89226863	89228289	1427	8	0.625	Promoter(2-3kb)	2229	ZNF778	197320
467998	17p	744946	746966	2021	6	1	3'UTR	5072	GEMIN4	50628
467998	17p	21300581	21300954	374	8	0.75	3'UTR	9112	MAP2K3	5606
467998	17p	21415470	21416431	962	7	0.714	Exon(exon3of3)	10334	KCNJ12	3768
467998	17q	76293419	76294016	598	6	0.5	Promoter(2-3kb)	-2167	QRICH2	84074
467998	17q	81645135	81645595	461	7	0.429	Promoter(2-3kb)	2722	TSPAN10	83882
467998	18p	11609904	11610469	566	8	0.625	Promoter(<=1kb)	308	SLC35G4	646000
467998	18q	47033929	47035145	1217	6	0.833	Promoter(<=1kb)	476	ELOA2	51224
467998	18q	58535186	58538030	2845	19	0.526	Promoter(<=1kb)	0	ALPK2	115701
467998	19p	1004688	1005532	845	6	0.5	Exon(exon3of9)	4269	GRIN3B	116444
467998	19p	1036457	1037830	1374	7	0.571	Promoter(2-3kb)	-2271	ABCA7	10347
467998	19p	4510548	4513547	3000	23	0.522	Exon(exon3of6)	4157	PLIN4	729359

467998	19p	5455600	5456439	840	7	0.571	Promoter(<=1kb)	183	ZNRF4	148066
467998	19p	8946313	8951868	5556	15	0.6	Exon(exon3of84)	29474	MUC16	94025
467998	19p	8959116	8962299	3184	10	0.7	Exon(exon3of84)	19043	MUC16	94025
467998	19p	8972751	8978096	5346	13	0.462	Exon(exon1of84)	3246	MUC16	94025
467998	19p	12430157	12432437	2281	9	0.333	3'UTR	8584	ZNF443	10224
467998	19p	15087213	15088040	828	10	0.3	Promoter(<=1kb)	233	OR1I1	126370
467998	19p	18264798	18267409	2612	12	0.583	5'UTR	7002	IQCN	80726
467998	19p	21971930	21974500	2571	7	0.714	Exon(exon4of4)	14408	ZNF208	7757
467998	19q	37885190	37888806	3617	8	0.625	Exon(exon6of6)	17788	WDR87	83889
467998	19q	39877222	39877880	659	6	0.5	Exon(exon20of28)	9412	FCGBP	8857
467998	19q	39886240	39886422	183	6	0.667	Promoter(<=1kb)	870	FCGBP	8857
467998	19q	43913423	43914878	1456	7	0.429	Exon(exon10of10)	4861	ZNF45	7596
467998	19q	43996326	43997366	1041	6	0.5	Exon(exon5of5)	5419	LOC101928063	101928063
467998	19q	44106512	44108078	1567	9	0.111	Exon(exon6of6)	-4103	ZNF225	7768
467998	19q	44327836	44329698	1863	6	0.5	Exon(exon4of4)	-22790	ZNF235	9310
467998	19q	52437918	52439242	1325	7	0.429	Exon(exon4of4)	6504	ZNF534	147658
467998	19q	53164551	53166239	1689	7	0.286	Exon(exon4of4)	-5476	ZNF347	84671
467998	19q	55911888	55913166	1279	7	0.714	Exon(exon5of12)	19145	NLRP13	126204
467998	19q	58368293	58368875	583	7	0.429	Exon(exon3of3)	-5445	ZNF497	162968
467998	20p	5922421	5923394	974	7	0.571	Exon(exon4of5)	6923	CHGB	1114
467998	20p	20052354	20052736	383	6	0.167	Promoter(<=1kb)	0	CFAP61	26074
467998	20q	63561666	63565531	3866	13	0.615	Promoter(<=1kb)	-61	HELZ2	85441
467998	21q	26843740	26844859	1120	6	0.667	Promoter(<=1kb)	0	ADAMTS1	9510
467998	21q	44550738	44551416	679	7	0.857	Promoter(<=1kb)	89	KRTAP10-2	386679
467998	21q	44637474	44638143	670	11	0.455	Promoter(<=1kb)	118	KRTAP10-10	353333
467998	22q	22352950	22353380	431	16	0.5	Exon(exon1of2)	30478	BMS1P20	96610
467998	22q	36191154	36191906	753	8	0.625	3'UTR	9971	APOL4	80832
467998	22q	49883704	49884994	1291	6	0.167	Exon(exon2of2)	22924	ALG12	79087
467998	23p	3320126	3323750	3625	9	0.556	Exon(exon5of7)	22902	MXRA5	25878
467998	23q	136874183	136874416	234	8	0.75	Promoter(<=1kb)	-201	RBMX	27316
469469	1p	12859108	12860212	1105	9	0.556	Promoter(2-3kb)	2022	PRAMEF2	65122
469469	1p	18481403	18483232	1830	8	0.75	Promoter(<=1kb)	421	KLHDC7A	127707
469469	1p	40067594	40067675	82	6	0	Promoter(<=1kb)	324	CAP1	10487
469469	1p	89186388	89186419	32	9	0.556	Promoter(<=1kb)	107	GBP4	115361
469469	1q	152213286	152213320	35	7	0.429	Exon(exon3of3)	10873	HRNR	388697
469469	1q	152219032	152221375	2344	16	0.75	Promoter(2-3kb)	2818	HRNR	388697
469469	1q	158765805	158766655	851	6	0.5	Promoter(<=1kb)	47	OR6N1	128372
469469	1q	201206099	201209738	3640	11	0.545	Promoter(1-2kb)	1017	IGFN1	91156
469469	1q	214640144	214642954	2811	12	0.5	Exon(exon12of20)	-5013	CENPF	1063
469469	1q	214644872	214647181	2310	10	0.4	Promoter(<=1kb)	-786	CENPF	1063
469469	1q	228315976	228318026	2051	6	0.5	Exon(exon50of81)	6492	OBSCN	84033
469469	1q	247841312	247841582	271	6	0.833	Promoter(<=1kb)	314	OR11L1	391189
469469	1q	247949325	247949738	414	9	0.333	Promoter(<=1kb)	467	OR2L8	391190
469469	2p	48580657	48582454	1798	7	0.571	Promoter(<=1kb)	0	STON1	11037
469469	2q	185789865	185794632	4768	10	0.8	Promoter(<=1kb)	0	FSIP2	401024
469469	2q	217847583	217848559	977	6	0.833	Exon(exon19of33)	-5423	TNS1	7145
469469	2q	233713134	233713783	650	13	0.692	Promoter(<=1kb)	142	UGT1A5	54579
469469	2q	233729143	233729909	767	7	0.286	Promoter(<=1kb)	35	UGT1A3	54659
469469	2q	233840612	233842185	1574	6	0.667	Promoter(<=1kb)	0	HJURP	55355
469469	2q	238130416	238131546	1131	7	0.429	Promoter(1-2kb)	1468	ESPNL	339768
469469	3p	31989532	31990905	1374	6	0.333	Exon(exon2of2)	7761	ZNF860	344787
469469	3p	75737230	75739007	1778	8	0.625	Promoter(<=1kb)	0	MIR4273	100422955
469469	3q	98264413	98265098	686	6	0.5	Promoter(<=1kb)	128	OR5H6	79295
469469	3q	194359607	194360906	1300	9	0.889	Exon(exon2of2)	-8279	CPN2	1370
469469	4p	5988383	5989749	1367	7	0.571	Promoter(<=1kb)	0	C4orf50	389197
469469	4p	6300792	6302360	1569	6	0.833	Exon(exon8of8)	6021	WFS1	7466
469469	4p	8227004	8228508	1505	8	0.125	Promoter(<=1kb)	-24	SH3TC1	54436
469469	4q	121036404	121037542	1139	6	0.333	Promoter(1-2kb)	1442	NDNF	79625
469469	4q	186617176	186621601	4426	11	0.364	Exon(exon10of27)	-7106	FAT1	2195
469469	4q	186706638	186709436	2799	7	0.714	Exon(exon2of27)	14397	FAT1	2195
469469	5q	67163994	67165736	1743	6	0.5	Exon(exon29of29)	20561	CD180	4064
469469	5q	79728956	79730716	1761	6	0.333	Exon(exon2of13)	-7426	CMYA5	202333
469469	5q	79731782	79734523	2742	13	0.308	Exon(exon2of13)	-3619	CMYA5	202333
469469	5q	140848579	140850786	2208	7	0.571	Promoter(<=1kb)	807	PCDHA9	9752
469469	5q	141174000	141175025	1026	6	0.833	Promoter(1-2kb)	1356	PCDHB7	56129
469469	5q	151565922	151568158	2237	9	0.778	Promoter(<=1kb)	786	FAT2	2196
469469	6p	1312843	1313745	903	6	0.5	Promoter(<=1kb)	745	FOXQ1	94234
469469	6p	46858771	46859502	732	8	0.5	Exon(exon17of21)	3802	ADGRF5	221395
469469	6q	159233455	159234370	916	10	0.5	Exon(exon11of23)	15158	FNDC1	84624
469469	7p	12369637	12370736	1100	6	0.667	3'UTR	-13307	VWDE	221806
469469	7p	45082725	45084866	2142	8	0.5	Promoter(1-2kb)	-1325	NACAD	23148
469469	7q	100958721	100960873	2153	56	0.446	Promoter(<=1kb)	756	MUC3A	4584
469469	7q	100991195	100992398	1204	8	0.625	Exon(exon5of15)	-20656	MUC12	10071
469469	7q	155357695	155358070	376	6	0.5	Exon(exon6of6)	9864	BLACE	338436
469469	8p	10607245	10608432	1188	8	0.5	Exon(exon4of4)	46711	RP1L1	94137
469469	8p	10609614	10610662	1049	8	0.5	Exon(exon4of4)	44481	RP1L1	94137
469469	8p	12132686	12133940	1255	7	0.714	Promoter(<=1kb)	498	USP17L7	392197

469469	8p	13021128	13022030	903	8	0.125	Exon(exon5of5)	9115	TRMT9B	57604
469469	8p	22163524	22164421	898	6	1	Promoter(<=1kb)	444	SFTPC	6440
469469	8q	138151949	138153046	1098	6	0.667	Promoter(<=1kb)	0	FAM135B	51059
469469	9q	76175241	76175266	26	6	1	Exon(exon14of14)	-13373	PCSK5	5125
469469	9q	76703555	76706360	2806	8	0.5	Promoter(<=1kb)	0	PCA3	50652
469469	9q	87885490	87888819	3330	8	0.625	Promoter(2-3kb)	2613	SPATA31E1	286234
469469	9q	104504315	104505071	757	6	0.5	Promoter(<=1kb)	52	OR13F1	138805
469469	9q	122553263	122554071	809	7	0.429	Promoter(<=1kb)	93	OR1N2	138882
469469	9q	135484803	135487213	2411	8	0.25	Promoter(1-2kb)	1440	PPP1R26	9858
469469	9q	135547960	135548795	836	8	0.625	Promoter(1-2kb)	1805	OBP2A	29991
469469	10q	46549378	46550723	1346	25	0.64	Exon(exon3of3)	4807	GPRIN2	9721
469469	10q	49323559	49326331	2773	9	0.444	Exon(exon3of3)	24285	C10orf71	118461
469469	10q	122084988	122087840	2853	8	0.75	Exon(exon4of23)	-25190	TACC2	10579
469469	11p	244106	244197	92	8	0.5	Promoter(<=1kb)	-232	PSMD13	5719
469469	11p	5177978	5178478	501	6	0.167	Promoter(<=1kb)	186	OR52Z1	283110
469469	11p	5389704	5390350	647	7	0.429	Promoter(<=1kb)	327	OR51M1	390059
469469	11p	5422212	5423123	912	11	0.636	Promoter(<=1kb)	101	OR51Q1	390061
469469	11p	5515185	5515931	747	6	0.333	Promoter(<=1kb)	768	UBQLNL	143630
469469	11p	5581045	5581738	694	8	0.375	Promoter(<=1kb)	168	OR52B6	340980
469469	11p	5841302	5841883	582	9	0.333	Promoter(<=1kb)	14	OR52E6	390078
469469	11p	11352040	11352736	697	8	0.25	Promoter(<=1kb)	514	CSNK2A3	283106
469469	11p	12293639	12294368	730	6	0.833	Exon(exon29of35)	6739	MICALCL	84953
469469	11p	48365634	48366039	406	7	0.286	Promoter(<=1kb)	426	OR4C5	79346
469469	11q	55827536	55827640	105	6	0.667	Promoter(<=1kb)	317	OR5L2	26338
469469	11q	58214757	58215722	966	7	0.286	Promoter(<=1kb)	12	OR1S1	219959
469469	11q	64116513	64118232	1720	7	0.714	Exon(exon2of2)	8702	MACROD1	28992
469469	11q	82732630	82733208	579	7	0.857	Promoter(<=1kb)	656	FAM181B	220382
469469	11q	85724687	85725825	1139	6	0.5	Promoter(<=1kb)	0	SYTL2	54843
469469	11q	123906595	123907324	730	7	0.429	Promoter(<=1kb)	449	OR8D4	338662
469469	11q	124015600	124016477	878	7	0.143	Promoter(<=1kb)	25	OR10G4	390264
469469	11q	124264847	124265676	830	6	0.833	Promoter(<=1kb)	20	OR8G5	219865
469469	12p	4626568	4628549	1982	11	0.455	Exon(exon5of6)	14051	DYRK4	8798
469469	12p	6453119	6453670	552	6	0.667	Promoter(<=1kb)	633	TAPBPL	55080
469469	12p	8222174	8223514	1341	6	0.667	Exon(exon5of6)	4073	FAM90A1	55138
469469	14q	19975713	19976448	736	10	0.5	Promoter(<=1kb)	269	OR4K15	81127
469469	14q	20060048	20060884	837	8	0.625	Promoter(<=1kb)	3	OR4L1	122742
469469	14q	20640982	20641567	586	6	0.5	Promoter(<=1kb)	124	OR6S1	341799
469469	14q	21634177	21634589	413	6	0.833	Promoter(<=1kb)	351	OR10G2	26534
469469	14q	22633879	22634450	572	9	0.333	Exon(exon2of2)	32212	ABHD4	63874
469469	14q	44504986	44506403	1418	6	0.667	Promoter(<=1kb)	880	FSCB	84075
469469	14q	70457532	70458238	707	9	0.333	Exon(exon2of2)	5358	ADAM21	8747
469469	14q	104175275	104177810	2536	7	0.286	Exon(exon12of15)	36235	KIF26A	26153
469469	14q	104939262	104942618	3357	10	0.2	5'UTR	7102	PLD4	122618
469469	14q	104943622	104945444	1823	6	0.667	Exon(exon6of6)	9958	AHNAK2	113146
469469	14q	104947943	104953878	5936	33	0.424	Promoter(1-2kb)	1524	AHNAK2	113146
469469	15q	23440196	23442067	1872	10	0.6	5'UTR	5167	GOLGA6L2	283685
469469	15q	40621642	40624434	2793	8	0.375	Promoter(<=1kb)	0	KNL1	57082
469469	15q	78766033	78766618	586	6	0.5	Promoter(<=1kb)	-920	ADAMTS7	11173
469469	15q	85579423	85582073	2651	15	0.6	Promoter(<=1kb)	-837	AKAP13	11214
469469	15q	99130060	99132517	2458	7	0.429	Exon(exon4of5)	7225	TTC23	64927
469469	15q	100569472	100570097	626	6	0.833	Promoter(<=1kb)	534	LINS1	55180
469469	16p	669592	672548	2957	7	0.571	Promoter(<=1kb)	0	RHOT2	89941
469469	16p	789084	790597	1514	6	0.167	Promoter(<=1kb)	0	CHTF18	63922
469469	16p	1256354	1256985	632	12	0.583	Promoter(<=1kb)	285	TPSD1	23430
469469	16q	74391650	74391897	248	9	0.778	Exon(exon7of7)	13772	NPIPB15	440348
469469	16q	89100686	89101050	365	8	0.625	Promoter(<=1kb)	24	ACSF3	197322
469469	16q	89227206	89228419	1214	8	0.625	Promoter(2-3kb)	2572	ZNF778	197320
469469	17p	21300581	21300954	374	8	0.75	3'UTR	9112	MAP2K3	5606
469469	17p	21415458	21416431	974	11	0.818	Exon(exon3of3)	10322	KCNJ12	3768
469469	17q	53823368	53824891	1524	6	0.833	Promoter(<=1kb)	441	KIF2B	84643
469469	17q	76291123	76294016	2894	11	0.455	Promoter(<=1kb)	0	QRICH2	84074
469469	17q	81645135	81645417	283	6	0.333	Promoter(2-3kb)	2722	TSPAN10	83882
469469	18p	11609728	11610333	606	7	0.857	Promoter(<=1kb)	132	SLC35G4	646000
469469	18p	14542649	14543140	492	7	0.571	Promoter(<=1kb)	6	POTEC	388468
469469	18q	58535186	58537515	2330	9	0.333	Promoter(<=1kb)	0	ALPK2	115701
469469	19p	603625	603874	250	6	0.5	Exon(exon2of8)	13744	HCN2	610
469469	19p	4510548	4513547	3000	22	0.409	Exon(exon3of6)	4157	PLIN4	729359
469469	19p	5455600	5456439	840	7	0.571	Promoter(<=1kb)	183	ZNRF4	148066
469469	19p	8948231	8951868	3638	11	0.545	Exon(exon3of84)	29474	MUC16	94025
469469	19p	12075333	12077046	1714	6	0.167	Promoter(<=1kb)	6	ZNF788P	388507
469469	19p	12430718	12431840	1123	6	0.5	3'UTR	9181	ZNF443	10224
469469	19p	15087452	15087953	502	7	0.143	Promoter(<=1kb)	472	OR1I1	126370
469469	19p	17281820	17284246	2427	9	0.556	Promoter(<=1kb)	0	ANKLE1	126549
469469	19p	18264753	18267409	2657	14	0.643	5'UTR	7002	IQCN	80726
469469	19p	23743906	23745300	1395	6	0.333	Exon(exon4of4)	13537	ZNF681	148213
469469	19q	39877222	39877880	659	6	0.5	Exon(exon20of28)	9412	FCGBP	8857
469469	19q	43913423	43914878	1456	8	0.5	Exon(exon10of10)	4861	ZNF45	7596

469469	19q	43966037	43967171	1135	6	0.167	Promoter(<=1kb)	-691	ZNF155	7711
469469	19q	44106512	44108078	1567	7	0	Exon(exon6of6)	-4103	ZNF225	7768
469469	19q	51745958	51746963	1006	6	0.333	Exon(exon3of3)	3848	FPR1	2357
469469	19q	52384029	52384992	964	7	0.143	Exon(exon4of4)	12677	ZNF528-AS1	102724105
469469	19q	52437918	52439242	1325	7	0.429	Exon(exon4of4)	6504	ZNF534	147658
469469	19q	52840798	52842068	1271	9	0.333	3'UTR	7457	ZNF468	90333
469469	19q	55857823	55858800	978	6	0.833	Promoter(<=1kb)	0	NLRP4	147945
469469	19q	55911888	55913077	1190	6	0.667	Exon(exon5of12)	19234	NLRP13	126204
469469	19q	58416932	58417769	838	6	0.5	3'UTR	8155	ZNF584	201514
469469	20p	20052354	20052736	383	6	0.167	Promoter(<=1kb)	0	CFAP61	26074
469469	20q	63561666	63565531	3866	11	0.636	Promoter(<=1kb)	-61	HELZ2	85441
469469	21q	26843740	26844859	1120	6	0.667	Promoter(<=1kb)	0	ADAMTS1	9510
469469	21q	44550835	44551416	582	8	0.875	Promoter(<=1kb)	89	KRTAP10-2	386679
469469	21q	44600627	44601692	1066	8	0.875	Promoter(<=1kb)	30	KRTAP10-7	386675
469469	22q	22352950	22353365	416	15	0.533	Exon(exon1of2)	30478	BMS1P20	96610
469469	22q	36191154	36191906	753	6	0.667	3'UTR	9971	APOL4	80832
469469	22q	39100331	39102033	1703	6	0.833	Promoter(<=1kb)	52	APOBEC3H	164668
469469	23p	8170039	8170141	103	6	0.5	Promoter(1-2kb)	1126	VCX2	51480
469469	23p	35802148	35803010	863	7	0.571	5'UTR	3357	MAGEB16	139604
469840	1p	16058547	16060000	1454	6	0.833	Exon(exon5of7)	6224	CLCNKB	1188
469840	1p	18481042	18482217	1176	12	0.75	Promoter(<=1kb)	60	KLHDC7A	127707
469840	1p	23874604	23875430	827	8	0.5	Exon(exon2of2)	-6310	FUCA1	2517
469840	1p	40067594	40067675	82	6	0	Promoter(<=1kb)	324	CAP1	10487
469840	1q	152218469	152221375	2907	17	0.706	Promoter(2-3kb)	2818	HRNR	388697
469840	1q	152302977	152304920	1944	10	0.6	Exon(exon3of3)	-7656	FLG-AS1	339400
469840	1q	152306380	152313891	7512	28	0.607	Promoter(<=1kb)	0	FLG-AS1	339400
469840	1q	201206099	201209738	3640	11	0.545	Promoter(1-2kb)	1017	IGFN1	91156
469840	1q	223393517	223394466	950	6	0.667	Promoter(<=1kb)	102	CCDC185	164127
469840	1q	228315976	228318038	2063	8	0.625	Exon(exon50of81)	6492	OBSCN	84033
469840	1q	232805117	232806800	1684	8	0.5	Promoter(<=1kb)	225	MAP10	54627
469840	1q	247841312	247841582	271	6	0.833	Promoter(<=1kb)	314	OR11L1	391189
469840	1q	247895950	247896410	461	6	0.5	Promoter(<=1kb)	363	OR2W3	343171
469840	1q	248060797	248061452	656	7	0.571	Exon(exon2of2)	22625	OR2L2	26246
469840	1q	248681658	248682198	541	7	0.571	Promoter(<=1kb)	130	OR14I1	401994
469840	2p	48580657	48582454	1798	7	0.571	Promoter(<=1kb)	0	STON1	11037
469840	2q	167243214	167244632	1419	6	0.333	Exon(exon9of11)	-4050	XIRP2	129446
469840	2q	167246794	167248478	1685	8	0.625	Promoter(<=1kb)	-204	XIRP2	129446
469840	2q	178714003	178715707	1705	6	0.333	Exon(exon8of8)	-11483	TTN	7273
469840	2q	178739433	178741811	2379	6	0.5	Exon(exon45of191)	26014	TTN	7273
469840	2q	184936178	184937636	1459	6	0.333	Exon(exon4of4)	69813	ZNF804A	91752
469840	2q	217847567	217848213	647	6	0.833	Exon(exon19of33)	-5407	TNS1	7145
469840	2q	237762685	237764060	1376	10	0.5	Exon(exon8of8)	-4137	LRRFIP1	9208
469840	3p	75737230	75739007	1778	9	0.556	Promoter(<=1kb)	0	MIR4273	100422955
469840	3q	98264413	98265098	686	6	0.5	Promoter(<=1kb)	128	OR5H6	79295
469840	3q	194341097	194342571	1475	7	0.714	Exon(exon2of2)	8747	CPN2	1370
469840	4p	5988383	5989749	1367	7	0.571	Promoter(<=1kb)	0	C4orf50	389197
469840	4p	6300792	6302360	1569	7	0.857	Exon(exon8of8)	6021	WFS1	7466
469840	4p	7433331	7434512	1182	6	0.833	Promoter(<=1kb)	418	PSAPL1	768239
469840	4p	38774486	38775552	1067	8	0.5	Promoter(<=1kb)	644	TLR10	81793
469840	4q	185458217	185460011	1795	9	0.556	Promoter(<=1kb)	0	CCDC110	256309
469840	5q	79731782	79734523	2742	13	0.308	Exon(exon2of13)	-3619	CMYA5	202333
469840	5q	83537326	83539905	2580	6	0.333	Promoter(1-2kb)	1712	VCAN	1462
469840	5q	140848579	140850786	2208	9	0.556	Promoter(<=1kb)	807	PCDHA9	9752
469840	5q	141174000	141175025	1026	6	0.833	Promoter(1-2kb)	1356	PCDHB7	56129
469840	5q	141187690	141189425	1736	6	0.5	Promoter(<=1kb)	529	PCDHB9	56127
469840	5q	151565922	151568455	2534	10	0.8	Promoter(<=1kb)	489	FAT2	2196
469840	6p	1312843	1313745	903	6	0.5	Promoter(<=1kb)	745	FOXQ1	94234
469840	6p	46858771	46859502	732	8	0.5	Exon(exon17of21)	3802	ADGRF5	221395
469840	6q	64591274	64591961	688	10	0.5	Exon(exon26of43)	121374	EYS	346007
469840	6q	149888581	149890867	2287	7	0.714	Promoter(<=1kb)	0	RAET1E-AS1	100652739
469840	6q	159231899	159234370	2472	12	0.583	Exon(exon11of23)	13602	FNDC1	84624
469840	7p	38353718	38353991	274	8	0.625	Exon(exon2of2)	3699	TRG-AS1	100506776
469840	7q	100958721	100960873	2153	58	0.431	Promoter(<=1kb)	756	MUC3A	4584
469840	7q	100991195	100993102	1908	9	0.556	Exon(exon5of15)	-19952	MUC12	10071
469840	8p	8376561	8377198	638	6	1	Exon(exon2of5)	4549	PRAG1	157285
469840	8p	10609614	10610667	1054	8	0.5	Exon(exon4of4)	44476	RP1L1	94137
469840	8p	13021128	13022030	903	7	0.143	Exon(exon5of5)	9115	TRMT9B	57604
469840	8q	141466429	141467514	1086	9	0.778	3'UTR	29245	MROH5	389690
469840	8q	143916351	143919209	2859	8	0.25	Exon(exon32of32)	20381	PLEC	5339
469840	9q	76175241	76175296	56	8	0.875	Exon(exon14of14)	-13343	PCSK5	5125
469840	9q	76705724	76707804	2081	7	0.571	Promoter(<=1kb)	666	PCA3	50652
469840	9q	87887543	87888819	1277	6	0.5	Exon(exon4of4)	4666	SPATA31E1	286234
469840	9q	104504315	104505071	757	6	0.5	Promoter(<=1kb)	52	OR13F1	138805
469840	9q	122628595	122629398	804	6	0.333	Promoter(<=1kb)	175	OR1B1	347169
469840	9q	133255902	133256264	363	6	0.833	Exon(exon7of7)	18950	ABO	28
469840	9q	135484803	135487213	2411	9	0.333	Promoter(1-2kb)	1440	PPP1R26	9858
469840	10q	46549378	46550723	1346	25	0.64	Exon(exon3of3)	4807	GPRIN2	9721

469840	10q	49323169	49326817	3649	12	0.583	Exon(exon3of3)	23895	C10orf71	118461
469840	10q	122084988	122087840	2853	8	0.75	Exon(exon4of23)	-25190	TACC2	10579
469840	10q	128102802	128106296	3495	13	0.538	Promoter(<=1kb)	0	MKI67	4288
469840	11p	244106	244197	92	8	0.5	Promoter(<=1kb)	-232	PSMD13	5719
469840	11p	1248397	1251628	3232	7	0.857	Promoter(<=1kb)	0	MUC5B-AS1	112577518
469840	11p	5177978	5178478	501	6	0.167	Promoter(<=1kb)	186	OR52Z1	283110
469840	11p	5389704	5390350	647	7	0.429	Promoter(<=1kb)	327	OR51M1	390059
469840	11p	5402638	5403322	685	10	0.5	Promoter(<=1kb)	41	OR51J1	79470
469840	11p	5422212	5423123	912	10	0.7	Promoter(<=1kb)	101	OR51Q1	390061
469840	11p	5515185	5515931	747	6	0.333	Promoter(<=1kb)	768	UBQLNL	143630
469840	11p	5581045	5581738	694	8	0.375	Promoter(<=1kb)	168	OR52B6	340980
469840	11p	5884818	5885061	244	6	0.333	Promoter(<=1kb)	547	OR52E4	390081
469840	11p	11352040	11352736	697	8	0.25	Promoter(<=1kb)	514	CSNK2A3	283106
469840	11p	12293639	12294368	730	6	0.833	Exon(exon29of35)	6739	MICALCL	84953
469840	11p	34916266	34916763	498	6	0.667	Promoter(<=1kb)	0	APIP	51074
469840	11q	58214757	58215722	966	8	0.25	Promoter(<=1kb)	12	OR1S1	219959
469840	11q	58402523	58403265	743	8	0.5	Promoter(<=1kb)	144	OR5B3	441608
469840	11q	85724687	85725825	1139	6	0.5	Promoter(<=1kb)	0	SYTL2	54843
469840	11q	123906790	123907324	535	6	0.667	Promoter(<=1kb)	644	OR8D4	338662
469840	11q	123943088	123943782	695	7	0.571	Promoter(<=1kb)	91	OR6T1	219874
469840	11q	124015601	124016477	877	6	0.167	Promoter(<=1kb)	26	OR10G4	390264
469840	11q	124023038	124023849	812	7	0.429	Promoter(<=1kb)	25	OR10G9	219870
469840	11q	124264847	124265676	830	9	0.778	Promoter(<=1kb)	20	OR8G5	219865
469840	11q	124382526	124383285	760	8	0.625	Promoter(<=1kb)	58	OR8B2	26595
469840	12p	4626568	4628549	1982	11	0.455	Exon(exon5of6)	14051	DYRK4	8798
469840	12q	50352028	50353222	1195	13	0.462	Promoter(1-2kb)	-1143	FAM186A	121006
469840	12q	52571389	52573652	2264	7	0.571	Promoter(<=1kb)	164	KRT74	121391
469840	13q	24434450	24435347	898	7	0.571	Exon(exon31of34)	19787	PARP4	143
469840	13q	24446725	24447185	461	6	0.5	Exon(exon26of34)	7949	PARP4	143
469840	13q	25096713	25097231	519	9	0.444	Promoter(<=1kb)	845	PABPC3	5042
469840	13q	102732474	102733933	1460	6	0.333	Exon(exon4of4)	25139	CCDC168	643677
469840	14q	20060048	20060884	837	8	0.625	Promoter(<=1kb)	3	OR4L1	122742
469840	14q	21634177	21634589	413	6	0.833	Promoter(<=1kb)	351	OR10G2	26534
469840	14q	44504986	44506403	1418	6	0.667	Promoter(<=1kb)	880	FSCB	84075
469840	15q	23440160	23442067	1908	10	0.6	5'UTR	5167	GOLGA6L2	283685
469840	15q	78766033	78766671	639	6	0.333	Promoter(<=1kb)	-920	ADAMTS7	11173
469840	15q	88854874	88855400	527	6	0.833	Exon(exon12of18)	7631	ACAN	176
469840	15q	88857108	88859365	2258	6	0	Exon(exon12of18)	9865	ACAN	176
469840	16p	1256354	1256985	632	11	0.636	Promoter(<=1kb)	285	TPSD1	23430
469840	16p	1486371	1488463	2093	8	0.75	Promoter(<=1kb)	4	PTX4	390667
469840	16q	74391650	74391897	248	9	0.778	Exon(exon7of7)	13772	NPIPB15	440348
469840	16q	88428339	88429600	1262	6	0.333	Exon(exon3of3)	-23680	ZFPM1	161882
469840	16q	88714632	88717113	2482	7	0.429	Promoter(<=1kb)	0	MIR4722	100616167
469840	16q	89100686	89101050	365	8	0.625	Promoter(<=1kb)	24	ACSF3	197322
469840	16q	89226863	89228289	1427	7	0.571	Promoter(2-3kb)	2229	ZNF778	197320
469840	17p	744946	746695	1750	6	0.833	3'UTR	5343	GEMIN4	50628
469840	17p	7482135	7482961	827	8	0.75	Promoter(<=1kb)	803	SLC35G6	643664
469840	17p	21300581	21300978	398	12	0.75	3'UTR	9112	MAP2K3	5606
469840	17p	21415458	21416431	974	11	0.818	Exon(exon3of3)	10322	KCNJ12	3768
469840	17q	53823368	53824891	1524	6	0.833	Promoter(<=1kb)	441	KIF2B	84643
469840	17q	73236508	73236991	484	6	0.333	Promoter(<=1kb)	0	FAM104A	84923
469840	17q	81645135	81645417	283	6	0.333	Promoter(2-3kb)	2722	TSPAN10	83882
469840	18q	58535186	58537515	2330	9	0.333	Promoter(<=1kb)	0	ALPK2	115701
469840	19p	4510548	4513547	3000	16	0.438	Exon(exon3of6)	4157	PLIN4	729359
469840	19p	5455600	5456439	840	6	0.5	Promoter(<=1kb)	183	ZNRF4	148066
469840	19p	8111125	8112061	937	6	0.833	Promoter(<=1kb)	0	FBN3	84467
469840	19p	8946313	8951868	5556	15	0.6	Exon(exon3of84)	29474	MUC16	94025
469840	19p	8959116	8962299	3184	10	0.7	Exon(exon3of84)	19043	MUC16	94025
469840	19p	8972467	8978096	5630	17	0.471	Exon(exon1of84)	3246	MUC16	94025
469840	19p	15087213	15088040	828	10	0.3	Promoter(<=1kb)	233	OR1I1	126370
469840	19p	18264798	18267409	2612	12	0.583	5'UTR	7002	IQCN	80726
469840	19p	21971930	21974500	2571	7	0.714	Exon(exon4of4)	14408	ZNF208	7757
469840	19p	23743906	23745300	1395	6	0.333	Exon(exon4of4)	13537	ZNF681	148213
469840	19q	39877222	39877880	659	6	0.5	Exon(exon20of28)	9412	FCGBP	8857
469840	19q	39886005	39886439	435	7	0.571	Promoter(<=1kb)	853	FCGBP	8857
469840	19q	43846955	43848536	1582	6	0.833	3'UTR	13450	ZNF283	284349
469840	19q	43913423	43914878	1456	8	0.5	Exon(exon10of10)	4861	ZNF45	7596
469840	19q	43966037	43967171	1135	6	0.167	Promoter(<=1kb)	-691	ZNF155	7711
469840	19q	44106512	44108078	1567	7	0	Exon(exon6of6)	-4103	ZNF225	7768
469840	19q	55911888	55913077	1190	6	0.667	Exon(exon5of12)	19234	NLRP13	126204
469840	20p	5922577	5923643	1067	9	0.667	Exon(exon4of5)	7079	CHGB	1114
469840	20p	20052354	20052736	383	6	0.167	Promoter(<=1kb)	0	CFAP61	26074
469840	20q	63561666	63565531	3866	14	0.643	Promoter(<=1kb)	-61	HELZ2	85441
469840	21q	26843740	26844859	1120	6	0.667	Promoter(<=1kb)	0	ADAMTS1	9510
469840	21q	44550835	44551416	582	6	0.833	Promoter(<=1kb)	89	KRTAP10-2	386679
469840	22q	22352950	22353348	399	14	0.571	Exon(exon1of2)	30478	BMS1P20	96610
469840	22q	36191154	36191906	753	6	0.667	3'UTR	9971	APOL4	80832

469840	22q	49884187	49884994	808	6	0.333	Exon(exon2of2)	22924	ALG12	79087
469840	23p	6533793	6533828	36	6	1	Promoter(1-2kb)	1290	VCX3A	51481
469840	23p	8170039	8170141	103	6	0.5	Promoter(1-2kb)	1126	VCX2	51480
469840	23p	35802148	35803010	863	7	0.571	5'UTR	3357	MAGEB16	139604
469840	23q	136874183	136874347	165	7	0.714	Promoter(<=1kb)	-201	RBMX	27316
470505	1p	11766028	11768307	2280	6	0.833	Promoter(<=1kb)	0	C1orf167	284498
470505	1p	13370686	13370989	304	8	0.5	Promoter(<=1kb)	911	PRAMEF19	645414
470505	1p	16058491	16059952	1462	7	0.857	Exon(exon5of7)	6168	CLCNKB	1188
470505	1p	23874604	23875430	827	8	0.5	Exon(exon2of2)	-6310	FUCA1	2517
470505	1p	36098176	36100138	1963	6	1	Promoter(<=1kb)	111	COL8A2	1296
470505	1p	40067594	40067675	82	6	0	Promoter(<=1kb)	324	CAP1	10487
470505	1q	152218469	152221071	2603	8	0.375	Exon(exon3of3)	3122	HRNR	388697
470505	1q	152302977	152304920	1944	8	0.5	Exon(exon3of3)	-7656	FLG-AS1	339400
470505	1q	152306380	152313891	7512	29	0.517	Promoter(<=1kb)	0	FLG-AS1	339400
470505	1q	156669844	156671817	1974	8	1	Exon(exon4of4)	5590	NES	10763
470505	1q	201206099	201209738	3640	10	0.6	Promoter(1-2kb)	1017	IGFN1	91156
470505	1q	214640144	214642954	2811	12	0.5	Exon(exon12of20)	-5013	CENPF	1063
470505	1q	214644872	214647181	2310	10	0.4	Promoter(<=1kb)	-786	CENPF	1063
470505	1q	222628664	222629929	1266	6	0.333	Promoter(<=1kb)	190	MIA3	375056
470505	1q	228315976	228318038	2063	8	0.625	Exon(exon50of81)	6492	OBSCN	84033
470505	1q	247841312	247841582	271	7	0.857	Promoter(<=1kb)	314	OR11L1	391189
470505	1q	247949443	247949738	296	9	0.333	Promoter(<=1kb)	585	OR2L8	391190
470505	1q	248294677	248295458	782	7	0.857	Promoter(<=1kb)	142	OR2T12	127064
470505	2p	48580657	48582454	1798	7	0.571	Promoter(<=1kb)	0	STON1	11037
470505	2q	132783032	132785012	1981	10	0.6	Promoter(1-2kb)	-1009	NCKAP5	344148
470505	2q	178739433	178741811	2379	6	0.5	Exon(exon45of191)	26014	TTN	7273
470505	2q	238130416	238131546	1131	6	0.333	Promoter(1-2kb)	1468	ESPNL	339768
470505	3p	75736880	75739243	2364	52	0.596	Promoter(<=1kb)	0	MIR4273	100422955
470505	3q	98264413	98265098	686	9	0.556	Promoter(<=1kb)	128	OR5H6	79295
470505	4p	5988383	5989749	1367	8	0.5	Promoter(<=1kb)	0	C4orf50	389197
470505	4p	6300792	6302360	1569	7	0.714	Exon(exon8of8)	6021	WFS1	7466
470505	4p	8227004	8228508	1505	8	0.125	Promoter(<=1kb)	-24	SH3TC1	54436
470505	4q	121036404	121037542	1139	6	0.333	Promoter(1-2kb)	1442	NDNF	79625
470505	4q	154489498	154491312	1815	9	0.556	Promoter(<=1kb)	22	DCHS2	54798
470505	4q	185458217	185460011	1795	9	0.556	Promoter(<=1kb)	0	CCDC110	256309
470505	5q	79728956	79730716	1761	7	0.286	Exon(exon2of13)	-7426	CMYA5	202333
470505	5q	79731782	79734523	2742	13	0.308	Exon(exon2of13)	-3619	CMYA5	202333
470505	5q	83537326	83539905	2580	6	0.333	Promoter(1-2kb)	1712	VCAN	1462
470505	5q	141174000	141175025	1026	6	0.833	Promoter(1-2kb)	1356	PCDHB7	56129
470505	5q	151521550	151522069	520	6	0.667	Promoter(<=1kb)	79	MIR6499	102465246
470505	5q	151565922	151568158	2237	9	0.778	Promoter(<=1kb)	786	FAT2	2196
470505	6p	1312843	1313745	903	6	0.5	Promoter(<=1kb)	745	FOXQ1	94234
470505	6p	42745312	42746041	730	6	0.667	Promoter(<=1kb)	62	TBCC	6903
470505	6p	46858771	46859502	732	8	0.5	Exon(exon17of21)	3802	ADGRF5	221395
470505	6q	149888581	149890867	2287	7	0.714	Promoter(<=1kb)	0	RAET1E-AS1	100652739
470505	6q	159231899	159234370	2472	12	0.583	Exon(exon11of23)	13602	FNDC1	84624
470505	7q	100958721	100960873	2153	58	0.431	Promoter(<=1kb)	756	MUC3A	4584
470505	7q	101034305	101038481	4177	25	0.52	Exon(exon3of12)	-5230	MUC17	140453
470505	8p	10607245	10608432	1188	8	0.5	Exon(exon4of4)	46711	RP1L1	94137
470505	8p	10609614	10610662	1049	8	0.5	Exon(exon4of4)	44481	RP1L1	94137
470505	8p	12132529	12133940	1412	10	0.7	Promoter(<=1kb)	498	USP17L7	392197
470505	8p	12137448	12138641	1194	6	0.833	Promoter(<=1kb)	436	USP17L2	377630
470505	8p	13021128	13022030	903	7	0.143	Exon(exon5of5)	9115	TRMT9B	57604
470505	8q	123651873	123652634	762	6	0.333	Promoter(<=1kb)	316	KLHL38	340359
470505	8q	141466455	141467514	1060	8	0.75	3'UTR	29245	MROH5	389690
470505	9q	76705179	76707804	2626	8	0.5	Promoter(<=1kb)	121	PCA3	50652
470505	9q	76709263	76710846	1584	9	0.556	Promoter(<=1kb)	0	PRUNE2	158471
470505	9q	87886533	87888536	2004	7	0.571	Exon(exon4of4)	3656	SPATA31E1	286234
470505	9q	104694482	104695444	963	6	0.5	Promoter(<=1kb)	0	OR13D1	286365
470505	9q	122567846	122568473	628	6	0.5	Promoter(<=1kb)	86	OR1L8	138881
470505	9q	133255635	133256205	571	11	0.909	3'UTR	19009	ABO	28
470505	9q	135484803	135487156	2354	7	0.143	Promoter(1-2kb)	1440	PPP1R26	9858
470505	10q	46549378	46550723	1346	26	0.654	Exon(exon3of3)	4807	GPRIN2	9721
470505	10q	128103129	128108204	5076	15	0.667	Promoter(<=1kb)	-1	MKI67	4288
470505	11p	244106	244197	92	8	0.5	Promoter(<=1kb)	-232	PSMD13	5719
470505	11p	5177978	5178478	501	6	0.167	Promoter(<=1kb)	186	OR52Z1	283110
470505	11p	5323451	5324256	806	6	0.5	Promoter(<=1kb)	41	OR51B2	79345
470505	11p	5389704	5390350	647	6	0.5	Promoter(<=1kb)	327	OR51M1	390059
470505	11p	5422212	5423123	912	11	0.636	Promoter(<=1kb)	101	OR51Q1	390061
470505	11p	5440761	5441472	712	6	0.833	Promoter(<=1kb)	42	OR51I1	390063
470505	11p	5515185	5516015	831	6	0.333	Promoter(<=1kb)	684	UBQLNL	143630
470505	11p	5581045	5581738	694	8	0.375	Promoter(<=1kb)	168	OR52B6	340980
470505	11p	5841302	5841883	582	9	0.333	Promoter(<=1kb)	14	OR52E6	390078
470505	11p	5884818	5885061	244	7	0.429	Promoter(<=1kb)	547	OR52E4	390081
470505	11p	11352040	11352736	697	8	0.25	Promoter(<=1kb)	514	CSNK2A3	283106
470505	11p	12293639	12294368	730	6	0.833	Exon(exon29of35)	6739	MICALCL	84953
470505	11p	34916266	34916763	498	6	0.667	Promoter(<=1kb)	0	APIP	51074

470505	11p	43942293	43943348	1056	8	0.75	Promoter(<=1kb)	0	C11orf96	387763
470505	11q	58214757	58215722	966	7	0.286	Promoter(<=1kb)	12	OR1S1	219959
470505	11q	58402523	58403265	743	8	0.5	Promoter(<=1kb)	144	OR5B3	441608
470505	11q	82732630	82733184	555	6	0.833	Promoter(<=1kb)	680	FAM181B	220382
470505	11q	123906790	123907324	535	6	0.667	Promoter(<=1kb)	644	OR8D4	338662
470505	11q	124038366	124038988	623	8	0.875	Promoter(<=1kb)	13	OR10G7	390265
470505	11q	124382526	124383285	760	7	0.571	Promoter(<=1kb)	58	OR8B2	26595
470505	12p	4626571	4628549	1979	10	0.4	Exon(exon5of6)	14054	DYRK4	8798
470505	12p	6453119	6453670	552	6	0.667	Promoter(<=1kb)	633	TAPBPL	55080
470505	12p	8222174	8223514	1341	6	0.667	Exon(exon5of6)	4073	FAM90A1	55138
470505	13q	102732474	102733933	1460	6	0.333	Exon(exon4of4)	25139	CCDC168	643677
470505	14q	20060048	20060884	837	8	0.625	Promoter(<=1kb)	3	OR4L1	122742
470505	14q	44504986	44506403	1418	6	0.667	Promoter(<=1kb)	880	FSCB	84075
470505	14q	70457733	70458385	653	11	0.455	Exon(exon2of2)	5559	ADAM21	8747
470505	14q	94587512	94587839	328	6	0.5	Exon(exon2of2)	-4219	SERPINA3	12
470505	14q	104177072	104177810	739	6	0.333	Exon(exon12of15)	38032	KIF26A	26153
470505	14q	104939262	104942618	3357	10	0.2	5'UTR	7102	PLD4	122618
470505	14q	104943622	104945444	1823	6	0.667	Exon(exon6of6)	9958	AHNAK2	113146
470505	14q	104948292	104953878	5587	25	0.48	Promoter(1-2kb)	1524	AHNAK2	113146
470505	15q	23440370	23442067	1698	9	0.667	5'UTR	5167	GOLGA6L2	283685
470505	15q	40621642	40624434	2793	8	0.375	Promoter(<=1kb)	0	KNL1	57082
470505	15q	52609086	52609780	695	6	0.333	Promoter(<=1kb)	-490	FAM214A	56204
470505	15q	78766033	78766626	594	6	0.333	Promoter(<=1kb)	-920	ADAMTS7	11173
470505	15q	85579423	85581800	2378	14	0.643	Promoter(1-2kb)	-1110	AKAP13	11214
470505	15q	88856849	88859365	2517	7	0	Exon(exon12of18)	9606	ACAN	176
470505	16p	1256354	1256985	632	11	0.636	Promoter(<=1kb)	285	TPSD1	23430
470505	16p	1486371	1488463	2093	8	0.75	Promoter(<=1kb)	4	PTX4	390667
470505	16p	27362551	27363079	529	6	0.167	3'UTR	7203	IL4R	3566
470505	16q	74391549	74391897	349	7	0.714	Exon(exon7of7)	13671	NPIPB15	440348
470505	16q	88430623	88431977	1355	6	0.5	Exon(exon3of3)	-21303	ZFPM1	161882
470505	16q	89100686	89101050	365	8	0.625	Promoter(<=1kb)	24	ACSF3	197322
470505	16q	89226863	89228419	1557	9	0.556	Promoter(2-3kb)	2229	ZNF778	197320
470505	17p	10638198	10641099	2902	7	0.286	Exon(exon19of41)	-8169	MYH3	4621
470505	17p	21300581	21300978	398	11	0.727	3'UTR	9112	MAP2K3	5606
470505	17p	21415458	21416431	974	11	0.818	Exon(exon3of3)	10322	KCNJ12	3768
470505	17q	81645135	81645607	473	7	0.286	Promoter(2-3kb)	2722	TSPAN10	83882
470505	18p	11609728	11610122	395	8	0.625	Promoter(<=1kb)	132	SLC35G4	646000
470505	18q	58535186	58537515	2330	9	0.333	Promoter(<=1kb)	0	ALPK2	115701
470505	19p	4510548	4513547	3000	19	0.368	Exon(exon3of6)	4157	PLIN4	729359
470505	19p	5455600	5456439	840	6	0.5	Promoter(<=1kb)	183	ZNRF4	148066
470505	19p	8937644	8939234	1591	6	0.833	Exon(exon5of84)	-41554	MUC16	94025
470505	19p	8946306	8951868	5563	19	0.684	Exon(exon3of84)	29474	MUC16	94025
470505	19p	8959116	8962299	3184	12	0.583	Exon(exon3of84)	19043	MUC16	94025
470505	19p	8963397	8967127	3731	21	0.667	Exon(exon3of84)	14215	MUC16	94025
470505	19p	8972751	8978096	5346	13	0.462	Exon(exon1of84)	3246	MUC16	94025
470505	19p	12430718	12431840	1123	6	0.5	3'UTR	9181	ZNF443	10224
470505	19p	17281820	17284246	2427	10	0.5	Promoter(<=1kb)	0	ANKLE1	126549
470505	19p	18264753	18267409	2657	14	0.643	5'UTR	7002	IQCN	80726
470505	19p	21971930	21974500	2571	10	0.7	Exon(exon4of4)	14408	ZNF208	7757
470505	19q	36996730	36997597	868	10	0.6	Exon(exon10of10)	5677	ZNF568	374900
470505	19q	39877222	39877880	659	7	0.429	Exon(exon20of28)	9412	FCGBP	8857
470505	19q	43913423	43914878	1456	8	0.5	Exon(exon10of10)	4861	ZNF45	7596
470505	19q	43996323	43997366	1044	6	0.333	Exon(exon5of5)	5419	LOC10192806	101928063
470505	19q	44106512	44108078	1567	9	0.111	Exon(exon6of6)	-4103	ZNF225	7768
470505	19q	44327836	44329698	1863	6	0.5	Exon(exon4of4)	-22790	ZNF235	9310
470505	19q	52384029	52384992	964	7	0.143	Exon(exon4of4)	12677	ZNF528-AS1	102724105
470505	19q	52437918	52439242	1325	7	0.429	Exon(exon4of4)	6504	ZNF534	147658
470505	19q	55481625	55483456	1832	7	0.429	Promoter(1-2kb)	-1732	NAT14	57106
470505	19q	55517821	55518642	822	8	1	Exon(exon14of14)	17661	SBK2	646643
470505	19q	57444351	57445460	1110	6	0.667	Exon(exon3of3)	9004	ZNF749	388567
470505	20p	5922421	5923394	974	6	0.5	Exon(exon4of5)	6923	CHGB	1114
470505	20q	63561666	63565531	3866	11	0.636	Promoter(<=1kb)	-61	HELZ2	85441
470505	21q	44539312	44540035	724	7	0.714	Promoter(<=1kb)	160	KRTAP10-1	386677
470505	21q	44600627	44601692	1066	9	0.778	Promoter(<=1kb)	30	KRTAP10-7	386675
470505	22q	22352950	22353365	416	15	0.533	Exon(exon1of2)	30478	BMS1P20	96610
470505	22q	36191154	36191906	753	6	0.667	3'UTR	9971	APOL4	80832
470505	23p	8170039	8170141	103	6	0.5	Promoter(1-2kb)	1126	VCX2	51480
470505	23q	136874183	136874347	165	7	0.714	Promoter(<=1kb)	-201	RBMX	27316
470505	23q	141906066	141906494	429	7	0.571	Promoter(1-2kb)	1264	MAGEC1	9947
471413	1p	18481403	18482217	815	6	0.667	Promoter(<=1kb)	421	KLHDC7A	127707
471413	1p	40067594	40067675	82	6	0	Promoter(<=1kb)	324	CAP1	10487
471413	1p	88983230	88983751	522	7	0.429	Exon(exon3of3)	4604	KYAT3	56267
471413	1p	89186388	89186414	27	8	0.625	Promoter(<=1kb)	112	GBP4	115361
471413	1q	152303673	152305138	1466	8	0.5	Exon(exon3of3)	-7438	FLG-AS1	339400
471413	1q	152306380	152313891	7512	28	0.536	Promoter(<=1kb)	0	FLG-AS1	339400
471413	1q	158765805	158766655	851	6	0.5	Promoter(<=1kb)	47	OR6N1	128372
471413	1q	169542317	169542882	566	7	0.143	Exon(exon13of25)	-26572	F5	2153

471413	1q	226735563	226737239	1677	6	0.833	Promoter(<=1kb)	219	ITPKB	3707
471413	1q	247841312	247841582	271	6	0.833	Promoter(<=1kb)	314	OR11L1	391189
471413	1q	247895625	247896502	878	7	0.571	Promoter(<=1kb)	38	OR2W3	343171
471413	1q	247949436	247949759	324	11	0.455	Promoter(<=1kb)	578	OR2L8	391190
471413	1q	248573992	248574210	219	6	0.833	Promoter(<=1kb)	547	OR2T34	127068
471413	1q	248681658	248682198	541	7	0.571	Promoter(<=1kb)	130	OR14I1	401994
471413	2q	102351547	102351902	356	7	0.429	Exon(exon11of11)	-4027	IL18R1	8809
471413	2q	132783032	132785012	1981	9	0.556	Promoter(1-2kb)	-1009	NCKAP5	344148
471413	2q	167246794	167248478	1685	8	0.625	Promoter(<=1kb)	-204	XIRP2	129446
471413	2q	178739433	178741811	2379	6	0.5	Exon(exon45of191)	26014	TTN	7273
471413	2q	185789865	185794632	4768	10	0.8	Promoter(<=1kb)	0	FSIP2	401024
471413	2q	217847583	217848746	1164	7	0.857	Exon(exon19of33)	-5423	TNS1	7145
471413	2q	233713134	233713783	650	13	0.692	Promoter(<=1kb)	142	UGT1A5	54579
471413	2q	235041233	235041640	408	6	0.333	Exon(exon4of6)	46393	SH3BP4	23677
471413	2q	238130416	238131546	1131	6	0.333	Promoter(1-2kb)	1468	ESPNL	339768
471413	3p	31989532	31990905	1374	6	0.333	Exon(exon2of2)	7761	ZNF860	344787
471413	3q	98169021	98169561	541	6	0.5	Exon(exon2of2)	19695	OR5H14	403273
471413	4p	5988383	5989749	1367	7	0.571	Promoter(<=1kb)	0	C4orf50	389197
471413	4p	6300792	6302360	1569	7	0.857	Exon(exon8of8)	6021	WFS1	7466
471413	4p	10443803	10446224	2422	6	0.333	Exon(exon3of3)	10952	ZNF518B	85460
471413	4q	121036404	121037542	1139	6	0.333	Promoter(1-2kb)	1442	NDNF	79625
471413	5p	795818	796237	420	6	0.667	3'UTR	4908	ZDHHC11	79844
471413	5q	83537326	83539905	2580	6	0.333	Promoter(1-2kb)	1712	VCAN	1462
471413	5q	140848579	140850786	2208	7	0.571	Promoter(<=1kb)	807	PCDHA9	9752
471413	5q	141174000	141175025	1026	7	0.714	Promoter(1-2kb)	1356	PCDHB7	56129
471413	5q	160565578	160565764	187	6	0.5	Exon(exon21of21)	37527	ATP10B	23120
471413	6p	1312843	1313745	903	6	0.5	Promoter(<=1kb)	745	FOXQ1	94234
471413	6p	42745312	42746041	730	6	0.667	Promoter(<=1kb)	62	TBCC	6903
471413	6p	46858771	46859502	732	7	0.571	Exon(exon17of21)	3802	ADGRF5	221395
471413	6q	64591274	64591961	688	10	0.5	Exon(exon26of43)	121374	EYS	346007
471413	6q	159231899	159234370	2472	12	0.583	Exon(exon11of23)	13602	FNDC1	84624
471413	7p	53035678	53036385	708	8	0.875	Promoter(<=1kb)	45	POM121L12	285877
471413	7q	100958721	100960873	2153	60	0.433	Promoter(<=1kb)	756	MUC3A	4584
471413	7q	100990920	100992398	1479	9	0.667	Exon(exon5of15)	-20656	MUC12	10071
471413	7q	100995575	100995896	322	8	0.625	Exon(exon5of15)	-17158	MUC12	10071
471413	7q	149818015	149819792	1778	6	0.667	Promoter(2-3kb)	-2352	SSPO	23145
471413	8p	10609614	10612307	2694	11	0.909	Exon(exon4of4)	42836	RP1L1	94137
471413	8p	12137448	12138641	1194	6	0.833	Promoter(<=1kb)	436	USP17L2	377630
471413	8p	13021128	13022030	903	8	0.125	Exon(exon5of5)	9115	TRMT9B	57604
471413	8q	123651655	123652634	980	7	0.571	Promoter(<=1kb)	316	KLHL38	340359
471413	8q	141466429	141467514	1086	9	0.778	3'UTR	29245	MROH5	389690
471413	8q	143916360	143919209	2850	7	0.143	Exon(exon32of32)	20381	PLEC	5339
471413	9q	76175237	76175271	35	8	0.875	Exon(exon14of14)	-13368	PCSK5	5125
471413	9q	76703555	76706360	2806	8	0.5	Promoter(<=1kb)	0	PCA3	50652
471413	9q	122553263	122554071	809	8	0.5	Promoter(<=1kb)	93	OR1N2	138882
471413	9q	122628595	122629398	804	7	0.286	Promoter(<=1kb)	175	OR1B1	347169
471413	9q	133255635	133256208	574	12	0.917	3'UTR	19006	ABO	28
471413	9q	135484803	135487213	2411	8	0.25	Promoter(1-2kb)	1440	PPP1R26	9858
471413	10q	46549378	46550723	1346	25	0.64	Exon(exon3of3)	4807	GPRIN2	9721
471413	10q	49323504	49326817	3314	10	0.5	Exon(exon3of3)	24230	C10orf71	118461
471413	10q	128102594	128106296	3703	8	0.625	Promoter(<=1kb)	0	MKI67	4288
471413	11p	244106	244197	92	8	0.5	Promoter(<=1kb)	-232	PSMD13	5719
471413	11p	308290	309148	859	6	0.5	Promoter(<=1kb)	0	IFITM2	10581
471413	11p	1194354	1196902	2549	7	0.571	Exon(exon34of49)	-26164	MUC5B	727897
471413	11p	1250091	1251524	1434	7	0.714	Promoter(<=1kb)	-415	MUC5B-AS1	112577518
471413	11p	5177978	5178478	501	6	0.167	Promoter(<=1kb)	186	OR52Z1	283110
471413	11p	5515185	5515931	747	6	0.333	Promoter(<=1kb)	768	UBQLNL	143630
471413	11p	5581045	5581738	694	8	0.375	Promoter(<=1kb)	168	OR52B6	340980
471413	11p	5755023	5755693	671	6	0.5	Promoter(<=1kb)	330	OR52N4	390072
471413	11p	5986042	5986669	628	7	1	Promoter(<=1kb)	316	OR52L1	338751
471413	11p	11352040	11352736	697	6	0.167	Promoter(<=1kb)	514	CSNK2A3	283106
471413	11p	12293639	12294842	1204	7	0.714	Exon(exon29of35)	6739	MICALCL	84953
471413	11p	43942293	43943348	1056	8	0.75	Promoter(<=1kb)	0	C11orf96	387763
471413	11q	58214757	58215722	966	7	0.286	Promoter(<=1kb)	12	OR1S1	219959
471413	11q	123906790	123907324	535	6	0.667	Promoter(<=1kb)	644	OR8D4	338662
471413	11q	124038366	124038988	623	7	1	Promoter(<=1kb)	13	OR10G7	390265
471413	11q	124264847	124265676	830	6	0.833	Promoter(<=1kb)	20	OR8G5	219865
471413	12p	4627329	4628549	1221	8	0.625	Exon(exon5of6)	14812	DYRK4	8798
471413	12p	6453119	6453670	552	6	0.667	Promoter(<=1kb)	633	TAPBPL	55080
471413	13q	25096659	25097231	573	9	0.556	Promoter(<=1kb)	791	PABPC3	5042
471413	13q	102732474	102733933	1460	6	0.333	Exon(exon4of4)	25139	CCDC168	643677
471413	14q	20640982	20641567	586	6	0.5	Promoter(<=1kb)	124	OR6S1	341799
471413	14q	21634177	21634589	413	6	0.833	Promoter(<=1kb)	351	OR10G2	26534
471413	14q	70457532	70457980	449	6	0.333	Exon(exon2of2)	5358	ADAM21	8747
471413	14q	94587512	94587839	328	6	0.5	Exon(exon2of2)	-4219	SERPINA3	12
471413	14q	104939262	104942618	3357	10	0.2	5'UTR	7102	PLD4	122618
471413	14q	104943622	104945444	1823	6	0.667	Exon(exon6of6)	9958	AHNAK2	113146

471413	14q	104947943	104953878	5936	41	0.512	Promoter(1-2kb)	1524	AHNAK2	113146
471413	15q	23440370	23442067	1698	9	0.667	5'UTR	5167	GOLGA6L2	283685
471413	15q	40621642	40623696	2055	6	0.333	Promoter(<=1kb)	0	KNL1	57082
471413	15q	85579423	85581800	2378	14	0.571	Promoter(1-2kb)	-1110	AKAP13	11214
471413	16p	669592	672548	2957	7	0.571	Promoter(<=1kb)	0	RHOT2	89941
471413	16p	1229573	1229716	144	7	0.571	Promoter(<=1kb)	446	TPSB2	64499
471413	16p	1256354	1256985	632	12	0.667	Promoter(<=1kb)	285	TPSD1	23430
471413	16p	4207130	4208004	875	6	0.5	Exon(exon2of7)	31739	SRL	6345
471413	16p	4883938	4885635	1698	6	1	Exon(exon22of22)	4701	PPL	5493
471413	16q	74391650	74391897	248	9	0.778	Exon(exon7of7)	13772	NPIPB15	440348
471413	16q	89100686	89101050	365	7	0.571	Promoter(<=1kb)	24	ACSF3	197322
471413	16q	89226863	89228289	1427	8	0.625	Promoter(2-3kb)	2229	ZNF778	197320
471413	17p	744917	746695	1779	7	0.857	3'UTR	5343	GEMIN4	50628
471413	17p	21300581	21300978	398	12	0.75	3'UTR	9112	MAP2K3	5606
471413	17p	21415458	21416416	959	10	0.9	Exon(exon3of3)	10322	KCNJ12	3768
471413	17q	76293419	76294016	598	6	0.5	Promoter(2-3kb)	-2167	QRICH2	84074
471413	17q	81645135	81645595	461	7	0.429	Promoter(2-3kb)	2722	TSPAN10	83882
471413	18q	58535186	58537515	2330	10	0.4	Promoter(<=1kb)	0	ALPK2	115701
471413	19p	4510548	4513547	3000	17	0.412	Exon(exon3of6)	4157	PLIN4	729359
471413	19p	5455600	5456439	840	7	0.571	Promoter(<=1kb)	183	ZNRF4	148066
471413	19p	8948231	8951868	3638	11	0.545	Exon(exon3of84)	29474	MUC16	94025
471413	19p	18264798	18267409	2612	12	0.583	5'UTR	7002	IQCN	80726
471413	19p	22757129	22759523	2395	8	0.625	3'UTR	10459	ZNF99	7652
471413	19q	36996730	36997597	868	10	0.6	Exon(exon10of10)	5677	ZNF568	374900
471413	19q	39877222	39877880	659	6	0.5	Exon(exon20of28)	9412	FCGBP	8857
471413	19q	39886005	39886439	435	8	0.5	Promoter(<=1kb)	853	FCGBP	8857
471413	19q	43913423	43914878	1456	9	0.556	Exon(exon10of10)	4861	ZNF45	7596
471413	19q	44106512	44108078	1567	7	0	Exon(exon6of6)	-4103	ZNF225	7768
471413	19q	44327836	44329698	1863	6	0.5	Exon(exon4of4)	-22790	ZNF235	9310
471413	19q	52437918	52439242	1325	7	0.429	Exon(exon4of4)	6504	ZNF534	147658
471413	19q	55911888	55913166	1279	7	0.714	Exon(exon5of12)	19145	NLRP13	126204
471413	19q	58368293	58368875	583	7	0.429	Exon(exon3of3)	-5445	ZNF497	162968
471413	20p	5922677	5923382	706	6	0.5	Exon(exon4of5)	7179	CHGB	1114
471413	20q	53575869	53577159	1291	6	0.833	Promoter(<=1kb)	0	ZNF217	7764
471413	20q	63561666	63565531	3866	12	0.667	Promoter(<=1kb)	-61	HELZ2	85441
471413	21q	44550835	44551416	582	7	0.857	Promoter(<=1kb)	89	KRTAP10-2	386679
471413	22q	22352950	22353348	399	14	0.571	Exon(exon1of2)	30478	BMS1P20	96610
471413	22q	36191154	36191906	753	7	0.714	3'UTR	9971	APOL4	80832
471413	23p	3320126	3323750	3625	10	0.6	Exon(exon5of7)	22902	MXRA5	25878
471413	23p	8170039	8170141	103	6	0.5	Promoter(1-2kb)	1126	VCX2	51480
471413	23p	35802148	35803010	863	7	0.571	5'UTR	3357	MAGEB16	139604
471413	23q	115190837	115192586	1750	6	0.667	Promoter(1-2kb)	1410	RBMXL3	139804
472286	1p	11766028	11768307	2280	6	0.833	Promoter(<=1kb)	0	C1orf167	284498
472286	1p	11778784	11779941	1158	7	0.714	Promoter(<=1kb)	0	C1orf167-AS1	102724659
472286	1p	16058491	16060000	1510	10	0.9	Exon(exon5of7)	6168	CLCNKB	1188
472286	1p	18481403	18482217	815	7	0.714	Promoter(<=1kb)	421	KLHDC7A	127707
472286	1p	23874604	23875430	827	8	0.5	Exon(exon2of2)	-6310	FUCA1	2517
472286	1p	40067594	40067675	82	6	0	Promoter(<=1kb)	324	CAP1	10487
472286	1p	89186388	89186419	32	9	0.556	Promoter(<=1kb)	107	GBP4	115361
472286	1q	152213286	152213320	35	7	0.429	Exon(exon3of3)	10873	HRNR	388697
472286	1q	152219233	152221375	2143	14	0.714	Promoter(2-3kb)	2818	HRNR	388697
472286	1q	156669844	156671745	1902	7	1	Exon(exon4of4)	5662	NES	10763
472286	1q	158765805	158766655	851	6	0.5	Promoter(<=1kb)	47	OR6N1	128372
472286	1q	226735683	226737239	1557	7	0.857	Promoter(<=1kb)	219	ITPKB	3707
472286	1q	228315976	228318038	2063	8	0.625	Exon(exon50of81)	6492	OBSCN	84033
472286	1q	247841312	247841582	271	6	0.833	Promoter(<=1kb)	314	OR11L1	391189
472286	1q	247895625	247896502	878	7	0.571	Promoter(<=1kb)	38	OR2W3	343171
472286	1q	247949436	247949759	324	11	0.455	Promoter(<=1kb)	578	OR2L8	391190
472286	1q	248294677	248295458	782	6	0.667	Promoter(<=1kb)	142	OR2T12	127064
472286	2p	48580657	48582454	1798	7	0.571	Promoter(<=1kb)	0	STON1	11037
472286	2q	130914528	130917154	2627	6	0.333	Promoter(<=1kb)	0	ARHGEF4	50649
472286	2q	134986437	134988152	1716	6	0.667	Promoter(<=1kb)	0	MAP3K19	80122
472286	2q	184936178	184937636	1459	6	0.333	Exon(exon4of4)	69813	ZNF804A	91752
472286	2q	185789865	185794632	4768	10	0.8	Promoter(<=1kb)	0	FSIP2	401024
472286	2q	185805377	185808170	2794	6	0.333	Promoter(<=1kb)	0	FSIP2	401024
472286	2q	217847583	217848559	977	6	0.833	Exon(exon19of33)	-5423	TNS1	7145
472286	2q	238130416	238131546	1131	6	0.333	Promoter(1-2kb)	1468	ESPNL	339768
472286	3p	75736929	75739007	2079	14	0.643	Promoter(<=1kb)	0	MIR4273	100422955
472286	4p	5988383	5989749	1367	8	0.625	Promoter(<=1kb)	0	C4orf50	389197
472286	4p	6300792	6302360	1569	6	0.833	Exon(exon8of8)	6021	WFS1	7466
472286	4p	8227004	8228508	1505	8	0.125	Promoter(<=1kb)	-24	SH3TC1	54436
472286	4q	112431241	112432293	1053	7	0.429	3'UTR	4735	ALPK1	80216
472286	4q	186617117	186621582	4466	11	0.364	Exon(exon10of27)	-7047	FAT1	2195
472286	4q	186706638	186708616	1979	6	0.667	Exon(exon2of27)	15217	FAT1	2195
472286	5q	79728956	79730716	1761	8	0.25	Exon(exon2of13)	-7426	CMYA5	202333
472286	5q	79731782	79734523	2742	13	0.308	Exon(exon2of13)	-3619	CMYA5	202333
472286	5q	83537326	83539905	2580	6	0.333	Promoter(1-2kb)	1712	VCAN	1462

472286	5q	140801551	140803473	1923	6	0.5	Promoter(<=1kb)	494	PCDHA3	56145
472286	5q	141174000	141175025	1026	6	0.833	Promoter(1-2kb)	1356	PCDHB7	56129
472286	5q	141187690	141189425	1736	6	0.5	Promoter(<=1kb)	529	PCDHB9	56127
472286	5q	141955676	141957660	1985	6	0.667	Promoter(<=1kb)	-668	RNF14	9604
472286	6p	26370344	26370479	136	6	0.5	Promoter(<=1kb)	0	BTN3A2	11118
472286	6p	46858771	46859502	732	8	0.5	Exon(exon17of21)	3802	ADGRF5	221395
472286	6q	149888581	149889987	1407	6	0.667	Promoter(<=1kb)	-116	RAET1E-AS1	100652739
472286	6q	159233455	159234370	916	10	0.5	Exon(exon11of23)	15158	FNDC1	84624
472286	7p	45082865	45084866	2002	9	0.444	Promoter(1-2kb)	-1465	NACAD	23148
472286	7q	64991278	64992758	1481	6	0.667	Promoter(<=1kb)	-242	ZNF117	51351
472286	7q	100958721	100960873	2153	53	0.453	Promoter(<=1kb)	756	MUC3A	4584
472286	7q	100991195	100992398	1204	8	0.625	Exon(exon5of15)	-20656	MUC12	10071
472286	7q	149818015	149819792	1778	6	0.667	Promoter(2-3kb)	-2352	SSPO	23145
472286	8p	10607245	10608432	1188	8	0.5	Exon(exon4of4)	46711	RP1L1	94137
472286	8p	10609614	10612307	2694	9	0.889	Exon(exon4of4)	42836	RP1L1	94137
472286	8p	11331194	11332082	889	8	0.625	Promoter(<=1kb)	306	SLC35G5	83650
472286	8p	12132529	12133940	1412	9	0.667	Promoter(<=1kb)	498	USP17L7	392197
472286	8p	12137553	12138641	1089	6	1	Promoter(<=1kb)	436	USP17L2	377630
472286	8q	123651655	123652634	980	7	0.571	Promoter(<=1kb)	316	KLHL38	340359
472286	8q	143916360	143919209	2850	7	0.143	Exon(exon32of32)	20381	PLEC	5339
472286	9p	34723747	34726527	2781	7	0.714	Promoter(2-3kb)	2961	FAM205A	259308
472286	9q	87885490	87888819	3330	8	0.625	Promoter(2-3kb)	2613	SPATA31E1	286234
472286	9q	104504315	104505071	757	6	0.5	Promoter(<=1kb)	52	OR13F1	138805
472286	9q	122553263	122554071	809	8	0.5	Promoter(<=1kb)	93	OR1N2	138882
472286	9q	122628595	122629130	536	6	0.333	Promoter(<=1kb)	443	OR1B1	347169
472286	9q	135484803	135487213	2411	8	0.25	Promoter(1-2kb)	1440	PPP1R26	9858
472286	10q	46549378	46550723	1346	26	0.654	Exon(exon3of3)	4807	GPRIN2	9721
472286	11p	244106	244197	92	8	0.5	Promoter(<=1kb)	-232	PSMD13	5719
472286	11p	1249774	1251524	1751	8	0.75	Promoter(<=1kb)	-98	MUC5B-AS1	112577518
472286	11p	5046754	5047432	679	7	0.571	Promoter(<=1kb)	228	OR52J3	119679
472286	11p	5177978	5178478	501	6	0.167	Promoter(<=1kb)	186	OR52Z1	283110
472286	11p	5323451	5324256	806	6	0.5	Promoter(<=1kb)	41	OR51B2	79345
472286	11p	5389704	5390350	647	7	0.429	Promoter(<=1kb)	327	OR51M1	390059
472286	11p	5422212	5423123	912	10	0.7	Promoter(<=1kb)	101	OR51Q1	390061
472286	11p	5581045	5581738	694	8	0.375	Promoter(<=1kb)	168	OR52B6	340980
472286	11p	5841302	5841883	582	9	0.333	Promoter(<=1kb)	14	OR52E6	390078
472286	11p	11352040	11352736	697	8	0.25	Promoter(<=1kb)	514	CSNK2A3	283106
472286	11p	12293639	12294538	900	8	0.625	Exon(exon29of35)	6739	MICALCL	84953
472286	11q	58402523	58403265	743	9	0.556	Promoter(<=1kb)	144	OR5B3	441608
472286	11q	64116513	64118232	1720	7	0.714	Exon(exon2of2)	8702	MACROD1	28992
472286	11q	82732630	82733184	555	6	0.833	Promoter(<=1kb)	680	FAM181B	220382
472286	11q	85724687	85725825	1139	6	0.5	Promoter(<=1kb)	0	SYTL2	54843
472286	11q	123906790	123907324	535	7	0.714	Promoter(<=1kb)	644	OR8D4	338662
472286	11q	124038366	124038988	623	7	1	Promoter(<=1kb)	13	OR10G7	390265
472286	12p	4626568	4628549	1982	11	0.455	Exon(exon5of6)	14051	DYRK4	8798
472286	12p	10434512	10435982	1471	6	0.5	Promoter(<=1kb)	0	KLRC2	3822
472286	13q	24434450	24435347	898	7	0.571	Exon(exon31of34)	19787	PARP4	143
472286	13q	25096713	25097231	519	12	0.5	Promoter(<=1kb)	845	PABPC3	5042
472286	13q	102732474	102733933	1460	6	0.333	Exon(exon4of4)	25139	CCDC168	643677
472286	14q	19975713	19976448	736	9	0.444	Promoter(<=1kb)	269	OR4K15	81127
472286	14q	20060048	20060884	837	8	0.625	Promoter(<=1kb)	3	OR4L1	122742
472286	14q	20640982	20641567	586	6	0.5	Promoter(<=1kb)	124	OR6S1	341799
472286	14q	22633879	22634450	572	9	0.333	Exon(exon2of2)	32212	ABHD4	63874
472286	14q	23080076	23080576	501	6	0.667	Promoter(<=1kb)	-483	ACIN1	22985
472286	14q	70457532	70458948	1417	12	0.417	Exon(exon2of2)	5358	ADAM21	8747
472286	14q	104175275	104177810	2536	10	0.3	Exon(exon12of15)	36235	KIF26A	26153
472286	14q	104947943	104950338	2396	8	0.625	Exon(exon6of6)	5064	AHNAK2	113146
472286	15q	23440160	23442067	1908	11	0.636	5'UTR	5167	GOLGA6L2	283685
472286	15q	40621642	40624434	2793	8	0.375	Promoter(<=1kb)	0	KNL1	57082
472286	15q	85579423	85581800	2378	14	0.571	Promoter(1-2kb)	-1110	AKAP13	11214
472286	15q	99130060	99132394	2335	6	0.5	Exon(exon4of5)	7348	TTC23	64927
472286	15q	101177475	101178745	1271	6	0.667	3'UTR	9125	CHSY1	22856
472286	16p	1229573	1229731	159	8	0.625	Promoter(<=1kb)	431	TPSB2	64499
472286	16p	1256345	1256985	641	12	0.667	Promoter(<=1kb)	276	TPSD1	23430
472286	16p	1486371	1488463	2093	8	0.75	Promoter(<=1kb)	4	PTX4	390667
472286	16q	88428416	88429600	1185	7	0.429	Exon(exon3of3)	-23680	ZFPM1	161882
472286	16q	89100686	89101050	365	7	0.571	Promoter(<=1kb)	24	ACSF3	197322
472286	16q	89226863	89228289	1427	8	0.625	Promoter(2-3kb)	2229	ZNF778	197320
472286	17p	21300581	21300978	398	12	0.75	3'UTR	9112	MAP2K3	5606
472286	17p	21415461	21416416	956	9	0.889	Exon(exon3of3)	10325	KCNJ12	3768
472286	17q	76291123	76294016	2894	11	0.455	Promoter(<=1kb)	0	QRICH2	84074
472286	17q	81645135	81645417	283	6	0.333	Promoter(2-3kb)	2722	TSPAN10	83882
472286	18q	58535186	58537515	2330	9	0.333	Promoter(<=1kb)	0	ALPK2	115701
472286	19p	4510548	4513547	3000	20	0.5	Exon(exon3of6)	4157	PLIN4	729359
472286	19p	5455600	5456439	840	7	0.571	Promoter(<=1kb)	183	ZNRF4	148066
472286	19p	8333830	8335276	1447	8	0.625	Promoter(<=1kb)	0	KANK3	256949
472286	19p	8937644	8939234	1591	6	0.667	Exon(exon5of84)	-41554	MUC16	94025

472286	19p	8946313	8951868	5556	20	0.65	Exon(exon3of84)	29474	MUC16	94025
472286	19p	8959116	8962299	3184	11	0.727	Exon(exon3of84)	19043	MUC16	94025
472286	19p	8971838	8978096	6259	15	0.533	Exon(exon1of84)	3246	MUC16	94025
472286	19p	9126210	9126946	737	9	0.556	Promoter(<=1kb)	4	OR7G3	390883
472286	19p	15087213	15088040	828	9	0.333	Promoter(<=1kb)	233	OR1I1	126370
472286	19p	21971930	21974500	2571	7	0.714	Exon(exon4of4)	14408	ZNF208	7757
472286	19q	33205448	33207385	1938	6	0.667	Promoter(1-2kb)	1578	LRP3	4037
472286	19q	39886005	39886439	435	7	0.571	Promoter(<=1kb)	853	FCGBP	8857
472286	19q	43913423	43914878	1456	8	0.5	Exon(exon10of10)	4861	ZNF45	7596
472286	19q	44106512	44108078	1567	8	0.125	Exon(exon6of6)	-4103	ZNF225	7768
472286	19q	52384029	52384992	964	7	0.143	Exon(exon4of4)	12677	ZNF528-AS1	102724105
472286	19q	55517821	55518176	356	7	1	Exon(exon14of14)	18127	SBK2	646643
472286	19q	55911888	55913077	1190	6	0.667	Exon(exon5of12)	19234	NLRP13	126204
472286	20p	5922421	5923394	974	8	0.5	Exon(exon4of5)	6923	CHGB	1114
472286	20q	63561666	63565531	3866	13	0.615	Promoter(<=1kb)	-61	HELZ2	85441
472286	22q	22352950	22353348	399	14	0.571	Exon(exon1of2)	30478	BMS1P20	96610
472286	22q	36191154	36191906	753	6	0.667	3'UTR	9971	APOL4	80832
472286	23p	8170039	8170141	103	6	0.5	Promoter(1-2kb)	1126	VCX2	51480
472286	23p	35802148	35803010	863	7	0.571	5'UTR	3357	MAGEB16	139604
473358	1p	11766028	11768307	2280	6	0.833	Promoter(<=1kb)	0	C1orf167	284498
473358	1p	11778784	11779941	1158	7	0.714	Promoter(<=1kb)	0	C1orf167-AS1	102724659
473358	1p	12859108	12860212	1105	10	0.5	Promoter(2-3kb)	2022	PRAMEF2	65122
473358	1p	18481403	18482217	815	7	0.714	Promoter(<=1kb)	421	KLHDC7A	127707
473358	1p	23874604	23875430	827	8	0.5	Exon(exon2of2)	-6310	FUCA1	2517
473358	1p	40067594	40067675	82	6	0	Promoter(<=1kb)	324	CAP1	10487
473358	1q	152303673	152305138	1466	11	0.545	Exon(exon3of3)	-7438	FLG-AS1	339400
473358	1q	152306380	152313891	7512	31	0.581	Promoter(<=1kb)	0	FLG-AS1	339400
473358	1q	169542317	169542882	566	6	0.167	Exon(exon13of25)	-26572	F5	2153
473358	1q	183647749	183648558	810	7	0.429	Exon(exon2of2)	4758	APOBEC4	403314
473358	1q	197101312	197101771	460	6	0.5	Exon(exon18of28)	33373	ASPM	259266
473358	1q	247841312	247841582	271	6	0.833	Promoter(<=1kb)	314	OR11L1	391189
473358	1q	247895625	247896410	786	7	0.571	Promoter(<=1kb)	38	OR2W3	343171
473358	1q	248294677	248295458	782	6	0.667	Promoter(<=1kb)	142	OR2T12	127064
473358	1q	248573992	248574210	219	6	0.833	Promoter(<=1kb)	547	OR2T34	127068
473358	1q	248681658	248682198	541	7	0.571	Promoter(<=1kb)	130	OR14I1	401994
473358	2p	29002636	29003646	1011	6	0.333	Exon(exon5of20)	-10821	TOGARAM2	165186
473358	2p	48580657	48582454	1798	7	0.571	Promoter(<=1kb)	0	STON1	11037
473358	2q	130193975	130195145	1171	7	0.714	Exon(exon3of5)	3294	TUBA3E	112714
473358	2q	130914200	130916712	2513	6	0.333	Promoter(<=1kb)	0	ARHGEF4	50649
473358	2q	132783061	132784972	1912	7	0.429	Promoter(1-2kb)	-1038	NCKAP5	344148
473358	2q	178739433	178741811	2379	6	0.5	Exon(exon45of191)	26014	TTN	7273
473358	2q	185789865	185794632	4768	10	0.8	Promoter(<=1kb)	0	FSIP2	401024
473358	2q	185805377	185808170	2794	6	0.333	Promoter(<=1kb)	0	FSIP2	401024
473358	2q	217847583	217848559	977	6	0.833	Exon(exon19of33)	-5423	TNS1	7145
473358	2q	237762685	237764060	1376	10	0.5	Exon(exon8of8)	-4137	LRRFIP1	9208
473358	2q	238130271	238131546	1276	7	0.286	Promoter(1-2kb)	1323	ESPNL	339768
473358	3p	75736880	75739243	2364	38	0.553	Promoter(<=1kb)	0	MIR4273	100422955
473358	3q	98169021	98169594	574	6	0.667	Exon(exon2of2)	19695	OR5H14	403273
473358	3q	98264413	98265137	725	8	0.625	Promoter(<=1kb)	128	OR5H6	79295
473358	3q	194341177	194342571	1395	6	0.667	Exon(exon2of2)	8747	CPN2	1370
473358	4p	5988383	5989749	1367	7	0.571	Promoter(<=1kb)	0	C4orf50	389197
473358	4p	6300792	6302360	1569	7	0.857	Exon(exon8of8)	6021	WFS1	7466
473358	4p	8227004	8228508	1505	8	0.125	Promoter(<=1kb)	-24	SH3TC1	54436
473358	4p	10443803	10446224	2422	6	0.333	Exon(exon3of3)	10952	ZNF518B	85460
473358	4q	121036404	121037542	1139	6	0.333	Promoter(1-2kb)	1442	NDNF	79625
473358	4q	185458217	185460011	1795	8	0.625	Promoter(<=1kb)	0	CCDC110	256309
473358	5q	54518000	54519665	1666	6	0.167	Promoter(<=1kb)	135	SNX18	112574
473358	5q	79728956	79730716	1761	8	0.25	Exon(exon2of13)	-7426	CMYA5	202333
473358	5q	79731782	79734523	2742	13	0.308	Exon(exon2of13)	-3619	CMYA5	202333
473358	5q	83537326	83539905	2580	6	0.333	Promoter(1-2kb)	1712	VCAN	1462
473358	5q	140848579	140850786	2208	6	0.5	Promoter(<=1kb)	807	PCDHA9	9752
473358	5q	141174000	141175025	1026	7	0.857	Promoter(1-2kb)	1356	PCDHB7	56129
473358	5q	141178745	141180333	1589	7	0.714	Promoter(<=1kb)	955	PCDHB8	56128
473358	5q	141187690	141188594	905	6	0.167	Promoter(<=1kb)	529	PCDHB9	56127
473358	5q	141955676	141957660	1985	6	0.667	Promoter(<=1kb)	-668	RNF14	9604
473358	5q	148826877	148828070	1194	6	1	Promoter(1-2kb)	1632	ADRB2	154
473358	5q	151565922	151568158	2237	9	0.778	Promoter(<=1kb)	786	FAT2	2196
473358	6p	26370344	26370479	136	6	0.5	Promoter(<=1kb)	0	BTN3A2	11118
473358	6p	46858771	46859389	619	6	0.5	Exon(exon17of21)	3915	ADGRF5	221395
473358	6q	149888581	149890867	2287	7	0.714	Promoter(<=1kb)	0	RAET1E-AS1	100652739
473358	6q	159233455	159234370	916	10	0.5	Exon(exon11of23)	15158	FNDC1	84624
473358	7p	12369637	12370736	1100	6	0.667	3'UTR	-13307	VWDE	221806
473358	7p	56021087	56021209	123	6	0.5	Exon(exon2of7)	12947	PSPH	5723
473358	7q	100958721	100960873	2153	54	0.463	Promoter(<=1kb)	756	MUC3A	4584
473358	7q	100991195	100992398	1204	6	0.5	Exon(exon5of15)	-20656	MUC12	10071
473358	7q	101034593	101038481	3889	27	0.519	Exon(exon3of12)	-5230	MUC17	140453
473358	8p	10607245	10608432	1188	8	0.5	Exon(exon4of4)	46711	RP1L1	94137

473358	8p	10609614	10610866	1253	10	0.5	Exon(exon4of4)	44277	RP1L1	94137
473358	8p	12132477	12133936	1460	11	0.727	Promoter(<=1kb)	502	USP17L7	392197
473358	8p	13021128	13022030	903	7	0.143	Exon(exon5of5)	9115	TRMT9B	57604
473358	8q	123651655	123652634	980	7	0.571	Promoter(<=1kb)	316	KLHL38	340359
473358	8q	141218050	141219792	1743	6	0.667	5'UTR	8778	SLC45A4	57210
473358	8q	141466429	141467514	1086	9	0.778	3'UTR	29245	MROH5	389690
473358	9p	34723747	34725050	1304	6	0.833	Exon(exon4of4)	4438	FAM205A	259308
473358	9q	76705724	76707804	2081	7	0.571	Promoter(<=1kb)	666	PCA3	50652
473358	9q	104598545	104599361	817	7	0.429	Promoter(<=1kb)	52	OR13C5	138799
473358	9q	122553278	122554071	794	7	0.429	Promoter(<=1kb)	108	OR1N2	138882
473358	9q	135484803	135487213	2411	8	0.25	Promoter(1-2kb)	1440	PPP1R26	9858
473358	10q	46549378	46550723	1346	26	0.654	Exon(exon3of3)	4807	GPRIN2	9721
473358	10q	89737450	89738561	1112	7	0.143	Exon(exon20of33)	13874	KIF20B	9585
473358	10q	128102594	128106296	3703	8	0.625	Promoter(<=1kb)	0	MKI67	4288
473358	11p	244106	244197	92	8	0.5	Promoter(<=1kb)	-232	PSMD13	5719
473358	11p	1194354	1196902	2549	7	0.571	Exon(exon34of49)	-26164	MUC5B	727897
473358	11p	5046754	5047432	679	7	0.571	Promoter(<=1kb)	228	OR52J3	119679
473358	11p	5177978	5178478	501	6	0.167	Promoter(<=1kb)	186	OR52Z1	283110
473358	11p	5323542	5324256	715	6	0.5	Promoter(<=1kb)	41	OR51B2	79345
473358	11p	5389704	5390350	647	7	0.429	Promoter(<=1kb)	327	OR51M1	390059
473358	11p	5422212	5423123	912	11	0.636	Promoter(<=1kb)	101	OR51Q1	390061
473358	11p	5515185	5515931	747	6	0.333	Promoter(<=1kb)	768	UBQLNL	143630
473358	11p	5581045	5581738	694	8	0.375	Promoter(<=1kb)	168	OR52B6	340980
473358	11p	5841302	5841883	582	9	0.333	Promoter(<=1kb)	14	OR52E6	390078
473358	11p	5884818	5885061	244	7	0.429	Promoter(<=1kb)	547	OR52E4	390081
473358	11p	5986042	5986669	628	7	1	Promoter(<=1kb)	316	OR52L1	338751
473358	11p	11352040	11352736	697	6	0.167	Promoter(<=1kb)	514	CSNK2A3	283106
473358	11p	12293639	12294842	1204	7	0.714	Exon(exon29of35)	6739	MICALCL	84953
473358	11p	43942293	43943348	1056	9	0.778	Promoter(<=1kb)	0	C11orf96	387763
473358	11q	55572176	55572903	728	6	0.667	Promoter(<=1kb)	48	OR4C16	219428
473358	11q	58214757	58215722	966	8	0.25	Promoter(<=1kb)	12	OR1S1	219959
473358	11q	64315818	64315856	39	6	0.833	Promoter(1-2kb)	1485	TRMT112	51504
473358	11q	66560202	66562261	2060	6	0.333	Exon(exon14of21)	3873	CTSF	8722
473358	11q	82732630	82733184	555	6	0.833	Promoter(<=1kb)	680	FAM181B	220382
473358	11q	85724687	85725825	1139	6	0.5	Promoter(<=1kb)	0	SYTL2	54843
473358	11q	123906790	123907324	535	6	0.667	Promoter(<=1kb)	644	OR8D4	338662
473358	11q	124015601	124016477	877	7	0.286	Promoter(<=1kb)	26	OR10G4	390264
473358	11q	124023038	124023849	812	7	0.429	Promoter(<=1kb)	25	OR10G9	219870
473358	11q	124382526	124383285	760	6	0.5	Promoter(<=1kb)	58	OR8B2	26595
473358	12p	4627329	4628549	1221	6	0.5	Exon(exon5of6)	14812	DYRK4	8798
473358	12q	49795819	49796884	1066	6	0.5	Promoter(<=1kb)	118	NCKAP5L	57701
473358	13q	24434450	24435347	898	7	0.571	Exon(exon31of34)	19787	PARP4	143
473358	13q	25096889	25097231	343	7	0.429	Promoter(1-2kb)	1021	PABPC3	5042
473358	13q	102732474	102733933	1460	6	0.333	Exon(exon4of4)	25139	CCDC168	643677
473358	14q	19975713	19976448	736	10	0.4	Promoter(<=1kb)	269	OR4K15	81127
473358	14q	20060048	20060884	837	9	0.667	Promoter(<=1kb)	3	OR4L1	122742
473358	14q	21634161	21634589	429	7	0.571	Promoter(<=1kb)	351	OR10G2	26534
473358	14q	22633879	22634450	572	9	0.333	Exon(exon2of2)	32212	ABHD4	63874
473358	14q	70457532	70458238	707	8	0.375	Exon(exon2of2)	5358	ADAM21	8747
473358	14q	104175275	104177810	2536	11	0.364	Exon(exon12of15)	36235	KIF26A	26153
473358	14q	104939262	104942618	3357	10	0.2	5'UTR	7102	PLD4	122618
473358	14q	104943622	104945444	1823	6	0.667	Exon(exon6of6)	9958	AHNAK2	113146
473358	14q	104947915	104949750	1836	16	0.562	Exon(exon6of6)	5652	AHNAK2	113146
473358	14q	104951557	104953878	2322	13	0.538	Promoter(1-2kb)	1524	AHNAK2	113146
473358	15q	23440196	23442067	1872	9	0.556	5'UTR	5167	GOLGA6L2	283685
473358	15q	40621642	40624434	2793	9	0.444	Promoter(<=1kb)	0	KNL1	57082
473358	15q	59207956	59208032	77	8	0.25	Promoter(<=1kb)	749	MYO1E	4643
473358	15q	85579423	85582016	2594	16	0.562	Promoter(<=1kb)	-894	AKAP13	11214
473358	16p	1228806	1229716	911	8	0.625	Promoter(<=1kb)	446	TPSB2	64499
473358	16p	1256354	1256985	632	11	0.636	Promoter(<=1kb)	285	TPSD1	23430
473358	16p	1486371	1488463	2093	8	0.75	Promoter(<=1kb)	4	PTX4	390667
473358	16p	3254463	3254762	300	6	0.5	Promoter(1-2kb)	1825	MEFV	4210
473358	16p	4207059	4208004	946	7	0.571	Exon(exon2of7)	31739	SRL	6345
473358	16q	89100686	89101050	365	7	0.571	Promoter(<=1kb)	24	ACSF3	197322
473358	16q	89226863	89228289	1427	8	0.625	Promoter(2-3kb)	2229	ZNF778	197320
473358	17p	744946	746966	2021	6	1	3'UTR	5072	GEMIN4	50628
473358	17p	10638198	10641099	2902	7	0.286	Exon(exon19of41)	-8169	MYH3	4621
473358	17p	21300581	21300954	374	10	0.7	3'UTR	9112	MAP2K3	5606
473358	17p	21415458	21416533	1076	18	0.778	Exon(exon3of3)	10322	KCNJ12	3768
473358	17q	41586466	41586829	364	6	0.833	Promoter(<=1kb)	66	KRT14	3861
473358	17q	41727098	41728331	1234	6	0.833	Promoter(1-2kb)	-1201	HAP1	9001
473358	17q	76293419	76294016	598	6	0.5	Promoter(2-3kb)	-2167	QRICH2	84074
473358	17q	81645135	81645417	283	6	0.333	Promoter(2-3kb)	2722	TSPAN10	83882
473358	18p	14542649	14543140	492	6	0.5	Promoter(<=1kb)	6	POTEC	388468
473358	18q	58535186	58537515	2330	9	0.333	Promoter(<=1kb)	0	ALPK2	115701
473358	19p	4510548	4513547	3000	23	0.435	Exon(exon3of6)	4157	PLIN4	729359
473358	19p	5455600	5456439	840	7	0.571	Promoter(<=1kb)	183	ZNRF4	148066

473358	19p	8946313	8951868	5556	15	0.6	Exon(exon3of84)	29474	MUC16	94025
473358	19p	8959116	8962299	3184	10	0.7	Exon(exon3of84)	19043	MUC16	94025
473358	19p	15087213	15088040	828	9	0.333	Promoter(<=1kb)	233	OR1I1	126370
473358	19p	17281820	17284246	2427	9	0.556	Promoter(<=1kb)	0	ANKLE1	126549
473358	19p	18264753	18267409	2657	8	0.5	5'UTR	7002	IQCN	80726
473358	19p	21971930	21974500	2571	7	0.714	Exon(exon4of4)	14408	ZNF208	7757
473358	19q	36996730	36997597	868	10	0.6	Exon(exon10of10)	5677	ZNF568	374900
473358	19q	39877222	39877880	659	6	0.5	Exon(exon20of28)	9412	FCGBP	8857
473358	19q	39886005	39886260	256	6	0.667	Promoter(1-2kb)	1032	FCGBP	8857
473358	19q	43913423	43914878	1456	9	0.556	Exon(exon10of10)	4861	ZNF45	7596
473358	19q	44106512	44108078	1567	9	0.111	Exon(exon6of6)	-4103	ZNF225	7768
473358	19q	51745958	51746963	1006	6	0.333	Exon(exon3of3)	3848	FPR1	2357
473358	19q	58368293	58368875	583	7	0.429	Exon(exon3of3)	-5445	ZNF497	162968
473358	20p	5922421	5923643	1223	9	0.667	Exon(exon4of5)	6923	CHGB	1114
473358	20p	20052354	20052736	383	6	0.167	Promoter(<=1kb)	0	CFAP61	26074
473358	20q	59193344	59194498	1155	6	0.667	Promoter(2-3kb)	2324	ZNF831	128611
473358	20q	63349752	63350772	1021	6	0.5	3'UTR	3794	CHRNA4	1137
473358	20q	63561666	63565531	3866	11	0.636	Promoter(<=1kb)	-61	HELZ2	85441
473358	21q	44550835	44551416	582	6	0.833	Promoter(<=1kb)	89	KRTAP10-2	386679
473358	21q	44558207	44558709	503	6	0.5	Promoter(<=1kb)	86	KRTAP10-3	386682
473358	21q	44637474	44638041	568	9	0.333	Promoter(<=1kb)	118	KRTAP10-10	353333
473358	22q	22352950	22353348	399	14	0.571	Exon(exon1of2)	30478	BMS1P20	96610
473358	22q	22704568	22704813	246	6	1	Exon(exon2of2)	57790	GGTLC2	91227
473358	22q	36191154	36191906	753	6	0.667	3'UTR	9971	APOL4	80832
473358	22q	49884187	49885288	1102	6	0.167	Exon(exon2of2)	22630	ALG12	79087
473358	23p	3320126	3323750	3625	9	0.556	Exon(exon5of7)	22902	MXRA5	25878
473358	23p	35802148	35803010	863	7	0.571	5'UTR	3357	MAGEB16	139604
480268	1p	978953	979884	932	6	0.5	Promoter(1-2kb)	1145	PERM1	84808
480268	1p	16058512	16060000	1489	8	0.75	Exon(exon5of7)	6189	CLCNKB	1188
480268	1p	18481042	18482032	991	11	0.727	Promoter(<=1kb)	60	KLHDC7A	127707
480268	1p	23874604	23875430	827	8	0.5	Exon(exon2of2)	-6310	FUCA1	2517
480268	1p	40067594	40067675	82	6	0	Promoter(<=1kb)	324	CAP1	10487
480268	1p	62273232	62275080	1849	7	0.429	Promoter(<=1kb)	-441	KANK4	163782
480268	1p	89186388	89186419	32	9	0.556	Promoter(<=1kb)	107	GBP4	115361
480268	1q	152213286	152213347	62	8	0.5	Exon(exon3of3)	10846	HRNR	388697
480268	1q	152219233	152221375	2143	15	0.733	Promoter(2-3kb)	2818	HRNR	388697
480268	1q	156669844	156670886	1043	6	1	Exon(exon4of4)	6521	NES	10763
480268	1q	201206099	201209856	3758	13	0.692	Promoter(1-2kb)	1017	IGFN1	91156
480268	1q	232805117	232806800	1684	7	0.429	Promoter(<=1kb)	225	MAP10	54627
480268	1q	247841312	247841582	271	6	0.833	Promoter(<=1kb)	314	OR11L1	391189
480268	1q	247895796	247896502	707	7	0.571	Promoter(<=1kb)	209	OR2W3	343171
480268	1q	247949325	247949738	414	10	0.3	Promoter(<=1kb)	467	OR2L8	391190
480268	1q	248273309	248273670	362	7	0.571	Promoter(<=1kb)	166	OR2T33	391195
480268	1q	248294677	248295458	782	7	0.571	Promoter(<=1kb)	142	OR2T12	127064
480268	1q	248649979	248650825	847	6	0.833	Promoter(<=1kb)	59	OR2T27	403239
480268	2p	48580657	48582454	1798	7	0.571	Promoter(<=1kb)	0	STON1	11037
480268	2q	102351547	102351902	356	7	0.429	Exon(exon11of11)	-4027	IL18R1	8809
480268	2q	178739433	178741811	2379	6	0.5	Exon(exon45of191)	26014	TTN	7273
480268	2q	184936178	184937636	1459	6	0.333	Exon(exon4of4)	69813	ZNF804A	91752
480268	2q	185789865	185794632	4768	10	0.8	Promoter(<=1kb)	0	FSIP2	401024
480268	2q	185805377	185808891	3515	7	0.429	Promoter(<=1kb)	0	FSIP2	401024
480268	2q	237762685	237764060	1376	10	0.5	Exon(exon8of8)	-4137	LRRFIP1	9208
480268	2q	238130416	238131546	1131	6	0.333	Promoter(1-2kb)	1468	ESPNL	339768
480268	3p	31989532	31990905	1374	7	0.429	Exon(exon2of2)	7761	ZNF860	344787
480268	3p	75737230	75739007	1778	9	0.556	Promoter(<=1kb)	0	MIR4273	100422955
480268	3q	98169021	98169594	574	6	0.667	Exon(exon2of2)	19695	OR5H14	403273
480268	3q	98264413	98265098	686	7	0.571	Promoter(<=1kb)	128	OR5H6	79295
480268	4p	5988383	5989749	1367	7	0.571	Promoter(<=1kb)	0	C4orf50	389197
480268	4p	6300792	6302360	1569	8	0.875	Exon(exon8of8)	6021	WFS1	7466
480268	4p	8227004	8228508	1505	8	0.125	Promoter(<=1kb)	-24	SH3TC1	54436
480268	4p	10443803	10446224	2422	6	0.333	Exon(exon3of3)	10952	ZNF518B	85460
480268	4q	56314397	56316592	2196	7	1	Promoter(<=1kb)	-71	CRACD	57482
480268	4q	154489498	154491312	1815	9	0.556	Promoter(<=1kb)	22	DCHS2	54798
480268	4q	185458217	185460011	1795	8	0.625	Promoter(<=1kb)	0	CCDC110	256309
480268	4q	186619481	186621582	2102	6	0.333	Exon(exon10of27)	-9411	FAT1	2195
480268	5p	795818	796218	401	6	0.833	3'UTR	4927	ZDHHC11	79844
480268	5q	79728956	79730716	1761	8	0.25	Exon(exon2of13)	-7426	CMYA5	202333
480268	5q	79731782	79734523	2742	13	0.308	Exon(exon2of13)	-3619	CMYA5	202333
480268	5q	83537326	83539905	2580	6	0.333	Promoter(1-2kb)	1712	VCAN	1462
480268	5q	140848579	140850786	2208	6	0.5	Promoter(<=1kb)	807	PCDHA9	9752
480268	5q	141122595	141123655	1061	6	0.833	Promoter(<=1kb)	777	PCDHB4	56131
480268	5q	141174000	141175025	1026	6	0.833	Promoter(1-2kb)	1356	PCDHB7	56129
480268	5q	141183999	141184688	690	6	1	Promoter(2-3kb)	-2473	PCDHB9	56127
480268	5q	151565922	151568158	2237	9	0.778	Promoter(<=1kb)	786	FAT2	2196
480268	6p	16326588	16327837	1250	6	0.167	Exon(exon8of9)	36643	GMPR	2766
480268	6p	26370344	26370479	136	6	0.5	Promoter(<=1kb)	0	BTN3A2	11118
480268	6p	46858771	46859389	619	6	0.5	Exon(exon17of21)	3915	ADGRF5	221395

480268	6q	149888581	149890867	2287	7	0.714	Promoter(<=1kb)	0	RAET1E-AS1	100652739
480268	6q	159231899	159234370	2472	10	0.5	Exon(exon11of23)	13602	FNDC1	84624
480268	7p	6330446	6330944	499	6	1	Exon(exon2of2)	7749	FAM220A	84792
480268	7p	12369637	12370736	1100	6	0.667	3'UTR	-13307	VWDE	221806
480268	7p	45082725	45084866	2142	8	0.5	Promoter(1-2kb)	-1325	NACAD	23148
480268	7p	56021087	56021209	123	6	0.5	Exon(exon2of7)	12947	PSPH	5723
480268	7q	100958721	100960873	2153	64	0.484	Promoter(<=1kb)	756	MUC3A	4584
480268	7q	100991195	100993127	1933	8	0.625	Exon(exon5of15)	-19927	MUC12	10071
480268	7q	100995575	100995785	211	6	0.833	Exon(exon5of15)	-17269	MUC12	10071
480268	7q	149818015	149819792	1778	6	0.667	Promoter(2-3kb)	-2352	SSPO	23145
480268	7q	149824094	149826495	2402	6	0.667	Promoter(<=1kb)	0	SSPO	23145
480268	8p	10607375	10612307	4933	16	0.625	Exon(exon4of4)	42836	RP1L1	94137
480268	8p	11331234	11332082	849	6	0.667	Promoter(<=1kb)	346	SLC35G5	83650
480268	8p	12132686	12133940	1255	6	0.667	Promoter(<=1kb)	498	USP17L7	392197
480268	8p	13021128	13022030	903	9	0.222	Exon(exon5of5)	9115	TRMT9B	57604
480268	8q	123651655	123652634	980	7	0.571	Promoter(<=1kb)	316	KLHL38	340359
480268	8q	141218050	141219792	1743	6	0.667	5'UTR	8778	SLC45A4	57210
480268	8q	143870008	143872631	2624	6	0.5	Exon(exon2of2)	5833	EPPK1	83481
480268	9p	116800	117800	1001	6	1	Promoter(<=1kb)	404	FOXD4	2298
480268	9p	34723747	34726716	2970	10	0.7	Promoter(2-3kb)	2772	FAM205A	259308
480268	9q	76175241	76175296	56	8	0.875	Exon(exon14of14)	-13343	PCSK5	5125
480268	9q	104598641	104599361	721	9	0.556	Promoter(<=1kb)	52	OR13C5	138799
480268	9q	122628595	122629398	804	6	0.333	Promoter(<=1kb)	175	OR1B1	347169
480268	9q	122749914	122750547	634	6	0.833	Promoter(<=1kb)	174	OR1L6	392390
480268	9q	135484803	135487213	2411	9	0.333	Promoter(1-2kb)	1440	PPP1R26	9858
480268	10q	46549378	46550723	1346	25	0.64	Exon(exon3of3)	4807	GPRIN2	9721
480268	10q	122084988	122087840	2853	8	0.75	Exon(exon4of23)	-25190	TACC2	10579
480268	11p	244106	244197	92	8	0.5	Promoter(<=1kb)	-232	PSMD13	5719
480268	11p	1194354	1196902	2549	7	0.571	Exon(exon34of49)	-26164	MUC5B	727897
480268	11p	1241677	1243593	1917	7	0.429	Exon(exon31of49)	6083	MUC5B-AS1	112577518
480268	11p	1246095	1248605	2511	12	0.5	Promoter(1-2kb)	1071	MUC5B-AS1	112577518
480268	11p	1250091	1251628	1538	9	0.778	Promoter(<=1kb)	-415	MUC5B-AS1	112577518
480268	11p	5177978	5178478	501	6	0.167	Promoter(<=1kb)	186	OR52Z1	283110
480268	11p	5323451	5324256	806	6	0.5	Promoter(<=1kb)	41	OR51B2	79345
480268	11p	5422212	5423123	912	11	0.636	Promoter(<=1kb)	101	OR51Q1	390061
480268	11p	5581045	5581738	694	8	0.375	Promoter(<=1kb)	168	OR52B6	340980
480268	11p	5884818	5885061	244	6	0.333	Promoter(<=1kb)	547	OR52E4	390081
480268	11p	11351961	11352736	776	7	0.143	Promoter(<=1kb)	514	CSNK2A3	283106
480268	11p	12293639	12294842	1204	8	0.75	Exon(exon29of35)	6739	MICALCL	84953
480268	11p	18173280	18173901	622	6	0.333	Promoter(<=1kb)	443	MRGPRX4	117196
480268	11q	58214757	58215722	966	8	0.25	Promoter(<=1kb)	12	OR1S1	219959
480268	11q	69295669	69296300	632	6	0.667	Promoter(1-2kb)	1495	MYEOV	26579
480268	11q	123906790	123907324	535	6	0.667	Promoter(<=1kb)	644	OR8D4	338662
480268	11q	124015601	124016477	877	9	0.333	Promoter(<=1kb)	26	OR10G4	390264
480268	11q	124023038	124023849	812	8	0.5	Promoter(<=1kb)	25	OR10G9	219870
480268	11q	124382526	124383285	760	8	0.625	Promoter(<=1kb)	58	OR8B2	26595
480268	12p	4626571	4628549	1979	8	0.5	Exon(exon5of6)	14054	DYRK4	8798
480268	12p	6453119	6453670	552	6	0.667	Promoter(<=1kb)	633	TAPBPL	55080
480268	13q	24434450	24435347	898	7	0.571	Exon(exon31of34)	19787	PARP4	143
480268	13q	25096659	25097182	524	11	0.455	Promoter(<=1kb)	791	PABPC3	5042
480268	13q	102732474	102733933	1460	6	0.333	Exon(exon4of4)	25139	CCDC168	643677
480268	14q	20060048	20060884	837	8	0.625	Promoter(<=1kb)	3	OR4L1	122742
480268	14q	21634137	21634589	453	9	0.556	Promoter(<=1kb)	351	OR10G2	26534
480268	14q	22633879	22634450	572	9	0.333	Exon(exon2of2)	32212	ABHD4	63874
480268	14q	70457520	70458540	1021	13	0.385	Exon(exon2of2)	5346	ADAM21	8747
480268	14q	94587512	94587839	328	6	0.5	Exon(exon2of2)	-4219	SERPINA3	12
480268	14q	104175275	104177810	2536	6	0.333	Exon(exon12of15)	36235	KIF26A	26153
480268	14q	104947943	104950338	2396	9	0.444	Exon(exon6of6)	5064	AHNAK2	113146
480268	15q	23440160	23442067	1908	10	0.6	5'UTR	5167	GOLGA6L2	283685
480268	15q	73702465	73703760	1296	6	0.833	Promoter(<=1kb)	-149	CD276	80381
480268	15q	78766033	78766581	549	6	0.667	Promoter(<=1kb)	-920	ADAMTS7	11173
480268	15q	85579423	85581800	2378	15	0.6	Promoter(1-2kb)	-1110	AKAP13	11214
480268	15q	88857108	88859365	2258	7	0.143	Exon(exon12of18)	9865	ACAN	176
480268	15q	99129423	99132517	3095	6	0.333	Exon(exon4of5)	7225	TTC23	64927
480268	16p	1486371	1488463	2093	8	0.75	Promoter(<=1kb)	4	PTX4	390667
480268	16q	84178965	84180270	1306	6	0.833	Promoter(1-2kb)	1148	TAF1C	9013
480268	16q	88428539	88429600	1062	6	0.333	Exon(exon3of3)	-23680	ZFPM1	161882
480268	16q	88714632	88717113	2482	7	0.429	Promoter(<=1kb)	0	MIR4722	100616167
480268	16q	89100686	89101050	365	7	0.571	Promoter(<=1kb)	24	ACSF3	197322
480268	16q	89226863	89228419	1557	9	0.556	Promoter(2-3kb)	2229	ZNF778	197320
480268	17p	744946	746966	2021	6	1	3'UTR	5072	GEMIN4	50628
480268	17p	21300581	21300978	398	12	0.75	3'UTR	9112	MAP2K3	5606
480268	17p	21415470	21416370	901	6	1	Exon(exon3of3)	10334	KCNJ12	3768
480268	17q	73236508	73236991	484	6	0.333	Promoter(<=1kb)	0	FAM104A	84923
480268	17q	76293419	76294016	598	6	0.5	Promoter(2-3kb)	-2167	QRICH2	84074
480268	17q	81645135	81645417	283	6	0.333	Promoter(2-3kb)	2722	TSPAN10	83882
480268	18p	11609646	11610350	705	11	0.818	Promoter(<=1kb)	50	SLC35G4	646000

480268	18q	75285722	75288019	2298	6	0.333	Exon(exon2of2)	41146	TSHZ1	10194
480268	19p	4510548	4513547	3000	20	0.45	Exon(exon3of6)	4157	PLIN4	729359
480268	19p	5455600	5456439	840	7	0.571	Promoter(<=1kb)	183	ZNRF4	148066
480268	19p	8948231	8951868	3638	14	0.643	Exon(exon3of84)	29474	MUC16	94025
480268	19p	8959403	8962066	2664	7	0.429	Exon(exon3of84)	19276	MUC16	94025
480268	19p	8964274	8967127	2854	21	0.667	Exon(exon3of84)	14215	MUC16	94025
480268	19p	12430718	12432437	1720	8	0.375	3'UTR	8584	ZNF443	10224
480268	19p	15087452	15087953	502	7	0.143	Promoter(<=1kb)	472	OR1I1	126370
480268	19p	17282085	17284246	2162	8	0.5	Promoter(<=1kb)	0	ANKLE1	126549
480268	19p	18264753	18267409	2657	8	0.5	5'UTR	7002	IQCN	80726
480268	19p	21971930	21974500	2571	7	0.714	Exon(exon4of4)	14408	ZNF208	7757
480268	19p	23743906	23745300	1395	6	0.333	Exon(exon4of4)	13537	ZNF681	148213
480268	19q	39877222	39877880	659	6	0.5	Exon(exon20of28)	9412	FCGBP	8857
480268	19q	43846955	43848536	1582	6	0.833	3'UTR	13450	ZNF283	284349
480268	19q	43913423	43914878	1456	8	0.5	Exon(exon10of10)	4861	ZNF45	7596
480268	19q	44106512	44108078	1567	7	0	Exon(exon6of6)	-4103	ZNF225	7768
480268	19q	51745958	51746963	1006	6	0.333	Exon(exon3of3)	3848	FPR1	2357
480268	19q	52437918	52439242	1325	7	0.429	Exon(exon4of4)	6504	ZNF534	147658
480268	19q	55912302	55913166	865	6	0.667	Exon(exon5of12)	19145	NLRP13	126204
480268	20p	5922421	5923394	974	6	0.5	Exon(exon4of5)	6923	CHGB	1114
480268	20p	20052354	20052736	383	6	0	Promoter(<=1kb)	0	CFAP61	26074
480268	21q	44591587	44592404	818	6	0.333	Promoter(<=1kb)	101	KRTAP10-6	386674
480268	21q	44600627	44601692	1066	9	0.667	Promoter(<=1kb)	30	KRTAP10-7	386675
480268	21q	44637500	44638041	542	7	0.429	Promoter(<=1kb)	144	KRTAP10-10	353333
480268	22q	22352950	22353380	431	16	0.5	Exon(exon1of2)	30478	BMS1P20	96610
480268	22q	36191154	36191906	753	6	0.667	3'UTR	9971	APOL4	80832
480268	23p	3320126	3323750	3625	10	0.6	Exon(exon5of7)	22902	MXRA5	25878
480268	23p	35802148	35803010	863	7	0.571	5'UTR	3357	MAGEB16	139604
480268	23q	101494051	101494598	548	7	0.571	Exon(exon2of2)	6007	ARMCX4	100131755
480268	23q	102937373	102937765	393	7	0.571	Promoter(<=1kb)	101	RAB40AL	282808
480501	1p	11766028	11768307	2280	6	0.833	Promoter(<=1kb)	0	C1orf167	284498
480501	1p	12847526	12847995	470	10	0.4	Promoter(<=1kb)	730	HNRNPCL1	343069
480501	1p	12859036	12860079	1044	7	0.429	Promoter(1-2kb)	1950	PRAMEF2	65122
480501	1p	12893249	12893425	177	6	0.667	Exon(exon4of4)	4845	PRAMEF10	343071
480501	1p	13370686	13371119	434	9	0.444	Promoter(<=1kb)	781	PRAMEF19	645414
480501	1p	16058491	16060000	1510	11	0.818	Exon(exon5of7)	6168	CLCNKB	1188
480501	1p	18481403	18482217	815	6	0.667	Promoter(<=1kb)	421	KLHDC7A	127707
480501	1p	23874604	23875430	827	8	0.5	Exon(exon2of2)	-6310	FUCA1	2517
480501	1p	40067594	40067675	82	6	0	Promoter(<=1kb)	324	CAP1	10487
480501	1q	152302977	152313891	10915	40	0.6	Promoter(<=1kb)	0	FLG-AS1	339400
480501	1q	156669844	156671745	1902	7	1	Exon(exon4of4)	5662	NES	10763
480501	1q	158765805	158766655	851	6	0.5	Promoter(<=1kb)	47	OR6N1	128372
480501	1q	247841312	247841582	271	6	0.833	Promoter(<=1kb)	314	OR11L1	391189
480501	1q	247895625	247896502	878	7	0.571	Promoter(<=1kb)	38	OR2W3	343171
480501	1q	247949436	247949759	324	8	0.375	Promoter(<=1kb)	578	OR2L8	391190
480501	2q	130914528	130916712	2185	6	0.333	Promoter(<=1kb)	0	ARHGEF4	50649
480501	2q	185789865	185794632	4768	10	0.8	Promoter(<=1kb)	0	FSIP2	401024
480501	2q	185805377	185807185	1809	6	0.5	Promoter(<=1kb)	0	FSIP2	401024
480501	2q	217847583	217848642	1060	7	0.857	Exon(exon19of33)	-5423	TNS1	7145
480501	2q	219271337	219271649	313	6	0.667	Exon(exon4of4)	6227	TUBA4A	7277
480501	2q	233681970	233682693	724	6	0.667	Promoter(<=1kb)	0	UGT1A7	54577
480501	2q	233713134	233713783	650	11	0.727	Promoter(<=1kb)	142	UGT1A5	54579
480501	2q	238130271	238131546	1276	8	0.375	Promoter(1-2kb)	1323	ESPNL	339768
480501	2q	240041845	240042154	310	6	0.167	Downstream(2-3kb)	3918	OR6B3	150681
480501	3p	75737230	75739007	1778	19	0.684	Promoter(<=1kb)	0	MIR4273	100422955
480501	3q	98264413	98265098	686	7	0.571	Promoter(<=1kb)	128	OR5H6	79295
480501	4p	3492873	3493392	520	6	0.667	5'UTR	7524	DOK7	285489
480501	4p	6300792	6302360	1569	6	0.833	Exon(exon8of8)	6021	WFS1	7466
480501	4p	7433331	7434759	1429	8	0.875	Promoter(<=1kb)	171	PSAPL1	768239
480501	4q	121036404	121037536	1133	6	0.333	Promoter(1-2kb)	1448	NDNF	79625
480501	4q	185458217	185460011	1795	8	0.625	Promoter(<=1kb)	0	CCDC110	256309
480501	4q	186706638	186708616	1979	6	0.667	Exon(exon2of27)	15217	FAT1	2195
480501	5q	79728956	79730716	1761	6	0.333	Exon(exon2of13)	-7426	CMYA5	202333
480501	5q	79731782	79734523	2742	13	0.308	Exon(exon2of13)	-3619	CMYA5	202333
480501	5q	140807352	140807737	386	6	0.833	Promoter(<=1kb)	271	PCDHA4	56144
480501	5q	140848579	140850786	2208	6	0.5	Promoter(<=1kb)	807	PCDHA9	9752
480501	5q	141174000	141175025	1026	6	0.833	Promoter(1-2kb)	1356	PCDHB7	56129
480501	5q	141955676	141957660	1985	6	0.667	Promoter(<=1kb)	-668	RNF14	9604
480501	5q	151521550	151522069	520	6	0.667	Promoter(<=1kb)	79	MIR6499	102465246
480501	6p	46858771	46859502	732	8	0.5	Exon(exon17of21)	3802	ADGRF5	221395
480501	6q	64591274	64591961	688	10	0.5	Exon(exon26of43)	121374	EYS	346007
480501	6q	149888581	149889987	1407	6	0.667	Promoter(<=1kb)	-116	RAET1E-AS1	100652739
480501	6q	159231899	159234370	2472	12	0.583	Exon(exon11of23)	13602	FNDC1	84624
480501	7p	6330446	6330944	499	6	1	Exon(exon2of2)	7749	FAM220A	84792
480501	7q	100958977	100960873	1897	50	0.46	Promoter(1-2kb)	1012	MUC3A	4584
480501	7q	100991195	100992398	1204	7	0.571	Exon(exon5of15)	-20656	MUC12	10071
480501	7q	100995415	100995785	371	8	0.625	Exon(exon5of15)	-17269	MUC12	10071

480501	7q	101004421	101004836	416	6	0.667	Exon(exon5of15)	-8218	MUC12	10071
480501	7q	101034361	101040583	6223	35	0.457	Exon(exon3of12)	-3128	MUC17	140453
480501	8p	10609614	10612543	2930	10	0.9	Exon(exon4of4)	42600	RP1L1	94137
480501	8p	11331234	11332082	849	7	0.571	Promoter(<=1kb)	346	SLC35G5	83650
480501	8p	12132686	12133940	1255	6	0.667	Promoter(<=1kb)	498	USP17L7	392197
480501	8p	13021128	13022030	903	8	0.125	Exon(exon5of5)	9115	TRMT9B	57604
480501	8q	123651655	123652634	980	7	0.571	Promoter(<=1kb)	316	KLHL38	340359
480501	9p	712060	713307	1248	7	0.714	Exon(exon7of16)	5171	KANK1	23189
480501	9q	76705724	76707804	2081	6	0.5	Promoter(<=1kb)	666	PCA3	50652
480501	9q	104504315	104505071	757	6	0.5	Promoter(<=1kb)	52	OR13F1	138805
480501	9q	104598641	104599361	721	10	0.6	Promoter(<=1kb)	52	OR13C5	138799
480501	9q	122553263	122554071	809	8	0.5	Promoter(<=1kb)	93	OR1N2	138882
480501	9q	122628595	122629398	804	6	0.333	Promoter(<=1kb)	175	OR1B1	347169
480501	9q	122749914	122750547	634	6	0.833	Promoter(<=1kb)	174	OR1L6	392390
480501	9q	135484803	135487340	2538	9	0.333	Promoter(1-2kb)	1440	PPP1R26	9858
480501	9q	135547960	135548795	836	8	0.625	Promoter(1-2kb)	1805	OBP2A	29991
480501	10q	46549378	46550723	1346	25	0.64	Exon(exon3of3)	4807	GPRIN2	9721
480501	10q	49323169	49326817	3649	13	0.615	Exon(exon3of3)	23895	C10orf71	118461
480501	11p	244106	244197	92	8	0.5	Promoter(<=1kb)	-232	PSMD13	5719
480501	11p	1194354	1196902	2549	7	0.571	Exon(exon34of49)	-26164	MUC5B	727897
480501	11p	1241677	1243593	1917	7	0.286	Exon(exon31of49)	6083	MUC5B-AS1	112577518
480501	11p	1244757	1248605	3849	13	0.692	Promoter(1-2kb)	1071	MUC5B-AS1	112577518
480501	11p	1250091	1251628	1538	9	0.778	Promoter(<=1kb)	-415	MUC5B-AS1	112577518
480501	11p	5046754	5047432	679	7	0.571	Promoter(<=1kb)	228	OR52J3	119679
480501	11p	5177978	5178478	501	6	0.167	Promoter(<=1kb)	186	OR52Z1	283110
480501	11p	5389416	5390350	935	7	0.571	Promoter(<=1kb)	39	OR51M1	390059
480501	11p	5402638	5403322	685	9	0.444	Promoter(<=1kb)	41	OR51J1	79470
480501	11p	5422212	5423123	912	11	0.636	Promoter(<=1kb)	101	OR51Q1	390061
480501	11p	5440604	5441472	869	6	0.833	Promoter(<=1kb)	42	OR51I1	390063
480501	11p	5515185	5515931	747	7	0.429	Promoter(<=1kb)	768	UBQLNL	143630
480501	11p	5581045	5581738	694	9	0.333	Promoter(<=1kb)	168	OR52B6	340980
480501	11p	5841302	5841883	582	9	0.333	Promoter(<=1kb)	14	OR52E6	390078
480501	11p	11351961	11352736	776	9	0.222	Promoter(<=1kb)	514	CSNK2A3	283106
480501	11p	12293788	12294842	1055	6	0.667	Exon(exon29of35)	6888	MICALCL	84953
480501	11p	18173280	18173901	622	6	0.333	Promoter(<=1kb)	443	MRGPRX4	117196
480501	11q	58214757	58215722	966	7	0.286	Promoter(<=1kb)	12	OR1S1	219959
480501	11q	58402523	58403265	743	8	0.5	Promoter(<=1kb)	144	OR5B3	441608
480501	11q	85724687	85725825	1139	6	0.5	Promoter(<=1kb)	0	SYTL2	54843
480501	11q	123906790	123907324	535	6	0.667	Promoter(<=1kb)	644	OR8D4	338662
480501	11q	124038930	124038988	59	6	1	Promoter(<=1kb)	13	OR10G7	390265
480501	11q	124382526	124383285	760	8	0.625	Promoter(<=1kb)	58	OR8B2	26595
480501	12p	4626568	4627524	957	7	0.429	Exon(exon5of6)	14051	DYRK4	8798
480501	12p	6453119	6453670	552	7	0.714	Promoter(<=1kb)	633	TAPBPL	55080
480501	12q	52316096	52317765	1670	6	0.667	Exon(exon4of9)	3633	KRT83	3889
480501	13q	24434450	24435347	898	7	0.571	Exon(exon31of34)	19787	PARP4	143
480501	13q	25096675	25097182	508	6	0.5	Promoter(<=1kb)	807	PABPC3	5042
480501	13q	49668389	49668431	43	6	0.167	Exon(exon3of3)	23007	EBPL	84650
480501	13q	102732474	102733933	1460	6	0.333	Exon(exon4of4)	25139	CCDC168	643677
480501	14q	19975713	19976448	736	9	0.444	Promoter(<=1kb)	269	OR4K15	81127
480501	14q	20060048	20060884	837	9	0.556	Promoter(<=1kb)	3	OR4L1	122742
480501	14q	20640982	20641567	586	6	0.5	Promoter(<=1kb)	124	OR6S1	341799
480501	14q	21634137	21634589	453	9	0.556	Promoter(<=1kb)	351	OR10G2	26534
480501	14q	44504986	44506403	1418	6	0.667	Promoter(<=1kb)	880	FSCB	84075
480501	14q	70457520	70458540	1021	6	0.333	Exon(exon2of2)	5346	ADAM21	8747
480501	14q	94587512	94587839	328	6	0.5	Exon(exon2of2)	-4219	SERPINA3	12
480501	14q	104175275	104177810	2536	9	0.333	Exon(exon12of15)	36235	KIF26A	26153
480501	14q	104939262	104942618	3357	10	0.2	5'UTR	7102	PLD4	122618
480501	14q	104943622	104945444	1823	7	0.571	Exon(exon6of6)	9958	AHNAK2	113146
480501	14q	104947901	104951557	3657	21	0.524	Exon(exon6of6)	3845	AHNAK2	113146
480501	15q	20534501	20534954	454	7	0.429	Exon(exon8of9)	6846	GOLGA6L6	727832
480501	15q	23440370	23442067	1698	9	0.667	5'UTR	5167	GOLGA6L2	283685
480501	15q	85579423	85582073	2651	15	0.6	Promoter(<=1kb)	-837	AKAP13	11214
480501	15q	99129423	99132517	3095	6	0.333	Exon(exon4of5)	7225	TTC23	64927
480501	16p	669592	672548	2957	7	0.571	Promoter(<=1kb)	0	RHOT2	89941
480501	16p	1256345	1256980	636	6	0.833	Promoter(<=1kb)	276	TPSD1	23430
480501	16p	1486371	1488463	2093	8	0.75	Promoter(<=1kb)	4	PTX4	390667
480501	16q	88428186	88429600	1415	7	0.429	Exon(exon3of3)	-23680	ZFPM1	161882
480501	16q	89100686	89101050	365	8	0.625	Promoter(<=1kb)	24	ACSF3	197322
480501	16q	89226883	89228419	1537	8	0.625	Promoter(2-3kb)	2249	ZNF778	197320
480501	17p	2299649	2300159	511	6	0.167	Exon(exon2of19)	-3224	SRR	63826
480501	17p	10638198	10641099	2902	7	0.286	Exon(exon19of41)	-8169	MYH3	4621
480501	17p	21300581	21300978	398	12	0.75	3'UTR	9112	MAP2K3	5606
480501	17q	76293419	76294016	598	6	0.5	Promoter(2-3kb)	-2167	QRICH2	84074
480501	17q	81645135	81645607	473	7	0.286	Promoter(2-3kb)	2722	TSPAN10	83882
480501	18p	11609646	11610509	864	14	0.786	Promoter(<=1kb)	50	SLC35G4	646000
480501	18q	58534999	58537536	2538	13	0.385	Promoter(<=1kb)	0	ALPK2	115701
480501	19p	1467668	1469531	1864	6	0.5	Exon(exon15of15)	9366	C19orf25	148223

480501	19p	4510711	4513547	2837	22	0.409	Exon(exon3of6)	4157	PLIN4	729359
480501	19p	5455600	5456439	840	7	0.571	Promoter(<=1kb)	183	ZNRF4	148066
480501	19p	8333830	8334965	1136	6	0.667	Promoter(<=1kb)	0	KANK3	256949
480501	19p	8946306	8951868	5563	17	0.588	Exon(exon3of84)	29474	MUC16	94025
480501	19p	8959403	8962066	2664	7	0.429	Exon(exon3of84)	19276	MUC16	94025
480501	19p	8964274	8967127	2854	21	0.667	Exon(exon3of84)	14215	MUC16	94025
480501	19p	12430157	12432437	2281	10	0.4	3'UTR	8584	ZNF443	10224
480501	19p	15087452	15087953	502	7	0.143	Promoter(<=1kb)	472	OR1I1	126370
480501	19p	15794192	15794719	528	6	0.333	Promoter(<=1kb)	241	OR10H5	284433
480501	19p	18264798	18267409	2612	12	0.583	5'UTR	7002	IQCN	80726
480501	19p	21971930	21974500	2571	7	0.714	Exon(exon4of4)	14408	ZNF208	7757
480501	19q	39877222	39877880	659	6	0.5	Exon(exon20of28)	9412	FCGBP	8857
480501	19q	39886240	39886422	183	6	0.667	Promoter(<=1kb)	870	FCGBP	8857
480501	19q	40880128	40880582	455	6	0.333	Promoter(<=1kb)	-38	CYP2A7	1549
480501	19q	43612804	43614201	1398	6	0.5	Promoter(<=1kb)	716	SRRM5	100170229
480501	19q	43846955	43848536	1582	6	0.833	3'UTR	13450	ZNF283	284349
480501	19q	43913423	43914878	1456	7	0.429	Exon(exon10of10)	4861	ZNF45	7596
480501	19q	43965860	43967171	1312	6	0.167	Promoter(<=1kb)	-691	ZNF155	7711
480501	19q	43996326	43997366	1041	6	0.5	Exon(exon5of5)	5419	LOC10192806:	101928063
480501	19q	44106512	44108078	1567	7	0	Exon(exon6of6)	-4103	ZNF225	7768
480501	19q	47679530	47681217	1688	6	0.667	Promoter(<=1kb)	568	BICRA	29998
480501	19q	52437918	52439242	1325	7	0.429	Exon(exon4of4)	6504	ZNF534	147658
480501	19q	53164551	53166239	1689	6	0.167	Exon(exon4of4)	-5476	ZNF347	84671
480501	19q	55481625	55483456	1832	6	0.5	Promoter(1-2kb)	-1732	NAT14	57106
480501	19q	55517821	55518176	356	6	1	Exon(exon14of14)	18127	SBK2	646643
480501	19q	55911888	55913077	1190	6	0.667	Exon(exon5of12)	19234	NLRP13	126204
480501	20p	5922677	5923382	706	6	0.5	Exon(exon4of5)	7179	CHGB	1114
480501	20q	63561666	63564319	2654	8	0.75	Promoter(<=1kb)	-61	HELZ2	85441
480501	21q	26843740	26844859	1120	6	0.667	Promoter(<=1kb)	0	ADAMTS1	9510
480501	21q	44637474	44638041	568	9	0.333	Promoter(<=1kb)	118	KRTAP10-10	353333
480501	22q	22352950	22353380	431	16	0.5	Exon(exon1of2)	30478	BMS1P20	96610
480501	22q	22646443	22647152	710	8	0.375	Promoter(<=1kb)	0	GGTLC2	91227
480501	22q	36191154	36191906	753	6	0.667	3'UTR	9971	APOL4	80832
480501	23p	8170039	8170141	103	6	0.5	Promoter(1-2kb)	1126	VCX2	51480
480501	23p	35802148	35803010	863	7	0.571	5'UTR	3357	MAGEB16	139604
480501	23q	136874183	136874416	234	8	0.75	Promoter(<=1kb)	-201	RBMX	27316
480850	1p	11766129	11768262	2134	8	0.875	Promoter(<=1kb)	0	C1orf167	284498
480850	1p	12029085	12030097	1013	6	0.833	Promoter(<=1kb)	0	MIR6729	102466982
480850	1p	16058491	16060000	1510	13	0.846	Exon(exon5of7)	6168	CLCNKB	1188
480850	1p	18481403	18482217	815	7	0.714	Promoter(<=1kb)	421	KLHDC7A	127707
480850	1p	23874604	23875430	827	9	0.556	Exon(exon2of2)	-6310	FUCA1	2517
480850	1p	40067594	40067675	82	6	0	Promoter(<=1kb)	324	CAP1	10487
480850	1p	74470408	74472144	1737	6	0.5	Exon(exon5of5)	31343	FPGT-TNNI3K	100526835
480850	1p	89186388	89186419	32	9	0.556	Promoter(<=1kb)	107	GBP4	115361
480850	1q	152213286	152213347	62	8	0.5	Exon(exon3of3)	10846	HRNR	388697
480850	1q	152218604	152221375	2772	17	0.765	Promoter(2-3kb)	2818	HRNR	388697
480850	1q	156669844	156670886	1043	6	1	Exon(exon4of4)	6521	NES	10763
480850	1q	169541237	169542882	1646	9	0.222	Exon(exon13of25)	-25492	F5	2153
480850	1q	197101312	197101771	460	6	0.5	Exon(exon18of28)	33373	ASPM	259266
480850	1q	214640144	214642954	2811	14	0.571	Exon(exon12of20)	-5013	CENPF	1063
480850	1q	214644872	214647181	2310	11	0.364	Promoter(<=1kb)	-786	CENPF	1063
480850	1q	222628664	222629929	1266	6	0.333	Promoter(<=1kb)	190	MIA3	375056
480850	1q	232805117	232806800	1684	6	0.5	Promoter(<=1kb)	225	MAP10	54627
480850	1q	247841312	247841582	271	6	0.833	Promoter(<=1kb)	314	OR11L1	391189
480850	1q	247949325	247949738	414	9	0.333	Promoter(<=1kb)	467	OR2L8	391190
480850	1q	248273309	248273700	392	8	0.5	Promoter(<=1kb)	136	OR2T33	391195
480850	2p	29071763	29073000	1238	6	0.667	Promoter(1-2kb)	1523	PCARE	388939
480850	2q	102351547	102351902	356	7	0.429	Exon(exon11of11)	-4027	IL18R1	8809
480850	2q	130914528	130916712	2185	6	0.333	Promoter(<=1kb)	0	ARHGEF4	50649
480850	2q	132783061	132784972	1912	7	0.429	Promoter(1-2kb)	-1038	NCKAP5	344148
480850	2q	240041845	240042811	967	8	0.375	Downstream(2-3kb)	3261	OR6B3	150681
480850	3p	75736880	75739243	2364	52	0.596	Promoter(<=1kb)	0	MIR4273	100422955
480850	3q	98264413	98265137	725	6	0.667	Promoter(<=1kb)	128	OR5H6	79295
480850	3q	196947388	196948102	715	7	0.571	3'UTR	3828	PIGZ	80235
480850	4p	5988612	5989749	1138	6	0.333	Promoter(<=1kb)	0	C4orf50	389197
480850	4p	6300792	6302360	1569	6	0.833	Exon(exon8of8)	6021	WFS1	7466
480850	4p	8227004	8228508	1505	8	0.125	Promoter(<=1kb)	-24	SH3TC1	54436
480850	4p	10443803	10446224	2422	7	0.429	Exon(exon3of3)	10952	ZNF518B	85460
480850	4q	186619481	186621582	2102	6	0.333	Exon(exon10of27)	-9411	FAT1	2195
480850	4q	186706638	186709751	3114	7	0.714	Exon(exon2of27)	14082	FAT1	2195
480850	5q	79728956	79730716	1761	7	0.286	Exon(exon2of13)	-7426	CMYA5	202333
480850	5q	79731782	79734523	2742	13	0.308	Exon(exon2of13)	-3619	CMYA5	202333
480850	5q	83537326	83539905	2580	6	0.333	Promoter(1-2kb)	1712	VCAN	1462
480850	5q	140807352	140807737	386	6	0.833	Promoter(<=1kb)	271	PCDHA4	56144
480850	5q	141100771	141102758	1988	12	0.25	Promoter(<=1kb)	298	PCDHB3	56132
480850	5q	141122595	141123949	1355	6	0.833	Promoter(<=1kb)	777	PCDHB4	56131
480850	5q	141174000	141175025	1026	7	0.714	Promoter(1-2kb)	1356	PCDHB7	56129

480850	5q	141178631	141180333	1703	6	0.833	Promoter(<=1kb)	841	PCDHB8	56128
480850	5q	141183999	141184688	690	6	0.833	Promoter(2-3kb)	-2473	PCDHB9	56127
480850	5q	141187109	141189425	2317	6	0.5	Promoter(<=1kb)	0	PCDHB9	56127
480850	5q	141955356	141957660	2305	6	0.667	Promoter(<=1kb)	-668	RNF14	9604
480850	5q	151565922	151568158	2237	9	0.778	Promoter(<=1kb)	786	FAT2	2196
480850	6p	46858771	46859502	732	8	0.5	Exon(exon17of21)	3802	ADGRF5	221395
480850	6q	64591274	64591961	688	10	0.5	Exon(exon26of43)	121374	EYS	346007
480850	6q	149888581	149889987	1407	6	0.667	Promoter(<=1kb)	-116	RAET1E-AS1	100652739
480850	6q	159231899	159234370	2472	12	0.583	Exon(exon11of23)	13602	FNDC1	84624
480850	6q	168307821	168309048	1228	6	0.333	5'UTR	10662	DACT2	168002
480850	7p	45082865	45084866	2002	7	0.429	Promoter(1-2kb)	-1465	NACAD	23148
480850	7p	53035678	53036385	708	7	1	Promoter(<=1kb)	45	POM121L12	285877
480850	7q	100958977	100960873	1897	56	0.446	Promoter(1-2kb)	1012	MUC3A	4584
480850	7q	100991195	100992398	1204	8	0.625	Exon(exon5of15)	-20656	MUC12	10071
480850	7q	100995575	100995785	211	7	0.714	Exon(exon5of15)	-17269	MUC12	10071
480850	7q	101004538	101004836	299	6	0.833	Exon(exon5of15)	-8218	MUC12	10071
480850	7q	101034361	101040583	6223	40	0.525	Exon(exon3of12)	-3128	MUC17	140453
480850	8p	10607245	10608261	1017	7	0.571	Exon(exon4of4)	46882	RP1L1	94137
480850	8p	10609650	10612307	2658	9	0.778	Exon(exon4of4)	42836	RP1L1	94137
480850	8p	12132686	12133940	1255	6	0.667	Promoter(<=1kb)	498	USP17L7	392197
480850	8p	13021128	13022030	903	7	0.143	Exon(exon5of5)	9115	TRMT9B	57604
480850	8q	138151949	138153046	1098	6	0.667	Promoter(<=1kb)	0	FAM135B	51059
480850	8q	143916360	143919209	2850	8	0.25	Exon(exon32of32)	20381	PLEC	5339
480850	9p	21206764	21207038	275	6	0.667	Promoter(<=1kb)	105	IFNA10	3446
480850	9q	76175237	76175301	65	11	0.727	Exon(exon14of14)	-13338	PCSK5	5125
480850	9q	76703555	76707804	4250	15	0.533	Promoter(<=1kb)	0	PCA3	50652
480850	9q	76709263	76710843	1581	8	0.5	Promoter(<=1kb)	0	PRUNE2	158471
480850	9q	87886533	87888536	2004	7	0.571	Exon(exon4of4)	3656	SPATA31E1	286234
480850	9q	104598641	104599361	721	8	0.625	Promoter(<=1kb)	52	OR13C5	138799
480850	9q	122553263	122554071	809	8	0.5	Promoter(<=1kb)	93	OR1N2	138882
480850	9q	122628595	122629130	536	6	0.333	Promoter(<=1kb)	443	OR1B1	347169
480850	9q	122749914	122750547	634	6	0.833	Promoter(<=1kb)	174	OR1L6	392390
480850	9q	133255635	133256205	571	7	1	3'UTR	19009	ABO	28
480850	9q	135484803	135487213	2411	9	0.333	Promoter(1-2kb)	1440	PPP1R26	9858
480850	10q	46549378	46550744	1367	26	0.615	5'UTR	4786	GPRIN2	9721
480850	10q	128102594	128105201	2608	6	0.667	Promoter(<=1kb)	0	MKI67	4288
480850	11p	244106	244197	92	8	0.5	Promoter(<=1kb)	-232	PSMD13	5719
480850	11p	1194354	1196902	2549	7	0.571	Exon(exon34of49)	-26164	MUC5B	727897
480850	11p	1246095	1247378	1284	8	0.375	Promoter(2-3kb)	2298	MUC5B-AS1	112577518
480850	11p	5177811	5178478	668	7	0.143	Promoter(<=1kb)	186	OR52Z1	283110
480850	11p	5323362	5324256	895	7	0.429	Promoter(<=1kb)	41	OR51B2	79345
480850	11p	5389704	5390350	647	7	0.429	Promoter(<=1kb)	327	OR51M1	390059
480850	11p	5402638	5403471	834	11	0.455	Promoter(<=1kb)	41	OR51J1	79470
480850	11p	5422212	5423123	912	10	0.7	Promoter(<=1kb)	101	OR51Q1	390061
480850	11p	5515185	5516015	831	6	0.333	Promoter(<=1kb)	684	UBQLNL	143630
480850	11p	5581045	5581738	694	8	0.375	Promoter(<=1kb)	168	OR52B6	340980
480850	11p	5841302	5841883	582	9	0.333	Promoter(<=1kb)	14	OR52E6	390078
480850	11p	11351961	11352736	776	7	0.143	Promoter(<=1kb)	514	CSNK2A3	283106
480850	11p	12293628	12294368	741	8	0.875	Exon(exon29of35)	6728	MICALCL	84953
480850	11p	18173280	18173901	622	6	0.333	Promoter(<=1kb)	443	MRGPRX4	117196
480850	11q	58214757	58215722	966	8	0.25	Promoter(<=1kb)	12	OR1S1	219959
480850	11q	82732630	82733184	555	6	0.833	Promoter(<=1kb)	680	FAM181B	220382
480850	11q	85724687	85725825	1139	7	0.571	Promoter(<=1kb)	0	SYTL2	54843
480850	11q	124015601	124016477	877	8	0.25	Promoter(<=1kb)	26	OR10G4	390264
480850	11q	124023038	124023849	812	8	0.5	Promoter(<=1kb)	25	OR10G9	219870
480850	11q	124185670	124186080	411	6	0.333	Promoter(<=1kb)	454	OR10D3	26497
480850	12p	4626568	4628549	1982	11	0.455	Exon(exon5of6)	14051	DYRK4	8798
480850	12p	6453119	6453670	552	6	0.667	Promoter(<=1kb)	633	TAPBPL	55080
480850	12p	8222174	8223514	1341	6	0.667	Exon(exon5of6)	4073	FAM90A1	55138
480850	12q	49795819	49797137	1319	6	0.5	Promoter(<=1kb)	0	NCKAP5L	57701
480850	12q	52316096	52317765	1670	6	0.667	Exon(exon4of9)	3633	KRT83	3889
480850	13q	24434450	24435347	898	7	0.571	Exon(exon31of34)	19787	PARP4	143
480850	13q	25096659	25097182	524	20	0.5	Promoter(<=1kb)	791	PABPC3	5042
480850	13q	49668389	49668431	43	6	0.167	Exon(exon3of3)	23007	EBPL	84650
480850	13q	102732474	102733933	1460	6	0.333	Exon(exon4of4)	25139	CCDC168	643677
480850	14q	19827604	19827851	248	6	0.833	Exon(exon2of2)	5669	OR4N2	390429
480850	14q	20060048	20060884	837	8	0.625	Promoter(<=1kb)	3	OR4L1	122742
480850	14q	20143999	20144604	606	8	0.5	Promoter(<=1kb)	263	OR4N5	390437
480850	14q	20428905	20430283	1379	6	0.667	3'UTR	5359	KLHL33	123103
480850	14q	21634137	21634589	453	9	0.556	Promoter(<=1kb)	351	OR10G2	26534
480850	14q	44504986	44506403	1418	6	0.667	Promoter(<=1kb)	880	FSCB	84075
480850	14q	94587512	94587839	328	7	0.571	Exon(exon2of2)	-4219	SERPINA3	12
480850	15q	20534480	20534954	475	6	0.333	Exon(exon8of9)	6846	GOLGA6L6	727832
480850	15q	23439979	23442067	2089	12	0.583	5'UTR	5167	GOLGA6L2	283685
480850	15q	40621642	40623696	2055	6	0.333	Promoter(<=1kb)	0	KNL1	57082
480850	15q	85579423	85582073	2651	16	0.625	Promoter(<=1kb)	-837	AKAP13	11214
480850	15q	99129423	99132517	3095	6	0.333	Exon(exon4of5)	7225	TTC23	64927

480850	16p	1241159	1241317	159	6	0.667	Promoter(<=1kb)	0	TPSAB1	7177
480850	16p	1486371	1488463	2093	8	0.75	Promoter(<=1kb)	4	PTX4	390667
480850	16q	74391650	74392004	355	7	0.571	Exon(exon7of7)	13772	NPIPB15	440348
480850	16q	88428339	88429600	1262	6	0.333	Exon(exon3of3)	-23680	ZFPM1	161882
480850	16q	89100686	89101050	365	7	0.571	Promoter(<=1kb)	24	ACSF3	197322
480850	16q	89226863	89228289	1427	8	0.625	Promoter(2-3kb)	2229	ZNF778	197320
480850	17p	2299649	2300159	511	6	0.167	Exon(exon2of19)	-3224	SRR	63826
480850	17p	21300581	21300978	398	11	0.727	3'UTR	9112	MAP2K3	5606
480850	17q	41586466	41586829	364	6	0.833	Promoter(<=1kb)	66	KRT14	3861
480850	17q	53823368	53824891	1524	6	0.833	Promoter(<=1kb)	441	KIF2B	84643
480850	17q	76293419	76294016	598	6	0.5	Promoter(2-3kb)	-2167	QRICH2	84074
480850	17q	81645135	81645595	461	7	0.429	Promoter(2-3kb)	2722	TSPAN10	83882
480850	18p	11609728	11610164	437	11	0.636	Promoter(<=1kb)	132	SLC35G4	646000
480850	18p	11644365	11644734	370	6	0.833	Exon(exon1of1)	10207	MIR7153	102465690
480850	18p	14542649	14543140	492	6	0.5	Promoter(<=1kb)	6	POTEC	388468
480850	18q	58535186	58537515	2330	9	0.333	Promoter(<=1kb)	0	ALPK2	115701
480850	19p	1036457	1036914	458	6	0.5	Exon(exon6of7)	-3187	ABCA7	10347
480850	19p	4510548	4513547	3000	17	0.471	Exon(exon3of6)	4157	PLIN4	729359
480850	19p	5455600	5456439	840	7	0.571	Promoter(<=1kb)	183	ZNRF4	148066
480850	19p	8937644	8939234	1591	6	0.667	Exon(exon5of84)	-41554	MUC16	94025
480850	19p	8946313	8951868	5556	19	0.632	Exon(exon3of84)	29474	MUC16	94025
480850	19p	8959116	8962299	3184	11	0.727	Exon(exon3of84)	19043	MUC16	94025
480850	19p	8971838	8978096	6259	15	0.533	Exon(exon1of84)	3246	MUC16	94025
480850	19p	12430400	12431840	1441	8	0.5	3'UTR	9181	ZNF443	10224
480850	19p	15087213	15088040	828	10	0.3	Promoter(<=1kb)	233	OR1I1	126370
480850	19p	18264753	18267409	2657	14	0.643	5'UTR	7002	IQCN	80726
480850	19p	22756205	22759523	3319	13	0.615	3'UTR	10459	ZNF99	7652
480850	19q	39886240	39886422	183	6	0.667	Promoter(<=1kb)	870	FCGBP	8857
480850	19q	39893459	39894014	556	6	0.833	Exon(exon12of28)	-6167	FCGBP	8857
480850	19q	40880231	40880622	392	6	0.333	Promoter(<=1kb)	-141	CYP2A7	1549
480850	19q	43204061	43205504	1444	6	0.833	Promoter(<=1kb)	0	PSG4	5672
480850	19q	43913423	43914878	1456	9	0.556	Exon(exon10of10)	4861	ZNF45	7596
480850	19q	43996326	43997366	1041	6	0.5	Exon(exon5of5)	5419	LOC10192806:	101928063
480850	19q	44106512	44108078	1567	9	0.111	Exon(exon6of6)	-4103	ZNF225	7768
480850	19q	52437918	52439242	1325	7	0.429	Exon(exon4of4)	6504	ZNF534	147658
480850	19q	55517821	55518176	356	7	1	Exon(exon14of14)	18127	SBK2	646643
480850	19q	55911888	55913166	1279	7	0.714	Exon(exon5of12)	19145	NLRP13	126204
480850	19q	58368293	58368875	583	7	0.429	Exon(exon3of3)	-5445	ZNF497	162968
480850	20q	63564900	63567360	2461	9	0.667	Promoter(<=1kb)	714	HELZ2	85441
480850	21q	26843740	26844859	1120	6	0.667	Promoter(<=1kb)	0	ADAMTS1	9510
480850	21q	44550835	44551416	582	6	0.833	Promoter(<=1kb)	89	KRTAP10-2	386679
480850	21q	44637474	44638041	568	9	0.333	Promoter(<=1kb)	118	KRTAP10-10	353333
480850	21q	46124685	46125999	1315	6	1	Exon(exon22of28)	4125	COL6A2	1292
480850	22q	22352950	22353380	431	16	0.5	Exon(exon1of2)	30478	BMS1P20	96610
480850	22q	22712431	22713141	711	8	0.875	Exon(exon2of3)	65653	GGTLC2	91227
480850	22q	36191154	36191906	753	6	0.667	3'UTR	9971	APOL4	80832
480850	22q	49884187	49884994	808	6	0.333	Exon(exon2of2)	22924	ALG12	79087
480850	23p	8170039	8170141	103	6	0.5	Promoter(1-2kb)	1126	VCX2	51480
480850	23p	35802148	35803010	863	7	0.571	5'UTR	3357	MAGEB16	139604
481025	1p	12847526	12847995	470	11	0.455	Promoter(<=1kb)	730	HNRNPCL1	343069
481025	1p	12859036	12860079	1044	6	0.333	Promoter(1-2kb)	1950	PRAMEF2	65122
481025	1p	13370686	13371119	434	7	0.429	Promoter(<=1kb)	781	PRAMEF19	645414
481025	1p	16058491	16060000	1510	13	0.846	Exon(exon5of7)	6168	CLCNKB	1188
481025	1p	23874604	23875430	827	8	0.5	Exon(exon2of2)	-6310	FUCA1	2517
481025	1p	40067594	40067675	82	6	0	Promoter(<=1kb)	324	CAP1	10487
481025	1p	89186388	89186419	32	9	0.556	Promoter(<=1kb)	107	GBP4	115361
481025	1q	152213274	152213320	47	8	0.5	Exon(exon3of3)	10873	HRNR	388697
481025	1q	152218469	152221375	2907	16	0.688	Promoter(2-3kb)	2818	HRNR	388697
481025	1q	152303673	152313891	10219	37	0.595	Promoter(<=1kb)	0	FLG-AS1	339400
481025	1q	156669844	156671745	1902	7	1	Exon(exon4of4)	5662	NES	10763
481025	1q	158765805	158766655	851	6	0.5	Promoter(<=1kb)	47	OR6N1	128372
481025	1q	228315976	228318026	2051	7	0.429	Exon(exon50of81)	6492	OBSCN	84033
481025	1q	228371172	228372999	1828	8	0.375	Promoter(<=1kb)	0	OBSCN	84033
481025	1q	232805117	232806800	1684	6	0.5	Promoter(<=1kb)	225	MAP10	54627
481025	1q	247841312	247841582	271	6	0.833	Promoter(<=1kb)	314	OR11L1	391189
481025	1q	247949325	247949738	414	9	0.333	Promoter(<=1kb)	467	OR2L8	391190
481025	1q	248681796	248682198	403	6	0.667	Promoter(<=1kb)	130	OR14I1	401994
481025	2q	102351547	102351902	356	7	0.429	Exon(exon11of11)	-4027	IL18R1	8809
481025	2q	132783534	132785001	1468	7	0.429	Promoter(1-2kb)	-1511	NCKAP5	344148
481025	2q	178739433	178741811	2379	6	0.5	Exon(exon45of191)	26014	TTN	7273
481025	2q	217847567	217848213	647	6	0.833	Exon(exon19of33)	-5407	TNS1	7145
481025	2q	219271337	219271649	313	6	0.667	Exon(exon4of4)	6227	TUBA4A	7277
481025	2q	238130271	238131546	1276	8	0.375	Promoter(1-2kb)	1323	ESPNL	339768
481025	2q	240041845	240042811	967	7	0.286	Downstream(2-3kb)	3261	OR6B3	150681
481025	3p	3844896	3845554	659	6	0.667	Exon(exon2of2)	-44021	LOC10013020'	100130207
481025	3p	75736929	75738859	1931	10	0.6	Promoter(<=1kb)	0	MIR4273	100422955
481025	3q	98264413	98265098	686	8	0.625	Promoter(<=1kb)	128	OR5H6	79295

481025	4p	5988383	5989749	1367	7	0.571	Promoter(<=1kb)	0	C4orf50	389197
481025	4p	6300792	6302360	1569	8	0.875	Exon(exon8of8)	6021	WFS1	7466
481025	4p	8227004	8228508	1505	7	0.143	Promoter(<=1kb)	-24	SH3TC1	54436
481025	4q	99651025	99654554	3530	8	0.75	Promoter(<=1kb)	94	C4orf54	285556
481025	4q	121036404	121037542	1139	6	0.333	Promoter(1-2kb)	1442	NDNF	79625
481025	4q	154489498	154491312	1815	9	0.556	Promoter(<=1kb)	22	DCHS2	54798
481025	5q	79731782	79734523	2742	13	0.308	Exon(exon2of13)	-3619	CMYA5	202333
481025	5q	83537326	83539905	2580	6	0.333	Promoter(1-2kb)	1712	VCAN	1462
481025	5q	140802149	140803473	1325	6	0.333	Promoter(1-2kb)	1092	PCDHA3	56145
481025	5q	140807352	140807737	386	6	0.833	Promoter(<=1kb)	271	PCDHA4	56144
481025	5q	140848579	140850786	2208	6	0.5	Promoter(<=1kb)	807	PCDHA9	9752
481025	5q	141955356	141957660	2305	6	0.667	Promoter(<=1kb)	-668	RNF14	9604
481025	5q	151565922	151568158	2237	10	0.8	Promoter(<=1kb)	786	FAT2	2196
481025	6p	46858771	46859502	732	8	0.5	Exon(exon17of21)	3802	ADGRF5	221395
481025	6q	64591274	64591961	688	10	0.5	Exon(exon26of43)	121374	EYS	346007
481025	6q	159233455	159234370	916	10	0.5	Exon(exon11of23)	15158	FNDC1	84624
481025	7q	64991282	64992758	1477	6	0.5	Promoter(<=1kb)	-246	ZNF117	51351
481025	7q	100958977	100960873	1897	53	0.472	Promoter(1-2kb)	1012	MUC3A	4584
481025	7q	100991195	100992398	1204	7	0.571	Exon(exon5of15)	-20656	MUC12	10071
481025	7q	100995575	100995785	211	7	0.714	Exon(exon5of15)	-17269	MUC12	10071
481025	7q	101004258	101004836	579	6	0.833	Exon(exon5of15)	-8218	MUC12	10071
481025	7q	101034361	101040583	6223	40	0.5	Exon(exon3of12)	-3128	MUC17	140453
481025	7q	149818015	149819792	1778	6	0.667	Promoter(2-3kb)	-2352	SSPO	23145
481025	8p	10607375	10608261	887	6	0.5	Exon(exon4of4)	46882	RP1L1	94137
481025	8p	12132686	12133940	1255	6	0.667	Promoter(<=1kb)	498	USP17L7	392197
481025	8p	13021128	13022030	903	8	0.125	Exon(exon5of5)	9115	TRMT9B	57604
481025	8q	143916360	143919209	2850	7	0.143	Exon(exon32of32)	20381	PLEC	5339
481025	9p	116800	117713	914	6	0.667	Promoter(<=1kb)	491	FOXD4	2298
481025	9p	39078723	39078846	124	6	0.667	Exon(exon22of24)	7302	CNTNAP3	79937
481025	9q	76175237	76175300	64	7	0.571	Exon(exon14of14)	-13339	PCSK5	5125
481025	9q	76703555	76707804	4250	13	0.462	Promoter(<=1kb)	0	PCA3	50652
481025	9q	76709263	76710843	1581	8	0.5	Promoter(<=1kb)	0	PRUNE2	158471
481025	9q	104598641	104599361	721	8	0.625	Promoter(<=1kb)	52	OR13C5	138799
481025	9q	122749914	122750547	634	6	0.833	Promoter(<=1kb)	174	OR1L6	392390
481025	9q	133255635	133256205	571	7	1	3'UTR	19009	ABO	28
481025	9q	135547960	135548795	836	6	0.667	Promoter(1-2kb)	1805	OBP2A	29991
481025	10q	46549378	46550723	1346	25	0.64	Exon(exon3of3)	4807	GPRIN2	9721
481025	10q	49323169	49326817	3649	12	0.583	Exon(exon3of3)	23895	C10orf71	118461
481025	10q	128102594	128105201	2608	6	0.667	Promoter(<=1kb)	0	MKI67	4288
481025	11p	244106	244197	92	8	0.5	Promoter(<=1kb)	-232	PSMD13	5719
481025	11p	5177978	5178478	501	6	0.167	Promoter(<=1kb)	186	OR52Z1	283110
481025	11p	5323362	5324256	895	7	0.429	Promoter(<=1kb)	41	OR51B2	79345
481025	11p	5389704	5390350	647	7	0.429	Promoter(<=1kb)	327	OR51M1	390059
481025	11p	5422212	5423123	912	10	0.7	Promoter(<=1kb)	101	OR51Q1	390061
481025	11p	5581045	5581738	694	8	0.375	Promoter(<=1kb)	168	OR52B6	340980
481025	11p	5841302	5841883	582	9	0.333	Promoter(<=1kb)	14	OR52E6	390078
481025	11p	11351961	11352736	776	7	0.143	Promoter(<=1kb)	514	CSNK2A3	283106
481025	11p	12293639	12294368	730	6	0.833	Exon(exon29of35)	6739	MICALCL	84953
481025	11q	58214757	58215722	966	8	0.25	Promoter(<=1kb)	12	OR1S1	219959
481025	11q	64315797	64315856	60	8	0.75	Promoter(1-2kb)	1485	TRMT112	51504
481025	11q	82732630	82733184	555	6	0.833	Promoter(<=1kb)	680	FAM181B	220382
481025	11q	123906790	123907324	535	6	0.667	Promoter(<=1kb)	644	OR8D4	338662
481025	11q	124038366	124038988	623	7	1	Promoter(<=1kb)	13	OR10G7	390265
481025	11q	124382526	124383285	760	8	0.625	Promoter(<=1kb)	58	OR8B2	26595
481025	12p	4627329	4628549	1221	8	0.625	Exon(exon5of6)	14812	DYRK4	8798
481025	12q	49795819	49796884	1066	6	0.5	Promoter(<=1kb)	118	NCKAP5L	57701
481025	13q	24434450	24435347	898	7	0.571	Exon(exon31of34)	19787	PARP4	143
481025	13q	25096877	25097021	145	7	0.286	Promoter(1-2kb)	1009	PABPC3	5042
481025	13q	102732474	102733933	1460	6	0.333	Exon(exon4of4)	25139	CCDC168	643677
481025	14q	19827604	19827851	248	6	0.833	Exon(exon2of2)	5669	OR4N2	390429
481025	14q	20060048	20060884	837	10	0.6	Promoter(<=1kb)	3	OR4L1	122742
481025	14q	21634137	21634589	453	10	0.6	Promoter(<=1kb)	351	OR10G2	26534
481025	14q	70457520	70458948	1429	9	0.444	Exon(exon2of2)	5346	ADAM21	8747
481025	14q	77377020	77378778	1759	7	0.286	Promoter(<=1kb)	0	SAMD15	161394
481025	14q	104939262	104942618	3357	10	0.2	5'UTR	7102	PLD4	122618
481025	14q	104943622	104945444	1823	7	0.571	Exon(exon6of6)	9958	AHNAK2	113146
481025	14q	104947943	104953878	5936	32	0.438	Promoter(1-2kb)	1524	AHNAK2	113146
481025	15q	23440081	23442067	1987	11	0.545	5'UTR	5167	GOLGA6L2	283685
481025	15q	99129423	99132517	3095	6	0.333	Exon(exon4of5)	7225	TTC23	64927
481025	16p	1228744	1229622	879	7	0.714	Promoter(<=1kb)	540	TPSB2	64499
481025	16p	1486322	1488463	2142	12	0.75	Promoter(<=1kb)	4	PTX4	390667
481025	16p	4207130	4208004	875	6	0.5	Exon(exon2of7)	31739	SRL	6345
481025	16p	28495551	28497395	1845	6	0.333	Promoter(<=1kb)	0	CLN3	1201
481025	16q	88428339	88429600	1262	6	0.333	Exon(exon3of3)	-23680	ZFPM1	161882
481025	16q	89226863	89228390	1528	10	0.6	Promoter(2-3kb)	2229	ZNF778	197320
481025	17p	2299649	2300159	511	6	0.167	Exon(exon2of19)	-3224	SRR	63826
481025	17p	7846914	7848460	1547	6	0.667	Promoter(1-2kb)	1999	KDM6B	23135

481025	17p	21300581	21300954	374	8	0.75	3'UTR	9112	MAP2K3	5606
481025	17q	41586466	41586829	364	6	0.833	Promoter(<=1kb)	66	KRT14	3861
481025	17q	81645135	81645417	283	6	0.333	Promoter(2-3kb)	2722	TSPAN10	83882
481025	18p	11609646	11610509	864	14	0.643	Promoter(<=1kb)	50	SLC35G4	646000
481025	18q	58535186	58537515	2330	9	0.333	Promoter(<=1kb)	0	ALPK2	115701
481025	19p	1036457	1036914	458	6	0.5	Exon(exon6of7)	-3187	ABCA7	10347
481025	19p	4510548	4513547	3000	18	0.556	Exon(exon3of6)	4157	PLIN4	729359
481025	19p	5455600	5456439	840	6	0.5	Promoter(<=1kb)	183	ZNRF4	148066
481025	19p	8937644	8939234	1591	6	0.667	Exon(exon5of84)	-41554	MUC16	94025
481025	19p	8946313	8951868	5556	18	0.611	Exon(exon3of84)	29474	MUC16	94025
481025	19p	8959116	8962299	3184	11	0.727	Exon(exon3of84)	19043	MUC16	94025
481025	19p	8971838	8978096	6259	15	0.533	Exon(exon1of84)	3246	MUC16	94025
481025	19p	12075333	12077046	1714	7	0.286	Promoter(<=1kb)	6	ZNF788P	388507
481025	19p	18264753	18267409	2657	8	0.5	5'UTR	7002	IQCN	80726
481025	19p	21971930	21974500	2571	7	0.714	Exon(exon4of4)	14408	ZNF208	7757
481025	19q	34943334	34944685	1352	6	0.333	3'UTR	9723	ZNF30	90075
481025	19q	36996730	36997597	868	10	0.6	Exon(exon10of10)	5677	ZNF568	374900
481025	19q	39886243	39886422	180	6	0.833	Promoter(<=1kb)	870	FCGBP	8857
481025	19q	43913423	43914878	1456	9	0.556	Exon(exon10of10)	4861	ZNF45	7596
481025	19q	44106512	44108078	1567	8	0.125	Exon(exon6of6)	-4103	ZNF225	7768
481025	19q	48873985	48875051	1067	6	0.5	Promoter(1-2kb)	1564	PPP1R15A	23645
481025	19q	51745958	51746963	1006	6	0.333	Exon(exon3of3)	3848	FPR1	2357
481025	19q	52384029	52384992	964	6	0	Exon(exon4of4)	12677	ZNF528-AS1	102724105
481025	19q	52437918	52439242	1325	7	0.429	Exon(exon4of4)	6504	ZNF534	147658
481025	19q	55912302	55913166	865	6	0.667	Exon(exon5of12)	19145	NLRP13	126204
481025	20q	63349752	63350772	1021	6	0.5	3'UTR	3794	CHRNA4	1137
481025	20q	63561666	63565531	3866	12	0.667	Promoter(<=1kb)	-61	HELZ2	85441
481025	21q	44573798	44574184	387	6	0.667	Promoter(<=1kb)	0	TSPEAR	54084
481025	21q	44600627	44601692	1066	9	0.778	Promoter(<=1kb)	30	KRTAP10-7	386675
481025	21q	44637476	44638143	668	9	0.444	Promoter(<=1kb)	120	KRTAP10-10	353333
481025	22q	22352950	22353298	349	13	0.538	Exon(exon1of2)	30478	BMS1P20	96610
481025	22q	36191154	36191906	753	7	0.714	3'UTR	9971	APOL4	80832
481025	22q	49884187	49884994	808	6	0.333	Exon(exon2of2)	22924	ALG12	79087
481025	23p	3320126	3323750	3625	9	0.556	Exon(exon5of7)	22902	MXRA5	25878
481025	23p	8170039	8170141	103	6	0.5	Promoter(1-2kb)	1126	VCX2	51480
481025	23p	35802148	35803010	863	7	0.571	5'UTR	3357	MAGEB16	139604
481301	1p	12893335	12893440	106	6	0.5	Exon(exon4of4)	4830	PRAMEF10	343071
481301	1p	16058491	16060000	1510	10	0.9	Exon(exon5of7)	6168	CLCNKB	1188
481301	1p	18481403	18482217	815	6	0.667	Promoter(<=1kb)	421	KLHDC7A	127707
481301	1p	23874604	23875430	827	9	0.556	Exon(exon2of2)	-6310	FUCA1	2517
481301	1p	40067594	40067675	82	6	0	Promoter(<=1kb)	324	CAP1	10487
481301	1q	152218469	152221375	2907	16	0.688	Promoter(2-3kb)	2818	HRNR	388697
481301	1q	152303673	152313911	10239	38	0.658	Promoter(<=1kb)	0	FLG-AS1	339400
481301	1q	156669844	156670886	1043	6	1	Exon(exon4of4)	6521	NES	10763
481301	1q	201206099	201209837	3739	11	0.545	Promoter(1-2kb)	1017	IGFN1	91156
481301	1q	201211373	201215147	3775	8	0.5	Promoter(<=1kb)	0	IGFN1	91156
481301	1q	223393517	223394466	950	7	0.714	Promoter(<=1kb)	102	CCDC185	164127
481301	1q	228315976	228318038	2063	8	0.625	Exon(exon50of81)	6492	OBSCN	84033
481301	1q	247841312	247841582	271	6	0.833	Promoter(<=1kb)	314	OR11L1	391189
481301	1q	247949443	247949738	296	9	0.333	Promoter(<=1kb)	585	OR2L8	391190
481301	1q	248273309	248273700	392	8	0.625	Promoter(<=1kb)	136	OR2T33	391195
481301	1q	248294677	248295458	782	7	0.571	Promoter(<=1kb)	142	OR2T12	127064
481301	2p	48580657	48582454	1798	7	0.571	Promoter(<=1kb)	0	STON1	11037
481301	2q	132783534	132785001	1468	7	0.429	Promoter(1-2kb)	-1511	NCKAP5	344148
481301	2q	178739433	178741811	2379	6	0.5	Exon(exon45of191)	26014	TTN	7273
481301	2q	184936178	184937636	1459	6	0.333	Exon(exon4of4)	69813	ZNF804A	91752
481301	2q	185789865	185794632	4768	10	0.8	Promoter(<=1kb)	0	FSIP2	401024
481301	2q	237762685	237764060	1376	10	0.5	Exon(exon8of8)	-4137	LRRFIP1	9208
481301	2q	238130271	238131546	1276	7	0.286	Promoter(1-2kb)	1323	ESPNL	339768
481301	3p	31989532	31990905	1374	8	0.375	Exon(exon2of2)	7761	ZNF860	344787
481301	3p	52521426	52524117	2692	6	0.167	Promoter(<=1kb)	0	STAB1	23166
481301	3p	75736880	75739007	2128	51	0.588	Promoter(<=1kb)	0	MIR4273	100422955
481301	3q	98264413	98265137	725	7	0.714	Promoter(<=1kb)	128	OR5H6	79295
481301	4p	1394843	1395373	531	9	0.333	Exon(exon1of1)	10031	UVSSA	57654
481301	4p	5988383	5989749	1367	7	0.571	Promoter(<=1kb)	0	C4orf50	389197
481301	4p	6300792	6302360	1569	8	0.875	Exon(exon8of8)	6021	WFS1	7466
481301	4p	8227004	8228508	1505	8	0.125	Promoter(<=1kb)	-24	SH3TC1	54436
481301	4q	154489498	154491312	1815	10	0.6	Promoter(<=1kb)	22	DCHS2	54798
481301	4q	186619481	186621601	2121	7	0.286	Exon(exon10of27)	-9411	FAT1	2195
481301	5q	83537326	83539905	2580	6	0.333	Promoter(1-2kb)	1712	VCAN	1462
481301	5q	140807352	140807737	386	6	0.833	Promoter(<=1kb)	271	PCDHA4	56144
481301	5q	141174000	141175025	1026	6	0.833	Promoter(1-2kb)	1356	PCDHB7	56129
481301	5q	141955356	141957660	2305	7	0.714	Promoter(<=1kb)	-668	RNF14	9604
481301	5q	151565922	151568158	2237	9	0.778	Promoter(<=1kb)	786	FAT2	2196
481301	6p	1312843	1313745	903	6	0.5	Promoter(<=1kb)	745	FOXQ1	94234
481301	6p	46858771	46859389	619	6	0.5	Exon(exon17of21)	3915	ADGRF5	221395
481301	6q	149888581	149890867	2287	7	0.714	Promoter(<=1kb)	0	RAET1E-AS1	100652739

481301	6q	159233455	159234370	916	10	0.5	Exon(exon11of23)	15158	FNDC1	84624
481301	7p	53035678	53036385	708	7	1	Promoter(<=1kb)	45	POM121L12	285877
481301	7q	89333725	89336565	2841	8	0.625	Exon(exon4of4)	115571	ZNF804B	219578
481301	7q	100958977	100960873	1897	56	0.446	Promoter(1-2kb)	1012	MUC3A	4584
481301	7q	100991195	100992398	1204	8	0.625	Exon(exon5of15)	-20656	MUC12	10071
481301	7q	100995547	100995785	239	7	0.857	Exon(exon5of15)	-17269	MUC12	10071
481301	7q	101003685	101004836	1152	6	0.667	Exon(exon5of15)	-8218	MUC12	10071
481301	8p	10607245	10612307	5063	18	0.722	Exon(exon4of4)	42836	RP1L1	94137
481301	8p	12132686	12133940	1255	6	0.667	Promoter(<=1kb)	498	USP17L7	392197
481301	8p	13021128	13022030	903	8	0.25	Exon(exon5of5)	9115	TRMT9B	57604
481301	9p	116800	117696	897	6	0.833	Promoter(<=1kb)	508	FOXD4	2298
481301	9p	39078723	39078846	124	7	0.714	Exon(exon22of24)	7302	CNTNAP3	79937
481301	9q	76175237	76175296	60	10	0.8	Exon(exon14of14)	-13343	PCSK5	5125
481301	9q	76703555	76706360	2806	9	0.556	Promoter(<=1kb)	0	PCA3	50652
481301	9q	87887543	87888819	1277	6	0.5	Exon(exon4of4)	4666	SPATA31E1	286234
481301	9q	104598545	104599361	817	12	0.583	Promoter(<=1kb)	52	OR13C5	138799
481301	9q	122553263	122554071	809	7	0.429	Promoter(<=1kb)	93	OR1N2	138882
481301	9q	122628595	122629398	804	7	0.429	Promoter(<=1kb)	175	OR1B1	347169
481301	9q	122749914	122750547	634	6	0.833	Promoter(<=1kb)	174	OR1L6	392390
481301	9q	133255635	133256205	571	7	1	3'UTR	19009	ABO	28
481301	10q	46549378	46550723	1346	25	0.64	Exon(exon3of3)	4807	GPRIN2	9721
481301	10q	49323559	49326305	2747	8	0.375	Exon(exon3of3)	24285	C10orf71	118461
481301	10q	122084988	122087840	2853	8	0.75	Exon(exon4of23)	-25190	TACC2	10579
481301	10q	128102594	128105201	2608	6	0.667	Promoter(<=1kb)	0	MKI67	4288
481301	11p	244106	244197	92	8	0.5	Promoter(<=1kb)	-232	PSMD13	5719
481301	11p	1246095	1248605	2511	12	0.5	Promoter(1-2kb)	1071	MUC5B-AS1	112577518
481301	11p	4954783	4955581	799	6	0.667	Promoter(<=1kb)	132	OR51A2	401667
481301	11p	5177978	5178478	501	6	0.167	Promoter(<=1kb)	186	OR52Z1	283110
481301	11p	5422212	5423123	912	11	0.636	Promoter(<=1kb)	101	OR51Q1	390061
481301	11p	5515185	5515931	747	6	0.333	Promoter(<=1kb)	768	UBQLNL	143630
481301	11p	5581045	5581738	694	8	0.375	Promoter(<=1kb)	168	OR52B6	340980
481301	11p	5841302	5841883	582	9	0.333	Promoter(<=1kb)	14	OR52E6	390078
481301	11p	5884818	5885061	244	7	0.429	Promoter(<=1kb)	547	OR52E4	390081
481301	11p	11351961	11352736	776	9	0.222	Promoter(<=1kb)	514	CSNK2A3	283106
481301	11p	12293639	12294368	730	6	0.833	Exon(exon29of35)	6739	MICALCL	84953
481301	11p	18173280	18173901	622	6	0.333	Promoter(<=1kb)	443	MRGPRX4	117196
481301	11q	55827536	55827640	105	6	0.667	Promoter(<=1kb)	317	OR5L2	26338
481301	11q	58214757	58215722	966	6	0.333	Promoter(<=1kb)	12	OR1S1	219959
481301	11q	58402523	58403265	743	8	0.5	Promoter(<=1kb)	144	OR5B3	441608
481301	11q	66560202	66562261	2060	6	0.333	Exon(exon14of21)	3873	CTSF	8722
481301	11q	78209517	78210481	965	7	0.857	Promoter(2-3kb)	2280	USP35	57558
481301	11q	85724687	85725825	1139	6	0.5	Promoter(<=1kb)	0	SYTL2	54843
481301	11q	124015601	124016477	877	8	0.25	Promoter(<=1kb)	26	OR10G4	390264
481301	11q	124023038	124023849	812	8	0.5	Promoter(<=1kb)	25	OR10G9	219870
481301	12p	4627329	4628549	1221	8	0.625	Exon(exon5of6)	14812	DYRK4	8798
481301	13q	25096889	25097182	294	6	0.333	Promoter(1-2kb)	1021	PABPC3	5042
481301	13q	102732474	102733933	1460	6	0.333	Exon(exon4of4)	25139	CCDC168	643677
481301	14q	19975713	19976448	736	9	0.444	Promoter(<=1kb)	269	OR4K15	81127
481301	14q	20060048	20060632	585	7	0.429	Promoter(<=1kb)	3	OR4L1	122742
481301	14q	20640982	20641567	586	6	0.5	Promoter(<=1kb)	124	OR6S1	341799
481301	14q	21634137	21634589	453	9	0.556	Promoter(<=1kb)	351	OR10G2	26534
481301	14q	22633879	22634450	572	8	0.375	Exon(exon2of2)	32212	ABHD4	63874
481301	14q	70457532	70458540	1009	9	0.333	Exon(exon2of2)	5358	ADAM21	8747
481301	14q	94587512	94587839	328	6	0.5	Exon(exon2of2)	-4219	SERPINA3	12
481301	14q	104175275	104177810	2536	11	0.364	Exon(exon12of15)	36235	KIF26A	26153
481301	14q	104949015	104950453	1439	8	0.25	Exon(exon6of6)	4949	AHNAK2	113146
481301	15q	40621642	40623696	2055	6	0.333	Promoter(<=1kb)	0	KNL1	57082
481301	15q	52609086	52609780	695	6	0.333	Promoter(<=1kb)	-490	FAM214A	56204
481301	15q	85579423	85582073	2651	15	0.6	Promoter(<=1kb)	-837	AKAP13	11214
481301	16p	1228744	1229731	988	10	0.7	Promoter(<=1kb)	431	TPSB2	64499
481301	16p	1256345	1256980	636	6	0.833	Promoter(<=1kb)	276	TPSD1	23430
481301	16p	1486371	1488463	2093	8	0.75	Promoter(<=1kb)	4	PTX4	390667
481301	16q	74391416	74392004	589	9	0.667	Exon(exon7of7)	13538	NPIPB15	440348
481301	16q	88428539	88429600	1062	6	0.333	Exon(exon3of3)	-23680	ZFPM1	161882
481301	16q	89100686	89101050	365	7	0.571	Promoter(<=1kb)	24	ACSF3	197322
481301	16q	89226863	89228289	1427	8	0.625	Promoter(2-3kb)	2229	ZNF778	197320
481301	17p	21300581	21300954	374	8	0.75	3'UTR	9112	MAP2K3	5606
481301	17q	76293419	76294016	598	6	0.5	Promoter(2-3kb)	-2167	QRICH2	84074
481301	17q	81645135	81645417	283	6	0.333	Promoter(2-3kb)	2722	TSPAN10	83882
481301	18p	11609904	11610509	606	9	0.667	Promoter(<=1kb)	308	SLC35G4	646000
481301	18q	58535186	58537515	2330	9	0.333	Promoter(<=1kb)	0	ALPK2	115701
481301	19p	1004711	1005532	822	8	0.625	Exon(exon3of9)	4292	GRIN3B	116444
481301	19p	4510548	4513547	3000	17	0.471	Exon(exon3of6)	4157	PLIN4	729359
481301	19p	5455600	5456439	840	7	0.571	Promoter(<=1kb)	183	ZNRF4	148066
481301	19p	8937644	8939234	1591	6	0.833	Exon(exon5of84)	-41554	MUC16	94025
481301	19p	8946306	8951868	5563	18	0.667	Exon(exon3of84)	29474	MUC16	94025
481301	19p	8959116	8962299	3184	13	0.538	Exon(exon3of84)	19043	MUC16	94025

481301	19p	8963397	8967127	3731	21	0.667	Exon(exon3of84)	14215	MUC16	94025
481301	19p	8972751	8978096	5346	13	0.462	Exon(exon1of84)	3246	MUC16	94025
481301	19p	12430718	12432437	1720	8	0.375	3'UTR	8584	ZNF443	10224
481301	19p	17281820	17284246	2427	9	0.556	Promoter(<=1kb)	0	ANKLE1	126549
481301	19p	18264798	18267409	2612	12	0.583	5'UTR	7002	IQCN	80726
481301	19p	21971930	21974500	2571	7	0.714	Exon(exon4of4)	14408	ZNF208	7757
481301	19p	23743906	23745300	1395	6	0.333	Exon(exon4of4)	13537	ZNF681	148213
481301	19q	39877222	39877880	659	6	0.5	Exon(exon20of28)	9412	FCGBP	8857
481301	19q	43846955	43848536	1582	6	0.833	3'UTR	13450	ZNF283	284349
481301	19q	43913423	43914878	1456	8	0.5	Exon(exon10of10)	4861	ZNF45	7596
481301	19q	43966037	43967171	1135	6	0.167	Promoter(<=1kb)	-691	ZNF155	7711
481301	19q	44106512	44108078	1567	8	0.125	Exon(exon6of6)	-4103	ZNF225	7768
481301	19q	51745958	51746963	1006	6	0.333	Exon(exon3of3)	3848	FPR1	2357
481301	19q	52437918	52439242	1325	7	0.429	Exon(exon4of4)	6504	ZNF534	147658
481301	19q	53164551	53166239	1689	6	0.167	Exon(exon4of4)	-5476	ZNF347	84671
481301	19q	55911888	55913077	1190	6	0.667	Exon(exon5of12)	19234	NLRP13	126204
481301	20p	5302073	5302730	658	6	1	Exon(exon2of2)	11639	PROKR2	128674
481301	20p	5922577	5923382	806	7	0.571	Exon(exon4of5)	7079	CHGB	1114
481301	21q	44637474	44638041	568	10	0.3	Promoter(<=1kb)	118	KRTAP10-10	353333
481301	22q	22352950	22353298	349	13	0.538	Exon(exon1of2)	30478	BMS1P20	96610
481301	22q	36191154	36191906	753	7	0.571	3'UTR	9971	APOL4	80832
481301	22q	46533795	46535941	2147	6	0.667	Promoter(<=1kb)	0	CELSR1	9620
481301	22q	49883704	49884994	1291	6	0.167	Exon(exon2of2)	22924	ALG12	79087
481301	23p	3320126	3323750	3625	10	0.6	Exon(exon5of7)	22902	MXRA5	25878
481301	23p	8170039	8170141	103	6	0.5	Promoter(1-2kb)	1126	VCX2	51480
481301	23q	102937373	102937765	393	6	0.5	Promoter(<=1kb)	101	RAB40AL	282808
481301	23q	141905856	141906385	530	8	0.75	Promoter(1-2kb)	1054	MAGEC1	9947
481670	1p	12847526	12847995	470	9	0.444	Promoter(<=1kb)	730	HNRNPCL1	343069
481670	1p	12859036	12860079	1044	6	0.333	Promoter(1-2kb)	1950	PRAMEF2	65122
481670	1p	13370686	13371119	434	7	0.429	Promoter(<=1kb)	781	PRAMEF19	645414
481670	1p	16058491	16060000	1510	10	0.9	Exon(exon5of7)	6168	CLCNKB	1188
481670	1p	18481403	18482217	815	6	0.667	Promoter(<=1kb)	421	KLHDC7A	127707
481670	1p	23874604	23875430	827	8	0.5	Exon(exon2of2)	-6310	FUCA1	2517
481670	1p	40067594	40067675	82	6	0	Promoter(<=1kb)	324	CAP1	10487
481670	1p	89186388	89186419	32	9	0.556	Promoter(<=1kb)	107	GBP4	115361
481670	1q	152213274	152213320	47	8	0.5	Exon(exon3of3)	10873	HRNR	388697
481670	1q	152218469	152221375	2907	18	0.667	Promoter(2-3kb)	2818	HRNR	388697
481670	1q	152303673	152313891	10219	37	0.595	Promoter(<=1kb)	0	FLG-AS1	339400
481670	1q	201206099	201209342	3244	10	0.6	Promoter(1-2kb)	1017	IGFN1	91156
481670	1q	201210956	201212792	1837	11	0.455	Promoter(<=1kb)	0	IGFN1	91156
481670	1q	223393517	223394466	950	6	0.667	Promoter(<=1kb)	102	CCDC185	164127
481670	1q	232805117	232806800	1684	7	0.429	Promoter(<=1kb)	225	MAP10	54627
481670	1q	247841312	247841582	271	6	0.833	Promoter(<=1kb)	314	OR11L1	391189
481670	1q	247949325	247949738	414	9	0.333	Promoter(<=1kb)	467	OR2L8	391190
481670	1q	248681658	248682198	541	7	0.571	Promoter(<=1kb)	130	OR14I1	401994
481670	2p	29002636	29003646	1011	6	0.333	Exon(exon5of20)	-10821	TOGARAM2	165186
481670	2p	48580657	48582454	1798	7	0.571	Promoter(<=1kb)	0	STON1	11037
481670	2q	102351547	102351902	356	7	0.429	Exon(exon11of11)	-4027	IL18R1	8809
481670	2q	130193975	130194376	402	7	0.571	Exon(exon4of5)	4063	TUBA3E	112714
481670	2q	130914528	130916712	2185	6	0.333	Promoter(<=1kb)	0	ARHGEF4	50649
481670	2q	185789865	185794632	4768	10	0.8	Promoter(<=1kb)	0	FSIP2	401024
481670	2q	185805377	185808170	2794	6	0.333	Promoter(<=1kb)	0	FSIP2	401024
481670	2q	237762685	237764060	1376	10	0.5	Exon(exon8of8)	-4137	LRRFIP1	9208
481670	2q	240041845	240042811	967	7	0.286	Downstream(2-3kb)	3261	OR6B3	150681
481670	3p	13570520	13571564	1045	6	0.167	Promoter(1-2kb)	1780	FBLN2	2199
481670	3p	31989532	31990905	1374	7	0.429	Exon(exon2of2)	7761	ZNF860	344787
481670	3p	75736929	75739007	2079	15	0.6	Promoter(<=1kb)	0	MIR4273	100422955
481670	3q	98169021	98169561	541	6	0.5	Exon(exon2of2)	19695	OR5H14	403273
481670	3q	98264409	98265098	690	8	0.625	Promoter(<=1kb)	124	OR5H6	79295
481670	3q	194359607	194360906	1300	9	0.889	Exon(exon2of2)	-8279	CPN2	1370
481670	4p	5988383	5989749	1367	8	0.5	Promoter(<=1kb)	0	C4orf50	389197
481670	4p	6300792	6302360	1569	7	0.857	Exon(exon8of8)	6021	WFS1	7466
481670	4p	7433331	7434512	1182	6	0.833	Promoter(<=1kb)	418	PSAPL1	768239
481670	4p	8227004	8228508	1505	7	0.143	Promoter(<=1kb)	-24	SH3TC1	54436
481670	4q	186706638	186709751	3114	7	0.714	Exon(exon2of27)	14082	FAT1	2195
481670	5q	83537326	83539905	2580	7	0.429	Promoter(1-2kb)	1712	VCAN	1462
481670	5q	140848579	140850786	2208	6	0.5	Promoter(<=1kb)	807	PCDHA9	9752
481670	5q	141174000	141174425	426	8	0.875	Promoter(1-2kb)	1356	PCDHB7	56129
481670	5q	141955676	141957660	1985	6	0.667	Promoter(<=1kb)	-668	RNF14	9604
481670	5q	148826877	148828070	1194	6	1	Promoter(1-2kb)	1632	ADRB2	154
481670	6p	1312843	1313745	903	6	0.5	Promoter(<=1kb)	745	FOXQ1	94234
481670	6p	46858771	46859502	732	8	0.5	Exon(exon17of21)	3802	ADGRF5	221395
481670	6q	149888581	149890867	2287	7	0.714	Promoter(<=1kb)	0	RAET1E-AS1	100652739
481670	6q	159233455	159234370	916	10	0.5	Exon(exon11of23)	15158	FNDC1	84624
481670	7p	5313004	5313713	710	6	0.667	Exon(exon27of30)	6894	TNRC18	84629
481670	7p	12369637	12370736	1100	6	0.667	3'UTR	-13307	VWDE	221806
481670	7q	100958977	100960873	1897	50	0.46	Promoter(1-2kb)	1012	MUC3A	4584

481670	7q	100991195	100993127	1933	8	0.625	Exon(exon5of15)	-19927	MUC12	10071
481670	7q	100995575	100995785	211	6	0.833	Exon(exon5of15)	-17269	MUC12	10071
481670	7q	101004230	101004836	607	6	0.833	Exon(exon5of15)	-8218	MUC12	10071
481670	7q	129126736	129127452	717	6	0.333	Exon(exon1of2)	-17255	TSPAN33	340348
481670	7q	149817987	149819792	1806	7	0.714	Promoter(2-3kb)	-2352	SSPO	23145
481670	7q	156949807	156950570	764	6	0.833	Promoter(<=1kb)	95	NOM1	64434
481670	8p	13021128	13022030	903	8	0.25	Exon(exon5of5)	9115	TRMT9B	57604
481670	9p	116800	117713	914	6	0.667	Promoter(<=1kb)	491	FOXD4	2298
481670	9q	76175237	76175296	60	9	0.778	Exon(exon14of14)	-13343	PCSK5	5125
481670	9q	76703555	76707804	4250	13	0.462	Promoter(<=1kb)	0	PCA3	50652
481670	9q	76709263	76710843	1581	8	0.5	Promoter(<=1kb)	0	PRUNE2	158471
481670	9q	87885490	87888819	3330	8	0.625	Promoter(2-3kb)	2613	SPATA31E1	286234
481670	9q	122749914	122750547	634	6	0.833	Promoter(<=1kb)	174	OR1L6	392390
481670	9q	135484803	135487213	2411	8	0.25	Promoter(1-2kb)	1440	PPP1R26	9858
481670	10p	47663	48605	943	6	0.833	Promoter(<=1kb)	664	TUBB8	347688
481670	10q	46549378	46550723	1346	25	0.64	Exon(exon3of3)	4807	GPRIN2	9721
481670	10q	122084988	122087840	2853	8	0.75	Exon(exon4of23)	-25190	TACC2	10579
481670	10q	128103129	128104752	1624	8	0.5	Promoter(<=1kb)	-1	MKI67	4288
481670	10q	128106210	128108204	1995	6	1	Exon(exon12of14)	-3082	MKI67	4288
481670	11p	244106	244197	92	8	0.5	Promoter(<=1kb)	-232	PSMD13	5719
481670	11p	1194354	1196902	2549	7	0.571	Exon(exon34of49)	-26164	MUC5B	727897
481670	11p	1241677	1243660	1984	9	0.444	Exon(exon31of49)	6016	MUC5B-AS1	112577518
481670	11p	1246440	1248605	2166	9	0.556	Promoter(1-2kb)	1071	MUC5B-AS1	112577518
481670	11p	1250091	1251524	1434	7	0.714	Promoter(<=1kb)	-415	MUC5B-AS1	112577518
481670	11p	5177978	5178478	501	6	0.167	Promoter(<=1kb)	186	OR52Z1	283110
481670	11p	5323362	5324256	895	7	0.429	Promoter(<=1kb)	41	OR51B2	79345
481670	11p	5389704	5390350	647	6	0.5	Promoter(<=1kb)	327	OR51M1	390059
481670	11p	5422212	5423123	912	11	0.636	Promoter(<=1kb)	101	OR51Q1	390061
481670	11p	5440761	5441472	712	6	0.833	Promoter(<=1kb)	42	OR51I1	390063
481670	11p	5581045	5581738	694	8	0.375	Promoter(<=1kb)	168	OR52B6	340980
481670	11p	5841302	5841883	582	9	0.333	Promoter(<=1kb)	14	OR52E6	390078
481670	11p	5884818	5885061	244	7	0.429	Promoter(<=1kb)	547	OR52E4	390081
481670	11p	11351961	11352736	776	7	0.143	Promoter(<=1kb)	514	CSNK2A3	283106
481670	11p	12293639	12294368	730	7	0.857	Exon(exon29of35)	6739	MICALCL	84953
481670	11p	18173280	18173901	622	6	0.333	Promoter(<=1kb)	443	MRGPRX4	117196
481670	11q	54603136	54603820	685	6	0.667	Promoter(<=1kb)	178	OR4C46	119749
481670	11q	58214757	58215722	966	8	0.25	Promoter(<=1kb)	12	OR1S1	219959
481670	11q	85724687	85725825	1139	6	0.5	Promoter(<=1kb)	0	SYTL2	54843
481670	11q	123906595	123907324	730	7	0.429	Promoter(<=1kb)	449	OR8D4	338662
481670	11q	124015600	124016477	878	7	0.143	Promoter(<=1kb)	25	OR10G4	390264
481670	12p	4626568	4628549	1982	11	0.455	Exon(exon5of6)	14051	DYRK4	8798
481670	13q	102732474	102733933	1460	6	0.333	Exon(exon4of4)	25139	CCDC168	643677
481670	14q	20060048	20060884	837	8	0.625	Promoter(<=1kb)	3	OR4L1	122742
481670	14q	21634137	21634589	453	9	0.556	Promoter(<=1kb)	351	OR10G2	26534
481670	14q	22633879	22634450	572	9	0.333	Exon(exon2of2)	32212	ABHD4	63874
481670	14q	44504986	44506403	1418	6	0.667	Promoter(<=1kb)	880	FSCB	84075
481670	14q	70457733	70458948	1216	7	0.571	Exon(exon2of2)	5559	ADAM21	8747
481670	14q	94587512	94587839	328	6	0.5	Exon(exon2of2)	-4219	SERPINA3	12
481670	14q	104175275	104177810	2536	6	0.333	Exon(exon12of15)	36235	KIF26A	26153
481670	14q	104939262	104945444	6183	18	0.389	5'UTR	7102	PLD4	122618
481670	14q	104947901	104949893	1993	15	0.6	Exon(exon6of6)	5509	AHNAK2	113146
481670	14q	104951557	104953878	2322	11	0.455	Promoter(1-2kb)	1524	AHNAK2	113146
481670	15q	20534480	20535129	650	8	0.375	Exon(exon8of9)	6671	GOLGA6L6	727832
481670	15q	23439979	23442067	2089	10	0.6	5'UTR	5167	GOLGA6L2	283685
481670	15q	52609086	52609780	695	6	0.333	Promoter(<=1kb)	-490	FAM214A	56204
481670	15q	78766049	78766671	623	7	0.714	Promoter(<=1kb)	-936	ADAMTS7	11173
481670	15q	85579423	85582073	2651	15	0.6	Promoter(<=1kb)	-837	AKAP13	11214
481670	15q	100569472	100570097	626	6	0.833	Promoter(<=1kb)	534	LINS1	55180
481670	16p	789084	790597	1514	7	0.286	Promoter(<=1kb)	0	CHTF18	63922
481670	16p	1228744	1229622	879	6	0.833	Promoter(<=1kb)	540	TPSB2	64499
481670	16p	8644541	8644765	225	6	0.5	Promoter(<=1kb)	104	METTL22	79091
481670	16q	74391401	74392004	604	10	0.7	Exon(exon7of7)	13523	NPIPB15	440348
481670	16q	88428416	88429600	1185	6	0.333	Exon(exon3of3)	-23680	ZFPM1	161882
481670	16q	88714632	88717113	2482	7	0.429	Promoter(<=1kb)	0	MIR4722	100616167
481670	16q	89100686	89101050	365	8	0.625	Promoter(<=1kb)	24	ACSF3	197322
481670	16q	89198519	89199058	540	6	0.667	Promoter(<=1kb)	0	SLC22A31	146429
481670	16q	89226863	89228419	1557	10	0.6	Promoter(2-3kb)	2229	ZNF778	197320
481670	17p	744946	747069	2124	8	0.875	3'UTR	4969	GEMIN4	50628
481670	17p	21300581	21300954	374	8	0.75	3'UTR	9112	MAP2K3	5606
481670	17p	21415470	21416404	935	6	1	Exon(exon3of3)	10334	KCNJ12	3768
481670	17q	41586466	41586829	364	6	0.833	Promoter(<=1kb)	66	KRT14	3861
481670	17q	76291123	76294016	2894	11	0.455	Promoter(<=1kb)	0	QRICH2	84074
481670	17q	81645135	81645417	283	6	0.333	Promoter(2-3kb)	2722	TSPAN10	83882
481670	18p	11609646	11610350	705	14	0.857	Promoter(<=1kb)	50	SLC35G4	646000
481670	18q	58535186	58537515	2330	10	0.4	Promoter(<=1kb)	0	ALPK2	115701
481670	19p	1004711	1005532	822	7	0.571	Exon(exon3of9)	4292	GRIN3B	116444
481670	19p	4510548	4513547	3000	19	0.474	Exon(exon3of6)	4157	PLIN4	729359

481670	19p	5455600	5456439	840	6	0.5	Promoter(<=1kb)	183	ZNRF4	148066
481670	19p	8333830	8335281	1452	7	0.571	Promoter(<=1kb)	0	KANK3	256949
481670	19p	8937644	8939234	1591	6	0.833	Exon(exon5of84)	-41554	MUC16	94025
481670	19p	8946306	8951868	5563	18	0.667	Exon(exon3of84)	29474	MUC16	94025
481670	19p	8959116	8962299	3184	12	0.583	Exon(exon3of84)	19043	MUC16	94025
481670	19p	8963397	8967127	3731	21	0.667	Exon(exon3of84)	14215	MUC16	94025
481670	19p	8972751	8978096	5346	14	0.5	Exon(exon1of84)	3246	MUC16	94025
481670	19p	17281820	17284246	2427	9	0.556	Promoter(<=1kb)	0	ANKLE1	126549
481670	19p	18264753	18267409	2657	8	0.5	5'UTR	7002	IQCN	80726
481670	19p	21971930	21974500	2571	7	0.714	Exon(exon4of4)	14408	ZNF208	7757
481670	19p	22756294	22759533	3240	14	0.5	3'UTR	10449	ZNF99	7652
481670	19q	39877222	39877880	659	6	0.5	Exon(exon20of28)	9412	FCGBP	8857
481670	19q	40880128	40880622	495	7	0.286	Promoter(<=1kb)	-38	CYP2A7	1549
481670	19q	43913423	43914878	1456	7	0.429	Exon(exon10of10)	4861	ZNF45	7596
481670	19q	43966037	43967171	1135	6	0.167	Promoter(<=1kb)	-691	ZNF155	7711
481670	19q	43996326	43997366	1041	6	0.5	Exon(exon5of5)	5419	LOC10192806:	101928063
481670	19q	44106512	44108078	1567	8	0.125	Exon(exon6of6)	-4103	ZNF225	7768
481670	19q	48873829	48875925	2097	9	0.444	Promoter(1-2kb)	1408	PPP1R15A	23645
481670	19q	52437918	52439242	1325	7	0.429	Exon(exon4of4)	6504	ZNF534	147658
481670	19q	55771923	55773169	1247	6	0.5	Promoter(2-3kb)	2782	RFPL4AL1	729974
481670	19q	55912302	55913166	865	6	0.667	Exon(exon5of12)	19145	NLRP13	126204
481670	19q	58368293	58368875	583	7	0.429	Exon(exon3of3)	-5445	ZNF497	162968
481670	20p	5922421	5923394	974	7	0.571	Exon(exon4of5)	6923	CHGB	1114
481670	20q	63349696	63350772	1077	7	0.429	3'UTR	3794	CHRNA4	1137
481670	20q	63561666	63564319	2654	9	0.667	Promoter(<=1kb)	-61	HELZ2	85441
481670	21q	26843740	26844859	1120	6	0.667	Promoter(<=1kb)	0	ADAMTS1	9510
481670	22q	22352950	22353380	431	16	0.5	Exon(exon1of2)	30478	BMS1P20	96610
481670	22q	22758758	22759209	452	6	0.833	Exon(exon1of2)	-63567	MIR650	723778
481670	22q	36191154	36191906	753	6	0.667	3'UTR	9971	APOL4	80832
481670	23p	8170039	8170141	103	6	0.5	Promoter(1-2kb)	1126	VCX2	51480
481670	23p	35802148	35803010	863	7	0.571	5'UTR	3357	MAGEB16	139604
481670	23q	136874183	136874416	234	8	0.75	Promoter(<=1kb)	-201	RBMX	27316
481813	1p	978953	979847	895	6	0.333	Promoter(1-2kb)	1182	PERM1	84808
481813	1p	12847526	12848032	507	11	0.455	Promoter(<=1kb)	693	HNRNPCL1	343069
481813	1p	12859036	12860079	1044	6	0.333	Promoter(1-2kb)	1950	PRAMEF2	65122
481813	1p	13370686	13371119	434	7	0.429	Promoter(<=1kb)	781	PRAMEF19	645414
481813	1p	13391965	13392142	178	6	0.333	Promoter(2-3kb)	2333	PRAMEF17	391004
481813	1p	18481403	18482217	815	6	0.667	Promoter(<=1kb)	421	KLHDC7A	127707
481813	1p	40067594	40067675	82	6	0	Promoter(<=1kb)	324	CAP1	10487
481813	1p	74469879	74472144	2266	6	0.333	Exon(exon5of5)	30814	FPGT-TNNI3K	100526835
481813	1p	89186388	89186419	32	9	0.556	Promoter(<=1kb)	107	GBP4	115361
481813	1q	152303164	152313891	10728	40	0.6	Promoter(<=1kb)	0	FLG-AS1	339400
481813	1q	156669844	156670886	1043	6	1	Exon(exon4of4)	6521	NES	10763
481813	1q	158765805	158766655	851	6	0.5	Promoter(<=1kb)	47	OR6N1	128372
481813	1q	169542317	169542882	566	6	0.167	Exon(exon13of25)	-26572	F5	2153
481813	1q	226735683	226737239	1557	8	0.875	Promoter(<=1kb)	219	ITPKB	3707
481813	1q	228315976	228318038	2063	8	0.625	Exon(exon50of81)	6492	OBSCN	84033
481813	1q	232805117	232806800	1684	6	0.5	Promoter(<=1kb)	225	MAP10	54627
481813	1q	247841312	247841582	271	6	0.833	Promoter(<=1kb)	314	OR11L1	391189
481813	1q	247949325	247949738	414	9	0.333	Promoter(<=1kb)	467	OR2L8	391190
481813	1q	248294677	248295458	782	7	0.571	Promoter(<=1kb)	142	OR2T12	127064
481813	1q	248626203	248626898	696	6	0.333	Promoter(<=1kb)	292	OR2T11	127077
481813	2p	29002636	29003646	1011	6	0.333	Exon(exon5of20)	-10821	TOGARAM2	165186
481813	2p	48580657	48582454	1798	7	0.571	Promoter(<=1kb)	0	STON1	11037
481813	2q	102351547	102351902	356	7	0.429	Exon(exon11of11)	-4027	IL18R1	8809
481813	2q	132783871	132785012	1142	6	0.5	Promoter(1-2kb)	-1848	NCKAP5	344148
481813	2q	233713134	233713664	531	8	0.75	Promoter(<=1kb)	142	UGT1A5	54579
481813	2q	238130271	238131546	1276	8	0.375	Promoter(1-2kb)	1323	ESPNL	339768
481813	2q	240041845	240042131	287	6	0.167	Downstream(2-3kb)	3941	OR6B3	150681
481813	3p	31989532	31990905	1374	7	0.286	Exon(exon2of2)	7761	ZNF860	344787
481813	3p	75736880	75739007	2128	43	0.605	Promoter(<=1kb)	0	MIR4273	100422955
481813	3q	98264413	98265137	725	8	0.625	Promoter(<=1kb)	128	OR5H6	79295
481813	4p	6300792	6302360	1569	8	0.75	Exon(exon8of8)	6021	WFS1	7466
481813	4p	8227004	8228508	1505	8	0.125	Promoter(<=1kb)	-24	SH3TC1	54436
481813	5p	795818	796218	401	8	0.625	3'UTR	4927	ZDHHC11	79844
481813	5q	83537326	83539905	2580	6	0.333	Promoter(1-2kb)	1712	VCAN	1462
481813	5q	140848579	140850786	2208	6	0.5	Promoter(<=1kb)	807	PCDHA9	9752
481813	5q	141174000	141175025	1026	6	0.833	Promoter(1-2kb)	1356	PCDHB7	56129
481813	5q	141183999	141184688	690	6	0.833	Promoter(2-3kb)	-2473	PCDHB9	56127
481813	5q	141187690	141189425	1736	6	0.5	Promoter(<=1kb)	529	PCDHB9	56127
481813	5q	141955356	141957660	2305	6	0.667	Promoter(<=1kb)	-668	RNF14	9604
481813	5q	148826877	148828070	1194	6	1	Promoter(1-2kb)	1632	ADRB2	154
481813	6p	46858771	46859502	732	8	0.5	Exon(exon17of21)	3802	ADGRF5	221395
481813	6q	159231899	159234370	2472	10	0.5	Exon(exon11of23)	13602	FNDC1	84624
481813	7q	100958977	100960873	1897	55	0.455	Promoter(1-2kb)	1012	MUC3A	4584
481813	7q	100991195	100994057	2863	11	0.727	Exon(exon5of15)	-18997	MUC12	10071
481813	7q	100995547	100995785	239	7	0.857	Exon(exon5of15)	-17269	MUC12	10071

481813	7q	149818015	149819792	1778	6	0.667	Promoter(2-3kb)	-2352	SSPO	23145
481813	8p	10607245	10608432	1188	8	0.5	Exon(exon4of4)	46711	RP1L1	94137
481813	8p	12132686	12133940	1255	7	0.714	Promoter(<=1kb)	498	USP17L7	392197
481813	8p	13021128	13022030	903	8	0.125	Exon(exon5of5)	9115	TRMT9B	57604
481813	8q	142664564	142665970	1407	6	0.833	Exon(exon2of2)	4000	JRK	8629
481813	9p	116800	117934	1135	7	0.714	Promoter(<=1kb)	270	FOXD4	2298
481813	9q	76175241	76175296	56	8	0.875	Exon(exon14of14)	-13343	PCSK5	5125
481813	9q	87887543	87888819	1277	6	0.5	Exon(exon4of4)	4666	SPATA31E1	286234
481813	9q	104598545	104599361	817	12	0.583	Promoter(<=1kb)	52	OR13C5	138799
481813	9q	104694482	104695444	963	6	0.5	Promoter(<=1kb)	0	OR13D1	286365
481813	9q	122553263	122554071	809	7	0.429	Promoter(<=1kb)	93	OR1N2	138882
481813	9q	122628595	122629398	804	6	0.333	Promoter(<=1kb)	175	OR1B1	347169
481813	9q	122749914	122750547	634	6	0.833	Promoter(<=1kb)	174	OR1L6	392390
481813	9q	133255635	133256205	571	7	1	3'UTR	19009	ABO	28
481813	10q	46549378	46550723	1346	25	0.64	Exon(exon3of3)	4807	GPRIN2	9721
481813	10q	49323504	49326817	3314	10	0.5	Exon(exon3of3)	24230	C10orf71	118461
481813	10q	128103129	128104752	1624	8	0.5	Promoter(<=1kb)	-1	MKI67	4288
481813	10q	128106210	128108204	1995	6	1	Exon(exon12of14)	-3082	MKI67	4288
481813	11p	244106	244197	92	8	0.5	Promoter(<=1kb)	-232	PSMD13	5719
481813	11p	1246095	1247378	1284	9	0.556	Promoter(2-3kb)	2298	MUC5B-AS1	112577518
481813	11p	4681935	4682546	612	6	0.833	Promoter(2-3kb)	2546	OR51E2	81285
481813	11p	5177978	5178478	501	6	0.167	Promoter(<=1kb)	186	OR52Z1	283110
481813	11p	5323362	5324256	895	7	0.429	Promoter(<=1kb)	41	OR51B2	79345
481813	11p	5422212	5423123	912	11	0.636	Promoter(<=1kb)	101	OR51Q1	390061
481813	11p	5515185	5515931	747	6	0.333	Promoter(<=1kb)	768	UBQLNL	143630
481813	11p	5581045	5581738	694	8	0.375	Promoter(<=1kb)	168	OR52B6	340980
481813	11p	5841302	5841883	582	10	0.4	Promoter(<=1kb)	14	OR52E6	390078
481813	11p	11351961	11352736	776	9	0.222	Promoter(<=1kb)	514	CSNK2A3	283106
481813	11p	34916266	34916763	498	6	0.667	Promoter(<=1kb)	0	APIP	51074
481813	11p	43942293	43943348	1056	9	0.778	Promoter(<=1kb)	0	C11orf96	387763
481813	11q	55572176	55572903	728	6	0.667	Promoter(<=1kb)	48	OR4C16	219428
481813	11q	58214757	58215722	966	6	0.333	Promoter(<=1kb)	12	OR1S1	219959
481813	11q	85724687	85725825	1139	6	0.5	Promoter(<=1kb)	0	SYTL2	54843
481813	11q	124015600	124016477	878	10	0.3	Promoter(<=1kb)	25	OR10G4	390264
481813	11q	124023038	124023849	812	11	0.364	Promoter(<=1kb)	25	OR10G9	219870
481813	11q	124382526	124383285	760	8	0.625	Promoter(<=1kb)	58	OR8B2	26595
481813	12p	4627329	4628152	824	6	0.333	Exon(exon5of6)	14812	DYRK4	8798
481813	12p	6453119	6453670	552	6	0.667	Promoter(<=1kb)	633	TAPBPL	55080
481813	13q	25096850	25096984	135	7	0.429	Promoter(<=1kb)	982	PABPC3	5042
481813	13q	102732474	102733933	1460	6	0.333	Exon(exon4of4)	25139	CCDC168	643677
481813	14q	20060048	20060523	476	6	0.5	Promoter(<=1kb)	3	OR4L1	122742
481813	14q	20640750	20641567	818	7	0.571	Promoter(<=1kb)	124	OR6S1	341799
481813	14q	22633879	22634450	572	9	0.333	Exon(exon2of2)	32212	ABHD4	63874
481813	14q	44504986	44506403	1418	6	0.667	Promoter(<=1kb)	880	FSCB	84075
481813	14q	70457532	70458540	1009	9	0.333	Exon(exon2of2)	5358	ADAM21	8747
481813	14q	104939262	104945444	6183	21	0.381	5'UTR	7102	PLD4	122618
481813	14q	104946867	104953878	7012	36	0.528	Promoter(1-2kb)	1524	AHNAK2	113146
481813	15q	23439979	23442067	2089	12	0.583	5'UTR	5167	GOLGA6L2	283685
481813	15q	85579423	85581800	2378	15	0.6	Promoter(1-2kb)	-1110	AKAP13	11214
481813	15q	99129423	99132517	3095	6	0.333	Exon(exon4of5)	7225	TTC23	64927
481813	15q	100569472	100570097	626	6	0.833	Promoter(<=1kb)	534	LINS1	55180
481813	16p	1486322	1488463	2142	11	0.636	Promoter(<=1kb)	4	PTX4	390667
481813	16p	4207130	4208004	875	6	0.5	Exon(exon2of7)	31739	SRL	6345
481813	16q	88428416	88429600	1185	6	0.333	Exon(exon3of3)	-23680	ZFPM1	161882
481813	16q	89100686	89101050	365	7	0.571	Promoter(<=1kb)	24	ACSF3	197322
481813	16q	89226863	89228289	1427	8	0.625	Promoter(2-3kb)	2229	ZNF778	197320
481813	17p	10638198	10641099	2902	6	0.333	Exon(exon19of41)	-8169	MYH3	4621
481813	17p	21300581	21300954	374	10	0.7	3'UTR	9112	MAP2K3	5606
481813	17q	53823368	53824891	1524	6	0.833	Promoter(<=1kb)	441	KIF2B	84643
481813	17q	76293419	76294016	598	6	0.5	Promoter(2-3kb)	-2167	QRICH2	84074
481813	17q	81645135	81645417	283	6	0.333	Promoter(2-3kb)	2722	TSPAN10	83882
481813	18p	11609904	11610469	566	8	0.625	Promoter(<=1kb)	308	SLC35G4	646000
481813	18q	58534888	58538030	3143	22	0.545	Promoter(<=1kb)	0	ALPK2	115701
481813	19p	4510548	4513547	3000	22	0.455	Exon(exon3of6)	4157	PLIN4	729359
481813	19p	5455600	5456439	840	8	0.625	Promoter(<=1kb)	183	ZNRF4	148066
481813	19p	8333830	8334965	1136	6	0.667	Promoter(<=1kb)	0	KANK3	256949
481813	19p	8948231	8950136	1906	7	0.571	Exon(exon3of84)	31206	MUC16	94025
481813	19p	8959518	8962066	2549	6	0.333	Exon(exon3of84)	19276	MUC16	94025
481813	19p	8964274	8967127	2854	19	0.632	Exon(exon3of84)	14215	MUC16	94025
481813	19p	15087213	15088040	828	11	0.273	Promoter(<=1kb)	233	OR1I1	126370
481813	19p	15794192	15794719	528	6	0.333	Promoter(<=1kb)	241	OR10H5	284433
481813	19p	21971930	21974500	2571	9	0.667	Exon(exon4of4)	14408	ZNF208	7757
481813	19p	22756205	22759523	3319	14	0.571	3'UTR	10459	ZNF99	7652
481813	19q	39877222	39877880	659	6	0.5	Exon(exon20of28)	9412	FCGBP	8857
481813	19q	39886240	39886422	183	6	0.667	Promoter(<=1kb)	870	FCGBP	8857
481813	19q	43846955	43848536	1582	6	0.833	3'UTR	13450	ZNF283	284349
481813	19q	43913423	43914878	1456	8	0.5	Exon(exon10of10)	4861	ZNF45	7596

481813	19q	43966037	43967171	1135	6	0.167	Promoter(<=1kb)	-691	ZNF155	7711
481813	19q	44106512	44108078	1567	8	0.125	Exon(exon6of6)	-4103	ZNF225	7768
481813	19q	48873325	48875925	2601	10	0.5	Promoter(<=1kb)	904	PPP1R15A	23645
481813	19q	52437918	52439242	1325	7	0.429	Exon(exon4of4)	6504	ZNF534	147658
481813	19q	53164551	53166239	1689	6	0.167	Exon(exon4of4)	-5476	ZNF347	84671
481813	19q	55481625	55483456	1832	7	0.429	Promoter(1-2kb)	-1732	NAT14	57106
481813	19q	55517821	55518176	356	11	0.727	Exon(exon14of14)	18127	SBK2	646643
481813	19q	55763087	55763165	79	6	0.667	Exon(exon3of3)	4073	RFPL4A	342931
481813	19q	55912302	55913166	865	6	0.667	Exon(exon5of12)	19145	NLRP13	126204
481813	19q	58368293	58368875	583	7	0.429	Exon(exon3of3)	-5445	ZNF497	162968
481813	20p	5922421	5923643	1223	9	0.667	Exon(exon4of5)	6923	CHGB	1114
481813	20q	63349752	63350772	1021	6	0.5	3'UTR	3794	CHRNA4	1137
481813	20q	63561666	63565531	3866	11	0.636	Promoter(<=1kb)	-61	HELZ2	85441
481813	21q	41740862	41741990	1129	6	0.833	Exon(exon2of2)	4851	MIR6814	102465488
481813	21q	44550835	44551416	582	6	0.833	Promoter(<=1kb)	89	KRTAP10-2	386679
481813	21q	44637476	44638143	668	6	0.667	Promoter(<=1kb)	120	KRTAP10-10	353333
481813	22q	22352950	22353380	431	16	0.5	Exon(exon1of2)	30478	BMS1P20	96610
481813	22q	22758758	22759209	452	6	0.833	Exon(exon1of2)	-63567	MIR650	723778
481813	22q	36191154	36191906	753	6	0.667	3'UTR	9971	APOL4	80832
481813	22q	39100331	39102033	1703	6	0.833	Promoter(<=1kb)	52	APOBEC3H	164668
481813	22q	49883704	49884994	1291	6	0.167	Exon(exon2of2)	22924	ALG12	79087
481813	23p	3320126	3323750	3625	9	0.556	Exon(exon5of7)	22902	MXRA5	25878
481813	23p	8170039	8170141	103	6	0.5	Promoter(1-2kb)	1126	VCX2	51480
481813	23p	35802148	35803010	863	7	0.571	5'UTR	3357	MAGEB16	139604
482217	1p	978953	980691	1739	7	0.571	Promoter(<=1kb)	338	PERM1	84808
482217	1p	12847526	12847995	470	8	0.5	Promoter(<=1kb)	730	HNRNPCL1	343069
482217	1p	12859036	12860079	1044	6	0.333	Promoter(1-2kb)	1950	PRAMEF2	65122
482217	1p	13369166	13369564	399	9	0.778	Promoter(2-3kb)	2336	PRAMEF19	645414
482217	1p	13370686	13371119	434	7	0.429	Promoter(<=1kb)	781	PRAMEF19	645414
482217	1p	16058491	16060000	1510	10	0.9	Exon(exon5of7)	6168	CLCNKB	1188
482217	1p	18481403	18483159	1757	7	0.714	Promoter(<=1kb)	421	KLHDC7A	127707
482217	1p	23874604	23875430	827	8	0.5	Exon(exon2of2)	-6310	FUCA1	2517
482217	1p	40067594	40067675	82	6	0	Promoter(<=1kb)	324	CAP1	10487
482217	1p	89186388	89186419	32	9	0.556	Promoter(<=1kb)	107	GBP4	115361
482217	1q	152303673	152313891	10219	39	0.59	Promoter(<=1kb)	0	FLG-AS1	339400
482217	1q	156669844	156671745	1902	7	1	Exon(exon4of4)	5662	NES	10763
482217	1q	158765805	158766655	851	6	0.5	Promoter(<=1kb)	47	OR6N1	128372
482217	1q	247841312	247841582	271	6	0.833	Promoter(<=1kb)	314	OR11L1	391189
482217	1q	247896121	247896502	382	6	0.5	Promoter(<=1kb)	534	OR2W3	343171
482217	1q	247921162	247921930	769	6	0.667	Promoter(<=1kb)	144	OR2T8	343172
482217	1q	247949436	247949759	324	8	0.375	Promoter(<=1kb)	578	OR2L8	391190
482217	2p	29071763	29073000	1238	6	0.667	Promoter(1-2kb)	1523	PCARE	388939
482217	2p	48580657	48582454	1798	7	0.571	Promoter(<=1kb)	0	STON1	11037
482217	2q	130193975	130195152	1178	6	0.667	Exon(exon3of5)	3287	TUBA3E	112714
482217	2q	178739433	178741811	2379	6	0.5	Exon(exon45of191)	26014	TTN	7273
482217	2q	184936178	184937636	1459	6	0.333	Exon(exon4of4)	69813	ZNF804A	91752
482217	2q	185789865	185794632	4768	10	0.8	Promoter(<=1kb)	0	FSIP2	401024
482217	2q	185805377	185808170	2794	6	0.333	Promoter(<=1kb)	0	FSIP2	401024
482217	2q	217847567	217848213	647	6	0.833	Exon(exon19of33)	-5407	TNS1	7145
482217	2q	219489386	219491798	2413	6	0.5	Promoter(<=1kb)	-69	SPEGNB	100996693
482217	2q	240041845	240042840	996	7	0.143	Downstream(2-3kb)	3232	OR6B3	150681
482217	3p	31989643	31990470	828	6	0.167	Exon(exon2of2)	7872	ZNF860	344787
482217	3p	75737230	75739007	1778	9	0.556	Promoter(<=1kb)	0	MIR4273	100422955
482217	3q	194341097	194342571	1475	7	0.714	Exon(exon2of2)	8747	CPN2	1370
482217	4p	5988383	5989749	1367	7	0.571	Promoter(<=1kb)	0	C4orf50	389197
482217	4p	6300792	6302360	1569	6	0.833	Exon(exon8of8)	6021	WFS1	7466
482217	4p	8227004	8228508	1505	8	0.125	Promoter(<=1kb)	-24	SH3TC1	54436
482217	4q	121036404	121037542	1139	6	0.333	Promoter(1-2kb)	1442	NDNF	79625
482217	4q	185458217	185460011	1795	8	0.625	Promoter(<=1kb)	0	CCDC110	256309
482217	4q	186619481	186621601	2121	7	0.286	Exon(exon10of27)	-9411	FAT1	2195
482217	4q	186706638	186708616	1979	6	0.667	Exon(exon2of27)	15217	FAT1	2195
482217	5p	796157	796237	81	6	0.333	3'UTR	4908	ZDHHC11	79844
482217	5q	79731782	79734523	2742	13	0.308	Exon(exon2of13)	-3619	CMYA5	202333
482217	5q	141174000	141175025	1026	6	0.833	Promoter(1-2kb)	1356	PCDHB7	56129
482217	5q	141183999	141184688	690	7	0.857	Promoter(2-3kb)	-2473	PCDHB9	56127
482217	5q	141187690	141189425	1736	6	0.5	Promoter(<=1kb)	529	PCDHB9	56127
482217	5q	148826877	148828070	1194	6	1	Promoter(1-2kb)	1632	ADRB2	154
482217	5q	151521550	151522069	520	6	0.667	Promoter(<=1kb)	79	MIR6499	102465246
482217	6p	16327099	16327684	586	8	0.5	Exon(exon8of9)	37154	GMPR	2766
482217	6p	46858771	46859502	732	7	0.571	Exon(exon17of21)	3802	ADGRF5	221395
482217	6q	106511549	106512572	1024	6	0.667	Promoter(<=1kb)	0	CRYBG1	202
482217	6q	159231899	159234370	2472	10	0.5	Exon(exon11of23)	13602	FNDC1	84624
482217	7p	6330446	6330944	499	6	1	Exon(exon2of2)	7749	FAM220A	84792
482217	7q	100958977	100960873	1897	48	0.479	Promoter(1-2kb)	1012	MUC3A	4584
482217	7q	100991195	100993127	1933	9	0.667	Exon(exon5of15)	-19927	MUC12	10071
482217	7q	100995575	100995785	211	7	0.714	Exon(exon5of15)	-17269	MUC12	10071
482217	7q	101004421	101004836	416	6	0.667	Exon(exon5of15)	-8218	MUC12	10071

482217	7q	101034361	101040583	6223	38	0.5	Exon(exon3of12)	-3128	MUC17	140453
482217	8p	8376561	8377994	1434	6	1	Exon(exon2of5)	3753	PRAG1	157285
482217	8p	11331234	11332082	849	6	0.667	Promoter(<=1kb)	346	SLC35G5	83650
482217	8p	12132686	12133940	1255	6	0.667	Promoter(<=1kb)	498	USP17L7	392197
482217	8p	13021128	13022030	903	7	0.143	Exon(exon5of5)	9115	TRMT9B	57604
482217	8q	142664573	142665852	1280	6	0.833	Exon(exon2of2)	4118	JRK	8629
482217	8q	143916185	143919209	3025	8	0.25	Exon(exon32of32)	20381	PLEC	5339
482217	9q	76705179	76707804	2626	8	0.5	Promoter(<=1kb)	121	PCA3	50652
482217	9q	76709263	76710843	1581	9	0.556	Promoter(<=1kb)	0	PRUNE2	158471
482217	9q	122553263	122554071	809	7	0.429	Promoter(<=1kb)	93	OR1N2	138882
482217	9q	122628595	122629130	536	6	0.333	Promoter(<=1kb)	443	OR1B1	347169
482217	9q	122749914	122750547	634	6	0.833	Promoter(<=1kb)	174	OR1L6	392390
482217	9q	128907497	128909159	1663	7	0.429	Exon(exon3of4)	-3585	LRRC8A	56262
482217	9q	133255635	133256205	571	7	1	3'UTR	19009	ABO	28
482217	9q	135484803	135487213	2411	8	0.25	Promoter(1-2kb)	1440	PPP1R26	9858
482217	10p	47663	48605	943	6	0.833	Promoter(<=1kb)	664	TUBB8	347688
482217	10q	46549378	46550723	1346	25	0.64	Exon(exon3of3)	4807	GPRIN2	9721
482217	10q	49323169	49325192	2024	7	0.571	Exon(exon3of3)	23895	C10orf71	118461
482217	10q	128103129	128104752	1624	8	0.5	Promoter(<=1kb)	-1	MKI67	4288
482217	11p	244106	244197	92	8	0.5	Promoter(<=1kb)	-232	PSMD13	5719
482217	11p	1194354	1196902	2549	7	0.571	Exon(exon34of49)	-26164	MUC5B	727897
482217	11p	1246332	1248605	2274	10	0.5	Promoter(1-2kb)	1071	MUC5B-AS1	112577518
482217	11p	5177811	5178478	668	7	0.143	Promoter(<=1kb)	186	OR52Z1	283110
482217	11p	5323542	5324256	715	6	0.5	Promoter(<=1kb)	41	OR51B2	79345
482217	11p	5422212	5423123	912	11	0.636	Promoter(<=1kb)	101	OR51Q1	390061
482217	11p	5581045	5581738	694	8	0.375	Promoter(<=1kb)	168	OR52B6	340980
482217	11p	5788000	5788760	761	8	0.75	Promoter(<=1kb)	56	OR52N1	79473
482217	11p	5841302	5841883	582	9	0.333	Promoter(<=1kb)	14	OR52E6	390078
482217	11p	11351961	11352736	776	9	0.222	Promoter(<=1kb)	514	CSNK2A3	283106
482217	11p	12293639	12294842	1204	7	0.714	Exon(exon29of35)	6739	MICALCL	84953
482217	11p	18173280	18173901	622	6	0.333	Promoter(<=1kb)	443	MRGPRX4	117196
482217	11q	55827536	55827640	105	6	0.667	Promoter(<=1kb)	317	OR5L2	26338
482217	11q	58214757	58215722	966	8	0.25	Promoter(<=1kb)	12	OR1S1	219959
482217	11q	85724687	85725825	1139	6	0.5	Promoter(<=1kb)	0	SYTL2	54843
482217	11q	123906790	123907324	535	6	0.667	Promoter(<=1kb)	644	OR8D4	338662
482217	11q	123943088	123943782	695	7	0.571	Promoter(<=1kb)	91	OR6T1	219874
482217	11q	124015601	124016477	877	7	0.143	Promoter(<=1kb)	26	OR10G4	390264
482217	11q	124023038	124023849	812	9	0.444	Promoter(<=1kb)	25	OR10G9	219870
482217	11q	124038366	124038988	623	9	0.889	Promoter(<=1kb)	13	OR10G7	390265
482217	11q	124382526	124383285	760	8	0.625	Promoter(<=1kb)	58	OR8B2	26595
482217	12p	4626568	4628549	1982	11	0.545	Exon(exon5of6)	14051	DYRK4	8798
482217	12p	31981273	31982507	1235	6	0.333	Exon(exon4of6)	-4659	RESF1	55196
482217	13q	24434450	24435347	898	7	0.571	Exon(exon31of34)	19787	PARP4	143
482217	13q	102732474	102733933	1460	6	0.333	Exon(exon4of4)	25139	CCDC168	643677
482217	14q	20640750	20641567	818	7	0.571	Promoter(<=1kb)	124	OR6S1	341799
482217	14q	70457745	70458948	1204	7	0.429	Exon(exon2of2)	5571	ADAM21	8747
482217	14q	104947794	104951938	4145	12	0.417	Exon(exon6of6)	3464	AHNAK2	113146
482217	15q	20534480	20535014	535	6	0.333	Exon(exon8of9)	6786	GOLGA6L6	727832
482217	15q	23439979	23442067	2089	12	0.583	5'UTR	5167	GOLGA6L2	283685
482217	15q	40621642	40623696	2055	6	0.333	Promoter(<=1kb)	0	KNL1	57082
482217	15q	52609086	52609780	695	6	0.333	Promoter(<=1kb)	-490	FAM214A	56204
482217	15q	99129423	99132854	3432	7	0.429	3'UTR	6888	TTC23	64927
482217	16p	789084	790597	1514	6	0.167	Promoter(<=1kb)	0	CHTF18	63922
482217	16p	1486371	1488463	2093	8	0.75	Promoter(<=1kb)	4	PTX4	390667
482217	16p	4883938	4885635	1698	6	1	Exon(exon22of22)	4701	PPL	5493
482217	16p	27362551	27363079	529	6	0.167	3'UTR	7203	IL4R	3566
482217	16q	89100686	89101050	365	9	0.667	Promoter(<=1kb)	24	ACSF3	197322
482217	16q	89227206	89228419	1214	8	0.625	Promoter(2-3kb)	2572	ZNF778	197320
482217	17p	21300581	21300978	398	12	0.75	3'UTR	9112	MAP2K3	5606
482217	17q	76293419	76294016	598	6	0.5	Promoter(2-3kb)	-2167	QRICH2	84074
482217	17q	81645135	81645417	283	6	0.333	Promoter(2-3kb)	2722	TSPAN10	83882
482217	18p	9886991	9888072	1082	7	0.857	Promoter(<=1kb)	977	TXNDC2	84203
482217	18p	11609646	11610363	718	12	0.833	Promoter(<=1kb)	50	SLC35G4	646000
482217	18p	14542649	14543140	492	6	0.5	Promoter(<=1kb)	6	POTEC	388468
482217	18q	58535186	58537515	2330	10	0.4	Promoter(<=1kb)	0	ALPK2	115701
482217	19p	1003159	1003440	282	6	1	Promoter(2-3kb)	2740	GRIN3B	116444
482217	19p	1036457	1036914	458	6	0.5	Exon(exon6of7)	-3187	ABCA7	10347
482217	19p	4510548	4513547	3000	17	0.471	Exon(exon3of6)	4157	PLIN4	729359
482217	19p	5455600	5456439	840	6	0.5	Promoter(<=1kb)	183	ZNRF4	148066
482217	19p	8333830	8334965	1136	7	0.714	Promoter(<=1kb)	0	KANK3	256949
482217	19p	8946313	8951868	5556	15	0.6	Exon(exon3of84)	29474	MUC16	94025
482217	19p	8959116	8962299	3184	10	0.7	Exon(exon3of84)	19043	MUC16	94025
482217	19p	8972751	8978096	5346	16	0.5	Exon(exon1of84)	3246	MUC16	94025
482217	19p	12430157	12431840	1684	9	0.444	3'UTR	9181	ZNF443	10224
482217	19p	17282085	17284246	2162	8	0.5	Promoter(<=1kb)	0	ANKLE1	126549
482217	19p	21971930	21974500	2571	10	0.7	Exon(exon4of4)	14408	ZNF208	7757
482217	19p	23743906	23745300	1395	6	0.333	Exon(exon4of4)	13537	ZNF681	148213

482217	19q	39886240	39886422	183	6	0.667	Promoter(<=1kb)	870	FCGBP	8857
482217	19q	40880231	40880622	392	6	0.333	Promoter(<=1kb)	-141	CYP2A7	1549
482217	19q	43846955	43848536	1582	6	0.833	3'UTR	13450	ZNF283	284349
482217	19q	43913423	43914878	1456	8	0.5	Exon(exon10of10)	4861	ZNF45	7596
482217	19q	44106512	44108078	1567	7	0	Exon(exon6of6)	-4103	ZNF225	7768
482217	19q	51872787	51873699	913	10	0.5	Promoter(<=1kb)	-940	ZNF577	84765
482217	19q	52437918	52439242	1325	7	0.429	Exon(exon4of4)	6504	ZNF534	147658
482217	19q	55517821	55518642	822	8	1	Exon(exon14of14)	17661	SBK2	646643
482217	19q	55857823	55858800	978	6	0.833	Promoter(<=1kb)	0	NLRP4	147945
482217	19q	55911888	55913077	1190	6	0.667	Exon(exon5of12)	19234	NLRP13	126204
482217	19q	58416932	58417769	838	6	0.5	3'UTR	8155	ZNF584	201514
482217	20p	5922421	5923394	974	6	0.5	Exon(exon4of5)	6923	CHGB	1114
482217	20q	63349752	63350772	1021	6	0.5	3'UTR	3794	CHRNA4	1137
482217	20q	63562677	63565531	2855	12	0.583	Promoter(1-2kb)	-1072	HELZ2	85441
482217	21q	26843740	26844859	1120	6	0.667	Promoter(<=1kb)	0	ADAMTS1	9510
482217	21q	44539312	44540035	724	6	1	Promoter(<=1kb)	160	KRTAP10-1	386677
482217	21q	44558207	44558709	503	6	0.333	Promoter(<=1kb)	86	KRTAP10-3	386682
482217	21q	44637474	44638041	568	9	0.333	Promoter(<=1kb)	118	KRTAP10-10	353333
482217	22q	22352950	22353298	349	13	0.538	Exon(exon1of2)	30478	BMS1P20	96610
482217	22q	22758758	22759209	452	6	0.833	Exon(exon1of2)	-63567	MIR650	723778
482217	22q	36191154	36191906	753	7	0.571	3'UTR	9971	APOL4	80832
482217	22q	49884187	49884994	808	6	0.333	Exon(exon2of2)	22924	ALG12	79087
482217	23p	8170039	8170141	103	6	0.5	Promoter(1-2kb)	1126	VCX2	51480
482217	23p	35802148	35803010	863	7	0.571	5'UTR	3357	MAGEB16	139604
482217	23q	102937373	102937765	393	7	0.571	Promoter(<=1kb)	101	RAB40AL	282808
485342	1p	11766028	11768307	2280	6	0.833	Promoter(<=1kb)	0	C1orf167	284498
485342	1p	11778784	11779941	1158	7	0.714	Promoter(<=1kb)	0	C1orf167-AS1	102724659
485342	1p	12029085	12030097	1013	6	0.833	Promoter(<=1kb)	0	MIR6729	102466982
485342	1p	12847526	12847995	470	9	0.556	Promoter(<=1kb)	730	HNRNPCL1	343069
485342	1p	12859036	12860079	1044	6	0.333	Promoter(1-2kb)	1950	PRAMEF2	65122
485342	1p	13370686	13371119	434	8	0.375	Promoter(<=1kb)	781	PRAMEF19	645414
485342	1p	16058491	16060000	1510	10	0.9	Exon(exon5of7)	6168	CLCNKB	1188
485342	1p	18481403	18483159	1757	7	0.714	Promoter(<=1kb)	421	KLHDC7A	127707
485342	1p	23874604	23875430	827	9	0.556	Exon(exon2of2)	-6310	FUCA1	2517
485342	1p	40067594	40067675	82	6	0	Promoter(<=1kb)	324	CAP1	10487
485342	1q	152213274	152213320	47	8	0.5	Exon(exon3of3)	10873	HRNR	388697
485342	1q	152218469	152221235	2767	15	0.6	Promoter(2-3kb)	2958	HRNR	388697
485342	1q	152303164	152313891	10728	55	0.673	Promoter(<=1kb)	0	FLG-AS1	339400
485342	1q	158765805	158766655	851	6	0.5	Promoter(<=1kb)	47	OR6N1	128372
485342	1q	197101312	197101771	460	6	0.5	Exon(exon18of28)	33373	ASPM	259266
485342	1q	223393517	223394466	950	6	0.667	Promoter(<=1kb)	102	CCDC185	164127
485342	1q	226735683	226737239	1557	7	0.857	Promoter(<=1kb)	219	ITPKB	3707
485342	1q	247841312	247841582	271	6	0.833	Promoter(<=1kb)	314	OR11L1	391189
485342	1q	247949325	247949738	414	10	0.3	Promoter(<=1kb)	467	OR2L8	391190
485342	1q	248294677	248295458	782	7	0.571	Promoter(<=1kb)	142	OR2T12	127064
485342	1q	248681658	248682198	541	7	0.571	Promoter(<=1kb)	130	OR14I1	401994
485342	2p	48580657	48582454	1798	7	0.571	Promoter(<=1kb)	0	STON1	11037
485342	2q	130193975	130194275	301	6	0.5	Exon(exon4of5)	4164	TUBA3E	112714
485342	2q	132783061	132785001	1941	9	0.444	Promoter(1-2kb)	-1038	NCKAP5	344148
485342	2q	185789865	185794632	4768	10	0.7	Promoter(<=1kb)	0	FSIP2	401024
485342	2q	185805377	185808170	2794	6	0.333	Promoter(<=1kb)	0	FSIP2	401024
485342	2q	219271337	219271649	313	6	0.667	Exon(exon4of4)	6227	TUBA4A	7277
485342	2q	233681970	233682693	724	6	0.667	Promoter(<=1kb)	0	UGT1A7	54577
485342	2q	233713134	233713664	531	8	0.75	Promoter(<=1kb)	142	UGT1A5	54579
485342	2q	238130416	238131546	1131	6	0.333	Promoter(1-2kb)	1468	ESPNL	339768
485342	2q	240041845	240042811	967	7	0.286	Downstream(2-3kb)	3261	OR6B3	150681
485342	3p	31989532	31990905	1374	6	0.333	Exon(exon2of2)	7761	ZNF860	344787
485342	3p	75736880	75739007	2128	53	0.604	Promoter(<=1kb)	0	MIR4273	100422955
485342	3q	194359607	194360906	1300	9	0.889	Exon(exon2of2)	-8279	CPN2	1370
485342	4p	5988383	5989749	1367	7	0.571	Promoter(<=1kb)	0	C4orf50	389197
485342	4p	6300792	6302360	1569	6	0.833	Exon(exon8of8)	6021	WFS1	7466
485342	4p	7433331	7434512	1182	6	0.833	Promoter(<=1kb)	418	PSAPL1	768239
485342	5p	795818	796218	401	6	0.833	3'UTR	4927	ZDHHC11	79844
485342	5q	83537326	83539905	2580	6	0.333	Promoter(1-2kb)	1712	VCAN	1462
485342	5q	140807352	140807737	386	6	0.833	Promoter(<=1kb)	271	PCDHA4	56144
485342	5q	140848579	140850786	2208	6	0.5	Promoter(<=1kb)	807	PCDHA9	9752
485342	5q	141174000	141175025	1026	6	0.833	Promoter(1-2kb)	1356	PCDHB7	56129
485342	5q	141183999	141184688	690	6	0.833	Promoter(2-3kb)	-2473	PCDHB9	56127
485342	5q	151565922	151568158	2237	9	0.778	Promoter(<=1kb)	786	FAT2	2196
485342	6p	46858771	46859389	619	6	0.5	Exon(exon17of21)	3915	ADGRF5	221395
485342	6q	149888581	149889987	1407	6	0.667	Promoter(<=1kb)	-116	RAET1E-AS1	100652739
485342	6q	159233455	159234370	916	10	0.5	Exon(exon11of23)	15158	FNDC1	84624
485342	7p	6330446	6330944	499	6	1	Exon(exon2of2)	7749	FAM220A	84792
485342	7p	12369637	12370736	1100	6	0.667	3'UTR	-13307	VWDE	221806
485342	7p	45082725	45084866	2142	7	0.429	Promoter(1-2kb)	-1325	NACAD	23148
485342	7p	53035678	53036385	708	7	1	Promoter(<=1kb)	45	POM121L12	285877
485342	7p	56021087	56021209	123	6	0.5	Exon(exon2of7)	12947	PSPH	5723

485342	7q	64708014	64708641	628	7	0.571	Exon(exon3of3)	18659	ZNF107	51427
485342	7q	100958977	100960873	1897	50	0.46	Promoter(1-2kb)	1012	MUC3A	4584
485342	7q	100991195	100993127	1933	9	0.667	Exon(exon5of15)	-19927	MUC12	10071
485342	7q	100995547	100996039	493	9	0.889	Exon(exon5of15)	-17015	MUC12	10071
485342	7q	149817984	149819792	1809	7	0.714	Promoter(2-3kb)	-2352	SSPO	23145
485342	7q	149824094	149826495	2402	6	0.667	Promoter(<=1kb)	0	SSPO	23145
485342	8p	10607245	10608432	1188	8	0.5	Exon(exon4of4)	46711	RP1L1	94137
485342	8p	10609614	10610142	529	7	0.429	Exon(exon4of4)	45001	RP1L1	94137
485342	8p	11331234	11332082	849	6	0.667	Promoter(<=1kb)	346	SLC35G5	83650
485342	8p	12132477	12133936	1460	8	0.625	Promoter(<=1kb)	502	USP17L7	392197
485342	8p	13021128	13022030	903	7	0.143	Exon(exon5of5)	9115	TRMT9B	57604
485342	8q	123651655	123652634	980	7	0.571	Promoter(<=1kb)	316	KLHL38	340359
485342	8q	143916360	143919209	2850	7	0.143	Exon(exon32of32)	20381	PLEC	5339
485342	9p	712060	713307	1248	7	0.714	Exon(exon7of16)	5171	KANK1	23189
485342	9p	21206764	21207074	311	6	0.5	Promoter(<=1kb)	69	IFNA10	3446
485342	9q	76175237	76175296	60	10	0.8	Exon(exon14of14)	-13343	PCSK5	5125
485342	9q	76705724	76707804	2081	6	0.5	Promoter(<=1kb)	666	PCA3	50652
485342	9q	87887543	87888819	1277	6	0.5	Exon(exon4of4)	4666	SPATA31E1	286234
485342	9q	104598545	104599361	817	12	0.583	Promoter(<=1kb)	52	OR13C5	138799
485342	9q	122749914	122750547	634	6	0.833	Promoter(<=1kb)	174	OR1L6	392390
485342	9q	135484803	135486007	1205	6	0.333	Promoter(1-2kb)	1440	PPP1R26	9858
485342	10q	46549378	46550723	1346	25	0.64	Exon(exon3of3)	4807	GPRIN2	9721
485342	10q	49323559	49326319	2761	9	0.444	Exon(exon3of3)	24285	C10orf71	118461
485342	10q	128102802	128104752	1951	10	0.5	Promoter(<=1kb)	0	MKI67	4288
485342	11p	244106	244197	92	8	0.5	Promoter(<=1kb)	-232	PSMD13	5719
485342	11p	1241677	1243593	1917	8	0.5	Exon(exon31of49)	6083	MUC5B-AS1	112577518
485342	11p	1244757	1248605	3849	14	0.643	Promoter(1-2kb)	1071	MUC5B-AS1	112577518
485342	11p	1250091	1251628	1538	9	0.778	Promoter(<=1kb)	-415	MUC5B-AS1	112577518
485342	11p	5177978	5178478	501	6	0.167	Promoter(<=1kb)	186	OR52Z1	283110
485342	11p	5422212	5423123	912	11	0.636	Promoter(<=1kb)	101	OR51Q1	390061
485342	11p	5515185	5515931	747	6	0.333	Promoter(<=1kb)	768	UBQLNL	143630
485342	11p	5581045	5581738	694	9	0.333	Promoter(<=1kb)	168	OR52B6	340980
485342	11p	5841302	5841883	582	9	0.333	Promoter(<=1kb)	14	OR52E6	390078
485342	11p	11351961	11352736	776	9	0.222	Promoter(<=1kb)	514	CSNK2A3	283106
485342	11p	12293639	12294842	1204	7	0.714	Exon(exon29of35)	6739	MICALCL	84953
485342	11q	55827536	55827640	105	6	0.667	Promoter(<=1kb)	317	OR5L2	26338
485342	11q	58214757	58215722	966	8	0.25	Promoter(<=1kb)	12	OR1S1	219959
485342	11q	64116513	64118232	1720	7	0.714	Exon(exon2of2)	8702	MACROD1	28992
485342	11q	66560202	66562261	2060	6	0.333	Exon(exon14of21)	3873	CTSF	8722
485342	11q	85724687	85725825	1139	6	0.5	Promoter(<=1kb)	0	SYTL2	54843
485342	11q	123906595	123907324	730	7	0.429	Promoter(<=1kb)	449	OR8D4	338662
485342	11q	124015600	124016477	878	9	0.333	Promoter(<=1kb)	25	OR10G4	390264
485342	11q	124023038	124023849	812	10	0.4	Promoter(<=1kb)	25	OR10G9	219870
485342	11q	124382526	124383285	760	8	0.625	Promoter(<=1kb)	58	OR8B2	26595
485342	12p	4626568	4628549	1982	11	0.455	Exon(exon5of6)	14051	DYRK4	8798
485342	12p	8222174	8223514	1341	6	0.667	Exon(exon5of6)	4073	FAM90A1	55138
485342	13q	24434450	24435347	898	7	0.571	Exon(exon31of34)	19787	PARP4	143
485342	13q	25096713	25097182	470	13	0.385	Promoter(<=1kb)	845	PABPC3	5042
485342	13q	49668389	49668431	43	6	0.167	Exon(exon3of3)	23007	EBPL	84650
485342	13q	102732474	102733933	1460	7	0.429	Exon(exon4of4)	25139	CCDC168	643677
485342	14q	20060048	20060884	837	8	0.625	Promoter(<=1kb)	3	OR4L1	122742
485342	14q	20223729	20224484	756	7	0.571	Promoter(<=1kb)	19	OR11H6	122748
485342	14q	20640982	20641567	586	6	0.5	Promoter(<=1kb)	124	OR6S1	341799
485342	14q	44504986	44506403	1418	6	0.667	Promoter(<=1kb)	880	FSCB	84075
485342	14q	70457520	70458540	1021	12	0.333	Exon(exon2of2)	5346	ADAM21	8747
485342	14q	94587512	94587839	328	6	0.5	Exon(exon2of2)	-4219	SERPINA3	12
485342	14q	104939262	104945444	6183	20	0.4	5'UTR	7102	PLD4	122618
485342	14q	104946867	104953878	7012	36	0.528	Promoter(1-2kb)	1524	AHNAK2	113146
485342	15q	23439979	23442067	2089	12	0.583	5'UTR	5167	GOLGA6L2	283685
485342	15q	40621642	40623696	2055	6	0.333	Promoter(<=1kb)	0	KNL1	57082
485342	15q	85579423	85581800	2378	14	0.571	Promoter(1-2kb)	-1110	AKAP13	11214
485342	15q	88854874	88855594	721	6	0.833	Exon(exon12of18)	7631	ACAN	176
485342	15q	88857108	88859365	2258	6	0	Exon(exon12of18)	9865	ACAN	176
485342	15q	99129423	99132517	3095	7	0.429	Exon(exon4of5)	7225	TTC23	64927
485342	15q	100569472	100570097	626	6	0.833	Promoter(<=1kb)	534	LINS1	55180
485342	16p	1228707	1229622	916	7	0.857	Promoter(<=1kb)	540	TPSB2	64499
485342	16p	1486371	1488463	2093	8	0.75	Promoter(<=1kb)	4	PTX4	390667
485342	16p	1998795	2000191	1397	6	0.667	Promoter(<=1kb)	0	ZNF598	90850
485342	16p	4207130	4208004	875	6	0.5	Exon(exon2of7)	31739	SRL	6345
485342	16q	74391401	74391928	528	7	0.714	Exon(exon7of7)	13523	NPIPB15	440348
485342	16q	89100686	89101050	365	7	0.571	Promoter(<=1kb)	24	ACSF3	197322
485342	16q	89226863	89228289	1427	8	0.625	Promoter(2-3kb)	2229	ZNF778	197320
485342	17p	21300581	21300954	374	8	0.75	3'UTR	9112	MAP2K3	5606
485342	17p	21415470	21416404	935	11	0.818	Exon(exon3of3)	10334	KCNJ12	3768
485342	17q	76293419	76294016	598	6	0.5	Promoter(2-3kb)	-2167	QRICH2	84074
485342	17q	80090158	80090265	108	6	0.333	Promoter(2-3kb)	2053	CCDC40	55036
485342	17q	81645135	81645607	473	7	0.286	Promoter(2-3kb)	2722	TSPAN10	83882

485342	18p	11609646	11610509	864	15	0.733	Promoter(<=1kb)	50	SLC35G4	646000
485342	18q	58535186	58537515	2330	9	0.333	Promoter(<=1kb)	0	ALPK2	115701
485342	19p	4510711	4513547	2837	19	0.474	Exon(exon3of6)	4157	PLIN4	729359
485342	19p	5455600	5456439	840	8	0.625	Promoter(<=1kb)	183	ZNRF4	148066
485342	19p	8948231	8952171	3941	12	0.5	Exon(exon3of84)	29171	MUC16	94025
485342	19p	8959403	8961248	1846	6	0.667	Exon(exon3of84)	20094	MUC16	94025
485342	19p	12430718	12432437	1720	8	0.375	3'UTR	8584	ZNF443	10224
485342	19p	15087213	15088040	828	10	0.3	Promoter(<=1kb)	233	OR1I1	126370
485342	19p	18264753	18267409	2657	8	0.5	5'UTR	7002	IQCN	80726
485342	19p	21971930	21974500	2571	9	0.667	Exon(exon4of4)	14408	ZNF208	7757
485342	19p	23743906	23745300	1395	6	0.333	Exon(exon4of4)	13537	ZNF681	148213
485342	19q	36996730	36997597	868	10	0.6	Exon(exon10of10)	5677	ZNF568	374900
485342	19q	43913423	43914878	1456	8	0.5	Exon(exon10of10)	4861	ZNF45	7596
485342	19q	44106512	44108078	1567	8	0.125	Exon(exon6of6)	-4103	ZNF225	7768
485342	19q	48873325	48875925	2601	11	0.545	Promoter(<=1kb)	904	PPP1R15A	23645
485342	19q	52437918	52439242	1325	7	0.429	Exon(exon4of4)	6504	ZNF534	147658
485342	19q	55771923	55773169	1247	6	0.5	Promoter(2-3kb)	2782	RFPL4AL1	729974
485342	19q	55911888	55913077	1190	7	0.714	Exon(exon5of12)	19234	NLRP13	126204
485342	19q	58368293	58368875	583	7	0.429	Exon(exon3of3)	-5445	ZNF497	162968
485342	20p	20052354	20052736	383	6	0	Promoter(<=1kb)	0	CFAP61	26074
485342	20q	63561666	63565531	3866	13	0.615	Promoter(<=1kb)	-61	HELZ2	85441
485342	21q	26843740	26844859	1120	6	0.667	Promoter(<=1kb)	0	ADAMTS1	9510
485342	21q	44600627	44601692	1066	9	0.778	Promoter(<=1kb)	30	KRTAP10-7	386675
485342	21q	44637500	44638041	542	7	0.429	Promoter(<=1kb)	144	KRTAP10-10	353333
485342	22q	22352950	22353380	431	16	0.5	Exon(exon1of2)	30478	BMS1P20	96610
485342	22q	22646443	22647152	710	7	0.286	Promoter(<=1kb)	0	GGTLC2	91227
485342	23p	3320126	3323750	3625	9	0.556	Exon(exon5of7)	22902	MXRA5	25878
485342	23p	8170039	8170141	103	6	0.5	Promoter(1-2kb)	1126	VCX2	51480
485342	23p	35802148	35803010	863	7	0.571	5'UTR	3357	MAGEB16	139604
486334	1p	12795518	12795749	232	6	0.5	Promoter(<=1kb)	827	PRAMEF1	65121
486334	1p	12847526	12848032	507	11	0.455	Promoter(<=1kb)	693	HNRNPCL1	343069
486334	1p	12859036	12860079	1044	6	0.333	Promoter(1-2kb)	1950	PRAMEF2	65122
486334	1p	12893187	12893472	286	10	0.4	Exon(exon4of4)	4798	PRAMEF10	343071
486334	1p	13370686	13371119	434	8	0.375	Promoter(<=1kb)	781	PRAMEF19	645414
486334	1p	16058491	16060000	1510	13	0.846	Exon(exon5of7)	6168	CLCNKB	1188
486334	1p	18481403	18482217	815	8	0.75	Promoter(<=1kb)	421	KLHDC7A	127707
486334	1p	23874604	23875430	827	8	0.5	Exon(exon2of2)	-6310	FUCA1	2517
486334	1p	40067594	40067675	82	6	0	Promoter(<=1kb)	324	CAP1	10487
486334	1p	89186388	89186419	32	9	0.556	Promoter(<=1kb)	107	GBP4	115361
486334	1q	145872200	145873487	1288	6	0.667	Exon(exon8of12)	-12364	PIAS3	10401
486334	1q	152213286	152213347	62	8	0.5	Exon(exon3of3)	10846	HRNR	388697
486334	1q	152219233	152221375	2143	15	0.733	Promoter(2-3kb)	2818	HRNR	388697
486334	1q	152910134	152911235	1102	8	0.5	Promoter(1-2kb)	1588	IVL	3713
486334	1q	228315976	228318038	2063	8	0.625	Exon(exon50of81)	6492	OBSCN	84033
486334	1q	247841312	247841582	271	6	0.833	Promoter(<=1kb)	314	OR11L1	391189
486334	1q	247949325	247949738	414	10	0.3	Promoter(<=1kb)	467	OR2L8	391190
486334	1q	248273309	248273670	362	7	0.571	Promoter(<=1kb)	166	OR2T33	391195
486334	1q	248294677	248295458	782	7	0.571	Promoter(<=1kb)	142	OR2T12	127064
486334	2p	48580657	48582454	1798	7	0.571	Promoter(<=1kb)	0	STON1	11037
486334	2q	102351547	102351902	356	7	0.429	Exon(exon11of11)	-4027	IL18R1	8809
486334	2q	132783534	132785001	1468	7	0.429	Promoter(1-2kb)	-1511	NCKAP5	344148
486334	2q	184936178	184937636	1459	6	0.333	Exon(exon4of4)	69813	ZNF804A	91752
486334	2q	185789865	185794632	4768	11	0.818	Promoter(<=1kb)	0	FSIP2	401024
486334	2q	185805377	185808170	2794	6	0.333	Promoter(<=1kb)	0	FSIP2	401024
486334	2q	217847567	217848213	647	6	0.833	Exon(exon19of33)	-5407	TNS1	7145
486334	2q	237762685	237764060	1376	10	0.5	Exon(exon8of8)	-4137	LRRFIP1	9208
486334	2q	238130271	238131546	1276	7	0.286	Promoter(1-2kb)	1323	ESPNL	339768
486334	2q	240041845	240042811	967	8	0.375	Downstream(2-3kb)	3261	OR6B3	150681
486334	3p	75737230	75739007	1778	9	0.556	Promoter(<=1kb)	0	MIR4273	100422955
486334	4p	5988383	5989749	1367	7	0.571	Promoter(<=1kb)	0	C4orf50	389197
486334	4p	6300792	6302360	1569	6	0.833	Exon(exon8of8)	6021	WFS1	7466
486334	4p	8227004	8228508	1505	8	0.125	Promoter(<=1kb)	-24	SH3TC1	54436
486334	4p	38796894	38798702	1809	6	0.5	Exon(exon4of4)	5674	TLR1	7096
486334	4q	87811379	87811766	388	6	0.333	Exon(exon7of7)	-9645	MEPE	56955
486334	4q	121036404	121037542	1139	6	0.333	Promoter(1-2kb)	1442	NDNF	79625
486334	4q	185458217	185460011	1795	9	0.556	Promoter(<=1kb)	0	CCDC110	256309
486334	4q	186617176	186621601	4426	11	0.364	Exon(exon10of27)	-7106	FAT1	2195
486334	5q	79728956	79730716	1761	9	0.222	Exon(exon2of13)	-7426	CMYA5	202333
486334	5q	79731782	79734523	2742	13	0.308	Exon(exon2of13)	-3619	CMYA5	202333
486334	5q	83537326	83539905	2580	6	0.333	Promoter(1-2kb)	1712	VCAN	1462
486334	5q	140848579	140850786	2208	6	0.5	Promoter(<=1kb)	807	PCDHA9	9752
486334	5q	141955356	141957660	2305	6	0.667	Promoter(<=1kb)	-668	RNF14	9604
486334	5q	151565922	151568455	2534	10	0.8	Promoter(<=1kb)	489	FAT2	2196
486334	6p	26370344	26370479	136	6	0.5	Promoter(<=1kb)	0	BTN3A2	11118
486334	6p	46858771	46859389	619	6	0.5	Exon(exon17of21)	3915	ADGRF5	221395
486334	6q	106511549	106512572	1024	6	0.667	Promoter(<=1kb)	0	CRYBG1	202
486334	6q	149888581	149890867	2287	7	0.714	Promoter(<=1kb)	0	RAET1E-AS1	100652739

486334	6q	159231899	159234370	2472	10	0.5	Exon(exon11of23)	13602	FNDC1	84624
486334	7p	6330446	6330944	499	6	1	Exon(exon2of2)	7749	FAM220A	84792
486334	7p	53035678	53036385	708	7	1	Promoter(<=1kb)	45	POM121L12	285877
486334	7q	100958977	100960873	1897	52	0.442	Promoter(1-2kb)	1012	MUC3A	4584
486334	7q	100991195	100995785	4591	15	0.6	Exon(exon5of15)	-17269	MUC12	10071
486334	7q	149818015	149819792	1778	6	0.667	Promoter(2-3kb)	-2352	SSPO	23145
486334	8p	8376561	8377198	638	6	1	Exon(exon2of5)	4549	PRAG1	157285
486334	8p	10607245	10608432	1188	7	0.429	Exon(exon4of4)	46711	RP1L1	94137
486334	8p	10609614	10610662	1049	7	0.429	Exon(exon4of4)	44481	RP1L1	94137
486334	8p	11331234	11332082	849	6	0.667	Promoter(<=1kb)	346	SLC35G5	83650
486334	8p	13021128	13022030	903	8	0.125	Exon(exon5of5)	9115	TRMT9B	57604
486334	9p	116800	117713	914	7	0.714	Promoter(<=1kb)	491	FOXD4	2298
486334	9p	34723747	34726473	2727	6	0.833	Exon(exon4of4)	3015	FAM205A	259308
486334	9q	76703451	76704082	632	6	0.333	Promoter(<=1kb)	-13	PRUNE2	158471
486334	9q	76705724	76707804	2081	6	0.5	Promoter(<=1kb)	666	PCA3	50652
486334	9q	87886533	87888536	2004	7	0.571	Exon(exon4of4)	3656	SPATA31E1	286234
486334	9q	104504315	104505071	757	6	0.5	Promoter(<=1kb)	52	OR13F1	138805
486334	9q	104598545	104599318	774	6	0.333	Promoter(<=1kb)	95	OR13C5	138799
486334	9q	122553263	122554071	809	8	0.5	Promoter(<=1kb)	93	OR1N2	138882
486334	9q	122628595	122629130	536	6	0.333	Promoter(<=1kb)	443	OR1B1	347169
486334	9q	122749914	122750547	634	6	0.833	Promoter(<=1kb)	174	OR1L6	392390
486334	9q	135484803	135487213	2411	8	0.25	Promoter(1-2kb)	1440	PPP1R26	9858
486334	10q	46549378	46550723	1346	27	0.667	Exon(exon3of3)	4807	GPRIN2	9721
486334	10q	49323169	49326817	3649	16	0.562	Exon(exon3of3)	23895	C10orf71	118461
486334	11p	244106	244197	92	8	0.5	Promoter(<=1kb)	-232	PSMD13	5719
486334	11p	1243512	1247378	3867	11	0.545	Promoter(2-3kb)	2298	MUC5B-AS1	112577518
486334	11p	5046754	5047432	679	7	0.571	Promoter(<=1kb)	228	OR52J3	119679
486334	11p	5177978	5178478	501	6	0.167	Promoter(<=1kb)	186	OR52Z1	283110
486334	11p	5389704	5390350	647	7	0.429	Promoter(<=1kb)	327	OR51M1	390059
486334	11p	5422212	5423123	912	11	0.636	Promoter(<=1kb)	101	OR51Q1	390061
486334	11p	5515185	5516015	831	6	0.333	Promoter(<=1kb)	684	UBQLNL	143630
486334	11p	5581045	5581738	694	8	0.375	Promoter(<=1kb)	168	OR52B6	340980
486334	11p	5884818	5885061	244	6	0.333	Promoter(<=1kb)	547	OR52E4	390081
486334	11p	11351961	11352736	776	7	0.143	Promoter(<=1kb)	514	CSNK2A3	283106
486334	11p	12293639	12294538	900	8	0.625	Exon(exon29of35)	6739	MICALCL	84953
486334	11p	18173280	18173901	622	6	0.333	Promoter(<=1kb)	443	MRGPRX4	117196
486334	11p	34916266	34916763	498	6	0.667	Promoter(<=1kb)	0	APIP	51074
486334	11q	58214757	58215722	966	8	0.25	Promoter(<=1kb)	12	OR1S1	219959
486334	11q	64116513	64118232	1720	7	0.714	Exon(exon2of2)	8702	MACROD1	28992
486334	11q	82732630	82733184	555	6	0.833	Promoter(<=1kb)	680	FAM181B	220382
486334	11q	85724687	85725825	1139	6	0.5	Promoter(<=1kb)	0	SYTL2	54843
486334	11q	93697503	93700096	2594	11	0.636	Promoter(<=1kb)	0	CEP295	85459
486334	11q	123906790	123907324	535	6	0.667	Promoter(<=1kb)	644	OR8D4	338662
486334	11q	124038366	124038988	623	9	0.889	Promoter(<=1kb)	13	OR10G7	390265
486334	11q	124382526	124383285	760	8	0.625	Promoter(<=1kb)	58	OR8B2	26595
486334	12p	4626568	4628549	1982	11	0.455	Exon(exon5of6)	14051	DYRK4	8798
486334	12p	6453119	6453670	552	6	0.667	Promoter(<=1kb)	633	TAPBPL	55080
486334	12p	8222174	8223514	1341	6	0.667	Exon(exon5of6)	4073	FAM90A1	55138
486334	13q	25096870	25097182	313	8	0.5	Promoter(1-2kb)	1002	PABPC3	5042
486334	13q	102732474	102733933	1460	6	0.333	Exon(exon4of4)	25139	CCDC168	643677
486334	14q	20060048	20060884	837	8	0.625	Promoter(<=1kb)	3	OR4L1	122742
486334	14q	20640982	20641567	586	6	0.5	Promoter(<=1kb)	124	OR6S1	341799
486334	14q	22633879	22634450	572	9	0.333	Exon(exon2of2)	32212	ABHD4	63874
486334	14q	44504986	44506403	1418	7	0.714	Promoter(<=1kb)	880	FSCB	84075
486334	14q	94587512	94587839	328	6	0.5	Exon(exon2of2)	-4219	SERPINA3	12
486334	14q	104947943	104951938	3996	11	0.455	Exon(exon6of6)	3464	AHNAK2	113146
486334	15q	20534501	20535129	629	6	0.333	Exon(exon8of9)	6671	GOLGA6L6	727832
486334	15q	23439979	23442067	2089	10	0.6	5'UTR	5167	GOLGA6L2	283685
486334	15q	40621642	40623696	2055	6	0.333	Promoter(<=1kb)	0	KNL1	57082
486334	15q	100569472	100570097	626	6	0.833	Promoter(<=1kb)	534	LINS1	55180
486334	15q	101177475	101178745	1271	6	0.667	3'UTR	9125	CHSY1	22856
486334	16p	789084	790597	1514	6	0.167	Promoter(<=1kb)	0	CHTF18	63922
486334	16p	1486371	1488463	2093	8	0.75	Promoter(<=1kb)	4	PTX4	390667
486334	16q	88428539	88429600	1062	6	0.333	Exon(exon3of3)	-23680	ZFPM1	161882
486334	16q	88714632	88717113	2482	7	0.429	Promoter(<=1kb)	0	MIR4722	100616167
486334	16q	89100686	89101050	365	8	0.625	Promoter(<=1kb)	24	ACSF3	197322
486334	16q	89226863	89228419	1557	10	0.6	Promoter(2-3kb)	2229	ZNF778	197320
486334	17p	2299649	2300159	511	6	0.167	Exon(exon2of19)	-3224	SRR	63826
486334	17p	10638198	10641099	2902	7	0.286	Exon(exon19of41)	-8169	MYH3	4621
486334	17p	21300581	21300954	374	8	0.75	3'UTR	9112	MAP2K3	5606
486334	17q	53823368	53824891	1524	6	0.833	Promoter(<=1kb)	441	KIF2B	84643
486334	17q	73236508	73236991	484	6	0.333	Promoter(<=1kb)	0	FAM104A	84923
486334	17q	76293419	76294016	598	6	0.5	Promoter(2-3kb)	-2167	QRICH2	84074
486334	17q	81645135	81645607	473	8	0.375	Promoter(2-3kb)	2722	TSPAN10	83882
486334	18p	11609904	11610509	606	8	0.625	Promoter(<=1kb)	308	SLC35G4	646000
486334	18p	14542649	14543140	492	6	0.5	Promoter(<=1kb)	6	POTEC	388468
486334	18q	58535186	58537515	2330	9	0.333	Promoter(<=1kb)	0	ALPK2	115701

486334	19p	4511338	4513547	2210	8	0.25	Exon(exon3of6)	4157	PLIN4	729359
486334	19p	5455600	5456439	840	6	0.5	Promoter(<=1kb)	183	ZNRF4	148066
486334	19p	8937644	8939234	1591	6	0.667	Exon(exon5of84)	-41554	MUC16	94025
486334	19p	8946313	8952171	5859	20	0.6	Exon(exon3of84)	29171	MUC16	94025
486334	19p	8959116	8962299	3184	12	0.75	Exon(exon3of84)	19043	MUC16	94025
486334	19p	8963397	8966905	3509	7	0.857	Exon(exon3of84)	14437	MUC16	94025
486334	19p	12430157	12432437	2281	9	0.333	3'UTR	8584	ZNF443	10224
486334	19p	15794192	15794719	528	6	0.333	Promoter(<=1kb)	241	OR10H5	284433
486334	19p	17281820	17284246	2427	9	0.556	Promoter(<=1kb)	0	ANKLE1	126549
486334	19p	18264798	18267409	2612	12	0.583	5'UTR	7002	IQCN	80726
486334	19p	21971930	21974500	2571	7	0.714	Exon(exon4of4)	14408	ZNF208	7757
486334	19p	23743906	23745300	1395	6	0.333	Exon(exon4of4)	13537	ZNF681	148213
486334	19q	39886241	39886422	182	6	0.833	Promoter(<=1kb)	870	FCGBP	8857
486334	19q	43846955	43848536	1582	6	0.833	3'UTR	13450	ZNF283	284349
486334	19q	43913423	43914878	1456	8	0.5	Exon(exon10of10)	4861	ZNF45	7596
486334	19q	44106512	44108078	1567	7	0	Exon(exon6of6)	-4103	ZNF225	7768
486334	19q	52365769	52366744	976	6	1	Exon(exon6of6)	-3173	ZNF880	400713
486334	19q	52437918	52439242	1325	7	0.429	Exon(exon4of4)	6504	ZNF534	147658
486334	19q	55481625	55483456	1832	7	0.429	Promoter(1-2kb)	-1732	NAT14	57106
486334	19q	55517821	55518176	356	7	1	Exon(exon14of14)	18127	SBK2	646643
486334	19q	55911888	55913077	1190	6	0.667	Exon(exon5of12)	19234	NLRP13	126204
486334	19q	57444351	57445460	1110	6	0.667	Exon(exon3of3)	9004	ZNF749	388567
486334	19q	58368293	58368875	583	7	0.429	Exon(exon3of3)	-5445	ZNF497	162968
486334	20p	5922677	5923382	706	7	0.571	Exon(exon4of5)	7179	CHGB	1114
486334	20q	56458364	56458660	297	6	0.833	Exon(exon7of7)	-9925	RTF2	51507
486334	20q	62812284	62813345	1062	7	0.857	Promoter(2-3kb)	-2899	COL9A3	1299
486334	20q	63561666	63565531	3866	13	0.615	Promoter(<=1kb)	-61	HELZ2	85441
486334	21q	44600627	44601692	1066	10	0.7	Promoter(<=1kb)	30	KRTAP10-7	386675
486334	21q	44637476	44638143	668	9	0.444	Promoter(<=1kb)	120	KRTAP10-10	353333
486334	22q	22352950	22353298	349	13	0.538	Exon(exon1of2)	30478	BMS1P20	96610
486334	22q	36191154	36191906	753	6	0.667	3'UTR	9971	APOL4	80832
486334	23p	3320126	3323750	3625	9	0.556	Exon(exon5of7)	22902	MXRA5	25878
486334	23p	8170039	8170243	205	7	0.571	Promoter(1-2kb)	1024	VCX2	51480
486334	23p	35802148	35803010	863	7	0.571	5'UTR	3357	MAGEB16	139604
486334	23q	136874183	136874416	234	8	0.75	Promoter(<=1kb)	-201	RBMX	27316
486502	1p	11766028	11768307	2280	6	0.833	Promoter(<=1kb)	0	C1orf167	284498
486502	1p	11778784	11779941	1158	7	0.714	Promoter(<=1kb)	0	C1orf167-AS1	102724659
486502	1p	16058491	16060000	1510	8	0.875	Exon(exon5of7)	6168	CLCNKB	1188
486502	1p	18481403	18482217	815	7	0.714	Promoter(<=1kb)	421	KLHDC7A	127707
486502	1p	23874604	23875430	827	8	0.5	Exon(exon2of2)	-6310	FUCA1	2517
486502	1p	31814028	31815123	1096	6	0.5	Promoter(<=1kb)	0	SPOCD1	90853
486502	1p	40067594	40067675	82	6	0	Promoter(<=1kb)	324	CAP1	10487
486502	1p	89186388	89186419	32	9	0.556	Promoter(<=1kb)	107	GBP4	115361
486502	1q	152213274	152213320	47	8	0.5	Exon(exon3of3)	10873	HRNR	388697
486502	1q	152218469	152221375	2907	17	0.706	Promoter(2-3kb)	2818	HRNR	388697
486502	1q	152302796	152313891	11096	38	0.553	Promoter(<=1kb)	0	FLG-AS1	339400
486502	1q	156669844	156670886	1043	6	1	Exon(exon4of4)	6521	NES	10763
486502	1q	158765805	158766655	851	6	0.5	Promoter(<=1kb)	47	OR6N1	128372
486502	1q	183647749	183648558	810	7	0.429	Exon(exon2of2)	4758	APOBEC4	403314
486502	1q	214640144	214642954	2811	12	0.5	Exon(exon12of20)	-5013	CENPF	1063
486502	1q	214644872	214647181	2310	10	0.4	Promoter(<=1kb)	-786	CENPF	1063
486502	1q	232805117	232806800	1684	6	0.333	Promoter(<=1kb)	225	MAP10	54627
486502	1q	236553659	236555893	2235	6	0.167	Exon(exon39of45)	10363	LGALS8	3964
486502	1q	247841312	247841582	271	6	0.833	Promoter(<=1kb)	314	OR11L1	391189
486502	1q	247949325	247949738	414	10	0.3	Promoter(<=1kb)	467	OR2L8	391190
486502	1q	248294677	248295458	782	7	0.571	Promoter(<=1kb)	142	OR2T12	127064
486502	2p	48580657	48582454	1798	7	0.571	Promoter(<=1kb)	0	STON1	11037
486502	2q	130914528	130916712	2185	6	0.167	Promoter(<=1kb)	0	ARHGEF4	50649
486502	2q	132783061	132784972	1912	7	0.429	Promoter(1-2kb)	-1038	NCKAP5	344148
486502	2q	167246794	167248478	1685	8	0.625	Promoter(<=1kb)	-204	XIRP2	129446
486502	2q	184936178	184937636	1459	6	0.333	Exon(exon4of4)	69813	ZNF804A	91752
486502	2q	185789865	185794632	4768	11	0.727	Promoter(<=1kb)	0	FSIP2	401024
486502	2q	217847583	217848642	1060	7	0.857	Exon(exon19of33)	-5423	TNS1	7145
486502	2q	233840612	233842185	1574	6	0.667	Promoter(<=1kb)	0	HJURP	55355
486502	2q	238130416	238131546	1131	6	0.333	Promoter(1-2kb)	1468	ESPNL	339768
486502	3p	31989532	31990905	1374	7	0.429	Exon(exon2of2)	7761	ZNF860	344787
486502	3p	75737230	75739007	1778	9	0.556	Promoter(<=1kb)	0	MIR4273	100422955
486502	3q	158732176	158732509	334	6	0.5	Promoter(<=1kb)	0	RARRES1	5918
486502	3q	196947388	196948448	1061	7	0.714	3'UTR	3482	PIGZ	80235
486502	4p	5988383	5989749	1367	7	0.571	Promoter(<=1kb)	0	C4orf50	389197
486502	4p	6300792	6302360	1569	7	0.857	Exon(exon8of8)	6021	WFS1	7466
486502	4p	8227004	8228508	1505	8	0.25	Promoter(<=1kb)	-24	SH3TC1	54436
486502	4p	38796894	38798702	1809	6	0.5	Exon(exon4of4)	5674	TLR1	7096
486502	4q	185458217	185460011	1795	9	0.556	Promoter(<=1kb)	0	CCDC110	256309
486502	5q	79728956	79730716	1761	6	0.333	Exon(exon2of13)	-7426	CMYA5	202333
486502	5q	79731782	79734523	2742	13	0.308	Exon(exon2of13)	-3619	CMYA5	202333
486502	5q	83537326	83539905	2580	6	0.333	Promoter(1-2kb)	1712	VCAN	1462

486502	5q	141174000	141175025	1026	6	0.833	Promoter(1-2kb)	1356	PCDHB7	56129
486502	5q	141183999	141184688	690	6	1	Promoter(2-3kb)	-2473	PCDHB9	56127
486502	5q	141955356	141957660	2305	6	0.667	Promoter(<=1kb)	-668	RNF14	9604
486502	5q	148826877	148828070	1194	6	1	Promoter(1-2kb)	1632	ADRB2	154
486502	5q	151521550	151522069	520	6	0.667	Promoter(<=1kb)	79	MIR6499	102465246
486502	5q	151565922	151568158	2237	9	0.778	Promoter(<=1kb)	786	FAT2	2196
486502	6p	46858771	46859502	732	8	0.5	Exon(exon17of21)	3802	ADGRF5	221395
486502	6q	64591274	64591961	688	10	0.5	Exon(exon26of43)	121374	EYS	346007
486502	6q	106511549	106512572	1024	6	0.667	Promoter(<=1kb)	0	CRYBG1	202
486502	6q	149888581	149890867	2287	7	0.714	Promoter(<=1kb)	0	RAET1E-AS1	100652739
486502	6q	159231899	159234370	2472	10	0.5	Exon(exon11of23)	13602	FNDC1	84624
486502	6q	168307929	168308705	777	6	0.5	5'UTR	11005	DACT2	168002
486502	7p	45082725	45084866	2142	8	0.5	Promoter(1-2kb)	-1325	NACAD	23148
486502	7p	53035678	53036385	708	7	1	Promoter(<=1kb)	45	POM121L12	285877
486502	7q	100958721	100960873	2153	55	0.473	Promoter(<=1kb)	756	MUC3A	4584
486502	7q	100991195	100993127	1933	9	0.667	Exon(exon5of15)	-19927	MUC12	10071
486502	7q	100995575	100995785	211	6	0.833	Exon(exon5of15)	-17269	MUC12	10071
486502	7q	129126736	129127452	717	6	0.333	Exon(exon1of2)	-17255	TSPAN33	340348
486502	8p	10607375	10608261	887	7	0.429	Exon(exon4of4)	46882	RP1L1	94137
486502	8p	11331194	11332026	833	8	0.625	Promoter(<=1kb)	306	SLC35G5	83650
486502	8p	12137448	12138641	1194	6	0.833	Promoter(<=1kb)	436	USP17L2	377630
486502	8p	13021128	13022030	903	7	0.143	Exon(exon5of5)	9115	TRMT9B	57604
486502	8q	143916360	143919209	2850	8	0.25	Exon(exon32of32)	20381	PLEC	5339
486502	9q	76175237	76175296	60	10	0.8	Exon(exon14of14)	-13343	PCSK5	5125
486502	9q	76703555	76707804	4250	13	0.462	Promoter(<=1kb)	0	PCA3	50652
486502	9q	76709263	76710843	1581	8	0.5	Promoter(<=1kb)	0	PRUNE2	158471
486502	9q	87885490	87888819	3330	8	0.625	Promoter(2-3kb)	2613	SPATA31E1	286234
486502	9q	104504315	104505071	757	6	0.5	Promoter(<=1kb)	52	OR13F1	138805
486502	9q	122553263	122554071	809	7	0.429	Promoter(<=1kb)	93	OR1N2	138882
486502	9q	122628595	122629130	536	6	0.333	Promoter(<=1kb)	443	OR1B1	347169
486502	9q	122749914	122750547	634	6	0.833	Promoter(<=1kb)	174	OR1L6	392390
486502	9q	133255635	133256205	571	7	1	3'UTR	19009	ABO	28
486502	9q	135484803	135487213	2411	8	0.25	Promoter(1-2kb)	1440	PPP1R26	9858
486502	10q	46549378	46550723	1346	25	0.64	Exon(exon3of3)	4807	GPRIN2	9721
486502	10q	128103129	128108204	5076	15	0.667	Promoter(<=1kb)	-1	MKI67	4288
486502	11p	244106	244197	92	8	0.5	Promoter(<=1kb)	-232	PSMD13	5719
486502	11p	1246095	1247378	1284	9	0.333	Promoter(2-3kb)	2298	MUC5B-AS1	112577518
486502	11p	1250091	1251628	1538	6	0.833	Promoter(<=1kb)	-415	MUC5B-AS1	112577518
486502	11p	1835943	1837342	1400	6	0.667	Promoter(<=1kb)	0	SYT8	90019
486502	11p	4681935	4682546	612	6	0.833	Promoter(2-3kb)	2546	OR51E2	81285
486502	11p	5177978	5178478	501	6	0.167	Promoter(<=1kb)	186	OR52Z1	283110
486502	11p	5323542	5324256	715	6	0.5	Promoter(<=1kb)	41	OR51B2	79345
486502	11p	5389704	5390350	647	7	0.429	Promoter(<=1kb)	327	OR51M1	390059
486502	11p	5422212	5423123	912	11	0.636	Promoter(<=1kb)	101	OR51Q1	390061
486502	11p	5581045	5581738	694	8	0.375	Promoter(<=1kb)	168	OR52B6	340980
486502	11p	5841302	5841883	582	9	0.333	Promoter(<=1kb)	14	OR52E6	390078
486502	11p	11351961	11352736	776	8	0.25	Promoter(<=1kb)	514	CSNK2A3	283106
486502	11p	12293639	12294368	730	6	0.833	Exon(exon29of35)	6739	MICALCL	84953
486502	11q	58214757	58215722	966	8	0.25	Promoter(<=1kb)	12	OR1S1	219959
486502	11q	66560202	66562261	2060	6	0.333	Exon(exon14of21)	3873	CTSF	8722
486502	11q	82732630	82733184	555	6	0.833	Promoter(<=1kb)	680	FAM181B	220382
486502	11q	85724687	85725825	1139	6	0.5	Promoter(<=1kb)	0	SYTL2	54843
486502	11q	124015601	124016477	877	9	0.333	Promoter(<=1kb)	26	OR10G4	390264
486502	11q	124023038	124023849	812	9	0.444	Promoter(<=1kb)	25	OR10G9	219870
486502	12p	4626568	4627524	957	8	0.5	Exon(exon5of6)	14051	DYRK4	8798
486502	12p	6453119	6453670	552	6	0.667	Promoter(<=1kb)	633	TAPBPL	55080
486502	13q	25096713	25097182	470	11	0.455	Promoter(<=1kb)	845	PABPC3	5042
486502	13q	49668389	49668560	172	6	0.333	Exon(exon3of3)	22878	EBPL	84650
486502	13q	102732474	102733933	1460	6	0.333	Exon(exon4of4)	25139	CCDC168	643677
486502	14q	19827604	19827851	248	6	0.833	Exon(exon2of2)	5669	OR4N2	390429
486502	14q	19975713	19976448	736	9	0.444	Promoter(<=1kb)	269	OR4K15	81127
486502	14q	20060048	20060884	837	8	0.625	Promoter(<=1kb)	3	OR4L1	122742
486502	14q	20640982	20641567	586	6	0.5	Promoter(<=1kb)	124	OR6S1	341799
486502	14q	21634137	21634589	453	9	0.556	Promoter(<=1kb)	351	OR10G2	26534
486502	14q	22633879	22634450	572	9	0.333	Exon(exon2of2)	32212	ABHD4	63874
486502	14q	70457520	70458540	1021	12	0.333	Exon(exon2of2)	5346	ADAM21	8747
486502	14q	104175275	104177810	2536	9	0.333	Exon(exon12of15)	36235	KIF26A	26153
486502	15q	23440160	23442067	1908	10	0.6	5'UTR	5167	GOLGA6L2	283685
486502	15q	40621642	40623696	2055	6	0.333	Promoter(<=1kb)	0	KNL1	57082
486502	15q	85579423	85581800	2378	15	0.6	Promoter(1-2kb)	-1110	AKAP13	11214
486502	16p	1228744	1229731	988	10	0.7	Promoter(<=1kb)	431	TPSB2	64499
486502	16p	1486011	1488463	2453	9	0.778	Promoter(<=1kb)	4	PTX4	390667
486502	16q	88428539	88429246	708	6	0.5	Exon(exon3of3)	-24034	ZFPM1	161882
486502	16q	88430623	88436097	5475	13	0.615	Exon(exon3of3)	-17183	ZFPM1	161882
486502	16q	89226863	89228289	1427	8	0.625	Promoter(2-3kb)	2229	ZNF778	197320
486502	17p	21300581	21300978	398	11	0.727	3'UTR	9112	MAP2K3	5606
486502	17q	76293419	76294016	598	6	0.5	Promoter(2-3kb)	-2167	QRICH2	84074

486502	17q	81645135	81645417	283	6	0.333	Promoter(2-3kb)	2722	TSPAN10	83882
486502	18p	11609646	11610164	519	9	0.778	Promoter(<=1kb)	50	SLC35G4	646000
486502	18q	47033929	47035145	1217	7	0.857	Promoter(<=1kb)	476	ELOA2	51224
486502	18q	58535186	58538030	2845	20	0.55	Promoter(<=1kb)	0	ALPK2	115701
486502	19p	4510548	4513547	3000	20	0.45	Exon(exon3of6)	4157	PLIN4	729359
486502	19p	5455600	5456439	840	6	0.5	Promoter(<=1kb)	183	ZNRF4	148066
486502	19p	8937644	8939234	1591	6	0.667	Exon(exon5of84)	-41554	MUC16	94025
486502	19p	8946313	8952171	5859	20	0.6	Exon(exon3of84)	29171	MUC16	94025
486502	19p	8959116	8962299	3184	12	0.75	Exon(exon3of84)	19043	MUC16	94025
486502	19p	12430718	12432437	1720	8	0.375	3'UTR	8584	ZNF443	10224
486502	19p	15087213	15088040	828	9	0.333	Promoter(<=1kb)	233	OR1I1	126370
486502	19p	15794192	15794829	638	8	0.375	Promoter(<=1kb)	241	OR10H5	284433
486502	19p	17282085	17284246	2162	9	0.556	Promoter(<=1kb)	0	ANKLE1	126549
486502	19p	18264798	18267409	2612	12	0.583	5'UTR	7002	IQCN	80726
486502	19p	21971930	21974500	2571	8	0.75	Exon(exon4of4)	14408	ZNF208	7757
486502	19q	36996730	36997597	868	10	0.7	Exon(exon10of10)	5677	ZNF568	374900
486502	19q	37151928	37153149	1222	6	0.833	Exon(exon5of5)	19287	ZNF585A	199704
486502	19q	37885190	37888806	3617	8	0.625	Exon(exon6of6)	17788	WDR87	83889
486502	19q	43913423	43914878	1456	8	0.5	Exon(exon10of10)	4861	ZNF45	7596
486502	19q	43966037	43967171	1135	6	0.167	Promoter(<=1kb)	-691	ZNF155	7711
486502	19q	43996326	43997366	1041	6	0.333	Exon(exon5of5)	5419	LOC10192806:	101928063
486502	19q	44106512	44108078	1567	8	0.125	Exon(exon6of6)	-4103	ZNF225	7768
486502	19q	52384129	52384992	864	6	0.167	Exon(exon4of4)	12677	ZNF528-AS1	102724105
486502	19q	52437918	52439242	1325	7	0.429	Exon(exon4of4)	6504	ZNF534	147658
486502	19q	53164551	53166239	1689	6	0.333	Exon(exon4of4)	-5476	ZNF347	84671
486502	19q	55481625	55483456	1832	6	0.5	Promoter(1-2kb)	-1732	NAT14	57106
486502	19q	55912302	55913166	865	6	0.667	Exon(exon5of12)	19145	NLRP13	126204
486502	19q	57444351	57445460	1110	6	0.667	Exon(exon3of3)	9004	ZNF749	388567
486502	19q	58368293	58368875	583	7	0.429	Exon(exon3of3)	-5445	ZNF497	162968
486502	20p	5922421	5923394	974	6	0.5	Exon(exon4of5)	6923	CHGB	1114
486502	20q	63561666	63565531	3866	13	0.615	Promoter(<=1kb)	-61	HELZ2	85441
486502	21q	26843740	26844859	1120	6	0.667	Promoter(<=1kb)	0	ADAMTS1	9510
486502	21q	44550835	44551416	582	6	0.833	Promoter(<=1kb)	89	KRTAP10-2	386679
486502	21q	44558207	44558709	503	7	0.571	Promoter(<=1kb)	86	KRTAP10-3	386682
486502	21q	44600627	44601692	1066	10	0.7	Promoter(<=1kb)	30	KRTAP10-7	386675
486502	21q	44637474	44638041	568	6	0.333	Promoter(<=1kb)	118	KRTAP10-10	353333
486502	22q	22352950	22353380	431	16	0.5	Exon(exon1of2)	30478	BMS1P20	96610
486502	22q	36191154	36191906	753	6	0.667	3'UTR	9971	APOL4	80832
486502	23p	8170039	8170243	205	7	0.571	Promoter(1-2kb)	1024	VCX2	51480
486502	23p	35802148	35803010	863	7	0.571	5'UTR	3357	MAGEB16	139604
486541	1p	12795591	12795813	223	6	0.667	Promoter(<=1kb)	900	PRAMEF1	65121
486541	1p	12847526	12847995	470	10	0.4	Promoter(<=1kb)	730	HNRNPCL1	343069
486541	1p	12859036	12860079	1044	6	0.333	Promoter(1-2kb)	1950	PRAMEF2	65122
486541	1p	12893249	12893472	224	6	0.5	Exon(exon4of4)	4798	PRAMEF10	343071
486541	1p	13369166	13369564	399	6	0.833	Promoter(2-3kb)	2336	PRAMEF19	645414
486541	1p	13370686	13371119	434	9	0.444	Promoter(<=1kb)	781	PRAMEF19	645414
486541	1p	16058491	16060000	1510	10	0.9	Exon(exon5of7)	6168	CLCNKB	1188
486541	1p	18481403	18482217	815	7	0.714	Promoter(<=1kb)	421	KLHDC7A	127707
486541	1p	40067594	40067675	82	6	0	Promoter(<=1kb)	324	CAP1	10487
486541	1p	89186388	89186419	32	9	0.556	Promoter(<=1kb)	107	GBP4	115361
486541	1q	152213286	152213347	62	8	0.5	Exon(exon3of3)	10846	HRNR	388697
486541	1q	152219233	152221375	2143	15	0.733	Promoter(2-3kb)	2818	HRNR	388697
486541	1q	158765805	158766655	851	6	0.5	Promoter(<=1kb)	47	OR6N1	128372
486541	1q	169540901	169542882	1982	10	0.3	Exon(exon13of25)	-25156	F5	2153
486541	1q	197101312	197102072	761	7	0.429	Exon(exon18of28)	33072	ASPM	259266
486541	1q	228315976	228318026	2051	6	0.5	Exon(exon50of81)	6492	OBSCN	84033
486541	1q	247841312	247841582	271	6	0.833	Promoter(<=1kb)	314	OR11L1	391189
486541	1q	247949325	247949738	414	10	0.3	Promoter(<=1kb)	467	OR2L8	391190
486541	1q	248681658	248682198	541	7	0.571	Promoter(<=1kb)	130	OR14I1	401994
486541	2p	29002636	29003646	1011	6	0.333	Exon(exon5of20)	-10821	TOGARAM2	165186
486541	2p	29071763	29073000	1238	6	0.667	Promoter(1-2kb)	1523	PCARE	388939
486541	2p	48580657	48582454	1798	7	0.571	Promoter(<=1kb)	0	STON1	11037
486541	2q	130193975	130194376	402	7	0.571	Exon(exon4of5)	4063	TUBA3E	112714
486541	2q	178739433	178741811	2379	6	0.5	Exon(exon45of191)	26014	TTN	7273
486541	2q	184936178	184937636	1459	6	0.333	Exon(exon4of4)	69813	ZNF804A	91752
486541	2q	185789591	185794632	5042	11	0.727	Promoter(<=1kb)	0	FSIP2	401024
486541	2q	238130271	238131546	1276	7	0.286	Promoter(1-2kb)	1323	ESPNL	339768
486541	2q	240041845	240042811	967	7	0.286	Downstream(2-3kb)	3261	OR6B3	150681
486541	3p	31989532	31990905	1374	7	0.286	Exon(exon2of2)	7761	ZNF860	344787
486541	3p	75736880	75739243	2364	63	0.619	Promoter(<=1kb)	0	MIR4273	100422955
486541	3q	98264413	98265137	725	6	0.667	Promoter(<=1kb)	128	OR5H6	79295
486541	4p	5988383	5989749	1367	7	0.571	Promoter(<=1kb)	0	C4orf50	389197
486541	4p	6300792	6302360	1569	7	0.857	Exon(exon8of8)	6021	WFS1	7466
486541	4p	10443803	10446224	2422	6	0.333	Exon(exon3of3)	10952	ZNF518B	85460
486541	5p	795818	796237	420	8	0.5	3'UTR	4908	ZDHHC11	79844
486541	5q	54518000	54519730	1731	6	0.167	Promoter(<=1kb)	135	SNX18	112574
486541	5q	140807352	140807737	386	6	0.833	Promoter(<=1kb)	271	PCDHA4	56144

486541	5q	141174000	141175025	1026	6	0.833	Promoter(1-2kb)	1356	PCDHB7	56129
486541	5q	141187690	141189425	1736	6	0.5	Promoter(<=1kb)	529	PCDHB9	56127
486541	5q	151521550	151522069	520	6	0.667	Promoter(<=1kb)	79	MIR6499	102465246
486541	5q	151565922	151568158	2237	10	0.7	Promoter(<=1kb)	786	FAT2	2196
486541	6p	1312843	1313745	903	6	0.5	Promoter(<=1kb)	745	FOXQ1	94234
486541	6p	46858771	46859389	619	6	0.5	Exon(exon17of21)	3915	ADGRF5	221395
486541	6q	106511549	106512572	1024	6	0.667	Promoter(<=1kb)	0	CRYBG1	202
486541	6q	149888581	149890867	2287	7	0.714	Promoter(<=1kb)	0	RAET1E-AS1	100652739
486541	6q	159233455	159234370	916	10	0.5	Exon(exon11of23)	15158	FNDC1	84624
486541	7p	53035678	53036385	708	6	1	Promoter(<=1kb)	45	POM121L12	285877
486541	7q	100952225	100953156	932	6	0.833	Promoter(2-3kb)	2600	MUC3A	4584
486541	7q	100958977	100960873	1897	49	0.469	Promoter(1-2kb)	1012	MUC3A	4584
486541	7q	100991195	100993127	1933	8	0.625	Exon(exon5of15)	-19927	MUC12	10071
486541	7q	100995575	100995785	211	6	0.833	Exon(exon5of15)	-17269	MUC12	10071
486541	7q	101004258	101004836	579	7	0.857	Exon(exon5of15)	-8218	MUC12	10071
486541	7q	149818015	149819792	1778	6	0.667	Promoter(2-3kb)	-2352	SSPO	23145
486541	7q	149824094	149826695	2602	6	0.667	Promoter(<=1kb)	0	SSPO	23145
486541	8p	10609614	10612307	2694	7	1	Exon(exon4of4)	42836	RP1L1	94137
486541	8p	12132686	12133940	1255	6	0.833	Promoter(<=1kb)	498	USP17L7	392197
486541	8p	12137448	12138641	1194	6	0.833	Promoter(<=1kb)	436	USP17L2	377630
486541	8p	13021128	13022030	903	11	0.182	Exon(exon5of5)	9115	TRMT9B	57604
486541	8q	144414297	144416618	2322	7	0.571	Promoter(<=1kb)	0	SLC39A4	55630
486541	9p	39078723	39078846	124	6	0.667	Exon(exon22of24)	7302	CNTNAP3	79937
486541	9q	76703555	76706360	2806	8	0.5	Promoter(<=1kb)	0	PCA3	50652
486541	9q	122553278	122554071	794	6	0.333	Promoter(<=1kb)	108	OR1N2	138882
486541	9q	122628595	122629398	804	8	0.375	Promoter(<=1kb)	175	OR1B1	347169
486541	9q	122749914	122750547	634	6	0.833	Promoter(<=1kb)	174	OR1L6	392390
486541	9q	133255635	133256205	571	7	0.857	3'UTR	19009	ABO	28
486541	9q	135484803	135487213	2411	8	0.25	Promoter(1-2kb)	1440	PPP1R26	9858
486541	10q	46549378	46550723	1346	26	0.654	Exon(exon3of3)	4807	GPRIN2	9721
486541	10q	49323169	49325192	2024	8	0.625	Exon(exon3of3)	23895	C10orf71	118461
486541	10q	128102594	128105201	2608	12	0.583	Promoter(<=1kb)	0	MKI67	4288
486541	10q	128106210	128109280	3071	8	0.875	Exon(exon12of14)	-3082	MKI67	4288
486541	11p	244106	244197	92	8	0.5	Promoter(<=1kb)	-232	PSMD13	5719
486541	11p	1194354	1196902	2549	7	0.571	Exon(exon34of49)	-26164	MUC5B	727897
486541	11p	1246095	1248605	2511	12	0.5	Promoter(1-2kb)	1071	MUC5B-AS1	112577518
486541	11p	5177978	5178478	501	6	0.167	Promoter(<=1kb)	186	OR52Z1	283110
486541	11p	5323451	5324256	806	6	0.5	Promoter(<=1kb)	41	OR51B2	79345
486541	11p	5389704	5390350	647	7	0.429	Promoter(<=1kb)	327	OR51M1	390059
486541	11p	5422212	5423123	912	10	0.7	Promoter(<=1kb)	101	OR51Q1	390061
486541	11p	5515185	5515931	747	6	0.333	Promoter(<=1kb)	768	UBQLNL	143630
486541	11p	5581045	5581738	694	8	0.375	Promoter(<=1kb)	168	OR52B6	340980
486541	11p	5841302	5841883	582	9	0.333	Promoter(<=1kb)	14	OR52E6	390078
486541	11p	5884818	5885061	244	7	0.429	Promoter(<=1kb)	547	OR52E4	390081
486541	11p	11351961	11352736	776	8	0.25	Promoter(<=1kb)	514	CSNK2A3	283106
486541	11p	12293639	12294842	1204	7	0.714	Exon(exon29of35)	6739	MICALCL	84953
486541	11p	43942293	43943348	1056	8	0.75	Promoter(<=1kb)	0	C11orf96	387763
486541	11q	54603136	54603820	685	6	0.667	Promoter(<=1kb)	178	OR4C46	119749
486541	11q	58214757	58215722	966	8	0.25	Promoter(<=1kb)	12	OR1S1	219959
486541	11q	64315797	64315856	60	8	0.75	Promoter(1-2kb)	1485	TRMT112	51504
486541	11q	85724687	85725825	1139	6	0.5	Promoter(<=1kb)	0	SYTL2	54843
486541	11q	123906595	123907324	730	7	0.429	Promoter(<=1kb)	449	OR8D4	338662
486541	11q	124015600	124016477	878	7	0.143	Promoter(<=1kb)	25	OR10G4	390264
486541	11q	124382526	124383285	760	8	0.625	Promoter(<=1kb)	58	OR8B2	26595
486541	12p	4626571	4628549	1979	8	0.5	Exon(exon5of6)	14054	DYRK4	8798
486541	12q	48525773	48526223	451	6	0.333	Promoter(<=1kb)	141	OR8S1	341568
486541	13q	24434450	24435347	898	8	0.625	Exon(exon31of34)	19787	PARP4	143
486541	13q	25096675	25097182	508	6	0.333	Promoter(<=1kb)	807	PABPC3	5042
486541	13q	102732474	102733933	1460	7	0.429	Exon(exon4of4)	25139	CCDC168	643677
486541	14q	20060048	20060884	837	8	0.625	Promoter(<=1kb)	3	OR4L1	122742
486541	14q	21634137	21634589	453	9	0.556	Promoter(<=1kb)	351	OR10G2	26534
486541	14q	22633879	22634450	572	8	0.375	Exon(exon2of2)	32212	ABHD4	63874
486541	14q	70457532	70458540	1009	11	0.273	Exon(exon2of2)	5358	ADAM21	8747
486541	14q	104175275	104177810	2536	6	0.333	Exon(exon12of15)	36235	KIF26A	26153
486541	14q	104938854	104942618	3765	11	0.182	5'UTR	6694	PLD4	122618
486541	14q	104943622	104945444	1823	7	0.571	Exon(exon6of6)	9958	AHNAK2	113146
486541	14q	104947901	104953878	5978	37	0.595	Promoter(1-2kb)	1524	AHNAK2	113146
486541	15q	20534480	20535129	650	8	0.375	Exon(exon8of9)	6671	GOLGA6L6	727832
486541	15q	23439979	23442067	2089	12	0.583	5'UTR	5167	GOLGA6L2	283685
486541	15q	40621642	40623696	2055	6	0.333	Promoter(<=1kb)	0	KNL1	57082
486541	15q	78765965	78766581	617	7	0.429	Promoter(<=1kb)	-852	ADAMTS7	11173
486541	15q	85579207	85581800	2594	15	0.6	Promoter(1-2kb)	-1110	AKAP13	11214
486541	15q	99129423	99132517	3095	6	0.333	Exon(exon4of5)	7225	TTC23	64927
486541	16p	669592	672548	2957	6	0.5	Promoter(<=1kb)	0	RHOT2	89941
486541	16p	789084	790597	1514	6	0.167	Promoter(<=1kb)	0	CHTF18	63922
486541	16p	1228744	1229731	988	10	0.7	Promoter(<=1kb)	431	TPSB2	64499
486541	16q	88428539	88429600	1062	6	0.333	Exon(exon3of3)	-23680	ZFPM1	161882

486541	16q	89100686	89101050	365	8	0.625	Promoter(<=1kb)	24	ACSF3	197322
486541	16q	89226863	89228390	1528	9	0.556	Promoter(2-3kb)	2229	ZNF778	197320
486541	17p	10638198	10641099	2902	7	0.286	Exon(exon19of41)	-8169	MYH3	4621
486541	17p	21300581	21300978	398	10	0.8	3'UTR	9112	MAP2K3	5606
486541	17q	53823368	53824891	1524	6	0.833	Promoter(<=1kb)	441	KIF2B	84643
486541	17q	76293419	76294016	598	6	0.5	Promoter(2-3kb)	-2167	QRICH2	84074
486541	17q	81645135	81645607	473	7	0.286	Promoter(2-3kb)	2722	TSPAN10	83882
486541	18p	11609904	11610444	541	7	0.714	Promoter(<=1kb)	308	SLC35G4	646000
486541	18p	14542649	14543140	492	6	0.5	Promoter(<=1kb)	6	POTEC	388468
486541	18q	58535186	58538030	2845	19	0.526	Promoter(<=1kb)	0	ALPK2	115701
486541	19p	4510548	4513547	3000	18	0.5	Exon(exon3of6)	4157	PLIN4	729359
486541	19p	5455600	5456439	840	7	0.571	Promoter(<=1kb)	183	ZNRF4	148066
486541	19p	8946313	8951868	5556	15	0.6	Exon(exon3of84)	29474	MUC16	94025
486541	19p	8959116	8962299	3184	10	0.7	Exon(exon3of84)	19043	MUC16	94025
486541	19p	8971838	8978096	6259	15	0.533	Exon(exon1of84)	3246	MUC16	94025
486541	19p	12430718	12432437	1720	8	0.375	3'UTR	8584	ZNF443	10224
486541	19p	15087452	15087953	502	7	0.143	Promoter(<=1kb)	472	OR1I1	126370
486541	19p	15794192	15794829	638	6	0.5	Promoter(<=1kb)	241	OR10H5	284433
486541	19p	18264798	18267409	2612	12	0.583	5'UTR	7002	IQCN	80726
486541	19p	21971930	21974500	2571	8	0.75	Exon(exon4of4)	14408	ZNF208	7757
486541	19p	23743741	23745300	1560	7	0.286	Exon(exon4of4)	13537	ZNF681	148213
486541	19q	36996730	36997597	868	10	0.6	Exon(exon10of10)	5677	ZNF568	374900
486541	19q	39877222	39877880	659	6	0.5	Exon(exon20of28)	9412	FCGBP	8857
486541	19q	39886240	39886355	116	6	0.833	Promoter(<=1kb)	937	FCGBP	8857
486541	19q	43846955	43848536	1582	7	0.857	3'UTR	13450	ZNF283	284349
486541	19q	43913423	43914878	1456	7	0.429	Exon(exon10of10)	4861	ZNF45	7596
486541	19q	44106512	44108078	1567	8	0.125	Exon(exon6of6)	-4103	ZNF225	7768
486541	19q	48874062	48875925	1864	8	0.5	Promoter(1-2kb)	1641	PPP1R15A	23645
486541	19q	52384174	52384992	819	8	0	Exon(exon4of4)	12677	ZNF528-AS1	102724105
486541	19q	52437918	52439242	1325	7	0.429	Exon(exon4of4)	6504	ZNF534	147658
486541	19q	53109058	53109736	679	6	0	5'UTR	-5622	ZNF160	90338
486541	19q	55911888	55913077	1190	6	0.667	Exon(exon5of12)	19234	NLRP13	126204
486541	20p	5922380	5923382	1003	8	0.625	Exon(exon4of5)	6882	CHGB	1114
486541	20q	62812284	62813345	1062	7	0.857	Promoter(2-3kb)	-2899	COL9A3	1299
486541	20q	63561666	63565531	3866	13	0.692	Promoter(<=1kb)	-61	HELZ2	85441
486541	21q	44573869	44574611	743	6	0.5	Promoter(<=1kb)	-53	TSPEAR	54084
486541	22q	22352950	22353380	431	16	0.5	Exon(exon1of2)	30478	BMS1P20	96610
486541	22q	36191154	36191906	753	6	0.667	3'UTR	9971	APOL4	80832
486541	23p	8170039	8170141	103	6	0.5	Promoter(1-2kb)	1126	VCX2	51480
486541	23p	35802148	35803010	863	7	0.571	5'UTR	3357	MAGEB16	139604
486541	23q	136874183	136874416	234	8	0.75	Promoter(<=1kb)	-201	RBMX	27316
486541	23q	141906066	141906494	429	10	0.5	Promoter(1-2kb)	1264	MAGEC1	9947
486566	1p	12795499	12795749	251	6	0.333	Promoter(<=1kb)	808	PRAMEF1	65121
486566	1p	12847526	12847780	255	7	0.429	Promoter(<=1kb)	945	HNRNPCL1	343069
486566	1p	12859036	12860079	1044	6	0.333	Promoter(1-2kb)	1950	PRAMEF2	65122
486566	1p	13370686	13370957	272	8	0.5	Promoter(<=1kb)	943	PRAMEF19	645414
486566	1p	13778554	13781269	2716	10	0.5	Exon(exon8of9)	6828	PRDM2	7799
486566	1p	16058512	16060000	1489	7	0.714	Exon(exon5of7)	6189	CLCNKB	1188
486566	1p	18481403	18482217	815	7	0.714	Promoter(<=1kb)	421	KLHDC7A	127707
486566	1p	23874604	23875430	827	8	0.5	Exon(exon2of2)	-6310	FUCA1	2517
486566	1p	40067594	40067675	82	6	0	Promoter(<=1kb)	324	CAP1	10487
486566	1q	152218469	152221375	2907	17	0.706	Promoter(2-3kb)	2818	HRNR	388697
486566	1q	152302796	152313891	11096	40	0.6	Promoter(<=1kb)	0	FLG-AS1	339400
486566	1q	158765805	158766655	851	6	0.5	Promoter(<=1kb)	47	OR6N1	128372
486566	1q	201206099	201209342	3244	11	0.636	Promoter(1-2kb)	1017	IGFN1	91156
486566	1q	201210956	201212792	1837	6	0.5	Promoter(<=1kb)	0	IGFN1	91156
486566	1q	226735683	226737239	1557	8	0.875	Promoter(<=1kb)	219	ITPKB	3707
486566	1q	228315976	228318026	2051	7	0.571	Exon(exon50of81)	6492	OBSCN	84033
486566	1q	247841312	247841582	271	6	0.833	Promoter(<=1kb)	314	OR11L1	391189
486566	1q	247895950	247896410	461	6	0.5	Promoter(<=1kb)	363	OR2W3	343171
486566	1q	247949452	247949738	287	6	0	Promoter(<=1kb)	594	OR2L8	391190
486566	2p	48580657	48582454	1798	7	0.571	Promoter(<=1kb)	0	STON1	11037
486566	2q	97547464	97547725	262	6	0.333	3'UTR	-35855	ANKRD36B	57730
486566	2q	130914211	130916712	2502	6	0.167	Promoter(<=1kb)	0	ARHGEF4	50649
486566	2q	132783032	132785012	1981	9	0.556	Promoter(1-2kb)	-1009	NCKAP5	344148
486566	2q	178739433	178741811	2379	6	0.5	Exon(exon45of191)	26014	TTN	7273
486566	2q	184936178	184937636	1459	6	0.333	Exon(exon4of4)	69813	ZNF804A	91752
486566	2q	185789865	185794632	4768	10	0.8	Promoter(<=1kb)	0	FSIP2	401024
486566	2q	185805377	185808170	2794	6	0.333	Promoter(<=1kb)	0	FSIP2	401024
486566	2q	238130416	238131546	1131	6	0.333	Promoter(1-2kb)	1468	ESPNL	339768
486566	2q	240041845	240042154	310	6	0.167	Downstream(2-3kb)	3918	OR6B3	150681
486566	3p	31989532	31990905	1374	7	0.286	Exon(exon2of2)	7761	ZNF860	344787
486566	3p	75736880	75739243	2364	52	0.596	Promoter(<=1kb)	0	MIR4273	100422955
486566	3q	98169021	98169594	574	6	0.667	Exon(exon2of2)	19695	OR5H14	403273
486566	3q	98264413	98265098	686	7	0.571	Promoter(<=1kb)	128	OR5H6	79295
486566	3q	196947878	196948448	571	7	0.571	3'UTR	3482	PIGZ	80235
486566	4p	5988383	5989749	1367	7	0.571	Promoter(<=1kb)	0	C4orf50	389197

486566	4p	6300792	6302360	1569	6	0.833	Exon(exon8of8)	6021	WFS1	7466
486566	4p	8227004	8228508	1505	8	0.125	Promoter(<=1kb)	-24	SH3TC1	54436
486566	4p	10443803	10446224	2422	6	0.333	Exon(exon3of3)	10952	ZNF518B	85460
486566	4q	87811379	87811766	388	6	0.333	Exon(exon7of7)	-9645	MEPE	56955
486566	4q	121036404	121037542	1139	6	0.333	Promoter(1-2kb)	1442	NDNF	79625
486566	4q	186706638	186708616	1979	6	0.667	Exon(exon2of27)	15217	FAT1	2195
486566	5q	54518000	54519665	1666	6	0.333	Promoter(<=1kb)	135	SNX18	112574
486566	5q	79728956	79730716	1761	6	0.333	Exon(exon2of13)	-7426	CMYA5	202333
486566	5q	79731782	79734523	2742	13	0.308	Exon(exon2of13)	-3619	CMYA5	202333
486566	5q	140807352	140807737	386	6	0.833	Promoter(<=1kb)	271	PCDHA4	56144
486566	5q	141172977	141174608	1632	9	0.889	Promoter(<=1kb)	333	PCDHB7	56129
486566	5q	141178745	141180333	1589	8	0.75	Promoter(<=1kb)	955	PCDHB8	56128
486566	5q	141955356	141957660	2305	6	0.667	Promoter(<=1kb)	-668	RNF14	9604
486566	6p	46858771	46859502	732	8	0.5	Exon(exon17of21)	3802	ADGRF5	221395
486566	6q	64591274	64591961	688	10	0.5	Exon(exon26of43)	121374	EYS	346007
486566	6q	159231899	159234370	2472	12	0.583	Exon(exon11of23)	13602	FNDC1	84624
486566	7p	6330446	6330944	499	6	1	Exon(exon2of2)	7749	FAM220A	84792
486566	7p	12369637	12370736	1100	6	0.667	3'UTR	-13307	VWDE	221806
486566	7p	53035678	53036385	708	6	1	Promoter(<=1kb)	45	POM121L12	285877
486566	7q	100958977	100960873	1897	52	0.442	Promoter(1-2kb)	1012	MUC3A	4584
486566	7q	100995547	100995785	239	7	0.714	Exon(exon5of15)	-17269	MUC12	10071
486566	7q	101034361	101040583	6223	36	0.528	Exon(exon3of12)	-3128	MUC17	140453
486566	7q	149818015	149819792	1778	6	0.667	Promoter(2-3kb)	-2352	SSPO	23145
486566	7q	149824094	149826495	2402	6	0.667	Promoter(<=1kb)	0	SSPO	23145
486566	8p	13021128	13022030	903	7	0.143	Exon(exon5of5)	9115	TRMT9B	57604
486566	8q	123651655	123652634	980	7	0.571	Promoter(<=1kb)	316	KLHL38	340359
486566	8q	143916360	143919209	2850	7	0.143	Exon(exon32of32)	20381	PLEC	5339
486566	9p	116800	117696	897	7	0.857	Promoter(<=1kb)	508	FOXD4	2298
486566	9q	76175237	76175296	60	9	0.778	Exon(exon14of14)	-13343	PCSK5	5125
486566	9q	87886533	87888536	2004	8	0.625	Exon(exon4of4)	3656	SPATA31E1	286234
486566	9q	104504315	104505071	757	6	0.5	Promoter(<=1kb)	52	OR13F1	138805
486566	9q	104598545	104599361	817	12	0.583	Promoter(<=1kb)	52	OR13C5	138799
486566	9q	104694482	104695444	963	6	0.5	Promoter(<=1kb)	0	OR13D1	286365
486566	9q	122749914	122750547	634	6	0.833	Promoter(<=1kb)	174	OR1L6	392390
486566	9q	135547960	135548795	836	8	0.625	Promoter(1-2kb)	1805	OBP2A	29991
486566	10q	46549378	46550723	1346	25	0.64	Exon(exon3of3)	4807	GPRIN2	9721
486566	10q	89737450	89738561	1112	6	0	Exon(exon20of33)	13874	KIF20B	9585
486566	11p	244106	244197	92	8	0.5	Promoter(<=1kb)	-232	PSMD13	5719
486566	11p	1246095	1247378	1284	8	0.375	Promoter(2-3kb)	2298	MUC5B-AS1	112577518
486566	11p	5177978	5178478	501	6	0.167	Promoter(<=1kb)	186	OR52Z1	283110
486566	11p	5323542	5324256	715	6	0.5	Promoter(<=1kb)	41	OR51B2	79345
486566	11p	5389704	5390350	647	6	0.5	Promoter(<=1kb)	327	OR51M1	390059
486566	11p	5402638	5403322	685	9	0.444	Promoter(<=1kb)	41	OR51J1	79470
486566	11p	5515079	5515931	853	7	0.429	Promoter(<=1kb)	768	UBQLNL	143630
486566	11p	5544676	5545259	584	6	0.5	Promoter(<=1kb)	290	OR52H1	390067
486566	11p	5581208	5581738	531	7	0.429	Promoter(<=1kb)	331	OR52B6	340980
486566	11p	5841302	5841883	582	9	0.333	Promoter(<=1kb)	14	OR52E6	390078
486566	11p	5884818	5885061	244	7	0.429	Promoter(<=1kb)	547	OR52E4	390081
486566	11p	11351961	11352736	776	9	0.222	Promoter(<=1kb)	514	CSNK2A3	283106
486566	11p	12293639	12294368	730	6	0.833	Exon(exon29of35)	6739	MICALCL	84953
486566	11p	18173280	18173901	622	6	0.333	Promoter(<=1kb)	443	MRGPRX4	117196
486566	11q	58214757	58215722	966	8	0.25	Promoter(<=1kb)	12	OR1S1	219959
486566	11q	85724687	85725825	1139	6	0.5	Promoter(<=1kb)	0	SYTL2	54843
486566	11q	124015601	124016477	877	9	0.222	Promoter(<=1kb)	26	OR10G4	390264
486566	11q	124023038	124023849	812	9	0.444	Promoter(<=1kb)	25	OR10G9	219870
486566	11q	124038392	124038988	597	6	0.833	Promoter(<=1kb)	13	OR10G7	390265
486566	11q	124382526	124383285	760	8	0.625	Promoter(<=1kb)	58	OR8B2	26595
486566	12p	4626568	4628549	1982	11	0.545	Exon(exon5of6)	14051	DYRK4	8798
486566	12q	50352073	50353545	1473	6	0.5	Promoter(1-2kb)	-1188	FAM186A	121006
486566	13q	24434450	24435347	898	7	0.571	Exon(exon31of34)	19787	PARP4	143
486566	13q	25096675	25097231	557	12	0.583	Promoter(<=1kb)	807	PABPC3	5042
486566	13q	102732474	102733933	1460	6	0.333	Exon(exon4of4)	25139	CCDC168	643677
486566	14q	20060048	20060884	837	8	0.625	Promoter(<=1kb)	3	OR4L1	122742
486566	14q	20640982	20641567	586	6	0.5	Promoter(<=1kb)	124	OR6S1	341799
486566	14q	21634137	21634589	453	9	0.556	Promoter(<=1kb)	351	OR10G2	26534
486566	14q	70457520	70458540	1021	13	0.385	Exon(exon2of2)	5346	ADAM21	8747
486566	14q	94587512	94587938	427	7	0.429	Exon(exon2of2)	-4120	SERPINA3	12
486566	14q	104175275	104177810	2536	8	0.25	Exon(exon12of15)	36235	KIF26A	26153
486566	14q	104939262	104945923	6662	21	0.476	5'UTR	7102	PLD4	122618
486566	14q	104947943	104953878	5936	33	0.545	Promoter(1-2kb)	1524	AHNAK2	113146
486566	15q	20534480	20535129	650	7	0.286	Exon(exon8of9)	6671	GOLGA6L6	727832
486566	15q	23440148	23442067	1920	10	0.7	5'UTR	5167	GOLGA6L2	283685
486566	15q	85579423	85581800	2378	14	0.571	Promoter(1-2kb)	-1110	AKAP13	11214
486566	15q	88854874	88855594	721	6	0.833	Exon(exon12of18)	7631	ACAN	176
486566	15q	88857108	88859365	2258	6	0	Exon(exon12of18)	9865	ACAN	176
486566	15q	99129423	99132517	3095	7	0.286	Exon(exon4of5)	7225	TTC23	64927
486566	16p	669592	672548	2957	7	0.571	Promoter(<=1kb)	0	RHOT2	89941

486566	16p	27362551	27363079	529	6	0.167	3'UTR	7203	IL4R	3566
486566	16q	74391401	74392004	604	10	0.7	Exon(exon7of7)	13523	NPIPB15	440348
486566	16q	89100686	89101050	365	8	0.625	Promoter(<=1kb)	24	ACSF3	197322
486566	16q	89226863	89228419	1557	9	0.556	Promoter(2-3kb)	2229	ZNF778	197320
486566	17p	744946	746966	2021	6	1	3'UTR	5072	GEMIN4	50628
486566	17p	7846914	7848939	2026	6	0.833	Promoter(1-2kb)	1999	KDM6B	23135
486566	17p	10638198	10641099	2902	7	0.286	Exon(exon19of41)	-8169	MYH3	4621
486566	17p	21300581	21300954	374	8	0.75	3'UTR	9112	MAP2K3	5606
486566	17p	21415470	21416370	901	6	0.833	Exon(exon3of3)	10334	KCNJ12	3768
486566	17q	76293419	76294016	598	6	0.5	Promoter(2-3kb)	-2167	QRICH2	84074
486566	17q	81645135	81645595	461	7	0.429	Promoter(2-3kb)	2722	TSPAN10	83882
486566	18p	11609728	11610350	623	12	0.667	Promoter(<=1kb)	132	SLC35G4	646000
486566	18p	11644365	11644598	234	6	1	Exon(exon1of1)	10343	MIR7153	102465690
486566	18q	58535186	58538030	2845	19	0.526	Promoter(<=1kb)	0	ALPK2	115701
486566	19p	4510548	4513547	3000	19	0.526	Exon(exon3of6)	4157	PLIN4	729359
486566	19p	5455600	5456439	840	7	0.571	Promoter(<=1kb)	183	ZNRF4	148066
486566	19p	8946313	8951868	5556	15	0.6	Exon(exon3of84)	29474	MUC16	94025
486566	19p	8959116	8962299	3184	10	0.7	Exon(exon3of84)	19043	MUC16	94025
486566	19p	8972751	8978096	5346	13	0.462	Exon(exon1of84)	3246	MUC16	94025
486566	19p	15087213	15088040	828	9	0.333	Promoter(<=1kb)	233	OR1I1	126370
486566	19p	18264798	18267409	2612	12	0.583	5'UTR	7002	IQCN	80726
486566	19p	22756294	22759533	3240	11	0.545	3'UTR	10449	ZNF99	7652
486566	19q	39886243	39886422	180	6	0.833	Promoter(<=1kb)	870	FCGBP	8857
486566	19q	43913423	43914878	1456	9	0.556	Exon(exon10of10)	4861	ZNF45	7596
486566	19q	43966037	43967171	1135	6	0.167	Promoter(<=1kb)	-691	ZNF155	7711
486566	19q	44106512	44108078	1567	8	0.125	Exon(exon6of6)	-4103	ZNF225	7768
486566	19q	48873985	48875925	1941	9	0.556	Promoter(1-2kb)	1564	PPP1R15A	23645
486566	19q	52437918	52439242	1325	7	0.429	Exon(exon4of4)	6504	ZNF534	147658
486566	19q	55481625	55483456	1832	6	0.5	Promoter(1-2kb)	-1732	NAT14	57106
486566	19q	55911888	55913077	1190	6	0.667	Exon(exon5of12)	19234	NLRP13	126204
486566	19q	57639918	57641641	1724	7	0.429	3'UTR	6693	ZNF211	10520
486566	20p	5922421	5923394	974	6	0.5	Exon(exon4of5)	6923	CHGB	1114
486566	20q	63561666	63565531	3866	13	0.615	Promoter(<=1kb)	-61	HELZ2	85441
486566	21q	26843740	26844859	1120	6	0.667	Promoter(<=1kb)	0	ADAMTS1	9510
486566	21q	44637476	44638143	668	8	0.5	Promoter(<=1kb)	120	KRTAP10-10	353333
486566	22q	22352950	22353380	431	16	0.5	Exon(exon1of2)	30478	BMS1P20	96610
486566	22q	36191154	36191906	753	8	0.625	3'UTR	9971	APOL4	80832
486566	23p	8170039	8170141	103	6	0.5	Promoter(1-2kb)	1126	VCX2	51480
488333	1p	978953	980214	1262	7	0.571	Promoter(<=1kb)	815	PERM1	84808
488333	1p	12795695	12795957	263	6	0.333	Promoter(1-2kb)	1004	PRAMEF1	65121
488333	1p	12847526	12847780	255	7	0.429	Promoter(<=1kb)	945	HNRNPCL1	343069
488333	1p	12859036	12860079	1044	6	0.333	Promoter(1-2kb)	1950	PRAMEF2	65122
488333	1p	13369166	13369564	399	6	0.833	Promoter(2-3kb)	2336	PRAMEF19	645414
488333	1p	13370686	13371119	434	7	0.429	Promoter(<=1kb)	781	PRAMEF19	645414
488333	1p	16058512	16060000	1489	8	0.75	Exon(exon5of7)	6189	CLCNKB	1188
488333	1p	18481403	18482217	815	6	0.667	Promoter(<=1kb)	421	KLHDC7A	127707
488333	1p	23874604	23875430	827	8	0.5	Exon(exon2of2)	-6310	FUCA1	2517
488333	1p	40067594	40067675	82	6	0	Promoter(<=1kb)	324	CAP1	10487
488333	1p	89186388	89186419	32	9	0.556	Promoter(<=1kb)	107	GBP4	115361
488333	1q	152218469	152221375	2907	18	0.722	Promoter(2-3kb)	2818	HRNR	388697
488333	1q	152302796	152313891	11096	39	0.59	Promoter(<=1kb)	0	FLG-AS1	339400
488333	1q	158765805	158766655	851	6	0.5	Promoter(<=1kb)	47	OR6N1	128372
488333	1q	169542317	169542882	566	6	0.167	Exon(exon13of25)	-26572	F5	2153
488333	1q	201206099	201209738	3640	11	0.545	Promoter(1-2kb)	1017	IGFN1	91156
488333	1q	223393517	223394466	950	6	0.667	Promoter(<=1kb)	102	CCDC185	164127
488333	1q	240207685	240207806	122	6	0.833	Exon(exon5of18)	-27965	FMN2	56776
488333	1q	247841312	247841582	271	6	0.833	Promoter(<=1kb)	314	OR11L1	391189
488333	1q	247949325	247949738	414	10	0.3	Promoter(<=1kb)	467	OR2L8	391190
488333	1q	248273309	248273718	410	6	0.667	Promoter(<=1kb)	118	OR2T33	391195
488333	2p	48580657	48582454	1798	7	0.571	Promoter(<=1kb)	0	STON1	11037
488333	2q	97547464	97547725	262	6	0.333	3'UTR	-35855	ANKRD36B	57730
488333	2q	167246794	167248478	1685	8	0.625	Promoter(<=1kb)	-204	XIRP2	129446
488333	2q	178739433	178741811	2379	6	0.5	Exon(exon45of191)	26014	TTN	7273
488333	2q	185789865	185794632	4768	10	0.8	Promoter(<=1kb)	0	FSIP2	401024
488333	2q	185805377	185807185	1809	6	0.5	Promoter(<=1kb)	0	FSIP2	401024
488333	2q	217817751	217818645	895	6	0.333	Promoter(2-3kb)	-2383	TNS1	7145
488333	2q	240041845	240042154	310	6	0.167	Downstream(2-3kb)	3918	OR6B3	150681
488333	3p	31989532	31990905	1374	7	0.286	Exon(exon2of2)	7761	ZNF860	344787
488333	3p	52521941	52524117	2177	6	0.333	Promoter(<=1kb)	0	STAB1	23166
488333	3p	75737230	75739007	1778	9	0.556	Promoter(<=1kb)	0	MIR4273	100422955
488333	3q	98264413	98265098	686	7	0.571	Promoter(<=1kb)	128	OR5H6	79295
488333	3q	194341097	194342571	1475	7	0.714	Exon(exon2of2)	8747	CPN2	1370
488333	4p	5988383	5989749	1367	7	0.571	Promoter(<=1kb)	0	C4orf50	389197
488333	4p	6300792	6302360	1569	7	0.857	Exon(exon8of8)	6021	WFS1	7466
488333	4p	8227004	8228508	1505	9	0.222	Promoter(<=1kb)	-24	SH3TC1	54436
488333	4q	185458217	185460011	1795	8	0.625	Promoter(<=1kb)	0	CCDC110	256309
488333	4q	186617176	186621582	4407	10	0.4	Exon(exon10of27)	-7106	FAT1	2195

488333	5q	79728956	79730716	1761	8	0.25	Exon(exon2of13)	-7426	CMYA5	202333
488333	5q	79731782	79734523	2742	13	0.308	Exon(exon2of13)	-3619	CMYA5	202333
488333	5q	83537326	83540746	3421	7	0.286	Promoter(1-2kb)	-1202	VCAN	1462
488333	5q	140848579	140850786	2208	9	0.556	Promoter(<=1kb)	807	PCDHA9	9752
488333	5q	141100771	141102758	1988	11	0.273	Promoter(<=1kb)	298	PCDHB3	56132
488333	5q	141122595	141123949	1355	6	0.833	Promoter(<=1kb)	777	PCDHB4	56131
488333	5q	141174000	141175025	1026	7	0.714	Promoter(1-2kb)	1356	PCDHB7	56129
488333	5q	141178631	141180333	1703	6	0.833	Promoter(<=1kb)	841	PCDHB8	56128
488333	5q	141183999	141184688	690	7	0.857	Promoter(2-3kb)	-2473	PCDHB9	56127
488333	5q	151521550	151522069	520	6	0.667	Promoter(<=1kb)	79	MIR6499	102465246
488333	6p	46858771	46859502	732	8	0.5	Exon(exon17of21)	3802	ADGRF5	221395
488333	6q	149888581	149890867	2287	7	0.714	Promoter(<=1kb)	0	RAET1E-AS1	100652739
488333	6q	159233455	159234370	916	10	0.5	Exon(exon11of23)	15158	FNDC1	84624
488333	7q	64991278	64992758	1481	6	0.667	Promoter(<=1kb)	-242	ZNF117	51351
488333	7q	100958721	100960873	2153	58	0.448	Promoter(<=1kb)	756	MUC3A	4584
488333	7q	100993912	100995922	2011	11	0.636	Exon(exon5of15)	-17132	MUC12	10071
488333	7q	101034361	101040583	6223	31	0.516	Exon(exon3of12)	-3128	MUC17	140453
488333	7q	149818015	149819792	1778	6	0.667	Promoter(2-3kb)	-2352	SSPO	23145
488333	8p	10609614	10612307	2694	9	0.889	Exon(exon4of4)	42836	RP1L1	94137
488333	8p	11331234	11332082	849	6	0.667	Promoter(<=1kb)	346	SLC35G5	83650
488333	8p	13021128	13022030	903	8	0.125	Exon(exon5of5)	9115	TRMT9B	57604
488333	8q	123651655	123652634	980	8	0.625	Promoter(<=1kb)	316	KLHL38	340359
488333	9p	214679	215269	591	6	0.5	Promoter(<=1kb)	0	DOCK8	81704
488333	9p	21206764	21207074	311	6	0.5	Promoter(<=1kb)	69	IFNA10	3446
488333	9p	39078723	39078846	124	8	0.75	Exon(exon22of24)	7302	CNTNAP3	79937
488333	9q	76175237	76175296	60	10	0.8	Exon(exon14of14)	-13343	PCSK5	5125
488333	9q	104598641	104599361	721	8	0.625	Promoter(<=1kb)	52	OR13C5	138799
488333	9q	122553263	122554071	809	7	0.429	Promoter(<=1kb)	93	OR1N2	138882
488333	9q	122628595	122629398	804	7	0.429	Promoter(<=1kb)	175	OR1B1	347169
488333	9q	122749914	122750547	634	6	0.833	Promoter(<=1kb)	174	OR1L6	392390
488333	9q	124855684	124856809	1126	7	0.571	Promoter(2-3kb)	2208	WDR38	401551
488333	9q	133255635	133256205	571	8	1	3'UTR	19009	ABO	28
488333	9q	133402909	133404019	1111	6	0.667	Exon(exon14of18)	8122	STKLD1	169436
488333	9q	135484803	135487213	2411	9	0.333	Promoter(1-2kb)	1440	PPP1R26	9858
488333	10q	46549378	46550723	1346	26	0.654	Exon(exon3of3)	4807	GPRIN2	9721
488333	10q	49323504	49325572	2069	7	0.429	Exon(exon3of3)	24230	C10orf71	118461
488333	10q	122084988	122087840	2853	8	0.75	Exon(exon4of23)	-25190	TACC2	10579
488333	10q	128103129	128104830	1702	10	0.6	Promoter(<=1kb)	-1	MKI67	4288
488333	10q	128106210	128109280	3071	7	1	Exon(exon12of14)	-3082	MKI67	4288
488333	11p	244106	244197	92	8	0.5	Promoter(<=1kb)	-232	PSMD13	5719
488333	11p	5046754	5047432	679	7	0.571	Promoter(<=1kb)	228	OR52J3	119679
488333	11p	5177978	5178478	501	6	0.167	Promoter(<=1kb)	186	OR52Z1	283110
488333	11p	5323451	5324256	806	6	0.5	Promoter(<=1kb)	41	OR51B2	79345
488333	11p	5389704	5390350	647	7	0.429	Promoter(<=1kb)	327	OR51M1	390059
488333	11p	5402638	5403322	685	9	0.444	Promoter(<=1kb)	41	OR51J1	79470
488333	11p	5515185	5515931	747	6	0.333	Promoter(<=1kb)	768	UBQLNL	143630
488333	11p	5581045	5581738	694	8	0.375	Promoter(<=1kb)	168	OR52B6	340980
488333	11p	5841302	5841883	582	10	0.4	Promoter(<=1kb)	14	OR52E6	390078
488333	11p	11351961	11352736	776	9	0.222	Promoter(<=1kb)	514	CSNK2A3	283106
488333	11p	12293639	12294538	900	6	0.667	Exon(exon29of35)	6739	MICALCL	84953
488333	11p	18173280	18173901	622	6	0.333	Promoter(<=1kb)	443	MRGPRX4	117196
488333	11q	58214757	58215722	966	7	0.286	Promoter(<=1kb)	12	OR1S1	219959
488333	11q	58402523	58403265	743	8	0.5	Promoter(<=1kb)	144	OR5B3	441608
488333	11q	82732630	82733184	555	6	0.833	Promoter(<=1kb)	680	FAM181B	220382
488333	11q	85724687	85725825	1139	6	0.5	Promoter(<=1kb)	0	SYTL2	54843
488333	11q	123906595	123907324	730	9	0.444	Promoter(<=1kb)	449	OR8D4	338662
488333	11q	124015600	124016477	878	7	0.143	Promoter(<=1kb)	25	OR10G4	390264
488333	11q	124038366	124038988	623	9	0.889	Promoter(<=1kb)	13	OR10G7	390265
488333	11q	124382526	124383285	760	8	0.625	Promoter(<=1kb)	58	OR8B2	26595
488333	12p	4626568	4628549	1982	11	0.545	Exon(exon5of6)	14051	DYRK4	8798
488333	12q	52316096	52317765	1670	6	0.667	Exon(exon4of9)	3633	KRT83	3889
488333	12q	52571389	52572653	1265	6	0.5	Promoter(1-2kb)	1163	KRT74	121391
488333	13q	24434450	24435347	898	7	0.571	Exon(exon31of34)	19787	PARP4	143
488333	13q	25096713	25097182	470	12	0.417	Promoter(<=1kb)	845	PABPC3	5042
488333	13q	102732474	102733933	1460	6	0.333	Exon(exon4of4)	25139	CCDC168	643677
488333	14q	20060048	20060884	837	8	0.625	Promoter(<=1kb)	3	OR4L1	122742
488333	14q	20640982	20641567	586	7	0.429	Promoter(<=1kb)	124	OR6S1	341799
488333	14q	70457532	70458540	1009	11	0.273	Exon(exon2of2)	5358	ADAM21	8747
488333	14q	94587512	94587839	328	6	0.5	Exon(exon2of2)	-4219	SERPINA3	12
488333	14q	104947661	104951938	4278	12	0.583	Exon(exon6of6)	3464	AHNAK2	113146
488333	15q	20534501	20535129	629	7	0.429	Exon(exon8of9)	6671	GOLGA6L6	727832
488333	15q	23439979	23442067	2089	12	0.583	5'UTR	5167	GOLGA6L2	283685
488333	15q	85579423	85581800	2378	14	0.571	Promoter(1-2kb)	-1110	AKAP13	11214
488333	16p	1486371	1488463	2093	8	0.75	Promoter(<=1kb)	4	PTX4	390667
488333	16q	74391401	74392004	604	10	0.6	Exon(exon7of7)	13523	NPIPB15	440348
488333	16q	88428539	88429600	1062	6	0.333	Exon(exon3of3)	-23680	ZFPM1	161882
488333	16q	89226863	89228419	1557	11	0.636	Promoter(2-3kb)	2229	ZNF778	197320

488333	17p	2299649	2300159	511	6	0.167	Exon(exon2of19)	-3224	SRR	63826
488333	17p	10638198	10641099	2902	7	0.286	Exon(exon19of41)	-8169	MYH3	4621
488333	17p	21300581	21300978	398	12	0.75	3'UTR	9112	MAP2K3	5606
488333	17q	76293419	76294016	598	6	0.5	Promoter(2-3kb)	-2167	QRICH2	84074
488333	17q	81510690	81511591	902	6	0.667	Promoter(<=1kb)	257	ACTG1	71
488333	17q	81645135	81645417	283	6	0.333	Promoter(2-3kb)	2722	TSPAN10	83882
488333	18p	11609646	11610383	738	8	0.875	Promoter(<=1kb)	50	SLC35G4	646000
488333	18q	58535186	58537515	2330	9	0.333	Promoter(<=1kb)	0	ALPK2	115701
488333	19p	4510548	4513547	3000	17	0.471	Exon(exon3of6)	4157	PLIN4	729359
488333	19p	5455600	5456439	840	7	0.571	Promoter(<=1kb)	183	ZNRF4	148066
488333	19p	5866643	5867737	1095	6	0.667	Promoter(2-3kb)	2803	FUT5	2527
488333	19p	8948231	8950136	1906	6	0.667	Exon(exon3of84)	31206	MUC16	94025
488333	19p	8959518	8962066	2549	6	0.333	Exon(exon3of84)	19276	MUC16	94025
488333	19p	8964274	8967127	2854	19	0.632	Exon(exon3of84)	14215	MUC16	94025
488333	19p	12430157	12431840	1684	9	0.444	3'UTR	9181	ZNF443	10224
488333	19p	18264798	18267409	2612	12	0.583	5'UTR	7002	IQCN	80726
488333	19p	21971930	21974500	2571	11	0.727	Exon(exon4of4)	14408	ZNF208	7757
488333	19p	22756294	22759533	3240	11	0.545	3'UTR	10449	ZNF99	7652
488333	19q	36996730	36997597	868	10	0.7	Exon(exon10of10)	5677	ZNF568	374900
488333	19q	37151928	37153149	1222	6	0.833	Exon(exon5of5)	19287	ZNF585A	199704
488333	19q	43913423	43914878	1456	9	0.556	Exon(exon10of10)	4861	ZNF45	7596
488333	19q	43996326	43997366	1041	6	0.5	Exon(exon5of5)	5419	LOC10192806	101928063
488333	19q	52437918	52439242	1325	7	0.429	Exon(exon4of4)	6504	ZNF534	147658
488333	19q	55911888	55913077	1190	6	0.667	Exon(exon5of12)	19234	NLRP13	126204
488333	19q	58368293	58368875	583	7	0.429	Exon(exon3of3)	-5445	ZNF497	162968
488333	20p	5922421	5923394	974	7	0.571	Exon(exon4of5)	6923	CHGB	1114
488333	20q	63561666	63565531	3866	11	0.636	Promoter(<=1kb)	-61	HELZ2	85441
488333	21q	44637474	44638143	670	10	0.4	Promoter(<=1kb)	118	KRTAP10-10	353333
488333	22q	22352950	22353380	431	16	0.5	Exon(exon1of2)	30478	BMS1P20	96610
488333	22q	36191154	36191906	753	6	0.667	3'UTR	9971	APOL4	80832
488333	23p	3320126	3323750	3625	10	0.6	Exon(exon5of7)	22902	MXRA5	25878
488333	23p	8170039	8170141	103	6	0.5	Promoter(1-2kb)	1126	VCX2	51480
488333	23p	35802148	35803010	863	7	0.571	5'UTR	3357	MAGEB16	139604
489705	1p	12847526	12847995	470	11	0.455	Promoter(<=1kb)	730	HNRNPCL1	343069
489705	1p	12859036	12860079	1044	6	0.333	Promoter(1-2kb)	1950	PRAMEF2	65122
489705	1p	13370686	13371119	434	7	0.429	Promoter(<=1kb)	781	PRAMEF19	645414
489705	1p	16058491	16060000	1510	10	0.9	Exon(exon5of7)	6168	CLCNKB	1188
489705	1p	23874604	23875430	827	8	0.5	Exon(exon2of2)	-6310	FUCA1	2517
489705	1p	31814028	31815123	1096	6	0.5	Promoter(<=1kb)	0	SPOCD1	90853
489705	1p	40067594	40067675	82	6	0	Promoter(<=1kb)	324	CAP1	10487
489705	1p	89186388	89186419	32	9	0.556	Promoter(<=1kb)	107	GBP4	115361
489705	1q	152219211	152221375	2165	18	0.722	Promoter(2-3kb)	2818	HRNR	388697
489705	1q	152307253	152307694	442	6	0.667	Exon(exon3of3)	-4882	FLG-AS1	339400
489705	1q	152310318	152313891	3574	13	0.615	Promoter(<=1kb)	0	FLG-AS1	339400
489705	1q	158765805	158766655	851	6	0.5	Promoter(<=1kb)	47	OR6N1	128372
489705	1q	201206099	201209342	3244	9	0.667	Promoter(1-2kb)	1017	IGFN1	91156
489705	1q	226735683	226737239	1557	7	0.857	Promoter(<=1kb)	219	ITPKB	3707
489705	1q	228315976	228317998	2023	6	0.5	Exon(exon50of81)	6492	OBSCN	84033
489705	1q	247841312	247841582	271	6	0.833	Promoter(<=1kb)	314	OR11L1	391189
489705	1q	247949325	247949738	414	10	0.3	Promoter(<=1kb)	467	OR2L8	391190
489705	1q	248681658	248682198	541	7	0.571	Promoter(<=1kb)	130	OR14I1	401994
489705	2p	48580657	48582454	1798	7	0.571	Promoter(<=1kb)	0	STON1	11037
489705	2q	132783061	132785001	1941	9	0.444	Promoter(1-2kb)	-1038	NCKAP5	344148
489705	2q	185789865	185794632	4768	10	0.8	Promoter(<=1kb)	0	FSIP2	401024
489705	2q	217847567	217848213	647	6	0.833	Exon(exon19of33)	-5407	TNS1	7145
489705	2q	219271337	219271649	313	6	0.667	Exon(exon4of4)	6227	TUBA4A	7277
489705	2q	233681970	233682693	724	6	0.667	Promoter(<=1kb)	0	UGT1A7	54577
489705	2q	233713134	233713664	531	8	0.75	Promoter(<=1kb)	142	UGT1A5	54579
489705	2q	240041845	240042154	310	6	0.167	Downstream(2-3kb)	3918	OR6B3	150681
489705	3p	75737230	75739007	1778	9	0.556	Promoter(<=1kb)	0	MIR4273	100422955
489705	3q	98264413	98265098	686	6	0.667	Promoter(<=1kb)	128	OR5H6	79295
489705	4p	1394795	1395368	574	6	0.5	Exon(exon1of1)	9983	UVSSA	57654
489705	4p	6300792	6302360	1569	6	0.833	Exon(exon8of8)	6021	WFS1	7466
489705	4p	8227004	8228508	1505	8	0.125	Promoter(<=1kb)	-24	SH3TC1	54436
489705	4q	112431241	112432293	1053	7	0.429	3'UTR	4735	ALPK1	80216
489705	4q	154489498	154491312	1815	9	0.556	Promoter(<=1kb)	22	DCHS2	54798
489705	5q	79728956	79730716	1761	8	0.25	Exon(exon2of13)	-7426	CMYA5	202333
489705	5q	79731782	79734523	2742	13	0.308	Exon(exon2of13)	-3619	CMYA5	202333
489705	5q	140848579	140850786	2208	6	0.5	Promoter(<=1kb)	807	PCDHA9	9752
489705	5q	141183999	141184688	690	6	0.833	Promoter(2-3kb)	-2473	PCDHB9	56127
489705	5q	141955356	141957660	2305	6	0.667	Promoter(<=1kb)	-668	RNF14	9604
489705	5q	151565922	151568158	2237	9	0.778	Promoter(<=1kb)	786	FAT2	2196
489705	6p	42745312	42746041	730	6	0.667	Promoter(<=1kb)	62	TBCC	6903
489705	6p	46858771	46859389	619	6	0.5	Exon(exon17of21)	3915	ADGRF5	221395
489705	6q	149888581	149890867	2287	7	0.714	Promoter(<=1kb)	0	RAET1E-AS1	100652739
489705	6q	159231899	159234370	2472	10	0.5	Exon(exon11of23)	13602	FNDC1	84624
489705	7p	12369637	12370736	1100	6	0.667	3'UTR	-13307	VWDE	221806

489705	7q	64991321	64992758	1438	6	0.667	Promoter(<=1kb)	-285	ZNF117	51351
489705	7q	100958721	100960873	2153	59	0.458	Promoter(<=1kb)	756	MUC3A	4584
489705	7q	100991195	100992398	1204	7	0.571	Exon(exon5of15)	-20656	MUC12	10071
489705	7q	100995575	100995785	211	7	0.714	Exon(exon5of15)	-17269	MUC12	10071
489705	7q	101033476	101041369	7894	42	0.548	Promoter(2-3kb)	-2342	MUC17	140453
489705	7q	156949807	156950570	764	6	0.833	Promoter(<=1kb)	95	NOM1	64434
489705	8p	10607245	10608432	1188	8	0.5	Exon(exon4of4)	46711	RP1L1	94137
489705	8p	10609614	10610662	1049	7	0.429	Exon(exon4of4)	44481	RP1L1	94137
489705	8p	12132686	12133940	1255	6	0.667	Promoter(<=1kb)	498	USP17L7	392197
489705	8p	13021128	13022030	903	7	0.143	Exon(exon5of5)	9115	TRMT9B	57604
489705	8q	142664552	142665852	1301	6	0.833	Exon(exon2of2)	4118	JRK	8629
489705	9p	39078723	39078846	124	7	0.714	Exon(exon22of24)	7302	CNTNAP3	79937
489705	9q	76703555	76706360	2806	8	0.5	Promoter(<=1kb)	0	PCA3	50652
489705	9q	87884955	87887543	2589	6	0.667	Promoter(2-3kb)	2078	SPATA31E1	286234
489705	9q	104598545	104599361	817	12	0.583	Promoter(<=1kb)	52	OR13C5	138799
489705	9q	122628595	122629130	536	6	0.333	Promoter(<=1kb)	443	OR1B1	347169
489705	9q	122749914	122750547	634	6	0.833	Promoter(<=1kb)	174	OR1L6	392390
489705	9q	135547960	135548795	836	8	0.625	Promoter(1-2kb)	1805	OBP2A	29991
489705	10q	46549378	46550723	1346	26	0.654	Exon(exon3of3)	4807	GPRIN2	9721
489705	10q	49322575	49325572	2998	8	0.5	Exon(exon3of3)	23301	C10orf71	118461
489705	10q	122084988	122087840	2853	8	0.75	Exon(exon4of23)	-25190	TACC2	10579
489705	10q	128103129	128104830	1702	10	0.6	Promoter(<=1kb)	-1	MKI67	4288
489705	11p	244106	244197	92	8	0.5	Promoter(<=1kb)	-232	PSMD13	5719
489705	11p	1246095	1247378	1284	6	0.333	Promoter(2-3kb)	2298	MUC5B-AS1	112577518
489705	11p	1248397	1251628	3232	9	0.778	Promoter(<=1kb)	0	MUC5B-AS1	112577518
489705	11p	5046754	5047432	679	7	0.571	Promoter(<=1kb)	228	OR52J3	119679
489705	11p	5177978	5178478	501	6	0.167	Promoter(<=1kb)	186	OR52Z1	283110
489705	11p	5389704	5390350	647	6	0.5	Promoter(<=1kb)	327	OR51M1	390059
489705	11p	5422212	5423123	912	11	0.636	Promoter(<=1kb)	101	OR51Q1	390061
489705	11p	5440604	5441472	869	6	0.833	Promoter(<=1kb)	42	OR51I1	390063
489705	11p	5581045	5581738	694	8	0.375	Promoter(<=1kb)	168	OR52B6	340980
489705	11p	5841302	5841883	582	9	0.333	Promoter(<=1kb)	14	OR52E6	390078
489705	11p	5884818	5885061	244	7	0.429	Promoter(<=1kb)	547	OR52E4	390081
489705	11p	11351961	11352736	776	9	0.222	Promoter(<=1kb)	514	CSNK2A3	283106
489705	11p	12293639	12294368	730	7	0.857	Exon(exon29of35)	6739	MICALCL	84953
489705	11p	18173280	18173901	622	6	0.333	Promoter(<=1kb)	443	MRGPRX4	117196
489705	11p	34916266	34916763	498	6	0.667	Promoter(<=1kb)	0	APIP	51074
489705	11q	58214757	58215722	966	6	0.333	Promoter(<=1kb)	12	OR1S1	219959
489705	11q	58402523	58403265	743	8	0.5	Promoter(<=1kb)	144	OR5B3	441608
489705	11q	85724687	85725825	1139	6	0.5	Promoter(<=1kb)	0	SYTL2	54843
489705	11q	123906510	123907324	815	7	0.714	Promoter(<=1kb)	364	OR8D4	338662
489705	11q	124038366	124038988	623	7	1	Promoter(<=1kb)	13	OR10G7	390265
489705	12p	4626571	4628549	1979	9	0.556	Exon(exon5of6)	14054	DYRK4	8798
489705	13q	102732474	102733933	1460	6	0.333	Exon(exon4of4)	25139	CCDC168	643677
489705	14q	20060048	20060884	837	8	0.625	Promoter(<=1kb)	3	OR4L1	122742
489705	14q	20143999	20144604	606	8	0.5	Promoter(<=1kb)	263	OR4N5	390437
489705	14q	21634137	21634589	453	9	0.556	Promoter(<=1kb)	351	OR10G2	26534
489705	14q	44504986	44506403	1418	6	0.667	Promoter(<=1kb)	880	FSCB	84075
489705	14q	70457532	70458540	1009	9	0.333	Exon(exon2of2)	5358	ADAM21	8747
489705	14q	104947943	104951938	3996	15	0.333	Exon(exon6of6)	3464	AHNAK2	113146
489705	15q	23440160	23442067	1908	11	0.545	5'UTR	5167	GOLGA6L2	283685
489705	15q	73702465	73703760	1296	6	0.833	Promoter(<=1kb)	-149	CD276	80381
489705	15q	75206207	75207654	1448	6	0.5	Promoter(<=1kb)	158	C15orf39	56905
489705	15q	85579423	85581800	2378	14	0.571	Promoter(1-2kb)	-1110	AKAP13	11214
489705	15q	99129423	99132517	3095	6	0.333	Exon(exon4of5)	7225	TTC23	64927
489705	16p	669592	672548	2957	7	0.429	Promoter(<=1kb)	0	RHOT2	89941
489705	16p	1228744	1229622	879	6	0.833	Promoter(<=1kb)	540	TPSB2	64499
489705	16p	27362551	27363079	529	6	0.167	3'UTR	7203	IL4R	3566
489705	16q	88714632	88717113	2482	8	0.5	Promoter(<=1kb)	0	MIR4722	100616167
489705	16q	89100686	89101050	365	8	0.625	Promoter(<=1kb)	24	ACSF3	197322
489705	16q	89226863	89228289	1427	8	0.625	Promoter(2-3kb)	2229	ZNF778	197320
489705	17p	744917	746966	2050	8	0.875	3'UTR	5072	GEMIN4	50628
489705	17p	21300581	21300954	374	11	0.727	3'UTR	9112	MAP2K3	5606
489705	17p	21415470	21416370	901	8	0.75	Exon(exon3of3)	10334	KCNJ12	3768
489705	17q	53823723	53824891	1169	6	0.833	Promoter(<=1kb)	796	KIF2B	84643
489705	17q	76293419	76294016	598	6	0.5	Promoter(2-3kb)	-2167	QRICH2	84074
489705	17q	81645135	81645417	283	6	0.333	Promoter(2-3kb)	2722	TSPAN10	83882
489705	18p	11609728	11610491	764	10	0.8	Promoter(<=1kb)	132	SLC35G4	646000
489705	18q	58535186	58538030	2845	19	0.579	Promoter(<=1kb)	0	ALPK2	115701
489705	19p	4511269	4511943	675	6	0.5	Exon(exon3of6)	5761	PLIN4	729359
489705	19p	5455600	5456439	840	8	0.625	Promoter(<=1kb)	183	ZNRF4	148066
489705	19p	8333830	8334965	1136	6	0.667	Promoter(<=1kb)	0	KANK3	256949
489705	19p	8948231	8952171	3941	13	0.538	Exon(exon3of84)	29171	MUC16	94025
489705	19p	8959403	8961248	1846	6	0.667	Exon(exon3of84)	20094	MUC16	94025
489705	19p	8971838	8973623	1786	7	0.714	Exon(exon1of84)	7719	MUC16	94025
489705	19p	12430157	12431840	1684	9	0.444	3'UTR	9181	ZNF443	10224
489705	19p	17281820	17284246	2427	9	0.556	Promoter(<=1kb)	0	ANKLE1	126549

489705	19p	18264798	18267409	2612	12	0.583	5'UTR	7002	IQCN	80726
489705	19q	39877222	39877880	659	6	0.5	Exon(exon20of28)	9412	FCGBP	8857
489705	19q	39886240	39886422	183	6	0.667	Promoter(<=1kb)	870	FCGBP	8857
489705	19q	43204774	43205502	729	6	0.833	Promoter(<=1kb)	49	PSG4	5672
489705	19q	43913423	43914878	1456	8	0.5	Exon(exon10of10)	4861	ZNF45	7596
489705	19q	43966037	43967171	1135	6	0.167	Promoter(<=1kb)	-691	ZNF155	7711
489705	19q	43996323	43997366	1044	6	0.333	Exon(exon5of5)	5419	LOC10192806	101928063
489705	19q	52437918	52439242	1325	7	0.429	Exon(exon4of4)	6504	ZNF534	147658
489705	19q	53164551	53166239	1689	6	0.167	Exon(exon4of4)	-5476	ZNF347	84671
489705	19q	55911888	55913077	1190	6	0.667	Exon(exon5of12)	19234	NLRP13	126204
489705	20p	5922421	5923643	1223	9	0.667	Exon(exon4of5)	6923	CHGB	1114
489705	20q	63349752	63350772	1021	6	0.5	3'UTR	3794	CHRNA4	1137
489705	20q	63561666	63565531	3866	11	0.636	Promoter(<=1kb)	-61	HELZ2	85441
489705	21q	26843740	26844859	1120	6	0.667	Promoter(<=1kb)	0	ADAMTS1	9510
489705	21q	44539312	44540035	724	7	0.857	Promoter(<=1kb)	160	KRTAP10-1	386677
489705	21q	44550738	44551416	679	7	0.857	Promoter(<=1kb)	89	KRTAP10-2	386679
489705	21q	44600627	44601692	1066	10	0.7	Promoter(<=1kb)	30	KRTAP10-7	386675
489705	21q	44637474	44638143	670	10	0.4	Promoter(<=1kb)	118	KRTAP10-10	353333
489705	21q	44666460	44666841	382	7	0.714	Promoter(<=1kb)	86	KRTAP12-2	353323
489705	22q	22352950	22353298	349	13	0.538	Exon(exon1of2)	30478	BMS1P20	96610
489705	22q	22758758	22759209	452	6	0.833	Exon(exon1of2)	-63567	MIR650	723778
489705	22q	36191154	36191906	753	6	0.667	3'UTR	9971	APOL4	80832
489705	23p	3320126	3323750	3625	9	0.556	Exon(exon5of7)	22902	MXRA5	25878
489705	23p	8170039	8170243	205	7	0.571	Promoter(1-2kb)	1024	VCX2	51480
489705	23p	38285603	38287917	2315	6	1	3'UTR	-11661	RPGR	6103
491318	1p	12847526	12847780	255	7	0.429	Promoter(<=1kb)	945	HNRNPCL1	343069
491318	1p	12859036	12860079	1044	6	0.333	Promoter(1-2kb)	1950	PRAMEF2	65122
491318	1p	13370686	13370957	272	7	0.429	Promoter(<=1kb)	943	PRAMEF19	645414
491318	1p	16058491	16060000	1510	10	0.9	Exon(exon5of7)	6168	CLCNKB	1188
491318	1p	18481042	18482217	1176	12	0.75	Promoter(<=1kb)	60	KLHDC7A	127707
491318	1p	23874604	23875430	827	9	0.556	Exon(exon2of2)	-6310	FUCA1	2517
491318	1p	40067594	40067675	82	6	0	Promoter(<=1kb)	324	CAP1	10487
491318	1q	152219233	152221375	2143	15	0.733	Promoter(2-3kb)	2818	HRNR	388697
491318	1q	156669844	156670886	1043	6	1	Exon(exon4of4)	6521	NES	10763
491318	1q	169542317	169542882	566	6	0.167	Exon(exon13of25)	-26572	F5	2153
491318	1q	201206099	201209342	3244	10	0.6	Promoter(1-2kb)	1017	IGFN1	91156
491318	1q	226735683	226737239	1557	7	0.857	Promoter(<=1kb)	219	ITPKB	3707
491318	1q	228315976	228318038	2063	8	0.625	Exon(exon50of81)	6492	OBSCN	84033
491318	1q	232805117	232806800	1684	6	0.5	Promoter(<=1kb)	225	MAP10	54627
491318	1q	247841312	247841582	271	6	0.833	Promoter(<=1kb)	314	OR11L1	391189
491318	1q	247949325	247949738	414	10	0.3	Promoter(<=1kb)	467	OR2L8	391190
491318	1q	248294677	248295458	782	7	0.571	Promoter(<=1kb)	142	OR2T12	127064
491318	2p	29071763	29073000	1238	6	0.667	Promoter(1-2kb)	1523	PCARE	388939
491318	2p	48580657	48582454	1798	7	0.571	Promoter(<=1kb)	0	STON1	11037
491318	2q	167246794	167248478	1685	7	0.714	Promoter(<=1kb)	-204	XIRP2	129446
491318	2q	167249575	167251847	2273	6	0.667	Promoter(<=1kb)	893	XIRP2	129446
491318	2q	184936178	184937636	1459	6	0.333	Exon(exon4of4)	69813	ZNF804A	91752
491318	2q	185789865	185794632	4768	11	0.818	Promoter(<=1kb)	0	FSIP2	401024
491318	2q	185805377	185808170	2794	6	0.333	Promoter(<=1kb)	0	FSIP2	401024
491318	2q	233840612	233842185	1574	6	0.667	Promoter(<=1kb)	0	HJURP	55355
491318	2q	238130271	238131546	1276	7	0.286	Promoter(1-2kb)	1323	ESPNL	339768
491318	2q	240041845	240042811	967	7	0.286	Downstream(2-3kb)	3261	OR6B3	150681
491318	3p	31989532	31990905	1374	7	0.286	Exon(exon2of2)	7761	ZNF860	344787
491318	3p	75736880	75739243	2364	48	0.667	Promoter(<=1kb)	0	MIR4273	100422955
491318	3q	98264413	98265098	686	7	0.571	Promoter(<=1kb)	128	OR5H6	79295
491318	4p	5988383	5989749	1367	7	0.571	Promoter(<=1kb)	0	C4orf50	389197
491318	4p	6300792	6302360	1569	7	0.714	Exon(exon8of8)	6021	WFS1	7466
491318	4p	8227004	8228508	1505	8	0.125	Promoter(<=1kb)	-24	SH3TC1	54436
491318	4q	121036404	121037542	1139	6	0.333	Promoter(1-2kb)	1442	NDNF	79625
491318	5q	83537326	83539905	2580	6	0.333	Promoter(1-2kb)	1712	VCAN	1462
491318	5q	140807352	140807737	386	6	0.833	Promoter(<=1kb)	271	PCDHA4	56144
491318	5q	140848579	140850786	2208	7	0.571	Promoter(<=1kb)	807	PCDHA9	9752
491318	5q	141955195	141957660	2466	7	0.714	Promoter(<=1kb)	-668	RNF14	9604
491318	5q	148826877	148828070	1194	6	1	Promoter(1-2kb)	1632	ADRB2	154
491318	5q	151565922	151568455	2534	10	0.8	Promoter(<=1kb)	489	FAT2	2196
491318	6p	1312843	1313745	903	6	0.5	Promoter(<=1kb)	745	FOXQ1	94234
491318	6p	42745312	42746041	730	6	0.667	Promoter(<=1kb)	62	TBCC	6903
491318	6p	46858771	46859502	732	7	0.571	Exon(exon17of21)	3802	ADGRF5	221395
491318	6q	159233455	159234370	916	10	0.5	Exon(exon11of23)	15158	FNDC1	84624
491318	7p	12369637	12370736	1100	6	0.667	3'UTR	-13307	VWDE	221806
491318	7q	100958721	100960873	2153	56	0.482	Promoter(<=1kb)	756	MUC3A	4584
491318	7q	100991195	100994057	2863	12	0.667	Exon(exon5of15)	-18997	MUC12	10071
491318	7q	100995424	100995785	362	7	0.857	Exon(exon5of15)	-17269	MUC12	10071
491318	7q	101034275	101038994	4720	31	0.419	Exon(exon3of12)	-4717	MUC17	140453
491318	7q	156949807	156950570	764	6	0.833	Promoter(<=1kb)	95	NOM1	64434
491318	8p	8376561	8377994	1434	6	1	Exon(exon2of5)	3753	PRAG1	157285
491318	8p	10607245	10608432	1188	8	0.5	Exon(exon4of4)	46711	RP1L1	94137

491318	8p	10609614	10610662	1049	7	0.429	Exon(exon4of4)	44481	RP1L1	94137
491318	8p	11331194	11332026	833	8	0.625	Promoter(<=1kb)	306	SLC35G5	83650
491318	8p	12132686	12133940	1255	6	0.667	Promoter(<=1kb)	498	USP17L7	392197
491318	8p	13021128	13022030	903	7	0.143	Exon(exon5of5)	9115	TRMT9B	57604
491318	8q	143870008	143872631	2624	6	0.5	Exon(exon2of2)	5833	EPPK1	83481
491318	9p	39078723	39078846	124	7	0.714	Exon(exon22of24)	7302	CNTNAP3	79937
491318	9q	76175237	76175296	60	10	0.8	Exon(exon14of14)	-13343	PCSK5	5125
491318	9q	76705179	76707804	2626	8	0.5	Promoter(<=1kb)	121	PCA3	50652
491318	9q	76709263	76710843	1581	8	0.5	Promoter(<=1kb)	0	PRUNE2	158471
491318	9q	87886533	87888398	1866	6	0.667	Exon(exon4of4)	3656	SPATA31E1	286234
491318	9q	104504315	104505071	757	6	0.5	Promoter(<=1kb)	52	OR13F1	138805
491318	9q	122553263	122554071	809	8	0.5	Promoter(<=1kb)	93	OR1N2	138882
491318	9q	122749914	122750547	634	6	0.833	Promoter(<=1kb)	174	OR1L6	392390
491318	9q	133255635	133256205	571	8	0.875	3'UTR	19009	ABO	28
491318	9q	135484803	135487213	2411	8	0.25	Promoter(1-2kb)	1440	PPP1R26	9858
491318	9q	135547960	135548795	836	6	0.667	Promoter(1-2kb)	1805	OBP2A	29991
491318	10q	46549378	46550622	1245	24	0.625	Exon(exon3of3)	4908	GPRIN2	9721
491318	10q	49323169	49326817	3649	14	0.643	Exon(exon3of3)	23895	C10orf71	118461
491318	10q	128103129	128106213	3085	12	0.667	Promoter(<=1kb)	-1	MKI67	4288
491318	11p	244106	244197	92	8	0.5	Promoter(<=1kb)	-232	PSMD13	5719
491318	11p	1246332	1248605	2274	11	0.455	Promoter(1-2kb)	1071	MUC5B-AS1	112577518
491318	11p	5177978	5178478	501	6	0.167	Promoter(<=1kb)	186	OR52Z1	283110
491318	11p	5323451	5324256	806	6	0.5	Promoter(<=1kb)	41	OR51B2	79345
491318	11p	5389704	5390350	647	6	0.5	Promoter(<=1kb)	327	OR51M1	390059
491318	11p	5402638	5403322	685	9	0.444	Promoter(<=1kb)	41	OR51J1	79470
491318	11p	5422212	5423123	912	11	0.636	Promoter(<=1kb)	101	OR51Q1	390061
491318	11p	5440604	5441472	869	6	0.833	Promoter(<=1kb)	42	OR51I1	390063
491318	11p	5515185	5515931	747	6	0.333	Promoter(<=1kb)	768	UBQLNL	143630
491318	11p	5581045	5581738	694	8	0.375	Promoter(<=1kb)	168	OR52B6	340980
491318	11p	5841302	5841883	582	9	0.333	Promoter(<=1kb)	14	OR52E6	390078
491318	11p	5884818	5885061	244	7	0.429	Promoter(<=1kb)	547	OR52E4	390081
491318	11p	11351961	11353007	1047	10	0.2	Promoter(<=1kb)	243	CSNK2A3	283106
491318	11p	12293628	12294368	741	7	0.857	Exon(exon29of35)	6728	MICALCL	84953
491318	11p	18173280	18173901	622	8	0.375	Promoter(<=1kb)	443	MRGPRX4	117196
491318	11q	58214757	58215722	966	8	0.25	Promoter(<=1kb)	12	OR1S1	219959
491318	11q	64315818	64315856	39	6	0.833	Promoter(1-2kb)	1485	TRMT112	51504
491318	11q	85724687	85725825	1139	6	0.5	Promoter(<=1kb)	0	SYTL2	54843
491318	11q	123906790	123907324	535	6	0.667	Promoter(<=1kb)	644	OR8D4	338662
491318	11q	124038366	124038988	623	7	1	Promoter(<=1kb)	13	OR10G7	390265
491318	11q	130914501	130915409	909	10	0.7	Promoter(1-2kb)	1035	SNX19	399979
491318	12p	4626568	4628549	1982	11	0.455	Exon(exon5of6)	14051	DYRK4	8798
491318	12p	6453119	6453670	552	6	0.667	Promoter(<=1kb)	633	TAPBPL	55080
491318	12q	52316096	52317765	1670	6	0.667	Exon(exon4of9)	3633	KRT83	3889
491318	13q	24434450	24435347	898	7	0.571	Exon(exon31of34)	19787	PARP4	143
491318	13q	25096659	25097034	376	14	0.571	Promoter(<=1kb)	791	PABPC3	5042
491318	13q	102732474	102733933	1460	6	0.333	Exon(exon4of4)	25139	CCDC168	643677
491318	14q	20060048	20060884	837	8	0.625	Promoter(<=1kb)	3	OR4L1	122742
491318	14q	21634137	21634589	453	9	0.556	Promoter(<=1kb)	351	OR10G2	26534
491318	14q	70457733	70458948	1216	8	0.5	Exon(exon2of2)	5559	ADAM21	8747
491318	14q	94587512	94587839	328	6	0.5	Exon(exon2of2)	-4219	SERPINA3	12
491318	15q	23440160	23442067	1908	12	0.583	5'UTR	5167	GOLGA6L2	283685
491318	15q	73702465	73703760	1296	6	0.833	Promoter(<=1kb)	-149	CD276	80381
491318	15q	78766049	78766626	578	6	0.667	Promoter(<=1kb)	-936	ADAMTS7	11173
491318	15q	85579423	85582073	2651	15	0.6	Promoter(<=1kb)	-837	AKAP13	11214
491318	15q	99129423	99132517	3095	10	0.5	Exon(exon4of5)	7225	TTC23	64927
491318	15q	100569472	100570097	626	6	0.833	Promoter(<=1kb)	534	LINS1	55180
491318	16p	1228744	1229622	879	6	0.833	Promoter(<=1kb)	540	TPSB2	64499
491318	16p	1486371	1488463	2093	8	0.75	Promoter(<=1kb)	4	PTX4	390667
491318	16q	88714632	88717113	2482	8	0.375	Promoter(<=1kb)	0	MIR4722	100616167
491318	16q	89100686	89101050	365	7	0.571	Promoter(<=1kb)	24	ACSF3	197322
491318	16q	89226863	89228289	1427	8	0.625	Promoter(2-3kb)	2229	ZNF778	197320
491318	17p	10638198	10641099	2902	6	0.333	Exon(exon19of41)	-8169	MYH3	4621
491318	17p	21300581	21300954	374	8	0.75	3'UTR	9112	MAP2K3	5606
491318	17q	76293419	76294016	598	6	0.5	Promoter(2-3kb)	-2167	QRICH2	84074
491318	17q	81645135	81645417	283	6	0.333	Promoter(2-3kb)	2722	TSPAN10	83882
491318	18p	11609646	11610581	936	14	0.643	Promoter(<=1kb)	50	SLC35G4	646000
491318	18q	58535186	58537515	2330	9	0.333	Promoter(<=1kb)	0	ALPK2	115701
491318	19p	1036457	1036914	458	6	0.5	Exon(exon6of7)	-3187	ABCA7	10347
491318	19p	4510548	4513547	3000	11	0.364	Exon(exon3of6)	4157	PLIN4	729359
491318	19p	5455600	5456439	840	7	0.571	Promoter(<=1kb)	183	ZNRF4	148066
491318	19p	8937583	8939234	1652	7	0.857	Exon(exon5of84)	-41493	MUC16	94025
491318	19p	8946306	8951868	5563	18	0.667	Exon(exon3of84)	29474	MUC16	94025
491318	19p	8959116	8967127	8012	33	0.636	Exon(exon3of84)	14215	MUC16	94025
491318	19p	8972751	8978096	5346	13	0.462	Exon(exon1of84)	3246	MUC16	94025
491318	19p	17281820	17284246	2427	9	0.556	Promoter(<=1kb)	0	ANKLE1	126549
491318	19p	18264798	18267409	2612	12	0.583	5'UTR	7002	IQCN	80726
491318	19p	23743906	23745300	1395	6	0.333	Exon(exon4of4)	13537	ZNF681	148213

491318	19q	36996730	36997597	868	10	0.6	Exon(exon10of10)	5677	ZNF568	374900
491318	19q	37886924	37889059	2136	6	0.833	Exon(exon6of6)	17535	WDR87	83889
491318	19q	39877222	39877880	659	6	0.5	Exon(exon20of28)	9412	FCGBP	8857
491318	19q	39886005	39886439	435	6	0.333	Promoter(<=1kb)	853	FCGBP	8857
491318	19q	40880231	40880622	392	6	0.333	Promoter(<=1kb)	-141	CYP2A7	1549
491318	19q	43913423	43914878	1456	9	0.556	Exon(exon10of10)	4861	ZNF45	7596
491318	19q	52365769	52366744	976	6	0.833	Exon(exon6of6)	-3173	ZNF880	400713
491318	19q	52437918	52439242	1325	7	0.429	Exon(exon4of4)	6504	ZNF534	147658
491318	19q	55481352	55483456	2105	7	0.571	Promoter(1-2kb)	-1732	NAT14	57106
491318	19q	55517821	55517975	155	6	0.833	Exon(exon14of14)	18328	SBK2	646643
491318	19q	58368293	58368994	702	8	0.5	Exon(exon3of3)	-5445	ZNF497	162968
491318	20p	5922421	5923394	974	6	0.5	Exon(exon4of5)	6923	CHGB	1114
491318	20q	63349752	63350772	1021	6	0.5	3'UTR	3794	CHRNA4	1137
491318	21q	26843740	26844859	1120	6	0.667	Promoter(<=1kb)	0	ADAMTS1	9510
491318	21q	44666460	44666841	382	7	0.714	Promoter(<=1kb)	86	KRTAP12-2	353323
491318	22q	22352950	22353348	399	14	0.571	Exon(exon1of2)	30478	BMS1P20	96610
491318	22q	36191154	36191906	753	6	0.667	3'UTR	9971	APOL4	80832
491318	23p	3320126	3323750	3625	10	0.5	Exon(exon5of7)	22902	MXRA5	25878
491318	23p	8170039	8170141	103	6	0.5	Promoter(1-2kb)	1126	VCX2	51480
491318	23p	35802148	35803010	863	7	0.571	5'UTR	3357	MAGEB16	139604
491915	1p	12795695	12795957	263	6	0.5	Promoter(1-2kb)	1004	PRAMEF1	65121
491915	1p	12847526	12847780	255	7	0.429	Promoter(<=1kb)	945	HNRNPCL1	343069
491915	1p	12859036	12860079	1044	6	0.333	Promoter(1-2kb)	1950	PRAMEF2	65122
491915	1p	13370686	13371119	434	7	0.429	Promoter(<=1kb)	781	PRAMEF19	645414
491915	1p	16058491	16060000	1510	10	0.9	Exon(exon5of7)	6168	CLCNKB	1188
491915	1p	18481403	18482217	815	6	0.667	Promoter(<=1kb)	421	KLHDC7A	127707
491915	1p	23874604	23875430	827	9	0.556	Exon(exon2of2)	-6310	FUCA1	2517
491915	1p	31814028	31815123	1096	6	0.5	Promoter(<=1kb)	0	SPOCD1	90853
491915	1p	40067594	40067675	82	6	0	Promoter(<=1kb)	324	CAP1	10487
491915	1p	89186388	89186419	32	9	0.556	Promoter(<=1kb)	107	GBP4	115361
491915	1q	152218469	152221375	2907	17	0.706	Promoter(2-3kb)	2818	HRNR	388697
491915	1q	152302796	152313891	11096	40	0.6	Promoter(<=1kb)	0	FLG-AS1	339400
491915	1q	156669844	156671745	1902	7	1	Exon(exon4of4)	5662	NES	10763
491915	1q	158765805	158766655	851	6	0.5	Promoter(<=1kb)	47	OR6N1	128372
491915	1q	201206099	201209342	3244	10	0.6	Promoter(1-2kb)	1017	IGFN1	91156
491915	1q	201210956	201212866	1911	6	0.333	Promoter(<=1kb)	0	IGFN1	91156
491915	1q	247841312	247841582	271	6	0.833	Promoter(<=1kb)	314	OR11L1	391189
491915	1q	247949325	247949738	414	9	0.333	Promoter(<=1kb)	467	OR2L8	391190
491915	1q	248681658	248682198	541	7	0.571	Promoter(<=1kb)	130	OR14I1	401994
491915	2q	102351547	102351902	356	7	0.429	Exon(exon11of11)	-4027	IL18R1	8809
491915	2q	178739433	178741811	2379	6	0.5	Exon(exon45of191)	26014	TTN	7273
491915	2q	184936178	184937636	1459	6	0.5	Exon(exon4of4)	69813	ZNF804A	91752
491915	2q	185790999	185794632	3634	9	0.778	Promoter(<=1kb)	0	FSIP2	401024
491915	2q	185805377	185808170	2794	7	0.286	Promoter(<=1kb)	0	FSIP2	401024
491915	2q	217847583	217848559	977	6	0.833	Exon(exon19of33)	-5423	TNS1	7145
491915	2q	219489386	219491798	2413	6	0.5	Promoter(<=1kb)	-69	SPEGNB	100996693
491915	2q	233840612	233842185	1574	7	0.714	Promoter(<=1kb)	0	HJURP	55355
491915	2q	238130416	238131546	1131	6	0.333	Promoter(1-2kb)	1468	ESPNL	339768
491915	3p	31989532	31990905	1374	7	0.286	Exon(exon2of2)	7761	ZNF860	344787
491915	3p	75736880	75739243	2364	66	0.591	Promoter(<=1kb)	0	MIR4273	100422955
491915	3q	98264413	98265098	686	7	0.571	Promoter(<=1kb)	128	OR5H6	79295
491915	3q	194341097	194342571	1475	7	0.714	Exon(exon2of2)	8747	CPN2	1370
491915	4p	5988383	5989749	1367	7	0.571	Promoter(<=1kb)	0	C4orf50	389197
491915	4p	6300792	6302360	1569	6	0.833	Exon(exon8of8)	6021	WFS1	7466
491915	4p	7433331	7434512	1182	7	0.714	Promoter(<=1kb)	418	PSAPL1	768239
491915	4p	8227004	8228508	1505	8	0.125	Promoter(<=1kb)	-24	SH3TC1	54436
491915	4p	10443803	10446224	2422	6	0.333	Exon(exon3of3)	10952	ZNF518B	85460
491915	4p	38796894	38798702	1809	6	0.5	Exon(exon4of4)	5674	TLR1	7096
491915	4q	121036404	121037542	1139	6	0.333	Promoter(1-2kb)	1442	NDNF	79625
491915	5p	795818	796237	420	7	0.714	3'UTR	4908	ZDHHC11	79844
491915	5q	79728956	79730716	1761	7	0.286	Exon(exon2of13)	-7426	CMYA5	202333
491915	5q	79731782	79734523	2742	13	0.308	Exon(exon2of13)	-3619	CMYA5	202333
491915	5q	83537326	83539905	2580	6	0.333	Promoter(1-2kb)	1712	VCAN	1462
491915	5q	140848579	140850786	2208	7	0.571	Promoter(<=1kb)	807	PCDHA9	9752
491915	5q	141174000	141175025	1026	7	0.714	Promoter(1-2kb)	1356	PCDHB7	56129
491915	5q	141178631	141180333	1703	6	0.833	Promoter(<=1kb)	841	PCDHB8	56128
491915	5q	141183999	141184688	690	7	0.857	Promoter(2-3kb)	-2473	PCDHB9	56127
491915	5q	141955356	141957660	2305	6	0.667	Promoter(<=1kb)	-668	RNF14	9604
491915	5q	148826877	148828070	1194	7	0.857	Promoter(1-2kb)	1632	ADRB2	154
491915	5q	151565922	151568158	2237	9	0.778	Promoter(<=1kb)	786	FAT2	2196
491915	6p	46858771	46859502	732	8	0.5	Exon(exon17of21)	3802	ADGRF5	221395
491915	6q	64591274	64591961	688	10	0.5	Exon(exon26of43)	121374	EYS	346007
491915	6q	149888581	149889987	1407	6	0.667	Promoter(<=1kb)	-116	RAET1E-AS1	100652739
491915	6q	159233455	159234370	916	10	0.5	Exon(exon11of23)	15158	FNDC1	84624
491915	7p	53035678	53036385	708	7	1	Promoter(<=1kb)	45	POM121L12	285877
491915	7q	100958977	100960873	1897	50	0.48	Promoter(1-2kb)	1012	MUC3A	4584
491915	7q	100991195	100992398	1204	9	0.667	Exon(exon5of15)	-20656	MUC12	10071

491915	7q	100995547	100995785	239	7	0.857	Exon(exon5of15)	-17269	MUC12	10071
491915	8p	10607375	10608261	887	6	0.5	Exon(exon4of4)	46882	RP1L1	94137
491915	8p	12137171	12138641	1471	7	0.857	Promoter(<=1kb)	436	USP17L2	377630
491915	8q	138151949	138153046	1098	6	0.667	Promoter(<=1kb)	0	FAM135B	51059
491915	9q	76705724	76707804	2081	6	0.5	Promoter(<=1kb)	666	PCA3	50652
491915	9q	104598641	104599361	721	9	0.556	Promoter(<=1kb)	52	OR13C5	138799
491915	9q	122553263	122554071	809	7	0.429	Promoter(<=1kb)	93	OR1N2	138882
491915	9q	122628595	122629398	804	7	0.286	Promoter(<=1kb)	175	OR1B1	347169
491915	9q	122749914	122750547	634	6	0.833	Promoter(<=1kb)	174	OR1L6	392390
491915	9q	135484803	135487213	2411	10	0.4	Promoter(1-2kb)	1440	PPP1R26	9858
491915	10p	30027143	30029814	2672	7	0.429	Exon(exon3of4)	29772	JCAD	57608
491915	10q	46549378	46550723	1346	25	0.64	Exon(exon3of3)	4807	GPRIN2	9721
491915	10q	49323169	49326817	3649	14	0.643	Exon(exon3of3)	23895	C10orf71	118461
491915	10q	122084988	122087840	2853	8	0.75	Exon(exon4of23)	-25190	TACC2	10579
491915	10q	128102594	128105201	2608	6	0.667	Promoter(<=1kb)	0	MKI67	4288
491915	11p	1194354	1196902	2549	7	0.571	Exon(exon34of49)	-26164	MUC5B	727897
491915	11p	1246095	1248605	2511	13	0.385	Promoter(1-2kb)	1071	MUC5B-AS1	112577518
491915	11p	1249698	1251628	1931	6	0.833	Promoter(<=1kb)	-22	MUC5B-AS1	112577518
491915	11p	5177978	5178478	501	6	0.167	Promoter(<=1kb)	186	OR52Z1	283110
491915	11p	5422212	5423123	912	11	0.636	Promoter(<=1kb)	101	OR51Q1	390061
491915	11p	5440761	5441472	712	6	0.833	Promoter(<=1kb)	42	OR51I1	390063
491915	11p	5515185	5515931	747	6	0.333	Promoter(<=1kb)	768	UBQLNL	143630
491915	11p	5581045	5581738	694	8	0.375	Promoter(<=1kb)	168	OR52B6	340980
491915	11p	5884818	5885061	244	6	0.333	Promoter(<=1kb)	547	OR52E4	390081
491915	11p	11351961	11352736	776	9	0.222	Promoter(<=1kb)	514	CSNK2A3	283106
491915	11p	12293639	12294368	730	6	0.833	Exon(exon29of35)	6739	MICALCL	84953
491915	11q	58214757	58215722	966	8	0.25	Promoter(<=1kb)	12	OR1S1	219959
491915	11q	64116513	64118232	1720	6	0.667	Exon(exon2of2)	8702	MACROD1	28992
491915	11q	64315797	64315856	60	8	0.75	Promoter(1-2kb)	1485	TRMT112	51504
491915	11q	85724687	85725825	1139	6	0.5	Promoter(<=1kb)	0	SYTL2	54843
491915	11q	123906790	123907324	535	6	0.667	Promoter(<=1kb)	644	OR8D4	338662
491915	11q	124038366	124038988	623	7	1	Promoter(<=1kb)	13	OR10G7	390265
491915	12p	4626571	4628549	1979	8	0.5	Exon(exon5of6)	14054	DYRK4	8798
491915	12p	6453119	6453670	552	6	0.667	Promoter(<=1kb)	633	TAPBPL	55080
491915	12q	49795819	49797137	1319	6	0.5	Promoter(<=1kb)	0	NCKAP5L	57701
491915	13q	24434450	24435347	898	7	0.571	Exon(exon31of34)	19787	PARP4	143
491915	13q	25096713	25097057	345	9	0.444	Promoter(<=1kb)	845	PABPC3	5042
491915	13q	102732424	102733933	1510	7	0.429	Exon(exon4of4)	25139	CCDC168	643677
491915	14q	20060048	20060884	837	8	0.625	Promoter(<=1kb)	3	OR4L1	122742
491915	14q	20640982	20641567	586	6	0.5	Promoter(<=1kb)	124	OR6S1	341799
491915	14q	21634137	21634589	453	9	0.556	Promoter(<=1kb)	351	OR10G2	26534
491915	14q	44504986	44506403	1418	6	0.667	Promoter(<=1kb)	880	FSCB	84075
491915	14q	70457520	70458540	1021	10	0.3	Exon(exon2of2)	5346	ADAM21	8747
491915	14q	94587512	94587839	328	6	0.5	Exon(exon2of2)	-4219	SERPINA3	12
491915	14q	104939262	104950538	11277	49	0.51	5'UTR	4864	AHNAK2	113146
491915	15q	23439979	23442067	2089	10	0.6	5'UTR	5167	GOLGA6L2	283685
491915	15q	73702465	73703760	1296	6	0.833	Promoter(<=1kb)	-149	CD276	80381
491915	15q	85579423	85581800	2378	14	0.571	Promoter(1-2kb)	-1110	AKAP13	11214
491915	16p	1486371	1488463	2093	8	0.75	Promoter(<=1kb)	4	PTX4	390667
491915	16q	74391549	74392004	456	8	0.625	Exon(exon7of7)	13671	NPIPB15	440348
491915	16q	88428539	88429600	1062	6	0.333	Exon(exon3of3)	-23680	ZFPM1	161882
491915	16q	88430623	88432051	1429	6	0.5	Exon(exon3of3)	-21229	ZFPM1	161882
491915	16q	88714632	88717113	2482	7	0.429	Promoter(<=1kb)	0	MIR4722	100616167
491915	16q	89226863	89228419	1557	9	0.556	Promoter(2-3kb)	2229	ZNF778	197320
491915	17p	7846914	7848460	1547	6	0.667	Promoter(1-2kb)	1999	KDM6B	23135
491915	17p	21300581	21300978	398	12	0.75	3'UTR	9112	MAP2K3	5606
491915	17p	21415470	21416404	935	7	1	Exon(exon3of3)	10334	KCNJ12	3768
491915	17q	76293419	76294016	598	6	0.5	Promoter(2-3kb)	-2167	QRICH2	84074
491915	17q	81645135	81645417	283	6	0.333	Promoter(2-3kb)	2722	TSPAN10	83882
491915	18p	11609646	11610491	846	13	0.769	Promoter(<=1kb)	50	SLC35G4	646000
491915	18p	14542649	14543140	492	6	0.5	Promoter(<=1kb)	6	POTEC	388468
491915	18q	58535186	58538030	2845	19	0.526	Promoter(<=1kb)	0	ALPK2	115701
491915	19p	620392	622336	1945	6	0.5	Promoter(1-2kb)	-1166	POLRMT	5442
491915	19p	1004711	1005532	822	7	0.571	Exon(exon3of9)	4292	GRIN3B	116444
491915	19p	4510711	4513547	2837	18	0.444	Exon(exon3of6)	4157	PLIN4	729359
491915	19p	5455600	5456439	840	7	0.571	Promoter(<=1kb)	183	ZNRF4	148066
491915	19p	8333830	8334965	1136	6	0.667	Promoter(<=1kb)	0	KANK3	256949
491915	19p	8948231	8951868	3638	12	0.583	Exon(exon3of84)	29474	MUC16	94025
491915	19p	8959403	8962066	2664	7	0.429	Exon(exon3of84)	19276	MUC16	94025
491915	19p	8964274	8967127	2854	21	0.667	Exon(exon3of84)	14215	MUC16	94025
491915	19p	15087213	15088040	828	9	0.333	Promoter(<=1kb)	233	OR1I1	126370
491915	19p	17281820	17284246	2427	9	0.556	Promoter(<=1kb)	0	ANKLE1	126549
491915	19p	18264753	18267409	2657	14	0.643	5'UTR	7002	IQCN	80726
491915	19p	21971930	21974500	2571	8	0.75	Exon(exon4of4)	14408	ZNF208	7757
491915	19q	39876927	39878231	1305	8	0.625	Exon(exon20of28)	9061	FCGBP	8857
491915	19q	39886240	39886422	183	6	0.667	Promoter(<=1kb)	870	FCGBP	8857
491915	19q	43846955	43848536	1582	6	0.833	3'UTR	13450	ZNF283	284349

491915	19q	43913423	43914878	1456	8	0.5	Exon(exon10of10)	4861	ZNF45	7596
491915	19q	44106512	44108078	1567	7	0	Exon(exon6of6)	-4103	ZNF225	7768
491915	19q	44327836	44329698	1863	6	0.5	Exon(exon4of4)	-22790	ZNF235	9310
491915	19q	48873325	48875925	2601	11	0.545	Promoter(<=1kb)	904	PPP1R15A	23645
491915	19q	51745958	51746963	1006	7	0.571	Exon(exon3of3)	3848	FPR1	2357
491915	19q	52437918	52439242	1325	7	0.429	Exon(exon4of4)	6504	ZNF534	147658
491915	19q	55481625	55483456	1832	8	0.5	Promoter(1-2kb)	-1732	NAT14	57106
491915	19q	55517821	55518851	1031	9	1	Exon(exon14of14)	17452	SBK2	646643
491915	19q	55911888	55913077	1190	6	0.667	Exon(exon5of12)	19234	NLRP13	126204
491915	19q	58368293	58368875	583	7	0.429	Exon(exon3of3)	-5445	ZNF497	162968
491915	20q	63349752	63350772	1021	6	0.5	3'UTR	3794	CHRNA4	1137
491915	20q	63561666	63565531	3866	11	0.636	Promoter(<=1kb)	-61	HELZ2	85441
491915	21q	41740862	41741990	1129	6	0.833	Exon(exon2of2)	4851	MIR6814	102465488
491915	21q	44600627	44601692	1066	9	0.778	Promoter(<=1kb)	30	KRTAP10-7	386675
491915	21q	44637474	44638041	568	10	0.4	Promoter(<=1kb)	118	KRTAP10-10	353333
491915	22q	22352950	22353380	431	16	0.5	Exon(exon1of2)	30478	BMS1P20	96610
491915	22q	22646441	22646811	371	6	0.333	Promoter(<=1kb)	0	GGTLC2	91227
491915	22q	36191154	36191906	753	7	0.714	3'UTR	9971	APOL4	80832
491915	22q	37723206	37726455	3250	6	0.667	3'UTR	-19778	TRIOBP	11078
491915	22q	49884187	49885288	1102	6	0.167	Exon(exon2of2)	22630	ALG12	79087
491915	23p	3320126	3323750	3625	9	0.556	Exon(exon5of7)	22902	MXRA5	25878
491915	23p	8170039	8170141	103	6	0.5	Promoter(1-2kb)	1126	VCX2	51480
491915	23p	35802148	35803010	863	7	0.571	5'UTR	3357	MAGEB16	139604
491915	23p	38285569	38287917	2349	7	1	3'UTR	-11627	RPGR	6103
492485	1p	13369166	13369564	399	7	0.714	Promoter(2-3kb)	2336	PRAMEF19	645414
492485	1p	13370686	13371119	434	7	0.429	Promoter(<=1kb)	781	PRAMEF19	645414
492485	1p	13391965	13392142	178	6	0.333	Promoter(2-3kb)	2333	PRAMEF17	391004
492485	1p	16058491	16060000	1510	10	0.9	Exon(exon5of7)	6168	CLCNKB	1188
492485	1p	18481403	18482217	815	6	0.667	Promoter(<=1kb)	421	KLHDC7A	127707
492485	1p	18875462	18877503	2042	6	0.5	Exon(exon10of16)	5772	MIR4695	100616120
492485	1p	23874604	23875430	827	8	0.5	Exon(exon2of2)	-6310	FUCA1	2517
492485	1p	40067594	40067675	82	6	0	Promoter(<=1kb)	324	CAP1	10487
492485	1p	62273232	62275080	1849	7	0.429	Promoter(<=1kb)	-441	KANK4	163782
492485	1p	89186388	89186419	32	9	0.556	Promoter(<=1kb)	107	GBP4	115361
492485	1q	152213274	152213316	43	7	0.571	Exon(exon3of3)	10877	HRNR	388697
492485	1q	152218469	152221375	2907	17	0.706	Promoter(2-3kb)	2818	HRNR	388697
492485	1q	152303673	152313891	10219	40	0.55	Promoter(<=1kb)	0	FLG-AS1	339400
492485	1q	223393517	223394466	950	6	0.667	Promoter(<=1kb)	102	CCDC185	164127
492485	1q	228315976	228318026	2051	7	0.571	Exon(exon50of81)	6492	OBSCN	84033
492485	1q	247841312	247841582	271	6	0.833	Promoter(<=1kb)	314	OR11L1	391189
492485	1q	247949443	247949738	296	9	0.333	Promoter(<=1kb)	585	OR2L8	391190
492485	1q	248294677	248295458	782	7	0.571	Promoter(<=1kb)	142	OR2T12	127064
492485	2p	48580657	48582454	1798	7	0.571	Promoter(<=1kb)	0	STON1	11037
492485	2q	130914528	130916712	2185	6	0.333	Promoter(<=1kb)	0	ARHGEF4	50649
492485	2q	132781993	132785001	3009	7	0.429	Promoter(<=1kb)	0	NCKAP5	344148
492485	2q	167246794	167248478	1685	7	0.714	Promoter(<=1kb)	-204	XIRP2	129446
492485	2q	184936178	184937636	1459	6	0.333	Exon(exon4of4)	69813	ZNF804A	91752
492485	2q	217847583	217848559	977	6	0.833	Exon(exon19of33)	-5423	TNS1	7145
492485	2q	219271337	219271649	313	6	0.667	Exon(exon4of4)	6227	TUBA4A	7277
492485	2q	238130271	238131546	1276	7	0.286	Promoter(1-2kb)	1323	ESPNL	339768
492485	2q	240041845	240042131	287	6	0.167	Downstream(2-3kb)	3941	OR6B3	150681
492485	3p	31989643	31990470	828	6	0.167	Exon(exon2of2)	7872	ZNF860	344787
492485	3p	75736880	75739007	2128	53	0.623	Promoter(<=1kb)	0	MIR4273	100422955
492485	4p	5988383	5989749	1367	7	0.571	Promoter(<=1kb)	0	C4orf50	389197
492485	4p	6300792	6302360	1569	7	0.857	Exon(exon8of8)	6021	WFS1	7466
492485	4p	7433331	7434512	1182	6	0.833	Promoter(<=1kb)	418	PSAPL1	768239
492485	4p	8227004	8228508	1505	8	0.125	Promoter(<=1kb)	-24	SH3TC1	54436
492485	4q	112431241	112432293	1053	7	0.429	3'UTR	4735	ALPK1	80216
492485	4q	185458217	185460011	1795	9	0.556	Promoter(<=1kb)	0	CCDC110	256309
492485	4q	186617176	186621601	4426	11	0.364	Exon(exon10of27)	-7106	FAT1	2195
492485	5p	795818	796218	401	6	0.833	3'UTR	4927	ZDHHC11	79844
492485	5q	83537326	83539905	2580	6	0.333	Promoter(1-2kb)	1712	VCAN	1462
492485	5q	140848579	140850786	2208	6	0.5	Promoter(<=1kb)	807	PCDHA9	9752
492485	5q	141174000	141175025	1026	6	0.833	Promoter(1-2kb)	1356	PCDHB7	56129
492485	5q	141183999	141184688	690	6	1	Promoter(2-3kb)	-2473	PCDHB9	56127
492485	5q	141955356	141957660	2305	6	0.667	Promoter(<=1kb)	-668	RNF14	9604
492485	5q	151521550	151522069	520	6	0.667	Promoter(<=1kb)	79	MIR6499	102465246
492485	6p	1312843	1313745	903	6	0.5	Promoter(<=1kb)	745	FOXQ1	94234
492485	6p	46858771	46859502	732	7	0.571	Exon(exon17of21)	3802	ADGRF5	221395
492485	6q	149888581	149890867	2287	7	0.714	Promoter(<=1kb)	0	RAET1E-AS1	100652739
492485	6q	159231899	159234370	2472	10	0.5	Exon(exon11of23)	13602	FNDC1	84624
492485	7p	12369637	12370736	1100	6	0.667	3'UTR	-13307	VWDE	221806
492485	7p	45082865	45084866	2002	6	0.333	Promoter(1-2kb)	-1465	NACAD	23148
492485	7q	100958977	100960873	1897	52	0.442	Promoter(1-2kb)	1012	MUC3A	4584
492485	7q	100991195	100992398	1204	7	0.571	Exon(exon5of15)	-20656	MUC12	10071
492485	7q	100995575	100995785	211	7	0.714	Exon(exon5of15)	-17269	MUC12	10071
492485	7q	101004421	101004836	416	6	0.667	Exon(exon5of15)	-8218	MUC12	10071

492485	7q	101034361	101040583	6223	37	0.486	Exon(exon3of12)	-3128	MUC17	140453
492485	8p	10609614	10612307	2694	10	0.9	Exon(exon4of4)	42836	RP1L1	94137
492485	8p	13021128	13022030	903	7	0.143	Exon(exon5of5)	9115	TRMT9B	57604
492485	9p	116800	117713	914	6	0.667	Promoter(<=1kb)	491	FOXD4	2298
492485	9q	76175237	76175300	64	6	0.5	Exon(exon14of14)	-13339	PCSK5	5125
492485	9q	76703555	76707804	4250	13	0.538	Promoter(<=1kb)	0	PCA3	50652
492485	9q	76709523	76710843	1321	6	0.5	Promoter(<=1kb)	0	PRUNE2	158471
492485	9q	87885490	87888819	3330	9	0.556	Promoter(2-3kb)	2613	SPATA31E1	286234
492485	9q	104504315	104505071	757	6	0.5	Promoter(<=1kb)	52	OR13F1	138805
492485	9q	104598545	104599361	817	12	0.583	Promoter(<=1kb)	52	OR13C5	138799
492485	9q	122553263	122554071	809	7	0.429	Promoter(<=1kb)	93	OR1N2	138882
492485	9q	122628595	122629130	536	6	0.333	Promoter(<=1kb)	443	OR1B1	347169
492485	9q	122749914	122750547	634	6	0.833	Promoter(<=1kb)	174	OR1L6	392390
492485	9q	135484803	135487213	2411	8	0.25	Promoter(1-2kb)	1440	PPP1R26	9858
492485	9q	135547960	135548795	836	8	0.625	Promoter(1-2kb)	1805	OBP2A	29991
492485	10q	46549378	46550723	1346	25	0.64	Exon(exon3of3)	4807	GPRIN2	9721
492485	10q	49323187	49326817	3631	11	0.545	Exon(exon3of3)	23913	C10orf71	118461
492485	10q	128102594	128106296	3703	8	0.625	Promoter(<=1kb)	0	MKI67	4288
492485	11p	244106	244197	92	8	0.5	Promoter(<=1kb)	-232	PSMD13	5719
492485	11p	1194354	1196902	2549	7	0.571	Exon(exon34of49)	-26164	MUC5B	727897
492485	11p	1241677	1243593	1917	8	0.5	Exon(exon31of49)	6083	MUC5B-AS1	112577518
492485	11p	1246095	1248605	2511	12	0.583	Promoter(1-2kb)	1071	MUC5B-AS1	112577518
492485	11p	5177978	5178478	501	6	0.167	Promoter(<=1kb)	186	OR52Z1	283110
492485	11p	5323451	5324256	806	6	0.5	Promoter(<=1kb)	41	OR51B2	79345
492485	11p	5389704	5390350	647	6	0.5	Promoter(<=1kb)	327	OR51M1	390059
492485	11p	5422212	5423123	912	10	0.7	Promoter(<=1kb)	101	OR51Q1	390061
492485	11p	5544676	5545259	584	6	0.5	Promoter(<=1kb)	290	OR52H1	390067
492485	11p	5581045	5581738	694	8	0.375	Promoter(<=1kb)	168	OR52B6	340980
492485	11p	11351961	11352736	776	9	0.222	Promoter(<=1kb)	514	CSNK2A3	283106
492485	11p	12293628	12294368	741	7	0.857	Exon(exon29of35)	6728	MICALCL	84953
492485	11q	58214757	58215722	966	8	0.25	Promoter(<=1kb)	12	OR1S1	219959
492485	11q	64315797	64315856	60	8	0.75	Promoter(1-2kb)	1485	TRMT112	51504
492485	11q	85724687	85725825	1139	6	0.5	Promoter(<=1kb)	0	SYTL2	54843
492485	11q	124015600	124016477	878	7	0.143	Promoter(<=1kb)	25	OR10G4	390264
492485	11q	124038366	124038988	623	9	1	Promoter(<=1kb)	13	OR10G7	390265
492485	12p	4626571	4628549	1979	9	0.556	Exon(exon5of6)	14054	DYRK4	8798
492485	12p	8222174	8223514	1341	6	0.667	Exon(exon5of6)	4073	FAM90A1	55138
492485	13q	24434450	24435347	898	7	0.571	Exon(exon31of34)	19787	PARP4	143
492485	13q	25096924	25097182	259	8	0.5	Promoter(1-2kb)	1056	PABPC3	5042
492485	14q	20060048	20060523	476	6	0.5	Promoter(<=1kb)	3	OR4L1	122742
492485	14q	21634137	21634644	508	10	0.6	Promoter(<=1kb)	296	OR10G2	26534
492485	14q	44504986	44506403	1418	6	0.667	Promoter(<=1kb)	880	FSCB	84075
492485	14q	70457520	70458540	1021	12	0.333	Exon(exon2of2)	5346	ADAM21	8747
492485	14q	104939262	104942618	3357	10	0.2	5'UTR	7102	PLD4	122618
492485	14q	104943622	104945444	1823	7	0.571	Exon(exon6of6)	9958	AHNAK2	113146
492485	14q	104947901	104953878	5978	35	0.486	Promoter(1-2kb)	1524	AHNAK2	113146
492485	15q	23440370	23442067	1698	11	0.636	5'UTR	5167	GOLGA6L2	283685
492485	15q	78766049	78766618	570	6	0.833	Promoter(<=1kb)	-936	ADAMTS7	11173
492485	15q	85579423	85581800	2378	15	0.533	Promoter(1-2kb)	-1110	AKAP13	11214
492485	15q	99130060	99132394	2335	6	0.5	Exon(exon4of5)	7348	TTC23	64927
492485	15q	100569472	100570097	626	6	0.833	Promoter(<=1kb)	534	LINS1	55180
492485	15q	101065940	101067308	1369	6	0.667	Promoter(1-2kb)	1524	LRRK1	79705
492485	16p	788343	790597	2255	7	0.286	Promoter(<=1kb)	0	RPUSD1	113000
492485	16p	1998795	2000191	1397	7	0.571	Promoter(<=1kb)	0	ZNF598	90850
492485	16p	4883938	4885635	1698	6	1	Exon(exon22of22)	4701	PPL	5493
492485	16q	74391549	74391928	380	8	0.75	Exon(exon7of7)	13671	NPIPB15	440348
492485	16q	84178965	84179828	864	6	0.833	Promoter(1-2kb)	1590	TAF1C	9013
492485	16q	88428539	88431889	3351	11	0.364	Exon(exon3of3)	-21391	ZFPM1	161882
492485	16q	88433049	88436097	3049	7	0.714	Exon(exon3of3)	-17183	ZFPM1	161882
492485	16q	89100686	89101050	365	7	0.571	Promoter(<=1kb)	24	ACSF3	197322
492485	16q	89226863	89228289	1427	8	0.625	Promoter(2-3kb)	2229	ZNF778	197320
492485	17p	10638198	10641099	2902	7	0.286	Exon(exon19of41)	-8169	MYH3	4621
492485	17p	21300581	21300978	398	12	0.75	3'UTR	9112	MAP2K3	5606
492485	17q	76293419	76294016	598	6	0.5	Promoter(2-3kb)	-2167	QRICH2	84074
492485	17q	81645135	81645607	473	8	0.375	Promoter(2-3kb)	2722	TSPAN10	83882
492485	18p	11609904	11610491	588	7	0.714	Promoter(<=1kb)	308	SLC35G4	646000
492485	18q	58535186	58538030	2845	18	0.556	Promoter(<=1kb)	0	ALPK2	115701
492485	19p	4510548	4513547	3000	25	0.52	Exon(exon3of6)	4157	PLIN4	729359
492485	19p	5455600	5456439	840	7	0.571	Promoter(<=1kb)	183	ZNRF4	148066
492485	19p	8946732	8951868	5137	15	0.533	Exon(exon3of84)	29474	MUC16	94025
492485	19p	12430400	12432437	2038	9	0.444	3'UTR	8584	ZNF443	10224
492485	19p	18264753	18267409	2657	8	0.5	5'UTR	7002	IQCN	80726
492485	19p	21971930	21974500	2571	8	0.75	Exon(exon4of4)	14408	ZNF208	7757
492485	19q	39877222	39877880	659	6	0.5	Exon(exon20of28)	9412	FCGBP	8857
492485	19q	39886240	39886422	183	6	0.667	Promoter(<=1kb)	870	FCGBP	8857
492485	19q	40875742	40878797	3056	8	0.25	Promoter(<=1kb)	0	CYP2A7	1549
492485	19q	40880231	40880622	392	6	0.333	Promoter(<=1kb)	-141	CYP2A7	1549

492485	19q	43204061	43205504	1444	6	0.833	Promoter(<=1kb)	0	PSG4	5672
492485	19q	43913423	43914878	1456	7	0.429	Exon(exon10of10)	4861	ZNF45	7596
492485	19q	43996326	43997366	1041	6	0.5	Exon(exon5of5)	5419	LOC10192806[illegible]	101928063
492485	19q	44327836	44329698	1863	6	0.5	Exon(exon4of4)	-22790	ZNF235	9310
492485	19q	52437918	52439242	1325	7	0.429	Exon(exon4of4)	6504	ZNF534	147658
492485	19q	55911888	55913077	1190	6	0.667	Exon(exon5of12)	19234	NLRP13	126204
492485	19q	58368217	58368875	659	8	0.5	Exon(exon3of3)	-5369	ZNF497	162968
492485	20q	63559968	63565825	5858	15	0.6	Promoter(<=1kb)	0	HELZ2	85441
492485	21q	26843740	26844859	1120	6	0.667	Promoter(<=1kb)	0	ADAMTS1	9510
492485	21q	44550835	44551416	582	6	0.833	Promoter(<=1kb)	89	KRTAP10-2	386679
492485	21q	44637476	44638143	668	10	0.5	Promoter(<=1kb)	120	KRTAP10-10	353333
492485	22q	22352950	22353380	431	16	0.5	Exon(exon1of2)	30478	BMS1P20	96610
492485	22q	36191154	36191906	753	6	0.667	3'UTR	9971	APOL4	80832
492485	23p	8170039	8170141	103	6	0.5	Promoter(1-2kb)	1126	VCX2	51480
492485	23p	35802148	35803010	863	7	0.571	5'UTR	3357	MAGEB16	139604
492794	1p	12847526	12847995	470	10	0.4	Promoter(<=1kb)	730	HNRNPCL1	343069
492794	1p	12859036	12860079	1044	6	0.333	Promoter(1-2kb)	1950	PRAMEF2	65122
492794	1p	13370686	13371119	434	7	0.429	Promoter(<=1kb)	781	PRAMEF19	645414
492794	1p	16048038	16049824	1787	6	0.5	Promoter(<=1kb)	0	CLCNKB	1188
492794	1p	16058491	16060000	1510	13	0.846	Exon(exon5of7)	6168	CLCNKB	1188
492794	1p	18481403	18482217	815	6	0.667	Promoter(<=1kb)	421	KLHDC7A	127707
492794	1p	23874604	23875430	827	8	0.5	Exon(exon2of2)	-6310	FUCA1	2517
492794	1p	40067594	40067675	82	6	0	Promoter(<=1kb)	324	CAP1	10487
492794	1p	89186388	89186419	32	9	0.556	Promoter(<=1kb)	107	GBP4	115361
492794	1q	152213286	152213347	62	8	0.5	Exon(exon3of3)	10846	HRNR	388697
492794	1q	152219233	152221375	2143	16	0.75	Promoter(2-3kb)	2818	HRNR	388697
492794	1q	169540901	169542882	1982	9	0.222	Exon(exon13of25)	-25156	F5	2153
492794	1q	183647749	183648558	810	7	0.429	Exon(exon2of2)	4758	APOBEC4	403314
492794	1q	222628664	222629929	1266	6	0.333	Promoter(<=1kb)	190	MIA3	375056
492794	1q	228315976	228318049	2074	8	0.625	Exon(exon50of81)	6492	OBSCN	84033
492794	1q	247841312	247841582	271	6	0.833	Promoter(<=1kb)	314	OR11L1	391189
492794	1q	247921132	247921847	716	6	0.833	Promoter(<=1kb)	114	OR2T8	343172
492794	1q	247949325	247949738	414	10	0.3	Promoter(<=1kb)	467	OR2L8	391190
492794	2q	132783061	132784972	1912	7	0.429	Promoter(1-2kb)	-1038	NCKAP5	344148
492794	2q	167246794	167248478	1685	7	0.714	Promoter(<=1kb)	-204	XIRP2	129446
492794	2q	178739433	178741811	2379	6	0.5	Exon(exon45of191)	26014	TTN	7273
492794	2q	185789865	185794632	4768	10	0.8	Promoter(<=1kb)	0	FSIP2	401024
492794	2q	185805377	185808170	2794	6	0.333	Promoter(<=1kb)	0	FSIP2	401024
492794	2q	217847583	217848559	977	6	0.833	Exon(exon19of33)	-5423	TNS1	7145
492794	2q	233681970	233682559	590	6	0.667	Promoter(<=1kb)	0	UGT1A7	54577
492794	2q	238130416	238131546	1131	6	0.333	Promoter(1-2kb)	1468	ESPNL	339768
492794	2q	240041845	240042154	310	6	0.167	Downstream(2-3kb)	3918	OR6B3	150681
492794	3p	75737230	75739007	1778	9	0.556	Promoter(<=1kb)	0	MIR4273	100422955
492794	3q	98169021	98169594	574	7	0.571	Exon(exon2of2)	19695	OR5H14	403273
492794	3q	98264413	98265098	686	7	0.571	Promoter(<=1kb)	128	OR5H6	79295
492794	3q	194341177	194342571	1395	6	0.667	Exon(exon2of2)	8747	CPN2	1370
492794	3q	196947388	196948662	1275	7	0.571	3'UTR	3268	PIGZ	80235
492794	4p	5988383	5989749	1367	7	0.571	Promoter(<=1kb)	0	C4orf50	389197
492794	4p	6300792	6302360	1569	7	0.857	Exon(exon8of8)	6021	WFS1	7466
492794	4p	8227004	8228508	1505	8	0.125	Promoter(<=1kb)	-24	SH3TC1	54436
492794	4q	185458217	185460011	1795	8	0.625	Promoter(<=1kb)	0	CCDC110	256309
492794	4q	186619481	186621601	2121	7	0.286	Exon(exon10of27)	-9411	FAT1	2195
492794	5q	112891825	112893068	1244	6	0.5	3'UTR	27216	SRP19	6728
492794	5q	140807352	140807737	386	6	0.833	Promoter(<=1kb)	271	PCDHA4	56144
492794	5q	141174000	141175025	1026	6	0.833	Promoter(1-2kb)	1356	PCDHB7	56129
492794	5q	141955356	141957660	2305	6	0.667	Promoter(<=1kb)	-668	RNF14	9604
492794	5q	148826877	148828070	1194	6	1	Promoter(1-2kb)	1632	ADRB2	154
492794	6p	46858771	46859502	732	8	0.5	Exon(exon17of21)	3802	ADGRF5	221395
492794	6q	64591274	64591961	688	10	0.5	Exon(exon26of43)	121374	EYS	346007
492794	6q	149888581	149890867	2287	7	0.714	Promoter(<=1kb)	0	RAET1E-AS1	100652739
492794	6q	159231899	159234370	2472	10	0.5	Exon(exon11of23)	13602	FNDC1	84624
492794	7q	100958721	100960873	2153	59	0.475	Promoter(<=1kb)	756	MUC3A	4584
492794	7q	100991195	100993127	1933	8	0.625	Exon(exon5of15)	-19927	MUC12	10071
492794	7q	100995575	100995785	211	6	0.833	Exon(exon5of15)	-17269	MUC12	10071
492794	7q	101036085	101038481	2397	7	0.429	Exon(exon3of12)	-5230	MUC17	140453
492794	8p	10609614	10612307	2694	10	0.9	Exon(exon4of4)	42836	RP1L1	94137
492794	8p	11331234	11332082	849	7	0.571	Promoter(<=1kb)	346	SLC35G5	83650
492794	8p	13021128	13022030	903	7	0.143	Exon(exon5of5)	9115	TRMT9B	57604
492794	9p	39078723	39078846	124	7	0.714	Exon(exon22of24)	7302	CNTNAP3	79937
492794	9q	76709523	76710843	1321	6	0.5	Promoter(<=1kb)	0	PRUNE2	158471
492794	9q	87885490	87888536	3047	9	0.556	Promoter(2-3kb)	2613	SPATA31E1	286234
492794	9q	104504315	104505071	757	6	0.333	Promoter(<=1kb)	52	OR13F1	138805
492794	9q	104598545	104599361	817	12	0.583	Promoter(<=1kb)	52	OR13C5	138799
492794	9q	122553278	122554071	794	6	0.333	Promoter(<=1kb)	108	OR1N2	138882
492794	9q	122749914	122750547	634	6	0.833	Promoter(<=1kb)	174	OR1L6	392390
492794	9q	124855684	124856809	1126	7	0.571	Promoter(2-3kb)	2208	WDR38	401551
492794	9q	133255635	133256205	571	7	1	3'UTR	19009	ABO	28

492794	9q	135484803	135487213	2411	9	0.333	Promoter(1-2kb)	1440	PPP1R26	9858
492794	9q	135547960	135548795	836	8	0.625	Promoter(1-2kb)	1805	OBP2A	29991
492794	10q	46549378	46550723	1346	25	0.64	Exon(exon3of3)	4807	GPRIN2	9721
492794	10q	49323504	49325572	2069	7	0.429	Exon(exon3of3)	24230	C10orf71	118461
492794	10q	128102594	128105201	2608	6	0.667	Promoter(<=1kb)	0	MKI67	4288
492794	11p	244106	244197	92	8	0.5	Promoter(<=1kb)	-232	PSMD13	5719
492794	11p	1194354	1196902	2549	7	0.571	Exon(exon34of49)	-26164	MUC5B	727897
492794	11p	1246095	1247378	1284	8	0.375	Promoter(2-3kb)	2298	MUC5B-AS1	112577518
492794	11p	5177978	5178478	501	6	0.167	Promoter(<=1kb)	186	OR52Z1	283110
492794	11p	5323451	5324256	806	6	0.5	Promoter(<=1kb)	41	OR51B2	79345
492794	11p	5389704	5390350	647	7	0.429	Promoter(<=1kb)	327	OR51M1	390059
492794	11p	5422212	5423123	912	11	0.636	Promoter(<=1kb)	101	OR51Q1	390061
492794	11p	5581045	5581738	694	8	0.375	Promoter(<=1kb)	168	OR52B6	340980
492794	11p	5841302	5841883	582	9	0.333	Promoter(<=1kb)	14	OR52E6	390078
492794	11p	11351961	11352736	776	9	0.222	Promoter(<=1kb)	514	CSNK2A3	283106
492794	11p	12293639	12294842	1204	7	0.714	Exon(exon29of35)	6739	MICALCL	84953
492794	11p	43942293	43943348	1056	9	0.778	Promoter(<=1kb)	0	C11orf96	387763
492794	11q	58214757	58215722	966	8	0.25	Promoter(<=1kb)	12	OR1S1	219959
492794	11q	58402523	58403265	743	8	0.5	Promoter(<=1kb)	144	OR5B3	441608
492794	11q	64116513	64118232	1720	7	0.714	Exon(exon2of2)	8702	MACROD1	28992
492794	11q	64315797	64315856	60	8	0.75	Promoter(1-2kb)	1485	TRMT112	51504
492794	11q	85724687	85725825	1139	6	0.5	Promoter(<=1kb)	0	SYTL2	54843
492794	11q	124015600	124016477	878	7	0.143	Promoter(<=1kb)	25	OR10G4	390264
492794	11q	124264847	124265809	963	6	1	Promoter(<=1kb)	20	OR8G5	219865
492794	11q	130914501	130915409	909	10	0.7	Promoter(1-2kb)	1035	SNX19	399979
492794	12p	4626568	4628549	1982	11	0.455	Exon(exon5of6)	14051	DYRK4	8798
492794	12p	6453119	6453670	552	6	0.667	Promoter(<=1kb)	633	TAPBPL	55080
492794	13q	24434450	24435347	898	7	0.571	Exon(exon31of34)	19787	PARP4	143
492794	13q	25096675	25097182	508	7	0.429	Promoter(<=1kb)	807	PABPC3	5042
492794	13q	102732474	102733933	1460	6	0.333	Exon(exon4of4)	25139	CCDC168	643677
492794	14q	19975713	19976448	736	9	0.444	Promoter(<=1kb)	269	OR4K15	81127
492794	14q	20060048	20060523	476	6	0.5	Promoter(<=1kb)	3	OR4L1	122742
492794	14q	21634137	21634686	550	10	0.5	Promoter(<=1kb)	254	OR10G2	26534
492794	14q	22633879	22634450	572	8	0.375	Exon(exon2of2)	32212	ABHD4	63874
492794	14q	70457532	70458540	1009	9	0.333	Exon(exon2of2)	5358	ADAM21	8747
492794	15q	23439979	23442067	2089	14	0.5	5'UTR	5167	GOLGA6L2	283685
492794	15q	40621642	40623696	2055	6	0.333	Promoter(<=1kb)	0	KNL1	57082
492794	15q	78765975	78766581	607	7	0.429	Promoter(<=1kb)	-862	ADAMTS7	11173
492794	15q	85579423	85582073	2651	15	0.6	Promoter(<=1kb)	-837	AKAP13	11214
492794	15q	88854874	88855594	721	7	0.857	Exon(exon12of18)	7631	ACAN	176
492794	15q	88857108	88859365	2258	6	0	Exon(exon12of18)	9865	ACAN	176
492794	16p	768559	770944	2386	12	0.5	Promoter(<=1kb)	0	MIR662	724032
492794	16p	1228744	1229713	970	8	0.625	Promoter(<=1kb)	449	TPSB2	64499
492794	16q	88428416	88429600	1185	6	0.333	Exon(exon3of3)	-23680	ZFPM1	161882
492794	16q	89100686	89101050	365	9	0.667	Promoter(<=1kb)	24	ACSF3	197322
492794	16q	89226863	89228289	1427	7	0.571	Promoter(2-3kb)	2229	ZNF778	197320
492794	17p	744946	746966	2021	6	1	3'UTR	5072	GEMIN4	50628
492794	17p	2299649	2300159	511	6	0.167	Exon(exon2of19)	-3224	SRR	63826
492794	17p	10638198	10641099	2902	7	0.286	Exon(exon19of41)	-8169	MYH3	4621
492794	17p	21300581	21300978	398	12	0.75	3'UTR	9112	MAP2K3	5606
492794	17p	21415470	21416416	947	6	0.833	Exon(exon3of3)	10334	KCNJ12	3768
492794	17q	76293419	76294016	598	6	0.5	Promoter(2-3kb)	-2167	QRICH2	84074
492794	17q	81510690	81511591	902	7	0.714	Promoter(<=1kb)	257	ACTG1	71
492794	17q	81645135	81645595	461	7	0.429	Promoter(2-3kb)	2722	TSPAN10	83882
492794	18p	11609904	11610509	606	10	0.5	Promoter(<=1kb)	308	SLC35G4	646000
492794	18q	58535186	58538030	2845	19	0.526	Promoter(<=1kb)	0	ALPK2	115701
492794	19p	1004711	1005532	822	7	0.571	Exon(exon3of9)	4292	GRIN3B	116444
492794	19p	4510548	4513547	3000	20	0.45	Exon(exon3of6)	4157	PLIN4	729359
492794	19p	5455600	5456439	840	8	0.625	Promoter(<=1kb)	183	ZNRF4	148066
492794	19p	8937644	8939234	1591	6	0.667	Exon(exon5of84)	-41554	MUC16	94025
492794	19p	8946313	8951868	5556	19	0.632	Exon(exon3of84)	29474	MUC16	94025
492794	19p	8959116	8962299	3184	11	0.727	Exon(exon3of84)	19043	MUC16	94025
492794	19p	8971838	8978096	6259	15	0.533	Exon(exon1of84)	3246	MUC16	94025
492794	19p	12430157	12431840	1684	8	0.375	3'UTR	9181	ZNF443	10224
492794	19p	14766987	14767036	50	6	0.833	Exon(exon6of21)	5338	ADGRE2	30817
492794	19p	15087213	15088040	828	9	0.333	Promoter(<=1kb)	233	OR1I1	126370
492794	19p	17281820	17284246	2427	9	0.556	Promoter(<=1kb)	0	ANKLE1	126549
492794	19p	18264753	18267409	2657	8	0.5	5'UTR	7002	IQCN	80726
492794	19q	37885190	37888806	3617	7	0.429	Exon(exon6of6)	17788	WDR87	83889
492794	19q	39877222	39877880	659	6	0.5	Exon(exon20of28)	9412	FCGBP	8857
492794	19q	40880128	40880622	495	7	0.286	Promoter(<=1kb)	-38	CYP2A7	1549
492794	19q	43913423	43914878	1456	9	0.556	Exon(exon10of10)	4861	ZNF45	7596
492794	19q	44106512	44108078	1567	8	0.125	Exon(exon6of6)	-4103	ZNF225	7768
492794	19q	52437918	52439242	1325	7	0.429	Exon(exon4of4)	6504	ZNF534	147658
492794	19q	53164551	53166239	1689	6	0.167	Exon(exon4of4)	-5476	ZNF347	84671
492794	19q	55911888	55913077	1190	6	0.667	Exon(exon5of12)	19234	NLRP13	126204
492794	19q	58368293	58368875	583	7	0.429	Exon(exon3of3)	-5445	ZNF497	162968

492794	20p	5922421	5923394	974	6	0.5	Exon(exon4of5)	6923	CHGB	1114
492794	20q	63561406	63565531	4126	12	0.667	Promoter(<=1kb)	0	HELZ2	85441
492794	21q	26843740	26844859	1120	6	0.667	Promoter(<=1kb)	0	ADAMTS1	9510
492794	21q	44637476	44638143	668	6	0.5	Promoter(<=1kb)	120	KRTAP10-10	353333
492794	22q	36191154	36191906	753	7	0.714	3'UTR	9971	APOL4	80832
492794	23q	136874183	136874416	234	8	0.75	Promoter(<=1kb)	-201	RBMX	27316
492884	1p	11766028	11768307	2280	6	0.833	Promoter(<=1kb)	0	C1orf167	284498
492884	1p	11778784	11779941	1158	7	0.714	Promoter(<=1kb)	0	C1orf167-AS1	102724659
492884	1p	18481403	18482217	815	6	0.667	Promoter(<=1kb)	421	KLHDC7A	127707
492884	1p	23874604	23875430	827	8	0.5	Exon(exon2of2)	-6310	FUCA1	2517
492884	1p	40067594	40067675	82	6	0	Promoter(<=1kb)	324	CAP1	10487
492884	1p	89186388	89186419	32	9	0.556	Promoter(<=1kb)	107	GBP4	115361
492884	1q	152220079	152221071	993	8	0.375	Exon(exon3of3)	3122	HRNR	388697
492884	1q	152303673	152313891	10219	40	0.6	Promoter(<=1kb)	0	FLG-AS1	339400
492884	1q	156669844	156670886	1043	6	1	Exon(exon4of4)	6521	NES	10763
492884	1q	158765805	158766655	851	6	0.5	Promoter(<=1kb)	47	OR6N1	128372
492884	1q	201206099	201207840	1742	6	0.5	Promoter(1-2kb)	1017	IGFN1	91156
492884	1q	232805117	232806800	1684	6	0.5	Promoter(<=1kb)	225	MAP10	54627
492884	1q	247841312	247841582	271	6	0.833	Promoter(<=1kb)	314	OR11L1	391189
492884	1q	247895625	247896410	786	6	0.5	Promoter(<=1kb)	38	OR2W3	343171
492884	1q	248273309	248273670	362	6	0.333	Promoter(<=1kb)	166	OR2T33	391195
492884	2p	48580657	48582454	1798	7	0.571	Promoter(<=1kb)	0	STON1	11037
492884	2q	102351547	102351902	356	7	0.429	Exon(exon11of11)	-4027	IL18R1	8809
492884	2q	132781078	132784972	3895	11	0.545	Promoter(<=1kb)	0	NCKAP5	344148
492884	2q	167246794	167248478	1685	7	0.714	Promoter(<=1kb)	-204	XIRP2	129446
492884	2q	184936178	184937636	1459	6	0.333	Exon(exon4of4)	69813	ZNF804A	91752
492884	2q	185790999	185794632	3634	9	0.778	Promoter(<=1kb)	0	FSIP2	401024
492884	2q	185805377	185808170	2794	7	0.286	Promoter(<=1kb)	0	FSIP2	401024
492884	2q	217847567	217848213	647	6	0.833	Exon(exon19of33)	-5407	TNS1	7145
492884	2q	233713134	233713664	531	8	0.75	Promoter(<=1kb)	142	UGT1A5	54579
492884	2q	238130416	238131546	1131	6	0.333	Promoter(1-2kb)	1468	ESPNL	339768
492884	2q	240041845	240042131	287	6	0.167	Downstream(2-3kb)	3941	OR6B3	150681
492884	3p	75736929	75738859	1931	10	0.6	Promoter(<=1kb)	0	MIR4273	100422955
492884	3q	98264413	98265098	686	7	0.571	Promoter(<=1kb)	128	OR5H6	79295
492884	4p	5988383	5989749	1367	7	0.571	Promoter(<=1kb)	0	C4orf50	389197
492884	4p	6300792	6302360	1569	6	0.833	Exon(exon8of8)	6021	WFS1	7466
492884	4p	8227004	8228508	1505	8	0.125	Promoter(<=1kb)	-24	SH3TC1	54436
492884	5p	795818	796237	420	6	0.667	3'UTR	4908	ZDHHC11	79844
492884	5q	83537326	83540746	3421	7	0.286	Promoter(1-2kb)	-1202	VCAN	1462
492884	5q	112891825	112893068	1244	6	0.5	3'UTR	27216	SRP19	6728
492884	5q	140848579	140850786	2208	6	0.5	Promoter(<=1kb)	807	PCDHA9	9752
492884	5q	141150948	141152171	1224	6	0.667	Promoter(<=1kb)	846	PCDHB6	56130
492884	5q	141174000	141175025	1026	7	0.714	Promoter(1-2kb)	1356	PCDHB7	56129
492884	6p	1312843	1313745	903	6	0.5	Promoter(<=1kb)	745	FOXQ1	94234
492884	6p	42745312	42746041	730	6	0.667	Promoter(<=1kb)	62	TBCC	6903
492884	6p	46858771	46859502	732	7	0.571	Exon(exon17of21)	3802	ADGRF5	221395
492884	6q	64591274	64591961	688	10	0.5	Exon(exon26of43)	121374	EYS	346007
492884	6q	149888581	149890867	2287	7	0.714	Promoter(<=1kb)	0	RAET1E-AS1	100652739
492884	6q	159231899	159234370	2472	12	0.583	Exon(exon11of23)	13602	FNDC1	84624
492884	7p	5063955	5065577	1623	6	0.833	Exon(exon6of6)	-6548	RBAKDN	389458
492884	7p	6330446	6330944	499	6	1	Exon(exon2of2)	7749	FAM220A	84792
492884	7p	53035678	53036385	708	7	1	Promoter(<=1kb)	45	POM121L12	285877
492884	7q	100958977	100960873	1897	50	0.46	Promoter(1-2kb)	1012	MUC3A	4584
492884	7q	100991195	100994057	2863	11	0.727	Exon(exon5of15)	-18997	MUC12	10071
492884	7q	100995547	100995785	239	7	0.857	Exon(exon5of15)	-17269	MUC12	10071
492884	7q	101003685	101004836	1152	6	0.667	Exon(exon5of15)	-8218	MUC12	10071
492884	7q	149818015	149819792	1778	6	0.667	Promoter(2-3kb)	-2352	SSPO	23145
492884	7q	149824094	149826695	2602	7	0.714	Promoter(<=1kb)	0	SSPO	23145
492884	8p	10609614	10612986	3373	7	1	Exon(exon4of4)	42157	RP1L1	94137
492884	8p	11331194	11332082	889	8	0.5	Promoter(<=1kb)	306	SLC35G5	83650
492884	8p	13021128	13022030	903	8	0.125	Exon(exon5of5)	9115	TRMT9B	57604
492884	8q	138151949	138153046	1098	6	0.667	Promoter(<=1kb)	0	FAM135B	51059
492884	9q	76175241	76175296	56	8	0.875	Exon(exon14of14)	-13343	PCSK5	5125
492884	9q	76705724	76707804	2081	6	0.5	Promoter(<=1kb)	666	PCA3	50652
492884	9q	104598641	104599361	721	8	0.625	Promoter(<=1kb)	52	OR13C5	138799
492884	9q	122553263	122554071	809	7	0.429	Promoter(<=1kb)	93	OR1N2	138882
492884	9q	122628595	122629398	804	6	0.333	Promoter(<=1kb)	175	OR1B1	347169
492884	9q	122749914	122750547	634	6	0.833	Promoter(<=1kb)	174	OR1L6	392390
492884	9q	124855684	124856809	1126	7	0.571	Promoter(2-3kb)	2208	WDR38	401551
492884	9q	131474936	131476383	1448	6	0.833	Promoter(<=1kb)	0	PRRC2B	84726
492884	9q	135484803	135487213	2411	9	0.333	Promoter(1-2kb)	1440	PPP1R26	9858
492884	10q	46549378	46550723	1346	25	0.64	Exon(exon3of3)	4807	GPRIN2	9721
492884	10q	49322575	49326319	3745	10	0.5	Exon(exon3of3)	23301	C10orf71	118461
492884	10q	89737450	89738561	1112	6	0	Exon(exon20of33)	13874	KIF20B	9585
492884	10q	128103129	128106213	3085	12	0.667	Promoter(<=1kb)	-1	MKI67	4288
492884	11p	244106	244197	92	8	0.5	Promoter(<=1kb)	-232	PSMD13	5719
492884	11p	1194354	1196902	2549	7	0.571	Exon(exon34of49)	-26164	MUC5B	727897

492884	11p	1246095	1247378	1284	7	0.286	Promoter(2-3kb)	2298	MUC5B-AS1	112577518
492884	11p	5177978	5178478	501	6	0.167	Promoter(<=1kb)	186	OR52Z1	283110
492884	11p	5323451	5324256	806	7	0.571	Promoter(<=1kb)	41	OR51B2	79345
492884	11p	5422212	5423123	912	11	0.636	Promoter(<=1kb)	101	OR51Q1	390061
492884	11p	5515185	5515931	747	6	0.333	Promoter(<=1kb)	768	UBQLNL	143630
492884	11p	5581045	5581738	694	8	0.375	Promoter(<=1kb)	168	OR52B6	340980
492884	11p	5841302	5841883	582	9	0.333	Promoter(<=1kb)	14	OR52E6	390078
492884	11p	11351961	11352736	776	9	0.222	Promoter(<=1kb)	514	CSNK2A3	283106
492884	11p	12293639	12294842	1204	7	0.714	Exon(exon29of35)	6739	MICALCL	84953
492884	11p	43942293	43943348	1056	9	0.778	Promoter(<=1kb)	0	C11orf96	387763
492884	11q	58214757	58215722	966	9	0.222	Promoter(<=1kb)	12	OR1S1	219959
492884	11q	58402523	58403265	743	8	0.5	Promoter(<=1kb)	144	OR5B3	441608
492884	11q	82732630	82733184	555	6	0.833	Promoter(<=1kb)	680	FAM181B	220382
492884	11q	85724687	85725825	1139	6	0.5	Promoter(<=1kb)	0	SYTL2	54843
492884	11q	123906790	123907324	535	6	0.667	Promoter(<=1kb)	644	OR8D4	338662
492884	11q	124038366	124038988	623	7	1	Promoter(<=1kb)	13	OR10G7	390265
492884	12p	4626568	4628549	1982	11	0.455	Exon(exon5of6)	14051	DYRK4	8798
492884	12p	6453119	6453670	552	6	0.667	Promoter(<=1kb)	633	TAPBPL	55080
492884	12p	8222174	8223514	1341	6	0.667	Exon(exon5of6)	4073	FAM90A1	55138
492884	13q	102732424	102733933	1510	7	0.429	Exon(exon4of4)	25139	CCDC168	643677
492884	14q	20060048	20060884	837	8	0.625	Promoter(<=1kb)	3	OR4L1	122742
492884	14q	70457532	70458540	1009	11	0.273	Exon(exon2of2)	5358	ADAM21	8747
492884	14q	104175275	104177810	2536	10	0.3	Exon(exon12of15)	36235	KIF26A	26153
492884	14q	104939262	104942618	3357	12	0.167	5'UTR	7102	PLD4	122618
492884	14q	104943622	104953878	10257	55	0.545	Promoter(1-2kb)	1524	AHNAK2	113146
492884	15q	23440160	23442067	1908	7	0.571	5'UTR	5167	GOLGA6L2	283685
492884	15q	85579423	85582073	2651	15	0.6	Promoter(<=1kb)	-837	AKAP13	11214
492884	15q	100569472	100570097	626	6	0.833	Promoter(<=1kb)	534	LINS1	55180
492884	16p	669592	672548	2957	6	0.5	Promoter(<=1kb)	0	RHOT2	89941
492884	16p	1228744	1229622	879	6	0.833	Promoter(<=1kb)	540	TPSB2	64499
492884	16p	1486371	1488463	2093	8	0.75	Promoter(<=1kb)	4	PTX4	390667
492884	16q	74391401	74392004	604	10	0.7	Exon(exon7of7)	13523	NPIPB15	440348
492884	16q	88428539	88429600	1062	6	0.333	Exon(exon3of3)	-23680	ZFPM1	161882
492884	16q	88430623	88431977	1355	6	0.5	Exon(exon3of3)	-21303	ZFPM1	161882
492884	16q	89100686	89101050	365	7	0.571	Promoter(<=1kb)	24	ACSF3	197322
492884	16q	89226863	89228390	1528	10	0.6	Promoter(2-3kb)	2229	ZNF778	197320
492884	17p	21300581	21300978	398	11	0.727	3'UTR	9112	MAP2K3	5606
492884	17p	21415470	21416404	935	6	1	Exon(exon3of3)	10334	KCNJ12	3768
492884	18p	11609904	11610469	566	8	0.625	Promoter(<=1kb)	308	SLC35G4	646000
492884	18q	58535186	58537515	2330	9	0.333	Promoter(<=1kb)	0	ALPK2	115701
492884	19p	1036457	1036914	458	6	0.5	Exon(exon6of7)	-3187	ABCA7	10347
492884	19p	4510548	4513547	3000	15	0.467	Exon(exon3of6)	4157	PLIN4	729359
492884	19p	5455600	5456439	840	6	0.5	Promoter(<=1kb)	183	ZNRF4	148066
492884	19p	8937644	8939234	1591	6	0.833	Exon(exon5of84)	-41554	MUC16	94025
492884	19p	8946306	8951868	5563	19	0.684	Exon(exon3of84)	29474	MUC16	94025
492884	19p	8959116	8962299	3184	12	0.583	Exon(exon3of84)	19043	MUC16	94025
492884	19p	8963397	8967127	3731	21	0.667	Exon(exon3of84)	14215	MUC16	94025
492884	19p	8972751	8978096	5346	13	0.462	Exon(exon1of84)	3246	MUC16	94025
492884	19p	12075333	12077046	1714	7	0.286	Promoter(<=1kb)	6	ZNF788P	388507
492884	19p	15794192	15794719	528	6	0.333	Promoter(<=1kb)	241	OR10H5	284433
492884	19p	21971930	21974500	2571	7	0.714	Exon(exon4of4)	14408	ZNF208	7757
492884	19p	23743906	23745300	1395	6	0.333	Exon(exon4of4)	13537	ZNF681	148213
492884	19q	34943334	34944685	1352	6	0.5	3'UTR	9723	ZNF30	90075
492884	19q	36996730	36997597	868	10	0.6	Exon(exon10of10)	5677	ZNF568	374900
492884	19q	39877222	39877880	659	6	0.5	Exon(exon20of28)	9412	FCGBP	8857
492884	19q	39886240	39886422	183	6	0.667	Promoter(<=1kb)	870	FCGBP	8857
492884	19q	43913423	43914878	1456	7	0.429	Exon(exon10of10)	4861	ZNF45	7596
492884	19q	43966037	43967171	1135	6	0.167	Promoter(<=1kb)	-691	ZNF155	7711
492884	19q	43996326	43997366	1041	6	0.5	Exon(exon5of5)	5419	LOC10192806[illegible]	101928063
492884	19q	44106512	44108078	1567	7	0	Exon(exon6of6)	-4103	ZNF225	7768
492884	19q	48873325	48875925	2601	10	0.5	Promoter(<=1kb)	904	PPP1R15A	23645
492884	19q	52437918	52439242	1325	7	0.429	Exon(exon4of4)	6504	ZNF534	147658
492884	19q	53108623	53109736	1114	6	0.167	5'UTR	-5187	ZNF160	90338
492884	19q	55911888	55913077	1190	6	0.667	Exon(exon5of12)	19234	NLRP13	126204
492884	20p	5922421	5923394	974	6	0.5	Exon(exon4of5)	6923	CHGB	1114
492884	20q	63349752	63350772	1021	6	0.5	3'UTR	3794	CHRNA4	1137
492884	20q	63561666	63565531	3866	12	0.667	Promoter(<=1kb)	-61	HELZ2	85441
492884	21q	26843740	26844859	1120	6	0.667	Promoter(<=1kb)	0	ADAMTS1	9510
492884	21q	44558207	44558709	503	7	0.571	Promoter(<=1kb)	86	KRTAP10-3	386682
492884	22q	22352950	22353380	431	16	0.5	Exon(exon1of2)	30478	BMS1P20	96610
492884	22q	36191154	36191906	753	6	0.667	3'UTR	9971	APOL4	80832
492884	22q	49883704	49884994	1291	6	0.167	Exon(exon2of2)	22924	ALG12	79087
492884	23p	35802148	35803010	863	7	0.571	5'UTR	3357	MAGEB16	139604
493359	1p	12847526	12847995	470	9	0.444	Promoter(<=1kb)	730	HNRNPCL1	343069
493359	1p	12859036	12860079	1044	6	0.333	Promoter(1-2kb)	1950	PRAMEF2	65122
493359	1p	13369166	13369564	399	9	0.778	Promoter(2-3kb)	2336	PRAMEF19	645414
493359	1p	13370686	13371119	434	7	0.429	Promoter(<=1kb)	781	PRAMEF19	645414

493359	1p	16058491	16060000	1510	10	0.9	Exon(exon5of7)	6168	CLCNKB	1188
493359	1p	23874604	23875430	827	8	0.5	Exon(exon2of2)	-6310	FUCA1	2517
493359	1p	40067594	40067675	82	6	0	Promoter(<=1kb)	324	CAP1	10487
493359	1p	74470690	74472144	1455	6	0.333	Exon(exon5of5)	31625	FPGT-TNNI3K	100526835
493359	1q	145872200	145873647	1448	7	0.571	Exon(exon8of12)	12219	ANKRD35	148741
493359	1q	152213286	152213347	62	8	0.5	Exon(exon3of3)	10846	HRNR	388697
493359	1q	152218569	152221375	2807	17	0.765	Promoter(2-3kb)	2818	HRNR	388697
493359	1q	169542317	169542882	566	6	0.167	Exon(exon13of25)	-26572	F5	2153
493359	1q	214640144	214642954	2811	12	0.5	Exon(exon12of20)	-5013	CENPF	1063
493359	1q	214644872	214647181	2310	10	0.4	Promoter(<=1kb)	-786	CENPF	1063
493359	1q	228315976	228318026	2051	6	0.5	Exon(exon50of81)	6492	OBSCN	84033
493359	1q	247841312	247841582	271	6	0.833	Promoter(<=1kb)	314	OR11L1	391189
493359	1q	247949325	247949738	414	9	0.333	Promoter(<=1kb)	467	OR2L8	391190
493359	2q	132783061	132784972	1912	8	0.5	Promoter(1-2kb)	-1038	NCKAP5	344148
493359	2q	167246794	167248478	1685	7	0.714	Promoter(<=1kb)	-204	XIRP2	129446
493359	2q	184936178	184937636	1459	7	0.429	Exon(exon4of4)	69813	ZNF804A	91752
493359	2q	185789865	185794632	4768	10	0.8	Promoter(<=1kb)	0	FSIP2	401024
493359	2q	185805377	185808170	2794	6	0.333	Promoter(<=1kb)	0	FSIP2	401024
493359	2q	217847583	217848559	977	6	0.833	Exon(exon19of33)	-5423	TNS1	7145
493359	2q	233713134	233713664	531	8	0.75	Promoter(<=1kb)	142	UGT1A5	54579
493359	2q	238130271	238131702	1432	11	0.545	Promoter(1-2kb)	1323	ESPNL	339768
493359	2q	240041845	240042154	310	6	0.167	Downstream(2-3kb)	3918	OR6B3	150681
493359	3p	31989532	31990905	1374	6	0.333	Exon(exon2of2)	7761	ZNF860	344787
493359	3p	75736880	75739243	2364	81	0.642	Promoter(<=1kb)	0	MIR4273	100422955
493359	3q	98264413	98265098	686	7	0.571	Promoter(<=1kb)	128	OR5H6	79295
493359	3q	194341097	194342571	1475	7	0.714	Exon(exon2of2)	8747	CPN2	1370
493359	3q	194359607	194360906	1300	9	0.889	Exon(exon2of2)	-8279	CPN2	1370
493359	4p	5988383	5989749	1367	7	0.571	Promoter(<=1kb)	0	C4orf50	389197
493359	4p	6300792	6302360	1569	7	0.857	Exon(exon8of8)	6021	WFS1	7466
493359	4q	112431241	112432293	1053	7	0.429	3'UTR	4735	ALPK1	80216
493359	4q	154489498	154491312	1815	9	0.556	Promoter(<=1kb)	22	DCHS2	54798
493359	4q	185458217	185460011	1795	8	0.625	Promoter(<=1kb)	0	CCDC110	256309
493359	5q	140807352	140807737	386	6	0.833	Promoter(<=1kb)	271	PCDHA4	56144
493359	5q	141122180	141123655	1476	6	0.833	Promoter(<=1kb)	362	PCDHB4	56131
493359	5q	141150948	141152584	1637	6	0.833	Promoter(<=1kb)	846	PCDHB6	56130
493359	5q	141172977	141175025	2049	11	0.818	Promoter(<=1kb)	333	PCDHB7	56129
493359	5q	141178745	141180333	1589	8	0.75	Promoter(<=1kb)	955	PCDHB8	56128
493359	5q	141183598	141184688	1091	7	0.857	Promoter(2-3kb)	2199	PCDHB16	57717
493359	5q	141187690	141189425	1736	8	0.25	Promoter(<=1kb)	529	PCDHB9	56127
493359	5q	141246961	141247287	327	6	0.667	Promoter(1-2kb)	1566	PCDHB15	56121
493359	5q	151565922	151568158	2237	9	0.778	Promoter(<=1kb)	786	FAT2	2196
493359	6p	16326440	16327684	1245	7	0.429	Exon(exon8of9)	36495	GMPR	2766
493359	6p	42745312	42746041	730	6	0.667	Promoter(<=1kb)	62	TBCC	6903
493359	6p	46858771	46859502	732	8	0.5	Exon(exon17of21)	3802	ADGRF5	221395
493359	6q	106511549	106512572	1024	6	0.667	Promoter(<=1kb)	0	CRYBG1	202
493359	6q	149888581	149890867	2287	7	0.714	Promoter(<=1kb)	0	RAET1E-AS1	100652739
493359	6q	159231899	159234370	2472	12	0.583	Exon(exon11of23)	13602	FNDC1	84624
493359	7p	45082865	45084866	2002	6	0.333	Promoter(1-2kb)	-1465	NACAD	23148
493359	7p	56021087	56021209	123	6	0.5	Exon(exon2of7)	12947	PSPH	5723
493359	7q	100958977	100960873	1897	51	0.471	Promoter(1-2kb)	1012	MUC3A	4584
493359	7q	100995547	100995785	239	6	0.833	Exon(exon5of15)	-17269	MUC12	10071
493359	8p	10609614	10612307	2694	9	0.889	Exon(exon4of4)	42836	RP1L1	94137
493359	8p	13021128	13022030	903	8	0.125	Exon(exon5of5)	9115	TRMT9B	57604
493359	8q	123651655	123652634	980	7	0.571	Promoter(<=1kb)	316	KLHL38	340359
493359	8q	142664633	142666038	1406	6	0.833	Exon(exon2of2)	3932	JRK	8629
493359	8q	143916360	143919209	2850	7	0.143	Exon(exon32of32)	20381	PLEC	5339
493359	8q	143921326	143924022	2697	10	0.7	Exon(exon31of32)	15568	PLEC	5339
493359	9q	76175237	76175300	64	6	0.5	Exon(exon14of14)	-13339	PCSK5	5125
493359	9q	76703555	76706360	2806	8	0.5	Promoter(<=1kb)	0	PCA3	50652
493359	9q	104598545	104599318	774	6	0.333	Promoter(<=1kb)	95	OR13C5	138799
493359	9q	122553263	122554071	809	7	0.429	Promoter(<=1kb)	93	OR1N2	138882
493359	9q	122628595	122629398	804	7	0.286	Promoter(<=1kb)	175	OR1B1	347169
493359	9q	122749914	122750547	634	6	0.833	Promoter(<=1kb)	174	OR1L6	392390
493359	9q	133255635	133256264	630	8	1	3'UTR	18950	ABO	28
493359	9q	135484695	135487213	2519	9	0.333	Promoter(1-2kb)	1332	PPP1R26	9858
493359	9q	135547960	135548795	836	6	0.667	Promoter(1-2kb)	1805	OBP2A	29991
493359	10q	46549378	46550723	1346	26	0.654	Exon(exon3of3)	4807	GPRIN2	9721
493359	10q	49323169	49326817	3649	11	0.545	Exon(exon3of3)	23895	C10orf71	118461
493359	10q	128103129	128104830	1702	10	0.6	Promoter(<=1kb)	-1	MKI67	4288
493359	11p	1245466	1247378	1913	8	0.375	Promoter(2-3kb)	2298	MUC5B-AS1	112577518
493359	11p	5046754	5047432	679	7	0.571	Promoter(<=1kb)	228	OR52J3	119679
493359	11p	5177978	5178478	501	6	0.167	Promoter(<=1kb)	186	OR52Z1	283110
493359	11p	5323451	5324256	806	7	0.571	Promoter(<=1kb)	41	OR51B2	79345
493359	11p	5422212	5423123	912	12	0.667	Promoter(<=1kb)	101	OR51Q1	390061
493359	11p	5440761	5441472	712	6	0.833	Promoter(<=1kb)	42	OR51I1	390063
493359	11p	5581045	5581738	694	8	0.375	Promoter(<=1kb)	168	OR52B6	340980
493359	11p	5841302	5841883	582	9	0.333	Promoter(<=1kb)	14	OR52E6	390078

493359	11p	5884818	5885061	244	7	0.429	Promoter(<=1kb)	547	OR52E4	390081
493359	11p	11351961	11352736	776	9	0.222	Promoter(<=1kb)	514	CSNK2A3	283106
493359	11p	12293639	12294538	900	8	0.625	Exon(exon29of35)	6739	MICALCL	84953
493359	11q	55827536	55827640	105	6	0.667	Promoter(<=1kb)	317	OR5L2	26338
493359	11q	58402523	58403265	743	8	0.5	Promoter(<=1kb)	144	OR5B3	441608
493359	11q	64116513	64118232	1720	6	0.667	Exon(exon2of2)	8702	MACROD1	28992
493359	11q	85724687	85725825	1139	6	0.5	Promoter(<=1kb)	0	SYTL2	54843
493359	11q	124015600	124016477	878	7	0.143	Promoter(<=1kb)	25	OR10G4	390264
493359	11q	124185670	124186080	411	6	0.333	Promoter(<=1kb)	454	OR10D3	26497
493359	12p	4627329	4628549	1221	8	0.625	Exon(exon5of6)	14812	DYRK4	8798
493359	12q	52316096	52317765	1670	6	0.667	Exon(exon4of9)	3633	KRT83	3889
493359	13q	25096659	25097182	524	16	0.5	Promoter(<=1kb)	791	PABPC3	5042
493359	13q	102732474	102733933	1460	6	0.333	Exon(exon4of4)	25139	CCDC168	643677
493359	13q	102743469	102745852	2384	6	0.5	Exon(exon1of2)	13220	CCDC168	643677
493359	14q	19975713	19976448	736	9	0.444	Promoter(<=1kb)	269	OR4K15	81127
493359	14q	20060048	20060884	837	8	0.625	Promoter(<=1kb)	3	OR4L1	122742
493359	14q	20640982	20641567	586	6	0.5	Promoter(<=1kb)	124	OR6S1	341799
493359	14q	21634137	21634589	453	9	0.556	Promoter(<=1kb)	351	OR10G2	26534
493359	14q	70457520	70458540	1021	14	0.357	Exon(exon2of2)	5346	ADAM21	8747
493359	14q	94587512	94587839	328	6	0.5	Exon(exon2of2)	-4219	SERPINA3	12
493359	14q	104175275	104177810	2536	9	0.222	Exon(exon12of15)	36235	KIF26A	26153
493359	14q	104939262	104942618	3357	15	0.267	5'UTR	7102	PLD4	122618
493359	14q	104943622	104953878	10257	57	0.614	Promoter(1-2kb)	1524	AHNAK2	113146
493359	15q	23440081	23442067	1987	12	0.5	5'UTR	5167	GOLGA6L2	283685
493359	15q	73702465	73703760	1296	6	0.833	Promoter(<=1kb)	-149	CD276	80381
493359	15q	78766033	78766626	594	7	0.571	Promoter(<=1kb)	-920	ADAMTS7	11173
493359	15q	85579423	85582016	2594	15	0.6	Promoter(<=1kb)	-894	AKAP13	11214
493359	15q	88854874	88855594	721	6	0.833	Exon(exon12of18)	7631	ACAN	176
493359	15q	88857108	88859365	2258	6	0	Exon(exon12of18)	9865	ACAN	176
493359	15q	99129423	99132517	3095	10	0.5	Exon(exon4of5)	7225	TTC23	64927
493359	16p	1486371	1488463	2093	8	0.75	Promoter(<=1kb)	4	PTX4	390667
493359	16p	28495551	28497395	1845	6	0.333	Promoter(<=1kb)	0	CLN3	1201
493359	16q	74391401	74391897	497	9	0.778	Exon(exon7of7)	13523	NPIPB15	440348
493359	16q	84195142	84196894	1753	6	0.667	Promoter(<=1kb)	0	ADAD2	161931
493359	16q	88428339	88429600	1262	7	0.429	Exon(exon3of3)	-23680	ZFPM1	161882
493359	16q	88714632	88717113	2482	7	0.429	Promoter(<=1kb)	0	MIR4722	100616167
493359	16q	89226863	89228289	1427	8	0.625	Promoter(2-3kb)	2229	ZNF778	197320
493359	17p	2299649	2300159	511	6	0.167	Exon(exon2of19)	-3224	SRR	63826
493359	17p	21300581	21300954	374	8	0.75	3'UTR	9112	MAP2K3	5606
493359	17p	21415470	21416404	935	11	0.818	Exon(exon3of3)	10334	KCNJ12	3768
493359	17q	76293419	76294016	598	6	0.5	Promoter(2-3kb)	-2167	QRICH2	84074
493359	17q	81645135	81645607	473	7	0.286	Promoter(2-3kb)	2722	TSPAN10	83882
493359	18p	11609646	11610469	824	15	0.8	Promoter(<=1kb)	50	SLC35G4	646000
493359	18p	14542649	14543140	492	6	0.5	Promoter(<=1kb)	6	POTEC	388468
493359	18q	58535186	58537515	2330	9	0.333	Promoter(<=1kb)	0	ALPK2	115701
493359	19p	4510711	4513547	2837	14	0.429	Exon(exon3of6)	4157	PLIN4	729359
493359	19p	8948231	8953259	5029	16	0.562	Exon(exon3of84)	28083	MUC16	94025
493359	19p	8959403	8962066	2664	8	0.5	Exon(exon3of84)	19276	MUC16	94025
493359	19p	8963283	8967127	3845	23	0.652	Exon(exon3of84)	14215	MUC16	94025
493359	19p	17281820	17284246	2427	9	0.556	Promoter(<=1kb)	0	ANKLE1	126549
493359	19p	18264798	18267409	2612	12	0.583	5'UTR	7002	IQCN	80726
493359	19p	21971930	21974500	2571	7	0.714	Exon(exon4of4)	14408	ZNF208	7757
493359	19q	36996730	36997597	868	10	0.7	Exon(exon10of10)	5677	ZNF568	374900
493359	19q	37151928	37153149	1222	6	0.833	Exon(exon5of5)	19287	ZNF585A	199704
493359	19q	37885190	37888806	3617	8	0.625	Exon(exon6of6)	17788	WDR87	83889
493359	19q	43913423	43914878	1456	8	0.5	Exon(exon10of10)	4861	ZNF45	7596
493359	19q	43966037	43967171	1135	6	0.167	Promoter(<=1kb)	-691	ZNF155	7711
493359	19q	44106512	44108078	1567	7	0	Exon(exon6of6)	-4103	ZNF225	7768
493359	19q	52437918	52439242	1325	7	0.429	Exon(exon4of4)	6504	ZNF534	147658
493359	19q	53164551	53166239	1689	6	0.167	Exon(exon4of4)	-5476	ZNF347	84671
493359	19q	55517821	55518176	356	11	0.818	Exon(exon14of14)	18127	SBK2	646643
493359	19q	55911888	55913077	1190	6	0.667	Exon(exon5of12)	19234	NLRP13	126204
493359	20p	5922421	5923643	1223	8	0.625	Exon(exon4of5)	6923	CHGB	1114
493359	20q	63349752	63350772	1021	6	0.5	3'UTR	3794	CHRNA4	1137
493359	20q	63561666	63565531	3866	12	0.667	Promoter(<=1kb)	-61	HELZ2	85441
493359	21q	26843740	26844859	1120	6	0.667	Promoter(<=1kb)	0	ADAMTS1	9510
493359	21q	44558207	44558709	503	7	0.286	Promoter(<=1kb)	86	KRTAP10-3	386682
493359	22q	22352950	22353380	431	16	0.5	Exon(exon1of2)	30478	BMS1P20	96610
493359	22q	36191154	36191906	753	7	0.714	3'UTR	9971	APOL4	80832
493359	23p	8170039	8170141	103	6	0.5	Promoter(1-2kb)	1126	VCX2	51480
493359	23p	35802148	35803010	863	7	0.571	5'UTR	3357	MAGEB16	139604
493359	23q	136874183	136874416	234	8	0.75	Promoter(<=1kb)	-201	RBMX	27316
493467	1p	12847526	12847780	255	8	0.375	Promoter(<=1kb)	945	HNRNPCL1	343069
493467	1p	12859036	12860079	1044	6	0.333	Promoter(1-2kb)	1950	PRAMEF2	65122
493467	1p	12893249	12893472	224	6	0.5	Exon(exon4of4)	4798	PRAMEF10	343071
493467	1p	13369166	13369564	399	7	0.714	Promoter(2-3kb)	2336	PRAMEF19	645414
493467	1p	13370686	13371119	434	8	0.375	Promoter(<=1kb)	781	PRAMEF19	645414

493467	1p	16048038	16049824	1787	6	0.5	Promoter(<=1kb)	0	CLCNKB	1188
493467	1p	16058491	16060000	1510	10	0.9	Exon(exon5of7)	6168	CLCNKB	1188
493467	1p	18481403	18482217	815	6	0.667	Promoter(<=1kb)	421	KLHDC7A	127707
493467	1p	23874604	23875430	827	8	0.5	Exon(exon2of2)	-6310	FUCA1	2517
493467	1p	40067594	40067675	82	6	0	Promoter(<=1kb)	324	CAP1	10487
493467	1p	62273232	62275080	1849	8	0.5	Promoter(<=1kb)	-441	KANK4	163782
493467	1p	89186388	89186419	32	9	0.556	Promoter(<=1kb)	107	GBP4	115361
493467	1q	152213286	152213406	121	9	0.556	Exon(exon3of3)	10787	HRNR	388697
493467	1q	152219233	152221375	2143	15	0.733	Promoter(2-3kb)	2818	HRNR	388697
493467	1q	156669844	156670886	1043	6	1	Exon(exon4of4)	6521	NES	10763
493467	1q	201206099	201209342	3244	10	0.7	Promoter(1-2kb)	1017	IGFN1	91156
493467	1q	201210956	201212866	1911	6	0.333	Promoter(<=1kb)	0	IGFN1	91156
493467	1q	228315976	228318049	2074	10	0.7	Exon(exon50of81)	6492	OBSCN	84033
493467	1q	247841312	247841582	271	6	0.833	Promoter(<=1kb)	314	OR11L1	391189
493467	1q	247949325	247949738	414	10	0.3	Promoter(<=1kb)	467	OR2L8	391190
493467	1q	248294677	248295415	739	6	0.667	Promoter(<=1kb)	185	OR2T12	127064
493467	1q	248681658	248682198	541	7	0.571	Promoter(<=1kb)	130	OR14I1	401994
493467	2q	130914528	130916712	2185	6	0.333	Promoter(<=1kb)	0	ARHGEF4	50649
493467	2q	167246794	167248478	1685	7	0.714	Promoter(<=1kb)	-204	XIRP2	129446
493467	2q	185790999	185794632	3634	8	0.875	Promoter(<=1kb)	0	FSIP2	401024
493467	2q	217847583	217848559	977	6	0.833	Exon(exon19of33)	-5423	TNS1	7145
493467	2q	233840612	233842185	1574	6	0.5	Promoter(<=1kb)	0	HJURP	55355
493467	2q	238130416	238131546	1131	6	0.333	Promoter(1-2kb)	1468	ESPNL	339768
493467	2q	240041845	240042154	310	6	0.167	Downstream(2-3kb)	3918	OR6B3	150681
493467	3p	31989532	31990905	1374	8	0.5	Exon(exon2of2)	7761	ZNF860	344787
493467	3p	75736929	75739007	2079	15	0.6	Promoter(<=1kb)	0	MIR4273	100422955
493467	3q	98264413	98265098	686	7	0.571	Promoter(<=1kb)	128	OR5H6	79295
493467	4p	5988383	5989749	1367	7	0.571	Promoter(<=1kb)	0	C4orf50	389197
493467	4p	6300792	6302360	1569	8	0.75	Exon(exon8of8)	6021	WFS1	7466
493467	4p	8227004	8228508	1505	7	0.143	Promoter(<=1kb)	-24	SH3TC1	54436
493467	4p	10443803	10446224	2422	6	0.333	Exon(exon3of3)	10952	ZNF518B	85460
493467	4q	185458217	185460011	1795	9	0.556	Promoter(<=1kb)	0	CCDC110	256309
493467	4q	186617117	186621582	4466	12	0.417	Exon(exon10of27)	-7047	FAT1	2195
493467	4q	186706638	186708616	1979	6	0.667	Exon(exon2of27)	15217	FAT1	2195
493467	5p	795818	796237	420	7	0.714	3'UTR	4908	ZDHHC11	79844
493467	5q	83537326	83539905	2580	7	0.286	Promoter(1-2kb)	1712	VCAN	1462
493467	5q	140848579	140850786	2208	6	0.5	Promoter(<=1kb)	807	PCDHA9	9752
493467	5q	141100771	141102758	1988	10	0.3	Promoter(<=1kb)	298	PCDHB3	56132
493467	5q	141122595	141123949	1355	7	0.714	Promoter(<=1kb)	777	PCDHB4	56131
493467	5q	141174000	141175025	1026	7	0.714	Promoter(1-2kb)	1356	PCDHB7	56129
493467	5q	141178631	141180333	1703	6	0.833	Promoter(<=1kb)	841	PCDHB8	56128
493467	5q	141183999	141184688	690	6	0.833	Promoter(2-3kb)	-2473	PCDHB9	56127
493467	5q	141187109	141189425	2317	7	0.571	Promoter(<=1kb)	0	PCDHB9	56127
493467	5q	141955676	141957660	1985	6	0.667	Promoter(<=1kb)	-668	RNF14	9604
493467	5q	151565922	151568158	2237	9	0.778	Promoter(<=1kb)	786	FAT2	2196
493467	6p	26370344	26370479	136	6	0.5	Promoter(<=1kb)	0	BTN3A2	11118
493467	6p	46858771	46859502	732	7	0.571	Exon(exon17of21)	3802	ADGRF5	221395
493467	6q	149888581	149890867	2287	7	0.714	Promoter(<=1kb)	0	RAET1E-AS1	100652739
493467	6q	159233455	159234370	916	10	0.5	Exon(exon11of23)	15158	FNDC1	84624
493467	7p	12369637	12370736	1100	6	0.667	3'UTR	-13307	VWDE	221806
493467	7p	45082725	45084866	2142	7	0.429	Promoter(1-2kb)	-1325	NACAD	23148
493467	7q	100958721	100960873	2153	59	0.441	Promoter(<=1kb)	756	MUC3A	4584
493467	7q	100993912	100995785	1874	10	0.7	Exon(exon5of15)	-17269	MUC12	10071
493467	7q	101034361	101040583	6223	36	0.528	Exon(exon3of12)	-3128	MUC17	140453
493467	7q	149818015	149819792	1778	6	0.667	Promoter(2-3kb)	-2352	SSPO	23145
493467	7q	149824094	149826495	2402	6	0.667	Promoter(<=1kb)	0	SSPO	23145
493467	8p	10609614	10612307	2694	10	0.9	Exon(exon4of4)	42836	RP1L1	94137
493467	8p	13021128	13022030	903	7	0.143	Exon(exon5of5)	9115	TRMT9B	57604
493467	8p	22163106	22164421	1316	6	1	Promoter(<=1kb)	26	SFTPC	6440
493467	8q	123651655	123652634	980	7	0.571	Promoter(<=1kb)	316	KLHL38	340359
493467	9p	116800	117934	1135	6	0.833	Promoter(<=1kb)	270	FOXD4	2298
493467	9p	21206764	21207038	275	6	0.667	Promoter(<=1kb)	105	IFNA10	3446
493467	9q	76175237	76175296	60	10	0.8	Exon(exon14of14)	-13343	PCSK5	5125
493467	9q	76705179	76707804	2626	8	0.5	Promoter(<=1kb)	121	PCA3	50652
493467	9q	76709263	76710843	1581	8	0.5	Promoter(<=1kb)	0	PRUNE2	158471
493467	9q	87885278	87888819	3542	13	0.692	Promoter(2-3kb)	2401	SPATA31E1	286234
493467	9q	104504315	104505071	757	6	0.5	Promoter(<=1kb)	52	OR13F1	138805
493467	9q	122749914	122750547	634	6	0.833	Promoter(<=1kb)	174	OR1L6	392390
493467	9q	133255635	133256205	571	10	0.9	3'UTR	19009	ABO	28
493467	9q	135484803	135487213	2411	9	0.333	Promoter(1-2kb)	1440	PPP1R26	9858
493467	10q	46549378	46550723	1346	26	0.654	Exon(exon3of3)	4807	GPRIN2	9721
493467	10q	122084988	122087840	2853	8	0.75	Exon(exon4of23)	-25190	TACC2	10579
493467	10q	128103129	128108204	5076	15	0.667	Promoter(<=1kb)	-1	MKI67	4288
493467	11p	244106	244197	92	8	0.5	Promoter(<=1kb)	-232	PSMD13	5719
493467	11p	1194354	1196902	2549	7	0.571	Exon(exon34of49)	-26164	MUC5B	727897
493467	11p	1246095	1247378	1284	8	0.375	Promoter(2-3kb)	2298	MUC5B-AS1	112577518
493467	11p	5177978	5178478	501	6	0.167	Promoter(<=1kb)	186	OR52Z1	283110

493467	11p	5389704	5390350	647	7	0.429	Promoter(<=1kb)	327	OR51M1	390059
493467	11p	5422212	5423123	912	11	0.636	Promoter(<=1kb)	101	OR51Q1	390061
493467	11p	5581045	5581738	694	8	0.375	Promoter(<=1kb)	168	OR52B6	340980
493467	11p	5841302	5841883	582	9	0.333	Promoter(<=1kb)	14	OR52E6	390078
493467	11p	5884818	5885061	244	7	0.429	Promoter(<=1kb)	547	OR52E4	390081
493467	11p	5986042	5986542	501	6	1	Promoter(<=1kb)	443	OR52L1	338751
493467	11p	11351961	11352736	776	7	0.143	Promoter(<=1kb)	514	CSNK2A3	283106
493467	11p	12293628	12294368	741	7	0.857	Exon(exon29of35)	6728	MICALCL	84953
493467	11p	18173280	18173901	622	6	0.333	Promoter(<=1kb)	443	MRGPRX4	117196
493467	11q	58214757	58215722	966	7	0.286	Promoter(<=1kb)	12	OR1S1	219959
493467	11q	58402523	58403265	743	8	0.5	Promoter(<=1kb)	144	OR5B3	441608
493467	11q	82732630	82733184	555	6	0.833	Promoter(<=1kb)	680	FAM181B	220382
493467	11q	85724687	85725825	1139	6	0.5	Promoter(<=1kb)	0	SYTL2	54843
493467	11q	123906790	123907324	535	6	0.667	Promoter(<=1kb)	644	OR8D4	338662
493467	11q	124038366	124038988	623	7	1	Promoter(<=1kb)	13	OR10G7	390265
493467	11q	124382526	124383285	760	10	0.6	Promoter(<=1kb)	58	OR8B2	26595
493467	12p	4626571	4628549	1979	9	0.444	Exon(exon5of6)	14054	DYRK4	8798
493467	12p	6018725	6019629	905	6	0.5	Promoter(2-3kb)	2297	VWF	7450
493467	12q	52316096	52317765	1670	6	0.667	Exon(exon4of9)	3633	KRT83	3889
493467	13q	25096713	25097005	293	7	0.143	Promoter(<=1kb)	845	PABPC3	5042
493467	13q	102732474	102733933	1460	6	0.333	Exon(exon4of4)	25139	CCDC168	643677
493467	14q	19935750	19936577	828	6	0.833	Promoter(<=1kb)	142	OR4K1	79544
493467	14q	20060048	20060884	837	8	0.625	Promoter(<=1kb)	3	OR4L1	122742
493467	14q	21634137	21634589	453	9	0.556	Promoter(<=1kb)	351	OR10G2	26534
493467	14q	44504986	44506403	1418	6	0.667	Promoter(<=1kb)	880	FSCB	84075
493467	14q	70457520	70458540	1021	12	0.333	Exon(exon2of2)	5346	ADAM21	8747
493467	14q	94587512	94587839	328	6	0.5	Exon(exon2of2)	-4219	SERPINA3	12
493467	14q	104949015	104951938	2924	10	0.4	Exon(exon6of6)	3464	AHNAK2	113146
493467	15q	20534480	20535129	650	8	0.375	Exon(exon8of9)	6671	GOLGA6L6	727832
493467	15q	23439979	23442067	2089	12	0.5	5'UTR	5167	GOLGA6L2	283685
493467	15q	40621642	40623696	2055	6	0.333	Promoter(<=1kb)	0	KNL1	57082
493467	15q	79456261	79457965	1705	6	0.5	Promoter(<=1kb)	165	MINAR1	23251
493467	16p	1486371	1488463	2093	8	0.75	Promoter(<=1kb)	4	PTX4	390667
493467	16q	88428539	88429600	1062	6	0.333	Exon(exon3of3)	-23680	ZFPM1	161882
493467	16q	88714632	88717113	2482	7	0.429	Promoter(<=1kb)	0	MIR4722	100616167
493467	16q	89100686	89101050	365	8	0.625	Promoter(<=1kb)	24	ACSF3	197322
493467	16q	89227116	89228419	1304	6	0.667	Promoter(2-3kb)	2482	ZNF778	197320
493467	17p	744917	746966	2050	8	0.875	3'UTR	5072	GEMIN4	50628
493467	17p	10638198	10641099	2902	7	0.286	Exon(exon19of41)	-8169	MYH3	4621
493467	17p	21300581	21300978	398	11	0.727	3'UTR	9112	MAP2K3	5606
493467	17p	21415470	21416404	935	6	1	Exon(exon3of3)	10334	KCNJ12	3768
493467	17q	73236508	73236991	484	6	0.333	Promoter(<=1kb)	0	FAM104A	84923
493467	17q	76293419	76294016	598	6	0.5	Promoter(2-3kb)	-2167	QRICH2	84074
493467	17q	81645135	81645607	473	7	0.286	Promoter(2-3kb)	2722	TSPAN10	83882
493467	18q	58535186	58538030	2845	19	0.526	Promoter(<=1kb)	0	ALPK2	115701
493467	18q	75285722	75287404	1683	6	0.333	Exon(exon2of2)	41146	TSHZ1	10194
493467	19p	5455600	5456439	840	6	0.5	Promoter(<=1kb)	183	ZNRF4	148066
493467	19p	8937644	8939234	1591	6	0.833	Exon(exon5of84)	-41554	MUC16	94025
493467	19p	8946306	8951868	5563	18	0.667	Exon(exon3of84)	29474	MUC16	94025
493467	19p	8959116	8962299	3184	12	0.583	Exon(exon3of84)	19043	MUC16	94025
493467	19p	8963397	8967127	3731	21	0.667	Exon(exon3of84)	14215	MUC16	94025
493467	19p	8972751	8978096	5346	13	0.462	Exon(exon1of84)	3246	MUC16	94025
493467	19p	12430157	12432437	2281	9	0.333	3'UTR	8584	ZNF443	10224
493467	19p	15794192	15794829	638	8	0.375	Promoter(<=1kb)	241	OR10H5	284433
493467	19p	18264798	18267409	2612	12	0.583	5'UTR	7002	IQCN	80726
493467	19p	21971930	21974500	2571	7	0.714	Exon(exon4of4)	14408	ZNF208	7757
493467	19p	22314389	22314412	24	6	0	Exon(exon4of4)	27948	ZNF729	100287226
493467	19q	37886882	37888806	1925	6	0.833	Exon(exon6of6)	17788	WDR87	83889
493467	19q	39886240	39886422	183	6	0.667	Promoter(<=1kb)	870	FCGBP	8857
493467	19q	43913423	43914878	1456	8	0.5	Exon(exon10of10)	4861	ZNF45	7596
493467	19q	43996326	43997366	1041	6	0.5	Exon(exon5of5)	5419	LOC10192806:	101928063
493467	19q	44106512	44108078	1567	7	0	Exon(exon6of6)	-4103	ZNF225	7768
493467	19q	44327836	44329698	1863	6	0.5	Exon(exon4of4)	-22790	ZNF235	9310
493467	19q	52437918	52439242	1325	7	0.429	Exon(exon4of4)	6504	ZNF534	147658
493467	19q	53164551	53166239	1689	6	0.167	Exon(exon4of4)	-5476	ZNF347	84671
493467	19q	55911888	55913077	1190	6	0.667	Exon(exon5of12)	19234	NLRP13	126204
493467	19q	56664116	56665114	999	6	0.5	Exon(exon2of2)	6643	ZNF835	90485
493467	20p	5922577	5923382	806	7	0.571	Exon(exon4of5)	7079	CHGB	1114
493467	20q	63561666	63565531	3866	14	0.714	Promoter(<=1kb)	-61	HELZ2	85441
493467	21q	44627405	44628008	604	6	0.667	Promoter(<=1kb)	312	KRTAP10-9	386676
493467	21q	44637474	44638143	670	8	0.5	Promoter(<=1kb)	118	KRTAP10-10	353333
493467	22q	22352950	22353380	431	16	0.5	Exon(exon1of2)	30478	BMS1P20	96610
493467	22q	22687062	22687232	171	6	1	Exon(exon2of2)	40284	GGTLC2	91227
493467	22q	36191154	36191906	753	7	0.714	3'UTR	9971	APOL4	80832
493467	22q	49883704	49884994	1291	6	0.167	Exon(exon2of2)	22924	ALG12	79087
493467	23p	3320126	3323750	3625	9	0.556	Exon(exon5of7)	22902	MXRA5	25878
493467	23p	8170039	8170141	103	6	0.5	Promoter(1-2kb)	1126	VCX2	51480

493467	23p	35802148	35803010	863	7	0.571	5'UTR	3357	MAGEB16	139604
493748	1p	12847526	12847995	470	9	0.444	Promoter(<=1kb)	730	HNRNPCL1	343069
493748	1p	12859036	12860079	1044	6	0.333	Promoter(1-2kb)	1950	PRAMEF2	65122
493748	1p	12893249	12893472	224	6	0.5	Exon(exon4of4)	4798	PRAMEF10	343071
493748	1p	13369166	13369564	399	6	0.833	Promoter(2-3kb)	2336	PRAMEF19	645414
493748	1p	13370686	13371119	434	7	0.429	Promoter(<=1kb)	781	PRAMEF19	645414
493748	1p	16058491	16060000	1510	13	0.846	Exon(exon5of7)	6168	CLCNKB	1188
493748	1p	18481403	18483159	1757	7	0.714	Promoter(<=1kb)	421	KLHDC7A	127707
493748	1p	23874604	23875430	827	8	0.5	Exon(exon2of2)	-6310	FUCA1	2517
493748	1p	40067594	40067675	82	6	0	Promoter(<=1kb)	324	CAP1	10487
493748	1q	152218469	152221375	2907	18	0.722	Promoter(2-3kb)	2818	HRNR	388697
493748	1q	152303673	152313911	10239	39	0.615	Promoter(<=1kb)	0	FLG-AS1	339400
493748	1q	156669844	156670886	1043	6	1	Exon(exon4of4)	6521	NES	10763
493748	1q	158765805	158766655	851	6	0.5	Promoter(<=1kb)	47	OR6N1	128372
493748	1q	201206099	201209342	3244	10	0.6	Promoter(1-2kb)	1017	IGFN1	91156
493748	1q	214640144	214642954	2811	12	0.5	Exon(exon12of20)	-5013	CENPF	1063
493748	1q	214644872	214647181	2310	10	0.4	Promoter(<=1kb)	-786	CENPF	1063
493748	1q	223393517	223394466	950	6	0.667	Promoter(<=1kb)	102	CCDC185	164127
493748	1q	228315976	228318038	2063	9	0.556	Exon(exon50of81)	6492	OBSCN	84033
493748	1q	228371172	228372999	1828	8	0.375	Promoter(<=1kb)	0	OBSCN	84033
493748	1q	247841312	247841582	271	6	0.833	Promoter(<=1kb)	314	OR11L1	391189
493748	1q	247949325	247949738	414	11	0.273	Promoter(<=1kb)	467	OR2L8	391190
493748	1q	248294677	248295458	782	7	0.571	Promoter(<=1kb)	142	OR2T12	127064
493748	2p	29002636	29003646	1011	6	0.333	Exon(exon5of20)	-10821	TOGARAM2	165186
493748	2p	48580657	48582454	1798	7	0.571	Promoter(<=1kb)	0	STON1	11037
493748	2q	132783032	132785012	1981	9	0.556	Promoter(1-2kb)	-1009	NCKAP5	344148
493748	2q	178739433	178741811	2379	6	0.5	Exon(exon45of191)	26014	TTN	7273
493748	2q	184936178	184937636	1459	6	0.333	Exon(exon4of4)	69813	ZNF804A	91752
493748	2q	185789865	185794632	4768	10	0.8	Promoter(<=1kb)	0	FSIP2	401024
493748	2q	217847583	217848559	977	6	0.833	Exon(exon19of33)	-5423	TNS1	7145
493748	2q	232408301	232409799	1499	6	1	Promoter(1-2kb)	1457	ALPG	251
493748	2q	240041845	240042811	967	8	0.25	Downstream(2-3kb)	3261	OR6B3	150681
493748	3p	75736880	75739007	2128	48	0.562	Promoter(<=1kb)	0	MIR4273	100422955
493748	3q	98264413	98265098	686	7	0.571	Promoter(<=1kb)	128	OR5H6	79295
493748	3q	194341097	194342571	1475	7	0.714	Exon(exon2of2)	8747	CPN2	1370
493748	3q	196947878	196948662	785	6	0.5	3'UTR	3268	PIGZ	80235
493748	4p	1394795	1395368	574	6	0.5	Exon(exon1of1)	9983	UVSSA	57654
493748	4p	6300792	6302360	1569	6	0.833	Exon(exon8of8)	6021	WFS1	7466
493748	4p	8227004	8228508	1505	7	0.143	Promoter(<=1kb)	-24	SH3TC1	54436
493748	4q	121036404	121037542	1139	6	0.333	Promoter(1-2kb)	1442	NDNF	79625
493748	4q	185458217	185460011	1795	8	0.625	Promoter(<=1kb)	0	CCDC110	256309
493748	4q	186619481	186621582	2102	6	0.333	Exon(exon10of27)	-9411	FAT1	2195
493748	5p	795818	796237	420	7	0.571	3'UTR	4908	ZDHHC11	79844
493748	5q	79731782	79734523	2742	13	0.308	Exon(exon2of13)	-3619	CMYA5	202333
493748	5q	140848579	140850786	2208	6	0.5	Promoter(<=1kb)	807	PCDHA9	9752
493748	5q	141122595	141123655	1061	6	0.833	Promoter(<=1kb)	777	PCDHB4	56131
493748	5q	141174000	141175025	1026	7	0.714	Promoter(1-2kb)	1356	PCDHB7	56129
493748	5q	148826877	148828070	1194	6	1	Promoter(1-2kb)	1632	ADRB2	154
493748	5q	151521550	151522069	520	6	0.667	Promoter(<=1kb)	79	MIR6499	102465246
493748	5q	151565922	151568158	2237	9	0.778	Promoter(<=1kb)	786	FAT2	2196
493748	6p	26370344	26370479	136	6	0.5	Promoter(<=1kb)	0	BTN3A2	11118
493748	6p	39314260	39316408	2149	6	0.667	Promoter(<=1kb)	0	KCNK17	89822
493748	6p	46858771	46859389	619	6	0.5	Exon(exon17of21)	3915	ADGRF5	221395
493748	6q	149888581	149890867	2287	7	0.714	Promoter(<=1kb)	0	RAET1E-AS1	100652739
493748	7q	64991278	64992758	1481	6	0.667	Promoter(<=1kb)	-242	ZNF117	51351
493748	7q	100958977	100960873	1897	54	0.444	Promoter(1-2kb)	1012	MUC3A	4584
493748	7q	100991195	100993127	1933	9	0.667	Exon(exon5of15)	-19927	MUC12	10071
493748	7q	100995575	100995785	211	6	0.833	Exon(exon5of15)	-17269	MUC12	10071
493748	8p	10607245	10608432	1188	8	0.5	Exon(exon4of4)	46711	RP1L1	94137
493748	8p	10609614	10610843	1230	9	0.556	Exon(exon4of4)	44300	RP1L1	94137
493748	8p	12132477	12133940	1464	11	0.727	Promoter(<=1kb)	498	USP17L7	392197
493748	8p	13021128	13022030	903	7	0.143	Exon(exon5of5)	9115	TRMT9B	57604
493748	8q	138151949	138153046	1098	6	0.667	Promoter(<=1kb)	0	FAM135B	51059
493748	8q	143870008	143872631	2624	6	0.5	Exon(exon2of2)	5833	EPPK1	83481
493748	9q	76703555	76707804	4250	14	0.5	Promoter(<=1kb)	0	PCA3	50652
493748	9q	76709263	76710843	1581	8	0.5	Promoter(<=1kb)	0	PRUNE2	158471
493748	9q	87886533	87888398	1866	6	0.667	Exon(exon4of4)	3656	SPATA31E1	286234
493748	9q	104504315	104505071	757	6	0.5	Promoter(<=1kb)	52	OR13F1	138805
493748	9q	104598545	104599361	817	12	0.583	Promoter(<=1kb)	52	OR13C5	138799
493748	9q	104694482	104695444	963	6	0.5	Promoter(<=1kb)	0	OR13D1	286365
493748	9q	122628595	122629130	536	6	0.333	Promoter(<=1kb)	443	OR1B1	347169
493748	9q	122749914	122750547	634	6	0.833	Promoter(<=1kb)	174	OR1L6	392390
493748	9q	133255635	133256205	571	7	1	3'UTR	19009	ABO	28
493748	9q	135484803	135487213	2411	8	0.25	Promoter(1-2kb)	1440	PPP1R26	9858
493748	9q	135547960	135548795	836	8	0.625	Promoter(1-2kb)	1805	OBP2A	29991
493748	10q	46549378	46550723	1346	27	0.667	Exon(exon3of3)	4807	GPRIN2	9721
493748	10q	49323169	49326817	3649	12	0.583	Exon(exon3of3)	23895	C10orf71	118461

493748	10q	89737450	89738561	1112	6	0	Exon(exon20of33)	13874	KIF20B	9585
493748	11p	244106	244197	92	8	0.5	Promoter(<=1kb)	-232	PSMD13	5719
493748	11p	1194354	1196902	2549	7	0.571	Exon(exon34of49)	-26164	MUC5B	727897
493748	11p	1246095	1251628	5534	19	0.579	Promoter(<=1kb)	0	MUC5B-AS1	112577518
493748	11p	5177811	5178478	668	7	0.143	Promoter(<=1kb)	186	OR52Z1	283110
493748	11p	5323451	5324256	806	6	0.5	Promoter(<=1kb)	41	OR51B2	79345
493748	11p	5422212	5423123	912	12	0.667	Promoter(<=1kb)	101	OR51Q1	390061
493748	11p	5581045	5581738	694	8	0.375	Promoter(<=1kb)	168	OR52B6	340980
493748	11p	5841302	5841883	582	9	0.333	Promoter(<=1kb)	14	OR52E6	390078
493748	11p	5986042	5986669	628	7	1	Promoter(<=1kb)	316	OR52L1	338751
493748	11p	11351961	11352736	776	9	0.222	Promoter(<=1kb)	514	CSNK2A3	283106
493748	11p	12293639	12294368	730	6	0.833	Exon(exon29of35)	6739	MICALCL	84953
493748	11q	58214757	58215722	966	8	0.25	Promoter(<=1kb)	12	OR1S1	219959
493748	11q	64116513	64118232	1720	7	0.714	Exon(exon2of2)	8702	MACROD1	28992
493748	11q	82732630	82733184	555	7	0.857	Promoter(<=1kb)	680	FAM181B	220382
493748	11q	85724687	85725825	1139	6	0.5	Promoter(<=1kb)	0	SYTL2	54843
493748	11q	124038366	124038988	623	7	1	Promoter(<=1kb)	13	OR10G7	390265
493748	11q	124382526	124383285	760	8	0.625	Promoter(<=1kb)	58	OR8B2	26595
493748	12p	4626571	4628549	1979	8	0.5	Exon(exon5of6)	14054	DYRK4	8798
493748	12p	6453119	6453670	552	7	0.571	Promoter(<=1kb)	633	TAPBPL	55080
493748	12q	49795819	49797137	1319	6	0.5	Promoter(<=1kb)	0	NCKAP5L	57701
493748	12q	50352073	50353545	1473	6	0.333	Promoter(1-2kb)	-1188	FAM186A	121006
493748	12q	52316096	52317765	1670	6	0.667	Exon(exon4of9)	3633	KRT83	3889
493748	13q	24434450	24435347	898	7	0.571	Exon(exon31of34)	19787	PARP4	143
493748	13q	102732474	102733933	1460	6	0.333	Exon(exon4of4)	25139	CCDC168	643677
493748	14q	20060048	20060884	837	8	0.625	Promoter(<=1kb)	3	OR4L1	122742
493748	14q	20640982	20641567	586	6	0.5	Promoter(<=1kb)	124	OR6S1	341799
493748	14q	21634137	21634589	453	9	0.556	Promoter(<=1kb)	351	OR10G2	26534
493748	14q	22633879	22634450	572	9	0.333	Exon(exon2of2)	32212	ABHD4	63874
493748	14q	70457532	70458540	1009	7	0.286	Exon(exon2of2)	5358	ADAM21	8747
493748	14q	104175275	104177810	2536	10	0.4	Exon(exon12of15)	36235	KIF26A	26153
493748	14q	104939262	104942618	3357	10	0.2	5'UTR	7102	PLD4	122618
493748	14q	104943622	104945444	1823	7	0.571	Exon(exon6of6)	9958	AHNAK2	113146
493748	14q	104947943	104949893	1951	12	0.5	Exon(exon6of6)	5509	AHNAK2	113146
493748	14q	104950949	104953878	2930	10	0.4	Promoter(1-2kb)	1524	AHNAK2	113146
493748	15q	20534480	20535063	584	7	0.429	Exon(exon8of9)	6737	GOLGA6L6	727832
493748	15q	85579423	85582073	2651	15	0.6	Promoter(<=1kb)	-837	AKAP13	11214
493748	15q	99129423	99132517	3095	7	0.429	Exon(exon4of5)	7225	TTC23	64927
493748	15q	101066056	101067308	1253	6	0.667	Promoter(1-2kb)	1640	LRRK1	79705
493748	16p	1228744	1229622	879	7	0.714	Promoter(<=1kb)	540	TPSB2	64499
493748	16p	1486371	1488463	2093	8	0.75	Promoter(<=1kb)	4	PTX4	390667
493748	16p	4207130	4208004	875	6	0.5	Exon(exon2of7)	31739	SRL	6345
493748	16q	74391401	74391897	497	8	0.75	Exon(exon7of7)	13523	NPIPB15	440348
493748	16q	88428539	88431889	3351	11	0.364	Exon(exon3of3)	-21391	ZFPM1	161882
493748	16q	89100686	89101050	365	9	0.667	Promoter(<=1kb)	24	ACSF3	197322
493748	16q	89226863	89228419	1557	12	0.667	Promoter(2-3kb)	2229	ZNF778	197320
493748	17p	2299649	2300159	511	6	0.167	Exon(exon2of19)	-3224	SRR	63826
493748	17p	21300581	21300978	398	11	0.727	3'UTR	9112	MAP2K3	5606
493748	17q	41586466	41586829	364	6	0.833	Promoter(<=1kb)	66	KRT14	3861
493748	17q	41727098	41728331	1234	6	0.833	Promoter(1-2kb)	-1201	HAP1	9001
493748	17q	81510690	81511591	902	6	0.667	Promoter(<=1kb)	257	ACTG1	71
493748	17q	81645135	81645417	283	6	0.333	Promoter(2-3kb)	2722	TSPAN10	83882
493748	18p	11609728	11610383	656	10	0.9	Promoter(<=1kb)	132	SLC35G4	646000
493748	18p	14542649	14543140	492	6	0.5	Promoter(<=1kb)	6	POTEC	388468
493748	18q	58535186	58538030	2845	19	0.526	Promoter(<=1kb)	0	ALPK2	115701
493748	19p	4510548	4513547	3000	20	0.45	Exon(exon3of6)	4157	PLIN4	729359
493748	19p	5455600	5456439	840	7	0.571	Promoter(<=1kb)	183	ZNRF4	148066
493748	19p	8937644	8939234	1591	6	0.833	Exon(exon5of84)	-41554	MUC16	94025
493748	19p	8946306	8951868	5563	19	0.632	Exon(exon3of84)	29474	MUC16	94025
493748	19p	8959116	8962299	3184	12	0.583	Exon(exon3of84)	19043	MUC16	94025
493748	19p	8963397	8967127	3731	21	0.667	Exon(exon3of84)	14215	MUC16	94025
493748	19p	8972448	8978096	5649	14	0.5	Exon(exon1of84)	3246	MUC16	94025
493748	19p	15087452	15087953	502	7	0.143	Promoter(<=1kb)	472	OR1I1	126370
493748	19p	18264798	18267409	2612	12	0.583	5'UTR	7002	IQCN	80726
493748	19p	21971930	21974500	2571	7	0.714	Exon(exon4of4)	14408	ZNF208	7757
493748	19p	23743906	23745300	1395	6	0.333	Exon(exon4of4)	13537	ZNF681	148213
493748	19q	37886924	37889059	2136	6	0.833	Exon(exon6of6)	17535	WDR87	83889
493748	19q	39876927	39877880	954	7	0.571	Exon(exon20of28)	9412	FCGBP	8857
493748	19q	39886240	39886422	183	6	0.667	Promoter(<=1kb)	870	FCGBP	8857
493748	19q	43612804	43614201	1398	6	0.5	Promoter(<=1kb)	716	SRRM5	100170229
493748	19q	43846955	43848536	1582	6	0.833	3'UTR	13450	ZNF283	284349
493748	19q	43913423	43914878	1456	7	0.429	Exon(exon10of10)	4861	ZNF45	7596
493748	19q	44106512	44108078	1567	7	0	Exon(exon6of6)	-4103	ZNF225	7768
493748	19q	52384174	52385296	1123	6	0	Exon(exon4of4)	12373	ZNF528-AS1	102724105
493748	19q	52437918	52439242	1325	7	0.429	Exon(exon4of4)	6504	ZNF534	147658
493748	19q	55911888	55913166	1279	7	0.714	Exon(exon5of12)	19145	NLRP13	126204
493748	19q	58368293	58368994	702	8	0.5	Exon(exon3of3)	-5445	ZNF497	162968

493748	20p	5922677	5923382	706	6	0.5	Exon(exon4of5)	7179	CHGB	1114
493748	20p	20052354	20052736	383	6	0.167	Promoter(<=1kb)	0	CFAP61	26074
493748	20q	63349752	63350772	1021	6	0.5	3'UTR	3794	CHRNA4	1137
493748	20q	63561666	63565531	3866	13	0.615	Promoter(<=1kb)	-61	HELZ2	85441
493748	21q	26843740	26844859	1120	6	0.667	Promoter(<=1kb)	0	ADAMTS1	9510
493748	22q	22352950	22353380	431	16	0.5	Exon(exon1of2)	30478	BMS1P20	96610
493748	22q	25028581	25029472	892	6	0.333	Promoter(<=1kb)	607	KIAA1671	85379
493748	22q	36191154	36191906	753	6	0.667	3'UTR	9971	APOL4	80832
493748	22q	49884187	49884994	808	6	0.333	Exon(exon2of2)	22924	ALG12	79087
493748	23p	3320126	3323750	3625	9	0.556	Exon(exon5of7)	22902	MXRA5	25878
493748	23p	8170039	8170141	103	6	0.5	Promoter(1-2kb)	1126	VCX2	51480
494208	1p	12847526	12847780	255	7	0.429	Promoter(<=1kb)	945	HNRNPCL1	343069
494208	1p	12859036	12860212	1177	14	0.5	Promoter(1-2kb)	1950	PRAMEF2	65122
494208	1p	12861255	12861721	467	8	0.375	Exon(exon4of4)	4169	PRAMEF2	65122
494208	1p	12879619	12879853	235	6	0.667	Exon(exon4of4)	6348	PRAMEF4	400735
494208	1p	13369166	13369564	399	7	0.714	Promoter(2-3kb)	2336	PRAMEF19	645414
494208	1p	13370686	13371119	434	7	0.429	Promoter(<=1kb)	781	PRAMEF19	645414
494208	1p	16058491	16060000	1510	10	0.9	Exon(exon5of7)	6168	CLCNKB	1188
494208	1p	18481403	18483159	1757	7	0.714	Promoter(<=1kb)	421	KLHDC7A	127707
494208	1p	23874604	23875430	827	8	0.5	Exon(exon2of2)	-6310	FUCA1	2517
494208	1p	40067594	40067675	82	6	0	Promoter(<=1kb)	324	CAP1	10487
494208	1p	89186388	89186419	32	9	0.556	Promoter(<=1kb)	107	GBP4	115361
494208	1q	152302977	152313891	10915	41	0.61	Promoter(<=1kb)	0	FLG-AS1	339400
494208	1q	156669844	156670886	1043	6	1	Exon(exon4of4)	6521	NES	10763
494208	1q	169540901	169542882	1982	10	0.3	Exon(exon13of25)	-25156	F5	2153
494208	1q	201206099	201209837	3739	12	0.583	Promoter(1-2kb)	1017	IGFN1	91156
494208	1q	223393517	223395217	1701	7	0.714	Promoter(<=1kb)	102	CCDC185	164127
494208	1q	228315976	228318038	2063	9	0.667	Exon(exon50of81)	6492	OBSCN	84033
494208	1q	247841312	247841582	271	6	0.833	Promoter(<=1kb)	314	OR11L1	391189
494208	1q	247921132	247921822	691	6	0.667	Promoter(<=1kb)	114	OR2T8	343172
494208	1q	247949325	247949738	414	10	0.3	Promoter(<=1kb)	467	OR2L8	391190
494208	1q	248294677	248295458	782	7	0.571	Promoter(<=1kb)	142	OR2T12	127064
494208	1q	248681658	248682198	541	7	0.571	Promoter(<=1kb)	130	OR14I1	401994
494208	2p	48580657	48582454	1798	7	0.571	Promoter(<=1kb)	0	STON1	11037
494208	2q	102351547	102351902	356	7	0.429	Exon(exon11of11)	-4027	IL18R1	8809
494208	2q	132783032	132785012	1981	7	0.571	Promoter(1-2kb)	-1009	NCKAP5	344148
494208	2q	178739433	178741811	2379	6	0.5	Exon(exon45of191)	26014	TTN	7273
494208	2q	233713134	233713783	650	13	0.692	Promoter(<=1kb)	142	UGT1A5	54579
494208	2q	237762685	237764060	1376	10	0.5	Exon(exon8of8)	-4137	LRRFIP1	9208
494208	2q	238130416	238131546	1131	7	0.429	Promoter(1-2kb)	1468	ESPNL	339768
494208	2q	240041845	240042131	287	6	0.167	Downstream(2-3kb)	3941	OR6B3	150681
494208	3p	31989532	31990905	1374	7	0.286	Exon(exon2of2)	7761	ZNF860	344787
494208	3p	75736929	75739007	2079	15	0.6	Promoter(<=1kb)	0	MIR4273	100422955
494208	3q	98264413	98265098	686	7	0.571	Promoter(<=1kb)	128	OR5H6	79295
494208	4p	5988383	5989749	1367	7	0.571	Promoter(<=1kb)	0	C4orf50	389197
494208	4p	6300792	6302391	1600	7	0.857	Exon(exon8of8)	6021	WFS1	7466
494208	4p	8227004	8228508	1505	7	0.143	Promoter(<=1kb)	-24	SH3TC1	54436
494208	4q	185458217	185460011	1795	8	0.625	Promoter(<=1kb)	0	CCDC110	256309
494208	4q	186706638	186709751	3114	7	0.714	Exon(exon2of27)	14082	FAT1	2195
494208	5q	79731782	79734523	2742	13	0.308	Exon(exon2of13)	-3619	CMYA5	202333
494208	5q	83537326	83539905	2580	6	0.333	Promoter(1-2kb)	1712	VCAN	1462
494208	5q	140848579	140850786	2208	6	0.5	Promoter(<=1kb)	807	PCDHA9	9752
494208	5q	141174000	141175025	1026	6	0.833	Promoter(1-2kb)	1356	PCDHB7	56129
494208	5q	141955356	141957660	2305	6	0.667	Promoter(<=1kb)	-668	RNF14	9604
494208	5q	151521550	151522069	520	6	0.667	Promoter(<=1kb)	79	MIR6499	102465246
494208	5q	151565922	151568455	2534	10	0.8	Promoter(<=1kb)	489	FAT2	2196
494208	6p	46858771	46859502	732	8	0.5	Exon(exon17of21)	3802	ADGRF5	221395
494208	6q	149888581	149890867	2287	7	0.714	Promoter(<=1kb)	0	RAET1E-AS1	100652739
494208	6q	159233455	159234370	916	10	0.5	Exon(exon11of23)	15158	FNDC1	84624
494208	7p	6330446	6330944	499	6	1	Exon(exon2of2)	7749	FAM220A	84792
494208	7p	38353718	38353991	274	8	0.625	Exon(exon2of2)	3699	TRG-AS1	100506776
494208	7p	53035678	53036385	708	7	1	Promoter(<=1kb)	45	POM121L12	285877
494208	7q	100958721	100960873	2153	55	0.473	Promoter(<=1kb)	756	MUC3A	4584
494208	7q	100991195	100993102	1908	9	0.667	Exon(exon5of15)	-19952	MUC12	10071
494208	7q	100995575	100995785	211	7	0.714	Exon(exon5of15)	-17269	MUC12	10071
494208	7q	101004230	101004836	607	7	0.714	Exon(exon5of15)	-8218	MUC12	10071
494208	7q	101034361	101040583	6223	39	0.513	Exon(exon3of12)	-3128	MUC17	140453
494208	7q	149818015	149819792	1778	6	0.667	Promoter(2-3kb)	-2352	SSPO	23145
494208	8p	10607245	10608432	1188	8	0.5	Exon(exon4of4)	46711	RP1L1	94137
494208	8p	10609614	10610662	1049	8	0.5	Exon(exon4of4)	44481	RP1L1	94137
494208	8p	11331234	11332082	849	7	0.714	Promoter(<=1kb)	346	SLC35G5	83650
494208	8p	12137448	12138752	1305	7	0.857	Promoter(<=1kb)	325	USP17L2	377630
494208	8p	13021128	13022030	903	8	0.25	Exon(exon5of5)	9115	TRMT9B	57604
494208	8p	22163175	22164421	1247	6	0.833	Promoter(<=1kb)	95	SFTPC	6440
494208	8q	141466455	141467514	1060	8	0.75	3'UTR	29245	MROH5	389690
494208	9p	116800	117934	1135	8	0.75	Promoter(<=1kb)	270	FOXD4	2298
494208	9q	76175241	76175296	56	9	0.889	Exon(exon14of14)	-13343	PCSK5	5125

494208	9q	76707758	76710843	3086	8	0.375	Promoter(<=1kb)	0	PRUNE2	158471
494208	9q	104598545	104599361	817	12	0.583	Promoter(<=1kb)	52	OR13C5	138799
494208	9q	122553278	122554071	794	7	0.429	Promoter(<=1kb)	108	OR1N2	138882
494208	9q	122628595	122629398	804	6	0.333	Promoter(<=1kb)	175	OR1B1	347169
494208	9q	122749914	122750547	634	6	0.833	Promoter(<=1kb)	174	OR1L6	392390
494208	9q	133255635	133256264	630	8	1	3'UTR	18950	ABO	28
494208	9q	135484803	135487573	2771	10	0.4	Promoter(1-2kb)	1440	PPP1R26	9858
494208	10p	47663	48605	943	7	0.571	Promoter(<=1kb)	664	TUBB8	347688
494208	10q	46549378	46550723	1346	25	0.64	Exon(exon3of3)	4807	GPRIN2	9721
494208	11p	244106	244197	92	8	0.5	Promoter(<=1kb)	-232	PSMD13	5719
494208	11p	308290	309127	838	6	0.5	Promoter(<=1kb)	0	IFITM2	10581
494208	11p	1246225	1247378	1154	7	0.571	Promoter(2-3kb)	2298	MUC5B-AS1	112577518
494208	11p	5323542	5324256	715	6	0.5	Promoter(<=1kb)	41	OR51B2	79345
494208	11p	5351521	5352416	896	15	0.467	Promoter(<=1kb)	13	OR51B6	390058
494208	11p	5389704	5390350	647	7	0.429	Promoter(<=1kb)	327	OR51M1	390059
494208	11p	5402638	5403322	685	10	0.5	Promoter(<=1kb)	41	OR51J1	79470
494208	11p	5422212	5423123	912	10	0.7	Promoter(<=1kb)	101	OR51Q1	390061
494208	11p	5515185	5515931	747	6	0.333	Promoter(<=1kb)	768	UBQLNL	143630
494208	11p	5581045	5581738	694	8	0.375	Promoter(<=1kb)	168	OR52B6	340980
494208	11p	5884818	5885061	244	6	0.333	Promoter(<=1kb)	547	OR52E4	390081
494208	11p	11351961	11352736	776	9	0.222	Promoter(<=1kb)	514	CSNK2A3	283106
494208	11p	12293639	12294368	730	6	0.833	Exon(exon29of35)	6739	MICALCL	84953
494208	11p	43942293	43943348	1056	9	0.889	Promoter(<=1kb)	0	C11orf96	387763
494208	11q	58214757	58215722	966	8	0.25	Promoter(<=1kb)	12	OR1S1	219959
494208	11q	64116513	64118232	1720	7	0.714	Exon(exon2of2)	8702	MACROD1	28992
494208	11q	85724687	85725825	1139	6	0.5	Promoter(<=1kb)	0	SYTL2	54843
494208	11q	123906790	123907324	535	6	0.667	Promoter(<=1kb)	644	OR8D4	338662
494208	11q	124038366	124038988	623	9	1	Promoter(<=1kb)	13	OR10G7	390265
494208	12p	4626568	4628152	1585	9	0.333	Exon(exon5of6)	14051	DYRK4	8798
494208	13q	25096877	25097182	306	11	0.364	Promoter(1-2kb)	1009	PABPC3	5042
494208	13q	102732474	102733933	1460	6	0.333	Exon(exon4of4)	25139	CCDC168	643677
494208	14q	19975713	19976448	736	9	0.444	Promoter(<=1kb)	269	OR4K15	81127
494208	14q	20060048	20060884	837	8	0.625	Promoter(<=1kb)	3	OR4L1	122742
494208	14q	20223729	20224484	756	7	0.571	Promoter(<=1kb)	19	OR11H6	122748
494208	14q	21634137	21634589	453	9	0.556	Promoter(<=1kb)	351	OR10G2	26534
494208	14q	70457520	70458540	1021	12	0.333	Exon(exon2of2)	5346	ADAM21	8747
494208	14q	104939262	104942618	3357	13	0.231	5'UTR	7102	PLD4	122618
494208	14q	104943622	104946886	3265	15	0.6	Exon(exon6of6)	8516	AHNAK2	113146
494208	14q	104947901	104953878	5978	36	0.5	Promoter(1-2kb)	1524	AHNAK2	113146
494208	15q	23439979	23442067	2089	10	0.6	5'UTR	5167	GOLGA6L2	283685
494208	15q	40621642	40624434	2793	8	0.375	Promoter(<=1kb)	0	KNL1	57082
494208	15q	78766033	78766671	639	8	0.625	Promoter(<=1kb)	-920	ADAMTS7	11173
494208	15q	85579423	85581800	2378	14	0.571	Promoter(1-2kb)	-1110	AKAP13	11214
494208	15q	100569472	100570097	626	7	0.714	Promoter(<=1kb)	534	LINS1	55180
494208	16p	1228744	1229622	879	6	0.833	Promoter(<=1kb)	540	TPSB2	64499
494208	16p	1486322	1488463	2142	12	0.75	Promoter(<=1kb)	4	PTX4	390667
494208	16p	4207130	4208004	875	6	0.5	Exon(exon2of7)	31739	SRL	6345
494208	16q	74391460	74392004	545	9	0.667	Exon(exon7of7)	13582	NPIPB15	440348
494208	16q	88428539	88429600	1062	6	0.333	Exon(exon3of3)	-23680	ZFPM1	161882
494208	16q	88430623	88432069	1447	6	0.333	Exon(exon3of3)	-21211	ZFPM1	161882
494208	16q	88433131	88436097	2967	6	0.667	Exon(exon3of3)	-17183	ZFPM1	161882
494208	16q	89226863	89228289	1427	8	0.625	Promoter(2-3kb)	2229	ZNF778	197320
494208	17p	10638198	10641099	2902	6	0.333	Exon(exon19of41)	-8169	MYH3	4621
494208	17p	21300581	21300978	398	12	0.75	3'UTR	9112	MAP2K3	5606
494208	17q	76291123	76294016	2894	11	0.455	Promoter(<=1kb)	0	QRICH2	84074
494208	17q	81645135	81645607	473	7	0.286	Promoter(2-3kb)	2722	TSPAN10	83882
494208	18p	9886991	9888072	1082	7	0.857	Promoter(<=1kb)	977	TXNDC2	84203
494208	18p	11609646	11610509	864	15	0.733	Promoter(<=1kb)	50	SLC35G4	646000
494208	18q	58535186	58537515	2330	10	0.4	Promoter(<=1kb)	0	ALPK2	115701
494208	19p	1004711	1005532	822	7	0.571	Exon(exon3of9)	4292	GRIN3B	116444
494208	19p	4510548	4513547	3000	23	0.522	Exon(exon3of6)	4157	PLIN4	729359
494208	19p	5455600	5456439	840	7	0.571	Promoter(<=1kb)	183	ZNRF4	148066
494208	19p	8936200	8939234	3035	11	0.909	Exon(exon5of84)	-40110	MUC16	94025
494208	19p	8946254	8951868	5615	23	0.522	Exon(exon3of84)	29474	MUC16	94025
494208	19p	8959116	8962299	3184	12	0.75	Exon(exon3of84)	19043	MUC16	94025
494208	19p	15794192	15794829	638	6	0.5	Promoter(<=1kb)	241	OR10H5	284433
494208	19p	18264798	18267409	2612	13	0.538	5'UTR	7002	IQCN	80726
494208	19p	21971930	21974500	2571	10	0.7	Exon(exon4of4)	14408	ZNF208	7757
494208	19p	23743906	23745300	1395	6	0.333	Exon(exon4of4)	13537	ZNF681	148213
494208	19q	36996640	36997597	958	11	0.636	Exon(exon10of10)	5587	ZNF568	374900
494208	19q	37885026	37888806	3781	7	0.571	Exon(exon6of6)	17788	WDR87	83889
494208	19q	39886240	39886422	183	6	0.667	Promoter(<=1kb)	870	FCGBP	8857
494208	19q	43966037	43967171	1135	6	0.167	Promoter(<=1kb)	-691	ZNF155	7711
494208	19q	44106512	44108078	1567	8	0.125	Exon(exon6of6)	-4103	ZNF225	7768
494208	19q	51745958	51746963	1006	6	0.333	Exon(exon3of3)	3848	FPR1	2357
494208	19q	52437918	52439242	1325	7	0.429	Exon(exon4of4)	6504	ZNF534	147658
494208	19q	55358736	55359651	916	6	0.667	Promoter(<=1kb)	0	FAM71E2	284418

494208	19q	55481625	55483456	1832	7	0.429	Promoter(1-2kb)	-1732	NAT14	57106
494208	19q	55911888	55913166	1279	7	0.714	Exon(exon5of12)	19145	NLRP13	126204
494208	20p	5922421	5923394	974	7	0.571	Exon(exon4of5)	6923	CHGB	1114
494208	20p	20052354	20052736	383	6	0.167	Promoter(<=1kb)	0	CFAP61	26074
494208	20q	63349752	63350772	1021	6	0.5	3'UTR	3794	CHRNA4	1137
494208	20q	63561666	63565531	3866	13	0.615	Promoter(<=1kb)	-61	HELZ2	85441
494208	21q	44637474	44638143	670	8	0.5	Promoter(<=1kb)	118	KRTAP10-10	353333
494208	21q	44666460	44666841	382	7	0.714	Promoter(<=1kb)	86	KRTAP12-2	353323
494208	22q	22352950	22353380	431	16	0.5	Exon(exon1of2)	30478	BMS1P20	96610
494208	22q	22704568	22704813	246	6	1	Exon(exon2of2)	57790	GGTLC2	91227
494208	22q	36191154	36191906	753	7	0.571	3'UTR	9971	APOL4	80832
494208	23p	3320126	3323750	3625	9	0.556	Exon(exon5of7)	22902	MXRA5	25878
494208	23p	8170039	8170141	103	6	0.5	Promoter(1-2kb)	1126	VCX2	51480
494208	23p	35802148	35803010	863	7	0.571	5'UTR	3357	MAGEB16	139604
494208	23q	136874183	136874416	234	6	1	Promoter(<=1kb)	-201	RBMX	27316
494208	23q	141906066	141906494	429	7	0.571	Promoter(1-2kb)	1264	MAGEC1	9947
494565	1p	12029085	12030097	1013	6	0.833	Promoter(<=1kb)	0	MIR6729	102466982
494565	1p	12847526	12847995	470	8	0.5	Promoter(<=1kb)	730	HNRNPCL1	343069
494565	1p	12859036	12860079	1044	6	0.333	Promoter(1-2kb)	1950	PRAMEF2	65122
494565	1p	13370686	13371119	434	7	0.429	Promoter(<=1kb)	781	PRAMEF19	645414
494565	1p	16058491	16060000	1510	13	0.846	Exon(exon5of7)	6168	CLCNKB	1188
494565	1p	18481403	18482217	815	7	0.714	Promoter(<=1kb)	421	KLHDC7A	127707
494565	1p	23874604	23875430	827	9	0.556	Exon(exon2of2)	-6310	FUCA1	2517
494565	1p	40067594	40067675	82	6	0	Promoter(<=1kb)	324	CAP1	10487
494565	1p	89186388	89186419	32	9	0.556	Promoter(<=1kb)	107	GBP4	115361
494565	1q	152213286	152213347	62	8	0.5	Exon(exon3of3)	10846	HRNR	388697
494565	1q	152219233	152221375	2143	16	0.75	Promoter(2-3kb)	2818	HRNR	388697
494565	1q	156669844	156670886	1043	6	1	Exon(exon4of4)	6521	NES	10763
494565	1q	169542317	169542882	566	6	0.167	Exon(exon13of25)	-26572	F5	2153
494565	1q	201206099	201209837	3739	12	0.583	Promoter(1-2kb)	1017	IGFN1	91156
494565	1q	223393517	223394466	950	6	0.667	Promoter(<=1kb)	102	CCDC185	164127
494565	1q	228315976	228318026	2051	6	0.5	Exon(exon50of81)	6492	OBSCN	84033
494565	1q	247841312	247841582	271	6	0.833	Promoter(<=1kb)	314	OR11L1	391189
494565	1q	247949325	247949759	435	8	0.375	Promoter(<=1kb)	467	OR2L8	391190
494565	1q	248273309	248273700	392	9	0.444	Promoter(<=1kb)	136	OR2T33	391195
494565	1q	248681658	248682198	541	7	0.571	Promoter(<=1kb)	130	OR14I1	401994
494565	2p	29071297	29073000	1704	7	0.714	Promoter(1-2kb)	1523	PCARE	388939
494565	2q	185789865	185794632	4768	10	0.8	Promoter(<=1kb)	0	FSIP2	401024
494565	2q	217847567	217848213	647	6	0.833	Exon(exon19of33)	-5407	TNS1	7145
494565	2q	233713134	233713664	531	8	0.75	Promoter(<=1kb)	142	UGT1A5	54579
494565	2q	238130416	238131546	1131	8	0.5	Promoter(1-2kb)	1468	ESPNL	339768
494565	2q	240041845	240042131	287	6	0.167	Downstream(2-3kb)	3941	OR6B3	150681
494565	3p	75736929	75738859	1931	10	0.6	Promoter(<=1kb)	0	MIR4273	100422955
494565	3q	98264413	98265098	686	7	0.571	Promoter(<=1kb)	128	OR5H6	79295
494565	3q	196947388	196948102	715	6	0.5	3'UTR	3828	PIGZ	80235
494565	4p	1394625	1395373	749	6	0.167	Exon(exon1of1)	9813	UVSSA	57654
494565	4p	5988383	5989749	1367	7	0.571	Promoter(<=1kb)	0	C4orf50	389197
494565	4p	6300792	6302360	1569	7	0.857	Exon(exon8of8)	6021	WFS1	7466
494565	4p	7433331	7434759	1429	8	0.875	Promoter(<=1kb)	171	PSAPL1	768239
494565	4p	8227004	8228508	1505	9	0.222	Promoter(<=1kb)	-24	SH3TC1	54436
494565	4q	154489498	154491312	1815	10	0.6	Promoter(<=1kb)	22	DCHS2	54798
494565	4q	185458217	185460011	1795	8	0.625	Promoter(<=1kb)	0	CCDC110	256309
494565	4q	186619481	186621601	2121	7	0.286	Exon(exon10of27)	-9411	FAT1	2195
494565	4q	186706638	186709436	2799	7	0.714	Exon(exon2of27)	14397	FAT1	2195
494565	5q	140848579	140850786	2208	15	0.533	Promoter(<=1kb)	807	PCDHA9	9752
494565	5q	141100771	141102758	1988	11	0.273	Promoter(<=1kb)	298	PCDHB3	56132
494565	5q	141174000	141175025	1026	7	0.714	Promoter(1-2kb)	1356	PCDHB7	56129
494565	5q	141183999	141184688	690	7	0.857	Promoter(2-3kb)	-2473	PCDHB9	56127
494565	5q	141955676	141957660	1985	6	0.667	Promoter(<=1kb)	-668	RNF14	9604
494565	5q	148826877	148828070	1194	6	1	Promoter(1-2kb)	1632	ADRB2	154
494565	5q	151565922	151568158	2237	9	0.778	Promoter(<=1kb)	786	FAT2	2196
494565	6p	16327099	16327849	751	9	0.556	Exon(exon8of9)	37154	GMPR	2766
494565	6p	46858771	46859502	732	8	0.5	Exon(exon17of21)	3802	ADGRF5	221395
494565	6q	159231899	159234370	2472	10	0.5	Exon(exon11of23)	13602	FNDC1	84624
494565	7p	56021087	56021209	123	6	0.5	Exon(exon2of7)	12947	PSPH	5723
494565	7q	100958977	100960873	1897	50	0.46	Promoter(1-2kb)	1012	MUC3A	4584
494565	7q	100991195	100993127	1933	10	0.6	Exon(exon5of15)	-19927	MUC12	10071
494565	7q	100995415	100995785	371	7	0.714	Exon(exon5of15)	-17269	MUC12	10071
494565	7q	101004440	101004836	397	6	0.833	Exon(exon5of15)	-8218	MUC12	10071
494565	8p	10607245	10608432	1188	8	0.5	Exon(exon4of4)	46711	RP1L1	94137
494565	8p	10609614	10610662	1049	8	0.5	Exon(exon4of4)	44481	RP1L1	94137
494565	8p	11331234	11332082	849	7	0.571	Promoter(<=1kb)	346	SLC35G5	83650
494565	8p	12137448	12138641	1194	6	0.833	Promoter(<=1kb)	436	USP17L2	377630
494565	8p	13021128	13022030	903	8	0.25	Exon(exon5of5)	9115	TRMT9B	57604
494565	8q	123651655	123652634	980	8	0.5	Promoter(<=1kb)	316	KLHL38	340359
494565	8q	138151548	138153046	1499	6	0.667	Promoter(<=1kb)	0	FAM135B	51059
494565	8q	143916360	143919209	2850	8	0.25	Exon(exon32of32)	20381	PLEC	5339

494565	8q	143921326	143924022	2697	9	0.667	Exon(exon31of32)	15568	PLEC	5339
494565	9p	116800	117934	1135	7	0.857	Promoter(<=1kb)	270	FOXD4	2298
494565	9p	34723747	34725050	1304	6	1	Exon(exon4of4)	4438	FAM205A	259308
494565	9p	39078723	39078846	124	6	0.667	Exon(exon22of24)	7302	CNTNAP3	79937
494565	9q	76175237	76175296	60	10	0.8	Exon(exon14of14)	-13343	PCSK5	5125
494565	9q	76705204	76707804	2601	7	0.429	Promoter(<=1kb)	146	PCA3	50652
494565	9q	87886533	87888536	2004	8	0.625	Exon(exon4of4)	3656	SPATA31E1	286234
494565	9q	104598545	104599318	774	9	0.556	Promoter(<=1kb)	95	OR13C5	138799
494565	9q	122553263	122554071	809	7	0.429	Promoter(<=1kb)	93	OR1N2	138882
494565	9q	122628595	122629398	804	6	0.333	Promoter(<=1kb)	175	OR1B1	347169
494565	9q	122749914	122750547	634	6	0.833	Promoter(<=1kb)	174	OR1L6	392390
494565	9q	135547960	135548795	836	6	0.667	Promoter(1-2kb)	1805	OBP2A	29991
494565	10q	46549378	46550744	1367	27	0.63	5'UTR	4786	GPRIN2	9721
494565	10q	49323559	49326331	2773	9	0.444	Exon(exon3of3)	24285	C10orf71	118461
494565	10q	89737450	89738561	1112	6	0	Exon(exon20of33)	13874	KIF20B	9585
494565	10q	122084988	122087840	2853	8	0.75	Exon(exon4of23)	-25190	TACC2	10579
494565	10q	128103129	128106213	3085	13	0.615	Promoter(<=1kb)	-1	MKI67	4288
494565	11p	244106	244197	92	8	0.5	Promoter(<=1kb)	-232	PSMD13	5719
494565	11p	308290	309127	838	6	0.5	Promoter(<=1kb)	0	IFITM2	10581
494565	11p	1246332	1247378	1047	6	0.5	Promoter(2-3kb)	2298	MUC5B-AS1	112577518
494565	11p	4946238	4946968	731	6	0.667	Promoter(<=1kb)	158	OR51A4	401666
494565	11p	5177978	5178478	501	6	0.167	Promoter(<=1kb)	186	OR52Z1	283110
494565	11p	5323451	5324256	806	6	0.5	Promoter(<=1kb)	41	OR51B2	79345
494565	11p	5389704	5390350	647	7	0.429	Promoter(<=1kb)	327	OR51M1	390059
494565	11p	5422212	5423123	912	10	0.7	Promoter(<=1kb)	101	OR51Q1	390061
494565	11p	5515185	5516015	831	6	0.333	Promoter(<=1kb)	684	UBQLNL	143630
494565	11p	5581045	5581738	694	8	0.375	Promoter(<=1kb)	168	OR52B6	340980
494565	11p	5841302	5841883	582	9	0.333	Promoter(<=1kb)	14	OR52E6	390078
494565	11p	5884818	5885061	244	7	0.429	Promoter(<=1kb)	547	OR52E4	390081
494565	11p	5986042	5986669	628	7	1	Promoter(<=1kb)	316	OR52L1	338751
494565	11p	11351961	11352736	776	10	0.3	Promoter(<=1kb)	514	CSNK2A3	283106
494565	11p	12293639	12294368	730	6	0.833	Exon(exon29of35)	6739	MICALCL	84953
494565	11p	18173280	18173901	622	6	0.333	Promoter(<=1kb)	443	MRGPRX4	117196
494565	11q	54603136	54603820	685	6	0.667	Promoter(<=1kb)	178	OR4C46	119749
494565	11q	58214757	58215722	966	7	0.286	Promoter(<=1kb)	12	OR1S1	219959
494565	11q	58402523	58403265	743	8	0.5	Promoter(<=1kb)	144	OR5B3	441608
494565	11q	85724687	85725825	1139	6	0.5	Promoter(<=1kb)	0	SYTL2	54843
494565	11q	123906790	123907324	535	6	0.667	Promoter(<=1kb)	644	OR8D4	338662
494565	12p	4626568	4627524	957	7	0.429	Exon(exon5of6)	14051	DYRK4	8798
494565	12p	6453119	6453670	552	6	0.667	Promoter(<=1kb)	633	TAPBPL	55080
494565	13q	25096713	25097682	970	14	0.5	Promoter(<=1kb)	845	PABPC3	5042
494565	13q	102732474	102733933	1460	6	0.333	Exon(exon4of4)	25139	CCDC168	643677
494565	14q	19975713	19976448	736	9	0.444	Promoter(<=1kb)	269	OR4K15	81127
494565	14q	20060048	20060884	837	8	0.625	Promoter(<=1kb)	3	OR4L1	122742
494565	14q	21634137	21634589	453	9	0.556	Promoter(<=1kb)	351	OR10G2	26534
494565	14q	22633879	22634450	572	9	0.333	Exon(exon2of2)	32212	ABHD4	63874
494565	14q	63599634	63599713	80	7	0.571	Exon(exon2of2)	41694	WDR89	112840
494565	14q	70457520	70458540	1021	13	0.385	Exon(exon2of2)	5346	ADAM21	8747
494565	14q	94587512	94587839	328	6	0.5	Exon(exon2of2)	-4219	SERPINA3	12
494565	14q	104175275	104177810	2536	6	0.333	Exon(exon12of15)	36235	KIF26A	26153
494565	14q	104939262	104942618	3357	10	0.2	5'UTR	7102	PLD4	122618
494565	14q	104943622	104945444	1823	8	0.625	Exon(exon6of6)	9958	AHNAK2	113146
494565	14q	104947901	104950338	2438	15	0.6	Exon(exon6of6)	5064	AHNAK2	113146
494565	15q	23439979	23442067	2089	13	0.615	5'UTR	5167	GOLGA6L2	283685
494565	15q	78765997	78766581	585	7	0.286	Promoter(<=1kb)	-884	ADAMTS7	11173
494565	15q	85579423	85581800	2378	14	0.571	Promoter(1-2kb)	-1110	AKAP13	11214
494565	15q	99129423	99132517	3095	7	0.429	Exon(exon4of5)	7225	TTC23	64927
494565	16p	1486371	1488463	2093	8	0.75	Promoter(<=1kb)	4	PTX4	390667
494565	16p	4207130	4208004	875	6	0.5	Exon(exon2of7)	31739	SRL	6345
494565	16q	74391650	74391928	279	7	0.714	Exon(exon7of7)	13772	NPIPB15	440348
494565	16q	88428539	88429600	1062	6	0.333	Exon(exon3of3)	-23680	ZFPM1	161882
494565	16q	89226863	89228390	1528	9	0.556	Promoter(2-3kb)	2229	ZNF778	197320
494565	17p	21300581	21300954	374	8	0.75	3'UTR	9112	MAP2K3	5606
494565	17p	21415470	21416370	901	11	0.727	Exon(exon3of3)	10334	KCNJ12	3768
494565	17q	41727098	41728331	1234	6	0.833	Promoter(1-2kb)	-1201	HAP1	9001
494565	17q	76293419	76294016	598	6	0.5	Promoter(2-3kb)	-2167	QRICH2	84074
494565	17q	81645135	81645417	283	6	0.333	Promoter(2-3kb)	2722	TSPAN10	83882
494565	18p	11609904	11610444	541	7	0.714	Promoter(<=1kb)	308	SLC35G4	646000
494565	18q	58535186	58537515	2330	9	0.333	Promoter(<=1kb)	0	ALPK2	115701
494565	19p	4510548	4513547	3000	21	0.571	Exon(exon3of6)	4157	PLIN4	729359
494565	19p	5455600	5456439	840	8	0.625	Promoter(<=1kb)	183	ZNRF4	148066
494565	19p	7899063	7900784	1722	7	0.714	Exon(exon4of4)	-3059	MAP2K7	5609
494565	19p	8333830	8334965	1136	6	0.667	Promoter(<=1kb)	0	KANK3	256949
494565	19p	8937644	8939234	1591	6	0.667	Exon(exon5of84)	-41554	MUC16	94025
494565	19p	8946313	8951868	5556	19	0.632	Exon(exon3of84)	29474	MUC16	94025
494565	19p	8959116	8962299	3184	11	0.727	Exon(exon3of84)	19043	MUC16	94025
494565	19p	8972751	8978096	5346	13	0.462	Exon(exon1of84)	3246	MUC16	94025

494565	19p	15087213	15088040	828	10	0.3	Promoter(<=1kb)	233	OR1I1	126370
494565	19p	15794192	15794719	528	6	0.333	Promoter(<=1kb)	241	OR10H5	284433
494565	19p	17281820	17284246	2427	9	0.556	Promoter(<=1kb)	0	ANKLE1	126549
494565	19p	18264753	18267409	2657	15	0.6	5'UTR	7002	IQCN	80726
494565	19p	21971930	21974500	2571	7	0.714	Exon(exon4of4)	14408	ZNF208	7757
494565	19p	23743906	23745300	1395	6	0.333	Exon(exon4of4)	13537	ZNF681	148213
494565	19q	34943334	34944685	1352	6	0.5	3'UTR	9723	ZNF30	90075
494565	19q	39877222	39877880	659	6	0.5	Exon(exon20of28)	9412	FCGBP	8857
494565	19q	39886240	39886439	200	7	0.571	Promoter(<=1kb)	853	FCGBP	8857
494565	19q	43846955	43848536	1582	7	0.857	3'UTR	13450	ZNF283	284349
494565	19q	43913423	43914878	1456	8	0.5	Exon(exon10of10)	4861	ZNF45	7596
494565	19q	52437918	52439242	1325	7	0.429	Exon(exon4of4)	6504	ZNF534	147658
494565	19q	55517821	55518642	822	8	1	Exon(exon14of14)	17661	SBK2	646643
494565	19q	55911888	55913077	1190	6	0.667	Exon(exon5of12)	19234	NLRP13	126204
494565	19q	57639918	57641641	1724	8	0.375	3'UTR	6693	ZNF211	10520
494565	19q	58368293	58368875	583	7	0.429	Exon(exon3of3)	-5445	ZNF497	162968
494565	20p	5922421	5923394	974	8	0.5	Exon(exon4of5)	6923	CHGB	1114
494565	20q	62812284	62813345	1062	7	0.857	Promoter(2-3kb)	-2899	COL9A3	1299
494565	20q	63349752	63350772	1021	6	0.5	3'UTR	3794	CHRNA4	1137
494565	20q	63561666	63567309	5644	16	0.688	Promoter(<=1kb)	-61	HELZ2	85441
494565	21q	44539312	44540035	724	7	0.714	Promoter(<=1kb)	160	KRTAP10-1	386677
494565	21q	44600627	44601692	1066	10	0.7	Promoter(<=1kb)	30	KRTAP10-7	386675
494565	21q	44637476	44638143	668	6	0.667	Promoter(<=1kb)	120	KRTAP10-10	353333
494565	22q	22352950	22353380	431	16	0.5	Exon(exon1of2)	30478	BMS1P20	96610
494565	22q	36191154	36191906	753	6	0.667	3'UTR	9971	APOL4	80832
494565	22q	36265284	36265796	513	6	1	Exon(exon6of6)	12120	APOL1	8542
494565	23q	136874183	136874416	234	8	0.75	Promoter(<=1kb)	-201	RBMX	27316
494728	1p	13369166	13369564	399	7	0.714	Promoter(2-3kb)	2336	PRAMEF19	645414
494728	1p	13370686	13370918	233	6	0.5	Promoter(<=1kb)	982	PRAMEF19	645414
494728	1p	16048038	16049824	1787	6	0.5	Promoter(<=1kb)	0	CLCNKB	1188
494728	1p	16058547	16060000	1454	7	0.857	Exon(exon5of7)	6224	CLCNKB	1188
494728	1p	23874604	23875430	827	8	0.5	Exon(exon2of2)	-6310	FUCA1	2517
494728	1p	40067594	40067675	82	6	0	Promoter(<=1kb)	324	CAP1	10487
494728	1p	89186388	89186419	32	9	0.556	Promoter(<=1kb)	107	GBP4	115361
494728	1q	152219233	152221375	2143	16	0.75	Promoter(2-3kb)	2818	HRNR	388697
494728	1q	158765805	158766655	851	6	0.5	Promoter(<=1kb)	47	OR6N1	128372
494728	1q	169542317	169542882	566	6	0.167	Exon(exon13of25)	-26572	F5	2153
494728	1q	214640144	214642954	2811	12	0.5	Exon(exon12of20)	-5013	CENPF	1063
494728	1q	214644872	214647181	2310	10	0.4	Promoter(<=1kb)	-786	CENPF	1063
494728	1q	247841312	247841582	271	6	0.833	Promoter(<=1kb)	314	OR11L1	391189
494728	1q	247949325	247949738	414	10	0.3	Promoter(<=1kb)	467	OR2L8	391190
494728	1q	248294677	248295458	782	7	0.857	Promoter(<=1kb)	142	OR2T12	127064
494728	2p	48580657	48582454	1798	7	0.571	Promoter(<=1kb)	0	STON1	11037
494728	2q	102351547	102351902	356	7	0.429	Exon(exon11of11)	-4027	IL18R1	8809
494728	2q	132783534	132785001	1468	7	0.429	Promoter(1-2kb)	-1511	NCKAP5	344148
494728	2q	185790999	185794632	3634	8	0.875	Promoter(<=1kb)	0	FSIP2	401024
494728	2q	185805377	185808170	2794	6	0.333	Promoter(<=1kb)	0	FSIP2	401024
494728	2q	219271337	219271649	313	6	0.667	Exon(exon4of4)	6227	TUBA4A	7277
494728	2q	219539659	219540599	941	6	0.667	Promoter(2-3kb)	2498	CHPF	79586
494728	3p	75736929	75739007	2079	14	0.643	Promoter(<=1kb)	0	MIR4273	100422955
494728	3q	98264413	98265098	686	7	0.571	Promoter(<=1kb)	128	OR5H6	79295
494728	4p	5988383	5989749	1367	7	0.571	Promoter(<=1kb)	0	C4orf50	389197
494728	4p	6300792	6302360	1569	7	0.857	Exon(exon8of8)	6021	WFS1	7466
494728	4p	8227004	8228508	1505	8	0.25	Promoter(<=1kb)	-24	SH3TC1	54436
494728	4q	185458217	185460011	1795	8	0.625	Promoter(<=1kb)	0	CCDC110	256309
494728	5q	140848579	140850786	2208	8	0.5	Promoter(<=1kb)	807	PCDHA9	9752
494728	5q	141174000	141175025	1026	6	0.833	Promoter(1-2kb)	1356	PCDHB7	56129
494728	5q	141246961	141247287	327	6	0.667	Promoter(1-2kb)	1566	PCDHB15	56121
494728	5q	141955356	141957660	2305	6	0.667	Promoter(<=1kb)	-668	RNF14	9604
494728	6p	1312843	1313745	903	6	0.5	Promoter(<=1kb)	745	FOXQ1	94234
494728	6p	46858771	46859502	732	8	0.5	Exon(exon17of21)	3802	ADGRF5	221395
494728	7p	12369637	12370736	1100	6	0.667	3'UTR	-13307	VWDE	221806
494728	7p	53035678	53036385	708	7	1	Promoter(<=1kb)	45	POM121L12	285877
494728	7q	100958721	100960873	2153	58	0.448	Promoter(<=1kb)	756	MUC3A	4584
494728	7q	100991195	100993127	1933	8	0.625	Exon(exon5of15)	-19927	MUC12	10071
494728	7q	100995575	100995785	211	6	0.833	Exon(exon5of15)	-17269	MUC12	10071
494728	7q	129126683	129127452	770	7	0.286	Exon(exon1of2)	-17255	TSPAN33	340348
494728	8p	8376561	8377198	638	6	1	Exon(exon2of5)	4549	PRAG1	157285
494728	8p	10607375	10610662	3288	13	0.538	Exon(exon4of4)	44481	RP1L1	94137
494728	8p	13021128	13022030	903	7	0.143	Exon(exon5of5)	9115	TRMT9B	57604
494728	8q	142664552	142665852	1301	6	0.833	Exon(exon2of2)	4118	JRK	8629
494728	9p	116800	117713	914	6	0.667	Promoter(<=1kb)	491	FOXD4	2298
494728	9p	39078723	39078846	124	6	0.667	Exon(exon22of24)	7302	CNTNAP3	79937
494728	9q	76175237	76175296	60	10	0.8	Exon(exon14of14)	-13343	PCSK5	5125
494728	9q	87886533	87888536	2004	6	0.5	Exon(exon4of4)	3656	SPATA31E1	286234
494728	9q	104598545	104599361	817	7	0.429	Promoter(<=1kb)	52	OR13C5	138799
494728	9q	122749914	122750547	634	6	0.833	Promoter(<=1kb)	174	OR1L6	392390

494728	9q	135484803	135487213	2411	9	0.333	Promoter(1-2kb)	1440	PPP1R26	9858
494728	10q	46549378	46550723	1346	25	0.64	Exon(exon3of3)	4807	GPRIN2	9721
494728	10q	49323169	49325192	2024	7	0.571	Exon(exon3of3)	23895	C10orf71	118461
494728	11p	244106	244197	92	8	0.5	Promoter(<=1kb)	-232	PSMD13	5719
494728	11p	1246095	1247378	1284	7	0.286	Promoter(2-3kb)	2298	MUC5B-AS1	112577518
494728	11p	1248397	1251628	3232	9	0.778	Promoter(<=1kb)	0	MUC5B-AS1	112577518
494728	11p	5177978	5178478	501	6	0.167	Promoter(<=1kb)	186	OR52Z1	283110
494728	11p	5323451	5324256	806	6	0.5	Promoter(<=1kb)	41	OR51B2	79345
494728	11p	5389704	5390350	647	7	0.429	Promoter(<=1kb)	327	OR51M1	390059
494728	11p	5422212	5423123	912	11	0.636	Promoter(<=1kb)	101	OR51Q1	390061
494728	11p	5515185	5515931	747	6	0.333	Promoter(<=1kb)	768	UBQLNL	143630
494728	11p	5581045	5581738	694	8	0.375	Promoter(<=1kb)	168	OR52B6	340980
494728	11p	5841302	5841883	582	9	0.333	Promoter(<=1kb)	14	OR52E6	390078
494728	11p	11351961	11352736	776	8	0.125	Promoter(<=1kb)	514	CSNK2A3	283106
494728	11p	18173280	18173901	622	6	0.333	Promoter(<=1kb)	443	MRGPRX4	117196
494728	11q	58214757	58215722	966	8	0.25	Promoter(<=1kb)	12	OR1S1	219959
494728	11q	82732630	82733184	555	6	0.833	Promoter(<=1kb)	680	FAM181B	220382
494728	11q	85724687	85725825	1139	6	0.5	Promoter(<=1kb)	0	SYTL2	54843
494728	11q	93697503	93700096	2594	12	0.667	Promoter(<=1kb)	0	CEP295	85459
494728	11q	123906595	123907324	730	7	0.429	Promoter(<=1kb)	449	OR8D4	338662
494728	11q	124015600	124016477	878	7	0.143	Promoter(<=1kb)	25	OR10G4	390264
494728	11q	124038366	124038988	623	9	1	Promoter(<=1kb)	13	OR10G7	390265
494728	11q	124382526	124383285	760	10	0.6	Promoter(<=1kb)	58	OR8B2	26595
494728	12p	4626568	4628549	1982	10	0.5	Exon(exon5of6)	14051	DYRK4	8798
494728	12p	6453119	6453670	552	6	0.667	Promoter(<=1kb)	633	TAPBPL	55080
494728	13q	24434450	24435347	898	7	0.571	Exon(exon31of34)	19787	PARP4	143
494728	13q	102732474	102733933	1460	6	0.333	Exon(exon4of4)	25139	CCDC168	643677
494728	14q	20143999	20144446	448	6	0.5	Promoter(<=1kb)	263	OR4N5	390437
494728	14q	20640982	20641567	586	6	0.5	Promoter(<=1kb)	124	OR6S1	341799
494728	14q	21634137	21634589	453	9	0.556	Promoter(<=1kb)	351	OR10G2	26534
494728	14q	70457520	70459101	1582	9	0.333	Exon(exon2of2)	5346	ADAM21	8747
494728	14q	94587512	94587839	328	6	0.5	Exon(exon2of2)	-4219	SERPINA3	12
494728	14q	104175275	104177810	2536	6	0.333	Exon(exon12of15)	36235	KIF26A	26153
494728	14q	104939605	104942618	3014	9	0.111	5'UTR	7445	PLD4	122618
494728	14q	104943622	104945444	1823	7	0.571	Exon(exon6of6)	9958	AHNAK2	113146
494728	14q	104947943	104953878	5936	32	0.469	Promoter(1-2kb)	1524	AHNAK2	113146
494728	15q	20534501	20535014	514	6	0.5	Exon(exon8of9)	6786	GOLGA6L6	727832
494728	15q	23439979	23442067	2089	14	0.5	5'UTR	5167	GOLGA6L2	283685
494728	15q	42690621	42692660	2040	6	0.5	Promoter(1-2kb)	1026	STARD9	57519
494728	15q	73702465	73703760	1296	6	0.833	Promoter(<=1kb)	-149	CD276	80381
494728	15q	85579423	85582073	2651	16	0.625	Promoter(<=1kb)	-837	AKAP13	11214
494728	15q	88857108	88859365	2258	6	0	Exon(exon12of18)	9865	ACAN	176
494728	15q	99129423	99132517	3095	6	0.333	Exon(exon4of5)	7225	TTC23	64927
494728	15q	100569472	100570097	626	7	0.714	Promoter(<=1kb)	534	LINS1	55180
494728	16p	1221890	1223490	1601	6	1	Promoter(1-2kb)	1285	TPSG1	25823
494728	16p	1228744	1229731	988	7	0.714	Promoter(<=1kb)	431	TPSB2	64499
494728	16q	88428539	88429600	1062	6	0.333	Exon(exon3of3)	-23680	ZFPM1	161882
494728	16q	88712207	88714717	2511	7	0.571	Promoter(<=1kb)	0	CTU2	348180
494728	16q	89100686	89101050	365	7	0.571	Promoter(<=1kb)	24	ACSF3	197322
494728	16q	89226863	89228289	1427	8	0.625	Promoter(2-3kb)	2229	ZNF778	197320
494728	17p	21300581	21300954	374	8	0.75	3'UTR	9112	MAP2K3	5606
494728	17q	76293419	76294016	598	6	0.5	Promoter(2-3kb)	-2167	QRICH2	84074
494728	17q	81645135	81645417	283	6	0.333	Promoter(2-3kb)	2722	TSPAN10	83882
494728	18p	11609646	11610491	846	14	0.786	Promoter(<=1kb)	50	SLC35G4	646000
494728	18q	58535186	58538030	2845	19	0.526	Promoter(<=1kb)	0	ALPK2	115701
494728	19p	4511338	4513547	2210	8	0.25	Exon(exon3of6)	4157	PLIN4	729359
494728	19p	5455600	5456439	840	6	0.5	Promoter(<=1kb)	183	ZNRF4	148066
494728	19p	8948231	8950136	1906	9	0.667	Exon(exon3of84)	31206	MUC16	94025
494728	19p	8959403	8962066	2664	8	0.5	Exon(exon3of84)	19276	MUC16	94025
494728	19p	8964274	8967127	2854	22	0.682	Exon(exon3of84)	14215	MUC16	94025
494728	19p	12430400	12431840	1441	8	0.5	3'UTR	9181	ZNF443	10224
494728	19p	14766987	14767045	59	7	0.857	Exon(exon6of21)	5329	ADGRE2	30817
494728	19p	18264753	18267409	2657	14	0.643	5'UTR	7002	IQCN	80726
494728	19p	21971930	21974500	2571	7	0.714	Exon(exon4of4)	14408	ZNF208	7757
494728	19q	33205448	33207385	1938	6	0.667	Promoter(1-2kb)	1578	LRP3	4037
494728	19q	43913423	43914878	1456	8	0.5	Exon(exon10of10)	4861	ZNF45	7596
494728	19q	43966037	43967171	1135	6	0.167	Promoter(<=1kb)	-691	ZNF155	7711
494728	19q	44106512	44108078	1567	7	0	Exon(exon6of6)	-4103	ZNF225	7768
494728	19q	48873985	48875925	1941	8	0.5	Promoter(1-2kb)	1564	PPP1R15A	23645
494728	19q	51872787	51873699	913	10	0.5	Promoter(<=1kb)	-940	ZNF577	84765
494728	19q	52437918	52439242	1325	7	0.429	Exon(exon4of4)	6504	ZNF534	147658
494728	19q	55773030	55773169	140	7	0.571	Exon(exon3of3)	3889	RFPL4AL1	729974
494728	19q	55911888	55913166	1279	8	0.75	Exon(exon5of12)	19145	NLRP13	126204
494728	19q	58368293	58368817	525	6	0.5	Exon(exon3of3)	-5445	ZNF497	162968
494728	20p	5922421	5923394	974	6	0.5	Exon(exon4of5)	6923	CHGB	1114
494728	20q	63349752	63350772	1021	6	0.5	3'UTR	3794	CHRNA4	1137
494728	21q	44637474	44638041	568	9	0.333	Promoter(<=1kb)	118	KRTAP10-10	353333

494728	22q	22352950	22353291	342	12	0.5	Exon(exon1of2)	30478	BMS1P20	96610
494728	22q	36191154	36191906	753	7	0.714	3'UTR	9971	APOL4	80832
494728	22q	49884187	49884994	808	6	0.333	Exon(exon2of2)	22924	ALG12	79087
494728	23p	8170039	8170141	103	6	0.5	Promoter(1-2kb)	1126	VCX2	51480
494728	23p	8466313	8466392	80	7	0.429	Promoter(<=1kb)	887	VCX3B	425054
494728	23p	35802148	35803010	863	7	0.571	5'UTR	3357	MAGEB16	139604
494728	23q	141906066	141906245	180	11	0.455	Promoter(1-2kb)	1264	MAGEC1	9947
495469	1p	12859108	12860212	1105	10	0.5	Promoter(2-3kb)	2022	PRAMEF2	65122
495469	1p	12861255	12861721	467	7	0.286	Exon(exon4of4)	4169	PRAMEF2	65122
495469	1p	16058491	16060000	1510	10	0.9	Exon(exon5of7)	6168	CLCNKB	1188
495469	1p	18481403	18482217	815	6	0.667	Promoter(<=1kb)	421	KLHDC7A	127707
495469	1p	23874604	23875430	827	9	0.556	Exon(exon2of2)	-6310	FUCA1	2517
495469	1p	40067594	40067675	82	6	0	Promoter(<=1kb)	324	CAP1	10487
495469	1p	89186388	89186419	32	9	0.556	Promoter(<=1kb)	107	GBP4	115361
495469	1q	152219233	152221375	2143	16	0.75	Promoter(2-3kb)	2818	HRNR	388697
495469	1q	201206099	201209954	3856	15	0.533	Promoter(1-2kb)	1017	IGFN1	91156
495469	1q	222628664	222630210	1547	6	0.333	Promoter(<=1kb)	190	MIA3	375056
495469	1q	232805117	232806800	1684	7	0.429	Promoter(<=1kb)	225	MAP10	54627
495469	1q	247841312	247841582	271	6	0.833	Promoter(<=1kb)	314	OR11L1	391189
495469	1q	247896121	247896502	382	6	0.5	Promoter(<=1kb)	534	OR2W3	343171
495469	1q	247949443	247949738	296	9	0.333	Promoter(<=1kb)	585	OR2L8	391190
495469	1q	248294677	248295458	782	7	0.571	Promoter(<=1kb)	142	OR2T12	127064
495469	2p	48580657	48582454	1798	7	0.571	Promoter(<=1kb)	0	STON1	11037
495469	2q	97547464	97547725	262	6	0.5	3'UTR	-35855	ANKRD36B	57730
495469	2q	130193975	130194465	491	7	0.714	Exon(exon4of5)	3974	TUBA3E	112714
495469	2q	132783032	132785012	1981	9	0.556	Promoter(1-2kb)	-1009	NCKAP5	344148
495469	2q	178739433	178741811	2379	6	0.5	Exon(exon45of191)	26014	TTN	7273
495469	2q	184936178	184937636	1459	6	0.333	Exon(exon4of4)	69813	ZNF804A	91752
495469	2q	185789865	185794632	4768	10	0.8	Promoter(<=1kb)	0	FSIP2	401024
495469	2q	219271337	219271649	313	6	0.667	Exon(exon4of4)	6227	TUBA4A	7277
495469	2q	238130416	238131546	1131	6	0.333	Promoter(1-2kb)	1468	ESPNL	339768
495469	3p	75736880	75739243	2364	59	0.576	Promoter(<=1kb)	0	MIR4273	100422955
495469	3q	194341097	194342571	1475	6	0.5	Exon(exon2of2)	8747	CPN2	1370
495469	3q	196947878	196948448	571	6	0.5	3'UTR	3482	PIGZ	80235
495469	4p	5988383	5989749	1367	7	0.571	Promoter(<=1kb)	0	C4orf50	389197
495469	4p	6300792	6302360	1569	6	0.833	Exon(exon8of8)	6021	WFS1	7466
495469	4p	8227004	8228508	1505	8	0.125	Promoter(<=1kb)	-24	SH3TC1	54436
495469	5q	79728956	79730716	1761	7	0.286	Exon(exon2of13)	-7426	CMYA5	202333
495469	5q	79731782	79734523	2742	13	0.308	Exon(exon2of13)	-3619	CMYA5	202333
495469	5q	140848579	140850786	2208	6	0.5	Promoter(<=1kb)	807	PCDHA9	9752
495469	5q	141174000	141175025	1026	6	0.833	Promoter(1-2kb)	1356	PCDHB7	56129
495469	5q	141183318	141184688	1371	7	1	Promoter(1-2kb)	1919	PCDHB16	57717
495469	5q	141955676	141957660	1985	6	0.667	Promoter(<=1kb)	-668	RNF14	9604
495469	6p	1312843	1313745	903	6	0.5	Promoter(<=1kb)	745	FOXQ1	94234
495469	6p	42745312	42746041	730	6	0.667	Promoter(<=1kb)	62	TBCC	6903
495469	6p	46858771	46859502	732	8	0.5	Exon(exon17of21)	3802	ADGRF5	221395
495469	6q	149888581	149890867	2287	7	0.714	Promoter(<=1kb)	0	RAET1E-AS1	100652739
495469	6q	159233455	159234370	916	10	0.5	Exon(exon11of23)	15158	FNDC1	84624
495469	7p	45082865	45084866	2002	7	0.429	Promoter(1-2kb)	-1465	NACAD	23148
495469	7p	53035678	53036385	708	7	1	Promoter(<=1kb)	45	POM121L12	285877
495469	7q	64991278	64992758	1481	7	0.714	Promoter(<=1kb)	-242	ZNF117	51351
495469	7q	100958977	100960873	1897	54	0.463	Promoter(1-2kb)	1012	MUC3A	4584
495469	7q	100991195	100992398	1204	7	0.571	Exon(exon5of15)	-20656	MUC12	10071
495469	7q	100995575	100995785	211	7	0.714	Exon(exon5of15)	-17269	MUC12	10071
495469	7q	101034361	101040583	6223	41	0.512	Exon(exon3of12)	-3128	MUC17	140453
495469	7q	149818015	149819792	1778	6	0.667	Promoter(2-3kb)	-2352	SSPO	23145
495469	8p	10607375	10608261	887	7	0.429	Exon(exon4of4)	46882	RP1L1	94137
495469	8p	12132686	12133940	1255	6	0.667	Promoter(<=1kb)	498	USP17L7	392197
495469	8p	12137553	12138641	1089	6	1	Promoter(<=1kb)	436	USP17L2	377630
495469	8p	13021128	13022030	903	7	0.143	Exon(exon5of5)	9115	TRMT9B	57604
495469	8q	123651655	123652634	980	7	0.571	Promoter(<=1kb)	316	KLHL38	340359
495469	8q	143870008	143872453	2446	6	0.5	Exon(exon2of2)	6011	EPPK1	83481
495469	8q	144108511	144110188	1678	6	0.5	Promoter(<=1kb)	0	WDR97	340390
495469	9p	21206764	21207038	275	6	0.667	Promoter(<=1kb)	105	IFNA10	3446
495469	9q	76705179	76707804	2626	8	0.5	Promoter(<=1kb)	121	PCA3	50652
495469	9q	76709523	76710843	1321	6	0.5	Promoter(<=1kb)	0	PRUNE2	158471
495469	9q	104598545	104599361	817	12	0.583	Promoter(<=1kb)	52	OR13C5	138799
495469	9q	122553263	122554071	809	8	0.5	Promoter(<=1kb)	93	OR1N2	138882
495469	9q	122628595	122629130	536	6	0.333	Promoter(<=1kb)	443	OR1B1	347169
495469	9q	122749914	122750547	634	6	0.833	Promoter(<=1kb)	174	OR1L6	392390
495469	9q	124855684	124856809	1126	7	0.571	Promoter(2-3kb)	2208	WDR38	401551
495469	9q	135484695	135486007	1313	6	0.333	Promoter(1-2kb)	1332	PPP1R26	9858
495469	10q	46549378	46550723	1346	26	0.654	Exon(exon3of3)	4807	GPRIN2	9721
495469	10q	49323169	49326817	3649	11	0.545	Exon(exon3of3)	23895	C10orf71	118461
495469	10q	128102594	128105201	2608	6	0.667	Promoter(<=1kb)	0	MKI67	4288
495469	11p	244106	244197	92	8	0.5	Promoter(<=1kb)	-232	PSMD13	5719
495469	11p	1194354	1196902	2549	8	0.625	Exon(exon34of49)	-26164	MUC5B	727897

495469	11p	1245785	1247378	1594	7	0.571	Promoter(2-3kb)	2298	MUC5B-AS1	112577518
495469	11p	5177978	5178478	501	6	0.167	Promoter(<=1kb)	186	OR52Z1	283110
495469	11p	5323362	5324256	895	7	0.429	Promoter(<=1kb)	41	OR51B2	79345
495469	11p	5389704	5390350	647	7	0.429	Promoter(<=1kb)	327	OR51M1	390059
495469	11p	5422212	5423123	912	11	0.636	Promoter(<=1kb)	101	OR51Q1	390061
495469	11p	5515185	5515931	747	6	0.333	Promoter(<=1kb)	768	UBQLNL	143630
495469	11p	5581045	5581738	694	9	0.333	Promoter(<=1kb)	168	OR52B6	340980
495469	11p	5841302	5841883	582	9	0.333	Promoter(<=1kb)	14	OR52E6	390078
495469	11p	5884818	5885061	244	7	0.429	Promoter(<=1kb)	547	OR52E4	390081
495469	11p	11351961	11352736	776	9	0.222	Promoter(<=1kb)	514	CSNK2A3	283106
495469	11p	12293639	12294368	730	6	0.833	Exon(exon29of35)	6739	MICALCL	84953
495469	11q	58402523	58403265	743	8	0.5	Promoter(<=1kb)	144	OR5B3	441608
495469	11q	64315797	64315856	60	8	0.75	Promoter(1-2kb)	1485	TRMT112	51504
495469	11q	85724687	85725825	1139	6	0.5	Promoter(<=1kb)	0	SYTL2	54843
495469	11q	123906595	123907324	730	9	0.444	Promoter(<=1kb)	449	OR8D4	338662
495469	11q	124015600	124016477	878	7	0.143	Promoter(<=1kb)	25	OR10G4	390264
495469	11q	124038366	124038988	623	9	1	Promoter(<=1kb)	13	OR10G7	390265
495469	11q	124382526	124383285	760	8	0.625	Promoter(<=1kb)	58	OR8B2	26595
495469	12p	4626568	4628549	1982	12	0.5	Exon(exon5of6)	14051	DYRK4	8798
495469	12p	8222174	8223514	1341	7	0.714	Exon(exon5of6)	4073	FAM90A1	55138
495469	12q	52316096	52317765	1670	6	0.667	Exon(exon4of9)	3633	KRT83	3889
495469	12q	52571389	52573652	2264	7	0.571	Promoter(<=1kb)	164	KRT74	121391
495469	12q	55247291	55248136	846	6	0.667	Promoter(<=1kb)	93	OR6C74	254783
495469	13q	25096675	25097182	508	23	0.522	Promoter(<=1kb)	807	PABPC3	5042
495469	13q	102732474	102733933	1460	6	0.333	Exon(exon4of4)	25139	CCDC168	643677
495469	14q	20640750	20641567	818	7	0.571	Promoter(<=1kb)	124	OR6S1	341799
495469	14q	21634137	21634589	453	9	0.556	Promoter(<=1kb)	351	OR10G2	26534
495469	14q	22633879	22634450	572	9	0.333	Exon(exon2of2)	32212	ABHD4	63874
495469	14q	70457520	70458948	1429	13	0.385	Exon(exon2of2)	5346	ADAM21	8747
495469	14q	94587512	94587839	328	6	0.5	Exon(exon2of2)	-4219	SERPINA3	12
495469	14q	104947943	104951938	3996	23	0.609	Exon(exon6of6)	3464	AHNAK2	113146
495469	15q	20534501	20535014	514	6	0.5	Exon(exon8of9)	6786	GOLGA6L6	727832
495469	15q	23439979	23442067	2089	11	0.545	5'UTR	5167	GOLGA6L2	283685
495469	15q	40356171	40356211	41	6	0.667	Promoter(2-3kb)	-2008	DISP2	85455
495469	15q	40621642	40623696	2055	6	0.333	Promoter(<=1kb)	0	KNL1	57082
495469	15q	78766033	78766581	549	6	0.667	Promoter(<=1kb)	-920	ADAMTS7	11173
495469	15q	85579207	85581800	2594	15	0.6	Promoter(1-2kb)	-1110	AKAP13	11214
495469	16p	1486322	1488362	2041	7	0.714	Promoter(<=1kb)	105	PTX4	390667
495469	16p	4207130	4208004	875	6	0.5	Exon(exon2of7)	31739	SRL	6345
495469	16q	74391401	74392004	604	11	0.636	Exon(exon7of7)	13523	NPIPB15	440348
495469	16q	89100686	89101050	365	7	0.571	Promoter(<=1kb)	24	ACSF3	197322
495469	16q	89226863	89228390	1528	10	0.6	Promoter(2-3kb)	2229	ZNF778	197320
495469	17p	21300581	21300978	398	12	0.75	3'UTR	9112	MAP2K3	5606
495469	17p	21415470	21416416	947	9	0.889	Exon(exon3of3)	10334	KCNJ12	3768
495469	17q	53823368	53824891	1524	6	0.833	Promoter(<=1kb)	441	KIF2B	84643
495469	17q	73236508	73236991	484	6	0.333	Promoter(<=1kb)	0	FAM104A	84923
495469	17q	76291123	76294016	2894	11	0.455	Promoter(<=1kb)	0	QRICH2	84074
495469	17q	81645135	81645607	473	7	0.286	Promoter(2-3kb)	2722	TSPAN10	83882
495469	18p	11609646	11610491	846	12	0.75	Promoter(<=1kb)	50	SLC35G4	646000
495469	18q	58535186	58537515	2330	9	0.333	Promoter(<=1kb)	0	ALPK2	115701
495469	19p	4510548	4513547	3000	21	0.476	Exon(exon3of6)	4157	PLIN4	729359
495469	19p	5455600	5456439	840	8	0.625	Promoter(<=1kb)	183	ZNRF4	148066
495469	19p	8948231	8950136	1906	6	0.667	Exon(exon3of84)	31206	MUC16	94025
495469	19p	8959518	8962066	2549	6	0.333	Exon(exon3of84)	19276	MUC16	94025
495469	19p	8964274	8967127	2854	19	0.632	Exon(exon3of84)	14215	MUC16	94025
495469	19p	12430718	12432437	1720	8	0.375	3'UTR	8584	ZNF443	10224
495469	19p	15087213	15088040	828	11	0.273	Promoter(<=1kb)	233	OR1I1	126370
495469	19p	17281820	17284246	2427	9	0.556	Promoter(<=1kb)	0	ANKLE1	126549
495469	19p	18264753	18267409	2657	8	0.5	5'UTR	7002	IQCN	80726
495469	19p	21971930	21974500	2571	7	0.714	Exon(exon4of4)	14408	ZNF208	7757
495469	19p	22756294	22759533	3240	15	0.533	3'UTR	10449	ZNF99	7652
495469	19q	43846955	43848536	1582	6	0.833	3'UTR	13450	ZNF283	284349
495469	19q	43913423	43914878	1456	7	0.429	Exon(exon10of10)	4861	ZNF45	7596
495469	19q	43996326	43997366	1041	6	0.5	Exon(exon5of5)	5419	LOC10192806[illegible]	101928063
495469	19q	44106512	44108078	1567	7	0	Exon(exon6of6)	-4103	ZNF225	7768
495469	19q	52365769	52366744	976	6	0.833	Exon(exon6of6)	-3173	ZNF880	400713
495469	19q	52437918	52439242	1325	7	0.429	Exon(exon4of4)	6504	ZNF534	147658
495469	19q	53164551	53166239	1689	6	0.167	Exon(exon4of4)	-5476	ZNF347	84671
495469	19q	55358400	55359651	1252	6	0.667	Promoter(<=1kb)	0	FAM71E2	284418
495469	19q	55481625	55483552	1928	7	0.571	Promoter(1-2kb)	-1636	NAT14	57106
495469	19q	55517821	55518642	822	9	1	Exon(exon14of14)	17661	SBK2	646643
495469	19q	55911888	55913077	1190	6	0.667	Exon(exon5of12)	19234	NLRP13	126204
495469	19q	58368293	58368875	583	7	0.429	Exon(exon3of3)	-5445	ZNF497	162968
495469	20p	5922677	5923382	706	7	0.571	Exon(exon4of5)	7179	CHGB	1114
495469	20p	20052354	20052736	383	6	0	Promoter(<=1kb)	0	CFAP61	26074
495469	20q	63562677	63565531	2855	10	0.7	Promoter(1-2kb)	-1072	HELZ2	85441
495469	21q	26843740	26844859	1120	6	0.667	Promoter(<=1kb)	0	ADAMTS1	9510

495469	21q	44550835	44551416	582	6	0.833	Promoter(<=1kb)	89	KRTAP10-2	386679
495469	22q	22352950	22353380	431	16	0.5	Exon(exon1of2)	30478	BMS1P20	96610
495469	22q	22646441	22646811	371	6	0.333	Promoter(<=1kb)	0	GGTLC2	91227
495469	22q	36191154	36191906	753	6	0.667	3'UTR	9971	APOL4	80832
495469	22q	39100331	39102033	1703	6	0.833	Promoter(<=1kb)	52	APOBEC3H	164668
495469	23p	8170039	8170141	103	6	0.5	Promoter(1-2kb)	1126	VCX2	51480
495469	23p	35802148	35803010	863	7	0.571	5'UTR	3357	MAGEB16	139604
495469	23q	136874183	136874416	234	8	0.75	Promoter(<=1kb)	-201	RBMX	27316
495930	1p	12847526	12847995	470	10	0.4	Promoter(<=1kb)	730	HNRNPCL1	343069
495930	1p	12859036	12860079	1044	6	0.333	Promoter(1-2kb)	1950	PRAMEF2	65122
495930	1p	13370686	13371119	434	7	0.429	Promoter(<=1kb)	781	PRAMEF19	645414
495930	1p	16058491	16060000	1510	10	0.9	Exon(exon5of7)	6168	CLCNKB	1188
495930	1p	18481403	18482217	815	7	0.714	Promoter(<=1kb)	421	KLHDC7A	127707
495930	1p	23874604	23875430	827	9	0.556	Exon(exon2of2)	-6310	FUCA1	2517
495930	1p	40067594	40067675	82	6	0	Promoter(<=1kb)	324	CAP1	10487
495930	1q	145872200	145873487	1288	6	0.667	Exon(exon8of12)	-12364	PIAS3	10401
495930	1q	152303673	152313891	10219	40	0.55	Promoter(<=1kb)	0	FLG-AS1	339400
495930	1q	156669844	156670886	1043	6	1	Exon(exon4of4)	6521	NES	10763
495930	1q	156907618	156909788	2171	6	0.333	Promoter(<=1kb)	-169	PEAR1	375033
495930	1q	158765805	158766655	851	6	0.5	Promoter(<=1kb)	47	OR6N1	128372
495930	1q	169542317	169542882	566	6	0.167	Exon(exon13of25)	-26572	F5	2153
495930	1q	201211017	201212095	1079	7	0.429	Promoter(<=1kb)	-547	IGFN1	91156
495930	1q	228315976	228318026	2051	6	0.5	Exon(exon50of81)	6492	OBSCN	84033
495930	1q	236553647	236555893	2247	6	0.333	Exon(exon39of45)	10351	LGALS8	3964
495930	1q	247841312	247841582	271	6	0.833	Promoter(<=1kb)	314	OR11L1	391189
495930	1q	247949325	247949738	414	10	0.3	Promoter(<=1kb)	467	OR2L8	391190
495930	2q	102351547	102351902	356	7	0.429	Exon(exon11of11)	-4027	IL18R1	8809
495930	2q	184936178	184937636	1459	6	0.333	Exon(exon4of4)	69813	ZNF804A	91752
495930	2q	217847583	217848746	1164	7	0.857	Exon(exon19of33)	-5423	TNS1	7145
495930	2q	219271337	219271649	313	6	0.667	Exon(exon4of4)	6227	TUBA4A	7277
495930	2q	233840612	233842185	1574	6	0.5	Promoter(<=1kb)	0	HJURP	55355
495930	2q	237762685	237764060	1376	10	0.5	Exon(exon8of8)	-4137	LRRFIP1	9208
495930	2q	238130416	238131702	1287	10	0.6	Promoter(1-2kb)	1468	ESPNL	339768
495930	3p	45967298	45968585	1288	6	0.833	Exon(exon8of19)	-8637	FYCO1	79443
495930	3p	75737230	75739007	1778	9	0.556	Promoter(<=1kb)	0	MIR4273	100422955
495930	3q	98264413	98265098	686	7	0.571	Promoter(<=1kb)	128	OR5H6	79295
495930	3q	194359607	194360906	1300	9	0.889	Exon(exon2of2)	-8279	CPN2	1370
495930	4p	5988383	5989749	1367	8	0.5	Promoter(<=1kb)	0	C4orf50	389197
495930	4p	6300792	6302360	1569	7	0.714	Exon(exon8of8)	6021	WFS1	7466
495930	4p	9175308	9175954	647	6	0.667	Exon(exon7of7)	4899	FAM90A26	100287045
495930	4q	186619481	186621582	2102	6	0.333	Exon(exon10of27)	-9411	FAT1	2195
495930	5q	79728956	79730716	1761	8	0.25	Exon(exon2of13)	-7426	CMYA5	202333
495930	5q	79731782	79734523	2742	13	0.308	Exon(exon2of13)	-3619	CMYA5	202333
495930	5q	83537326	83539905	2580	6	0.333	Promoter(1-2kb)	1712	VCAN	1462
495930	5q	140848579	140850786	2208	6	0.5	Promoter(<=1kb)	807	PCDHA9	9752
495930	5q	141174000	141175025	1026	6	0.833	Promoter(1-2kb)	1356	PCDHB7	56129
495930	5q	141183999	141184688	690	6	0.833	Promoter(2-3kb)	-2473	PCDHB9	56127
495930	5q	141955356	141957660	2305	6	0.667	Promoter(<=1kb)	-668	RNF14	9604
495930	5q	151565922	151568158	2237	9	0.778	Promoter(<=1kb)	786	FAT2	2196
495930	6p	46858771	46859502	732	8	0.5	Exon(exon17of21)	3802	ADGRF5	221395
495930	6q	159231899	159234370	2472	12	0.583	Exon(exon11of23)	13602	FNDC1	84624
495930	7p	6330446	6330944	499	6	1	Exon(exon2of2)	7749	FAM220A	84792
495930	7p	12369637	12370736	1100	6	0.667	3'UTR	-13307	VWDE	221806
495930	7p	45082865	45084866	2002	7	0.429	Promoter(1-2kb)	-1465	NACAD	23148
495930	7p	56021087	56021209	123	6	0.5	Exon(exon2of7)	12947	PSPH	5723
495930	7q	100958721	100960873	2153	58	0.448	Promoter(<=1kb)	756	MUC3A	4584
495930	7q	100991195	100992398	1204	8	0.625	Exon(exon5of15)	-20656	MUC12	10071
495930	7q	100995547	100996058	512	8	0.875	Exon(exon5of15)	-16996	MUC12	10071
495930	7q	101003685	101004836	1152	6	0.667	Exon(exon5of15)	-8218	MUC12	10071
495930	7q	101034305	101038994	4690	30	0.433	Exon(exon3of12)	-4717	MUC17	140453
495930	7q	149818015	149819792	1778	6	0.667	Promoter(2-3kb)	-2352	SSPO	23145
495930	8p	10607245	10612307	5063	16	0.75	Exon(exon4of4)	42836	RP1L1	94137
495930	8p	13021128	13022030	903	7	0.143	Exon(exon5of5)	9115	TRMT9B	57604
495930	9p	34723747	34726527	2781	9	0.778	Promoter(2-3kb)	2961	FAM205A	259308
495930	9p	39078723	39078846	124	6	0.667	Exon(exon22of24)	7302	CNTNAP3	79937
495930	9q	76175237	76175300	64	6	0.5	Exon(exon14of14)	-13339	PCSK5	5125
495930	9q	76705724	76707804	2081	6	0.5	Promoter(<=1kb)	666	PCA3	50652
495930	9q	87885490	87888819	3330	8	0.625	Promoter(2-3kb)	2613	SPATA31E1	286234
495930	9q	104598545	104599318	774	6	0.333	Promoter(<=1kb)	95	OR13C5	138799
495930	9q	122553263	122554071	809	8	0.5	Promoter(<=1kb)	93	OR1N2	138882
495930	9q	122628595	122629130	536	6	0.333	Promoter(<=1kb)	443	OR1B1	347169
495930	9q	122749914	122750547	634	6	0.833	Promoter(<=1kb)	174	OR1L6	392390
495930	9q	135484803	135487213	2411	10	0.4	Promoter(1-2kb)	1440	PPP1R26	9858
495930	10q	46549378	46550723	1346	25	0.64	Exon(exon3of3)	4807	GPRIN2	9721
495930	10q	49323169	49326817	3649	11	0.545	Exon(exon3of3)	23895	C10orf71	118461
495930	10q	122084988	122087840	2853	9	0.778	Exon(exon4of23)	-25190	TACC2	10579
495930	11p	244106	244197	92	8	0.5	Promoter(<=1kb)	-232	PSMD13	5719

495930	11p	1194354	1196902	2549	7	0.571	Exon(exon34of49)	-26164	MUC5B	727897
495930	11p	1246095	1247378	1284	7	0.286	Promoter(2-3kb)	2298	MUC5B-AS1	112577518
495930	11p	1250091	1251628	1538	6	0.833	Promoter(<=1kb)	-415	MUC5B-AS1	112577518
495930	11p	5177978	5178478	501	6	0.167	Promoter(<=1kb)	186	OR52Z1	283110
495930	11p	5323542	5324256	715	6	0.5	Promoter(<=1kb)	41	OR51B2	79345
495930	11p	5389704	5390350	647	7	0.429	Promoter(<=1kb)	327	OR51M1	390059
495930	11p	5422212	5423123	912	10	0.7	Promoter(<=1kb)	101	OR51Q1	390061
495930	11p	5515185	5516015	831	6	0.333	Promoter(<=1kb)	684	UBQLNL	143630
495930	11p	5581045	5581738	694	8	0.375	Promoter(<=1kb)	168	OR52B6	340980
495930	11p	5788000	5788760	761	8	0.75	Promoter(<=1kb)	56	OR52N1	79473
495930	11p	5841302	5841883	582	9	0.333	Promoter(<=1kb)	14	OR52E6	390078
495930	11p	7000807	7001486	680	6	0	3'UTR	18749	ZNF214	7761
495930	11p	11351961	11352736	776	9	0.222	Promoter(<=1kb)	514	CSNK2A3	283106
495930	11p	12293639	12294842	1204	7	0.714	Exon(exon29of35)	6739	MICALCL	84953
495930	11p	18173280	18173901	622	7	0.429	Promoter(<=1kb)	443	MRGPRX4	117196
495930	11q	58214757	58215722	966	8	0.25	Promoter(<=1kb)	12	OR1S1	219959
495930	11q	64116513	64118232	1720	7	0.714	Exon(exon2of2)	8702	MACROD1	28992
495930	11q	85724687	85725825	1139	6	0.5	Promoter(<=1kb)	0	SYTL2	54843
495930	11q	123906790	123907324	535	6	0.667	Promoter(<=1kb)	644	OR8D4	338662
495930	11q	124038366	124038988	623	7	1	Promoter(<=1kb)	13	OR10G7	390265
495930	11q	124382526	124383285	760	8	0.625	Promoter(<=1kb)	58	OR8B2	26595
495930	12p	4626571	4628549	1979	8	0.5	Exon(exon5of6)	14054	DYRK4	8798
495930	12p	6018725	6019629	905	6	0.5	Promoter(2-3kb)	2297	VWF	7450
495930	12p	6453119	6453670	552	7	0.714	Promoter(<=1kb)	633	TAPBPL	55080
495930	13q	24434450	24435347	898	7	0.571	Exon(exon31of34)	19787	PARP4	143
495930	13q	25096659	25097682	1024	19	0.474	Promoter(<=1kb)	791	PABPC3	5042
495930	13q	102732474	102733933	1460	6	0.333	Exon(exon4of4)	25139	CCDC168	643677
495930	14q	20060048	20060884	837	8	0.625	Promoter(<=1kb)	3	OR4L1	122742
495930	14q	21634137	21634589	453	9	0.556	Promoter(<=1kb)	351	OR10G2	26534
495930	14q	70457532	70458540	1009	9	0.333	Exon(exon2of2)	5358	ADAM21	8747
495930	15q	20534480	20534954	475	6	0.333	Exon(exon8of9)	6846	GOLGA6L6	727832
495930	15q	23439979	23442067	2089	8	0.625	5'UTR	5167	GOLGA6L2	283685
495930	15q	40621642	40623696	2055	6	0.333	Promoter(<=1kb)	0	KNL1	57082
495930	15q	85579423	85581800	2378	15	0.6	Promoter(1-2kb)	-1110	AKAP13	11214
495930	15q	99129423	99132517	3095	7	0.429	Exon(exon4of5)	7225	TTC23	64927
495930	16p	789084	790597	1514	6	0.167	Promoter(<=1kb)	0	CHTF18	63922
495930	16q	88428539	88429600	1062	6	0.333	Exon(exon3of3)	-23680	ZFPM1	161882
495930	16q	89100686	89101050	365	8	0.625	Promoter(<=1kb)	24	ACSF3	197322
495930	16q	89226863	89228419	1557	10	0.6	Promoter(2-3kb)	2229	ZNF778	197320
495930	17p	744946	746966	2021	7	1	3'UTR	5072	GEMIN4	50628
495930	17p	10638198	10641099	2902	7	0.286	Exon(exon19of41)	-8169	MYH3	4621
495930	17p	21300581	21300978	398	12	0.75	3'UTR	9112	MAP2K3	5606
495930	17q	76291123	76294016	2894	11	0.455	Promoter(<=1kb)	0	QRICH2	84074
495930	17q	81645135	81645417	283	6	0.333	Promoter(2-3kb)	2722	TSPAN10	83882
495930	18p	11609646	11610350	705	14	0.857	Promoter(<=1kb)	50	SLC35G4	646000
495930	18p	14542649	14543140	492	6	0.5	Promoter(<=1kb)	6	POTEC	388468
495930	18q	58535186	58538030	2845	19	0.526	Promoter(<=1kb)	0	ALPK2	115701
495930	19p	1004688	1005532	845	8	0.5	Exon(exon3of9)	4269	GRIN3B	116444
495930	19p	1036445	1036914	470	7	0.571	Exon(exon6of7)	-3187	ABCA7	10347
495930	19p	4510548	4513547	3000	22	0.545	Exon(exon3of6)	4157	PLIN4	729359
495930	19p	5455600	5456439	840	7	0.571	Promoter(<=1kb)	183	ZNRF4	148066
495930	19p	8972751	8978096	5346	13	0.462	Exon(exon1of84)	3246	MUC16	94025
495930	19p	12430157	12432437	2281	9	0.333	3'UTR	8584	ZNF443	10224
495930	19p	17281820	17284246	2427	9	0.556	Promoter(<=1kb)	0	ANKLE1	126549
495930	19p	18264753	18267409	2657	14	0.643	5'UTR	7002	IQCN	80726
495930	19p	21971930	21974500	2571	9	0.667	Exon(exon4of4)	14408	ZNF208	7757
495930	19p	23743906	23745300	1395	6	0.333	Exon(exon4of4)	13537	ZNF681	148213
495930	19q	39886005	39886422	418	8	0.625	Promoter(<=1kb)	870	FCGBP	8857
495930	19q	40880231	40880589	359	6	0.333	Promoter(<=1kb)	-141	CYP2A7	1549
495930	19q	43203948	43205504	1557	6	0.667	Promoter(<=1kb)	0	PSG4	5672
495930	19q	43913423	43914878	1456	9	0.556	Exon(exon10of10)	4861	ZNF45	7596
495930	19q	43996326	43997366	1041	6	0.5	Exon(exon5of5)	5419	LOC10192806:	101928063
495930	19q	44106512	44108078	1567	7	0	Exon(exon6of6)	-4103	ZNF225	7768
495930	19q	48873327	48875925	2599	10	0.6	Promoter(<=1kb)	906	PPP1R15A	23645
495930	19q	52437918	52439242	1325	7	0.429	Exon(exon4of4)	6504	ZNF534	147658
495930	19q	55911888	55913077	1190	6	0.667	Exon(exon5of12)	19234	NLRP13	126204
495930	19q	56663753	56665114	1362	6	0.5	Exon(exon2of2)	6643	ZNF835	90485
495930	19q	58368293	58368875	583	7	0.429	Exon(exon3of3)	-5445	ZNF497	162968
495930	20p	5922421	5923394	974	6	0.5	Exon(exon4of5)	6923	CHGB	1114
495930	20p	20052354	20052736	383	6	0.167	Promoter(<=1kb)	0	CFAP61	26074
495930	20q	63561666	63565531	3866	11	0.636	Promoter(<=1kb)	-61	HELZ2	85441
495930	21q	26843740	26844859	1120	6	0.667	Promoter(<=1kb)	0	ADAMTS1	9510
495930	21q	44637474	44638143	670	10	0.4	Promoter(<=1kb)	118	KRTAP10-10	353333
495930	22q	22352950	22353380	431	16	0.5	Exon(exon1of2)	30478	BMS1P20	96610
495930	22q	36191154	36191906	753	7	0.571	3'UTR	9971	APOL4	80832
495930	23p	8170039	8170141	103	6	0.5	Promoter(1-2kb)	1126	VCX2	51480
495930	23q	136874183	136874416	234	8	0.75	Promoter(<=1kb)	-201	RBMX	27316

495995	1p	978953	980691	1739	7	0.571	Promoter(<=1kb)	338	PERM1	84808
495995	1p	12859108	12860212	1105	10	0.5	Promoter(2-3kb)	2022	PRAMEF2	65122
495995	1p	12861255	12861721	467	7	0.286	Exon(exon4of4)	4169	PRAMEF2	65122
495995	1p	16058491	16060000	1510	10	0.9	Exon(exon5of7)	6168	CLCNKB	1188
495995	1p	23874604	23875430	827	9	0.556	Exon(exon2of2)	-6310	FUCA1	2517
495995	1p	40067594	40067675	82	6	0	Promoter(<=1kb)	324	CAP1	10487
495995	1p	89186388	89186419	32	9	0.556	Promoter(<=1kb)	107	GBP4	115361
495995	1q	152303673	152314394	10722	42	0.595	Promoter(<=1kb)	0	FLG-AS1	339400
495995	1q	158765805	158766655	851	6	0.5	Promoter(<=1kb)	47	OR6N1	128372
495995	1q	201206099	201209342	3244	10	0.7	Promoter(1-2kb)	1017	IGFN1	91156
495995	1q	201210956	201212792	1837	11	0.455	Promoter(<=1kb)	0	IGFN1	91156
495995	1q	223393517	223394466	950	6	0.667	Promoter(<=1kb)	102	CCDC185	164127
495995	1q	247841312	247841582	271	6	0.833	Promoter(<=1kb)	314	OR11L1	391189
495995	1q	247949325	247949738	414	10	0.3	Promoter(<=1kb)	467	OR2L8	391190
495995	1q	248294677	248295458	782	7	0.571	Promoter(<=1kb)	142	OR2T12	127064
495995	2p	29071763	29073000	1238	6	0.667	Promoter(1-2kb)	1523	PCARE	388939
495995	2q	130193975	130194376	402	6	0.5	Exon(exon4of5)	4063	TUBA3E	112714
495995	2q	178739433	178741811	2379	6	0.5	Exon(exon45of191)	26014	TTN	7273
495995	2q	185789591	185794632	5042	11	0.727	Promoter(<=1kb)	0	FSIP2	401024
495995	2q	217847583	217848559	977	6	0.833	Exon(exon19of33)	-5423	TNS1	7145
495995	2q	233681970	233682693	724	6	0.667	Promoter(<=1kb)	0	UGT1A7	54577
495995	2q	233840612	233842185	1574	7	0.571	Promoter(<=1kb)	0	HJURP	55355
495995	2q	238130416	238131702	1287	9	0.667	Promoter(1-2kb)	1468	ESPNL	339768
495995	3p	75736880	75739243	2364	54	0.611	Promoter(<=1kb)	0	MIR4273	100422955
495995	3q	98169021	98169594	574	6	0.667	Exon(exon2of2)	19695	OR5H14	403273
495995	3q	98264413	98265137	725	8	0.625	Promoter(<=1kb)	128	OR5H6	79295
495995	3q	194359607	194360906	1300	9	0.889	Exon(exon2of2)	-8279	CPN2	1370
495995	4p	5988383	5989749	1367	7	0.571	Promoter(<=1kb)	0	C4orf50	389197
495995	4p	6300792	6302360	1569	7	0.857	Exon(exon8of8)	6021	WFS1	7466
495995	4q	121036404	121037542	1139	6	0.333	Promoter(1-2kb)	1442	NDNF	79625
495995	4q	154489670	154491312	1643	7	0.429	Promoter(<=1kb)	22	DCHS2	54798
495995	4q	185458217	185460011	1795	9	0.556	Promoter(<=1kb)	0	CCDC110	256309
495995	5q	79728956	79734523	5568	19	0.316	Exon(exon2of13)	-3619	CMYA5	202333
495995	5q	83537326	83539905	2580	6	0.333	Promoter(1-2kb)	1712	VCAN	1462
495995	5q	140848579	140850786	2208	7	0.571	Promoter(<=1kb)	807	PCDHA9	9752
495995	5q	141174000	141175025	1026	10	0.9	Promoter(1-2kb)	1356	PCDHB7	56129
495995	5q	141178745	141180333	1589	11	0.727	Promoter(<=1kb)	955	PCDHB8	56128
495995	5q	141183598	141184688	1091	7	0.857	Promoter(2-3kb)	2199	PCDHB16	57717
495995	5q	141187690	141189425	1736	8	0.25	Promoter(<=1kb)	529	PCDHB9	56127
495995	5q	141246961	141247287	327	6	0.667	Promoter(1-2kb)	1566	PCDHB15	56121
495995	5q	141955676	141957660	1985	6	0.667	Promoter(<=1kb)	-668	RNF14	9604
495995	5q	151565922	151568158	2237	9	0.778	Promoter(<=1kb)	786	FAT2	2196
495995	6p	1312843	1313745	903	6	0.5	Promoter(<=1kb)	745	FOXQ1	94234
495995	6p	26370344	26370479	136	7	0.571	Promoter(<=1kb)	0	BTN3A2	11118
495995	6p	46858771	46859389	619	6	0.5	Exon(exon17of21)	3915	ADGRF5	221395
495995	6q	149888581	149890867	2287	7	0.714	Promoter(<=1kb)	0	RAET1E-AS1	100652739
495995	6q	159233455	159234370	916	10	0.5	Exon(exon11of23)	15158	FNDC1	84624
495995	7p	12369637	12370736	1100	6	0.667	3'UTR	-13307	VWDE	221806
495995	7q	64991321	64992758	1438	6	0.667	Promoter(<=1kb)	-285	ZNF117	51351
495995	7q	100958721	100960873	2153	60	0.433	Promoter(<=1kb)	756	MUC3A	4584
495995	7q	100991195	100994057	2863	11	0.727	Exon(exon5of15)	-18997	MUC12	10071
495995	7q	100995547	100995785	239	7	0.857	Exon(exon5of15)	-17269	MUC12	10071
495995	7q	101003685	101004836	1152	6	0.667	Exon(exon5of15)	-8218	MUC12	10071
495995	8p	10607245	10610662	3418	15	0.6	Exon(exon4of4)	44481	RP1L1	94137
495995	8p	13021128	13022030	903	7	0.143	Exon(exon5of5)	9115	TRMT9B	57604
495995	8q	123651873	123652634	762	6	0.333	Promoter(<=1kb)	316	KLHL38	340359
495995	8q	142664552	142665852	1301	6	0.833	Exon(exon2of2)	4118	JRK	8629
495995	8q	143916360	143919209	2850	7	0.143	Exon(exon32of32)	20381	PLEC	5339
495995	9q	76175237	76175296	60	10	0.8	Exon(exon14of14)	-13343	PCSK5	5125
495995	9q	76705724	76707804	2081	6	0.5	Promoter(<=1kb)	666	PCA3	50652
495995	9q	87885490	87888819	3330	8	0.625	Promoter(2-3kb)	2613	SPATA31E1	286234
495995	9q	104598641	104599361	721	9	0.556	Promoter(<=1kb)	52	OR13C5	138799
495995	9q	122553263	122554071	809	8	0.5	Promoter(<=1kb)	93	OR1N2	138882
495995	9q	122628595	122629398	804	7	0.286	Promoter(<=1kb)	175	OR1B1	347169
495995	9q	122749914	122750547	634	6	0.833	Promoter(<=1kb)	174	OR1L6	392390
495995	9q	131474936	131475983	1048	6	0.667	Promoter(<=1kb)	0	PRRC2B	84726
495995	9q	135547960	135548795	836	6	0.667	Promoter(1-2kb)	1805	OBP2A	29991
495995	10q	46549378	46550723	1346	26	0.654	Exon(exon3of3)	4807	GPRIN2	9721
495995	10q	49323169	49326817	3649	12	0.583	Exon(exon3of3)	23895	C10orf71	118461
495995	10q	122084548	122087840	3293	11	0.818	Exon(exon4of23)	-25190	TACC2	10579
495995	10q	128103129	128106296	3168	14	0.643	Promoter(<=1kb)	-1	MKI67	4288
495995	11p	244106	244197	92	8	0.5	Promoter(<=1kb)	-232	PSMD13	5719
495995	11p	1194354	1196902	2549	7	0.571	Exon(exon34of49)	-26164	MUC5B	727897
495995	11p	1241082	1245331	4250	12	0.75	Exon(exon31of49)	4345	MUC5B-AS1	112577518
495995	11p	1250091	1251524	1434	6	0.667	Promoter(<=1kb)	-415	MUC5B-AS1	112577518
495995	11p	5177978	5178478	501	6	0.167	Promoter(<=1kb)	186	OR52Z1	283110
495995	11p	5323451	5324256	806	6	0.5	Promoter(<=1kb)	41	OR51B2	79345

495995	11p	5389704	5390350	647	7	0.429	Promoter(<=1kb)	327	OR51M1	390059
495995	11p	5422212	5423123	912	10	0.7	Promoter(<=1kb)	101	OR51Q1	390061
495995	11p	5581045	5581738	694	8	0.375	Promoter(<=1kb)	168	OR52B6	340980
495995	11p	5841302	5841883	582	9	0.333	Promoter(<=1kb)	14	OR52E6	390078
495995	11p	5884818	5885061	244	7	0.429	Promoter(<=1kb)	547	OR52E4	390081
495995	11p	11351961	11352736	776	8	0.25	Promoter(<=1kb)	514	CSNK2A3	283106
495995	11p	12293639	12294368	730	8	0.875	Exon(exon29of35)	6739	MICALCL	84953
495995	11p	18173280	18173901	622	6	0.333	Promoter(<=1kb)	443	MRGPRX4	117196
495995	11p	34916266	34916763	498	6	0.667	Promoter(<=1kb)	0	APIP	51074
495995	11q	55572176	55572903	728	6	0.667	Promoter(<=1kb)	48	OR4C16	219428
495995	11q	58214757	58215722	966	8	0.25	Promoter(<=1kb)	12	OR1S1	219959
495995	11q	58402523	58403265	743	9	0.556	Promoter(<=1kb)	144	OR5B3	441608
495995	11q	64315797	64315856	60	8	0.75	Promoter(1-2kb)	1485	TRMT112	51504
495995	11q	82732630	82733184	555	6	0.833	Promoter(<=1kb)	680	FAM181B	220382
495995	11q	85724687	85725825	1139	6	0.5	Promoter(<=1kb)	0	SYTL2	54843
495995	11q	93697503	93700096	2594	12	0.667	Promoter(<=1kb)	0	CEP295	85459
495995	12p	4626571	4628549	1979	9	0.556	Exon(exon5of6)	14054	DYRK4	8798
495995	12p	6453119	6453670	552	6	0.667	Promoter(<=1kb)	633	TAPBPL	55080
495995	12p	8222174	8223514	1341	6	0.667	Exon(exon5of6)	4073	FAM90A1	55138
495995	13q	24434450	24435347	898	7	0.571	Exon(exon31of34)	19787	PARP4	143
495995	13q	49668389	49668447	59	7	0.286	Exon(exon3of3)	22991	EBPL	84650
495995	13q	102732474	102733933	1460	6	0.333	Exon(exon4of4)	25139	CCDC168	643677
495995	14q	20060048	20060884	837	8	0.625	Promoter(<=1kb)	3	OR4L1	122742
495995	14q	21634137	21634589	453	9	0.556	Promoter(<=1kb)	351	OR10G2	26534
495995	14q	70457520	70458540	1021	13	0.385	Exon(exon2of2)	5346	ADAM21	8747
495995	14q	104939262	104954713	15452	68	0.485	Promoter(<=1kb)	689	AHNAK2	113146
495995	15q	23439979	23442067	2089	13	0.538	5'UTR	5167	GOLGA6L2	283685
495995	15q	79456261	79457965	1705	6	0.5	Promoter(<=1kb)	165	MINAR1	23251
495995	15q	85579423	85582073	2651	14	0.571	Promoter(<=1kb)	-837	AKAP13	11214
495995	15q	88856792	88859365	2574	10	0.3	Exon(exon12of18)	9549	ACAN	176
495995	15q	99129423	99132517	3095	6	0.333	Exon(exon4of5)	7225	TTC23	64927
495995	15q	100569472	100570060	589	6	0.667	Promoter(<=1kb)	571	LINS1	55180
495995	16p	789084	790597	1514	7	0.286	Promoter(<=1kb)	0	CHTF18	63922
495995	16p	1228744	1229622	879	6	0.833	Promoter(<=1kb)	540	TPSB2	64499
495995	16p	1486371	1488463	2093	8	0.75	Promoter(<=1kb)	4	PTX4	390667
495995	16q	88714632	88717113	2482	8	0.5	Promoter(<=1kb)	0	MIR4722	100616167
495995	16q	89226863	89228289	1427	8	0.625	Promoter(2-3kb)	2229	ZNF778	197320
495995	17p	21295710	21296172	463	6	0.333	5'UTR	4241	MAP2K3	5606
495995	17p	21300581	21300954	374	11	0.727	3'UTR	9112	MAP2K3	5606
495995	17q	76293419	76294016	598	6	0.5	Promoter(2-3kb)	-2167	QRICH2	84074
495995	17q	81645135	81645607	473	7	0.286	Promoter(2-3kb)	2722	TSPAN10	83882
495995	18p	11609728	11610164	437	11	0.636	Promoter(<=1kb)	132	SLC35G4	646000
495995	18p	11644365	11644598	234	6	1	Exon(exon1of1)	10343	MIR7153	102465690
495995	18q	58535186	58538030	2845	18	0.556	Promoter(<=1kb)	0	ALPK2	115701
495995	19p	1004688	1005532	845	6	0.5	Exon(exon3of9)	4269	GRIN3B	116444
495995	19p	1036445	1036914	470	7	0.571	Exon(exon6of7)	-3187	ABCA7	10347
495995	19p	4510548	4513547	3000	22	0.455	Exon(exon3of6)	4157	PLIN4	729359
495995	19p	8937419	8939234	1816	7	0.714	Exon(exon5of84)	-41329	MUC16	94025
495995	19p	8946313	8951868	5556	19	0.632	Exon(exon3of84)	29474	MUC16	94025
495995	19p	8959116	8962299	3184	11	0.727	Exon(exon3of84)	19043	MUC16	94025
495995	19p	8971838	8978096	6259	15	0.533	Exon(exon1of84)	3246	MUC16	94025
495995	19p	12430157	12432437	2281	9	0.333	3'UTR	8584	ZNF443	10224
495995	19p	15087213	15088040	828	9	0.333	Promoter(<=1kb)	233	OR1I1	126370
495995	19p	17281820	17284246	2427	9	0.556	Promoter(<=1kb)	0	ANKLE1	126549
495995	19p	18264798	18267409	2612	12	0.583	5'UTR	7002	IQCN	80726
495995	19p	21971930	21974500	2571	7	0.714	Exon(exon4of4)	14408	ZNF208	7757
495995	19p	22756294	22759533	3240	14	0.5	3'UTR	10449	ZNF99	7652
495995	19q	36996730	36997597	868	10	0.7	Exon(exon10of10)	5677	ZNF568	374900
495995	19q	37151928	37153149	1222	6	0.833	Exon(exon5of5)	19287	ZNF585A	199704
495995	19q	40880231	40880622	392	6	0.333	Promoter(<=1kb)	-141	CYP2A7	1549
495995	19q	43846955	43848536	1582	6	0.833	3'UTR	13450	ZNF283	284349
495995	19q	43913423	43914878	1456	8	0.5	Exon(exon10of10)	4861	ZNF45	7596
495995	19q	44106512	44108078	1567	7	0	Exon(exon6of6)	-4103	ZNF225	7768
495995	19q	44327836	44329698	1863	6	0.5	Exon(exon4of4)	-22790	ZNF235	9310
495995	19q	52437918	52439242	1325	7	0.429	Exon(exon4of4)	6504	ZNF534	147658
495995	19q	55481625	55483456	1832	7	0.429	Promoter(1-2kb)	-1732	NAT14	57106
495995	19q	55911888	55913077	1190	6	0.667	Exon(exon5of12)	19234	NLRP13	126204
495995	19q	58368293	58368875	583	7	0.429	Exon(exon3of3)	-5445	ZNF497	162968
495995	20p	5922421	5923394	974	8	0.5	Exon(exon4of5)	6923	CHGB	1114
495995	20p	20052354	20052736	383	6	0.167	Promoter(<=1kb)	0	CFAP61	26074
495995	20q	63349752	63350772	1021	6	0.5	3'UTR	3794	CHRNA4	1137
495995	20q	63561666	63565531	3866	11	0.636	Promoter(<=1kb)	-61	HELZ2	85441
495995	21q	44591587	44592404	818	7	0.286	Promoter(<=1kb)	101	KRTAP10-6	386674
495995	21q	44600627	44601692	1066	10	0.7	Promoter(<=1kb)	30	KRTAP10-7	386675
495995	21q	44637474	44638041	568	9	0.333	Promoter(<=1kb)	118	KRTAP10-10	353333
495995	21q	45455650	45456803	1154	6	0.667	Promoter(<=1kb)	119	COL18A1	80781
495995	22q	22352950	22353380	431	16	0.5	Exon(exon1of2)	30478	BMS1P20	96610

495995	22q	36191154	36191906	753	6	0.667	3'UTR	9971	APOL4	80832
495995	22q	36265284	36265796	513	6	1	Exon(exon6of6)	12120	APOL1	8542
495995	22q	39100331	39102033	1703	6	0.833	Promoter(<=1kb)	52	APOBEC3H	164668
495995	23p	3320126	3323750	3625	9	0.556	Exon(exon5of7)	22902	MXRA5	25878
495995	23p	8170039	8170141	103	6	0.5	Promoter(1-2kb)	1126	VCX2	51480
495995	23p	35802148	35803010	863	7	0.571	5'UTR	3357	MAGEB16	139604
495995	23q	141906066	141906494	429	8	0.625	Promoter(1-2kb)	1264	MAGEC1	9947
496527	1p	978953	980637	1685	6	0.5	Promoter(<=1kb)	392	PERM1	84808
496527	1p	16058491	16060000	1510	10	0.9	Exon(exon5of7)	6168	CLCNKB	1188
496527	1p	18481403	18483159	1757	7	0.714	Promoter(<=1kb)	421	KLHDC7A	127707
496527	1p	23874604	23875430	827	8	0.5	Exon(exon2of2)	-6310	FUCA1	2517
496527	1p	40067594	40067675	82	6	0	Promoter(<=1kb)	324	CAP1	10487
496527	1p	89186388	89186419	32	9	0.556	Promoter(<=1kb)	107	GBP4	115361
496527	1q	152302977	152313891	10915	40	0.6	Promoter(<=1kb)	0	FLG-AS1	339400
496527	1q	156669844	156670886	1043	6	1	Exon(exon4of4)	6521	NES	10763
496527	1q	158765805	158766655	851	6	0.5	Promoter(<=1kb)	47	OR6N1	128372
496527	1q	197101312	197101771	460	6	0.5	Exon(exon18of28)	33373	ASPM	259266
496527	1q	201206099	201209342	3244	10	0.6	Promoter(1-2kb)	1017	IGFN1	91156
496527	1q	201210956	201215147	4192	9	0.556	Promoter(<=1kb)	0	IGFN1	91156
496527	1q	223393517	223394466	950	6	0.667	Promoter(<=1kb)	102	CCDC185	164127
496527	1q	232805117	232806800	1684	7	0.429	Promoter(<=1kb)	225	MAP10	54627
496527	1q	247841312	247841582	271	6	0.833	Promoter(<=1kb)	314	OR11L1	391189
496527	2p	48580657	48582454	1798	7	0.571	Promoter(<=1kb)	0	STON1	11037
496527	2q	130914528	130916712	2185	6	0.333	Promoter(<=1kb)	0	ARHGEF4	50649
496527	2q	132783534	132785001	1468	7	0.429	Promoter(1-2kb)	-1511	NCKAP5	344148
496527	2q	184936178	184937636	1459	6	0.333	Exon(exon4of4)	69813	ZNF804A	91752
496527	2q	217847583	217848559	977	6	0.833	Exon(exon19of33)	-5423	TNS1	7145
496527	2q	233713134	233713783	650	13	0.692	Promoter(<=1kb)	142	UGT1A5	54579
496527	3p	31989532	31990905	1374	7	0.429	Exon(exon2of2)	7761	ZNF860	344787
496527	3p	75736929	75739007	2079	14	0.643	Promoter(<=1kb)	0	MIR4273	100422955
496527	3q	98264413	98265098	686	8	0.5	Promoter(<=1kb)	128	OR5H6	79295
496527	4p	1394795	1395368	574	6	0.5	Exon(exon1of1)	9983	UVSSA	57654
496527	4p	5988383	5989749	1367	7	0.571	Promoter(<=1kb)	0	C4orf50	389197
496527	4p	6300792	6302360	1569	6	0.833	Exon(exon8of8)	6021	WFS1	7466
496527	4p	8227004	8228508	1505	8	0.25	Promoter(<=1kb)	-24	SH3TC1	54436
496527	4p	38774486	38775552	1067	9	0.444	Promoter(<=1kb)	644	TLR10	81793
496527	4p	38796894	38798702	1809	6	0.5	Exon(exon4of4)	5674	TLR1	7096
496527	4q	99653038	99654557	1520	6	0.833	Promoter(<=1kb)	91	C4orf54	285556
496527	5q	79731782	79734523	2742	14	0.357	Exon(exon2of13)	-3619	CMYA5	202333
496527	5q	140807352	140807737	386	6	0.833	Promoter(<=1kb)	271	PCDHA4	56144
496527	5q	140848579	140850786	2208	6	0.5	Promoter(<=1kb)	807	PCDHA9	9752
496527	5q	141174000	141175025	1026	6	0.833	Promoter(1-2kb)	1356	PCDHB7	56129
496527	5q	141187690	141189425	1736	6	0.5	Promoter(<=1kb)	529	PCDHB9	56127
496527	5q	141955356	141957660	2305	6	0.667	Promoter(<=1kb)	-668	RNF14	9604
496527	5q	151521550	151522069	520	6	0.667	Promoter(<=1kb)	79	MIR6499	102465246
496527	6p	46858978	46859502	525	7	0.429	Exon(exon17of21)	3802	ADGRF5	221395
496527	6q	64591274	64591961	688	10	0.5	Exon(exon26of43)	121374	EYS	346007
496527	6q	159231899	159234370	2472	12	0.583	Exon(exon11of23)	13602	FNDC1	84624
496527	7p	6330446	6330944	499	6	1	Exon(exon2of2)	7749	FAM220A	84792
496527	7q	100958721	100960873	2153	53	0.491	Promoter(<=1kb)	756	MUC3A	4584
496527	7q	100991195	100992398	1204	8	0.625	Exon(exon5of15)	-20656	MUC12	10071
496527	7q	100995547	100995896	350	8	0.875	Exon(exon5of15)	-17158	MUC12	10071
496527	7q	101004258	101004836	579	6	0.833	Exon(exon5of15)	-8218	MUC12	10071
496527	8p	8376561	8377198	638	6	1	Exon(exon2of5)	4549	PRAG1	157285
496527	8p	10607375	10608261	887	6	0.5	Exon(exon4of4)	46882	RP1L1	94137
496527	8p	13021128	13022030	903	8	0.25	Exon(exon5of5)	9115	TRMT9B	57604
496527	8q	138151949	138153046	1098	6	0.667	Promoter(<=1kb)	0	FAM135B	51059
496527	8q	142664564	142665970	1407	9	0.889	Exon(exon2of2)	4000	JRK	8629
496527	8q	143916360	143919209	2850	6	0	Exon(exon32of32)	20381	PLEC	5339
496527	9p	34723747	34725815	2069	6	0.833	Exon(exon4of4)	3673	FAM205A	259308
496527	9q	76705179	76707804	2626	8	0.5	Promoter(<=1kb)	121	PCA3	50652
496527	9q	76709263	76710843	1581	8	0.5	Promoter(<=1kb)	0	PRUNE2	158471
496527	9q	87887543	87888819	1277	6	0.5	Exon(exon4of4)	4666	SPATA31E1	286234
496527	9q	104598641	104599361	721	8	0.625	Promoter(<=1kb)	52	OR13C5	138799
496527	9q	122628595	122629398	804	6	0.333	Promoter(<=1kb)	175	OR1B1	347169
496527	9q	122749914	122750547	634	6	0.833	Promoter(<=1kb)	174	OR1L6	392390
496527	9q	135484803	135487213	2411	9	0.333	Promoter(1-2kb)	1440	PPP1R26	9858
496527	10q	46549378	46550723	1346	25	0.64	Exon(exon3of3)	4807	GPRIN2	9721
496527	10q	128102594	128109280	6687	21	0.714	Promoter(<=1kb)	0	MKI67	4288
496527	11p	244106	244197	92	8	0.5	Promoter(<=1kb)	-232	PSMD13	5719
496527	11p	5046754	5047432	679	7	0.571	Promoter(<=1kb)	228	OR52J3	119679
496527	11p	5177978	5178478	501	6	0.167	Promoter(<=1kb)	186	OR52Z1	283110
496527	11p	5323542	5324256	715	6	0.5	Promoter(<=1kb)	41	OR51B2	79345
496527	11p	5389704	5390350	647	6	0.5	Promoter(<=1kb)	327	OR51M1	390059
496527	11p	5581045	5581738	694	8	0.375	Promoter(<=1kb)	168	OR52B6	340980
496527	11p	5884818	5885061	244	6	0.333	Promoter(<=1kb)	547	OR52E4	390081
496527	11p	11351961	11352736	776	9	0.222	Promoter(<=1kb)	514	CSNK2A3	283106

496527	11p	12293639	12294538	900	8	0.625	Exon(exon29of35)	6739	MICALCL	84953
496527	11p	18173280	18173901	622	6	0.333	Promoter(<=1kb)	443	MRGPRX4	117196
496527	11q	58214757	58215722	966	8	0.25	Promoter(<=1kb)	12	OR1S1	219959
496527	11q	64315797	64315856	60	8	0.75	Promoter(1-2kb)	1485	TRMT112	51504
496527	11q	82732630	82733184	555	6	0.833	Promoter(<=1kb)	680	FAM181B	220382
496527	11q	85724687	85725825	1139	6	0.5	Promoter(<=1kb)	0	SYTL2	54843
496527	11q	124015601	124016477	877	9	0.333	Promoter(<=1kb)	26	OR10G4	390264
496527	11q	124023038	124023849	812	9	0.444	Promoter(<=1kb)	25	OR10G9	219870
496527	12p	4626571	4628549	1979	10	0.5	Exon(exon5of6)	14054	DYRK4	8798
496527	13q	24434450	24435347	898	7	0.571	Exon(exon31of34)	19787	PARP4	143
496527	13q	25096659	25097182	524	22	0.5	Promoter(<=1kb)	791	PABPC3	5042
496527	13q	102732474	102733933	1460	6	0.333	Exon(exon4of4)	25139	CCDC168	643677
496527	14q	19827604	19827851	248	6	0.833	Exon(exon2of2)	5669	OR4N2	390429
496527	14q	20060048	20060884	837	8	0.625	Promoter(<=1kb)	3	OR4L1	122742
496527	14q	70457532	70458540	1009	9	0.333	Exon(exon2of2)	5358	ADAM21	8747
496527	14q	94587512	94587839	328	6	0.5	Exon(exon2of2)	-4219	SERPINA3	12
496527	14q	104175275	104177810	2536	7	0.143	Exon(exon12of15)	36235	KIF26A	26153
496527	14q	104939262	104954713	15452	80	0.475	Promoter(<=1kb)	689	AHNAK2	113146
496527	15q	20534501	20535014	514	6	0.5	Exon(exon8of9)	6786	GOLGA6L6	727832
496527	15q	23439979	23442067	2089	12	0.5	5'UTR	5167	GOLGA6L2	283685
496527	15q	85579423	85581800	2378	16	0.625	Promoter(1-2kb)	-1110	AKAP13	11214
496527	15q	88854874	88855594	721	6	0.833	Exon(exon12of18)	7631	ACAN	176
496527	15q	88857108	88859365	2258	6	0	Exon(exon12of18)	9865	ACAN	176
496527	15q	99129423	99132517	3095	6	0.333	Exon(exon4of5)	7225	TTC23	64927
496527	16p	1221890	1223490	1601	7	1	Promoter(1-2kb)	1285	TPSG1	25823
496527	16p	1228744	1229622	879	7	0.714	Promoter(<=1kb)	540	TPSB2	64499
496527	16p	1256345	1256816	472	6	0.833	Promoter(<=1kb)	276	TPSD1	23430
496527	16p	1486322	1488463	2142	12	0.75	Promoter(<=1kb)	4	PTX4	390667
496527	16q	74391460	74392004	545	12	0.75	Exon(exon7of7)	13582	NPIPB15	440348
496527	16q	88428416	88429600	1185	6	0.333	Exon(exon3of3)	-23680	ZFPM1	161882
496527	16q	89100686	89101050	365	8	0.625	Promoter(<=1kb)	24	ACSF3	197322
496527	16q	89226863	89228419	1557	9	0.556	Promoter(2-3kb)	2229	ZNF778	197320
496527	17p	744946	747270	2325	6	0.833	3'UTR	4768	GEMIN4	50628
496527	17p	10638198	10641099	2902	7	0.286	Exon(exon19of41)	-8169	MYH3	4621
496527	17p	21300581	21300954	374	9	0.778	3'UTR	9112	MAP2K3	5606
496527	17q	81510690	81511591	902	6	0.667	Promoter(<=1kb)	257	ACTG1	71
496527	17q	81645135	81645417	283	6	0.333	Promoter(2-3kb)	2722	TSPAN10	83882
496527	18p	11609646	11610350	705	11	0.818	Promoter(<=1kb)	50	SLC35G4	646000
496527	18q	58535186	58538030	2845	19	0.526	Promoter(<=1kb)	0	ALPK2	115701
496527	19p	1036445	1036914	470	7	0.571	Exon(exon6of7)	-3187	ABCA7	10347
496527	19p	4510548	4513547	3000	22	0.455	Exon(exon3of6)	4157	PLIN4	729359
496527	19p	5455600	5456439	840	7	0.571	Promoter(<=1kb)	183	ZNRF4	148066
496527	19p	5866643	5867737	1095	7	0.714	Promoter(2-3kb)	2803	FUT5	2527
496527	19p	8936200	8939234	3035	11	0.909	Exon(exon5of84)	-40110	MUC16	94025
496527	19p	8946254	8951868	5615	22	0.591	Exon(exon3of84)	29474	MUC16	94025
496527	19p	8959116	8962299	3184	12	0.583	Exon(exon3of84)	19043	MUC16	94025
496527	19p	8963397	8967127	3731	21	0.667	Exon(exon3of84)	14215	MUC16	94025
496527	19p	8972751	8978096	5346	13	0.462	Exon(exon1of84)	3246	MUC16	94025
496527	19p	12430157	12432437	2281	9	0.333	3'UTR	8584	ZNF443	10224
496527	19p	15087213	15088040	828	10	0.3	Promoter(<=1kb)	233	OR1I1	126370
496527	19p	18264798	18267409	2612	12	0.583	5'UTR	7002	IQCN	80726
496527	19p	21971930	21974500	2571	7	0.714	Exon(exon4of4)	14408	ZNF208	7757
496527	19q	34943334	34944685	1352	6	0.333	3'UTR	9723	ZNF30	90075
496527	19q	36996730	36997597	868	10	0.7	Exon(exon10of10)	5677	ZNF568	374900
496527	19q	37151928	37153149	1222	6	0.833	Exon(exon5of5)	19287	ZNF585A	199704
496527	19q	37885190	37888806	3617	8	0.625	Exon(exon6of6)	17788	WDR87	83889
496527	19q	39886240	39886422	183	6	0.667	Promoter(<=1kb)	870	FCGBP	8857
496527	19q	43846955	43848536	1582	6	0.833	3'UTR	13450	ZNF283	284349
496527	19q	43913423	43914878	1456	8	0.5	Exon(exon10of10)	4861	ZNF45	7596
496527	19q	43996326	43997415	1090	6	0.5	Exon(exon5of5)	5370	LOC10192806:	101928063
496527	19q	44106512	44108078	1567	7	0	Exon(exon6of6)	-4103	ZNF225	7768
496527	19q	44327836	44329698	1863	6	0.5	Exon(exon4of4)	-22790	ZNF235	9310
496527	19q	48873325	48875925	2601	11	0.545	Promoter(<=1kb)	904	PPP1R15A	23645
496527	19q	50855077	50856247	1171	6	0.667	Promoter(<=1kb)	105	KLK3	354
496527	19q	55911888	55913077	1190	6	0.667	Exon(exon5of12)	19234	NLRP13	126204
496527	19q	58368293	58368875	583	7	0.429	Exon(exon3of3)	-5445	ZNF497	162968
496527	20q	63349752	63350949	1198	6	0.5	3'UTR	3617	CHRNA4	1137
496527	20q	63561666	63565531	3866	12	0.667	Promoter(<=1kb)	-61	HELZ2	85441
496527	21q	26843740	26844859	1120	6	0.667	Promoter(<=1kb)	0	ADAMTS1	9510
496527	21q	41740862	41741990	1129	6	0.833	Exon(exon2of2)	4851	MIR6814	102465488
496527	21q	44591587	44592404	818	7	0.286	Promoter(<=1kb)	101	KRTAP10-6	386674
496527	21q	44600627	44601692	1066	10	0.7	Promoter(<=1kb)	30	KRTAP10-7	386675
496527	22q	22352950	22353380	431	16	0.5	Exon(exon1of2)	30478	BMS1P20	96610
496527	22q	36191154	36191906	753	6	0.667	3'UTR	9971	APOL4	80832
496527	22q	39100331	39102033	1703	6	0.833	Promoter(<=1kb)	52	APOBEC3H	164668
496527	23p	8170039	8170243	205	7	0.571	Promoter(1-2kb)	1024	VCX2	51480
496527	23p	35802148	35803010	863	7	0.571	5'UTR	3357	MAGEB16	139604

496527	23q	102937373	102937765	393	7	0.571	Promoter(<=1kb)	101	RAB40AL	282808
496527	23q	136874183	136874347	165	7	0.714	Promoter(<=1kb)	-201	RBMX	27316
487160	1p	12795695	12795963	269	6	0.667	Promoter(1-2kb)	1004	PRAMEF1	65121
487160	1p	12847526	12847780	255	7	0.429	Promoter(<=1kb)	945	HNRNPCL1	343069
487160	1p	12859036	12860079	1044	6	0.333	Promoter(1-2kb)	1950	PRAMEF2	65122
487160	1p	13370686	13371119	434	7	0.429	Promoter(<=1kb)	781	PRAMEF19	645414
487160	1p	16058491	16060000	1510	13	0.846	Exon(exon5of7)	6168	CLCNKB	1188
487160	1p	18481403	18483159	1757	7	0.714	Promoter(<=1kb)	421	KLHDC7A	127707
487160	1p	23874604	23875430	827	9	0.556	Exon(exon2of2)	-6310	FUCA1	2517
487160	1p	40067594	40067675	82	6	0	Promoter(<=1kb)	324	CAP1	10487
487160	1p	89186388	89186419	32	9	0.556	Promoter(<=1kb)	107	GBP4	115361
487160	1q	152303673	152313891	10219	41	0.561	Promoter(<=1kb)	0	FLG-AS1	339400
487160	1q	156669844	156670886	1043	6	1	Exon(exon4of4)	6521	NES	10763
487160	1q	156906687	156909788	3102	6	0.5	Promoter(<=1kb)	-169	PEAR1	375033
487160	1q	158765805	158766655	851	6	0.5	Promoter(<=1kb)	47	OR6N1	128372
487160	1q	201206099	201209342	3244	9	0.667	Promoter(1-2kb)	1017	IGFN1	91156
487160	1q	214640144	214642954	2811	12	0.5	Exon(exon12of20)	-5013	CENPF	1063
487160	1q	214644872	214647181	2310	10	0.4	Promoter(<=1kb)	-786	CENPF	1063
487160	1q	228315976	228318049	2074	7	0.571	Exon(exon50of81)	6492	OBSCN	84033
487160	1q	247841312	247841582	271	6	0.833	Promoter(<=1kb)	314	OR11L1	391189
487160	1q	247921132	247921847	716	8	0.625	Promoter(<=1kb)	114	OR2T8	343172
487160	1q	247949325	247949738	414	10	0.3	Promoter(<=1kb)	467	OR2L8	391190
487160	1q	248294677	248295458	782	7	0.571	Promoter(<=1kb)	142	OR2T12	127064
487160	2p	48580657	48582454	1798	7	0.571	Promoter(<=1kb)	0	STON1	11037
487160	2q	132783534	132785001	1468	7	0.429	Promoter(1-2kb)	-1511	NCKAP5	344148
487160	2q	184936178	184937636	1459	8	0.375	Exon(exon4of4)	69813	ZNF804A	91752
487160	2q	185789865	185794632	4768	11	0.727	Promoter(<=1kb)	0	FSIP2	401024
487160	2q	217847583	217848559	977	6	0.833	Exon(exon19of33)	-5423	TNS1	7145
487160	2q	219271337	219271649	313	6	0.667	Exon(exon4of4)	6227	TUBA4A	7277
487160	2q	240041845	240042811	967	7	0.286	Downstream(2-3kb)	3261	OR6B3	150681
487160	3p	31989532	31990905	1374	6	0.333	Exon(exon2of2)	7761	ZNF860	344787
487160	3p	75737230	75739007	1778	9	0.556	Promoter(<=1kb)	0	MIR4273	100422955
487160	3q	98264413	98265137	725	7	0.714	Promoter(<=1kb)	128	OR5H6	79295
487160	3q	194341097	194342571	1475	7	0.714	Exon(exon2of2)	8747	CPN2	1370
487160	3q	196947388	196948102	715	6	0.5	3'UTR	3828	PIGZ	80235
487160	4p	5988383	5989749	1367	7	0.571	Promoter(<=1kb)	0	C4orf50	389197
487160	4p	6300792	6302360	1569	6	0.833	Exon(exon8of8)	6021	WFS1	7466
487160	4p	7433331	7434512	1182	6	0.833	Promoter(<=1kb)	418	PSAPL1	768239
487160	4p	8227004	8228508	1505	8	0.125	Promoter(<=1kb)	-24	SH3TC1	54436
487160	4q	121036404	121037542	1139	6	0.333	Promoter(1-2kb)	1442	NDNF	79625
487160	4q	154489498	154491312	1815	9	0.556	Promoter(<=1kb)	22	DCHS2	54798
487160	4q	185458217	185460011	1795	8	0.625	Promoter(<=1kb)	0	CCDC110	256309
487160	4q	186617176	186621601	4426	11	0.364	Exon(exon10of27)	-7106	FAT1	2195
487160	5q	79731782	79734523	2742	13	0.308	Exon(exon2of13)	-3619	CMYA5	202333
487160	5q	83537326	83540746	3421	7	0.286	Promoter(1-2kb)	-1202	VCAN	1462
487160	5q	140807352	140807737	386	6	0.833	Promoter(<=1kb)	271	PCDHA4	56144
487160	5q	141174000	141175025	1026	6	0.833	Promoter(1-2kb)	1356	PCDHB7	56129
487160	5q	141955676	141957660	1985	6	0.667	Promoter(<=1kb)	-668	RNF14	9604
487160	5q	148826877	148828070	1194	6	1	Promoter(1-2kb)	1632	ADRB2	154
487160	5q	151565922	151568158	2237	10	0.8	Promoter(<=1kb)	786	FAT2	2196
487160	6p	46858771	46859502	732	8	0.5	Exon(exon17of21)	3802	ADGRF5	221395
487160	6q	159231899	159234370	2472	10	0.5	Exon(exon11of23)	13602	FNDC1	84624
487160	7p	12369637	12370736	1100	6	0.667	3'UTR	-13307	VWDE	221806
487160	7q	100958977	100960873	1897	53	0.453	Promoter(1-2kb)	1012	MUC3A	4584
487160	7q	100991195	100994057	2863	16	0.688	Exon(exon5of15)	-18997	MUC12	10071
487160	7q	100995424	100995785	362	7	0.857	Exon(exon5of15)	-17269	MUC12	10071
487160	8p	10607245	10608432	1188	8	0.5	Exon(exon4of4)	46711	RP1L1	94137
487160	8p	10609614	10612307	2694	7	1	Exon(exon4of4)	42836	RP1L1	94137
487160	8p	11331234	11332082	849	7	0.714	Promoter(<=1kb)	346	SLC35G5	83650
487160	8p	12132686	12133940	1255	6	0.667	Promoter(<=1kb)	498	USP17L7	392197
487160	8p	12137553	12138641	1089	6	1	Promoter(<=1kb)	436	USP17L2	377630
487160	8p	13021128	13022030	903	8	0.25	Exon(exon5of5)	9115	TRMT9B	57604
487160	8q	123651655	123652634	980	7	0.571	Promoter(<=1kb)	316	KLHL38	340359
487160	9q	76703555	76707804	4250	14	0.429	Promoter(<=1kb)	0	PCA3	50652
487160	9q	76709263	76710846	1584	9	0.556	Promoter(<=1kb)	0	PRUNE2	158471
487160	9q	104504315	104505071	757	6	0.5	Promoter(<=1kb)	52	OR13F1	138805
487160	9q	104598641	104599361	721	8	0.625	Promoter(<=1kb)	52	OR13C5	138799
487160	9q	122553263	122554071	809	7	0.429	Promoter(<=1kb)	93	OR1N2	138882
487160	9q	122628595	122629398	804	6	0.167	Promoter(<=1kb)	175	OR1B1	347169
487160	9q	122749914	122750547	634	6	0.833	Promoter(<=1kb)	174	OR1L6	392390
487160	9q	133255635	133256264	630	8	1	3'UTR	18950	ABO	28
487160	9q	135547960	135548795	836	8	0.625	Promoter(1-2kb)	1805	OBP2A	29991
487160	10q	46549378	46550723	1346	26	0.654	Exon(exon3of3)	4807	GPRIN2	9721
487160	10q	49323559	49326554	2996	11	0.545	Exon(exon3of3)	24285	C10orf71	118461
487160	10q	122084988	122087840	2853	8	0.75	Exon(exon4of23)	-25190	TACC2	10579
487160	10q	128103129	128104752	1624	8	0.5	Promoter(<=1kb)	-1	MKI67	4288
487160	10q	128106210	128108204	1995	6	1	Exon(exon12of14)	-3082	MKI67	4288

487160	11p	244106	244197	92	8	0.5	Promoter(<=1kb)	-232	PSMD13	5719
487160	11p	1241677	1243593	1917	6	0.333	Exon(exon31of49)	6083	MUC5B-AS1	112577518
487160	11p	1244757	1248605	3849	15	0.667	Promoter(1-2kb)	1071	MUC5B-AS1	112577518
487160	11p	1250091	1251628	1538	9	0.778	Promoter(<=1kb)	-415	MUC5B-AS1	112577518
487160	11p	5177811	5178478	668	7	0.143	Promoter(<=1kb)	186	OR52Z1	283110
487160	11p	5323451	5324256	806	6	0.5	Promoter(<=1kb)	41	OR51B2	79345
487160	11p	5389704	5390350	647	6	0.5	Promoter(<=1kb)	327	OR51M1	390059
487160	11p	5515185	5516015	831	6	0.333	Promoter(<=1kb)	684	UBQLNL	143630
487160	11p	5581045	5581738	694	8	0.375	Promoter(<=1kb)	168	OR52B6	340980
487160	11p	5841302	5841883	582	9	0.333	Promoter(<=1kb)	14	OR52E6	390078
487160	11p	5884818	5885061	244	7	0.429	Promoter(<=1kb)	547	OR52E4	390081
487160	11p	11351961	11352736	776	9	0.222	Promoter(<=1kb)	514	CSNK2A3	283106
487160	11p	12293639	12294368	730	6	0.833	Exon(exon29of35)	6739	MICALCL	84953
487160	11q	58214757	58215722	966	8	0.25	Promoter(<=1kb)	12	OR1S1	219959
487160	11q	64116513	64118232	1720	7	0.714	Exon(exon2of2)	8702	MACROD1	28992
487160	11q	82732630	82733184	555	6	0.833	Promoter(<=1kb)	680	FAM181B	220382
487160	11q	123906510	123907324	815	7	0.714	Promoter(<=1kb)	364	OR8D4	338662
487160	11q	124038366	124038988	623	7	1	Promoter(<=1kb)	13	OR10G7	390265
487160	12p	4626571	4628549	1979	8	0.5	Exon(exon5of6)	14054	DYRK4	8798
487160	12q	52316096	52317765	1670	6	0.667	Exon(exon4of9)	3633	KRT83	3889
487160	13q	102732474	102733933	1460	6	0.333	Exon(exon4of4)	25139	CCDC168	643677
487160	14q	20060048	20060884	837	8	0.625	Promoter(<=1kb)	3	OR4L1	122742
487160	14q	21634137	21634589	453	9	0.556	Promoter(<=1kb)	351	OR10G2	26534
487160	14q	44504986	44506403	1418	6	0.667	Promoter(<=1kb)	880	FSCB	84075
487160	14q	70457532	70458540	1009	11	0.273	Exon(exon2of2)	5358	ADAM21	8747
487160	14q	104175275	104177810	2536	10	0.3	Exon(exon12of15)	36235	KIF26A	26153
487160	14q	104947943	104950338	2396	8	0.5	Exon(exon6of6)	5064	AHNAK2	113146
487160	15q	23440160	23442067	1908	11	0.636	5'UTR	5167	GOLGA6L2	283685
487160	15q	78766071	78766626	556	6	0.5	Promoter(<=1kb)	-958	ADAMTS7	11173
487160	15q	85579207	85581800	2594	14	0.643	Promoter(1-2kb)	-1110	AKAP13	11214
487160	15q	88857878	88859365	1488	6	0.167	Exon(exon12of18)	10635	ACAN	176
487160	15q	100569472	100570097	626	6	0.833	Promoter(<=1kb)	534	LINS1	55180
487160	16p	789084	790597	1514	6	0.167	Promoter(<=1kb)	0	CHTF18	63922
487160	16p	1256345	1256980	636	6	0.833	Promoter(<=1kb)	276	TPSD1	23430
487160	16p	1486322	1488463	2142	13	0.692	Promoter(<=1kb)	4	PTX4	390667
487160	16q	88714632	88717113	2482	7	0.429	Promoter(<=1kb)	0	MIR4722	100616167
487160	16q	89226863	89228289	1427	8	0.625	Promoter(2-3kb)	2229	ZNF778	197320
487160	17p	744946	746966	2021	6	1	3'UTR	5072	GEMIN4	50628
487160	17p	10638198	10641099	2902	6	0.333	Exon(exon19of41)	-8169	MYH3	4621
487160	17p	21300581	21300978	398	12	0.75	3'UTR	9112	MAP2K3	5606
487160	17p	21415470	21416370	901	6	0.833	Exon(exon3of3)	10334	KCNJ12	3768
487160	17q	53823368	53824891	1524	6	0.833	Promoter(<=1kb)	441	KIF2B	84643
487160	17q	76291123	76294016	2894	11	0.455	Promoter(<=1kb)	0	QRICH2	84074
487160	17q	81645135	81645595	461	7	0.429	Promoter(2-3kb)	2722	TSPAN10	83882
487160	18p	11609904	11610491	588	6	0.667	Promoter(<=1kb)	308	SLC35G4	646000
487160	18p	14542649	14543140	492	6	0.5	Promoter(<=1kb)	6	POTEC	388468
487160	18q	58535186	58537515	2330	10	0.4	Promoter(<=1kb)	0	ALPK2	115701
487160	19p	1036457	1036914	458	6	0.5	Exon(exon6of7)	-3187	ABCA7	10347
487160	19p	4510548	4513547	3000	20	0.45	Exon(exon3of6)	4157	PLIN4	729359
487160	19p	5455600	5456439	840	6	0.5	Promoter(<=1kb)	183	ZNRF4	148066
487160	19p	8937644	8939234	1591	6	0.667	Exon(exon5of84)	-41554	MUC16	94025
487160	19p	8946313	8952171	5859	20	0.6	Exon(exon3of84)	29171	MUC16	94025
487160	19p	8959116	8962299	3184	12	0.75	Exon(exon3of84)	19043	MUC16	94025
487160	19p	8971838	8978096	6259	15	0.533	Exon(exon1of84)	3246	MUC16	94025
487160	19p	12430718	12432437	1720	8	0.375	3'UTR	8584	ZNF443	10224
487160	19p	17281820	17284246	2427	9	0.556	Promoter(<=1kb)	0	ANKLE1	126549
487160	19p	18264798	18267409	2612	12	0.583	5'UTR	7002	IQCN	80726
487160	19p	21971930	21974500	2571	7	0.714	Exon(exon4of4)	14408	ZNF208	7757
487160	19p	22756205	22759523	3319	9	0.667	3'UTR	10459	ZNF99	7652
487160	19q	39877222	39877880	659	6	0.5	Exon(exon20of28)	9412	FCGBP	8857
487160	19q	39886240	39886422	183	6	0.667	Promoter(<=1kb)	870	FCGBP	8857
487160	19q	43203948	43205504	1557	7	0.714	Promoter(<=1kb)	0	PSG4	5672
487160	19q	43913423	43914878	1456	8	0.5	Exon(exon10of10)	4861	ZNF45	7596
487160	19q	44106512	44108078	1567	7	0	Exon(exon6of6)	-4103	ZNF225	7768
487160	19q	52437918	52439242	1325	7	0.429	Exon(exon4of4)	6504	ZNF534	147658
487160	19q	55481625	55484116	2492	7	0.571	Promoter(1-2kb)	-1072	NAT14	57106
487160	19q	55911888	55913077	1190	6	0.667	Exon(exon5of12)	19234	NLRP13	126204
487160	19q	57639918	57641641	1724	7	0.429	3'UTR	6693	ZNF211	10520
487160	19q	58368260	58368875	616	8	0.5	Exon(exon3of3)	-5412	ZNF497	162968
487160	20q	63349752	63350772	1021	6	0.5	3'UTR	3794	CHRNA4	1137
487160	20q	63561666	63565531	3866	12	0.667	Promoter(<=1kb)	-61	HELZ2	85441
487160	21q	44573869	44574611	743	6	0.667	Promoter(<=1kb)	-53	TSPEAR	54084
487160	21q	44600627	44601692	1066	9	0.778	Promoter(<=1kb)	30	KRTAP10-7	386675
487160	22q	22352950	22353380	431	16	0.5	Exon(exon1of2)	30478	BMS1P20	96610
487160	22q	36191154	36191906	753	6	0.667	3'UTR	9971	APOL4	80832
487160	22q	49884187	49885288	1102	6	0.167	Exon(exon2of2)	22630	ALG12	79087
487160	23p	3320126	3323750	3625	10	0.6	Exon(exon5of7)	22902	MXRA5	25878

487160	23p	8170039	8170141	103	6	0.5	Promoter(1-2kb)	1126	VCX2	51480
487160	23p	35802148	35803010	863	7	0.571	5'UTR	3357	MAGEB16	139604
487160	23q	115189945	115192586	2642	6	0.833	Promoter(<=1kb)	518	RBMXL3	139804
497356	1p	12847526	12847995	470	12	0.333	Promoter(<=1kb)	730	HNRNPCL1	343069
497356	1p	12859036	12860128	1093	9	0.444	Promoter(1-2kb)	1950	PRAMEF2	65122
497356	1p	12893213	12893472	260	8	0.375	Exon(exon4of4)	4798	PRAMEF10	343071
497356	1p	13369166	13369564	399	6	0.667	Promoter(2-3kb)	2336	PRAMEF19	645414
497356	1p	13370686	13371119	434	7	0.429	Promoter(<=1kb)	781	PRAMEF19	645414
497356	1p	16058491	16060000	1510	13	0.846	Exon(exon5of7)	6168	CLCNKB	1188
497356	1p	18481403	18482217	815	7	0.714	Promoter(<=1kb)	421	KLHDC7A	127707
497356	1p	40067594	40067675	82	6	0	Promoter(<=1kb)	324	CAP1	10487
497356	1q	152213274	152213320	47	8	0.5	Exon(exon3of3)	10873	HRNR	388697
497356	1q	152218469	152221375	2907	16	0.688	Promoter(2-3kb)	2818	HRNR	388697
497356	1q	152303673	152313891	10219	38	0.605	Promoter(<=1kb)	0	FLG-AS1	339400
497356	1q	156669844	156670886	1043	6	1	Exon(exon4of4)	6521	NES	10763
497356	1q	158398525	158399274	750	6	0.333	Promoter(<=1kb)	192	OR10T2	128360
497356	1q	158765805	158766655	851	6	0.5	Promoter(<=1kb)	47	OR6N1	128372
497356	1q	197101312	197101771	460	6	0.5	Exon(exon18of28)	33373	ASPM	259266
497356	1q	201210718	201211044	327	6	0.333	Promoter(1-2kb)	-1598	IGFN1	91156
497356	1q	228315976	228318026	2051	6	0.5	Exon(exon50of81)	6492	OBSCN	84033
497356	1q	247841312	247841582	271	6	0.833	Promoter(<=1kb)	314	OR11L1	391189
497356	1q	247949325	247949738	414	10	0.3	Promoter(<=1kb)	467	OR2L8	391190
497356	1q	248681658	248682198	541	7	0.571	Promoter(<=1kb)	130	OR14I1	401994
497356	2p	29002636	29003646	1011	6	0.333	Exon(exon5of20)	-10821	TOGARAM2	165186
497356	2p	48580657	48582454	1798	8	0.625	Promoter(<=1kb)	0	STON1	11037
497356	2q	132783534	132785001	1468	7	0.429	Promoter(1-2kb)	-1511	NCKAP5	344148
497356	2q	167246794	167248478	1685	7	0.714	Promoter(<=1kb)	-204	XIRP2	129446
497356	2q	167249575	167251847	2273	6	0.667	Promoter(<=1kb)	893	XIRP2	129446
497356	2q	185789865	185794632	4768	10	0.8	Promoter(<=1kb)	0	FSIP2	401024
497356	2q	185799746	185802394	2649	6	0.5	Promoter(<=1kb)	0	FSIP2-AS1	107985781
497356	2q	185805377	185808170	2794	6	0.333	Promoter(<=1kb)	0	FSIP2	401024
497356	2q	238130162	238131546	1385	6	0.5	Promoter(1-2kb)	1214	ESPNL	339768
497356	2q	240041845	240042811	967	7	0.286	Downstream(2-3kb)	3261	OR6B3	150681
497356	3p	75664404	75665948	1545	6	0.5	Promoter(<=1kb)	74	FRG2C	100288801
497356	3p	75736880	75738934	2055	65	0.585	Promoter(<=1kb)	0	MIR4273	100422955
497356	3q	98264413	98265098	686	7	0.571	Promoter(<=1kb)	128	OR5H6	79295
497356	3q	194359607	194360906	1300	9	0.889	Exon(exon2of2)	-8279	CPN2	1370
497356	3q	196947388	196948102	715	6	0.5	3'UTR	3828	PIGZ	80235
497356	4p	5988383	5989749	1367	7	0.571	Promoter(<=1kb)	0	C4orf50	389197
497356	4p	6300792	6302360	1569	6	0.833	Exon(exon8of8)	6021	WFS1	7466
497356	4p	8227004	8228508	1505	8	0.125	Promoter(<=1kb)	-24	SH3TC1	54436
497356	4p	38773881	38775552	1672	6	0.333	Promoter(<=1kb)	644	TLR10	81793
497356	4q	121036404	121037542	1139	6	0.333	Promoter(1-2kb)	1442	NDNF	79625
497356	4q	186706638	186708616	1979	6	0.5	Exon(exon2of27)	15217	FAT1	2195
497356	5p	795818	796237	420	9	0.556	3'UTR	4908	ZDHHC11	79844
497356	5q	79731782	79734523	2742	13	0.308	Exon(exon2of13)	-3619	CMYA5	202333
497356	5q	140807352	140807737	386	6	0.833	Promoter(<=1kb)	271	PCDHA4	56144
497356	5q	141100771	141102758	1988	11	0.273	Promoter(<=1kb)	298	PCDHB3	56132
497356	5q	141174000	141175025	1026	7	0.714	Promoter(1-2kb)	1356	PCDHB7	56129
497356	5q	141178631	141180333	1703	6	0.833	Promoter(<=1kb)	841	PCDHB8	56128
497356	5q	141183999	141184688	690	6	0.833	Promoter(2-3kb)	-2473	PCDHB9	56127
497356	5q	141187109	141189425	2317	6	0.5	Promoter(<=1kb)	0	PCDHB9	56127
497356	5q	141192663	141194472	1810	6	1	Promoter(<=1kb)	310	PCDHB10	56126
497356	5q	141955356	141957660	2305	6	0.667	Promoter(<=1kb)	-668	RNF14	9604
497356	5q	151565922	151568158	2237	9	0.778	Promoter(<=1kb)	786	FAT2	2196
497356	6p	42745312	42746041	730	6	0.667	Promoter(<=1kb)	62	TBCC	6903
497356	6p	46858771	46859389	619	6	0.5	Exon(exon17of21)	3915	ADGRF5	221395
497356	6q	149888581	149890867	2287	7	0.714	Promoter(<=1kb)	0	RAET1E-AS1	100652739
497356	6q	159231899	159234370	2472	11	0.545	Exon(exon11of23)	13602	FNDC1	84624
497356	7p	12369637	12370736	1100	6	0.667	3'UTR	-13307	VWDE	221806
497356	7p	45082725	45084999	2275	8	0.5	Promoter(1-2kb)	-1325	NACAD	23148
497356	7q	100958721	100960873	2153	55	0.473	Promoter(<=1kb)	756	MUC3A	4584
497356	7q	100995547	100995785	239	7	0.857	Exon(exon5of15)	-17269	MUC12	10071
497356	7q	129126683	129127452	770	7	0.286	Exon(exon1of2)	-17255	TSPAN33	340348
497356	7q	138917080	138919164	2085	6	0.667	Exon(exon2of20)	62154	KIAA1549	57670
497356	8p	10607245	10608432	1188	8	0.5	Exon(exon4of4)	46711	RP1L1	94137
497356	8p	10609614	10610662	1049	8	0.5	Exon(exon4of4)	44481	RP1L1	94137
497356	8p	12132477	12133936	1460	10	0.7	Promoter(<=1kb)	502	USP17L7	392197
497356	8p	13021128	13022030	903	8	0.25	Exon(exon5of5)	9115	TRMT9B	57604
497356	8q	141218050	141219792	1743	6	0.667	5'UTR	8778	SLC45A4	57210
497356	9p	39078723	39078846	124	6	0.667	Exon(exon22of24)	7302	CNTNAP3	79937
497356	9q	104598545	104599361	817	12	0.583	Promoter(<=1kb)	52	OR13C5	138799
497356	9q	122553263	122554071	809	7	0.429	Promoter(<=1kb)	93	OR1N2	138882
497356	9q	122628595	122629398	804	6	0.333	Promoter(<=1kb)	175	OR1B1	347169
497356	9q	122749914	122750547	634	6	0.833	Promoter(<=1kb)	174	OR1L6	392390
497356	9q	135484803	135487573	2771	9	0.333	Promoter(1-2kb)	1440	PPP1R26	9858
497356	9q	135547960	135548795	836	8	0.625	Promoter(1-2kb)	1805	OBP2A	29991

497356	10q	46549378	46550723	1346	25	0.64	Exon(exon3of3)	4807	GPRIN2	9721
497356	11p	244106	244197	92	8	0.5	Promoter(<=1kb)	-232	PSMD13	5719
497356	11p	1194354	1196902	2549	7	0.571	Exon(exon34of49)	-26164	MUC5B	727897
497356	11p	1246095	1247378	1284	9	0.444	Promoter(2-3kb)	2298	MUC5B-AS1	112577518
497356	11p	1250091	1251628	1538	6	0.833	Promoter(<=1kb)	-415	MUC5B-AS1	112577518
497356	11p	5177811	5178478	668	7	0.143	Promoter(<=1kb)	186	OR52Z1	283110
497356	11p	5323362	5324256	895	7	0.429	Promoter(<=1kb)	41	OR51B2	79345
497356	11p	5389704	5390350	647	7	0.429	Promoter(<=1kb)	327	OR51M1	390059
497356	11p	5422212	5423123	912	10	0.7	Promoter(<=1kb)	101	OR51Q1	390061
497356	11p	5581045	5581738	694	8	0.375	Promoter(<=1kb)	168	OR52B6	340980
497356	11p	5884818	5885061	244	6	0.333	Promoter(<=1kb)	547	OR52E4	390081
497356	11p	5986042	5986669	628	7	1	Promoter(<=1kb)	316	OR52L1	338751
497356	11p	11351961	11352736	776	8	0.25	Promoter(<=1kb)	514	CSNK2A3	283106
497356	11p	12293639	12294538	900	8	0.625	Exon(exon29of35)	6739	MICALCL	84953
497356	11p	18173280	18173901	622	7	0.286	Promoter(<=1kb)	443	MRGPRX4	117196
497356	11q	55827393	55827640	248	7	0.714	Promoter(<=1kb)	174	OR5L2	26338
497356	11q	58214757	58215722	966	8	0.25	Promoter(<=1kb)	12	OR1S1	219959
497356	11q	64315749	64315856	108	9	0.778	Promoter(1-2kb)	1465	ESRRA	2101
497356	11q	82732630	82733184	555	6	0.833	Promoter(<=1kb)	680	FAM181B	220382
497356	11q	85724687	85725825	1139	6	0.5	Promoter(<=1kb)	0	SYTL2	54843
497356	11q	93697503	93700096	2594	11	0.636	Promoter(<=1kb)	0	CEP295	85459
497356	11q	123906595	123907324	730	7	0.429	Promoter(<=1kb)	449	OR8D4	338662
497356	11q	124015600	124016477	878	10	0.3	Promoter(<=1kb)	25	OR10G4	390264
497356	11q	124023038	124023849	812	9	0.444	Promoter(<=1kb)	25	OR10G9	219870
497356	12p	4626568	4628549	1982	11	0.455	Exon(exon5of6)	14051	DYRK4	8798
497356	12p	8222174	8223514	1341	6	0.667	Exon(exon5of6)	4073	FAM90A1	55138
497356	12p	31981130	31983004	1875	6	0.167	Exon(exon4of6)	-4162	RESF1	55196
497356	13q	25096870	25097182	313	14	0.5	Promoter(1-2kb)	1002	PABPC3	5042
497356	13q	102732474	102733933	1460	6	0.333	Exon(exon4of4)	25139	CCDC168	643677
497356	14q	20060048	20060884	837	8	0.625	Promoter(<=1kb)	3	OR4L1	122742
497356	14q	20640982	20641567	586	6	0.5	Promoter(<=1kb)	124	OR6S1	341799
497356	14q	21634137	21634589	453	9	0.556	Promoter(<=1kb)	351	OR10G2	26534
497356	14q	70457532	70458540	1009	9	0.333	Exon(exon2of2)	5358	ADAM21	8747
497356	14q	104939262	104942618	3357	10	0.2	5'UTR	7102	PLD4	122618
497356	14q	104943622	104946886	3265	11	0.636	Exon(exon6of6)	8516	AHNAK2	113146
497356	14q	104947901	104953878	5978	34	0.5	Promoter(1-2kb)	1524	AHNAK2	113146
497356	15q	23439979	23442067	2089	13	0.538	5'UTR	5167	GOLGA6L2	283685
497356	15q	100569472	100570097	626	6	0.833	Promoter(<=1kb)	534	LINS1	55180
497356	16p	669592	672548	2957	7	0.571	Promoter(<=1kb)	0	RHOT2	89941
497356	16p	1228744	1229731	988	9	0.667	Promoter(<=1kb)	431	TPSB2	64499
497356	16p	1256345	1256980	636	8	0.75	Promoter(<=1kb)	276	TPSD1	23430
497356	16p	1486371	1488463	2093	10	0.8	Promoter(<=1kb)	4	PTX4	390667
497356	16q	88713331	88717113	3783	10	0.5	Promoter(<=1kb)	0	MIR4722	100616167
497356	16q	89226863	89228419	1557	9	0.556	Promoter(2-3kb)	2229	ZNF778	197320
497356	17p	10638198	10641099	2902	7	0.286	Exon(exon19of41)	-8169	MYH3	4621
497356	17p	21300581	21300954	374	10	0.7	3'UTR	9112	MAP2K3	5606
497356	17p	21415470	21416416	947	7	0.857	Exon(exon3of3)	10334	KCNJ12	3768
497356	17q	41586466	41586829	364	6	0.833	Promoter(<=1kb)	66	KRT14	3861
497356	17q	76293419	76294016	598	6	0.5	Promoter(2-3kb)	-2167	QRICH2	84074
497356	17q	80090158	80090265	108	6	0.333	Promoter(2-3kb)	2053	CCDC40	55036
497356	17q	81645135	81645595	461	7	0.429	Promoter(2-3kb)	2722	TSPAN10	83882
497356	18p	11609646	11610219	574	15	0.867	Promoter(<=1kb)	50	SLC35G4	646000
497356	18q	58535186	58537515	2330	10	0.4	Promoter(<=1kb)	0	ALPK2	115701
497356	19p	1036457	1036914	458	6	0.5	Exon(exon6of7)	-3187	ABCA7	10347
497356	19p	4510548	4513547	3000	23	0.478	Exon(exon3of6)	4157	PLIN4	729359
497356	19p	5455600	5456439	840	7	0.571	Promoter(<=1kb)	183	ZNRF4	148066
497356	19p	8333830	8334965	1136	6	0.667	Promoter(<=1kb)	0	KANK3	256949
497356	19p	8937644	8939234	1591	6	0.833	Exon(exon5of84)	-41554	MUC16	94025
497356	19p	8946306	8951868	5563	16	0.625	Exon(exon3of84)	29474	MUC16	94025
497356	19p	8959116	8962299	3184	10	0.7	Exon(exon3of84)	19043	MUC16	94025
497356	19p	8972751	8978096	5346	16	0.5	Exon(exon1of84)	3246	MUC16	94025
497356	19p	12430157	12432437	2281	10	0.4	3'UTR	8584	ZNF443	10224
497356	19p	15087213	15088040	828	10	0.3	Promoter(<=1kb)	233	OR1I1	126370
497356	19p	17281820	17284246	2427	9	0.556	Promoter(<=1kb)	0	ANKLE1	126549
497356	19p	18264798	18267409	2612	12	0.583	5'UTR	7002	IQCN	80726
497356	19p	21971930	21974500	2571	7	0.714	Exon(exon4of4)	14408	ZNF208	7757
497356	19q	36996730	36997597	868	10	0.6	Exon(exon10of10)	5677	ZNF568	374900
497356	19q	40880231	40880622	392	6	0.333	Promoter(<=1kb)	-141	CYP2A7	1549
497356	19q	43846955	43848536	1582	7	0.857	3'UTR	13450	ZNF283	284349
497356	19q	43913423	43914878	1456	8	0.5	Exon(exon10of10)	4861	ZNF45	7596
497356	19q	44106512	44108078	1567	7	0	Exon(exon6of6)	-4103	ZNF225	7768
497356	19q	44327836	44329698	1863	6	0.5	Exon(exon4of4)	-22790	ZNF235	9310
497356	19q	52437918	52439242	1325	7	0.429	Exon(exon4of4)	6504	ZNF534	147658
497356	19q	55160006	55162286	2281	6	0.333	Promoter(<=1kb)	0	DNAAF3	352909
497356	19q	55911888	55913077	1190	6	0.667	Exon(exon5of12)	19234	NLRP13	126204
497356	21q	44600627	44601692	1066	10	0.7	Promoter(<=1kb)	30	KRTAP10-7	386675
497356	21q	44637474	44638143	670	8	0.5	Promoter(<=1kb)	118	KRTAP10-10	353333

497356	22q	22352950	22353348	399	14	0.571	Exon(exon1of2)	30478	BMS1P20	96610
497356	22q	36191154	36191906	753	6	0.667	3'UTR	9971	APOL4	80832
497356	22q	36265284	36265796	513	6	1	Exon(exon6of6)	12120	APOL1	8542
497356	22q	39100331	39102033	1703	6	0.833	Promoter(<=1kb)	52	APOBEC3H	164668
497356	23p	3320126	3323750	3625	9	0.556	Exon(exon5of7)	22902	MXRA5	25878
497356	23p	8170039	8170141	103	6	0.5	Promoter(1-2kb)	1126	VCX2	51480
497356	23p	35802148	35803010	863	7	0.571	5'UTR	3357	MAGEB16	139604
501796	1p	11766028	11768307	2280	6	0.833	Promoter(<=1kb)	0	C1orf167	284498
501796	1p	11778784	11779941	1158	7	0.714	Promoter(<=1kb)	0	C1orf167-AS1	102724659
501796	1p	16048038	16049824	1787	6	0.5	Promoter(<=1kb)	0	CLCNKB	1188
501796	1p	16058491	16060000	1510	14	0.857	Exon(exon5of7)	6168	CLCNKB	1188
501796	1p	18481403	18483159	1757	7	0.714	Promoter(<=1kb)	421	KLHDC7A	127707
501796	1p	23874604	23875430	827	8	0.5	Exon(exon2of2)	-6310	FUCA1	2517
501796	1p	40067594	40067675	82	6	0	Promoter(<=1kb)	324	CAP1	10487
501796	1q	152303673	152313891	10219	39	0.59	Promoter(<=1kb)	0	FLG-AS1	339400
501796	1q	156669844	156670886	1043	6	1	Exon(exon4of4)	6521	NES	10763
501796	1q	158765805	158766655	851	6	0.5	Promoter(<=1kb)	47	OR6N1	128372
501796	1q	226735563	226737239	1677	8	0.875	Promoter(<=1kb)	219	ITPKB	3707
501796	1q	228315976	228318026	2051	7	0.571	Exon(exon50of81)	6492	OBSCN	84033
501796	1q	247841312	247841582	271	6	0.833	Promoter(<=1kb)	314	OR11L1	391189
501796	1q	247949443	247949738	296	10	0.3	Promoter(<=1kb)	585	OR2L8	391190
501796	1q	248294677	248295458	782	6	0.833	Promoter(<=1kb)	142	OR2T12	127064
501796	1q	248574028	248574210	183	6	0.667	Promoter(<=1kb)	547	OR2T34	127068
501796	2p	29002636	29003646	1011	6	0.333	Exon(exon5of20)	-10821	TOGARAM2	165186
501796	2p	48580657	48582454	1798	7	0.571	Promoter(<=1kb)	0	STON1	11037
501796	2q	178739433	178741811	2379	6	0.5	Exon(exon45of191)	26014	TTN	7273
501796	2q	185789865	185794632	4768	10	0.8	Promoter(<=1kb)	0	FSIP2	401024
501796	2q	185805377	185808170	2794	6	0.333	Promoter(<=1kb)	0	FSIP2	401024
501796	2q	237762685	237764060	1376	10	0.5	Exon(exon8of8)	-4137	LRRFIP1	9208
501796	2q	240041845	240042154	310	6	0.167	Downstream(2-3kb)	3918	OR6B3	150681
501796	3p	75736880	75739007	2128	56	0.589	Promoter(<=1kb)	0	MIR4273	100422955
501796	3q	98264413	98265137	725	6	0.667	Promoter(<=1kb)	128	OR5H6	79295
501796	4p	5988383	5989749	1367	7	0.571	Promoter(<=1kb)	0	C4orf50	389197
501796	4p	6300792	6302360	1569	6	0.833	Exon(exon8of8)	6021	WFS1	7466
501796	4p	8227004	8228508	1505	8	0.125	Promoter(<=1kb)	-24	SH3TC1	54436
501796	4q	121036404	121037542	1139	6	0.333	Promoter(1-2kb)	1442	NDNF	79625
501796	4q	186619481	186621601	2121	7	0.286	Exon(exon10of27)	-9411	FAT1	2195
501796	4q	186706638	186708616	1979	6	0.5	Exon(exon2of27)	15217	FAT1	2195
501796	5q	140848579	140850786	2208	6	0.5	Promoter(<=1kb)	807	PCDHA9	9752
501796	5q	141174000	141175025	1026	6	0.833	Promoter(1-2kb)	1356	PCDHB7	56129
501796	5q	141183999	141184688	690	7	0.714	Promoter(2-3kb)	-2473	PCDHB9	56127
501796	5q	141187690	141189425	1736	6	0.5	Promoter(<=1kb)	529	PCDHB9	56127
501796	5q	141955676	141957660	1985	6	0.667	Promoter(<=1kb)	-668	RNF14	9604
501796	5q	148826877	148828070	1194	6	1	Promoter(1-2kb)	1632	ADRB2	154
501796	5q	151521550	151522069	520	6	0.667	Promoter(<=1kb)	79	MIR6499	102465246
501796	6p	1312843	1313745	903	6	0.5	Promoter(<=1kb)	745	FOXQ1	94234
501796	6p	46858771	46859502	732	8	0.5	Exon(exon17of21)	3802	ADGRF5	221395
501796	6q	64591274	64591961	688	10	0.5	Exon(exon26of43)	121374	EYS	346007
501796	6q	149888581	149890867	2287	7	0.714	Promoter(<=1kb)	0	RAET1E-AS1	100652739
501796	6q	159233455	159234370	916	10	0.5	Exon(exon11of23)	15158	FNDC1	84624
501796	7p	45082725	45084866	2142	8	0.5	Promoter(1-2kb)	-1325	NACAD	23148
501796	7q	64991321	64992758	1438	6	0.667	Promoter(<=1kb)	-285	ZNF117	51351
501796	7q	100958721	100960873	2153	55	0.473	Promoter(<=1kb)	756	MUC3A	4584
501796	7q	100991195	100992398	1204	7	0.571	Exon(exon5of15)	-20656	MUC12	10071
501796	7q	100995575	100995785	211	7	0.714	Exon(exon5of15)	-17269	MUC12	10071
501796	8p	8376561	8377198	638	6	1	Exon(exon2of5)	4549	PRAG1	157285
501796	8p	10607245	10608261	1017	7	0.571	Exon(exon4of4)	46882	RP1L1	94137
501796	8p	10609650	10612307	2658	10	0.7	Exon(exon4of4)	42836	RP1L1	94137
501796	8p	11331234	11332082	849	6	0.667	Promoter(<=1kb)	346	SLC35G5	83650
501796	8p	13021128	13022030	903	7	0.143	Exon(exon5of5)	9115	TRMT9B	57604
501796	8q	123651873	123652634	762	6	0.333	Promoter(<=1kb)	316	KLHL38	340359
501796	8q	138151949	138153046	1098	6	0.667	Promoter(<=1kb)	0	FAM135B	51059
501796	9p	21206764	21207074	311	6	0.5	Promoter(<=1kb)	69	IFNA10	3446
501796	9p	34723747	34726527	2781	11	0.727	Promoter(2-3kb)	2961	FAM205A	259308
501796	9q	76705724	76707804	2081	6	0.5	Promoter(<=1kb)	666	PCA3	50652
501796	9q	87885490	87888819	3330	8	0.625	Promoter(2-3kb)	2613	SPATA31E1	286234
501796	9q	104598545	104599318	774	6	0.333	Promoter(<=1kb)	95	OR13C5	138799
501796	9q	122553278	122554071	794	6	0.333	Promoter(<=1kb)	108	OR1N2	138882
501796	9q	122628595	122629398	804	7	0.286	Promoter(<=1kb)	175	OR1B1	347169
501796	9q	122749914	122750547	634	6	0.833	Promoter(<=1kb)	174	OR1L6	392390
501796	9q	135547960	135548795	836	6	0.667	Promoter(1-2kb)	1805	OBP2A	29991
501796	10q	46549378	46550723	1346	25	0.64	Exon(exon3of3)	4807	GPRIN2	9721
501796	10q	49323169	49326817	3649	13	0.615	Exon(exon3of3)	23895	C10orf71	118461
501796	10q	125026145	125027175	1031	7	0.571	Promoter(<=1kb)	853	CTBP2	1488
501796	11p	244106	244197	92	8	0.5	Promoter(<=1kb)	-232	PSMD13	5719
501796	11p	1246332	1248605	2274	11	0.545	Promoter(1-2kb)	1071	MUC5B-AS1	112577518
501796	11p	1835943	1837419	1477	6	0.667	Promoter(<=1kb)	0	SYT8	90019

501796	11p	4954783	4955581	799	7	0.714	Promoter(<=1kb)	132	OR51A2	401667
501796	11p	5177978	5178478	501	6	0.167	Promoter(<=1kb)	186	OR52Z1	283110
501796	11p	5323451	5324256	806	6	0.5	Promoter(<=1kb)	41	OR51B2	79345
501796	11p	5389704	5390350	647	6	0.5	Promoter(<=1kb)	327	OR51M1	390059
501796	11p	5422212	5423123	912	11	0.636	Promoter(<=1kb)	101	OR51Q1	390061
501796	11p	5440761	5441472	712	6	0.833	Promoter(<=1kb)	42	OR51I1	390063
501796	11p	5515185	5516015	831	6	0.333	Promoter(<=1kb)	684	UBQLNL	143630
501796	11p	5581045	5581738	694	8	0.375	Promoter(<=1kb)	168	OR52B6	340980
501796	11p	5841302	5841883	582	9	0.333	Promoter(<=1kb)	14	OR52E6	390078
501796	11p	5884818	5885061	244	7	0.429	Promoter(<=1kb)	547	OR52E4	390081
501796	11p	11351961	11352736	776	9	0.222	Promoter(<=1kb)	514	CSNK2A3	283106
501796	11p	12293788	12294842	1055	6	0.667	Exon(exon29of35)	6888	MICALCL	84953
501796	11p	34916266	34916763	498	6	0.667	Promoter(<=1kb)	0	APIP	51074
501796	11p	43942293	43943348	1056	10	0.8	Promoter(<=1kb)	0	C11orf96	387763
501796	11q	54603136	54603820	685	6	0.667	Promoter(<=1kb)	178	OR4C46	119749
501796	11q	58214757	58215722	966	7	0.286	Promoter(<=1kb)	12	OR1S1	219959
501796	11q	64315797	64315856	60	8	0.75	Promoter(1-2kb)	1485	TRMT112	51504
501796	11q	123906790	123907324	535	7	0.714	Promoter(<=1kb)	644	OR8D4	338662
501796	11q	124382526	124383285	760	8	0.625	Promoter(<=1kb)	58	OR8B2	26595
501796	12p	4626571	4628549	1979	8	0.5	Exon(exon5of6)	14054	DYRK4	8798
501796	12p	6453119	6453670	552	6	0.667	Promoter(<=1kb)	633	TAPBPL	55080
501796	13q	25096713	25097182	470	11	0.455	Promoter(<=1kb)	845	PABPC3	5042
501796	13q	39012206	39013752	1547	6	0.333	Promoter(<=1kb)	0	PROSER1	80209
501796	13q	49668389	49668431	43	6	0.167	Exon(exon3of3)	23007	EBPL	84650
501796	13q	102732474	102733933	1460	6	0.333	Exon(exon4of4)	25139	CCDC168	643677
501796	14q	20060048	20060884	837	8	0.625	Promoter(<=1kb)	3	OR4L1	122742
501796	14q	20224029	20224484	456	6	0.5	Promoter(<=1kb)	319	OR11H6	122748
501796	14q	21634137	21634589	453	9	0.556	Promoter(<=1kb)	351	OR10G2	26534
501796	14q	44504986	44506403	1418	6	0.667	Promoter(<=1kb)	880	FSCB	84075
501796	14q	70457520	70458540	1021	13	0.385	Exon(exon2of2)	5346	ADAM21	8747
501796	14q	104175275	104179622	4348	11	0.455	Exon(exon12of15)	36235	KIF26A	26153
501796	14q	104939262	104942618	3357	10	0.2	5'UTR	7102	PLD4	122618
501796	14q	104943622	104945444	1823	7	0.571	Exon(exon6of6)	9958	AHNAK2	113146
501796	14q	104947901	104953878	5978	34	0.559	Promoter(1-2kb)	1524	AHNAK2	113146
501796	15q	23439762	23442067	2306	13	0.538	5'UTR	5167	GOLGA6L2	283685
501796	15q	85579423	85582073	2651	15	0.6	Promoter(<=1kb)	-837	AKAP13	11214
501796	15q	99129423	99132517	3095	6	0.333	Exon(exon4of5)	7225	TTC23	64927
501796	15q	100569472	100570097	626	6	0.833	Promoter(<=1kb)	534	LINS1	55180
501796	16p	1228744	1229716	973	9	0.667	Promoter(<=1kb)	446	TPSB2	64499
501796	16p	1486371	1488463	2093	8	0.75	Promoter(<=1kb)	4	PTX4	390667
501796	16p	1998795	2000191	1397	7	0.571	Promoter(<=1kb)	0	ZNF598	90850
501796	16p	4207130	4208004	875	6	0.5	Exon(exon2of7)	31739	SRL	6345
501796	16q	74391549	74392004	456	9	0.667	Exon(exon7of7)	13671	NPIPB15	440348
501796	16q	88428539	88429600	1062	6	0.333	Exon(exon3of3)	-23680	ZFPM1	161882
501796	16q	89100686	89101050	365	7	0.571	Promoter(<=1kb)	24	ACSF3	197322
501796	16q	89226863	89228419	1557	10	0.6	Promoter(2-3kb)	2229	ZNF778	197320
501796	17p	744946	746966	2021	6	1	3'UTR	5072	GEMIN4	50628
501796	17p	10638198	10641099	2902	7	0.286	Exon(exon19of41)	-8169	MYH3	4621
501796	17p	21300581	21300954	374	8	0.75	3'UTR	9112	MAP2K3	5606
501796	17q	76293419	76294016	598	6	0.5	Promoter(2-3kb)	-2167	QRICH2	84074
501796	17q	81645135	81645417	283	6	0.333	Promoter(2-3kb)	2722	TSPAN10	83882
501796	18p	11609904	11610509	606	10	0.5	Promoter(<=1kb)	308	SLC35G4	646000
501796	18q	47033929	47035145	1217	6	1	Promoter(<=1kb)	476	ELOA2	51224
501796	18q	58535186	58538030	2845	19	0.526	Promoter(<=1kb)	0	ALPK2	115701
501796	19p	1004688	1005532	845	6	0.5	Exon(exon3of9)	4269	GRIN3B	116444
501796	19p	4510548	4513547	3000	18	0.5	Exon(exon3of6)	4157	PLIN4	729359
501796	19p	5455600	5456439	840	7	0.571	Promoter(<=1kb)	183	ZNRF4	148066
501796	19p	8948231	8952511	4281	12	0.5	Exon(exon3of84)	28831	MUC16	94025
501796	19p	17281820	17284246	2427	9	0.556	Promoter(<=1kb)	0	ANKLE1	126549
501796	19p	18264753	18267409	2657	8	0.5	5'UTR	7002	IQCN	80726
501796	19p	21971930	21974500	2571	8	0.75	Exon(exon4of4)	14408	ZNF208	7757
501796	19p	23743906	23745300	1395	6	0.333	Exon(exon4of4)	13537	ZNF681	148213
501796	19q	36996730	36997597	868	10	0.7	Exon(exon10of10)	5677	ZNF568	374900
501796	19q	37151928	37153149	1222	6	0.833	Exon(exon5of5)	19287	ZNF585A	199704
501796	19q	39877222	39877880	659	6	0.5	Exon(exon20of28)	9412	FCGBP	8857
501796	19q	40880231	40880622	392	6	0.333	Promoter(<=1kb)	-141	CYP2A7	1549
501796	19q	43846955	43848536	1582	6	0.833	3'UTR	13450	ZNF283	284349
501796	19q	43913423	43914878	1456	9	0.556	Exon(exon10of10)	4861	ZNF45	7596
501796	19q	44106512	44108078	1567	7	0	Exon(exon6of6)	-4103	ZNF225	7768
501796	19q	51745958	51746963	1006	6	0.5	Exon(exon3of3)	3848	FPR1	2357
501796	19q	52437918	52439242	1325	7	0.429	Exon(exon4of4)	6504	ZNF534	147658
501796	19q	55911888	55913077	1190	6	0.667	Exon(exon5of12)	19234	NLRP13	126204
501796	20p	5922421	5923394	974	7	0.571	Exon(exon4of5)	6923	CHGB	1114
501796	20p	20052354	20052736	383	6	0.167	Promoter(<=1kb)	0	CFAP61	26074
501796	20q	63349752	63350772	1021	6	0.5	3'UTR	3794	CHRNA4	1137
501796	20q	63561666	63565531	3866	12	0.667	Promoter(<=1kb)	-61	HELZ2	85441
501796	21q	44539312	44540070	759	8	0.625	Promoter(<=1kb)	125	KRTAP10-1	386677

501796	21q	44573798	44574611	814	6	0.5	Promoter(<=1kb)	0	TSPEAR	54084
501796	21q	44600627	44601692	1066	10	0.7	Promoter(<=1kb)	30	KRTAP10-7	386675
501796	21q	44637474	44638143	670	7	0.429	Promoter(<=1kb)	118	KRTAP10-10	353333
501796	22q	22352950	22353380	431	16	0.5	Exon(exon1of2)	30478	BMS1P20	96610
501796	22q	36191154	36191906	753	7	0.571	3'UTR	9971	APOL4	80832
501796	22q	37723206	37726455	3250	6	0.667	3'UTR	-19778	TRIOBP	11078
501796	23p	8170039	8170141	103	6	0.5	Promoter(1-2kb)	1126	VCX2	51480
501796	23p	35802148	35803010	863	7	0.571	5'UTR	3357	MAGEB16	139604
501919	1p	978953	979884	932	6	0.5	Promoter(1-2kb)	1145	PERM1	84808
501919	1p	12795495	12795749	255	7	0.286	Promoter(<=1kb)	804	PRAMEF1	65121
501919	1p	12847526	12847995	470	10	0.4	Promoter(<=1kb)	730	HNRNPCL1	343069
501919	1p	12859036	12860079	1044	6	0.333	Promoter(1-2kb)	1950	PRAMEF2	65122
501919	1p	12893187	12893472	286	12	0.333	Exon(exon4of4)	4798	PRAMEF10	343071
501919	1p	13370686	13370957	272	7	0.429	Promoter(<=1kb)	943	PRAMEF19	645414
501919	1p	16058491	16060000	1510	13	0.846	Exon(exon5of7)	6168	CLCNKB	1188
501919	1p	18481403	18482217	815	6	0.667	Promoter(<=1kb)	421	KLHDC7A	127707
501919	1p	23874604	23875430	827	8	0.5	Exon(exon2of2)	-6310	FUCA1	2517
501919	1p	40067594	40067675	82	6	0	Promoter(<=1kb)	324	CAP1	10487
501919	1p	89186388	89186419	32	9	0.556	Promoter(<=1kb)	107	GBP4	115361
501919	1q	152218469	152221375	2907	16	0.688	Promoter(2-3kb)	2818	HRNR	388697
501919	1q	152303673	152307444	3772	15	0.533	Exon(exon3of3)	-5132	FLG-AS1	339400
501919	1q	152308531	152313891	5361	18	0.722	Promoter(<=1kb)	0	FLG-AS1	339400
501919	1q	156669844	156670886	1043	6	1	Exon(exon4of4)	6521	NES	10763
501919	1q	158765805	158766655	851	6	0.5	Promoter(<=1kb)	47	OR6N1	128372
501919	1q	169542317	169542882	566	6	0.167	Exon(exon13of25)	-26572	F5	2153
501919	1q	197101312	197101771	460	6	0.5	Exon(exon18of28)	33373	ASPM	259266
501919	1q	201206099	201209342	3244	10	0.6	Promoter(1-2kb)	1017	IGFN1	91156
501919	1q	228315976	228318049	2074	8	0.625	Exon(exon50of81)	6492	OBSCN	84033
501919	1q	247841312	247841582	271	6	0.833	Promoter(<=1kb)	314	OR11L1	391189
501919	1q	247895950	247896410	461	6	0.5	Promoter(<=1kb)	363	OR2W3	343171
501919	1q	248294677	248295458	782	9	0.667	Promoter(<=1kb)	142	OR2T12	127064
501919	2q	130193975	130195087	1113	6	0.667	Exon(exon3of5)	3352	TUBA3E	112714
501919	2q	132783061	132784972	1912	7	0.429	Promoter(1-2kb)	-1038	NCKAP5	344148
501919	2q	184936178	184937636	1459	6	0.333	Exon(exon4of4)	69813	ZNF804A	91752
501919	2q	217847583	217848559	977	6	0.833	Exon(exon19of33)	-5423	TNS1	7145
501919	2q	233681970	233682693	724	6	0.667	Promoter(<=1kb)	0	UGT1A7	54577
501919	2q	233713134	233713783	650	13	0.692	Promoter(<=1kb)	142	UGT1A5	54579
501919	2q	233840612	233842185	1574	6	0.667	Promoter(<=1kb)	0	HJURP	55355
501919	2q	238130416	238131546	1131	6	0.333	Promoter(1-2kb)	1468	ESPNL	339768
501919	2q	240041845	240042154	310	6	0.167	Downstream(2-3kb)	3918	OR6B3	150681
501919	3p	75736880	75739243	2364	41	0.61	Promoter(<=1kb)	0	MIR4273	100422955
501919	3q	196947878	196948448	571	7	0.571	3'UTR	3482	PIGZ	80235
501919	4p	5988383	5989749	1367	7	0.571	Promoter(<=1kb)	0	C4orf50	389197
501919	4p	6300792	6302360	1569	8	0.75	Exon(exon8of8)	6021	WFS1	7466
501919	4p	8227004	8228508	1505	8	0.25	Promoter(<=1kb)	-24	SH3TC1	54436
501919	4p	38796894	38798702	1809	6	0.5	Exon(exon4of4)	5674	TLR1	7096
501919	4q	121036404	121037542	1139	6	0.333	Promoter(1-2kb)	1442	NDNF	79625
501919	4q	186619481	186621601	2121	7	0.286	Exon(exon10of27)	-9411	FAT1	2195
501919	4q	186706638	186709751	3114	7	0.714	Exon(exon2of27)	14082	FAT1	2195
501919	5q	79728956	79730716	1761	6	0.333	Exon(exon2of13)	-7426	CMYA5	202333
501919	5q	79731782	79734523	2742	13	0.308	Exon(exon2of13)	-3619	CMYA5	202333
501919	5q	141174000	141175025	1026	6	0.833	Promoter(1-2kb)	1356	PCDHB7	56129
501919	5q	141955676	141957689	2014	7	0.714	Promoter(<=1kb)	-639	RNF14	9604
501919	5q	151565922	151568158	2237	9	0.778	Promoter(<=1kb)	786	FAT2	2196
501919	6p	16327099	16328014	916	7	0.429	Exon(exon8of9)	37154	GMPR	2766
501919	6p	46858771	46859502	732	7	0.571	Exon(exon17of21)	3802	ADGRF5	221395
501919	6q	149888581	149890867	2287	7	0.714	Promoter(<=1kb)	0	RAET1E-AS1	100652739
501919	6q	159233455	159234370	916	10	0.5	Exon(exon11of23)	15158	FNDC1	84624
501919	7p	6330446	6330944	499	6	1	Exon(exon2of2)	7749	FAM220A	84792
501919	7q	100958721	100960873	2153	57	0.456	Promoter(<=1kb)	756	MUC3A	4584
501919	7q	100991195	100993127	1933	9	0.667	Exon(exon5of15)	-19927	MUC12	10071
501919	7q	100995575	100995785	211	6	0.833	Exon(exon5of15)	-17269	MUC12	10071
501919	8p	10607245	10608432	1188	8	0.5	Exon(exon4of4)	46711	RP1L1	94137
501919	8p	10609614	10610662	1049	8	0.5	Exon(exon4of4)	44481	RP1L1	94137
501919	8p	11331234	11332082	849	6	0.667	Promoter(<=1kb)	346	SLC35G5	83650
501919	8p	12132477	12133940	1464	11	0.636	Promoter(<=1kb)	498	USP17L7	392197
501919	8p	13021128	13022030	903	7	0.143	Exon(exon5of5)	9115	TRMT9B	57604
501919	8q	123651655	123652634	980	7	0.571	Promoter(<=1kb)	316	KLHL38	340359
501919	8q	143916360	143919209	2850	7	0.143	Exon(exon32of32)	20381	PLEC	5339
501919	9q	76175237	76175296	60	9	0.778	Exon(exon14of14)	-13343	PCSK5	5125
501919	9q	76705179	76707804	2626	8	0.5	Promoter(<=1kb)	121	PCA3	50652
501919	9q	76709263	76710843	1581	8	0.5	Promoter(<=1kb)	0	PRUNE2	158471
501919	9q	87886533	87888536	2004	7	0.571	Exon(exon4of4)	3656	SPATA31E1	286234
501919	9q	114167914	114169457	1544	6	0.5	Promoter(<=1kb)	0	COL27A1	85301
501919	9q	122553263	122554071	809	7	0.429	Promoter(<=1kb)	93	OR1N2	138882
501919	9q	122628595	122629398	804	6	0.333	Promoter(<=1kb)	175	OR1B1	347169
501919	9q	122749914	122750547	634	6	0.833	Promoter(<=1kb)	174	OR1L6	392390

501919	9q	133255635	133256205	571	7	1	3'UTR	19009	ABO	28
501919	9q	135484803	135487213	2411	9	0.333	Promoter(1-2kb)	1440	PPP1R26	9858
501919	10p	30027143	30029814	2672	7	0.429	Exon(exon3of4)	29772	JCAD	57608
501919	10q	46549378	46550723	1346	26	0.654	Exon(exon3of3)	4807	GPRIN2	9721
501919	10q	49323169	49326817	3649	13	0.615	Exon(exon3of3)	23895	C10orf71	118461
501919	10q	122084988	122087840	2853	9	0.778	Exon(exon4of23)	-25190	TACC2	10579
501919	10q	128103129	128104830	1702	10	0.6	Promoter(<=1kb)	-1	MKI67	4288
501919	11p	244106	244197	92	8	0.5	Promoter(<=1kb)	-232	PSMD13	5719
501919	11p	1246332	1248605	2274	10	0.5	Promoter(1-2kb)	1071	MUC5B-AS1	112577518
501919	11p	5177811	5178478	668	7	0.143	Promoter(<=1kb)	186	OR52Z1	283110
501919	11p	5323362	5324256	895	7	0.429	Promoter(<=1kb)	41	OR51B2	79345
501919	11p	5389704	5390350	647	7	0.429	Promoter(<=1kb)	327	OR51M1	390059
501919	11p	5402638	5403471	834	11	0.455	Promoter(<=1kb)	41	OR51J1	79470
501919	11p	5422212	5423123	912	10	0.7	Promoter(<=1kb)	101	OR51Q1	390061
501919	11p	5581045	5581738	694	8	0.375	Promoter(<=1kb)	168	OR52B6	340980
501919	11p	5841302	5841883	582	9	0.333	Promoter(<=1kb)	14	OR52E6	390078
501919	11p	11351961	11352736	776	9	0.222	Promoter(<=1kb)	514	CSNK2A3	283106
501919	11p	12293628	12294842	1215	8	0.75	Exon(exon29of35)	6728	MICALCL	84953
501919	11p	18173280	18173901	622	6	0.333	Promoter(<=1kb)	443	MRGPRX4	117196
501919	11q	58402523	58403265	743	8	0.5	Promoter(<=1kb)	144	OR5B3	441608
501919	11q	85724687	85725825	1139	6	0.5	Promoter(<=1kb)	0	SYTL2	54843
501919	11q	130914501	130915409	909	10	0.7	Promoter(1-2kb)	1035	SNX19	399979
501919	12p	4626571	4628549	1979	8	0.5	Exon(exon5of6)	14054	DYRK4	8798
501919	12p	6453119	6453670	552	6	0.667	Promoter(<=1kb)	633	TAPBPL	55080
501919	12q	52316096	52317765	1670	6	0.667	Exon(exon4of9)	3633	KRT83	3889
501919	12q	52571389	52573652	2264	6	0.667	Promoter(<=1kb)	164	KRT74	121391
501919	13q	102732474	102733933	1460	6	0.333	Exon(exon4of4)	25139	CCDC168	643677
501919	14q	20060048	20060884	837	8	0.625	Promoter(<=1kb)	3	OR4L1	122742
501919	14q	21634137	21634589	453	9	0.556	Promoter(<=1kb)	351	OR10G2	26534
501919	14q	70457532	70458540	1009	9	0.333	Exon(exon2of2)	5358	ADAM21	8747
501919	14q	104939262	104951557	12296	53	0.547	5'UTR	3845	AHNAK2	113146
501919	15q	23439979	23442067	2089	11	0.545	5'UTR	5167	GOLGA6L2	283685
501919	15q	73702465	73703760	1296	6	0.833	Promoter(<=1kb)	-149	CD276	80381
501919	15q	78766033	78766626	594	7	0.571	Promoter(<=1kb)	-920	ADAMTS7	11173
501919	15q	85579423	85581800	2378	14	0.571	Promoter(1-2kb)	-1110	AKAP13	11214
501919	15q	88854874	88855594	721	6	0.833	Exon(exon12of18)	7631	ACAN	176
501919	15q	88857108	88859365	2258	6	0	Exon(exon12of18)	9865	ACAN	176
501919	16p	789084	790597	1514	6	0.167	Promoter(<=1kb)	0	CHTF18	63922
501919	16p	1256345	1256980	636	6	0.833	Promoter(<=1kb)	276	TPSD1	23430
501919	16p	1486371	1488432	2062	7	0.714	Promoter(<=1kb)	35	PTX4	390667
501919	16p	1998795	2000191	1397	8	0.75	Promoter(<=1kb)	0	ZNF598	90850
501919	16q	74391650	74392004	355	7	0.571	Exon(exon7of7)	13772	NPIPB15	440348
501919	16q	88430623	88431933	1311	6	0.5	Exon(exon3of3)	-21347	ZFPM1	161882
501919	16q	88714717	88717113	2397	6	0.5	Promoter(<=1kb)	0	MIR4722	100616167
501919	16q	89226863	89228289	1427	7	0.571	Promoter(2-3kb)	2229	ZNF778	197320
501919	17p	10638198	10641099	2902	7	0.286	Exon(exon19of41)	-8169	MYH3	4621
501919	17p	21300581	21300978	398	11	0.727	3'UTR	9112	MAP2K3	5606
501919	17q	76293419	76294016	598	6	0.5	Promoter(2-3kb)	-2167	QRICH2	84074
501919	17q	81645135	81645417	283	6	0.333	Promoter(2-3kb)	2722	TSPAN10	83882
501919	18p	11609904	11610469	566	8	0.625	Promoter(<=1kb)	308	SLC35G4	646000
501919	18p	14542649	14543140	492	6	0.5	Promoter(<=1kb)	6	POTEC	388468
501919	18q	58535186	58538030	2845	19	0.526	Promoter(<=1kb)	0	ALPK2	115701
501919	19p	1004711	1005532	822	7	0.571	Exon(exon3of9)	4292	GRIN3B	116444
501919	19p	1036457	1036914	458	6	0.5	Exon(exon6of7)	-3187	ABCA7	10347
501919	19p	4510711	4513547	2837	15	0.467	Exon(exon3of6)	4157	PLIN4	729359
501919	19p	5455600	5456439	840	8	0.625	Promoter(<=1kb)	183	ZNRF4	148066
501919	19p	8937644	8939234	1591	6	0.667	Exon(exon5of84)	-41554	MUC16	94025
501919	19p	8946313	8951868	5556	19	0.632	Exon(exon3of84)	29474	MUC16	94025
501919	19p	8959116	8962299	3184	11	0.727	Exon(exon3of84)	19043	MUC16	94025
501919	19p	8971838	8978096	6259	15	0.533	Exon(exon1of84)	3246	MUC16	94025
501919	19p	12430718	12432437	1720	8	0.375	3'UTR	8584	ZNF443	10224
501919	19p	15087213	15088040	828	11	0.273	Promoter(<=1kb)	233	OR1I1	126370
501919	19p	18264798	18267409	2612	13	0.615	5'UTR	7002	IQCN	80726
501919	19p	21971930	21974500	2571	11	0.636	Exon(exon4of4)	14408	ZNF208	7757
501919	19p	22756294	22759533	3240	11	0.545	3'UTR	10449	ZNF99	7652
501919	19q	39877222	39877880	659	6	0.5	Exon(exon20of28)	9412	FCGBP	8857
501919	19q	39886240	39886422	183	6	0.667	Promoter(<=1kb)	870	FCGBP	8857
501919	19q	40880128	40880622	495	7	0.286	Promoter(<=1kb)	-38	CYP2A7	1549
501919	19q	43846955	43848536	1582	6	0.833	3'UTR	13450	ZNF283	284349
501919	19q	43913423	43914878	1456	7	0.429	Exon(exon10of10)	4861	ZNF45	7596
501919	19q	43996326	43997366	1041	6	0.5	Exon(exon5of5)	5419	LOC10192806:	101928063
501919	19q	44106512	44108078	1567	7	0	Exon(exon6of6)	-4103	ZNF225	7768
501919	19q	44327836	44329698	1863	6	0.5	Exon(exon4of4)	-22790	ZNF235	9310
501919	19q	48873325	48875925	2601	11	0.545	Promoter(<=1kb)	904	PPP1R15A	23645
501919	19q	52437918	52439242	1325	7	0.429	Exon(exon4of4)	6504	ZNF534	147658
501919	19q	52766361	52767718	1358	6	0.667	Exon(exon5of9)	18982	ZNF600	162966
501919	19q	55358736	55359651	916	7	0.714	Promoter(<=1kb)	0	FAM71E2	284418

501919	19q	55517821	55518642	822	8	1	Exon(exon14of14)	17661	SBK2	646643
501919	19q	58368293	58368875	583	7	0.429	Exon(exon3of3)	-5445	ZNF497	162968
501919	20p	1635288	1636423	1136	6	0.333	Promoter(1-2kb)	1662	SIRPG-AS1	101929010
501919	20p	20052354	20052736	383	6	0	Promoter(<=1kb)	0	CFAP61	26074
501919	20q	63561666	63565531	3866	12	0.667	Promoter(<=1kb)	-61	HELZ2	85441
501919	21q	26843740	26844859	1120	6	0.667	Promoter(<=1kb)	0	ADAMTS1	9510
501919	21q	44550835	44551416	582	6	0.833	Promoter(<=1kb)	89	KRTAP10-2	386679
501919	21q	44637476	44638143	668	9	0.444	Promoter(<=1kb)	120	KRTAP10-10	353333
501919	22q	22352950	22353380	431	16	0.5	Exon(exon1of2)	30478	BMS1P20	96610
501919	22q	36191154	36191906	753	6	0.667	3'UTR	9971	APOL4	80832
501919	23p	8170039	8170141	103	6	0.5	Promoter(1-2kb)	1126	VCX2	51480
501919	23p	35802148	35803010	863	7	0.571	5'UTR	3357	MAGEB16	139604
502448	1p	16058491	16060000	1510	11	0.818	Exon(exon5of7)	6168	CLCNKB	1188
502448	1p	18481403	18482217	815	6	0.667	Promoter(<=1kb)	421	KLHDC7A	127707
502448	1p	40067594	40067675	82	6	0	Promoter(<=1kb)	324	CAP1	10487
502448	1q	152303673	152313911	10239	41	0.61	Promoter(<=1kb)	0	FLG-AS1	339400
502448	1q	156669844	156670886	1043	7	0.857	Exon(exon4of4)	6521	NES	10763
502448	1q	158765805	158766655	851	6	0.5	Promoter(<=1kb)	47	OR6N1	128372
502448	1q	201206099	201209342	3244	10	0.7	Promoter(1-2kb)	1017	IGFN1	91156
502448	1q	201210956	201212866	1911	6	0.333	Promoter(<=1kb)	0	IGFN1	91156
502448	1q	214640144	214642954	2811	12	0.5	Exon(exon12of20)	-5013	CENPF	1063
502448	1q	214644872	214647181	2310	10	0.4	Promoter(<=1kb)	-786	CENPF	1063
502448	1q	228315976	228318049	2074	7	0.571	Exon(exon50of81)	6492	OBSCN	84033
502448	1q	247841312	247841582	271	6	0.833	Promoter(<=1kb)	314	OR11L1	391189
502448	1q	247949325	247949738	414	10	0.3	Promoter(<=1kb)	467	OR2L8	391190
502448	1q	248273309	248273670	362	6	0.5	Promoter(<=1kb)	166	OR2T33	391195
502448	1q	248294677	248295458	782	6	0.667	Promoter(<=1kb)	142	OR2T12	127064
502448	2p	29002636	29003646	1011	6	0.333	Exon(exon5of20)	-10821	TOGARAM2	165186
502448	2p	48580657	48582454	1798	7	0.571	Promoter(<=1kb)	0	STON1	11037
502448	2q	130914528	130916712	2185	6	0.333	Promoter(<=1kb)	0	ARHGEF4	50649
502448	2q	132781078	132784972	3895	11	0.545	Promoter(<=1kb)	0	NCKAP5	344148
502448	2q	184936178	184937636	1459	6	0.333	Exon(exon4of4)	69813	ZNF804A	91752
502448	2q	217847583	217848746	1164	7	0.857	Exon(exon19of33)	-5423	TNS1	7145
502448	2q	238130416	238131546	1131	6	0.333	Promoter(1-2kb)	1468	ESPNL	339768
502448	2q	240041845	240042811	967	8	0.375	Downstream(2-3kb)	3261	OR6B3	150681
502448	3p	31989532	31990905	1374	7	0.286	Exon(exon2of2)	7761	ZNF860	344787
502448	3p	75737230	75739007	1778	9	0.556	Promoter(<=1kb)	0	MIR4273	100422955
502448	3q	98169021	98169594	574	6	0.667	Exon(exon2of2)	19695	OR5H14	403273
502448	3q	98264413	98265098	686	7	0.571	Promoter(<=1kb)	128	OR5H6	79295
502448	4p	1394625	1395373	749	11	0.364	Exon(exon1of1)	9813	UVSSA	57654
502448	4p	5988383	5989749	1367	7	0.571	Promoter(<=1kb)	0	C4orf50	389197
502448	4p	6300792	6302360	1569	7	0.857	Exon(exon8of8)	6021	WFS1	7466
502448	4p	7433331	7434512	1182	6	0.833	Promoter(<=1kb)	418	PSAPL1	768239
502448	4p	8227004	8228508	1505	8	0.25	Promoter(<=1kb)	-24	SH3TC1	54436
502448	4p	38773164	38774870	1707	6	0.333	Promoter(1-2kb)	1326	TLR10	81793
502448	4q	112431241	112432293	1053	8	0.5	3'UTR	4735	ALPK1	80216
502448	5q	140848579	140850786	2208	6	0.5	Promoter(<=1kb)	807	PCDHA9	9752
502448	5q	141174000	141175025	1026	6	0.833	Promoter(1-2kb)	1356	PCDHB7	56129
502448	5q	141955676	141957660	1985	6	0.667	Promoter(<=1kb)	-668	RNF14	9604
502448	5q	151565922	151568455	2534	10	0.8	Promoter(<=1kb)	489	FAT2	2196
502448	6p	46858771	46859502	732	7	0.571	Exon(exon17of21)	3802	ADGRF5	221395
502448	6q	159231899	159234370	2472	10	0.5	Exon(exon11of23)	13602	FNDC1	84624
502448	7q	100958977	100960873	1897	54	0.444	Promoter(1-2kb)	1012	MUC3A	4584
502448	7q	100991195	100992438	1244	8	0.5	Exon(exon5of15)	-20616	MUC12	10071
502448	7q	100995575	100995785	211	7	0.714	Exon(exon5of15)	-17269	MUC12	10071
502448	7q	101034221	101041312	7092	48	0.542	Promoter(2-3kb)	-2399	MUC17	140453
502448	8p	8376561	8377220	660	6	1	Exon(exon2of5)	4527	PRAG1	157285
502448	8p	10607245	10608432	1188	8	0.5	Exon(exon4of4)	46711	RP1L1	94137
502448	8p	10609697	10610142	446	6	0.333	Exon(exon4of4)	45001	RP1L1	94137
502448	8p	12132686	12133940	1255	7	0.714	Promoter(<=1kb)	498	USP17L7	392197
502448	8p	13021128	13022030	903	7	0.143	Exon(exon5of5)	9115	TRMT9B	57604
502448	9p	116800	117800	1001	7	0.857	Promoter(<=1kb)	404	FOXD4	2298
502448	9p	21206764	21207074	311	6	0.5	Promoter(<=1kb)	69	IFNA10	3446
502448	9q	76175237	76175300	64	6	0.5	Exon(exon14of14)	-13339	PCSK5	5125
502448	9q	87886533	87888536	2004	7	0.571	Exon(exon4of4)	3656	SPATA31E1	286234
502448	9q	104504315	104505071	757	6	0.5	Promoter(<=1kb)	52	OR13F1	138805
502448	9q	104598545	104599318	774	6	0.333	Promoter(<=1kb)	95	OR13C5	138799
502448	9q	122553263	122554071	809	7	0.429	Promoter(<=1kb)	93	OR1N2	138882
502448	9q	122749914	122750547	634	6	0.833	Promoter(<=1kb)	174	OR1L6	392390
502448	9q	133255635	133256205	571	8	0.875	3'UTR	19009	ABO	28
502448	9q	135484803	135487156	2354	7	0.143	Promoter(1-2kb)	1440	PPP1R26	9858
502448	10q	46549378	46550723	1346	27	0.667	Exon(exon3of3)	4807	GPRIN2	9721
502448	11p	244106	244197	92	8	0.5	Promoter(<=1kb)	-232	PSMD13	5719
502448	11p	1246095	1248605	2511	13	0.538	Promoter(1-2kb)	1071	MUC5B-AS1	112577518
502448	11p	1250091	1252374	2284	7	0.857	Promoter(<=1kb)	-415	MUC5B-AS1	112577518
502448	11p	5177978	5178478	501	6	0.167	Promoter(<=1kb)	186	OR52Z1	283110
502448	11p	5323542	5324256	715	6	0.5	Promoter(<=1kb)	41	OR51B2	79345

502448	11p	5351521	5352416	896	15	0.467	Promoter(<=1kb)	13	OR51B6	390058
502448	11p	5389704	5390350	647	7	0.429	Promoter(<=1kb)	327	OR51M1	390059
502448	11p	5422212	5423123	912	11	0.636	Promoter(<=1kb)	101	OR51Q1	390061
502448	11p	5515079	5515931	853	7	0.429	Promoter(<=1kb)	768	UBQLNL	143630
502448	11p	5544676	5545259	584	6	0.5	Promoter(<=1kb)	290	OR52H1	390067
502448	11p	5581045	5581738	694	9	0.444	Promoter(<=1kb)	168	OR52B6	340980
502448	11p	5788000	5788760	761	8	0.75	Promoter(<=1kb)	56	OR52N1	79473
502448	11p	5841302	5841883	582	9	0.333	Promoter(<=1kb)	14	OR52E6	390078
502448	11p	11351961	11352736	776	9	0.222	Promoter(<=1kb)	514	CSNK2A3	283106
502448	11p	12293639	12294538	900	8	0.625	Exon(exon29of35)	6739	MICALCL	84953
502448	11p	18173280	18173901	622	6	0.333	Promoter(<=1kb)	443	MRGPRX4	117196
502448	11p	43942293	43943348	1056	9	0.778	Promoter(<=1kb)	0	C11orf96	387763
502448	11q	55827536	55827640	105	6	0.667	Promoter(<=1kb)	317	OR5L2	26338
502448	11q	58214757	58215722	966	8	0.25	Promoter(<=1kb)	12	OR1S1	219959
502448	11q	85724687	85725825	1139	6	0.5	Promoter(<=1kb)	0	SYTL2	54843
502448	11q	124015601	124016477	877	8	0.25	Promoter(<=1kb)	26	OR10G4	390264
502448	11q	124023038	124023849	812	8	0.5	Promoter(<=1kb)	25	OR10G9	219870
502448	11q	130914501	130915409	909	10	0.7	Promoter(1-2kb)	1035	SNX19	399979
502448	12p	4626568	4628549	1982	11	0.455	Exon(exon5of6)	14051	DYRK4	8798
502448	12q	52316096	52317765	1670	6	0.667	Exon(exon4of9)	3633	KRT83	3889
502448	13q	102732474	102733933	1460	6	0.333	Exon(exon4of4)	25139	CCDC168	643677
502448	14q	20060048	20060884	837	8	0.625	Promoter(<=1kb)	3	OR4L1	122742
502448	14q	20640982	20641567	586	6	0.5	Promoter(<=1kb)	124	OR6S1	341799
502448	14q	21634137	21634589	453	9	0.556	Promoter(<=1kb)	351	OR10G2	26534
502448	14q	70457520	70458540	1021	9	0.333	Exon(exon2of2)	5346	ADAM21	8747
502448	14q	94587512	94587839	328	6	0.5	Exon(exon2of2)	-4219	SERPINA3	12
502448	15q	20534480	20535056	577	8	0.375	Exon(exon8of9)	6744	GOLGA6L6	727832
502448	15q	23439979	23442067	2089	12	0.583	5'UTR	5167	GOLGA6L2	283685
502448	15q	40621642	40623696	2055	6	0.333	Promoter(<=1kb)	0	KNL1	57082
502448	15q	100569472	100570097	626	6	0.833	Promoter(<=1kb)	534	LINS1	55180
502448	16p	1486371	1488463	2093	8	0.75	Promoter(<=1kb)	4	PTX4	390667
502448	16q	88430623	88431933	1311	6	0.5	Exon(exon3of3)	-21347	ZFPM1	161882
502448	16q	89226863	89228289	1427	7	0.571	Promoter(2-3kb)	2229	ZNF778	197320
502448	17p	413503	413831	329	7	0.571	Promoter(<=1kb)	-120	LOC10537143(	105371430
502448	17p	744917	746695	1779	7	0.857	3'UTR	5343	GEMIN4	50628
502448	17p	10638198	10641099	2902	7	0.286	Exon(exon19of41)	-8169	MYH3	4621
502448	17p	21300581	21300978	398	9	0.778	3'UTR	9112	MAP2K3	5606
502448	17q	76293419	76294016	598	6	0.5	Promoter(2-3kb)	-2167	QRICH2	84074
502448	17q	81645135	81645417	283	6	0.333	Promoter(2-3kb)	2722	TSPAN10	83882
502448	18p	11609646	11610581	936	16	0.688	Promoter(<=1kb)	50	SLC35G4	646000
502448	18q	58534999	58537536	2538	12	0.417	Promoter(<=1kb)	0	ALPK2	115701
502448	19p	1036457	1036914	458	6	0.5	Exon(exon6of7)	-3187	ABCA7	10347
502448	19p	4510548	4513547	3000	21	0.429	Exon(exon3of6)	4157	PLIN4	729359
502448	19p	5455600	5456439	840	8	0.625	Promoter(<=1kb)	183	ZNRF4	148066
502448	19p	8937644	8939234	1591	6	0.667	Exon(exon5of84)	-41554	MUC16	94025
502448	19p	8946313	8952171	5859	20	0.6	Exon(exon3of84)	29171	MUC16	94025
502448	19p	8959116	8962299	3184	12	0.75	Exon(exon3of84)	19043	MUC16	94025
502448	19p	8971838	8978096	6259	16	0.562	Exon(exon1of84)	3246	MUC16	94025
502448	19p	17282085	17284246	2162	8	0.5	Promoter(<=1kb)	0	ANKLE1	126549
502448	19p	18264798	18267409	2612	12	0.583	5'UTR	7002	IQCN	80726
502448	19p	21971930	21974500	2571	10	0.7	Exon(exon4of4)	14408	ZNF208	7757
502448	19p	22756205	22759533	3329	22	0.545	3'UTR	10449	ZNF99	7652
502448	19q	37886924	37889059	2136	6	0.833	Exon(exon6of6)	17535	WDR87	83889
502448	19q	39877222	39877880	659	6	0.5	Exon(exon20of28)	9412	FCGBP	8857
502448	19q	39886240	39886422	183	6	0.667	Promoter(<=1kb)	870	FCGBP	8857
502448	19q	40880231	40880622	392	6	0.333	Promoter(<=1kb)	-141	CYP2A7	1549
502448	19q	43203948	43205504	1557	6	0.667	Promoter(<=1kb)	0	PSG4	5672
502448	19q	43846955	43848536	1582	6	0.833	3'UTR	13450	ZNF283	284349
502448	19q	43913423	43914878	1456	8	0.5	Exon(exon10of10)	4861	ZNF45	7596
502448	19q	43966037	43967171	1135	6	0.167	Promoter(<=1kb)	-691	ZNF155	7711
502448	19q	44106512	44108078	1567	8	0.125	Exon(exon6of6)	-4103	ZNF225	7768
502448	19q	48873325	48875925	2601	11	0.545	Promoter(<=1kb)	904	PPP1R15A	23645
502448	19q	55911888	55913077	1190	6	0.667	Exon(exon5of12)	19234	NLRP13	126204
502448	20q	63349752	63350772	1021	6	0.5	3'UTR	3794	CHRNA4	1137
502448	20q	63561666	63565531	3866	13	0.615	Promoter(<=1kb)	-61	HELZ2	85441
502448	22q	22352950	22353380	431	16	0.5	Exon(exon1of2)	30478	BMS1P20	96610
502448	22q	36191154	36191906	753	7	0.571	3'UTR	9971	APOL4	80832
502448	23p	3320126	3323750	3625	10	0.6	Exon(exon5of7)	22902	MXRA5	25878
502448	23p	8170039	8170141	103	6	0.5	Promoter(1-2kb)	1126	VCX2	51480
502448	23p	35802148	35803010	863	7	0.571	5'UTR	3357	MAGEB16	139604
502448	23p	38285569	38287917	2349	7	1	3'UTR	-11627	RPGR	6103
502828	1p	11766028	11768307	2280	6	0.833	Promoter(<=1kb)	0	C1orf167	284498
502828	1p	11778784	11779941	1158	7	0.714	Promoter(<=1kb)	0	C1orf167-AS1	102724659
502828	1p	12892936	12893472	537	19	0.421	Exon(exon4of4)	4798	PRAMEF10	343071
502828	1p	16058512	16060000	1489	8	0.75	Exon(exon5of7)	6189	CLCNKB	1188
502828	1p	18481403	18483159	1757	8	0.75	Promoter(<=1kb)	421	KLHDC7A	127707
502828	1p	23874604	23875430	827	8	0.5	Exon(exon2of2)	-6310	FUCA1	2517

502828	1p	40067594	40067675	82	6	0	Promoter(<=1kb)	324	CAP1	10487
502828	1p	89186388	89186419	32	9	0.556	Promoter(<=1kb)	107	GBP4	115361
502828	1q	145872200	145873487	1288	6	0.667	Exon(exon8of12)	-12364	PIAS3	10401
502828	1q	152218469	152221375	2907	16	0.688	Promoter(2-3kb)	2818	HRNR	388697
502828	1q	152303673	152313891	10219	37	0.595	Promoter(<=1kb)	0	FLG-AS1	339400
502828	1q	158765805	158766655	851	6	0.5	Promoter(<=1kb)	47	OR6N1	128372
502828	1q	169542317	169542882	566	6	0.167	Exon(exon13of25)	-26572	F5	2153
502828	1q	228315976	228318026	2051	6	0.5	Exon(exon50of81)	6492	OBSCN	84033
502828	1q	247841312	247841582	271	6	0.833	Promoter(<=1kb)	314	OR11L1	391189
502828	1q	247949443	247949738	296	9	0.333	Promoter(<=1kb)	585	OR2L8	391190
502828	1q	248294677	248295458	782	7	0.571	Promoter(<=1kb)	142	OR2T12	127064
502828	2p	48580657	48582454	1798	7	0.571	Promoter(<=1kb)	0	STON1	11037
502828	2q	184936178	184937636	1459	6	0.333	Exon(exon4of4)	69813	ZNF804A	91752
502828	2q	185789865	185794632	4768	10	0.8	Promoter(<=1kb)	0	FSIP2	401024
502828	2q	219271337	219271649	313	6	0.667	Exon(exon4of4)	6227	TUBA4A	7277
502828	2q	238130271	238131546	1276	7	0.286	Promoter(1-2kb)	1323	ESPNL	339768
502828	2q	240041845	240042154	310	6	0.167	Downstream(2-3kb)	3918	OR6B3	150681
502828	3p	52521941	52524117	2177	6	0.333	Promoter(<=1kb)	0	STAB1	23166
502828	3p	75736929	75738859	1931	11	0.545	Promoter(<=1kb)	0	MIR4273	100422955
502828	3q	98264413	98265098	686	7	0.571	Promoter(<=1kb)	128	OR5H6	79295
502828	4p	5988383	5989749	1367	7	0.571	Promoter(<=1kb)	0	C4orf50	389197
502828	4p	6300792	6302360	1569	9	0.778	Exon(exon8of8)	6021	WFS1	7466
502828	4p	8227004	8228508	1505	8	0.125	Promoter(<=1kb)	-24	SH3TC1	54436
502828	4q	112431241	112432293	1053	7	0.429	3'UTR	4735	ALPK1	80216
502828	4q	121036404	121037542	1139	6	0.333	Promoter(1-2kb)	1442	NDNF	79625
502828	4q	185458217	185460011	1795	9	0.556	Promoter(<=1kb)	0	CCDC110	256309
502828	5q	67163994	67165736	1743	6	0.5	Exon(exon29of29)	20561	CD180	4064
502828	5q	140807352	140807737	386	6	0.833	Promoter(<=1kb)	271	PCDHA4	56144
502828	5q	141174000	141175025	1026	6	0.833	Promoter(1-2kb)	1356	PCDHB7	56129
502828	5q	141955676	141957660	1985	6	0.667	Promoter(<=1kb)	-668	RNF14	9604
502828	6p	1312843	1313717	875	6	0.333	Promoter(<=1kb)	745	FOXQ1	94234
502828	6p	16327099	16327837	739	7	0.286	Exon(exon8of9)	37154	GMPR	2766
502828	6p	46858771	46859389	619	6	0.5	Exon(exon17of21)	3915	ADGRF5	221395
502828	6q	64591274	64591961	688	10	0.5	Exon(exon26of43)	121374	EYS	346007
502828	6q	159233455	159234370	916	10	0.5	Exon(exon11of23)	15158	FNDC1	84624
502828	7p	5313004	5313713	710	6	0.667	Exon(exon27of30)	6894	TNRC18	84629
502828	7p	45082725	45084866	2142	8	0.5	Promoter(1-2kb)	-1325	NACAD	23148
502828	7q	64991278	64992758	1481	6	0.667	Promoter(<=1kb)	-242	ZNF117	51351
502828	7q	100958977	100960873	1897	54	0.463	Promoter(1-2kb)	1012	MUC3A	4584
502828	7q	100991195	100993127	1933	8	0.625	Exon(exon5of15)	-19927	MUC12	10071
502828	7q	100995575	100995785	211	6	0.833	Exon(exon5of15)	-17269	MUC12	10071
502828	7q	101036085	101038841	2757	8	0.5	Exon(exon3of12)	-4870	MUC17	140453
502828	7q	149818015	149819792	1778	6	0.667	Promoter(2-3kb)	-2352	SSPO	23145
502828	7q	149824094	149826495	2402	6	0.667	Promoter(<=1kb)	0	SSPO	23145
502828	8p	8376561	8377198	638	6	1	Exon(exon2of5)	4549	PRAG1	157285
502828	8p	10607375	10608261	887	6	0.5	Exon(exon4of4)	46882	RP1L1	94137
502828	8p	10609697	10610843	1147	8	0.5	Exon(exon4of4)	44300	RP1L1	94137
502828	8p	12132686	12133940	1255	6	0.833	Promoter(<=1kb)	498	USP17L7	392197
502828	8p	12137448	12138641	1194	6	0.833	Promoter(<=1kb)	436	USP17L2	377630
502828	8p	13021128	13022030	903	7	0.143	Exon(exon5of5)	9115	TRMT9B	57604
502828	8p	17754377	17755366	990	6	0.333	Promoter(<=1kb)	0	MTUS1	57509
502828	8q	123651655	123652634	980	7	0.571	Promoter(<=1kb)	316	KLHL38	340359
502828	9p	21206764	21207074	311	6	0.5	Promoter(<=1kb)	69	IFNA10	3446
502828	9q	76705179	76707804	2626	8	0.5	Promoter(<=1kb)	121	PCA3	50652
502828	9q	76709263	76710843	1581	8	0.5	Promoter(<=1kb)	0	PRUNE2	158471
502828	9q	104598545	104599361	817	12	0.583	Promoter(<=1kb)	52	OR13C5	138799
502828	9q	122553263	122554071	809	8	0.5	Promoter(<=1kb)	93	OR1N2	138882
502828	9q	122749914	122750547	634	6	0.833	Promoter(<=1kb)	174	OR1L6	392390
502828	9q	131474844	131476113	1270	6	0.833	Promoter(<=1kb)	0	PRRC2B	84726
502828	9q	135484803	135487213	2411	9	0.222	Promoter(1-2kb)	1440	PPP1R26	9858
502828	10p	47663	48605	943	6	0.833	Promoter(<=1kb)	664	TUBB8	347688
502828	10p	30027143	30029020	1878	7	0.429	Exon(exon3of4)	30566	JCAD	57608
502828	10q	46549378	46550723	1346	25	0.64	Exon(exon3of3)	4807	GPRIN2	9721
502828	10q	49322575	49326817	4243	12	0.583	Exon(exon3of3)	23301	C10orf71	118461
502828	10q	89737450	89738561	1112	6	0	Exon(exon20of33)	13874	KIF20B	9585
502828	10q	122084988	122087840	2853	8	0.75	Exon(exon4of23)	-25190	TACC2	10579
502828	10q	128103129	128109280	6152	16	0.75	Promoter(<=1kb)	-1	MKI67	4288
502828	11p	244106	244197	92	8	0.5	Promoter(<=1kb)	-232	PSMD13	5719
502828	11p	1194354	1196902	2549	7	0.571	Exon(exon34of49)	-26164	MUC5B	727897
502828	11p	1246095	1247378	1284	10	0.5	Promoter(2-3kb)	2298	MUC5B-AS1	112577518
502828	11p	5177978	5178478	501	6	0.167	Promoter(<=1kb)	186	OR52Z1	283110
502828	11p	5323451	5324256	806	6	0.5	Promoter(<=1kb)	41	OR51B2	79345
502828	11p	5422212	5423123	912	11	0.636	Promoter(<=1kb)	101	OR51Q1	390061
502828	11p	5581045	5581738	694	8	0.375	Promoter(<=1kb)	168	OR52B6	340980
502828	11p	5841302	5841883	582	9	0.333	Promoter(<=1kb)	14	OR52E6	390078
502828	11p	11351961	11352736	776	9	0.222	Promoter(<=1kb)	514	CSNK2A3	283106
502828	11p	12293639	12294368	730	7	0.857	Exon(exon29of35)	6739	MICALCL	84953

502828	11p	18137446	18138122	677	6	0.333	Exon(exon3of3)	4834	MRGPRX3	117195
502828	11p	18173280	18173901	622	6	0.333	Promoter(<=1kb)	443	MRGPRX4	117196
502828	11q	58402523	58403265	743	9	0.444	Promoter(<=1kb)	144	OR5B3	441608
502828	11q	66560202	66562261	2060	6	0.333	Exon(exon14of21)	3873	CTSF	8722
502828	11q	85724687	85725825	1139	6	0.5	Promoter(<=1kb)	0	SYTL2	54843
502828	11q	124038366	124038988	623	7	1	Promoter(<=1kb)	13	OR10G7	390265
502828	11q	124382526	124383285	760	8	0.625	Promoter(<=1kb)	58	OR8B2	26595
502828	11q	130914501	130915580	1080	12	0.75	Promoter(<=1kb)	864	SNX19	399979
502828	12p	4626568	4628549	1982	11	0.455	Exon(exon5of6)	14051	DYRK4	8798
502828	12p	6453119	6453670	552	6	0.667	Promoter(<=1kb)	633	TAPBPL	55080
502828	13q	24434450	24435347	898	7	0.571	Exon(exon31of34)	19787	PARP4	143
502828	13q	25096870	25097182	313	8	0.375	Promoter(1-2kb)	1002	PABPC3	5042
502828	13q	102732474	102733933	1460	6	0.333	Exon(exon4of4)	25139	CCDC168	643677
502828	14q	20060048	20060884	837	8	0.625	Promoter(<=1kb)	3	OR4L1	122742
502828	14q	20223729	20224484	756	7	0.571	Promoter(<=1kb)	19	OR11H6	122748
502828	14q	21634137	21634589	453	9	0.556	Promoter(<=1kb)	351	OR10G2	26534
502828	14q	70457532	70458540	1009	11	0.273	Exon(exon2of2)	5358	ADAM21	8747
502828	14q	94587512	94587839	328	6	0.5	Exon(exon2of2)	-4219	SERPINA3	12
502828	14q	104939262	104950447	11186	39	0.513	5'UTR	4955	AHNAK2	113146
502828	15q	23439979	23442067	2089	14	0.5	5'UTR	5167	GOLGA6L2	283685
502828	15q	40621642	40623696	2055	6	0.333	Promoter(<=1kb)	0	KNL1	57082
502828	15q	73702465	73703760	1296	6	0.833	Promoter(<=1kb)	-149	CD276	80381
502828	15q	85579423	85581800	2378	15	0.6	Promoter(1-2kb)	-1110	AKAP13	11214
502828	15q	88854874	88855594	721	6	0.833	Exon(exon12of18)	7631	ACAN	176
502828	15q	88857108	88859365	2258	7	0.143	Exon(exon12of18)	9865	ACAN	176
502828	15q	99129423	99132517	3095	7	0.286	Exon(exon4of5)	7225	TTC23	64927
502828	16p	1486371	1488463	2093	8	0.75	Promoter(<=1kb)	4	PTX4	390667
502828	16p	27362551	27363079	529	6	0.167	3'UTR	7203	IL4R	3566
502828	16q	88428339	88429600	1262	6	0.333	Exon(exon3of3)	-23680	ZFPM1	161882
502828	16q	88714717	88717113	2397	7	0.571	Promoter(<=1kb)	0	MIR4722	100616167
502828	16q	89227206	89228419	1214	8	0.625	Promoter(2-3kb)	2572	ZNF778	197320
502828	17p	744946	746966	2021	6	1	3'UTR	5072	GEMIN4	50628
502828	17p	10638198	10641099	2902	7	0.286	Exon(exon19of41)	-8169	MYH3	4621
502828	17p	21300581	21300954	374	8	0.75	3'UTR	9112	MAP2K3	5606
502828	17q	76293419	76294016	598	6	0.5	Promoter(2-3kb)	-2167	QRICH2	84074
502828	17q	81510690	81511591	902	6	0.667	Promoter(<=1kb)	257	ACTG1	71
502828	17q	81645135	81645417	283	6	0.333	Promoter(2-3kb)	2722	TSPAN10	83882
502828	18p	11609728	11610491	764	13	0.692	Promoter(<=1kb)	132	SLC35G4	646000
502828	18p	14542649	14543140	492	6	0.5	Promoter(<=1kb)	6	POTEC	388468
502828	18q	58535186	58538030	2845	19	0.526	Promoter(<=1kb)	0	ALPK2	115701
502828	19p	4510548	4513547	3000	21	0.429	Exon(exon3of6)	4157	PLIN4	729359
502828	19p	5455600	5456439	840	8	0.625	Promoter(<=1kb)	183	ZNRF4	148066
502828	19p	8937644	8939234	1591	6	0.667	Exon(exon5of84)	-41554	MUC16	94025
502828	19p	8946313	8951868	5556	19	0.632	Exon(exon3of84)	29474	MUC16	94025
502828	19p	8959116	8962299	3184	11	0.727	Exon(exon3of84)	19043	MUC16	94025
502828	19p	9126210	9126946	737	9	0.556	Promoter(<=1kb)	4	OR7G3	390883
502828	19p	15087213	15088040	828	9	0.333	Promoter(<=1kb)	233	OR1I1	126370
502828	19p	17281820	17284246	2427	9	0.556	Promoter(<=1kb)	0	ANKLE1	126549
502828	19p	18264798	18267409	2612	13	0.615	5'UTR	7002	IQCN	80726
502828	19p	21971930	21974500	2571	7	0.714	Exon(exon4of4)	14408	ZNF208	7757
502828	19p	22756205	22759523	3319	14	0.571	3'UTR	10459	ZNF99	7652
502828	19q	39893459	39894014	556	6	0.667	Exon(exon12of28)	-6167	FCGBP	8857
502828	19q	40880231	40880622	392	7	0.429	Promoter(<=1kb)	-141	CYP2A7	1549
502828	19q	43203948	43205504	1557	7	0.714	Promoter(<=1kb)	0	PSG4	5672
502828	19q	43913423	43914878	1456	8	0.5	Exon(exon10of10)	4861	ZNF45	7596
502828	19q	44327836	44329698	1863	6	0.5	Exon(exon4of4)	-22790	ZNF235	9310
502828	19q	52437918	52439242	1325	7	0.429	Exon(exon4of4)	6504	ZNF534	147658
502828	19q	55911888	55913077	1190	6	0.667	Exon(exon5of12)	19234	NLRP13	126204
502828	19q	58416932	58417769	838	6	0.5	3'UTR	8155	ZNF584	201514
502828	20p	5922421	5923394	974	6	0.5	Exon(exon4of5)	6923	CHGB	1114
502828	20q	62812284	62813345	1062	7	0.857	Promoter(2-3kb)	-2899	COL9A3	1299
502828	20q	63561666	63565531	3866	11	0.636	Promoter(<=1kb)	-61	HELZ2	85441
502828	21q	26843740	26844859	1120	6	0.667	Promoter(<=1kb)	0	ADAMTS1	9510
502828	22q	17119403	17120750	1348	6	0.833	Promoter(<=1kb)	610	TMEM121B	27439
502828	22q	22352950	22353380	431	16	0.5	Exon(exon1of2)	30478	BMS1P20	96610
502828	22q	36191154	36191906	753	6	0.667	3'UTR	9971	APOL4	80832
502828	22q	39100331	39102033	1703	6	0.833	Promoter(<=1kb)	52	APOBEC3H	164668
502828	23p	8170039	8170141	103	6	0.5	Promoter(1-2kb)	1126	VCX2	51480
502828	23p	35802148	35803010	863	7	0.571	5'UTR	3357	MAGEB16	139604
502828	23q	102937373	102937765	393	7	0.571	Promoter(<=1kb)	101	RAB40AL	282808
503050	1p	11766028	11768307	2280	6	0.833	Promoter(<=1kb)	0	C1orf167	284498
503050	1p	11778784	11779941	1158	7	0.714	Promoter(<=1kb)	0	C1orf167-AS1	102724659
503050	1p	16048038	16049824	1787	6	0.5	Promoter(<=1kb)	0	CLCNKB	1188
503050	1p	16058491	16060000	1510	10	0.9	Exon(exon5of7)	6168	CLCNKB	1188
503050	1p	18481403	18482217	815	7	0.714	Promoter(<=1kb)	421	KLHDC7A	127707
503050	1p	23874604	23875430	827	8	0.5	Exon(exon2of2)	-6310	FUCA1	2517
503050	1p	40067594	40067675	82	6	0	Promoter(<=1kb)	324	CAP1	10487

503050	1p	89186388	89186419	32	9	0.556	Promoter(<=1kb)	107	GBP4	115361
503050	1q	152219233	152221375	2143	15	0.733	Promoter(2-3kb)	2818	HRNR	388697
503050	1q	156669844	156670886	1043	7	1	Exon(exon4of4)	6521	NES	10763
503050	1q	158765805	158766655	851	6	0.5	Promoter(<=1kb)	47	OR6N1	128372
503050	1q	197101312	197101771	460	6	0.5	Exon(exon18of28)	33373	ASPM	259266
503050	1q	201206099	201209342	3244	9	0.667	Promoter(1-2kb)	1017	IGFN1	91156
503050	1q	226735563	226737239	1677	8	0.875	Promoter(<=1kb)	219	ITPKB	3707
503050	1q	228315976	228318026	2051	6	0.5	Exon(exon50of81)	6492	OBSCN	84033
503050	1q	247841312	247841582	271	6	0.833	Promoter(<=1kb)	314	OR11L1	391189
503050	1q	247895950	247896410	461	7	0.571	Promoter(<=1kb)	363	OR2W3	343171
503050	1q	248294677	248295415	739	6	0.667	Promoter(<=1kb)	185	OR2T12	127064
503050	2p	48580657	48582454	1798	7	0.571	Promoter(<=1kb)	0	STON1	11037
503050	2q	102351547	102351902	356	7	0.429	Exon(exon11of11)	-4027	IL18R1	8809
503050	2q	132783061	132784972	1912	7	0.429	Promoter(1-2kb)	-1038	NCKAP5	344148
503050	2q	178739433	178741811	2379	6	0.5	Exon(exon45of191)	26014	TTN	7273
503050	2q	184936178	184937636	1459	7	0.429	Exon(exon4of4)	69813	ZNF804A	91752
503050	2q	185789865	185794632	4768	10	0.8	Promoter(<=1kb)	0	FSIP2	401024
503050	2q	185805377	185808170	2794	7	0.429	Promoter(<=1kb)	0	FSIP2	401024
503050	2q	233713134	233713664	531	8	0.75	Promoter(<=1kb)	142	UGT1A5	54579
503050	2q	237762685	237764060	1376	10	0.5	Exon(exon8of8)	-4137	LRRFIP1	9208
503050	2q	240041845	240042811	967	7	0.286	Downstream(2-3kb)	3261	OR6B3	150681
503050	3p	75736929	75738859	1931	11	0.545	Promoter(<=1kb)	0	MIR4273	100422955
503050	3q	98264413	98265098	686	7	0.571	Promoter(<=1kb)	128	OR5H6	79295
503050	3q	194359607	194360906	1300	9	0.889	Exon(exon2of2)	-8279	CPN2	1370
503050	4p	6300792	6302360	1569	8	0.75	Exon(exon8of8)	6021	WFS1	7466
503050	4p	8227004	8228508	1505	8	0.125	Promoter(<=1kb)	-24	SH3TC1	54436
503050	4p	10443803	10446224	2422	6	0.333	Exon(exon3of3)	10952	ZNF518B	85460
503050	4p	38774486	38775552	1067	8	0.5	Promoter(<=1kb)	644	TLR10	81793
503050	4p	38796894	38798702	1809	6	0.5	Exon(exon4of4)	5674	TLR1	7096
503050	4q	154489498	154491312	1815	9	0.556	Promoter(<=1kb)	22	DCHS2	54798
503050	5q	83537326	83539905	2580	6	0.333	Promoter(1-2kb)	1712	VCAN	1462
503050	5q	140848579	140850786	2208	6	0.5	Promoter(<=1kb)	807	PCDHA9	9752
503050	5q	141174000	141175025	1026	7	0.714	Promoter(1-2kb)	1356	PCDHB7	56129
503050	5q	141955356	141957660	2305	6	0.667	Promoter(<=1kb)	-668	RNF14	9604
503050	6p	46858771	46859502	732	8	0.5	Exon(exon17of21)	3802	ADGRF5	221395
503050	6q	149888581	149890867	2287	7	0.714	Promoter(<=1kb)	0	RAET1E-AS1	100652739
503050	6q	159231899	159234370	2472	12	0.583	Exon(exon11of23)	13602	FNDC1	84624
503050	7p	31642839	31643796	958	6	0.5	Exon(exon12of15)	-8186	ITPRID1	223075
503050	7q	100958977	100960873	1897	47	0.489	Promoter(1-2kb)	1012	MUC3A	4584
503050	7q	100993912	100995785	1874	10	0.7	Exon(exon5of15)	-17269	MUC12	10071
503050	7q	101034361	101040583	6223	38	0.553	Exon(exon3of12)	-3128	MUC17	140453
503050	8p	8376561	8377220	660	6	1	Exon(exon2of5)	4527	PRAG1	157285
503050	8p	10607375	10608261	887	7	0.429	Exon(exon4of4)	46882	RP1L1	94137
503050	8p	11331194	11332082	889	9	0.667	Promoter(<=1kb)	306	SLC35G5	83650
503050	8p	12132477	12133940	1464	10	0.7	Promoter(<=1kb)	498	USP17L7	392197
503050	8p	12137448	12138641	1194	7	0.857	Promoter(<=1kb)	436	USP17L2	377630
503050	8p	13021128	13022030	903	7	0.143	Exon(exon5of5)	9115	TRMT9B	57604
503050	8q	141218050	141219792	1743	6	0.667	5'UTR	8778	SLC45A4	57210
503050	9p	116800	117713	914	8	0.75	Promoter(<=1kb)	491	FOXD4	2298
503050	9p	39078723	39078846	124	6	0.667	Exon(exon22of24)	7302	CNTNAP3	79937
503050	9q	76175237	76175296	60	10	0.8	Exon(exon14of14)	-13343	PCSK5	5125
503050	9q	76705179	76707804	2626	8	0.5	Promoter(<=1kb)	121	PCA3	50652
503050	9q	76709263	76710843	1581	8	0.5	Promoter(<=1kb)	0	PRUNE2	158471
503050	9q	87886533	87888536	2004	7	0.571	Exon(exon4of4)	3656	SPATA31E1	286234
503050	9q	104598545	104599318	774	6	0.333	Promoter(<=1kb)	95	OR13C5	138799
503050	9q	122553263	122554071	809	8	0.5	Promoter(<=1kb)	93	OR1N2	138882
503050	9q	122628595	122629130	536	6	0.333	Promoter(<=1kb)	443	OR1B1	347169
503050	9q	122749914	122750547	634	6	0.833	Promoter(<=1kb)	174	OR1L6	392390
503050	9q	133255635	133256264	630	8	1	3'UTR	18950	ABO	28
503050	9q	135484803	135487573	2771	8	0.25	Promoter(1-2kb)	1440	PPP1R26	9858
503050	10q	46549378	46550723	1346	25	0.64	Exon(exon3of3)	4807	GPRIN2	9721
503050	10q	103602059	103602687	629	6	0.667	Exon(exon15of15)	30382	NEURL1	9148
503050	10q	128103129	128104830	1702	10	0.6	Promoter(<=1kb)	-1	MKI67	4288
503050	11p	244106	244197	92	8	0.5	Promoter(<=1kb)	-232	PSMD13	5719
503050	11p	1241677	1243593	1917	8	0.375	Exon(exon31of49)	6083	MUC5B-AS1	112577518
503050	11p	1246095	1251628	5534	20	0.7	Promoter(<=1kb)	0	MUC5B-AS1	112577518
503050	11p	5177978	5178478	501	6	0.167	Promoter(<=1kb)	186	OR52Z1	283110
503050	11p	5323542	5324256	715	6	0.5	Promoter(<=1kb)	41	OR51B2	79345
503050	11p	5351521	5352416	896	16	0.438	Promoter(<=1kb)	13	OR51B6	390058
503050	11p	5389704	5390350	647	7	0.429	Promoter(<=1kb)	327	OR51M1	390059
503050	11p	5422212	5423123	912	10	0.7	Promoter(<=1kb)	101	OR51Q1	390061
503050	11p	5581045	5581738	694	8	0.375	Promoter(<=1kb)	168	OR52B6	340980
503050	11p	5841302	5841883	582	9	0.333	Promoter(<=1kb)	14	OR52E6	390078
503050	11p	11351961	11352736	776	9	0.222	Promoter(<=1kb)	514	CSNK2A3	283106
503050	11p	12293639	12294538	900	8	0.625	Exon(exon29of35)	6739	MICALCL	84953
503050	11q	58214757	58215722	966	8	0.25	Promoter(<=1kb)	12	OR1S1	219959
503050	11q	85724687	85725825	1139	6	0.5	Promoter(<=1kb)	0	SYTL2	54843

503050	11q	123906790	123907324	535	6	0.667	Promoter(<=1kb)	644	OR8D4	338662
503050	11q	124015601	124016477	877	8	0.25	Promoter(<=1kb)	26	OR10G4	390264
503050	11q	124023038	124023849	812	9	0.444	Promoter(<=1kb)	25	OR10G9	219870
503050	11q	124038366	124038988	623	9	0.889	Promoter(<=1kb)	13	OR10G7	390265
503050	11q	124382526	124383285	760	9	0.667	Promoter(<=1kb)	58	OR8B2	26595
503050	11q	130914501	130915409	909	10	0.7	Promoter(1-2kb)	1035	SNX19	399979
503050	12p	4626568	4628549	1982	11	0.455	Exon(exon5of6)	14051	DYRK4	8798
503050	12p	6453119	6453670	552	6	0.667	Promoter(<=1kb)	633	TAPBPL	55080
503050	13q	102732474	102733933	1460	6	0.333	Exon(exon4of4)	25139	CCDC168	643677
503050	14q	19975713	19976448	736	10	0.5	Promoter(<=1kb)	269	OR4K15	81127
503050	14q	20060048	20060884	837	8	0.625	Promoter(<=1kb)	3	OR4L1	122742
503050	14q	70457532	70458540	1009	9	0.333	Exon(exon2of2)	5358	ADAM21	8747
503050	14q	104175455	104177762	2308	8	0.375	Exon(exon12of15)	36415	KIF26A	26153
503050	14q	104939262	104942618	3357	13	0.231	5'UTR	7102	PLD4	122618
503050	14q	104943622	104953878	10257	49	0.551	Promoter(1-2kb)	1524	AHNAK2	113146
503050	15q	23440160	23442067	1908	10	0.6	5'UTR	5167	GOLGA6L2	283685
503050	16p	1486371	1488463	2093	8	0.75	Promoter(<=1kb)	4	PTX4	390667
503050	16q	74391650	74391928	279	7	0.714	Exon(exon7of7)	13772	NPIPB15	440348
503050	16q	88428539	88431889	3351	11	0.455	Exon(exon3of3)	-21391	ZFPM1	161882
503050	16q	88433131	88436684	3554	7	0.714	Exon(exon3of3)	-16596	ZFPM1	161882
503050	16q	89226863	89228289	1427	7	0.571	Promoter(2-3kb)	2229	ZNF778	197320
503050	17p	744946	746966	2021	6	1	3'UTR	5072	GEMIN4	50628
503050	17p	21300581	21300954	374	8	0.75	3'UTR	9112	MAP2K3	5606
503050	17q	41727098	41728331	1234	6	0.833	Promoter(1-2kb)	-1201	HAP1	9001
503050	17q	81645135	81645595	461	7	0.429	Promoter(2-3kb)	2722	TSPAN10	83882
503050	18p	11609728	11610491	764	10	0.8	Promoter(<=1kb)	132	SLC35G4	646000
503050	18q	58535186	58537515	2330	9	0.333	Promoter(<=1kb)	0	ALPK2	115701
503050	19p	4510548	4513547	3000	21	0.476	Exon(exon3of6)	4157	PLIN4	729359
503050	19p	5455600	5456439	840	7	0.571	Promoter(<=1kb)	183	ZNRF4	148066
503050	19p	8333830	8334965	1136	7	0.714	Promoter(<=1kb)	0	KANK3	256949
503050	19p	8948231	8953259	5029	16	0.562	Exon(exon3of84)	28083	MUC16	94025
503050	19p	8959403	8962066	2664	8	0.5	Exon(exon3of84)	19276	MUC16	94025
503050	19p	8964274	8967127	2854	22	0.682	Exon(exon3of84)	14215	MUC16	94025
503050	19p	15087389	15087953	565	6	0.333	Promoter(<=1kb)	409	OR1I1	126370
503050	19p	18264798	18267409	2612	12	0.583	5'UTR	7002	IQCN	80726
503050	19p	21971930	21974500	2571	6	0.667	Exon(exon4of4)	14408	ZNF208	7757
503050	19q	36996730	36997597	868	10	0.7	Exon(exon10of10)	5677	ZNF568	374900
503050	19q	37151928	37153149	1222	6	0.833	Exon(exon5of5)	19287	ZNF585A	199704
503050	19q	37885190	37888806	3617	9	0.667	Exon(exon6of6)	17788	WDR87	83889
503050	19q	39877222	39877880	659	6	0.5	Exon(exon20of28)	9412	FCGBP	8857
503050	19q	39886243	39886422	180	6	0.833	Promoter(<=1kb)	870	FCGBP	8857
503050	19q	40880128	40880622	495	7	0.286	Promoter(<=1kb)	-38	CYP2A7	1549
503050	19q	43204774	43205504	731	6	0.833	Promoter(<=1kb)	47	PSG4	5672
503050	19q	43846955	43848536	1582	6	0.833	3'UTR	13450	ZNF283	284349
503050	19q	43913423	43914878	1456	9	0.444	Exon(exon10of10)	4861	ZNF45	7596
503050	19q	44106512	44108078	1567	7	0	Exon(exon6of6)	-4103	ZNF225	7768
503050	19q	51745958	51746963	1006	6	0.5	Exon(exon3of3)	3848	FPR1	2357
503050	19q	52365769	52366744	976	6	1	Exon(exon6of6)	-3173	ZNF880	400713
503050	19q	52384029	52384992	964	6	0	Exon(exon4of4)	12677	ZNF528-AS1	102724105
503050	19q	52437918	52439242	1325	7	0.429	Exon(exon4of4)	6504	ZNF534	147658
503050	19q	53164551	53166239	1689	6	0.167	Exon(exon4of4)	-5476	ZNF347	84671
503050	19q	55358736	55359651	916	6	0.5	Promoter(<=1kb)	0	FAM71E2	284418
503050	19q	55481625	55483456	1832	7	0.571	Promoter(1-2kb)	-1732	NAT14	57106
503050	19q	55911888	55913077	1190	6	0.667	Exon(exon5of12)	19234	NLRP13	126204
503050	19q	56664065	56665114	1050	6	0.5	Exon(exon2of2)	6643	ZNF835	90485
503050	19q	58368293	58368875	583	7	0.429	Exon(exon3of3)	-5445	ZNF497	162968
503050	20p	5922421	5923394	974	6	0.5	Exon(exon4of5)	6923	CHGB	1114
503050	20q	53575869	53577159	1291	6	0.833	Promoter(<=1kb)	0	ZNF217	7764
503050	20q	63559968	63565531	5564	17	0.647	Promoter(<=1kb)	0	HELZ2	85441
503050	21q	26843740	26844859	1120	6	0.667	Promoter(<=1kb)	0	ADAMTS1	9510
503050	22q	22352950	22353380	431	16	0.5	Exon(exon1of2)	30478	BMS1P20	96610
503050	22q	36191154	36191906	753	6	0.667	3'UTR	9971	APOL4	80832
503050	23p	8170039	8170141	103	6	0.5	Promoter(1-2kb)	1126	VCX2	51480
504198	1p	16058491	16060000	1510	13	0.846	Exon(exon5of7)	6168	CLCNKB	1188
504198	1p	23874604	23875430	827	8	0.5	Exon(exon2of2)	-6310	FUCA1	2517
504198	1p	40067594	40067675	82	6	0	Promoter(<=1kb)	324	CAP1	10487
504198	1q	152218469	152221375	2907	14	0.643	Promoter(2-3kb)	2818	HRNR	388697
504198	1q	152303673	152313891	10219	37	0.622	Promoter(<=1kb)	0	FLG-AS1	339400
504198	1q	156669844	156670886	1043	6	1	Exon(exon4of4)	6521	NES	10763
504198	1q	201211017	201212095	1079	7	0.429	Promoter(<=1kb)	-547	IGFN1	91156
504198	1q	228315976	228318038	2063	8	0.625	Exon(exon50of81)	6492	OBSCN	84033
504198	1q	232805117	232806800	1684	7	0.429	Promoter(<=1kb)	225	MAP10	54627
504198	1q	247841312	247841582	271	6	0.833	Promoter(<=1kb)	314	OR11L1	391189
504198	1q	247895625	247896502	878	7	0.571	Promoter(<=1kb)	38	OR2W3	343171
504198	1q	247949325	247949759	435	12	0.417	Promoter(<=1kb)	467	OR2L8	391190
504198	1q	248294677	248295458	782	7	0.571	Promoter(<=1kb)	142	OR2T12	127064
504198	1q	248681658	248682198	541	7	0.571	Promoter(<=1kb)	130	OR14I1	401994

504198	2p	29071763	29073000	1238	6	0.667	Promoter(1-2kb)	1523	PCARE	388939
504198	2p	48580657	48582454	1798	7	0.571	Promoter(<=1kb)	0	STON1	11037
504198	2q	184936178	184937636	1459	7	0.429	Exon(exon4of4)	69813	ZNF804A	91752
504198	2q	185789591	185794632	5042	11	0.727	Promoter(<=1kb)	0	FSIP2	401024
504198	2q	217847583	217848559	977	6	0.833	Exon(exon19of33)	-5423	TNS1	7145
504198	2q	238130416	238131546	1131	6	0.333	Promoter(1-2kb)	1468	ESPNL	339768
504198	2q	240041845	240042811	967	7	0.286	Downstream(2-3kb)	3261	OR6B3	150681
504198	3p	31989532	31990905	1374	7	0.429	Exon(exon2of2)	7761	ZNF860	344787
504198	3p	75736929	75739007	2079	14	0.643	Promoter(<=1kb)	0	MIR4273	100422955
504198	3q	98169021	98169594	574	6	0.667	Exon(exon2of2)	19695	OR5H14	403273
504198	3q	98264413	98265098	686	7	0.571	Promoter(<=1kb)	128	OR5H6	79295
504198	4p	1394625	1395368	744	7	0.429	Exon(exon1of1)	9813	UVSSA	57654
504198	4p	5988383	5989749	1367	7	0.571	Promoter(<=1kb)	0	C4orf50	389197
504198	4p	6300792	6302360	1569	6	0.833	Exon(exon8of8)	6021	WFS1	7466
504198	4p	8227004	8228508	1505	8	0.25	Promoter(<=1kb)	-24	SH3TC1	54436
504198	4p	10443803	10446224	2422	7	0.429	Exon(exon3of3)	10952	ZNF518B	85460
504198	4p	38827472	38829115	1644	6	0.5	Exon(exon2of2)	-21894	TLR1	7096
504198	4q	185458217	185460011	1795	8	0.625	Promoter(<=1kb)	0	CCDC110	256309
504198	4q	186706638	186708616	1979	6	0.667	Exon(exon2of27)	15217	FAT1	2195
504198	5q	79728956	79730716	1761	7	0.571	Exon(exon2of13)	-7426	CMYA5	202333
504198	5q	79731782	79734523	2742	13	0.308	Exon(exon2of13)	-3619	CMYA5	202333
504198	5q	83537326	83539905	2580	6	0.333	Promoter(1-2kb)	1712	VCAN	1462
504198	5q	140848579	140850786	2208	7	0.571	Promoter(<=1kb)	807	PCDHA9	9752
504198	5q	141955676	141957660	1985	6	0.667	Promoter(<=1kb)	-668	RNF14	9604
504198	5q	155014517	155017304	2788	9	0.778	Promoter(<=1kb)	762	KIF4B	285643
504198	6p	46858771	46859389	619	6	0.5	Exon(exon17of21)	3915	ADGRF5	221395
504198	7q	64991321	64992758	1438	6	0.667	Promoter(<=1kb)	-285	ZNF117	51351
504198	7q	100958721	100960873	2153	56	0.464	Promoter(<=1kb)	756	MUC3A	4584
504198	7q	100991195	100993127	1933	9	0.667	Exon(exon5of15)	-19927	MUC12	10071
504198	7q	100995575	100995785	211	6	0.833	Exon(exon5of15)	-17269	MUC12	10071
504198	8p	11331234	11332082	849	6	0.667	Promoter(<=1kb)	346	SLC35G5	83650
504198	8p	13021128	13022030	903	7	0.143	Exon(exon5of5)	9115	TRMT9B	57604
504198	8q	138151949	138153046	1098	6	0.667	Promoter(<=1kb)	0	FAM135B	51059
504198	8q	143916360	143919209	2850	7	0.143	Exon(exon32of32)	20381	PLEC	5339
504198	8q	143921326	143924022	2697	9	0.667	Exon(exon31of32)	15568	PLEC	5339
504198	9q	76175241	76175301	61	9	0.778	Exon(exon14of14)	-13338	PCSK5	5125
504198	9q	76703555	76706360	2806	8	0.5	Promoter(<=1kb)	0	PCA3	50652
504198	9q	104504315	104505071	757	6	0.5	Promoter(<=1kb)	52	OR13F1	138805
504198	9q	104598545	104599361	817	12	0.583	Promoter(<=1kb)	52	OR13C5	138799
504198	9q	122553263	122554071	809	7	0.429	Promoter(<=1kb)	93	OR1N2	138882
504198	9q	122567771	122568460	690	6	0.667	Promoter(<=1kb)	99	OR1L8	138881
504198	9q	122628595	122629130	536	6	0.333	Promoter(<=1kb)	443	OR1B1	347169
504198	9q	122749914	122750547	634	6	0.833	Promoter(<=1kb)	174	OR1L6	392390
504198	9q	135484803	135487213	2411	8	0.25	Promoter(1-2kb)	1440	PPP1R26	9858
504198	10q	46549378	46550723	1346	25	0.64	Exon(exon3of3)	4807	GPRIN2	9721
504198	10q	49323504	49325572	2069	7	0.429	Exon(exon3of3)	24230	C10orf71	118461
504198	10q	89737450	89738561	1112	6	0	Exon(exon20of33)	13874	KIF20B	9585
504198	10q	122084988	122087840	2853	9	0.778	Exon(exon4of23)	-25190	TACC2	10579
504198	11p	244106	244197	92	8	0.5	Promoter(<=1kb)	-232	PSMD13	5719
504198	11p	1194354	1196902	2549	7	0.571	Exon(exon34of49)	-26164	MUC5B	727897
504198	11p	1246095	1247378	1284	7	0.286	Promoter(2-3kb)	2298	MUC5B-AS1	112577518
504198	11p	5177978	5178478	501	6	0.167	Promoter(<=1kb)	186	OR52Z1	283110
504198	11p	5323451	5324256	806	6	0.5	Promoter(<=1kb)	41	OR51B2	79345
504198	11p	5422212	5423123	912	12	0.667	Promoter(<=1kb)	101	OR51Q1	390061
504198	11p	5581045	5581738	694	8	0.375	Promoter(<=1kb)	168	OR52B6	340980
504198	11p	11351961	11352736	776	9	0.222	Promoter(<=1kb)	514	CSNK2A3	283106
504198	11q	58214757	58215722	966	8	0.25	Promoter(<=1kb)	12	OR1S1	219959
504198	11q	82732630	82733184	555	6	0.833	Promoter(<=1kb)	680	FAM181B	220382
504198	11q	85724687	85725825	1139	6	0.5	Promoter(<=1kb)	0	SYTL2	54843
504198	11q	123906790	123907324	535	6	0.667	Promoter(<=1kb)	644	OR8D4	338662
504198	11q	123943088	123943782	695	7	0.571	Promoter(<=1kb)	91	OR6T1	219874
504198	11q	124015601	124016477	877	8	0.125	Promoter(<=1kb)	26	OR10G4	390264
504198	11q	124023038	124023849	812	9	0.444	Promoter(<=1kb)	25	OR10G9	219870
504198	11q	124038366	124038988	623	6	0.833	Promoter(<=1kb)	13	OR10G7	390265
504198	12p	4626568	4628549	1982	10	0.5	Exon(exon5of6)	14051	DYRK4	8798
504198	12p	8222174	8223514	1341	6	0.667	Exon(exon5of6)	4073	FAM90A1	55138
504198	12q	111598955	111599196	242	6	0.5	Promoter(<=1kb)	0	ATXN2	6311
504198	13q	25096877	25097182	306	7	0.429	Promoter(1-2kb)	1009	PABPC3	5042
504198	13q	102732474	102733933	1460	6	0.333	Exon(exon4of4)	25139	CCDC168	643677
504198	14q	19935750	19936577	828	6	0.667	Promoter(<=1kb)	142	OR4K1	79544
504198	14q	19975713	19976448	736	9	0.444	Promoter(<=1kb)	269	OR4K15	81127
504198	14q	20060048	20060884	837	8	0.625	Promoter(<=1kb)	3	OR4L1	122742
504198	14q	21634137	21634589	453	9	0.556	Promoter(<=1kb)	351	OR10G2	26534
504198	14q	70457520	70458540	1021	13	0.385	Exon(exon2of2)	5346	ADAM21	8747
504198	14q	94587512	94587839	328	6	0.5	Exon(exon2of2)	-4219	SERPINA3	12
504198	14q	104175275	104177810	2536	6	0.333	Exon(exon12of15)	36235	KIF26A	26153
504198	14q	104883051	104884531	1481	6	0.5	Exon(exon8of19)	-9403	CEP170B	283638

504198	14q	104947943	104951938	3996	12	0.583	Exon(exon6of6)	3464	AHNAK2	113146
504198	15q	23440160	23442067	1908	12	0.583	5'UTR	5167	GOLGA6L2	283685
504198	15q	40621642	40624434	2793	8	0.375	Promoter(<=1kb)	0	KNL1	57082
504198	15q	85579423	85581800	2378	14	0.571	Promoter(1-2kb)	-1110	AKAP13	11214
504198	15q	99129423	99132517	3095	6	0.333	Exon(exon4of5)	7225	TTC23	64927
504198	16p	789084	790597	1514	6	0.167	Promoter(<=1kb)	0	CHTF18	63922
504198	16p	1228744	1229731	988	10	0.7	Promoter(<=1kb)	431	TPSB2	64499
504198	16p	1256345	1256980	636	7	0.857	Promoter(<=1kb)	276	TPSD1	23430
504198	16p	1486371	1488463	2093	8	0.75	Promoter(<=1kb)	4	PTX4	390667
504198	16q	88428539	88431889	3351	11	0.364	Exon(exon3of3)	-21391	ZFPM1	161882
504198	16q	89100686	89101050	365	7	0.571	Promoter(<=1kb)	24	ACSF3	197322
504198	16q	89226863	89228289	1427	7	0.571	Promoter(2-3kb)	2229	ZNF778	197320
504198	17p	744946	747069	2124	7	1	3'UTR	4969	GEMIN4	50628
504198	17p	21300581	21300978	398	12	0.75	3'UTR	9112	MAP2K3	5606
504198	17q	76293419	76294016	598	6	0.5	Promoter(2-3kb)	-2167	QRICH2	84074
504198	17q	81645135	81645417	283	6	0.333	Promoter(2-3kb)	2722	TSPAN10	83882
504198	18p	11609904	11610469	566	7	0.571	Promoter(<=1kb)	308	SLC35G4	646000
504198	18q	58535186	58537515	2330	10	0.4	Promoter(<=1kb)	0	ALPK2	115701
504198	19p	4510548	4513547	3000	22	0.455	Exon(exon3of6)	4157	PLIN4	729359
504198	19p	7899063	7900784	1722	6	0.667	Exon(exon4of4)	-3059	MAP2K7	5609
504198	19p	8948231	8952171	3941	12	0.5	Exon(exon3of84)	29171	MUC16	94025
504198	19p	8959403	8961248	1846	6	0.667	Exon(exon3of84)	20094	MUC16	94025
504198	19p	12430400	12431840	1441	8	0.5	3'UTR	9181	ZNF443	10224
504198	19p	15087452	15087953	502	7	0.143	Promoter(<=1kb)	472	OR1I1	126370
504198	19p	17281820	17284246	2427	9	0.556	Promoter(<=1kb)	0	ANKLE1	126549
504198	19p	18264798	18267409	2612	12	0.583	5'UTR	7002	IQCN	80726
504198	19p	21971930	21974500	2571	8	0.75	Exon(exon4of4)	14408	ZNF208	7757
504198	19p	22757857	22759533	1677	8	0.5	Exon(exon4of4)	10449	ZNF99	7652
504198	19p	23743906	23745300	1395	6	0.333	Exon(exon4of4)	13537	ZNF681	148213
504198	19q	36996730	36997597	868	10	0.6	Exon(exon10of10)	5677	ZNF568	374900
504198	19q	37885026	37888806	3781	7	0.571	Exon(exon6of6)	17788	WDR87	83889
504198	19q	39886005	39886422	418	6	0.667	Promoter(<=1kb)	870	FCGBP	8857
504198	19q	40880128	40880622	495	7	0.286	Promoter(<=1kb)	-38	CYP2A7	1549
504198	19q	43612804	43614201	1398	6	0.5	Promoter(<=1kb)	716	SRRM5	100170229
504198	19q	43913423	43914878	1456	8	0.5	Exon(exon10of10)	4861	ZNF45	7596
504198	19q	55911888	55913077	1190	6	0.667	Exon(exon5of12)	19234	NLRP13	126204
504198	19q	58368293	58368875	583	7	0.429	Exon(exon3of3)	-5445	ZNF497	162968
504198	20p	5922577	5923643	1067	8	0.625	Exon(exon4of5)	7079	CHGB	1114
504198	20q	53575869	53577159	1291	6	0.833	Promoter(<=1kb)	0	ZNF217	7764
504198	20q	63349752	63350772	1021	6	0.5	3'UTR	3794	CHRNA4	1137
504198	20q	63561666	63565531	3866	14	0.643	Promoter(<=1kb)	-61	HELZ2	85441
504198	21q	44637474	44638143	670	10	0.4	Promoter(<=1kb)	118	KRTAP10-10	353333
504198	22q	22352950	22353380	431	16	0.5	Exon(exon1of2)	30478	BMS1P20	96610
504198	22q	36191154	36191906	753	7	0.714	3'UTR	9971	APOL4	80832
504198	22q	49884187	49884994	808	6	0.333	Exon(exon2of2)	22924	ALG12	79087
504198	23p	3320126	3323750	3625	10	0.6	Exon(exon5of7)	22902	MXRA5	25878
504198	23p	8170039	8170141	103	6	0.5	Promoter(1-2kb)	1126	VCX2	51480
504198	23p	35802148	35803010	863	7	0.571	5'UTR	3357	MAGEB16	139604
506263	1p	12847526	12847780	255	8	0.375	Promoter(<=1kb)	945	HNRNPCL1	343069
506263	1p	12859036	12860079	1044	6	0.333	Promoter(1-2kb)	1950	PRAMEF2	65122
506263	1p	13369166	13369564	399	7	0.714	Promoter(2-3kb)	2336	PRAMEF19	645414
506263	1p	13370686	13370957	272	7	0.429	Promoter(<=1kb)	943	PRAMEF19	645414
506263	1p	16058491	16060000	1510	10	0.9	Exon(exon5of7)	6168	CLCNKB	1188
506263	1p	40067594	40067675	82	6	0	Promoter(<=1kb)	324	CAP1	10487
506263	1q	152303673	152313891	10219	39	0.59	Promoter(<=1kb)	0	FLG-AS1	339400
506263	1q	197101312	197101771	460	6	0.5	Exon(exon18of28)	33373	ASPM	259266
506263	1q	228315976	228318049	2074	8	0.5	Exon(exon50of81)	6492	OBSCN	84033
506263	1q	228371172	228372999	1828	8	0.375	Promoter(<=1kb)	0	OBSCN	84033
506263	1q	247841312	247841582	271	6	0.833	Promoter(<=1kb)	314	OR11L1	391189
506263	2p	48580657	48582454	1798	7	0.571	Promoter(<=1kb)	0	STON1	11037
506263	2q	132783061	132784972	1912	7	0.429	Promoter(1-2kb)	-1038	NCKAP5	344148
506263	2q	167243228	167245060	1833	6	0.333	Exon(exon9of11)	-3622	XIRP2	129446
506263	2q	185789865	185794632	4768	10	0.8	Promoter(<=1kb)	0	FSIP2	401024
506263	2q	185805377	185808170	2794	6	0.333	Promoter(<=1kb)	0	FSIP2	401024
506263	2q	233840612	233842185	1574	7	0.714	Promoter(<=1kb)	0	HJURP	55355
506263	2q	238130416	238131546	1131	6	0.333	Promoter(1-2kb)	1468	ESPNL	339768
506263	2q	240041845	240042131	287	6	0.167	Downstream(2-3kb)	3941	OR6B3	150681
506263	3p	31989532	31990905	1374	7	0.286	Exon(exon2of2)	7761	ZNF860	344787
506263	3p	75737230	75739007	1778	9	0.556	Promoter(<=1kb)	0	MIR4273	100422955
506263	3q	194341097	194342571	1475	7	0.714	Exon(exon2of2)	8747	CPN2	1370
506263	4p	5988383	5989749	1367	7	0.571	Promoter(<=1kb)	0	C4orf50	389197
506263	4p	6300792	6302360	1569	6	0.833	Exon(exon8of8)	6021	WFS1	7466
506263	4q	186619481	186621601	2121	7	0.286	Exon(exon10of27)	-9411	FAT1	2195
506263	5q	83537326	83539905	2580	6	0.333	Promoter(1-2kb)	1712	VCAN	1462
506263	5q	140807352	140807737	386	6	0.833	Promoter(<=1kb)	271	PCDHA4	56144
506263	5q	140848579	140850786	2208	6	0.5	Promoter(<=1kb)	807	PCDHA9	9752
506263	5q	141174000	141175025	1026	6	0.833	Promoter(1-2kb)	1356	PCDHB7	56129

506263	5q	148826877	148828070	1194	6	1	Promoter(1-2kb)	1632	ADRB2	154
506263	6p	26370344	26370479	136	6	0.5	Promoter(<=1kb)	0	BTN3A2	11118
506263	6p	39314105	39316408	2304	6	0.667	Promoter(<=1kb)	0	KCNK17	89822
506263	6p	46858771	46859502	732	7	0.571	Exon(exon17of21)	3802	ADGRF5	221395
506263	6q	149888581	149890867	2287	7	0.714	Promoter(<=1kb)	0	RAET1E-AS1	100652739
506263	6q	159231899	159234370	2472	12	0.583	Exon(exon11of23)	13602	FNDC1	84624
506263	7p	6330446	6330944	499	6	1	Exon(exon2of2)	7749	FAM220A	84792
506263	7p	45082725	45084866	2142	7	0.429	Promoter(1-2kb)	-1325	NACAD	23148
506263	7q	100958977	100960873	1897	47	0.489	Promoter(1-2kb)	1012	MUC3A	4584
506263	7q	100991195	100995785	4591	15	0.6	Exon(exon5of15)	-17269	MUC12	10071
506263	7q	101034361	101040583	6223	38	0.526	Exon(exon3of12)	-3128	MUC17	140453
506263	7q	149818015	149819792	1778	6	0.667	Promoter(2-3kb)	-2352	SSPO	23145
506263	7q	156949807	156950570	764	6	0.833	Promoter(<=1kb)	95	NOM1	64434
506263	8p	10607245	10608432	1188	8	0.5	Exon(exon4of4)	46711	RP1L1	94137
506263	8p	10609614	10610662	1049	8	0.5	Exon(exon4of4)	44481	RP1L1	94137
506263	8p	11331194	11332026	833	8	0.625	Promoter(<=1kb)	306	SLC35G5	83650
506263	8p	12132686	12133940	1255	6	0.667	Promoter(<=1kb)	498	USP17L7	392197
506263	8p	13021128	13022030	903	8	0.25	Exon(exon5of5)	9115	TRMT9B	57604
506263	8q	123651655	123652634	980	7	0.571	Promoter(<=1kb)	316	KLHL38	340359
506263	8q	141466429	141467514	1086	9	0.778	3'UTR	29245	MROH5	389690
506263	9p	34723747	34726527	2781	10	0.7	Promoter(2-3kb)	2961	FAM205A	259308
506263	9q	104598545	104599361	817	12	0.583	Promoter(<=1kb)	52	OR13C5	138799
506263	9q	122553278	122554071	794	6	0.333	Promoter(<=1kb)	108	OR1N2	138882
506263	9q	122628595	122629398	804	6	0.333	Promoter(<=1kb)	175	OR1B1	347169
506263	9q	122749914	122750547	634	6	0.833	Promoter(<=1kb)	174	OR1L6	392390
506263	10p	47663	48605	943	7	0.857	Promoter(<=1kb)	664	TUBB8	347688
506263	10q	46549378	46550723	1346	25	0.64	Exon(exon3of3)	4807	GPRIN2	9721
506263	10q	128103129	128104752	1624	8	0.5	Promoter(<=1kb)	-1	MKI67	4288
506263	10q	128106210	128108204	1995	6	1	Exon(exon12of14)	-3082	MKI67	4288
506263	11p	244106	244197	92	8	0.5	Promoter(<=1kb)	-232	PSMD13	5719
506263	11p	1194354	1196902	2549	7	0.571	Exon(exon34of49)	-26164	MUC5B	727897
506263	11p	1243578	1245307	1730	6	0.667	Exon(exon31of49)	4369	MUC5B-AS1	112577518
506263	11p	1250091	1251524	1434	7	0.714	Promoter(<=1kb)	-415	MUC5B-AS1	112577518
506263	11p	5177811	5178478	668	7	0.143	Promoter(<=1kb)	186	OR52Z1	283110
506263	11p	5323362	5324256	895	7	0.429	Promoter(<=1kb)	41	OR51B2	79345
506263	11p	5389704	5390350	647	7	0.429	Promoter(<=1kb)	327	OR51M1	390059
506263	11p	5422212	5423123	912	10	0.7	Promoter(<=1kb)	101	OR51Q1	390061
506263	11p	5544676	5545259	584	6	0.5	Promoter(<=1kb)	290	OR52H1	390067
506263	11p	5581045	5581738	694	8	0.375	Promoter(<=1kb)	168	OR52B6	340980
506263	11p	5884818	5885061	244	6	0.333	Promoter(<=1kb)	547	OR52E4	390081
506263	11p	5986042	5986669	628	7	1	Promoter(<=1kb)	316	OR52L1	338751
506263	11p	11351961	11352736	776	9	0.222	Promoter(<=1kb)	514	CSNK2A3	283106
506263	11p	12293639	12294368	730	6	0.833	Exon(exon29of35)	6739	MICALCL	84953
506263	11p	34916266	34916763	498	6	0.667	Promoter(<=1kb)	0	APIP	51074
506263	11q	58214757	58215722	966	7	0.286	Promoter(<=1kb)	12	OR1S1	219959
506263	11q	58402523	58403265	743	8	0.5	Promoter(<=1kb)	144	OR5B3	441608
506263	11q	64315797	64315856	60	8	0.75	Promoter(1-2kb)	1485	TRMT112	51504
506263	11q	85724687	85725825	1139	6	0.5	Promoter(<=1kb)	0	SYTL2	54843
506263	11q	93697503	93700096	2594	11	0.636	Promoter(<=1kb)	0	CEP295	85459
506263	11q	123906589	123907324	736	7	0.714	Promoter(<=1kb)	443	OR8D4	338662
506263	11q	124015600	124016477	878	7	0.143	Promoter(<=1kb)	25	OR10G4	390264
506263	11q	124038366	124038988	623	9	0.889	Promoter(<=1kb)	13	OR10G7	390265
506263	12p	4626568	4627524	957	7	0.429	Exon(exon5of6)	14051	DYRK4	8798
506263	12p	6453119	6453670	552	7	0.714	Promoter(<=1kb)	633	TAPBPL	55080
506263	13q	25096713	25097182	470	16	0.438	Promoter(<=1kb)	845	PABPC3	5042
506263	13q	102732474	102733933	1460	6	0.333	Exon(exon4of4)	25139	CCDC168	643677
506263	14q	20060048	20060884	837	8	0.625	Promoter(<=1kb)	3	OR4L1	122742
506263	14q	21634137	21634589	453	9	0.556	Promoter(<=1kb)	351	OR10G2	26534
506263	14q	22633879	22634450	572	8	0.375	Exon(exon2of2)	32212	ABHD4	63874
506263	14q	44504986	44506403	1418	6	0.667	Promoter(<=1kb)	880	FSCB	84075
506263	14q	70457520	70458540	1021	12	0.333	Exon(exon2of2)	5346	ADAM21	8747
506263	14q	94587512	94587839	328	6	0.5	Exon(exon2of2)	-4219	SERPINA3	12
506263	14q	104939262	104945444	6183	18	0.389	5'UTR	7102	PLD4	122618
506263	14q	104947901	104953878	5978	33	0.545	Promoter(1-2kb)	1524	AHNAK2	113146
506263	15q	23439979	23442067	2089	12	0.583	5'UTR	5167	GOLGA6L2	283685
506263	15q	85579423	85581800	2378	16	0.5	Promoter(1-2kb)	-1110	AKAP13	11214
506263	15q	88854874	88855594	721	6	0.833	Exon(exon12of18)	7631	ACAN	176
506263	15q	88857108	88859365	2258	7	0.143	Exon(exon12of18)	9865	ACAN	176
506263	15q	100569472	100570097	626	6	0.833	Promoter(<=1kb)	534	LINS1	55180
506263	16p	1228744	1229731	988	10	0.7	Promoter(<=1kb)	431	TPSB2	64499
506263	16p	1256345	1256980	636	8	0.75	Promoter(<=1kb)	276	TPSD1	23430
506263	16p	1486371	1488463	2093	8	0.75	Promoter(<=1kb)	4	PTX4	390667
506263	16q	74391401	74392004	604	10	0.7	Exon(exon7of7)	13523	NPIPB15	440348
506263	16q	79211868	79212064	197	6	0.667	3'UTR	15509	WWOX	51741
506263	16q	84195142	84196894	1753	6	0.667	Promoter(<=1kb)	0	ADAD2	161931
506263	16q	88428539	88436097	7559	20	0.55	Exon(exon3of3)	-17183	ZFPM1	161882
506263	16q	89226863	89228289	1427	9	0.667	Promoter(2-3kb)	2229	ZNF778	197320

506263	17p	2299649	2300159	511	6	0.167	Exon(exon2of19)	-3224	SRR	63826
506263	17p	10638198	10641099	2902	7	0.286	Exon(exon19of41)	-8169	MYH3	4621
506263	17p	21300581	21300978	398	12	0.75	3'UTR	9112	MAP2K3	5606
506263	17q	73236548	73236991	444	6	0.333	Promoter(<=1kb)	0	FAM104A	84923
506263	17q	76293419	76294016	598	6	0.5	Promoter(2-3kb)	-2167	QRICH2	84074
506263	17q	81645135	81645417	283	6	0.333	Promoter(2-3kb)	2722	TSPAN10	83882
506263	18p	11609728	11610581	854	11	0.636	Promoter(<=1kb)	132	SLC35G4	646000
506263	18p	11644365	11644734	370	7	0.714	Exon(exon1of1)	10207	MIR7153	102465690
506263	18q	58535186	58537515	2330	9	0.333	Promoter(<=1kb)	0	ALPK2	115701
506263	18q	75285722	75287404	1683	6	0.333	Exon(exon2of2)	41146	TSHZ1	10194
506263	19p	4510548	4513547	3000	21	0.476	Exon(exon3of6)	4157	PLIN4	729359
506263	19p	8333830	8334965	1136	6	0.667	Promoter(<=1kb)	0	KANK3	256949
506263	19p	8948231	8952171	3941	12	0.5	Exon(exon3of84)	29171	MUC16	94025
506263	19p	8959403	8961248	1846	6	0.667	Exon(exon3of84)	20094	MUC16	94025
506263	19p	9126210	9126946	737	9	0.556	Promoter(<=1kb)	4	OR7G3	390883
506263	19p	12430718	12432437	1720	8	0.375	3'UTR	8584	ZNF443	10224
506263	19p	21971930	21974500	2571	10	0.7	Exon(exon4of4)	14408	ZNF208	7757
506263	19q	37886924	37889059	2136	6	0.833	Exon(exon6of6)	17535	WDR87	83889
506263	19q	39877222	39877880	659	6	0.5	Exon(exon20of28)	9412	FCGBP	8857
506263	19q	39886240	39886422	183	6	0.667	Promoter(<=1kb)	870	FCGBP	8857
506263	19q	40880128	40880622	495	7	0.286	Promoter(<=1kb)	-38	CYP2A7	1549
506263	19q	43913423	43914878	1456	9	0.556	Exon(exon10of10)	4861	ZNF45	7596
506263	19q	43966037	43967171	1135	6	0.167	Promoter(<=1kb)	-691	ZNF155	7711
506263	19q	43996326	43997366	1041	6	0.5	Exon(exon5of5)	5419	LOC101928063	101928063
506263	19q	44106512	44108078	1567	8	0.125	Exon(exon6of6)	-4103	ZNF225	7768
506263	19q	52437918	52439242	1325	7	0.429	Exon(exon4of4)	6504	ZNF534	147658
506263	19q	55911888	55913166	1279	7	0.714	Exon(exon5of12)	19145	NLRP13	126204
506263	20p	5922421	5923394	974	6	0.5	Exon(exon4of5)	6923	CHGB	1114
506263	20q	63349752	63350772	1021	6	0.5	3'UTR	3794	CHRNA4	1137
506263	20q	63561666	63565531	3866	13	0.615	Promoter(<=1kb)	-61	HELZ2	85441
506263	21q	26843740	26844859	1120	6	0.667	Promoter(<=1kb)	0	ADAMTS1	9510
506263	21q	44579776	44580521	746	6	0.667	Promoter(<=1kb)	83	KRTAP10-5	386680
506263	21q	44591587	44592404	818	6	0.333	Promoter(<=1kb)	101	KRTAP10-6	386674
506263	21q	44600627	44601692	1066	10	0.7	Promoter(<=1kb)	30	KRTAP10-7	386675
506263	21q	44637474	44638143	670	8	0.5	Promoter(<=1kb)	118	KRTAP10-10	353333
506263	22q	22352950	22353298	349	13	0.538	Exon(exon1of2)	30478	BMS1P20	96610
506263	22q	22646441	22646811	371	6	0.333	Promoter(<=1kb)	0	GGTLC2	91227
506263	22q	36191154	36191906	753	6	0.667	3'UTR	9971	APOL4	80832
506263	23p	8170039	8170141	103	6	0.5	Promoter(1-2kb)	1126	VCX2	51480
506263	23p	35802148	35803010	863	7	0.571	5'UTR	3357	MAGEB16	139604
506616	1p	16048038	16049824	1787	6	0.5	Promoter(<=1kb)	0	CLCNKB	1188
506616	1p	16058491	16060000	1510	13	0.846	Exon(exon5of7)	6168	CLCNKB	1188
506616	1p	18481403	18482217	815	7	0.714	Promoter(<=1kb)	421	KLHDC7A	127707
506616	1p	32695043	32696011	969	6	0.5	5'UTR	6745	SYNC	81493
506616	1p	40067594	40067675	82	6	0	Promoter(<=1kb)	324	CAP1	10487
506616	1p	62273232	62274777	1546	6	0.333	Promoter(<=1kb)	-441	KANK4	163782
506616	1p	89186388	89186419	32	9	0.556	Promoter(<=1kb)	107	GBP4	115361
506616	1q	152218469	152221375	2907	16	0.688	Promoter(2-3kb)	2818	HRNR	388697
506616	1q	152303673	152305241	1569	9	0.556	Exon(exon3of3)	-7335	FLG-AS1	339400
506616	1q	152307253	152313891	6639	27	0.667	Promoter(<=1kb)	0	FLG-AS1	339400
506616	1q	156669844	156670886	1043	6	1	Exon(exon4of4)	6521	NES	10763
506616	1q	158765805	158766655	851	6	0.5	Promoter(<=1kb)	47	OR6N1	128372
506616	1q	169542317	169542882	566	6	0.167	Exon(exon13of25)	-26572	F5	2153
506616	1q	214640144	214642954	2811	12	0.5	Exon(exon12of20)	-5013	CENPF	1063
506616	1q	214644872	214647181	2310	10	0.4	Promoter(<=1kb)	-786	CENPF	1063
506616	1q	223393517	223394466	950	6	0.667	Promoter(<=1kb)	102	CCDC185	164127
506616	1q	228315976	228318049	2074	7	0.571	Exon(exon50of81)	6492	OBSCN	84033
506616	1q	247841312	247841582	271	6	0.833	Promoter(<=1kb)	314	OR11L1	391189
506616	1q	247949325	247949738	414	11	0.273	Promoter(<=1kb)	467	OR2L8	391190
506616	1q	248681658	248682198	541	7	0.571	Promoter(<=1kb)	130	OR14I1	401994
506616	2p	29002636	29003646	1011	6	0.333	Exon(exon5of20)	-10821	TOGARAM2	165186
506616	2p	29071763	29073000	1238	6	0.667	Promoter(1-2kb)	1523	PCARE	388939
506616	2p	48580657	48582454	1798	7	0.571	Promoter(<=1kb)	0	STON1	11037
506616	2q	130914528	130916712	2185	6	0.333	Promoter(<=1kb)	0	ARHGEF4	50649
506616	2q	132783032	132785012	1981	7	0.571	Promoter(1-2kb)	-1009	NCKAP5	344148
506616	2q	178739433	178741811	2379	6	0.5	Exon(exon45of191)	26014	TTN	7273
506616	2q	217847583	217848559	977	6	0.833	Exon(exon19of33)	-5423	TNS1	7145
506616	2q	233713134	233713664	531	8	0.75	Promoter(<=1kb)	142	UGT1A5	54579
506616	2q	238130271	238131546	1276	7	0.286	Promoter(1-2kb)	1323	ESPNL	339768
506616	2q	240041845	240042131	287	6	0.167	Downstream(2-3kb)	3941	OR6B3	150681
506616	3p	31989532	31990905	1374	7	0.286	Exon(exon2of2)	7761	ZNF860	344787
506616	3p	75737230	75739007	1778	9	0.556	Promoter(<=1kb)	0	MIR4273	100422955
506616	3q	98264413	98265137	725	6	0.667	Promoter(<=1kb)	128	OR5H6	79295
506616	4p	5988383	5989749	1367	7	0.571	Promoter(<=1kb)	0	C4orf50	389197
506616	4p	6300792	6302360	1569	6	0.833	Exon(exon8of8)	6021	WFS1	7466
506616	4p	8227004	8228508	1505	8	0.125	Promoter(<=1kb)	-24	SH3TC1	54436
506616	4q	121036404	121037542	1139	6	0.333	Promoter(1-2kb)	1442	NDNF	79625

506616	4q	185458217	185460011	1795	8	0.625	Promoter(<=1kb)	0	CCDC110	256309
506616	5p	795818	796237	420	9	0.556	3'UTR	4908	ZDHHC11	79844
506616	5q	54518000	54519730	1731	6	0.167	Promoter(<=1kb)	135	SNX18	112574
506616	5q	79728956	79730716	1761	6	0.333	Exon(exon2of13)	-7426	CMYA5	202333
506616	5q	79731782	79734523	2742	13	0.308	Exon(exon2of13)	-3619	CMYA5	202333
506616	5q	140848579	140850786	2208	6	0.5	Promoter(<=1kb)	807	PCDHA9	9752
506616	5q	141174000	141175025	1026	6	0.833	Promoter(1-2kb)	1356	PCDHB7	56129
506616	5q	141183999	141184688	690	6	0.833	Promoter(2-3kb)	-2473	PCDHB9	56127
506616	5q	141187690	141189425	1736	7	0.571	Promoter(<=1kb)	529	PCDHB9	56127
506616	5q	141955356	141957660	2305	7	0.714	Promoter(<=1kb)	-668	RNF14	9604
506616	5q	151565922	151568158	2237	9	0.778	Promoter(<=1kb)	786	FAT2	2196
506616	6p	1312843	1313745	903	6	0.5	Promoter(<=1kb)	745	FOXQ1	94234
506616	6p	46858771	46859389	619	6	0.5	Exon(exon17of21)	3915	ADGRF5	221395
506616	6q	159231899	159234370	2472	12	0.583	Exon(exon11of23)	13602	FNDC1	84624
506616	7q	100958721	100960873	2153	55	0.473	Promoter(<=1kb)	756	MUC3A	4584
506616	7q	100991195	100992398	1204	7	0.571	Exon(exon5of15)	-20656	MUC12	10071
506616	7q	100995575	100995896	322	9	0.778	Exon(exon5of15)	-17158	MUC12	10071
506616	7q	101004421	101004836	416	6	0.667	Exon(exon5of15)	-8218	MUC12	10071
506616	7q	101034361	101040583	6223	40	0.525	Exon(exon3of12)	-3128	MUC17	140453
506616	7q	149818015	149819792	1778	6	0.667	Promoter(2-3kb)	-2352	SSPO	23145
506616	7q	149824094	149826495	2402	6	0.667	Promoter(<=1kb)	0	SSPO	23145
506616	8p	10607245	10608261	1017	7	0.571	Exon(exon4of4)	46882	RP1L1	94137
506616	8p	10609650	10612607	2958	9	0.889	Exon(exon4of4)	42536	RP1L1	94137
506616	8p	12137448	12138641	1194	6	0.833	Promoter(<=1kb)	436	USP17L2	377630
506616	8p	13021128	13022030	903	7	0.143	Exon(exon5of5)	9115	TRMT9B	57604
506616	8q	141218050	141219792	1743	6	0.667	5'UTR	8778	SLC45A4	57210
506616	8q	141466429	141467514	1086	9	0.778	3'UTR	29245	MROH5	389690
506616	9q	76703555	76707804	4250	15	0.533	Promoter(<=1kb)	0	PCA3	50652
506616	9q	76709263	76710843	1581	8	0.5	Promoter(<=1kb)	0	PRUNE2	158471
506616	9q	87886533	87888713	2181	8	0.625	Exon(exon4of4)	3656	SPATA31E1	286234
506616	9q	104598545	104599361	817	12	0.583	Promoter(<=1kb)	52	OR13C5	138799
506616	9q	122553263	122554071	809	7	0.429	Promoter(<=1kb)	93	OR1N2	138882
506616	9q	122749914	122750547	634	6	0.833	Promoter(<=1kb)	174	OR1L6	392390
506616	9q	135484803	135487213	2411	8	0.25	Promoter(1-2kb)	1440	PPP1R26	9858
506616	9q	135547960	135548795	836	8	0.625	Promoter(1-2kb)	1805	OBP2A	29991
506616	10q	46549378	46550723	1346	26	0.654	Exon(exon3of3)	4807	GPRIN2	9721
506616	10q	49323169	49326817	3649	15	0.533	Exon(exon3of3)	23895	C10orf71	118461
506616	10q	122084988	122087840	2853	8	0.75	Exon(exon4of23)	-25190	TACC2	10579
506616	10q	128102594	128105201	2608	6	0.667	Promoter(<=1kb)	0	MKI67	4288
506616	11p	244106	244197	92	8	0.5	Promoter(<=1kb)	-232	PSMD13	5719
506616	11p	308290	309127	838	6	0.5	Promoter(<=1kb)	0	IFITM2	10581
506616	11p	1194354	1196902	2549	7	0.571	Exon(exon34of49)	-26164	MUC5B	727897
506616	11p	1246095	1252431	6337	21	0.619	Promoter(<=1kb)	0	MUC5B-AS1	112577518
506616	11p	5046754	5047432	679	7	0.571	Promoter(<=1kb)	228	OR52J3	119679
506616	11p	5177978	5178478	501	6	0.167	Promoter(<=1kb)	186	OR52Z1	283110
506616	11p	5323451	5324256	806	6	0.5	Promoter(<=1kb)	41	OR51B2	79345
506616	11p	5422212	5423123	912	11	0.636	Promoter(<=1kb)	101	OR51Q1	390061
506616	11p	5515185	5515931	747	6	0.333	Promoter(<=1kb)	768	UBQLNL	143630
506616	11p	5581045	5581738	694	8	0.375	Promoter(<=1kb)	168	OR52B6	340980
506616	11p	5841302	5841883	582	9	0.333	Promoter(<=1kb)	14	OR52E6	390078
506616	11p	5884818	5885061	244	7	0.429	Promoter(<=1kb)	547	OR52E4	390081
506616	11p	5986042	5986542	501	6	1	Promoter(<=1kb)	443	OR52L1	338751
506616	11p	11351961	11352736	776	9	0.222	Promoter(<=1kb)	514	CSNK2A3	283106
506616	11p	12293639	12294538	900	8	0.625	Exon(exon29of35)	6739	MICALCL	84953
506616	11q	58214757	58215722	966	8	0.25	Promoter(<=1kb)	12	OR1S1	219959
506616	11q	85724687	85725825	1139	6	0.5	Promoter(<=1kb)	0	SYTL2	54843
506616	11q	123906790	123907324	535	6	0.667	Promoter(<=1kb)	644	OR8D4	338662
506616	11q	130914501	130915409	909	10	0.7	Promoter(1-2kb)	1035	SNX19	399979
506616	12p	4626568	4628549	1982	10	0.5	Exon(exon5of6)	14051	DYRK4	8798
506616	12p	6453119	6453670	552	6	0.667	Promoter(<=1kb)	633	TAPBPL	55080
506616	12p	31980987	31982507	1521	6	0.5	Exon(exon3of3)	-4659	RESF1	55196
506616	13q	24434450	24435347	898	7	0.571	Exon(exon31of34)	19787	PARP4	143
506616	13q	25096675	25096984	310	9	0.444	Promoter(<=1kb)	807	PABPC3	5042
506616	13q	102732424	102733933	1510	7	0.429	Exon(exon4of4)	25139	CCDC168	643677
506616	14q	19975713	19976448	736	9	0.444	Promoter(<=1kb)	269	OR4K15	81127
506616	14q	20060048	20060523	476	6	0.5	Promoter(<=1kb)	3	OR4L1	122742
506616	14q	70457532	70458540	1009	9	0.333	Exon(exon2of2)	5358	ADAM21	8747
506616	14q	94587512	94587839	328	6	0.5	Exon(exon2of2)	-4219	SERPINA3	12
506616	14q	104175455	104178720	3266	9	0.444	Exon(exon12of15)	36415	KIF26A	26153
506616	15q	23439979	23442067	2089	12	0.583	5'UTR	5167	GOLGA6L2	283685
506616	15q	40621642	40624434	2793	8	0.375	Promoter(<=1kb)	0	KNL1	57082
506616	15q	73702465	73703760	1296	6	0.833	Promoter(<=1kb)	-149	CD276	80381
506616	15q	85579423	85582073	2651	15	0.6	Promoter(<=1kb)	-837	AKAP13	11214
506616	15q	99129423	99132517	3095	10	0.5	Exon(exon4of5)	7225	TTC23	64927
506616	15q	100569472	100570097	626	6	0.833	Promoter(<=1kb)	534	LINS1	55180
506616	16p	670707	672942	2236	7	0.857	Promoter(<=1kb)	0	RHOT2	89941
506616	16p	1228744	1229622	879	12	0.75	Promoter(<=1kb)	540	TPSB2	64499

506616	16p	1486371	1488463	2093	8	0.75	Promoter(<=1kb)	4	PTX4	390667
506616	16p	4207130	4208004	875	6	0.5	Exon(exon2of7)	31739	SRL	6345
506616	16q	88714632	88717113	2482	7	0.429	Promoter(<=1kb)	0	MIR4722	100616167
506616	16q	89100686	89101050	365	8	0.625	Promoter(<=1kb)	24	ACSF3	197322
506616	16q	89227116	89228419	1304	6	0.667	Promoter(2-3kb)	2482	ZNF778	197320
506616	17p	10638198	10641099	2902	7	0.286	Exon(exon19of41)	-8169	MYH3	4621
506616	17p	21300581	21300954	374	8	0.75	3'UTR	9112	MAP2K3	5606
506616	17q	76293419	76294016	598	6	0.5	Promoter(2-3kb)	-2167	QRICH2	84074
506616	17q	80090158	80090265	108	6	0.333	Promoter(2-3kb)	2053	CCDC40	55036
506616	17q	81645135	81645607	473	7	0.286	Promoter(2-3kb)	2722	TSPAN10	83882
506616	18p	11609728	11610383	656	11	0.909	Promoter(<=1kb)	132	SLC35G4	646000
506616	18q	58535186	58537515	2330	9	0.333	Promoter(<=1kb)	0	ALPK2	115701
506616	19p	4510548	4513547	3000	19	0.526	Exon(exon3of6)	4157	PLIN4	729359
506616	19p	5455600	5456439	840	7	0.571	Promoter(<=1kb)	183	ZNRF4	148066
506616	19p	5831566	5832198	633	6	1	Promoter(2-3kb)	2879	FUT6	2528
506616	19p	8937644	8939234	1591	6	0.833	Exon(exon5of84)	-41554	MUC16	94025
506616	19p	8946306	8951868	5563	16	0.625	Exon(exon3of84)	29474	MUC16	94025
506616	19p	8959116	8962299	3184	11	0.727	Exon(exon3of84)	19043	MUC16	94025
506616	19p	8972751	8978164	5414	17	0.471	Exon(exon1of84)	3178	MUC16	94025
506616	19p	12430718	12432437	1720	10	0.4	3'UTR	8584	ZNF443	10224
506616	19p	18264753	18267409	2657	8	0.5	5'UTR	7002	IQCN	80726
506616	19p	21971930	21974500	2571	7	0.714	Exon(exon4of4)	14408	ZNF208	7757
506616	19p	22756294	22759533	3240	11	0.545	3'UTR	10449	ZNF99	7652
506616	19p	23743906	23745300	1395	6	0.333	Exon(exon4of4)	13537	ZNF681	148213
506616	19q	34943334	34944685	1352	6	0.333	3'UTR	9723	ZNF30	90075
506616	19q	36996730	36997597	868	10	0.6	Exon(exon10of10)	5677	ZNF568	374900
506616	19q	37886924	37888806	1883	6	0.667	Exon(exon6of6)	17788	WDR87	83889
506616	19q	39877222	39877880	659	6	0.5	Exon(exon20of28)	9412	FCGBP	8857
506616	19q	39886005	39886422	418	7	0.571	Promoter(<=1kb)	870	FCGBP	8857
506616	19q	43846955	43848536	1582	6	0.833	3'UTR	13450	ZNF283	284349
506616	19q	43913423	43914878	1456	8	0.5	Exon(exon10of10)	4861	ZNF45	7596
506616	19q	43966037	43967171	1135	6	0.167	Promoter(<=1kb)	-691	ZNF155	7711
506616	19q	44106512	44108078	1567	9	0.111	Exon(exon6of6)	-4103	ZNF225	7768
506616	19q	44327836	44329698	1863	6	0.5	Exon(exon4of4)	-22790	ZNF235	9310
506616	19q	52437918	52439242	1325	7	0.429	Exon(exon4of4)	6504	ZNF534	147658
506616	19q	58368293	58368875	583	7	0.429	Exon(exon3of3)	-5445	ZNF497	162968
506616	20p	5922577	5923382	806	8	0.625	Exon(exon4of5)	7079	CHGB	1114
506616	20p	20052354	20052736	383	6	0.167	Promoter(<=1kb)	0	CFAP61	26074
506616	20q	63561666	63565531	3866	13	0.615	Promoter(<=1kb)	-61	HELZ2	85441
506616	21q	26843740	26844859	1120	6	0.667	Promoter(<=1kb)	0	ADAMTS1	9510
506616	21q	44550835	44551416	582	6	0.833	Promoter(<=1kb)	89	KRTAP10-2	386679
506616	21q	44591587	44592404	818	9	0.222	Promoter(<=1kb)	101	KRTAP10-6	386674
506616	21q	44600627	44601692	1066	11	0.636	Promoter(<=1kb)	30	KRTAP10-7	386675
506616	21q	44637476	44638143	668	6	0.667	Promoter(<=1kb)	120	KRTAP10-10	353333
506616	22q	22352950	22353380	431	16	0.5	Exon(exon1of2)	30478	BMS1P20	96610
506616	22q	36191154	36191906	753	6	0.667	3'UTR	9971	APOL4	80832
506616	23p	8170039	8170141	103	6	0.5	Promoter(1-2kb)	1126	VCX2	51480
506616	23p	35802148	35803010	863	7	0.571	5'UTR	3357	MAGEB16	139604
506820	1p	12859108	12860212	1105	10	0.5	Promoter(2-3kb)	2022	PRAMEF2	65122
506820	1p	16058491	16060000	1510	13	0.846	Exon(exon5of7)	6168	CLCNKB	1188
506820	1p	18481403	18482217	815	7	0.714	Promoter(<=1kb)	421	KLHDC7A	127707
506820	1p	40067594	40067675	82	7	0	Promoter(<=1kb)	324	CAP1	10487
506820	1p	89186388	89186419	32	9	0.556	Promoter(<=1kb)	107	GBP4	115361
506820	1q	152219233	152221375	2143	16	0.75	Promoter(2-3kb)	2818	HRNR	388697
506820	1q	156669844	156671745	1902	7	1	Exon(exon4of4)	5662	NES	10763
506820	1q	158765805	158766655	851	6	0.5	Promoter(<=1kb)	47	OR6N1	128372
506820	1q	169542317	169542882	566	6	0.167	Exon(exon13of25)	-26572	F5	2153
506820	1q	201206099	201209342	3244	10	0.6	Promoter(1-2kb)	1017	IGFN1	91156
506820	1q	201210956	201212792	1837	7	0.429	Promoter(<=1kb)	0	IGFN1	91156
506820	1q	226735695	226737239	1545	6	0.833	Promoter(<=1kb)	219	ITPKB	3707
506820	1q	228315976	228318038	2063	9	0.667	Exon(exon50of81)	6492	OBSCN	84033
506820	1q	247841312	247841582	271	6	0.833	Promoter(<=1kb)	314	OR11L1	391189
506820	1q	247895625	247896410	786	6	0.5	Promoter(<=1kb)	38	OR2W3	343171
506820	1q	247949325	247949759	435	9	0.333	Promoter(<=1kb)	467	OR2L8	391190
506820	2q	132783534	132785001	1468	7	0.429	Promoter(1-2kb)	-1511	NCKAP5	344148
506820	2q	178564277	178567262	2986	7	0.571	Exon(exon275of312)	-17311	TTN-AS1	100506866
506820	2q	178739433	178741811	2379	6	0.5	Exon(exon45of191)	26014	TTN	7273
506820	2q	185790999	185794632	3634	9	0.778	Promoter(<=1kb)	0	FSIP2	401024
506820	2q	185805377	185807800	2424	6	0.333	Promoter(<=1kb)	0	FSIP2	401024
506820	2q	217847567	217848213	647	6	0.833	Exon(exon19of33)	-5407	TNS1	7145
506820	2q	233681970	233682693	724	6	0.667	Promoter(<=1kb)	0	UGT1A7	54577
506820	2q	233713134	233713664	531	8	0.75	Promoter(<=1kb)	142	UGT1A5	54579
506820	2q	238130416	238131546	1131	6	0.333	Promoter(1-2kb)	1468	ESPNL	339768
506820	3p	75736880	75739243	2364	46	0.587	Promoter(<=1kb)	0	MIR4273	100422955
506820	3q	196947878	196948102	225	6	0.5	3'UTR	3828	PIGZ	80235
506820	4p	5988383	5989749	1367	7	0.571	Promoter(<=1kb)	0	C4orf50	389197
506820	4p	6300792	6302360	1569	7	0.857	Exon(exon8of8)	6021	WFS1	7466

506820	4p	38774486	38775586	1101	7	0.286	Promoter(<=1kb)	610	TLR10	81793
506820	5q	79731782	79734523	2742	13	0.308	Exon(exon2of13)	-3619	CMYA5	202333
506820	5q	140848579	140850786	2208	7	0.571	Promoter(<=1kb)	807	PCDHA9	9752
506820	5q	141955195	141957660	2466	7	0.714	Promoter(<=1kb)	-668	RNF14	9604
506820	5q	148100464	148101867	1404	6	0.333	Exon(exon13of28)	9536	SPINK5	11005
506820	5q	151565922	151568158	2237	9	0.778	Promoter(<=1kb)	786	FAT2	2196
506820	6p	46858771	46859389	619	6	0.5	Exon(exon17of21)	3915	ADGRF5	221395
506820	6q	106511549	106512572	1024	6	0.667	Promoter(<=1kb)	0	CRYBG1	202
506820	6q	149888581	149890867	2287	7	0.714	Promoter(<=1kb)	0	RAET1E-AS1	100652739
506820	6q	159233455	159234370	916	10	0.5	Exon(exon11of23)	15158	FNDC1	84624
506820	7p	12369637	12370736	1100	6	0.667	3'UTR	-13307	VWDE	221806
506820	7p	45082725	45084866	2142	7	0.429	Promoter(1-2kb)	-1325	NACAD	23148
506820	7p	53035678	53036385	708	7	1	Promoter(<=1kb)	45	POM121L12	285877
506820	7q	64991278	64992758	1481	6	0.667	Promoter(<=1kb)	-242	ZNF117	51351
506820	7q	100958977	100960873	1897	50	0.46	Promoter(1-2kb)	1012	MUC3A	4584
506820	7q	100991195	100993165	1971	10	0.7	Exon(exon5of15)	-19889	MUC12	10071
506820	7q	100995575	100995785	211	6	0.833	Exon(exon5of15)	-17269	MUC12	10071
506820	7q	149818015	149819792	1778	6	0.667	Promoter(2-3kb)	-2352	SSPO	23145
506820	7q	156949807	156950570	764	6	0.833	Promoter(<=1kb)	95	NOM1	64434
506820	8p	8376561	8377198	638	6	1	Exon(exon2of5)	4549	PRAG1	157285
506820	8p	10609614	10612307	2694	10	0.9	Exon(exon4of4)	42836	RP1L1	94137
506820	8p	11331234	11332082	849	6	0.667	Promoter(<=1kb)	346	SLC35G5	83650
506820	8p	13021128	13022030	903	8	0.25	Exon(exon5of5)	9115	TRMT9B	57604
506820	8q	123651873	123652634	762	6	0.333	Promoter(<=1kb)	316	KLHL38	340359
506820	8q	138151949	138153046	1098	6	0.667	Promoter(<=1kb)	0	FAM135B	51059
506820	9p	116800	117696	897	7	0.857	Promoter(<=1kb)	508	FOXD4	2298
506820	9p	21206764	21207074	311	6	0.5	Promoter(<=1kb)	69	IFNA10	3446
506820	9p	34723747	34726473	2727	7	0.714	Exon(exon4of4)	3015	FAM205A	259308
506820	9q	76175237	76175300	64	6	0.5	Exon(exon14of14)	-13339	PCSK5	5125
506820	9q	76705179	76707804	2626	8	0.5	Promoter(<=1kb)	121	PCA3	50652
506820	9q	76709263	76710843	1581	8	0.5	Promoter(<=1kb)	0	PRUNE2	158471
506820	9q	122553263	122554071	809	7	0.429	Promoter(<=1kb)	93	OR1N2	138882
506820	9q	122749914	122750547	634	6	0.833	Promoter(<=1kb)	174	OR1L6	392390
506820	10q	46549378	46550723	1346	25	0.64	Exon(exon3of3)	4807	GPRIN2	9721
506820	10q	49323169	49325572	2404	8	0.5	Exon(exon3of3)	23895	C10orf71	118461
506820	10q	103602059	103602530	472	6	0.667	Exon(exon15of15)	30382	NEURL1	9148
506820	10q	128102595	128104752	2158	9	0.556	Promoter(<=1kb)	0	MKI67	4288
506820	10q	128106210	128108204	1995	6	1	Exon(exon12of14)	-3082	MKI67	4288
506820	10q	133625384	133625511	128	7	0.714	Promoter(1-2kb)	1281	FRG2B	441581
506820	11p	244106	244197	92	8	0.5	Promoter(<=1kb)	-232	PSMD13	5719
506820	11p	1245466	1247378	1913	8	0.5	Promoter(2-3kb)	2298	MUC5B-AS1	112577518
506820	11p	5177978	5178478	501	6	0.167	Promoter(<=1kb)	186	OR52Z1	283110
506820	11p	5389704	5390350	647	7	0.429	Promoter(<=1kb)	327	OR51M1	390059
506820	11p	5422212	5423123	912	11	0.727	Promoter(<=1kb)	101	OR51Q1	390061
506820	11p	5581045	5581738	694	8	0.375	Promoter(<=1kb)	168	OR52B6	340980
506820	11p	5788000	5788760	761	8	0.75	Promoter(<=1kb)	56	OR52N1	79473
506820	11p	5841302	5841883	582	9	0.333	Promoter(<=1kb)	14	OR52E6	390078
506820	11p	11351961	11352736	776	9	0.222	Promoter(<=1kb)	514	CSNK2A3	283106
506820	11p	12293584	12294368	785	7	0.857	Exon(exon29of35)	6684	MICALCL	84953
506820	11p	34916266	34916763	498	6	0.667	Promoter(<=1kb)	0	APIP	51074
506820	11q	58214757	58215722	966	8	0.25	Promoter(<=1kb)	12	OR1S1	219959
506820	11q	85724687	85725825	1139	6	0.5	Promoter(<=1kb)	0	SYTL2	54843
506820	11q	93697503	93700096	2594	12	0.667	Promoter(<=1kb)	0	CEP295	85459
506820	11q	123906790	123907324	535	6	0.667	Promoter(<=1kb)	644	OR8D4	338662
506820	11q	124038366	124038988	623	7	1	Promoter(<=1kb)	13	OR10G7	390265
506820	11q	124185670	124186080	411	6	0.333	Promoter(<=1kb)	454	OR10D3	26497
506820	11q	130914501	130915409	909	10	0.7	Promoter(1-2kb)	1035	SNX19	399979
506820	12p	4626571	4628549	1979	9	0.444	Exon(exon5of6)	14054	DYRK4	8798
506820	12q	52316096	52317765	1670	6	0.667	Exon(exon4of9)	3633	KRT83	3889
506820	13q	102732474	102733933	1460	6	0.333	Exon(exon4of4)	25139	CCDC168	643677
506820	14q	19975713	19976448	736	9	0.444	Promoter(<=1kb)	269	OR4K15	81127
506820	14q	20060048	20060884	837	8	0.625	Promoter(<=1kb)	3	OR4L1	122742
506820	14q	21634137	21634589	453	9	0.556	Promoter(<=1kb)	351	OR10G2	26534
506820	14q	44504986	44506403	1418	7	0.714	Promoter(<=1kb)	880	FSCB	84075
506820	14q	70457520	70458540	1021	9	0.333	Exon(exon2of2)	5346	ADAM21	8747
506820	14q	94587512	94587839	328	6	0.5	Exon(exon2of2)	-4219	SERPINA3	12
506820	14q	104939262	104942618	3357	10	0.2	5'UTR	7102	PLD4	122618
506820	14q	104943622	104945444	1823	7	0.571	Exon(exon6of6)	9958	AHNAK2	113146
506820	14q	104947943	104950411	2469	26	0.538	Exon(exon6of6)	4991	AHNAK2	113146
506820	14q	104951557	104953878	2322	12	0.417	Promoter(1-2kb)	1524	AHNAK2	113146
506820	15q	23440370	23442067	1698	11	0.636	5'UTR	5167	GOLGA6L2	283685
506820	15q	40621642	40624039	2398	8	0.25	Promoter(<=1kb)	0	KNL1	57082
506820	15q	59206980	59208056	1077	7	0.714	Promoter(<=1kb)	137	LDHAL6B	92483
506820	15q	78766033	78766632	600	8	0.625	Promoter(<=1kb)	-920	ADAMTS7	11173
506820	15q	85579423	85581800	2378	14	0.571	Promoter(1-2kb)	-1110	AKAP13	11214
506820	16p	1228744	1229583	840	9	0.778	Promoter(<=1kb)	579	TPSB2	64499
506820	16q	89227206	89228289	1084	6	0.667	Promoter(2-3kb)	2572	ZNF778	197320

506820	17p	21300581	21300978	398	12	0.75	3'UTR	9112	MAP2K3	5606
506820	17p	21415470	21416370	901	8	0.75	Exon(exon3of3)	10334	KCNJ12	3768
506820	17q	81510690	81511591	902	6	0.667	Promoter(<=1kb)	257	ACTG1	71
506820	17q	81645135	81645607	473	7	0.286	Promoter(2-3kb)	2722	TSPAN10	83882
506820	18p	9887397	9888072	676	6	0.667	Promoter(1-2kb)	1383	TXNDC2	84203
506820	18p	11609646	11610164	519	9	0.778	Promoter(<=1kb)	50	SLC35G4	646000
506820	18q	47033929	47035145	1217	6	1	Promoter(<=1kb)	476	ELOA2	51224
506820	18q	58535186	58538030	2845	19	0.526	Promoter(<=1kb)	0	ALPK2	115701
506820	19p	4510548	4513547	3000	19	0.526	Exon(exon3of6)	4157	PLIN4	729359
506820	19p	5455600	5456439	840	8	0.625	Promoter(<=1kb)	183	ZNRF4	148066
506820	19p	8946313	8951868	5556	15	0.6	Exon(exon3of84)	29474	MUC16	94025
506820	19p	8959116	8962299	3184	10	0.7	Exon(exon3of84)	19043	MUC16	94025
506820	19p	8971838	8978096	6259	15	0.533	Exon(exon1of84)	3246	MUC16	94025
506820	19p	12075333	12077046	1714	6	0.167	Promoter(<=1kb)	6	ZNF788P	388507
506820	19p	18264798	18267409	2612	12	0.583	5'UTR	7002	IQCN	80726
506820	19p	21971930	21974500	2571	8	0.75	Exon(exon4of4)	14408	ZNF208	7757
506820	19p	22757857	22759533	1677	11	0.455	Exon(exon4of4)	10449	ZNF99	7652
506820	19p	23743906	23745300	1395	6	0.333	Exon(exon4of4)	13537	ZNF681	148213
506820	19q	36996730	36997597	868	11	0.636	Exon(exon10of10)	5677	ZNF568	374900
506820	19q	37151928	37153149	1222	6	0.833	Exon(exon5of5)	19287	ZNF585A	199704
506820	19q	37885190	37888806	3617	9	0.556	Exon(exon6of6)	17788	WDR87	83889
506820	19q	39886005	39886422	418	8	0.625	Promoter(<=1kb)	870	FCGBP	8857
506820	19q	40880128	40880622	495	7	0.286	Promoter(<=1kb)	-38	CYP2A7	1549
506820	19q	43204061	43205504	1444	6	0.833	Promoter(<=1kb)	0	PSG4	5672
506820	19q	43913423	43914878	1456	9	0.556	Exon(exon10of10)	4861	ZNF45	7596
506820	19q	44106512	44108078	1567	7	0	Exon(exon6of6)	-4103	ZNF225	7768
506820	19q	51745958	51746963	1006	6	0.5	Exon(exon3of3)	3848	FPR1	2357
506820	19q	52437918	52439242	1325	7	0.429	Exon(exon4of4)	6504	ZNF534	147658
506820	19q	55160006	55162286	2281	6	0.333	Promoter(<=1kb)	0	DNAAF3	352909
506820	19q	55481625	55483456	1832	6	0.5	Promoter(1-2kb)	-1732	NAT14	57106
506820	19q	55911888	55913166	1279	7	0.714	Exon(exon5of12)	19145	NLRP13	126204
506820	20p	5922421	5923643	1223	9	0.667	Exon(exon4of5)	6923	CHGB	1114
506820	20q	63561666	63565531	3866	12	0.667	Promoter(<=1kb)	-61	HELZ2	85441
506820	21q	44600627	44601692	1066	9	0.778	Promoter(<=1kb)	30	KRTAP10-7	386675
506820	21q	44637476	44638143	668	6	0.5	Promoter(<=1kb)	120	KRTAP10-10	353333
506820	22q	22352950	22353380	431	16	0.5	Exon(exon1of2)	30478	BMS1P20	96610
506820	22q	36191154	36191906	753	6	0.667	3'UTR	9971	APOL4	80832
506820	22q	39100331	39102033	1703	6	0.833	Promoter(<=1kb)	52	APOBEC3H	164668
506820	22q	49884187	49884994	808	6	0.333	Exon(exon2of2)	22924	ALG12	79087
506820	23p	3320126	3323750	3625	10	0.6	Exon(exon5of7)	22902	MXRA5	25878
506820	23p	8170039	8170141	103	6	0.5	Promoter(1-2kb)	1126	VCX2	51480
506820	23p	35802148	35803010	863	7	0.571	5'UTR	3357	MAGEB16	139604
507045	1p	11766028	11768307	2280	6	0.833	Promoter(<=1kb)	0	C1orf167	284498
507045	1p	16058491	16059570	1080	8	0.75	Exon(exon6of7)	6168	CLCNKB	1188
507045	1p	18481042	18482217	1176	12	0.75	Promoter(<=1kb)	60	KLHDC7A	127707
507045	1p	23874604	23875430	827	8	0.5	Exon(exon2of2)	-6310	FUCA1	2517
507045	1p	32695043	32696011	969	6	0.5	5'UTR	6745	SYNC	81493
507045	1p	40067594	40067675	82	6	0	Promoter(<=1kb)	324	CAP1	10487
507045	1p	89186388	89186419	32	9	0.556	Promoter(<=1kb)	107	GBP4	115361
507045	1q	152213286	152213347	62	8	0.5	Exon(exon3of3)	10846	HRNR	388697
507045	1q	152219233	152221375	2143	16	0.75	Promoter(2-3kb)	2818	HRNR	388697
507045	1q	158765805	158766655	851	6	0.5	Promoter(<=1kb)	47	OR6N1	128372
507045	1q	169542317	169542882	566	6	0.167	Exon(exon13of25)	-26572	F5	2153
507045	1q	223393517	223394466	950	6	0.667	Promoter(<=1kb)	102	CCDC185	164127
507045	1q	226735683	226737239	1557	8	0.875	Promoter(<=1kb)	219	ITPKB	3707
507045	1q	228315976	228317998	2023	6	0.333	Exon(exon50of81)	6492	OBSCN	84033
507045	1q	228371172	228372999	1828	8	0.375	Promoter(<=1kb)	0	OBSCN	84033
507045	1q	232805117	232806800	1684	6	0.5	Promoter(<=1kb)	225	MAP10	54627
507045	1q	247841312	247841582	271	6	0.833	Promoter(<=1kb)	314	OR11L1	391189
507045	1q	247949325	247949738	414	10	0.3	Promoter(<=1kb)	467	OR2L8	391190
507045	1q	248273309	248273700	392	8	0.5	Promoter(<=1kb)	136	OR2T33	391195
507045	1q	248294677	248295415	739	6	0.667	Promoter(<=1kb)	185	OR2T12	127064
507045	2p	48580657	48582454	1798	7	0.571	Promoter(<=1kb)	0	STON1	11037
507045	2q	97547464	97547725	262	6	0.333	3'UTR	-35855	ANKRD36B	57730
507045	2q	130914528	130916898	2371	9	0.556	Promoter(<=1kb)	0	ARHGEF4	50649
507045	2q	132783061	132785001	1941	9	0.444	Promoter(1-2kb)	-1038	NCKAP5	344148
507045	2q	167246794	167251847	5054	13	0.692	Promoter(<=1kb)	0	XIRP2	129446
507045	2q	178739433	178741811	2379	6	0.5	Exon(exon45of191)	26014	TTN	7273
507045	2q	185789865	185794632	4768	10	0.8	Promoter(<=1kb)	0	FSIP2	401024
507045	3p	75736880	75739243	2364	46	0.609	Promoter(<=1kb)	0	MIR4273	100422955
507045	3q	98264413	98265098	686	7	0.571	Promoter(<=1kb)	128	OR5H6	79295
507045	3q	194341097	194342571	1475	7	0.714	Exon(exon2of2)	8747	CPN2	1370
507045	4p	5988383	5989749	1367	8	0.5	Promoter(<=1kb)	0	C4orf50	389197
507045	4p	6300792	6302360	1569	6	0.833	Exon(exon8of8)	6021	WFS1	7466
507045	4p	7433331	7434512	1182	7	0.857	Promoter(<=1kb)	418	PSAPL1	768239
507045	4p	8227004	8228508	1505	8	0.125	Promoter(<=1kb)	-24	SH3TC1	54436
507045	4p	10443803	10446224	2422	6	0.333	Exon(exon3of3)	10952	ZNF518B	85460

507045	4q	121036404	121037542	1139	6	0.333	Promoter(1-2kb)	1442	NDNF	79625
507045	4q	185458217	185460011	1795	8	0.625	Promoter(<=1kb)	0	CCDC110	256309
507045	5q	79731782	79734523	2742	13	0.308	Exon(exon2of13)	-3619	CMYA5	202333
507045	5q	83537326	83539905	2580	7	0.429	Promoter(1-2kb)	1712	VCAN	1462
507045	5q	140848579	140850786	2208	7	0.571	Promoter(<=1kb)	807	PCDHA9	9752
507045	5q	141174000	141175025	1026	7	0.714	Promoter(1-2kb)	1356	PCDHB7	56129
507045	5q	141178631	141180333	1703	6	0.833	Promoter(<=1kb)	841	PCDHB8	56128
507045	5q	141183999	141184688	690	7	0.857	Promoter(2-3kb)	-2473	PCDHB9	56127
507045	5q	141187109	141189425	2317	6	0.5	Promoter(<=1kb)	0	PCDHB9	56127
507045	5q	141955356	141957660	2305	6	0.667	Promoter(<=1kb)	-668	RNF14	9604
507045	5q	148826877	148828070	1194	6	1	Promoter(1-2kb)	1632	ADRB2	154
507045	5q	151521550	151522069	520	6	0.667	Promoter(<=1kb)	79	MIR6499	102465246
507045	6p	16327099	16327684	586	6	0.333	Exon(exon8of9)	37154	GMPR	2766
507045	6p	26370344	26370479	136	6	0.5	Promoter(<=1kb)	0	BTN3A2	11118
507045	6p	46858771	46859389	619	6	0.5	Exon(exon17of21)	3915	ADGRF5	221395
507045	6q	159233455	159234370	916	10	0.5	Exon(exon11of23)	15158	FNDC1	84624
507045	7p	1091758	1093669	1912	8	1	3'UTR	3645	GPER1	2852
507045	7p	38353718	38353991	274	9	0.667	Exon(exon2of2)	3699	TRG-AS1	100506776
507045	7q	100958721	100960873	2153	56	0.464	Promoter(<=1kb)	756	MUC3A	4584
507045	7q	100991195	100992398	1204	7	0.571	Exon(exon5of15)	-20656	MUC12	10071
507045	7q	100995575	100995785	211	7	0.714	Exon(exon5of15)	-17269	MUC12	10071
507045	7q	101004439	101004836	398	6	0.833	Exon(exon5of15)	-8218	MUC12	10071
507045	7q	101034361	101041369	7009	40	0.55	Promoter(2-3kb)	-2342	MUC17	140453
507045	8p	8376561	8377198	638	6	1	Exon(exon2of5)	4549	PRAG1	157285
507045	8p	10607245	10610662	3418	11	0.727	Exon(exon4of4)	44481	RP1L1	94137
507045	8p	11331234	11332082	849	6	0.667	Promoter(<=1kb)	346	SLC35G5	83650
507045	8p	12137448	12138641	1194	7	0.857	Promoter(<=1kb)	436	USP17L2	377630
507045	8p	13021128	13022030	903	9	0.222	Exon(exon5of5)	9115	TRMT9B	57604
507045	9p	39078723	39078854	132	7	0.857	Exon(exon22of24)	7294	CNTNAP3	79937
507045	9q	76705724	76707804	2081	6	0.5	Promoter(<=1kb)	666	PCA3	50652
507045	9q	104598641	104599361	721	8	0.625	Promoter(<=1kb)	52	OR13C5	138799
507045	9q	122553263	122554071	809	7	0.429	Promoter(<=1kb)	93	OR1N2	138882
507045	9q	122628595	122629398	804	7	0.429	Promoter(<=1kb)	175	OR1B1	347169
507045	9q	122749914	122750547	634	6	0.833	Promoter(<=1kb)	174	OR1L6	392390
507045	9q	133255635	133256264	630	7	1	3'UTR	18950	ABO	28
507045	10q	46549378	46550723	1346	27	0.667	Exon(exon3of3)	4807	GPRIN2	9721
507045	10q	128102594	128106296	3703	8	0.625	Promoter(<=1kb)	0	MKI67	4288
507045	11p	244106	244197	92	8	0.5	Promoter(<=1kb)	-232	PSMD13	5719
507045	11p	1194354	1196902	2549	7	0.571	Exon(exon34of49)	-26164	MUC5B	727897
507045	11p	1246095	1247378	1284	6	0.333	Promoter(2-3kb)	2298	MUC5B-AS1	112577518
507045	11p	5046754	5047432	679	7	0.571	Promoter(<=1kb)	228	OR52J3	119679
507045	11p	5177978	5178478	501	6	0.167	Promoter(<=1kb)	186	OR52Z1	283110
507045	11p	5323451	5324256	806	7	0.571	Promoter(<=1kb)	41	OR51B2	79345
507045	11p	5389704	5390350	647	6	0.5	Promoter(<=1kb)	327	OR51M1	390059
507045	11p	5581045	5581738	694	8	0.375	Promoter(<=1kb)	168	OR52B6	340980
507045	11p	5841302	5841883	582	9	0.333	Promoter(<=1kb)	14	OR52E6	390078
507045	11p	11351961	11352736	776	7	0.143	Promoter(<=1kb)	514	CSNK2A3	283106
507045	11p	12293639	12294368	730	7	0.857	Exon(exon29of35)	6739	MICALCL	84953
507045	11p	34916266	34916763	498	6	0.667	Promoter(<=1kb)	0	APIP	51074
507045	11q	58402523	58403265	743	8	0.5	Promoter(<=1kb)	144	OR5B3	441608
507045	11q	64315818	64315856	39	6	0.833	Promoter(1-2kb)	1485	TRMT112	51504
507045	11q	66560202	66562261	2060	6	0.333	Exon(exon14of21)	3873	CTSF	8722
507045	11q	85724687	85725825	1139	6	0.5	Promoter(<=1kb)	0	SYTL2	54843
507045	11q	123906790	123907324	535	6	0.667	Promoter(<=1kb)	644	OR8D4	338662
507045	11q	123943088	123943782	695	7	0.571	Promoter(<=1kb)	91	OR6T1	219874
507045	11q	124015601	124016477	877	8	0.25	Promoter(<=1kb)	26	OR10G4	390264
507045	11q	124023038	124023849	812	9	0.444	Promoter(<=1kb)	25	OR10G9	219870
507045	11q	124038392	124038988	597	7	0.857	Promoter(<=1kb)	13	OR10G7	390265
507045	12p	4626568	4628549	1982	11	0.455	Exon(exon5of6)	14051	DYRK4	8798
507045	12p	6453119	6453670	552	7	0.714	Promoter(<=1kb)	633	TAPBPL	55080
507045	12q	49795290	49796884	1595	6	0.5	Promoter(<=1kb)	118	NCKAP5L	57701
507045	12q	52316096	52317765	1670	6	0.667	Exon(exon4of9)	3633	KRT83	3889
507045	13q	25096870	25097231	362	11	0.545	Promoter(1-2kb)	1002	PABPC3	5042
507045	13q	102732474	102733933	1460	6	0.333	Exon(exon4of4)	25139	CCDC168	643677
507045	14q	19975713	19976448	736	9	0.444	Promoter(<=1kb)	269	OR4K15	81127
507045	14q	20060048	20060884	837	8	0.625	Promoter(<=1kb)	3	OR4L1	122742
507045	14q	21634137	21634644	508	7	0.429	Promoter(<=1kb)	296	OR10G2	26534
507045	14q	70457532	70458540	1009	10	0.3	Exon(exon2of2)	5358	ADAM21	8747
507045	15q	23440160	23442067	1908	11	0.636	5'UTR	5167	GOLGA6L2	283685
507045	15q	40621642	40623696	2055	6	0.333	Promoter(<=1kb)	0	KNL1	57082
507045	15q	79456261	79457965	1705	6	0.5	Promoter(<=1kb)	165	MINAR1	23251
507045	15q	85579423	85581800	2378	14	0.571	Promoter(1-2kb)	-1110	AKAP13	11214
507045	15q	99129423	99132517	3095	6	0.333	Exon(exon4of5)	7225	TTC23	64927
507045	16p	669592	672548	2957	6	0.5	Promoter(<=1kb)	0	RHOT2	89941
507045	16p	1486148	1488463	2316	12	0.667	Promoter(<=1kb)	4	PTX4	390667
507045	16p	27362551	27363079	529	6	0.167	3'UTR	7203	IL4R	3566
507045	16q	88428539	88429600	1062	6	0.333	Exon(exon3of3)	-23680	ZFPM1	161882

507045	16q	89100686	89101050	365	7	0.571	Promoter(<=1kb)	24	ACSF3	197322
507045	16q	89226863	89228289	1427	8	0.625	Promoter(2-3kb)	2229	ZNF778	197320
507045	17p	744946	746966	2021	6	1	3'UTR	5072	GEMIN4	50628
507045	17p	21300581	21300954	374	8	0.75	3'UTR	9112	MAP2K3	5606
507045	17p	21415470	21416370	901	6	1	Exon(exon3of3)	10334	KCNJ12	3768
507045	17q	53823368	53824891	1524	6	0.833	Promoter(<=1kb)	441	KIF2B	84643
507045	17q	76293419	76294016	598	6	0.5	Promoter(2-3kb)	-2167	QRICH2	84074
507045	17q	81645135	81645417	283	6	0.333	Promoter(2-3kb)	2722	TSPAN10	83882
507045	18p	11609904	11610491	588	7	0.714	Promoter(<=1kb)	308	SLC35G4	646000
507045	18p	14542649	14543140	492	7	0.571	Promoter(<=1kb)	6	POTEC	388468
507045	19p	1004711	1005532	822	7	0.571	Exon(exon3of9)	4292	GRIN3B	116444
507045	19p	4510548	4513547	3000	21	0.429	Exon(exon3of6)	4157	PLIN4	729359
507045	19p	5455600	5456439	840	6	0.5	Promoter(<=1kb)	183	ZNRF4	148066
507045	19p	8937644	8939234	1591	6	0.833	Exon(exon5of84)	-41554	MUC16	94025
507045	19p	8946306	8951868	5563	18	0.667	Exon(exon3of84)	29474	MUC16	94025
507045	19p	8959116	8962299	3184	12	0.583	Exon(exon3of84)	19043	MUC16	94025
507045	19p	8963397	8967127	3731	21	0.667	Exon(exon3of84)	14215	MUC16	94025
507045	19p	8972751	8978096	5346	15	0.533	Exon(exon1of84)	3246	MUC16	94025
507045	19p	12430157	12432437	2281	9	0.333	3'UTR	8584	ZNF443	10224
507045	19p	15087213	15088040	828	10	0.3	Promoter(<=1kb)	233	OR1I1	126370
507045	19p	17282085	17284246	2162	8	0.5	Promoter(<=1kb)	0	ANKLE1	126549
507045	19p	18264798	18267409	2612	12	0.583	5'UTR	7002	IQCN	80726
507045	19p	21971930	21974500	2571	7	0.714	Exon(exon4of4)	14408	ZNF208	7757
507045	19p	23743906	23745300	1395	6	0.333	Exon(exon4of4)	13537	ZNF681	148213
507045	19q	36996730	36997597	868	10	0.6	Exon(exon10of10)	5677	ZNF568	374900
507045	19q	39886005	39886439	435	6	0.333	Promoter(<=1kb)	853	FCGBP	8857
507045	19q	40880231	40880622	392	6	0.333	Promoter(<=1kb)	-141	CYP2A7	1549
507045	19q	43846955	43848536	1582	6	0.833	3'UTR	13450	ZNF283	284349
507045	19q	43913423	43914878	1456	8	0.5	Exon(exon10of10)	4861	ZNF45	7596
507045	19q	44106512	44108078	1567	7	0	Exon(exon6of6)	-4103	ZNF225	7768
507045	19q	44327836	44329698	1863	6	0.5	Exon(exon4of4)	-22790	ZNF235	9310
507045	19q	51745958	51746963	1006	6	0.5	Exon(exon3of3)	3848	FPR1	2357
507045	19q	52437918	52439242	1325	7	0.429	Exon(exon4of4)	6504	ZNF534	147658
507045	19q	55517821	55518642	822	8	1	Exon(exon14of14)	17661	SBK2	646643
507045	19q	55911888	55913166	1279	7	0.714	Exon(exon5of12)	19145	NLRP13	126204
507045	20p	5922677	5923382	706	6	0.5	Exon(exon4of5)	7179	CHGB	1114
507045	20q	63349752	63350772	1021	6	0.5	3'UTR	3794	CHRNA4	1137
507045	20q	63561666	63565531	3866	12	0.667	Promoter(<=1kb)	-61	HELZ2	85441
507045	21q	26843740	26844859	1120	6	0.667	Promoter(<=1kb)	0	ADAMTS1	9510
507045	21q	44600627	44601692	1066	9	0.778	Promoter(<=1kb)	30	KRTAP10-7	386675
507045	22q	22352950	22353298	349	13	0.538	Exon(exon1of2)	30478	BMS1P20	96610
507045	22q	36191154	36191906	753	6	0.667	3'UTR	9971	APOL4	80832
507045	22q	39100331	39102033	1703	6	0.833	Promoter(<=1kb)	52	APOBEC3H	164668
507045	22q	49883704	49884994	1291	6	0.167	Exon(exon2of2)	22924	ALG12	79087
507045	23p	8170039	8170141	103	6	0.5	Promoter(1-2kb)	1126	VCX2	51480
507045	23p	35802148	35803010	863	7	0.571	5'UTR	3357	MAGEB16	139604
507045	23q	136347344	136349199	1856	6	0.333	Exon(exon6of26)	41323	ADGRG4	139378
507111	1p	16048038	16049824	1787	6	0.5	Promoter(<=1kb)	0	CLCNKB	1188
507111	1p	16058512	16060000	1489	8	0.75	Exon(exon5of7)	6189	CLCNKB	1188
507111	1p	18481403	18482217	815	6	0.667	Promoter(<=1kb)	421	KLHDC7A	127707
507111	1p	23874604	23875430	827	8	0.5	Exon(exon2of2)	-6310	FUCA1	2517
507111	1p	40067594	40067675	82	6	0	Promoter(<=1kb)	324	CAP1	10487
507111	1p	62273232	62275080	1849	7	0.429	Promoter(<=1kb)	-441	KANK4	163782
507111	1p	89186388	89186419	32	9	0.556	Promoter(<=1kb)	107	GBP4	115361
507111	1q	152218469	152221375	2907	14	0.643	Promoter(2-3kb)	2818	HRNR	388697
507111	1q	152302822	152313891	11070	37	0.595	Promoter(<=1kb)	0	FLG-AS1	339400
507111	1q	156669844	156670886	1043	6	1	Exon(exon4of4)	6521	NES	10763
507111	1q	228315976	228318049	2074	10	0.7	Exon(exon50of81)	6492	OBSCN	84033
507111	1q	247841312	247841582	271	6	0.833	Promoter(<=1kb)	314	OR11L1	391189
507111	1q	247949443	247949738	296	9	0.333	Promoter(<=1kb)	585	OR2L8	391190
507111	2p	48580657	48582454	1798	7	0.571	Promoter(<=1kb)	0	STON1	11037
507111	2q	132783534	132785001	1468	7	0.429	Promoter(1-2kb)	-1511	NCKAP5	344148
507111	2q	178739433	178741811	2379	6	0.5	Exon(exon45of191)	26014	TTN	7273
507111	2q	184936178	184937636	1459	6	0.333	Exon(exon4of4)	69813	ZNF804A	91752
507111	2q	185789865	185794632	4768	10	0.8	Promoter(<=1kb)	0	FSIP2	401024
507111	2q	217847583	217848559	977	6	0.833	Exon(exon19of33)	-5423	TNS1	7145
507111	3p	31989532	31990905	1374	6	0.333	Exon(exon2of2)	7761	ZNF860	344787
507111	3p	75736929	75738859	1931	10	0.6	Promoter(<=1kb)	0	MIR4273	100422955
507111	4p	5988383	5989749	1367	7	0.571	Promoter(<=1kb)	0	C4orf50	389197
507111	4p	6300792	6302360	1569	7	0.857	Exon(exon8of8)	6021	WFS1	7466
507111	4p	10443803	10446224	2422	7	0.429	Exon(exon3of3)	10952	ZNF518B	85460
507111	4q	186619481	186621582	2102	6	0.333	Exon(exon10of27)	-9411	FAT1	2195
507111	5p	795818	796261	444	7	0.714	3'UTR	4884	ZDHHC11	79844
507111	5q	79728956	79730716	1761	7	0.286	Exon(exon2of13)	-7426	CMYA5	202333
507111	5q	79731782	79734523	2742	13	0.308	Exon(exon2of13)	-3619	CMYA5	202333
507111	5q	140807352	140808990	1639	10	0.7	Promoter(<=1kb)	271	PCDHA4	56144
507111	5q	141100771	141102758	1988	11	0.273	Promoter(<=1kb)	298	PCDHB3	56132

507111	5q	141174000	141175025	1026	7	0.714	Promoter(1-2kb)	1356	PCDHB7	56129
507111	5q	141178631	141180333	1703	6	0.833	Promoter(<=1kb)	841	PCDHB8	56128
507111	5q	141183999	141184688	690	6	0.833	Promoter(2-3kb)	-2473	PCDHB9	56127
507111	5q	141192663	141194472	1810	6	1	Promoter(<=1kb)	310	PCDHB10	56126
507111	5q	141955676	141957660	1985	6	0.667	Promoter(<=1kb)	-668	RNF14	9604
507111	5q	148826877	148828070	1194	6	1	Promoter(1-2kb)	1632	ADRB2	154
507111	6p	1312733	1313717	985	6	0.5	Promoter(<=1kb)	635	FOXQ1	94234
507111	6p	16327099	16327684	586	6	0.333	Exon(exon8of9)	37154	GMPR	2766
507111	6p	46858771	46859389	619	6	0.5	Exon(exon17of21)	3915	ADGRF5	221395
507111	6q	159233455	159234370	916	10	0.5	Exon(exon11of23)	15158	FNDC1	84624
507111	7p	6330446	6330944	499	6	1	Exon(exon2of2)	7749	FAM220A	84792
507111	7p	12369637	12370736	1100	6	0.667	3'UTR	-13307	VWDE	221806
507111	7q	64991278	64992758	1481	6	0.667	Promoter(<=1kb)	-242	ZNF117	51351
507111	7q	89334686	89336565	1880	7	0.571	Exon(exon4of4)	116532	ZNF804B	219578
507111	7q	100958721	100960873	2153	59	0.441	Promoter(<=1kb)	756	MUC3A	4584
507111	7q	100991195	100993127	1933	8	0.625	Exon(exon5of15)	-19927	MUC12	10071
507111	7q	100995575	100995785	211	6	0.833	Exon(exon5of15)	-17269	MUC12	10071
507111	8p	8376561	8377198	638	6	1	Exon(exon2of5)	4549	PRAG1	157285
507111	8p	10607245	10608261	1017	7	0.571	Exon(exon4of4)	46882	RP1L1	94137
507111	8p	10609650	10612307	2658	10	0.7	Exon(exon4of4)	42836	RP1L1	94137
507111	8p	11331234	11332082	849	6	0.667	Promoter(<=1kb)	346	SLC35G5	83650
507111	8p	12132686	12133940	1255	8	0.75	Promoter(<=1kb)	498	USP17L7	392197
507111	8p	12137448	12138752	1305	7	0.857	Promoter(<=1kb)	325	USP17L2	377630
507111	8q	123651873	123652634	762	6	0.333	Promoter(<=1kb)	316	KLHL38	340359
507111	8q	141466455	141467514	1060	8	0.75	3'UTR	29245	MROH5	389690
507111	9p	39078723	39078846	124	6	0.667	Exon(exon22of24)	7302	CNTNAP3	79937
507111	9q	70535957	70537247	1291	6	0.667	Exon(exon25of25)	-121333	KLF9	687
507111	9q	76705724	76707804	2081	7	0.571	Promoter(<=1kb)	666	PCA3	50652
507111	9q	104598641	104599361	721	8	0.625	Promoter(<=1kb)	52	OR13C5	138799
507111	9q	122553278	122554071	794	7	0.429	Promoter(<=1kb)	108	OR1N2	138882
507111	9q	122628595	122629398	804	6	0.333	Promoter(<=1kb)	175	OR1B1	347169
507111	9q	122749914	122750547	634	6	0.833	Promoter(<=1kb)	174	OR1L6	392390
507111	9q	135484803	135487213	2411	8	0.25	Promoter(1-2kb)	1440	PPP1R26	9858
507111	9q	135547960	135548795	836	6	0.667	Promoter(1-2kb)	1805	OBP2A	29991
507111	10q	46549378	46550723	1346	26	0.654	Exon(exon3of3)	4807	GPRIN2	9721
507111	10q	49323169	49325572	2404	8	0.5	Exon(exon3of3)	23895	C10orf71	118461
507111	10q	122084988	122087840	2853	8	0.75	Exon(exon4of23)	-25190	TACC2	10579
507111	10q	128102594	128105328	2735	7	0.714	Promoter(<=1kb)	0	MKI67	4288
507111	11p	244106	244197	92	8	0.5	Promoter(<=1kb)	-232	PSMD13	5719
507111	11p	5177978	5178478	501	6	0.167	Promoter(<=1kb)	186	OR52Z1	283110
507111	11p	5323542	5324256	715	6	0.5	Promoter(<=1kb)	41	OR51B2	79345
507111	11p	5351521	5352416	896	16	0.438	Promoter(<=1kb)	13	OR51B6	390058
507111	11p	5389704	5390350	647	7	0.429	Promoter(<=1kb)	327	OR51M1	390059
507111	11p	5422212	5423123	912	10	0.7	Promoter(<=1kb)	101	OR51Q1	390061
507111	11p	5581045	5581738	694	8	0.375	Promoter(<=1kb)	168	OR52B6	340980
507111	11p	5884818	5885061	244	6	0.333	Promoter(<=1kb)	547	OR52E4	390081
507111	11p	11351961	11352736	776	9	0.222	Promoter(<=1kb)	514	CSNK2A3	283106
507111	11p	12293639	12294368	730	6	0.833	Exon(exon29of35)	6739	MICALCL	84953
507111	11p	34916266	34916763	498	6	0.667	Promoter(<=1kb)	0	APIP	51074
507111	11q	58031251	58031899	649	6	0.667	Promoter(<=1kb)	321	OR6Q1	219952
507111	11q	58402523	58403265	743	8	0.5	Promoter(<=1kb)	144	OR5B3	441608
507111	11q	82732630	82733184	555	6	0.833	Promoter(<=1kb)	680	FAM181B	220382
507111	11q	85724687	85725825	1139	6	0.5	Promoter(<=1kb)	0	SYTL2	54843
507111	11q	93697503	93700096	2594	12	0.667	Promoter(<=1kb)	0	CEP295	85459
507111	11q	123906790	123907324	535	6	0.667	Promoter(<=1kb)	644	OR8D4	338662
507111	11q	123943088	123943782	695	7	0.571	Promoter(<=1kb)	91	OR6T1	219874
507111	11q	124015601	124016477	877	8	0.25	Promoter(<=1kb)	26	OR10G4	390264
507111	11q	124023038	124023849	812	9	0.444	Promoter(<=1kb)	25	OR10G9	219870
507111	11q	124382526	124383285	760	8	0.625	Promoter(<=1kb)	58	OR8B2	26595
507111	12p	4626571	4628549	1979	9	0.444	Exon(exon5of6)	14054	DYRK4	8798
507111	12p	6453119	6453670	552	6	0.667	Promoter(<=1kb)	633	TAPBPL	55080
507111	13q	25096870	25097182	313	14	0.429	Promoter(1-2kb)	1002	PABPC3	5042
507111	13q	49668389	49668431	43	6	0.167	Exon(exon3of3)	23007	EBPL	84650
507111	13q	102732474	102733933	1460	6	0.333	Exon(exon4of4)	25139	CCDC168	643677
507111	14q	20060048	20060884	837	10	0.6	Promoter(<=1kb)	3	OR4L1	122742
507111	14q	21634137	21634589	453	9	0.556	Promoter(<=1kb)	351	OR10G2	26534
507111	14q	70457520	70458540	1021	14	0.429	Exon(exon2of2)	5346	ADAM21	8747
507111	14q	104175275	104177810	2536	7	0.429	Exon(exon12of15)	36235	KIF26A	26153
507111	14q	104947943	104949883	1941	6	0.5	Exon(exon6of6)	5519	AHNAK2	113146
507111	15q	23439979	23442067	2089	14	0.5	5'UTR	5167	GOLGA6L2	283685
507111	15q	85579423	85582073	2651	15	0.6	Promoter(<=1kb)	-837	AKAP13	11214
507111	15q	88857108	88859365	2258	6	0	Exon(exon12of18)	9865	ACAN	176
507111	15q	99129423	99132517	3095	6	0.333	Exon(exon4of5)	7225	TTC23	64927
507111	16p	1228744	1229622	879	7	0.714	Promoter(<=1kb)	540	TPSB2	64499
507111	16p	1486371	1488463	2093	8	0.75	Promoter(<=1kb)	4	PTX4	390667
507111	16p	4207130	4207728	599	6	0.333	Exon(exon2of7)	32015	SRL	6345
507111	16q	74391650	74392004	355	8	0.625	Exon(exon7of7)	13772	NPIPB15	440348

507111	16q	88714632	88717113	2482	7	0.429	Promoter(<=1kb)	0	MIR4722	100616167
507111	16q	89226863	89228289	1427	7	0.571	Promoter(2-3kb)	2229	ZNF778	197320
507111	17p	21300581	21300954	374	8	0.75	3'UTR	9112	MAP2K3	5606
507111	17q	41586466	41586829	364	6	0.833	Promoter(<=1kb)	66	KRT14	3861
507111	17q	53823368	53824891	1524	6	0.833	Promoter(<=1kb)	441	KIF2B	84643
507111	17q	76293419	76294016	598	6	0.5	Promoter(2-3kb)	-2167	QRICH2	84074
507111	17q	81645135	81645515	381	7	0.429	Promoter(2-3kb)	2722	TSPAN10	83882
507111	18p	11609728	11610350	623	8	0.875	Promoter(<=1kb)	132	SLC35G4	646000
507111	18q	58535186	58537515	2330	9	0.333	Promoter(<=1kb)	0	ALPK2	115701
507111	19p	1004688	1005532	845	6	0.5	Exon(exon3of9)	4269	GRIN3B	116444
507111	19p	4510548	4513547	3000	20	0.45	Exon(exon3of6)	4157	PLIN4	729359
507111	19p	5455600	5456439	840	7	0.571	Promoter(<=1kb)	183	ZNRF4	148066
507111	19p	8333830	8334965	1136	6	0.667	Promoter(<=1kb)	0	KANK3	256949
507111	19p	8948231	8953259	5029	16	0.562	Exon(exon3of84)	28083	MUC16	94025
507111	19p	8959403	8962066	2664	8	0.5	Exon(exon3of84)	19276	MUC16	94025
507111	19p	8964274	8967127	2854	22	0.682	Exon(exon3of84)	14215	MUC16	94025
507111	19p	12430718	12432437	1720	8	0.375	3'UTR	8584	ZNF443	10224
507111	19p	15087213	15088040	828	11	0.273	Promoter(<=1kb)	233	OR1I1	126370
507111	19p	18264753	18267409	2657	8	0.5	5'UTR	7002	IQCN	80726
507111	19p	23743906	23745300	1395	6	0.333	Exon(exon4of4)	13537	ZNF681	148213
507111	19q	39877222	39877880	659	6	0.5	Exon(exon20of28)	9412	FCGBP	8857
507111	19q	43204774	43205504	731	6	0.833	Promoter(<=1kb)	47	PSG4	5672
507111	19q	43966037	43967171	1135	6	0.167	Promoter(<=1kb)	-691	ZNF155	7711
507111	19q	52437918	52439242	1325	7	0.429	Exon(exon4of4)	6504	ZNF534	147658
507111	19q	52766233	52767718	1486	7	0.714	Exon(exon5of9)	18982	ZNF600	162966
507111	19q	55911888	55913077	1190	6	0.667	Exon(exon5of12)	19234	NLRP13	126204
507111	20p	5922421	5923394	974	8	0.5	Exon(exon4of5)	6923	CHGB	1114
507111	20p	20052354	20052736	383	6	0	Promoter(<=1kb)	0	CFAP61	26074
507111	20q	63561666	63565737	4072	12	0.667	Promoter(<=1kb)	-61	HELZ2	85441
507111	22q	22352950	22353380	431	16	0.5	Exon(exon1of2)	30478	BMS1P20	96610
507111	22q	36191154	36191906	753	7	0.714	3'UTR	9971	APOL4	80832
507111	23p	3320126	3323750	3625	10	0.6	Exon(exon5of7)	22902	MXRA5	25878
507111	23p	8170039	8170141	103	6	0.5	Promoter(1-2kb)	1126	VCX2	51480
507111	23p	35802148	35803010	863	7	0.571	5'UTR	3357	MAGEB16	139604
507111	23q	115190034	115192586	2553	6	0.833	Promoter(<=1kb)	607	RBMXL3	139804
507427	1p	12029085	12030097	1013	6	0.833	Promoter(<=1kb)	0	MIR6729	102466982
507427	1p	16048038	16049824	1787	6	0.5	Promoter(<=1kb)	0	CLCNKB	1188
507427	1p	16058491	16060000	1510	10	0.9	Exon(exon5of7)	6168	CLCNKB	1188
507427	1p	18481403	18482217	815	7	0.714	Promoter(<=1kb)	421	KLHDC7A	127707
507427	1p	40067594	40067675	82	6	0	Promoter(<=1kb)	324	CAP1	10487
507427	1p	74469879	74472144	2266	6	0.333	Exon(exon5of5)	30814	FPGT-TNNI3K	100526835
507427	1q	152213274	152213316	43	7	0.571	Exon(exon3of3)	10877	HRNR	388697
507427	1q	152218469	152221375	2907	16	0.688	Promoter(2-3kb)	2818	HRNR	388697
507427	1q	152302977	152313891	10915	40	0.625	Promoter(<=1kb)	0	FLG-AS1	339400
507427	1q	169542317	169542882	566	6	0.167	Exon(exon13of25)	-26572	F5	2153
507427	1q	201206099	201209342	3244	10	0.6	Promoter(1-2kb)	1017	IGFN1	91156
507427	1q	201210956	201212792	1837	6	0.333	Promoter(<=1kb)	0	IGFN1	91156
507427	1q	228315976	228318049	2074	8	0.625	Exon(exon50of81)	6492	OBSCN	84033
507427	1q	247841312	247841582	271	6	0.833	Promoter(<=1kb)	314	OR11L1	391189
507427	1q	247895745	247896410	666	7	0.571	Promoter(<=1kb)	158	OR2W3	343171
507427	1q	248294677	248295458	782	9	0.667	Promoter(<=1kb)	142	OR2T12	127064
507427	1q	248681658	248682198	541	7	0.571	Promoter(<=1kb)	130	OR14I1	401994
507427	2p	29002636	29003646	1011	6	0.333	Exon(exon5of20)	-10821	TOGARAM2	165186
507427	2q	102351547	102351902	356	7	0.429	Exon(exon11of11)	-4027	IL18R1	8809
507427	2q	130193975	130194376	402	6	0.667	Exon(exon4of5)	4063	TUBA3E	112714
507427	2q	167246794	167248478	1685	7	0.714	Promoter(<=1kb)	-204	XIRP2	129446
507427	2q	178739433	178741811	2379	6	0.5	Exon(exon45of191)	26014	TTN	7273
507427	2q	184936178	184937636	1459	6	0.333	Exon(exon4of4)	69813	ZNF804A	91752
507427	2q	185789865	185794632	4768	10	0.8	Promoter(<=1kb)	0	FSIP2	401024
507427	2q	185805377	185808170	2794	6	0.333	Promoter(<=1kb)	0	FSIP2	401024
507427	2q	217847583	217848559	977	6	0.833	Exon(exon19of33)	-5423	TNS1	7145
507427	3p	31989532	31990905	1374	6	0.333	Exon(exon2of2)	7761	ZNF860	344787
507427	3p	52520288	52524117	3830	9	0.444	Promoter(<=1kb)	0	STAB1	23166
507427	3p	75737230	75739007	1778	9	0.556	Promoter(<=1kb)	0	MIR4273	100422955
507427	3q	98149575	98150126	552	6	0.667	Promoter(<=1kb)	249	OR5H14	403273
507427	3q	98264413	98265098	686	7	0.571	Promoter(<=1kb)	128	OR5H6	79295
507427	3q	194341097	194342571	1475	7	0.714	Exon(exon2of2)	8747	CPN2	1370
507427	4p	5988383	5989749	1367	7	0.571	Promoter(<=1kb)	0	C4orf50	389197
507427	4p	6300792	6302360	1569	6	0.833	Exon(exon8of8)	6021	WFS1	7466
507427	4p	10443803	10446224	2422	7	0.429	Exon(exon3of3)	10952	ZNF518B	85460
507427	4q	87811379	87811766	388	6	0.333	Exon(exon7of7)	-9645	MEPE	56955
507427	5p	795818	796237	420	8	0.625	3'UTR	4908	ZDHHC11	79844
507427	5q	83537326	83539905	2580	6	0.333	Promoter(1-2kb)	1712	VCAN	1462
507427	5q	140807352	140807737	386	6	0.833	Promoter(<=1kb)	271	PCDHA4	56144
507427	5q	141174000	141175025	1026	6	0.833	Promoter(1-2kb)	1356	PCDHB7	56129
507427	5q	141187690	141189425	1736	6	0.5	Promoter(<=1kb)	529	PCDHB9	56127
507427	5q	151565922	151568158	2237	9	0.778	Promoter(<=1kb)	786	FAT2	2196

507427	6p	46858771	46859389	619	6	0.5	Exon(exon17of21)	3915	ADGRF5	221395
507427	6q	106511549	106512572	1024	6	0.667	Promoter(<=1kb)	0	CRYBG1	202
507427	6q	159231899	159234370	2472	12	0.583	Exon(exon11of23)	13602	FNDC1	84624
507427	7p	53035678	53036385	708	6	1	Promoter(<=1kb)	45	POM121L12	285877
507427	7q	100958977	100960873	1897	47	0.489	Promoter(1-2kb)	1012	MUC3A	4584
507427	7q	100991195	100992398	1204	7	0.571	Exon(exon5of15)	-20656	MUC12	10071
507427	7q	100995575	100995785	211	7	0.714	Exon(exon5of15)	-17269	MUC12	10071
507427	7q	101003515	101004836	1322	7	0.714	Exon(exon5of15)	-8218	MUC12	10071
507427	7q	101034361	101040583	6223	32	0.469	Exon(exon3of12)	-3128	MUC17	140453
507427	8p	10607245	10610662	3418	13	0.692	Exon(exon4of4)	44481	RP1L1	94137
507427	8p	11331194	11332026	833	7	0.429	Promoter(<=1kb)	306	SLC35G5	83650
507427	8p	13021128	13022030	903	7	0.143	Exon(exon5of5)	9115	TRMT9B	57604
507427	8q	123651655	123652634	980	7	0.571	Promoter(<=1kb)	316	KLHL38	340359
507427	8q	142664564	142665970	1407	9	0.889	Exon(exon2of2)	4000	JRK	8629
507427	9p	116800	117713	914	7	0.714	Promoter(<=1kb)	491	FOXD4	2298
507427	9p	39078723	39078753	31	6	0.833	Exon(exon22of24)	7395	CNTNAP3	79937
507427	9q	76175237	76175296	60	10	0.8	Exon(exon14of14)	-13343	PCSK5	5125
507427	9q	76705724	76707804	2081	6	0.5	Promoter(<=1kb)	666	PCA3	50652
507427	9q	87886533	87888713	2181	8	0.625	Exon(exon4of4)	3656	SPATA31E1	286234
507427	9q	104504315	104505071	757	6	0.5	Promoter(<=1kb)	52	OR13F1	138805
507427	9q	104598545	104599361	817	13	0.538	Promoter(<=1kb)	52	OR13C5	138799
507427	9q	122553263	122554071	809	7	0.429	Promoter(<=1kb)	93	OR1N2	138882
507427	9q	122749914	122750547	634	6	0.833	Promoter(<=1kb)	174	OR1L6	392390
507427	9q	124855684	124856809	1126	7	0.571	Promoter(2-3kb)	2208	WDR38	401551
507427	9q	135484803	135487213	2411	8	0.25	Promoter(1-2kb)	1440	PPP1R26	9858
507427	10q	46549378	46550723	1346	28	0.679	Exon(exon3of3)	4807	GPRIN2	9721
507427	10q	49323169	49326817	3649	11	0.545	Exon(exon3of3)	23895	C10orf71	118461
507427	10q	122084988	122087840	2853	8	0.75	Exon(exon4of23)	-25190	TACC2	10579
507427	10q	128102802	128104752	1951	10	0.5	Promoter(<=1kb)	0	MKI67	4288
507427	11p	244106	244197	92	8	0.5	Promoter(<=1kb)	-232	PSMD13	5719
507427	11p	1241677	1243593	1917	6	0.333	Exon(exon31of49)	6083	MUC5B-AS1	112577518
507427	11p	1246095	1248605	2511	11	0.545	Promoter(1-2kb)	1071	MUC5B-AS1	112577518
507427	11p	1250091	1251628	1538	9	0.778	Promoter(<=1kb)	-415	MUC5B-AS1	112577518
507427	11p	5177978	5178478	501	6	0.167	Promoter(<=1kb)	186	OR52Z1	283110
507427	11p	5389704	5390350	647	7	0.429	Promoter(<=1kb)	327	OR51M1	390059
507427	11p	5402638	5403471	834	11	0.455	Promoter(<=1kb)	41	OR51J1	79470
507427	11p	5422212	5423123	912	10	0.7	Promoter(<=1kb)	101	OR51Q1	390061
507427	11p	5581045	5581738	694	8	0.375	Promoter(<=1kb)	168	OR52B6	340980
507427	11p	5884818	5885061	244	6	0.333	Promoter(<=1kb)	547	OR52E4	390081
507427	11p	11351961	11352736	776	7	0.143	Promoter(<=1kb)	514	CSNK2A3	283106
507427	11p	12293639	12294368	730	6	0.833	Exon(exon29of35)	6739	MICALCL	84953
507427	11q	55572176	55572903	728	6	0.667	Promoter(<=1kb)	48	OR4C16	219428
507427	11q	58214757	58215722	966	8	0.25	Promoter(<=1kb)	12	OR1S1	219959
507427	11q	85724687	85725825	1139	6	0.5	Promoter(<=1kb)	0	SYTL2	54843
507427	11q	123906790	123907324	535	6	0.667	Promoter(<=1kb)	644	OR8D4	338662
507427	11q	124038366	124038988	623	7	1	Promoter(<=1kb)	13	OR10G7	390265
507427	12p	4626571	4628549	1979	8	0.5	Exon(exon5of6)	14054	DYRK4	8798
507427	12p	6453119	6453670	552	6	0.667	Promoter(<=1kb)	633	TAPBPL	55080
507427	12p	8222174	8223514	1341	6	0.667	Exon(exon5of6)	4073	FAM90A1	55138
507427	12q	52316096	52317765	1670	6	0.667	Exon(exon4of9)	3633	KRT83	3889
507427	13q	25096713	25097182	470	13	0.462	Promoter(<=1kb)	845	PABPC3	5042
507427	13q	49668389	49668431	43	6	0.167	Exon(exon3of3)	23007	EBPL	84650
507427	13q	102732474	102733933	1460	6	0.333	Exon(exon4of4)	25139	CCDC168	643677
507427	14q	20060048	20060884	837	8	0.625	Promoter(<=1kb)	3	OR4L1	122742
507427	14q	21634137	21634589	453	9	0.556	Promoter(<=1kb)	351	OR10G2	26534
507427	14q	22633879	22634450	572	9	0.333	Exon(exon2of2)	32212	ABHD4	63874
507427	14q	44504986	44506403	1418	6	0.667	Promoter(<=1kb)	880	FSCB	84075
507427	14q	70457745	70458948	1204	7	0.429	Exon(exon2of2)	5571	ADAM21	8747
507427	14q	104947943	104951938	3996	13	0.308	Exon(exon6of6)	3464	AHNAK2	113146
507427	15q	23439979	23442067	2089	13	0.538	5'UTR	5167	GOLGA6L2	283685
507427	15q	52609086	52609780	695	6	0.333	Promoter(<=1kb)	-490	FAM214A	56204
507427	15q	88857878	88859365	1488	7	0.143	Exon(exon12of18)	10635	ACAN	176
507427	15q	101065940	101067308	1369	6	0.667	Promoter(1-2kb)	1524	LRRK1	79705
507427	16p	1486371	1488463	2093	9	0.778	Promoter(<=1kb)	4	PTX4	390667
507427	16p	1998795	2000191	1397	7	0.571	Promoter(<=1kb)	0	ZNF598	90850
507427	16p	4207059	4208004	946	7	0.571	Exon(exon2of7)	31739	SRL	6345
507427	16q	74391460	74392004	545	9	0.667	Exon(exon7of7)	13582	NPIPB15	440348
507427	16q	88428539	88429600	1062	6	0.333	Exon(exon3of3)	-23680	ZFPM1	161882
507427	16q	89100686	89101050	365	7	0.571	Promoter(<=1kb)	24	ACSF3	197322
507427	16q	89226863	89228390	1528	9	0.556	Promoter(2-3kb)	2229	ZNF778	197320
507427	17p	744946	746966	2021	7	1	3'UTR	5072	GEMIN4	50628
507427	17p	10638198	10641099	2902	7	0.286	Exon(exon19of41)	-8169	MYH3	4621
507427	17p	21300581	21300978	398	11	0.727	3'UTR	9112	MAP2K3	5606
507427	17q	81645135	81645607	473	7	0.286	Promoter(2-3kb)	2722	TSPAN10	83882
507427	18p	11609904	11610509	606	8	0.625	Promoter(<=1kb)	308	SLC35G4	646000
507427	19p	4510548	4513547	3000	20	0.45	Exon(exon3of6)	4157	PLIN4	729359
507427	19p	5455600	5456439	840	7	0.571	Promoter(<=1kb)	183	ZNRF4	148066

507427	19p	8948231	8951868	3638	11	0.545	Exon(exon3of84)	29474	MUC16	94025
507427	19p	15087213	15088040	828	9	0.333	Promoter(<=1kb)	233	OR1I1	126370
507427	19p	17281820	17284246	2427	9	0.556	Promoter(<=1kb)	0	ANKLE1	126549
507427	19p	18264798	18267409	2612	12	0.583	5'UTR	7002	IQCN	80726
507427	19p	21971930	21974500	2571	8	0.75	Exon(exon4of4)	14408	ZNF208	7757
507427	19p	23743906	23745300	1395	6	0.333	Exon(exon4of4)	13537	ZNF681	148213
507427	19q	39877222	39877880	659	7	0.571	Exon(exon20of28)	9412	FCGBP	8857
507427	19q	39886240	39886422	183	6	0.667	Promoter(<=1kb)	870	FCGBP	8857
507427	19q	43913423	43914878	1456	8	0.5	Exon(exon10of10)	4861	ZNF45	7596
507427	19q	43966037	43967171	1135	6	0.167	Promoter(<=1kb)	-691	ZNF155	7711
507427	19q	44106512	44108078	1567	7	0	Exon(exon6of6)	-4103	ZNF225	7768
507427	19q	51745958	51746963	1006	6	0.333	Exon(exon3of3)	3848	FPR1	2357
507427	19q	51872787	51873699	913	10	0.5	Promoter(<=1kb)	-940	ZNF577	84765
507427	19q	52437918	52439242	1325	7	0.429	Exon(exon4of4)	6504	ZNF534	147658
507427	19q	55911888	55913166	1279	7	0.714	Exon(exon5of12)	19145	NLRP13	126204
507427	19q	57639918	57641641	1724	7	0.429	3'UTR	6693	ZNF211	10520
507427	20p	5922421	5923394	974	7	0.571	Exon(exon4of5)	6923	CHGB	1114
507427	20p	20052354	20052736	383	6	0.167	Promoter(<=1kb)	0	CFAP61	26074
507427	20q	63561666	63565531	3866	12	0.667	Promoter(<=1kb)	-61	HELZ2	85441
507427	21q	44550835	44551416	582	6	0.833	Promoter(<=1kb)	89	KRTAP10-2	386679
507427	21q	44591587	44592359	773	7	0.286	Promoter(<=1kb)	146	KRTAP10-6	386674
507427	21q	44637474	44638041	568	9	0.333	Promoter(<=1kb)	118	KRTAP10-10	353333
507427	22q	22352950	22353380	431	16	0.5	Exon(exon1of2)	30478	BMS1P20	96610
507427	22q	36191154	36191906	753	7	0.571	3'UTR	9971	APOL4	80832
507427	22q	39100331	39102033	1703	6	0.833	Promoter(<=1kb)	52	APOBEC3H	164668
507427	23p	8170039	8170141	103	6	0.5	Promoter(1-2kb)	1126	VCX2	51480
507427	23q	141906066	141906385	320	9	0.556	Promoter(1-2kb)	1264	MAGEC1	9947
507544	1p	11778784	11779941	1158	7	0.714	Promoter(<=1kb)	0	C1orf167-AS1	102724659
507544	1p	12795695	12795957	263	6	0.5	Promoter(1-2kb)	1004	PRAMEF1	65121
507544	1p	12847526	12847995	470	10	0.4	Promoter(<=1kb)	730	HNRNPCL1	343069
507544	1p	12859036	12860079	1044	6	0.333	Promoter(1-2kb)	1950	PRAMEF2	65122
507544	1p	13370686	13371119	434	7	0.429	Promoter(<=1kb)	781	PRAMEF19	645414
507544	1p	16058491	16060000	1510	10	0.9	Exon(exon5of7)	6168	CLCNKB	1188
507544	1p	18481403	18483159	1757	8	0.75	Promoter(<=1kb)	421	KLHDC7A	127707
507544	1p	23874604	23875430	827	8	0.5	Exon(exon2of2)	-6310	FUCA1	2517
507544	1p	40067594	40067675	82	6	0	Promoter(<=1kb)	324	CAP1	10487
507544	1p	89186388	89186419	32	9	0.556	Promoter(<=1kb)	107	GBP4	115361
507544	1q	152303673	152313891	10219	38	0.579	Promoter(<=1kb)	0	FLG-AS1	339400
507544	1q	156669844	156670886	1043	6	1	Exon(exon4of4)	6521	NES	10763
507544	1q	158765805	158766655	851	6	0.5	Promoter(<=1kb)	47	OR6N1	128372
507544	1q	169542317	169542882	566	6	0.167	Exon(exon13of25)	-26572	F5	2153
507544	1q	197101312	197101771	460	6	0.5	Exon(exon18of28)	33373	ASPM	259266
507544	1q	201206099	201209837	3739	12	0.5	Promoter(1-2kb)	1017	IGFN1	91156
507544	1q	201210956	201212792	1837	7	0.286	Promoter(<=1kb)	0	IGFN1	91156
507544	1q	228315976	228318026	2051	6	0.5	Exon(exon50of81)	6492	OBSCN	84033
507544	1q	247841312	247841582	271	6	0.833	Promoter(<=1kb)	314	OR11L1	391189
507544	1q	247949325	247949738	414	9	0.333	Promoter(<=1kb)	467	OR2L8	391190
507544	2p	29002636	29003646	1011	6	0.333	Exon(exon5of20)	-10821	TOGARAM2	165186
507544	2q	102351547	102351902	356	7	0.429	Exon(exon11of11)	-4027	IL18R1	8809
507544	2q	167246794	167248478	1685	8	0.625	Promoter(<=1kb)	-204	XIRP2	129446
507544	2q	184936178	184937636	1459	6	0.333	Exon(exon4of4)	69813	ZNF804A	91752
507544	2q	185790999	185794632	3634	8	0.875	Promoter(<=1kb)	0	FSIP2	401024
507544	2q	185805377	185808336	2960	7	0.429	Promoter(<=1kb)	0	FSIP2	401024
507544	2q	240041845	240042131	287	6	0.167	Downstream(2-3kb)	3941	OR6B3	150681
507544	3p	31989532	31990905	1374	6	0.333	Exon(exon2of2)	7761	ZNF860	344787
507544	3p	75736929	75739007	2079	63	0.651	Promoter(<=1kb)	0	MIR4273	100422955
507544	3q	98264413	98265137	725	6	0.667	Promoter(<=1kb)	128	OR5H6	79295
507544	3q	196947388	196948102	715	6	0.5	3'UTR	3828	PIGZ	80235
507544	4p	5988383	5989749	1367	7	0.571	Promoter(<=1kb)	0	C4orf50	389197
507544	4p	6300792	6302360	1569	6	0.833	Exon(exon8of8)	6021	WFS1	7466
507544	4p	8227004	8228508	1505	8	0.125	Promoter(<=1kb)	-24	SH3TC1	54436
507544	4p	9175308	9175954	647	6	0.667	Exon(exon7of7)	4899	FAM90A26	100287045
507544	4q	154489498	154491312	1815	9	0.556	Promoter(<=1kb)	22	DCHS2	54798
507544	4q	185458217	185460011	1795	9	0.556	Promoter(<=1kb)	0	CCDC110	256309
507544	5q	79728956	79730716	1761	7	0.286	Exon(exon2of13)	-7426	CMYA5	202333
507544	5q	79731782	79734523	2742	13	0.308	Exon(exon2of13)	-3619	CMYA5	202333
507544	5q	83537326	83539905	2580	7	0.286	Promoter(1-2kb)	1712	VCAN	1462
507544	5q	140807352	140807737	386	6	0.833	Promoter(<=1kb)	271	PCDHA4	56144
507544	5q	140848579	140850786	2208	6	0.5	Promoter(<=1kb)	807	PCDHA9	9752
507544	5q	141174000	141175025	1026	6	0.833	Promoter(1-2kb)	1356	PCDHB7	56129
507544	5q	141183999	141184688	690	6	1	Promoter(2-3kb)	-2473	PCDHB9	56127
507544	5q	141955356	141957660	2305	6	0.667	Promoter(<=1kb)	-668	RNF14	9604
507544	6p	1312843	1313717	875	6	0.333	Promoter(<=1kb)	745	FOXQ1	94234
507544	6p	46858771	46859502	732	8	0.5	Exon(exon17of21)	3802	ADGRF5	221395
507544	6q	149888581	149889987	1407	6	0.667	Promoter(<=1kb)	-116	RAET1E-AS1	100652739
507544	6q	159233455	159234370	916	10	0.5	Exon(exon11of23)	15158	FNDC1	84624
507544	7q	64991278	64992758	1481	6	0.667	Promoter(<=1kb)	-242	ZNF117	51351

507544	7q	100958721	100960873	2153	56	0.464	Promoter(<=1kb)	756	MUC3A	4584
507544	7q	100991195	100992398	1204	8	0.625	Exon(exon5of15)	-20656	MUC12	10071
507544	7q	100995547	100995896	350	9	0.889	Exon(exon5of15)	-17158	MUC12	10071
507544	8p	8376561	8377198	638	6	1	Exon(exon2of5)	4549	PRAG1	157285
507544	8p	10607375	10608261	887	6	0.5	Exon(exon4of4)	46882	RP1L1	94137
507544	8p	11331194	11332082	889	8	0.625	Promoter(<=1kb)	306	SLC35G5	83650
507544	8p	17754008	17755366	1359	6	0.5	Promoter(<=1kb)	0	MTUS1	57509
507544	8q	123651873	123652634	762	6	0.333	Promoter(<=1kb)	316	KLHL38	340359
507544	8q	141466455	141467514	1060	8	0.75	3'UTR	29245	MROH5	389690
507544	8q	143870008	143872453	2446	6	0.5	Exon(exon2of2)	6011	EPPK1	83481
507544	9q	76175237	76175296	60	9	0.778	Exon(exon14of14)	-13343	PCSK5	5125
507544	9q	76705724	76707804	2081	6	0.5	Promoter(<=1kb)	666	PCA3	50652
507544	9q	87885278	87888819	3542	13	0.692	Promoter(2-3kb)	2401	SPATA31E1	286234
507544	9q	104598545	104599361	817	12	0.583	Promoter(<=1kb)	52	OR13C5	138799
507544	9q	122553263	122554071	809	8	0.5	Promoter(<=1kb)	93	OR1N2	138882
507544	9q	122628595	122629130	536	6	0.333	Promoter(<=1kb)	443	OR1B1	347169
507544	9q	122749914	122750547	634	6	0.833	Promoter(<=1kb)	174	OR1L6	392390
507544	10q	46549378	46550723	1346	26	0.654	Exon(exon3of3)	4807	GPRIN2	9721
507544	10q	49323169	49326817	3649	11	0.545	Exon(exon3of3)	23895	C10orf71	118461
507544	10q	128103129	128106213	3085	12	0.667	Promoter(<=1kb)	-1	MKI67	4288
507544	11p	244106	244197	92	8	0.5	Promoter(<=1kb)	-232	PSMD13	5719
507544	11p	1246332	1247378	1047	7	0.429	Promoter(2-3kb)	2298	MUC5B-AS1	112577518
507544	11p	4946238	4946968	731	6	0.667	Promoter(<=1kb)	158	OR51A4	401666
507544	11p	5177978	5178478	501	6	0.167	Promoter(<=1kb)	186	OR52Z1	283110
507544	11p	5422212	5423123	912	11	0.636	Promoter(<=1kb)	101	OR51Q1	390061
507544	11p	5581045	5581738	694	9	0.333	Promoter(<=1kb)	168	OR52B6	340980
507544	11p	5841302	5841883	582	9	0.333	Promoter(<=1kb)	14	OR52E6	390078
507544	11p	5986042	5986542	501	6	1	Promoter(<=1kb)	443	OR52L1	338751
507544	11p	11351961	11352736	776	9	0.222	Promoter(<=1kb)	514	CSNK2A3	283106
507544	11p	12293639	12294368	730	6	0.833	Exon(exon29of35)	6739	MICALCL	84953
507544	11p	18173280	18173901	622	6	0.333	Promoter(<=1kb)	443	MRGPRX4	117196
507544	11q	58214757	58215722	966	8	0.25	Promoter(<=1kb)	12	OR1S1	219959
507544	11q	123906790	123907324	535	6	0.667	Promoter(<=1kb)	644	OR8D4	338662
507544	11q	124015600	124016477	878	7	0.143	Promoter(<=1kb)	25	OR10G4	390264
507544	12p	4626568	4628549	1982	11	0.455	Exon(exon5of6)	14051	DYRK4	8798
507544	12p	8222174	8223514	1341	6	0.667	Exon(exon5of6)	4073	FAM90A1	55138
507544	12q	52316096	52317765	1670	6	0.667	Exon(exon4of9)	3633	KRT83	3889
507544	13q	24434450	24435347	898	7	0.571	Exon(exon31of34)	19787	PARP4	143
507544	13q	102732474	102733933	1460	7	0.429	Exon(exon4of4)	25139	CCDC168	643677
507544	14q	20060048	20060884	837	8	0.625	Promoter(<=1kb)	3	OR4L1	122742
507544	14q	20640982	20641567	586	6	0.5	Promoter(<=1kb)	124	OR6S1	341799
507544	14q	21634137	21634589	453	9	0.556	Promoter(<=1kb)	351	OR10G2	26534
507544	14q	70457532	70458540	1009	9	0.333	Exon(exon2of2)	5358	ADAM21	8747
507544	14q	94587512	94587839	328	6	0.5	Exon(exon2of2)	-4219	SERPINA3	12
507544	15q	23439979	23442067	2089	12	0.5	5'UTR	5167	GOLGA6L2	283685
507544	15q	78766033	78766609	577	7	0.571	Promoter(<=1kb)	-920	ADAMTS7	11173
507544	15q	85579423	85581800	2378	15	0.533	Promoter(1-2kb)	-1110	AKAP13	11214
507544	15q	100569472	100570097	626	6	0.833	Promoter(<=1kb)	534	LINS1	55180
507544	15q	101065952	101067308	1357	6	0.5	Promoter(1-2kb)	1536	LRRK1	79705
507544	16p	669592	672548	2957	7	0.571	Promoter(<=1kb)	0	RHOT2	89941
507544	16p	1486322	1488463	2142	11	0.636	Promoter(<=1kb)	4	PTX4	390667
507544	16q	74391650	74392004	355	7	0.571	Exon(exon7of7)	13772	NPIPB15	440348
507544	16q	88428539	88431997	3459	13	0.462	Exon(exon3of3)	-21283	ZFPM1	161882
507544	16q	88433131	88436097	2967	6	0.667	Exon(exon3of3)	-17183	ZFPM1	161882
507544	16q	89100686	89101050	365	8	0.625	Promoter(<=1kb)	24	ACSF3	197322
507544	16q	89226863	89228419	1557	10	0.6	Promoter(2-3kb)	2229	ZNF778	197320
507544	17p	21300581	21300978	398	12	0.75	3'UTR	9112	MAP2K3	5606
507544	17q	76293419	76294016	598	6	0.5	Promoter(2-3kb)	-2167	QRICH2	84074
507544	17q	81645135	81645417	283	6	0.333	Promoter(2-3kb)	2722	TSPAN10	83882
507544	18p	11609646	11610350	705	11	0.818	Promoter(<=1kb)	50	SLC35G4	646000
507544	18q	58535186	58537515	2330	9	0.333	Promoter(<=1kb)	0	ALPK2	115701
507544	19p	4510548	4513547	3000	17	0.471	Exon(exon3of6)	4157	PLIN4	729359
507544	19p	8937644	8939234	1591	6	0.833	Exon(exon5of84)	-41554	MUC16	94025
507544	19p	8946306	8951868	5563	18	0.667	Exon(exon3of84)	29474	MUC16	94025
507544	19p	8959116	8962299	3184	12	0.583	Exon(exon3of84)	19043	MUC16	94025
507544	19p	8963397	8967127	3731	21	0.667	Exon(exon3of84)	14215	MUC16	94025
507544	19p	8972751	8978096	5346	13	0.462	Exon(exon1of84)	3246	MUC16	94025
507544	19p	12430718	12432437	1720	8	0.375	3'UTR	8584	ZNF443	10224
507544	19p	17281820	17284246	2427	9	0.556	Promoter(<=1kb)	0	ANKLE1	126549
507544	19p	18264753	18267409	2657	8	0.5	5'UTR	7002	IQCN	80726
507544	19p	21971930	21974500	2571	8	0.75	Exon(exon4of4)	14408	ZNF208	7757
507544	19q	39877222	39877880	659	6	0.5	Exon(exon20of28)	9412	FCGBP	8857
507544	19q	39886240	39886422	183	6	0.667	Promoter(<=1kb)	870	FCGBP	8857
507544	19q	40880231	40880622	392	6	0.333	Promoter(<=1kb)	-141	CYP2A7	1549
507544	19q	43913423	43914878	1456	9	0.556	Exon(exon10of10)	4861	ZNF45	7596
507544	19q	44106512	44108078	1567	7	0	Exon(exon6of6)	-4103	ZNF225	7768
507544	19q	48873985	48875925	1941	9	0.556	Promoter(1-2kb)	1564	PPP1R15A	23645

507544	19q	52437918	52439242	1325	7	0.429	Exon(exon4of4)	6504	ZNF534	147658
507544	19q	55911888	55913077	1190	6	0.667	Exon(exon5of12)	19234	NLRP13	126204
507544	20p	5922421	5923394	974	7	0.571	Exon(exon4of5)	6923	CHGB	1114
507544	20q	63561666	63567360	5695	18	0.667	Promoter(<=1kb)	-61	HELZ2	85441
507544	21q	26843740	26844859	1120	6	0.667	Promoter(<=1kb)	0	ADAMTS1	9510
507544	21q	44539410	44540035	626	6	0.667	Promoter(<=1kb)	160	KRTAP10-1	386677
507544	21q	44558207	44558709	503	6	0.5	Promoter(<=1kb)	86	KRTAP10-3	386682
507544	21q	44591429	44592404	976	10	0.2	Promoter(<=1kb)	101	KRTAP10-6	386674
507544	21q	44600627	44601692	1066	11	0.636	Promoter(<=1kb)	30	KRTAP10-7	386675
507544	22q	22352950	22353380	431	16	0.5	Exon(exon1of2)	30478	BMS1P20	96610
507544	22q	36191154	36191906	753	6	0.667	3'UTR	9971	APOL4	80832
507544	22q	37724535	37726455	1921	6	0.667	3'UTR	-19778	TRIOBP	11078
507544	22q	50503556	50505699	2144	6	0.833	Promoter(<=1kb)	0	LMF2	91289
507544	23p	8170039	8170243	205	7	0.571	Promoter(1-2kb)	1024	VCX2	51480
507544	23p	35802148	35803010	863	7	0.571	5'UTR	3357	MAGEB16	139604
507694	1p	16048038	16049824	1787	6	0.5	Promoter(<=1kb)	0	CLCNKB	1188
507694	1p	16058491	16060000	1510	10	0.9	Exon(exon5of7)	6168	CLCNKB	1188
507694	1p	18481403	18482217	815	6	0.667	Promoter(<=1kb)	421	KLHDC7A	127707
507694	1p	40067594	40067675	82	6	0	Promoter(<=1kb)	324	CAP1	10487
507694	1q	152213286	152213347	62	8	0.5	Exon(exon3of3)	10846	HRNR	388697
507694	1q	152219233	152221375	2143	16	0.75	Promoter(2-3kb)	2818	HRNR	388697
507694	1q	158765805	158766655	851	6	0.5	Promoter(<=1kb)	47	OR6N1	128372
507694	1q	169542317	169542882	566	7	0.143	Exon(exon13of25)	-26572	F5	2153
507694	1q	214640144	214642954	2811	12	0.5	Exon(exon12of20)	-5013	CENPF	1063
507694	1q	214644872	214647386	2515	11	0.364	Promoter(<=1kb)	-581	CENPF	1063
507694	1q	228315976	228318038	2063	9	0.667	Exon(exon50of81)	6492	OBSCN	84033
507694	1q	247841312	247841582	271	6	0.833	Promoter(<=1kb)	314	OR11L1	391189
507694	1q	247949325	247949738	414	10	0.3	Promoter(<=1kb)	467	OR2L8	391190
507694	2p	29071763	29073000	1238	6	0.667	Promoter(1-2kb)	1523	PCARE	388939
507694	2p	48580657	48582454	1798	7	0.571	Promoter(<=1kb)	0	STON1	11037
507694	2q	132783061	132784972	1912	7	0.429	Promoter(1-2kb)	-1038	NCKAP5	344148
507694	2q	178739433	178741811	2379	6	0.5	Exon(exon45of191)	26014	TTN	7273
507694	2q	184936178	184937636	1459	6	0.333	Exon(exon4of4)	69813	ZNF804A	91752
507694	2q	208325018	208326375	1358	6	0.5	3'UTR	-7997	PIKFYVE	200576
507694	2q	217847567	217848559	993	7	0.857	Exon(exon19of33)	-5407	TNS1	7145
507694	2q	238130271	238131546	1276	7	0.286	Promoter(1-2kb)	1323	ESPNL	339768
507694	2q	240041845	240042187	343	7	0.286	Downstream(2-3kb)	3885	OR6B3	150681
507694	3p	75737230	75739007	1778	9	0.556	Promoter(<=1kb)	0	MIR4273	100422955
507694	3q	196947878	196948448	571	6	0.5	3'UTR	3482	PIGZ	80235
507694	4p	5988383	5989749	1367	7	0.571	Promoter(<=1kb)	0	C4orf50	389197
507694	4p	6300792	6302360	1569	6	0.833	Exon(exon8of8)	6021	WFS1	7466
507694	4p	8227004	8228508	1505	8	0.125	Promoter(<=1kb)	-24	SH3TC1	54436
507694	4q	154489498	154491312	1815	9	0.556	Promoter(<=1kb)	22	DCHS2	54798
507694	4q	186617176	186621582	4407	10	0.4	Exon(exon10of27)	-7106	FAT1	2195
507694	5q	79731782	79734523	2742	13	0.308	Exon(exon2of13)	-3619	CMYA5	202333
507694	5q	140807352	140807737	386	6	0.833	Promoter(<=1kb)	271	PCDHA4	56144
507694	5q	141100771	141102758	1988	10	0.3	Promoter(<=1kb)	298	PCDHB3	56132
507694	5q	141174000	141175025	1026	6	0.833	Promoter(1-2kb)	1356	PCDHB7	56129
507694	5q	141183999	141184688	690	6	0.833	Promoter(2-3kb)	-2473	PCDHB9	56127
507694	6p	1312723	1313745	1023	7	0.571	Promoter(<=1kb)	625	FOXQ1	94234
507694	6p	46858771	46859502	732	8	0.5	Exon(exon17of21)	3802	ADGRF5	221395
507694	6q	106511549	106512572	1024	6	0.667	Promoter(<=1kb)	0	CRYBG1	202
507694	6q	159233455	159234370	916	10	0.5	Exon(exon11of23)	15158	FNDC1	84624
507694	6q	168307636	168308954	1319	6	0.5	5'UTR	10756	DACT2	168002
507694	7p	12369637	12370736	1100	6	0.667	3'UTR	-13307	VWDE	221806
507694	7q	100958977	100960873	1897	56	0.429	Promoter(1-2kb)	1012	MUC3A	4584
507694	7q	100991195	100992398	1204	7	0.571	Exon(exon5of15)	-20656	MUC12	10071
507694	7q	100995420	100995785	366	8	0.75	Exon(exon5of15)	-17269	MUC12	10071
507694	7q	101004258	101004836	579	8	0.875	Exon(exon5of15)	-8218	MUC12	10071
507694	7q	101034361	101040583	6223	36	0.528	Exon(exon3of12)	-3128	MUC17	140453
507694	8p	10607245	10608432	1188	8	0.5	Exon(exon4of4)	46711	RP1L1	94137
507694	8p	11331234	11332082	849	7	0.571	Promoter(<=1kb)	346	SLC35G5	83650
507694	8p	12132686	12133940	1255	6	0.667	Promoter(<=1kb)	498	USP17L7	392197
507694	8p	13021128	13022030	903	7	0.143	Exon(exon5of5)	9115	TRMT9B	57604
507694	8q	141466429	141467514	1086	9	0.778	3'UTR	29245	MROH5	389690
507694	8q	143916360	143919209	2850	8	0.25	Exon(exon32of32)	20381	PLEC	5339
507694	9p	116800	117934	1135	7	0.857	Promoter(<=1kb)	270	FOXD4	2298
507694	9p	21206714	21207074	361	7	0.571	Promoter(<=1kb)	69	IFNA10	3446
507694	9p	39078723	39078846	124	6	0.667	Exon(exon22of24)	7302	CNTNAP3	79937
507694	9q	76175237	76175296	60	9	0.778	Exon(exon14of14)	-13343	PCSK5	5125
507694	9q	76703451	76707804	4354	14	0.429	Promoter(<=1kb)	0	PCA3	50652
507694	9q	76709263	76710843	1581	8	0.5	Promoter(<=1kb)	0	PRUNE2	158471
507694	9q	87887543	87888819	1277	6	0.5	Exon(exon4of4)	4666	SPATA31E1	286234
507694	9q	122553263	122554071	809	7	0.429	Promoter(<=1kb)	93	OR1N2	138882
507694	9q	122628595	122629398	804	6	0.333	Promoter(<=1kb)	175	OR1B1	347169
507694	9q	122749914	122750547	634	6	0.833	Promoter(<=1kb)	174	OR1L6	392390
507694	9q	133255635	133256205	571	7	1	3'UTR	19009	ABO	28

507694	9q	135484803	135487213	2411	8	0.25	Promoter(1-2kb)	1440	PPP1R26	9858
507694	9q	135547960	135548795	836	8	0.625	Promoter(1-2kb)	1805	OBP2A	29991
507694	10q	46549378	46550723	1346	26	0.654	Exon(exon3of3)	4807	GPRIN2	9721
507694	10q	49323169	49325192	2024	7	0.571	Exon(exon3of3)	23895	C10orf71	118461
507694	10q	128102594	128105201	2608	6	0.667	Promoter(<=1kb)	0	MKI67	4288
507694	11p	244106	244197	92	8	0.5	Promoter(<=1kb)	-232	PSMD13	5719
507694	11p	1246095	1247407	1313	6	0.333	Promoter(2-3kb)	2269	MUC5B-AS1	112577518
507694	11p	1250091	1251524	1434	7	0.714	Promoter(<=1kb)	-415	MUC5B-AS1	112577518
507694	11p	5046754	5047432	679	7	0.571	Promoter(<=1kb)	228	OR52J3	119679
507694	11p	5177978	5178478	501	6	0.167	Promoter(<=1kb)	186	OR52Z1	283110
507694	11p	5323362	5324256	895	7	0.429	Promoter(<=1kb)	41	OR51B2	79345
507694	11p	5389704	5390350	647	7	0.429	Promoter(<=1kb)	327	OR51M1	390059
507694	11p	5422212	5423123	912	10	0.7	Promoter(<=1kb)	101	OR51Q1	390061
507694	11p	5515185	5516015	831	6	0.333	Promoter(<=1kb)	684	UBQLNL	143630
507694	11p	5581045	5581738	694	8	0.375	Promoter(<=1kb)	168	OR52B6	340980
507694	11p	5841302	5841883	582	9	0.333	Promoter(<=1kb)	14	OR52E6	390078
507694	11p	11351961	11352736	776	9	0.222	Promoter(<=1kb)	514	CSNK2A3	283106
507694	11p	12293639	12294842	1204	7	0.714	Exon(exon29of35)	6739	MICALCL	84953
507694	11p	43942293	43943348	1056	9	0.778	Promoter(<=1kb)	0	C11orf96	387763
507694	11q	58214757	58215722	966	8	0.25	Promoter(<=1kb)	12	OR1S1	219959
507694	11q	64116513	64118232	1720	7	0.714	Exon(exon2of2)	8702	MACROD1	28992
507694	11q	66560202	66562261	2060	6	0.333	Exon(exon14of21)	3873	CTSF	8722
507694	11q	85724687	85726853	2167	9	0.444	Promoter(<=1kb)	0	SYTL2	54843
507694	11q	124015601	124016477	877	9	0.333	Promoter(<=1kb)	26	OR10G4	390264
507694	11q	124023038	124023849	812	9	0.444	Promoter(<=1kb)	25	OR10G9	219870
507694	12p	4626571	4628549	1979	9	0.444	Exon(exon5of6)	14054	DYRK4	8798
507694	12p	6453119	6453670	552	6	0.667	Promoter(<=1kb)	633	TAPBPL	55080
507694	12p	31981306	31983288	1983	6	0.5	Exon(exon4of6)	-3878	RESF1	55196
507694	13q	24434450	24435347	898	7	0.571	Exon(exon31of34)	19787	PARP4	143
507694	13q	25096870	25097182	313	8	0.375	Promoter(1-2kb)	1002	PABPC3	5042
507694	13q	102732474	102733933	1460	6	0.333	Exon(exon4of4)	25139	CCDC168	643677
507694	14q	20060048	20060884	837	8	0.625	Promoter(<=1kb)	3	OR4L1	122742
507694	14q	20223729	20224484	756	7	0.571	Promoter(<=1kb)	19	OR11H6	122748
507694	14q	20640982	20641567	586	7	0.429	Promoter(<=1kb)	124	OR6S1	341799
507694	14q	21634137	21634589	453	9	0.556	Promoter(<=1kb)	351	OR10G2	26534
507694	14q	22633879	22634450	572	9	0.333	Exon(exon2of2)	32212	ABHD4	63874
507694	14q	70457520	70458540	1021	12	0.333	Exon(exon2of2)	5346	ADAM21	8747
507694	14q	94587512	94587839	328	6	0.5	Exon(exon2of2)	-4219	SERPINA3	12
507694	14q	104175275	104177810	2536	6	0.167	Exon(exon12of15)	36235	KIF26A	26153
507694	14q	104939262	104945444	6183	20	0.4	5'UTR	7102	PLD4	122618
507694	14q	104946451	104953878	7428	44	0.545	Promoter(1-2kb)	1524	AHNAK2	113146
507694	15q	20534480	20534954	475	6	0.333	Exon(exon8of9)	6846	GOLGA6L6	727832
507694	15q	23439979	23442067	2089	10	0.6	5'UTR	5167	GOLGA6L2	283685
507694	15q	73702465	73703760	1296	6	0.833	Promoter(<=1kb)	-149	CD276	80381
507694	15q	78766033	78766581	549	6	0.667	Promoter(<=1kb)	-920	ADAMTS7	11173
507694	16p	768559	770944	2386	12	0.5	Promoter(<=1kb)	0	MIR662	724032
507694	16p	1228744	1229583	840	9	0.778	Promoter(<=1kb)	579	TPSB2	64499
507694	16p	1256345	1256816	472	6	0.833	Promoter(<=1kb)	276	TPSD1	23430
507694	16p	1486371	1488463	2093	8	0.75	Promoter(<=1kb)	4	PTX4	390667
507694	16q	74391401	74391928	528	7	0.714	Exon(exon7of7)	13523	NPIPB15	440348
507694	16q	89226863	89228289	1427	8	0.625	Promoter(2-3kb)	2229	ZNF778	197320
507694	17p	10638198	10641099	2902	6	0.333	Exon(exon19of41)	-8169	MYH3	4621
507694	17p	21300581	21300978	398	12	0.75	3'UTR	9112	MAP2K3	5606
507694	17p	21415470	21416404	935	6	1	Exon(exon3of3)	10334	KCNJ12	3768
507694	17q	41462841	41462967	127	6	0.833	Exon(exon6of7)	4462	KRT32	3882
507694	17q	81645135	81645607	473	7	0.286	Promoter(2-3kb)	2722	TSPAN10	83882
507694	18p	11609646	11610164	519	13	0.846	Promoter(<=1kb)	50	SLC35G4	646000
507694	18p	14542649	14543140	492	6	0.5	Promoter(<=1kb)	6	POTEC	388468
507694	18q	58535186	58538030	2845	19	0.526	Promoter(<=1kb)	0	ALPK2	115701
507694	19p	1003159	1003440	282	7	1	Promoter(2-3kb)	2740	GRIN3B	116444
507694	19p	1004688	1005532	845	6	0.333	Exon(exon3of9)	4269	GRIN3B	116444
507694	19p	1036457	1036914	458	6	0.5	Exon(exon6of7)	-3187	ABCA7	10347
507694	19p	4510548	4513547	3000	21	0.476	Exon(exon3of6)	4157	PLIN4	729359
507694	19p	8946732	8951868	5137	13	0.462	Exon(exon3of84)	29474	MUC16	94025
507694	19p	12430400	12431840	1441	8	0.5	3'UTR	9181	ZNF443	10224
507694	19p	17282085	17284246	2162	8	0.5	Promoter(<=1kb)	0	ANKLE1	126549
507694	19p	18264798	18267409	2612	12	0.583	5'UTR	7002	IQCN	80726
507694	19p	21971930	21974500	2571	7	0.714	Exon(exon4of4)	14408	ZNF208	7757
507694	19q	36996730	36997597	868	10	0.7	Exon(exon10of10)	5677	ZNF568	374900
507694	19q	40880231	40880622	392	6	0.333	Promoter(<=1kb)	-141	CYP2A7	1549
507694	19q	43846955	43848536	1582	6	0.833	3'UTR	13450	ZNF283	284349
507694	19q	43913423	43914878	1456	8	0.5	Exon(exon10of10)	4861	ZNF45	7596
507694	19q	43966037	43967171	1135	6	0.167	Promoter(<=1kb)	-691	ZNF155	7711
507694	19q	44106512	44108078	1567	7	0	Exon(exon6of6)	-4103	ZNF225	7768
507694	19q	44327836	44329698	1863	6	0.5	Exon(exon4of4)	-22790	ZNF235	9310
507694	19q	52437918	52439242	1325	7	0.429	Exon(exon4of4)	6504	ZNF534	147658
507694	19q	55911888	55913077	1190	6	0.667	Exon(exon5of12)	19234	NLRP13	126204

507694	19q	58368293	58368875	583	8	0.375	Exon(exon3of3)	-5445	ZNF497	162968
507694	20p	5922421	5923394	974	7	0.571	Exon(exon4of5)	6923	CHGB	1114
507694	20q	63349752	63350772	1021	6	0.5	3'UTR	3794	CHRNA4	1137
507694	20q	63561666	63567360	5695	19	0.684	Promoter(<=1kb)	-61	HELZ2	85441
507694	21q	26843740	26844859	1120	6	0.667	Promoter(<=1kb)	0	ADAMTS1	9510
507694	21q	44539312	44540035	724	6	0.833	Promoter(<=1kb)	160	KRTAP10-1	386677
507694	21q	44550835	44551416	582	6	0.833	Promoter(<=1kb)	89	KRTAP10-2	386679
507694	22q	22352950	22353380	431	16	0.5	Exon(exon1of2)	30478	BMS1P20	96610
507694	22q	36191154	36191906	753	6	0.667	3'UTR	9971	APOL4	80832
507694	23p	8170039	8170141	103	6	0.5	Promoter(1-2kb)	1126	VCX2	51480
507694	23p	35802148	35803010	863	7	0.571	5'UTR	3357	MAGEB16	139604
508046	1p	978953	979884	932	6	0.5	Promoter(1-2kb)	1145	PERM1	84808
508046	1p	12847526	12847780	255	7	0.429	Promoter(<=1kb)	945	HNRNPCL1	343069
508046	1p	12859036	12860079	1044	6	0.333	Promoter(1-2kb)	1950	PRAMEF2	65122
508046	1p	13369166	13369564	399	7	0.857	Promoter(2-3kb)	2336	PRAMEF19	645414
508046	1p	13370686	13370957	272	7	0.429	Promoter(<=1kb)	943	PRAMEF19	645414
508046	1p	16058491	16060000	1510	13	0.846	Exon(exon5of7)	6168	CLCNKB	1188
508046	1p	18481403	18482217	815	6	0.667	Promoter(<=1kb)	421	KLHDC7A	127707
508046	1p	23874604	23875430	827	9	0.556	Exon(exon2of2)	-6310	FUCA1	2517
508046	1p	40067594	40067675	82	6	0	Promoter(<=1kb)	324	CAP1	10487
508046	1p	89186388	89186419	32	9	0.556	Promoter(<=1kb)	107	GBP4	115361
508046	1q	152302977	152313891	10915	39	0.59	Promoter(<=1kb)	0	FLG-AS1	339400
508046	1q	156669844	156670886	1043	6	1	Exon(exon4of4)	6521	NES	10763
508046	1q	158765805	158766655	851	6	0.5	Promoter(<=1kb)	47	OR6N1	128372
508046	1q	169542317	169542882	566	6	0.167	Exon(exon13of25)	-26572	F5	2153
508046	1q	201211017	201212095	1079	7	0.429	Promoter(<=1kb)	-547	IGFN1	91156
508046	1q	228315976	228318049	2074	10	0.7	Exon(exon50of81)	6492	OBSCN	84033
508046	1q	247841312	247841582	271	6	0.833	Promoter(<=1kb)	314	OR11L1	391189
508046	1q	247949443	247949738	296	10	0.3	Promoter(<=1kb)	585	OR2L8	391190
508046	2q	102351547	102351902	356	7	0.429	Exon(exon11of11)	-4027	IL18R1	8809
508046	2q	132783032	132785125	2094	8	0.625	Promoter(1-2kb)	-1009	NCKAP5	344148
508046	2q	185788819	185794632	5814	12	0.75	Promoter(<=1kb)	0	FSIP2	401024
508046	2q	217847567	217848213	647	6	0.833	Exon(exon19of33)	-5407	TNS1	7145
508046	2q	219271337	219271649	313	6	0.667	Exon(exon4of4)	6227	TUBA4A	7277
508046	2q	233713134	233713664	531	8	0.75	Promoter(<=1kb)	142	UGT1A5	54579
508046	2q	233840612	233842185	1574	6	0.667	Promoter(<=1kb)	0	HJURP	55355
508046	2q	240041845	240042154	310	6	0.167	Downstream(2-3kb)	3918	OR6B3	150681
508046	3p	31989532	31990905	1374	7	0.286	Exon(exon2of2)	7761	ZNF860	344787
508046	3p	52521941	52524117	2177	6	0.333	Promoter(<=1kb)	0	STAB1	23166
508046	3p	75736880	75738859	1980	49	0.612	Promoter(<=1kb)	0	MIR4273	100422955
508046	3q	98264413	98265098	686	7	0.571	Promoter(<=1kb)	128	OR5H6	79295
508046	4p	5988383	5989749	1367	7	0.571	Promoter(<=1kb)	0	C4orf50	389197
508046	4p	6300792	6302360	1569	6	0.833	Exon(exon8of8)	6021	WFS1	7466
508046	4q	121036404	121037542	1139	6	0.333	Promoter(1-2kb)	1442	NDNF	79625
508046	4q	185458217	185460011	1795	8	0.625	Promoter(<=1kb)	0	CCDC110	256309
508046	5q	79728956	79730716	1761	6	0.333	Exon(exon2of13)	-7426	CMYA5	202333
508046	5q	79731782	79734523	2742	13	0.308	Exon(exon2of13)	-3619	CMYA5	202333
508046	5q	140848579	140850786	2208	6	0.5	Promoter(<=1kb)	807	PCDHA9	9752
508046	5q	141174000	141175025	1026	6	0.833	Promoter(1-2kb)	1356	PCDHB7	56129
508046	5q	151565922	151568455	2534	10	0.8	Promoter(<=1kb)	489	FAT2	2196
508046	6p	46858771	46859502	732	8	0.5	Exon(exon17of21)	3802	ADGRF5	221395
508046	6q	149888581	149890867	2287	7	0.714	Promoter(<=1kb)	0	RAET1E-AS1	100652739
508046	6q	159233455	159234370	916	10	0.5	Exon(exon11of23)	15158	FNDC1	84624
508046	7q	100958977	100960873	1897	56	0.446	Promoter(1-2kb)	1012	MUC3A	4584
508046	7q	100991195	100993127	1933	8	0.625	Exon(exon5of15)	-19927	MUC12	10071
508046	7q	100995575	100995785	211	6	0.833	Exon(exon5of15)	-17269	MUC12	10071
508046	7q	149818015	149819792	1778	6	0.667	Promoter(2-3kb)	-2352	SSPO	23145
508046	8p	10607245	10612307	5063	18	0.722	Exon(exon4of4)	42836	RP1L1	94137
508046	8p	11331234	11332082	849	6	0.667	Promoter(<=1kb)	346	SLC35G5	83650
508046	8p	13021128	13022030	903	8	0.25	Exon(exon5of5)	9115	TRMT9B	57604
508046	8q	123651873	123652634	762	6	0.333	Promoter(<=1kb)	316	KLHL38	340359
508046	9p	712060	713307	1248	7	0.714	Exon(exon7of16)	5171	KANK1	23189
508046	9q	76703555	76706360	2806	8	0.5	Promoter(<=1kb)	0	PCA3	50652
508046	9q	122553263	122554071	809	8	0.5	Promoter(<=1kb)	93	OR1N2	138882
508046	9q	122628595	122629398	804	8	0.375	Promoter(<=1kb)	175	OR1B1	347169
508046	9q	122749914	122750547	634	6	0.833	Promoter(<=1kb)	174	OR1L6	392390
508046	9q	135484803	135487213	2411	8	0.25	Promoter(1-2kb)	1440	PPP1R26	9858
508046	9q	135547960	135548795	836	8	0.625	Promoter(1-2kb)	1805	OBP2A	29991
508046	10q	46549378	46550723	1346	25	0.64	Exon(exon3of3)	4807	GPRIN2	9721
508046	10q	49323559	49326554	2996	10	0.5	Exon(exon3of3)	24285	C10orf71	118461
508046	10q	128103129	128106296	3168	14	0.643	Promoter(<=1kb)	-1	MKI67	4288
508046	11p	244106	244197	92	8	0.5	Promoter(<=1kb)	-232	PSMD13	5719
508046	11p	1194354	1196902	2549	7	0.571	Exon(exon34of49)	-26164	MUC5B	727897
508046	11p	1245466	1247378	1913	8	0.375	Promoter(2-3kb)	2298	MUC5B-AS1	112577518
508046	11p	5177978	5178478	501	6	0.167	Promoter(<=1kb)	186	OR52Z1	283110
508046	11p	5323542	5324256	715	6	0.5	Promoter(<=1kb)	41	OR51B2	79345
508046	11p	5389704	5390350	647	6	0.5	Promoter(<=1kb)	327	OR51M1	390059

508046	11p	5402638	5403322	685	9	0.444	Promoter(<=1kb)	41	OR51J1	79470
508046	11p	5440604	5441472	869	6	0.833	Promoter(<=1kb)	42	OR51I1	390063
508046	11p	5515185	5515931	747	7	0.286	Promoter(<=1kb)	768	UBQLNL	143630
508046	11p	5581045	5581738	694	8	0.375	Promoter(<=1kb)	168	OR52B6	340980
508046	11p	5841302	5841883	582	9	0.333	Promoter(<=1kb)	14	OR52E6	390078
508046	11p	11351961	11352736	776	10	0.2	Promoter(<=1kb)	514	CSNK2A3	283106
508046	11p	12293639	12294368	730	6	0.833	Exon(exon29of35)	6739	MICALCL	84953
508046	11q	55827536	55827640	105	6	0.667	Promoter(<=1kb)	317	OR5L2	26338
508046	11q	58214757	58215722	966	8	0.25	Promoter(<=1kb)	12	OR1S1	219959
508046	11q	82732630	82733184	555	6	0.833	Promoter(<=1kb)	680	FAM181B	220382
508046	11q	85724687	85725825	1139	6	0.5	Promoter(<=1kb)	0	SYTL2	54843
508046	11q	93697503	93700096	2594	11	0.636	Promoter(<=1kb)	0	CEP295	85459
508046	11q	130914501	130915409	909	10	0.7	Promoter(1-2kb)	1035	SNX19	399979
508046	12p	4626568	4628549	1982	10	0.5	Exon(exon5of6)	14051	DYRK4	8798
508046	12p	6018369	6019277	909	6	0.167	Promoter(2-3kb)	2649	VWF	7450
508046	12p	6453119	6453670	552	6	0.667	Promoter(<=1kb)	633	TAPBPL	55080
508046	12q	52316096	52317765	1670	6	0.667	Exon(exon4of9)	3633	KRT83	3889
508046	12q	52571389	52573652	2264	6	0.667	Promoter(<=1kb)	164	KRT74	121391
508046	13q	49668389	49668560	172	6	0.333	Exon(exon3of3)	22878	EBPL	84650
508046	13q	102732474	102733933	1460	6	0.333	Exon(exon4of4)	25139	CCDC168	643677
508046	14q	19975713	19976448	736	9	0.444	Promoter(<=1kb)	269	OR4K15	81127
508046	14q	44504986	44506403	1418	6	0.667	Promoter(<=1kb)	880	FSCB	84075
508046	14q	70457532	70458540	1009	9	0.333	Exon(exon2of2)	5358	ADAM21	8747
508046	14q	94587512	94587839	328	6	0.5	Exon(exon2of2)	-4219	SERPINA3	12
508046	14q	104939262	104942618	3357	10	0.2	5'UTR	7102	PLD4	122618
508046	14q	104943622	104945444	1823	7	0.571	Exon(exon6of6)	9958	AHNAK2	113146
508046	14q	104947943	104953878	5936	35	0.457	Promoter(1-2kb)	1524	AHNAK2	113146
508046	15q	20534480	20535129	650	7	0.429	Exon(exon8of9)	6671	GOLGA6L6	727832
508046	15q	22465655	22465901	247	7	0.429	Exon(exon8of11)	18939	HERC2P7	100132101
508046	15q	23439979	23442067	2089	10	0.6	5'UTR	5167	GOLGA6L2	283685
508046	15q	85579423	85581800	2378	15	0.6	Promoter(1-2kb)	-1110	AKAP13	11214
508046	15q	88856792	88859365	2574	9	0.222	Exon(exon12of18)	9549	ACAN	176
508046	15q	99129423	99132517	3095	6	0.333	Exon(exon4of5)	7225	TTC23	64927
508046	15q	101177475	101178745	1271	6	0.667	3'UTR	9125	CHSY1	22856
508046	16p	1228744	1229731	988	10	0.7	Promoter(<=1kb)	431	TPSB2	64499
508046	16p	1256345	1256980	636	6	0.833	Promoter(<=1kb)	276	TPSD1	23430
508046	16p	1486371	1488463	2093	8	0.75	Promoter(<=1kb)	4	PTX4	390667
508046	16p	4207130	4208004	875	6	0.5	Exon(exon2of7)	31739	SRL	6345
508046	16p	28495551	28497395	1845	6	0.333	Promoter(<=1kb)	0	CLN3	1201
508046	16q	88428539	88429600	1062	6	0.333	Exon(exon3of3)	-23680	ZFPM1	161882
508046	16q	88714632	88717113	2482	7	0.429	Promoter(<=1kb)	0	MIR4722	100616167
508046	16q	89226863	89228390	1528	10	0.6	Promoter(2-3kb)	2229	ZNF778	197320
508046	17p	744946	746966	2021	6	1	3'UTR	5072	GEMIN4	50628
508046	17p	21300581	21300954	374	8	0.75	3'UTR	9112	MAP2K3	5606
508046	17q	76293419	76294016	598	6	0.5	Promoter(2-3kb)	-2167	QRICH2	84074
508046	17q	81645135	81645417	283	6	0.333	Promoter(2-3kb)	2722	TSPAN10	83882
508046	18p	11609646	11610491	846	12	0.75	Promoter(<=1kb)	50	SLC35G4	646000
508046	18p	14542649	14543140	492	6	0.5	Promoter(<=1kb)	6	POTEC	388468
508046	18q	58535186	58538030	2845	19	0.579	Promoter(<=1kb)	0	ALPK2	115701
508046	19p	4510711	4511943	1233	10	0.6	Exon(exon3of6)	5761	PLIN4	729359
508046	19p	5455600	5456439	840	6	0.5	Promoter(<=1kb)	183	ZNRF4	148066
508046	19p	8946313	8951868	5556	16	0.625	Exon(exon3of84)	29474	MUC16	94025
508046	19p	8959116	8962299	3184	10	0.7	Exon(exon3of84)	19043	MUC16	94025
508046	19p	8972751	8978096	5346	13	0.462	Exon(exon1of84)	3246	MUC16	94025
508046	19p	17281820	17284246	2427	9	0.556	Promoter(<=1kb)	0	ANKLE1	126549
508046	19p	18264753	18267409	2657	8	0.5	5'UTR	7002	IQCN	80726
508046	19p	21971930	21974500	2571	7	0.714	Exon(exon4of4)	14408	ZNF208	7757
508046	19p	22756205	22759523	3319	13	0.615	3'UTR	10459	ZNF99	7652
508046	19p	23743906	23745300	1395	6	0.333	Exon(exon4of4)	13537	ZNF681	148213
508046	19q	34958015	34959325	1311	6	1	Promoter(1-2kb)	1378	ZNF792	126375
508046	19q	36996730	36997597	868	10	0.7	Exon(exon10of10)	5677	ZNF568	374900
508046	19q	37151928	37153149	1222	6	0.833	Exon(exon5of5)	19287	ZNF585A	199704
508046	19q	39877143	39877880	738	6	0.5	Exon(exon20of28)	9412	FCGBP	8857
508046	19q	43913423	43914878	1456	9	0.556	Exon(exon10of10)	4861	ZNF45	7596
508046	19q	43966037	43967171	1135	6	0.167	Promoter(<=1kb)	-691	ZNF155	7711
508046	19q	43996326	43997366	1041	6	0.5	Exon(exon5of5)	5419	LOC10192806	101928063
508046	19q	44106512	44108078	1567	7	0	Exon(exon6of6)	-4103	ZNF225	7768
508046	19q	51413748	51415963	2216	6	0.667	Promoter(<=1kb)	0	LOC10012908	100129083
508046	19q	51872787	51873699	913	10	0.5	Promoter(<=1kb)	-940	ZNF577	84765
508046	19q	52437918	52439242	1325	7	0.429	Exon(exon4of4)	6504	ZNF534	147658
508046	19q	55481625	55483456	1832	7	0.429	Promoter(1-2kb)	-1732	NAT14	57106
508046	19q	55517821	55518642	822	9	1	Exon(exon14of14)	17661	SBK2	646643
508046	19q	55911888	55913166	1279	7	0.714	Exon(exon5of12)	19145	NLRP13	126204
508046	19q	58368293	58368875	583	7	0.429	Exon(exon3of3)	-5445	ZNF497	162968
508046	20p	5922421	5923394	974	6	0.5	Exon(exon4of5)	6923	CHGB	1114
508046	20p	20052354	20052736	383	6	0	Promoter(<=1kb)	0	CFAP61	26074
508046	20q	63349752	63350772	1021	6	0.5	3'UTR	3794	CHRNA4	1137

508046	20q	63561666	63565531	3866	15	0.667	Promoter(<=1kb)	-61	HELZ2	85441
508046	21q	26843740	26844859	1120	6	0.667	Promoter(<=1kb)	0	ADAMTS1	9510
508046	21q	44600627	44601692	1066	10	0.7	Promoter(<=1kb)	30	KRTAP10-7	386675
508046	21q	44637474	44638041	568	6	0.333	Promoter(<=1kb)	118	KRTAP10-10	353333
508046	21q	45455650	45456666	1017	6	0.667	Promoter(<=1kb)	119	COL18A1	80781
508046	22q	22352950	22353298	349	13	0.538	Exon(exon1of2)	30478	BMS1P20	96610
508046	22q	22646441	22646811	371	6	0.333	Promoter(<=1kb)	0	GGTLC2	91227
508046	22q	36191154	36191906	753	7	0.571	3'UTR	9971	APOL4	80832
508046	22q	36265284	36265796	513	6	1	Exon(exon6of6)	12120	APOL1	8542
508046	22q	49883704	49884994	1291	6	0.167	Exon(exon2of2)	22924	ALG12	79087
508046	23p	8170039	8170141	103	6	0.5	Promoter(1-2kb)	1126	VCX2	51480
508046	23p	35802148	35803010	863	7	0.571	5'UTR	3357	MAGEB16	139604
508046	23q	136874183	136874416	234	7	0.857	Promoter(<=1kb)	-201	RBMX	27316
508496	1p	12795518	12795749	232	7	0.429	Promoter(<=1kb)	827	PRAMEF1	65121
508496	1p	12847526	12847995	470	11	0.364	Promoter(<=1kb)	730	HNRNPCL1	343069
508496	1p	12859036	12860079	1044	6	0.333	Promoter(1-2kb)	1950	PRAMEF2	65122
508496	1p	12893187	12893437	251	10	0.5	Exon(exon4of4)	4833	PRAMEF10	343071
508496	1p	13370686	13371119	434	9	0.444	Promoter(<=1kb)	781	PRAMEF19	645414
508496	1p	16058491	16060000	1510	12	0.833	Exon(exon5of7)	6168	CLCNKB	1188
508496	1p	18481403	18483159	1757	7	0.714	Promoter(<=1kb)	421	KLHDC7A	127707
508496	1p	40067594	40067675	82	6	0	Promoter(<=1kb)	324	CAP1	10487
508496	1p	89186388	89186419	32	9	0.556	Promoter(<=1kb)	107	GBP4	115361
508496	1q	152303673	152313911	10239	41	0.585	Promoter(<=1kb)	0	FLG-AS1	339400
508496	1q	169542317	169542882	566	6	0.167	Exon(exon13of25)	-26572	F5	2153
508496	1q	228315933	228318038	2106	9	0.667	Exon(exon50of81)	6449	OBSCN	84033
508496	1q	232805117	232806800	1684	6	0.5	Promoter(<=1kb)	225	MAP10	54627
508496	1q	247841312	247841582	271	6	0.833	Promoter(<=1kb)	314	OR11L1	391189
508496	1q	247949443	247949738	296	9	0.333	Promoter(<=1kb)	585	OR2L8	391190
508496	2p	48580657	48582454	1798	7	0.571	Promoter(<=1kb)	0	STON1	11037
508496	2q	178739433	178741811	2379	6	0.5	Exon(exon45of191)	26014	TTN	7273
508496	2q	184936178	184937636	1459	6	0.333	Exon(exon4of4)	69813	ZNF804A	91752
508496	2q	185789865	185794632	4768	10	0.8	Promoter(<=1kb)	0	FSIP2	401024
508496	2q	185804558	185807185	2628	6	0.5	Promoter(<=1kb)	0	FSIP2	401024
508496	2q	217847583	217848559	977	6	0.833	Exon(exon19of33)	-5423	TNS1	7145
508496	2q	219489386	219491798	2413	6	0.5	Promoter(<=1kb)	-69	SPEGNB	100996693
508496	2q	238130416	238131546	1131	7	0.429	Promoter(1-2kb)	1468	ESPNL	339768
508496	2q	240041845	240042131	287	6	0.167	Downstream(2-3kb)	3941	OR6B3	150681
508496	3p	13570520	13571564	1045	6	0.167	Promoter(1-2kb)	1780	FBLN2	2199
508496	3p	31989532	31990905	1374	7	0.286	Exon(exon2of2)	7761	ZNF860	344787
508496	3p	75737230	75739007	1778	9	0.556	Promoter(<=1kb)	0	MIR4273	100422955
508496	3q	98264413	98265137	725	6	0.667	Promoter(<=1kb)	128	OR5H6	79295
508496	3q	196947878	196948662	785	6	0.5	3'UTR	3268	PIGZ	80235
508496	4p	5988383	5989749	1367	7	0.571	Promoter(<=1kb)	0	C4orf50	389197
508496	4p	6300792	6302360	1569	6	0.833	Exon(exon8of8)	6021	WFS1	7466
508496	4p	38773164	38775552	2389	11	0.455	Promoter(<=1kb)	644	TLR10	81793
508496	4q	121036404	121037542	1139	6	0.333	Promoter(1-2kb)	1442	NDNF	79625
508496	4q	186619481	186621582	2102	7	0.429	Exon(exon10of27)	-9411	FAT1	2195
508496	4q	186706638	186708616	1979	6	0.5	Exon(exon2of27)	15217	FAT1	2195
508496	5p	795818	796237	420	9	0.556	3'UTR	4908	ZDHHC11	79844
508496	5q	79728956	79730716	1761	7	0.286	Exon(exon2of13)	-7426	CMYA5	202333
508496	5q	79731782	79735549	3768	16	0.438	Promoter(2-3kb)	-2593	CMYA5	202333
508496	5q	83537326	83539905	2580	6	0.333	Promoter(1-2kb)	1712	VCAN	1462
508496	5q	140807352	140807737	386	6	0.833	Promoter(<=1kb)	271	PCDHA4	56144
508496	5q	140848579	140850786	2208	6	0.5	Promoter(<=1kb)	807	PCDHA9	9752
508496	5q	151565922	151568455	2534	10	0.8	Promoter(<=1kb)	489	FAT2	2196
508496	6p	46858771	46859389	619	6	0.5	Exon(exon17of21)	3915	ADGRF5	221395
508496	6p	47681486	47682172	687	6	0.333	Exon(exon6of6)	-3692	ADGRF4	221393
508496	6q	149888581	149889987	1407	6	0.667	Promoter(<=1kb)	-116	RAET1E-AS1	100652739
508496	6q	159233455	159234370	916	10	0.5	Exon(exon11of23)	15158	FNDC1	84624
508496	7p	38353718	38353991	274	9	0.667	Exon(exon2of2)	3699	TRG-AS1	100506776
508496	7p	45082865	45084866	2002	7	0.429	Promoter(1-2kb)	-1465	NACAD	23148
508496	7p	53035678	53036385	708	7	1	Promoter(<=1kb)	45	POM121L12	285877
508496	7q	100958721	100960873	2153	59	0.441	Promoter(<=1kb)	756	MUC3A	4584
508496	7q	100990701	100994184	3484	14	0.714	Exon(exon5of15)	-18870	MUC12	10071
508496	7q	100995547	100995785	239	7	0.857	Exon(exon5of15)	-17269	MUC12	10071
508496	8p	10607245	10612307	5063	19	0.737	Exon(exon4of4)	42836	RP1L1	94137
508496	8p	11331234	11332082	849	7	0.571	Promoter(<=1kb)	346	SLC35G5	83650
508496	8p	13021128	13022030	903	7	0.143	Exon(exon5of5)	9115	TRMT9B	57604
508496	8q	100706704	100706893	190	6	1	Promoter(<=1kb)	0	PABPC1	26986
508496	8q	123651655	123652634	980	7	0.571	Promoter(<=1kb)	316	KLHL38	340359
508496	8q	143916360	143919209	2850	7	0.143	Exon(exon32of32)	20381	PLEC	5339
508496	8q	143923488	143925516	2029	9	0.667	Exon(exon31of32)	14074	PLEC	5339
508496	9p	116800	117934	1135	8	0.75	Promoter(<=1kb)	270	FOXD4	2298
508496	9q	76703555	76707804	4250	13	0.462	Promoter(<=1kb)	0	PCA3	50652
508496	9q	76709263	76710843	1581	9	0.556	Promoter(<=1kb)	0	PRUNE2	158471
508496	9q	87887543	87888819	1277	6	0.5	Exon(exon4of4)	4666	SPATA31E1	286234
508496	9q	104598545	104599361	817	12	0.583	Promoter(<=1kb)	52	OR13C5	138799

508496	9q	122553278	122554071	794	7	0.429	Promoter(<=1kb)	108	OR1N2	138882
508496	9q	122749914	122750547	634	6	0.833	Promoter(<=1kb)	174	OR1L6	392390
508496	9q	131474844	131476113	1270	6	0.833	Promoter(<=1kb)	0	PRRC2B	84726
508496	9q	135484803	135487213	2411	9	0.333	Promoter(1-2kb)	1440	PPP1R26	9858
508496	10q	46549378	46550723	1346	26	0.654	Exon(exon3of3)	4807	GPRIN2	9721
508496	10q	49323169	49325192	2024	7	0.571	Exon(exon3of3)	23895	C10orf71	118461
508496	10q	89737450	89738561	1112	6	0	Exon(exon20of33)	13874	KIF20B	9585
508496	10q	122084988	122087840	2853	8	0.75	Exon(exon4of23)	-25190	TACC2	10579
508496	10q	128103129	128104830	1702	10	0.6	Promoter(<=1kb)	-1	MKI67	4288
508496	10q	128106210	128109280	3071	7	1	Exon(exon12of14)	-3082	MKI67	4288
508496	11p	244106	244197	92	8	0.5	Promoter(<=1kb)	-232	PSMD13	5719
508496	11p	1241046	1243660	2615	11	0.545	Exon(exon31of49)	6016	MUC5B-AS1	112577518
508496	11p	1246687	1248605	1919	8	0.5	Promoter(1-2kb)	1071	MUC5B-AS1	112577518
508496	11p	1250091	1251524	1434	7	0.714	Promoter(<=1kb)	-415	MUC5B-AS1	112577518
508496	11p	5177978	5178478	501	6	0.167	Promoter(<=1kb)	186	OR52Z1	283110
508496	11p	5323451	5324256	806	6	0.5	Promoter(<=1kb)	41	OR51B2	79345
508496	11p	5389704	5390350	647	6	0.5	Promoter(<=1kb)	327	OR51M1	390059
508496	11p	5402638	5403322	685	9	0.444	Promoter(<=1kb)	41	OR51J1	79470
508496	11p	5422212	5423123	912	11	0.636	Promoter(<=1kb)	101	OR51Q1	390061
508496	11p	5440604	5441472	869	6	0.833	Promoter(<=1kb)	42	OR51I1	390063
508496	11p	5515079	5515931	853	7	0.429	Promoter(<=1kb)	768	UBQLNL	143630
508496	11p	5544676	5545259	584	6	0.5	Promoter(<=1kb)	290	OR52H1	390067
508496	11p	5581045	5581738	694	8	0.375	Promoter(<=1kb)	168	OR52B6	340980
508496	11p	5884818	5885061	244	6	0.333	Promoter(<=1kb)	547	OR52E4	390081
508496	11p	11351961	11352736	776	8	0.25	Promoter(<=1kb)	514	CSNK2A3	283106
508496	11p	12293639	12294538	900	8	0.625	Exon(exon29of35)	6739	MICALCL	84953
508496	11p	18173280	18173901	622	6	0.333	Promoter(<=1kb)	443	MRGPRX4	117196
508496	11p	34916266	34916763	498	6	0.667	Promoter(<=1kb)	0	APIP	51074
508496	11q	58214757	58215722	966	8	0.25	Promoter(<=1kb)	12	OR1S1	219959
508496	11q	64315797	64315856	60	8	0.75	Promoter(1-2kb)	1485	TRMT112	51504
508496	11q	85724582	85725825	1244	7	0.571	Promoter(<=1kb)	0	SYTL2	54843
508496	11q	123906790	123907324	535	6	0.667	Promoter(<=1kb)	644	OR8D4	338662
508496	11q	123943088	123943782	695	7	0.571	Promoter(<=1kb)	91	OR6T1	219874
508496	11q	124015601	124016477	877	12	0.25	Promoter(<=1kb)	26	OR10G4	390264
508496	11q	124023038	124023849	812	9	0.444	Promoter(<=1kb)	25	OR10G9	219870
508496	11q	124038366	124038988	623	9	0.889	Promoter(<=1kb)	13	OR10G7	390265
508496	12p	4626568	4628549	1982	11	0.455	Exon(exon5of6)	14051	DYRK4	8798
508496	13q	25096889	25097231	343	14	0.5	Promoter(1-2kb)	1021	PABPC3	5042
508496	13q	102732474	102733933	1460	6	0.333	Exon(exon4of4)	25139	CCDC168	643677
508496	14q	21634137	21634589	453	9	0.556	Promoter(<=1kb)	351	OR10G2	26534
508496	14q	70457532	70458540	1009	9	0.333	Exon(exon2of2)	5358	ADAM21	8747
508496	14q	104939262	104942618	3357	13	0.154	5'UTR	7102	PLD4	122618
508496	14q	104943622	104946886	3265	10	0.6	Exon(exon6of6)	8516	AHNAK2	113146
508496	14q	104947901	104950411	2511	20	0.65	Exon(exon6of6)	4991	AHNAK2	113146
508496	14q	104951428	104953878	2451	17	0.529	Promoter(1-2kb)	1524	AHNAK2	113146
508496	15q	20534501	20535129	629	6	0.333	Exon(exon8of9)	6671	GOLGA6L6	727832
508496	15q	23439979	23442067	2089	10	0.6	5'UTR	5167	GOLGA6L2	283685
508496	15q	73702465	73703760	1296	6	0.833	Promoter(<=1kb)	-149	CD276	80381
508496	15q	78766049	78766618	570	6	0.833	Promoter(<=1kb)	-936	ADAMTS7	11173
508496	15q	88857108	88859365	2258	7	0.143	Exon(exon12of18)	9865	ACAN	176
508496	15q	99129423	99132517	3095	6	0.333	Exon(exon4of5)	7225	TTC23	64927
508496	15q	100569472	100570060	589	6	0.667	Promoter(<=1kb)	571	LINS1	55180
508496	16p	669592	672548	2957	7	0.571	Promoter(<=1kb)	0	RHOT2	89941
508496	16p	1228744	1229731	988	10	0.7	Promoter(<=1kb)	431	TPSB2	64499
508496	16p	1256345	1256985	641	12	0.583	Promoter(<=1kb)	276	TPSD1	23430
508496	16p	1486371	1488463	2093	8	0.75	Promoter(<=1kb)	4	PTX4	390667
508496	16p	28495551	28498085	2535	7	0.429	Promoter(<=1kb)	0	CLN3	1201
508496	16q	88714717	88717113	2397	8	0.5	Promoter(<=1kb)	0	MIR4722	100616167
508496	16q	89100686	89101050	365	7	0.571	Promoter(<=1kb)	24	ACSF3	197322
508496	16q	89226863	89228419	1557	9	0.556	Promoter(2-3kb)	2229	ZNF778	197320
508496	17p	10638198	10641099	2902	7	0.286	Exon(exon19of41)	-8169	MYH3	4621
508496	17p	21300581	21300954	374	8	0.75	3'UTR	9112	MAP2K3	5606
508496	17p	21415470	21416404	935	20	0.85	Exon(exon3of3)	10334	KCNJ12	3768
508496	17q	76293419	76294016	598	6	0.5	Promoter(2-3kb)	-2167	QRICH2	84074
508496	17q	81645135	81645607	473	7	0.286	Promoter(2-3kb)	2722	TSPAN10	83882
508496	18p	11609683	11610596	914	23	0.783	Promoter(<=1kb)	87	SLC35G4	646000
508496	18p	11644378	11644747	370	8	0.75	Exon(exon1of1)	10194	MIR7153	102465690
508496	18p	14542649	14543140	492	7	0.571	Promoter(<=1kb)	6	POTEC	388468
508496	18q	58535186	58538030	2845	18	0.556	Promoter(<=1kb)	0	ALPK2	115701
508496	19p	1004711	1005532	822	8	0.625	Exon(exon3of9)	4292	GRIN3B	116444
508496	19p	4216913	4217959	1047	6	0.5	Exon(exon18of22)	7950	ANKRD24	170961
508496	19p	4510548	4513547	3000	23	0.478	Exon(exon3of6)	4157	PLIN4	729359
508496	19p	5455600	5456439	840	6	0.5	Promoter(<=1kb)	183	ZNRF4	148066
508496	19p	8948231	8953259	5029	16	0.562	Exon(exon3of84)	28083	MUC16	94025
508496	19p	8959403	8962066	2664	8	0.5	Exon(exon3of84)	19276	MUC16	94025
508496	19p	8964274	8967127	2854	22	0.682	Exon(exon3of84)	14215	MUC16	94025
508496	19p	8972467	8973623	1157	6	0.667	Exon(exon1of84)	7719	MUC16	94025

508496	19p	17282085	17284246	2162	8	0.5	Promoter(<=1kb)	0	ANKLE1	126549
508496	19p	18264798	18267409	2612	12	0.583	5'UTR	7002	IQCN	80726
508496	19p	21971930	21974500	2571	7	0.714	Exon(exon4of4)	14408	ZNF208	7757
508496	19p	22314389	22314427	39	8	0.125	Exon(exon4of4)	27948	ZNF729	100287226
508496	19p	23743906	23745300	1395	6	0.333	Exon(exon4of4)	13537	ZNF681	148213
508496	19q	39886005	39886439	435	8	0.625	Promoter(<=1kb)	853	FCGBP	8857
508496	19q	43846955	43848536	1582	6	0.833	3'UTR	13450	ZNF283	284349
508496	19q	43913423	43914878	1456	8	0.5	Exon(exon10of10)	4861	ZNF45	7596
508496	19q	44106512	44108078	1567	7	0	Exon(exon6of6)	-4103	ZNF225	7768
508496	19q	48873325	48875925	2601	12	0.583	Promoter(<=1kb)	904	PPP1R15A	23645
508496	19q	52437918	52439242	1325	8	0.5	Exon(exon4of4)	6504	ZNF534	147658
508496	19q	53164551	53166239	1689	6	0.167	Exon(exon4of4)	-5476	ZNF347	84671
508496	19q	55771923	55773169	1247	6	0.5	Promoter(2-3kb)	2782	RFPL4AL1	729974
508496	19q	55911888	55913077	1190	6	0.667	Exon(exon5of12)	19234	NLRP13	126204
508496	19q	58368013	58368875	863	8	0.5	Exon(exon3of3)	-5165	ZNF497	162968
508496	20p	5922421	5923394	974	8	0.5	Exon(exon4of5)	6923	CHGB	1114
508496	20q	63562677	63565531	2855	13	0.615	Promoter(1-2kb)	-1072	HELZ2	85441
508496	21q	44600627	44601692	1066	10	0.7	Promoter(<=1kb)	30	KRTAP10-7	386675
508496	21q	44637474	44638041	568	9	0.333	Promoter(<=1kb)	118	KRTAP10-10	353333
508496	22q	22352950	22353380	431	16	0.5	Exon(exon1of2)	30478	BMS1P20	96610
508496	22q	36191154	36191906	753	6	0.667	3'UTR	9971	APOL4	80832
508496	22q	36265284	36265796	513	6	0.833	Exon(exon6of6)	12120	APOL1	8542
508496	23p	8170039	8170243	205	7	0.571	Promoter(1-2kb)	1024	VCX2	51480
508496	23p	35802148	35803010	863	7	0.571	5'UTR	3357	MAGEB16	139604
509959	1p	16058491	16060000	1510	10	0.9	Exon(exon5of7)	6168	CLCNKB	1188
509959	1p	18481403	18482217	815	6	0.667	Promoter(<=1kb)	421	KLHDC7A	127707
509959	1p	40067594	40067675	82	6	0	Promoter(<=1kb)	324	CAP1	10487
509959	1p	89186388	89186419	32	9	0.556	Promoter(<=1kb)	107	GBP4	115361
509959	1q	152218469	152221375	2907	14	0.643	Promoter(2-3kb)	2818	HRNR	388697
509959	1q	152302822	152313891	11070	36	0.583	Promoter(<=1kb)	0	FLG-AS1	339400
509959	1q	158765805	158766655	851	6	0.5	Promoter(<=1kb)	47	OR6N1	128372
509959	1q	197101312	197101771	460	6	0.5	Exon(exon18of28)	33373	ASPM	259266
509959	1q	232805207	232806505	1299	6	0.333	Promoter(<=1kb)	315	MAP10	54627
509959	1q	236553659	236555893	2235	6	0.167	Exon(exon39of45)	10363	LGALS8	3964
509959	1q	247841312	247841582	271	6	0.833	Promoter(<=1kb)	314	OR11L1	391189
509959	2p	48580657	48582454	1798	7	0.571	Promoter(<=1kb)	0	STON1	11037
509959	2q	132783534	132785001	1468	7	0.429	Promoter(1-2kb)	-1511	NCKAP5	344148
509959	2q	217847583	217848559	977	6	0.833	Exon(exon19of33)	-5423	TNS1	7145
509959	2q	219271337	219271649	313	6	0.667	Exon(exon4of4)	6227	TUBA4A	7277
509959	2q	232408301	232409799	1499	6	1	Promoter(1-2kb)	1457	ALPG	251
509959	2q	233840612	233842185	1574	6	0.667	Promoter(<=1kb)	0	HJURP	55355
509959	2q	238130271	238131546	1276	7	0.286	Promoter(1-2kb)	1323	ESPNL	339768
509959	3p	31989532	31990905	1374	7	0.286	Exon(exon2of2)	7761	ZNF860	344787
509959	3p	75736880	75739007	2128	48	0.583	Promoter(<=1kb)	0	MIR4273	100422955
509959	3q	98264413	98265098	686	7	0.571	Promoter(<=1kb)	128	OR5H6	79295
509959	3q	194359607	194360906	1300	9	0.889	Exon(exon2of2)	-8279	CPN2	1370
509959	4p	5988383	5989749	1367	7	0.571	Promoter(<=1kb)	0	C4orf50	389197
509959	4p	6300792	6302360	1569	6	0.833	Exon(exon8of8)	6021	WFS1	7466
509959	4p	8227004	8228508	1505	7	0.143	Promoter(<=1kb)	-24	SH3TC1	54436
509959	4q	112431241	112432293	1053	7	0.429	3'UTR	4735	ALPK1	80216
509959	4q	185458217	185460011	1795	9	0.556	Promoter(<=1kb)	0	CCDC110	256309
509959	4q	186619481	186621582	2102	6	0.333	Exon(exon10of27)	-9411	FAT1	2195
509959	5q	79728956	79730716	1761	7	0.286	Exon(exon2of13)	-7426	CMYA5	202333
509959	5q	79731782	79734523	2742	13	0.308	Exon(exon2of13)	-3619	CMYA5	202333
509959	5q	140807352	140807737	386	6	0.833	Promoter(<=1kb)	271	PCDHA4	56144
509959	5q	140848579	140850786	2208	7	0.571	Promoter(<=1kb)	807	PCDHA9	9752
509959	5q	141174000	141174900	901	6	1	Promoter(1-2kb)	1356	PCDHB7	56129
509959	5q	141187690	141189425	1736	7	0.571	Promoter(<=1kb)	529	PCDHB9	56127
509959	6p	1312843	1313745	903	6	0.5	Promoter(<=1kb)	745	FOXQ1	94234
509959	6q	64591274	64591961	688	10	0.5	Exon(exon26of43)	121374	EYS	346007
509959	6q	159231899	159234370	2472	12	0.583	Exon(exon11of23)	13602	FNDC1	84624
509959	7p	45082725	45084866	2142	8	0.5	Promoter(1-2kb)	-1325	NACAD	23148
509959	7p	53035678	53036385	708	6	1	Promoter(<=1kb)	45	POM121L12	285877
509959	7q	64991278	64992758	1481	6	0.667	Promoter(<=1kb)	-242	ZNF117	51351
509959	7q	100958721	100960873	2153	58	0.431	Promoter(<=1kb)	756	MUC3A	4584
509959	7q	100991195	100992398	1204	8	0.625	Exon(exon5of15)	-20656	MUC12	10071
509959	7q	100995575	100995785	211	7	0.714	Exon(exon5of15)	-17269	MUC12	10071
509959	8p	10607375	10608261	887	6	0.5	Exon(exon4of4)	46882	RP1L1	94137
509959	8p	10609614	10610662	1049	8	0.5	Exon(exon4of4)	44481	RP1L1	94137
509959	8p	11331234	11332082	849	6	0.667	Promoter(<=1kb)	346	SLC35G5	83650
509959	8p	13021128	13022030	903	9	0.222	Exon(exon5of5)	9115	TRMT9B	57604
509959	8q	123651655	123652634	980	7	0.571	Promoter(<=1kb)	316	KLHL38	340359
509959	9p	21206764	21207074	311	6	0.5	Promoter(<=1kb)	69	IFNA10	3446
509959	9p	39078723	39078846	124	6	0.667	Exon(exon22of24)	7302	CNTNAP3	79937
509959	9q	76705179	76707804	2626	8	0.5	Promoter(<=1kb)	121	PCA3	50652
509959	9q	76709263	76710843	1581	8	0.5	Promoter(<=1kb)	0	PRUNE2	158471
509959	9q	87886533	87888536	2004	8	0.625	Exon(exon4of4)	3656	SPATA31E1	286234

509959	9q	122553263	122554071	809	8	0.5	Promoter(<=1kb)	93	OR1N2	138882
509959	9q	122628595	122629130	536	6	0.333	Promoter(<=1kb)	443	OR1B1	347169
509959	9q	122749914	122750547	634	6	0.833	Promoter(<=1kb)	174	OR1L6	392390
509959	9q	133255635	133256205	571	7	1	3'UTR	19009	ABO	28
509959	10q	46549378	46550723	1346	26	0.654	Exon(exon3of3)	4807	GPRIN2	9721
509959	10q	49323504	49326817	3314	10	0.5	Exon(exon3of3)	24230	C10orf71	118461
509959	10q	128103129	128104830	1702	10	0.6	Promoter(<=1kb)	-1	MKI67	4288
509959	11p	244106	244197	92	8	0.5	Promoter(<=1kb)	-232	PSMD13	5719
509959	11p	1241677	1243593	1917	6	0.333	Exon(exon31of49)	6083	MUC5B-AS1	112577518
509959	11p	1244757	1248605	3849	14	0.571	Promoter(1-2kb)	1071	MUC5B-AS1	112577518
509959	11p	1250091	1251628	1538	9	0.778	Promoter(<=1kb)	-415	MUC5B-AS1	112577518
509959	11p	5177978	5178478	501	6	0.167	Promoter(<=1kb)	186	OR52Z1	283110
509959	11p	5323362	5324256	895	7	0.429	Promoter(<=1kb)	41	OR51B2	79345
509959	11p	5389704	5390350	647	7	0.429	Promoter(<=1kb)	327	OR51M1	390059
509959	11p	5422212	5423123	912	10	0.7	Promoter(<=1kb)	101	OR51Q1	390061
509959	11p	5515185	5516015	831	6	0.333	Promoter(<=1kb)	684	UBQLNL	143630
509959	11p	5581045	5581738	694	8	0.375	Promoter(<=1kb)	168	OR52B6	340980
509959	11p	5841302	5841883	582	9	0.333	Promoter(<=1kb)	14	OR52E6	390078
509959	11p	5884818	5885061	244	7	0.429	Promoter(<=1kb)	547	OR52E4	390081
509959	11p	11351961	11352736	776	9	0.222	Promoter(<=1kb)	514	CSNK2A3	283106
509959	11p	12293639	12294538	900	8	0.625	Exon(exon29of35)	6739	MICALCL	84953
509959	11p	18173280	18173901	622	6	0.333	Promoter(<=1kb)	443	MRGPRX4	117196
509959	11q	58214757	58215722	966	8	0.25	Promoter(<=1kb)	12	OR1S1	219959
509959	11q	64116513	64118232	1720	7	0.714	Exon(exon2of2)	8702	MACROD1	28992
509959	11q	85724687	85725825	1139	6	0.5	Promoter(<=1kb)	0	SYTL2	54843
509959	11q	123906595	123907324	730	9	0.444	Promoter(<=1kb)	449	OR8D4	338662
509959	11q	124015600	124016477	878	7	0.143	Promoter(<=1kb)	25	OR10G4	390264
509959	11q	124038366	124038988	623	7	0.857	Promoter(<=1kb)	13	OR10G7	390265
509959	11q	124382526	124383285	760	8	0.625	Promoter(<=1kb)	58	OR8B2	26595
509959	12p	4626568	4628549	1982	11	0.455	Exon(exon5of6)	14051	DYRK4	8798
509959	12q	52571389	52573652	2264	7	0.571	Promoter(<=1kb)	164	KRT74	121391
509959	13q	25096659	25097231	573	8	0.5	Promoter(<=1kb)	791	PABPC3	5042
509959	13q	102732474	102733933	1460	6	0.333	Exon(exon4of4)	25139	CCDC168	643677
509959	14q	20060048	20060884	837	8	0.625	Promoter(<=1kb)	3	OR4L1	122742
509959	14q	20640750	20641567	818	7	0.571	Promoter(<=1kb)	124	OR6S1	341799
509959	14q	21634137	21634589	453	9	0.556	Promoter(<=1kb)	351	OR10G2	26534
509959	14q	63599622	63599735	114	13	0.462	Exon(exon2of2)	41672	WDR89	112840
509959	14q	70457532	70458540	1009	11	0.273	Exon(exon2of2)	5358	ADAM21	8747
509959	14q	94587512	94587839	328	6	0.5	Exon(exon2of2)	-4219	SERPINA3	12
509959	14q	104175275	104177810	2536	11	0.364	Exon(exon12of15)	36235	KIF26A	26153
509959	14q	104947943	104950338	2396	6	0.333	Exon(exon6of6)	5064	AHNAK2	113146
509959	15q	20534647	20535129	483	6	0.333	Exon(exon8of9)	6671	GOLGA6L6	727832
509959	15q	23439979	23442067	2089	12	0.583	5'UTR	5167	GOLGA6L2	283685
509959	15q	73702465	73703760	1296	6	0.833	Promoter(<=1kb)	-149	CD276	80381
509959	15q	99129423	99132517	3095	6	0.333	Exon(exon4of5)	7225	TTC23	64927
509959	16p	1228744	1229731	988	10	0.7	Promoter(<=1kb)	431	TPSB2	64499
509959	16p	1256345	1256980	636	6	0.833	Promoter(<=1kb)	276	TPSD1	23430
509959	16p	1998795	2000191	1397	7	0.571	Promoter(<=1kb)	0	ZNF598	90850
509959	16p	4206969	4208004	1036	7	0.571	Exon(exon2of7)	31739	SRL	6345
509959	16p	4883938	4885635	1698	6	1	Exon(exon22of22)	4701	PPL	5493
509959	16q	74391416	74391928	513	7	0.714	Exon(exon7of7)	13538	NPIPB15	440348
509959	16q	89226863	89228289	1427	7	0.571	Promoter(2-3kb)	2229	ZNF778	197320
509959	17p	744946	746966	2021	6	1	3'UTR	5072	GEMIN4	50628
509959	17p	10638198	10641099	2902	7	0.286	Exon(exon19of41)	-8169	MYH3	4621
509959	17p	21300581	21300978	398	12	0.75	3'UTR	9112	MAP2K3	5606
509959	17p	21415470	21416370	901	10	0.8	Exon(exon3of3)	10334	KCNJ12	3768
509959	17q	53823368	53824891	1524	6	0.833	Promoter(<=1kb)	441	KIF2B	84643
509959	17q	76293419	76294016	598	6	0.5	Promoter(2-3kb)	-2167	QRICH2	84074
509959	17q	81645135	81645417	283	6	0.333	Promoter(2-3kb)	2722	TSPAN10	83882
509959	18p	9887397	9888072	676	6	0.667	Promoter(1-2kb)	1383	TXNDC2	84203
509959	18p	11609646	11610581	936	11	0.636	Promoter(<=1kb)	50	SLC35G4	646000
509959	18q	58535186	58537515	2330	10	0.4	Promoter(<=1kb)	0	ALPK2	115701
509959	18q	75285495	75287404	1910	6	0.333	5'UTR	40919	TSHZ1	10194
509959	19p	4510548	4513547	3000	17	0.471	Exon(exon3of6)	4157	PLIN4	729359
509959	19p	8946313	8951868	5556	15	0.6	Exon(exon3of84)	29474	MUC16	94025
509959	19p	8959116	8962299	3184	10	0.7	Exon(exon3of84)	19043	MUC16	94025
509959	19p	8972751	8978096	5346	16	0.5	Exon(exon1of84)	3246	MUC16	94025
509959	19p	12430718	12432437	1720	8	0.375	3'UTR	8584	ZNF443	10224
509959	19p	14841061	14841331	271	7	0.571	Promoter(<=1kb)	546	OR7A10	390892
509959	19p	14880627	14881233	607	6	0.5	Promoter(<=1kb)	219	OR7A17	26333
509959	19p	15087213	15088040	828	9	0.333	Promoter(<=1kb)	233	OR1I1	126370
509959	19p	18264753	18267409	2657	14	0.643	5'UTR	7002	IQCN	80726
509959	19p	21971930	21974500	2571	7	0.714	Exon(exon4of4)	14408	ZNF208	7757
509959	19q	34943334	34944685	1352	6	0.333	3'UTR	9723	ZNF30	90075
509959	19q	37885190	37888806	3617	8	0.625	Exon(exon6of6)	17788	WDR87	83889
509959	19q	39877222	39877880	659	6	0.5	Exon(exon20of28)	9412	FCGBP	8857
509959	19q	39886240	39886422	183	6	0.667	Promoter(<=1kb)	870	FCGBP	8857

509959	19q	40394958	40395707	750	6	0.5	3'UTR	-4777	HIPK4	147746
509959	19q	43913423	43914878	1456	8	0.5	Exon(exon10of10)	4861	ZNF45	7596
509959	19q	44106512	44108078	1567	7	0	Exon(exon6of6)	-4103	ZNF225	7768
509959	19q	52437918	52439242	1325	7	0.429	Exon(exon4of4)	6504	ZNF534	147658
509959	19q	53164551	53166239	1689	6	0.167	Exon(exon4of4)	-5476	ZNF347	84671
509959	19q	55911888	55913077	1190	6	0.667	Exon(exon5of12)	19234	NLRP13	126204
509959	20p	5922421	5923394	974	6	0.5	Exon(exon4of5)	6923	CHGB	1114
509959	20p	20052354	20052736	383	6	0.167	Promoter(<=1kb)	0	CFAP61	26074
509959	20q	63349752	63350772	1021	6	0.5	3'UTR	3794	CHRNA4	1137
509959	20q	63561666	63565531	3866	11	0.636	Promoter(<=1kb)	-61	HELZ2	85441
509959	21q	36070334	36072675	2342	6	0.833	Promoter(<=1kb)	299	CBR1	873
509959	21q	44539312	44540035	724	6	0.833	Promoter(<=1kb)	160	KRTAP10-1	386677
509959	21q	44550835	44551416	582	6	0.833	Promoter(<=1kb)	89	KRTAP10-2	386679
509959	22q	22352950	22353380	431	16	0.5	Exon(exon1of2)	30478	BMS1P20	96610
509959	22q	22704568	22704813	246	6	1	Exon(exon2of2)	57790	GGTLC2	91227
509959	22q	36191154	36191906	753	6	0.667	3'UTR	9971	APOL4	80832
509959	23p	8170039	8170141	103	6	0.5	Promoter(1-2kb)	1126	VCX2	51480
509959	23p	35802148	35803010	863	7	0.571	5'UTR	3357	MAGEB16	139604
510286	1p	16058512	16060000	1489	7	0.714	Exon(exon5of7)	6189	CLCNKB	1188
510286	1p	18481403	18482217	815	6	0.667	Promoter(<=1kb)	421	KLHDC7A	127707
510286	1p	23874604	23875430	827	9	0.556	Exon(exon2of2)	-6310	FUCA1	2517
510286	1p	40067594	40067675	82	6	0	Promoter(<=1kb)	324	CAP1	10487
510286	1p	89186388	89186419	32	9	0.556	Promoter(<=1kb)	107	GBP4	115361
510286	1q	152213274	152213320	47	8	0.5	Exon(exon3of3)	10873	HRNR	388697
510286	1q	152218469	152221375	2907	16	0.688	Promoter(2-3kb)	2818	HRNR	388697
510286	1q	152303673	152313891	10219	41	0.585	Promoter(<=1kb)	0	FLG-AS1	339400
510286	1q	158765805	158766655	851	6	0.5	Promoter(<=1kb)	47	OR6N1	128372
510286	1q	201206099	201209342	3244	10	0.6	Promoter(1-2kb)	1017	IGFN1	91156
510286	1q	214640144	214642954	2811	12	0.5	Exon(exon12of20)	-5013	CENPF	1063
510286	1q	214644872	214647181	2310	10	0.4	Promoter(<=1kb)	-786	CENPF	1063
510286	1q	223393517	223394466	950	6	0.667	Promoter(<=1kb)	102	CCDC185	164127
510286	1q	226735563	226737239	1677	9	0.889	Promoter(<=1kb)	219	ITPKB	3707
510286	1q	228315976	228318038	2063	8	0.625	Exon(exon50of81)	6492	OBSCN	84033
510286	1q	247841312	247841582	271	6	0.833	Promoter(<=1kb)	314	OR11L1	391189
510286	1q	247949325	247949738	414	11	0.273	Promoter(<=1kb)	467	OR2L8	391190
510286	1q	248272863	248273670	808	6	0.333	Promoter(<=1kb)	166	OR2T33	391195
510286	1q	248294677	248295458	782	7	0.571	Promoter(<=1kb)	142	OR2T12	127064
510286	2p	48580657	48582454	1798	7	0.571	Promoter(<=1kb)	0	STON1	11037
510286	2q	132783061	132784972	1912	7	0.429	Promoter(1-2kb)	-1038	NCKAP5	344148
510286	2q	178739433	178741811	2379	6	0.5	Exon(exon45of191)	26014	TTN	7273
510286	2q	184936178	184937636	1459	6	0.333	Exon(exon4of4)	69813	ZNF804A	91752
510286	2q	185789865	185794632	4768	10	0.8	Promoter(<=1kb)	0	FSIP2	401024
510286	2q	185805377	185808170	2794	6	0.333	Promoter(<=1kb)	0	FSIP2	401024
510286	2q	217847583	217848746	1164	7	0.857	Exon(exon19of33)	-5423	TNS1	7145
510286	2q	237762685	237764060	1376	10	0.5	Exon(exon8of8)	-4137	LRRFIP1	9208
510286	2q	240041845	240042811	967	7	0.286	Downstream(2-3kb)	3261	OR6B3	150681
510286	3p	75736880	75739243	2364	53	0.585	Promoter(<=1kb)	0	MIR4273	100422955
510286	3q	98264413	98265137	725	8	0.625	Promoter(<=1kb)	128	OR5H6	79295
510286	4p	5988383	5989749	1367	7	0.571	Promoter(<=1kb)	0	C4orf50	389197
510286	4p	6300792	6302415	1624	7	0.857	Exon(exon8of8)	6021	WFS1	7466
510286	4p	8227004	8228508	1505	8	0.125	Promoter(<=1kb)	-24	SH3TC1	54436
510286	4q	87616226	87616370	145	11	0.455	Exon(exon5of5)	7697	DSPP	1834
510286	4q	185458217	185460011	1795	8	0.625	Promoter(<=1kb)	0	CCDC110	256309
510286	5q	79728956	79730716	1761	7	0.286	Exon(exon2of13)	-7426	CMYA5	202333
510286	5q	79731782	79734523	2742	13	0.308	Exon(exon2of13)	-3619	CMYA5	202333
510286	5q	83537326	83539905	2580	6	0.333	Promoter(1-2kb)	1712	VCAN	1462
510286	5q	140807352	140807737	386	6	0.833	Promoter(<=1kb)	271	PCDHA4	56144
510286	5q	140848579	140850786	2208	7	0.571	Promoter(<=1kb)	807	PCDHA9	9752
510286	5q	141100771	141102728	1958	8	0.25	Promoter(<=1kb)	298	PCDHB3	56132
510286	5q	141955676	141957660	1985	6	0.667	Promoter(<=1kb)	-668	RNF14	9604
510286	5q	151521550	151522069	520	6	0.667	Promoter(<=1kb)	79	MIR6499	102465246
510286	5q	151565922	151568158	2237	9	0.778	Promoter(<=1kb)	786	FAT2	2196
510286	6p	16327099	16328014	916	7	0.429	Exon(exon8of9)	37154	GMPR	2766
510286	6p	46858771	46859502	732	8	0.5	Exon(exon17of21)	3802	ADGRF5	221395
510286	6q	64591274	64591961	688	10	0.5	Exon(exon26of43)	121374	EYS	346007
510286	6q	149888581	149890867	2287	7	0.714	Promoter(<=1kb)	0	RAET1E-AS1	100652739
510286	6q	159231899	159234370	2472	11	0.545	Exon(exon11of23)	13602	FNDC1	84624
510286	7p	6330446	6330944	499	6	1	Exon(exon2of2)	7749	FAM220A	84792
510286	7p	12369637	12370736	1100	6	0.667	3'UTR	-13307	VWDE	221806
510286	7p	53035678	53036385	708	7	1	Promoter(<=1kb)	45	POM121L12	285877
510286	7p	56021087	56021209	123	6	0.5	Exon(exon2of7)	12947	PSPH	5723
510286	7q	100958977	100960873	1897	57	0.439	Promoter(1-2kb)	1012	MUC3A	4584
510286	7q	100991195	100993102	1908	9	0.667	Exon(exon5of15)	-19952	MUC12	10071
510286	7q	100995575	100995785	211	7	0.714	Exon(exon5of15)	-17269	MUC12	10071
510286	7q	101034361	101040583	6223	39	0.513	Exon(exon3of12)	-3128	MUC17	140453
510286	7q	149818015	149819792	1778	6	0.667	Promoter(2-3kb)	-2352	SSPO	23145
510286	7q	156949807	156950570	764	6	0.833	Promoter(<=1kb)	95	NOM1	64434

510286	8p	10607375	10608261	887	6	0.5	Exon(exon4of4)	46882	RP1L1	94137
510286	8p	11331234	11332082	849	6	0.667	Promoter(<=1kb)	346	SLC35G5	83650
510286	8p	12132686	12133940	1255	6	0.667	Promoter(<=1kb)	498	USP17L7	392197
510286	8p	13021128	13022030	903	7	0.143	Exon(exon5of5)	9115	TRMT9B	57604
510286	8q	123651655	123652634	980	7	0.571	Promoter(<=1kb)	316	KLHL38	340359
510286	8q	143916351	143919209	2859	8	0.25	Exon(exon32of32)	20381	PLEC	5339
510286	8q	143923488	143925516	2029	9	0.667	Exon(exon31of32)	14074	PLEC	5339
510286	9p	116800	117934	1135	6	1	Promoter(<=1kb)	270	FOXD4	2298
510286	9p	21206764	21207038	275	6	0.667	Promoter(<=1kb)	105	IFNA10	3446
510286	9q	76175237	76175271	35	7	0.857	Exon(exon14of14)	-13368	PCSK5	5125
510286	9q	76705724	76707804	2081	6	0.5	Promoter(<=1kb)	666	PCA3	50652
510286	9q	104504315	104505071	757	6	0.5	Promoter(<=1kb)	52	OR13F1	138805
510286	9q	122553263	122554071	809	7	0.429	Promoter(<=1kb)	93	OR1N2	138882
510286	9q	122628595	122629398	804	6	0.333	Promoter(<=1kb)	175	OR1B1	347169
510286	9q	122749914	122750547	634	6	0.833	Promoter(<=1kb)	174	OR1L6	392390
510286	9q	133255635	133256205	571	7	1	3'UTR	19009	ABO	28
510286	9q	135547960	135548795	836	8	0.625	Promoter(1-2kb)	1805	OBP2A	29991
510286	10q	46549378	46550723	1346	25	0.64	Exon(exon3of3)	4807	GPRIN2	9721
510286	10q	49323504	49325572	2069	7	0.429	Exon(exon3of3)	24230	C10orf71	118461
510286	10q	128103129	128104830	1702	10	0.6	Promoter(<=1kb)	-1	MKI67	4288
510286	11p	244106	244197	92	8	0.5	Promoter(<=1kb)	-232	PSMD13	5719
510286	11p	1194354	1196902	2549	7	0.571	Exon(exon34of49)	-26164	MUC5B	727897
510286	11p	1246332	1248605	2274	11	0.455	Promoter(1-2kb)	1071	MUC5B-AS1	112577518
510286	11p	5177811	5178478	668	7	0.143	Promoter(<=1kb)	186	OR52Z1	283110
510286	11p	5389704	5390350	647	7	0.429	Promoter(<=1kb)	327	OR51M1	390059
510286	11p	5422212	5423123	912	11	0.636	Promoter(<=1kb)	101	OR51Q1	390061
510286	11p	5515185	5516015	831	6	0.333	Promoter(<=1kb)	684	UBQLNL	143630
510286	11p	5581045	5581738	694	8	0.375	Promoter(<=1kb)	168	OR52B6	340980
510286	11p	5884818	5885061	244	6	0.333	Promoter(<=1kb)	547	OR52E4	390081
510286	11p	11351961	11352736	776	8	0.25	Promoter(<=1kb)	514	CSNK2A3	283106
510286	11p	12293639	12294538	900	8	0.625	Exon(exon29of35)	6739	MICALCL	84953
510286	11p	43942293	43943348	1056	8	0.75	Promoter(<=1kb)	0	C11orf96	387763
510286	11q	58214757	58215722	966	9	0.222	Promoter(<=1kb)	12	OR1S1	219959
510286	11q	85724687	85725825	1139	6	0.5	Promoter(<=1kb)	0	SYTL2	54843
510286	11q	123906790	123907324	535	6	0.667	Promoter(<=1kb)	644	OR8D4	338662
510286	11q	123943088	123943782	695	7	0.571	Promoter(<=1kb)	91	OR6T1	219874
510286	11q	124015601	124016477	877	10	0.2	Promoter(<=1kb)	26	OR10G4	390264
510286	11q	124023038	124023849	812	9	0.444	Promoter(<=1kb)	25	OR10G9	219870
510286	11q	124038366	124038988	623	9	0.889	Promoter(<=1kb)	13	OR10G7	390265
510286	12p	4626571	4628549	1979	10	0.5	Exon(exon5of6)	14054	DYRK4	8798
510286	12p	8222174	8223514	1341	6	0.667	Exon(exon5of6)	4073	FAM90A1	55138
510286	12q	52316096	52317765	1670	6	0.667	Exon(exon4of9)	3633	KRT83	3889
510286	13q	24434450	24435347	898	7	0.571	Exon(exon31of34)	19787	PARP4	143
510286	13q	25096429	25097182	754	16	0.438	Promoter(<=1kb)	561	PABPC3	5042
510286	13q	49668389	49668431	43	6	0.167	Exon(exon3of3)	23007	EBPL	84650
510286	13q	102732474	102733933	1460	6	0.333	Exon(exon4of4)	25139	CCDC168	643677
510286	14q	20060048	20060884	837	8	0.625	Promoter(<=1kb)	3	OR4L1	122742
510286	14q	21634137	21634589	453	9	0.556	Promoter(<=1kb)	351	OR10G2	26534
510286	14q	44504986	44506403	1418	7	0.714	Promoter(<=1kb)	880	FSCB	84075
510286	14q	70457532	70458540	1009	10	0.4	Exon(exon2of2)	5358	ADAM21	8747
510286	14q	104939262	104942618	3357	10	0.2	5'UTR	7102	PLD4	122618
510286	14q	104943622	104946886	3265	10	0.6	Exon(exon6of6)	8516	AHNAK2	113146
510286	14q	104947901	104954103	6203	44	0.5	Promoter(1-2kb)	1299	AHNAK2	113146
510286	15q	20534480	20535129	650	7	0.286	Exon(exon8of9)	6671	GOLGA6L6	727832
510286	15q	23439979	23442067	2089	14	0.5	5'UTR	5167	GOLGA6L2	283685
510286	15q	40621642	40623696	2055	6	0.333	Promoter(<=1kb)	0	KNL1	57082
510286	15q	59206980	59208056	1077	7	0.714	Promoter(<=1kb)	137	LDHAL6B	92483
510286	15q	73702465	73703760	1296	6	0.833	Promoter(<=1kb)	-149	CD276	80381
510286	15q	88857108	88859365	2258	7	0.143	Exon(exon12of18)	9865	ACAN	176
510286	16p	1228744	1229622	879	10	0.8	Promoter(<=1kb)	540	TPSB2	64499
510286	16p	1256345	1256896	552	6	0.667	Promoter(<=1kb)	276	TPSD1	23430
510286	16p	1486371	1488463	2093	8	0.75	Promoter(<=1kb)	4	PTX4	390667
510286	16p	4207130	4208004	875	6	0.5	Exon(exon2of7)	31739	SRL	6345
510286	16p	28495551	28497395	1845	6	0.333	Promoter(<=1kb)	0	CLN3	1201
510286	16q	84178965	84180270	1306	6	0.833	Promoter(1-2kb)	1148	TAF1C	9013
510286	16q	88428416	88429600	1185	6	0.333	Exon(exon3of3)	-23680	ZFPM1	161882
510286	16q	88714632	88717113	2482	7	0.429	Promoter(<=1kb)	0	MIR4722	100616167
510286	16q	89226883	89228390	1508	8	0.625	Promoter(2-3kb)	2249	ZNF778	197320
510286	17p	21300581	21300978	398	12	0.75	3'UTR	9112	MAP2K3	5606
510286	17q	81510690	81511591	902	6	0.667	Promoter(<=1kb)	257	ACTG1	71
510286	17q	81645135	81645607	473	8	0.375	Promoter(2-3kb)	2722	TSPAN10	83882
510286	18p	11609728	11610350	623	9	0.889	Promoter(<=1kb)	132	SLC35G4	646000
510286	18p	11644378	11644724	347	7	0.857	Exon(exon1of1)	10217	MIR7153	102465690
510286	18q	58535186	58537515	2330	10	0.4	Promoter(<=1kb)	0	ALPK2	115701
510286	19p	1004688	1005532	845	6	0.5	Exon(exon3of9)	4269	GRIN3B	116444
510286	19p	4510548	4513547	3000	22	0.455	Exon(exon3of6)	4157	PLIN4	729359
510286	19p	5455600	5456439	840	7	0.571	Promoter(<=1kb)	183	ZNRF4	148066

510286	19p	8948231	8950136	1906	6	0.667	Exon(exon3of84)	31206	MUC16	94025
510286	19p	8959518	8962066	2549	6	0.333	Exon(exon3of84)	19276	MUC16	94025
510286	19p	8964274	8967127	2854	19	0.632	Exon(exon3of84)	14215	MUC16	94025
510286	19p	12430718	12432437	1720	8	0.375	3'UTR	8584	ZNF443	10224
510286	19p	14841061	14841331	271	6	0.5	Promoter(<=1kb)	546	OR7A10	390892
510286	19p	18264798	18267409	2612	12	0.583	5'UTR	7002	IQCN	80726
510286	19p	21971930	21974500	2571	7	0.714	Exon(exon4of4)	14408	ZNF208	7757
510286	19q	37885190	37888806	3617	8	0.625	Exon(exon6of6)	17788	WDR87	83889
510286	19q	39877222	39877880	659	6	0.5	Exon(exon20of28)	9412	FCGBP	8857
510286	19q	39886240	39886422	183	6	0.667	Promoter(<=1kb)	870	FCGBP	8857
510286	19q	40880231	40880622	392	6	0.333	Promoter(<=1kb)	-141	CYP2A7	1549
510286	19q	43913423	43914878	1456	8	0.5	Exon(exon10of10)	4861	ZNF45	7596
510286	19q	44106512	44108078	1567	8	0.125	Exon(exon6of6)	-4103	ZNF225	7768
510286	19q	44327836	44329698	1863	6	0.5	Exon(exon4of4)	-22790	ZNF235	9310
510286	19q	51745958	51746963	1006	6	0.5	Exon(exon3of3)	3848	FPR1	2357
510286	19q	52437918	52439242	1325	7	0.429	Exon(exon4of4)	6504	ZNF534	147658
510286	19q	53164551	53166239	1689	6	0.167	Exon(exon4of4)	-5476	ZNF347	84671
510286	19q	55358379	55359651	1273	6	0.667	Promoter(<=1kb)	0	FAM71E2	284418
510286	19q	55517821	55518642	822	10	1	Exon(exon14of14)	17661	SBK2	646643
510286	20q	62812284	62813345	1062	7	0.857	Promoter(2-3kb)	-2899	COL9A3	1299
510286	20q	63561666	63565531	3866	12	0.667	Promoter(<=1kb)	-61	HELZ2	85441
510286	21q	26843740	26844859	1120	6	0.667	Promoter(<=1kb)	0	ADAMTS1	9510
510286	21q	44550835	44551416	582	6	0.833	Promoter(<=1kb)	89	KRTAP10-2	386679
510286	21q	44600627	44601692	1066	10	0.7	Promoter(<=1kb)	30	KRTAP10-7	386675
510286	22q	22352950	22353380	431	16	0.5	Exon(exon1of2)	30478	BMS1P20	96610
510286	22q	36191154	36191906	753	7	0.714	3'UTR	9971	APOL4	80832
510286	23p	3320126	3323750	3625	10	0.6	Exon(exon5of7)	22902	MXRA5	25878
510286	23p	8170039	8170141	103	6	0.5	Promoter(1-2kb)	1126	VCX2	51480
510286	23p	35802148	35803010	863	7	0.571	5'UTR	3357	MAGEB16	139604
510286	23q	102937373	102937765	393	7	0.571	Promoter(<=1kb)	101	RAB40AL	282808
510590	1p	16058491	16060000	1510	13	0.846	Exon(exon5of7)	6168	CLCNKB	1188
510590	1p	18481403	18482217	815	6	0.667	Promoter(<=1kb)	421	KLHDC7A	127707
510590	1p	40067594	40067675	82	6	0	Promoter(<=1kb)	324	CAP1	10487
510590	1p	89186388	89186419	32	9	0.556	Promoter(<=1kb)	107	GBP4	115361
510590	1q	152213286	152213347	62	8	0.5	Exon(exon3of3)	10846	HRNR	388697
510590	1q	152219233	152221375	2143	15	0.733	Promoter(2-3kb)	2818	HRNR	388697
510590	1q	158765805	158766655	851	6	0.5	Promoter(<=1kb)	47	OR6N1	128372
510590	1q	201206099	201209342	3244	9	0.667	Promoter(1-2kb)	1017	IGFN1	91156
510590	1q	201210956	201212866	1911	6	0.333	Promoter(<=1kb)	0	IGFN1	91156
510590	1q	228315976	228318038	2063	9	0.667	Exon(exon50of81)	6492	OBSCN	84033
510590	1q	232805117	232806800	1684	6	0.5	Promoter(<=1kb)	225	MAP10	54627
510590	1q	247841312	247841582	271	6	0.833	Promoter(<=1kb)	314	OR11L1	391189
510590	1q	248294677	248295458	782	8	0.625	Promoter(<=1kb)	142	OR2T12	127064
510590	2q	167246794	167248478	1685	7	0.714	Promoter(<=1kb)	-204	XIRP2	129446
510590	2q	178739433	178741811	2379	6	0.5	Exon(exon45of191)	26014	TTN	7273
510590	2q	184936178	184937636	1459	6	0.333	Exon(exon4of4)	69813	ZNF804A	91752
510590	2q	217847567	217848213	647	6	0.833	Exon(exon19of33)	-5407	TNS1	7145
510590	2q	237762685	237764060	1376	10	0.5	Exon(exon8of8)	-4137	LRRFIP1	9208
510590	2q	240041845	240042154	310	6	0.167	Downstream(2-3kb)	3918	OR6B3	150681
510590	3p	13637703	13638208	506	6	0.833	Promoter(1-2kb)	1258	FBLN2	2199
510590	3p	75736880	75738859	1980	48	0.604	Promoter(<=1kb)	0	MIR4273	100422955
510590	3q	98264413	98265137	725	8	0.625	Promoter(<=1kb)	128	OR5H6	79295
510590	4p	5988383	5989749	1367	7	0.571	Promoter(<=1kb)	0	C4orf50	389197
510590	4p	6300792	6302360	1569	6	0.833	Exon(exon8of8)	6021	WFS1	7466
510590	4q	121036404	121037542	1139	6	0.333	Promoter(1-2kb)	1442	NDNF	79625
510590	5p	795818	796218	401	6	0.667	3'UTR	4927	ZDHHC11	79844
510590	5q	140807352	140807737	386	6	0.833	Promoter(<=1kb)	271	PCDHA4	56144
510590	5q	141174000	141175025	1026	6	0.833	Promoter(1-2kb)	1356	PCDHB7	56129
510590	5q	141955356	141957660	2305	6	0.667	Promoter(<=1kb)	-668	RNF14	9604
510590	5q	151565922	151568158	2237	9	0.778	Promoter(<=1kb)	786	FAT2	2196
510590	6p	1312843	1313745	903	6	0.5	Promoter(<=1kb)	745	FOXQ1	94234
510590	6p	46858771	46859502	732	8	0.5	Exon(exon17of21)	3802	ADGRF5	221395
510590	7p	6330446	6330944	499	6	1	Exon(exon2of2)	7749	FAM220A	84792
510590	7q	64991321	64992758	1438	6	0.667	Promoter(<=1kb)	-285	ZNF117	51351
510590	7q	100958721	100960873	2153	57	0.474	Promoter(<=1kb)	756	MUC3A	4584
510590	7q	100991195	100993127	1933	9	0.667	Exon(exon5of15)	-19927	MUC12	10071
510590	7q	100995575	100995785	211	6	0.833	Exon(exon5of15)	-17269	MUC12	10071
510590	7q	101036085	101038481	2397	7	0.429	Exon(exon3of12)	-5230	MUC17	140453
510590	8p	8376561	8377994	1434	6	1	Exon(exon2of5)	3753	PRAG1	157285
510590	8p	10607375	10608261	887	6	0.5	Exon(exon4of4)	46882	RP1L1	94137
510590	8p	12132686	12133940	1255	6	0.833	Promoter(<=1kb)	498	USP17L7	392197
510590	8p	12137448	12138641	1194	7	0.857	Promoter(<=1kb)	436	USP17L2	377630
510590	8p	13021128	13022030	903	8	0.25	Exon(exon5of5)	9115	TRMT9B	57604
510590	9q	76705724	76707804	2081	6	0.5	Promoter(<=1kb)	666	PCA3	50652
510590	9q	104504315	104505071	757	6	0.5	Promoter(<=1kb)	52	OR13F1	138805
510590	9q	104598545	104599361	817	12	0.583	Promoter(<=1kb)	52	OR13C5	138799
510590	9q	122553263	122554071	809	7	0.429	Promoter(<=1kb)	93	OR1N2	138882

510590	9q	122749914	122750547	634	6	0.833	Promoter(<=1kb)	174	OR1L6	392390
510590	10q	46549378	46550723	1346	25	0.64	Exon(exon3of3)	4807	GPRIN2	9721
510590	10q	49323169	49325572	2404	8	0.5	Exon(exon3of3)	23895	C10orf71	118461
510590	11p	244106	244197	92	8	0.5	Promoter(<=1kb)	-232	PSMD13	5719
510590	11p	1246095	1248605	2511	11	0.545	Promoter(1-2kb)	1071	MUC5B-AS1	112577518
510590	11p	1250091	1251628	1538	6	0.833	Promoter(<=1kb)	-415	MUC5B-AS1	112577518
510590	11p	5177811	5178478	668	7	0.143	Promoter(<=1kb)	186	OR52Z1	283110
510590	11p	5323362	5324256	895	7	0.429	Promoter(<=1kb)	41	OR51B2	79345
510590	11p	5389704	5390350	647	7	0.429	Promoter(<=1kb)	327	OR51M1	390059
510590	11p	5402638	5403471	834	11	0.455	Promoter(<=1kb)	41	OR51J1	79470
510590	11p	5422212	5423123	912	10	0.7	Promoter(<=1kb)	101	OR51Q1	390061
510590	11p	5515185	5515931	747	6	0.333	Promoter(<=1kb)	768	UBQLNL	143630
510590	11p	5581208	5581738	531	6	0.333	Promoter(<=1kb)	331	OR52B6	340980
510590	11p	5841302	5841883	582	9	0.333	Promoter(<=1kb)	14	OR52E6	390078
510590	11p	5986042	5986542	501	6	1	Promoter(<=1kb)	443	OR52L1	338751
510590	11p	11351961	11352736	776	9	0.222	Promoter(<=1kb)	514	CSNK2A3	283106
510590	11p	43942293	43943348	1056	8	0.875	Promoter(<=1kb)	0	C11orf96	387763
510590	11q	58214757	58215722	966	7	0.286	Promoter(<=1kb)	12	OR1S1	219959
510590	11q	58402523	58403265	743	8	0.5	Promoter(<=1kb)	144	OR5B3	441608
510590	11q	66560202	66562261	2060	6	0.333	Exon(exon14of21)	3873	CTSF	8722
510590	11q	85724687	85725825	1139	6	0.5	Promoter(<=1kb)	0	SYTL2	54843
510590	11q	123906595	123907324	730	9	0.444	Promoter(<=1kb)	449	OR8D4	338662
510590	11q	124015600	124016477	878	7	0.143	Promoter(<=1kb)	25	OR10G4	390264
510590	11q	124038366	124038988	623	9	0.889	Promoter(<=1kb)	13	OR10G7	390265
510590	12p	4627329	4628549	1221	6	0.5	Exon(exon5of6)	14812	DYRK4	8798
510590	12p	6453119	6453670	552	6	0.667	Promoter(<=1kb)	633	TAPBPL	55080
510590	12q	49795819	49797137	1319	6	0.5	Promoter(<=1kb)	0	NCKAP5L	57701
510590	12q	52316096	52317765	1670	6	0.667	Exon(exon4of9)	3633	KRT83	3889
510590	13q	25096870	25097182	313	6	0.333	Promoter(1-2kb)	1002	PABPC3	5042
510590	13q	102732474	102733933	1460	6	0.333	Exon(exon4of4)	25139	CCDC168	643677
510590	14q	19975713	19976448	736	9	0.444	Promoter(<=1kb)	269	OR4K15	81127
510590	14q	20060048	20060884	837	8	0.625	Promoter(<=1kb)	3	OR4L1	122742
510590	14q	20143999	20144604	606	8	0.5	Promoter(<=1kb)	263	OR4N5	390437
510590	14q	20640982	20641567	586	6	0.5	Promoter(<=1kb)	124	OR6S1	341799
510590	14q	21634137	21634644	508	7	0.429	Promoter(<=1kb)	296	OR10G2	26534
510590	14q	70457733	70458948	1216	8	0.5	Exon(exon2of2)	5559	ADAM21	8747
510590	14q	104947943	104950453	2511	11	0.364	Exon(exon6of6)	4949	AHNAK2	113146
510590	15q	23439979	23442067	2089	10	0.6	5'UTR	5167	GOLGA6L2	283685
510590	15q	40621642	40624039	2398	7	0.286	Promoter(<=1kb)	0	KNL1	57082
510590	15q	85579423	85582073	2651	16	0.625	Promoter(<=1kb)	-837	AKAP13	11214
510590	15q	99129423	99132517	3095	6	0.333	Exon(exon4of5)	7225	TTC23	64927
510590	16p	1486371	1488463	2093	8	0.75	Promoter(<=1kb)	4	PTX4	390667
510590	16p	27362551	27363079	529	6	0.167	3'UTR	7203	IL4R	3566
510590	16q	88428539	88431889	3351	12	0.333	Exon(exon3of3)	-21391	ZFPM1	161882
510590	16q	88433131	88436097	2967	6	0.667	Exon(exon3of3)	-17183	ZFPM1	161882
510590	16q	88714632	88717113	2482	7	0.429	Promoter(<=1kb)	0	MIR4722	100616167
510590	16q	89100686	89101050	365	7	0.571	Promoter(<=1kb)	24	ACSF3	197322
510590	16q	89226863	89228419	1557	9	0.556	Promoter(2-3kb)	2229	ZNF778	197320
510590	17p	744946	746966	2021	6	1	3'UTR	5072	GEMIN4	50628
510590	17p	10638198	10641099	2902	7	0.286	Exon(exon19of41)	-8169	MYH3	4621
510590	17p	21300581	21300978	398	12	0.75	3'UTR	9112	MAP2K3	5606
510590	17q	81645135	81645607	473	7	0.286	Promoter(2-3kb)	2722	TSPAN10	83882
510590	18q	58535186	58537515	2330	9	0.333	Promoter(<=1kb)	0	ALPK2	115701
510590	19p	1036457	1036914	458	6	0.5	Exon(exon6of7)	-3187	ABCA7	10347
510590	19p	4510548	4513547	3000	23	0.478	Exon(exon3of6)	4157	PLIN4	729359
510590	19p	5455600	5456439	840	6	0.5	Promoter(<=1kb)	183	ZNRF4	148066
510590	19p	8946313	8951868	5556	15	0.6	Exon(exon3of84)	29474	MUC16	94025
510590	19p	8959116	8962299	3184	10	0.7	Exon(exon3of84)	19043	MUC16	94025
510590	19p	8972751	8978096	5346	13	0.462	Exon(exon1of84)	3246	MUC16	94025
510590	19p	12430157	12432437	2281	11	0.364	3'UTR	8584	ZNF443	10224
510590	19p	15794192	15794719	528	6	0.333	Promoter(<=1kb)	241	OR10H5	284433
510590	19p	17281820	17284246	2427	9	0.556	Promoter(<=1kb)	0	ANKLE1	126549
510590	19p	18264753	18267409	2657	8	0.5	5'UTR	7002	IQCN	80726
510590	19p	21971930	21974500	2571	8	0.75	Exon(exon4of4)	14408	ZNF208	7757
510590	19q	34943334	34944685	1352	6	0.333	3'UTR	9723	ZNF30	90075
510590	19q	36996730	36997597	868	10	0.7	Exon(exon10of10)	5677	ZNF568	374900
510590	19q	37151928	37153149	1222	6	0.833	Exon(exon5of5)	19287	ZNF585A	199704
510590	19q	39877222	39877880	659	6	0.5	Exon(exon20of28)	9412	FCGBP	8857
510590	19q	43846955	43848536	1582	6	0.833	3'UTR	13450	ZNF283	284349
510590	19q	43913423	43914878	1456	9	0.556	Exon(exon10of10)	4861	ZNF45	7596
510590	19q	44106512	44108078	1567	7	0	Exon(exon6of6)	-4103	ZNF225	7768
510590	19q	48873325	48875925	2601	11	0.545	Promoter(<=1kb)	904	PPP1R15A	23645
510590	19q	52437918	52439242	1325	7	0.429	Exon(exon4of4)	6504	ZNF534	147658
510590	19q	53164551	53166239	1689	6	0.167	Exon(exon4of4)	-5476	ZNF347	84671
510590	19q	55771923	55773169	1247	6	0.5	Promoter(2-3kb)	2782	RFPL4AL1	729974
510590	19q	55911888	55913077	1190	6	0.667	Exon(exon5of12)	19234	NLRP13	126204
510590	20p	5922677	5923382	706	7	0.571	Exon(exon4of5)	7179	CHGB	1114

510590	20p	20052354	20052736	383	6	0.167	Promoter(<=1kb)	0	CFAP61	26074
510590	20q	63561666	63565531	3866	13	0.615	Promoter(<=1kb)	-61	HELZ2	85441
510590	21q	45455650	45456803	1154	6	0.667	Promoter(<=1kb)	119	COL18A1	80781
510590	22q	22352950	22353380	431	16	0.5	Exon(exon1of2)	30478	BMS1P20	96610
510590	22q	22758758	22759209	452	6	0.833	Exon(exon1of2)	-63567	MIR650	723778
510590	22q	36191154	36191906	753	6	0.667	3'UTR	9971	APOL4	80832
510590	22q	39100331	39102033	1703	6	0.833	Promoter(<=1kb)	52	APOBEC3H	164668
510590	23p	8170039	8170141	103	6	0.5	Promoter(1-2kb)	1126	VCX2	51480
510590	23p	35802148	35803010	863	7	0.571	5'UTR	3357	MAGEB16	139604
510590	23q	102937373	102937765	393	7	0.571	Promoter(<=1kb)	101	RAB40AL	282808
511534	1p	16058491	16060000	1510	10	0.9	Exon(exon5of7)	6168	CLCNKB	1188
511534	1p	18481403	18483159	1757	7	0.714	Promoter(<=1kb)	421	KLHDC7A	127707
511534	1p	23874604	23875430	827	8	0.5	Exon(exon2of2)	-6310	FUCA1	2517
511534	1p	40067594	40067675	82	6	0	Promoter(<=1kb)	324	CAP1	10487
511534	1p	89186388	89186419	32	9	0.556	Promoter(<=1kb)	107	GBP4	115361
511534	1q	152303673	152313891	10219	42	0.595	Promoter(<=1kb)	0	FLG-AS1	339400
511534	1q	158765805	158766655	851	6	0.5	Promoter(<=1kb)	47	OR6N1	128372
511534	1q	169542317	169542882	566	6	0.167	Exon(exon13of25)	-26572	F5	2153
511534	1q	214640144	214642954	2811	12	0.5	Exon(exon12of20)	-5013	CENPF	1063
511534	1q	214644872	214647181	2310	10	0.4	Promoter(<=1kb)	-786	CENPF	1063
511534	1q	222628664	222629929	1266	6	0.333	Promoter(<=1kb)	190	MIA3	375056
511534	1q	228315976	228318026	2051	6	0.5	Exon(exon50of81)	6492	OBSCN	84033
511534	1q	247841312	247841582	271	6	0.833	Promoter(<=1kb)	314	OR11L1	391189
511534	1q	247895625	247896410	786	6	0.5	Promoter(<=1kb)	38	OR2W3	343171
511534	1q	247921132	247921822	691	6	0.667	Promoter(<=1kb)	114	OR2T8	343172
511534	1q	247949325	247949759	435	8	0.375	Promoter(<=1kb)	467	OR2L8	391190
511534	1q	248273309	248273700	392	8	0.5	Promoter(<=1kb)	136	OR2T33	391195
511534	2p	48580657	48582454	1798	7	0.571	Promoter(<=1kb)	0	STON1	11037
511534	2q	102351547	102351902	356	7	0.429	Exon(exon11of11)	-4027	IL18R1	8809
511534	2q	130193975	130194181	207	6	0.667	Exon(exon4of5)	4258	TUBA3E	112714
511534	2q	132783534	132785001	1468	7	0.429	Promoter(1-2kb)	-1511	NCKAP5	344148
511534	2q	167249736	167251847	2112	6	0.333	Promoter(1-2kb)	1054	XIRP2	129446
511534	2q	184936178	184937636	1459	6	0.333	Exon(exon4of4)	69813	ZNF804A	91752
511534	2q	185789865	185794632	4768	11	0.727	Promoter(<=1kb)	0	FSIP2	401024
511534	2q	185805377	185807800	2424	6	0.333	Promoter(<=1kb)	0	FSIP2	401024
511534	2q	217847583	217848559	977	6	0.833	Exon(exon19of33)	-5423	TNS1	7145
511534	2q	238130416	238131546	1131	7	0.429	Promoter(1-2kb)	1468	ESPNL	339768
511534	2q	240041845	240042154	310	6	0.167	Downstream(2-3kb)	3918	OR6B3	150681
511534	3p	75736880	75739007	2128	47	0.638	Promoter(<=1kb)	0	MIR4273	100422955
511534	3q	98264413	98265098	686	7	0.571	Promoter(<=1kb)	128	OR5H6	79295
511534	3q	196947805	196948102	298	6	0.5	3'UTR	3828	PIGZ	80235
511534	4p	5988383	5989749	1367	7	0.571	Promoter(<=1kb)	0	C4orf50	389197
511534	4p	6300792	6302360	1569	6	0.833	Exon(exon8of8)	6021	WFS1	7466
511534	4p	7433331	7434512	1182	7	0.857	Promoter(<=1kb)	418	PSAPL1	768239
511534	4p	38774449	38775552	1104	9	0.556	Promoter(<=1kb)	644	TLR10	81793
511534	4q	121036404	121037542	1139	6	0.333	Promoter(1-2kb)	1442	NDNF	79625
511534	4q	154489498	154491312	1815	8	0.5	Promoter(<=1kb)	22	DCHS2	54798
511534	4q	185458217	185460011	1795	8	0.625	Promoter(<=1kb)	0	CCDC110	256309
511534	4q	186617176	186621582	4407	10	0.4	Exon(exon10of27)	-7106	FAT1	2195
511534	4q	186706638	186709436	2799	7	0.714	Exon(exon2of27)	14397	FAT1	2195
511534	5q	83537326	83539905	2580	6	0.333	Promoter(1-2kb)	1712	VCAN	1462
511534	5q	140848579	140850786	2208	6	0.5	Promoter(<=1kb)	807	PCDHA9	9752
511534	5q	151565922	151568158	2237	9	0.778	Promoter(<=1kb)	786	FAT2	2196
511534	6p	16326440	16327837	1398	7	0.429	Exon(exon8of9)	36495	GMPR	2766
511534	6p	26370344	26370479	136	6	0.5	Promoter(<=1kb)	0	BTN3A2	11118
511534	6p	46858771	46859389	619	6	0.5	Exon(exon17of21)	3915	ADGRF5	221395
511534	6q	64591274	64591961	688	10	0.5	Exon(exon26of43)	121374	EYS	346007
511534	6q	106511549	106512572	1024	6	0.667	Promoter(<=1kb)	0	CRYBG1	202
511534	6q	149888581	149890867	2287	7	0.714	Promoter(<=1kb)	0	RAET1E-AS1	100652739
511534	6q	159233455	159234370	916	10	0.5	Exon(exon11of23)	15158	FNDC1	84624
511534	7p	6330446	6330944	499	6	1	Exon(exon2of2)	7749	FAM220A	84792
511534	7p	12369637	12370736	1100	6	0.667	3'UTR	-13307	VWDE	221806
511534	7p	53035678	53036385	708	7	1	Promoter(<=1kb)	45	POM121L12	285877
511534	7q	100958977	100960873	1897	56	0.446	Promoter(1-2kb)	1012	MUC3A	4584
511534	7q	100991195	100992398	1204	8	0.625	Exon(exon5of15)	-20656	MUC12	10071
511534	7q	100995575	100995785	211	7	0.714	Exon(exon5of15)	-17269	MUC12	10071
511534	7q	101034361	101040583	6223	40	0.525	Exon(exon3of12)	-3128	MUC17	140453
511534	7q	149818015	149819792	1778	6	0.667	Promoter(2-3kb)	-2352	SSPO	23145
511534	8p	10609614	10610662	1049	6	0.667	Exon(exon4of4)	44481	RP1L1	94137
511534	8p	13021128	13022030	903	7	0.143	Exon(exon5of5)	9115	TRMT9B	57604
511534	8q	138151949	138153046	1098	6	0.667	Promoter(<=1kb)	0	FAM135B	51059
511534	8q	141466429	141467514	1086	9	0.778	3'UTR	29245	MROH5	389690
511534	8q	143916360	143919209	2850	7	0.143	Exon(exon32of32)	20381	PLEC	5339
511534	8q	143923488	143925516	2029	8	0.625	Exon(exon31of32)	14074	PLEC	5339
511534	9p	116800	117713	914	6	0.667	Promoter(<=1kb)	491	FOXD4	2298
511534	9p	38395931	38397218	1288	6	0.833	Exon(exon2of2)	3219	ALDH1B1	219
511534	9q	76175241	76175296	56	9	0.889	Exon(exon14of14)	-13343	PCSK5	5125

511534	9q	76703555	76706360	2806	8	0.5	Promoter(<=1kb)	0	PCA3	50652
511534	9q	104598545	104599361	817	12	0.583	Promoter(<=1kb)	52	OR13C5	138799
511534	9q	122553263	122554071	809	7	0.429	Promoter(<=1kb)	93	OR1N2	138882
511534	9q	122749914	122750547	634	6	0.833	Promoter(<=1kb)	174	OR1L6	392390
511534	9q	135484803	135487213	2411	9	0.333	Promoter(1-2kb)	1440	PPP1R26	9858
511534	9q	135547960	135548795	836	8	0.625	Promoter(1-2kb)	1805	OBP2A	29991
511534	10p	47663	48605	943	6	0.833	Promoter(<=1kb)	664	TUBB8	347688
511534	10q	46549378	46550723	1346	25	0.64	Exon(exon3of3)	4807	GPRIN2	9721
511534	10q	49323559	49326817	3259	10	0.4	Exon(exon3of3)	24285	C10orf71	118461
511534	10q	128103129	128104752	1624	8	0.5	Promoter(<=1kb)	-1	MKI67	4288
511534	11p	244106	244197	92	8	0.5	Promoter(<=1kb)	-232	PSMD13	5719
511534	11p	1194354	1196902	2549	7	0.571	Exon(exon34of49)	-26164	MUC5B	727897
511534	11p	1246332	1247378	1047	6	0.5	Promoter(2-3kb)	2298	MUC5B-AS1	112577518
511534	11p	5046754	5047432	679	7	0.571	Promoter(<=1kb)	228	OR52J3	119679
511534	11p	5177978	5178478	501	6	0.167	Promoter(<=1kb)	186	OR52Z1	283110
511534	11p	5323451	5324256	806	6	0.5	Promoter(<=1kb)	41	OR51B2	79345
511534	11p	5389704	5390350	647	7	0.429	Promoter(<=1kb)	327	OR51M1	390059
511534	11p	5422212	5423123	912	11	0.636	Promoter(<=1kb)	101	OR51Q1	390061
511534	11p	5581045	5581738	694	8	0.375	Promoter(<=1kb)	168	OR52B6	340980
511534	11p	5788000	5788760	761	8	0.75	Promoter(<=1kb)	56	OR52N1	79473
511534	11p	5841302	5841883	582	9	0.333	Promoter(<=1kb)	14	OR52E6	390078
511534	11p	11351961	11352736	776	8	0.25	Promoter(<=1kb)	514	CSNK2A3	283106
511534	11p	12293639	12294842	1204	7	0.714	Exon(exon29of35)	6739	MICALCL	84953
511534	11q	58214757	58215722	966	8	0.25	Promoter(<=1kb)	12	OR1S1	219959
511534	11q	85724687	85725825	1139	6	0.5	Promoter(<=1kb)	0	SYTL2	54843
511534	11q	93697503	93700096	2594	11	0.636	Promoter(<=1kb)	0	CEP295	85459
511534	11q	123906790	123907324	535	6	0.667	Promoter(<=1kb)	644	OR8D4	338662
511534	11q	123943088	123943782	695	7	0.571	Promoter(<=1kb)	91	OR6T1	219874
511534	11q	124015601	124016477	877	8	0.25	Promoter(<=1kb)	26	OR10G4	390264
511534	11q	124023038	124023849	812	9	0.444	Promoter(<=1kb)	25	OR10G9	219870
511534	11q	124038366	124038988	623	9	0.889	Promoter(<=1kb)	13	OR10G7	390265
511534	11q	130914501	130915409	909	10	0.7	Promoter(1-2kb)	1035	SNX19	399979
511534	12p	4626568	4628549	1982	11	0.455	Exon(exon5of6)	14051	DYRK4	8798
511534	12q	48525773	48526514	742	6	0.5	Promoter(<=1kb)	141	OR8S1	341568
511534	12q	52571389	52573652	2264	7	0.571	Promoter(<=1kb)	164	KRT74	121391
511534	13q	102732474	102734850	2377	7	0.286	Exon(exon4of4)	24222	CCDC168	643677
511534	14q	20060048	20060884	837	8	0.625	Promoter(<=1kb)	3	OR4L1	122742
511534	14q	70457532	70458540	1009	9	0.333	Exon(exon2of2)	5358	ADAM21	8747
511534	14q	77026548	77027532	985	6	0.5	Promoter(1-2kb)	1176	IRF2BPL	64207
511534	14q	104939262	104942618	3357	12	0.167	5'UTR	7102	PLD4	122618
511534	14q	104943622	104946886	3265	15	0.6	Exon(exon6of6)	8516	AHNAK2	113146
511534	14q	104947901	104953878	5978	40	0.475	Promoter(1-2kb)	1524	AHNAK2	113146
511534	15q	23439979	23442067	2089	14	0.5	5'UTR	5167	GOLGA6L2	283685
511534	15q	73702465	73703760	1296	6	0.833	Promoter(<=1kb)	-149	CD276	80381
511534	15q	79456261	79457965	1705	6	0.5	Promoter(<=1kb)	165	MINAR1	23251
511534	15q	85579423	85582073	2651	15	0.6	Promoter(<=1kb)	-837	AKAP13	11214
511534	15q	88854874	88855594	721	6	0.833	Exon(exon12of18)	7631	ACAN	176
511534	15q	88857108	88859365	2258	6	0	Exon(exon12of18)	9865	ACAN	176
511534	15q	99129423	99132517	3095	7	0.429	Exon(exon4of5)	7225	TTC23	64927
511534	16p	1256345	1256980	636	6	0.833	Promoter(<=1kb)	276	TPSD1	23430
511534	16p	1486322	1488463	2142	13	0.692	Promoter(<=1kb)	4	PTX4	390667
511534	16q	74391416	74392004	589	9	0.667	Exon(exon7of7)	13538	NPIPB15	440348
511534	16q	88428539	88429600	1062	6	0.333	Exon(exon3of3)	-23680	ZFPM1	161882
511534	16q	89226883	89228390	1508	8	0.625	Promoter(2-3kb)	2249	ZNF778	197320
511534	17p	21300581	21300978	398	11	0.727	3'UTR	9112	MAP2K3	5606
511534	17q	53823368	53824891	1524	6	0.833	Promoter(<=1kb)	441	KIF2B	84643
511534	17q	81645135	81645607	473	7	0.286	Promoter(2-3kb)	2722	TSPAN10	83882
511534	18q	58535186	58538030	2845	19	0.526	Promoter(<=1kb)	0	ALPK2	115701
511534	19p	4510548	4513547	3000	21	0.429	Exon(exon3of6)	4157	PLIN4	729359
511534	19p	5455600	5456439	840	7	0.571	Promoter(<=1kb)	183	ZNRF4	148066
511534	19p	8333830	8335281	1452	7	0.571	Promoter(<=1kb)	0	KANK3	256949
511534	19p	8937644	8939234	1591	6	0.667	Exon(exon5of84)	-41554	MUC16	94025
511534	19p	8946313	8951868	5556	19	0.632	Exon(exon3of84)	29474	MUC16	94025
511534	19p	8959116	8962299	3184	12	0.75	Exon(exon3of84)	19043	MUC16	94025
511534	19p	8971777	8978096	6320	16	0.562	Exon(exon1of84)	3246	MUC16	94025
511534	19p	17281820	17284246	2427	9	0.556	Promoter(<=1kb)	0	ANKLE1	126549
511534	19p	18264798	18267409	2612	12	0.583	5'UTR	7002	IQCN	80726
511534	19p	21971930	21974500	2571	7	0.714	Exon(exon4of4)	14408	ZNF208	7757
511534	19q	36996730	36997597	868	10	0.6	Exon(exon10of10)	5677	ZNF568	374900
511534	19q	37886924	37889059	2136	6	0.833	Exon(exon6of6)	17535	WDR87	83889
511534	19q	39886240	39886422	183	6	0.667	Promoter(<=1kb)	870	FCGBP	8857
511534	19q	43846955	43848536	1582	6	0.833	3'UTR	13450	ZNF283	284349
511534	19q	43913423	43914878	1456	7	0.429	Exon(exon10of10)	4861	ZNF45	7596
511534	19q	44106512	44108078	1567	7	0	Exon(exon6of6)	-4103	ZNF225	7768
511534	19q	52437918	52439242	1325	7	0.429	Exon(exon4of4)	6504	ZNF534	147658
511534	19q	55911888	55913166	1279	7	0.714	Exon(exon5of12)	19145	NLRP13	126204
511534	20p	5922421	5923394	974	9	0.556	Exon(exon4of5)	6923	CHGB	1114

511534	20q	62812284	62813587	1304	6	1	Promoter(2-3kb)	-2657	COL9A3	1299
511534	20q	63349752	63350772	1021	6	0.5	3'UTR	3794	CHRNA4	1137
511534	21q	26843740	26844859	1120	6	0.667	Promoter(<=1kb)	0	ADAMTS1	9510
511534	22q	36191154	36191906	753	6	0.667	3'UTR	9971	APOL4	80832
511534	22q	39100331	39102033	1703	6	0.833	Promoter(<=1kb)	52	APOBEC3H	164668
511534	22q	49884187	49884994	808	6	0.333	Exon(exon2of2)	22924	ALG12	79087
511534	23p	3320126	3323750	3625	10	0.6	Exon(exon5of7)	22902	MXRA5	25878
511534	23p	8170039	8170141	103	6	0.5	Promoter(1-2kb)	1126	VCX2	51480
511534	23p	8466313	8466392	80	7	0.429	Promoter(<=1kb)	887	VCX3B	425054
511534	23p	35802148	35803010	863	7	0.571	5'UTR	3357	MAGEB16	139604
512636	1p	978953	979847	895	6	0.5	Promoter(1-2kb)	1182	PERM1	84808
512636	1p	16058491	16060000	1510	10	0.9	Exon(exon5of7)	6168	CLCNKB	1188
512636	1p	40067594	40067675	82	6	0	Promoter(<=1kb)	324	CAP1	10487
512636	1p	89186388	89186419	32	9	0.556	Promoter(<=1kb)	107	GBP4	115361
512636	1q	152218469	152221375	2907	16	0.688	Promoter(2-3kb)	2818	HRNR	388697
512636	1q	152303673	152313891	10219	34	0.618	Promoter(<=1kb)	0	FLG-AS1	339400
512636	1q	158765805	158766655	851	6	0.5	Promoter(<=1kb)	47	OR6N1	128372
512636	1q	197101312	197101771	460	6	0.5	Exon(exon18of28)	33373	ASPM	259266
512636	1q	226735683	226737239	1557	7	0.857	Promoter(<=1kb)	219	ITPKB	3707
512636	1q	228315976	228318038	2063	8	0.625	Exon(exon50of81)	6492	OBSCN	84033
512636	1q	247841312	247841582	271	6	0.833	Promoter(<=1kb)	314	OR11L1	391189
512636	1q	247949325	247949738	414	11	0.273	Promoter(<=1kb)	467	OR2L8	391190
512636	1q	248273309	248273670	362	7	0.571	Promoter(<=1kb)	166	OR2T33	391195
512636	1q	248294677	248295458	782	7	0.571	Promoter(<=1kb)	142	OR2T12	127064
512636	1q	248681658	248682198	541	7	0.571	Promoter(<=1kb)	130	OR14I1	401994
512636	2p	48580657	48582454	1798	7	0.571	Promoter(<=1kb)	0	STON1	11037
512636	2q	102351547	102351902	356	7	0.429	Exon(exon11of11)	-4027	IL18R1	8809
512636	2q	178739433	178741811	2379	6	0.5	Exon(exon45of191)	26014	TTN	7273
512636	2q	185789865	185794632	4768	10	0.8	Promoter(<=1kb)	0	FSIP2	401024
512636	2q	185805377	185808170	2794	6	0.333	Promoter(<=1kb)	0	FSIP2	401024
512636	2q	217847567	217848213	647	6	0.833	Exon(exon19of33)	-5407	TNS1	7145
512636	2q	232378876	232380431	1556	7	0.429	Promoter(<=1kb)	125	ALPP	250
512636	2q	233681970	233682559	590	6	0.5	Promoter(<=1kb)	0	UGT1A7	54577
512636	2q	238130271	238131546	1276	7	0.286	Promoter(1-2kb)	1323	ESPNL	339768
512636	2q	240041845	240042154	310	6	0.167	Downstream(2-3kb)	3918	OR6B3	150681
512636	3p	31989532	31990905	1374	7	0.286	Exon(exon2of2)	7761	ZNF860	344787
512636	3p	75736880	75739243	2364	47	0.617	Promoter(<=1kb)	0	MIR4273	100422955
512636	3q	98169021	98169561	541	6	0.5	Exon(exon2of2)	19695	OR5H14	403273
512636	3q	98264413	98265098	686	7	0.571	Promoter(<=1kb)	128	OR5H6	79295
512636	3q	196947177	196948102	926	6	0.5	3'UTR	3828	PIGZ	80235
512636	4p	1394795	1395605	811	6	0.333	Exon(exon1of1)	9983	UVSSA	57654
512636	4p	5988383	5989749	1367	7	0.571	Promoter(<=1kb)	0	C4orf50	389197
512636	4p	6300792	6302360	1569	6	0.833	Exon(exon8of8)	6021	WFS1	7466
512636	4p	7433331	7434759	1429	7	0.857	Promoter(<=1kb)	171	PSAPL1	768239
512636	4p	10443803	10446224	2422	6	0.333	Exon(exon3of3)	10952	ZNF518B	85460
512636	4q	186619481	186621582	2102	6	0.333	Exon(exon10of27)	-9411	FAT1	2195
512636	4q	186706638	186708616	1979	7	0.571	Exon(exon2of27)	15217	FAT1	2195
512636	5q	83537326	83539905	2580	6	0.333	Promoter(1-2kb)	1712	VCAN	1462
512636	5q	140848579	140850786	2208	6	0.5	Promoter(<=1kb)	807	PCDHA9	9752
512636	5q	141100771	141102758	1988	11	0.273	Promoter(<=1kb)	298	PCDHB3	56132
512636	5q	141955676	141957660	1985	7	0.714	Promoter(<=1kb)	-668	RNF14	9604
512636	5q	148826877	148828070	1194	6	1	Promoter(1-2kb)	1632	ADRB2	154
512636	6p	46858771	46859502	732	8	0.5	Exon(exon17of21)	3802	ADGRF5	221395
512636	6q	106511549	106512572	1024	6	0.667	Promoter(<=1kb)	0	CRYBG1	202
512636	6q	149888581	149890867	2287	7	0.714	Promoter(<=1kb)	0	RAET1E-AS1	100652739
512636	7p	6330446	6330944	499	6	1	Exon(exon2of2)	7749	FAM220A	84792
512636	7p	45082865	45084866	2002	6	0.333	Promoter(1-2kb)	-1465	NACAD	23148
512636	7q	100958721	100960873	2153	59	0.441	Promoter(<=1kb)	756	MUC3A	4584
512636	7q	100991195	100993127	1933	8	0.625	Exon(exon5of15)	-19927	MUC12	10071
512636	7q	100995575	100995785	211	6	0.833	Exon(exon5of15)	-17269	MUC12	10071
512636	7q	101036085	101038481	2397	7	0.429	Exon(exon3of12)	-5230	MUC17	140453
512636	8p	10607245	10608432	1188	8	0.5	Exon(exon4of4)	46711	RP1L1	94137
512636	8p	11331234	11332082	849	6	0.667	Promoter(<=1kb)	346	SLC35G5	83650
512636	9p	116800	117934	1135	7	0.857	Promoter(<=1kb)	270	FOXD4	2298
512636	9p	34723747	34726824	3078	9	0.667	Promoter(2-3kb)	2664	FAM205A	259308
512636	9q	122553263	122554071	809	7	0.429	Promoter(<=1kb)	93	OR1N2	138882
512636	9q	122749914	122750547	634	6	0.833	Promoter(<=1kb)	174	OR1L6	392390
512636	10p	47663	48605	943	6	0.833	Promoter(<=1kb)	664	TUBB8	347688
512636	10q	46549378	46550744	1367	27	0.63	5'UTR	4786	GPRIN2	9721
512636	10q	128103129	128104830	1702	10	0.6	Promoter(<=1kb)	-1	MKI67	4288
512636	11p	244106	244197	92	8	0.5	Promoter(<=1kb)	-232	PSMD13	5719
512636	11p	1194354	1196902	2549	7	0.571	Exon(exon34of49)	-26164	MUC5B	727897
512636	11p	1246095	1251628	5534	18	0.667	Promoter(<=1kb)	0	MUC5B-AS1	112577518
512636	11p	5323451	5324256	806	7	0.571	Promoter(<=1kb)	41	OR51B2	79345
512636	11p	5351521	5352416	896	16	0.438	Promoter(<=1kb)	13	OR51B6	390058
512636	11p	5389704	5390350	647	7	0.429	Promoter(<=1kb)	327	OR51M1	390059
512636	11p	5422212	5423123	912	10	0.7	Promoter(<=1kb)	101	OR51Q1	390061

512636	11p	5581045	5581738	694	8	0.375	Promoter(<=1kb)	168	OR52B6	340980
512636	11p	5841302	5841883	582	9	0.333	Promoter(<=1kb)	14	OR52E6	390078
512636	11p	5884818	5885061	244	7	0.429	Promoter(<=1kb)	547	OR52E4	390081
512636	11p	11351961	11353022	1062	10	0.3	Promoter(<=1kb)	228	CSNK2A3	283106
512636	11p	12293639	12294368	730	6	0.833	Exon(exon29of35)	6739	MICALCL	84953
512636	11p	18173280	18173901	622	6	0.333	Promoter(<=1kb)	443	MRGPRX4	117196
512636	11q	58214757	58215722	966	8	0.25	Promoter(<=1kb)	12	OR1S1	219959
512636	11q	64116513	64118232	1720	7	0.714	Exon(exon2of2)	8702	MACROD1	28992
512636	11q	66560202	66562261	2060	6	0.333	Exon(exon14of21)	3873	CTSF	8722
512636	11q	82732630	82733184	555	6	0.833	Promoter(<=1kb)	680	FAM181B	220382
512636	11q	124015600	124016477	878	10	0.3	Promoter(<=1kb)	25	OR10G4	390264
512636	11q	124023038	124023849	812	10	0.4	Promoter(<=1kb)	25	OR10G9	219870
512636	12p	4626571	4628549	1979	9	0.556	Exon(exon5of6)	14054	DYRK4	8798
512636	12p	31981273	31982507	1235	6	0.5	Exon(exon4of6)	-4659	RESF1	55196
512636	12q	52571389	52573652	2264	7	0.571	Promoter(<=1kb)	164	KRT74	121391
512636	13q	24434450	24435347	898	7	0.571	Exon(exon31of34)	19787	PARP4	143
512636	13q	25096638	25097231	594	19	0.526	Promoter(<=1kb)	770	PABPC3	5042
512636	13q	102732474	102733933	1460	6	0.333	Exon(exon4of4)	25139	CCDC168	643677
512636	14q	20060048	20060884	837	8	0.625	Promoter(<=1kb)	3	OR4L1	122742
512636	14q	22633879	22634450	572	9	0.333	Exon(exon2of2)	32212	ABHD4	63874
512636	14q	70457733	70458948	1216	7	0.571	Exon(exon2of2)	5559	ADAM21	8747
512636	14q	94587512	94587839	328	6	0.5	Exon(exon2of2)	-4219	SERPINA3	12
512636	14q	104175275	104177810	2536	10	0.3	Exon(exon12of15)	36235	KIF26A	26153
512636	14q	104939262	104942618	3357	10	0.2	5'UTR	7102	PLD4	122618
512636	14q	104943622	104946886	3265	11	0.636	Exon(exon6of6)	8516	AHNAK2	113146
512636	14q	104947901	104950447	2547	16	0.5	Exon(exon6of6)	4955	AHNAK2	113146
512636	15q	20534501	20535014	514	6	0.5	Exon(exon8of9)	6786	GOLGA6L6	727832
512636	15q	23439979	23442067	2089	12	0.5	5'UTR	5167	GOLGA6L2	283685
512636	15q	40621642	40623696	2055	6	0.333	Promoter(<=1kb)	0	KNL1	57082
512636	16p	1228744	1229622	879	6	0.833	Promoter(<=1kb)	540	TPSB2	64499
512636	16p	1256345	1256816	472	6	0.833	Promoter(<=1kb)	276	TPSD1	23430
512636	16q	89100686	89101050	365	8	0.625	Promoter(<=1kb)	24	ACSF3	197322
512636	16q	89226863	89228419	1557	10	0.6	Promoter(2-3kb)	2229	ZNF778	197320
512636	17p	744917	746695	1779	7	0.857	3'UTR	5343	GEMIN4	50628
512636	17p	10638198	10641099	2902	7	0.286	Exon(exon19of41)	-8169	MYH3	4621
512636	17p	21300581	21300978	398	12	0.75	3'UTR	9112	MAP2K3	5606
512636	17q	81645135	81645607	473	7	0.286	Promoter(2-3kb)	2722	TSPAN10	83882
512636	18p	11609646	11610164	519	14	0.857	Promoter(<=1kb)	50	SLC35G4	646000
512636	18p	14542649	14543140	492	6	0.5	Promoter(<=1kb)	6	POTEC	388468
512636	18q	58535186	58538030	2845	19	0.526	Promoter(<=1kb)	0	ALPK2	115701
512636	19p	4510548	4511943	1396	15	0.533	Exon(exon3of6)	5761	PLIN4	729359
512636	19p	5455600	5456439	840	6	0.5	Promoter(<=1kb)	183	ZNRF4	148066
512636	19p	8333830	8334965	1136	6	0.667	Promoter(<=1kb)	0	KANK3	256949
512636	19p	8946313	8951868	5556	15	0.6	Exon(exon3of84)	29474	MUC16	94025
512636	19p	8959116	8962299	3184	10	0.7	Exon(exon3of84)	19043	MUC16	94025
512636	19p	8972751	8978096	5346	16	0.5	Exon(exon1of84)	3246	MUC16	94025
512636	19p	12430157	12431840	1684	9	0.444	3'UTR	9181	ZNF443	10224
512636	19p	15087213	15088040	828	10	0.3	Promoter(<=1kb)	233	OR1I1	126370
512636	19p	15794192	15794719	528	6	0.333	Promoter(<=1kb)	241	OR10H5	284433
512636	19p	18264753	18267409	2657	8	0.5	5'UTR	7002	IQCN	80726
512636	19p	21971930	21974500	2571	9	0.667	Exon(exon4of4)	14408	ZNF208	7757
512636	19p	22756294	22759533	3240	11	0.545	3'UTR	10449	ZNF99	7652
512636	19q	36996730	36997597	868	10	0.7	Exon(exon10of10)	5677	ZNF568	374900
512636	19q	37151928	37153149	1222	6	0.833	Exon(exon5of5)	19287	ZNF585A	199704
512636	19q	37885190	37888806	3617	9	0.667	Exon(exon6of6)	17788	WDR87	83889
512636	19q	39886240	39886422	183	6	0.667	Promoter(<=1kb)	870	FCGBP	8857
512636	19q	40875742	40878797	3056	8	0.125	Promoter(<=1kb)	0	CYP2A7	1549
512636	19q	40880231	40880622	392	6	0.333	Promoter(<=1kb)	-141	CYP2A7	1549
512636	19q	43846955	43848536	1582	6	0.833	3'UTR	13450	ZNF283	284349
512636	19q	43913423	43914878	1456	7	0.429	Exon(exon10of10)	4861	ZNF45	7596
512636	19q	44106512	44108078	1567	7	0	Exon(exon6of6)	-4103	ZNF225	7768
512636	19q	52437918	52439242	1325	7	0.429	Exon(exon4of4)	6504	ZNF534	147658
512636	19q	55911888	55913077	1190	6	0.667	Exon(exon5of12)	19234	NLRP13	126204
512636	19q	58368293	58368875	583	7	0.429	Exon(exon3of3)	-5445	ZNF497	162968
512636	20p	5922421	5923394	974	7	0.571	Exon(exon4of5)	6923	CHGB	1114
512636	20q	63561666	63565531	3866	11	0.636	Promoter(<=1kb)	-61	HELZ2	85441
512636	21q	41740862	41741990	1129	6	0.833	Exon(exon2of2)	4851	MIR6814	102465488
512636	21q	44600627	44601692	1066	10	0.7	Promoter(<=1kb)	30	KRTAP10-7	386675
512636	21q	44627276	44628008	733	6	0.667	Promoter(<=1kb)	183	KRTAP10-9	386676
512636	21q	44637474	44638143	670	8	0.5	Promoter(<=1kb)	118	KRTAP10-10	353333
512636	22q	22352950	22353298	349	13	0.538	Exon(exon1of2)	30478	BMS1P20	96610
512636	22q	36191154	36191906	753	6	0.667	3'UTR	9971	APOL4	80832
512636	23p	8170039	8170141	103	6	0.5	Promoter(1-2kb)	1126	VCX2	51480
512636	23p	35802148	35803010	863	7	0.571	5'UTR	3357	MAGEB16	139604
512636	23q	102937373	102937765	393	7	0.571	Promoter(<=1kb)	101	RAB40AL	282808
512965	1p	16058491	16060000	1510	10	0.9	Exon(exon5of7)	6168	CLCNKB	1188
512965	1p	18481403	18482217	815	6	0.667	Promoter(<=1kb)	421	KLHDC7A	127707

512965	1p	23874604	23875430	827	9	0.556	Exon(exon2of2)	-6310	FUCA1	2517
512965	1p	40067594	40067675	82	6	0	Promoter(<=1kb)	324	CAP1	10487
512965	1p	89186388	89186419	32	9	0.556	Promoter(<=1kb)	107	GBP4	115361
512965	1q	152218469	152221375	2907	16	0.688	Promoter(2-3kb)	2818	HRNR	388697
512965	1q	152303673	152313891	10219	38	0.605	Promoter(<=1kb)	0	FLG-AS1	339400
512965	1q	158765805	158766655	851	6	0.5	Promoter(<=1kb)	47	OR6N1	128372
512965	1q	169540901	169542882	1982	10	0.3	Exon(exon13of25)	-25156	F5	2153
512965	1q	197101312	197101771	460	6	0.5	Exon(exon18of28)	33373	ASPM	259266
512965	1q	201206099	201209342	3244	10	0.6	Promoter(1-2kb)	1017	IGFN1	91156
512965	1q	201210956	201212792	1837	6	0.333	Promoter(<=1kb)	0	IGFN1	91156
512965	1q	223393517	223394466	950	6	0.667	Promoter(<=1kb)	102	CCDC185	164127
512965	1q	228315976	228318026	2051	6	0.5	Exon(exon50of81)	6492	OBSCN	84033
512965	1q	232805207	232807473	2267	7	0.286	Promoter(<=1kb)	315	MAP10	54627
512965	1q	247841312	247841582	271	6	0.833	Promoter(<=1kb)	314	OR11L1	391189
512965	1q	247949325	247949738	414	10	0.3	Promoter(<=1kb)	467	OR2L8	391190
512965	2q	132783032	132785012	1981	7	0.571	Promoter(1-2kb)	-1009	NCKAP5	344148
512965	2q	178739433	178741811	2379	6	0.5	Exon(exon45of191)	26014	TTN	7273
512965	2q	185789865	185794632	4768	11	0.727	Promoter(<=1kb)	0	FSIP2	401024
512965	2q	185805377	185808170	2794	6	0.333	Promoter(<=1kb)	0	FSIP2	401024
512965	2q	217847583	217848559	977	6	0.833	Exon(exon19of33)	-5423	TNS1	7145
512965	2q	233681970	233682693	724	6	0.667	Promoter(<=1kb)	0	UGT1A7	54577
512965	2q	233713134	233713783	650	13	0.692	Promoter(<=1kb)	142	UGT1A5	54579
512965	2q	233840612	233842185	1574	6	0.667	Promoter(<=1kb)	0	HJURP	55355
512965	2q	238130416	238131546	1131	6	0.333	Promoter(1-2kb)	1468	ESPNL	339768
512965	2q	240041845	240042131	287	6	0.167	Downstream(2-3kb)	3941	OR6B3	150681
512965	3p	31989532	31990905	1374	7	0.286	Exon(exon2of2)	7761	ZNF860	344787
512965	3p	75736880	75739007	2128	54	0.648	Promoter(<=1kb)	0	MIR4273	100422955
512965	3q	98264413	98265137	725	8	0.625	Promoter(<=1kb)	128	OR5H6	79295
512965	3q	158732176	158732509	334	6	0.5	Promoter(<=1kb)	0	RARRES1	5918
512965	3q	194359607	194360906	1300	9	0.889	Exon(exon2of2)	-8279	CPN2	1370
512965	4p	5988383	5989749	1367	8	0.5	Promoter(<=1kb)	0	C4orf50	389197
512965	4p	6300792	6302360	1569	6	0.833	Exon(exon8of8)	6021	WFS1	7466
512965	4p	8227004	8228508	1505	8	0.125	Promoter(<=1kb)	-24	SH3TC1	54436
512965	4q	112431241	112432293	1053	7	0.429	3'UTR	4735	ALPK1	80216
512965	4q	154489498	154491312	1815	9	0.556	Promoter(<=1kb)	22	DCHS2	54798
512965	4q	186619481	186621582	2102	6	0.333	Exon(exon10of27)	-9411	FAT1	2195
512965	5p	795818	796261	444	7	0.714	3'UTR	4884	ZDHHC11	79844
512965	5q	79728956	79730716	1761	6	0.333	Exon(exon2of13)	-7426	CMYA5	202333
512965	5q	79731782	79734523	2742	13	0.308	Exon(exon2of13)	-3619	CMYA5	202333
512965	5q	83537326	83539905	2580	6	0.333	Promoter(1-2kb)	1712	VCAN	1462
512965	5q	140848579	140850786	2208	6	0.5	Promoter(<=1kb)	807	PCDHA9	9752
512965	5q	141100771	141102758	1988	11	0.273	Promoter(<=1kb)	298	PCDHB3	56132
512965	5q	151565922	151568158	2237	9	0.778	Promoter(<=1kb)	786	FAT2	2196
512965	6p	46858771	46859389	619	6	0.5	Exon(exon17of21)	3915	ADGRF5	221395
512965	6q	64591274	64591961	688	10	0.5	Exon(exon26of43)	121374	EYS	346007
512965	6q	159233455	159234370	916	10	0.5	Exon(exon11of23)	15158	FNDC1	84624
512965	7p	12369637	12370736	1100	6	0.667	3'UTR	-13307	VWDE	221806
512965	7p	38353718	38353991	274	8	0.625	Exon(exon2of2)	3699	TRG-AS1	100506776
512965	7p	53035678	53036385	708	7	1	Promoter(<=1kb)	45	POM121L12	285877
512965	7q	64991278	64992758	1481	6	0.667	Promoter(<=1kb)	-242	ZNF117	51351
512965	7q	100958977	100960873	1897	48	0.479	Promoter(1-2kb)	1012	MUC3A	4584
512965	7q	100991195	100992398	1204	9	0.667	Exon(exon5of15)	-20656	MUC12	10071
512965	7q	100995547	100995785	239	7	0.857	Exon(exon5of15)	-17269	MUC12	10071
512965	7q	101034275	101038994	4720	35	0.429	Exon(exon3of12)	-4717	MUC17	140453
512965	8p	8376561	8377860	1300	6	1	Exon(exon2of5)	3887	PRAG1	157285
512965	8p	10607245	10612307	5063	18	0.722	Exon(exon4of4)	42836	RP1L1	94137
512965	8p	11331234	11332082	849	6	0.667	Promoter(<=1kb)	346	SLC35G5	83650
512965	8p	12132686	12133940	1255	6	0.667	Promoter(<=1kb)	498	USP17L7	392197
512965	8p	13021128	13022030	903	8	0.25	Exon(exon5of5)	9115	TRMT9B	57604
512965	8p	17754377	17755366	990	6	0.5	Promoter(<=1kb)	0	MTUS1	57509
512965	8q	123651655	123652634	980	7	0.571	Promoter(<=1kb)	316	KLHL38	340359
512965	8q	142664633	142665852	1220	6	0.833	Exon(exon2of2)	4118	JRK	8629
512965	9p	116800	117934	1135	7	0.714	Promoter(<=1kb)	270	FOXD4	2298
512965	9p	712060	713307	1248	7	0.714	Exon(exon7of16)	5171	KANK1	23189
512965	9p	39078723	39078846	124	7	0.714	Exon(exon22of24)	7302	CNTNAP3	79937
512965	9q	76705724	76707804	2081	6	0.5	Promoter(<=1kb)	666	PCA3	50652
512965	9q	87886533	87888713	2181	8	0.625	Exon(exon4of4)	3656	SPATA31E1	286234
512965	9q	104504315	104505071	757	6	0.5	Promoter(<=1kb)	52	OR13F1	138805
512965	9q	122553278	122554071	794	6	0.333	Promoter(<=1kb)	108	OR1N2	138882
512965	9q	122749914	122750547	634	6	0.833	Promoter(<=1kb)	174	OR1L6	392390
512965	9q	135484803	135487213	2411	8	0.25	Promoter(1-2kb)	1440	PPP1R26	9858
512965	10q	46549378	46550723	1346	25	0.64	Exon(exon3of3)	4807	GPRIN2	9721
512965	10q	128102594	128106296	3703	8	0.625	Promoter(<=1kb)	0	MKI67	4288
512965	11p	244106	244197	92	8	0.5	Promoter(<=1kb)	-232	PSMD13	5719
512965	11p	1194354	1196902	2549	7	0.571	Exon(exon34of49)	-26164	MUC5B	727897
512965	11p	1246095	1247378	1284	8	0.375	Promoter(2-3kb)	2298	MUC5B-AS1	112577518
512965	11p	5177978	5178478	501	6	0.167	Promoter(<=1kb)	186	OR52Z1	283110

512965	11p	5323542	5324256	715	6	0.5	Promoter(<=1kb)	41	OR51B2	79345
512965	11p	5389704	5390350	647	6	0.5	Promoter(<=1kb)	327	OR51M1	390059
512965	11p	5422212	5423123	912	11	0.636	Promoter(<=1kb)	101	OR51Q1	390061
512965	11p	5440761	5441472	712	6	0.833	Promoter(<=1kb)	42	OR51I1	390063
512965	11p	5515185	5516015	831	6	0.333	Promoter(<=1kb)	684	UBQLNL	143630
512965	11p	5581045	5581738	694	8	0.375	Promoter(<=1kb)	168	OR52B6	340980
512965	11p	5884818	5885061	244	6	0.333	Promoter(<=1kb)	547	OR52E4	390081
512965	11p	5986042	5986542	501	6	1	Promoter(<=1kb)	443	OR52L1	338751
512965	11p	11351961	11352736	776	7	0.143	Promoter(<=1kb)	514	CSNK2A3	283106
512965	11p	12293639	12294368	730	7	0.857	Exon(exon29of35)	6739	MICALCL	84953
512965	11q	55572176	55572903	728	6	0.667	Promoter(<=1kb)	48	OR4C16	219428
512965	11q	58214757	58215722	966	8	0.25	Promoter(<=1kb)	12	OR1S1	219959
512965	11q	64315797	64315856	60	8	0.75	Promoter(1-2kb)	1485	TRMT112	51504
512965	11q	85724687	85725825	1139	6	0.5	Promoter(<=1kb)	0	SYTL2	54843
512965	11q	123906595	123907324	730	7	0.429	Promoter(<=1kb)	449	OR8D4	338662
512965	11q	124015600	124016477	878	10	0.3	Promoter(<=1kb)	25	OR10G4	390264
512965	11q	124023038	124023849	812	10	0.4	Promoter(<=1kb)	25	OR10G9	219870
512965	11q	124382526	124383285	760	9	0.667	Promoter(<=1kb)	58	OR8B2	26595
512965	11q	130914501	130915409	909	10	0.7	Promoter(1-2kb)	1035	SNX19	399979
512965	12p	4626568	4628549	1982	11	0.545	Exon(exon5of6)	14051	DYRK4	8798
512965	12p	6453119	6453670	552	6	0.667	Promoter(<=1kb)	633	TAPBPL	55080
512965	12q	50352073	50353762	1690	6	0.167	Promoter(1-2kb)	-1188	FAM186A	121006
512965	13q	24434450	24435347	898	7	0.571	Exon(exon31of34)	19787	PARP4	143
512965	13q	25096713	25097182	470	9	0.444	Promoter(<=1kb)	845	PABPC3	5042
512965	13q	102732474	102733933	1460	6	0.333	Exon(exon4of4)	25139	CCDC168	643677
512965	14q	20060048	20060884	837	8	0.625	Promoter(<=1kb)	3	OR4L1	122742
512965	14q	20640750	20641567	818	6	0.5	Promoter(<=1kb)	124	OR6S1	341799
512965	14q	21634137	21634589	453	9	0.556	Promoter(<=1kb)	351	OR10G2	26534
512965	14q	44504986	44506403	1418	7	0.714	Promoter(<=1kb)	880	FSCB	84075
512965	14q	70457532	70458540	1009	10	0.4	Exon(exon2of2)	5358	ADAM21	8747
512965	14q	104175275	104178677	3403	6	0.333	Exon(exon12of15)	36235	KIF26A	26153
512965	14q	104939262	104946886	7625	28	0.393	5'UTR	7102	PLD4	122618
512965	14q	104947901	104953878	5978	40	0.5	Promoter(1-2kb)	1524	AHNAK2	113146
512965	15q	20534480	20535014	535	7	0.429	Exon(exon8of9)	6786	GOLGA6L6	727832
512965	15q	40621642	40623696	2055	6	0.333	Promoter(<=1kb)	0	KNL1	57082
512965	15q	85579423	85581800	2378	14	0.571	Promoter(1-2kb)	-1110	AKAP13	11214
512965	15q	99129423	99132517	3095	6	0.333	Exon(exon4of5)	7225	TTC23	64927
512965	15q	101065640	101067308	1669	6	0.5	Promoter(1-2kb)	1224	LRRK1	79705
512965	16p	1256345	1256896	552	6	0.667	Promoter(<=1kb)	276	TPSD1	23430
512965	16p	1486371	1488463	2093	8	0.75	Promoter(<=1kb)	4	PTX4	390667
512965	16q	74391401	74391897	497	8	0.75	Exon(exon7of7)	13523	NPIPB15	440348
512965	16q	89100686	89101050	365	8	0.625	Promoter(<=1kb)	24	ACSF3	197322
512965	16q	89226863	89228419	1557	9	0.556	Promoter(2-3kb)	2229	ZNF778	197320
512965	17p	744946	746966	2021	6	1	3'UTR	5072	GEMIN4	50628
512965	17p	10638198	10641099	2902	7	0.286	Exon(exon19of41)	-8169	MYH3	4621
512965	17p	21300581	21300954	374	8	0.75	3'UTR	9112	MAP2K3	5606
512965	17p	21415470	21416404	935	6	1	Exon(exon3of3)	10334	KCNJ12	3768
512965	17q	43091983	43094240	2258	6	0.333	Promoter(<=1kb)	-132	BRCA1	672
512965	17q	76293419	76294016	598	6	0.5	Promoter(2-3kb)	-2167	QRICH2	84074
512965	17q	81645135	81645595	461	7	0.429	Promoter(2-3kb)	2722	TSPAN10	83882
512965	18p	11609646	11610164	519	13	0.846	Promoter(<=1kb)	50	SLC35G4	646000
512965	18q	58535186	58537515	2330	9	0.333	Promoter(<=1kb)	0	ALPK2	115701
512965	19p	1036457	1036914	458	7	0.571	Exon(exon6of7)	-3187	ABCA7	10347
512965	19p	4510711	4513547	2837	20	0.5	Exon(exon3of6)	4157	PLIN4	729359
512965	19p	5455600	5456439	840	7	0.571	Promoter(<=1kb)	183	ZNRF4	148066
512965	19p	8946313	8951868	5556	16	0.625	Exon(exon3of84)	29474	MUC16	94025
512965	19p	8959116	8962299	3184	10	0.7	Exon(exon3of84)	19043	MUC16	94025
512965	19p	8972751	8978096	5346	13	0.462	Exon(exon1of84)	3246	MUC16	94025
512965	19p	15087213	15088040	828	10	0.3	Promoter(<=1kb)	233	OR1I1	126370
512965	19p	17282085	17284246	2162	8	0.5	Promoter(<=1kb)	0	ANKLE1	126549
512965	19p	18264753	18267409	2657	14	0.643	5'UTR	7002	IQCN	80726
512965	19p	21971930	21974500	2571	8	0.625	Exon(exon4of4)	14408	ZNF208	7757
512965	19p	22756205	22759523	3319	14	0.571	3'UTR	10459	ZNF99	7652
512965	19p	23743906	23745300	1395	6	0.333	Exon(exon4of4)	13537	ZNF681	148213
512965	19q	36996730	36997597	868	10	0.6	Exon(exon10of10)	5677	ZNF568	374900
512965	19q	39893459	39894316	858	6	0.833	Exon(exon12of28)	-6167	FCGBP	8857
512965	19q	43913423	43914878	1456	8	0.5	Exon(exon10of10)	4861	ZNF45	7596
512965	19q	43966037	43967171	1135	6	0.167	Promoter(<=1kb)	-691	ZNF155	7711
512965	19q	44106512	44108078	1567	8	0.125	Exon(exon6of6)	-4103	ZNF225	7768
512965	19q	52437918	52439242	1325	7	0.429	Exon(exon4of4)	6504	ZNF534	147658
512965	19q	55517821	55518642	822	8	1	Exon(exon14of14)	17661	SBK2	646643
512965	19q	57640993	57641802	810	6	0.667	3'UTR	7768	ZNF211	10520
512965	20p	5922421	5923394	974	6	0.5	Exon(exon4of5)	6923	CHGB	1114
512965	20q	63349752	63350772	1021	6	0.5	3'UTR	3794	CHRNA4	1137
512965	20q	63561666	63565531	3866	11	0.636	Promoter(<=1kb)	-61	HELZ2	85441
512965	21q	26843740	26844859	1120	6	0.667	Promoter(<=1kb)	0	ADAMTS1	9510
512965	21q	44637474	44638041	568	11	0.364	Promoter(<=1kb)	118	KRTAP10-10	353333

512965	21q	44666460	44666841	382	7	0.714	Promoter(<=1kb)	86	KRTAP12-2	353323
512965	22q	22352950	22353380	431	16	0.5	Exon(exon1of2)	30478	BMS1P20	96610
512965	22q	36191154	36191906	753	6	0.667	3'UTR	9971	APOL4	80832
512965	23p	3320126	3323750	3625	9	0.556	Exon(exon5of7)	22902	MXRA5	25878
512965	23p	8170039	8170141	103	6	0.5	Promoter(1-2kb)	1126	VCX2	51480
512965	23p	35802148	35803010	863	7	0.571	5'UTR	3357	MAGEB16	139604
513003	1p	16058491	16060000	1510	10	0.9	Exon(exon5of7)	6168	CLCNKB	1188
513003	1p	18481403	18482217	815	7	0.714	Promoter(<=1kb)	421	KLHDC7A	127707
513003	1p	23874604	23875430	827	8	0.5	Exon(exon2of2)	-6310	FUCA1	2517
513003	1p	40067594	40067675	82	6	0	Promoter(<=1kb)	324	CAP1	10487
513003	1q	152213274	152213316	43	7	0.571	Exon(exon3of3)	10877	HRNR	388697
513003	1q	152218469	152221375	2907	17	0.706	Promoter(2-3kb)	2818	HRNR	388697
513003	1q	152303673	152313891	10219	39	0.59	Promoter(<=1kb)	0	FLG-AS1	339400
513003	1q	158765805	158766655	851	6	0.5	Promoter(<=1kb)	47	OR6N1	128372
513003	1q	169542317	169542882	566	6	0.167	Exon(exon13of25)	-26572	F5	2153
513003	1q	183647749	183648558	810	7	0.429	Exon(exon2of2)	4758	APOBEC4	403314
513003	1q	197101312	197101771	460	6	0.5	Exon(exon18of28)	33373	ASPM	259266
513003	1q	201206099	201209837	3739	12	0.583	Promoter(1-2kb)	1017	IGFN1	91156
513003	1q	201210956	201215147	4192	9	0.556	Promoter(<=1kb)	0	IGFN1	91156
513003	1q	223393517	223394466	950	6	0.667	Promoter(<=1kb)	102	CCDC185	164127
513003	1q	247841312	247841582	271	6	0.833	Promoter(<=1kb)	314	OR11L1	391189
513003	1q	247949325	247949738	414	10	0.3	Promoter(<=1kb)	467	OR2L8	391190
513003	1q	248294677	248295458	782	7	0.571	Promoter(<=1kb)	142	OR2T12	127064
513003	2p	29002636	29003646	1011	6	0.333	Exon(exon5of20)	-10821	TOGARAM2	165186
513003	2p	48580657	48582454	1798	7	0.571	Promoter(<=1kb)	0	STON1	11037
513003	2q	102351547	102351902	356	7	0.429	Exon(exon11of11)	-4027	IL18R1	8809
513003	2q	132781078	132784972	3895	10	0.5	Promoter(<=1kb)	0	NCKAP5	344148
513003	2q	178739433	178741811	2379	6	0.5	Exon(exon45of191)	26014	TTN	7273
513003	2q	184936178	184937636	1459	6	0.333	Exon(exon4of4)	69813	ZNF804A	91752
513003	2q	217847583	217848559	977	6	0.833	Exon(exon19of33)	-5423	TNS1	7145
513003	2q	238130416	238131546	1131	7	0.429	Promoter(1-2kb)	1468	ESPNL	339768
513003	3p	31989532	31990905	1374	6	0.333	Exon(exon2of2)	7761	ZNF860	344787
513003	3p	75736880	75739243	2364	55	0.618	Promoter(<=1kb)	0	MIR4273	100422955
513003	3q	98264413	98265098	686	7	0.571	Promoter(<=1kb)	128	OR5H6	79295
513003	4p	1394625	1395373	749	6	0.167	Exon(exon1of1)	9813	UVSSA	57654
513003	4p	6300792	6302360	1569	6	0.833	Exon(exon8of8)	6021	WFS1	7466
513003	4p	8227004	8228508	1505	8	0.125	Promoter(<=1kb)	-24	SH3TC1	54436
513003	4q	125316816	125320716	3901	7	0.714	Promoter(<=1kb)	417	FAT4	79633
513003	4q	185458217	185460011	1795	8	0.625	Promoter(<=1kb)	0	CCDC110	256309
513003	4q	186706638	186709751	3114	7	0.714	Exon(exon2of27)	14082	FAT1	2195
513003	5q	79728956	79730716	1761	7	0.286	Exon(exon2of13)	-7426	CMYA5	202333
513003	5q	79731782	79734523	2742	13	0.308	Exon(exon2of13)	-3619	CMYA5	202333
513003	5q	83537326	83539905	2580	6	0.333	Promoter(1-2kb)	1712	VCAN	1462
513003	5q	140848579	140850786	2208	7	0.571	Promoter(<=1kb)	807	PCDHA9	9752
513003	5q	141100771	141102758	1988	11	0.273	Promoter(<=1kb)	298	PCDHB3	56132
513003	5q	141174000	141175025	1026	6	0.833	Promoter(1-2kb)	1356	PCDHB7	56129
513003	5q	141183999	141184688	690	6	0.833	Promoter(2-3kb)	-2473	PCDHB9	56127
513003	5q	141187690	141189425	1736	6	0.5	Promoter(<=1kb)	529	PCDHB9	56127
513003	5q	141955676	141957660	1985	6	0.667	Promoter(<=1kb)	-668	RNF14	9604
513003	5q	151521550	151522069	520	6	0.667	Promoter(<=1kb)	79	MIR6499	102465246
513003	5q	151565922	151568158	2237	9	0.778	Promoter(<=1kb)	786	FAT2	2196
513003	6p	42104954	42107331	2378	6	0.5	Exon(exon4of5)	35288	C6orf132	647024
513003	6p	46858771	46859502	732	8	0.5	Exon(exon17of21)	3802	ADGRF5	221395
513003	6q	149888581	149889987	1407	6	0.667	Promoter(<=1kb)	-116	RAET1E-AS1	100652739
513003	6q	159231899	159234370	2472	12	0.583	Exon(exon11of23)	13602	FNDC1	84624
513003	7p	38353718	38353991	274	8	0.625	Exon(exon2of2)	3699	TRG-AS1	100506776
513003	7q	100958977	100960873	1897	50	0.46	Promoter(1-2kb)	1012	MUC3A	4584
513003	7q	100991195	100992398	1204	8	0.625	Exon(exon5of15)	-20656	MUC12	10071
513003	7q	100995547	100995785	239	7	0.857	Exon(exon5of15)	-17269	MUC12	10071
513003	7q	101003685	101004836	1152	7	0.714	Exon(exon5of15)	-8218	MUC12	10071
513003	7q	101034305	101038994	4690	32	0.406	Exon(exon3of12)	-4717	MUC17	140453
513003	7q	156949807	156950570	764	6	0.833	Promoter(<=1kb)	95	NOM1	64434
513003	8p	11331234	11332082	849	6	0.667	Promoter(<=1kb)	346	SLC35G5	83650
513003	8p	12132686	12133940	1255	6	0.667	Promoter(<=1kb)	498	USP17L7	392197
513003	8p	13021128	13022030	903	7	0.143	Exon(exon5of5)	9115	TRMT9B	57604
513003	8q	123651655	123652634	980	7	0.571	Promoter(<=1kb)	316	KLHL38	340359
513003	8q	141218050	141219792	1743	7	0.714	5'UTR	8778	SLC45A4	57210
513003	9p	116800	117713	914	9	0.667	Promoter(<=1kb)	491	FOXD4	2298
513003	9q	76175237	76175296	60	10	0.8	Exon(exon14of14)	-13343	PCSK5	5125
513003	9q	76705179	76707804	2626	8	0.5	Promoter(<=1kb)	121	PCA3	50652
513003	9q	76709523	76710843	1321	6	0.5	Promoter(<=1kb)	0	PRUNE2	158471
513003	9q	87885490	87888819	3330	8	0.625	Promoter(2-3kb)	2613	SPATA31E1	286234
513003	9q	104598545	104599361	817	12	0.583	Promoter(<=1kb)	52	OR13C5	138799
513003	9q	122553263	122554071	809	7	0.429	Promoter(<=1kb)	93	OR1N2	138882
513003	9q	122628595	122629130	536	6	0.333	Promoter(<=1kb)	443	OR1B1	347169
513003	9q	122749914	122750547	634	6	0.833	Promoter(<=1kb)	174	OR1L6	392390
513003	9q	131474844	131476113	1270	6	0.833	Promoter(<=1kb)	0	PRRC2B	84726

513003	9q	135547960	135548795	836	6	0.667	Promoter(1-2kb)	1805	OBP2A	29991
513003	10p	47663	48605	943	6	0.833	Promoter(<=1kb)	664	TUBB8	347688
513003	10q	46549378	46550723	1346	25	0.64	Exon(exon3of3)	4807	GPRIN2	9721
513003	10q	49323559	49326554	2996	10	0.5	Exon(exon3of3)	24285	C10orf71	118461
513003	10q	128103129	128106296	3168	13	0.615	Promoter(<=1kb)	-1	MKI67	4288
513003	11p	244106	244197	92	8	0.5	Promoter(<=1kb)	-232	PSMD13	5719
513003	11p	1241677	1243593	1917	7	0.429	Exon(exon31of49)	6083	MUC5B-AS1	112577518
513003	11p	1246095	1248605	2511	11	0.545	Promoter(1-2kb)	1071	MUC5B-AS1	112577518
513003	11p	1250091	1251628	1538	9	0.778	Promoter(<=1kb)	-415	MUC5B-AS1	112577518
513003	11p	5177978	5178478	501	6	0.167	Promoter(<=1kb)	186	OR52Z1	283110
513003	11p	5323362	5324256	895	7	0.429	Promoter(<=1kb)	41	OR51B2	79345
513003	11p	5389704	5390350	647	7	0.429	Promoter(<=1kb)	327	OR51M1	390059
513003	11p	5422212	5423123	912	11	0.636	Promoter(<=1kb)	101	OR51Q1	390061
513003	11p	5515185	5515931	747	6	0.333	Promoter(<=1kb)	768	UBQLNL	143630
513003	11p	5581045	5581738	694	8	0.375	Promoter(<=1kb)	168	OR52B6	340980
513003	11p	5841302	5841883	582	9	0.333	Promoter(<=1kb)	14	OR52E6	390078
513003	11p	11351961	11352736	776	9	0.222	Promoter(<=1kb)	514	CSNK2A3	283106
513003	11p	12293639	12294842	1204	7	0.714	Exon(exon29of35)	6739	MICALCL	84953
513003	11p	18173280	18173901	622	6	0.333	Promoter(<=1kb)	443	MRGPRX4	117196
513003	11p	34916266	34916763	498	6	0.667	Promoter(<=1kb)	0	APIP	51074
513003	11q	54603136	54603820	685	6	0.667	Promoter(<=1kb)	178	OR4C46	119749
513003	11q	58214757	58215722	966	8	0.25	Promoter(<=1kb)	12	OR1S1	219959
513003	11q	85724687	85725825	1139	6	0.5	Promoter(<=1kb)	0	SYTL2	54843
513003	11q	123906790	123907324	535	6	0.667	Promoter(<=1kb)	644	OR8D4	338662
513003	11q	123943088	123943782	695	7	0.571	Promoter(<=1kb)	91	OR6T1	219874
513003	11q	124015601	124016477	877	8	0.25	Promoter(<=1kb)	26	OR10G4	390264
513003	11q	124023038	124023849	812	9	0.444	Promoter(<=1kb)	25	OR10G9	219870
513003	11q	124038366	124038988	623	9	0.889	Promoter(<=1kb)	13	OR10G7	390265
513003	11q	130914501	130915580	1080	12	0.75	Promoter(<=1kb)	864	SNX19	399979
513003	12p	4626571	4628549	1979	8	0.5	Exon(exon5of6)	14054	DYRK4	8798
513003	12q	48525773	48526223	451	6	0.5	Promoter(<=1kb)	141	OR8S1	341568
513003	13q	25096659	25096984	326	6	0.5	Promoter(<=1kb)	791	PABPC3	5042
513003	13q	102732474	102733933	1460	6	0.333	Exon(exon4of4)	25139	CCDC168	643677
513003	14q	19975713	19976448	736	10	0.4	Promoter(<=1kb)	269	OR4K15	81127
513003	14q	20060048	20060884	837	8	0.625	Promoter(<=1kb)	3	OR4L1	122742
513003	14q	20143999	20144604	606	8	0.5	Promoter(<=1kb)	263	OR4N5	390437
513003	14q	20640750	20641567	818	7	0.571	Promoter(<=1kb)	124	OR6S1	341799
513003	14q	21634137	21634589	453	9	0.556	Promoter(<=1kb)	351	OR10G2	26534
513003	14q	44504986	44506403	1418	7	0.714	Promoter(<=1kb)	880	FSCB	84075
513003	14q	70457520	70459292	1773	13	0.385	Exon(exon2of2)	5346	ADAM21	8747
513003	14q	104175275	104177810	2536	9	0.333	Exon(exon12of15)	36235	KIF26A	26153
513003	14q	104939262	104942618	3357	12	0.167	5'UTR	7102	PLD4	122618
513003	14q	104943622	104946886	3265	15	0.6	Exon(exon6of6)	8516	AHNAK2	113146
513003	14q	104947901	104953878	5978	33	0.515	Promoter(1-2kb)	1524	AHNAK2	113146
513003	15q	23440160	23442067	1908	12	0.583	5'UTR	5167	GOLGA6L2	283685
513003	15q	40621642	40623696	2055	6	0.333	Promoter(<=1kb)	0	KNL1	57082
513003	15q	73702465	73703760	1296	6	0.833	Promoter(<=1kb)	-149	CD276	80381
513003	15q	85579423	85582073	2651	15	0.6	Promoter(<=1kb)	-837	AKAP13	11214
513003	15q	88854874	88855594	721	7	0.857	Exon(exon12of18)	7631	ACAN	176
513003	15q	88857108	88859365	2258	6	0	Exon(exon12of18)	9865	ACAN	176
513003	15q	99129423	99132517	3095	7	0.429	Exon(exon4of5)	7225	TTC23	64927
513003	15q	100569472	100570097	626	6	0.833	Promoter(<=1kb)	534	LINS1	55180
513003	16p	1228744	1229622	879	10	0.8	Promoter(<=1kb)	540	TPSB2	64499
513003	16p	1486371	1488463	2093	9	0.778	Promoter(<=1kb)	4	PTX4	390667
513003	16q	74391650	74391928	279	8	0.75	Exon(exon7of7)	13772	NPIPB15	440348
513003	16q	88433131	88436097	2967	6	0.667	Exon(exon3of3)	-17183	ZFPM1	161882
513003	16q	88714632	88717113	2482	7	0.429	Promoter(<=1kb)	0	MIR4722	100616167
513003	16q	89100686	89101050	365	8	0.625	Promoter(<=1kb)	24	ACSF3	197322
513003	16q	89226863	89228289	1427	8	0.625	Promoter(2-3kb)	2229	ZNF778	197320
513003	17p	744946	746966	2021	6	1	3'UTR	5072	GEMIN4	50628
513003	17p	10638198	10641099	2902	7	0.286	Exon(exon19of41)	-8169	MYH3	4621
513003	17p	21300581	21300978	398	12	0.75	3'UTR	9112	MAP2K3	5606
513003	17q	53823368	53824891	1524	6	0.833	Promoter(<=1kb)	441	KIF2B	84643
513003	17q	81645135	81645607	473	7	0.286	Promoter(2-3kb)	2722	TSPAN10	83882
513003	18p	11609728	11610509	782	13	0.692	Promoter(<=1kb)	132	SLC35G4	646000
513003	18q	47033473	47035259	1787	9	1	Promoter(<=1kb)	362	ELOA2	51224
513003	18q	58535186	58537515	2330	9	0.333	Promoter(<=1kb)	0	ALPK2	115701
513003	19p	1036457	1036914	458	6	0.5	Exon(exon6of7)	-3187	ABCA7	10347
513003	19p	4510548	4513547	3000	22	0.455	Exon(exon3of6)	4157	PLIN4	729359
513003	19p	5455600	5456439	840	8	0.625	Promoter(<=1kb)	183	ZNRF4	148066
513003	19p	8937644	8939234	1591	6	0.833	Exon(exon5of84)	-41554	MUC16	94025
513003	19p	8946306	8951868	5563	18	0.667	Exon(exon3of84)	29474	MUC16	94025
513003	19p	8959116	8962299	3184	12	0.583	Exon(exon3of84)	19043	MUC16	94025
513003	19p	8963397	8967127	3731	21	0.667	Exon(exon3of84)	14215	MUC16	94025
513003	19p	8972751	8978096	5346	13	0.462	Exon(exon1of84)	3246	MUC16	94025
513003	19p	12430400	12431840	1441	8	0.5	3'UTR	9181	ZNF443	10224
513003	19p	17281820	17284246	2427	9	0.556	Promoter(<=1kb)	0	ANKLE1	126549

513003	19p	18264798	18267409	2612	12	0.583	5'UTR	7002	IQCN	80726
513003	19p	21971930	21974500	2571	10	0.7	Exon(exon4of4)	14408	ZNF208	7757
513003	19q	36996730	36997597	868	10	0.7	Exon(exon10of10)	5677	ZNF568	374900
513003	19q	37151928	37153149	1222	6	0.833	Exon(exon5of5)	19287	ZNF585A	199704
513003	19q	39877222	39877880	659	6	0.5	Exon(exon20of28)	9412	FCGBP	8857
513003	19q	39886240	39886422	183	6	0.667	Promoter(<=1kb)	870	FCGBP	8857
513003	19q	43913423	43914878	1456	9	0.556	Exon(exon10of10)	4861	ZNF45	7596
513003	19q	43996326	43997366	1041	6	0.5	Exon(exon5of5)	5419	LOC10192806:	101928063
513003	19q	44106512	44108078	1567	7	0	Exon(exon6of6)	-4103	ZNF225	7768
513003	19q	48873325	48875925	2601	12	0.583	Promoter(<=1kb)	904	PPP1R15A	23645
513003	19q	52437918	52439242	1325	7	0.429	Exon(exon4of4)	6504	ZNF534	147658
513003	19q	55911888	55913077	1190	6	0.667	Exon(exon5of12)	19234	NLRP13	126204
513003	20p	5922421	5923643	1223	9	0.667	Exon(exon4of5)	6923	CHGB	1114
513003	20p	20052354	20052736	383	6	0	Promoter(<=1kb)	0	CFAP61	26074
513003	20q	63561666	63565531	3866	12	0.667	Promoter(<=1kb)	-61	HELZ2	85441
513003	21q	41740862	41741990	1129	6	0.833	Exon(exon2of2)	4851	MIR6814	102465488
513003	21q	44637474	44638143	670	11	0.455	Promoter(<=1kb)	118	KRTAP10-10	353333
513003	22q	22352950	22353380	431	16	0.5	Exon(exon1of2)	30478	BMS1P20	96610
513003	22q	36191154	36191906	753	6	0.667	3'UTR	9971	APOL4	80832
513003	23p	35802148	35803010	863	7	0.571	5'UTR	3357	MAGEB16	139604
513003	23q	136874183	136874416	234	8	0.75	Promoter(<=1kb)	-201	RBMX	27316
513866	1p	16058491	16060000	1510	13	0.846	Exon(exon5of7)	6168	CLCNKB	1188
513866	1p	18481403	18482217	815	7	0.714	Promoter(<=1kb)	421	KLHDC7A	127707
513866	1p	23874604	23875430	827	8	0.5	Exon(exon2of2)	-6310	FUCA1	2517
513866	1p	40067594	40067675	82	6	0	Promoter(<=1kb)	324	CAP1	10487
513866	1p	89186388	89186419	32	9	0.556	Promoter(<=1kb)	107	GBP4	115361
513866	1q	152303673	152313891	10219	39	0.59	Promoter(<=1kb)	0	FLG-AS1	339400
513866	1q	156669844	156670886	1043	7	1	Exon(exon4of4)	6521	NES	10763
513866	1q	201206099	201209342	3244	10	0.6	Promoter(1-2kb)	1017	IGFN1	91156
513866	1q	214640144	214642954	2811	12	0.5	Exon(exon12of20)	-5013	CENPF	1063
513866	1q	214644872	214647181	2310	10	0.4	Promoter(<=1kb)	-786	CENPF	1063
513866	1q	223393517	223394466	950	6	0.667	Promoter(<=1kb)	102	CCDC185	164127
513866	1q	226735683	226737239	1557	7	0.857	Promoter(<=1kb)	219	ITPKB	3707
513866	1q	247841312	247841582	271	6	0.833	Promoter(<=1kb)	314	OR11L1	391189
513866	1q	247921132	247921847	716	7	0.714	Promoter(<=1kb)	114	OR2T8	343172
513866	1q	247949325	247949738	414	11	0.273	Promoter(<=1kb)	467	OR2L8	391190
513866	1q	248294677	248295458	782	7	0.571	Promoter(<=1kb)	142	OR2T12	127064
513866	1q	248681796	248682226	431	6	0.667	Promoter(<=1kb)	102	OR14I1	401994
513866	2q	130914528	130916712	2185	6	0.333	Promoter(<=1kb)	0	ARHGEF4	50649
513866	2q	132781993	132785012	3020	10	0.6	Promoter(<=1kb)	0	NCKAP5	344148
513866	2q	184936178	184937636	1459	6	0.333	Exon(exon4of4)	69813	ZNF804A	91752
513866	2q	185790999	185794632	3634	8	0.875	Promoter(<=1kb)	0	FSIP2	401024
513866	2q	185805377	185808170	2794	6	0.333	Promoter(<=1kb)	0	FSIP2	401024
513866	2q	233713134	233713783	650	13	0.692	Promoter(<=1kb)	142	UGT1A5	54579
513866	2q	238130416	238131546	1131	6	0.333	Promoter(1-2kb)	1468	ESPNL	339768
513866	3p	31989532	31990905	1374	8	0.5	Exon(exon2of2)	7761	ZNF860	344787
513866	3p	75736880	75739243	2364	52	0.596	Promoter(<=1kb)	0	MIR4273	100422955
513866	3q	98264413	98265098	686	7	0.571	Promoter(<=1kb)	128	OR5H6	79295
513866	4p	1394795	1395368	574	6	0.5	Exon(exon1of1)	9983	UVSSA	57654
513866	4p	5988383	5989749	1367	7	0.571	Promoter(<=1kb)	0	C4orf50	389197
513866	4p	6300792	6302391	1600	8	0.875	Exon(exon8of8)	6021	WFS1	7466
513866	4p	8227004	8228508	1505	9	0.222	Promoter(<=1kb)	-24	SH3TC1	54436
513866	4p	10443803	10446224	2422	7	0.429	Exon(exon3of3)	10952	ZNF518B	85460
513866	4q	121036404	121037542	1139	6	0.333	Promoter(1-2kb)	1442	NDNF	79625
513866	4q	186619481	186621582	2102	7	0.429	Exon(exon10of27)	-9411	FAT1	2195
513866	4q	186706638	186709436	2799	7	0.714	Exon(exon2of27)	14397	FAT1	2195
513866	5q	79728956	79730716	1761	6	0.333	Exon(exon2of13)	-7426	CMYA5	202333
513866	5q	79731782	79734523	2742	13	0.308	Exon(exon2of13)	-3619	CMYA5	202333
513866	5q	140848579	140850786	2208	6	0.5	Promoter(<=1kb)	807	PCDHA9	9752
513866	5q	141955356	141957660	2305	6	0.667	Promoter(<=1kb)	-668	RNF14	9604
513866	5q	151565922	151568158	2237	10	0.7	Promoter(<=1kb)	786	FAT2	2196
513866	6p	46858771	46859502	732	8	0.5	Exon(exon17of21)	3802	ADGRF5	221395
513866	6q	106511549	106512572	1024	6	0.667	Promoter(<=1kb)	0	CRYBG1	202
513866	6q	159233455	159234370	916	10	0.5	Exon(exon11of23)	15158	FNDC1	84624
513866	7p	48271966	48274682	2717	6	0.667	3'UTR	-4594	ABCA13	154664
513866	7q	100958721	100960873	2153	54	0.463	Promoter(<=1kb)	756	MUC3A	4584
513866	7q	100991195	100993102	1908	8	0.625	Exon(exon5of15)	-19952	MUC12	10071
513866	7q	100995575	100995785	211	7	0.714	Exon(exon5of15)	-17269	MUC12	10071
513866	7q	101003685	101004836	1152	6	0.667	Exon(exon5of15)	-8218	MUC12	10071
513866	7q	101034361	101040583	6223	38	0.5	Exon(exon3of12)	-3128	MUC17	140453
513866	7q	149818015	149819792	1778	6	0.667	Promoter(2-3kb)	-2352	SSPO	23145
513866	8p	10607245	10608432	1188	8	0.5	Exon(exon4of4)	46711	RP1L1	94137
513866	8p	10609614	10610142	529	7	0.429	Exon(exon4of4)	45001	RP1L1	94137
513866	8p	13021128	13022030	903	7	0.143	Exon(exon5of5)	9115	TRMT9B	57604
513866	8q	123651873	123652634	762	6	0.333	Promoter(<=1kb)	316	KLHL38	340359
513866	8q	143870008	143872631	2624	6	0.5	Exon(exon2of2)	5833	EPPK1	83481
513866	8q	143916360	143919209	2850	7	0.143	Exon(exon32of32)	20381	PLEC	5339

513866	9p	116800	117713	914	6	0.667	Promoter(<=1kb)	491	FOXD4	2298
513866	9p	21206764	21207074	311	6	0.5	Promoter(<=1kb)	69	IFNA10	3446
513866	9q	76175237	76175296	60	10	0.8	Exon(exon14of14)	-13343	PCSK5	5125
513866	9q	76703555	76707804	4250	13	0.462	Promoter(<=1kb)	0	PCA3	50652
513866	9q	76709263	76710843	1581	8	0.5	Promoter(<=1kb)	0	PRUNE2	158471
513866	9q	104598545	104599361	817	13	0.538	Promoter(<=1kb)	52	OR13C5	138799
513866	9q	122553263	122554071	809	7	0.429	Promoter(<=1kb)	93	OR1N2	138882
513866	9q	122628595	122629398	804	8	0.375	Promoter(<=1kb)	175	OR1B1	347169
513866	9q	122749914	122750547	634	6	0.833	Promoter(<=1kb)	174	OR1L6	392390
513866	9q	131474844	131476113	1270	6	0.833	Promoter(<=1kb)	0	PRRC2B	84726
513866	9q	135484803	135487213	2411	10	0.4	Promoter(1-2kb)	1440	PPP1R26	9858
513866	9q	135547960	135548795	836	6	0.667	Promoter(1-2kb)	1805	OBP2A	29991
513866	10q	46549378	46550744	1367	26	0.615	5'UTR	4786	GPRIN2	9721
513866	10q	122084988	122087840	2853	8	0.75	Exon(exon4of23)	-25190	TACC2	10579
513866	10q	128102594	128105201	2608	6	0.667	Promoter(<=1kb)	0	MKI67	4288
513866	11p	244106	244197	92	8	0.5	Promoter(<=1kb)	-232	PSMD13	5719
513866	11p	1246095	1247378	1284	7	0.286	Promoter(2-3kb)	2298	MUC5B-AS1	112577518
513866	11p	5177978	5178478	501	6	0.167	Promoter(<=1kb)	186	OR52Z1	283110
513866	11p	5323451	5324256	806	6	0.5	Promoter(<=1kb)	41	OR51B2	79345
513866	11p	5389704	5390350	647	6	0.5	Promoter(<=1kb)	327	OR51M1	390059
513866	11p	5515185	5515931	747	6	0.333	Promoter(<=1kb)	768	UBQLNL	143630
513866	11p	5581045	5581738	694	8	0.375	Promoter(<=1kb)	168	OR52B6	340980
513866	11p	5884818	5885061	244	6	0.333	Promoter(<=1kb)	547	OR52E4	390081
513866	11p	11351961	11352736	776	9	0.222	Promoter(<=1kb)	514	CSNK2A3	283106
513866	11p	12293639	12294842	1204	7	0.714	Exon(exon29of35)	6739	MICALCL	84953
513866	11q	54603136	54603820	685	6	0.667	Promoter(<=1kb)	178	OR4C46	119749
513866	11q	58214757	58215722	966	8	0.25	Promoter(<=1kb)	12	OR1S1	219959
513866	11q	82732630	82733184	555	6	0.833	Promoter(<=1kb)	680	FAM181B	220382
513866	11q	85724687	85725825	1139	6	0.5	Promoter(<=1kb)	0	SYTL2	54843
513866	11q	123906790	123907324	535	6	0.667	Promoter(<=1kb)	644	OR8D4	338662
513866	11q	124038366	124038988	623	7	1	Promoter(<=1kb)	13	OR10G7	390265
513866	12p	4626568	4628549	1982	11	0.455	Exon(exon5of6)	14051	DYRK4	8798
513866	12q	48525773	48526223	451	6	0.333	Promoter(<=1kb)	141	OR8S1	341568
513866	12q	52316096	52317765	1670	6	0.667	Exon(exon4of9)	3633	KRT83	3889
513866	13q	25096870	25097682	813	11	0.545	Promoter(1-2kb)	1002	PABPC3	5042
513866	13q	102732474	102733933	1460	6	0.333	Exon(exon4of4)	25139	CCDC168	643677
513866	14q	20640982	20641567	586	6	0.5	Promoter(<=1kb)	124	OR6S1	341799
513866	14q	21634137	21634589	453	9	0.556	Promoter(<=1kb)	351	OR10G2	26534
513866	14q	44505064	44506403	1340	6	0.667	Promoter(<=1kb)	880	FSCB	84075
513866	14q	104949015	104951557	2543	7	0.286	Exon(exon6of6)	3845	AHNAK2	113146
513866	15q	20534501	20535129	629	6	0.333	Exon(exon8of9)	6671	GOLGA6L6	727832
513866	15q	23440160	23442067	1908	11	0.545	5'UTR	5167	GOLGA6L2	283685
513866	15q	99129423	99132517	3095	6	0.333	Exon(exon4of5)	7225	TTC23	64927
513866	16p	1228744	1229622	879	6	0.667	Promoter(<=1kb)	540	TPSB2	64499
513866	16p	1486371	1488463	2093	8	0.75	Promoter(<=1kb)	4	PTX4	390667
513866	16p	3589138	3590852	1715	6	0.833	Exon(exon12of15)	-11735	NLRC3	197358
513866	16p	28495551	28497395	1845	6	0.333	Promoter(<=1kb)	0	CLN3	1201
513866	16q	74391401	74391897	497	9	0.778	Exon(exon7of7)	13523	NPIPB15	440348
513866	16q	89100686	89101050	365	8	0.625	Promoter(<=1kb)	24	ACSF3	197322
513866	16q	89227116	89228419	1304	9	0.667	Promoter(2-3kb)	2482	ZNF778	197320
513866	17p	744946	747069	2124	7	1	3'UTR	4969	GEMIN4	50628
513866	17p	21300581	21300978	398	12	0.75	3'UTR	9112	MAP2K3	5606
513866	17p	21415470	21416404	935	7	0.857	Exon(exon3of3)	10334	KCNJ12	3768
513866	17q	41586466	41586829	364	6	0.833	Promoter(<=1kb)	66	KRT14	3861
513866	17q	41727098	41728331	1234	6	0.833	Promoter(1-2kb)	-1201	HAP1	9001
513866	17q	76293419	76294016	598	6	0.5	Promoter(2-3kb)	-2167	QRICH2	84074
513866	17q	81645135	81645595	461	7	0.429	Promoter(2-3kb)	2722	TSPAN10	83882
513866	18p	11609646	11610164	519	9	0.778	Promoter(<=1kb)	50	SLC35G4	646000
513866	18q	58534888	58537515	2628	12	0.417	Promoter(<=1kb)	0	ALPK2	115701
513866	19p	4510548	4513547	3000	22	0.455	Exon(exon3of6)	4157	PLIN4	729359
513866	19p	5455600	5456439	840	8	0.625	Promoter(<=1kb)	183	ZNRF4	148066
513866	19p	8946313	8951868	5556	15	0.6	Exon(exon3of84)	29474	MUC16	94025
513866	19p	8959116	8962299	3184	10	0.7	Exon(exon3of84)	19043	MUC16	94025
513866	19p	8972751	8978096	5346	16	0.5	Exon(exon1of84)	3246	MUC16	94025
513866	19p	12430400	12431840	1441	8	0.5	3'UTR	9181	ZNF443	10224
513866	19p	15794192	15794719	528	6	0.333	Promoter(<=1kb)	241	OR10H5	284433
513866	19p	18264798	18267409	2612	12	0.583	5'UTR	7002	IQCN	80726
513866	19p	21971930	21974500	2571	8	0.75	Exon(exon4of4)	14408	ZNF208	7757
513866	19p	22663799	22665021	1223	6	0.667	Exon(exon4of4)	29475	ZNF492	57615
513866	19p	22756205	22759523	3319	13	0.615	3'UTR	10459	ZNF99	7652
513866	19q	36996730	36997597	868	10	0.6	Exon(exon10of10)	5677	ZNF568	374900
513866	19q	39886240	39886422	183	6	0.667	Promoter(<=1kb)	870	FCGBP	8857
513866	19q	43846955	43848536	1582	6	0.833	3'UTR	13450	ZNF283	284349
513866	19q	43913423	43914878	1456	8	0.5	Exon(exon10of10)	4861	ZNF45	7596
513866	19q	43966037	43967171	1135	6	0.167	Promoter(<=1kb)	-691	ZNF155	7711
513866	19q	44106512	44108078	1567	7	0	Exon(exon6of6)	-4103	ZNF225	7768
513866	19q	52384029	52384992	964	6	0	Exon(exon4of4)	12677	ZNF528-AS1	102724105

513866	19q	52437918	52439242	1325	7	0.429	Exon(exon4of4)	6504	ZNF534	147658
513866	19q	53490416	53491750	1335	6	0.5	Exon(exon4of4)	22673	ZNF813	126017
513866	19q	55911888	55913077	1190	6	0.667	Exon(exon5of12)	19234	NLRP13	126204
513866	20p	5922421	5923394	974	9	0.556	Exon(exon4of5)	6923	CHGB	1114
513866	20p	20052354	20052736	383	6	0.167	Promoter(<=1kb)	0	CFAP61	26074
513866	20q	63349752	63350772	1021	6	0.5	3'UTR	3794	CHRNA4	1137
513866	20q	63561666	63565531	3866	12	0.667	Promoter(<=1kb)	-61	HELZ2	85441
513866	22q	22352950	22353380	431	16	0.5	Exon(exon1of2)	30478	BMS1P20	96610
513866	22q	22758758	22759209	452	6	0.833	Exon(exon1of2)	-63567	MIR650	723778
513866	22q	36191154	36191906	753	6	0.667	3'UTR	9971	APOL4	80832
513866	22q	39100331	39102033	1703	6	0.833	Promoter(<=1kb)	52	APOBEC3H	164668
513866	22q	50503556	50505734	2179	7	0.857	Promoter(<=1kb)	0	LMF2	91289
513866	23p	3320126	3323750	3625	9	0.556	Exon(exon5of7)	22902	MXRA5	25878
513866	23p	8170039	8170141	103	6	0.5	Promoter(1-2kb)	1126	VCX2	51480
513866	23p	35802148	35803010	863	7	0.571	5'UTR	3357	MAGEB16	139604
513886	1p	16058491	16060000	1510	10	0.9	Exon(exon5of7)	6168	CLCNKB	1188
513886	1p	18481403	18482217	815	6	0.667	Promoter(<=1kb)	421	KLHDC7A	127707
513886	1p	23874604	23875430	827	8	0.5	Exon(exon2of2)	-6310	FUCA1	2517
513886	1p	40067594	40067675	82	6	0	Promoter(<=1kb)	324	CAP1	10487
513886	1q	152213274	152213320	47	8	0.5	Exon(exon3of3)	10873	HRNR	388697
513886	1q	152218469	152221375	2907	17	0.706	Promoter(2-3kb)	2818	HRNR	388697
513886	1q	152303673	152313891	10219	41	0.585	Promoter(<=1kb)	0	FLG-AS1	339400
513886	1q	158765805	158766655	851	6	0.5	Promoter(<=1kb)	47	OR6N1	128372
513886	1q	201206099	201209342	3244	10	0.6	Promoter(1-2kb)	1017	IGFN1	91156
513886	1q	201210956	201215147	4192	9	0.556	Promoter(<=1kb)	0	IGFN1	91156
513886	1q	247841312	247841582	271	6	0.833	Promoter(<=1kb)	314	OR11L1	391189
513886	1q	247949325	247949738	414	10	0.3	Promoter(<=1kb)	467	OR2L8	391190
513886	1q	248294677	248295458	782	7	0.571	Promoter(<=1kb)	142	OR2T12	127064
513886	2q	185789865	185794632	4768	11	0.727	Promoter(<=1kb)	0	FSIP2	401024
513886	2q	185805377	185808170	2794	6	0.333	Promoter(<=1kb)	0	FSIP2	401024
513886	2q	217847583	217848559	977	6	0.833	Exon(exon19of33)	-5423	TNS1	7145
513886	2q	219539659	219540599	941	6	0.667	Promoter(2-3kb)	2498	CHPF	79586
513886	2q	233713134	233713664	531	8	0.75	Promoter(<=1kb)	142	UGT1A5	54579
513886	2q	238130236	238131546	1311	8	0.375	Promoter(1-2kb)	1288	ESPNL	339768
513886	3p	75736880	75738859	1980	52	0.577	Promoter(<=1kb)	0	MIR4273	100422955
513886	3q	98264413	98265098	686	7	0.571	Promoter(<=1kb)	128	OR5H6	79295
513886	4p	3492873	3493455	583	6	0.667	5'UTR	7524	DOK7	285489
513886	4p	5988383	5989749	1367	7	0.571	Promoter(<=1kb)	0	C4orf50	389197
513886	4p	6300792	6302360	1569	7	0.857	Exon(exon8of8)	6021	WFS1	7466
513886	4p	8227004	8228508	1505	8	0.125	Promoter(<=1kb)	-24	SH3TC1	54436
513886	4q	186619481	186621582	2102	7	0.286	Exon(exon10of27)	-9411	FAT1	2195
513886	5q	54518000	54519730	1731	6	0.167	Promoter(<=1kb)	135	SNX18	112574
513886	5q	79731782	79734523	2742	14	0.357	Exon(exon2of13)	-3619	CMYA5	202333
513886	5q	140848579	140850786	2208	9	0.556	Promoter(<=1kb)	807	PCDHA9	9752
513886	5q	141174000	141175025	1026	6	0.833	Promoter(1-2kb)	1356	PCDHB7	56129
513886	5q	151565922	151568158	2237	9	0.778	Promoter(<=1kb)	786	FAT2	2196
513886	6p	46858771	46859389	619	6	0.5	Exon(exon17of21)	3915	ADGRF5	221395
513886	6p	56605300	56607197	1898	7	0.571	Exon(exon36of98)	-6787	DST	667
513886	6q	149888581	149890867	2287	8	0.625	Promoter(<=1kb)	0	RAET1E-AS1	100652739
513886	6q	159233455	159234370	916	10	0.5	Exon(exon11of23)	15158	FNDC1	84624
513886	7p	6330446	6330944	499	6	1	Exon(exon2of2)	7749	FAM220A	84792
513886	7p	12369637	12370736	1100	6	0.667	3'UTR	-13307	VWDE	221806
513886	7p	38353718	38353991	274	8	0.625	Exon(exon2of2)	3699	TRG-AS1	100506776
513886	7q	100958977	100960873	1897	55	0.473	Promoter(1-2kb)	1012	MUC3A	4584
513886	7q	100991195	100993127	1933	9	0.667	Exon(exon5of15)	-19927	MUC12	10071
513886	7q	100995575	100995879	305	7	0.857	Exon(exon5of15)	-17175	MUC12	10071
513886	8p	8376561	8377198	638	6	1	Exon(exon2of5)	4549	PRAG1	157285
513886	8p	10607245	10608261	1017	7	0.571	Exon(exon4of4)	46882	RP1L1	94137
513886	8p	10609650	10612307	2658	9	0.778	Exon(exon4of4)	42836	RP1L1	94137
513886	8p	12132686	12133940	1255	6	0.667	Promoter(<=1kb)	498	USP17L7	392197
513886	8p	13021128	13022030	903	8	0.125	Exon(exon5of5)	9115	TRMT9B	57604
513886	8q	138151949	138153046	1098	6	0.5	Promoter(<=1kb)	0	FAM135B	51059
513886	8q	141466455	141467514	1060	8	0.75	3'UTR	29245	MROH5	389690
513886	9p	214661	215269	609	6	0.5	Promoter(<=1kb)	0	DOCK8	81704
513886	9p	34723747	34726527	2781	11	0.727	Promoter(2-3kb)	2961	FAM205A	259308
513886	9q	76175237	76175301	65	12	0.75	Exon(exon14of14)	-13338	PCSK5	5125
513886	9q	76705724	76707804	2081	6	0.5	Promoter(<=1kb)	666	PCA3	50652
513886	9q	104598641	104599361	721	8	0.625	Promoter(<=1kb)	52	OR13C5	138799
513886	9q	122628595	122629398	804	6	0.333	Promoter(<=1kb)	175	OR1B1	347169
513886	9q	122749914	122750547	634	6	0.833	Promoter(<=1kb)	174	OR1L6	392390
513886	10q	46549378	46550723	1346	25	0.64	Exon(exon3of3)	4807	GPRIN2	9721
513886	10q	49323559	49326331	2773	9	0.444	Exon(exon3of3)	24285	C10orf71	118461
513886	11p	244106	244197	92	8	0.5	Promoter(<=1kb)	-232	PSMD13	5719
513886	11p	1194354	1196902	2549	7	0.571	Exon(exon34of49)	-26164	MUC5B	727897
513886	11p	1246332	1247378	1047	6	0.5	Promoter(2-3kb)	2298	MUC5B-AS1	112577518
513886	11p	1835943	1837342	1400	6	0.667	Promoter(<=1kb)	0	SYT8	90019
513886	11p	5177811	5178478	668	7	0.143	Promoter(<=1kb)	186	OR52Z1	283110

513886	11p	5422212	5423123	912	11	0.636	Promoter(<=1kb)	101	OR51Q1	390061
513886	11p	5544676	5545259	584	6	0.5	Promoter(<=1kb)	290	OR52H1	390067
513886	11p	5581045	5581738	694	8	0.375	Promoter(<=1kb)	168	OR52B6	340980
513886	11p	5884818	5885061	244	6	0.333	Promoter(<=1kb)	547	OR52E4	390081
513886	11p	11351961	11352736	776	9	0.222	Promoter(<=1kb)	514	CSNK2A3	283106
513886	11p	12293639	12294842	1204	7	0.714	Exon(exon29of35)	6739	MICALCL	84953
513886	11p	18173280	18173901	622	6	0.333	Promoter(<=1kb)	443	MRGPRX4	117196
513886	11p	43942293	43943348	1056	8	0.75	Promoter(<=1kb)	0	C11orf96	387763
513886	11q	58214757	58215722	966	7	0.286	Promoter(<=1kb)	12	OR1S1	219959
513886	11q	58402523	58403265	743	8	0.5	Promoter(<=1kb)	144	OR5B3	441608
513886	11q	64315818	64315856	39	6	0.833	Promoter(1-2kb)	1485	TRMT112	51504
513886	11q	66560202	66562261	2060	6	0.333	Exon(exon14of21)	3873	CTSF	8722
513886	11q	93697503	93700096	2594	11	0.636	Promoter(<=1kb)	0	CEP295	85459
513886	11q	130914501	130915409	909	10	0.7	Promoter(1-2kb)	1035	SNX19	399979
513886	12p	4626571	4628549	1979	8	0.5	Exon(exon5of6)	14054	DYRK4	8798
513886	12p	8222174	8223514	1341	6	0.667	Exon(exon5of6)	4073	FAM90A1	55138
513886	12p	31981130	31983004	1875	8	0.25	Exon(exon4of6)	-4162	RESF1	55196
513886	13q	102732474	102733933	1460	6	0.333	Exon(exon4of4)	25139	CCDC168	643677
513886	14q	20060048	20060884	837	8	0.625	Promoter(<=1kb)	3	OR4L1	122742
513886	14q	20640982	20641567	586	6	0.5	Promoter(<=1kb)	124	OR6S1	341799
513886	14q	21634137	21634589	453	9	0.556	Promoter(<=1kb)	351	OR10G2	26534
513886	14q	22633879	22634450	572	9	0.333	Exon(exon2of2)	32212	ABHD4	63874
513886	14q	70457532	70458540	1009	9	0.333	Exon(exon2of2)	5358	ADAM21	8747
513886	14q	94587512	94587839	328	6	0.5	Exon(exon2of2)	-4219	SERPINA3	12
513886	14q	104175455	104177762	2308	8	0.375	Exon(exon12of15)	36415	KIF26A	26153
513886	14q	104939262	104942618	3357	10	0.2	5'UTR	7102	PLD4	122618
513886	14q	104943622	104945444	1823	7	0.571	Exon(exon6of6)	9958	AHNAK2	113146
513886	14q	104946867	104953878	7012	42	0.548	Promoter(1-2kb)	1524	AHNAK2	113146
513886	15q	23439979	23442067	2089	12	0.583	5'UTR	5167	GOLGA6L2	283685
513886	15q	40356171	40356211	41	6	0.667	Promoter(2-3kb)	-2008	DISP2	85455
513886	15q	59206980	59208056	1077	7	0.714	Promoter(<=1kb)	137	LDHAL6B	92483
513886	15q	73702465	73703760	1296	6	0.833	Promoter(<=1kb)	-149	CD276	80381
513886	15q	85579423	85582073	2651	15	0.6	Promoter(<=1kb)	-837	AKAP13	11214
513886	15q	88857340	88859365	2026	6	0.333	Exon(exon12of18)	10097	ACAN	176
513886	16p	768559	770215	1657	8	0.5	Promoter(<=1kb)	0	MIR662	724032
513886	16p	1486371	1488463	2093	8	0.75	Promoter(<=1kb)	4	PTX4	390667
513886	16q	88428539	88436097	7559	21	0.571	Exon(exon3of3)	-17183	ZFPM1	161882
513886	16q	88714632	88717113	2482	7	0.429	Promoter(<=1kb)	0	MIR4722	100616167
513886	16q	89100686	89101050	365	7	0.571	Promoter(<=1kb)	24	ACSF3	197322
513886	16q	89226863	89228289	1427	7	0.571	Promoter(2-3kb)	2229	ZNF778	197320
513886	17p	10638198	10641099	2902	7	0.286	Exon(exon19of41)	-8169	MYH3	4621
513886	17p	21300581	21300954	374	8	0.75	3'UTR	9112	MAP2K3	5606
513886	17q	81645135	81645607	473	7	0.286	Promoter(2-3kb)	2722	TSPAN10	83882
513886	18p	11609646	11610383	738	13	0.846	Promoter(<=1kb)	50	SLC35G4	646000
513886	18q	47033556	47035145	1590	7	0.857	Promoter(<=1kb)	476	ELOA2	51224
513886	18q	58535186	58537515	2330	10	0.4	Promoter(<=1kb)	0	ALPK2	115701
513886	19p	4510548	4513547	3000	20	0.45	Exon(exon3of6)	4157	PLIN4	729359
513886	19p	5455600	5456439	840	6	0.5	Promoter(<=1kb)	183	ZNRF4	148066
513886	19p	8937644	8939234	1591	6	0.833	Exon(exon5of84)	-41554	MUC16	94025
513886	19p	8946306	8951868	5563	18	0.667	Exon(exon3of84)	29474	MUC16	94025
513886	19p	8959116	8962299	3184	12	0.583	Exon(exon3of84)	19043	MUC16	94025
513886	19p	8963397	8967127	3731	21	0.667	Exon(exon3of84)	14215	MUC16	94025
513886	19p	8971838	8978096	6259	15	0.533	Exon(exon1of84)	3246	MUC16	94025
513886	19p	15087213	15088040	828	9	0.333	Promoter(<=1kb)	233	OR1I1	126370
513886	19p	18264798	18267409	2612	12	0.583	5'UTR	7002	IQCN	80726
513886	19p	21971930	21974500	2571	10	0.7	Exon(exon4of4)	14408	ZNF208	7757
513886	19q	40880128	40880622	495	7	0.286	Promoter(<=1kb)	-38	CYP2A7	1549
513886	19q	43913423	43914878	1456	9	0.556	Exon(exon10of10)	4861	ZNF45	7596
513886	19q	44106512	44108078	1567	7	0	Exon(exon6of6)	-4103	ZNF225	7768
513886	19q	52384174	52385296	1123	6	0	Exon(exon4of4)	12373	ZNF528-AS1	102724105
513886	19q	52437918	52439242	1325	7	0.429	Exon(exon4of4)	6504	ZNF534	147658
513886	19q	55771923	55773169	1247	6	0.5	Promoter(2-3kb)	2782	RFPL4AL1	729974
513886	19q	55912302	55913166	865	6	0.667	Exon(exon5of12)	19145	NLRP13	126204
513886	20p	1635288	1636423	1136	6	0.333	Promoter(1-2kb)	1662	SIRPG-AS1	101929010
513886	20p	5922421	5923643	1223	9	0.667	Exon(exon4of5)	6923	CHGB	1114
513886	20q	63349752	63350772	1021	6	0.5	3'UTR	3794	CHRNA4	1137
513886	20q	63561666	63565531	3866	12	0.667	Promoter(<=1kb)	-61	HELZ2	85441
513886	21q	44550835	44551416	582	6	0.833	Promoter(<=1kb)	89	KRTAP10-2	386679
513886	21q	44637474	44638041	568	9	0.333	Promoter(<=1kb)	118	KRTAP10-10	353333
513886	22q	22352950	22353380	431	16	0.5	Exon(exon1of2)	30478	BMS1P20	96610
513886	22q	36191154	36191906	753	6	0.667	3'UTR	9971	APOL4	80832
513886	22q	49884187	49884994	808	6	0.333	Exon(exon2of2)	22924	ALG12	79087
513886	23p	8170039	8170141	103	6	0.5	Promoter(1-2kb)	1126	VCX2	51480
513886	23p	35802148	35803010	863	7	0.571	5'UTR	3357	MAGEB16	139604
513886	23q	141905856	141906385	530	9	0.778	Promoter(1-2kb)	1054	MAGEC1	9947
513944	1p	11766028	11768307	2280	6	0.833	Promoter(<=1kb)	0	C1orf167	284498
513944	1p	12893249	12893472	224	9	0.444	Exon(exon4of4)	4798	PRAMEF10	343071

513944	1p	13369166	13369564	399	7	0.714	Promoter(2-3kb)	2336	PRAMEF19	645414
513944	1p	13370686	13371119	434	7	0.429	Promoter(<=1kb)	781	PRAMEF19	645414
513944	1p	16058491	16060000	1510	10	0.9	Exon(exon5of7)	6168	CLCNKB	1188
513944	1p	18481403	18482217	815	7	0.714	Promoter(<=1kb)	421	KLHDC7A	127707
513944	1p	23874604	23875430	827	9	0.556	Exon(exon2of2)	-6310	FUCA1	2517
513944	1p	40067594	40067675	82	6	0	Promoter(<=1kb)	324	CAP1	10487
513944	1p	89186388	89186419	32	9	0.556	Promoter(<=1kb)	107	GBP4	115361
513944	1q	152303673	152313891	10219	38	0.579	Promoter(<=1kb)	0	FLG-AS1	339400
513944	1q	152759630	152760825	1196	6	0.5	Exon(exon1of1)	-15547	LCE1F	353137
513944	1q	158765805	158766655	851	6	0.5	Promoter(<=1kb)	47	OR6N1	128372
513944	1q	169540901	169542882	1982	10	0.3	Exon(exon13of25)	-25156	F5	2153
513944	1q	183647749	183648558	810	7	0.429	Exon(exon2of2)	4758	APOBEC4	403314
513944	1q	214640144	214642954	2811	13	0.462	Exon(exon12of20)	-5013	CENPF	1063
513944	1q	214644872	214647181	2310	10	0.4	Promoter(<=1kb)	-786	CENPF	1063
513944	1q	228315976	228318026	2051	6	0.5	Exon(exon50of81)	6492	OBSCN	84033
513944	1q	247841312	247841582	271	6	0.833	Promoter(<=1kb)	314	OR11L1	391189
513944	1q	248294677	248295458	782	9	0.667	Promoter(<=1kb)	142	OR2T12	127064
513944	2p	29002636	29003646	1011	6	0.333	Exon(exon5of20)	-10821	TOGARAM2	165186
513944	2p	29071763	29073000	1238	6	0.667	Promoter(1-2kb)	1523	PCARE	388939
513944	2p	48580657	48582454	1798	7	0.571	Promoter(<=1kb)	0	STON1	11037
513944	2q	130193975	130194181	207	6	0.667	Exon(exon4of5)	4258	TUBA3E	112714
513944	2q	130914528	130916712	2185	6	0.333	Promoter(<=1kb)	0	ARHGEF4	50649
513944	2q	132781993	132784972	2980	8	0.5	Promoter(<=1kb)	0	NCKAP5	344148
513944	2q	167246794	167248478	1685	7	0.714	Promoter(<=1kb)	-204	XIRP2	129446
513944	2q	184936178	184937636	1459	6	0.333	Exon(exon4of4)	69813	ZNF804A	91752
513944	2q	185789865	185794632	4768	10	0.8	Promoter(<=1kb)	0	FSIP2	401024
513944	2q	185805377	185808170	2794	6	0.333	Promoter(<=1kb)	0	FSIP2	401024
513944	2q	237762685	237764060	1376	10	0.5	Exon(exon8of8)	-4137	LRRFIP1	9208
513944	2q	238130416	238131546	1131	6	0.333	Promoter(1-2kb)	1468	ESPNL	339768
513944	2q	240041845	240042131	287	6	0.167	Downstream(2-3kb)	3941	OR6B3	150681
513944	3p	31989532	31990905	1374	7	0.286	Exon(exon2of2)	7761	ZNF860	344787
513944	3p	75736880	75739007	2128	54	0.611	Promoter(<=1kb)	0	MIR4273	100422955
513944	3q	98264413	98265098	686	7	0.571	Promoter(<=1kb)	128	OR5H6	79295
513944	4p	5988383	5989749	1367	7	0.571	Promoter(<=1kb)	0	C4orf50	389197
513944	4p	6300792	6302360	1569	6	0.833	Exon(exon8of8)	6021	WFS1	7466
513944	4p	7433331	7434512	1182	6	0.833	Promoter(<=1kb)	418	PSAPL1	768239
513944	4q	186619481	186621601	2121	7	0.286	Exon(exon10of27)	-9411	FAT1	2195
513944	4q	186706638	186709461	2824	7	0.714	Exon(exon2of27)	14372	FAT1	2195
513944	5p	795818	796218	401	6	0.833	3'UTR	4927	ZDHHC11	79844
513944	5q	79728956	79735735	6780	23	0.435	Promoter(2-3kb)	-2407	CMYA5	202333
513944	5q	83537326	83539905	2580	6	0.333	Promoter(1-2kb)	1712	VCAN	1462
513944	5q	140848579	140850786	2208	6	0.5	Promoter(<=1kb)	807	PCDHA9	9752
513944	5q	141174000	141175025	1026	7	0.857	Promoter(1-2kb)	1356	PCDHB7	56129
513944	5q	141187690	141188770	1081	6	0.5	Promoter(<=1kb)	529	PCDHB9	56127
513944	5q	141955676	141957660	1985	6	0.667	Promoter(<=1kb)	-668	RNF14	9604
513944	5q	148826877	148828070	1194	6	1	Promoter(1-2kb)	1632	ADRB2	154
513944	5q	151521550	151522069	520	6	0.667	Promoter(<=1kb)	79	MIR6499	102465246
513944	6p	46858771	46859502	732	8	0.5	Exon(exon17of21)	3802	ADGRF5	221395
513944	6q	159233455	159234370	916	10	0.5	Exon(exon11of23)	15158	FNDC1	84624
513944	7p	6330446	6330944	499	6	1	Exon(exon2of2)	7749	FAM220A	84792
513944	7q	64991278	64992758	1481	6	0.667	Promoter(<=1kb)	-242	ZNF117	51351
513944	7q	100958721	100960873	2153	58	0.448	Promoter(<=1kb)	756	MUC3A	4584
513944	7q	100995547	100995785	239	6	0.833	Exon(exon5of15)	-17269	MUC12	10071
513944	7q	101034361	101040583	6223	39	0.513	Exon(exon3of12)	-3128	MUC17	140453
513944	7q	149818015	149819792	1778	6	0.667	Promoter(2-3kb)	-2352	SSPO	23145
513944	8p	10607375	10612307	4933	16	0.625	Exon(exon4of4)	42836	RP1L1	94137
513944	8p	11331234	11332082	849	6	0.667	Promoter(<=1kb)	346	SLC35G5	83650
513944	8p	12132477	12133940	1464	11	0.727	Promoter(<=1kb)	498	USP17L7	392197
513944	8q	123651873	123652634	762	6	0.333	Promoter(<=1kb)	316	KLHL38	340359
513944	8q	142664439	142665852	1414	7	0.857	Exon(exon2of2)	4118	JRK	8629
513944	9p	116800	117934	1135	7	0.714	Promoter(<=1kb)	270	FOXD4	2298
513944	9p	214679	215269	591	6	0.5	Promoter(<=1kb)	0	DOCK8	81704
513944	9q	76175237	76175296	60	10	0.8	Exon(exon14of14)	-13343	PCSK5	5125
513944	9q	76703555	76706360	2806	8	0.5	Promoter(<=1kb)	0	PCA3	50652
513944	9q	104598545	104599361	817	12	0.583	Promoter(<=1kb)	52	OR13C5	138799
513944	9q	122553263	122554071	809	8	0.5	Promoter(<=1kb)	93	OR1N2	138882
513944	9q	122628595	122629398	804	8	0.375	Promoter(<=1kb)	175	OR1B1	347169
513944	9q	122749914	122750547	634	6	0.833	Promoter(<=1kb)	174	OR1L6	392390
513944	9q	135484695	135487213	2519	9	0.333	Promoter(1-2kb)	1332	PPP1R26	9858
513944	9q	135547960	135548795	836	6	0.667	Promoter(1-2kb)	1805	OBP2A	29991
513944	10p	47663	48605	943	7	0.714	Promoter(<=1kb)	664	TUBB8	347688
513944	10q	46549378	46550723	1346	26	0.654	Exon(exon3of3)	4807	GPRIN2	9721
513944	10q	49323504	49326817	3314	10	0.5	Exon(exon3of3)	24230	C10orf71	118461
513944	10q	128103129	128108204	5076	15	0.667	Promoter(<=1kb)	-1	MKI67	4288
513944	11p	244106	244197	92	8	0.5	Promoter(<=1kb)	-232	PSMD13	5719
513944	11p	1246095	1248605	2511	12	0.5	Promoter(1-2kb)	1071	MUC5B-AS1	112577518
513944	11p	5177978	5178478	501	6	0.167	Promoter(<=1kb)	186	OR52Z1	283110

513944	11p	5323542	5324256	715	6	0.5	Promoter(<=1kb)	41	OR51B2	79345
513944	11p	5389704	5390350	647	7	0.429	Promoter(<=1kb)	327	OR51M1	390059
513944	11p	5422212	5423123	912	11	0.636	Promoter(<=1kb)	101	OR51Q1	390061
513944	11p	5581045	5581738	694	8	0.375	Promoter(<=1kb)	168	OR52B6	340980
513944	11p	5884818	5885061	244	6	0.333	Promoter(<=1kb)	547	OR52E4	390081
513944	11p	11351961	11352736	776	9	0.222	Promoter(<=1kb)	514	CSNK2A3	283106
513944	11p	12293628	12294368	741	7	0.857	Exon(exon29of35)	6728	MICALCL	84953
513944	11q	58214757	58215722	966	6	0.333	Promoter(<=1kb)	12	OR1S1	219959
513944	11q	64315797	64315856	60	8	0.75	Promoter(1-2kb)	1485	TRMT112	51504
513944	11q	66560202	66562261	2060	6	0.333	Exon(exon14of21)	3873	CTSF	8722
513944	11q	123906790	123907324	535	6	0.667	Promoter(<=1kb)	644	OR8D4	338662
513944	11q	124015600	124016477	878	7	0.143	Promoter(<=1kb)	25	OR10G4	390264
513944	11q	124038366	124038988	623	9	0.889	Promoter(<=1kb)	13	OR10G7	390265
513944	12p	4626568	4628152	1585	9	0.333	Exon(exon5of6)	14051	DYRK4	8798
513944	12p	6453119	6453670	552	6	0.667	Promoter(<=1kb)	633	TAPBPL	55080
513944	12p	31981130	31983004	1875	6	0.167	Exon(exon4of6)	-4162	RESF1	55196
513944	12q	52571389	52572653	1265	6	0.5	Promoter(1-2kb)	1163	KRT74	121391
513944	13q	24434450	24435347	898	7	0.571	Exon(exon31of34)	19787	PARP4	143
513944	13q	25096659	25097182	524	10	0.4	Promoter(<=1kb)	791	PABPC3	5042
513944	13q	102732474	102733933	1460	7	0.286	Exon(exon4of4)	25139	CCDC168	643677
513944	14q	19975713	19976448	736	9	0.444	Promoter(<=1kb)	269	OR4K15	81127
513944	14q	20060048	20060884	837	8	0.625	Promoter(<=1kb)	3	OR4L1	122742
513944	14q	70457520	70458948	1429	8	0.5	Exon(exon2of2)	5346	ADAM21	8747
513944	14q	103110058	103110438	381	6	0.667	Promoter(2-3kb)	2436	EXOC3L4	91828
513944	14q	104175275	104177810	2536	9	0.333	Exon(exon12of15)	36235	KIF26A	26153
513944	14q	104939262	104942618	3357	10	0.2	5'UTR	7102	PLD4	122618
513944	14q	104943622	104945444	1823	7	0.571	Exon(exon6of6)	9958	AHNAK2	113146
513944	14q	104947901	104949943	2043	14	0.571	Exon(exon6of6)	5459	AHNAK2	113146
513944	15q	23439979	23442067	2089	13	0.538	5'UTR	5167	GOLGA6L2	283685
513944	15q	88854874	88855594	721	6	0.833	Exon(exon12of18)	7631	ACAN	176
513944	15q	88857108	88859365	2258	6	0	Exon(exon12of18)	9865	ACAN	176
513944	15q	99130060	99132394	2335	6	0.5	Exon(exon4of5)	7348	TTC23	64927
513944	16p	1228744	1229622	879	6	0.833	Promoter(<=1kb)	540	TPSB2	64499
513944	16p	1256345	1256816	472	6	0.833	Promoter(<=1kb)	276	TPSD1	23430
513944	16p	1486371	1488463	2093	8	0.75	Promoter(<=1kb)	4	PTX4	390667
513944	16p	4207130	4208004	875	6	0.5	Exon(exon2of7)	31739	SRL	6345
513944	16q	88714632	88717113	2482	8	0.375	Promoter(<=1kb)	0	MIR4722	100616167
513944	16q	89226895	89228289	1395	7	0.571	Promoter(2-3kb)	2261	ZNF778	197320
513944	17p	744917	746966	2050	8	0.875	3'UTR	5072	GEMIN4	50628
513944	17p	2299649	2300159	511	6	0.167	Exon(exon2of19)	-3224	SRR	63826
513944	17p	10638198	10641099	2902	7	0.286	Exon(exon19of41)	-8169	MYH3	4621
513944	17p	21300581	21300954	374	11	0.727	3'UTR	9112	MAP2K3	5606
513944	17q	53823368	53824891	1524	7	0.857	Promoter(<=1kb)	441	KIF2B	84643
513944	17q	81645135	81645417	283	6	0.333	Promoter(2-3kb)	2722	TSPAN10	83882
513944	18p	11609646	11610164	519	9	0.778	Promoter(<=1kb)	50	SLC35G4	646000
513944	18p	14542649	14543140	492	6	0.5	Promoter(<=1kb)	6	POTEC	388468
513944	18q	58535186	58538030	2845	19	0.526	Promoter(<=1kb)	0	ALPK2	115701
513944	19p	4510548	4513547	3000	21	0.476	Exon(exon3of6)	4157	PLIN4	729359
513944	19p	5455600	5456439	840	8	0.625	Promoter(<=1kb)	183	ZNRF4	148066
513944	19p	8936591	8938720	2130	7	1	Exon(exon5of84)	-40501	MUC16	94025
513944	19p	8946254	8947220	967	7	0.286	Exon(exon3of84)	34122	MUC16	94025
513944	19p	8959518	8962066	2549	6	0.333	Exon(exon3of84)	19276	MUC16	94025
513944	19p	8964274	8967127	2854	19	0.632	Exon(exon3of84)	14215	MUC16	94025
513944	19p	10338682	10339609	928	6	0.333	Promoter(<=1kb)	10	ICAM3	3385
513944	19p	12075333	12077046	1714	7	0.286	Promoter(<=1kb)	6	ZNF788P	388507
513944	19p	12430718	12432437	1720	8	0.375	3'UTR	8584	ZNF443	10224
513944	19p	18264798	18267409	2612	12	0.583	5'UTR	7002	IQCN	80726
513944	19q	39886005	39886439	435	6	0.333	Promoter(<=1kb)	853	FCGBP	8857
513944	19q	43204774	43205504	731	6	0.833	Promoter(<=1kb)	47	PSG4	5672
513944	19q	43913423	43914878	1456	8	0.5	Exon(exon10of10)	4861	ZNF45	7596
513944	19q	43966037	43967171	1135	6	0.167	Promoter(<=1kb)	-691	ZNF155	7711
513944	19q	44327836	44329698	1863	6	0.5	Exon(exon4of4)	-22790	ZNF235	9310
513944	19q	48873325	48875925	2601	11	0.545	Promoter(<=1kb)	904	PPP1R15A	23645
513944	19q	51745958	51746963	1006	6	0.333	Exon(exon3of3)	3848	FPR1	2357
513944	19q	51872787	51873699	913	10	0.5	Promoter(<=1kb)	-940	ZNF577	84765
513944	19q	52437918	52439242	1325	7	0.429	Exon(exon4of4)	6504	ZNF534	147658
513944	19q	55911888	55913166	1279	7	0.714	Exon(exon5of12)	19145	NLRP13	126204
513944	19q	58368293	58368875	583	7	0.429	Exon(exon3of3)	-5445	ZNF497	162968
513944	20p	5922421	5923394	974	7	0.571	Exon(exon4of5)	6923	CHGB	1114
513944	20q	59191988	59194085	2098	6	0.667	Promoter(<=1kb)	968	ZNF831	128611
513944	20q	62812284	62813587	1304	6	1	Promoter(2-3kb)	-2657	COL9A3	1299
513944	20q	63561666	63565531	3866	12	0.667	Promoter(<=1kb)	-61	HELZ2	85441
513944	21q	26843740	26844859	1120	6	0.667	Promoter(<=1kb)	0	ADAMTS1	9510
513944	21q	44591587	44592404	818	7	0.286	Promoter(<=1kb)	101	KRTAP10-6	386674
513944	21q	44600627	44601692	1066	11	0.636	Promoter(<=1kb)	30	KRTAP10-7	386675
513944	21q	44637474	44638143	670	11	0.455	Promoter(<=1kb)	118	KRTAP10-10	353333
513944	22q	22352950	22353298	349	13	0.538	Exon(exon1of2)	30478	BMS1P20	96610

513944	22q	36191154	36191906	753	6	0.667	3'UTR	9971	APOL4	80832
513944	22q	37723206	37726455	3250	6	0.667	3'UTR	-19778	TRIOBP	11078
513944	22q	39100331	39102033	1703	6	0.833	Promoter(<=1kb)	52	APOBEC3H	164668
513944	22q	49884187	49885288	1102	6	0.167	Exon(exon2of2)	22630	ALG12	79087
513944	23p	8170039	8170141	103	6	0.5	Promoter(1-2kb)	1126	VCX2	51480
513944	23q	136874183	136874347	165	7	0.714	Promoter(<=1kb)	-201	RBMX	27316
514442	1p	16058491	16059570	1080	6	0.667	Exon(exon6of7)	6168	CLCNKB	1188
514442	1p	18481042	18482032	991	10	0.7	Promoter(<=1kb)	60	KLHDC7A	127707
514442	1p	23874604	23875430	827	8	0.5	Exon(exon2of2)	-6310	FUCA1	2517
514442	1p	40067594	40067675	82	6	0	Promoter(<=1kb)	324	CAP1	10487
514442	1p	88983595	88983800	206	6	0.5	Exon(exon3of3)	4555	KYAT3	56267
514442	1p	89186388	89186419	32	9	0.556	Promoter(<=1kb)	107	GBP4	115361
514442	1q	152218469	152221375	2907	16	0.688	Promoter(2-3kb)	2818	HRNR	388697
514442	1q	152303673	152313891	10219	38	0.605	Promoter(<=1kb)	0	FLG-AS1	339400
514442	1q	156669844	156670886	1043	6	1	Exon(exon4of4)	6521	NES	10763
514442	1q	158765805	158766655	851	6	0.5	Promoter(<=1kb)	47	OR6N1	128372
514442	1q	169542317	169542882	566	8	0.125	Exon(exon13of25)	-26572	F5	2153
514442	1q	183647749	183648558	810	7	0.429	Exon(exon2of2)	4758	APOBEC4	403314
514442	1q	201206099	201209342	3244	10	0.6	Promoter(1-2kb)	1017	IGFN1	91156
514442	1q	201210956	201212792	1837	6	0.333	Promoter(<=1kb)	0	IGFN1	91156
514442	1q	228315976	228318026	2051	7	0.571	Exon(exon50of81)	6492	OBSCN	84033
514442	1q	247841312	247841582	271	6	0.833	Promoter(<=1kb)	314	OR11L1	391189
514442	1q	247949325	247949738	414	10	0.3	Promoter(<=1kb)	467	OR2L8	391190
514442	1q	248294677	248295458	782	7	0.571	Promoter(<=1kb)	142	OR2T12	127064
514442	2p	48580657	48582454	1798	8	0.625	Promoter(<=1kb)	0	STON1	11037
514442	2q	130914528	130916712	2185	6	0.333	Promoter(<=1kb)	0	ARHGEF4	50649
514442	2q	132781993	132785012	3020	10	0.6	Promoter(<=1kb)	0	NCKAP5	344148
514442	2q	167246794	167248478	1685	8	0.625	Promoter(<=1kb)	-204	XIRP2	129446
514442	2q	178739433	178741811	2379	6	0.5	Exon(exon45of191)	26014	TTN	7273
514442	2q	238130416	238131546	1131	6	0.333	Promoter(1-2kb)	1468	ESPNL	339768
514442	3p	31989643	31990470	828	6	0.167	Exon(exon2of2)	7872	ZNF860	344787
514442	3p	75736880	75739007	2128	45	0.6	Promoter(<=1kb)	0	MIR4273	100422955
514442	3q	98169021	98169594	574	6	0.667	Exon(exon2of2)	19695	OR5H14	403273
514442	3q	98264413	98265137	725	6	0.667	Promoter(<=1kb)	128	OR5H6	79295
514442	4p	5988383	5989749	1367	7	0.571	Promoter(<=1kb)	0	C4orf50	389197
514442	4p	6300792	6302360	1569	7	0.857	Exon(exon8of8)	6021	WFS1	7466
514442	4p	8227004	8228508	1505	7	0.143	Promoter(<=1kb)	-24	SH3TC1	54436
514442	4p	38774486	38775552	1067	8	0.5	Promoter(<=1kb)	644	TLR10	81793
514442	4q	186706638	186709436	2799	7	0.714	Exon(exon2of27)	14397	FAT1	2195
514442	5p	41158761	41160209	1449	6	0.833	Promoter(<=1kb)	160	C6	729
514442	5q	83537326	83539905	2580	6	0.333	Promoter(1-2kb)	1712	VCAN	1462
514442	5q	141174000	141175025	1026	7	0.714	Promoter(1-2kb)	1356	PCDHB7	56129
514442	5q	141178631	141180333	1703	6	0.833	Promoter(<=1kb)	841	PCDHB8	56128
514442	5q	141183999	141184688	690	6	0.833	Promoter(2-3kb)	-2473	PCDHB9	56127
514442	5q	141187109	141189425	2317	6	0.5	Promoter(<=1kb)	0	PCDHB9	56127
514442	5q	141192663	141194472	1810	6	1	Promoter(<=1kb)	310	PCDHB10	56126
514442	5q	141955356	141957660	2305	7	0.714	Promoter(<=1kb)	-668	RNF14	9604
514442	5q	148826877	148828070	1194	6	1	Promoter(1-2kb)	1632	ADRB2	154
514442	5q	151565922	151568158	2237	9	0.778	Promoter(<=1kb)	786	FAT2	2196
514442	6p	46858771	46859502	732	8	0.5	Exon(exon17of21)	3802	ADGRF5	221395
514442	6q	149888581	149890867	2287	7	0.714	Promoter(<=1kb)	0	RAET1E-AS1	100652739
514442	6q	159233455	159234370	916	10	0.5	Exon(exon11of23)	15158	FNDC1	84624
514442	7p	12369637	12370736	1100	6	0.667	3'UTR	-13307	VWDE	221806
514442	7p	38353718	38353991	274	8	0.625	Exon(exon2of2)	3699	TRG-AS1	100506776
514442	7q	89334686	89336295	1610	6	0.5	Exon(exon4of4)	116532	ZNF804B	219578
514442	7q	100958721	100960873	2153	58	0.448	Promoter(<=1kb)	756	MUC3A	4584
514442	7q	100991195	100992398	1204	7	0.571	Exon(exon5of15)	-20656	MUC12	10071
514442	7q	100995575	100995785	211	7	0.714	Exon(exon5of15)	-17269	MUC12	10071
514442	7q	101004421	101004836	416	6	0.667	Exon(exon5of15)	-8218	MUC12	10071
514442	7q	101034361	101040583	6223	39	0.487	Exon(exon3of12)	-3128	MUC17	140453
514442	8p	10607245	10608432	1188	8	0.5	Exon(exon4of4)	46711	RP1L1	94137
514442	8p	10609614	10610662	1049	8	0.5	Exon(exon4of4)	44481	RP1L1	94137
514442	8p	11331234	11332082	849	6	0.667	Promoter(<=1kb)	346	SLC35G5	83650
514442	8p	13021128	13022030	903	8	0.125	Exon(exon5of5)	9115	TRMT9B	57604
514442	8q	123651655	123652634	980	7	0.571	Promoter(<=1kb)	316	KLHL38	340359
514442	8q	142664564	142665970	1407	6	0.833	Exon(exon2of2)	4000	JRK	8629
514442	9p	116800	117800	1001	9	0.778	Promoter(<=1kb)	404	FOXD4	2298
514442	9q	76175237	76175296	60	11	0.818	Exon(exon14of14)	-13343	PCSK5	5125
514442	9q	76705724	76707804	2081	6	0.5	Promoter(<=1kb)	666	PCA3	50652
514442	9q	87886533	87888536	2004	8	0.625	Exon(exon4of4)	3656	SPATA31E1	286234
514442	9q	104504315	104505071	757	6	0.5	Promoter(<=1kb)	52	OR13F1	138805
514442	9q	122553263	122554071	809	8	0.5	Promoter(<=1kb)	93	OR1N2	138882
514442	9q	122628595	122629398	804	6	0.333	Promoter(<=1kb)	175	OR1B1	347169
514442	9q	122749914	122750547	634	6	0.833	Promoter(<=1kb)	174	OR1L6	392390
514442	10q	46549378	46550723	1346	25	0.64	Exon(exon3of3)	4807	GPRIN2	9721
514442	10q	49323504	49326817	3314	10	0.5	Exon(exon3of3)	24230	C10orf71	118461
514442	10q	128103129	128104830	1702	10	0.6	Promoter(<=1kb)	-1	MKI67	4288

514442	11p	244106	244197	92	8	0.5	Promoter(<=1kb)	-232	PSMD13	5719
514442	11p	5058716	5059541	826	6	0.667	Promoter(<=1kb)	86	OR52E2	119678
514442	11p	5177978	5178478	501	6	0.167	Promoter(<=1kb)	186	OR52Z1	283110
514442	11p	5323451	5324256	806	6	0.5	Promoter(<=1kb)	41	OR51B2	79345
514442	11p	5515079	5515931	853	7	0.429	Promoter(<=1kb)	768	UBQLNL	143630
514442	11p	5544676	5545259	584	6	0.5	Promoter(<=1kb)	290	OR52H1	390067
514442	11p	5581045	5581738	694	9	0.444	Promoter(<=1kb)	168	OR52B6	340980
514442	11p	5788000	5788760	761	8	0.75	Promoter(<=1kb)	56	OR52N1	79473
514442	11p	5841302	5841883	582	9	0.333	Promoter(<=1kb)	14	OR52E6	390078
514442	11p	11351961	11352736	776	9	0.222	Promoter(<=1kb)	514	CSNK2A3	283106
514442	11p	12293639	12294842	1204	7	0.714	Exon(exon29of35)	6739	MICALCL	84953
514442	11p	43942293	43943348	1056	9	0.778	Promoter(<=1kb)	0	C11orf96	387763
514442	11q	54603136	54603820	685	6	0.667	Promoter(<=1kb)	178	OR4C46	119749
514442	11q	58214757	58215722	966	8	0.25	Promoter(<=1kb)	12	OR1S1	219959
514442	11q	64315797	64315856	60	8	0.75	Promoter(1-2kb)	1485	TRMT112	51504
514442	11q	82732630	82733184	555	6	0.833	Promoter(<=1kb)	680	FAM181B	220382
514442	11q	85724687	85725825	1139	6	0.5	Promoter(<=1kb)	0	SYTL2	54843
514442	11q	123906790	123907324	535	6	0.667	Promoter(<=1kb)	644	OR8D4	338662
514442	11q	124038930	124038988	59	6	1	Promoter(<=1kb)	13	OR10G7	390265
514442	11q	124382526	124383285	760	8	0.625	Promoter(<=1kb)	58	OR8B2	26595
514442	12p	4626568	4628549	1982	11	0.455	Exon(exon5of6)	14051	DYRK4	8798
514442	12p	8222174	8223514	1341	6	0.667	Exon(exon5of6)	4073	FAM90A1	55138
514442	12q	48525773	48526223	451	6	0.5	Promoter(<=1kb)	141	OR8S1	341568
514442	12q	52571389	52572653	1265	6	0.5	Promoter(1-2kb)	1163	KRT74	121391
514442	13q	25096870	25097182	313	8	0.5	Promoter(1-2kb)	1002	PABPC3	5042
514442	13q	102732474	102733933	1460	6	0.333	Exon(exon4of4)	25139	CCDC168	643677
514442	14q	19935750	19936577	828	6	0.833	Promoter(<=1kb)	142	OR4K1	79544
514442	14q	22633879	22634450	572	10	0.4	Exon(exon2of2)	32212	ABHD4	63874
514442	14q	44504986	44506403	1418	6	0.667	Promoter(<=1kb)	880	FSCB	84075
514442	14q	70457532	70458540	1009	10	0.3	Exon(exon2of2)	5358	ADAM21	8747
514442	14q	104175275	104177810	2536	11	0.364	Exon(exon12of15)	36235	KIF26A	26153
514442	14q	104947943	104951938	3996	11	0.364	Exon(exon6of6)	3464	AHNAK2	113146
514442	15q	20534501	20535129	629	7	0.429	Exon(exon8of9)	6671	GOLGA6L6	727832
514442	15q	23439979	23442067	2089	12	0.583	5'UTR	5167	GOLGA6L2	283685
514442	15q	40621642	40624039	2398	7	0.286	Promoter(<=1kb)	0	KNL1	57082
514442	15q	85579423	85581800	2378	14	0.571	Promoter(1-2kb)	-1110	AKAP13	11214
514442	16q	74391650	74392004	355	8	0.625	Exon(exon7of7)	13772	NPIPB15	440348
514442	16q	89100686	89101050	365	8	0.625	Promoter(<=1kb)	24	ACSF3	197322
514442	16q	89226863	89228289	1427	8	0.625	Promoter(2-3kb)	2229	ZNF778	197320
514442	17p	744946	746966	2021	6	1	3'UTR	5072	GEMIN4	50628
514442	17p	2299649	2300159	511	6	0.167	Exon(exon2of19)	-3224	SRR	63826
514442	17p	10638198	10641099	2902	6	0.333	Exon(exon19of41)	-8169	MYH3	4621
514442	17p	21300581	21300954	374	8	0.75	3'UTR	9112	MAP2K3	5606
514442	17p	21415470	21416370	901	6	1	Exon(exon3of3)	10334	KCNJ12	3768
514442	17q	76293419	76294016	598	6	0.5	Promoter(2-3kb)	-2167	QRICH2	84074
514442	17q	81645135	81645595	461	7	0.429	Promoter(2-3kb)	2722	TSPAN10	83882
514442	18p	11609904	11610469	566	8	0.625	Promoter(<=1kb)	308	SLC35G4	646000
514442	18q	58535186	58537515	2330	10	0.4	Promoter(<=1kb)	0	ALPK2	115701
514442	19p	4510711	4513547	2837	19	0.474	Exon(exon3of6)	4157	PLIN4	729359
514442	19p	5455600	5456439	840	7	0.571	Promoter(<=1kb)	183	ZNRF4	148066
514442	19p	8948231	8951868	3638	13	0.615	Exon(exon3of84)	29474	MUC16	94025
514442	19p	8959403	8962066	2664	7	0.429	Exon(exon3of84)	19276	MUC16	94025
514442	19p	8964274	8967127	2854	21	0.667	Exon(exon3of84)	14215	MUC16	94025
514442	19p	10113850	10115580	1731	6	0.833	Promoter(1-2kb)	1108	EIF3G	8666
514442	19p	12075333	12077046	1714	7	0.286	Promoter(<=1kb)	6	ZNF788P	388507
514442	19p	12430718	12432437	1720	10	0.4	3'UTR	8584	ZNF443	10224
514442	19p	21971930	21974500	2571	8	0.75	Exon(exon4of4)	14408	ZNF208	7757
514442	19p	22756294	22759533	3240	14	0.5	3'UTR	10449	ZNF99	7652
514442	19q	33205409	33207385	1977	6	0.667	Promoter(1-2kb)	1539	LRP3	4037
514442	19q	39893949	39894238	290	6	0.5	Exon(exon12of28)	-6657	FCGBP	8857
514442	19q	40880231	40880622	392	6	0.333	Promoter(<=1kb)	-141	CYP2A7	1549
514442	19q	43913423	43914878	1456	7	0.429	Exon(exon10of10)	4861	ZNF45	7596
514442	19q	43996326	43997366	1041	6	0.5	Exon(exon5of5)	5419	LOC10192806:	101928063
514442	19q	44106512	44108078	1567	7	0	Exon(exon6of6)	-4103	ZNF225	7768
514442	19q	47679530	47681217	1688	6	0.667	Promoter(<=1kb)	568	BICRA	29998
514442	19q	51745958	51746963	1006	6	0.333	Exon(exon3of3)	3848	FPR1	2357
514442	19q	52437918	52439242	1325	7	0.429	Exon(exon4of4)	6504	ZNF534	147658
514442	19q	53164551	53166239	1689	6	0.167	Exon(exon4of4)	-5476	ZNF347	84671
514442	19q	55911888	55913077	1190	6	0.667	Exon(exon5of12)	19234	NLRP13	126204
514442	20p	20052354	20052736	383	6	0	Promoter(<=1kb)	0	CFAP61	26074
514442	20q	63540866	63543452	2587	10	0.8	Exon(exon1of1)	-3490	PTK6	5753
514442	20q	63561666	63565531	3866	13	0.692	Promoter(<=1kb)	-61	HELZ2	85441
514442	21q	44637474	44638041	568	10	0.4	Promoter(<=1kb)	118	KRTAP10-10	353333
514442	22q	22352950	22353298	349	13	0.538	Exon(exon1of2)	30478	BMS1P20	96610
514442	22q	36191154	36191906	753	6	0.667	3'UTR	9971	APOL4	80832
514442	22q	36265284	36265796	513	6	1	Exon(exon6of6)	12120	APOL1	8542
514442	23p	8170039	8170141	103	6	0.5	Promoter(1-2kb)	1126	VCX2	51480

514442	23p	35802148	35803010	863	7	0.571	5'UTR	3357	MAGEB16	139604
514442	23q	136874183	136874416	234	6	1	Promoter(<=1kb)	-201	RBMX	27316
514970	1p	12795695	12795957	263	6	0.5	Promoter(1-2kb)	1004	PRAMEF1	65121
514970	1p	12847526	12847780	255	7	0.429	Promoter(<=1kb)	945	HNRNPCL1	343069
514970	1p	12859036	12860079	1044	6	0.333	Promoter(1-2kb)	1950	PRAMEF2	65122
514970	1p	13370686	13371119	434	7	0.429	Promoter(<=1kb)	781	PRAMEF19	645414
514970	1p	16058491	16060000	1510	13	0.846	Exon(exon5of7)	6168	CLCNKB	1188
514970	1p	18481403	18482217	815	6	0.667	Promoter(<=1kb)	421	KLHDC7A	127707
514970	1p	23874604	23875430	827	9	0.556	Exon(exon2of2)	-6310	FUCA1	2517
514970	1p	40067594	40067675	82	6	0	Promoter(<=1kb)	324	CAP1	10487
514970	1p	62273232	62274934	1703	6	0.167	Promoter(<=1kb)	-441	KANK4	163782
514970	1p	89186388	89186419	32	9	0.556	Promoter(<=1kb)	107	GBP4	115361
514970	1q	152218469	152221129	2661	10	0.5	Exon(exon3of3)	3064	HRNR	388697
514970	1q	152303673	152313891	10219	47	0.617	Promoter(<=1kb)	0	FLG-AS1	339400
514970	1q	158765805	158766655	851	6	0.5	Promoter(<=1kb)	47	OR6N1	128372
514970	1q	201206099	201209342	3244	11	0.636	Promoter(1-2kb)	1017	IGFN1	91156
514970	1q	228315976	228318049	2074	7	0.571	Exon(exon50of81)	6492	OBSCN	84033
514970	1q	232805207	232806505	1299	6	0.333	Promoter(<=1kb)	315	MAP10	54627
514970	1q	247841312	247841582	271	6	0.833	Promoter(<=1kb)	314	OR11L1	391189
514970	1q	247949325	247949738	414	10	0.3	Promoter(<=1kb)	467	OR2L8	391190
514970	2p	48580657	48582454	1798	7	0.571	Promoter(<=1kb)	0	STON1	11037
514970	2q	130914528	130916712	2185	6	0.333	Promoter(<=1kb)	0	ARHGEF4	50649
514970	2q	132781078	132784972	3895	10	0.5	Promoter(<=1kb)	0	NCKAP5	344148
514970	2q	217847583	217848559	977	6	0.833	Exon(exon19of33)	-5423	TNS1	7145
514970	2q	219539659	219540599	941	6	0.667	Promoter(2-3kb)	2498	CHPF	79586
514970	2q	233681970	233682693	724	6	0.667	Promoter(<=1kb)	0	UGT1A7	54577
514970	2q	233840612	233842185	1574	6	0.667	Promoter(<=1kb)	0	HJURP	55355
514970	2q	237762685	237764060	1376	10	0.5	Exon(exon8of8)	-4137	LRRFIP1	9208
514970	2q	238130416	238131546	1131	6	0.333	Promoter(1-2kb)	1468	ESPNL	339768
514970	3p	13570520	13571564	1045	6	0.167	Promoter(1-2kb)	1780	FBLN2	2199
514970	3p	75736880	75739243	2364	49	0.633	Promoter(<=1kb)	0	MIR4273	100422955
514970	4p	5988383	5989749	1367	7	0.571	Promoter(<=1kb)	0	C4orf50	389197
514970	4p	6300792	6302360	1569	8	0.75	Exon(exon8of8)	6021	WFS1	7466
514970	4q	154489498	154491312	1815	9	0.556	Promoter(<=1kb)	22	DCHS2	54798
514970	5q	79728956	79730716	1761	7	0.286	Exon(exon2of13)	-7426	CMYA5	202333
514970	5q	79731782	79734523	2742	13	0.308	Exon(exon2of13)	-3619	CMYA5	202333
514970	5q	83537326	83539905	2580	6	0.333	Promoter(1-2kb)	1712	VCAN	1462
514970	5q	140848579	140850786	2208	7	0.571	Promoter(<=1kb)	807	PCDHA9	9752
514970	5q	141174000	141175025	1026	6	0.833	Promoter(1-2kb)	1356	PCDHB7	56129
514970	5q	141183999	141184688	690	6	1	Promoter(2-3kb)	-2473	PCDHB9	56127
514970	5q	141955356	141957660	2305	6	0.667	Promoter(<=1kb)	-668	RNF14	9604
514970	5q	151565922	151568158	2237	9	0.778	Promoter(<=1kb)	786	FAT2	2196
514970	6p	27309272	27310722	1451	6	0.833	Promoter(1-2kb)	1448	POM121L2	94026
514970	6p	42745312	42746041	730	6	0.667	Promoter(<=1kb)	62	TBCC	6903
514970	6p	46858771	46859502	732	8	0.5	Exon(exon17of21)	3802	ADGRF5	221395
514970	6q	64591274	64591961	688	10	0.5	Exon(exon26of43)	121374	EYS	346007
514970	6q	149888581	149890867	2287	7	0.714	Promoter(<=1kb)	0	RAET1E-AS1	100652739
514970	6q	159233455	159234370	916	10	0.5	Exon(exon11of23)	15158	FNDC1	84624
514970	7p	20784995	20785358	364	6	1	Promoter(1-2kb)	1528	SP8	221833
514970	7p	28956184	28957525	1342	6	0.667	Promoter(<=1kb)	805	TRIL	9865
514970	7q	100958977	100960873	1897	52	0.442	Promoter(1-2kb)	1012	MUC3A	4584
514970	7q	100995547	100995785	239	6	0.833	Exon(exon5of15)	-17269	MUC12	10071
514970	7q	149818015	149819792	1778	6	0.667	Promoter(2-3kb)	-2352	SSPO	23145
514970	7q	149824094	149826695	2602	6	0.667	Promoter(<=1kb)	0	SSPO	23145
514970	8p	10607245	10608432	1188	8	0.5	Exon(exon4of4)	46711	RP1L1	94137
514970	8p	10609614	10612307	2694	10	0.7	Exon(exon4of4)	42836	RP1L1	94137
514970	8p	12132686	12133940	1255	6	0.667	Promoter(<=1kb)	498	USP17L7	392197
514970	8p	13021128	13022030	903	8	0.25	Exon(exon5of5)	9115	TRMT9B	57604
514970	8q	138151949	138153046	1098	6	0.667	Promoter(<=1kb)	0	FAM135B	51059
514970	9p	116832	117720	889	6	0.5	Promoter(<=1kb)	484	FOXD4	2298
514970	9q	70535957	70537292	1336	6	0.833	Exon(exon25of25)	-121333	KLF9	687
514970	9q	76705179	76707804	2626	8	0.5	Promoter(<=1kb)	121	PCA3	50652
514970	9q	76709523	76710843	1321	6	0.5	Promoter(<=1kb)	0	PRUNE2	158471
514970	9q	104504315	104505071	757	6	0.5	Promoter(<=1kb)	52	OR13F1	138805
514970	9q	104598545	104599318	774	6	0.333	Promoter(<=1kb)	95	OR13C5	138799
514970	9q	122553263	122554071	809	7	0.429	Promoter(<=1kb)	93	OR1N2	138882
514970	9q	122749914	122750547	634	6	0.833	Promoter(<=1kb)	174	OR1L6	392390
514970	9q	133255635	133256205	571	7	1	3'UTR	19009	ABO	28
514970	9q	135484803	135487156	2354	7	0.143	Promoter(1-2kb)	1440	PPP1R26	9858
514970	10q	46549378	46550723	1346	26	0.654	Exon(exon3of3)	4807	GPRIN2	9721
514970	10q	49323504	49325572	2069	7	0.429	Exon(exon3of3)	24230	C10orf71	118461
514970	11p	244106	244197	92	8	0.5	Promoter(<=1kb)	-232	PSMD13	5719
514970	11p	1194354	1196902	2549	7	0.571	Exon(exon34of49)	-26164	MUC5B	727897
514970	11p	1246095	1247378	1284	7	0.286	Promoter(2-3kb)	2298	MUC5B-AS1	112577518
514970	11p	5177978	5178478	501	6	0.167	Promoter(<=1kb)	186	OR52Z1	283110
514970	11p	5323362	5324256	895	7	0.429	Promoter(<=1kb)	41	OR51B2	79345
514970	11p	5389704	5390350	647	6	0.5	Promoter(<=1kb)	327	OR51M1	390059

514970	11p	5402638	5403322	685	9	0.444	Promoter(<=1kb)	41	OR51J1	79470
514970	11p	5422212	5423123	912	11	0.636	Promoter(<=1kb)	101	OR51Q1	390061
514970	11p	5440604	5441472	869	6	0.833	Promoter(<=1kb)	42	OR51I1	390063
514970	11p	5515185	5515931	747	6	0.333	Promoter(<=1kb)	768	UBQLNL	143630
514970	11p	5544676	5545259	584	6	0.5	Promoter(<=1kb)	290	OR52H1	390067
514970	11p	5581208	5581777	570	7	0.429	Promoter(<=1kb)	331	OR52B6	340980
514970	11p	5841302	5841883	582	9	0.333	Promoter(<=1kb)	14	OR52E6	390078
514970	11p	11351961	11352736	776	9	0.222	Promoter(<=1kb)	514	CSNK2A3	283106
514970	11p	12293639	12294538	900	8	0.625	Exon(exon29of35)	6739	MICALCL	84953
514970	11q	58214757	58215722	966	6	0.333	Promoter(<=1kb)	12	OR1S1	219959
514970	11q	58402523	58403265	743	8	0.5	Promoter(<=1kb)	144	OR5B3	441608
514970	11q	64116513	64118232	1720	7	0.714	Exon(exon2of2)	8702	MACROD1	28992
514970	11q	64315818	64315856	39	6	0.833	Promoter(1-2kb)	1485	TRMT112	51504
514970	11q	123906790	123907324	535	6	0.667	Promoter(<=1kb)	644	OR8D4	338662
514970	11q	124038366	124038988	623	7	1	Promoter(<=1kb)	13	OR10G7	390265
514970	11q	130914501	130915409	909	10	0.7	Promoter(1-2kb)	1035	SNX19	399979
514970	12p	4626568	4628549	1982	11	0.545	Exon(exon5of6)	14051	DYRK4	8798
514970	12p	8222174	8223514	1341	6	0.667	Exon(exon5of6)	4073	FAM90A1	55138
514970	13q	102732474	102733933	1460	6	0.333	Exon(exon4of4)	25139	CCDC168	643677
514970	14q	21634137	21634589	453	9	0.556	Promoter(<=1kb)	351	OR10G2	26534
514970	14q	22633879	22634450	572	9	0.333	Exon(exon2of2)	32212	ABHD4	63874
514970	14q	63599608	63599713	106	14	0.571	Exon(exon2of2)	41694	WDR89	112840
514970	14q	70457520	70458540	1021	12	0.417	Exon(exon2of2)	5346	ADAM21	8747
514970	14q	104175275	104177810	2536	10	0.3	Exon(exon12of15)	36235	KIF26A	26153
514970	14q	104939262	104942618	3357	12	0.167	5'UTR	7102	PLD4	122618
514970	14q	104943622	104953878	10257	66	0.545	Promoter(1-2kb)	1524	AHNAK2	113146
514970	15q	20534501	20535129	629	6	0.333	Exon(exon8of9)	6671	GOLGA6L6	727832
514970	15q	23439979	23442067	2089	10	0.6	5'UTR	5167	GOLGA6L2	283685
514970	15q	42690621	42692660	2040	6	0.5	Promoter(1-2kb)	1026	STARD9	57519
514970	15q	85579423	85581800	2378	14	0.571	Promoter(1-2kb)	-1110	AKAP13	11214
514970	16p	1228744	1229731	988	7	0.857	Promoter(<=1kb)	431	TPSB2	64499
514970	16q	74391650	74392004	355	8	0.625	Exon(exon7of7)	13772	NPIPB15	440348
514970	16q	89100686	89101050	365	8	0.625	Promoter(<=1kb)	24	ACSF3	197322
514970	16q	89226863	89228419	1557	11	0.636	Promoter(2-3kb)	2229	ZNF778	197320
514970	17p	744946	746966	2021	6	1	3'UTR	5072	GEMIN4	50628
514970	17p	21300581	21300978	398	11	0.727	3'UTR	9112	MAP2K3	5606
514970	17p	21415470	21416370	901	6	0.833	Exon(exon3of3)	10334	KCNJ12	3768
514970	17q	73236548	73236991	444	6	0.5	Promoter(<=1kb)	0	FAM104A	84923
514970	17q	81645135	81645417	283	6	0.333	Promoter(2-3kb)	2722	TSPAN10	83882
514970	18p	11609904	11610444	541	7	0.714	Promoter(<=1kb)	308	SLC35G4	646000
514970	18q	58535186	58538030	2845	19	0.526	Promoter(<=1kb)	0	ALPK2	115701
514970	19p	4511338	4513547	2210	11	0.273	Exon(exon3of6)	4157	PLIN4	729359
514970	19p	5455600	5456439	840	6	0.5	Promoter(<=1kb)	183	ZNRF4	148066
514970	19p	8946306	8950136	3831	12	0.5	Exon(exon3of84)	31206	MUC16	94025
514970	19p	8959518	8962066	2549	6	0.333	Exon(exon3of84)	19276	MUC16	94025
514970	19p	8964274	8967127	2854	19	0.632	Exon(exon3of84)	14215	MUC16	94025
514970	19p	15087452	15087953	502	6	0.167	Promoter(<=1kb)	472	OR1I1	126370
514970	19p	18264798	18267409	2612	12	0.583	5'UTR	7002	IQCN	80726
514970	19p	21971930	21974500	2571	6	0.667	Exon(exon4of4)	14408	ZNF208	7757
514970	19q	36996730	36997597	868	11	0.636	Exon(exon10of10)	5677	ZNF568	374900
514970	19q	37151928	37153149	1222	6	0.833	Exon(exon5of5)	19287	ZNF585A	199704
514970	19q	37885190	37888806	3617	8	0.625	Exon(exon6of6)	17788	WDR87	83889
514970	19q	39877222	39877880	659	6	0.5	Exon(exon20of28)	9412	FCGBP	8857
514970	19q	39886215	39886422	208	7	0.571	Promoter(<=1kb)	870	FCGBP	8857
514970	19q	40880231	40880622	392	6	0.333	Promoter(<=1kb)	-141	CYP2A7	1549
514970	19q	43913423	43914878	1456	9	0.556	Exon(exon10of10)	4861	ZNF45	7596
514970	19q	43996326	43997366	1041	6	0.5	Exon(exon5of5)	5419	LOC10192806[illegible]	101928063
514970	19q	44106512	44108078	1567	7	0	Exon(exon6of6)	-4103	ZNF225	7768
514970	19q	44327836	44329698	1863	6	0.5	Exon(exon4of4)	-22790	ZNF235	9310
514970	19q	52437918	52439242	1325	7	0.429	Exon(exon4of4)	6504	ZNF534	147658
514970	19q	55481625	55483456	1832	6	0.5	Promoter(1-2kb)	-1732	NAT14	57106
514970	19q	55857823	55858737	915	6	0.833	Promoter(<=1kb)	0	NLRP4	147945
514970	19q	55911888	55913077	1190	6	0.667	Exon(exon5of12)	19234	NLRP13	126204
514970	20p	5922421	5923394	974	7	0.571	Exon(exon4of5)	6923	CHGB	1114
514970	20p	20052354	20052736	383	6	0.167	Promoter(<=1kb)	0	CFAP61	26074
514970	20q	63349752	63350772	1021	6	0.5	3'UTR	3794	CHRNA4	1137
514970	20q	63561666	63565531	3866	12	0.667	Promoter(<=1kb)	-61	HELZ2	85441
514970	21q	44550835	44551416	582	6	0.833	Promoter(<=1kb)	89	KRTAP10-2	386679
514970	22q	22352950	22353298	349	13	0.538	Exon(exon1of2)	30478	BMS1P20	96610
514970	22q	36191154	36191906	753	6	0.667	3'UTR	9971	APOL4	80832
514970	22q	49883704	49886488	2785	8	0.25	Exon(exon2of2)	21430	ALG12	79087
514970	23p	8170039	8170141	103	6	0.5	Promoter(1-2kb)	1126	VCX2	51480
514970	23p	35802148	35803010	863	7	0.571	5'UTR	3357	MAGEB16	139604
514970	23q	136874183	136874347	165	7	0.714	Promoter(<=1kb)	-201	RBMX	27316
515125	1p	11766028	11768307	2280	6	0.833	Promoter(<=1kb)	0	C1orf167	284498
515125	1p	11778784	11779941	1158	7	0.714	Promoter(<=1kb)	0	C1orf167-AS1	102724659
515125	1p	16058491	16060000	1510	10	0.9	Exon(exon5of7)	6168	CLCNKB	1188

515125	1p	23874604	23875430	827	8	0.5	Exon(exon2of2)	-6310	FUCA1	2517
515125	1p	40067594	40067675	82	6	0	Promoter(<=1kb)	324	CAP1	10487
515125	1p	89186388	89186419	32	9	0.556	Promoter(<=1kb)	107	GBP4	115361
515125	1q	152213286	152213347	62	8	0.5	Exon(exon3of3)	10846	HRNR	388697
515125	1q	152219233	152221375	2143	16	0.75	Promoter(2-3kb)	2818	HRNR	388697
515125	1q	156669844	156670886	1043	6	1	Exon(exon4of4)	6521	NES	10763
515125	1q	158765805	158766655	851	6	0.5	Promoter(<=1kb)	47	OR6N1	128372
515125	1q	169542317	169542882	566	6	0.167	Exon(exon13of25)	-26572	F5	2153
515125	1q	214640144	214642954	2811	12	0.5	Exon(exon12of20)	-5013	CENPF	1063
515125	1q	214644872	214647291	2420	11	0.455	Promoter(<=1kb)	-676	CENPF	1063
515125	1q	236553659	236555893	2235	6	0.167	Exon(exon39of45)	10363	LGALS8	3964
515125	1q	247841312	247841582	271	6	0.833	Promoter(<=1kb)	314	OR11L1	391189
515125	1q	247895625	247896410	786	7	0.571	Promoter(<=1kb)	38	OR2W3	343171
515125	1q	247949436	247949759	324	7	0.429	Promoter(<=1kb)	578	OR2L8	391190
515125	2p	29002636	29003646	1011	6	0.333	Exon(exon5of20)	-10821	TOGARAM2	165186
515125	2p	48580657	48582454	1798	7	0.571	Promoter(<=1kb)	0	STON1	11037
515125	2q	102351547	102351902	356	7	0.429	Exon(exon11of11)	-4027	IL18R1	8809
515125	2q	130193975	130194181	207	6	0.5	Exon(exon4of5)	4258	TUBA3E	112714
515125	2q	130914528	130916712	2185	6	0.333	Promoter(<=1kb)	0	ARHGEF4	50649
515125	2q	132783032	132785012	1981	9	0.556	Promoter(1-2kb)	-1009	NCKAP5	344148
515125	2q	167246794	167248478	1685	7	0.714	Promoter(<=1kb)	-204	XIRP2	129446
515125	2q	178739433	178741811	2379	6	0.5	Exon(exon45of191)	26014	TTN	7273
515125	2q	184936178	184937636	1459	8	0.375	Exon(exon4of4)	69813	ZNF804A	91752
515125	2q	185790999	185794632	3634	8	0.875	Promoter(<=1kb)	0	FSIP2	401024
515125	2q	185805377	185808170	2794	6	0.333	Promoter(<=1kb)	0	FSIP2	401024
515125	2q	217847567	217848559	993	7	0.857	Exon(exon19of33)	-5407	TNS1	7145
515125	2q	238130416	238131546	1131	6	0.333	Promoter(1-2kb)	1468	ESPNL	339768
515125	2q	240041845	240042811	967	8	0.375	Downstream(2-3kb)	3261	OR6B3	150681
515125	3p	75736929	75738859	1931	10	0.6	Promoter(<=1kb)	0	MIR4273	100422955
515125	3q	98264413	98265137	725	8	0.625	Promoter(<=1kb)	128	OR5H6	79295
515125	3q	194341177	194342571	1395	6	0.667	Exon(exon2of2)	8747	CPN2	1370
515125	3q	196947878	196948579	702	6	0.5	3'UTR	3351	PIGZ	80235
515125	4p	5988612	5989749	1138	6	0.5	Promoter(<=1kb)	0	C4orf50	389197
515125	4p	6300792	6302360	1569	7	0.714	Exon(exon8of8)	6021	WFS1	7466
515125	4p	10443803	10446224	2422	6	0.333	Exon(exon3of3)	10952	ZNF518B	85460
515125	4q	121036404	121037542	1139	6	0.333	Promoter(1-2kb)	1442	NDNF	79625
515125	4q	186619481	186621601	2121	7	0.286	Exon(exon10of27)	-9411	FAT1	2195
515125	5q	83537326	83539905	2580	6	0.333	Promoter(1-2kb)	1712	VCAN	1462
515125	5q	140807352	140807737	386	6	0.833	Promoter(<=1kb)	271	PCDHA4	56144
515125	5q	140841480	140843056	1577	7	0.714	Promoter(<=1kb)	293	PCDHA8	56140
515125	5q	141174000	141175025	1026	6	0.833	Promoter(1-2kb)	1356	PCDHB7	56129
515125	5q	141183999	141184688	690	6	1	Promoter(2-3kb)	-2473	PCDHB9	56127
515125	5q	141955356	141957660	2305	6	0.667	Promoter(<=1kb)	-668	RNF14	9604
515125	5q	151565922	151568158	2237	9	0.778	Promoter(<=1kb)	786	FAT2	2196
515125	6p	42745312	42746041	730	6	0.667	Promoter(<=1kb)	62	TBCC	6903
515125	6p	46858771	46859502	732	7	0.571	Exon(exon17of21)	3802	ADGRF5	221395
515125	6q	149888581	149890867	2287	7	0.714	Promoter(<=1kb)	0	RAET1E-AS1	100652739
515125	7p	6330446	6330944	499	6	1	Exon(exon2of2)	7749	FAM220A	84792
515125	7q	100958977	100960873	1897	56	0.446	Promoter(1-2kb)	1012	MUC3A	4584
515125	7q	100991195	100992398	1204	7	0.571	Exon(exon5of15)	-20656	MUC12	10071
515125	7q	100995575	100995785	211	7	0.714	Exon(exon5of15)	-17269	MUC12	10071
515125	7q	101004421	101004836	416	6	0.667	Exon(exon5of15)	-8218	MUC12	10071
515125	7q	101034361	101040583	6223	40	0.525	Exon(exon3of12)	-3128	MUC17	140453
515125	7q	149818015	149819792	1778	6	0.667	Promoter(2-3kb)	-2352	SSPO	23145
515125	7q	149824094	149826495	2402	6	0.667	Promoter(<=1kb)	0	SSPO	23145
515125	8p	10607245	10608432	1188	8	0.5	Exon(exon4of4)	46711	RP1L1	94137
515125	8p	10609614	10610662	1049	7	0.571	Exon(exon4of4)	44481	RP1L1	94137
515125	8p	12132686	12133940	1255	7	0.714	Promoter(<=1kb)	498	USP17L7	392197
515125	8p	13021128	13022030	903	8	0.25	Exon(exon5of5)	9115	TRMT9B	57604
515125	8q	123651655	123652634	980	7	0.571	Promoter(<=1kb)	316	KLHL38	340359
515125	8q	141466429	141467514	1086	9	0.778	3'UTR	29245	MROH5	389690
515125	9p	34723747	34726473	2727	6	0.833	Exon(exon4of4)	3015	FAM205A	259308
515125	9p	39078723	39078753	31	6	0.833	Exon(exon22of24)	7395	CNTNAP3	79937
515125	9q	76175237	76175296	60	8	0.75	Exon(exon14of14)	-13343	PCSK5	5125
515125	9q	76703555	76706360	2806	8	0.5	Promoter(<=1kb)	0	PCA3	50652
515125	9q	87887543	87888819	1277	6	0.5	Exon(exon4of4)	4666	SPATA31E1	286234
515125	9q	104504315	104505071	757	6	0.5	Promoter(<=1kb)	52	OR13F1	138805
515125	9q	104598545	104599361	817	11	0.545	Promoter(<=1kb)	52	OR13C5	138799
515125	9q	122553263	122554071	809	7	0.429	Promoter(<=1kb)	93	OR1N2	138882
515125	9q	122628595	122629398	804	6	0.333	Promoter(<=1kb)	175	OR1B1	347169
515125	9q	122749914	122750547	634	6	0.833	Promoter(<=1kb)	174	OR1L6	392390
515125	9q	131474844	131476113	1270	7	0.714	Promoter(<=1kb)	0	PRRC2B	84726
515125	9q	133255635	133256205	571	7	1	3'UTR	19009	ABO	28
515125	9q	135484803	135487156	2354	8	0.25	Promoter(1-2kb)	1440	PPP1R26	9858
515125	10q	46549378	46550723	1346	26	0.654	Exon(exon3of3)	4807	GPRIN2	9721
515125	10q	122084988	122087840	2853	8	0.75	Exon(exon4of23)	-25190	TACC2	10579
515125	10q	128103129	128104830	1702	10	0.6	Promoter(<=1kb)	-1	MKI67	4288

515125	11p	244106	244197	92	8	0.5	Promoter(<=1kb)	-232	PSMD13	5719
515125	11p	1194354	1196902	2549	7	0.571	Exon(exon34of49)	-26164	MUC5B	727897
515125	11p	1246095	1247378	1284	8	0.375	Promoter(2-3kb)	2298	MUC5B-AS1	112577518
515125	11p	5177978	5178478	501	6	0.167	Promoter(<=1kb)	186	OR52Z1	283110
515125	11p	5323362	5324256	895	7	0.429	Promoter(<=1kb)	41	OR51B2	79345
515125	11p	5389704	5390350	647	7	0.429	Promoter(<=1kb)	327	OR51M1	390059
515125	11p	5402638	5403471	834	11	0.455	Promoter(<=1kb)	41	OR51J1	79470
515125	11p	5422212	5423123	912	10	0.7	Promoter(<=1kb)	101	OR51Q1	390061
515125	11p	5581045	5581738	694	9	0.333	Promoter(<=1kb)	168	OR52B6	340980
515125	11p	5884818	5885061	244	6	0.333	Promoter(<=1kb)	547	OR52E4	390081
515125	11p	11351961	11352736	776	9	0.222	Promoter(<=1kb)	514	CSNK2A3	283106
515125	11p	12293639	12294842	1204	7	0.714	Exon(exon29of35)	6739	MICALCL	84953
515125	11p	18173280	18173901	622	6	0.333	Promoter(<=1kb)	443	MRGPRX4	117196
515125	11p	34916266	34916763	498	6	0.667	Promoter(<=1kb)	0	APIP	51074
515125	11q	58214757	58215722	966	6	0.333	Promoter(<=1kb)	12	OR1S1	219959
515125	11q	64116513	64118232	1720	7	0.714	Exon(exon2of2)	8702	MACROD1	28992
515125	11q	82732630	82733184	555	6	0.833	Promoter(<=1kb)	680	FAM181B	220382
515125	11q	85724687	85725825	1139	6	0.5	Promoter(<=1kb)	0	SYTL2	54843
515125	11q	123906790	123907324	535	6	0.667	Promoter(<=1kb)	644	OR8D4	338662
515125	11q	123943088	123943782	695	7	0.571	Promoter(<=1kb)	91	OR6T1	219874
515125	11q	124015600	124016477	878	7	0.143	Promoter(<=1kb)	25	OR10G4	390264
515125	12p	4626571	4628549	1979	9	0.556	Exon(exon5of6)	14054	DYRK4	8798
515125	13q	24434450	24435347	898	7	0.571	Exon(exon31of34)	19787	PARP4	143
515125	13q	25096638	25097231	594	19	0.474	Promoter(<=1kb)	770	PABPC3	5042
515125	13q	102732474	102733933	1460	6	0.333	Exon(exon4of4)	25139	CCDC168	643677
515125	14q	19827604	19828161	558	7	0.857	Exon(exon2of2)	5669	OR4N2	390429
515125	14q	20640982	20641567	586	6	0.5	Promoter(<=1kb)	124	OR6S1	341799
515125	14q	70457520	70458540	1021	13	0.385	Exon(exon2of2)	5346	ADAM21	8747
515125	14q	77026548	77027532	985	6	0.5	Promoter(1-2kb)	1176	IRF2BPL	64207
515125	14q	94587512	94587839	328	6	0.5	Exon(exon2of2)	-4219	SERPINA3	12
515125	14q	104175275	104177810	2536	10	0.3	Exon(exon12of15)	36235	KIF26A	26153
515125	15q	23439979	23442067	2089	12	0.583	5'UTR	5167	GOLGA6L2	283685
515125	15q	40621642	40623696	2055	6	0.333	Promoter(<=1kb)	0	KNL1	57082
515125	15q	42690621	42692660	2040	7	0.571	Promoter(1-2kb)	1026	STARD9	57519
515125	15q	73702465	73703972	1508	8	0.875	Promoter(<=1kb)	0	CD276	80381
515125	15q	85579207	85581800	2594	14	0.643	Promoter(1-2kb)	-1110	AKAP13	11214
515125	15q	99129423	99132517	3095	6	0.333	Exon(exon4of5)	7225	TTC23	64927
515125	16p	1228744	1229622	879	7	0.714	Promoter(<=1kb)	540	TPSB2	64499
515125	16p	1486371	1488463	2093	8	0.75	Promoter(<=1kb)	4	PTX4	390667
515125	16p	28495551	28497395	1845	6	0.333	Promoter(<=1kb)	0	CLN3	1201
515125	16q	74391460	74392004	545	9	0.667	Exon(exon7of7)	13582	NPIPB15	440348
515125	16q	89100686	89101050	365	8	0.625	Promoter(<=1kb)	24	ACSF3	197322
515125	16q	89226863	89228289	1427	7	0.571	Promoter(2-3kb)	2229	ZNF778	197320
515125	17p	2299649	2300159	511	6	0.167	Exon(exon2of19)	-3224	SRR	63826
515125	17p	10638198	10641099	2902	6	0.333	Exon(exon19of41)	-8169	MYH3	4621
515125	17p	21300581	21300954	374	10	0.7	3'UTR	9112	MAP2K3	5606
515125	17q	53823368	53824891	1524	6	0.833	Promoter(<=1kb)	441	KIF2B	84643
515125	17q	76293419	76294016	598	7	0.571	Promoter(2-3kb)	-2167	QRICH2	84074
515125	17q	81645135	81645417	283	6	0.333	Promoter(2-3kb)	2722	TSPAN10	83882
515125	18q	58535186	58537515	2330	9	0.333	Promoter(<=1kb)	0	ALPK2	115701
515125	19p	4510548	4513547	3000	20	0.45	Exon(exon3of6)	4157	PLIN4	729359
515125	19p	5455600	5456439	840	7	0.571	Promoter(<=1kb)	183	ZNRF4	148066
515125	19p	8948231	8950136	1906	6	0.667	Exon(exon3of84)	31206	MUC16	94025
515125	19p	8959518	8962066	2549	6	0.333	Exon(exon3of84)	19276	MUC16	94025
515125	19p	8964274	8967127	2854	19	0.632	Exon(exon3of84)	14215	MUC16	94025
515125	19p	15087213	15088040	828	10	0.3	Promoter(<=1kb)	233	OR1I1	126370
515125	19p	21971930	21974500	2571	9	0.667	Exon(exon4of4)	14408	ZNF208	7757
515125	19p	22756294	22759533	3240	14	0.5	3'UTR	10449	ZNF99	7652
515125	19q	34943334	34944685	1352	6	0.333	3'UTR	9723	ZNF30	90075
515125	19q	36996730	36997597	868	10	0.6	Exon(exon10of10)	5677	ZNF568	374900
515125	19q	39877222	39877880	659	6	0.5	Exon(exon20of28)	9412	FCGBP	8857
515125	19q	43204061	43205504	1444	6	0.833	Promoter(<=1kb)	0	PSG4	5672
515125	19q	43258867	43258908	42	6	0.5	Promoter(1-2kb)	1838	LOC284344	284344
515125	19q	43913423	43914878	1456	8	0.5	Exon(exon10of10)	4861	ZNF45	7596
515125	19q	43966037	43967171	1135	6	0.167	Promoter(<=1kb)	-691	ZNF155	7711
515125	19q	44106512	44108078	1567	7	0	Exon(exon6of6)	-4103	ZNF225	7768
515125	19q	44327836	44329698	1863	6	0.5	Exon(exon4of4)	-22790	ZNF235	9310
515125	19q	48873325	48875925	2601	11	0.545	Promoter(<=1kb)	904	PPP1R15A	23645
515125	19q	51872787	51873699	913	10	0.5	Promoter(<=1kb)	-940	ZNF577	84765
515125	19q	52437918	52439242	1325	7	0.429	Exon(exon4of4)	6504	ZNF534	147658
515125	19q	55911888	55913077	1190	6	0.667	Exon(exon5of12)	19234	NLRP13	126204
515125	19q	57444351	57445460	1110	6	0.667	Exon(exon3of3)	9004	ZNF749	388567
515125	19q	57639918	57641641	1724	7	0.429	3'UTR	6693	ZNF211	10520
515125	19q	58368293	58368875	583	7	0.429	Exon(exon3of3)	-5445	ZNF497	162968
515125	20p	5922421	5923394	974	7	0.571	Exon(exon4of5)	6923	CHGB	1114
515125	20q	63561666	63564319	2654	8	0.75	Promoter(<=1kb)	-61	HELZ2	85441
515125	21q	44600627	44601692	1066	10	0.7	Promoter(<=1kb)	30	KRTAP10-7	386675

515125	22q	22352950	22353380	431	16	0.5	Exon(exon1of2)	30478	BMS1P20	96610
515125	22q	36191154	36191906	753	7	0.571	3'UTR	9971	APOL4	80832
515125	23p	8170039	8170141	103	6	0.5	Promoter(1-2kb)	1126	VCX2	51480
515125	23q	101494054	101494895	842	7	0.571	Exon(exon2of2)	6010	ARMCX4	100131755
515125	23q	136874183	136874347	165	7	0.714	Promoter(<=1kb)	-201	RBMX	27316
517353	1p	12029085	12030097	1013	6	0.833	Promoter(<=1kb)	0	MIR6729	102466982
517353	1p	12795518	12795813	296	8	0.5	Promoter(<=1kb)	827	PRAMEF1	65121
517353	1p	12847526	12847995	470	10	0.4	Promoter(<=1kb)	730	HNRNPCL1	343069
517353	1p	12859036	12860212	1177	15	0.533	Promoter(1-2kb)	1950	PRAMEF2	65122
517353	1p	12861255	12861478	224	7	0.143	Exon(exon4of4)	4169	PRAMEF2	65122
517353	1p	12893187	12893425	239	10	0.3	Exon(exon4of4)	4845	PRAMEF10	343071
517353	1p	13369166	13369564	399	7	0.714	Promoter(2-3kb)	2336	PRAMEF19	645414
517353	1p	13370686	13371119	434	7	0.429	Promoter(<=1kb)	781	PRAMEF19	645414
517353	1p	16058491	16060000	1510	10	0.9	Exon(exon5of7)	6168	CLCNKB	1188
517353	1p	18481403	18482217	815	6	0.667	Promoter(<=1kb)	421	KLHDC7A	127707
517353	1p	23874604	23875430	827	8	0.5	Exon(exon2of2)	-6310	FUCA1	2517
517353	1p	40067594	40067675	82	6	0	Promoter(<=1kb)	324	CAP1	10487
517353	1p	89186388	89186419	32	9	0.556	Promoter(<=1kb)	107	GBP4	115361
517353	1q	152213274	152213316	43	7	0.571	Exon(exon3of3)	10877	HRNR	388697
517353	1q	152218469	152221375	2907	17	0.706	Promoter(2-3kb)	2818	HRNR	388697
517353	1q	152303673	152313891	10219	39	0.59	Promoter(<=1kb)	0	FLG-AS1	339400
517353	1q	158765805	158766655	851	6	0.5	Promoter(<=1kb)	47	OR6N1	128372
517353	1q	247841312	247841582	271	6	0.833	Promoter(<=1kb)	314	OR11L1	391189
517353	1q	247896121	247896502	382	6	0.5	Promoter(<=1kb)	534	OR2W3	343171
517353	1q	247949325	247949738	414	10	0.3	Promoter(<=1kb)	467	OR2L8	391190
517353	1q	248273309	248273670	362	7	0.571	Promoter(<=1kb)	166	OR2T33	391195
517353	1q	248294677	248295458	782	7	0.571	Promoter(<=1kb)	142	OR2T12	127064
517353	1q	248681658	248682198	541	7	0.571	Promoter(<=1kb)	130	OR14I1	401994
517353	2q	102351547	102351902	356	7	0.429	Exon(exon11of11)	-4027	IL18R1	8809
517353	2q	178739433	178741811	2379	6	0.5	Exon(exon45of191)	26014	TTN	7273
517353	2q	184936178	184937636	1459	6	0.333	Exon(exon4of4)	69813	ZNF804A	91752
517353	2q	185789865	185794632	4768	10	0.8	Promoter(<=1kb)	0	FSIP2	401024
517353	3p	31989532	31990905	1374	7	0.286	Exon(exon2of2)	7761	ZNF860	344787
517353	3p	75736929	75738859	1931	11	0.545	Promoter(<=1kb)	0	MIR4273	100422955
517353	4p	5988383	5989749	1367	7	0.571	Promoter(<=1kb)	0	C4orf50	389197
517353	4p	6300792	6302360	1569	6	0.833	Exon(exon8of8)	6021	WFS1	7466
517353	4p	8227004	8228508	1505	8	0.125	Promoter(<=1kb)	-24	SH3TC1	54436
517353	4p	38774486	38775552	1067	8	0.5	Promoter(<=1kb)	644	TLR10	81793
517353	4q	112431241	112432293	1053	7	0.429	3'UTR	4735	ALPK1	80216
517353	4q	121036404	121037542	1139	6	0.333	Promoter(1-2kb)	1442	NDNF	79625
517353	4q	185458217	185460011	1795	9	0.556	Promoter(<=1kb)	0	CCDC110	256309
517353	4q	186706638	186709805	3168	8	0.625	Exon(exon2of27)	14028	FAT1	2195
517353	5q	140807352	140807737	386	6	0.833	Promoter(<=1kb)	271	PCDHA4	56144
517353	5q	140848579	140850786	2208	6	0.5	Promoter(<=1kb)	807	PCDHA9	9752
517353	5q	141174000	141175025	1026	6	0.833	Promoter(1-2kb)	1356	PCDHB7	56129
517353	5q	141183999	141184688	690	6	0.833	Promoter(2-3kb)	-2473	PCDHB9	56127
517353	5q	141187690	141189425	1736	6	0.5	Promoter(<=1kb)	529	PCDHB9	56127
517353	5q	141955356	141957660	2305	6	0.667	Promoter(<=1kb)	-668	RNF14	9604
517353	5q	151565922	151568158	2237	9	0.778	Promoter(<=1kb)	786	FAT2	2196
517353	5q	155014517	155016129	1613	6	0.833	Promoter(<=1kb)	762	KIF4B	285643
517353	6p	1312843	1313745	903	6	0.5	Promoter(<=1kb)	745	FOXQ1	94234
517353	6p	46858771	46859389	619	6	0.5	Exon(exon17of21)	3915	ADGRF5	221395
517353	6q	159231899	159234370	2472	10	0.5	Exon(exon11of23)	13602	FNDC1	84624
517353	7p	12369637	12370736	1100	6	0.667	3'UTR	-13307	VWDE	221806
517353	7p	53035678	53036385	708	7	1	Promoter(<=1kb)	45	POM121L12	285877
517353	7q	100958977	100960873	1897	47	0.489	Promoter(1-2kb)	1012	MUC3A	4584
517353	7q	100995547	100995785	239	6	0.833	Exon(exon5of15)	-17269	MUC12	10071
517353	7q	149818015	149819792	1778	6	0.667	Promoter(2-3kb)	-2352	SSPO	23145
517353	7q	149824094	149826495	2402	6	0.667	Promoter(<=1kb)	0	SSPO	23145
517353	8p	8376561	8377994	1434	6	1	Exon(exon2of5)	3753	PRAG1	157285
517353	8p	11331234	11332082	849	7	0.714	Promoter(<=1kb)	346	SLC35G5	83650
517353	8p	12132686	12133940	1255	6	0.667	Promoter(<=1kb)	498	USP17L7	392197
517353	8p	13021128	13022030	903	10	0.2	Exon(exon5of5)	9115	TRMT9B	57604
517353	8q	123651655	123652634	980	7	0.571	Promoter(<=1kb)	316	KLHL38	340359
517353	8q	143916360	143919209	2850	8	0.25	Exon(exon32of32)	20381	PLEC	5339
517353	9p	214679	215269	591	6	0.5	Promoter(<=1kb)	0	DOCK8	81704
517353	9p	34723747	34726527	2781	11	0.727	Promoter(2-3kb)	2961	FAM205A	259308
517353	9q	76175237	76175296	60	10	0.8	Exon(exon14of14)	-13343	PCSK5	5125
517353	9q	76705724	76707804	2081	6	0.5	Promoter(<=1kb)	666	PCA3	50652
517353	9q	104598545	104599361	817	12	0.583	Promoter(<=1kb)	52	OR13C5	138799
517353	9q	122553263	122554071	809	8	0.5	Promoter(<=1kb)	93	OR1N2	138882
517353	9q	122628595	122629398	804	6	0.333	Promoter(<=1kb)	175	OR1B1	347169
517353	9q	122749914	122750547	634	6	0.833	Promoter(<=1kb)	174	OR1L6	392390
517353	9q	133255635	133256205	571	7	1	3'UTR	19009	ABO	28
517353	9q	135484803	135487573	2771	9	0.333	Promoter(1-2kb)	1440	PPP1R26	9858
517353	10q	46549378	46550723	1346	25	0.64	Exon(exon3of3)	4807	GPRIN2	9721
517353	10q	49322575	49326817	4243	12	0.583	Exon(exon3of3)	23301	C10orf71	118461

517353	11p	244106	244197	92	8	0.5	Promoter(<=1kb)	-232	PSMD13	5719
517353	11p	1194354	1196902	2549	7	0.571	Exon(exon34of49)	-26164	MUC5B	727897
517353	11p	1246095	1247378	1284	8	0.375	Promoter(2-3kb)	2298	MUC5B-AS1	112577518
517353	11p	5177978	5178478	501	6	0.167	Promoter(<=1kb)	186	OR52Z1	283110
517353	11p	5323362	5324256	895	7	0.429	Promoter(<=1kb)	41	OR51B2	79345
517353	11p	5389704	5390350	647	7	0.429	Promoter(<=1kb)	327	OR51M1	390059
517353	11p	5402638	5403471	834	11	0.455	Promoter(<=1kb)	41	OR51J1	79470
517353	11p	5422212	5423123	912	10	0.7	Promoter(<=1kb)	101	OR51Q1	390061
517353	11p	5581045	5581738	694	8	0.375	Promoter(<=1kb)	168	OR52B6	340980
517353	11p	5841302	5841883	582	9	0.333	Promoter(<=1kb)	14	OR52E6	390078
517353	11p	11351961	11352736	776	7	0.143	Promoter(<=1kb)	514	CSNK2A3	283106
517353	11p	12293639	12294368	730	6	0.833	Exon(exon29of35)	6739	MICALCL	84953
517353	11p	18173280	18173901	622	6	0.333	Promoter(<=1kb)	443	MRGPRX4	117196
517353	11p	34916266	34916763	498	6	0.667	Promoter(<=1kb)	0	APIP	51074
517353	11q	55827536	55827640	105	6	0.667	Promoter(<=1kb)	317	OR5L2	26338
517353	11q	58214757	58215722	966	8	0.25	Promoter(<=1kb)	12	OR1S1	219959
517353	11q	66560202	66562261	2060	6	0.333	Exon(exon14of21)	3873	CTSF	8722
517353	11q	85724687	85725825	1139	6	0.5	Promoter(<=1kb)	0	SYTL2	54843
517353	11q	124015600	124016477	878	7	0.143	Promoter(<=1kb)	25	OR10G4	390264
517353	11q	124382526	124383285	760	9	0.667	Promoter(<=1kb)	58	OR8B2	26595
517353	12p	4626571	4628549	1979	9	0.556	Exon(exon5of6)	14054	DYRK4	8798
517353	12p	6453119	6453670	552	6	0.667	Promoter(<=1kb)	633	TAPBPL	55080
517353	12p	8222174	8223514	1341	6	0.667	Exon(exon5of6)	4073	FAM90A1	55138
517353	12q	48525773	48526223	451	6	0.333	Promoter(<=1kb)	141	OR8S1	341568
517353	12q	52316096	52317765	1670	6	0.667	Exon(exon4of9)	3633	KRT83	3889
517353	13q	24434450	24435347	898	7	0.571	Exon(exon31of34)	19787	PARP4	143
517353	13q	25096870	25097182	313	10	0.5	Promoter(1-2kb)	1002	PABPC3	5042
517353	13q	102732474	102733933	1460	7	0.286	Exon(exon4of4)	25139	CCDC168	643677
517353	14q	20060048	20060884	837	8	0.625	Promoter(<=1kb)	3	OR4L1	122742
517353	14q	20640982	20641567	586	6	0.5	Promoter(<=1kb)	124	OR6S1	341799
517353	14q	21634137	21634589	453	9	0.556	Promoter(<=1kb)	351	OR10G2	26534
517353	14q	22633879	22634450	572	9	0.333	Exon(exon2of2)	32212	ABHD4	63874
517353	14q	44504986	44506403	1418	6	0.667	Promoter(<=1kb)	880	FSCB	84075
517353	14q	70457532	70458540	1009	9	0.333	Exon(exon2of2)	5358	ADAM21	8747
517353	14q	94587512	94587839	328	6	0.5	Exon(exon2of2)	-4219	SERPINA3	12
517353	14q	104939262	104942618	3357	10	0.2	5'UTR	7102	PLD4	122618
517353	14q	104943622	104945444	1823	7	0.571	Exon(exon6of6)	9958	AHNAK2	113146
517353	14q	104946867	104953878	7012	50	0.6	Promoter(1-2kb)	1524	AHNAK2	113146
517353	15q	23439979	23442067	2089	10	0.6	5'UTR	5167	GOLGA6L2	283685
517353	15q	73702465	73703760	1296	7	0.857	Promoter(<=1kb)	-149	CD276	80381
517353	15q	85579423	85581800	2378	14	0.571	Promoter(1-2kb)	-1110	AKAP13	11214
517353	15q	99129423	99132797	3375	7	0.429	3'UTR	6945	TTC23	64927
517353	15q	100569472	100570097	626	6	0.833	Promoter(<=1kb)	534	LINS1	55180
517353	16p	1228744	1229622	879	7	0.714	Promoter(<=1kb)	540	TPSB2	64499
517353	16p	4206969	4208004	1036	7	0.571	Exon(exon2of7)	31739	SRL	6345
517353	16q	74391460	74392004	545	9	0.667	Exon(exon7of7)	13582	NPIPB15	440348
517353	16q	88428339	88429600	1262	6	0.333	Exon(exon3of3)	-23680	ZFPM1	161882
517353	16q	88430623	88431903	1281	6	0.5	Exon(exon3of3)	-21377	ZFPM1	161882
517353	16q	89100686	89101050	365	7	0.571	Promoter(<=1kb)	24	ACSF3	197322
517353	16q	89226863	89228289	1427	7	0.571	Promoter(2-3kb)	2229	ZNF778	197320
517353	17p	744946	746966	2021	7	1	3'UTR	5072	GEMIN4	50628
517353	17p	2299649	2300159	511	6	0.167	Exon(exon2of19)	-3224	SRR	63826
517353	17p	21300581	21300954	374	10	0.8	3'UTR	9112	MAP2K3	5606
517353	17p	21415470	21416404	935	6	1	Exon(exon3of3)	10334	KCNJ12	3768
517353	17q	81645135	81645607	473	7	0.286	Promoter(2-3kb)	2722	TSPAN10	83882
517353	18p	9886991	9888072	1082	7	0.857	Promoter(<=1kb)	977	TXNDC2	84203
517353	18p	11609646	11610491	846	14	0.714	Promoter(<=1kb)	50	SLC35G4	646000
517353	19p	4510548	4513547	3000	23	0.478	Exon(exon3of6)	4157	PLIN4	729359
517353	19p	5455600	5456439	840	7	0.571	Promoter(<=1kb)	183	ZNRF4	148066
517353	19p	8937644	8939234	1591	6	0.667	Exon(exon5of84)	-41554	MUC16	94025
517353	19p	8946313	8952171	5859	21	0.619	Exon(exon3of84)	29171	MUC16	94025
517353	19p	8959116	8962299	3184	12	0.75	Exon(exon3of84)	19043	MUC16	94025
517353	19p	8972751	8978096	5346	16	0.5	Exon(exon1of84)	3246	MUC16	94025
517353	19p	12075333	12077046	1714	6	0.167	Promoter(<=1kb)	6	ZNF788P	388507
517353	19p	12430718	12432437	1720	8	0.375	3'UTR	8584	ZNF443	10224
517353	19p	15087452	15087953	502	7	0.143	Promoter(<=1kb)	472	OR1I1	126370
517353	19p	18264798	18267409	2612	12	0.583	5'UTR	7002	IQCN	80726
517353	19p	21971930	21974500	2571	7	0.714	Exon(exon4of4)	14408	ZNF208	7757
517353	19q	36996730	36997597	868	10	0.7	Exon(exon10of10)	5677	ZNF568	374900
517353	19q	37151928	37153149	1222	6	0.833	Exon(exon5of5)	19287	ZNF585A	199704
517353	19q	39886005	39886422	418	6	0.667	Promoter(<=1kb)	870	FCGBP	8857
517353	19q	40875742	40878797	3056	8	0.125	Promoter(<=1kb)	0	CYP2A7	1549
517353	19q	40880231	40880622	392	6	0.333	Promoter(<=1kb)	-141	CYP2A7	1549
517353	19q	43846955	43848536	1582	6	0.833	3'UTR	13450	ZNF283	284349
517353	19q	43913423	43914878	1456	9	0.556	Exon(exon10of10)	4861	ZNF45	7596
517353	19q	52437918	52439242	1325	8	0.5	Exon(exon4of4)	6504	ZNF534	147658
517353	19q	52840798	52841923	1126	6	0.333	3'UTR	7602	ZNF468	90333

517353	20p	5922421	5923394	974	6	0.5	Exon(exon4of5)	6923	CHGB	1114
517353	20p	20052354	20052736	383	6	0	Promoter(<=1kb)	0	CFAP61	26074
517353	20q	63349752	63350772	1021	6	0.5	3'UTR	3794	CHRNA4	1137
517353	20q	63561666	63565531	3866	11	0.636	Promoter(<=1kb)	-61	HELZ2	85441
517353	21q	44550835	44551416	582	6	0.833	Promoter(<=1kb)	89	KRTAP10-2	386679
517353	21q	44600627	44601692	1066	14	0.571	Promoter(<=1kb)	30	KRTAP10-7	386675
517353	22q	22352950	22353380	431	16	0.5	Exon(exon1of2)	30478	BMS1P20	96610
517353	22q	36191154	36191906	753	7	0.714	3'UTR	9971	APOL4	80832
517353	22q	39100331	39102033	1703	6	0.833	Promoter(<=1kb)	52	APOBEC3H	164668
517353	23p	8170039	8170141	103	6	0.5	Promoter(1-2kb)	1126	VCX2	51480
517353	23p	35802148	35803010	863	7	0.571	5'UTR	3357	MAGEB16	139604
517514	1p	978953	979847	895	6	0.5	Promoter(1-2kb)	1182	PERM1	84808
517514	1p	12795695	12795957	263	6	0.5	Promoter(1-2kb)	1004	PRAMEF1	65121
517514	1p	12847526	12847780	255	7	0.429	Promoter(<=1kb)	945	HNRNPCL1	343069
517514	1p	12859036	12860212	1177	14	0.5	Promoter(1-2kb)	1950	PRAMEF2	65122
517514	1p	12861255	12861684	430	6	0.167	Exon(exon4of4)	4169	PRAMEF2	65122
517514	1p	13369166	13369564	399	8	0.875	Promoter(2-3kb)	2336	PRAMEF19	645414
517514	1p	13370686	13371119	434	8	0.375	Promoter(<=1kb)	781	PRAMEF19	645414
517514	1p	16058491	16060000	1510	10	0.9	Exon(exon5of7)	6168	CLCNKB	1188
517514	1p	18481403	18482217	815	6	0.667	Promoter(<=1kb)	421	KLHDC7A	127707
517514	1p	23874604	23875430	827	8	0.5	Exon(exon2of2)	-6310	FUCA1	2517
517514	1p	40067594	40067675	82	6	0	Promoter(<=1kb)	324	CAP1	10487
517514	1q	145872200	145873487	1288	6	0.667	Exon(exon8of12)	-12364	PIAS3	10401
517514	1q	152213274	152213320	47	8	0.5	Exon(exon3of3)	10873	HRNR	388697
517514	1q	152218469	152221375	2907	17	0.706	Promoter(2-3kb)	2818	HRNR	388697
517514	1q	152303673	152313891	10219	39	0.538	Promoter(<=1kb)	0	FLG-AS1	339400
517514	1q	156669844	156670886	1043	6	1	Exon(exon4of4)	6521	NES	10763
517514	1q	158765805	158766655	851	6	0.5	Promoter(<=1kb)	47	OR6N1	128372
517514	1q	197101312	197101771	460	6	0.5	Exon(exon18of28)	33373	ASPM	259266
517514	1q	228315976	228318049	2074	7	0.571	Exon(exon50of81)	6492	OBSCN	84033
517514	1q	247841312	247841582	271	6	0.833	Promoter(<=1kb)	314	OR11L1	391189
517514	1q	247949325	247949738	414	10	0.3	Promoter(<=1kb)	467	OR2L8	391190
517514	2p	26476258	26477885	1628	6	1	Promoter(<=1kb)	8	OTOF	9381
517514	2p	29002636	29003646	1011	6	0.333	Exon(exon5of20)	-10821	TOGARAM2	165186
517514	2p	48580657	48582454	1798	7	0.571	Promoter(<=1kb)	0	STON1	11037
517514	2q	130914528	130916712	2185	6	0.333	Promoter(<=1kb)	0	ARHGEF4	50649
517514	2q	132781993	132785001	3009	9	0.444	Promoter(<=1kb)	0	NCKAP5	344148
517514	2q	178739433	178741811	2379	6	0.5	Exon(exon45of191)	26014	TTN	7273
517514	2q	185790999	185794632	3634	8	0.875	Promoter(<=1kb)	0	FSIP2	401024
517514	2q	185799746	185802394	2649	6	0.5	Promoter(<=1kb)	0	FSIP2-AS1	107985781
517514	2q	185805377	185808170	2794	6	0.333	Promoter(<=1kb)	0	FSIP2	401024
517514	2q	233713134	233713783	650	13	0.692	Promoter(<=1kb)	142	UGT1A5	54579
517514	2q	240041845	240042131	287	6	0.167	Downstream(2-3kb)	3941	OR6B3	150681
517514	3p	75736880	75738859	1980	35	0.571	Promoter(<=1kb)	0	MIR4273	100422955
517514	3q	98264413	98265098	686	7	0.571	Promoter(<=1kb)	128	OR5H6	79295
517514	4p	1394795	1395368	574	7	0.429	Exon(exon1of1)	9983	UVSSA	57654
517514	4p	5988383	5989749	1367	8	0.625	Promoter(<=1kb)	0	C4orf50	389197
517514	4p	6300792	6302360	1569	6	0.833	Exon(exon8of8)	6021	WFS1	7466
517514	4p	7433331	7434512	1182	6	0.833	Promoter(<=1kb)	418	PSAPL1	768239
517514	4q	112431241	112432464	1224	8	0.5	3'UTR	4735	ALPK1	80216
517514	4q	168511522	168512293	772	6	0.833	Promoter(<=1kb)	0	PALLD	23022
517514	5q	79728956	79730716	1761	7	0.286	Exon(exon2of13)	-7426	CMYA5	202333
517514	5q	79731782	79734523	2742	13	0.308	Exon(exon2of13)	-3619	CMYA5	202333
517514	5q	83537326	83540746	3421	8	0.375	Promoter(1-2kb)	-1202	VCAN	1462
517514	5q	140802063	140803473	1411	6	0.5	Promoter(1-2kb)	1006	PCDHA3	56145
517514	5q	140848579	140850786	2208	7	0.571	Promoter(<=1kb)	807	PCDHA9	9752
517514	5q	141173510	141175025	1516	7	0.857	Promoter(<=1kb)	866	PCDHB7	56129
517514	5q	141183999	141184688	690	6	0.833	Promoter(2-3kb)	-2473	PCDHB9	56127
517514	5q	141187690	141189425	1736	7	0.571	Promoter(<=1kb)	529	PCDHB9	56127
517514	5q	141955676	141957660	1985	6	0.5	Promoter(<=1kb)	-668	RNF14	9604
517514	5q	148826877	148828070	1194	6	1	Promoter(1-2kb)	1632	ADRB2	154
517514	6p	26370344	26370479	136	6	0.5	Promoter(<=1kb)	0	BTN3A2	11118
517514	6p	46858771	46859502	732	8	0.5	Exon(exon17of21)	3802	ADGRF5	221395
517514	6q	159233455	159234370	916	10	0.5	Exon(exon11of23)	15158	FNDC1	84624
517514	7p	12369637	12370736	1100	6	0.667	3'UTR	-13307	VWDE	221806
517514	7p	45082865	45084866	2002	7	0.429	Promoter(1-2kb)	-1465	NACAD	23148
517514	7q	100958721	100960873	2153	61	0.443	Promoter(<=1kb)	756	MUC3A	4584
517514	7q	100991195	100993127	1933	9	0.667	Exon(exon5of15)	-19927	MUC12	10071
517514	7q	100995549	100995785	237	7	0.857	Exon(exon5of15)	-17269	MUC12	10071
517514	8p	10607245	10612307	5063	14	0.786	Exon(exon4of4)	42836	RP1L1	94137
517514	8p	12137448	12138641	1194	6	0.833	Promoter(<=1kb)	436	USP17L2	377630
517514	8p	13021128	13022030	903	8	0.25	Exon(exon5of5)	9115	TRMT9B	57604
517514	8q	143916360	143919209	2850	7	0.143	Exon(exon32of32)	20381	PLEC	5339
517514	9p	117371	118032	662	8	0.75	Promoter(<=1kb)	172	FOXD4	2298
517514	9p	39078723	39078846	124	6	0.667	Exon(exon22of24)	7302	CNTNAP3	79937
517514	9q	76175237	76175296	60	10	0.8	Exon(exon14of14)	-13343	PCSK5	5125
517514	9q	76705179	76707804	2626	8	0.5	Promoter(<=1kb)	121	PCA3	50652

517514	9q	76709523	76710843	1321	6	0.5	Promoter(<=1kb)	0	PRUNE2	158471
517514	9q	104504315	104505071	757	6	0.5	Promoter(<=1kb)	52	OR13F1	138805
517514	9q	104598545	104599361	817	12	0.583	Promoter(<=1kb)	52	OR13C5	138799
517514	9q	122553263	122554071	809	7	0.429	Promoter(<=1kb)	93	OR1N2	138882
517514	9q	122749914	122750547	634	6	0.833	Promoter(<=1kb)	174	OR1L6	392390
517514	9q	131474936	131475956	1021	6	0.667	Promoter(<=1kb)	0	PRRC2B	84726
517514	9q	133255635	133256205	571	7	1	3'UTR	19009	ABO	28
517514	9q	135484803	135487213	2411	8	0.25	Promoter(1-2kb)	1440	PPP1R26	9858
517514	9q	135547240	135548795	1556	9	0.556	Promoter(1-2kb)	1085	OBP2A	29991
517514	10p	47663	48605	943	6	0.833	Promoter(<=1kb)	664	TUBB8	347688
517514	10q	46549378	46550723	1346	26	0.654	Exon(exon3of3)	4807	GPRIN2	9721
517514	10q	49323504	49326817	3314	13	0.462	Exon(exon3of3)	24230	C10orf71	118461
517514	11p	244106	244197	92	8	0.5	Promoter(<=1kb)	-232	PSMD13	5719
517514	11p	1243401	1244556	1156	6	0.333	Exon(exon31of49)	5120	MUC5B-AS1	112577518
517514	11p	1246332	1248605	2274	10	0.5	Promoter(1-2kb)	1071	MUC5B-AS1	112577518
517514	11p	5046754	5047432	679	7	0.571	Promoter(<=1kb)	228	OR52J3	119679
517514	11p	5177811	5178478	668	7	0.143	Promoter(<=1kb)	186	OR52Z1	283110
517514	11p	5323451	5324256	806	6	0.5	Promoter(<=1kb)	41	OR51B2	79345
517514	11p	5389704	5390350	647	7	0.429	Promoter(<=1kb)	327	OR51M1	390059
517514	11p	5422212	5423123	912	10	0.7	Promoter(<=1kb)	101	OR51Q1	390061
517514	11p	5581208	5581738	531	6	0.333	Promoter(<=1kb)	331	OR52B6	340980
517514	11p	5841302	5841883	582	9	0.333	Promoter(<=1kb)	14	OR52E6	390078
517514	11p	5884818	5885061	244	7	0.429	Promoter(<=1kb)	547	OR52E4	390081
517514	11p	11351961	11352736	776	9	0.222	Promoter(<=1kb)	514	CSNK2A3	283106
517514	11p	12293639	12294368	730	6	0.833	Exon(exon29of35)	6739	MICALCL	84953
517514	11q	58214757	58215722	966	8	0.25	Promoter(<=1kb)	12	OR1S1	219959
517514	11q	58402523	58403265	743	8	0.5	Promoter(<=1kb)	144	OR5B3	441608
517514	11q	64116513	64118232	1720	6	0.667	Exon(exon2of2)	8702	MACROD1	28992
517514	11q	66560202	66562261	2060	6	0.333	Exon(exon14of21)	3873	CTSF	8722
517514	11q	85724687	85725825	1139	6	0.5	Promoter(<=1kb)	0	SYTL2	54843
517514	11q	123906790	123907324	535	6	0.667	Promoter(<=1kb)	644	OR8D4	338662
517514	11q	124038366	124038988	623	7	1	Promoter(<=1kb)	13	OR10G7	390265
517514	11q	130914501	130915409	909	10	0.7	Promoter(1-2kb)	1035	SNX19	399979
517514	12p	4626571	4628549	1979	9	0.444	Exon(exon5of6)	14054	DYRK4	8798
517514	12q	55247291	55248136	846	6	0.667	Promoter(<=1kb)	93	OR6C74	254783
517514	13q	25096675	25097182	508	14	0.571	Promoter(<=1kb)	807	PABPC3	5042
517514	13q	102732424	102733933	1510	7	0.429	Exon(exon4of4)	25139	CCDC168	643677
517514	13q	102741653	102745587	3935	10	0.5	Exon(exon1of2)	13485	CCDC168	643677
517514	14q	19975713	19976448	736	9	0.444	Promoter(<=1kb)	269	OR4K15	81127
517514	14q	20060048	20060884	837	8	0.625	Promoter(<=1kb)	3	OR4L1	122742
517514	14q	70457532	70458540	1009	9	0.333	Exon(exon2of2)	5358	ADAM21	8747
517514	14q	103110032	103110438	407	6	0.667	Promoter(2-3kb)	2410	EXOC3L4	91828
517514	14q	104939262	104945444	6183	20	0.4	5'UTR	7102	PLD4	122618
517514	14q	104946867	104953878	7012	37	0.514	Promoter(1-2kb)	1524	AHNAK2	113146
517514	15q	23439979	23442067	2089	12	0.583	5'UTR	5167	GOLGA6L2	283685
517514	15q	40621642	40624039	2398	7	0.286	Promoter(<=1kb)	0	KNL1	57082
517514	15q	73702465	73703760	1296	6	0.833	Promoter(<=1kb)	-149	CD276	80381
517514	15q	99129423	99132517	3095	6	0.333	Exon(exon4of5)	7225	TTC23	64927
517514	16p	1228744	1229731	988	10	0.7	Promoter(<=1kb)	431	TPSB2	64499
517514	16p	1256345	1256980	636	9	0.778	Promoter(<=1kb)	276	TPSD1	23430
517514	16p	1486371	1488463	2093	8	0.75	Promoter(<=1kb)	4	PTX4	390667
517514	16p	28495551	28497395	1845	6	0.333	Promoter(<=1kb)	0	CLN3	1201
517514	16q	74391650	74391928	279	7	0.714	Exon(exon7of7)	13772	NPIPB15	440348
517514	16q	88430623	88432069	1447	6	0.333	Exon(exon3of3)	-21211	ZFPM1	161882
517514	16q	89100686	89101050	365	7	0.571	Promoter(<=1kb)	24	ACSF3	197322
517514	16q	89226863	89228390	1528	10	0.6	Promoter(2-3kb)	2229	ZNF778	197320
517514	17p	21300581	21300954	374	8	0.75	3'UTR	9112	MAP2K3	5606
517514	17q	76293419	76294016	598	6	0.5	Promoter(2-3kb)	-2167	QRICH2	84074
517514	17q	81645135	81645417	283	6	0.333	Promoter(2-3kb)	2722	TSPAN10	83882
517514	18p	11609646	11610444	799	13	0.769	Promoter(<=1kb)	50	SLC35G4	646000
517514	18q	58535186	58537515	2330	9	0.333	Promoter(<=1kb)	0	ALPK2	115701
517514	19p	4511269	4513547	2279	9	0.333	Exon(exon3of6)	4157	PLIN4	729359
517514	19p	5455600	5456439	840	8	0.625	Promoter(<=1kb)	183	ZNRF4	148066
517514	19p	8937644	8939234	1591	6	0.833	Exon(exon5of84)	-41554	MUC16	94025
517514	19p	8945654	8951868	6215	19	0.632	Exon(exon3of84)	29474	MUC16	94025
517514	19p	8959116	8962299	3184	12	0.583	Exon(exon3of84)	19043	MUC16	94025
517514	19p	8963397	8967127	3731	21	0.667	Exon(exon3of84)	14215	MUC16	94025
517514	19p	8972448	8978096	5649	16	0.5	Exon(exon1of84)	3246	MUC16	94025
517514	19p	12430157	12432437	2281	9	0.333	3'UTR	8584	ZNF443	10224
517514	19p	14841061	14841331	271	6	0.5	Promoter(<=1kb)	546	OR7A10	390892
517514	19p	14880627	14881233	607	6	0.5	Promoter(<=1kb)	219	OR7A17	26333
517514	19p	17282085	17284246	2162	8	0.5	Promoter(<=1kb)	0	ANKLE1	126549
517514	19p	23743906	23745300	1395	6	0.333	Exon(exon4of4)	13537	ZNF681	148213
517514	19q	39886240	39886422	183	6	0.667	Promoter(<=1kb)	870	FCGBP	8857
517514	19q	43846955	43848536	1582	6	0.833	3'UTR	13450	ZNF283	284349
517514	19q	43913423	43914878	1456	8	0.5	Exon(exon10of10)	4861	ZNF45	7596
517514	19q	43966037	43967171	1135	6	0.167	Promoter(<=1kb)	-691	ZNF155	7711

517514	19q	44106512	44108078	1567	7	0	Exon(exon6of6)	-4103	ZNF225	7768
517514	19q	52437918	52439242	1325	7	0.429	Exon(exon4of4)	6504	ZNF534	147658
517514	19q	55912302	55913166	865	6	0.667	Exon(exon5of12)	19145	NLRP13	126204
517514	19q	58368293	58368875	583	7	0.429	Exon(exon3of3)	-5445	ZNF497	162968
517514	20p	5922577	5923382	806	7	0.571	Exon(exon4of5)	7079	CHGB	1114
517514	20q	63349752	63350772	1021	6	0.5	3'UTR	3794	CHRNA4	1137
517514	20q	63561666	63565531	3866	11	0.636	Promoter(<=1kb)	-61	HELZ2	85441
517514	21q	44591587	44592404	818	6	0.333	Promoter(<=1kb)	101	KRTAP10-6	386674
517514	21q	44600627	44601692	1066	10	0.7	Promoter(<=1kb)	30	KRTAP10-7	386675
517514	22q	22352950	22353380	431	16	0.5	Exon(exon1of2)	30478	BMS1P20	96610
517514	22q	36191154	36191906	753	7	0.714	3'UTR	9971	APOL4	80832
517514	23p	3320126	3323750	3625	10	0.6	Exon(exon5of7)	22902	MXRA5	25878
517514	23p	8170039	8170141	103	6	0.5	Promoter(1-2kb)	1126	VCX2	51480
517514	23p	35802148	35803010	863	7	0.571	5'UTR	3357	MAGEB16	139604
519684	1p	11766028	11768307	2280	6	0.833	Promoter(<=1kb)	0	C1orf167	284498
519684	1p	16058491	16060000	1510	10	0.9	Exon(exon5of7)	6168	CLCNKB	1188
519684	1p	18481042	18482217	1176	12	0.75	Promoter(<=1kb)	60	KLHDC7A	127707
519684	1p	23874604	23875430	827	9	0.556	Exon(exon2of2)	-6310	FUCA1	2517
519684	1p	40067594	40067675	82	6	0	Promoter(<=1kb)	324	CAP1	10487
519684	1p	89186388	89186419	32	9	0.556	Promoter(<=1kb)	107	GBP4	115361
519684	1q	152303673	152313891	10219	39	0.59	Promoter(<=1kb)	0	FLG-AS1	339400
519684	1q	197100802	197101771	970	6	0.5	Exon(exon18of28)	33373	ASPM	259266
519684	1q	201206099	201209837	3739	11	0.545	Promoter(1-2kb)	1017	IGFN1	91156
519684	1q	223393517	223394466	950	6	0.667	Promoter(<=1kb)	102	CCDC185	164127
519684	1q	228315976	228318026	2051	8	0.625	Exon(exon50of81)	6492	OBSCN	84033
519684	1q	247841312	247841582	271	6	0.833	Promoter(<=1kb)	314	OR11L1	391189
519684	1q	247949325	247949738	414	10	0.3	Promoter(<=1kb)	467	OR2L8	391190
519684	1q	248272863	248273670	808	6	0.333	Promoter(<=1kb)	166	OR2T33	391195
519684	1q	248294677	248295458	782	7	0.571	Promoter(<=1kb)	142	OR2T12	127064
519684	2p	48580657	48582454	1798	7	0.571	Promoter(<=1kb)	0	STON1	11037
519684	2q	132783061	132785001	1941	9	0.444	Promoter(1-2kb)	-1038	NCKAP5	344148
519684	2q	185789591	185794632	5042	12	0.667	Promoter(<=1kb)	0	FSIP2	401024
519684	2q	217847583	217848746	1164	7	0.857	Exon(exon19of33)	-5423	TNS1	7145
519684	2q	237762685	237764060	1376	10	0.5	Exon(exon8of8)	-4137	LRRFIP1	9208
519684	3p	13570520	13571564	1045	6	0.167	Promoter(1-2kb)	1780	FBLN2	2199
519684	3p	31989532	31990905	1374	8	0.375	Exon(exon2of2)	7761	ZNF860	344787
519684	3p	75736880	75739243	2364	48	0.604	Promoter(<=1kb)	0	MIR4273	100422955
519684	3q	98264413	98265098	686	7	0.571	Promoter(<=1kb)	128	OR5H6	79295
519684	4p	5988383	5989749	1367	7	0.571	Promoter(<=1kb)	0	C4orf50	389197
519684	4p	6300792	6302360	1569	6	0.833	Exon(exon8of8)	6021	WFS1	7466
519684	4p	8227004	8228508	1505	8	0.25	Promoter(<=1kb)	-24	SH3TC1	54436
519684	4q	121036404	121037542	1139	6	0.333	Promoter(1-2kb)	1442	NDNF	79625
519684	4q	185458217	185460011	1795	9	0.556	Promoter(<=1kb)	0	CCDC110	256309
519684	5q	79731782	79734523	2742	13	0.308	Exon(exon2of13)	-3619	CMYA5	202333
519684	5q	79735549	79737483	1935	6	0.833	Promoter(<=1kb)	-659	CMYA5	202333
519684	5q	140807352	140807737	386	6	0.833	Promoter(<=1kb)	271	PCDHA4	56144
519684	5q	141174000	141175025	1026	6	0.833	Promoter(1-2kb)	1356	PCDHB7	56129
519684	5q	141183999	141184688	690	8	1	Promoter(2-3kb)	-2473	PCDHB9	56127
519684	5q	148826877	148828070	1194	6	1	Promoter(1-2kb)	1632	ADRB2	154
519684	5q	151521550	151522069	520	6	0.667	Promoter(<=1kb)	79	MIR6499	102465246
519684	5q	151565922	151568158	2237	9	0.778	Promoter(<=1kb)	786	FAT2	2196
519684	6p	46858771	46859502	732	8	0.5	Exon(exon17of21)	3802	ADGRF5	221395
519684	6q	159231899	159234370	2472	13	0.615	Exon(exon11of23)	13602	FNDC1	84624
519684	7p	12369637	12370736	1100	6	0.667	3'UTR	-13307	VWDE	221806
519684	7p	38353718	38353991	274	8	0.625	Exon(exon2of2)	3699	TRG-AS1	100506776
519684	7q	100958977	100960873	1897	54	0.463	Promoter(1-2kb)	1012	MUC3A	4584
519684	7q	100991195	100992398	1204	7	0.571	Exon(exon5of15)	-20656	MUC12	10071
519684	7q	100995575	100995922	348	8	0.625	Exon(exon5of15)	-17132	MUC12	10071
519684	7q	101034361	101040583	6223	40	0.5	Exon(exon3of12)	-3128	MUC17	140453
519684	7q	149818015	149819792	1778	6	0.667	Promoter(2-3kb)	-2352	SSPO	23145
519684	7q	149823389	149826495	3107	7	0.714	Promoter(<=1kb)	0	SSPO	23145
519684	8p	10607375	10610662	3288	11	0.545	Exon(exon4of4)	44481	RP1L1	94137
519684	8p	13021128	13022030	903	8	0.125	Exon(exon5of5)	9115	TRMT9B	57604
519684	8q	123651655	123652634	980	7	0.571	Promoter(<=1kb)	316	KLHL38	340359
519684	9p	116800	118032	1233	9	0.889	Promoter(<=1kb)	172	FOXD4	2298
519684	9q	76175237	76175301	65	11	0.727	Exon(exon14of14)	-13338	PCSK5	5125
519684	9q	76705724	76707804	2081	6	0.5	Promoter(<=1kb)	666	PCA3	50652
519684	9q	104598641	104599361	721	8	0.625	Promoter(<=1kb)	52	OR13C5	138799
519684	9q	122553263	122554071	809	8	0.5	Promoter(<=1kb)	93	OR1N2	138882
519684	9q	122628595	122629130	536	6	0.333	Promoter(<=1kb)	443	OR1B1	347169
519684	9q	122749914	122750547	634	6	0.833	Promoter(<=1kb)	174	OR1L6	392390
519684	9q	131474844	131476113	1270	6	0.833	Promoter(<=1kb)	0	PRRC2B	84726
519684	9q	135484803	135487213	2411	8	0.25	Promoter(1-2kb)	1440	PPP1R26	9858
519684	10q	46549378	46550723	1346	25	0.64	Exon(exon3of3)	4807	GPRIN2	9721
519684	10q	89737450	89738561	1112	6	0	Exon(exon20of33)	13874	KIF20B	9585
519684	10q	128103129	128108204	5076	15	0.667	Promoter(<=1kb)	-1	MKI67	4288
519684	11p	244106	244197	92	8	0.5	Promoter(<=1kb)	-232	PSMD13	5719

519684	11p	1246095	1248605	2511	12	0.5	Promoter(1-2kb)	1071	MUC5B-AS1	112577518
519684	11p	1249711	1251628	1918	7	0.857	Promoter(<=1kb)	-35	MUC5B-AS1	112577518
519684	11p	5177811	5178478	668	7	0.143	Promoter(<=1kb)	186	OR52Z1	283110
519684	11p	5323542	5324256	715	6	0.5	Promoter(<=1kb)	41	OR51B2	79345
519684	11p	5389704	5390350	647	6	0.5	Promoter(<=1kb)	327	OR51M1	390059
519684	11p	5422212	5423123	912	10	0.7	Promoter(<=1kb)	101	OR51Q1	390061
519684	11p	5581045	5581738	694	8	0.375	Promoter(<=1kb)	168	OR52B6	340980
519684	11p	5884818	5885061	244	6	0.333	Promoter(<=1kb)	547	OR52E4	390081
519684	11p	11351961	11352736	776	9	0.222	Promoter(<=1kb)	514	CSNK2A3	283106
519684	11p	12293639	12294842	1204	7	0.571	Exon(exon29of35)	6739	MICALCL	84953
519684	11q	56105181	56105892	712	6	0.5	Promoter(<=1kb)	163	OR8H2	390151
519684	11q	58214757	58215722	966	8	0.25	Promoter(<=1kb)	12	OR1S1	219959
519684	11q	58402523	58403265	743	8	0.5	Promoter(<=1kb)	144	OR5B3	441608
519684	11q	64315818	64315856	39	6	0.833	Promoter(1-2kb)	1485	TRMT112	51504
519684	11q	82732630	82733184	555	6	0.833	Promoter(<=1kb)	680	FAM181B	220382
519684	11q	85724687	85725825	1139	6	0.5	Promoter(<=1kb)	0	SYTL2	54843
519684	11q	124015601	124016477	877	10	0.3	Promoter(<=1kb)	26	OR10G4	390264
519684	11q	124023038	124023849	812	9	0.556	Promoter(<=1kb)	25	OR10G9	219870
519684	11q	124382526	124383285	760	6	0.667	Promoter(<=1kb)	58	OR8B2	26595
519684	11q	130914501	130915409	909	10	0.7	Promoter(1-2kb)	1035	SNX19	399979
519684	12p	4626571	4628549	1979	8	0.5	Exon(exon5of6)	14054	DYRK4	8798
519684	12p	6453119	6453670	552	6	0.667	Promoter(<=1kb)	633	TAPBPL	55080
519684	12q	52571389	52573652	2264	7	0.571	Promoter(<=1kb)	164	KRT74	121391
519684	13q	25096713	25097154	442	27	0.519	Promoter(<=1kb)	845	PABPC3	5042
519684	13q	102732474	102733933	1460	6	0.333	Exon(exon4of4)	25139	CCDC168	643677
519684	14q	20060048	20060884	837	10	0.6	Promoter(<=1kb)	3	OR4L1	122742
519684	14q	20640750	20641567	818	7	0.571	Promoter(<=1kb)	124	OR6S1	341799
519684	14q	21634137	21634589	453	9	0.556	Promoter(<=1kb)	351	OR10G2	26534
519684	14q	22633879	22634450	572	8	0.375	Exon(exon2of2)	32212	ABHD4	63874
519684	14q	70457520	70458948	1429	13	0.385	Exon(exon2of2)	5346	ADAM21	8747
519684	15q	23439979	23442067	2089	12	0.583	5'UTR	5167	GOLGA6L2	283685
519684	15q	73702465	73703760	1296	7	0.857	Promoter(<=1kb)	-149	CD276	80381
519684	15q	85579423	85582073	2651	15	0.6	Promoter(<=1kb)	-837	AKAP13	11214
519684	15q	88857108	88859365	2258	6	0	Exon(exon12of18)	9865	ACAN	176
519684	15q	99129423	99132517	3095	6	0.333	Exon(exon4of5)	7225	TTC23	64927
519684	16p	1256345	1256985	641	10	0.6	Promoter(<=1kb)	276	TPSD1	23430
519684	16p	1998795	2001059	2265	8	0.625	Promoter(<=1kb)	0	ZNF598	90850
519684	16p	4883938	4885635	1698	6	1	Exon(exon22of22)	4701	PPL	5493
519684	16q	74391650	74392004	355	8	0.625	Exon(exon7of7)	13772	NPIPB15	440348
519684	16q	89100686	89101050	365	8	0.625	Promoter(<=1kb)	24	ACSF3	197322
519684	16q	89226863	89228419	1557	9	0.556	Promoter(2-3kb)	2229	ZNF778	197320
519684	17p	7846914	7848460	1547	6	0.667	Promoter(1-2kb)	1999	KDM6B	23135
519684	17p	10638198	10641099	2902	7	0.286	Exon(exon19of41)	-8169	MYH3	4621
519684	17p	21300581	21300954	374	8	0.75	3'UTR	9112	MAP2K3	5606
519684	17p	21415470	21416416	947	10	0.9	Exon(exon3of3)	10334	KCNJ12	3768
519684	17q	53823368	53824891	1524	6	0.833	Promoter(<=1kb)	441	KIF2B	84643
519684	17q	81645135	81645607	473	7	0.286	Promoter(2-3kb)	2722	TSPAN10	83882
519684	18p	11609646	11610164	519	9	0.778	Promoter(<=1kb)	50	SLC35G4	646000
519684	18q	58535186	58538030	2845	18	0.556	Promoter(<=1kb)	0	ALPK2	115701
519684	18q	75285722	75287404	1683	6	0.333	Exon(exon2of2)	41146	TSHZ1	10194
519684	19p	4510548	4513547	3000	23	0.522	Exon(exon3of6)	4157	PLIN4	729359
519684	19p	5455600	5456439	840	7	0.571	Promoter(<=1kb)	183	ZNRF4	148066
519684	19p	8946732	8952171	5440	15	0.467	Exon(exon3of84)	29171	MUC16	94025
519684	19p	8959403	8961248	1846	6	0.667	Exon(exon3of84)	20094	MUC16	94025
519684	19p	12430157	12432437	2281	10	0.4	3'UTR	8584	ZNF443	10224
519684	19p	15087213	15088040	828	9	0.333	Promoter(<=1kb)	233	OR1I1	126370
519684	19p	15794192	15794719	528	6	0.333	Promoter(<=1kb)	241	OR10H5	284433
519684	19p	18264798	18267409	2612	12	0.583	5'UTR	7002	IQCN	80726
519684	19p	21971930	21974500	2571	7	0.714	Exon(exon4of4)	14408	ZNF208	7757
519684	19p	23743906	23745300	1395	6	0.333	Exon(exon4of4)	13537	ZNF681	148213
519684	19q	39877222	39877880	659	6	0.5	Exon(exon20of28)	9412	FCGBP	8857
519684	19q	39886240	39886422	183	6	0.667	Promoter(<=1kb)	870	FCGBP	8857
519684	19q	40880128	40880622	495	7	0.286	Promoter(<=1kb)	-38	CYP2A7	1549
519684	19q	43846955	43848536	1582	6	0.833	3'UTR	13450	ZNF283	284349
519684	19q	43913423	43914878	1456	8	0.5	Exon(exon10of10)	4861	ZNF45	7596
519684	19q	44106512	44108078	1567	7	0	Exon(exon6of6)	-4103	ZNF225	7768
519684	19q	51745958	51746963	1006	6	0.333	Exon(exon3of3)	3848	FPR1	2357
519684	19q	52365769	52366744	976	6	0.833	Exon(exon6of6)	-3173	ZNF880	400713
519684	19q	52384029	52384992	964	6	0	Exon(exon4of4)	12677	ZNF528-AS1	102724105
519684	19q	52437918	52439242	1325	7	0.429	Exon(exon4of4)	6504	ZNF534	147658
519684	19q	55911888	55913077	1190	6	0.667	Exon(exon5of12)	19234	NLRP13	126204
519684	19q	58368293	58368875	583	7	0.429	Exon(exon3of3)	-5445	ZNF497	162968
519684	20p	5922421	5923643	1223	9	0.667	Exon(exon4of5)	6923	CHGB	1114
519684	20q	63349752	63350772	1021	6	0.5	3'UTR	3794	CHRNA4	1137
519684	20q	63561666	63565980	4315	12	0.583	Promoter(<=1kb)	-61	HELZ2	85441
519684	21q	44600627	44601692	1066	10	0.7	Promoter(<=1kb)	30	KRTAP10-7	386675
519684	22q	22352950	22353380	431	16	0.5	Exon(exon1of2)	30478	BMS1P20	96610

519684	22q	36191154	36191906	753	7	0.571	3'UTR	9971	APOL4	80832
519684	22q	39100331	39102033	1703	6	0.833	Promoter(<=1kb)	52	APOBEC3H	164668
519684	22q	49884187	49884994	808	6	0.333	Exon(exon2of2)	22924	ALG12	79087
519684	23p	3320126	3323750	3625	10	0.5	Exon(exon5of7)	22902	MXRA5	25878
519684	23p	8170039	8170141	103	6	0.5	Promoter(1-2kb)	1126	VCX2	51480
519684	23p	35802148	35803010	863	7	0.571	5'UTR	3357	MAGEB16	139604
519684	23q	136874183	136874416	234	8	0.75	Promoter(<=1kb)	-201	RBMX	27316
527571	1p	12847526	12847995	470	11	0.455	Promoter(<=1kb)	730	HNRNPCL1	343069
527571	1p	12859036	12860079	1044	6	0.333	Promoter(1-2kb)	1950	PRAMEF2	65122
527571	1p	13321724	13322134	411	6	0.167	Exon(exon4of4)	6143	PRAMEF15	653619
527571	1p	13370686	13371119	434	9	0.444	Promoter(<=1kb)	781	PRAMEF19	645414
527571	1p	13391965	13392142	178	6	0.333	Promoter(2-3kb)	2333	PRAMEF17	391004
527571	1p	16058491	16060000	1510	13	0.846	Exon(exon5of7)	6168	CLCNKB	1188
527571	1p	18481403	18482217	815	6	0.667	Promoter(<=1kb)	421	KLHDC7A	127707
527571	1p	23874604	23875430	827	9	0.556	Exon(exon2of2)	-6310	FUCA1	2517
527571	1p	40067594	40067675	82	6	0	Promoter(<=1kb)	324	CAP1	10487
527571	1p	89186388	89186419	32	9	0.556	Promoter(<=1kb)	107	GBP4	115361
527571	1q	152213274	152213316	43	7	0.571	Exon(exon3of3)	10877	HRNR	388697
527571	1q	152218469	152221375	2907	16	0.688	Promoter(2-3kb)	2818	HRNR	388697
527571	1q	152303673	152314098	10426	38	0.632	Promoter(<=1kb)	0	FLG-AS1	339400
527571	1q	158765805	158766655	851	6	0.5	Promoter(<=1kb)	47	OR6N1	128372
527571	1q	169542317	169542882	566	6	0.167	Exon(exon13of25)	-26572	F5	2153
527571	1q	223393517	223394466	950	6	0.667	Promoter(<=1kb)	102	CCDC185	164127
527571	1q	228315976	228318026	2051	6	0.5	Exon(exon50of81)	6492	OBSCN	84033
527571	1q	236553659	236555893	2235	6	0.333	Exon(exon39of45)	10363	LGALS8	3964
527571	1q	247841312	247841582	271	6	0.833	Promoter(<=1kb)	314	OR11L1	391189
527571	1q	247949325	247949738	414	10	0.3	Promoter(<=1kb)	467	OR2L8	391190
527571	1q	248294677	248295458	782	7	0.571	Promoter(<=1kb)	142	OR2T12	127064
527571	2q	130193975	130194181	207	6	0.667	Exon(exon4of5)	4258	TUBA3E	112714
527571	2q	184936178	184937636	1459	7	0.429	Exon(exon4of4)	69813	ZNF804A	91752
527571	2q	185789865	185794632	4768	10	0.8	Promoter(<=1kb)	0	FSIP2	401024
527571	2q	233681970	233682693	724	6	0.667	Promoter(<=1kb)	0	UGT1A7	54577
527571	2q	233713134	233713783	650	12	0.667	Promoter(<=1kb)	142	UGT1A5	54579
527571	2q	233840612	233842185	1574	6	0.667	Promoter(<=1kb)	0	HJURP	55355
527571	2q	238130271	238131702	1432	9	0.556	Promoter(1-2kb)	1323	ESPNL	339768
527571	2q	240041845	240042154	310	6	0.167	Downstream(2-3kb)	3918	OR6B3	150681
527571	3p	75736929	75738859	1931	10	0.6	Promoter(<=1kb)	0	MIR4273	100422955
527571	3q	98264413	98265098	686	7	0.571	Promoter(<=1kb)	128	OR5H6	79295
527571	4p	5988383	5989749	1367	7	0.571	Promoter(<=1kb)	0	C4orf50	389197
527571	4p	6300792	6302360	1569	6	0.833	Exon(exon8of8)	6021	WFS1	7466
527571	4q	121036404	121037542	1139	6	0.333	Promoter(1-2kb)	1442	NDNF	79625
527571	4q	185458217	185460011	1795	9	0.556	Promoter(<=1kb)	0	CCDC110	256309
527571	4q	186706638	186708616	1979	6	0.667	Exon(exon2of27)	15217	FAT1	2195
527571	5p	795818	796237	420	8	0.5	3'UTR	4908	ZDHHC11	79844
527571	5q	79728956	79730716	1761	7	0.286	Exon(exon2of13)	-7426	CMYA5	202333
527571	5q	79731782	79734523	2742	13	0.308	Exon(exon2of13)	-3619	CMYA5	202333
527571	5q	83537326	83539905	2580	6	0.333	Promoter(1-2kb)	1712	VCAN	1462
527571	5q	140807352	140807737	386	7	0.857	Promoter(<=1kb)	271	PCDHA4	56144
527571	5q	140848579	140850786	2208	6	0.5	Promoter(<=1kb)	807	PCDHA9	9752
527571	5q	141174000	141175025	1026	6	0.833	Promoter(1-2kb)	1356	PCDHB7	56129
527571	5q	141183999	141184688	690	7	0.714	Promoter(2-3kb)	-2473	PCDHB9	56127
527571	5q	141187690	141189425	1736	6	0.5	Promoter(<=1kb)	529	PCDHB9	56127
527571	5q	148826877	148828070	1194	6	1	Promoter(1-2kb)	1632	ADRB2	154
527571	5q	151565922	151568158	2237	9	0.778	Promoter(<=1kb)	786	FAT2	2196
527571	6p	46858771	46859502	732	8	0.5	Exon(exon17of21)	3802	ADGRF5	221395
527571	6p	56605300	56607197	1898	6	0.667	Exon(exon36of98)	-6787	DST	667
527571	6q	149888581	149889987	1407	6	0.667	Promoter(<=1kb)	-116	RAET1E-AS1	100652739
527571	6q	159231899	159234370	2472	12	0.583	Exon(exon11of23)	13602	FNDC1	84624
527571	7p	12369637	12370736	1100	6	0.667	3'UTR	-13307	VWDE	221806
527571	7p	45082865	45084866	2002	6	0.333	Promoter(1-2kb)	-1465	NACAD	23148
527571	7p	56021087	56021209	123	6	0.5	Exon(exon2of7)	12947	PSPH	5723
527571	7q	100958721	100960873	2153	57	0.474	Promoter(<=1kb)	756	MUC3A	4584
527571	7q	100991195	100993127	1933	8	0.625	Exon(exon5of15)	-19927	MUC12	10071
527571	7q	100995575	100995785	211	6	0.833	Exon(exon5of15)	-17269	MUC12	10071
527571	7q	101034221	101038994	4774	34	0.412	Exon(exon3of12)	-4717	MUC17	140453
527571	8p	8376561	8377994	1434	6	1	Exon(exon2of5)	3753	PRAG1	157285
527571	8p	10607245	10608432	1188	8	0.5	Exon(exon4of4)	46711	RP1L1	94137
527571	8p	10609614	10612607	2994	10	0.9	Exon(exon4of4)	42536	RP1L1	94137
527571	8p	12132686	12133940	1255	7	0.714	Promoter(<=1kb)	498	USP17L7	392197
527571	8p	13021128	13022030	903	7	0.143	Exon(exon5of5)	9115	TRMT9B	57604
527571	8q	123651802	123652634	833	6	0.5	Promoter(<=1kb)	316	KLHL38	340359
527571	8q	143916360	143920173	3814	8	0.125	Exon(exon32of32)	19417	PLEC	5339
527571	8q	143923488	143925516	2029	10	0.7	Exon(exon31of32)	14074	PLEC	5339
527571	9q	76175237	76175296	60	10	0.8	Exon(exon14of14)	-13343	PCSK5	5125
527571	9q	76703555	76707804	4250	13	0.462	Promoter(<=1kb)	0	PCA3	50652
527571	9q	76709263	76710843	1581	8	0.5	Promoter(<=1kb)	0	PRUNE2	158471
527571	9q	87885490	87888819	3330	8	0.625	Promoter(2-3kb)	2613	SPATA31E1	286234

527571	9q	104504315	104505023	709	6	0.5	Promoter(<=1kb)	52	OR13F1	138805
527571	9q	122553263	122554071	809	8	0.5	Promoter(<=1kb)	93	OR1N2	138882
527571	9q	122628595	122629398	804	7	0.286	Promoter(<=1kb)	175	OR1B1	347169
527571	9q	122749914	122750547	634	6	0.833	Promoter(<=1kb)	174	OR1L6	392390
527571	9q	133255635	133256205	571	7	1	3'UTR	19009	ABO	28
527571	9q	135484803	135487213	2411	9	0.333	Promoter(1-2kb)	1440	PPP1R26	9858
527571	10q	46549378	46550723	1346	25	0.64	Exon(exon3of3)	4807	GPRIN2	9721
527571	10q	128102594	128105201	2608	12	0.583	Promoter(<=1kb)	0	MKI67	4288
527571	10q	128106210	128109280	3071	8	0.875	Exon(exon12of14)	-3082	MKI67	4288
527571	11p	244106	244197	92	8	0.5	Promoter(<=1kb)	-232	PSMD13	5719
527571	11p	308290	309127	838	6	0.5	Promoter(<=1kb)	0	IFITM2	10581
527571	11p	1194354	1196902	2549	7	0.571	Exon(exon34of49)	-26164	MUC5B	727897
527571	11p	1246332	1247378	1047	6	0.5	Promoter(2-3kb)	2298	MUC5B-AS1	112577518
527571	11p	5177978	5178478	501	6	0.167	Promoter(<=1kb)	186	OR52Z1	283110
527571	11p	5389704	5390350	647	6	0.5	Promoter(<=1kb)	327	OR51M1	390059
527571	11p	5422212	5423123	912	11	0.636	Promoter(<=1kb)	101	OR51Q1	390061
527571	11p	5440761	5441472	712	6	0.833	Promoter(<=1kb)	42	OR51I1	390063
527571	11p	5544676	5545259	584	6	0.5	Promoter(<=1kb)	290	OR52H1	390067
527571	11p	5581045	5581738	694	8	0.375	Promoter(<=1kb)	168	OR52B6	340980
527571	11p	5841302	5841883	582	9	0.333	Promoter(<=1kb)	14	OR52E6	390078
527571	11p	11351961	11352736	776	9	0.222	Promoter(<=1kb)	514	CSNK2A3	283106
527571	11p	12293639	12294842	1204	7	0.714	Exon(exon29of35)	6739	MICALCL	84953
527571	11p	18173280	18173901	622	7	0.429	Promoter(<=1kb)	443	MRGPRX4	117196
527571	11q	55827393	55827640	248	7	0.714	Promoter(<=1kb)	174	OR5L2	26338
527571	11q	58214757	58215722	966	8	0.25	Promoter(<=1kb)	12	OR1S1	219959
527571	11q	66560202	66562261	2060	6	0.333	Exon(exon14of21)	3873	CTSF	8722
527571	11q	82732630	82733184	555	6	0.833	Promoter(<=1kb)	680	FAM181B	220382
527571	11q	85724687	85725825	1139	6	0.5	Promoter(<=1kb)	0	SYTL2	54843
527571	11q	123906790	123907324	535	6	0.667	Promoter(<=1kb)	644	OR8D4	338662
527571	11q	123943088	123943782	695	7	0.571	Promoter(<=1kb)	91	OR6T1	219874
527571	11q	124015601	124016477	877	9	0.333	Promoter(<=1kb)	26	OR10G4	390264
527571	11q	124023038	124023849	812	10	0.4	Promoter(<=1kb)	25	OR10G9	219870
527571	11q	124382526	124383285	760	8	0.625	Promoter(<=1kb)	58	OR8B2	26595
527571	11q	130914501	130915409	909	10	0.7	Promoter(1-2kb)	1035	SNX19	399979
527571	12p	4626571	4628549	1979	10	0.5	Exon(exon5of6)	14054	DYRK4	8798
527571	12p	6453119	6453670	552	6	0.667	Promoter(<=1kb)	633	TAPBPL	55080
527571	12p	8222174	8223514	1341	6	0.667	Exon(exon5of6)	4073	FAM90A1	55138
527571	12q	52316096	52317765	1670	6	0.667	Exon(exon4of9)	3633	KRT83	3889
527571	13q	24434450	24435347	898	7	0.571	Exon(exon31of34)	19787	PARP4	143
527571	13q	25096675	25097182	508	31	0.581	Promoter(<=1kb)	807	PABPC3	5042
527571	13q	102732474	102733933	1460	6	0.333	Exon(exon4of4)	25139	CCDC168	643677
527571	14q	19827604	19827851	248	6	0.833	Exon(exon2of2)	5669	OR4N2	390429
527571	14q	69572069	69573506	1438	6	0.667	Promoter(1-2kb)	1365	CCDC177	56936
527571	14q	70457520	70458540	1021	13	0.385	Exon(exon2of2)	5346	ADAM21	8747
527571	14q	94587512	94587839	328	6	0.5	Exon(exon2of2)	-4219	SERPINA3	12
527571	14q	104175275	104177810	2536	6	0.167	Exon(exon12of15)	36235	KIF26A	26153
527571	14q	104947943	104949947	2005	7	0.429	Exon(exon6of6)	5455	AHNAK2	113146
527571	15q	20534613	20535129	517	6	0.5	Exon(exon8of9)	6671	GOLGA6L6	727832
527571	15q	23439979	23442067	2089	12	0.583	5'UTR	5167	GOLGA6L2	283685
527571	15q	85579423	85581800	2378	14	0.571	Promoter(1-2kb)	-1110	AKAP13	11214
527571	15q	88854874	88855594	721	6	0.833	Exon(exon12of18)	7631	ACAN	176
527571	15q	88857108	88859365	2258	6	0	Exon(exon12of18)	9865	ACAN	176
527571	15q	99129423	99132517	3095	6	0.333	Exon(exon4of5)	7225	TTC23	64927
527571	16q	74391650	74392004	355	9	0.667	Exon(exon7of7)	13772	NPIPB15	440348
527571	16q	88712207	88714717	2511	7	0.571	Promoter(<=1kb)	0	CTU2	348180
527571	16q	89100686	89101050	365	8	0.625	Promoter(<=1kb)	24	ACSF3	197322
527571	16q	89227206	89228419	1214	6	0.667	Promoter(2-3kb)	2572	ZNF778	197320
527571	17p	10638198	10641099	2902	7	0.286	Exon(exon19of41)	-8169	MYH3	4621
527571	17p	21300581	21300978	398	11	0.727	3'UTR	9112	MAP2K3	5606
527571	17p	21415456	21416416	961	8	0.875	Exon(exon3of3)	10320	KCNJ12	3768
527571	17q	76293419	76294016	598	6	0.5	Promoter(2-3kb)	-2167	QRICH2	84074
527571	17q	80090158	80090265	108	6	0.333	Promoter(2-3kb)	2053	CCDC40	55036
527571	17q	81510690	81511591	902	6	0.667	Promoter(<=1kb)	257	ACTG1	71
527571	17q	81645135	81645607	473	7	0.286	Promoter(2-3kb)	2722	TSPAN10	83882
527571	18p	11609646	11610333	688	10	0.8	Promoter(<=1kb)	50	SLC35G4	646000
527571	18q	58535186	58537515	2330	9	0.333	Promoter(<=1kb)	0	ALPK2	115701
527571	19p	4510548	4513547	3000	19	0.526	Exon(exon3of6)	4157	PLIN4	729359
527571	19p	5455600	5456439	840	7	0.571	Promoter(<=1kb)	183	ZNRF4	148066
527571	19p	8937644	8939234	1591	7	0.571	Exon(exon5of84)	-41554	MUC16	94025
527571	19p	8946313	8952171	5859	19	0.579	Exon(exon3of84)	29171	MUC16	94025
527571	19p	8959116	8962299	3184	12	0.75	Exon(exon3of84)	19043	MUC16	94025
527571	19p	8971838	8978096	6259	15	0.533	Exon(exon1of84)	3246	MUC16	94025
527571	19p	12430718	12432437	1720	8	0.375	3'UTR	8584	ZNF443	10224
527571	19p	18264753	18267409	2657	8	0.5	5'UTR	7002	IQCN	80726
527571	19p	21971930	21974500	2571	7	0.714	Exon(exon4of4)	14408	ZNF208	7757
527571	19p	22663799	22665021	1223	7	0.714	Exon(exon4of4)	29475	ZNF492	57615
527571	19p	22756205	22759523	3319	15	0.6	3'UTR	10459	ZNF99	7652

527571	19q	39877222	39877880	659	6	0.5	Exon(exon20of28)	9412	FCGBP	8857
527571	19q	39886240	39886422	183	6	0.667	Promoter(<=1kb)	870	FCGBP	8857
527571	19q	40880231	40880622	392	6	0.333	Promoter(<=1kb)	-141	CYP2A7	1549
527571	19q	43913423	43914878	1456	9	0.556	Exon(exon10of10)	4861	ZNF45	7596
527571	19q	44327836	44329698	1863	6	0.5	Exon(exon4of4)	-22790	ZNF235	9310
527571	19q	52384029	52384992	964	6	0	Exon(exon4of4)	12677	ZNF528-AS1	102724105
527571	19q	52437918	52439242	1325	7	0.429	Exon(exon4of4)	6504	ZNF534	147658
527571	19q	53164551	53166239	1689	6	0.167	Exon(exon4of4)	-5476	ZNF347	84671
527571	19q	55911888	55913166	1279	7	0.714	Exon(exon5of12)	19145	NLRP13	126204
527571	19q	56664116	56665114	999	6	0.5	Exon(exon2of2)	6643	ZNF835	90485
527571	19q	58368013	58368875	863	8	0.5	Exon(exon3of3)	-5165	ZNF497	162968
527571	20p	5922577	5923382	806	8	0.625	Exon(exon4of5)	7079	CHGB	1114
527571	20p	20052354	20052736	383	6	0	Promoter(<=1kb)	0	CFAP61	26074
527571	20q	63561666	63565947	4282	13	0.615	Promoter(<=1kb)	-61	HELZ2	85441
527571	22q	22352950	22353298	349	13	0.538	Exon(exon1of2)	30478	BMS1P20	96610
527571	22q	22758758	22759209	452	6	0.833	Exon(exon1of2)	-63567	MIR650	723778
527571	22q	36191154	36191906	753	8	0.625	3'UTR	9971	APOL4	80832
527571	22q	39100331	39102033	1703	6	0.833	Promoter(<=1kb)	52	APOBEC3H	164668
527571	23p	3320126	3324529	4404	10	0.6	Exon(exon5of7)	22123	MXRA5	25878
527571	23p	8170039	8170141	103	6	0.5	Promoter(1-2kb)	1126	VCX2	51480
527571	23p	35802148	35803010	863	7	0.571	5'UTR	3357	MAGEB16	139604
527571	23p	38285569	38287917	2349	7	1	3'UTR	-11627	RPGR	6103
527571	23q	102937373	102937765	393	7	0.571	Promoter(<=1kb)	101	RAB40AL	282808
528372	1p	12859108	12860212	1105	10	0.5	Promoter(2-3kb)	2022	PRAMEF2	65122
528372	1p	12861255	12861684	430	6	0.167	Exon(exon4of4)	4169	PRAMEF2	65122
528372	1p	16058491	16060000	1510	13	0.846	Exon(exon5of7)	6168	CLCNKB	1188
528372	1p	18481403	18483159	1757	7	0.714	Promoter(<=1kb)	421	KLHDC7A	127707
528372	1p	40067594	40067675	82	6	0	Promoter(<=1kb)	324	CAP1	10487
528372	1p	89186388	89186419	32	9	0.556	Promoter(<=1kb)	107	GBP4	115361
528372	1q	152218469	152221375	2907	17	0.706	Promoter(2-3kb)	2818	HRNR	388697
528372	1q	152303673	152313891	10219	40	0.575	Promoter(<=1kb)	0	FLG-AS1	339400
528372	1q	156669844	156670886	1043	6	1	Exon(exon4of4)	6521	NES	10763
528372	1q	214640144	214642954	2811	12	0.5	Exon(exon12of20)	-5013	CENPF	1063
528372	1q	232805117	232806964	1848	7	0.429	Promoter(<=1kb)	225	MAP10	54627
528372	1q	247841312	247841582	271	7	0.857	Promoter(<=1kb)	314	OR11L1	391189
528372	1q	247949325	247949738	414	10	0.3	Promoter(<=1kb)	467	OR2L8	391190
528372	2p	48580657	48582454	1798	7	0.571	Promoter(<=1kb)	0	STON1	11037
528372	2q	130914528	130916712	2185	6	0.333	Promoter(<=1kb)	0	ARHGEF4	50649
528372	2q	132783032	132785012	1981	9	0.556	Promoter(1-2kb)	-1009	NCKAP5	344148
528372	2q	167246794	167248478	1685	7	0.714	Promoter(<=1kb)	-204	XIRP2	129446
528372	2q	184936178	184937636	1459	6	0.333	Exon(exon4of4)	69813	ZNF804A	91752
528372	2q	217847583	217848559	977	6	0.833	Exon(exon19of33)	-5423	TNS1	7145
528372	2q	238130542	238131702	1161	8	0.75	Promoter(1-2kb)	1594	ESPNL	339768
528372	2q	240041845	240042131	287	6	0.167	Downstream(2-3kb)	3941	OR6B3	150681
528372	3p	31989532	31990905	1374	7	0.286	Exon(exon2of2)	7761	ZNF860	344787
528372	3p	42638005	42639610	1606	6	0.667	Promoter(2-3kb)	2925	NKTR	4820
528372	3p	75736880	75739007	2128	67	0.597	Promoter(<=1kb)	0	MIR4273	100422955
528372	3q	98264413	98265137	725	7	0.714	Promoter(<=1kb)	128	OR5H6	79295
528372	4p	5988383	5989749	1367	8	0.5	Promoter(<=1kb)	0	C4orf50	389197
528372	4p	6300792	6302360	1569	8	0.875	Exon(exon8of8)	6021	WFS1	7466
528372	5q	79728956	79730716	1761	7	0.286	Exon(exon2of13)	-7426	CMYA5	202333
528372	5q	79731782	79734523	2742	13	0.308	Exon(exon2of13)	-3619	CMYA5	202333
528372	5q	140807352	140807737	386	6	0.833	Promoter(<=1kb)	271	PCDHA4	56144
528372	5q	140848579	140850786	2208	6	0.5	Promoter(<=1kb)	807	PCDHA9	9752
528372	5q	141174000	141175025	1026	6	0.833	Promoter(1-2kb)	1356	PCDHB7	56129
528372	5q	141955356	141957660	2305	6	0.667	Promoter(<=1kb)	-668	RNF14	9604
528372	5q	151565922	151568455	2534	10	0.8	Promoter(<=1kb)	489	FAT2	2196
528372	6p	16327099	16327684	586	7	0.429	Exon(exon8of9)	37154	GMPR	2766
528372	6p	46858771	46859502	732	8	0.5	Exon(exon17of21)	3802	ADGRF5	221395
528372	6q	159233455	159234370	916	10	0.5	Exon(exon11of23)	15158	FNDC1	84624
528372	7p	38353718	38353991	274	9	0.667	Exon(exon2of2)	3699	TRG-AS1	100506776
528372	7q	64991278	64992758	1481	6	0.667	Promoter(<=1kb)	-242	ZNF117	51351
528372	7q	100958721	100960873	2153	67	0.463	Promoter(<=1kb)	756	MUC3A	4584
528372	7q	100991287	100992462	1176	8	0.375	Exon(exon5of15)	-20592	MUC12	10071
528372	7q	100995415	100995785	371	8	0.625	Exon(exon5of15)	-17269	MUC12	10071
528372	7q	101034361	101040583	6223	37	0.514	Exon(exon3of12)	-3128	MUC17	140453
528372	8p	12132686	12133940	1255	6	0.667	Promoter(<=1kb)	498	USP17L7	392197
528372	8p	13021128	13022030	903	8	0.25	Exon(exon5of5)	9115	TRMT9B	57604
528372	8q	123651873	123652634	762	6	0.333	Promoter(<=1kb)	316	KLHL38	340359
528372	9p	21206764	21207074	311	6	0.5	Promoter(<=1kb)	69	IFNA10	3446
528372	9q	76175237	76175300	64	6	0.5	Exon(exon14of14)	-13339	PCSK5	5125
528372	9q	76703555	76706360	2806	8	0.5	Promoter(<=1kb)	0	PCA3	50652
528372	9q	104504315	104505023	709	6	0.5	Promoter(<=1kb)	52	OR13F1	138805
528372	9q	122553263	122554071	809	8	0.5	Promoter(<=1kb)	93	OR1N2	138882
528372	9q	122628595	122629398	804	6	0.333	Promoter(<=1kb)	175	OR1B1	347169
528372	9q	122749914	122750547	634	6	0.833	Promoter(<=1kb)	174	OR1L6	392390
528372	9q	135484803	135487340	2538	9	0.333	Promoter(1-2kb)	1440	PPP1R26	9858

528372	9q	135547960	135548795	836	8	0.625	Promoter(1-2kb)	1805	OBP2A	29991
528372	10q	46549378	46550723	1346	27	0.667	Exon(exon3of3)	4807	GPRIN2	9721
528372	11p	244106	244197	92	8	0.5	Promoter(<=1kb)	-232	PSMD13	5719
528372	11p	1246095	1248605	2511	12	0.5	Promoter(1-2kb)	1071	MUC5B-AS1	112577518
528372	11p	5177978	5178478	501	6	0.167	Promoter(<=1kb)	186	OR52Z1	283110
528372	11p	5323362	5324256	895	8	0.5	Promoter(<=1kb)	41	OR51B2	79345
528372	11p	5389704	5390350	647	7	0.429	Promoter(<=1kb)	327	OR51M1	390059
528372	11p	5422212	5423123	912	11	0.727	Promoter(<=1kb)	101	OR51Q1	390061
528372	11p	5581045	5581738	694	8	0.375	Promoter(<=1kb)	168	OR52B6	340980
528372	11p	5841302	5841883	582	9	0.333	Promoter(<=1kb)	14	OR52E6	390078
528372	11p	11351961	11352736	776	9	0.222	Promoter(<=1kb)	514	CSNK2A3	283106
528372	11q	58214757	58215722	966	7	0.286	Promoter(<=1kb)	12	OR1S1	219959
528372	11q	58402523	58403265	743	9	0.556	Promoter(<=1kb)	144	OR5B3	441608
528372	11q	64315797	64315856	60	8	0.75	Promoter(1-2kb)	1485	TRMT112	51504
528372	11q	85724687	85725825	1139	6	0.5	Promoter(<=1kb)	0	SYTL2	54843
528372	11q	130914501	130915409	909	10	0.7	Promoter(1-2kb)	1035	SNX19	399979
528372	12p	4626568	4628549	1982	11	0.455	Exon(exon5of6)	14051	DYRK4	8798
528372	12p	6453119	6453670	552	6	0.667	Promoter(<=1kb)	633	TAPBPL	55080
528372	13q	24434450	24435347	898	7	0.571	Exon(exon31of34)	19787	PARP4	143
528372	13q	25096665	25097182	518	15	0.533	Promoter(<=1kb)	797	PABPC3	5042
528372	13q	102732474	102733933	1460	6	0.333	Exon(exon4of4)	25139	CCDC168	643677
528372	14q	19827604	19827851	248	6	0.833	Exon(exon2of2)	5669	OR4N2	390429
528372	14q	20060048	20060884	837	8	0.625	Promoter(<=1kb)	3	OR4L1	122742
528372	14q	20143999	20144604	606	9	0.556	Promoter(<=1kb)	263	OR4N5	390437
528372	14q	20640982	20641567	586	6	0.5	Promoter(<=1kb)	124	OR6S1	341799
528372	14q	21634137	21634589	453	9	0.556	Promoter(<=1kb)	351	OR10G2	26534
528372	14q	22633879	22634450	572	9	0.333	Exon(exon2of2)	32212	ABHD4	63874
528372	14q	44504986	44506403	1418	6	0.667	Promoter(<=1kb)	880	FSCB	84075
528372	14q	70457532	70458540	1009	11	0.364	Exon(exon2of2)	5358	ADAM21	8747
528372	14q	77026548	77027532	985	6	0.5	Promoter(1-2kb)	1176	IRF2BPL	64207
528372	14q	104175275	104177762	2488	9	0.222	Exon(exon12of15)	36235	KIF26A	26153
528372	14q	104939262	104945444	6183	20	0.4	5'UTR	7102	PLD4	122618
528372	14q	104946867	104950766	3900	18	0.556	Exon(exon6of6)	4636	AHNAK2	113146
528372	15q	23439979	23442067	2089	12	0.5	5'UTR	5167	GOLGA6L2	283685
528372	15q	40621642	40623696	2055	6	0.333	Promoter(<=1kb)	0	KNL1	57082
528372	15q	73702465	73703760	1296	6	0.833	Promoter(<=1kb)	-149	CD276	80381
528372	15q	85579423	85582073	2651	15	0.6	Promoter(<=1kb)	-837	AKAP13	11214
528372	15q	99129423	99132517	3095	7	0.429	Exon(exon4of5)	7225	TTC23	64927
528372	16p	669592	672548	2957	7	0.571	Promoter(<=1kb)	0	RHOT2	89941
528372	16p	768559	770215	1657	7	0.429	Promoter(<=1kb)	0	MIR662	724032
528372	16p	3355986	3356626	641	6	0.667	Promoter(<=1kb)	97	OR2C1	4993
528372	16p	4207130	4208004	875	6	0.5	Exon(exon2of7)	31739	SRL	6345
528372	16q	89226863	89228289	1427	8	0.625	Promoter(2-3kb)	2229	ZNF778	197320
528372	17p	744946	746966	2021	6	1	3'UTR	5072	GEMIN4	50628
528372	17p	2299649	2300159	511	6	0.167	Exon(exon2of19)	-3224	SRR	63826
528372	17p	10638198	10641099	2902	7	0.286	Exon(exon19of41)	-8169	MYH3	4621
528372	17p	21300581	21300954	374	10	0.7	3'UTR	9112	MAP2K3	5606
528372	18p	11609904	11610469	566	8	0.625	Promoter(<=1kb)	308	SLC35G4	646000
528372	18q	58535186	58537515	2330	10	0.3	Promoter(<=1kb)	0	ALPK2	115701
528372	19p	1036457	1036914	458	7	0.571	Exon(exon6of7)	-3187	ABCA7	10347
528372	19p	4510711	4513547	2837	19	0.421	Exon(exon3of6)	4157	PLIN4	729359
528372	19p	5455600	5456439	840	8	0.625	Promoter(<=1kb)	183	ZNRF4	148066
528372	19p	8937644	8939234	1591	6	0.667	Exon(exon5of84)	-41554	MUC16	94025
528372	19p	8946313	8951868	5556	22	0.636	Exon(exon3of84)	29474	MUC16	94025
528372	19p	8959116	8962299	3184	11	0.727	Exon(exon3of84)	19043	MUC16	94025
528372	19p	8971838	8978096	6259	15	0.533	Exon(exon1of84)	3246	MUC16	94025
528372	19p	10338682	10339609	928	6	0.333	Promoter(<=1kb)	10	ICAM3	3385
528372	19p	12075333	12077046	1714	6	0.167	Promoter(<=1kb)	6	ZNF788P	388507
528372	19p	15087452	15087953	502	7	0.143	Promoter(<=1kb)	472	OR1I1	126370
528372	19p	18264798	18267409	2612	12	0.583	5'UTR	7002	IQCN	80726
528372	19p	21971930	21974500	2571	7	0.714	Exon(exon4of4)	14408	ZNF208	7757
528372	19p	23743906	23745300	1395	6	0.333	Exon(exon4of4)	13537	ZNF681	148213
528372	19q	36996730	36997597	868	10	0.6	Exon(exon10of10)	5677	ZNF568	374900
528372	19q	40880231	40880622	392	6	0.333	Promoter(<=1kb)	-141	CYP2A7	1549
528372	19q	43913423	43914878	1456	8	0.5	Exon(exon10of10)	4861	ZNF45	7596
528372	19q	43996323	43997366	1044	6	0.333	Exon(exon5of5)	5419	LOC10192806:	101928063
528372	19q	44106512	44108078	1567	6	0.167	Exon(exon6of6)	-4103	ZNF225	7768
528372	19q	51745958	51746963	1006	6	0.5	Exon(exon3of3)	3848	FPR1	2357
528372	19q	52437918	52439242	1325	7	0.429	Exon(exon4of4)	6504	ZNF534	147658
528372	19q	55517821	55518176	356	10	0.9	Exon(exon14of14)	18127	SBK2	646643
528372	19q	55912302	55913166	865	6	0.667	Exon(exon5of12)	19145	NLRP13	126204
528372	20p	5922421	5923394	974	8	0.5	Exon(exon4of5)	6923	CHGB	1114
528372	20q	41361473	41362531	1059	6	0.667	Exon(exon4of4)	3497	LPIN3	64900
528372	20q	63349752	63350772	1021	6	0.5	3'UTR	3794	CHRNA4	1137
528372	21q	26843740	26844859	1120	6	0.667	Promoter(<=1kb)	0	ADAMTS1	9510
528372	21q	44539312	44540035	724	6	0.833	Promoter(<=1kb)	160	KRTAP10-1	386677
528372	21q	44637476	44638143	668	6	0.667	Promoter(<=1kb)	120	KRTAP10-10	353333

528372	22q	22352950	22353298	349	13	0.538	Exon(exon1of2)	30478	BMS1P20	96610
528372	22q	22758758	22759209	452	6	0.833	Exon(exon1of2)	-63567	MIR650	723778
528372	22q	36191154	36191906	753	6	0.667	3'UTR	9971	APOL4	80832
528372	23p	3320126	3323750	3625	9	0.556	Exon(exon5of7)	22902	MXRA5	25878
528372	23p	8170039	8170141	103	6	0.5	Promoter(1-2kb)	1126	VCX2	51480
528372	23p	35802148	35803010	863	7	0.571	5'UTR	3357	MAGEB16	139604
528372	23q	136874183	136874416	234	8	0.75	Promoter(<=1kb)	-201	RBMX	27316
605279	1p	11778784	11779941	1158	7	0.714	Promoter(<=1kb)	0	C1orf167-AS1	102724659
605279	1p	12847526	12847995	470	7	0.429	Promoter(<=1kb)	730	HNRNPCL1	343069
605279	1p	12859036	12860079	1044	6	0.333	Promoter(1-2kb)	1950	PRAMEF2	65122
605279	1p	13369166	13369564	399	7	0.714	Promoter(2-3kb)	2336	PRAMEF19	645414
605279	1p	13370686	13371119	434	8	0.375	Promoter(<=1kb)	781	PRAMEF19	645414
605279	1p	18481403	18482217	815	6	0.667	Promoter(<=1kb)	421	KLHDC7A	127707
605279	1p	23874604	23875430	827	9	0.556	Exon(exon2of2)	-6310	FUCA1	2517
605279	1p	40067594	40067675	82	6	0	Promoter(<=1kb)	324	CAP1	10487
605279	1p	89186388	89186419	32	9	0.556	Promoter(<=1kb)	107	GBP4	115361
605279	1q	152303164	152305350	2187	12	0.5	Exon(exon3of3)	-7226	FLG-AS1	339400
605279	1q	152306380	152313891	7512	30	0.567	Promoter(<=1kb)	0	FLG-AS1	339400
605279	1q	169542317	169542882	566	6	0.167	Exon(exon13of25)	-26572	F5	2153
605279	1q	201206099	201209738	3640	11	0.545	Promoter(1-2kb)	1017	IGFN1	91156
605279	1q	201212089	201215147	3059	7	0.571	Promoter(<=1kb)	0	IGFN1	91156
605279	1q	247841312	247841582	271	6	0.833	Promoter(<=1kb)	314	OR11L1	391189
605279	1q	247895950	247896410	461	6	0.5	Promoter(<=1kb)	363	OR2W3	343171
605279	1q	247949325	247949738	414	8	0.25	Promoter(<=1kb)	467	OR2L8	391190
605279	2q	184936178	184937636	1459	6	0.333	Exon(exon4of4)	69813	ZNF804A	91752
605279	2q	185789865	185794632	4768	12	0.75	Promoter(<=1kb)	0	FSIP2	401024
605279	2q	185805377	185807800	2424	6	0.333	Promoter(<=1kb)	0	FSIP2	401024
605279	2q	217847583	217848559	977	6	0.833	Exon(exon19of33)	-5423	TNS1	7145
605279	2q	237762685	237764060	1376	10	0.5	Exon(exon8of8)	-4137	LRRFIP1	9208
605279	2q	238130416	238131546	1131	6	0.333	Promoter(1-2kb)	1468	ESPNL	339768
605279	2q	240041845	240042811	967	8	0.375	Downstream(2-3kb)	3261	OR6B3	150681
605279	3p	31989532	31990905	1374	7	0.429	Exon(exon2of2)	7761	ZNF860	344787
605279	3p	75736880	75739243	2364	55	0.6	Promoter(<=1kb)	0	MIR4273	100422955
605279	4p	5988383	5989749	1367	7	0.571	Promoter(<=1kb)	0	C4orf50	389197
605279	4p	6300792	6302360	1569	8	0.75	Exon(exon8of8)	6021	WFS1	7466
605279	4p	8227004	8228508	1505	8	0.125	Promoter(<=1kb)	-24	SH3TC1	54436
605279	4p	10443803	10446224	2422	7	0.429	Exon(exon3of3)	10952	ZNF518B	85460
605279	4q	154489498	154491312	1815	9	0.556	Promoter(<=1kb)	22	DCHS2	54798
605279	4q	185458217	185460011	1795	8	0.625	Promoter(<=1kb)	0	CCDC110	256309
605279	5q	79728956	79730716	1761	7	0.286	Exon(exon2of13)	-7426	CMYA5	202333
605279	5q	79731782	79734523	2742	13	0.308	Exon(exon2of13)	-3619	CMYA5	202333
605279	5q	83537326	83539905	2580	6	0.333	Promoter(1-2kb)	1712	VCAN	1462
605279	5q	141174000	141175025	1026	7	0.857	Promoter(1-2kb)	1356	PCDHB7	56129
605279	5q	141187690	141189425	1736	6	0.5	Promoter(<=1kb)	529	PCDHB9	56127
605279	6p	26370344	26370479	136	6	0.5	Promoter(<=1kb)	0	BTN3A2	11118
605279	6p	42745312	42746041	730	6	0.667	Promoter(<=1kb)	62	TBCC	6903
605279	6p	46858771	46859502	732	8	0.5	Exon(exon17of21)	3802	ADGRF5	221395
605279	6q	159233455	159234370	916	10	0.5	Exon(exon11of23)	15158	FNDC1	84624
605279	7p	6330446	6330944	499	6	1	Exon(exon2of2)	7749	FAM220A	84792
605279	7p	12369637	12370736	1100	6	0.667	3'UTR	-13307	VWDE	221806
605279	7p	56021087	56021209	123	6	0.5	Exon(exon2of7)	12947	PSPH	5723
605279	7q	100958721	100960873	2153	51	0.471	Promoter(<=1kb)	756	MUC3A	4584
605279	7q	101034305	101038481	4177	20	0.55	Exon(exon3of12)	-5230	MUC17	140453
605279	7q	149818015	149819792	1778	6	0.667	Promoter(2-3kb)	-2352	SSPO	23145
605279	8p	10607245	10612307	5063	18	0.722	Exon(exon4of4)	42836	RP1L1	94137
605279	8p	12132686	12133940	1255	6	0.833	Promoter(<=1kb)	498	USP17L7	392197
605279	8p	12137448	12138641	1194	6	0.833	Promoter(<=1kb)	436	USP17L2	377630
605279	9q	76705179	76707804	2626	8	0.5	Promoter(<=1kb)	121	PCA3	50652
605279	9q	76709263	76710843	1581	8	0.5	Promoter(<=1kb)	0	PRUNE2	158471
605279	9q	104504315	104505071	757	6	0.5	Promoter(<=1kb)	52	OR13F1	138805
605279	9q	104598545	104599361	817	6	0.333	Promoter(<=1kb)	52	OR13C5	138799
605279	9q	122553278	122554071	794	6	0.333	Promoter(<=1kb)	108	OR1N2	138882
605279	9q	133255635	133256205	571	7	1	3'UTR	19009	ABO	28
605279	9q	135484803	135487213	2411	10	0.4	Promoter(1-2kb)	1440	PPP1R26	9858
605279	10q	46549378	46550723	1346	25	0.64	Exon(exon3of3)	4807	GPRIN2	9721
605279	10q	49323169	49326817	3649	16	0.562	Exon(exon3of3)	23895	C10orf71	118461
605279	11p	244106	244197	92	8	0.5	Promoter(<=1kb)	-232	PSMD13	5719
605279	11p	1194354	1196902	2549	7	0.571	Exon(exon34of49)	-26164	MUC5B	727897
605279	11p	5177978	5178478	501	6	0.167	Promoter(<=1kb)	186	OR52Z1	283110
605279	11p	5323451	5324256	806	7	0.571	Promoter(<=1kb)	41	OR51B2	79345
605279	11p	5422212	5423123	912	12	0.667	Promoter(<=1kb)	101	OR51Q1	390061
605279	11p	5440761	5441472	712	6	0.833	Promoter(<=1kb)	42	OR51I1	390063
605279	11p	5581045	5581738	694	8	0.375	Promoter(<=1kb)	168	OR52B6	340980
605279	11p	5788000	5788760	761	8	0.75	Promoter(<=1kb)	56	OR52N1	79473
605279	11p	5841302	5841883	582	9	0.333	Promoter(<=1kb)	14	OR52E6	390078
605279	11p	11352040	11352736	697	8	0.25	Promoter(<=1kb)	514	CSNK2A3	283106
605279	11p	12293639	12294842	1204	7	0.714	Exon(exon29of35)	6739	MICALCL	84953

605279	11p	34916266	34916763	498	6	0.667	Promoter(<=1kb)	0	APIP	51074
605279	11p	43942293	43943348	1056	9	0.778	Promoter(<=1kb)	0	C11orf96	387763
605279	11q	58214757	58215722	966	7	0.286	Promoter(<=1kb)	12	OR1S1	219959
605279	11q	85724687	85725825	1139	6	0.5	Promoter(<=1kb)	0	SYTL2	54843
605279	11q	123906790	123907324	535	6	0.667	Promoter(<=1kb)	644	OR8D4	338662
605279	11q	124038366	124038949	584	6	1	Promoter(<=1kb)	52	OR10G7	390265
605279	11q	124382526	124383285	760	7	0.571	Promoter(<=1kb)	58	OR8B2	26595
605279	12p	4626568	4628549	1982	10	0.5	Exon(exon5of6)	14051	DYRK4	8798
605279	12p	31981306	31983423	2118	6	0.5	Exon(exon4of6)	-3743	RESF1	55196
605279	12q	49795819	49797347	1529	6	0.5	Promoter(<=1kb)	0	NCKAP5L	57701
605279	12q	52571389	52573652	2264	7	0.571	Promoter(<=1kb)	164	KRT74	121391
605279	13q	24434450	24435347	898	7	0.571	Exon(exon31of34)	19787	PARP4	143
605279	13q	25096271	25097182	912	7	0.429	Promoter(<=1kb)	403	PABPC3	5042
605279	13q	102732474	102733933	1460	6	0.333	Exon(exon4of4)	25139	CCDC168	643677
605279	14q	20060048	20060523	476	6	0.5	Promoter(<=1kb)	3	OR4L1	122742
605279	14q	21634218	21634644	427	6	0.833	Promoter(<=1kb)	296	OR10G2	26534
605279	14q	63599676	63599713	38	6	0.5	Exon(exon2of2)	41694	WDR89	112840
605279	14q	70457745	70458238	494	8	0.375	Exon(exon2of2)	5571	ADAM21	8747
605279	15q	23440196	23442067	1872	9	0.556	5'UTR	5167	GOLGA6L2	283685
605279	15q	40621642	40623696	2055	6	0.333	Promoter(<=1kb)	0	KNL1	57082
605279	15q	88854874	88855400	527	6	0.833	Exon(exon12of18)	7631	ACAN	176
605279	15q	88857108	88859365	2258	6	0	Exon(exon12of18)	9865	ACAN	176
605279	16p	789084	790597	1514	6	0.167	Promoter(<=1kb)	0	CHTF18	63922
605279	16p	1256354	1256985	632	12	0.667	Promoter(<=1kb)	285	TPSD1	23430
605279	16p	1486371	1488463	2093	8	0.75	Promoter(<=1kb)	4	PTX4	390667
605279	16p	28495551	28497395	1845	6	0.333	Promoter(<=1kb)	0	CLN3	1201
605279	16q	88428539	88429600	1062	6	0.5	Exon(exon3of3)	-23680	ZFPM1	161882
605279	16q	88430623	88432396	1774	7	0.571	Exon(exon3of3)	-20884	ZFPM1	161882
605279	16q	89226863	89228390	1528	9	0.556	Promoter(2-3kb)	2229	ZNF778	197320
605279	17p	744917	746966	2050	8	0.875	3'UTR	5072	GEMIN4	50628
605279	17p	7482736	7482961	226	6	0.667	Promoter(1-2kb)	1302	ZBTB4	57659
605279	17p	10638424	10641099	2676	6	0.167	Exon(exon19of41)	-8395	MYH3	4621
605279	17p	21300581	21300954	374	8	0.75	3'UTR	9112	MAP2K3	5606
605279	17p	21415458	21416431	974	11	0.818	Exon(exon3of3)	10322	KCNJ12	3768
605279	17q	41586401	41586829	429	6	0.667	Promoter(<=1kb)	66	KRT14	3861
605279	17q	76291123	76294016	2894	11	0.455	Promoter(<=1kb)	0	QRICH2	84074
605279	17q	81645135	81645417	283	6	0.333	Promoter(2-3kb)	2722	TSPAN10	83882
605279	18p	9887397	9888072	676	6	0.667	Promoter(1-2kb)	1383	TXNDC2	84203
605279	18p	11609646	11610333	688	10	0.8	Promoter(<=1kb)	50	SLC35G4	646000
605279	18q	58534888	58537515	2628	12	0.417	Promoter(<=1kb)	0	ALPK2	115701
605279	19p	4510548	4513547	3000	22	0.455	Exon(exon3of6)	4157	PLIN4	729359
605279	19p	5455600	5456439	840	6	0.5	Promoter(<=1kb)	183	ZNRF4	148066
605279	19p	8333830	8334965	1136	7	0.714	Promoter(<=1kb)	0	KANK3	256949
605279	19p	8946313	8951868	5556	15	0.6	Exon(exon3of84)	29474	MUC16	94025
605279	19p	8959116	8962299	3184	10	0.7	Exon(exon3of84)	19043	MUC16	94025
605279	19p	8972467	8978490	6024	18	0.444	Promoter(2-3kb)	2852	MUC16	94025
605279	19p	9126210	9126946	737	9	0.556	Promoter(<=1kb)	4	OR7G3	390883
605279	19p	12430400	12432437	2038	10	0.5	3'UTR	8584	ZNF443	10224
605279	19p	14841061	14841331	271	6	0.5	Promoter(<=1kb)	546	OR7A10	390892
605279	19p	15087213	15088040	828	10	0.3	Promoter(<=1kb)	233	OR1I1	126370
605279	19p	17282085	17284246	2162	8	0.5	Promoter(<=1kb)	0	ANKLE1	126549
605279	19p	18264798	18267409	2612	12	0.583	5'UTR	7002	IQCN	80726
605279	19p	21971930	21974500	2571	7	0.714	Exon(exon4of4)	14408	ZNF208	7757
605279	19q	39877222	39877880	659	6	0.5	Exon(exon20of28)	9412	FCGBP	8857
605279	19q	39886005	39886439	435	7	0.571	Promoter(<=1kb)	853	FCGBP	8857
605279	19q	43913423	43914878	1456	8	0.5	Exon(exon10of10)	4861	ZNF45	7596
605279	19q	43966037	43967171	1135	6	0.167	Promoter(<=1kb)	-691	ZNF155	7711
605279	19q	44106512	44108078	1567	7	0	Exon(exon6of6)	-4103	ZNF225	7768
605279	19q	51745958	51746963	1006	6	0.333	Exon(exon3of3)	3848	FPR1	2357
605279	19q	51872787	51873699	913	10	0.5	Promoter(<=1kb)	-940	ZNF577	84765
605279	19q	52437918	52439242	1325	7	0.429	Exon(exon4of4)	6504	ZNF534	147658
605279	19q	55912302	55913166	865	6	0.667	Exon(exon5of12)	19145	NLRP13	126204
605279	20q	63349752	63350772	1021	6	0.5	3'UTR	3794	CHRNA4	1137
605279	20q	63540866	63543452	2587	10	0.8	Exon(exon1of1)	-3490	PTK6	5753
605279	20q	63561666	63565531	3866	12	0.667	Promoter(<=1kb)	-61	HELZ2	85441
605279	21q	26843740	26844859	1120	6	0.667	Promoter(<=1kb)	0	ADAMTS1	9510
605279	21q	44550835	44551416	582	6	0.833	Promoter(<=1kb)	89	KRTAP10-2	386679
605279	21q	44600627	44601692	1066	11	0.636	Promoter(<=1kb)	30	KRTAP10-7	386675
605279	21q	44637476	44638143	668	7	0.571	Promoter(<=1kb)	120	KRTAP10-10	353333
605279	21q	44666460	44666841	382	7	0.714	Promoter(<=1kb)	86	KRTAP12-2	353323
605279	22q	36191154	36191656	503	6	0.5	Exon(exon4of4)	10221	APOL4	80832
605279	23p	8170039	8170141	103	6	0.5	Promoter(1-2kb)	1126	VCX2	51480
605279	23p	35802148	35803010	863	7	0.571	5'UTR	3357	MAGEB16	139604
605279	23q	136874183	136874347	165	7	0.714	Promoter(<=1kb)	-201	RBMX	27316
609014	1p	978953	979847	895	6	0.5	Promoter(1-2kb)	1182	PERM1	84808
609014	1p	11766028	11768307	2280	6	0.833	Promoter(<=1kb)	0	C1orf167	284498
609014	1p	18481403	18482217	815	7	0.714	Promoter(<=1kb)	421	KLHDC7A	127707

609014	1p	23874604	23875430	827	9	0.556	Exon(exon2of2)	-6310	FUCA1	2517
609014	1p	40067594	40067675	82	6	0	Promoter(<=1kb)	324	CAP1	10487
609014	1q	152218469	152221375	2907	17	0.706	Promoter(2-3kb)	2818	HRNR	388697
609014	1q	152303673	152304920	1248	8	0.5	Exon(exon3of3)	-7656	FLG-AS1	339400
609014	1q	152306380	152313891	7512	30	0.6	Promoter(<=1kb)	0	FLG-AS1	339400
609014	1q	158765805	158766655	851	6	0.5	Promoter(<=1kb)	47	OR6N1	128372
609014	1q	169540901	169542882	1982	9	0.333	Exon(exon13of25)	-25156	F5	2153
609014	1q	201206099	201209738	3640	10	0.6	Promoter(1-2kb)	1017	IGFN1	91156
609014	1q	228315976	228318038	2063	10	0.6	Exon(exon50of81)	6492	OBSCN	84033
609014	1q	240207634	240208254	621	11	0.545	Exon(exon5of18)	-27517	FMN2	56776
609014	1q	247841312	247841582	271	6	0.833	Promoter(<=1kb)	314	OR11L1	391189
609014	1q	247949325	247949738	414	9	0.333	Promoter(<=1kb)	467	OR2L8	391190
609014	1q	248294677	248295458	782	6	0.667	Promoter(<=1kb)	142	OR2T12	127064
609014	2p	29071763	29073000	1238	6	0.667	Promoter(1-2kb)	1523	PCARE	388939
609014	2p	48580657	48582454	1798	7	0.571	Promoter(<=1kb)	0	STON1	11037
609014	2q	132781993	132785001	3009	9	0.444	Promoter(<=1kb)	0	NCKAP5	344148
609014	2q	185789865	185794632	4768	10	0.8	Promoter(<=1kb)	0	FSIP2	401024
609014	2q	185805377	185808170	2794	6	0.333	Promoter(<=1kb)	0	FSIP2	401024
609014	2q	235041167	235041640	474	6	0.167	Exon(exon4of6)	46327	SH3BP4	23677
609014	2q	238130416	238131573	1158	7	0.429	Promoter(1-2kb)	1468	ESPNL	339768
609014	3p	75736929	75739007	2079	13	0.692	Promoter(<=1kb)	0	MIR4273	100422955
609014	3q	98264413	98265137	725	8	0.625	Promoter(<=1kb)	128	OR5H6	79295
609014	3q	194341097	194342571	1475	7	0.714	Exon(exon2of2)	8747	CPN2	1370
609014	4p	5988383	5989749	1367	7	0.571	Promoter(<=1kb)	0	C4orf50	389197
609014	4p	6300792	6302360	1569	6	0.833	Exon(exon8of8)	6021	WFS1	7466
609014	4q	56314397	56316592	2196	7	1	Promoter(<=1kb)	-71	CRACD	57482
609014	5q	79728956	79730716	1761	6	0.333	Exon(exon2of13)	-7426	CMYA5	202333
609014	5q	79731782	79734523	2742	13	0.308	Exon(exon2of13)	-3619	CMYA5	202333
609014	5q	140807352	140808990	1639	8	0.5	Promoter(<=1kb)	271	PCDHA4	56144
609014	5q	141174000	141175025	1026	6	0.833	Promoter(1-2kb)	1356	PCDHB7	56129
609014	5q	141187690	141189425	1736	6	0.5	Promoter(<=1kb)	529	PCDHB9	56127
609014	5q	141955676	141957660	1985	6	0.667	Promoter(<=1kb)	-668	RNF14	9604
609014	5q	148826877	148828070	1194	7	0.857	Promoter(1-2kb)	1632	ADRB2	154
609014	6p	46858771	46859389	619	6	0.5	Exon(exon17of21)	3915	ADGRF5	221395
609014	6q	64591274	64591961	688	10	0.5	Exon(exon26of43)	121374	EYS	346007
609014	6q	149888581	149889987	1407	6	0.667	Promoter(<=1kb)	-116	RAET1E-AS1	100652739
609014	6q	159231899	159234370	2472	10	0.5	Exon(exon11of23)	13602	FNDC1	84624
609014	7p	12369637	12370736	1100	6	0.667	3'UTR	-13307	VWDE	221806
609014	7q	100958721	100960873	2153	51	0.471	Promoter(<=1kb)	756	MUC3A	4584
609014	7q	100991195	100992398	1204	6	0.5	Exon(exon5of15)	-20656	MUC12	10071
609014	8p	10609614	10612307	2694	10	0.9	Exon(exon4of4)	42836	RP1L1	94137
609014	8p	12132477	12133936	1460	8	0.625	Promoter(<=1kb)	502	USP17L7	392197
609014	8p	13021128	13022030	903	7	0.143	Exon(exon5of5)	9115	TRMT9B	57604
609014	8q	100706704	100706973	270	7	0.857	Promoter(<=1kb)	0	PABPC1	26986
609014	8q	123651873	123652634	762	6	0.333	Promoter(<=1kb)	316	KLHL38	340359
609014	9p	34723747	34725072	1326	6	0.667	Exon(exon4of4)	4416	FAM205A	259308
609014	9q	76705724	76707804	2081	6	0.5	Promoter(<=1kb)	666	PCA3	50652
609014	9q	122553278	122554071	794	7	0.286	Promoter(<=1kb)	108	OR1N2	138882
609014	9q	122628595	122629398	804	6	0.333	Promoter(<=1kb)	175	OR1B1	347169
609014	9q	135484803	135487213	2411	8	0.25	Promoter(1-2kb)	1440	PPP1R26	9858
609014	10q	46549378	46550723	1346	26	0.654	Exon(exon3of3)	4807	GPRIN2	9721
609014	10q	128102594	128106296	3703	8	0.625	Promoter(<=1kb)	0	MKI67	4288
609014	11p	244106	244197	92	8	0.5	Promoter(<=1kb)	-232	PSMD13	5719
609014	11p	5046754	5047432	679	7	0.571	Promoter(<=1kb)	228	OR52J3	119679
609014	11p	5177978	5178478	501	6	0.167	Promoter(<=1kb)	186	OR52Z1	283110
609014	11p	5351521	5352416	896	14	0.429	Promoter(<=1kb)	13	OR51B6	390058
609014	11p	5389704	5390350	647	7	0.429	Promoter(<=1kb)	327	OR51M1	390059
609014	11p	5422212	5423123	912	11	0.636	Promoter(<=1kb)	101	OR51Q1	390061
609014	11p	5544676	5545259	584	6	0.5	Promoter(<=1kb)	290	OR52H1	390067
609014	11p	5581045	5581738	694	9	0.444	Promoter(<=1kb)	168	OR52B6	340980
609014	11p	5841302	5841883	582	9	0.333	Promoter(<=1kb)	14	OR52E6	390078
609014	11p	11352040	11352736	697	6	0.167	Promoter(<=1kb)	514	CSNK2A3	283106
609014	11p	12293639	12294368	730	6	0.833	Exon(exon29of35)	6739	MICALCL	84953
609014	11q	55572176	55572903	728	6	0.667	Promoter(<=1kb)	48	OR4C16	219428
609014	11q	58214757	58215722	966	7	0.286	Promoter(<=1kb)	12	OR1S1	219959
609014	11q	64116513	64118232	1720	8	0.75	Exon(exon2of2)	8702	MACROD1	28992
609014	11q	82732630	82733184	555	6	0.833	Promoter(<=1kb)	680	FAM181B	220382
609014	11q	85724687	85725825	1139	6	0.5	Promoter(<=1kb)	0	SYTL2	54843
609014	11q	123906595	123907324	730	7	0.429	Promoter(<=1kb)	449	OR8D4	338662
609014	11q	124015600	124016477	878	7	0.143	Promoter(<=1kb)	25	OR10G4	390264
609014	11q	124038920	124038988	69	6	0.833	Promoter(<=1kb)	13	OR10G7	390265
609014	11q	124382526	124383285	760	9	0.556	Promoter(<=1kb)	58	OR8B2	26595
609014	11q	130914501	130915409	909	10	0.7	Promoter(1-2kb)	1035	SNX19	399979
609014	12p	4626568	4628549	1982	11	0.455	Exon(exon5of6)	14051	DYRK4	8798
609014	12q	49795819	49797084	1266	6	0.5	Promoter(<=1kb)	0	NCKAP5L	57701
609014	12q	52316096	52317765	1670	6	0.667	Exon(exon4of9)	3633	KRT83	3889
609014	13q	25096713	25097231	519	9	0.444	Promoter(<=1kb)	845	PABPC3	5042

609014	13q	102732474	102733933	1460	6	0.333	Exon(exon4of4)	25139	CCDC168	643677
609014	14q	20640982	20641567	586	6	0.5	Promoter(<=1kb)	124	OR6S1	341799
609014	14q	21634162	21634589	428	7	0.714	Promoter(<=1kb)	351	OR10G2	26534
609014	14q	44504986	44506403	1418	6	0.667	Promoter(<=1kb)	880	FSCB	84075
609014	14q	70457745	70458784	1040	11	0.364	Exon(exon2of2)	5571	ADAM21	8747
609014	15q	23440196	23442067	1872	10	0.6	5'UTR	5167	GOLGA6L2	283685
609014	15q	40621642	40623696	2055	6	0.333	Promoter(<=1kb)	0	KNL1	57082
609014	15q	59206980	59208041	1062	14	0.357	Promoter(<=1kb)	137	LDHAL6B	92483
609014	15q	85579423	85581800	2378	14	0.571	Promoter(1-2kb)	-1110	AKAP13	11214
609014	15q	99130060	99132394	2335	6	0.5	Exon(exon4of5)	7348	TTC23	64927
609014	16p	1229573	1229716	144	6	0.667	Promoter(<=1kb)	446	TPSB2	64499
609014	16p	1256354	1256985	632	11	0.636	Promoter(<=1kb)	285	TPSD1	23430
609014	16p	1486371	1488463	2093	8	0.75	Promoter(<=1kb)	4	PTX4	390667
609014	16q	74391650	74391897	248	9	0.778	Exon(exon7of7)	13772	NPIPB15	440348
609014	16q	89226863	89228289	1427	7	0.571	Promoter(2-3kb)	2229	ZNF778	197320
609014	17p	744946	746966	2021	6	1	3'UTR	5072	GEMIN4	50628
609014	17p	21300581	21300954	374	8	0.75	3'UTR	9112	MAP2K3	5606
609014	17p	21415458	21416431	974	14	0.857	Exon(exon3of3)	10322	KCNJ12	3768
609014	17q	76293419	76294016	598	6	0.5	Promoter(2-3kb)	-2167	QRICH2	84074
609014	17q	81645135	81645417	283	6	0.333	Promoter(2-3kb)	2722	TSPAN10	83882
609014	18p	9887152	9888072	921	6	0.833	Promoter(1-2kb)	1138	TXNDC2	84203
609014	18q	58535186	58537515	2330	9	0.333	Promoter(<=1kb)	0	ALPK2	115701
609014	19p	4511338	4513547	2210	11	0.273	Exon(exon3of6)	4157	PLIN4	729359
609014	19p	5455600	5456439	840	6	0.5	Promoter(<=1kb)	183	ZNRF4	148066
609014	19p	8937644	8939234	1591	6	0.667	Exon(exon5of84)	-41554	MUC16	94025
609014	19p	8948231	8953259	5029	15	0.533	Exon(exon3of84)	28083	MUC16	94025
609014	19p	8959403	8962479	3077	9	0.556	Exon(exon3of84)	18863	MUC16	94025
609014	19p	8964274	8966760	2487	19	0.737	Exon(exon3of84)	14582	MUC16	94025
609014	19p	15087389	15087953	565	6	0.333	Promoter(<=1kb)	409	OR1I1	126370
609014	19p	18264798	18267409	2612	12	0.583	5'UTR	7002	IQCN	80726
609014	19p	21971930	21974500	2571	7	0.714	Exon(exon4of4)	14408	ZNF208	7757
609014	19p	23743906	23745300	1395	6	0.333	Exon(exon4of4)	13537	ZNF681	148213
609014	19q	36996730	36997597	868	10	0.7	Exon(exon10of10)	5677	ZNF568	374900
609014	19q	37885190	37888806	3617	8	0.625	Exon(exon6of6)	17788	WDR87	83889
609014	19q	43846955	43848536	1582	6	0.833	3'UTR	13450	ZNF283	284349
609014	19q	43913423	43914878	1456	7	0.429	Exon(exon10of10)	4861	ZNF45	7596
609014	19q	43996326	43997366	1041	6	0.5	Exon(exon5of5)	5419	LOC10192806[illegible]	101928063
609014	19q	44106512	44108078	1567	7	0	Exon(exon6of6)	-4103	ZNF225	7768
609014	19q	44327836	44329698	1863	6	0.5	Exon(exon4of4)	-22790	ZNF235	9310
609014	19q	55517821	55518642	822	8	1	Exon(exon14of14)	17661	SBK2	646643
609014	19q	55911888	55913077	1190	6	0.667	Exon(exon5of12)	19234	NLRP13	126204
609014	19q	58368293	58368875	583	7	0.429	Exon(exon3of3)	-5445	ZNF497	162968
609014	20p	5922421	5923394	974	6	0.5	Exon(exon4of5)	6923	CHGB	1114
609014	20p	20052354	20052736	383	7	0.143	Promoter(<=1kb)	0	CFAP61	26074
609014	20q	63540866	63543452	2587	10	0.8	Exon(exon1of1)	-3490	PTK6	5753
609014	21q	26843740	26844859	1120	6	0.667	Promoter(<=1kb)	0	ADAMTS1	9510
609014	21q	44550835	44551416	582	7	0.857	Promoter(<=1kb)	89	KRTAP10-2	386679
609014	21q	44558207	44558709	503	6	0.5	Promoter(<=1kb)	86	KRTAP10-3	386682
609014	21q	44637474	44638143	670	11	0.455	Promoter(<=1kb)	118	KRTAP10-10	353333
609014	21q	44666460	44666841	382	7	0.714	Promoter(<=1kb)	86	KRTAP12-2	353323
609014	21q	45455650	45456666	1017	6	0.667	Promoter(<=1kb)	119	COL18A1	80781
609014	22q	22352950	22353365	416	15	0.533	Exon(exon1of2)	30478	BMS1P20	96610
609014	22q	36191154	36191906	753	6	0.667	3'UTR	9971	APOL4	80832
609014	22q	39100331	39102033	1703	6	0.833	Promoter(<=1kb)	52	APOBEC3H	164668
609014	22q	49884187	49884994	808	6	0.333	Exon(exon2of2)	22924	ALG12	79087
609014	23p	3320126	3323750	3625	10	0.6	Exon(exon5of7)	22902	MXRA5	25878
609014	23p	8170039	8170141	103	6	0.5	Promoter(1-2kb)	1126	VCX2	51480
609014	23p	35802148	35803010	863	7	0.571	5'UTR	3357	MAGEB16	139604
609548	1p	978953	979847	895	6	0.333	Promoter(1-2kb)	1182	PERM1	84808
609548	1p	16058491	16059952	1462	7	0.857	Exon(exon5of7)	6168	CLCNKB	1188
609548	1p	18481403	18482217	815	6	0.667	Promoter(<=1kb)	421	KLHDC7A	127707
609548	1p	40067594	40067675	82	6	0	Promoter(<=1kb)	324	CAP1	10487
609548	1p	89186388	89186419	32	9	0.556	Promoter(<=1kb)	107	GBP4	115361
609548	1q	152219233	152221375	2143	17	0.706	Promoter(2-3kb)	2818	HRNR	388697
609548	1q	158765805	158766655	851	6	0.5	Promoter(<=1kb)	47	OR6N1	128372
609548	1q	169542317	169542882	566	6	0.167	Exon(exon13of25)	-26572	F5	2153
609548	1q	197101312	197101771	460	6	0.5	Exon(exon18of28)	33373	ASPM	259266
609548	1q	201206099	201209738	3640	11	0.545	Promoter(1-2kb)	1017	IGFN1	91156
609548	1q	201210956	201212792	1837	6	0.5	Promoter(<=1kb)	0	IGFN1	91156
609548	1q	226735683	226737239	1557	7	0.857	Promoter(<=1kb)	219	ITPKB	3707
609548	1q	228315976	228318038	2063	8	0.625	Exon(exon50of81)	6492	OBSCN	84033
609548	1q	247841312	247841582	271	6	0.833	Promoter(<=1kb)	314	OR11L1	391189
609548	1q	248060797	248061452	656	7	0.571	Exon(exon2of2)	22625	OR2L2	26246
609548	1q	248294677	248295458	782	7	0.571	Promoter(<=1kb)	142	OR2T12	127064
609548	2p	29071763	29073000	1238	6	0.667	Promoter(1-2kb)	1523	PCARE	388939
609548	2q	185789865	185794632	4768	10	0.8	Promoter(<=1kb)	0	FSIP2	401024
609548	2q	219271337	219271649	313	6	0.667	Exon(exon4of4)	6227	TUBA4A	7277

609548	2q	219539659	219540599	941	6	0.667	Promoter(2-3kb)	2498	CHPF	79586
609548	2q	237762685	237764060	1376	10	0.5	Exon(exon8of8)	-4137	LRRFIP1	9208
609548	3p	31989532	31990905	1374	6	0.333	Exon(exon2of2)	7761	ZNF860	344787
609548	3p	75736880	75739243	2364	40	0.575	Promoter(<=1kb)	0	MIR4273	100422955
609548	3q	195867934	195868534	601	6	0.833	Promoter(1-2kb)	1156	TNK2	10188
609548	4p	5988383	5989749	1367	7	0.571	Promoter(<=1kb)	0	C4orf50	389197
609548	4p	6300792	6302360	1569	6	0.833	Exon(exon8of8)	6021	WFS1	7466
609548	4p	7433331	7434759	1429	8	0.875	Promoter(<=1kb)	171	PSAPL1	768239
609548	4p	8227004	8228508	1505	8	0.125	Promoter(<=1kb)	-24	SH3TC1	54436
609548	4q	185458217	185460011	1795	8	0.625	Promoter(<=1kb)	0	CCDC110	256309
609548	4q	186619481	186621582	2102	6	0.333	Exon(exon10of27)	-9411	FAT1	2195
609548	4q	186706638	186709751	3114	7	0.714	Exon(exon2of27)	14082	FAT1	2195
609548	5q	83537326	83539905	2580	8	0.375	Promoter(1-2kb)	1712	VCAN	1462
609548	5q	140848579	140850786	2208	7	0.571	Promoter(<=1kb)	807	PCDHA9	9752
609548	5q	141174000	141175025	1026	6	0.833	Promoter(1-2kb)	1356	PCDHB7	56129
609548	5q	141187690	141189425	1736	6	0.5	Promoter(<=1kb)	529	PCDHB9	56127
609548	5q	141955676	141957660	1985	6	0.667	Promoter(<=1kb)	-668	RNF14	9604
609548	5q	151521550	151522069	520	6	0.667	Promoter(<=1kb)	79	MIR6499	102465246
609548	6p	46858771	46859502	732	8	0.5	Exon(exon17of21)	3802	ADGRF5	221395
609548	6q	149888581	149890867	2287	7	0.714	Promoter(<=1kb)	0	RAET1E-AS1	100652739
609548	6q	167340203	167341487	1285	6	0.5	Exon(exon2of2)	15098	TTLL2	83887
609548	7p	12369637	12370736	1100	6	0.667	3'UTR	-13307	VWDE	221806
609548	7q	89334686	89336565	1880	7	0.571	Exon(exon4of4)	116532	ZNF804B	219578
609548	7q	100958721	100960873	2153	52	0.462	Promoter(<=1kb)	756	MUC3A	4584
609548	7q	100991195	100992398	1204	7	0.571	Exon(exon5of15)	-20656	MUC12	10071
609548	7q	100995547	100995858	312	6	0.833	Exon(exon5of15)	-17196	MUC12	10071
609548	7q	149818015	149819792	1778	6	0.667	Promoter(2-3kb)	-2352	SSPO	23145
609548	8p	10607375	10608261	887	6	0.5	Exon(exon4of4)	46882	RP1L1	94137
609548	8p	12132686	12133940	1255	6	0.667	Promoter(<=1kb)	498	USP17L7	392197
609548	8p	13021128	13022030	903	7	0.143	Exon(exon5of5)	9115	TRMT9B	57604
609548	8q	123651622	123652634	1013	6	0.5	Promoter(<=1kb)	316	KLHL38	340359
609548	8q	138151949	138153046	1098	6	0.667	Promoter(<=1kb)	0	FAM135B	51059
609548	9q	76175237	76175261	25	6	0.833	Exon(exon14of14)	-13378	PCSK5	5125
609548	9q	76703555	76707804	4250	13	0.538	Promoter(<=1kb)	0	PCA3	50652
609548	9q	76709523	76710843	1321	7	0.571	Promoter(<=1kb)	0	PRUNE2	158471
609548	9q	104694482	104695444	963	6	0.5	Promoter(<=1kb)	0	OR13D1	286365
609548	9q	122553263	122554071	809	7	0.429	Promoter(<=1kb)	93	OR1N2	138882
609548	10q	46549378	46550723	1346	25	0.64	Exon(exon3of3)	4807	GPRIN2	9721
609548	10q	49323504	49325572	2069	7	0.429	Exon(exon3of3)	24230	C10orf71	118461
609548	10q	125026145	125027175	1031	6	0.5	Promoter(<=1kb)	853	CTBP2	1488
609548	11p	244106	244197	92	8	0.5	Promoter(<=1kb)	-232	PSMD13	5719
609548	11p	1194354	1196902	2549	7	0.571	Exon(exon34of49)	-26164	MUC5B	727897
609548	11p	5177978	5178478	501	6	0.167	Promoter(<=1kb)	186	OR52Z1	283110
609548	11p	5323362	5324256	895	7	0.429	Promoter(<=1kb)	41	OR51B2	79345
609548	11p	5389704	5390350	647	7	0.429	Promoter(<=1kb)	327	OR51M1	390059
609548	11p	5422212	5423123	912	11	0.636	Promoter(<=1kb)	101	OR51Q1	390061
609548	11p	5581045	5581738	694	8	0.375	Promoter(<=1kb)	168	OR52B6	340980
609548	11p	5788000	5788760	761	8	0.75	Promoter(<=1kb)	56	OR52N1	79473
609548	11p	5841302	5841883	582	9	0.333	Promoter(<=1kb)	14	OR52E6	390078
609548	11p	11352040	11352736	697	8	0.25	Promoter(<=1kb)	514	CSNK2A3	283106
609548	11p	12293639	12294368	730	6	0.833	Exon(exon29of35)	6739	MICALCL	84953
609548	11q	55572176	55572903	728	6	0.667	Promoter(<=1kb)	48	OR4C16	219428
609548	11q	58214757	58215722	966	9	0.222	Promoter(<=1kb)	12	OR1S1	219959
609548	11q	58402523	58403265	743	8	0.5	Promoter(<=1kb)	144	OR5B3	441608
609548	11q	123906790	123907324	535	6	0.667	Promoter(<=1kb)	644	OR8D4	338662
609548	11q	123943088	123943782	695	7	0.571	Promoter(<=1kb)	91	OR6T1	219874
609548	11q	124015601	124016477	877	7	0.143	Promoter(<=1kb)	26	OR10G4	390264
609548	11q	124023038	124023849	812	7	0.429	Promoter(<=1kb)	25	OR10G9	219870
609548	11q	124038366	124038988	623	8	1	Promoter(<=1kb)	13	OR10G7	390265
609548	11q	124382526	124383285	760	6	0.5	Promoter(<=1kb)	58	OR8B2	26595
609548	12p	4626571	4628549	1979	9	0.444	Exon(exon5of6)	14054	DYRK4	8798
609548	12q	52316096	52317765	1670	6	0.667	Exon(exon4of9)	3633	KRT83	3889
609548	13q	25096713	25097231	519	14	0.5	Promoter(<=1kb)	845	PABPC3	5042
609548	13q	102732474	102734807	2334	7	0.286	Exon(exon4of4)	24265	CCDC168	643677
609548	14q	19935750	19936577	828	6	0.833	Promoter(<=1kb)	142	OR4K1	79544
609548	14q	20060048	20060884	837	8	0.625	Promoter(<=1kb)	3	OR4L1	122742
609548	14q	21634162	21634589	428	7	0.714	Promoter(<=1kb)	351	OR10G2	26534
609548	14q	63599645	63599684	40	8	0.625	Exon(exon2of2)	41723	WDR89	112840
609548	14q	70457532	70458948	1417	11	0.364	Exon(exon2of2)	5358	ADAM21	8747
609548	14q	104177072	104178677	1606	6	0.333	Exon(exon12of15)	38032	KIF26A	26153
609548	15q	23440370	23442067	1698	9	0.667	5'UTR	5167	GOLGA6L2	283685
609548	15q	59206980	59208056	1077	12	0.5	Promoter(<=1kb)	137	LDHAL6B	92483
609548	15q	78766033	78766632	600	9	0.444	Promoter(<=1kb)	-920	ADAMTS7	11173
609548	15q	85579423	85581800	2378	15	0.533	Promoter(1-2kb)	-1110	AKAP13	11214
609548	15q	88854874	88855400	527	6	0.833	Exon(exon12of18)	7631	ACAN	176
609548	15q	88857108	88859365	2258	6	0	Exon(exon12of18)	9865	ACAN	176
609548	16p	1229573	1229731	159	8	0.625	Promoter(<=1kb)	431	TPSB2	64499

609548	16p	1256345	1256985	641	14	0.714	Promoter(<=1kb)	276	TPSD1	23430
609548	16p	1486371	1488463	2093	8	0.75	Promoter(<=1kb)	4	PTX4	390667
609548	16q	74391549	74391897	349	10	0.8	Exon(exon7of7)	13671	NPIPB15	440348
609548	16q	88430623	88436097	5475	13	0.615	Exon(exon3of3)	-17183	ZFPM1	161882
609548	16q	89226863	89228390	1528	9	0.556	Promoter(2-3kb)	2229	ZNF778	197320
609548	17p	744917	746695	1779	8	0.875	3'UTR	5343	GEMIN4	50628
609548	17p	21300581	21300954	374	10	0.8	3'UTR	9112	MAP2K3	5606
609548	17p	21415458	21416431	974	14	0.857	Exon(exon3of3)	10322	KCNJ12	3768
609548	17q	76293419	76294016	598	6	0.5	Promoter(2-3kb)	-2167	QRICH2	84074
609548	17q	81645135	81645417	283	6	0.333	Promoter(2-3kb)	2722	TSPAN10	83882
609548	18q	58535186	58538030	2845	19	0.526	Promoter(<=1kb)	0	ALPK2	115701
609548	18q	75285722	75287404	1683	6	0.333	Exon(exon2of2)	41146	TSHZ1	10194
609548	19p	4510548	4513547	3000	20	0.45	Exon(exon3of6)	4157	PLIN4	729359
609548	19p	8937644	8939234	1591	6	0.667	Exon(exon5of84)	-41554	MUC16	94025
609548	19p	8946313	8952171	5859	20	0.6	Exon(exon3of84)	29171	MUC16	94025
609548	19p	8959116	8962299	3184	12	0.75	Exon(exon3of84)	19043	MUC16	94025
609548	19p	8971838	8978096	6259	15	0.533	Exon(exon1of84)	3246	MUC16	94025
609548	19p	15087452	15087953	502	7	0.143	Promoter(<=1kb)	472	OR1I1	126370
609548	19p	17281820	17284246	2427	9	0.556	Promoter(<=1kb)	0	ANKLE1	126549
609548	19p	18264798	18267409	2612	12	0.583	5'UTR	7002	IQCN	80726
609548	19p	21971930	21974500	2571	6	0.667	Exon(exon4of4)	14408	ZNF208	7757
609548	19p	22757892	22759533	1642	9	0.444	Exon(exon4of4)	10449	ZNF99	7652
609548	19p	23743906	23745300	1395	6	0.333	Exon(exon4of4)	13537	ZNF681	148213
609548	19q	36996730	36997597	868	10	0.7	Exon(exon10of10)	5677	ZNF568	374900
609548	19q	37151928	37153149	1222	6	0.833	Exon(exon5of5)	19287	ZNF585A	199704
609548	19q	37885190	37888806	3617	8	0.625	Exon(exon6of6)	17788	WDR87	83889
609548	19q	39877222	39877880	659	6	0.5	Exon(exon20of28)	9412	FCGBP	8857
609548	19q	39886005	39886439	435	7	0.571	Promoter(<=1kb)	853	FCGBP	8857
609548	19q	43204774	43205504	731	6	0.833	Promoter(<=1kb)	47	PSG4	5672
609548	19q	43913423	43914878	1456	8	0.5	Exon(exon10of10)	4861	ZNF45	7596
609548	19q	44106645	44108078	1434	6	0	Exon(exon6of6)	-4103	ZNF225	7768
609548	19q	44327836	44329698	1863	6	0.5	Exon(exon4of4)	-22790	ZNF235	9310
609548	19q	51745958	51746963	1006	6	0.333	Exon(exon3of3)	3848	FPR1	2357
609548	19q	52437918	52439242	1325	7	0.429	Exon(exon4of4)	6504	ZNF534	147658
609548	19q	55911888	55913077	1190	6	0.667	Exon(exon5of12)	19234	NLRP13	126204
609548	19q	58368293	58368875	583	7	0.429	Exon(exon3of3)	-5445	ZNF497	162968
609548	20p	5922577	5923382	806	7	0.571	Exon(exon4of5)	7079	CHGB	1114
609548	20q	63349752	63350772	1021	6	0.5	3'UTR	3794	CHRNA4	1137
609548	20q	63561666	63565531	3866	12	0.667	Promoter(<=1kb)	-61	HELZ2	85441
609548	21q	44550738	44551416	679	7	0.857	Promoter(<=1kb)	89	KRTAP10-2	386679
609548	21q	44637474	44638041	568	9	0.333	Promoter(<=1kb)	118	KRTAP10-10	353333
609548	22q	21029917	21031730	1814	6	0.833	Promoter(<=1kb)	819	SLC7A4	6545
609548	22q	22352950	22353348	399	14	0.571	Exon(exon1of2)	30478	BMS1P20	96610
609548	22q	36191154	36191906	753	7	0.714	3'UTR	9971	APOL4	80832
609548	23p	8170039	8170141	103	6	0.5	Promoter(1-2kb)	1126	VCX2	51480
609548	23q	136874183	136874347	165	7	0.714	Promoter(<=1kb)	-201	RBMX	27316
609548	23q	141906066	141906494	429	6	0.5	Promoter(1-2kb)	1264	MAGEC1	9947
611358	1p	978953	979884	932	7	0.571	Promoter(1-2kb)	1145	PERM1	84808
611358	1p	18481403	18482217	815	6	0.667	Promoter(<=1kb)	421	KLHDC7A	127707
611358	1p	23874604	23875430	827	8	0.5	Exon(exon2of2)	-6310	FUCA1	2517
611358	1p	40067594	40067675	82	6	0	Promoter(<=1kb)	324	CAP1	10487
611358	1p	89186388	89186419	32	9	0.556	Promoter(<=1kb)	107	GBP4	115361
611358	1q	152302977	152313891	10915	43	0.581	Promoter(<=1kb)	0	FLG-AS1	339400
611358	1q	156669844	156670886	1043	6	1	Exon(exon4of4)	6521	NES	10763
611358	1q	158765805	158766655	851	6	0.5	Promoter(<=1kb)	47	OR6N1	128372
611358	1q	201206099	201209738	3640	10	0.6	Promoter(1-2kb)	1017	IGFN1	91156
611358	1q	223393517	223394466	950	7	0.714	Promoter(<=1kb)	102	CCDC185	164127
611358	1q	236553659	236555893	2235	6	0.167	Exon(exon39of45)	10363	LGALS8	3964
611358	1q	247841312	247841582	271	6	0.833	Promoter(<=1kb)	314	OR11L1	391189
611358	1q	247949325	247949738	414	9	0.333	Promoter(<=1kb)	467	OR2L8	391190
611358	1q	248573992	248574210	219	6	0.833	Promoter(<=1kb)	547	OR2T34	127068
611358	1q	248681658	248682198	541	7	0.571	Promoter(<=1kb)	130	OR14I1	401994
611358	2p	29002636	29003646	1011	6	0.333	Exon(exon5of20)	-10821	TOGARAM2	165186
611358	2q	184936178	184937636	1459	6	0.333	Exon(exon4of4)	69813	ZNF804A	91752
611358	2q	185789865	185794632	4768	10	0.8	Promoter(<=1kb)	0	FSIP2	401024
611358	2q	185805377	185808170	2794	6	0.333	Promoter(<=1kb)	0	FSIP2	401024
611358	2q	217847583	217848559	977	6	0.833	Exon(exon19of33)	-5423	TNS1	7145
611358	2q	238130416	238131546	1131	6	0.333	Promoter(1-2kb)	1468	ESPNL	339768
611358	3p	75736880	75738859	1980	47	0.574	Promoter(<=1kb)	0	MIR4273	100422955
611358	3q	98264413	98265137	725	6	0.667	Promoter(<=1kb)	128	OR5H6	79295
611358	4p	5988383	5989749	1367	7	0.571	Promoter(<=1kb)	0	C4orf50	389197
611358	4p	6300792	6302360	1569	8	0.875	Exon(exon8of8)	6021	WFS1	7466
611358	4p	8227004	8228508	1505	8	0.125	Promoter(<=1kb)	-24	SH3TC1	54436
611358	4p	10443803	10446224	2422	6	0.333	Exon(exon3of3)	10952	ZNF518B	85460
611358	4q	121036404	121037542	1139	6	0.333	Promoter(1-2kb)	1442	NDNF	79625
611358	4q	185458217	185460011	1795	8	0.625	Promoter(<=1kb)	0	CCDC110	256309
611358	4q	186617176	186621582	4407	10	0.4	Exon(exon10of27)	-7106	FAT1	2195

611358	5q	79731782	79734523	2742	13	0.308	Exon(exon2of13)	-3619	CMYA5	202333
611358	5q	83537326	83539905	2580	6	0.333	Promoter(1-2kb)	1712	VCAN	1462
611358	5q	140848579	140850786	2208	6	0.5	Promoter(<=1kb)	807	PCDHA9	9752
611358	5q	141955356	141957660	2305	6	0.667	Promoter(<=1kb)	-668	RNF14	9604
611358	5q	148826877	148828070	1194	7	0.857	Promoter(1-2kb)	1632	ADRB2	154
611358	5q	151565922	151568158	2237	10	0.8	Promoter(<=1kb)	786	FAT2	2196
611358	6p	46858771	46859502	732	8	0.5	Exon(exon17of21)	3802	ADGRF5	221395
611358	6q	159233455	159234370	916	10	0.5	Exon(exon11of23)	15158	FNDC1	84624
611358	7p	12369637	12370736	1100	6	0.667	3'UTR	-13307	VWDE	221806
611358	7q	100958744	100960873	2130	55	0.436	Promoter(<=1kb)	779	MUC3A	4584
611358	7q	100991195	100992398	1204	7	0.571	Exon(exon5of15)	-20656	MUC12	10071
611358	7q	149818015	149819792	1778	6	0.667	Promoter(2-3kb)	-2352	SSPO	23145
611358	7q	149824094	149826495	2402	6	0.667	Promoter(<=1kb)	0	SSPO	23145
611358	8p	10607245	10608432	1188	8	0.5	Exon(exon4of4)	46711	RP1L1	94137
611358	8p	12132686	12133940	1255	6	0.667	Promoter(<=1kb)	498	USP17L7	392197
611358	8p	13021128	13022030	903	7	0.143	Exon(exon5of5)	9115	TRMT9B	57604
611358	8q	143916360	143919209	2850	7	0.143	Exon(exon32of32)	20381	PLEC	5339
611358	9p	34723747	34726527	2781	10	0.6	Promoter(2-3kb)	2961	FAM205A	259308
611358	9q	76703555	76707804	4250	13	0.462	Promoter(<=1kb)	0	PCA3	50652
611358	9q	76709263	76710843	1581	8	0.5	Promoter(<=1kb)	0	PRUNE2	158471
611358	9q	122553263	122554071	809	7	0.429	Promoter(<=1kb)	93	OR1N2	138882
611358	9q	135484803	135487213	2411	8	0.25	Promoter(1-2kb)	1440	PPP1R26	9858
611358	10q	46549378	46550723	1346	25	0.64	Exon(exon3of3)	4807	GPRIN2	9721
611358	10q	49323169	49326817	3649	11	0.545	Exon(exon3of3)	23895	C10orf71	118461
611358	10q	128102594	128105201	2608	6	0.667	Promoter(<=1kb)	0	MKI67	4288
611358	11p	244106	244197	92	8	0.5	Promoter(<=1kb)	-232	PSMD13	5719
611358	11p	5177978	5178478	501	6	0.167	Promoter(<=1kb)	186	OR52Z1	283110
611358	11p	5323451	5324256	806	6	0.5	Promoter(<=1kb)	41	OR51B2	79345
611358	11p	5422212	5423123	912	11	0.636	Promoter(<=1kb)	101	OR51Q1	390061
611358	11p	5515185	5516015	831	6	0.333	Promoter(<=1kb)	684	UBQLNL	143630
611358	11p	5581045	5581738	694	8	0.375	Promoter(<=1kb)	168	OR52B6	340980
611358	11p	5841302	5841883	582	9	0.333	Promoter(<=1kb)	14	OR52E6	390078
611358	11p	11352040	11352736	697	8	0.25	Promoter(<=1kb)	514	CSNK2A3	283106
611358	11p	12293639	12294368	730	6	0.833	Exon(exon29of35)	6739	MICALCL	84953
611358	11q	58214757	58215424	668	8	0.375	Promoter(<=1kb)	12	OR1S1	219959
611358	11q	66560202	66562261	2060	6	0.333	Exon(exon14of21)	3873	CTSF	8722
611358	11q	82732630	82733184	555	7	0.857	Promoter(<=1kb)	680	FAM181B	220382
611358	11q	85724687	85725825	1139	6	0.5	Promoter(<=1kb)	0	SYTL2	54843
611358	11q	123906790	123907324	535	6	0.667	Promoter(<=1kb)	644	OR8D4	338662
611358	11q	124038366	124038988	623	7	1	Promoter(<=1kb)	13	OR10G7	390265
611358	11q	124264847	124265676	830	6	0.833	Promoter(<=1kb)	20	OR8G5	219865
611358	11q	124382526	124383285	760	6	0.5	Promoter(<=1kb)	58	OR8B2	26595
611358	11q	130914501	130915409	909	10	0.7	Promoter(1-2kb)	1035	SNX19	399979
611358	12p	4626568	4628152	1585	9	0.333	Exon(exon5of6)	14051	DYRK4	8798
611358	12p	6453119	6453670	552	7	0.714	Promoter(<=1kb)	633	TAPBPL	55080
611358	12p	8222174	8223514	1341	6	0.667	Exon(exon5of6)	4073	FAM90A1	55138
611358	12q	52316096	52317765	1670	6	0.667	Exon(exon4of9)	3633	KRT83	3889
611358	13q	24434450	24435347	898	7	0.571	Exon(exon31of34)	19787	PARP4	143
611358	13q	25096713	25097182	470	6	0.333	Promoter(<=1kb)	845	PABPC3	5042
611358	13q	102732474	102733933	1460	6	0.333	Exon(exon4of4)	25139	CCDC168	643677
611358	14q	19975713	19976448	736	9	0.444	Promoter(<=1kb)	269	OR4K15	81127
611358	14q	20060048	20060884	837	8	0.625	Promoter(<=1kb)	3	OR4L1	122742
611358	14q	20640750	20641567	818	7	0.571	Promoter(<=1kb)	124	OR6S1	341799
611358	14q	21634177	21634589	413	6	0.833	Promoter(<=1kb)	351	OR10G2	26534
611358	14q	22633879	22634450	572	9	0.333	Exon(exon2of2)	32212	ABHD4	63874
611358	14q	70457532	70458948	1417	11	0.364	Exon(exon2of2)	5358	ADAM21	8747
611358	14q	94587512	94587839	328	6	0.5	Exon(exon2of2)	-4219	SERPINA3	12
611358	15q	23440370	23442067	1698	9	0.667	5'UTR	5167	GOLGA6L2	283685
611358	15q	59206980	59208056	1077	7	0.714	Promoter(<=1kb)	137	LDHAL6B	92483
611358	16p	1486371	1488463	2093	8	0.75	Promoter(<=1kb)	4	PTX4	390667
611358	16q	88714632	88717113	2482	7	0.429	Promoter(<=1kb)	0	MIR4722	100616167
611358	16q	89226863	89228289	1427	7	0.571	Promoter(2-3kb)	2229	ZNF778	197320
611358	17p	21300581	21300954	374	10	0.7	3'UTR	9112	MAP2K3	5606
611358	17p	21415458	21416533	1076	13	0.846	Exon(exon3of3)	10322	KCNJ12	3768
611358	17q	76291123	76294016	2894	11	0.455	Promoter(<=1kb)	0	QRICH2	84074
611358	17q	81645135	81645607	473	7	0.286	Promoter(2-3kb)	2722	TSPAN10	83882
611358	18p	11609728	11610350	623	8	0.75	Promoter(<=1kb)	132	SLC35G4	646000
611358	18q	58535186	58538030	2845	19	0.526	Promoter(<=1kb)	0	ALPK2	115701
611358	19p	4510548	4513547	3000	19	0.474	Exon(exon3of6)	4157	PLIN4	729359
611358	19p	5455600	5456439	840	7	0.571	Promoter(<=1kb)	183	ZNRF4	148066
611358	19p	8333830	8334965	1136	6	0.667	Promoter(<=1kb)	0	KANK3	256949
611358	19p	8937644	8939234	1591	6	0.667	Exon(exon5of84)	-41554	MUC16	94025
611358	19p	8946313	8951868	5556	19	0.632	Exon(exon3of84)	29474	MUC16	94025
611358	19p	8959116	8962299	3184	11	0.727	Exon(exon3of84)	19043	MUC16	94025
611358	19p	8971838	8978096	6259	15	0.533	Exon(exon1of84)	3246	MUC16	94025
611358	19p	17281820	17284246	2427	8	0.5	Promoter(<=1kb)	0	ANKLE1	126549
611358	19p	18264798	18267409	2612	12	0.583	5'UTR	7002	IQCN	80726

611358	19p	21971930	21974500	2571	10	0.7	Exon(exon4of4)	14408	ZNF208	7757
611358	19p	23743906	23745300	1395	6	0.333	Exon(exon4of4)	13537	ZNF681	148213
611358	19q	36996730	36997597	868	10	0.6	Exon(exon10of10)	5677	ZNF568	374900
611358	19q	39877222	39877880	659	7	0.429	Exon(exon20of28)	9412	FCGBP	8857
611358	19q	43913423	43914878	1456	8	0.5	Exon(exon10of10)	4861	ZNF45	7596
611358	19q	44106512	44108078	1567	8	0.125	Exon(exon6of6)	-4103	ZNF225	7768
611358	19q	52437918	52439242	1325	7	0.429	Exon(exon4of4)	6504	ZNF534	147658
611358	19q	58368293	58368875	583	7	0.429	Exon(exon3of3)	-5445	ZNF497	162968
611358	20p	5922421	5923394	974	7	0.571	Exon(exon4of5)	6923	CHGB	1114
611358	20q	63561666	63565531	3866	11	0.636	Promoter(<=1kb)	-61	HELZ2	85441
611358	21q	26843740	26844859	1120	6	0.667	Promoter(<=1kb)	0	ADAMTS1	9510
611358	21q	36070334	36072675	2342	6	0.833	Promoter(<=1kb)	299	CBR1	873
611358	21q	44591587	44592404	818	6	0.167	Promoter(<=1kb)	101	KRTAP10-6	386674
611358	21q	44600627	44601692	1066	10	0.7	Promoter(<=1kb)	30	KRTAP10-7	386675
611358	21q	44637474	44638143	670	10	0.4	Promoter(<=1kb)	118	KRTAP10-10	353333
611358	22q	22352950	22353365	416	13	0.462	Exon(exon1of2)	30478	BMS1P20	96610
611358	22q	36191154	36191906	753	7	0.571	3'UTR	9971	APOL4	80832
611358	22q	49884187	49884994	808	6	0.167	Exon(exon2of2)	22924	ALG12	79087
611358	23p	3320126	3323750	3625	9	0.556	Exon(exon5of7)	22902	MXRA5	25878
611358	23p	8170039	8170141	103	6	0.5	Promoter(1-2kb)	1126	VCX2	51480
611358	23p	35802148	35803010	863	7	0.571	5'UTR	3357	MAGEB16	139604
611358	23q	136874183	136874347	165	7	0.714	Promoter(<=1kb)	-201	RBMX	27316
611773	1p	12029085	12030097	1013	6	0.833	Promoter(<=1kb)	0	MIR6729	102466982
611773	1p	12847526	12847995	470	7	0.429	Promoter(<=1kb)	730	HNRNPCL1	343069
611773	1p	12859036	12860079	1044	6	0.333	Promoter(1-2kb)	1950	PRAMEF2	65122
611773	1p	13370686	13371119	434	9	0.444	Promoter(<=1kb)	781	PRAMEF19	645414
611773	1p	18481403	18483159	1757	7	0.714	Promoter(<=1kb)	421	KLHDC7A	127707
611773	1p	23874604	23875430	827	9	0.556	Exon(exon2of2)	-6310	FUCA1	2517
611773	1p	40067594	40067675	82	6	0	Promoter(<=1kb)	324	CAP1	10487
611773	1p	88983230	88983751	522	7	0.429	Exon(exon3of3)	4604	KYAT3	56267
611773	1p	89186388	89186419	32	9	0.556	Promoter(<=1kb)	107	GBP4	115361
611773	1q	152303673	152304920	1248	8	0.5	Exon(exon3of3)	-7656	FLG-AS1	339400
611773	1q	152306380	152313891	7512	32	0.594	Promoter(<=1kb)	0	FLG-AS1	339400
611773	1q	169542317	169542882	566	6	0.167	Exon(exon13of25)	-26572	F5	2153
611773	1q	197101312	197101771	460	6	0.5	Exon(exon18of28)	33373	ASPM	259266
611773	1q	201206099	201209738	3640	10	0.6	Promoter(1-2kb)	1017	IGFN1	91156
611773	1q	222628664	222629929	1266	6	0.333	Promoter(<=1kb)	190	MIA3	375056
611773	1q	226735563	226737239	1677	8	0.875	Promoter(<=1kb)	219	ITPKB	3707
611773	1q	232805207	232806505	1299	6	0.333	Promoter(<=1kb)	315	MAP10	54627
611773	1q	247841312	247841582	271	6	0.833	Promoter(<=1kb)	314	OR11L1	391189
611773	1q	247949443	247949738	296	9	0.333	Promoter(<=1kb)	585	OR2L8	391190
611773	2p	29002636	29003646	1011	6	0.333	Exon(exon5of20)	-10821	TOGARAM2	165186
611773	2p	48580657	48582454	1798	7	0.571	Promoter(<=1kb)	0	STON1	11037
611773	2q	132783061	132785001	1941	9	0.444	Promoter(1-2kb)	-1038	NCKAP5	344148
611773	2q	185790999	185794632	3634	8	0.875	Promoter(<=1kb)	0	FSIP2	401024
611773	2q	185805377	185808170	2794	6	0.333	Promoter(<=1kb)	0	FSIP2	401024
611773	2q	217847583	217848559	977	6	0.833	Exon(exon19of33)	-5423	TNS1	7145
611773	2q	238130271	238131546	1276	8	0.375	Promoter(1-2kb)	1323	ESPNL	339768
611773	3p	75736929	75739239	2311	21	0.667	Promoter(<=1kb)	0	MIR4273	100422955
611773	3q	98264413	98265098	686	7	0.571	Promoter(<=1kb)	128	OR5H6	79295
611773	3q	194359607	194360906	1300	9	0.889	Exon(exon2of2)	-8279	CPN2	1370
611773	4p	5988383	5989749	1367	8	0.625	Promoter(<=1kb)	0	C4orf50	389197
611773	4p	6300792	6302360	1569	7	0.857	Exon(exon8of8)	6021	WFS1	7466
611773	4p	8227004	8228508	1505	8	0.125	Promoter(<=1kb)	-24	SH3TC1	54436
611773	4p	38773164	38775552	2389	11	0.455	Promoter(<=1kb)	644	TLR10	81793
611773	4q	185458217	185460011	1795	8	0.625	Promoter(<=1kb)	0	CCDC110	256309
611773	4q	186706638	186709751	3114	7	0.714	Exon(exon2of27)	14082	FAT1	2195
611773	5q	140848579	140850786	2208	6	0.5	Promoter(<=1kb)	807	PCDHA9	9752
611773	5q	141174000	141175025	1026	6	0.833	Promoter(1-2kb)	1356	PCDHB7	56129
611773	5q	141187690	141189425	1736	6	0.5	Promoter(<=1kb)	529	PCDHB9	56127
611773	5q	141955676	141957660	1985	6	0.667	Promoter(<=1kb)	-668	RNF14	9604
611773	5q	148826877	148828070	1194	6	1	Promoter(1-2kb)	1632	ADRB2	154
611773	5q	151565922	151568158	2237	9	0.778	Promoter(<=1kb)	786	FAT2	2196
611773	6p	46858771	46859389	619	6	0.5	Exon(exon17of21)	3915	ADGRF5	221395
611773	6q	159231899	159234370	2472	12	0.583	Exon(exon11of23)	13602	FNDC1	84624
611773	7p	12369637	12370736	1100	6	0.667	3'UTR	-13307	VWDE	221806
611773	7p	53035678	53036385	708	7	1	Promoter(<=1kb)	45	POM121L12	285877
611773	7q	100958721	100960873	2153	58	0.448	Promoter(<=1kb)	756	MUC3A	4584
611773	7q	101034305	101038481	4177	24	0.542	Exon(exon3of12)	-5230	MUC17	140453
611773	7q	149818015	149819792	1778	6	0.667	Promoter(2-3kb)	-2352	SSPO	23145
611773	7q	149824094	149826495	2402	6	0.667	Promoter(<=1kb)	0	SSPO	23145
611773	8p	10607375	10608261	887	6	0.5	Exon(exon4of4)	46882	RP1L1	94137
611773	8p	13021128	13022030	903	8	0.125	Exon(exon5of5)	9115	TRMT9B	57604
611773	8q	123651655	123652634	980	7	0.571	Promoter(<=1kb)	316	KLHL38	340359
611773	8q	138151949	138153046	1098	6	0.667	Promoter(<=1kb)	0	FAM135B	51059
611773	9q	76705179	76707804	2626	8	0.5	Promoter(<=1kb)	121	PCA3	50652
611773	9q	76709263	76710843	1581	8	0.5	Promoter(<=1kb)	0	PRUNE2	158471

611773	9q	104694482	104695444	963	6	0.5	Promoter(<=1kb)	0	OR13D1	286365
611773	9q	122553263	122554071	809	7	0.429	Promoter(<=1kb)	93	OR1N2	138882
611773	9q	122628595	122629130	536	6	0.333	Promoter(<=1kb)	443	OR1B1	347169
611773	9q	133255635	133256205	571	7	1	3'UTR	19009	ABO	28
611773	9q	135484803	135487213	2411	8	0.25	Promoter(1-2kb)	1440	PPP1R26	9858
611773	10q	46549378	46550723	1346	25	0.64	Exon(exon3of3)	4807	GPRIN2	9721
611773	10q	89737450	89738561	1112	6	0	Exon(exon20of33)	13874	KIF20B	9585
611773	10q	128103129	128104752	1624	9	0.444	Promoter(<=1kb)	-1	MKI67	4288
611773	11p	244106	244197	92	8	0.5	Promoter(<=1kb)	-232	PSMD13	5719
611773	11p	1241677	1243593	1917	8	0.5	Exon(exon31of49)	6083	MUC5B-AS1	112577518
611773	11p	1249617	1251524	1908	7	0.714	Promoter(<=1kb)	0	MUC5B-AS1	112577518
611773	11p	5046754	5047432	679	7	0.571	Promoter(<=1kb)	228	OR52J3	119679
611773	11p	5177978	5178478	501	6	0.167	Promoter(<=1kb)	186	OR52Z1	283110
611773	11p	5389704	5390350	647	7	0.429	Promoter(<=1kb)	327	OR51M1	390059
611773	11p	5422212	5423123	912	11	0.636	Promoter(<=1kb)	101	OR51Q1	390061
611773	11p	5544676	5545259	584	6	0.5	Promoter(<=1kb)	290	OR52H1	390067
611773	11p	5581045	5581738	694	8	0.375	Promoter(<=1kb)	168	OR52B6	340980
611773	11p	5841302	5841883	582	9	0.333	Promoter(<=1kb)	14	OR52E6	390078
611773	11p	11352040	11352736	697	8	0.25	Promoter(<=1kb)	514	CSNK2A3	283106
611773	11p	12293639	12294538	900	6	0.667	Exon(exon29of35)	6739	MICALCL	84953
611773	11q	58214757	58215722	966	7	0.286	Promoter(<=1kb)	12	OR1S1	219959
611773	11q	64315818	64315856	39	6	0.833	Promoter(1-2kb)	1485	TRMT112	51504
611773	11q	66560202	66562261	2060	6	0.333	Exon(exon14of21)	3873	CTSF	8722
611773	11q	82732630	82733184	555	6	0.833	Promoter(<=1kb)	680	FAM181B	220382
611773	11q	85724687	85725825	1139	6	0.5	Promoter(<=1kb)	0	SYTL2	54843
611773	11q	123906790	123907324	535	6	0.667	Promoter(<=1kb)	644	OR8D4	338662
611773	11q	124382526	124383285	760	6	0.5	Promoter(<=1kb)	58	OR8B2	26595
611773	12p	4626568	4628549	1982	11	0.455	Exon(exon5of6)	14051	DYRK4	8798
611773	13q	24446725	24447185	461	6	0.333	Exon(exon26of34)	7949	PARP4	143
611773	13q	25096675	25097231	557	14	0.5	Promoter(<=1kb)	807	PABPC3	5042
611773	13q	102732424	102733933	1510	7	0.429	Exon(exon4of4)	25139	CCDC168	643677
611773	14q	19975713	19976448	736	9	0.444	Promoter(<=1kb)	269	OR4K15	81127
611773	14q	20060048	20060884	837	8	0.625	Promoter(<=1kb)	3	OR4L1	122742
611773	14q	20640750	20641567	818	6	0.5	Promoter(<=1kb)	124	OR6S1	341799
611773	14q	21634177	21634589	413	6	0.833	Promoter(<=1kb)	351	OR10G2	26534
611773	14q	70457532	70459101	1570	11	0.273	Exon(exon2of2)	5358	ADAM21	8747
611773	14q	104939262	104942618	3357	11	0.273	5'UTR	7102	PLD4	122618
611773	14q	104943622	104945444	1823	7	0.714	Exon(exon6of6)	9958	AHNAK2	113146
611773	14q	104947453	104951938	4486	22	0.591	Exon(exon6of6)	3464	AHNAK2	113146
611773	15q	23440370	23442067	1698	9	0.667	5'UTR	5167	GOLGA6L2	283685
611773	15q	40621642	40623696	2055	6	0.333	Promoter(<=1kb)	0	KNL1	57082
611773	15q	78766033	78766626	594	6	0.167	Promoter(<=1kb)	-920	ADAMTS7	11173
611773	15q	85579423	85582073	2651	15	0.667	Promoter(<=1kb)	-837	AKAP13	11214
611773	15q	99129423	99132517	3095	6	0.333	Exon(exon4of5)	7225	TTC23	64927
611773	16p	1256345	1256985	641	14	0.714	Promoter(<=1kb)	276	TPSD1	23430
611773	16p	1486371	1488463	2093	9	0.778	Promoter(<=1kb)	4	PTX4	390667
611773	16p	4207130	4208004	875	6	0.5	Exon(exon2of7)	31739	SRL	6345
611773	16q	74391401	74391897	497	11	0.818	Exon(exon7of7)	13523	NPIPB15	440348
611773	16q	88712207	88714717	2511	7	0.571	Promoter(<=1kb)	0	CTU2	348180
611773	16q	89100686	89101050	365	7	0.571	Promoter(<=1kb)	24	ACSF3	197322
611773	16q	89226863	89228289	1427	7	0.571	Promoter(2-3kb)	2229	ZNF778	197320
611773	17p	744946	746966	2021	6	1	3'UTR	5072	GEMIN4	50628
611773	17p	2299649	2300159	511	6	0.167	Exon(exon2of19)	-3224	SRR	63826
611773	17p	7482135	7482961	827	6	0.667	Promoter(<=1kb)	803	SLC35G6	643664
611773	17p	21300581	21300954	374	8	0.75	3'UTR	9112	MAP2K3	5606
611773	17p	21415458	21416548	1091	17	0.824	Exon(exon3of3)	10322	KCNJ12	3768
611773	17q	76293419	76294016	598	6	0.5	Promoter(2-3kb)	-2167	QRICH2	84074
611773	17q	81645135	81645595	461	7	0.429	Promoter(2-3kb)	2722	TSPAN10	83882
611773	18p	11609646	11610350	705	11	0.818	Promoter(<=1kb)	50	SLC35G4	646000
611773	18q	58535186	58538030	2845	19	0.579	Promoter(<=1kb)	0	ALPK2	115701
611773	19p	1004688	1005532	845	6	0.5	Exon(exon3of9)	4269	GRIN3B	116444
611773	19p	1036457	1036914	458	6	0.5	Exon(exon6of7)	-3187	ABCA7	10347
611773	19p	4510548	4513547	3000	20	0.45	Exon(exon3of6)	4157	PLIN4	729359
611773	19p	5455600	5456439	840	7	0.571	Promoter(<=1kb)	183	ZNRF4	148066
611773	19p	8333830	8335276	1447	8	0.625	Promoter(<=1kb)	0	KANK3	256949
611773	19p	8937644	8939234	1591	6	0.667	Exon(exon5of84)	-41554	MUC16	94025
611773	19p	8946313	8951868	5556	19	0.632	Exon(exon3of84)	29474	MUC16	94025
611773	19p	8959116	8962299	3184	11	0.727	Exon(exon3of84)	19043	MUC16	94025
611773	19p	12430400	12431840	1441	7	0.571	3'UTR	9181	ZNF443	10224
611773	19p	17281820	17284246	2427	9	0.556	Promoter(<=1kb)	0	ANKLE1	126549
611773	19p	18264753	18267409	2657	8	0.5	5'UTR	7002	IQCN	80726
611773	19p	21971930	21974500	2571	7	0.714	Exon(exon4of4)	14408	ZNF208	7757
611773	19q	39877222	39877880	659	6	0.5	Exon(exon20of28)	9412	FCGBP	8857
611773	19q	39886005	39886439	435	7	0.571	Promoter(<=1kb)	853	FCGBP	8857
611773	19q	43913423	43914878	1456	8	0.5	Exon(exon10of10)	4861	ZNF45	7596
611773	19q	44106512	44108078	1567	7	0	Exon(exon6of6)	-4103	ZNF225	7768
611773	19q	44327836	44329698	1863	6	0.5	Exon(exon4of4)	-22790	ZNF235	9310

611773	19q	58368293	58368875	583	7	0.429	Exon(exon3of3)	-5445	ZNF497	162968
611773	20p	5922421	5923394	974	6	0.5	Exon(exon4of5)	6923	CHGB	1114
611773	20p	20052354	20052736	383	6	0.167	Promoter(<=1kb)	0	CFAP61	26074
611773	20q	63561666	63565531	3866	12	0.667	Promoter(<=1kb)	-61	HELZ2	85441
611773	21q	44550835	44551416	582	6	0.833	Promoter(<=1kb)	89	KRTAP10-2	386679
611773	21q	44600627	44601692	1066	8	0.875	Promoter(<=1kb)	30	KRTAP10-7	386675
611773	21q	44637474	44638041	568	11	0.455	Promoter(<=1kb)	118	KRTAP10-10	353333
611773	22q	22352950	22353348	399	14	0.571	Exon(exon1of2)	30478	BMS1P20	96610
617572	1p	12847526	12847995	470	7	0.429	Promoter(<=1kb)	730	HNRNPCL1	343069
617572	1p	12859036	12860079	1044	6	0.333	Promoter(1-2kb)	1950	PRAMEF2	65122
617572	1p	13370686	13370989	304	7	0.571	Promoter(<=1kb)	911	PRAMEF19	645414
617572	1p	16048038	16049824	1787	6	0.5	Promoter(<=1kb)	0	CLCNKB	1188
617572	1p	18481403	18482217	815	7	0.714	Promoter(<=1kb)	421	KLHDC7A	127707
617572	1p	23874604	23875430	827	8	0.5	Exon(exon2of2)	-6310	FUCA1	2517
617572	1p	40067594	40067675	82	6	0	Promoter(<=1kb)	324	CAP1	10487
617572	1q	152303673	152305350	1678	10	0.4	Exon(exon3of3)	-7226	FLG-AS1	339400
617572	1q	152306380	152313891	7512	30	0.567	Promoter(<=1kb)	0	FLG-AS1	339400
617572	1q	158765805	158766655	851	6	0.5	Promoter(<=1kb)	47	OR6N1	128372
617572	1q	201206099	201209738	3640	11	0.545	Promoter(1-2kb)	1017	IGFN1	91156
617572	1q	214640144	214642954	2811	12	0.5	Exon(exon12of20)	-5013	CENPF	1063
617572	1q	214644872	214647181	2310	10	0.4	Promoter(<=1kb)	-786	CENPF	1063
617572	1q	223393517	223394466	950	6	0.667	Promoter(<=1kb)	102	CCDC185	164127
617572	1q	228315976	228318049	2074	7	0.571	Exon(exon50of81)	6492	OBSCN	84033
617572	1q	232805117	232806800	1684	7	0.429	Promoter(<=1kb)	225	MAP10	54627
617572	1q	240207775	240208254	480	9	0.667	Exon(exon5of18)	-27517	FMN2	56776
617572	1q	247841312	247841582	271	6	0.833	Promoter(<=1kb)	314	OR11L1	391189
617572	1q	247949325	247949738	414	10	0.3	Promoter(<=1kb)	467	OR2L8	391190
617572	2p	48580657	48582454	1798	7	0.571	Promoter(<=1kb)	0	STON1	11037
617572	2q	95944651	95945175	525	7	0.571	Promoter(<=1kb)	0	ANKRD36C	400986
617572	2q	178739433	178741811	2379	6	0.5	Exon(exon45of191)	26014	TTN	7273
617572	2q	219271337	219271649	313	6	0.667	Exon(exon4of4)	6227	TUBA4A	7277
617572	3p	31989532	31990905	1374	6	0.333	Exon(exon2of2)	7761	ZNF860	344787
617572	3p	75736880	75739231	2352	42	0.595	Promoter(<=1kb)	0	MIR4273	100422955
617572	3q	194341097	194342571	1475	7	0.714	Exon(exon2of2)	8747	CPN2	1370
617572	4p	5988383	5989749	1367	7	0.571	Promoter(<=1kb)	0	C4orf50	389197
617572	4p	6300792	6302360	1569	7	0.857	Exon(exon8of8)	6021	WFS1	7466
617572	4q	186617176	186621582	4407	10	0.4	Exon(exon10of27)	-7106	FAT1	2195
617572	5q	140848579	140850786	2208	6	0.5	Promoter(<=1kb)	807	PCDHA9	9752
617572	5q	141174000	141175025	1026	6	0.833	Promoter(1-2kb)	1356	PCDHB7	56129
617572	5q	151565922	151568455	2534	10	0.8	Promoter(<=1kb)	489	FAT2	2196
617572	6p	42745312	42746041	730	6	0.667	Promoter(<=1kb)	62	TBCC	6903
617572	6p	46858771	46859502	732	8	0.5	Exon(exon17of21)	3802	ADGRF5	221395
617572	6q	64591274	64591961	688	10	0.5	Exon(exon26of43)	121374	EYS	346007
617572	6q	149888581	149889987	1407	6	0.667	Promoter(<=1kb)	-116	RAET1E-AS1	100652739
617572	6q	159231899	159234370	2472	12	0.583	Exon(exon11of23)	13602	FNDC1	84624
617572	7p	53035678	53036385	708	6	1	Promoter(<=1kb)	45	POM121L12	285877
617572	7q	100958721	100960873	2153	59	0.441	Promoter(<=1kb)	756	MUC3A	4584
617572	7q	100991195	100993102	1908	8	0.625	Exon(exon5of15)	-19952	MUC12	10071
617572	8p	8376561	8377198	638	6	1	Exon(exon2of5)	4549	PRAG1	157285
617572	8p	10606895	10608432	1538	6	0.5	Exon(exon4of4)	46711	RP1L1	94137
617572	8p	10609614	10612307	2694	10	0.9	Exon(exon4of4)	42836	RP1L1	94137
617572	8p	13021128	13022030	903	8	0.25	Exon(exon5of5)	9115	TRMT9B	57604
617572	9p	214679	215269	591	6	0.5	Promoter(<=1kb)	0	DOCK8	81704
617572	9q	76175241	76175266	26	6	1	Exon(exon14of14)	-13373	PCSK5	5125
617572	9q	76705724	76707804	2081	6	0.5	Promoter(<=1kb)	666	PCA3	50652
617572	9q	87886533	87888536	2004	7	0.571	Exon(exon4of4)	3656	SPATA31E1	286234
617572	9q	122553263	122554071	809	7	0.429	Promoter(<=1kb)	93	OR1N2	138882
617572	9q	122628595	122629398	804	6	0.333	Promoter(<=1kb)	175	OR1B1	347169
617572	9q	133255722	133256264	543	6	0.667	Exon(exon7of7)	18950	ABO	28
617572	9q	135484803	135487213	2411	8	0.25	Promoter(1-2kb)	1440	PPP1R26	9858
617572	10q	46549378	46550723	1346	25	0.64	Exon(exon3of3)	4807	GPRIN2	9721
617572	10q	128103129	128104752	1624	8	0.5	Promoter(<=1kb)	-1	MKI67	4288
617572	10q	128106210	128108204	1995	6	1	Exon(exon12of14)	-3082	MKI67	4288
617572	11p	244106	244197	92	8	0.5	Promoter(<=1kb)	-232	PSMD13	5719
617572	11p	5177978	5178478	501	6	0.167	Promoter(<=1kb)	186	OR52Z1	283110
617572	11p	5323451	5324256	806	6	0.5	Promoter(<=1kb)	41	OR51B2	79345
617572	11p	5422212	5423123	912	12	0.667	Promoter(<=1kb)	101	OR51Q1	390061
617572	11p	5440761	5441472	712	6	0.833	Promoter(<=1kb)	42	OR51I1	390063
617572	11p	5515185	5516015	831	6	0.333	Promoter(<=1kb)	684	UBQLNL	143630
617572	11p	5581208	5581738	531	6	0.333	Promoter(<=1kb)	331	OR52B6	340980
617572	11p	5841302	5841883	582	9	0.333	Promoter(<=1kb)	14	OR52E6	390078
617572	11p	11352040	11352736	697	7	0.286	Promoter(<=1kb)	514	CSNK2A3	283106
617572	11p	12293639	12294368	730	7	0.857	Exon(exon29of35)	6739	MICALCL	84953
617572	11q	58214757	58215722	966	8	0.25	Promoter(<=1kb)	12	OR1S1	219959
617572	11q	66560202	66562261	2060	6	0.333	Exon(exon14of21)	3873	CTSF	8722
617572	11q	82732630	82733184	555	6	0.833	Promoter(<=1kb)	680	FAM181B	220382
617572	11q	85724687	85725825	1139	6	0.5	Promoter(<=1kb)	0	SYTL2	54843

617572	11q	123906790	123907324	535	6	0.667	Promoter(<=1kb)	644	OR8D4	338662
617572	11q	124038930	124038988	59	6	1	Promoter(<=1kb)	13	OR10G7	390265
617572	11q	124382526	124383285	760	6	0.5	Promoter(<=1kb)	58	OR8B2	26595
617572	11q	130914501	130915409	909	10	0.7	Promoter(1-2kb)	1035	SNX19	399979
617572	12p	4626571	4628549	1979	8	0.5	Exon(exon5of6)	14054	DYRK4	8798
617572	12q	52316096	52317765	1670	6	0.667	Exon(exon4of9)	3633	KRT83	3889
617572	13q	102732474	102733933	1460	6	0.333	Exon(exon4of4)	25139	CCDC168	643677
617572	14q	20060048	20060884	837	8	0.625	Promoter(<=1kb)	3	OR4L1	122742
617572	14q	20143999	20144604	606	8	0.5	Promoter(<=1kb)	263	OR4N5	390437
617572	14q	20640982	20641567	586	6	0.5	Promoter(<=1kb)	124	OR6S1	341799
617572	14q	21634177	21634589	413	6	0.833	Promoter(<=1kb)	351	OR10G2	26534
617572	14q	70457532	70458238	707	9	0.333	Exon(exon2of2)	5358	ADAM21	8747
617572	14q	94587512	94587839	328	6	0.5	Exon(exon2of2)	-4219	SERPINA3	12
617572	14q	103110058	103110438	381	6	0.667	Promoter(2-3kb)	2436	EXOC3L4	91828
617572	14q	104175275	104177810	2536	6	0.333	Exon(exon12of15)	36235	KIF26A	26153
617572	15q	23439979	23442067	2089	10	0.6	5'UTR	5167	GOLGA6L2	283685
617572	15q	85579423	85581800	2378	14	0.571	Promoter(1-2kb)	-1110	AKAP13	11214
617572	16p	669592	672548	2957	7	0.571	Promoter(<=1kb)	0	RHOT2	89941
617572	16p	1256354	1256985	632	11	0.636	Promoter(<=1kb)	285	TPSD1	23430
617572	16p	1486371	1488463	2093	8	0.75	Promoter(<=1kb)	4	PTX4	390667
617572	16p	1998795	2000191	1397	6	0.667	Promoter(<=1kb)	0	ZNF598	90850
617572	16p	4207059	4208004	946	7	0.571	Exon(exon2of7)	31739	SRL	6345
617572	16q	88428539	88431889	3351	11	0.364	Exon(exon3of3)	-21391	ZFPM1	161882
617572	16q	88714632	88717113	2482	7	0.429	Promoter(<=1kb)	0	MIR4722	100616167
617572	16q	89100686	89101050	365	8	0.625	Promoter(<=1kb)	24	ACSF3	197322
617572	16q	89226863	89228419	1557	9	0.556	Promoter(2-3kb)	2229	ZNF778	197320
617572	17p	744946	746966	2021	6	1	3'UTR	5072	GEMIN4	50628
617572	17p	5558351	5559959	1609	6	0.833	Exon(exon4of17)	23998	NLRP1	22861
617572	17p	10638198	10640146	1949	6	0.167	Exon(exon22of41)	-8169	MYH3	4621
617572	17p	21300581	21300954	374	8	0.75	3'UTR	9112	MAP2K3	5606
617572	17p	21415458	21416455	998	13	0.846	Exon(exon3of3)	10322	KCNJ12	3768
617572	17q	76293419	76294016	598	6	0.5	Promoter(2-3kb)	-2167	QRICH2	84074
617572	17q	81645135	81645417	283	6	0.333	Promoter(2-3kb)	2722	TSPAN10	83882
617572	18p	11609646	11610350	705	14	0.786	Promoter(<=1kb)	50	SLC35G4	646000
617572	18p	14542649	14543140	492	6	0.5	Promoter(<=1kb)	6	POTEC	388468
617572	18q	58535186	58537515	2330	9	0.333	Promoter(<=1kb)	0	ALPK2	115701
617572	19p	4510548	4513547	3000	18	0.389	Exon(exon3of6)	4157	PLIN4	729359
617572	19p	5455600	5456439	840	7	0.571	Promoter(<=1kb)	183	ZNRF4	148066
617572	19p	8948231	8950136	1906	6	0.667	Exon(exon3of84)	31206	MUC16	94025
617572	19p	8959518	8962066	2549	6	0.333	Exon(exon3of84)	19276	MUC16	94025
617572	19p	8964274	8967127	2854	19	0.632	Exon(exon3of84)	14215	MUC16	94025
617572	19p	9126210	9126946	737	9	0.556	Promoter(<=1kb)	4	OR7G3	390883
617572	19p	12430718	12432437	1720	8	0.375	3'UTR	8584	ZNF443	10224
617572	19p	15087213	15088040	828	10	0.3	Promoter(<=1kb)	233	OR1I1	126370
617572	19p	18264753	18267409	2657	8	0.5	5'UTR	7002	IQCN	80726
617572	19p	20624087	20625568	1482	6	0.167	3'UTR	35939	ZNF626	199777
617572	19p	21971930	21974500	2571	7	0.714	Exon(exon4of4)	14408	ZNF208	7757
617572	19p	23743906	23745300	1395	6	0.333	Exon(exon4of4)	13537	ZNF681	148213
617572	19q	39877222	39877880	659	6	0.5	Exon(exon20of28)	9412	FCGBP	8857
617572	19q	39886005	39886439	435	7	0.571	Promoter(<=1kb)	853	FCGBP	8857
617572	19q	43846955	43848536	1582	7	0.857	3'UTR	13450	ZNF283	284349
617572	19q	43913423	43914878	1456	8	0.5	Exon(exon10of10)	4861	ZNF45	7596
617572	19q	43966037	43967171	1135	6	0.167	Promoter(<=1kb)	-691	ZNF155	7711
617572	19q	44106512	44108078	1567	8	0.125	Exon(exon6of6)	-4103	ZNF225	7768
617572	19q	52437918	52439242	1325	7	0.429	Exon(exon4of4)	6504	ZNF534	147658
617572	19q	55358736	55359651	916	6	0.667	Promoter(<=1kb)	0	FAM71E2	284418
617572	19q	55481625	55483456	1832	7	0.429	Promoter(1-2kb)	-1732	NAT14	57106
617572	19q	55517821	55518642	822	9	1	Exon(exon14of14)	17661	SBK2	646643
617572	19q	55911888	55913077	1190	6	0.667	Exon(exon5of12)	19234	NLRP13	126204
617572	20p	5922421	5923643	1223	9	0.667	Exon(exon4of5)	6923	CHGB	1114
617572	20q	63349752	63350772	1021	6	0.5	3'UTR	3794	CHRNA4	1137
617572	20q	63561666	63565531	3866	12	0.667	Promoter(<=1kb)	-61	HELZ2	85441
617572	21q	44591417	44592404	988	6	0.5	Promoter(<=1kb)	101	KRTAP10-6	386674
617572	21q	44600627	44601692	1066	8	0.875	Promoter(<=1kb)	30	KRTAP10-7	386675
617572	22q	22352950	22353348	399	14	0.571	Exon(exon1of2)	30478	BMS1P20	96610
617572	22q	36191154	36191906	753	6	0.667	3'UTR	9971	APOL4	80832
617572	22q	49884187	49884994	808	6	0.333	Exon(exon2of2)	22924	ALG12	79087
617572	23p	8170039	8170141	103	6	0.5	Promoter(1-2kb)	1126	VCX2	51480
617572	23p	35802148	35803010	863	7	0.571	5'UTR	3357	MAGEB16	139604
618520	1p	11766028	11768307	2280	6	0.833	Promoter(<=1kb)	0	C1orf167	284498
618520	1p	11778784	11779941	1158	7	0.714	Promoter(<=1kb)	0	C1orf167-AS1	102724659
618520	1p	12847526	12847995	470	7	0.429	Promoter(<=1kb)	730	HNRNPCL1	343069
618520	1p	12859036	12860212	1177	13	0.538	Promoter(1-2kb)	1950	PRAMEF2	65122
618520	1p	13370686	13371119	434	8	0.375	Promoter(<=1kb)	781	PRAMEF19	645414
618520	1p	18481403	18482217	815	6	0.667	Promoter(<=1kb)	421	KLHDC7A	127707
618520	1p	23874604	23875430	827	8	0.5	Exon(exon2of2)	-6310	FUCA1	2517
618520	1p	40067594	40067675	82	6	0	Promoter(<=1kb)	324	CAP1	10487

618520	1q	152213286	152213316	31	6	0.5	Exon(exon3of3)	10877	HRNR	388697
618520	1q	152219233	152221375	2143	16	0.688	Promoter(2-3kb)	2818	HRNR	388697
618520	1q	156669844	156670886	1043	6	1	Exon(exon4of4)	6521	NES	10763
618520	1q	158765805	158766655	851	6	0.5	Promoter(<=1kb)	47	OR6N1	128372
618520	1q	201206099	201209738	3640	11	0.545	Promoter(1-2kb)	1017	IGFN1	91156
618520	1q	228315976	228318026	2051	6	0.5	Exon(exon50of81)	6492	OBSCN	84033
618520	1q	247841312	247841582	271	6	0.833	Promoter(<=1kb)	314	OR11L1	391189
618520	1q	247949460	247949738	279	8	0.25	Promoter(<=1kb)	602	OR2L8	391190
618520	1q	248060797	248061452	656	7	0.571	Exon(exon2of2)	22625	OR2L2	26246
618520	1q	248294677	248295458	782	9	0.667	Promoter(<=1kb)	142	OR2T12	127064
618520	2p	48580657	48582454	1798	7	0.571	Promoter(<=1kb)	0	STON1	11037
618520	2q	102351547	102351902	356	7	0.429	Exon(exon11of11)	-4027	IL18R1	8809
618520	2q	130193953	130194376	424	6	0.667	Exon(exon4of5)	4063	TUBA3E	112714
618520	2q	132783061	132784972	1912	7	0.429	Promoter(1-2kb)	-1038	NCKAP5	344148
618520	2q	184936178	184937636	1459	7	0.429	Exon(exon4of4)	69813	ZNF804A	91752
618520	2q	185789591	185794632	5042	11	0.727	Promoter(<=1kb)	0	FSIP2	401024
618520	2q	233713134	233713736	603	10	0.8	Promoter(<=1kb)	142	UGT1A5	54579
618520	2q	238130271	238131546	1276	7	0.286	Promoter(1-2kb)	1323	ESPNL	339768
618520	3p	31989532	31990905	1374	6	0.333	Exon(exon2of2)	7761	ZNF860	344787
618520	3p	75736880	75739243	2364	42	0.643	Promoter(<=1kb)	0	MIR4273	100422955
618520	3q	98264413	98265098	686	6	0.5	Promoter(<=1kb)	128	OR5H6	79295
618520	4p	5988383	5989749	1367	7	0.571	Promoter(<=1kb)	0	C4orf50	389197
618520	4p	6300792	6302360	1569	6	0.833	Exon(exon8of8)	6021	WFS1	7466
618520	4p	8227004	8228508	1505	8	0.125	Promoter(<=1kb)	-24	SH3TC1	54436
618520	4q	186619481	186621582	2102	6	0.333	Exon(exon10of27)	-9411	FAT1	2195
618520	4q	186706638	186709751	3114	7	0.714	Exon(exon2of27)	14082	FAT1	2195
618520	5q	79728956	79730716	1761	6	0.5	Exon(exon2of13)	-7426	CMYA5	202333
618520	5q	79731782	79734523	2742	13	0.308	Exon(exon2of13)	-3619	CMYA5	202333
618520	5q	141174000	141175025	1026	6	0.833	Promoter(1-2kb)	1356	PCDHB7	56129
618520	5q	141955356	141957660	2305	6	0.667	Promoter(<=1kb)	-668	RNF14	9604
618520	5q	148826877	148828070	1194	6	1	Promoter(1-2kb)	1632	ADRB2	154
618520	5q	151521550	151522069	520	6	0.667	Promoter(<=1kb)	79	MIR6499	102465246
618520	5q	151565922	151568158	2237	9	0.778	Promoter(<=1kb)	786	FAT2	2196
618520	6p	46858771	46859502	732	8	0.5	Exon(exon17of21)	3802	ADGRF5	221395
618520	6q	159231899	159234370	2472	12	0.583	Exon(exon11of23)	13602	FNDC1	84624
618520	7p	6330446	6330944	499	6	1	Exon(exon2of2)	7749	FAM220A	84792
618520	7p	12369637	12370736	1100	6	0.667	3'UTR	-13307	VWDE	221806
618520	7q	64991278	64992758	1481	6	0.667	Promoter(<=1kb)	-242	ZNF117	51351
618520	7q	100958721	100960873	2153	57	0.439	Promoter(<=1kb)	756	MUC3A	4584
618520	7q	100991195	100994358	3164	11	0.727	Exon(exon5of15)	-18696	MUC12	10071
618520	7q	100995547	100995858	312	6	0.667	Exon(exon5of15)	-17196	MUC12	10071
618520	7q	149818015	149819792	1778	6	0.667	Promoter(2-3kb)	-2352	SSPO	23145
618520	8p	8376561	8377198	638	7	1	Exon(exon2of5)	4549	PRAG1	157285
618520	8p	10607375	10612307	4933	16	0.625	Exon(exon4of4)	42836	RP1L1	94137
618520	8p	11331194	11332082	889	8	0.5	Promoter(<=1kb)	306	SLC35G5	83650
618520	8p	13021128	13022030	903	7	0.143	Exon(exon5of5)	9115	TRMT9B	57604
618520	8q	141218050	141219792	1743	6	0.667	5'UTR	8778	SLC45A4	57210
618520	9p	34723747	34725745	1999	8	0.625	Exon(exon4of4)	3743	FAM205A	259308
618520	9q	76703555	76706360	2806	8	0.5	Promoter(<=1kb)	0	PCA3	50652
618520	9q	104504315	104505023	709	6	0.5	Promoter(<=1kb)	52	OR13F1	138805
618520	9q	122553263	122554071	809	8	0.5	Promoter(<=1kb)	93	OR1N2	138882
618520	9q	122628595	122629130	536	6	0.333	Promoter(<=1kb)	443	OR1B1	347169
618520	9q	135484803	135487213	2411	8	0.25	Promoter(1-2kb)	1440	PPP1R26	9858
618520	10q	46549378	46550723	1346	26	0.654	Exon(exon3of3)	4807	GPRIN2	9721
618520	11p	244106	244197	92	8	0.5	Promoter(<=1kb)	-232	PSMD13	5719
618520	11p	1194354	1196902	2549	7	0.571	Exon(exon34of49)	-26164	MUC5B	727897
618520	11p	5046754	5047432	679	7	0.571	Promoter(<=1kb)	228	OR52J3	119679
618520	11p	5177978	5178478	501	6	0.167	Promoter(<=1kb)	186	OR52Z1	283110
618520	11p	5323542	5324256	715	6	0.5	Promoter(<=1kb)	41	OR51B2	79345
618520	11p	5389704	5390350	647	7	0.429	Promoter(<=1kb)	327	OR51M1	390059
618520	11p	5422212	5423123	912	10	0.7	Promoter(<=1kb)	101	OR51Q1	390061
618520	11p	5581045	5581738	694	8	0.375	Promoter(<=1kb)	168	OR52B6	340980
618520	11p	5841302	5841883	582	9	0.333	Promoter(<=1kb)	14	OR52E6	390078
618520	11p	5884818	5885061	244	7	0.429	Promoter(<=1kb)	547	OR52E4	390081
618520	11p	5986042	5986542	501	6	1	Promoter(<=1kb)	443	OR52L1	338751
618520	11p	11352040	11352736	697	8	0.25	Promoter(<=1kb)	514	CSNK2A3	283106
618520	11p	12293639	12294538	900	8	0.625	Exon(exon29of35)	6739	MICALCL	84953
618520	11q	58402523	58403265	743	8	0.5	Promoter(<=1kb)	144	OR5B3	441608
618520	11q	64116513	64118232	1720	7	0.714	Exon(exon2of2)	8702	MACROD1	28992
618520	11q	64315818	64315856	39	6	0.833	Promoter(1-2kb)	1485	TRMT112	51504
618520	11q	66560202	66562261	2060	6	0.333	Exon(exon14of21)	3873	CTSF	8722
618520	11q	82732630	82733184	555	6	0.833	Promoter(<=1kb)	680	FAM181B	220382
618520	11q	85724687	85725825	1139	6	0.5	Promoter(<=1kb)	0	SYTL2	54843
618520	11q	123906790	123907324	535	6	0.667	Promoter(<=1kb)	644	OR8D4	338662
618520	11q	123943088	123943782	695	7	0.571	Promoter(<=1kb)	91	OR6T1	219874
618520	11q	124015601	124016477	877	7	0.286	Promoter(<=1kb)	26	OR10G4	390264
618520	11q	124023038	124023849	812	7	0.429	Promoter(<=1kb)	25	OR10G9	219870

618520	11q	124038366	124038988	623	10	0.9	Promoter(<=1kb)	13	OR10G7	390265
618520	11q	124264847	124265676	830	6	0.833	Promoter(<=1kb)	20	OR8G5	219865
618520	11q	130914501	130915409	909	10	0.7	Promoter(1-2kb)	1035	SNX19	399979
618520	12p	4626571	4628549	1979	9	0.556	Exon(exon5of6)	14054	DYRK4	8798
618520	12p	6453119	6453670	552	6	0.667	Promoter(<=1kb)	633	TAPBPL	55080
618520	12q	52316096	52317765	1670	6	0.667	Exon(exon4of9)	3633	KRT83	3889
618520	13q	24434450	24435347	898	7	0.571	Exon(exon31of34)	19787	PARP4	143
618520	13q	25096713	25097182	470	10	0.4	Promoter(<=1kb)	845	PABPC3	5042
618520	13q	102732474	102733933	1460	6	0.333	Exon(exon4of4)	25139	CCDC168	643677
618520	14q	20060048	20060884	837	8	0.625	Promoter(<=1kb)	3	OR4L1	122742
618520	14q	21634177	21634589	413	6	0.833	Promoter(<=1kb)	351	OR10G2	26534
618520	14q	70457532	70458238	707	8	0.375	Exon(exon2of2)	5358	ADAM21	8747
618520	14q	104175275	104177810	2536	10	0.3	Exon(exon12of15)	36235	KIF26A	26153
618520	15q	23440081	23442067	1987	11	0.545	5'UTR	5167	GOLGA6L2	283685
618520	15q	59206980	59208023	1044	8	0.5	Promoter(<=1kb)	137	LDHAL6B	92483
618520	15q	78766033	78766632	600	7	0.286	Promoter(<=1kb)	-920	ADAMTS7	11173
618520	15q	85579423	85582073	2651	15	0.6	Promoter(<=1kb)	-837	AKAP13	11214
618520	15q	88857108	88859365	2258	6	0	Exon(exon12of18)	9865	ACAN	176
618520	16p	1256354	1256985	632	11	0.636	Promoter(<=1kb)	285	TPSD1	23430
618520	16p	1486371	1488463	2093	8	0.75	Promoter(<=1kb)	4	PTX4	390667
618520	16p	27362551	27363079	529	6	0.167	3'UTR	7203	IL4R	3566
618520	16q	89226863	89228390	1528	9	0.556	Promoter(2-3kb)	2229	ZNF778	197320
618520	17p	10638198	10641099	2902	7	0.286	Exon(exon19of41)	-8169	MYH3	4621
618520	17p	21300581	21300954	374	9	0.778	3'UTR	9112	MAP2K3	5606
618520	17p	21415458	21416455	998	19	0.895	Exon(exon3of3)	10322	KCNJ12	3768
618520	17q	81645135	81645417	283	6	0.333	Promoter(2-3kb)	2722	TSPAN10	83882
618520	18p	14542649	14543140	492	6	0.5	Promoter(<=1kb)	6	POTEC	388468
618520	18q	58535186	58537515	2330	9	0.333	Promoter(<=1kb)	0	ALPK2	115701
618520	19p	4510711	4513547	2837	19	0.474	Exon(exon3of6)	4157	PLIN4	729359
618520	19p	5455600	5456439	840	6	0.5	Promoter(<=1kb)	183	ZNRF4	148066
618520	19p	8937644	8939234	1591	6	0.833	Exon(exon5of84)	-41554	MUC16	94025
618520	19p	8945559	8951868	6310	19	0.684	Exon(exon3of84)	29474	MUC16	94025
618520	19p	8959116	8962299	3184	12	0.583	Exon(exon3of84)	19043	MUC16	94025
618520	19p	8963397	8967127	3731	21	0.667	Exon(exon3of84)	14215	MUC16	94025
618520	19p	8972751	8978096	5346	13	0.462	Exon(exon1of84)	3246	MUC16	94025
618520	19p	15087213	15088040	828	11	0.273	Promoter(<=1kb)	233	OR1I1	126370
618520	19p	17281820	17284246	2427	9	0.556	Promoter(<=1kb)	0	ANKLE1	126549
618520	19p	18264798	18267409	2612	12	0.583	5'UTR	7002	IQCN	80726
618520	19p	21971930	21974500	2571	8	0.75	Exon(exon4of4)	14408	ZNF208	7757
618520	19p	23743906	23745300	1395	6	0.333	Exon(exon4of4)	13537	ZNF681	148213
618520	19q	39877222	39877880	659	7	0.429	Exon(exon20of28)	9412	FCGBP	8857
618520	19q	39886005	39886439	435	6	0.667	Promoter(<=1kb)	853	FCGBP	8857
618520	19q	43913423	43914878	1456	8	0.5	Exon(exon10of10)	4861	ZNF45	7596
618520	19q	44106512	44108078	1567	8	0.125	Exon(exon6of6)	-4103	ZNF225	7768
618520	19q	52365769	52366744	976	7	0.857	Exon(exon6of6)	-3173	ZNF880	400713
618520	19q	52437918	52439242	1325	7	0.429	Exon(exon4of4)	6504	ZNF534	147658
618520	19q	55481625	55483456	1832	7	0.429	Promoter(1-2kb)	-1732	NAT14	57106
618520	19q	55517821	55518642	822	8	1	Exon(exon14of14)	17661	SBK2	646643
618520	19q	55912302	55913166	865	6	0.667	Exon(exon5of12)	19145	NLRP13	126204
618520	19q	58368293	58368875	583	7	0.429	Exon(exon3of3)	-5445	ZNF497	162968
618520	21q	26843740	26844859	1120	6	0.667	Promoter(<=1kb)	0	ADAMTS1	9510
618520	21q	44637474	44638041	568	9	0.333	Promoter(<=1kb)	118	KRTAP10-10	353333
618520	22q	22352950	22353348	399	14	0.571	Exon(exon1of2)	30478	BMS1P20	96610
618520	22q	35658986	35659117	132	6	0.833	Exon(exon3of3)	10540	APOL6	80830
618520	22q	36191154	36191906	753	7	0.571	3'UTR	9971	APOL4	80832
618520	22q	39100331	39102033	1703	6	0.833	Promoter(<=1kb)	52	APOBEC3H	164668
618520	23p	3320126	3323750	3625	9	0.556	Exon(exon5of7)	22902	MXRA5	25878
618520	23p	8170039	8170141	103	6	0.5	Promoter(1-2kb)	1126	VCX2	51480
618520	23p	35802148	35803010	863	7	0.571	5'UTR	3357	MAGEB16	139604
619096	1p	13369166	13369564	399	6	0.667	Promoter(2-3kb)	2336	PRAMEF19	645414
619096	1p	13370686	13371119	434	9	0.444	Promoter(<=1kb)	781	PRAMEF19	645414
619096	1p	23874604	23875430	827	8	0.5	Exon(exon2of2)	-6310	FUCA1	2517
619096	1p	26343953	26345408	1456	6	0.833	Exon(exon4of22)	-4607	CRYBG2	55057
619096	1p	40067594	40067675	82	6	0	Promoter(<=1kb)	324	CAP1	10487
619096	1p	89186388	89186419	32	9	0.556	Promoter(<=1kb)	107	GBP4	115361
619096	1q	152218469	152221375	2907	17	0.706	Promoter(2-3kb)	2818	HRNR	388697
619096	1q	152303673	152304920	1248	8	0.5	Exon(exon3of3)	-7656	FLG-AS1	339400
619096	1q	152306380	152313891	7512	30	0.6	Promoter(<=1kb)	0	FLG-AS1	339400
619096	1q	158765805	158766425	621	6	0.5	Promoter(<=1kb)	277	OR6N1	128372
619096	1q	223393517	223394466	950	6	0.667	Promoter(<=1kb)	102	CCDC185	164127
619096	1q	226735683	226737239	1557	7	0.857	Promoter(<=1kb)	219	ITPKB	3707
619096	1q	232805117	232806800	1684	6	0.333	Promoter(<=1kb)	225	MAP10	54627
619096	1q	247841312	247841582	271	6	0.833	Promoter(<=1kb)	314	OR11L1	391189
619096	1q	247895625	247896410	786	6	0.5	Promoter(<=1kb)	38	OR2W3	343171
619096	1q	247949325	247949738	414	6	0.167	Promoter(<=1kb)	467	OR2L8	391190
619096	2p	48580657	48582454	1798	7	0.571	Promoter(<=1kb)	0	STON1	11037
619096	2q	167246721	167248478	1758	8	0.75	Promoter(<=1kb)	-204	XIRP2	129446

619096	2q	184936178	184937636	1459	8	0.375	Exon(exon4of4)	69813	ZNF804A	91752
619096	2q	185789591	185794632	5042	11	0.727	Promoter(<=1kb)	0	FSIP2	401024
619096	2q	238130416	238131702	1287	10	0.7	Promoter(1-2kb)	1468	ESPNL	339768
619096	3p	31989532	31990905	1374	6	0.333	Exon(exon2of2)	7761	ZNF860	344787
619096	3p	75736929	75738859	1931	10	0.6	Promoter(<=1kb)	0	MIR4273	100422955
619096	3q	98169021	98169561	541	6	0.5	Exon(exon2of2)	19695	OR5H14	403273
619096	3q	98264413	98265098	686	6	0.5	Promoter(<=1kb)	128	OR5H6	79295
619096	4p	5988383	5989749	1367	7	0.571	Promoter(<=1kb)	0	C4orf50	389197
619096	4p	6300792	6302360	1569	9	0.778	Exon(exon8of8)	6021	WFS1	7466
619096	4p	8227004	8228508	1505	8	0.125	Promoter(<=1kb)	-24	SH3TC1	54436
619096	4q	99653038	99654557	1520	7	0.857	Promoter(<=1kb)	91	C4orf54	285556
619096	4q	154489498	154491312	1815	9	0.556	Promoter(<=1kb)	22	DCHS2	54798
619096	4q	186619481	186621601	2121	7	0.286	Exon(exon10of27)	-9411	FAT1	2195
619096	4q	186706638	186708616	1979	6	0.667	Exon(exon2of27)	15217	FAT1	2195
619096	5q	79728956	79730716	1761	7	0.286	Exon(exon2of13)	-7426	CMYA5	202333
619096	5q	79731782	79734523	2742	13	0.308	Exon(exon2of13)	-3619	CMYA5	202333
619096	5q	83537326	83539905	2580	7	0.286	Promoter(1-2kb)	1712	VCAN	1462
619096	5q	140802063	140803636	1574	6	0.333	Promoter(1-2kb)	1006	PCDHA3	56145
619096	5q	140848579	140850786	2208	6	0.5	Promoter(<=1kb)	807	PCDHA9	9752
619096	6p	1312843	1313745	903	6	0.5	Promoter(<=1kb)	745	FOXQ1	94234
619096	6p	42745312	42746041	730	6	0.667	Promoter(<=1kb)	62	TBCC	6903
619096	6p	46858771	46859502	732	8	0.5	Exon(exon17of21)	3802	ADGRF5	221395
619096	6q	64591274	64591961	688	10	0.5	Exon(exon26of43)	121374	EYS	346007
619096	6q	146434004	146434706	703	6	0.5	3'UTR	-109127	RAB32	10981
619096	6q	149888581	149890867	2287	7	0.714	Promoter(<=1kb)	0	RAET1E-AS1	100652739
619096	6q	159231899	159234370	2472	10	0.5	Exon(exon11of23)	13602	FNDC1	84624
619096	7p	38353718	38353991	274	8	0.625	Exon(exon2of2)	3699	TRG-AS1	100506776
619096	7p	53035678	53036385	708	6	1	Promoter(<=1kb)	45	POM121L12	285877
619096	7p	56021087	56021209	123	6	0.5	Exon(exon2of7)	12947	PSPH	5723
619096	7q	64991278	64992758	1481	6	0.667	Promoter(<=1kb)	-242	ZNF117	51351
619096	7q	100958721	100960873	2153	58	0.448	Promoter(<=1kb)	756	MUC3A	4584
619096	7q	100991195	100992398	1204	7	0.571	Exon(exon5of15)	-20656	MUC12	10071
619096	7q	100995575	100995872	298	6	0.667	Exon(exon5of15)	-17182	MUC12	10071
619096	7q	149818015	149819792	1778	6	0.667	Promoter(2-3kb)	-2352	SSPO	23145
619096	7q	149824094	149826695	2602	6	0.667	Promoter(<=1kb)	0	SSPO	23145
619096	8p	10607245	10608432	1188	8	0.5	Exon(exon4of4)	46711	RP1L1	94137
619096	8p	10609614	10610662	1049	9	0.556	Exon(exon4of4)	44481	RP1L1	94137
619096	8p	13021128	13022030	903	10	0.2	Exon(exon5of5)	9115	TRMT9B	57604
619096	8q	123651873	123652634	762	6	0.333	Promoter(<=1kb)	316	KLHL38	340359
619096	9p	34723747	34725745	1999	7	0.571	Exon(exon4of4)	3743	FAM205A	259308
619096	9q	76175237	76175266	30	7	0.857	Exon(exon14of14)	-13373	PCSK5	5125
619096	9q	76705179	76707804	2626	8	0.5	Promoter(<=1kb)	121	PCA3	50652
619096	9q	76709263	76710843	1581	8	0.5	Promoter(<=1kb)	0	PRUNE2	158471
619096	9q	104598545	104599361	817	7	0.429	Promoter(<=1kb)	52	OR13C5	138799
619096	9q	122553263	122554071	809	7	0.429	Promoter(<=1kb)	93	OR1N2	138882
619096	9q	133255635	133256264	630	8	1	3'UTR	18950	ABO	28
619096	9q	135484803	135487213	2411	9	0.333	Promoter(1-2kb)	1440	PPP1R26	9858
619096	10q	46549378	46550744	1367	27	0.63	5'UTR	4786	GPRIN2	9721
619096	10q	128103129	128106296	3168	13	0.615	Promoter(<=1kb)	-1	MKI67	4288
619096	11p	244106	244197	92	8	0.5	Promoter(<=1kb)	-232	PSMD13	5719
619096	11p	5177978	5178478	501	6	0.167	Promoter(<=1kb)	186	OR52Z1	283110
619096	11p	5323542	5324256	715	6	0.5	Promoter(<=1kb)	41	OR51B2	79345
619096	11p	5389704	5390350	647	7	0.429	Promoter(<=1kb)	327	OR51M1	390059
619096	11p	5422212	5423123	912	11	0.636	Promoter(<=1kb)	101	OR51Q1	390061
619096	11p	5581045	5581738	694	9	0.333	Promoter(<=1kb)	168	OR52B6	340980
619096	11p	5841302	5841883	582	9	0.333	Promoter(<=1kb)	14	OR52E6	390078
619096	11p	11352040	11352736	697	8	0.25	Promoter(<=1kb)	514	CSNK2A3	283106
619096	11p	12293639	12294368	730	6	0.833	Exon(exon29of35)	6739	MICALCL	84953
619096	11q	58214757	58215722	966	9	0.222	Promoter(<=1kb)	12	OR1S1	219959
619096	11q	66560202	66562261	2060	6	0.333	Exon(exon14of21)	3873	CTSF	8722
619096	11q	85724687	85725825	1139	6	0.5	Promoter(<=1kb)	0	SYTL2	54843
619096	11q	124015600	124016477	878	7	0.143	Promoter(<=1kb)	25	OR10G4	390264
619096	11q	124038366	124038988	623	7	1	Promoter(<=1kb)	13	OR10G7	390265
619096	11q	124264847	124265766	920	7	0.857	Promoter(<=1kb)	20	OR8G5	219865
619096	11q	130914501	130915409	909	10	0.7	Promoter(1-2kb)	1035	SNX19	399979
619096	12p	4626571	4628549	1979	9	0.444	Exon(exon5of6)	14054	DYRK4	8798
619096	12q	52316096	52317765	1670	6	0.667	Exon(exon4of9)	3633	KRT83	3889
619096	13q	24434450	24435347	898	7	0.571	Exon(exon31of34)	19787	PARP4	143
619096	13q	25096713	25097182	470	13	0.462	Promoter(<=1kb)	845	PABPC3	5042
619096	13q	102732474	102733933	1460	6	0.333	Exon(exon4of4)	25139	CCDC168	643677
619096	14q	21634177	21634589	413	6	0.833	Promoter(<=1kb)	351	OR10G2	26534
619096	14q	70457532	70458238	707	8	0.375	Exon(exon2of2)	5358	ADAM21	8747
619096	14q	94587512	94587839	328	6	0.5	Exon(exon2of2)	-4219	SERPINA3	12
619096	14q	104939262	104942618	3357	10	0.2	5'UTR	7102	PLD4	122618
619096	14q	104943622	104945444	1823	6	0.667	Exon(exon6of6)	9958	AHNAK2	113146
619096	14q	104947943	104953878	5936	33	0.485	Promoter(1-2kb)	1524	AHNAK2	113146
619096	15q	23440370	23442067	1698	9	0.667	5'UTR	5167	GOLGA6L2	283685

619096	15q	78766033	78766626	594	7	0.429	Promoter(<=1kb)	-920	ADAMTS7	11173
619096	15q	85579423	85581800	2378	15	0.533	Promoter(1-2kb)	-1110	AKAP13	11214
619096	15q	88857108	88859365	2258	6	0	Exon(exon12of18)	9865	ACAN	176
619096	16p	1256354	1256985	632	11	0.636	Promoter(<=1kb)	285	TPSD1	23430
619096	16p	1486371	1488463	2093	8	0.75	Promoter(<=1kb)	4	PTX4	390667
619096	16q	74391401	74391897	497	11	0.818	Exon(exon7of7)	13523	NPIPB15	440348
619096	16q	81127796	81128003	208	6	0.5	Exon(exon4of11)	8369	PKD1L2	114780
619096	16q	89100686	89101050	365	8	0.625	Promoter(<=1kb)	24	ACSF3	197322
619096	16q	89226863	89228419	1557	10	0.6	Promoter(2-3kb)	2229	ZNF778	197320
619096	17p	7482135	7482961	827	6	0.667	Promoter(<=1kb)	803	SLC35G6	643664
619096	17p	21300581	21300954	374	10	0.7	3'UTR	9112	MAP2K3	5606
619096	17p	21415458	21416431	974	16	0.875	Exon(exon3of3)	10322	KCNJ12	3768
619096	17q	76291123	76294016	2894	11	0.455	Promoter(<=1kb)	0	QRICH2	84074
619096	17q	81645135	81645417	283	6	0.333	Promoter(2-3kb)	2722	TSPAN10	83882
619096	18p	11609646	11610350	705	11	0.818	Promoter(<=1kb)	50	SLC35G4	646000
619096	18p	14542649	14543140	492	6	0.5	Promoter(<=1kb)	6	POTEC	388468
619096	18q	47033929	47035145	1217	6	0.833	Promoter(<=1kb)	476	ELOA2	51224
619096	18q	58535186	58537515	2330	9	0.333	Promoter(<=1kb)	0	ALPK2	115701
619096	19p	4510548	4513547	3000	20	0.4	Exon(exon3of6)	4157	PLIN4	729359
619096	19p	5455600	5456439	840	7	0.571	Promoter(<=1kb)	183	ZNRF4	148066
619096	19p	8333830	8334965	1136	6	0.667	Promoter(<=1kb)	0	KANK3	256949
619096	19p	8948231	8952171	3941	12	0.5	Exon(exon3of84)	29171	MUC16	94025
619096	19p	8959403	8961248	1846	6	0.667	Exon(exon3of84)	20094	MUC16	94025
619096	19p	8971838	8978096	6259	15	0.533	Exon(exon1of84)	3246	MUC16	94025
619096	19p	12430157	12431840	1684	9	0.444	3'UTR	9181	ZNF443	10224
619096	19p	15087213	15088040	828	11	0.273	Promoter(<=1kb)	233	OR1I1	126370
619096	19p	21971930	21974500	2571	7	0.714	Exon(exon4of4)	14408	ZNF208	7757
619096	19p	23743906	23745300	1395	6	0.333	Exon(exon4of4)	13537	ZNF681	148213
619096	19q	36996730	36997597	868	10	0.7	Exon(exon10of10)	5677	ZNF568	374900
619096	19q	37151928	37153149	1222	6	0.833	Exon(exon5of5)	19287	ZNF585A	199704
619096	19q	37885190	37888806	3617	8	0.625	Exon(exon6of6)	17788	WDR87	83889
619096	19q	39877222	39877880	659	6	0.5	Exon(exon20of28)	9412	FCGBP	8857
619096	19q	39886005	39886260	256	6	0.667	Promoter(1-2kb)	1032	FCGBP	8857
619096	19q	43913423	43914878	1456	8	0.5	Exon(exon10of10)	4861	ZNF45	7596
619096	19q	44106512	44108078	1567	8	0.125	Exon(exon6of6)	-4103	ZNF225	7768
619096	19q	51745958	51746963	1006	6	0.5	Exon(exon3of3)	3848	FPR1	2357
619096	19q	52437918	52439242	1325	7	0.429	Exon(exon4of4)	6504	ZNF534	147658
619096	19q	55912302	55913166	865	6	0.667	Exon(exon5of12)	19145	NLRP13	126204
619096	19q	58368293	58368875	583	7	0.429	Exon(exon3of3)	-5445	ZNF497	162968
619096	20p	20052354	20052736	383	6	0.167	Promoter(<=1kb)	0	CFAP61	26074
619096	20q	63562677	63565531	2855	10	0.6	Promoter(1-2kb)	-1072	HELZ2	85441
619096	21q	44550835	44551416	582	8	0.875	Promoter(<=1kb)	89	KRTAP10-2	386679
619096	22q	22352950	22353348	399	14	0.571	Exon(exon1of2)	30478	BMS1P20	96610
619096	22q	36191154	36191906	753	6	0.667	3'UTR	9971	APOL4	80832
619096	23p	3320126	3323750	3625	10	0.6	Exon(exon5of7)	22902	MXRA5	25878
619096	23p	8170039	8170141	103	6	0.5	Promoter(1-2kb)	1126	VCX2	51480
619096	23p	35802148	35803010	863	7	0.571	5'UTR	3357	MAGEB16	139604
621478	1p	18481403	18482163	761	7	0.714	Promoter(<=1kb)	421	KLHDC7A	127707
621478	1p	23874604	23875430	827	8	0.5	Exon(exon2of2)	-6310	FUCA1	2517
621478	1p	40067594	40067675	82	6	0	Promoter(<=1kb)	324	CAP1	10487
621478	1p	89186388	89186419	32	9	0.556	Promoter(<=1kb)	107	GBP4	115361
621478	1q	152213286	152213320	35	7	0.429	Exon(exon3of3)	10873	HRNR	388697
621478	1q	152219233	152221375	2143	14	0.714	Promoter(2-3kb)	2818	HRNR	388697
621478	1q	214640144	214642954	2811	12	0.5	Exon(exon12of20)	-5013	CENPF	1063
621478	1q	214644872	214647181	2310	10	0.4	Promoter(<=1kb)	-786	CENPF	1063
621478	1q	228315976	228318026	2051	6	0.5	Exon(exon50of81)	6492	OBSCN	84033
621478	1q	240207634	240208254	621	11	0.545	Exon(exon5of18)	-27517	FMN2	56776
621478	1q	247841312	247841582	271	6	0.833	Promoter(<=1kb)	314	OR11L1	391189
621478	2q	167243228	167245065	1838	7	0.286	Exon(exon9of11)	-3617	XIRP2	129446
621478	2q	184936178	184937636	1459	7	0.429	Exon(exon4of4)	69813	ZNF804A	91752
621478	2q	185789865	185794632	4768	10	0.8	Promoter(<=1kb)	0	FSIP2	401024
621478	2q	185805377	185808170	2794	6	0.333	Promoter(<=1kb)	0	FSIP2	401024
621478	2q	237762685	237764060	1376	10	0.5	Exon(exon8of8)	-4137	LRRFIP1	9208
621478	2q	238130542	238131702	1161	8	0.625	Promoter(1-2kb)	1594	ESPNL	339768
621478	3p	75736929	75738859	1931	10	0.6	Promoter(<=1kb)	0	MIR4273	100422955
621478	3q	194341097	194342571	1475	6	0.667	Exon(exon2of2)	8747	CPN2	1370
621478	4p	5988383	5989749	1367	7	0.571	Promoter(<=1kb)	0	C4orf50	389197
621478	4p	6300792	6302360	1569	6	0.833	Exon(exon8of8)	6021	WFS1	7466
621478	4p	8227004	8228508	1505	8	0.125	Promoter(<=1kb)	-24	SH3TC1	54436
621478	4p	38774486	38775552	1067	6	0.167	Promoter(<=1kb)	644	TLR10	81793
621478	4q	87811379	87811766	388	6	0.333	Exon(exon7of7)	-9645	MEPE	56955
621478	4q	154489498	154491312	1815	10	0.6	Promoter(<=1kb)	22	DCHS2	54798
621478	4q	185458217	185460011	1795	8	0.625	Promoter(<=1kb)	0	CCDC110	256309
621478	5q	79728956	79730716	1761	7	0.286	Exon(exon2of13)	-7426	CMYA5	202333
621478	5q	79731782	79734523	2742	13	0.308	Exon(exon2of13)	-3619	CMYA5	202333
621478	5q	83537326	83539905	2580	6	0.333	Promoter(1-2kb)	1712	VCAN	1462
621478	5q	140848579	140850786	2208	6	0.5	Promoter(<=1kb)	807	PCDHA9	9752

621478	5q	151565922	151568158	2237	9	0.778	Promoter(<=1kb)	786	FAT2	2196
621478	6p	46858771	46859502	732	7	0.571	Exon(exon17of21)	3802	ADGRF5	221395
621478	6q	111373870	111375649	1780	6	0.833	3'UTR	-6512	REV3L	5980
621478	6q	149888581	149890867	2287	7	0.714	Promoter(<=1kb)	0	RAET1E-AS1	100652739
621478	6q	159231899	159234370	2472	10	0.5	Exon(exon11of23)	13602	FNDC1	84624
621478	7p	38353718	38353991	274	8	0.625	Exon(exon2of2)	3699	TRG-AS1	100506776
621478	7q	100958721	100960873	2153	60	0.45	Promoter(<=1kb)	756	MUC3A	4584
621478	7q	100991195	100992398	1204	6	0.5	Exon(exon5of15)	-20656	MUC12	10071
621478	7q	100995575	100995831	257	6	0.5	Exon(exon5of15)	-17223	MUC12	10071
621478	7q	101034305	101038481	4177	26	0.5	Exon(exon3of12)	-5230	MUC17	140453
621478	8p	10607245	10610662	3418	9	0.667	Exon(exon4of4)	44481	RP1L1	94137
621478	8p	11331234	11332082	849	6	1	Promoter(<=1kb)	346	SLC35G5	83650
621478	8p	13021128	13022030	903	8	0.25	Exon(exon5of5)	9115	TRMT9B	57604
621478	8q	141218050	141219792	1743	6	0.667	5'UTR	8778	SLC45A4	57210
621478	9q	76705179	76707804	2626	8	0.5	Promoter(<=1kb)	121	PCA3	50652
621478	9q	76709263	76710846	1584	9	0.556	Promoter(<=1kb)	0	PRUNE2	158471
621478	9q	87886533	87888713	2181	8	0.625	Exon(exon4of4)	3656	SPATA31E1	286234
621478	9q	122553263	122554071	809	7	0.429	Promoter(<=1kb)	93	OR1N2	138882
621478	10q	46549378	46550723	1346	26	0.654	Exon(exon3of3)	4807	GPRIN2	9721
621478	10q	122084988	122087840	2853	8	0.75	Exon(exon4of23)	-25190	TACC2	10579
621478	11p	244106	244197	92	8	0.5	Promoter(<=1kb)	-232	PSMD13	5719
621478	11p	1194354	1196902	2549	7	0.571	Exon(exon34of49)	-26164	MUC5B	727897
621478	11p	5177978	5178478	501	6	0.167	Promoter(<=1kb)	186	OR52Z1	283110
621478	11p	5323362	5324256	895	8	0.5	Promoter(<=1kb)	41	OR51B2	79345
621478	11p	5389704	5390350	647	7	0.429	Promoter(<=1kb)	327	OR51M1	390059
621478	11p	5422212	5423123	912	11	0.636	Promoter(<=1kb)	101	OR51Q1	390061
621478	11p	5515185	5515931	747	6	0.333	Promoter(<=1kb)	768	UBQLNL	143630
621478	11p	5581045	5581738	694	9	0.333	Promoter(<=1kb)	168	OR52B6	340980
621478	11p	5841302	5841883	582	9	0.333	Promoter(<=1kb)	14	OR52E6	390078
621478	11p	5884818	5885061	244	7	0.429	Promoter(<=1kb)	547	OR52E4	390081
621478	11p	11352040	11352736	697	8	0.25	Promoter(<=1kb)	514	CSNK2A3	283106
621478	11p	12293639	12294368	730	7	0.857	Exon(exon29of35)	6739	MICALCL	84953
621478	11q	58214757	58215722	966	9	0.222	Promoter(<=1kb)	12	OR1S1	219959
621478	11q	58402523	58403307	785	9	0.444	Promoter(<=1kb)	102	OR5B3	441608
621478	11q	123906790	123907324	535	6	0.667	Promoter(<=1kb)	644	OR8D4	338662
621478	11q	124015600	124016477	878	7	0.143	Promoter(<=1kb)	25	OR10G4	390264
621478	11q	124264847	124265676	830	6	0.833	Promoter(<=1kb)	20	OR8G5	219865
621478	12p	4626568	4628549	1982	11	0.455	Exon(exon5of6)	14051	DYRK4	8798
621478	12p	6453119	6453670	552	6	0.667	Promoter(<=1kb)	633	TAPBPL	55080
621478	13q	25096713	25097182	470	6	0.333	Promoter(<=1kb)	845	PABPC3	5042
621478	13q	102732474	102733933	1460	6	0.333	Exon(exon4of4)	25139	CCDC168	643677
621478	14q	20060048	20060884	837	8	0.625	Promoter(<=1kb)	3	OR4L1	122742
621478	14q	20223729	20224484	756	7	0.571	Promoter(<=1kb)	19	OR11H6	122748
621478	14q	20640750	20641567	818	7	0.571	Promoter(<=1kb)	124	OR6S1	341799
621478	14q	21634161	21634644	484	6	0.5	Promoter(<=1kb)	296	OR10G2	26534
621478	14q	63599645	63599735	91	12	0.583	Exon(exon2of2)	41672	WDR89	112840
621478	14q	104943622	104948473	4852	12	0.667	Exon(exon6of6)	6929	AHNAK2	113146
621478	14q	104949673	104950766	1094	6	0.667	Exon(exon6of6)	4636	AHNAK2	113146
621478	15q	23440492	23442067	1576	7	0.714	5'UTR	5167	GOLGA6L2	283685
621478	15q	40621642	40623696	2055	6	0.333	Promoter(<=1kb)	0	KNL1	57082
621478	15q	59206980	59208056	1077	12	0.5	Promoter(<=1kb)	137	LDHAL6B	92483
621478	15q	85579423	85581800	2378	15	0.6	Promoter(1-2kb)	-1110	AKAP13	11214
621478	15q	99129423	99132517	3095	6	0.333	Exon(exon4of5)	7225	TTC23	64927
621478	16p	1256345	1256980	636	6	0.833	Promoter(<=1kb)	276	TPSD1	23430
621478	16p	1486371	1488463	2093	8	0.75	Promoter(<=1kb)	4	PTX4	390667
621478	16q	74391650	74391897	248	9	0.778	Exon(exon7of7)	13772	NPIPB15	440348
621478	16q	89226863	89228289	1427	8	0.625	Promoter(2-3kb)	2229	ZNF778	197320
621478	17p	10638198	10641099	2902	6	0.333	Exon(exon19of41)	-8169	MYH3	4621
621478	17p	21300581	21300954	374	9	0.667	3'UTR	9112	MAP2K3	5606
621478	17p	21415470	21416431	962	11	0.818	Exon(exon3of3)	10334	KCNJ12	3768
621478	17q	41727098	41728331	1234	6	0.833	Promoter(1-2kb)	-1201	HAP1	9001
621478	17q	53823368	53824891	1524	6	0.833	Promoter(<=1kb)	441	KIF2B	84643
621478	17q	76291123	76294016	2894	11	0.455	Promoter(<=1kb)	0	QRICH2	84074
621478	17q	81645135	81645417	283	6	0.333	Promoter(2-3kb)	2722	TSPAN10	83882
621478	18q	58535186	58537515	2330	12	0.417	Promoter(<=1kb)	0	ALPK2	115701
621478	19p	1036457	1036914	458	6	0.5	Exon(exon6of7)	-3187	ABCA7	10347
621478	19p	4510548	4513547	3000	16	0.5	Exon(exon3of6)	4157	PLIN4	729359
621478	19p	5455600	5456439	840	7	0.571	Promoter(<=1kb)	183	ZNRF4	148066
621478	19p	8948231	8951868	3638	11	0.545	Exon(exon3of84)	29474	MUC16	94025
621478	19p	15087242	15087953	712	6	0.333	Promoter(<=1kb)	262	OR1I1	126370
621478	19p	17281820	17284246	2427	9	0.556	Promoter(<=1kb)	0	ANKLE1	126549
[truncated: 251,681 more chars]
